# Hybrid Origins of Citrus Varieties Inferred from DNA Marker Analysis of Nuclear and Organelle Genomes

**DOI:** 10.1371/journal.pone.0166969

**Published:** 2016-11-30

**Authors:** Tokurou Shimizu, Akira Kitajima, Keisuke Nonaka, Terutaka Yoshioka, Satoshi Ohta, Shingo Goto, Atsushi Toyoda, Asao Fujiyama, Takako Mochizuki, Hideki Nagasaki, Eli Kaminuma, Yasukazu Nakamura

**Affiliations:** 1 Division of Citrus Research, Institute of Fruit Tree and Tea Science, NARO, Shimizu, Shizuoka, Japan; 2 Experimental Farm, Graduate School of Agriculture, Kyoto University, Kizugawa, Kyoto, Japan; 3 National Institute of Genetics, Comparative Genomics laboratory, National Institute of Genetics, Mishima, Shizuoka, Japan; 4 National Institute of Genetics, Center for Information Biology, National Institute of Genetics, Mishima, Shizuoka, Japan; USDA-ARS Southern Regional Research Center, UNITED STATES

## Abstract

Most indigenous citrus varieties are assumed to be natural hybrids, but their parentage has so far been determined in only a few cases because of their wide genetic diversity and the low transferability of DNA markers. Here we infer the parentage of indigenous citrus varieties using simple sequence repeat and indel markers developed from various citrus genome sequence resources. Parentage tests with 122 known hybrids using the selected DNA markers certify their transferability among those hybrids. Identity tests confirm that most variant strains are selected mutants, but we find four types of kunenbo (*Citrus nobilis*) and three types of tachibana (*Citrus tachibana*) for which we suggest different origins. Structure analysis with DNA markers that are in Hardy–Weinberg equilibrium deduce three basic taxa coinciding with the current understanding of citrus ancestors. Genotyping analysis of 101 indigenous citrus varieties with 123 selected DNA markers infers the parentages of 22 indigenous citrus varieties including Satsuma, Temple, and iyo, and single parents of 45 indigenous citrus varieties, including kunenbo, *C*. *ichangensis*, and Ichang lemon by allele-sharing and parentage tests. Genotyping analysis of chloroplast and mitochondrial genomes using 11 DNA markers classifies their cytoplasmic genotypes into 18 categories and deduces the combination of seed and pollen parents. Likelihood ratio analysis verifies the inferred parentages with significant scores. The reconstructed genealogy identifies 12 types of varieties consisting of Kishu, kunenbo, yuzu, koji, sour orange, dancy, kobeni mikan, sweet orange, tachibana, Cleopatra, willowleaf mandarin, and pummelo, which have played pivotal roles in the occurrence of these indigenous varieties. The inferred parentage of the indigenous varieties confirms their hybrid origins, as found by recent studies.

## Introduction

The genus *Citrus* L. (Family Rutaceae, subfamily Aurantiodeae) covers a wide range of edible and commercial varieties, including sweet orange, lemon, lime, grapefruit, and mandarins such as Clementine, Satsuma, King, and ponkan [1–4]. The production of major citrus varieties in tropical to sub-tropical and temperate zones exceeds 90 million tons, and the citrus industry occupies a significant position not only in the fruit industry but also in global agriculture [5,6]. In addition to the worldwide production of these major citrus varieties, numerous indigenous citrus varieties have also been produced in specific regions, and consumed locally [2,7]. Wide genetic diversity observed in *Citrus*, however, has made it difficult for taxonomists to draw a clear picture of their classification. Furthermore, mutants have occasionally been selected from limb sports or nucellar seedlings, and these constitute large variant strains [2,8–10]. Understanding how these modern citrus varieties arose from the ancestral basic species would bring us important insights for future citrus breeding.

Many botanists and taxonomists have proposed various approaches for the classification of a wide range of citrus varieties. Among them, two systems proposed by Swingle [11] and Tanaka [7,12] have been used in many studies. These two systems presume that most indigenous and commercial varieties arose from hybridization of ancestral ones, but differ in the way they treat indigenous varieties and cultivated varieties. Swingle primarily classified indigenous varieties rather than the cultivated varieties, placing two subgenera Papeda and Citrus in the genus *Citrus* [11]. The subgenus Papeda consists of section Papeda with four species, and section Papedocitrus with two species. He classified ten species in the subgenus Citrus, and regarded most cultivated varieties as natural hybrids of these indigenous species. He assigned most mandarin varieties to the scientific name *Citrus reticulata*, classified tachibana separately as *C*. *tachibana*, and also classified grapefruit, which arose from a chance seedling [2,9], separately as *C*. *paradisi*. In contrast, Tanaka stressed the importance of both indigenous varieties and cultivated varieties, and classified them equally as a species. He primarily placed two subgenera (Archicitrus and Metacitrus) in genus *Citrus*. The subgenus Archicitrus consists of five sections (Papeda, Limonellus, Citrophorum, Cephalocitrus and Aurantium) with 111 species, including grapefruit as *C*. *paradisi*. The subgenus Metacitrus consists of three sections (Osmocitrus, Acrumen and Pseudofortunella) with 48 species [12]. According to Tanaka’s system, individual mandarin varieties and tachibana were classified as a species with individual scientific names, and *C*. *reticulata* was assigned to the ponkan mandarin. Tanaka classified 145 citrus species in 22 different categories [12]. Since then, he has added several indigenous varieties to his classification system, and he released the ultimate list consisting of 159 species in 1969 [13]. Swingle considered *C*. *ichangensis* as a species of subgenus Papeda, and did not assign a scientific name to yuzu because he regarded it as a natural hybrid of *C*. *ichangensis*. In contrast, Tanaka classified *C*. *ichangensis* in subgenus Metacitrus section Osmocitrus, and classified yuzu to subgenus Metacitrus section Euosmocitrus as *C*. *junos* [12].

By the 1970s, various studies had been launched to classify citrus varieties using biochemical markers. In 1975, Scora published a novel paper based on his own chemotaxonomical study of citrus together with a survey of past literature [14]. He postulated three hypothetical taxa, mandarin (*C*. *reticulata*), citron (*C*. *medica*) and pummelo (*C*. *maxima*, formerly *C*. *grandis*), as the ancestors, and proposed that modern citrus varieties arose from repeated hybridization of these ancestors. In 1976, Barrett and Rhodes examined correlations among 22 indigenous varieties based on similarities for 146 traits, then estimated their affinities according to their deduced distance [15]. Similar chemotaxonomical studies gradually revealed the phylogenies of citrus varieties [16–21]. When DNA marker technology became available, taxonomical studies attempted classification of citrus using various DNA markers such as RAPD [22–26], RFLP [27], AFLP [28,29], ISSR [29–31] and SRAP [8,32]. Nicolosi and colleagues deduced a citrus phylogeny according to the genotypes of nuclear and chloroplast markers, and demonstrated that the origins of citrus varieties proposed by Scora [14] and Barrett and Rhodes [15] were acceptable [33,34]. Since then, the origins of some citrus varieties have gradually been revealed, and new classifications have been proposed [35,36]. Nowadays, codominant precision simple sequence repeat (SSR) or single nucleotide polymorphism (SNP) markers have been developed and used in most studies (see the reviews [34,37–40]). In addition, the chloroplast genome sequence of sweet orange has been released [41], and genome sequences of major citrus varieties are now public [42,43]. These genome sequence resources enable the design of precision DNA markers, and have revealed the parentage of Clementine, grapefruit, sweet orange, and limes and lemons [43–48]. However, the parentage of most indigenous varieties has not yet been determined.

Identifying the combination of seed parent and pollen parent is another important issue to be solved in parentage analysis. Many studies have revealed the phylogeny of citrus varieties by evaluating polymorphisms in the chloroplast or mitochondrial genome, or both [33,47,49–57]. However, some of these studies have only evaluated local citrus varieties [51,52], or limited numbers of varieties in the genus *Citrus* [50,57,58]. Next generation sequencing (NGS) technology has become commonplace, and it has been applied to the genotyping of citrus chloroplast genomes [56], but it is still a costly and time-consuming approach. Simple but reproducible and low-cost technologies that reveal sufficient polymorphisms are needed for the parentage analysis of a wide range of citrus varieties.

DNA marker analysis has been used in forensic genetics for inferring parentage or paternity, and identifying missing persons from their remains [59,60]. These techniques have also been used to infer sibships of wild populations [61–64], and are anticipated to be able to reveal unknown genealogy among indigenous citrus varieties. Two basic approaches have been adopted for parentage estimation with DNA marker analysis [64]. The first uses allele-sharing tests that estimate the number of alleles shared between two individuals at codominant DNA markers according to the Mendelian rules of inheritance. These tests estimate the probability of parentage from the proportion of DNA markers with shared alleles, and can also eliminate unrelated individuals. The discriminatory power of the test is proportional to the number of loci evaluated and the polymorphism of each DNA marker. However, these tests are susceptible to genotyping errors, and may give false positive or negative results [64]. Another approach is a likelihood ratio analysis, which compares the probabilities of alternate hypotheses for the parentage of two individuals (e.g., whether they are parent and offspring or unrelated) then estimates an odds score between these two hypotheses [62–64]. This is a widely used technique for examining proposed paternity or parentage and also to identify individuals [59,60,65]. The likelihood ratio analysis estimates the probability of the proposed parentage according to the likelihood of alleged parents and child, then compares it with a null relation between them deduced from the allele frequency within the population. The logarithm of likelihood ratio odds (LOD score) is often used to indicate the estimated score, but the number of DNA markers used for the evaluation and their allele frequency in the population influence the score [64]. Genotyping errors can also influence the score, and it is thus difficult to demonstrate a clear threshold for discrimination [63]. These two methods each have pros and cons; therefore, an approach that first excludes unrelated individuals using an allele-sharing test, then examines the probability of the proposed parentage using likelihood ratio analysis, will be a simple but effective way to infer parentage in a given population.

Because genotyping error severely affects the reliability of both methods, detecting such error and evaluating parentage with error-free DNA markers is a prerequisite for reliability. In the genotyping analysis of citrus varieties, however, wide genetic diversity among natural varieties reduces the transferability of DNA markers, resulting in false genotypes [44,46,64,66]. Selected somatic mutants could also be a drawback because some of them, but not all, have mutations in their genotype that make it difficult to estimate their identity.

The objective of the present study is to infer parentage among various citrus varieties using DNA marker analysis, and verify the inferred parentage statistically. We have attempted 1) to develop sufficient DNA markers for parentage analysis and eliminate erroneous DNA markers by examining them with a large enough set of known hybrid varieties, 2) to estimate genetic structures of indigenous varieties using these certified DNA markers, 3) to determine the cytosolic genotypes of individual varieties by evaluating chloroplast and mitochondrial genomes with DNA marker analysis, 4) to infer parentage among indigenous citrus varieties and verify it using a likelihood ratio approach.

## Materials and Methods

### Plant materials

We selected 371 citrus accessions consisting of 208 indigenous varieties, 78 hybrid varieties, and 85 selected strains (Table 1 and S1 Table). The indigenous varieties are from the collections of the Institute of Fruit Tree and Tea Science, NARO (NIFTS) that have been maintained at the Okitsu Citrus Research Division in Shizuoka prefecture, Japan. These varieties were selected from major mandarins (*C*. *reticulata*, *C*. *tangerina*, *C*. *unshiu*, *C*. *clementina*, *C*. *kinokuni*, *C*. *tachibana*, *C*. *nobilis*), pummelos (*C*. *maxima* and its hybrids), lemon (*C*. *limon*), sweet orange (*C*. *sinensis*), yuzu (*C*. *junos*), ichanchii (*C*. *ichangensis*) and their assumed natural hybrids. Sixteen varieties included variant selections to evaluate their genetic identity: four Clementines, two varieties classified to *C*. *tangerine* hort. ex Tanaka (Dancy and Obeni mikan), three grapefruits, five hyuganatsu, two iyos, 16 Kishus, 10 kunenbos, four ponkans, 12 pummelos, 21 Satsumas, two shiikuwashas, five sour oranges, 20 sweet oranges, 12 tachibanas, four tankans, and two willowleaf mandarins, respectively. Among them, kunenbo included both *C*. *nobilis* Lour. (King) and *C*. *nobilis* Lour. var. kunep Tanaka. Hybrid varieties used in this study are from the collections of NIFTS. Forty-five of them were developed by NIFTS, 11 by UC Riverside, 10 by the USDA, and the other 12 varieties were developed by seven other institutes or by farmers. We also used 85 strains that were selections from various crosses in NIFTS.

**Table 1:** Summary of citrus samples used in this study.

Category		Scientific names		Samples	Genotyped samples	Representative samples
		Swingle's system	Tanaka's system			
**Indigenous varieties**				**208**	**269**	**101**
	Clementine	*C*. *reticulata* Blanco	*C*. *clementina* hort. ex Tanaka	4	4	1
	Dancy	*C*. *reticulata* Blanco	*C*. *tangerina* hort. ex Tanaka	2	2	1
	Grapefruit	*C*. *paradisi* Macf.	*C*. *paradisi* Macf.	3	3	1
	Hyuganatsu	*C*. *sinensis* (L.) Osbeck	*C*. *tamurana* hort. ex Tanaka	5	6	1
	Iyo	*C*. *sinensis* (L.) Osbeck	*C*. *iyo* hort. ex Tanaka	2	2	1
	Kishu	*C*. *reticulata* Blanco	*C*. *kinokuni* hort. ex Tanaka	16	21	1
	Kunenbo ^1^^)^	*C*. *reticulata* Blanco	*C*. *nobilis* Lour. var. kunep Tanaka	10	13	4
	Natsudaidai	*C*. *paradisi* Macf.	*C*. *natsudaidai* Hayata	4	5	1
	Ponkan	*C*. *reticulata* Blanco	*C*. *reticulata* Blanco	4	5	1
	Pummelo	*C*. *grandis* Osbeck ^2^^)^	*C*. *grandis* Osbeck ^2^^)^	12	14	12
	Satsuma	*C*. *reticulata* Blanco	*C*. *unshiu* Marcov.	21	33	1
	Shiikuwasha	*C*. *indica*	*C*. *depressa* Hayata	2	3	2
	Sour orange	*C*. *aurantium* L.	*C*. *aurantium* L.	5	6	1
	Sweet orange	*C*. *sinensis* (L.) Osbeck	*C*. *sinensis* (L.) Osbeck	20	22	1
	Tachibana	*C*. *tachibana* Makino	*C*. *tachibana* (Makino) Tanaka	12	13	3
	Tankan	*C*. *sinensis* (L.) Osbeck	*C*. *tankan* Hayata	4	4	1
	Willowleaf mandarin	*C*. *reticulata* Blanco	*C*. *deliciosa* Ten.	2	2	2
	Others			80	111	66
Hybrid varieties		*C*.spp	*C*.spp	**78**	**83**	**75**
Selected strains		*C*.spp	*C*.spp	**85**	**90**	**85**
			**Total**	**371**	**442**	**261**

1) Kunenbo (*C*. *nobilis* Lour. var. kunep Tanaka) includes King mandarin (*C*. *nobilis* Lour.)

2) Now classified as *C*. *maxima* Merr.

### DNA extraction

Fully matured leaves were collected from each sample in the field at Okitsu, Shizuoka, then provided for DNA extraction using a modified protocol with a Nucleon Phytopure kit (GE Healthcare Life Science, NJ, USA) [67]. For certain varieties, several samples were collected from different trees. These were used as biological replicates to confirm the reproducibility of genotyping (RA in S1 Table). DNA concentration of the prepared DNA samples was determined using a Qubit Assay kit (ThermoFisher Scientific, Tokyo, Japan). UV absorbance analysis was used to confirm sample quality (A_260_/A_280_ > 1.8, and A_260_/A_230_ > 2.0), and gel electrophoresis analysis to verify the size and integrity of the extracted DNA samples.

### Citrus sequence resources for DNA marker design

Nucleotide sequences of expressed genes of citrus were obtained from public cDNA sequence databases dbEST (http://www.ncbi.nlm.nih.gov/dbEST/), RefSeq (http://www.ncbi.nlm.nih.gov/refseq/) and HarvEST (http://harvest.ucr.edu/) [68]. Citrus genome sequence resources in public databases, including BAC end sequences of Clementine [69] and Satsuma [70,71], and whole genome shotgun sequences of sweet orange ‘Ridge Pineapple’ in the trace file repository of Sanger reads (ftp://ftp.ncbi.nlm.nih.gov/pub/TraceDB/citrus_sinensis/), were also used for DNA marker design. Preliminary evaluation of the quality and length of each of these data sets was carried out using pregap4 [72], then a consensus sequence set was obtained for each set with Mira assembler [73] to reduce redundancy.

### NGS analysis of citrus varieties

NGS analysis of citrus varieties for mining SSR and indel regions was performed with a HiSeq 2000 sequencing system (Illumina, CA, USA) in paired-end mode [67]. Quality-checked NGS reads were mapped to the haploid Clementine reference sequence v.0.9 or v.1.0 [43] using BWA [74]. Candidate SSR or indel regions in the re-sequenced data were scored and identified using SAMtools and BCFtools [75], or using mreps [76].

### DNA marker design for genotyping nuclear genomes

SSR regions of each sequence were mined using mreps [76], then candidate regions with motif length between two and six nucleotides were selected. The identified candidate regions found in expressed genes or genomic sequences were used for oligonucleotide primer design with PerlPrimer [77] or Primer3 [78]. Previously reported SSR markers designed from BAC end sequences [46], or from EST sequences [79,80] were also used in this study.

### DNA marker design for genotyping organelle genomes

SSR markers for detecting polymorphisms in the chloroplast genome were designed from the chloroplast genome sequence of sweet orange ‘Ridge Pineapple’ (accession No. DQ864733) [41] by searching candidate SSR regions using mreps [76] as described in the previous section. Oligonucleotide primer sets for citrus mitochondrial genomes [53], and universal primer sets for the chloroplast genomes of dicotyledonous angiosperms [81] were also used for genotyping organelle genomes.

### Genotyping analysis

All genotyping analysis of nuclear or organelle genomes followed the multiplexed and multicolored post-labeling method in single tube with BStag reported by Shimizu and Yano [82]. Post-labeling of the PCR product with BStag is a simple but inexpensive method that does not require large alteration of the PCR program, and it reduces the total cost of analysis significantly. One of the six standard BStag sequences or an additional BStag sequence (F9TCC: 5'-CTAGTATCAGGACTCC-3') was added at the 5' end of the designed forward primer. A short ‘pigtail’ sequence was added at the 5' end of the reverse primer in order to suppress stuttering of the detected peak [83]. For each genotyping analysis, four oligonucleotide primer sets that were individually attached to different BStag sequences were mixed with the corresponding fluorescently labeled BStag primers. A typical PCR program for the amplification and post-labeling of the target region of the nuclear genome was: initial denaturation at 94°C for 3 min; 32 cycles of target amplification (20 s at 94°C followed by 35 s at 52–65°C); then three post-labeling cycles (20 s at 94°C followed by 10 s at 49°C and 5 s at 72°C); and final extension at 72°C for 10 min then terminated at 4°C. Each DNA marker was labeled separately with one of four different fluorescent dyes in a single tube at the labeling step. The reaction mixture was diluted twofold with water after the PCR. Then, a 0.4-μL aliquot of the diluted mixture was mixed with 0.1 μL GeneScan 600 LIZ® dye Size Standard (ThermoFisher Scientific, Tokyo, Japan) and adjusted to be 10 μL with deionized formamide, and then heat denatured at 95°C for 4 min. Electrophoresis of the labeled product was carried out on an ABI 3130xl DNA sequencer (ThermoFisher Scientific, Tokyo, Japan) with 36 cm length capillary using the standard program. Genotypes of each DNA marker/sample were called using GeneMapper 4.0 software (ThermoFisher Scientific, Tokyo, Japan).

### Parentage test and identity test

Parentage was confirmed for assumed parent–offspring triads by considering the inheritance of each allele from parents to offspring according to the Mendelian rule. Any DNA markers showing discrepancies in known hybrids were excluded from the analysis. The evaluation was carried out using a function of GUGS (General Utilities for Genotyping Study) software (Shimizu, T. in preparation). The identity test is a simple exact match test of each genotype to others for all combinations. If a pair of samples coincided with each other for the genotypes of all of the DNA markers, they were treated as identical. In this study, we counted the number of DNA markers that did not agree between any given pair of samples.

### Statistical evaluation of the genotype data

Observed heterozygosity (*H*_o_), expected heterozygosity (*H*_e_, equivalent to the unbiased estimator of gene diversity given by equation 8.4 of Nei [84]), number of unique alleles, and polymorphic information content (*PIC*, representing the probability of distinguishing a marker allele derived from either one of the parents [85]) were calculated using the frequency analysis function of Cervus [62] and confirmed with GUGS. The probability of match (*PM*), representing the probability that an unrelated individual happens to have the same genotype to others [60] is given by:

$$PM=\sum_{k=1}^{m}p_{k}^{2}.$$ (1)

Here, *p*_*k*_ is the observed frequency of each unique genotype *k* in the population, and *m* is the number of unique genotypes at a given nuclear locus. The gene diversity (*GD*) of a single allelic organelle genotype at a given locus was evaluated by

$$GD=1-\sum_{i=1}^{m}x_{i}^{2}$$ (2)

(equation 8.1 of Nei [84]). Here, *x*_*i*_ is the observed frequency of the *i*th single allele in the population, and *m* is the number of alleles at an organelle locus. This parameter (Nei’s *GD*) is an equivalent of the expected heterozygosity for diploid organisms. The values of the unique genotypes, *PM* and *GD*, were obtained using a function of GUGS. Wright’s fixation index (*F*_*w*_) was obtained by the equation *F*_*w*_ = (*H*_e_−*H*_o_)/*H*_e_ (equation 12.9 of Nei and Kumar [86]).

All statistical evaluations of the normal distribution (Shapiro–Wilk test) and one-way ANOVA (Kruskal–Wallis test) were conducted with the stats package of R (version 3.1.3, https://www.r-project.org/) in the Rstudio environment (version 0.99.893, https://www.rstudio.com/). Tests for equal variance and stochastic equality of two samples were conducted according to Brown–Forsythe test and Brunner–Munzel test using functions levene.test and brunner.munzel.test in the lawstat package [87]. The *p*-value adjustment for multiple samples was carried out by Benjamini–Hochberg (BH) correction with the p.adjust function of R. *F*-statistics for population analysis (*F*_*IT*_, *F*_*IS*_) [86,88,89] were estimated for each sample category or individual DNA marker using R packages hierfstat [90] and pegas [91] in combination with adegenet [92]. Additionally, Hedrick’s *G''*_*ST*_ [88], which is an equivalent of *F*_*ST*_ extended to multiallelic DNA markers, was estimated globally or pairwise using the mmod package of R [93] in combination with adegenet [92].

### Evaluation of Hardy–Weinberg equilibrium

An exact test of Hardy–Weinberg proportions for multiallelic genotype data was estimated with a Markov Chain Monte Carlo (MCMC) simulation method developed by Guo and Thompson [94], that was implemented as a function of Arlequin (version 3.5.2.2) [95]. The genotype data file used as input for Arlequin was formatted with CONVERT software [96] with no prior inferred population structure. We continued the MCMC simulation runs 10 times each for 1,000,000 iterations in both initial burn-in and de-memorization steps, and then the average of the estimated *p*-values was provided for evaluation.

### Factorial analysis and phylogenetic evaluation

Principal coordinate analysis (PCoA) and phylogenetic analysis of the obtained genotype data were carried out with DARWin (version 6.0.13) [97,98]. A dissimilarity matrix was obtained from the genotypes of each sample pair using a simple matching method (nuclear genotypes) or from modalities by Rogers and Tanimoto’s coefficient (organelle genotypes). The PCoA analysis assumed two to six axes (typically five), and data for the first two axes were used to draw a scatter plot. A consensus phylogenetic tree was inferred from the bootstrapped dissimilarity matrices obtained from 30,000 iterations for the nuclear genotype data or 5,000 iterations for the organelle genotype data using the weighted neighbor-joining method [99], then obtained consensus trees.

### Structure analysis

Structure analysis for the inference of the basic taxa and their proportions was carried out using STRUCTURE [100]. The genotype data for the 101 representative indigenous varieties obtained with the 123 selected DNA markers were formatted using CONVERT software [96] with no prior inferred population structure. Missing data were treated as lost (assigned ‘-9’ for the genotype data). The analysis assumed the admixture model for ancestry and that allele frequencies were correlated. In the estimation of the number of basic taxa (*K*), we varied *K* stepwise from two to ten, then evaluated the probability ten times for each *K* with 100,000 iterations of the initial burn-in and 1,000,000 MCMC runs. The inferred proportions of the *K* populations, and the estimated ln*Pr*(*X*|*K*), mean ln*P*(*K*) and its variance were used to obtain stdev Ln*P*(*K*), *L'*(*K*) and |*L''*(*K*)|, then Δ*K* was estimated as the mean of (|*L''*(*K*)| / stdev Ln*P*(*K*)), following Evanno et al [101]. We used the Structure Harvester web service [102] at http://taylor0.biology.ucla.edu/structureHarvester/ for this purpose. The inferred proportions of the *K* basic taxa were deduced individually from the output of Structure Harvester using the *Greedy* algorithm of CLUMPP [103]. We compared the full search and random input order running modes of CLUMPP, and also changed the running period for the permutation analysis from 1,000 to 1,000,000, but all results were identical. We therefore used the simulation results from CLUMPP run in *Greedy* mode with 100,000 permutation runs. The bar plot of inferred proportions was drawn with MS Excel.

### Allele-sharing test and stochastic verification of inferred parentage

Possible parent-to-offspring relationships between varieties were examined using an allele-sharing test. The test evaluates the ratio of the number of DNA markers that share at least one allele between two varieties to the total number of DNA markers. Any pair of varieties in which nearly all DNA markers shared an allele between the two varieties was selected as a candidate parent–offspring pair. When two varieties were assumed to be the parents of a particular offspring variety, the parentage of the assumed triad was examined using the parentage test.

The probability of the inferred dyad or triad being true single parent-to-offspring or parents-to-offspring combinations was examined by likelihood ratio analysis according to Marshal et al and Jones and Ardren [62,63]. In this analysis, the probabilities of two hypotheses (*H*_1_ and *H*_2_) are compared. Assume *P*(*G*|*H*_1_) is the probability of observing a particular pair of genotypes *G* under the hypothesis *H*_1_, and *P*(*G*|*H*_2_) is the probability of *G* under the hypothesis *H*_2_. The evaluated *P*(*G*|*H*_1_) relative to the evaluated *P*(*G*|*H*_2_) will give a likelihood ratio *L*(*H*_1_, *H*_2_ | *G*) that the *G* will be observed under the two hypotheses *H*_1_ and *H*_2_:

$$L\;(H_{1},H_{2}|G)=\frac{P(G|H_{1})}{P(G|H_{2})}.$$ (3)

In the parentage test, *H*_1_ presumes that a particular variety is an offspring of the alleged parent or parents, and *H*_2_ presumes that it is not an offspring of the alleged parents but a chance seedling that has arisen from a given population. The likelihood ratio *L* represents the probability that the offspring was obtained from the alleged parent(s) rather than being a chance seedling.

For the stochastic evaluation of the parentage test, let *g*_*S*_, *g*_*P*_ and *g*_*O*_ represent the genotypes of the alleged seed parent, alleged pollen parent and offspring, respectively, at a DNA marker. The likelihood ratio that the alleged parents are the true parents of the given offspring variety was estimated according to Eq (3) from Jones and Ardren [63]:

$$L(H_{1},H_{2}|g_{S},g_{P},g_{O})=\frac{T(g_{O}|g_{S},g_{P})}{P(g_{B})}.$$ (4)

Here, the numerator *T*(*g*_*O*_|*g*_*S*_,*g*_*P*_) is the transition probability of *g*_*O*_ given *g*_*S*_ and *g*_*P*_. This probability was estimated from the allele frequencies and a genotype combination according to Table 1 of Marshall et al [62]. The denominator *P*(*g*_*B*_) is the frequency of the offspring’s genotype in a particular population obtained according to Table 2 of Marshall et al [62]. The value *L* is the likelihood ratio that the parentage of this triad is correct compared to the offspring obtained its genotype from an unknown hybrid combination.

**Table 2:** Summary of DNA markers used in this study.

Type/source		Evaluated	Selected	(%)	Certified	(%)	Reference
Genomic SSR/INDEL		154	104	67.5%	58	37.7%	This study
EST/cDNA SSR		201	110	54.7%	87	43.3%	This study
Ollitrault, F et al. 2010		79	6	7.6%	6	7.6%	1)
Chen, C. et al. 2008		106	19	17.9%	12	11.3%	2)
Chen, C. et al. 2006		56	7	12.5%	6	10.7%	3)
	Total	596	246	41.3%	169	28.4%	

1) Ollitrault, F et al. (2010) Am. J. Bot. e124-e129.

2) Chen, C et al. (2008) Tree Genet. Genom. 4:1–10.

3) Chen, C et al. (2006) Theor Appl Genet. 112:1248–1257.

In a similar manner, another likelihood ratio for the alleged single parent to an offspring was estimated according to Eq (2) of Jones and Ardren [63], or Eq (5) of Marshall et al [62]:

$$L(H_{1},H_{2}|g_{S},g_{O})=\frac{T(g_{O}|g_{S})}{P(g_{B})}.$$ (5)

Here, the numerator *T*(*g*_*O*_|*g*_*S*_) is the transition probability of *g*_*O*_ given *g*_*S*_, estimated from their allele frequencies and genotype combination according to Brenner [104] or Table 2 of Marshall et al [62]. In most parentage analyses of wild plant populations, it is unknown which variety is the seed parent or the pollen parent. Thus, a particular alleged parent sample without any prior supporting information was assigned to either *g*_*S*_ or *g*_*P*_ arbitrarily. The probability of obtaining a particular genotype in a population was estimated from the allele frequencies at a given DNA marker, as *x*^2^ for homozygous genotype, or 2*xy* for a heterozygous genotype, where *x* and *y* are the allele frequencies in a population. The obtained value *L* is the ratio of the likelihood that this is a parent–offspring dyad to the likelihood that the offspring is from some unknown hybrid combination. All DNA markers used in the parentage test were presumed to be at Hardy–Weinberg equilibrium (HWE) in the given population. The *LOD* score (the natural logarithm of the likelihood ratio, *LR*) for the set of genotypes at multiple DNA markers is given by the product of *LR*:

$$LOD\;score=log(\prod_{m=1}^{k}{LR}_{m}),$$ (6)

where *LR*_*m*_ is a likelihood ratio for a triad or dyad at the *m*th DNA marker. Any DNA markers that showed discrepancies in the parentage test or allele-sharing test were excluded from *LOD* score estimation. The required cross trial index (*RCI*) was obtained by:

$$RCI=log\left(\frac{1}{N\prod_{k=1}^{m}f_{k}}\right).$$ (7)

Here, *N* is the number of individuals with unique genotype in the proposed population, *f*_*k*_ is the expected frequency of a particular genotype at the *k*th DNA marker estimated from the allele frequencies of the two alleles in the population (equation 7.4 in Nei [84]), and *m* is the total number of DNA markers used for the evaluation. Single parent–offspring probability (*SPP*) is not a likelihood ratio value but a cumulative probability between two particular individuals assuming that one is the alleged parent of a particular offspring variety without prior information on the other parent. The *SPP* value for the particular offspring (*g*_*O*_) and the alleged parent (*g*_*P*_) is obtained from the transition probability *T*(*g*_*O*_|*g*_*P*_) of *g*_*O*_ given *g*_*P*_ in a similar manner to that described above by:

$$SPP=\sum_{k=1}^{m}T_{k}(g_{O}|g_{P}),$$ (8)

where *m* is the total number of DNA markers used for the evaluation. These tests, frequency analyses and probability estimations were carried out using functions of GUGS software. The inferred genealogy was drawn as a family tree manually, or using Helium [105].

## Results

### Development and evaluation of DNA markers for nuclear genotyping of citrus

DNA sequences of citrus expressed genes from cloned cDNA, EST, and RefSeq in public sequence database repositories or the harvEST citrus database were used for DNA marker design. Preliminary clustering analysis of EST sequences with a sequence assembler reduced duplication in these data sets, and yielded 98,869 consensus sequences from 582,270 EST sequences. Another clustering analysis of whole genome shotgun sequences of sweet orange ‘Ridge Pineapple’ yielded 381,909 consensus sequences from 866,700 reads, but 46,341 Clementine BAC end sequences were not used for assembly because of their low redundancy. SSR mining of these data sets with mreps [76] identified 143,825 candidate regions from the consensus EST sequences, 314,967 from the consensus sweet orange whole genome shotgun sequences, and 16,159 from the Clementine BAC end sequences. SSR mining of the Clementine haploid genome sequence [43] (https://www.citrusgenomedb.org/) also identified 310,413 candidate SSR regions for both v0.9 (release 165) and v1.0 (release 182) genomes. These candidate regions were verified with resequencing data obtained from NGS analysis of 15 citrus varieties (banpeiyu A004, Clementine A009, dancy A016, hyuganatsu A036 and A038, King A054, Kishu A066, ponkan A108, Satsuma A113 and A122, sweet orange A162, willowleaf (Mediterranean) mandarin A200, ‘Encore’ B014, ‘Harehime’ B017, and ‘Kiyomi’ tangor B031). Candidate SSR regions that were supported with more than 40× Illumina read coverage were selected for primer design by referring their motif size, repeat length, genome position, gene annotation, specificity and versatility among citrus varieties. We also identified indel regions by referring to resequencing data, and these were also used for primer design. Consequently, we designed SSR and indel markers (S2 Table lists DNA markers by type and gives their sources).

### Verifying genotyping errors to select certified DNA markers

The genotypes of the DNA markers were preliminarily evaluated for peak height and peak height ratio, product size, and number of alleles in a small sample set consisting of Satsuma, sweet orange, Clementine, pummelos, lemon, ponkan and Kishu. Most of the evaluated primers successfully amplified PCR products, but a portion of them failed to amplify in particular varieties, or yielded multiple peaks in lemon. Consequently, 154 genomic SSR/indel markers and 201 EST/cDNA markers were primarily selected. The selected DNA markers were further examined for inconsistency using the parentage test with two hybrid varieties ‘Kiyomi’ tangor (Satsuma × sweet orange) and ‘Harumi’ (‘Kiyomi’ tangor × ponkan). Consequently, 104 genomic SSR/indel markers and 110 EST/cDNA SSR markers were selected for further evaluation (Table 2). Genomic SSR markers reported by Ollitrault et al [46] were also evaluated in a similar manner, and six valid markers were selected (Table 2). EST SSR markers reported by Chen et al [79,80] were also examined and 19 and seven SSR markers were selected.

Genotyping analyses of 371 plant samples (Table 1 and S1 Table) were conducted with the 246 selected SSR/indel markers and their genotype data were obtained (S3 Table). Genotyping error in these data was examined using the parentage test with 59 known hybrid varieties (Table 3, S1 Fig), and also with 63 of 85 selected strains. The hybrid varieties used for the test were developed from various crosses of Satsuma, Clementine, sweet orange, grapefruit, hassaku, ponkan, several pummelos, hyuganatsu, dancy, King, willowleaf mandarin, Kishu, and offspring of these varieties (Table 3). The parentage test using multiallelic DNA markers is strict to the combination; it will fail even when the correct triad is examined but their parents-to-child combination is incorrect (eg. AB × CD will give AC, but AC × CD will not give AB). Accordingly, this test examined not only erroneous DNA markers, but also incorrect hybrid combinations. For example, we found during the evaluation that ‘Fortune’ (Clementine × dancy) was discrepant to the reported parents [2]. Therefore, ‘Fortune’ was excluded from the reference varieties for the parentage test (the correct parentage of ‘Fortune’ will be discussed in a later section).

**Table 3:** Parentage of hybrid varieties with their organellar genotypes and the LOD scores used for error checking of DNA markers.

#	Hybrid variety			Seed parent			Pollen parent			LOD ^2)^	*RCI* ^3)^	*SPP* ^4)^	
	ID	Variety name	CT^1)^	ID	Variety name	CT ^1)^	ID	Variety name	CT^1)^	score		Seed	Pollen
1	B001	Akemi	C12	B031	Kiyomi	C12	B056	Seminole	C04	91.3	172.3	51.8	48.1
2	B003	Aki Tangor	C12	A125	Satsuma	C12	A162	Sweet orange	C07	78.8	162.3	51.3	49.2
3	B005	Amaka	C12	B031	Kiyomi	C12	B014	Encore	C12	95.9	169.0	47.5	55.3
4	B006	Ariake	C07	A162	Sweet orange	C07	A009	Clementine	C12	108.0	185.3	49.2	50.9
5	B007	Asumi	C12	B077	Okitsu-46	C12	B021	Harumi	C12	99.5	171.8	49.3	53.7
6	B008	Aurastar	C04	B072	H/FD-1	C04	A004	Banpeiyu	C04	— ^5)^	— ^5)^	36.4	34.5
7	B009	Awa Orange	C05	A036	Hyuganatsu	C05	A162	Sweet orange	C07	72.9	175.2	46.8	45.4
8	B010	Benibae	C12	B073	HF9	C12	B014	Encore	C12	100.9	161.1	54.3	55.5
9	B011	Benimadoka	C04	A088	Mato buntan	C04	A032	Hirado buntan	C04	210.0	256.9	45.0	44.9
10	B014	Encore	C12	A054	King	C12	A201	Willowleaf mandarin	C12	102.5	167.2	53.1	58.9
11	B015	Fairchild	C12	A009	Clementine	C12	B045	Orland	C04	84.0	156.2	56.3	50.1
12	B017	Harehime	C12	B070	E-647	C12	A125	Satsuma	C12	103.3	179.5	49.6	46.4
13	B018	Hareyaka	C12	B014	Encore	C12	A107	Ponkan	C12	104.0	163.9	56.7	55.1
14	B019	Haruhi	C12	B077	Okitsu-46	C12	B009	Awa Orange	C05	97.5	179.3	49.8	49.3
15	B021	Harumi	C12	B031	Kiyomi	C12	A107	Ponkan	C12	94.6	172.0	49.5	51.9
16	B022	Hayaka	C12	A125	Satsuma	C12	A107	Ponkan	C12	91.8	169.1	48.8	51.7
17	B023	Hayasaki	C04	A088	Mato buntan	C04	A032	Hirado buntan	C04	209.5	257.1	44.4	44.2
18	B024	Himekoharu	C12	B031	Kiyomi	C12	A101	Oogonkan	C05	81.4	171.5	49.0	47.0
19	B026	Honey	C12	A054	King	C12	A201	Willowleaf mandarin	C12	114.5	181.6	52.9	56.0
20	B027	Kanpei	C12	B039	Nishinokaori	C12	A107	Ponkan	C12	98.4	167.7	51.4	48.7
21	B028	Kara mandarin	C12	A125	Satsuma	C12	A054	King	C12	77.2	148.8	53.9	54.0
22	B030	Kinnow mandarin	C12	A054	King	C12	A201	Willowleaf mandarin	C12	104.2	171.6	52.3	57.5
23	B031	Kiyomi	C12	A125	Satsuma	C12	A162	Sweet orange	C07	81.4	169.2	47.3	48.4
24	B033	Lee	C12	A009	Clementine	C12	B045	Orland	C04	103.9	178.9	53.7	48.1
25	B034	May Pomelo	C04	A028	Hassaku	C04	A032	Hirado buntan	C04	139.7	216.0	41.9	50.2
26	B035	Mihaya	C12	B066	Tsunonozomi	C12	B076	No.1408	C12	98.7	160.1	53.9	56.2
27	B036	Mihocore	C12	A125	Satsuma	C12	B014	Encore	C12	97.5	163.9	53.9	56.2
28	B037	Mineola	C04	A024	Grapefruit	C04	A016	Dancy tangerine	C12	90.2	164.0	47.5	52.1
29	B038	Nankou	C12	A125	Satsuma	C12	A009	Clementine	C12	104.8	175.7	49.7	48.9
30	B039	Nishinokaori	C12	B031	Kiyomi	C12	A162	Sweet orange	C07	94.8	186.5	47.9	44.6
31	B040	Nou 5 gou	C12	B033	Lee	C12	A059	Kishu	C12	113.9	175.5	53.0	54.0
32	B041	Nou 6 gou	C12	A054	King	C12	A059	Kishu	C12	106.3	174.4	53.6	53.0
33	B042	Nou 7 gou	C04	B072	H/FD-1	C04	A004	Banpeiyu	C04	— ^5)^	— ^5)^	37.7	38.8
34	B043	Nova	C12	A009	Clementine	C12	B045	Orland	C04	119.1	193.3	51.2	46.3
35	B045	Orland	C04	A024	Grapefruit	C04	A016	Dancy tangerine	C12	101.1	174.0	46.5	49.9
36	B047	Osceola	C12	A009	Clementine	C12	B045	Orland	C04	100.5	176.9	53.9	48.7
37	B048	Page	C04	B037	Mineola	C04	A009	Clementine	C12	110.7	180.3	49.3	53.5
38	B051	Robinson	C12	A009	Clementine	C12	B045	Orland	C04	104.2	180.5	54.5	47.6
39	B052	Saga Mandarin	C12	A125	Satsuma	C12	B015	Fairchild	C12	93.8	166.1	52.7	50.7
40	B054	Seihou	C12	B031	Kiyomi	C12	B037	Mineola	C04	98.0	180.7	50.3	49.4
41	B055	Seinannohikari	C12	B071	EnOw21	C12	B069	Youkou	C12	92.7	160.1	54.5	52.7
42	B056	Seminole	C04	A024	Grapefruit	C04	A016	Dancy tangerine	C12	93.3	171.6	46.3	51.1
43	B057	Setomi	C12	B031	Kiyomi	C12	A107	Ponkan	C12	107.5	185.5	47.7	48.4
44	B058	Shiranuhi	C12	B031	Kiyomi	C12	A107	Ponkan	C12	82.4	161.9	52.8	49.5
45	B059	Southern Yellow	C05	B063	Tanikawa Buntan	C05	A059	Kishu	C12	104.8	185.1	46.1	42.0
46	B060	Summer Fresh	C04	A028	Hassaku	C04	A098	Natsudaidai	C04	94.0	191.0	45.6	42.9
47	B061	Sweet Spring	C12	A125	Satsuma	C12	A028	Hassaku	C04	69.3	155.5	52.3	48.1
48	B062	Tamami	C12	B031	Kiyomi	C12	B067	Willking	C12	109.6	174.5	49.9	53.9
49	B064	Tsunokagayaki	C12	B074	KyOw14	C12	B014	Encore	C12	94.7	164.2	52.5	52.3
50	B065	Tsunokaori	C12	B031	Kiyomi	C12	A125	Satsuma	C12	84.9	159.5	52.0	45.9
51	B066	Tsunonozomi	C12	B031	Kiyomi	C12	B014	Encore	C12	94.1	165.4	50.4	53.3
52	B068	Yellow Pummelo	C04	A028	Hassaku	C04	A032	Hirado buntan	C04	108.5	183.5	47.5	52.6
53	B069	Youkou	C12	B031	Kiyomi	C12	A107	Ponkan	C12	77.2	159.1	50.3	52.4
54	B070	E-647	C12	B031	Kiyomi	C12	B047	Osceola	C12	111.8	183.7	49.3	49.9
55	B071	EnOw21	C12	B014	Encore	C12	A125	Satsuma	C12	97.2	165.5	52.5	52.2
56	B073	HF9	C12	A125	Satsuma	C12	A162	Sweet orange	C07	72.9	165.8	48.4	46.3
57	B074	KyOw14	C12	B031	Kiyomi	C12	A125	Satsuma	C12	91.7	170.1	51.3	50.6
58	B075	KyOw21	C12	B031	Kiyomi	C12	A125	Satsuma	C12	96.9	174.3	51.4	48.8
59	B077	Okitsu-46	C12	B061	Sweet Spring	C12	A162	Sweet orange	C07	76.9	173.0	46.8	47.0

1) Category of organellar genotype (see Table 9).

2) LOD: See the section ‘Stochastic evaluation of inferred parentage’.

3) RCI: required cross trial index obtained by Eq 7.

4) SPP: Single parent–offspring probability for seed parent or pollen parent obtained by Eq 8.

^5^) Score or value was indeterminate due to lack of allele in H/FD-1.

All DNA markers were also evaluated for error with 64 selected strains.

The parentage test confirmed that 176 DNA markers were consistent on all hybrid varieties, and 182 DNA markers were consistent on all of the selected strains (S4 Table). However, 31 DNA markers showed discrepancies in more than two hybrid varieties, and 24 in more than two selected strains. Thirteen DNA markers failed to give an amplified product in just one hybrid variety, and 27 in just one selected strain. Most of these failures were due to simple technical error, and they were ignored in the following analysis. However, four DNA markers failed to amplify in the hybrid varieties and two in the selected strains (S4 Table). These DNA markers were assumed to contain a null allele, and they were excluded. Accordingly, the parentage test selected 58 certified genomic markers and 87 certified EST/cDNA markers. A similar evaluation for the published SSR markers also selected 6 certified SSR markers from the Clementine BAC end sequence [46], and 12 and 6 certified SSR markers from the EST sequence [79,80]. These 169 certified markers (166 SSR and 3 indel markers) are indicated by asterisks in S2, S3, S4, S6 and S7 Tables.

Most of the varieties used for the error check were offspring of mandarin, sweet orange or pummelo. Therefore, the selected DNA markers were expected to show less discrepant genotypes for those varieties or their offspring. On the contrary, lemon, yuzu, sour orange and citron were less frequently used as breeding parents for the hybrids. Consequently, the selected DNA markers could show discrepant genotypes for parentage analysis when these varieties or their offspring were used.

The 246 selected DNA markers were also used to construct a linkage map for two cross populations (Shimizu, T. in preparation). As a result, 225 of the selected DNA markers, including 154 certified markers, were mapped to one of the maps, or to both as a single locus (S2 Table gives the mapped linkage group in the ‘LG’ column). The mapped linkage groups of all DNA markers present in both maps agreed with each other. Among the mapped markers, 16 exactly matched the positions of other DNA markers on the two maps, and they were regarded as duplicate markers. These duplicate markers are indicated with double asterisks in S2, S3, S4, S6 and S7 Tables, and were excluded from the statistical evaluation. According to this selection and validation process, 153 certified DNA markers (150 SSR markers and 3 indel markers) that were consistent with 148 crosses, and uniquely mapped to a linkage group as a single locus without duplication, were finally selected.

### Genetic identity of indigenous varieties

All citrus samples were examined for their genetic identity to each other using the 169 certified DNA markers. The number of mismatched genotypes between all combinations of two indigenous varieties was scored using the identity test, and then summarized (S5 Table). Considerable numbers of mismatches were confirmed between most unrelated varieties or strains. None of the hybrid varieties except ‘Kuchinotsu-41’ (B032) and ‘Sagakashi 34’ (B053) coincide with any other varieties or strains. ‘Kuchinotsu-41’ is an autotetraploid selection of hyuganatsu and its genotypes completely agreed with those of hyuganatsu (Table 4, S5 Table). Likewise, ‘Sagakashi 34’ was confirmed to be a nucellar selection of ‘Shiranuhi’. The genotypes of each selected strain showed no coincidence with other varieties or strains, and these strains were confirmed to have been selected from diverse crosses. Twelve pummelo varieties (*C*. *maxima* or *C*. *maxima* hybrid) did not agree in their genotypes with the others; therefore we conclude that these pummelo varieties were not mutant selections.

**Table 4:** List of varieties that hold identical genotypes to the representative variety.

Variety	(Sub type)	No.	Representative variety		ND	Identical varieties				
			Variety/strain name	Scientitific name		No.	Variety name	Strain name	MM	ND
Andoukan		A001	(stock strain)	C. maxima (hybrid)	0	A056	Kinukawa	(stock strain)	2	2
Clementine		A009	(stock strain)	*C*. *clementina* hort. ex Tanaka	0	A010	Clementine	A peau fin	0	0
						A011	Clementine	Caffin	0	0
						A012	Clementine	de nules	0	0
Dancy		A016	(stock strain)	*C*. *tangerina* hort. ex Tanaka	0	A017	Dancy tangerine	Obenimikan	0	0
Girimikan		A023	(stock strain)	*C*. *tardiva* hort. ex Shirai	0	A180	Tajima mikan	(stock strain)	0	0
Grapefruit		A024	Marsh	*C*. *paradisi* Macfad.	0	A025	Grapefruit	Red blush	0	0
						A026	Grapefruit	Triumph	0	0
Henka mikan		A030	(stock strain)	*C*. *pseudo-aurantium* hort. ex Yu.Tanaka	0	A094	Nansho daidai	(stock strain)	0	0
Hiroshimanatsubuntan		A033	(stock strain)	*C*. *hiroshimana* hort. ex Yu.Tanaka	0	A181	Takumanatsukunenbo		0	0
Hyuganatsu		A036	(stock strain)	*C*. *tamurana* hort. ex Tanaka	0	A037	Hyuganatsu	Ihara 1	0	0
						A038	Hyuganatsu	Muroto konatsu	0	0
						A039	Hyuganatsu	Orange hyuga	1	0
						A040	Hyuganatsu	Shoukakukei hyuganatsu	0	0
						B032	'Kuchinotsu-41'	Hyuganatasu NC	0	0
Iyo		A044	Ootani Iyo	*C*. *iyo* hort. ex Tanaka	0	A043	Iyo	Miyauchi Iyo	0	0
Kishu		A059	Kishu	*C*. *kinokuni* hort. ex Tanaka	0	A057	Kishu	Hira Kishu	0	0
						A058	Kishu	Hisago komikan	1	1
						A060	Kishu	Kishu mikan	0	0
						A061	Kishu	Kishu mikan Ihara Ichijoji	0	0
						A062	Kishu	Komikan Fukuyama (Kinkou pearl)	0	0
						A063	Kishu	Komikan Kawachi	0	0
						A064	Kishu	Komikan Tensui	0	0
						A065	Kishu	Kouda mikan	0	0
						A066	Kishu	Mukaku Kishu (seedless Kishu)	0	0
						A067	Kishu	Nanfengmiju	0	0
						A068	Kishu	Ozaki komikan	0	0
						A069	Kishu	Sakurajima komikan Matsuura	0	0
						A070	Kishu	Sakurajima komikan senbatsu 1gou	0	0
						A071	Kishu	Sakurajima komikan Shirahama	0	0
						A072	Kishu	Taka mikan	0	0
Kizu		A073	(stock strain)	*C*. *kizu* hort. ex Yu.Tanaka	0	A029	Hebesu	(stock strain)	0	0
Koji		A076	(stock strain)	*C*. *leiocarpa* hort. ex Tanaka	0	A077	Komikan 2009–130	(stock strain)	0	0
						A189	Toukan	(stock strain)	0	0
Kunenbo	Kunenbo A	A081	(stock strain)	*C*. *nobilis* Lour. var. kunep Tanaka	0	A007	Bendiguangju	(Honchi kokitsu)	0	0
						A083	Kunenbo	Kagoshima 0027	1	1
						A084	Kunenbo	Kamikoshikijima	1	1
						A190	Tookunin	(stock strain)	2	2
						A193	Twukkunin	(stock strain)	0	0
						A194	Twukunihu	(stock strain)	0	0
						A195	Twuukuribu	(stock strain)	0	0
	Kunenbo B	A082	Kagoshima 0007	*C*. *nobilis* Lour. var. kunep Tanaka	0	(none)			—	
	Twukkuni	A192	(stock strain)	*C*. *nobilis* Lour. var. kunep Tanaka	1	(none)			—	
	King	A054	(stock strain)	*C*. *nobilis* Lour.	0	(none)			—	
Natsudaidai		A098	Kawano	*C*. *natsudaidai* Hayata	0	A096	Natsudaidai	(stock strain)	0	0
						A097	Natsudaidai	Beniamanatsu	0	0
						A099	Natsudaidai	Tachibana orange	0	0
Ponkan		A107	Oota	*C*. *reticulata* Blanco	0	A105	Ponkan	Ihara ponkan	0	0
						A106	Ponkan	Morita ponkan	0	0
						A108	Ponkan	Yoshida ponkan	0	0
Rokugatsumikan		A111	(stock strain)	*C*. *rokugatsu* hort. ex Yu.Tanaka	0	A020	Fukushukan	(stock strain)	0	0
Satsuma		A125	Okitsu Wase	*C*. *unshiu* Marcov.		A113	Satsuma	Aoshima unshu	0	0
						A114	Satsuma	Dobashi beni	1	0
						A115	Satsuma	Haraguchi wase	0	0
						A116	Satsuma	Imamura unshu	0	0
						A117	Satsuma	Iwasaki wase	0	0
						A118	Satsuma	Juman unshu	0	0
						A119	Satsuma	Jutaro unshu NC	0	0
						A120	Satsuma	Kinokuni unshu	0	0
						A121	Satsuma	Kuno unshu	0	0
						A122	Satsuma	Miyagawa wase	0	0
						A123	Satsuma	Nagahashi unshu NC	0	0
						A124	Satsuma	Niu unshu	0	0
						A126	Satsuma	Original tree	0	0
						A127	Satsuma	Otsu-4 (NC)	0	0
						A128	Satsuma	Shirakawa unshu	0	0
						A129	Satsuma	Sugiyama unshu	0	0
						A130	Satsuma	Suruga beni	0	0
						A131	Satsuma	Ueno wase	0	0
						A132	Satsuma	Yamada unshu NC	0	0
						A133	Satsuma	Yamashita beni	0	0
Satsuma Kikoku		A134	(stock strain)	*C*. spp	0	A078	Konejime	(stock strain)	0	0
Sour orange		A141	(stock strain)	*C*. *aurantium* L. var. crispa Yu.Tanaka	0	A139	Sour orange	Bouquet de fleurs	0	0
						A140	Sour orange	Chaozhouchen	0	0
						A142	Sour orange	Kaiseito	2	2
						A143	Sour orange	Zadaidai	0	0
						A093	Myrttle leaf orange	Chinott	1	0
Sweet orange		A162	Trovita	*C*. *sinensis* (L.) Osbeck	0	A148	Sweet orange	Cadenera	0	0
						A149	Sweet orange	Cara cara	3	2
						A150	Sweet orange	Crescent	0	0
						A151	Sweet orange	Hamlin	0	0
						A152	Sweet orange	Jincheng	0	0
						A153	Sweet orange	Joppa	0	0
						A154	Sweet orange	Mediterranean sweet orange	0	0
						A155	Sweet orange	Moro NC	1	0
						A156	Sweet orange	Parson Brown	0	0
						A157	Sweet orange	Pineapple	0	0
						A158	Sweet orange	Santa Catarina	0	0
						A159	Sweet orange	Seike navel	0	0
						A160	Sweet orange	Shamouti	0	0
						A161	Sweet orange	Tongzigan	0	0
						A163	Sweet orange	Washington navel	0	0
						A164	Sweet orange	Valencia	0	0
						A165	Sweet orange	Wuyuecheng	0	0
						A166	Sweet orange	Xuegan	0	0
						A167	Sweet orange	Yinzigan	1	0
Tachibana	Tachibana A	A172	Heda 1	*C*. *tachibana* (Makino) Tanaka	1	A168	Tachibana	(stock strain)	0	1
						A173	Tachibana	Heda 2	0	1
						A178	Tachibana	Okitsu	0	1
	Tachibana B	A175	Ishinami No.1	*C*. *tachibana* (Makino) Tanaka	0	A169	Tachibana	Anettaishijou	0	0
						A171	Tachibana	Hananoiwaya	0	0
						A176	Tachibana	Ishinami No.2	0	0
						A177	Tachibana	Oodomari OP-2	0	0
						A179	Tachibana	Reizanji	0	0
	Tachibana C	A174	Ishinami Minka	*C*. *tachibana* (Makino) Tanaka	0	(none)				
Tankan		A183	Taishun	*C*. *tankan* Hayata	0	A182	Tankan	(stock strain)	0	0
						A184	Tankan	Tarumizu 1	0	0
						A185	Tankan	T-132	0	0
Tosa buntan		A191	(stock strain)	*C*. *maxima* (L.) Merr.	0	A102	Ootachibana	(stock strain)	0	0
Ujukitsu		A197	(stock strain)	*C*. *ujukitsu* hort. ex Tanaka	0	A034	Houraikan	(stock strain)	0	0
'Shiranuhi'		B058	(stock strain)	(Hybrid)	0	B053	'Sagakashi 34'	(stock strain)	0	0

NC: Nucellar seedling

ND: Number of failed genotypes

MM: Mismatched genotypes

Any pair of samples that showed fewer than four mismatches were assumed identical. This threshold was determined empirically. According to this criterion, all genotypes of the four Clementine strains (A009 to A012) were identical, and they were confirmed as selected somatic mutants (Table 4, S5 Table). In the same way, genotypes of two *C*. *tangerina* varieties (A016: dancy and A017: obeni mikan), three grapefruit strains (A024–A026), two iyo strains (A043 and A044), four natsudaidai strains (A096–A099), four ponkan strains (A105–A108) and four tankan strains (A182–A185) agreed exactly among themselves, and were revealed to be somatic mutants. Except for one mismatch observed in the strain Hisago komikan (A058), the genotypes of 16 strains of Kishu (A057–A072) agreed with each other exactly, and they were confirmed as somatic mutants. Interestingly, these 15 strains of Kishu were collected in Japan, but a Chinese strain nanfengmiju (A067) exactly matched Kishu. The identity tests of hyuganatsu, Satsuma and sweet orange demonstrated slight mismatches within them. These mismatches were attributed to accidental technical failure. However, biological replication of Cara cara (A149) and the nucellar seedling selection Moro (A155) confirmed that their discrepancies were reproducible. These observations confirmed that the mutation of SSR markers is not frequent but a rare event, and unlikely to alter many genotypes of a strain from the original. We therefore concluded that all evaluated strains of hyuganatsu, Satsuma and sweet orange were somatic mutants. The identity test of five strains of sour orange (A141: stock strain of sour orange, A139: bouquet de fleurs, A140: chaozhouchen, A142: kaiseito, and A143: za daidai) confirmed them to be somatic mutants of sour orange. The examined genotype of myrtle-leaf orange Chinott (A093: *C*. *myrtifolia* Raf.) was discrepant in one DNA marker (NSX23) but otherwise identical to sour orange, and it was consequently confirmed to be another somatic mutant of sour orange. Though significant differences are widely recognized in their fruit shape, tree architecture and leaf size, such discrepancies between sour orange strains and myrtle-leaf orange strains were confirmed to be natural variations within sour orange.

The identity test also revealed unforeseen relationships between particular varieties. A possible pummelo hybrid variety andoukan (A001) coincided in its genotypes, except for two with missing data, with those of kinukawa (A056: *C*. *glaberrima* hort. ex Tanaka), which was thought to be a chance seedling of pummelo. A mandarin variety girimikan (A023: *C*. *tardiva* hort. ex Shirai) exactly matched in genotypes with those of Tajima mikan (A180: *C*. spp). Such identical relationships were also revealed between henka mikan (A030: *C*. *pseudo-aurantium* hort. ex Yu.Tanaka) and nansho daidai (A094: *C*. *taiwanica* Tanaka et Shimada), Hiroshimanatsubuntan (A033: *C*. *hiroshimana* hort. ex Yu.Tanaka) and Takumanatsukunenbo (A181: *C*. spp), kizu (A073: *C*. *kizu* hort. ex Yu.Tanaka) and hebesu (A029: *C*. *junos* hybrid), rokugatsumikan (A111: *C*. *rokugatsu* hort. ex Yu.Tanaka) and fukushukan (A020: *C*. spp), Satsuma kikoku (A134: *C*. spp) and konejime (A078: *C*. *junos* hybrid), and Tosa buntan (A191: *C*. *maxima* Merr.) and Ootachibana (A102: *C*. *otachibana* hort. ex Yu.Tanaka). Koji (A076: *C*. *leiocarpa* hort. ex Tanaka) matched in its genotypes with two varieties: komikan 2009–130 (A077: *C*. spp), which was a collection of NIFTS found in the Southwest Islands of Japan; and toukan (A189: *C*. spp). Ujukitsu (A197: *C*. *ujukitsu* hort. ex Tanaka) and horaikan (A034: *C*. *ujukitsu* hort. ex Tanaka) were presumed to be synonymous with each other, and this study confirmed the assumption with evidence. Tanaka described kizu (A073) and mochiyu (A091) as synonyms from different localities [12], but the identity test revealed that they arose from different origins. With these observations, we selected one of the natural variations from each set of identical genotypes, and regarded them as representatives of each genotype in the subsequent analysis.

### Genetic variation in the indigenous varieties

In contrast to the genetic identity found among the strains of various indigenous citrus varieties, variations in the strains of kunenbo and tachibana were identified (Table 4 and S5 Table). One kunenbo strain (A081: *C*. *nobilis* Lour. var. kunep Tanaka) agreed in its genotypes with six other strains (A083: kunenbo Kagoshima 0027, A084: kunenbo Kamikoshikijima, A190: tookunin, A193: twukkunin, A194: twukunihu, and A195: twuukuribu) that were classified to the same scientific name. Although three of them (A083, A084, A190) showed one or two mismatches to kunenbo, these were attributed to technical failure (S5 Table). Furthermore, bendi guangju (A007: *C*. spp, also known as honchi kokitsu in Japanese) exactly agreed in its genotypes with kunenbo (A081) (S5 Table). Two *C*. *nobilis* strains kunenbo Kagoshima 007 (A082: *C*. *nobilis* Lour. var. kunep Tanaka) and twukkuni (A192: *C*. *nobilis* Lour. var. kunep Tanaka) revealed 133 and 115 mismatches to kunenbo (A081) among the 169 DNA markers used, and kunenbo Kagoshima 007 (A082) disagreed with twukkuni (A192) for 100 markers (S5 Table). Additionally, King (A054: *C*. *nobilis* Lour.) revealed 99, 110 and 107 mismatches to kunenbo (A081), kunenbo Kagoshima 007 (A082) and twukkuni (A192), respectively (S5 Table). Although twukkuni (A192) contained one missing marker, these four varieties are obviously derived from different origins considering the frequency of mismatches among them. Accordingly, we selected these four unique genotypes as the representative varieties of *C*. *nobilis*, and tentatively assigned ‘kunenbo-A’ to kunenbo (A081), ‘kunenbo-B’ to kunenbo Kagoshima 007 (A082), ‘twukkuni’ to twukkuni (A192), and ‘King’ to King (A054) in subsequent study.

Similar genetic variations were also found among the strains of tachibana (*C*. *tachibana* (Makino) Tanaka). One tachibana strain, Heda 1 (A172), agreed in its genotype with three others (A168: tachibana stock strain, A173: Heda 2 and A178: Okitsu), but large discrepancies were found for tachibana Ishinami No.1 (A175) and tachibana ishinami minka (A174), with 45 and 72 mismatches (S5 Table). Tachibana ishinami No.1 (A175) and tachibana ishinami minka (A174) disagreed at 72 DNA markers (S5 Table). Tachibana ishinami minka (A176) agreed only with itself. However, tachibana ishinami No.1 (A175) agreed in genotype with five other tachibana strains (A169: anettaishijou, A171: hananoiwaya, A176: ishinami No.2, A177: Oodomari OP-2 and A179: Reizanji). On the basis of these observations, we selected these three unique genotypes as the representative varieties of tachibana, and tentatively assigned tachibana-A to Heda 1 (A172), tachibana-B to ishinami No.1 (A175), and tachibana-C to ishinami minka (A174) in subsequent study. Likewise, two shiikuwasha strains (A135 stock strain, and A136 Ogimikugani) disagreed at 44 DNA markers (S5 Table), and are therefore regarded as different strains of *C*. *depressa* Hayata.

According to these observations revealed by the genetic identity test, we selected 101 representative indigenous varieties that have unique genotypes. Kobayashi mikan (A074) was excluded from the representatives because it is a chimera and often gave three alleles. We also selected 75 representatives from 78 hybrid varieties by excluding one nucellar selection (B053: ‘Sagakashi 34’), one triploid variety (B046: ‘Oroblanco’), and one tetraploid variety (B032: ‘Kuchinotsu-41’). All 85 selected strains were selected as representatives since their genotypes were unique and did not overlap with others. Consequently, 261 unique representative varieties or strains were selected (Table 1). These are indicated by asterisks in S1, S3, S5 and S9 Tables.

### Statistical evaluation of genetic characteristics

The 261 selected representative varieties or strains in the three sample categories were evaluated for seven genetic parameters: number of unique genotypes (*Ng*); number of unique alleles (*Na*); observed heterozygosity (*Ho*); expected heterozygosity (*He*); polymorphic information content (*PIC*); match probability (*PM*); and Wright’s fixation index (*F*_*w*_); using the 169 certified DNA markers. Table 5 shows a summary of each parameter for the three sample categories (indigenous varieties, hybrid varieties and selected strains). S6 Table gives all the data for these seven parameters and the number of valid samples obtained with both certified and non-certified DNA markers.

**Table 5:** Summary of genetic characteristics and deduced population structure. A. Genetic characteristics for three sample categories.

Feature	Sample	N_50_	N_25_	N_75_	Mean	S-W P	B-F P	K-W P	Sig	Pairs	95% CI			P_adj_
*Ng*	IV	9.0	6.0	17.0	12.0	1.5.E-10	2.05E-15	1.30E-11	A	IV-HV	0.267	-	0.381	4.94E-09
	HV	6.0	4.0	10.0	7.1	1.8.E-09			B	IV-SL	0.241	-	0.352	8.98E-12
	SL	6.0	3.0	9.0	6.4	1.3.E-08			B	HV-SL	0.404	-	0.527	0.268
	ALL	10.0	6.0	18.0	13.4	8.4.E-11	—	—		—	—		—	—
*Na*	IV	5.0	3.0	7.0	5.6	2.5.E-09	7.05E-07	2.20E-09	A	IV-HV	0.291	-	0.406	5.38E-07
	HV	3.5	3.0	5.0	4.1	3.7.E-09			B	IV-SL	0.263	-	0.374	1.29E-09
	SL	3.0	2.0	5.0	3.8	1.2.E-08			B	HV-SL	0.403	-	0.524	0.231
	ALL	5.0	4.0	8.0	5.9	3.4.E-09	—	—		—	—		—	—
*Ho*	IV	0.567	0.455	0.703	0.567	1.0.E-03	0.4994	0.005462	A	IV-HV	0.350	-	0.472	7.99E-03
	HV	0.507	0.373	0.653	0.500	1.6.E-03			B	IV-SL	0.353	-	0.474	7.99E-03
	SL	0.529	0.388	0.647	0.504	5.5.E-04			B	HV-SL	0.443	-	0.567	0.868
	ALL	0.548	0.425	0.659	0.534	6.1.E-04	—	—		—	—		—	—
*He*	IV	0.567	0.478	0.716	0.570	2.4.E-05	0.8272	2.43E-09	A	IV-HV	0.295	-	0.412	2.06E-06
	HV	0.499	0.870	0.602	0.484	3.5.E-04			B	IV-SL	0.255	-	0.368	6.24E-10
	SL	0.489	0.361	0.572	0.461	1.8.E-04			B	HV-SL	0.397	-	0.520	0.159
	ALL	0.529	0.439	0.643	0.527	3.4.E-05	—	—		—	—		—	—
*PIC*	IV	0.495	0.385	0.668	0.512	1.1.E-02	0.03945	1.08E-09	A	IV-HV	0.290	-	0.406	7.98E-07
	HV	0.394	0.324	0.541	0.421	1.0.E-02			B	IV-SL	0.253	-	0.366	3.81E-10
	SL	0.375	0.318	0.506	0.397	2.6.E-03			B	HV-SL	0.396	-	0.519	0.1793
	ALL	0.460	0.366	0.589	0.468	1.8.E-03	—	—		—	—		—	—
*PM*	IV	0.261	0.132	0.361	0.279	7.8.E-10	0.8338	2.89E-13	A	IV-HV	0.623	-	0.737	2.34E-09
	HV	0.364	0.232	0.471	0.381	1.5.E-07			B	IV-SL	0.672	-	0.780	1.47E-14
	SL	0.403	0.277	0.489	0.408	3.4.E-08			B	HV-SL	0.488	-	0.611	0.117
	ALL	0.307	0.184	0.404	0.323	2.4.E-09	—	—		—	—		—	—
*F*_*W*_	IV	0.004	-0.052	0.075	0.008	0.01428	8.94E-05	2.23E-13	A	IV-HV	0.298	-	0.420	1.21E-05
	HV	-0.055	-0.106	0.004	-0.019	1.2E-10			B	IV-SL	0.213	-	0.324	4.32E-14
	SL	0.090	-0.169	0.000	-0.083	5.9.E-03			C	HV-SL	0.325	-	0.446	2.29E-04
	ALL	-0.021	-0.067	-0.043	-0.005	4.4.E-04	—	—		—	—		—	—

Average value of genetic characteristics with standard deviation for each sample category obtained with the 169 certified DNA markers. **IV**: 101 indigenous varieties, **HV**: 78 hybrid varieties, **SL**: 85 selected strains, **ALL**: 261 representative samples of these three categories.

***N***_**g**_: number of unique genotypes, ***N***_**a**_: number of unique alleles, ***H***_**o**_: observed heterozygosity, ***H***_**e**_: expected heterozygosity, ***PIC***: polymorphic information content, ***PM***: probability of match, ***F***_***w***_: fixation index.

***N***_**50**_, ***N***_**25**_, ***N***_**75**_: 50th, 25th and 75th percentile values.

**S-W P**: *p*-value of the normal distribution for each sample estimated by Shapiro–Wilk test.

**B-F P**: *p*-value of homogeneity of variance among the three sample categories estimated by Brown–Forsythe test.

**K-W P**: *p*-value of one-way ANOVA among the three sample categories estimated by Kruskal–Wallis test.

**Sig**: different letters represent significance between them at *p* < .01.

**95% CI** and **P**_**adj**_: The adjusted 95% confidence interval and *p*-value for each sample pair by Brunner–Munzel test. The *p*-value was adjusted by Benjamini–Hochberg (BH) correction for multiple sample comparison.

**Pairs**: combinations for the pairwise Brunner–Munzel test or *G''*_ST_ analysis.

Statistical evaluation of the data did not confirm the normal distribution of these data even after several data conversion methods were applied, and equal variance was not confirmed for *Ng*, *Na* and *F*_*w*_. Consequently, we adopted nonparametric analysis methods for the evaluation of these data. The medians (*N*_50_) of *N*_*g*_, *N*_*a*_, *H*_*o*_, *H*_*e*_, *PIC*, *PM* and *F*_*w*_ for the indigenous varieties were 9.0, 5.0, 0.567, 0.567, 0.495, 0.261 and 0.04, respectively. The median and mean values of *N*_*g*_, *N*_*a*_, *H*_o_ and *H*_e_ were higher than those reported by Curk et al [47], confirming that they were sufficiently polymorphic for the following genetic analysis. The observed *H*_o_ value demonstrated the heterozygous nature of the indigenous citrus varieties, and the *H*_e_ value demonstrated wide genetic diversity among them. The observed heterozygosity was high enough to use these DNA markers for the genetic mapping of crossed citrus populations. The observed high *PIC* value and low *PM* value confirmed their discriminatory power and indicates a low chance of misidentification of plant samples when using them.

The *N*_50_ and *N*_75_ values of *N*_*g*_ for the hybrid varieties (6.0 and 10.0) and the selected strains (6.0 and 9.0) were lower than for the indigenous varieties (9.0 and 12.0, Table 5). The observed differences between these were considered significant (*p* < .01), but not between the hybrid varieties and the selected strains (*p* > .05). Likewise, the *N*_*a*_ values for the hybrid varieties and the selected strains were significantly lower than that of the indigenous varieties (*p* < .01), but the difference between the hybrid varieties and the selected strains was not obvious (*p* > .05). These decreases in *N*_*g*_ and *N*_*a*_ strongly suggest that certain genotypes or alleles have been selected during the breeding program. These selected allele sets could be beneficial for citrus breeding. The differences in *N*_*g*_ and *N*_*a*_ were not significant between the hybrid varieties and the selected strains, suggesting that the usefulness of the selected alleles in breeding continues in these offspring.

Similarly, the *H*_*o*_, *H*_*e*_ and *PIC* values were significantly decreased in the hybrid varieties and the selected strains compared with the indigenous varieties (*p* < .01), but it was not obvious between the hybrid varieties and the selected strains. The observed decrease in *PIC* value coincided with the loss of alleles in the hybrid varieties and the selected strains. Though significant decreases were observed in *H*_*o*_ and *H*_*e*_, these values in the hybrid varieties and the selected strains remained high, confirming their higher heterozygosity. On the contrary, *PM* was 0.261 for the indigenous varieties but was increased to 0.364 and 0.403 in the hybrid varieties and the selected strains, respectively. The observed increase in *PM* coinciding with the loss of alleles resulted in an increase in the probability that unrelated individuals show the same genotype.

The estimated fixation index (*F*_*w*_) was not consistent among the three sample categories (*p* < .01). The *F*_*w*_ value for the indigenous varieties (0.004) suggested inbreeding within them. However, it was decreased for the hybrid varieties (-0.005) and this decrease is considered to have been achieved through artificial outcrossing. Interestingly, the *F*_*w*_ value was increased again in the selected strains (0.090). This increase is consistent with consanguineous mating among the indigenous varieties and the hybrid varieties during development of the selected strains, resulting in the loss of alleles.

The *F*_*IT*_ value [86,88,89] suggests that inbreeding of all citrus samples within the three sample categories is not obvious (Table 6). The *F*_*IS*_ value estimates that the inbreeding of individual varieties or lines in each sample category is not significant. However, the global *G''*_*ST*_ value [88] was as high as 0.0703, suggesting substantial inbreeding in each of the three sample categories. The within-population inbreeding between each sample category is demonstrated by the increase of *G''*_*ST*_ value between the indigenous varieties and the hybrid varieties (0.08637) or the selected strains (0.11925) (Table 6). In contrast, the increase was not significant between the hybrid varieties and the selected strains (0.00253). The deduced inbreeding within the hybrid varieties and the selected strains coincide well with the decrease in genotypes (*N*_*g*_), alleles (*N*_*a*_), observed heterozygosity (*H*_*o*_), expected heterozygosity (*H*_*e*_) and *PIC* values, and also the increase in match probability (*PM*) among them (Table 5). These observations support the initial hypothesis that the indigenous varieties used in this study are high in genetic diversity but that hybrid varieties have selected particular alleles from the indigenous varieties, and fewer alleles are maintained in the selected strains. The decreased variation in alleles would increase the probability of sharing the same allele by crossing, as suggested by the increase in match probability, and results in an increase in inbreeding in the hybrid varieties and the selected strains by frequent use of particular varieties as breeding parents.

**Table 6:** Deduced *F* statistics and *G''*_ST_ values among three sample categories.

Statistics	Value	Pairs	*G''*_*ST*_
*F*_*IT*_	0.0002	IV—HV	0.08637
*G''*_*ST*_	0.0703	IV—SL	0.11925
*F*_*IS*_	-0.0384	HV—SL	0.00253

Refer Table 5 for the symbols.

### Evaluation of genetic disequilibrium

Prior to estimating the population structure and analyzing the parentage of the indigenous varieties, which assume Hardy–Weinberg equilibrium (HWE) [100,106], we tested for HWE in the certified markers. Because SSR markers are highly polymorphic, we applied a Markov Chain Monte Carlo (MCMC) simulation method [94,107]. This method was implemented in Arlequin [95], and estimated the *p*-value for individual DNA markers, but it showed a slight variation in separate analyses because of its simulation principle. Accordingly, we tested for HWE in the 169 certified DNA markers ten times, and 31 DNA markers were considered not to satisfy HWE in the indigenous variety samples according to their average *p*-value (*p* < .05). These 31 DNA markers, along with the duplicated markers, were excluded in the following analysis, and 123 representative DNA markers that were confirmed to satisfy genetic consistency, singularity in the genome, and HWE in the indigenous varieties, were selected and provided to the following analysis.

### Factorial analysis and phylogenetic evaluation based on nuclear genotypes

**Fig 1:**
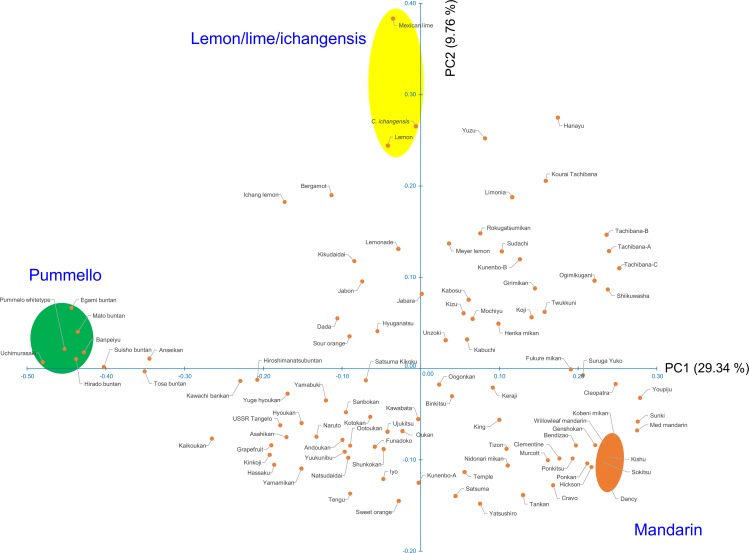
Principal coordinate analysis plot of 101 citrus indigenous varieties using genomic DNA markers. The plot was produced from the dissimilarity index deduced from 169 genomic DNA markers.

The population structure of the 101 representative indigenous varieties that excluded all identical genotype plant samples was examined with the 123 representative genomic DNA markers by principal coordinate analysis using DARWin [97,98]. The number of assumed axes was changed from two to six, but the values of the first two coordinates did not change. Therefore, the values of two major coordinates from five assumed coordinates were used to draw a scatter plot (Fig 1). These two coordinates explain about 39.1% of the total variation among the indigenous varieties. Five mandarin varieties (Kishu, dancy, willowleaf mandarin, sokitsu and kobeni mikan) are located together in the lower right region of the plot. Six pummelo varieties (banpeiyu, Egami buntan, Hirado buntan, Mato buntan, pummelo white type and uchimurasaki) are located on the left side of the plot. These five mandarins and six pummelos are located on opposite sides of the abscissa, and are considered to represent mandarin (*C*. *reticulata*) and pummelo (*C*. *maxima*), respectively. Meanwhile, three varieties (lemon, Mexican lime and ichanchii) that represent *C*. *medica* or *C*. *ichangensis* are located at the top center of the plot (Fig 1). The positions of these major citrus varieties, *C*. *reticulata*, *C*. *maxima* and *C*. *medica* or *C*. *ichangensis*, in the plot are reciprocal to each other, and constitute representative apexes in the plot. Though the absolute positions of these three basic groups are different, their triangular relationship is similar to previous reports [15,45], and confirms that these major citrus variety groups are well separated on this plot with the selected DNA markers.

*C*. *medica* and *C*. *ichangensis* are located close together in the upper apex of the scatter plot. Preliminary evaluation of the DNA markers found that a considerable portion of the evaluated DNA markers yielded three or more PCR products, or failed on amplification, for the citrus varieties of *C*. *medica*. These defective DNA markers were eliminated in this study, but this may have suppressed the separation between *C*. *medica* and *C*. *ichangensis*. Satsuma and sweet orange are located between pummelos and mandarins but closer to mandarins, suggesting the contribution of mandarin and pummelo for their occurrence. Yuzu is close to *C*. *ichangensis*, agreeing with its proposed origin as a chance seedling of *C*. *ichangensis* as suggested by Swingle [11]. Likewise, sour orange is located close to the middle among these three apexes, and it is considered an offspring of *C*. *maxima*, *C*. *reticulata* and *C*. *medica*. Other indigenous citrus varieties are located anywhere between these three apexes on the scatter plot. However, their distribution is not discrete but continuous, and there is no clear isolated or aggregated structure. These observations suggest a complex admixture history for the occurrence of these varieties.

**Fig 2:**
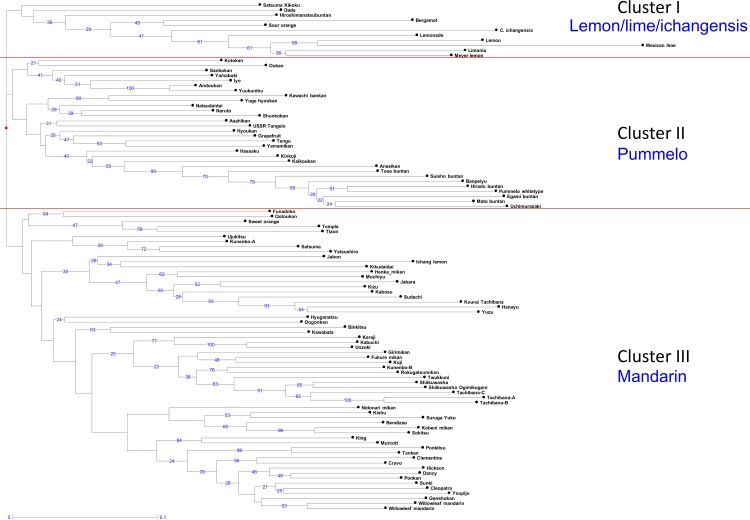
Phylogenetic tree of 101 citrus indigenous varieties estimated from genomic DNA markers. The tree was produced using the neighbor-joining method from genetic distances by the simple matching method. Node labels show bootstrap support values larger than 20.

A phylogenetic tree of the 101 representative indigenous varieties based on 123 representative genomic DNA markers was constructed using the neighbor-joining method [99] from bootstrap analysis. We prepared the consensus tree from 30,000 bootstrap trials, but runs with 5,000 or 10,000 MCMC iterations produced identical trees. The tree classifies the 101 indigenous citrus varieties into three major clusters (Fig 2). Cluster I consists of 11 varieties including the three varieties (lemon, Mexican lime and ichanchii) that constitute the upper central apex in Fig 1. Sour orange and the offspring of lemon were classified into this cluster, and *C*. *medica* and *C*. *ichangensis* with their varieties are thus considered to be classified in this cluster. Cluster II consists of 30 varieties including the five pummelo varieties that constitute the left apex in the PCoA plot (Fig 1). All pummelo offspring varieties are found in this cluster; therefore, this cluster is considered to represent *C*. *maxima* and its offspring. Cluster III consists of 60 varieties, and the six mandarin varieties that constitute the remaining apex in Fig 1 are found in this cluster. However, various varieties that are located at diverse positions in the PCoA plot (Fig 1), for example Temple, ujukitsu, henka mikan, mochiyu, jabara, kizu, kabosu, sudachi, which are regarded as natural hybrids [7,11], are also found in this cluster. Consequently, this cluster is considered to represent *C*. *reticulata* and its offspring varieties. All representative varieties are classified into different clades, and no consolidated clade structure reminiscent of the PCoA plot is obvious in the tree.

### Inferring basic taxa and their proportions in individuals of the indigenous varieties

**Fig 3:**
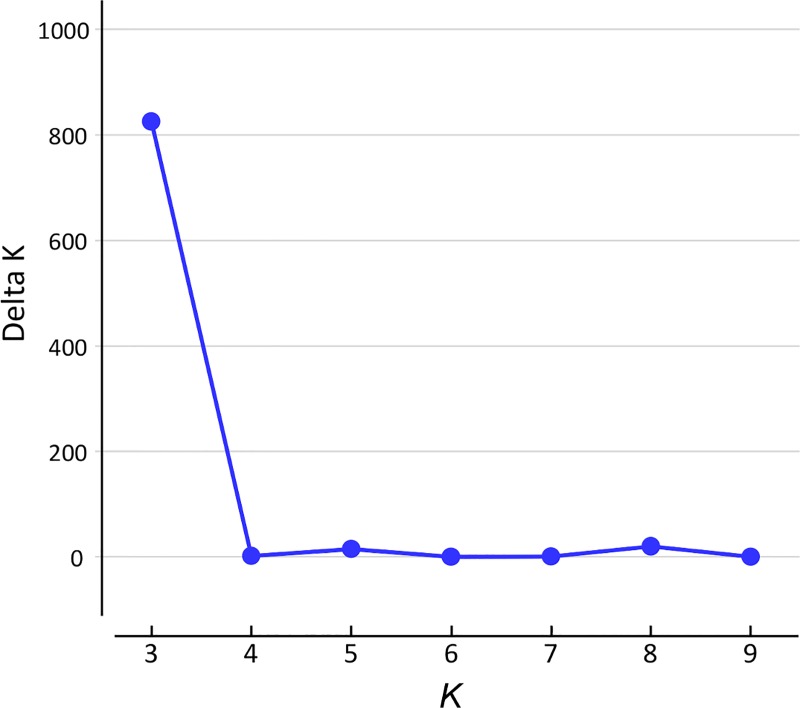
Magnitude of Δ*K* deduced from structure analysis as a function of *K*. Simulation runs were performed for *K* = 2 to 10 with 10 iterations for each *K*, and the mean values of Δ*K* are plotted against *K*.

The proportions of basic taxa for 101 indigenous varieties were inferred by a model-based clustering method with a Bayesian MCMC approach according to Pritchard et al [108]. The deduced number of basic taxa (*K*) was obtained from the Δ*K* value according to Evanno et al [102] by varying *K* from two to ten (Table 7). The magnitude plot of Δ*K* against *K* shows a large single peak at *K* = 3 (Fig 3). The delta *K* values for *K* = 4 to 9 were close to zero (Table 7). Changing the iteration period for initial burn-in (50,000 or 100,000) and MCMC runs (500,000 or 1,000,000) in the simulations gave the same result with a single large peak at *K* = 3 (data not shown). Another large peak at *K* = 5 appeared when all genotype data that deviated from HWE were included in the structure analysis (data not shown). However, this disappeared when the genotype data that deviated from HWE were removed. Therefore, that peak was considered to be spuriously caused by disequilibrium in particular DNA markers, and discarded. The deduced *K* value conforms to the current understanding that the basic citrus ancestral taxa consist of *C*. *medica*, *C*. *maxima* and *C*. *reticulata* [14,15,35,109].

**Table 7:** Summary of Δ*K* estimation for Structure analysis.

K	Reps	Mean LnP(K)	Stdev LnP(K)	Ln'(K)	|Ln''(K)|	Delta K
2	10	-22738.00	0.49	—	—	—
3	10	-21305.92	1.07	1432.08	681.49	637.78
4	10	-20555.33	73.18	750.59	209.44	2.86
5	10	-20014.18	182.62	541.15	2388.97	13.08
6	10	-21862.00	3550.42	-1847.82	4291.36	1.21
7	10	-19418.46	259.15	2443.54	5009.38	19.33
8	10	-21984.30	8041.19	-2565.84	37.24	0.00
9	10	-24587.38	4307.95	-2603.08	5124.23	1.19
10	10	-22066.23	5352.53	2521.15	—	—

The magnitude of Δ*K* from STRUCTURE analysis was calculated according to Evanno et al (2005) for 101 indigenous varieties with 123 DNA markers. The prior *K* value was varied from 2 to 10, with 10 simulation runs for each *K*. Simulations for each *K* used 100,000 iterations for initial burn-in, and 1,000,000 iterations for the subsequent MCMC estimation.

**Fig 4:**
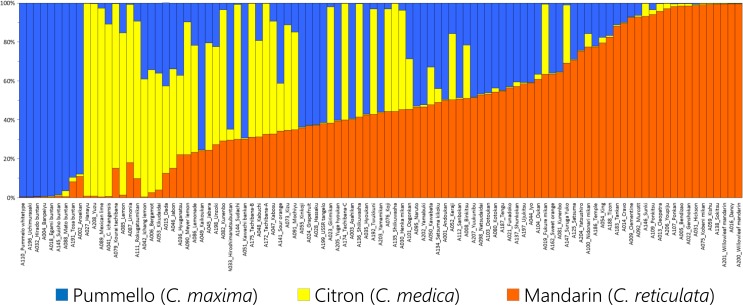
Inferred admixture populations (Q) of 101 indigenous citrus varieties for a *K* = 3 population model in STRUCTURE. Three clusters correspond to the deduced basic taxa at *K* = 3 (pummelo, citron and mandarin).

The inferred admixture population (Q) plot of the 101 indigenous varieties at *K* = 3 demonstrates that several varieties mostly consisted of one of the three populations (Fig 4, S8 Table). The inferred admixture proportion of the first population was over 90% for seven pummelo varieties (banpeiyu, Egami buntan, Hirado buntan, Mato buntan, suisho buntan, pummelo white type and uchimurasaki), and these were regarded as a taxon representing pummelo (*C*. *maxima*). Likewise, eight varieties (hanayu, ichanchii, Kourai Tachibana, lemon, limonia, Mexican line, rokugatsumikan and yuzu) had proportions of more than 80% for the second population, and this group was regarded as a taxon representing citron (*C*. *medica*). Sixteen varieties (bendizao, Clementine, Cleopatra, dancy, Hickson, Kishu, kobeni mikan, genshokan, Murcott, sokitsu, sunki, ponkitsu, youpiju, ponkan, willowleaf mandarin and Mediterranean mandarin) had proportions of more than 90% for the third population, and were regarded as a taxon representing mandarin (*C*. *reticulata*). The deduced proportions of these three basic taxa in individual varieties will be discussed in the following section.

### Development and evaluation of DNA markers for genotyping chloroplast and mitochondrial genomes

For the purpose of categorizing citrus varieties according to their cytoplasmic organellar genotypes (referred to as ‘cytotypes’ hereafter), genotypes of both chloroplast and mitochondria genomes were evaluated using DNA markers for each genome. Phylogenetic analyses of citrus varieties based on chloroplast genome polymorphisms have been reported for *trn*L–*trn*F and *trn*T–*trn*L sequences [50], *rbc*L–ORF106, *psa*A–*trn*S, *trn*H–*trn*K and *trn*D–*trn*T intergenic regions [57], *trn*L–*trn*F sequences [51], *trn*L–*trn*F intergenic regions [52], nine chloroplast genomic intergenic regions [110], *mat*K gene sequences [55], and *trn*S–*trn*G, *rps*16, *rpl*16, *atp*B–*rbc*L and *acc*D–*psa*I sequences [111]. However, our preliminary attempts at genotyping chloroplast genomes with previously reported DNA markers showed occasional failures or less amplification on particular varieties (data not shown). Therefore, we designed new SSR markers for the chloroplast genome by referring to the chloroplast genome sequence of sweet orange ‘Ridge Pineapple’ [41]. SSR mining of the sweet orange genome using mreps [76] identified 94 candidate regions. The forward and reverse primers of four SSR markers in the short single copy region and nine SSR markers in the large single copy region were designed to anchor at two adjacent genes to amplify an SSR found in an untranslated region between these genes. Preliminary evaluation selected two SSR markers for the short single copy region (CSS03: *ndh*E–*ndh*G and CSS04: *ndh*D–*psa*C) and two SSR markers for the large single copy region (CSL01: *psb*A–*trn*K and CSL09: *rpl*16–*rps*3) (Table 8). These regions have not been evaluated in citrus, or in other plant species. However, they were confirmed to be stable and versatile in a wide range of citrus varieties.

**Table 8:** SSR markers for the genotyping of organelle genomes.

**Chloroplast (this study**^**1**^**)**								
Name	Target region	SSR From	To	Size	Program	F/R	BStag	Sequence
CSS03	*ndh*E-*ndh*G	122,864	122,878	292	54/28	F	F9GTC	ctagtatgaggacgtcTCATTAACCAACTCCGTACCA
						R	-	gtttcttGGCGCGTCAATAACAAATCT
CSS04	*ndh*D-*psa*C	121,941	121,955	211	56/28	F	F9GCC	ctagtattaggacgccGTGGTAAAGACAAGATACACTTGG
						R	-	gtttcttATGGCATGAAACAACCCGA
CSL01	*psb*A-*trn*K	1,743	1,775	355	60/28	F	F9GAC	ctagtatcaggacgacCAGTGCTAGTTATCCAGTTACAGA
						R	-	gtttcttCGGGCAACCCATTCTTATTATT
CSL09	*rpl*16-*rps*3	86,713	86,730	276	54/32	F	F9TAC	ctagtatcaggactacCGCACACTAAGCATAGCAAT
						R	-	gtttcttCCTCTACAAACCATTGGAGCTA
**Chloroplast (Weising and Gardner, 1991**^**2**^**)**								
Name	Target region	SSR From	To	Size	Program		BStag	Sequence
ccmp2.2	5' to *trn*S	8,609		189	54/28	F	F9GAC	ctagtatcaggacgacATCGTACCGAGGGTTCGAAT
						R	-	GATCCAGGGCGTAATCCCG *)
ccmp6.2	ORF77-ORF82 intergenic	45,119		103	60/28	F	F9GCC	ctagtattaggacgccCGATGCATATGTAGAAAGCC
						R	-	CATTACGTGCGACTATCTCT
ccmp7.2	*atp*-B-*rbc*L intergenic	57,339		133	52/36	F	F9CCG	ctagtattaggacccgACATCATTATTGTATACTCTTTC
						R	-	CAACAGATAAAACTGTCAAG
ccmp10.2	*rpl*2-*rps*19 intergenic	86,694		103	54/32	F	F9AGG	ctagtattaggacaggTTTTTTTTTAGTGAACGTGTCA
						R	-	TTCGCCGCCGTACTAAATAG
**Mitochondria (Froelicher, et al. 2011**^**3**^**)**								
Name	Target region	SSR From	To	Size	Program		BStag	Sequence
*rrn*5/*rrn*18-1	*rrn*5-*rrn*18	161,129	161,383	273	56/28	F	F9GTC	ctagtatgaggacgtcGGGTGAAGTCGTAACAAGGT
						R	-	GAGGTCGGAATGGGATCGGG
*nad*2/4-3	*nad*2-*nad*4-3	330,666	331,566	239	52/28	F	F9CCG	ctagtattaggacccgGACCTTCACCTCAAATCA
						R	-	TTCAGATAACACGCACC
*nad*7/1-2	*nad*7-*nad*1-2	132,914	133,077	163	52/28	F	F9AGG	ctagtattaggacaggGGAACATAGCATAGGG
						R	-	TTTGATATAGGCTCGCT

^1^ SSR markers developed in this study with reference to the sweet orange chloroplast genome (DQ864733).

^2^ SSR markers for chloroplast genomes by Weising and Gardner, 1991. The underlined nucleotides represent the modifications from their original sequences.

*) Forward and reverse primers were swapped from the original report.

^3^ DNA markers for genotyping mitochondrial genomes by Froelicher et al 2011.

All these DNA markers were designed for the single tube post labeling protocol with BStag. F/R: Forward/reverse primers.

We also evaluated the availability of recently published universal SSR markers for chloroplast genomes [81] and citrus mitochondrial genomes [53]. Preliminary evaluation of the 10 SSR markers for chloroplast genomes [81] failed to amplify or yielded a low amplification product with some citrus varieties. The primer sequences of these SSR markers were modified with reference to the sweet orange chloroplast genome [41], and four redesigned SSR markers (ccmp2.2, ccmp6.2, ccmp7.2 and ccmp10.2) were selected that show stable and consistent genotypes for a wide range of samples. Likewise, we evaluated 15 citrus DNA markers for mitochondria [53], but nine of them yielded amplified products too long for fragment analysis and were excluded. Three (*rrn*5/*rrn*18-1, *nad*2/4-3, and *nad*7/1-2) were selected according to their product size and stability on various citrus samples. Consequently, we selected 11 SSR markers, eight for chloroplast genomes and three for mitochondrial genomes.

### Genetic characteristics estimated using organellar DNA markers

Genotyping analysis of 371 plant samples in three sample categories using the 11 selected organelle SSR markers yielded a single product in each sample, with no failure to amplify (S9 Table). The observed product sizes of these SSR markers almost agreed with previous reports [53,81]. All plant samples that were assumed to be somatic mutants on the basis of their nuclear genotypes (Table 4) revealed identical cytotypes.

The 11 organelle SSR markers evaluated each produced two to eight alleles among the samples and a total of 43 alleles were identified (Table 9). Their product sizes ranged from 129 to 370 bp (Table 9). The average number of alleles for the four chloroplast DNA markers from Weising was 3.8 [81], and that for the three mitochondrial DNA markers from Froelicher was 2.3 [53]. In contrast, the average number of alleles for the chloroplast DNA markers developed in this study was as high as 5.3. The genetic diversity (Nei’s *GD*) of the indigenous varieties ranged from 0.040 to 0.765 (Table 9). The median number of genotypes for all samples was 3.0 and the median *GD* was 0.477 (Table 10), and this SSR marker set was confirmed to be polymorphic enough to classify the cytotypes of a wide range of citrus samples. Previous studies classified the cytotypes of sweet orange (*C*. *sinensis*) and pummelo (*C*. *maxima*) into the same category [33,47,50,53,54,57], but this study separated them into different categories. Curk and colleagues classified citrus varieties into six categories with three chloroplast DNA markers and three mitochondrial DNA markers [47]. Their study classified sunki (*C*. *sunki*) and Cleopatra (*C*. *reshni*) into the same group as wild mandarin (*C*. *reticulata*) and limonia (*C*. *limonia*), and also classified Ichang lemon (*C*. *sp*.) into the group of *C*. *maxima*. In this study, those varieties were classified into independent groups, confirming the usefulness of the four chloroplast DNA markers developed in this study for fine separation and parentage analysis of citrus varieties.

**Table 9:** Organellar genotype characteristics of plant samples observed using SSR markers.

**Summary of identified genotypes for three sample categories**														
Markers			CSS03	CSS04	CSL01	CSL09	ccmp2.2	ccmp6.2	ccmp7.2	ccmp10.2	*rrn*5/*rrn*18-1	*nad*2/4-3	*nad*7/1-2	Total
	Indigenous varieties		2	4	7	8	3	8	2	2	2	3	2	43
	Hybrid varieties		1	3	3	2	1	3	1	1	2	2	2	21
	Selected lines		1	3	3	3	1	3	1	1	2	2	2	22
	All samples		2	4	7	8	3	8	2	2	2	3	2	43
**Size distribution of the amplified product (bp)** *														
Markers			CSS03	CSS04	CSL01	CSL09	ccmp2.2	ccmp6.2	ccmp7.2	ccmp10.2	*rrn*5/*rrn*18-1	*nad*2/4-3	*nad*7/1-2	Total
	Max		335	237	379	311	224	149	159	130	274	269	172	379
	Min		315	233	370	298	210	139	148	129	269	253	164	129
**Genetic diversity of each SSR markers estimated by Nei's GD**														
Markers			CSS03	CSS04	CSL01	CSL09	ccmp2.2	ccmp6.2	ccmp7.2	ccmp10.2	*rrn*5/*rrn*18-1	*nad*2/4-3	*nad*7/1-2	Average
	Indigenous varieties		0.0776	0.6673	0.7651	0.6467	0.0398	0.6402	0.0776	0.0776	0.4321	0.5236	0.4321	0.3981
	Hybrid varieties		0.0000	0.4193	0.4193	0.3930	0.0000	0.4154	0.0000	0.0000	0.3930	0.3930	0.3930	0.2569
	Selected lines		0.0000	0.2570	0.2570	0.2510	0.0000	0.2372	0.0000	0.0000	0.2478	0.2478	0.2478	0.1587
	All samples		0.0304	0.5736	0.6087	0.5658	0.0154	0.5300	0.0304	0.0304	0.4770	0.5218	0.4770	0.3509

*) Product size include BStag sequence (16 bp).

**Table 10:** Differences in genetic characteristics of organellar genotype among three sample categories.

Feature	Sample	N_50_	N_25_	N_75_	Mean	S-W P	B-F P	K-W P	Sig	Pair	95% CI			P_adj_
*Ng*	IV	3.0	2.0	5.5	3.9	0.00167	0.04179	0.04813	a	IV-SV	0.033	-	0.429	0.031
	HV	2.0	1.0	2.5	1.9	0.01659			b	IV-SL	0.045	-	0.475	0.046
	SL	2.0	1.0	3.0	2.0	0.00805			b	SV-SL	0.275	-	0.783	0.815
	ALL	3.0	2.0	5.5	3.9	8.4.E-11	—	—		—	—		—	—
*GD*	IV	0.4320	0.0780	0.6418	0.3982	0.07757	0.1605	0.01515	a	IV-SV	0.019	-	0.467	0.041
	HV	0.3930	0.0000	0.3985	0.2568	0.00093			b	IV-SL	0.000	-	0.447	0.041
	SL	0.2425	0.0000	0.2487	0.1588	0.00082			b	SV-SL	0.024	-	0.546	0.098
	ALL	0.4770	0.0300	0.5390	0.3509	0.00562	—	—		—	—		—	—

IV: indigenous varieties, HV: hybrid varieties, SL: selected strains, ALL: 261.

Refer Table 5 for other symbols.

The cytotypes obtained were categorized into 18 classes (C01 to C18) by the nonredundant allele set of 11 SSR markers (Table 11). Two SSR markers for the chloroplast genome (ccmp7.2 and ccmp10.2) showed identical genotype patterns, as did two SSR markers for the mitochondrial genome (*rrn*5/*rrn*18-1 and *nad*7/1-2) (Table 11). Each of the 18 classes consisted of a unique and non-redundant genotype set, and is referred to by its representative variety (Table 11). All representative plant samples of the three sample categories were classified into one of these 18 cytotype classes (Table 12 and S9 Table). The 18 proposed cytotypes agreed with the hybrid varieties that were used to evaluate genotyping error (Table 3), and showed no discrepancies. Therefore, we conclude that these classes demonstrate genuine cytotypes.

**Table 11:** The classes of citrus cytoplasmic genotype (cytotype) according to the nonredundant alleles of eight chloroplast and three mitochondrial SSR markers.

Class	Cytotype	Chloroplast markers								Mitochondria markers		
		CSS03	CSS04	CSL01	CSL09	ccmp2.2	ccmp6.2	ccmp7.2	ccmp10.2	rrn5/rrn18-1	nad2/4-3	nad7/1-2
C01	C. ichangensis type	315	237	371	306	224	149	159	129	274	261	172
C02	Mexican lime type	315	233	379	305	217	148	148	130	274	261	172
C03	Limonia type	315	233	370	304	210	141	148	130	269	261	164
C04	pummelo type	315	234	379	298	210	143	148	130	274	269	172
C05	Hyuganatsu type	315	233	377	298	210	144	148	130	274	269	172
C06	Lemon type	315	233	377	305	210	141	148	130	274	269	172
C07	Sweet orange type	315	233	377	298	210	143	148	130	274	269	172
C08	Satsumakikoku type	315	233	377	299	210	143	148	130	274	269	172
C09	Yuzu type	315	233	371	311	210	139	159	129	274	261	172
C10	Ichang lemon type	315	233	377	298	210	145	148	130	274	269	172
C11	Kunenbo_B type	335	233	374	310	210	141	148	130	269	253	164
C12	Mandarin type	315	235	372	303	210	141	148	130	269	253	164
C13	Sunki type	315	235	370	305	210	141	148	130	269	261	164
C14	Tachibana type	335	233	374	310	210	142	148	130	269	253	164
C15	Tachibana C type	315	234	371	305	210	141	148	130	269	261	164
C16	Ogimikugani type	335	234	374	310	210	142	148	130	269	253	164
C17	Cleopatra type	315	234	370	304	210	141	148	130	269	261	164
C18	Koji type	315	234	378	304	210	142	148	130	274	269	172

**Table 12:** Summary of all plant samples and representative samples according to the organelle genotype classes.

Class	Type	Swingle's system (1943)	Tanaka's system (1961)		All plant samples			Representative samples		
					IV	HV	SL	IV	HV	SL
C01	*C*. *ichangensis* type	*C*. *ichangensis*	*C*. *ichangensis Swingle*		1	0	0	1	0	0
C02	Mexican lime type	*C*. *aurantifolia*	*Citrus aurantifolia(Christm*.) Swingle		1	0	0	1	0	0
C03	Limonia type	*C*. *limon*	*C*. *limonia Osbeck*		1	0	0	1	0	0
C04	pummelo type	*C*. *grandis*, *C*. *paradisi*	*C*. *grandis*, *C*. *paradisi*		43	16	8	33	15	8
C05	Hyuganatsu type	*C*. *sinensis*,*C*. *paradisi*	*C*. *grandisC*. *aurantium L*.		9	5	3	5	4	3
C06	Lemon type	*C*. *limon*, *C*. *aurantium*	*C*. *limon Burm*.*f*., *C*. *aurantium L*.		12	0	1	5	0	1
C07	Sweet orange type	*C*. *limon*, *C*. *sinensi*, *C*. *nobilis hybrid*	*C*. *limon*, *C*. *sinensis*, *C*. *nobilis*, *C*. *reticulata*		44	1	0	16	1	0
C08	Satsumakikoku type	*C*. *aurantium*	*C*. *aurantium L*.		2	0	0	1	0	0
C09	Yuzu type	*(C*. *ichangensis hybrid)*	*C*. *junos*		3	0	0	3	0	0
C10	Ichang lemon type	*(C*. *ichangensis hybrid)*	*C*. *wilsonii Tanaka*		1	0	0	1	0	0
C11	Kunenbo B type	*C*. *reticulata hybrid*	*C*. *nobilis Lour*.		1	0	0	1	0	0
C12	Mandarin type	*C*. *reticulata*	*C*. *nobilis*, *C*. *reticulata*, *C*. *unshiu*		63	56	73	21	55	73
C13	Sunki type	*C*.*reticulata var*. *austera*	*C*. *sunki Hort*. *ex Tanaka*		7	0	0	4	0	0
C14	Tachibana type	*C*. *tachibana*	*C*. *tachibana*		11	0	0	2	0	0
C15	Tachibana C type	*C*. *tachibana*	*C*. *tachibana*		1	0	0	1	0	0
C16	Ogimikugani type	*C*. *reticulata hybrid*	*C*. *depressa Hayata*		1	0	0	1	0	0
C17	Cleopatra type	*C*.*reticulata var*. *austera*	*C*. *reshni Hort*. *ex Tanaka*		1	0	0	1	0	0
C18	Koji type	*(C*. *ichangensis hybrid)*, *(C*. *indica hybrid)*	*C*. *leiocarpa*, *C*.*sudachi Hort*. *ex Shirai*		6	0	0	3	0	0
				Total	208	78	85	101	75	85

IV: indigenous varieties, HV: hybrid varieties, SL: selected strains (see Table 1).

Among these cytotypes, C04 (pummelo type) was dominant in the indigenous varieties, followed by C12 (mandarin type) and C07 (sweet orange type) (Table 12). In contrast, nine indigenous varieties were exclusively classified with their own cytotypes (C01: *C*. *ichangensis* type, C02: Mexican lime type, C03: limonia type, C08: Satsumakikoku type, C10: Ichang lemon type, C11: kunenbo B type, C15: tachibana C type, C16: Ogimikugani type and C17: Cleopatra type). Two kunenbo varieties (A081: kunenbo A and A194: twukkuni) shared the same cytotype C07 (sweet orange type), in accord with the report by Yamamoto and colleagues [49]. However, two other kunenbo varieties were revealed to have different cytotypes (C12: mandarin type for A054: King and C11: kunenbo B type for A082: kunenbo B). Likewise, the cytotypes of two tachibana varieties (A174: tachibana A and A177: tachibana B) were identical (C14: tachibana type), but another tachibana (A176: tachibana C) had its own unique cytotype C15 and we refer to it as tachibana C type (S9 Table). One shiikuwasha variety (A136: shiikuwasha) shared same cytotype C13 with sunki and others, but another shiikuwasha variety (A137: shiikuwasha Ogimikugani) had its own unique cytotype C16 (S9 Table). These observations confirm their divergent origins as suggested by their nuclear genotypes (Table 4).

Other cytotypes (C05, C06, C09 and C18) were shared among three to five varieties. The cytotype C05 (hyuganatsu type) was shared among hyuganatsu, kawabata, lemonade, oogonkan and tengu (S9 Table). Similarly, bergamot, lemon, Hiroshimanatsubuntan, rokugatsumikan and sour oranges shared cytotype C06 (lemon type). Hanayu, Kourai tachibana and yuzu shared cytotype C09 (yuzu type). Cytotype C18 (koji type) was shared among girimikan, koji and sudachi. These observations enabled us to estimate their origin and possible hybrid combinations, and this is discussed in the following section.

**Fig 5:**
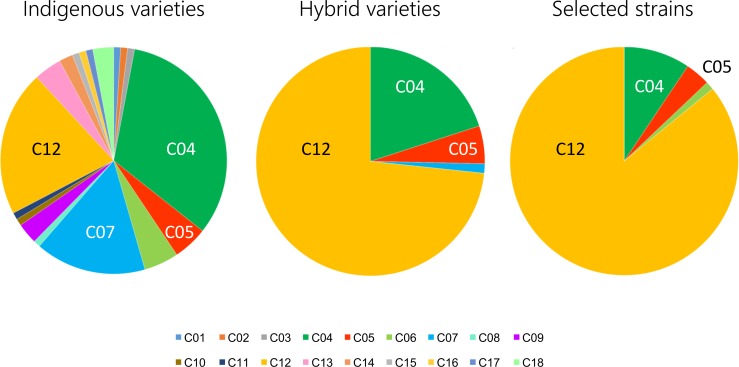
Organelle genome composition of the three sample categories. Each pie chart shows the relative abundance of each cytotype within the three sample categories. C01: C. ichangensis type, C02: Mexican lime, C03: limonia type, C04: pummelo type, C05: hyuganatsu type, C06: lemon type, C07: sweet orange type, C08: Satsumakikoku type, C09: yuzu type, C10: Ichang lemon type, C11: kunenbo B type, C12: mandarin type, C13: sunki type, C14: tachibana type, C15: tachibana C type, C16: Ogimikugani type, C17: Cleopatra type, C18: koji type.

As observed in the genotyping analysis of the nuclear genome (Table 5), a significant decrease in the number of genotypes and *GD* between the indigenous varieties and the hybrid varieties or the selected strains were also confirmed in the organelle genomes (Table 10). The observed decrease suggests the frequent use of specific cytotypes during cross breeding programs. Comparing the observed cytotype among three sample categories demonstrates that four out of 18 cytotypes have been selected during the breeding process (Table 12, Fig 5). The cytotype C12 (mandarin type) has been selected preferentially in the hybrid varieties and the selected strains from all cytotypes.

### Factorial analysis and phylogenetic evaluation based on organellar genotypes

**Fig 6:**
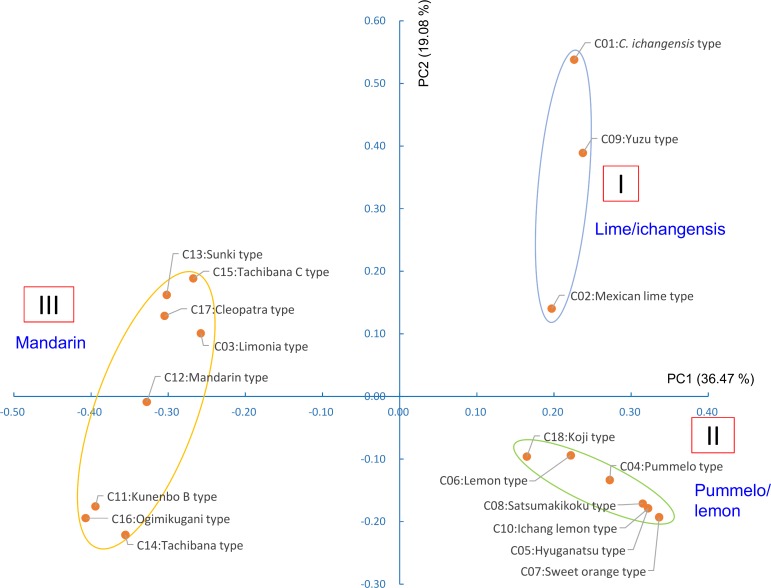
Principal coordinate analysis plot of 18 representative organelle genomes observed within 101 citrus indigenous varieties. The plot was produced from modalities by Rogers and Tanimoto’s coefficient estimated from 11 organellar DNA markers. Three groups of genomes that cluster together are circled (I, II and III).

PCoA analysis of the 18 cytotypes clearly distinguishes them into three clusters (Fig 6). As with the PCoA analysis of the nuclear genome, the first two coordinates showed identical values when the number of assumed axes was changed from two to six, and the values of the first two of five assumed coordinates were used to draw the plot. These two coordinates explained about 55.6% of the total variation of 18 cytotypes. The three clusters observed were reminiscent of the three apexes in the PCoA plot from the nuclear genome (Fig 1). No cytotypes were classified in intermediate positions as observed in the nuclear PCoA plot (Fig 1). Thus, the classified cytotypes are considered to be a good measure to confirm parentage and the combination of seed parent and pollen parent. Among them, three cytotypes (C01, C02 and C09) are grouped in cluster I, corresponding to lime, yuzu and *C*. *ichangensis*. Seven cytotypes (C04, C05, C06, C07, C08, C10 and C18) are grouped in cluster II, which corresponds to pummelo and lemon. Eight cytotypes (C03, C11, C12, C13, C14, C15, C16, and C17) are grouped in cluster III, which corresponds to mandarin. Although the PCoA analysis of nuclear markers placed lemon (*C*. *limon*) with lime and *C*. *ichangensis* (Fig 1), it is classified in cluster II, which consists of the pummelo cytotype. Cytotypes C01 (*C*. *ichangensis* type) and C09 (yuzu type) are classified close together in cluster I, corresponding to the proposed relationship between them that was mentioned by Swingle [11] and Tanaka [12]. In cluster I, polymorphisms between C01 and C09 were observed in four chloroplast DNA markers (CSS04, CSL09, ccmp2.2m and ccmp6.2), and polymorphisms between C02 (Mexican lime type) and C09 in six chloroplast DNA markers (CSL01, CSL09, ccmp2.2, ccmp6.2, ccmp7.2 and ccmp10.2). However, no polymorphism was observed in mitochondrial markers within cluster I (Table 11). Consequently, distributions of these three cytotypes in cluster I represent polymorphism of chloroplast markers.

Five varieties in cluster II (hyuganatsu, Ichang lemon, koji, Satsumakikoku and sweet orange) are placed in intermediate positions among the three apexes in the nuclear PCoA plot (Fig 1), but their corresponding cytotypes are classified to the same pummelo and lemon cluster (Fig 6). Of those grouped in cluster II, C05 (hyuganatsu type) and C10 (Ichang lemon type) show just one polymorphism in ccmp6.2, and map to the same position. All of the cytotypes grouped in cluster II show polymorphism for the chloroplast markers, but no polymorphism was observed for the mitochondrial markers (Table 11). Six varieties in cluster III (Cleopatra, kunenbo-B, limonia, tachibana, tachibana-C, and shiikuwasha Ogimikugani) are placed in intermediate positions among the three apexes in the nuclear PCoA plot (Fig 1), but they are grouped in the same mandarin cluster (Fig 6). The cytotype of kunenbo-A (A081) is C07 (sweet orange type) and it falls in cluster II with pummelo, but the cytotype of kunenbo-B (C11) is in cluster III. These inconsistencies suggest different origins of their cytoplasmic genomes. All of the eight cytotypes in cluster III harbor unique mitochondrial alleles for *rrn*5/*rrn*18-1 and *nad*7/1-2 of length 269 and 164, respectively. Accordingly, we conclude that the first coordinate (horizontal axis) corresponds to the polymorphism of mitochondrial markers, and the second coordinate (vertical axis) corresponds to the polymorphism of chloroplast markers.

**Fig 7:**
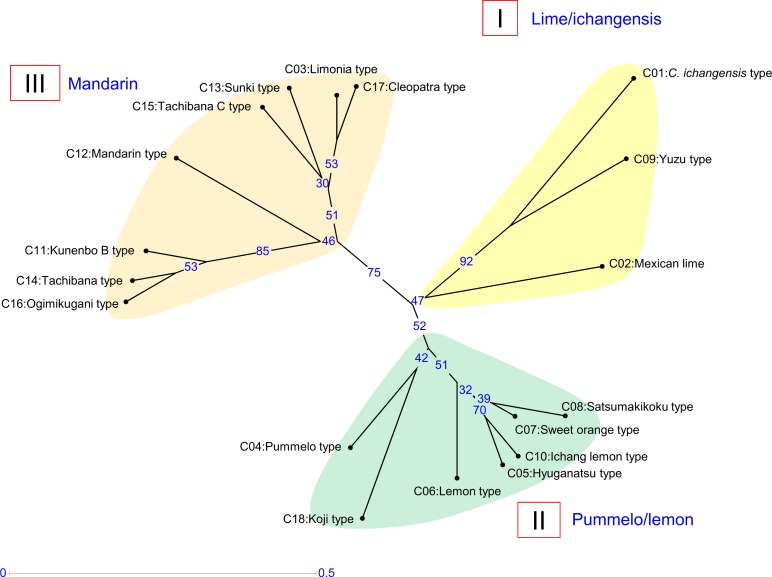
Estimated phylogenetic tree of the 18 representative organelle genomes observed in 101 citrus indigenous varieties. The tree was produced using the neighbor-joining method. The three main clades observed are indicated by different colors (I, II and III). Node labels show bootstrap support values.

Of the eight cytotypes that are grouped in cluster III, four cytotypes (C03, C13, C15 and C17) and three cytotypes (C11, C14 and C16) are placed at opposite ends of the cluster along the second coordinate, with C12 (mandarin type) in the center (Fig 6). Interestingly, two cytotypes of tachibana (C14: tachibana type and C15: tachibana C type) are positioned at opposite ends of cluster III (Fig 6). The group of three cytotypes (C11, C14 and C16) harbored the 374 and 310 alleles for chloroplast CSL01 and CSL09, respectively, but the remaining five cytotypes harbored different alleles. Thus, these differences are considered to separate these groups in cluster III. Furthermore, the group of three cytotypes (C11, C14 and C16) and C12 harbored the 253 allele at the *nad*2/4-3 marker for mitochondria, which was not observed in other cytotypes (Table 11), suggesting that the mitochondria of these four cytotypes could be derived from the same origin. The phylogenetic tree estimated using the neighbor-joining method demonstrates the same three clusters (Fig 7).

Consequently, clusters I and II were revealed to harbor the same mitochondrial genotypes, and their differences were due to polymorphism in chloroplast genotypes. In contrast, cluster III was distinguished from clusters I and II by a polymorphism in mitochondrial genotypes. The observed isolation of two groups in cluster III was caused by polymorphisms in CSL01 and CSL09, and the observation that the mandarin type (C12) was placed between these groups could contribute to understanding the evolution of this cytotype.

### Parentage analysis of parent–offspring triads in the indigenous varieties

Parentage was evaluated for all possible dyad combinations in the 101 indigenous varieties using the allele-sharing test with the 123 selected DNA markers confirmed to have Hardy–Weinberg proportions (Table 13). This test will succeed when a particular dyad shares at least one allele, but will fail whenever no allele is shared between them according to Mendel’s laws of inheritance [64]. The number of DNA markers not sharing any alleles was scored for each pairwise combination of the indigenous varieties using the allele-sharing test, and these scores are given in S10 Table. The test reveals that 74 varieties share all alleles with others, and 92 varieties share alleles with others when up to four mismatches are allowed (Table 13). Among those varieties, kaikoukan (A049), Kishu (A059), kunenbo-A (A081), sour orange (A141) and sweet orange (A162) were shown to share all alleles with more than five varieties without mismatch (S10 Table). Yuzu (A208) was shown to share alleles with 10 varieties when up to four mismatches were allowed.

**Table 13:** Summary of allele sharing test within the indigenous varieties for inferring parentage.

**Evaluated DNA markers**		123
**Examined indigenous varieties**		101
(Combinations examined by the allele sharing test)		10,201
**Inferred parent-offspring dyads**		
	No mismatch allowed	74
	(No shared alleles with others)	27
	Up to 4 mismatches allowed	92
	(No shared alleles with others)	9

Parent–offspring relationships were examined using the parentage test for all varieties that matched more than two other varieties. We allowed up to four mismatches among 123 DNA markers on the test in case of mutations or genotyping error (Table 13). The identities of seed parent and pollen parent were also deduced from their cytotypes (S9 Table). Any examined triads that disagreed on their parentage by more than five mismatches were rejected in this study. As an example, the parentage of ‘Fortune’ (B016) was inconsistent with the reported parentage (Clementine × dancy) [2], but the allele-sharing test proposed Clementine and Orland as the candidate parents with no mismatched DNA markers, and their cytotypes confirmed the true parentage with Clementine as seed parent and Orland as pollen parent (Table 14). In addition, the pollen parent of the hybrid variety ‘Haruka’ (B020), which was a selection of open pollinated hyuganatsu, was identified as natsudaidai. The cytotype of ‘Haruka’ agreed with the inferred parentage (Table 14).

**Table 14:** List of the inferred parentage in the indigenous varieties and orphan hybrid varieties.

No.	Offspring variety			Inferred seed parent			Inferred pollen parent			Scores			
	ID	Variety	CT	ID	Variety	CT^)^	ID	Variety	CT	MM	Matched(%)	LOD ^1)^score	*RCI* ^2)^
1	A001	Andoukan	C04	A049	Kaikoukan	C04	A059	Kishu	C12	0	100.0%	80.9	158.0
2	A006	Bergamot	C06	A085	Lemon	C06	A141	Sour orange	C06	4	96.7%	125.9	221.7
3	A009	Clementine	C12	A201	Willowleaf mandarin	C12	A162	Sweet orange	C07	0	100.0%	92.6	176.0
4	A014	Cravo	C12	A201	Willowleaf mandarin	C12	A162	Sweet orange	C07	0	100.0%	90.7	168.5
5	A019	Fukure mikan	C12	A059	Kishu	C12	A076	Koji	C18	0	100.0%	100.0	170.2
6	A027	Hanayu	C09	A208	Yuzu	C09	A172	Tachibana-A	C14	2	98.4%	173.3	251.8
7	A030	Henka mikan	C07	A081	Kunenbo-A	C07	A208	Yuzu	C09	2	98.4%	99.7	201.2
8	A044	Iyo	C04	A049	Kaikoukan	C04	A016	Dancy	C12	0	100.0%	92.7	163.6
9	A045	Jabara	C07	A081	Kunenbo-A	C07	A208	Yuzu	C09	3	97.6%	110.6	212.8
10	A047	Kabosu	C07	A081	Kunenbo-A	C07	A208	Yuzu	C09	1	99.2%	101.4	202.8
11	A052	Keraji	C07	A048	Kabuchi	C07	A081	Kunenbo-A	C07	0	100.0%	89.3	187.9
12	A073	Kizu	C07	A081	Kunenbo-A	C07	A208	Yuzu	C09	2	98.4%	102.9	203.0
13	A091	Mochiyu	C07	A081	Kunenbo-A	C07	A208	Yuzu	C09	2	98.4%	97.5	196.8
14	A100	Nidonari mikan	C12	A059	Kishu mandarin	C12	A141	Sour orange	C06	0	100.0%	88.7	177.0
15	A112	Sanbokan	C04	A049	Kaikoukan	C04	A059	Kishu	C12	0	100.0%	82.3	161.5
16	A125	Satsuma	C12	A059	Kishu	C12	A081	Kunenbo-A	C07	0	100.0%	75.3	162.2
17	A138	Sokitsu	C12	A059	Kishu	C12	A075	Kobeni mikan	C12	0	100.0%	127.1	178.6
18	A147	Suruga Yuko	C12	A059	Kishu	C12	A076	Koji	C18	0	100.0%	114.6	189.0
19	A186	Temple	C12	A201	Willowleaf mandarin	C12	A162	Sweet orange	C07	4	96.7%	81.9	160.4
20	A188	Tizon	C07	A162	Sweet orange	C07	A013	Cleopatra	C17	0	100.0%	88.3	162.0
21	A204	Yatsushiro	C07	A081	Kunenbo-A	C07	A059	Kishu	C12	1	99.2%	82.3	169.2
22	A207	Yuukunibu	C04	A001	Andoukan	C04	A112	Sanbokan	C04	1	99.2%	74.1	164.5
23	B016	Fortune	C12	A009	Clementine	C12	B045	Orland	C04	0	100.0%	98.9	174.7
24	B020	Haruka	C05	A036	Hyuganatsu	C05	A096	Natsudaidai	C04	0	100.0%	65.5	171.8

**CT**: Classes of organelle genotype (see Table 11), **MM**: Number of mismatched markers.

1) **LOD**: see the section ‘Stochastic evaluation of inferred parentage’

2) **RCI**: required cross trial index obtained using Eq 7.

**Fig 8:**
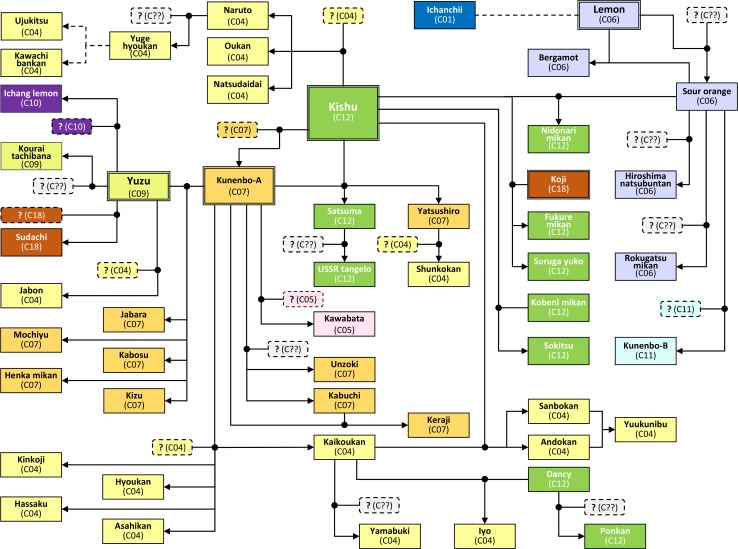
An inferred genealogy of citrus indigenous varieties, part 1. The plot shows the pedigrees of Kishu, yuzu, lemon, sour orange and their inferred offspring. Codes in parentheses represent individual cytotypes, and the same color represents the same cytotype. Dashed boxes indicate postulated parents. Double-lined boxes correspond to key varieties in this plot.

Consistent with their cytotypes, Satsuma (A125) was inferred to be an offspring of Kishu (A059) as the seed parent, and kunenbo-A (A081) as the pollen parent (Table 14, Fig 8). All of the genotypes obtained not only from the 123 certified DNA markers but also the 169 passed DNA markers supported the parentage. Parentage analysis further identified Yatsushiro (A204) as another offspring of the same parents (Kishu and kunenbo-A), but their cross combinations were opposite to each other. Satsuma and Yatsushiro are therefore recognized as siblings (Table 14, Fig 8).

**Fig 9:**
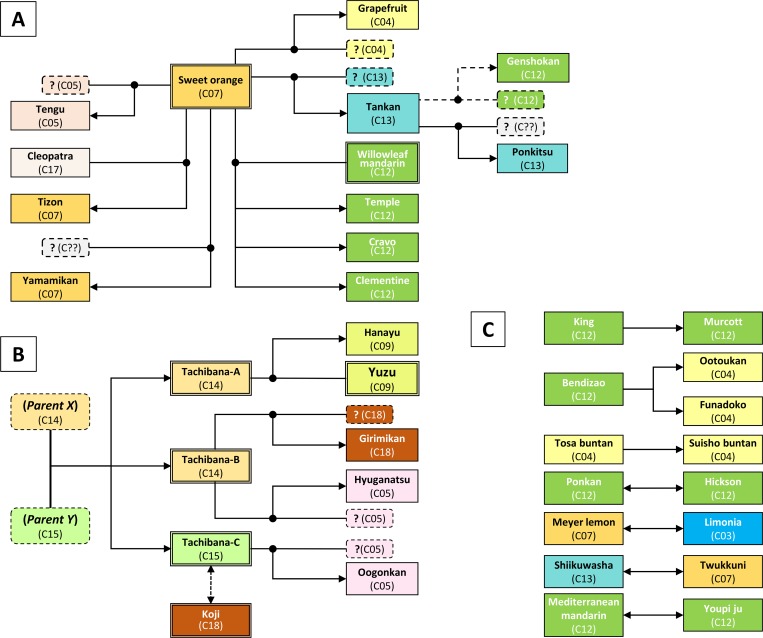
An inferred genealogy of citrus indigenous varieties, part 2. A: The pedigree plot of sweet orange and its inferred offspring. B: The pedigree plot of three tachibanas and their inferred offspring with postulated parents. C: Various proposed pedigrees. Their codes, colors and lines are as described for Fig 8.

In a similar fashion, Clementine was inferred to be an offspring of willowleaf mandarin × sweet orange as previously demonstrated [43,45]. Though Cravo (A014; Laranja Cravo) had been recognized as a variety of unknown origin [2], the parentage test proposed that it was an offspring of willowleaf mandarin × sweet orange. Furthermore, Temple (A186; *C*. *temple* Hort. ex Y. Tanaka) [2] was inferred to be another offspring of the same parents despite it showing four mismatches (Table 14, Fig 9A). Their cytotypes and the results of structure analysis supported these inferred parentages. Consequently, Clementine, Cravo and Temple are recognized as siblings from a willowleaf mandarin × sweet orange cross.

The allele-sharing test revealed close relationships of sour orange (A141) to six varieties: bergamot (A006), Hiroshimanatsubuntan (A033), kunenbo-B (A082), lemon (A085), nidonari mikan (A100), and rokugatsumikan (A111) (S10 Table). Among these varieties, nidonari mikan (*C*. *nidonari* Hort. ex Y. Tanaka) is an old mandarin variety of unknown origin, but it demonstrated no mismatch in any DNA marker with sour orange or Kishu (S10 Table). The parentage test inferred it to be an offspring of Kishu × sour orange with no mismatch, and their cytotypes proposed Kishu as the seed parent, and sour orange as the pollen parent. Likewise, the parentage test inferred bergamot (A006; *C*. *bergamina* Risso) to be an offspring of lemon (A085) × sour orange (Table 14). According to the cytotypes, bergamot (A006) is assumed to be a hybrid of lemon (A085) as the seed parent and sour orange (A141) as the pollen parent. Though four mismatches were observed in the score of bergamot, this is thought to result from residual genotyping error because lemon and its relatives were not provided for the initial verification of the DNA markers in sufficient numbers. Bergamot has been considered a natural hybrid of sour orange [2], and the inferred parentage agrees with the proposed one [45,47]. Very recently, Curk et al reported the identical combination for bergamot [47]. The parentage of the remaining four varieties (lemon, Hiroshimanatsubuntan, kunenbo-B and rokugatsumikan) will be examined in the next section.

Allele-sharing tests on Kishu revealed that at least 18 varieties could be kindred to it (S10 Table). Parentage tests on these inferred that both andoukan (A001) and sanbokan (A112) are offspring of kaikoukan (A049) as the seed parent and Kishu (A059) as the pollen parent without mismatches. Although one mismatch was observed, yuukunibu (A207) is inferred to be an offspring of these hybrid varieties (andoukan × sanbokan). Kaikoukan was inferred to be the seed parent of iyo (A044), crossed with dancy (A016) as the pollen parent. Consequently, both andoukan and sanbokan are revealed to be half-siblings of iyo. Sokitsu (A138) was inferred to be an offspring of Kishu × kobeni mikan (A075), but it is not possible to determine which is the seed parent and which is the pollen parent because of their identical cytotypes.

Five varieties (A030: henka mikan, A045: jabara, A047: kabosu, A073: kizu and A091: mochiyu) were inferred to be offspring of yuzu × kunenbo-A, though one to three mismatches were observed for them (Table 14). Likewise, hanayu (A027) was inferred to be a hybrid of yuzu as the seed parent and tachibana-A (A172) as the pollen parent with two mismatches. Given that control hybrids used to verify the DNA markers did not include sufficient numbers of yuzu or its relatives, the observed mismatches could be due to either unforeseen null alleles or mutations, or both.

Fukure mikan (A019) and Suruga yuko (A147) were thought to be mutant varieties of koji (A076) [112], but parentage analysis revealed that they are not mutants but offspring of Kishu × koji. Tanaka proposed a close relationship among koji, fukure mikan and tachibana [7] and this agrees with the inferred parentage. Tizon (A188; *C*. *papillaris* Blanco) [12] was inferred to be a hybrid of sweet orange × Cleopatra. Kabuchi (A048) shared all alleles with kunenbo-A (A081) and keraji (A052), and keraji also shared all alleles with kabuchi and kunenbo-A. The parentage test rejected kunenbo-A and keraji as the parents of kabuchi with 14 mismatches, but kunenbo-A and kabuchi were inferred to be the parents of keraji with no mismatch. Thus, kabuchi was inferred to be an offspring of kunenbo-A as seed parent and an unidentified variety, and keraji was inferred to be an offspring of kabuchi and kunenbo-A, but their combination was indeterminate. This inferred parentage suggests that keraji is a backcrossed offspring of kunenbo-A.

Despite these inferred parentages, most of the proposed parent–offspring combinations were rejected by significant discrepancies on the parentage test. Three varieties, Kawachi bankan (A051), ujukitsu (A197) and yuge hyoukan (A205) shared all alleles among them. Likewise, ujukitsu, Kishu (A059) and yuge hyoukan shared all alleles among them, and this was confirmed with Naruto (A095), Kishu and yuge hyoukan. These observed perfect matches suggested their parentage, but the parentage test rejected all combinations of them. We hypothesize that Naruto and ujukitsu are the offspring of Kishu with unknown parents. According to their cytotype, Kishu is thought to be their pollen parent. The unknown other parents of Naruto and ujukitsu should hold a pummelo-type cytotype. The result of structure analysis coincides with this hypothesis. In this fashion, yuge hyoukan was inferred to be the offspring of ujukitsu or Naruto and an unidentified variety with uncertain cytotype, but the parentage of Kawachi bankan remained uncertain. These assumed relationships are examined further using stochastic methods, as discussed in a later section.

Tankan has been considered a natural tangor [2,7], and the allele-sharing test revealed no mismatch to sweet orange, ponkitsu (A109) and genshokan (A022). Ponkitsu showed five and eight mismatches to genshokan and sweet orange, respectively. Likewise, genshokan showed 10 mismatches to sweet orange. Consequently, sweet orange and genshokan were proposed as the parents of tankan, but the parentage test rejected their parentage. Furthermore, the cytotypes of tankan, sweet orange and genshokan were sunki type (C13), sweet orange type (C07) and mandarin type (C12), respectively, and did not coincide with each other. Therefore, tankan is assumed to be an offspring of sweet orange and an unidentified variety with sunki-type cytotype. Ponkitsu is assumed to be an offspring of tankan since they share the same cytotype. The parentage of genshokan is unclear, but it could be an offspring of tankan.

Except for the inferred triads, kunenbo-A revealed no mismatch to asahikan, hassaku, hyoukan, kabuchi, kaikoukan, kawabata, kinkoji, Kishu and unzoki, and one mismatch to sweet orange. Because kunenbo-A and sweet orange share the same cytotype (C07; sweet orange type), all possible combinations of these were examined using the parentage test but rejected with 15–25 mismatches. Sweet orange shared the same cytotype with kunenbo-A, but showed allele mismatches when evaluated with the genotype data obtained from 169 certified DNA markers. None of the other indigenous varieties that have the sweet orange-type cytotype (Table 12) shared significant number of alleles with kunenbo-A, and they were thus rejected from the parentage test. In contrast, kunenbo-A did not show any mismatch to Kishu when evaluated with all of the DNA markers (data not shown). The deduced proportions of the three basic taxa in Kishu were 0.2%, 0.5% and 99.3%, and those of kunenbo-A were 35.2%, 0.4% and 64.2% for pummelo, citron and mandarin, respectively (S8 Table). The proportion of mandarin genome would decrease when Kishu is crossed with an unknown variety, as observed in kunenbo-A. On the contrary, it seems difficult to purge the entire pummelo genome portion in kunenbo-A by a single crossing event with an unknown variety to result in Kishu that has a minimum of pummelo genome. Consequently, kunenbo-A is thought to be an offspring of Kishu as pollen parent crossed with an unidentified variety that hold sweet orange-type cytotype as seed parent.

Suisho buntan (A145) shared all alleles with Tosa buntan (A191), and shared all except for one mismatch with Hirado buntan (A032). The parentage test rejected both of these as parents of suisho buntan with seven mismatches (data not shown). Suisho buntan has been selected from open pollinated pummelo, and banoukan and Tosa buntan were proposed to be the parents [112]. Although banoukan was not examined in this study, the allele-sharing test supports the parentage of Tosa buntan.

The inferred parentages agreed well with the population compositions deduced by structure analysis with *K* = 3 (Fig 4, S8 Table). The proportions of the assumed basic taxa coincided for those triads (Table 14). For example, the deduced proportions of the three basic taxa (P1: pummelo, P2: citron and P3: mandarin) in tizon (A188) were 16.6%, 1.0% and 82.4%, respectively (S8 Table), and these are close to the proportions (18.2%, 2.3% and 79.6%) estimated from the inferred parents sweet orange (36.1%, 0.5% and 63.4%) and Cleopatra (0.3%, 4.0% and 95.7%). The proposed proportions in sokitsu (A138) also agreed well with the inferred parents (Kishu × kobeni mikan). Meanwhile, measurable discrepancies in the proportions of the populations were observed occasionally. The estimated proportions in Kishu (0.2%, 0.5% and 99.3% for the three genomes) × kunenbo-A (35.2%, 0.4% and 64.2%) were 17.7%, 0.5% and 81.8%; however, the corresponding proportions estimated from the inferred offspring were 28.9%, 0.5% and 70.6% for Satsuma, and 24.3%, 0.5% and 75.2% for Yatsushiro. Interestingly, the deduced proportions in these offspring were not identical but fluctuated in these siblings. Similar discrepancies and fluctuation were also observed among other inferred full or half-siblings. Iyo was one of two inferred offspring of kaikoukan and Kishu, and the deduced proportions in iyo (40.9%, 0.3% and 58.8%) were close to those in kaikoukan × dancy (37.9%, 0.4% and 61.8%). In contrast, andoukan (49.6%, 0.4% and 50.0%) and sanbokan (48.6%, 0.7% and 50.7%) differed from the expected values of kaikoukan × Kishu (37.9%, 0.4% and 61.7%). Likewise, four offspring of kunenbo-A × yuzu (henka mikan, jabara, kabosu and mochiyu), two offspring of willowleaf mandarin × sweet orange (Clementine and Cravo), two siblings of Kishu × koji (fukure mikan and Suruga yuko) were consistent in their genome composition with those of the inferred parents but showed fluctuation between siblings. The deduced proportion of the pummelo genome in the four siblings of kunenbo-A × yuzu fluctuated widely, from 3.7% (henka mikan) to 20.5% (jabara), and the proportions of the two other genomes also fluctuated in a coordinated manner. Similar discrepancies were also observed in bergamot and hanayu, and were still evident at *K* = 4 (S2 Fig). These observed variations suggest that two alleles at particular heterozygous loci would have different effects on the estimation of the proportions of basic taxa, or that the lack of ‘pure’ citron or papeda varieties in this study might lead to underestimation of their contribution in the indigenous varieties.

### Three types of tachibana and their relatives

We evaluated the mutual relationships between three types of tachibanas. The shared allele frequencies estimated using the allele-sharing test (S10 Table) were 93.5% (tachibana-A–B), 97.6% (tachibana-A–C) and 91.9% (tachibana-B–C). The observed shared allele frequencies were higher than those between the three types of tachibanas and other varieties; 66.2% (tachibana-A), 70.7% (tachibana-B), and 67.9% (tachibana-C). Hirai and colleagues reported similar genetic variation among wild tachibana collections using three isozymes [20]. The deduced proportions for the genomes of the three basic taxa for these types were similar to one another (Fig 4), and also suggested their hybrid origin as mandarin × citron. The cytotype of tachibana-A and B was the same (C14; tachibana type), but it was different in tachibana-C (C15; tachibana-C type). These observations suggest that these three types of tachibana might be siblings. Accordingly, a model was proposed in which these three types of tachibanas are offspring of two ancestors, one harboring tachibana-type cytotype (C14) and the other harboring tachibana-C type (C15) (Fig 9B). Because these cytotypes (C14 and C15) were not found in other varieties, those hypothetical ancestors may have been lost.

The allele-sharing test suggested that hanayu is an offspring of yuzu × tachibana-A as described above. Similarly, girimikan (A023) and hyuganatsu (A036) were proposed to be offspring of tachibana-B (A175), but their cytotypes were different to each other. Oogonkan (A101) was proposed to be the offspring of tachibana-C (Table 15). Although the seed parents of these three varieties were unidentified, cytotypes of hyuganatsu and oogonkan were identical (C05; hyuganatsu). Iwamasa has pointed out the close relationship among hyuganatsu, oogonkan and yuzu [112], and their unidentified seed parents could be siblings of yuzu. On the other hand, no previous studies have proposed the involvement of any of the tachibanas as the parents of hanayu, girimikan, hyuganatsu, or oogonkan.

**Table 15:** Inferred parent-to-child combinations in the indigenous varieties.

	Offspring variety			Inferred seed parent			Inferred pollen parent			Scores				
#	ID	Variety	CT	ID	Variety *^)^	CT	ID	Variety *^)^	CT	MM	Matched (%)	LOD ^1)^ score	*RCI* ^2)^	*SPP* ^3)^
1	A003	Asahikan	C04		*(pummelo type)*	C04	A081	Kunenbo-A	C07	0	100.0	6.6	173.4	41.2
2	A021	Funadoko	C04		*(pummelo type)*	C04	A005	Bendizao	C12	0	100.0	17.7	164.1	47.2
3	A023	Girimikan	C18		*(Koji type)*	C18	A175	Tachibana-B	C14	1	99.2	61.8	186.3	53.1
4	A024	Grapefruit	C04		*(pummelo type)*	C04	A162	Sweet orange	C07	0	100.0	14.5	177.9	42.0
5	A028	Hassaku	C04		*(pummelo type)*	C04	A081	Kunenbo-A	C07	0	100.0	16.2	176.6	42.6
6	A031	Hickson	C12	A107	Ponkan	C12		*(Unidentified)*		0	100.0	50.9	179.6	51.7
7	A033	Hiroshimanatsubuntan	C06	A141	Sour orange	C06		*(Unidentified)*		0	100.0	41.7	187.3	47.6
8	A035	Hyoukan	C04		*(pummelo type)*	C04	A081	Kunenbo-A	C07	0	100.0	4.0	169.9	42.1
9	A036	Hyuganatsu	C05		*(Hyuganatsu type)*	C05	A175	Tachibana-B	C14	0	100.0	34.0	188.4	44.5
10	A041	Ichanchii (*C*.*ichangensis*)	C01		*(C*. *ichangensis type)*	C01	A085	Lemon	C06	1	99.2	52.9	298.4	35.5
	A085	Lemon	C06		*(lemon type)*	C06	A041	Ichanchii (*C*.*ichangensis*)	C01	1	99.2	52.9	278.5	37.3
11	A042	Ichang lemon	C10		*(Ichang lemon type)*	C10	A208	Yuzu	C09	1	99.2	59.1	255.8	38.3
12	A046	Jabon	C04		*(pummelo type)*	C04	A208	Yuzu	C09	0	100.0	40.3	194.8	43.9
13	A048	Kabuchi	C07	A081	Kunenbo-A	C07		*(Unidentified)*		0	100.0	7.6	189.8	42.6
14	A049	Kaikoukan	C04		*(pummelo type)*	C04	A081	Kunenbo-A	C07	0	100.0	16.5	192.3	38.5
15	A050	Kawabata	C05		*(Hyuganatsu type)*	C05	A081	Kunenbo-A	C07	0	100.0	18.5	177.6	44.8
16	A051	Kawachi bankan	C04	A197	Ujukitsu	C04		*(Unidentified)*		0	100.0	17.0	186.4	41.1
	A051	Kawachi bankan	C04	A205	Yuge hyoukan	C04		*(Unidentified)*		0	100.0	34.9	186.4	45.6
17	A076	Koji	C18		*(Koji type)*	C18	A174	Tachibana-C	C15	2	98.4	36.8	184.8	47.9
18	A055	Kinkoji	C04		*(pummelo type)*	C04	A081	Kunenbo-A	C07	0	100.0	19.0	187.4	41.0
19	A079	Kourai Tachibana	C09	A208	Yuzu	C09		*(Unidentified)*		2	98.4	72.5	231.6	47.5
20	A081	Kunenbo-A	C07		*(Sweet orange type)*	C07	A059	Kishu	C12	0	100.0	3.3	152.7	45.9
21	A082	Kunenbo-B	C11		*(Kunenbo_B type)*	C11	A141	Sour orange	C06	0	100.0	15.5	188.3	42.4
22	A085	Lemon	C06	A141	Sour orange	C06		*(Unidentified)*		0	100.0	13.4	280.8	31.8
	A141	Sour orange	C06	A085	Lemon	C06		*(Unidentified)*		0	100.0	13.4	180.6	42.2
23	A090	Meyer lemon	C07		*(sweet orange type)*	C07	A087	Limonia	C03	1	99.2	113.1	266.7	43.5
24	A092	Murcott	C12	A054	King	C12		*(Unidentified)*		0	100.0	40.8	177.0	50.8
25	A098	Natsudaidai	C04		*(pummelo type)*	C04	A059	Kishu	C12	1	99.2	-1.4	163.5	42.7
26	A095	Naruto	C04		*(pummelo type)*	C04	A059	Kishu	C12	0	100.0	1.3	169.9	40.0
27	A101	Oogonkan	C05		*(Hyuganatsu type)*	C05	A174	Tachibana-C	C15	0	100.0	34.6	180.9	47.7
28	A103	Ootoukan	C04		*(pummelo type)*	C04	A005	Bendizao	C12	0	100.0	22.0	178.1	43.7
29	A104	Oukan	C04		*(pummelo type)*	C04	A059	Kishu	C12	0	100.0	11.1	171.3	42.8
30	A107	Ponkan	C12	A016	Dancy	C12		*(Unidentified)*		1	99.2	63.6	179.4	54.7
31	A109	Ponkitsu	C13	A183	Tankan	C13		*(Unidentified)*		0	100.0	61.4	190.5	52.1
32	A111	Rokugatsumikan	C06	A141	Sour orange	C06		*(Unidentified)*		0	100.0	17.0	208.5	40.4
33	A135	Shiikuwasha	C13		*(Sunki type)*	C13	A192	Twukkuni	C07	1	99.2	63.6	196.0	52.0
34	A137	Shunkokan	C04		*(pummelo type)*	C04	A204	Yatsushiro	C07	0	100.0	19.8	167.6	47.8
35	A144	Sudachi	C18		*(Koji type)*	C18	A208	Yuzu	C09	3	97.6	67.2	210.1	47.8
36	A145	Suisho buntan	C04	A191	Tosa buntan	C04		*(Unidentified)*		0	100.0	73.3	237.7	42.9
37	A183	Tankan	C13		*(Sunki type)*	C13	A162	Sweet orange	C07	0	100.0	28.4	188.8	44.6
38	A187	Tengu	C05		*(Hyuganatsu type)*	C05	A162	Sweet orange	C07	0	100.0	14.5	167.8	43.2
39	A197	Ujukitsu	C04		(pummelo type)	C04	A059	Kishu	C12	0	100.0	9.3	161.8	46.4
	A197	Ujukitsu	C04	A205	Yuge hyoukan	C04		*(Unidentified)*		0	100.0	22.1	161.8	49.2
40	A198	Unzoki	C07	A081	Kunenbo-A	C07		*(Unidentified)*		0	100.0	6.3	188.8	42.0
41	A199	USSR Tangelo	C12	A125	Satsuma	C12		*(Unidentified)*		0	100.0	9.1	177.0	42.0
42	A202	Yamabuki	C04	A049	Kaikoukan	C04		*(Unidentified)*		0	100.0	38.3	166.3	50.2
43	A203	Yamamikan	C07	A162	Sweet orange	C07		*(Unidentified)*		0	100.0	15.3	181.0	42.4
44	A205	Yuge hyoukan	C04	A197	Ujukitsu	C04		*(Unidentified)*		0	100.0	22.1	179.0	44.4
	A205	Yuge hyoukan	C04	A095	Naruto	C04		*(Unidentified)*		0	100.0	14.7	179.0	43.5
45	A206	Youpi ju (Yuhikitsu)	C12	A200	Willowleaf mandarin	C12		*(Unidentified)*		0	100.0	52.7	194.3	54.4

**CT**: Classes of organelle genotype (see Table 9), **MM**: Number of mismatched markers.

1) **LOD**: see the section ‘Stochastic evaluation of inferred parentage’.

2) ***RCI***: required cross trial index obtained using Eq 7.

3) ***SPP***: Single parent–offspring probability obtained using Eq 8.

### Parentage analysis of parent–offspring dyads in the indigenous varieties

The inferred triads were excluded from the proposed dyads, and the remaining dyads were further evaluated (Table 15). Unlike the parentage test, the allele-sharing test does not predict which variety is the parent and which is the offspring. Accordingly, parentage was estimated from cytotypes, asymmetry of parentage, the result of structure analysis, and past literatures.

The allele-sharing test found a close relationship of Kishu to oukan (A104) and natsudaidai (A098) (S10 Table). Oukan (*C*. *suavissima* Hort. ex Tanaka) is an old mandarin variety from China [7]. From the evidence obtained, it was suggested to be a hybrid of *C*. *maxima* and Kishu. Natsudaidai (*C*. *natsudaidai* Hayata) was a chance seedling in Yamaguchi prefecture [2,11], and the evidence suggests that this arose from hybridization between pummelo and Kishu.

Murcott (A092) and King (A054) shared all alleles and an identical cytotype (S9 and S10 Tables). This suggests that they could be a parent–offspring pair, but their deduced proportions of the ancestral populations were also similar (S8 Table), and it seemed difficult to determine which one would be the parent. According to Hodgson [2], Murcott was recognized as a tangor of unknown origin resulting from the breeding program of the USDA. Because King was frequently used in the USDA citrus breeding program [2], it is likely that Murcott was a selection of King. Nicolosi and colleagues also reported similarity between them [33]. Consequently, we postulate that Murcott is an offspring of King (Table 15).

Grapefruit has been regarded as a natural hybrid of sweet orange and pummelo [109,113], and recent molecular work supports this [34,43,45]. The allele-sharing test coincided with the current consensus on grapefruit, with no mismatches between sweet orange and grapefruit. The population structure analysis suggests the involvement of pummelo as the other parent, agreeing with the cytotype of grapefruit that is not the same as sweet orange but rather the pummelo type. Accordingly, grapefruit is assumed to be an offspring of sweet orange as the pollen parent with an unidentified variety harboring the pummelo cytotype. In addition, tengu (A187) and yamamikan (A203) are suggested their parentages with sweet orange (S11 Table). Because the cytotype of tengu (*C*. *tengu* Hort. ex Tanaka) [7,12] was hyuganatsu type (C05), sweet orange was assumed to be the pollen parent of tengu. Although the pollen parent of yamamikan (*C*. *intermedia* hort. ex Tanaka) is unidentified, the deduced genome proportions of the basic taxa suggest that yamamikan is a hybrid of mandarin and pummelo (Fig 4), and this agrees with the hypothesis of Tanaka [7]. Both tengu and yamamikan showed just one mismatch between them under the allele-sharing test, supporting the hypothesis that they are siblings of sweet orange.

Three mandarin varieties (Mediterranean mandarin, willowleaf mandarin and youpiju) are not mutant selections, but Mediterranean mandarin and willowleaf mandarin share 95.1% of alleles, suggesting a common ancestral origin. Mediterranean mandarin and youpiju shared all alleles including the excluded markers. They are old mandarin varieties of uncertain origin, and it has been suggested that they are kindred varieties. Furthermore, Hickson (A031) and ponkan (A107) shared all alleles, and these two varieties also shared significant numbers of alleles with willowleaf mandarin (S10 Table). Hickson was found as a sporting limb on ‘Ellendate’ tangor [2,114], but the deduced genome structure suggests that Hickson is almost a mandarin (Fig 4). These varieties share the same cytotype (C12; mandarin type) and their genome structures are quite similar to each other. Therefore, their parentage is indeterminate. We propose an alternative hypothesis that they are siblings.

Bendizao (A005) shared all alleles with two Japanese local varieties ootoukan (A103) and funadoko (A021), but their cytotypes were different to that of bendizao. Since the probability of selecting an identical genotype from two different varieties is negligible, the observed asymmetry confirms that both ootoukan and funadoko are the offspring of bendizao as pollen parent and an unidentified variety with pummelo-type organelle genomes. The deduced genome structure of basic taxa support their proposed kinship.

Similar asymmetric relationships were also found in sour orange and four varieties (A033: Hiroshimanatsubuntan, A082: kunenbo-B, A085: lemon, A111: rokugatsumikan) by the allele-sharing test (S10 Table). Since sour orange was inferred to be the parent of nidonari mikan and bergamot, those four varieties were also assumed to be either parents or offspring of sour orange. These varieties except for kunenbo-B share the same cytotype (C06; lemon type), but the allele-sharing test revealed that Hiroshimanatsubuntan, kunenbo-B and rokugatsumikan have 18, 20 and 15 mismatches with lemon, respectively (S10 Table). On the basis of these scores, sour orange is assumed to be an offspring of lemon, and Hiroshimanatsubuntan, kunenbo-B and rokugatsumikan are assumed to be offspring of sour orange. Their cytotypes suggest that sour orange is the seed parent of Hiroshimanatsubuntan and rokugatsumikan, but the pollen parent of kunenbo-B. The deduced genome structure of basic taxa agrees well with this inferred parentage. The inferred parentage also revealed that the two types of kunenbo (kunenbo-A and kunenbo-B) are different in origin.

It is interesting that ichanchii (*C*. *ichangensis* Swingle) shares all alleles with lemon but their cytotypes are different. Swingle considered it a unique variety related to papeda, and regarded yuzu as a chance seedling of *C*. *ichangensis* [11]. However, the allele-sharing test clearly refutes this proposal, with 31 out of 123 DNA markers not shared between yuzu and *C*. *ichangensis*. Because the cytotype of *C*. *ichangensis* was unique, but the lemon-type cytotype was found in 13 varieties (S9 Table), *C*. *ichangensis* could be an offspring of lemon with an unidentified seed parent whose cytotype should be identical to that of *C*. *ichangensis*. The position of *C*. *ichangensis* on the PCoA plot of the nuclear genome is close to lemon (Fig 1), as supported by the allele-sharing test. However, their cytotypes are different in eight out of 13 organelle DNA markers, and they are far apart in the organellar PCoA plot (Fig 6). These observations hypothesized that *C*. *ichangensis* could an offspring of an unidentified papeda × lemon, and yuzu might also be an offspring of this unidentified papeda.

As observed in the parentage analysis of the proposed triads, the allele-sharing test revealed a possible parent–offspring relationship of yuzu with Ichang lemon (A042), Kourai tachibana (A079), jabon (A046) and sudachi (A144). Swingle considered Ichang lemon (*C*. *wilsonii* Tanaka) to be a hybrid of *C*. *ichangensis* and *C*. *maxima* [11]. In contrast, Tanaka regarded it as an indigenous variety related to yuzu, and classified both *C*. *ichangensis* and *C*. *wilsonii* to subgenus *Eucitrus* [12]. Their inferred parentage in this study confirm that *C*. *wilsonii* is an offspring of yuzu as Tanaka stated [12]. However, there is no evidence to suggest kinship of *C*. *ichangensis* and yuzu, and direct parentage of *C*. *ichangensis* and *C*. *wilsonii* are consequently refuted. Their cytotypes also suggest no direct kinship between them (Table 15, Fig 8). Kourai tachibana (A079) was found in Yamaguchi prefecture [7] and initially classified as *C*. *tachibana* Tanaka, but later reclassified to *C*. *nippokoreana* Tanaka [7,12]. Although the allele-sharing test identified two mismatches between Kourai tachibana and yuzu (S10 Table), these mismatches were considered to be caused by unidentified genotyping error. They share the same cytotype (C09; yuzu type), suggesting that yuzu would be the seed parent of Kourai tachibana (S9 Table). The allele-sharing test did not identify the candidate pollen parent of Kourai tachibana but demonstrated fewer mismatches with the three types of tachibanas (11, 12 and 8 mismatches with tachibana-A, B and C, respectively) than with other varieties. The three types of tachibana and their proposed sibships suggest that there might be another sibling of tachibana, and it could be the pollen parent of Kourai tachibana.

The inferred relationships agreed that sudachi (*C*. *sudachi* Hort. ex Shirai) is a hybrid of yuzu [7]. The cytotype of sudachi was koji-type (C18), but neither koji nor any other variety with koji-type organelle genome was assumed to be the seed parent of sudachi. Jabon (A046) is an indigenous variety of unknown origin found in Hiroshima prefecture, Japan. ‘Jabon’ is a Japanese synonym of pummelo [7,12], but the morphological features of jabon are not reminiscent of a typical pummelo but suggest a hybrid of pummelo [115]. The inferred hybrid combination coincides with the observed features. Furthermore, koji is another indigenous variety in Japan and its cytotype (C18; koji type) is unique among the evaluated varieties. However, it shares all alleles except for two mismatches with tachibana-C (Table 15). The deduced genomic proportions of the basic taxa suggest that koji is a hybrid of mandarin (Fig 4). Furthermore, the cytotype of sudachi is identical to that of koji. These observations might imply that the unidentified parents of tachibana-C, koji and sudachi could be identical or very close to each other.

USSR tangelo (A199) is a germplasm collection of NIFTS of unknown origin, but it is recognized as a hybrid of Satsuma × pummelo [116]. The allele-sharing test confirmed it as an offspring of Satsuma as the seed parent (S10 Table). Its pollen parent was not identified but structure analysis suggests introgression of the pummelo genome (Fig 4). This is just one inferred offspring of Satsuma in the evaluated varieties. Satsuma possesses strong and stable male sterility, parthenocarpy, and apomixes [117], and these traits could make it difficult to obtain offspring of Satsuma. Yatsushiro (A204) was inferred to be an offspring of kunenbo-A × Kishu, and it was assumed to be the pollen parent of shunkokan (A137). Because the cytotype of shunkokan is pummelo type (C04), kunenbo-A is inferred to be its pollen grandparent. Shunkokan (*C*. *shunkokan* hort. ex Tanaka) was found in Wakayama prefecture in Japan, and Tanaka mentioned its resemblance to kunenbo and pummelo [7].

Despite these assumed parentages, several dyads remain uncertain. Dancy (A016) shares all alleles with ponkan (A107). Likewise, limonia (A087) and Meyer lemon (A090), and also shiikuwasha (A135) and the kunenbo variety twukkuni (A192) share all alleles except for one mismatch (S10 Table).

Thirty-eight of the 45 selected dyads revealed no mismatches between them. A single mismatch was observed in seven dyads, two and three mismatches were observed in two and one variety, respectively (Table 15). As observed in the parentage analysis of triads, kunenbo-A, sweet orange, sour orange, Kishu, yuzu, lemon and bendizao were found in eight, four, three, four, four, two and two dyads, respectively (Table 15). Four types of kunenbo (kunenbo-A, kunenbo-B, twukkuni and King) were found in these dyads. According to the score of the allele-sharing test and their cytotypes, seven varieties (asahikan, hassaku, hyoukan, kaikoukan, kawabata, kinkoji and unzoki) were assumed to be offspring of kunenbo-A and various unidentified varieties.

### Stochastic evaluation of inferred parentage

While the allele-sharing test and the parentage test, coupled with the cytotypes, are an excellent approach to infer the parentage of uncertain varieties, these tests do not estimate the probability of the inferred parentage in the citrus population. In addition, these tests are susceptible to genotyping error or mutations, and could fail to estimate the correct combination. Although using the parentage test with known hybrid varieties or strains in this study eliminated all of the suspicious DNA markers, this does not guarantee the genotype to be perfect. As can be observed in Table 4, mutation is occasionally detected within mutant lines. Accordingly, the inferred parentage of triads or dyads was further examined by stochastic evaluation using a likelihood ratio approach. The likelihood ratio analysis is preferred over the parentage test because it can provide a basis for estimating the reliability of the inferred parentage. This approach has been used widely in forensic genetics in combination with Bayes’ theorem for missing person identification, paternity examination and kinship testing [59,60], and also in the field genetics [62–64]. A likelihood ratio represents the relative odds of two alternative hypotheses, and it has the advantage that it avoids postulating a posterior probability for the hypothesis. However, the likelihood ratio approach is implicitly premised on the minimum occurrence of relatives (full sib or half sib) in the given population [63]. Furthermore, it is known that this score is affected by the allele frequency in the population [60,118]. As observed in the previous section, a significant number of the indigenous varieties are thought to share kinship relations. Such a strained family structure might alter the LOD score due to uneven allele frequencies in populations.

Hence, we first evaluated the behavior of the LOD score with known hybrid triads (Table 3) using Eq 4. Their LOD scores show a wide range, from 69.3 (sweet spring) to 210.0 (benimadoka) (Table 3). In the case of the identification of a suspect from stains or remains, a likelihood ratio of 1,000 to 10,000 obtained from 10 to 15 (typically 13) STR markers is considered strong support for the prosecution hypothesis [60,118]. These values correspond to LOD scores of 6.9 to 9.2. Thus, the LOD scores of the known hybrid varieties obtained with 123 DNA markers were high enough to confirm their parentage (Table 3). Although all DNA markers used for the evaluation were confirmed to hold Hardy–Weinberg equilibrium, the indigenous varieties used in this study have been revealed to result from frequent and repeated crosses between several key varieties. Such complex population structure could unbalance the genotype frequencies of particular varieties, and could result in changes in their LOD scores. We therefore evaluated the whole genotype frequency of individuals in a population with a new score ‘required cross trial index’ (*RCI*). The *RCI* is a simple measure to estimate how many cross trials would be required to obtain a particular individual from the proposed population. This is a unitless logarithmic natural value, and observed differences in *RCI* value depend on allele frequency and abundance of sibs in the population. Higher *RCI* values mean there is less of an opportunity to select such an individual because of the lower allele frequency in the population of indigenous varieties, and *vice versa*. The observed *RCI* values of the known hybrid varieties range from 148.8 (kara mandarin) to 257.1 (Hayasaki) (Table 3). The structure analysis (Fig 4) suggests that these variations correlate with the less frequent occurrence of the pummelo genome, and the abundant occurrence of the mandarin genome within the indigenous varieties. On that premise, their *LOD* scores and *RCI* values show a high regression coefficient (*r*^2^ = 0.821), and the observed variations found in the *LOD* score were accordingly considered mostly due to allele abundance in the indigenous variety population. A similar influence of related individuals on frequency in the population was found in Marshall et al [62]. The estimated *RCI* values for the indigenous varieties showed a nonuniform distribution (S3 Fig) and the estimated *p*-value by the Shapiro–Wilk test was 2.07 × 10^−10^, supporting the hypothesis. Despite these constraints, the likelihood ratio analysis was considered valuable for the evaluation of confidence in the assumed triads even when applied to a small and structured population because it was avoidable by evaluating them with a sufficient number of DNA markers.

The LOD scores of the inferred parentage of indigenous varieties range from 75.3 (Satsuma) to 127.1 (sokitsu) (Table 14), and these values are comparable to those observed in the known hybrid varieties (Table 3). Therefore, we consider them sufficiently high enough to confirm the inferred combination. The observed LOD scores show a correlation to their *RCI* values (*r*^2^ = 0.711), as observed in the known hybrid varieties (Table 3). The observed LOD score of Satsuma was lower than others but the *RCI* value was proportionately low (Table 14). The inferred parents of Satsuma (Kishu and kunenbo-A) were also found in other parentage frequently as indicated by their low *RCI* values (161.5 for Kishu and 156.4 for kunenbo-A). This evidence confirms that frequent occurrence of sibship in the population depresses the LOD score of Satsuma. The LOD score of andoukan (80.9) was also considered to be depressed for similar reasons on the basis of its *RCI* value.

Likelihood ratio estimation of single parent and offspring dyads using Eq 5 was less informative than estimates from parent and offspring triads due to the lack of information of the second parent [65], resulting in lower LOD scores (Table 15). The observed LOD scores of most inferred single parent–offspring dyads in the indigenous varieties confirmed their relationships, but with a wide range from -1.4 (natsudaidai) to 113.1 (Meyer lemon) (Table 15). The likelihood ratio and LOD score were more susceptible to family structure than those observed in the triads [50]. The *RCI* values also showed large variation from 152.7 (kunenbo-A) to 298.4 (ichanchii) (Table 15). Meagher also pointed out a similar dependence although he evaluated the likelihood ratio in a different manner [61]. Because of these large variations, these measures were considered to be affected severely by the population structure of the indigenous varieties and not reliable for examining inferred parentage. Therefore, we verified the validity of the inferred dyads with another measure, ‘single parent–offspring probability’ (*SPP*). The *SPP* was a cumulative probability of two particular individuals being a single parent–offspring dyad, obtained from their transition probability (Eq 8). This score depends on the allele frequencies and combination of the single parent and offspring, but is less susceptible to family structure than the likelihood ratio. The score will increase according to the number of DNA markers for the analysis or the use of highly polymorphic DNA markers. In the estimation of the inferred single parent–offspring dyads, their obtained *SPP* values ranged from 35.5 to 54.7, and no large variation was observed (Table 15). The *SPP* values were comparable to those obtained from the known hybrid varieties (Table 3). Consequently, the inferred parentages were considered to be correct sufficiently.

The inferred parentage of several dyads was further examined with these scores. Direct parentage between ichanchii (*C*. *ichangensis*; A041) and lemon (A085) was suggested by the allele-sharing test, but their LOD scores were identical when either of them was assumed to be the parent and the other to the offspring. The *SPP* scores of these combinations were close, but slightly higher when ichanchii was assumed to be the parent of lemon. Structure analysis suggested a hybrid origin for ichanchii (Fig 4), they were insufficient to conclude the parentage between ichanchii and lemon (Table 15). The allele-sharing test suggested close kinship among Kawachi bankan (A051), yuge hyoukan (A205), ujukitsu (A197), Naruto (A095) and Kishu (A059), but their parentage relationships were not obvious. LOD and SPP scores suggested that Naruto was an offspring of Kishu with another unidentified parent harboring the pummelo-type cytotype (C04), and yuge hyoukan was inferred to be an offspring of Naruto and an unidentified parent. The parentage of ujukitsu and Kawachi bankan was not obvious, but they were assumed to be offspring of yuge hyoukan on the basis of their *SPP* scores (Fig 8). The possible parentage relationships between lemon and sour orange showed identical LOD scores, but the *RCI* score was higher when lemon was assumed to be the offspring of sour orange, and SPP score was higher when sour orange was assumed to be the offspring of lemon (Table 15). The result of structure analysis suggested admixture of the three basic taxa in sour orange, while lemon derived mostly from a single taxon. The PCoA analysis placed lemon close to one of the basic taxa; however, sour orange was placed in the middle of the three taxa. With this evidence, we infer that sour orange arose from a hybridization of lemon with an unidentified male variety.

## Discussion

This study intended to infer the parentage of indigenous citrus varieties by the allele sharing test, parentage test, and likelihood ratio analysis. The identity test was used to identify mutant strains or synonymous varieties, and 101 representative varieties were selected. These selected representatives are valid as a core collection of citrus varieties. Similar approaches to infer parentage with DNA marker analysis have also been reported in pine [119], grape [120], and apple [121]. Genotypes of chloroplast and mitochondrial genomes were evaluated to estimate the combination of seed parent and pollen parent. Very recently, Curk et al revealed the parentage of lime, lemon and sour orange according to nuclear and organelle genome analysis [47]. However, this only revealed their parentage of a limited number of varieties, and the parentage of many indigenous varieties remained uncertain.

The allele-sharing test recognized the correct parentage of ‘Fortune’ (Table 14). The test also identified the unidentified parental variety of ‘Haruka’ (Table 14). Genotyping analysis of chloroplast and mitochondrial genomes enabled fine classification of the cytoplasmic genotype (cytotype) into 18 categories, and confirmed the inferred parentage of ‘Fortune’ and ‘Haruka’. Thus, the allele-sharing test with DNA markers, after eliminating erroneous ones by parentage test with 122 known hybrid triads (59 hybrid varieties and 63 selected strains), was confirmed to be a valid approach to infer parentage as described by Sieberts et al [122], and the deduced cytotype was sufficient to understand the combination of seed parent and pollen parent.

Consequently, the parentage of 22 indigenous varieties was inferred, and 12 of them revealed no mismatch in the parentage test (Table 14). Their cytotypes matched the inferred parentage entirely and contributed to determining the combination of these parents. LOD scores for all 22 varieties were sufficient to support the inferred parentage. The allele-sharing test also inferred 46 single parent–offspring parentages, and 36 of them showed no mismatches. The cytotypes of these inferred combinations were valuable to estimate whether the alleged single parent was the seed parent or the pollen parent. The reconstructed genealogy of the indigenous varieties was not tree-like, but reminiscent of a route map of a city with its ‘hub’ structure (Fig 8 and Fig 9).

Although the LOD score of the inferred parentage varied widely between varieties according to their *RCI* scores, it demonstrated that the inferred combination would be authoritative. The fixation index (*F*_*w*_) of the indigenous varieties was not large enough and suggested that inbreeding did not affect the LOD score (Table 5). On the contrary, LOD showed a significant correlation to the *RCI* value, suggesting that uneven distribution of allele frequency affected the LOD score. Kunenbo-A, Kishu, yuzu, sweet orange and sour orange were found in 17, 13, 10, 8 or 5 inferred parentage combinations as parents, respectively (Tables 14 and 15). The frequent usage of particular varieties as parents would accumulate alleles specific to them in the given population and change allele frequencies, and this is considered to increase the difference in the LOD scores (S3 Fig). The influence of uneven distribution on the LOD score became prominent when inferring single parent–offspring dyads using Eq 5. Their deduced LOD scores showed large changes, and some of them had negative values (Table 15). The clear correlation between *RCI* and LOD scores was also observed in these varieties. The observed large influence of allele frequency on the LOD score strongly suggested that LOD score would not be a measure for inferring single parent–offspring parentage in citrus. To overcome the disadvantages of the LOD score, we proposed a simple *SPP* score to determine the inferred parentage. The *SPP* score was estimated from the genotypes and allele frequencies of the alleged single parent and offspring. The *SPP* scores for the known hybrid varieties support the parentage of the single parent in the known hybrids (Table 3). The *SPP* values of the inferred single parent–offspring were close to those of the known hybrids (Table 15).

### Involvement of pummelo in the occurrence of the indigenous varieties

No pummelo varieties in this study were recognized as parents in triads (Table 14). However, the allele-sharing test with their cytotypes suggested five varieties (A003:asahikan, A028:hassaku, A035:hyoukan, A049:kaikoukan and A055:kinkoji) were hybrids of kunenbo-A and an unidentified variety or varieties harboring the pummelo-type cytotype. Likewise, three varieties (Naruto:A095, natsudaidai:A098 and oukan: A104) were inferred to be hybrids of Kishu (A059), shunkokan (A137) a hybrid of Yatsushiro (A204), jabon (A046) a hybrid of yuzu (A208), and two varieties (A021:funadoko and A103:ootoukan) hybrids of bendizao (A005), with unidentified varieties harboring the pummelo cytotype as the other parent in each case. Since pummelo is monoembryonic [2], its offspring should have different genotypes to the parent. We considered that the 12 evaluated pummelo varieties were not sufficient to find correct parentage, or some of them could be lost. However, these findings suggest that these unidentified pummelo varieties were cultivated close to kunenbo-A, Kishu, yuzu or bendizao in the past. Of those probable pummelo offspring inferred by the allele-sharing test, kaikoukan (A049) was inferred to be the parent of iyo (A044), and of sanbokan (A112) or andoukan (A001), with dancy (A016) and Kishu (A059) as pollen parents, respectively. Fukuba reported that 33 citrus varieties consisting of Kishu, Satsuma, Yatsushiro, kaikoukan, kunenbo, dancy, sour orange, sweet orange koji, ujukitsu, yuzu, citron and various pummelo varieties had been cultivated widely in the Wakayama region of Japan for a long time when his report was published in 1882 [123]. Similar records were found in old Japanese articles, suggesting that these varieties were selected in these regions.

### The inferred roles of Kishu, kunenbo and yuzu in the occurrence of citrus varieties

The genetic identity test recognized four different types of kunenbo (*C*. *nobilis* Lour. var. kunep Tanaka; kunenbo-A, kunenbo-B, twukkuni and King) in the evaluated indigenous varieties (Table 4). These four types were inferred to be the parents of others, and kunenbo-B was inferred to be the offspring of sour orange (Table 14 and Table 15). Yamamoto et al reported that kunenbo is self-incompatible [124], and this would contribute to the many offspring of kunenbo-A. Interestingly, the allele-sharing test inferred that kunenbo-A was an offspring of Kishu (*C*. *kinokuni* hort. ex Tanaka) and an unidentified variety harboring the sweet orange-type cytotype. This observation revealed that both Satsuma and Yatsushiro would be BC1 selections of Kishu, and demonstrated the introgression of the Kishu genome into at least 30 varieties through kunenbo-A. Consequently, the inferred parentage revealed the pivotal role of Kishu in the occurrence of these indigenous varieties.

The allele-sharing test also inferred yuzu (*C*. *junos* Siebold ex Tanaka) to be the parent of 10 varieties, five of which were hybrids with kunenbo-A (Table 14). The involvement of yuzu and kunenbo-A in these varieties suggests that they have been cultivated together for a considerable period. With these observations, not only kunenbo-A, but also yuzu was regarded as another key variety for the occurrence of the indigenous varieties. Tanaka stated that *C*. *nobilis* must have played some important part in creating a new subsection Microacrumen in Japanese southern islands [12], and this evidence supports his proposal.

### Three types of tachibana and their offspring

Three types of tachibana (*C*. *tachibana* (Makino) Tanaka) were inferred to be parents of hanayu, girimikan, hyuganatsu and ogonkan (aka ogonto), which are indigenous varieties in Japan [7,12]. The name ‘Tachibana’ appears in the historic Japanese article ‘Kojiki’ published in 712 A.D. Kaibara describes tachibana as having been cultivated in diverse regions of Japan in his book published in 1709 [125]. The allele-sharing test and structure analysis raise the hypothesis that the three types of tachibana found in this study arose from hybridization of the same unknown parents. Hirai et al reported isozyme polymorphism in tachibana accessions collected from various regions of Japan [20], and these results support this hypothesis. These tachibana varieties are presumed to have crossed with others close to them at various places in Japan.

Kourai tachibana (*C*. *nippokoreana* Tanaka) was found in Yamaguchi prefecture in Japan, and it was initially misidentified as tachibana [7]. Though these three types of tachibana were not inferred to be parents of Kourai tachibana, the allele-sharing test suggested that an unidentified type of tachibana not evaluated in this study might be its parent. Additionally, the allele-sharing test revealed that koji, another indigenous variety of Japan, shared the same parental variety with tachibana, suggesting their unidentified kinship. Tanaka stated that koji might have arisen from a cross between tachibana and fukure mikan [7]. Though koji is not an offspring of tachibana × fukure mikan, close kinship of koji and tachibana was suggested in this study.

### Sour orange, lemons and Cleopatra

Sour orange (*C*. *aurantium* L.) was initially regarded as an offspring of citron (*C*. *medica*), mandarin (*C*. *reticulata*) and pummelo (*C*. *maxima*) [14–16]. Recent molecular studies suggest that regular lemon (*C*. *limon* (L.) Burm.f.) arose from hybridization between sour orange and mandarin [33,34,45,47,126]. The allele-sharing test inferred that bergamot arose from hybridization of lemon and sour orange as previously demonstrated [48]. Three varieties (Hiroshimanatsubuntan, kunenbo-B and rokugatsumikan) were also inferred to be hybrids of sour orange and unidentified varieties (Table 15, Fig 8). The allele-sharing test revealed a close relationship between sour orange and lemon as Curk et al reported [47]. However, all of the evidence from the allele-sharing test, PCoA, structure analysis and their cytotypes suggest that sour orange is an offspring of lemon (Fig 8). The deduced admixture of three basic taxa suggests that lemon might be a BC2 of citron and pummelo, and sour orange was deduced to be the offspring of lemon and the F1 of pummelo and mandarin. Another admixture analysis with *K* = 4 also demonstrated a similar result (S2 Fig). The reason for the discrepancy between the parentage of lemon and sour orange and that reported by Curk et al [47] is unclear, but genotyping error or any DNA markers that deviate from HWE might change allele frequencies in the population and could result in the opposite results.

Cleopatra was regarded as a variety of Indian origin [2]. Its cytotype was unique and no similar ones were found. Tizon (*C*. *papillaris* Blanco) was inferred to arise from hybridization of Cleopatra and sweet orange (Table 14). With these inferred parentages, lemon and sour orange, and also Cleopatra and sweet orange are considered to have been cultivated in the same regions for considerable durations. Future evaluation with this approach could identify the parent of sour orange or Cleopatra unless they have been lost.

### Origins of *C*. *ichangensis* and other acid citrus varieties

Many studies based on DNA marker analysis of nuclear genomes have reported the unique position of *C*. *ichangensis* (ichanchii) in citrus taxonomy [33,34,55,57]. Swingle considered *C*. *ichangensis* as a variation of Papeda, and he classified it to subgenus Papeda section Papedocitrus [11]. He defined ‘Ichandarin’ as a hybrid of *C*. *ichangensis* × mandarin, and assumed yuzu as an Ichandarin [11]. Tanaka also recognized the similarity between *C*. *ichangensis* and yuzu, but he classified *C*. *ichangensis* to section Osmocitrus subsection Euosmocitrus by yuzu [12]. The allele-sharing test and parentage analysis in this study did not confirm direct parentage of *C*. *ichangensis* to yuzu as Swingle assumed, and their cytotypes unfortunately did not coincide. The allele-sharing test suggests the direct parentage between *C*. *ichangensis* and lemon, but the cytotype of *C*. *ichangensis* is unique and does not match that of lemon (Table 15). Because this study did not include sufficient number of papeda or citron as reference, the parentage of *C*. *ichangensis* and lemon was required further investigation. The origin of yuzu must be examined in detail with further evidence, but the similarity between yuzu and *C*. *ichangensis* suggests the unidentified parent of *C*. *ichangensis* as a primary candidate. Meanwhile, Swingle regarded Ichang lemon (*C*. *wilsoni*) as a probable hybrid of *C*. *ichangensis* and pummelo [11], but Tanaka classified it in the same section with yuzu (section Papedocitrus). The allele-sharing test revealed that the Ichang lemon is not an offspring of *C*. *ichangensis* but an offspring of yuzu as the pollen parent and an unidentified variety having the unique Ichang lemon cytotype as the seed parent (Table 15, Fig 8). In a similar fashion, Swingle regarded sudachi (*C*. *sudachi* hort. ex Shirai) as an Ichandarin [11], but Tanaka regarded sudachi, kizu and hanayu to be natural hybrids of yuzu and classified them in the same section [7,12]. The allele-sharing test inferred them to be hybrids of yuzu with various varieties (Table 14 and Table 15, Fig 8 and Fig 9), and confirmed the implications of Tanaka.

### Origins of Satsuma and kunenbo

The allele-sharing test and parentage analysis inferred the parentages among Satsuma, Yatsushiro, kunenbo-A and Kishu. Many studies have pointed out similarities among Satsuma, kunenbo and Yatsushiro [12,13,123,127–130]. The close relationship between Satsuma and kunenbo has been observed in the DNA marker analysis [8,33,34]. Yatsushiro (*C*. *yatsushiro* hort. ex Yu. Tanaka) is an old but abandoned variety that has been produced in several regions as a substitute for Satsuma recently [123,127,128].

In 1709, Kaibara described 15 citrus varieties including ‘Unshukitsu’, which is an old name for Satsuma [125], but Tanaka considered it to represent Kishu [129]. The first document that is considered to describe Satsuma appeared in 1848 by Okamura [127]. In this document, he reported that Satsuma had been cultivated in many regions of Japan for several hundred years as a high quality seedless variety. George R. Hall introduced the first Satsuma trees to Florida from Japan in 1876 [2], indicating that Satsuma was already widely recognized for superior fruit characteristics by this time. Abe described nine and 15 local names for Kishu and Satsuma, respectively, in his book published in 1904 [128]. Together with these old documents and his own survey of old Satsuma trees in the Kyushu region, Tanaka proposed that Satsuma arose at Nagashima town in Kagoshima prefecture from the 15th to 16th centuries [129,130]. Kishu was a major citrus variety from the 12th to 18th centuries in Japan that was produced in wide regions including Kagoshima [127–130]. The origin of Kishu is not known. However, the occurrence of a Chinese biotype (nanfengmiju) agreed with recent speculation that it was transmitted from China to Japan in ancient times [12]. Kunenbo (*C*. *nobilis* Lour. var. kunep Tanaka) is not an indigenous variety of Japan but is regarded to have been transmitted from South China through Taiwan, Sakishima Islands and Ryukyu Islands to the Kyushu region around the 8th century [125,129,130]. The inferred parentage of kunenbo-A suggests that kunenbo is a hybrid of Kishu selected in ancient China or else, then propagated to many places. Therefore, it is likely that kunenbo was backcrossed to Kishu in the Kagoshima region of Japan several times and Satsuma and Yatsushiro were selected from their offspring.

Some characteristics of Satsuma contrast to those of its parents Kishu and kunenbo. For example, kunenbo is self-incompatible [124], but Kishu and Satsuma are not. Both kunenbo and Satsuma are polyembryonic but Kishu is monoembryonic [70]. Nakano et al isolated candidate genes involved in the polyembryony of Satsuma [131]. Likewise, Kishu shows no parthenocarpy (Shimizu, T., unpublished) but Satsuma is an exceptionally high and stable parthenocarpic variety [117]. Kotoda et al isolated two gibberellin 20-oxidase genes from Satsuma thought to be involved in parthenocarpy, and demonstrated their different biological functions [71]. Very recently, Kotoda et al isolated three gibberellin 2-oxidase genes of Satsuma involved in the degradation of bioactive gibberellic acid [132]. Furthermore, Satsuma is an entirely male sterile variety but Kishu and kunenbo are fertile varieties [117,133]. Goto et al recently revealed that male sterility of Satsuma is mostly caused by a decrease in pollen in the anther, and suggested the involvement of a nuclear gene to decrease pollen number [134]. The inferred parentage of Satsuma, Kishu and kunenbo with yuzu, sweet orange, sour orange, koji, tachibana and various pummelos is anticipated to enable a deep understanding of these traits of importance to the citrus industry. Another genotyping study with more than 1,000 certified SNP markers confirmed these inferred parentages (Shimizu, T. et al, in preparation). Whole genome sequence analysis and a comparative genomic approach for Satsuma, Kishu and kunenbo will reveal them at a molecular level.

In conclusion, the allele-sharing test and parentage test with the certified DNA markers inferred the parentage of 22 indigenous citrus varieties, and single parents of 46 indigenous citrus varieties. Genotyping analysis of chloroplast and mitochondrial genomes with 11 DNA markers classified cytotypes into 18 categories, and these were helpful in confirming the inferred parentages. Likelihood ratio analysis of triads verified the inferred parentages with significant scores. However, the scores of the triads were susceptible to the allele frequencies of particular varieties in a given population and showed large changes. Such susceptibility of the score became evident when it was applied to validate the parentage of single parents to offspring. Alternatively, a single parent–offspring probability (*SPP*) score was proposed to verify the inferred single parent to offspring parentage. The inferred parentage identified 12 types of varieties, consisting of Kishu, several types of kunenbo, yuzu, koji, sour orange, dancy, kobeni mikan, sweet orange, three types of tachibana, Cleopatra, willowleaf mandarin, and unidentified pummelo varieties, that were deeply involved in the occurrence of these indigenous varieties. The inferred parentage of the indigenous varieties confirmed their hybrid origins as stated by recent studies [11,12,14,15]. This study will also contribute to a reconsideration of their taxonomy.

## Supporting Information

S1 Fig Pedigree tree of hybrid varieties used for error checking of DNA markers. This chart was drawn using Helium software.
(PNG)

S2 Fig Inferred admixture proportions (Q) of 101 indigenous citrus varieties obtained by structure analysis with *K* = 4. Three clusters correspond to the deduced basic taxa at *K* = 4 (pummelo, citron mandarin, and probable papeda).
(TIF)

S3 Fig Histogram of *RCI* values of the 101 indigenous varieties estimated with 123 DNA markers. (TIF)

S1 Table All plant materials used in this study. (PDF)

S2 Table Details of the DNA markers used in this study. (PDF)

S3 Table Genotypes of 371 plant samples obtained with 246 preliminary selected markers. (PDF)

S4 Table Summary of DNA marker inconsistency for known parent-offsrping trios. (PDF)

S5 Table Summary of matched genotypes between a pair of samples by the 169 validated markers. (PDF)

S6 Table Genetic characteristics of all DNA markers for representative varieties/strains. (XLS)

S7 Table Summary of the Hardy–Weinberg equilibrium test of the 169 certified DNA markers with the 101 indigenous varieties. (PDF)

S8 Table Inferred admixture proportions (Q) of 101 indigenous citrus varieties obtained with *K* = 3. (PDF)

S9 Table Genotypes of all plant samples obtained with 11 SSR markers for organelle genomes. (PDF)

S10 Table Numbers of DNA markers that are inconsistent under the allele-sharing test between all combinations of representative indigenous varieties. (PDF)

S11 Table Representative genotypes of the 22 indigenous citrus varieties and their inferred parental varieties. (PDF)
