# Supplementary figures and images for: Hybrid Origins of Citrus Varieties Inferred from DNA Marker Analysis of Nuclear and Organelle Genomes
Source: PLoS One. 2016 Nov 30;11(11):e0166969. doi: 10.1371/journal.pone.0166969 (PMC5130255; doi:10.1371/journal.pone.0166969)

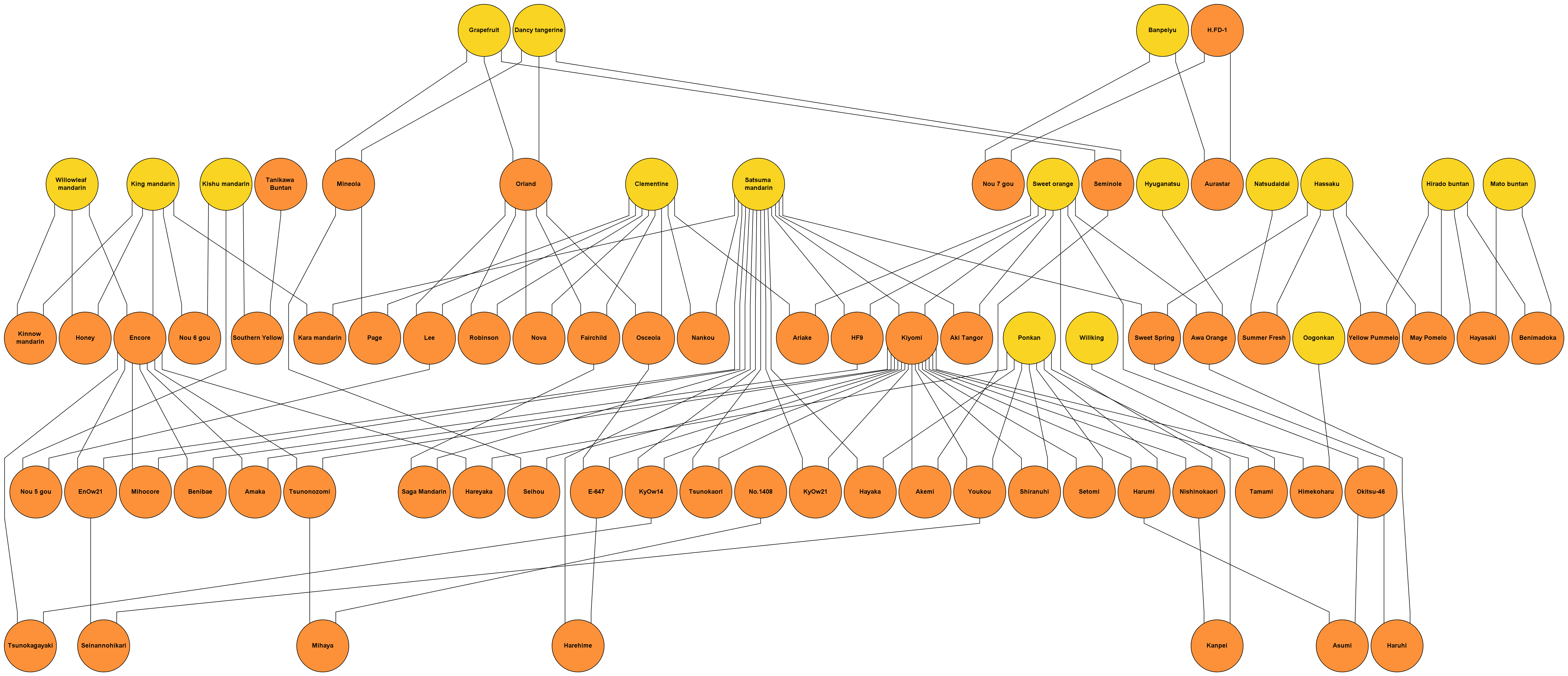

Supplement: S1 Fig — This chart was drawn using Helium software. (PNG) [file pone.0166969.s001.png]

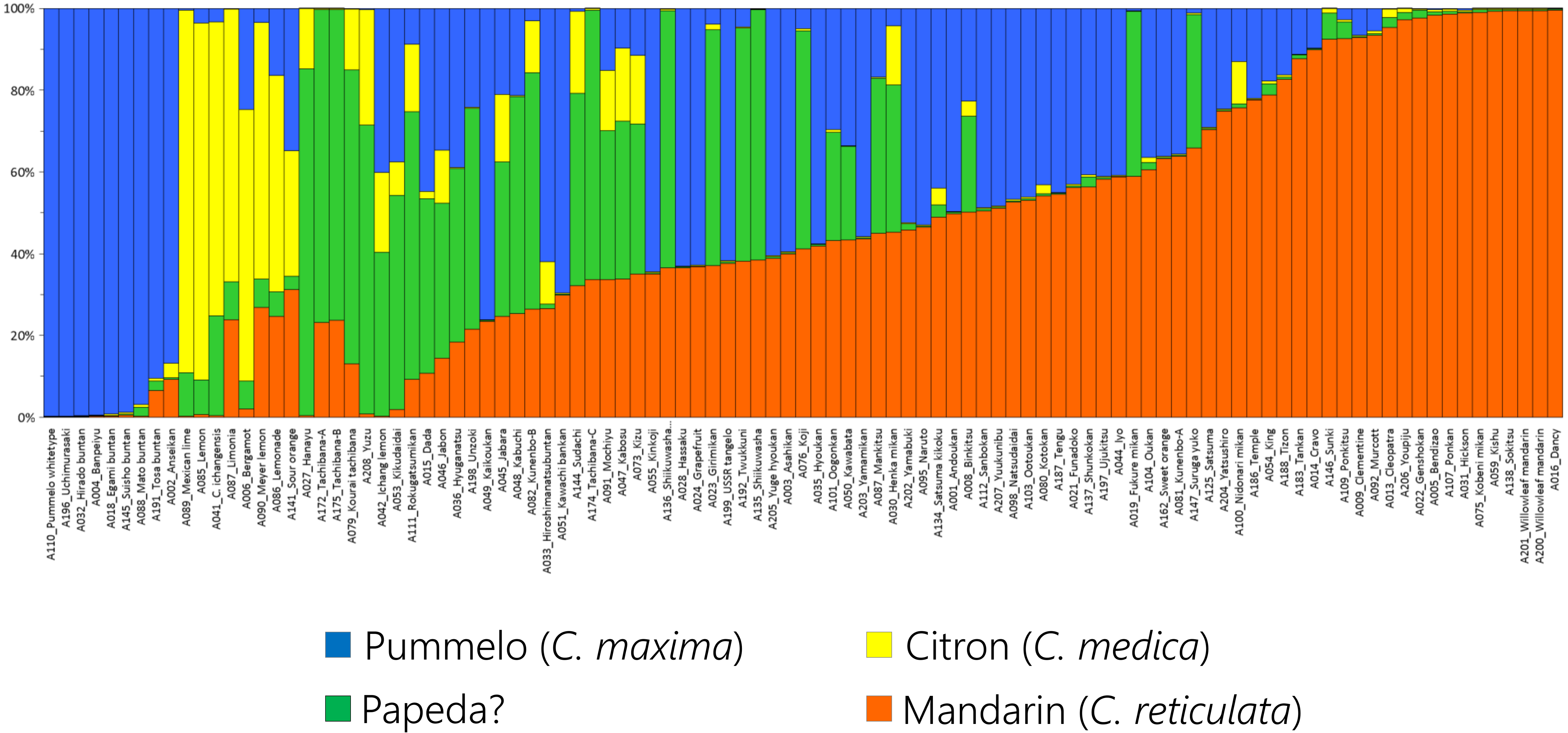

Supplement: S2 Fig — Three clusters correspond to the deduced basic taxa at K = 4 (pummelo, citron mandarin, and probable papeda). (TIF) [file pone.0166969.s002.tif]

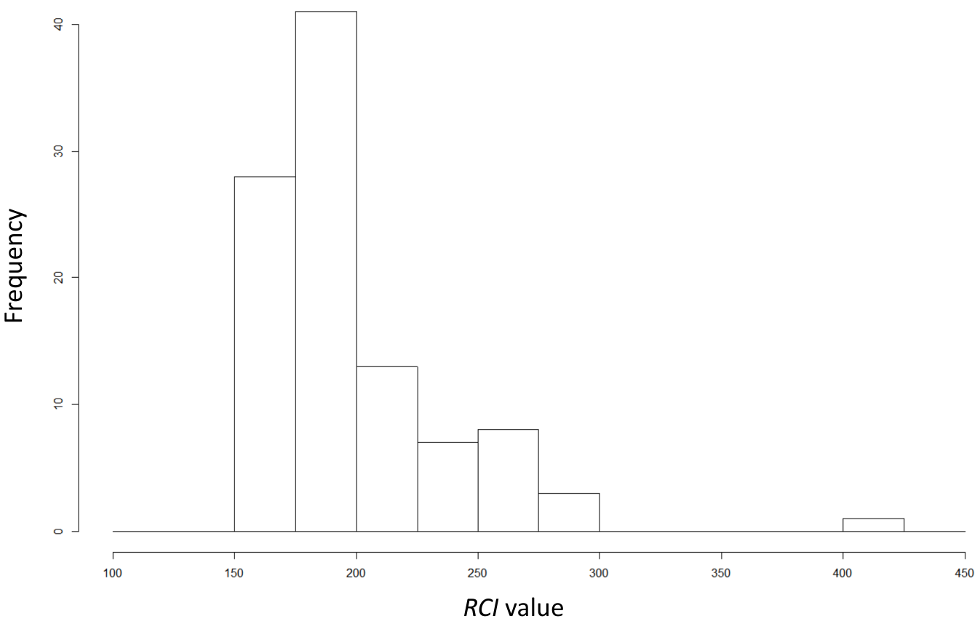

Supplement: S3 Fig — (TIF) [file pone.0166969.s003.tif]
