# Supplementary material for: Hybrid Origins of Citrus Varieties Inferred from DNA Marker Analysis of Nuclear and Organelle Genomes
Source: PLoS One. 2016 Nov 30;11(11):e0166969. doi: 10.1371/journal.pone.0166969 (PMC5130255; doi:10.1371/journal.pone.0166969)
Supplement: S1 Table — (PDF) [file pone.0166969.s004.pdf]

Table S1. All plant materials used in this study

A. Indigenous varieties

ID	Rep	Vaiety name	Strain name	RA	Scientific name	Accession I
A001	*	Andoukan	(Stock strain)	1	*C. maxima* (hybrid)	115520
A002	*	Anseikan	(Stock strain)	1	*C. grandis* Osbeck var. anseikan hort. ex Tanaka	117431
A003	*	Asahikan	(Stock strain)	1	*C. asahikan* hort. ex Tanaka	113333
A004	*	Banpeiyu	(Stock strain)	1	*C. maxima* Merr.	171506
A005	*	Bendizao	(Stock strain)	1	*C. succosa* hort. ex Tanaka	171488
A006	*	Bergamot	(Stock strain)	1	*C. bergamia* Risso	113223
A007		Bendiguangju	(Honchi Kokitsu)	1	C. spp	113459
A008	*	Binkitsu	(Stock strain)	2	*C. platymamma* hort. ex Tanaka	113168
A009	*	Clementine	(Stock strain)	1	*C. clementina* hort. ex Tanaka	113161
A010		Clementine	A Peau Fin	1	*C. clementina* hort. ex Tanaka	117392
A011		Clementine	Caffin	1	*C. clementina* hort. ex Tanaka	113163
A012		Clementine	de Nules	1	*C. clementina* hort. ex Tanaka	181745
A013	*	Cleopatra	(Stock strain)	2	*C. reshni* hort. ex Tanaka	117402
A014	*	Cravo	(Stock strain)	1	C. spp	113160
A015	*	Dada	(Stock strain)	1	*C. luteo-turgida* Tanaka	117375
A016	*	Dancy	(Stock strain)	1	*C. tangerina* hort. ex Tanaka	117396
A017		Dancy	Obenimikan	2	*C. tangerina* hort. ex Tanaka	117395
A018	*	Egami buntan	(Stock strain)	1	*C. maxima* Merr.	168898
A019	*	Fukure mikan	(Stock strain)	1	*C. tumida* hort. ex Tanaka	117407
A020		Fukushukan	(Stock strain)	1	C. spp	113371
A021	*	Funadoko	(Stock strain)	2	*C. funadoko* hort. ex Yu.Tanaka	117372
A022	*	Genshokan	(Stock strain)	1	*C. genshokan* hort. ex Tanaka	113159
A023	*	Girimikan	(Stock strain)	2	*C. tardiva* hort. ex Shirai	117404
A024	*	Grapefruit	Marsh	1	*C. paradisi* Macfad.	171496
A025		Grapefruit	Red blush	1	*C. paradisi* Macfad.	117298
A026		Grapefruit	Triumph	1	*C. paradisi* Macfad.	113255
A027	*	Hanayu	(Stock strain)	1	*C. hanaju* Siebold ex Shirai	117378
A028	*	Hassaku	(Stock strain)	2	*C. hassaku* hort. ex Tanaka	117286
A029		Hebesu	(Stock strain)	1	*C. junos* (hybrid)	168863
A030	*	Henka mikan	(Stock strain)	1	*C. pseudo-aurantium* hort. ex Yu.Tanaka	117382
A031	*	Hickson	(Sporting limb on 'Ellendale' tangor)	1	C. spp	113369
A032	*	Hirado buntan	(Stock strain)	3	*C. maxima* Merr.	171507
A033	*	Hiroshimanatsubuntan	(Stock strain)	1	*C. hiroshimana* hort. ex Yu.Tanaka	171491
A034		Houraikan	(Stock strain)	1	*C. ujukitsu* hort. ex Tanaka	117376
A035	*	Hyoukan	(Stock strain)	2	*C. ampullacea* hort. ex Tanaka	113365
A036	*	Hyuganatsu	(Stock strain)	1	*C. tamurana* hort. ex Tanaka	117317
A037		Hyuganatsu	Ihara 1	1	*C. tamurana* hort. ex Tanaka	223668
A038		Hyuganatsu	Muroto Konatsu	2	*C. tamurana* hort. ex Tanaka	115758
A039		Hyuganatsu	Orange Hyuga	1	*C. tamurana* hort. ex Tanaka	117316
A040		Hyuganatsu	Shoukakukei Hyuganatsu	1	*C. tamurana* hort. ex Tanaka	116984
A041	*	Ichanchii	(Stock strain)	2	*C. ichangensis* Swingle	NA
A042	*	Ichang lemon	(Stock strain)	2	*C. wilsonii* Tanaka	117384
A043		Iyo	Miyauchi Iyo	1	*C. iyo* hort. ex Tanaka	117373
A044	*	Iyo	Ootani Iyo	1	*C. iyo* hort. ex Tanaka	115518
A045	*	Jabara	(Stock strain)	1	*C. junos* (hybrid)	113193
A046	*	Jabbon	(Stock strain)	1	*C. junos* (hybrid)	117463
A047	*	Kabosu	(Stock strain)	2	*C. sphaerocarpa* hort. ex Tanaka	117381
A048	*	Kabuchi	(Stock strain)	2	*C. keraji* hort. ex Tanaka var. kabuchii hort. ex Tanaka	117390
A049	*	Kaikoukan	(Stock strain)	1	*C. truncata* hort. ex Tanaka	113343
A050	*	Kawabata	(Stock strain)	1	*C. aurea* hort. ex Tanaka	113344
A051	*	Kawachi bankan	(Stock strain)	1	*C. maxima* Merr.	117412
A052	*	Keraji	(Stock strain)	3	*C. keraji* hort. ex Tanaka	117389
A053	*	Kikudaidai	(Stock strain)	1	*C. canariculata* hort. ex Yu.Tanaka	117371
A054	*	King	(Stock strain)	2	*C. nobilis* Lour.	117386
A055	*	Kinkoji	(Stock strain)	1	*C. obovoidea* hort. ex I.Takah.	117361
A056		Kinukawa	(Stock strain)	1	*C. glaberrima* hort. ex Tanaka	117278
A057		Kishu	Hira Kishu	2	*C. kinokuni* hort. ex Tanaka	117398
A058		Kishu	Hisago Komikan	1	*C. kinokuni* hort. ex Tanaka	117457
A059	*	Kishu	Kishu	2	*C. kinokuni* hort. ex Tanaka	171490
A060		Kishu	Kishu mikan	1	*C. kinokuni* hort. ex Tanaka	171490
A061		Kishu	Kishu mikan Ihara Ichijoji	2	*C. kinokuni* hort. ex Tanaka	117948
A062		Kishu	Komikan Fukuyama (Kinkou PEARL)	1	*C. kinokuni* hort. ex Tanaka	117947
A063		Kishu	Komikan Kawachi	1	*C. kinokuni* hort. ex Tanaka	117951
A064		Kishu	Komikan Tensui	1	*C. kinokuni* hort. ex Tanaka	117949
A065		Kishu	Kouda mikan	1	*C. kinokuni* hort. ex Tanaka	117458
A066		Kishu	Mukaku Kishu (seedless Kishu)	2	*C. kinokuni* hort. ex Tanaka	117399
A067		Kishu	Nan feng mi ju	2	*C. kinokuni* hort. ex Tanaka	117731
A068		Kishu	Ozaki Komikan	1	*C. kinokuni* hort. ex Tanaka	203329
A069		Kishu	Sakurajima Komikan Matsuura	1	*C. kinokuni* hort. ex Tanaka	117459
A070		Kishu	Sakurajima Komikan senbatsu 1gou	1	*C. kinokuni* hort. ex Tanaka	117460
A071		Kishu	Sakurajima Komikan Shirahama	1	*C. kinokuni* hort. ex Tanaka	117461
A072		Kishu	Taka Mkan	1	*C. kinokuni* hort. ex Tanaka	(113181)
A073	*	Kizu	(Stock strain)	1	*C. kizu* hort. ex Yu.Tanaka	181849
A074		Kobayashi mikan	(Stock strain)	1	*C*. spp	NA
A075	*	Kobeni mikan	(Stock strain)	2	*C. erythrosa* hort. ex Tanaka	117397
A076	*	Koji	(Stock strain)	1	*C. leiocarpa* hort. ex Tanaka	113156
A077		Komikan 2009-130	(Stock strain)	1	*C*. spp	NA
A078		Konejime	(Stock strain)	1	*C. junos* (hybrid)	170627
A079	*	Kourai Tachibana	(Stock strain)	2	*C. nippokoreana* Tanaka	171493
A080	*	Kotokan	(Stock strain)	1	*C. kotokan* Hayata	113342
A081	*	Kunenbo	(Stock strain)	3	*C. nobilis* Lour. var. kunep Tanaka	117387
A082	*	Kunenbo	Kagoshima 0007	1	*C. nobilis* Lour. var. kunep Tanaka	NA
A083		Kunenbo	Kagoshima 0027	1	*C. nobilis* Lour. var. kunep Tanaka	NA
A084		Kunenbo	Kunenbo Kamikoshikijima	1	*C. nobilis* Lour. var. kunep Tanaka	117950
A085	*	Lemon	Lisbon	2	*C. limon* (L.) Burm. f.	117289
A086	*	Lemonade	(Stock strain)	1	*C*. spp	117481
A087	*	Limonia	(Stock strain)	1	*C. limonia* (L.) Osbeck	113240
A088	*	Mato buntan	(Stock strain)	1	*C. maxima* Merr.	117283
A089	*	Mexican lime	(Stock strain)	2	*C. aurantifolia* (Christm.) Swingle	113215
A090	*	Meyer lemon	(Stock strain)	1	*C. meyerii* Yu.Tanaka	113237
A091	*	Mochiyu	(Stock strain)	1	*C. inflata* hort. ex Tanaka	117379
A092	*	Murcott	(Stock strain)	1	*C*. spp	113374
A093		Myrttle leaf orange	Chinott	1	*C. myrtifolia* Raf.	113378
A094		Nansho daidai	(Stock strain)	1	*C. taiwanica* Tanaka et Shimada	117363
A095	*	Naruto	(Stock strain)	2	*C. medioglobosa* hort. ex Tanaka	117293
A096		Natsudaidai	(Stock strain)	1	*C. natsudaidai* Hayata	(117296)
A097		Natsudaidai	Beniamanatsu	1	*C. natsudaidai* Hayata	113504
A098	*	Natsudaidai	Kawano Natsudaidai	2	*C. natsudaidai* Hayata	117297
A099		Natsudaidai	Tachibana orange	1	*C. natsudaidai* Hayata	113208
A100	*	Nidonari mikan	(Stock strain)	1	*C*. spp	113362
A101	*	Oogonkan	(Stock strain)	1	*C*. spp	172148
A102		Ootachibana	(Stock strain)	1	*C. otachibana* hort. ex Yu.Tanaka	117362
A103	*	Ootoukan	(Stock strain)	1	*C. sinomaxima* hort. ex Tanaka	113332

A104	*	Oukan	(Stock strain)	2	*C. suavissima* hort. ex Tanaka	117394
A105		Ponkan	Ihara ponkan	1	*C. reticulata* Blanco	172349
A106		Ponkan	Morita ponkan	1	*C. reticulata* Blanco	168862
A107	*	Ponkan	Oota ponkan	2	*C. reticulata* Blanco	171505
A108		Ponkan	Yoshida ponkan	1	*C. reticulata* Blanco	113178
A109	*	Ponkitsu	(Stock strain)	2	*C. ponki* hort. ex Tanaka	113171
A110	*	Pummelo whitetype	(Stock strain)	1	*C. maxima* Merr.	113251
A111	*	Rokugatsumikan	(Stock strain)	1	*C. rokugatsu* hort. ex Yu.Tanaka	113387
A112	*	Sanbokan	(Stock strain)	1	*C. sulcata* hort. ex I.Takah.	117315
A113		Satsuma	Aoshima unshu	2	*C. unshiu* Marcov.	117320
A114		Satsuma	Dobashi beni	1	*C. unshiu* Marcov.	117325
A115		Satsuma	Haraguchi wase	1	*C. unshiu* Marcov.	170625
A116		Satsuma	Imamura unshu	1	*C. unshiu* Marcov.	117339
A117		Satsuma	Iwasaki wase	1	*C. unshiu* Marcov.	115489
A118		Satsuma	Juman unshu	1	*C. unshiu* Marcov.	117337
A119		Satsuma	Jutaro unshu NC	1	*C. unshiu* Marcov.	NA
A120		Satsuma	Kinokuni unshu	1	*C. unshiu* Marcov.	115749
A121		Satsuma	Kuno unshu	1	*C. unshiu* Marcov.	170633
A122		Satsuma	Miyagawa wase	2	*C. unshiu* Marcov.	117351
A123		Satsuma	Nagahashi unshu NC	2	*C. unshiu* Marcov.	NA
A124		Satsuma	Niu unshu	2	*C. unshiu* Marcov.	113499
A125	*	Satsuma	Okitsu wase (NC)	2	*C. unshiu* Marcov.	170630
A126		Satsuma	Original tree	5	*C. unshiu* Marcov.	168849
A127		Satsuma	Otsu-4 (NC)	2	*C. unshiu* Marcov.	117319
A128		Satsuma	Shirakawa unshu	1	*C. unshiu* Marcov.	117452
A129		Satsuma	Sugiyama unshu	1	*C. unshiu* Marcov.	117516
A130		Satsuma	Suruga beni	1	*C. unshiu* Marcov.	113153
A131		Satsuma	Ueno wase	2	*C. unshiu* Marcov.	168844
A132		Satsuma	Yamada unshu NC	2	*C. unshiu* Marcov.	NA
A133		Satsuma	Yamashita beni	1	*C. unshiu* Marcov.	113125
A134	*	Satsuma Kikoku	(Stock strain)	1	*C*. spp	117498
A135	*	Shiikuwasha	(Stock strain)	2	*C. depressa* Hayata	117406
A136	*	Shiikuwasha	Oogimi Kuganii	1	*C. depressa* Hayata	117514
A137	*	Shunkokan	(Stock strain)	1	*C. shunkokan* hort. ex Tanaka	117377
A138	*	Sokitsu	(Stock strain)	3	*C. kinokuni* hort. ex Tanaka var. subcompressa hort. ex Tanaka	117400
A139		Sour orange	Bouquet de Fleurs	1	*C. aurantium* L. var. crispa Yu.Tanaka	117370
A140		Sour orange	Chaozhouchen	1	*C. aurantium* L.	117367
A141	*	Sour orange	Daidai	2	*C. aurantium* L.	117365
A142		Sour orange	Kaiseito	1	*C. aurantium* L.	117369
A143		Sour orange	Za daidai	1	*C. aurantium* L.	NA
A144	*	Sudachi	(Stock strain)	2	*C. sudachi* hort. ex Shirai	117383
A145	*	Suisho buntan	(Stock strain)	1	*C. maxima* Merr.	117279
A146	*	Sunki	(Stock strain)	2	*C. sunki* (Hayata) hort. ex Tanaka	117403
A147	*	Suruga Yuko	(Stock strain)	1	*C. leiocarpa* hort. ex Tanaka forma monoembryota Tanaka	113164
A148		Sweet orange	Cadenera	1	*C. sinensis* (L.) Osbeck	117526
A149		Sweet orange	Cara Cara	1	*C. sinensis* (L.) Osbeck	118404
A150		Sweet orange	Crescent	1	*C. sinensis* (L.) Osbeck	117529
A151		Sweet orange	Hamlin	1	*C. sinensis* (L.) Osbeck	117307
A152		Sweet orange	Jin Cheng	1	*C. sinensis* (L.) Osbeck	117912
A153		Sweet orange	Joppa	1	*C. sinensis* (L.) Osbeck	113273
A154		Sweet orange	Mediterranean Sweet Orange	1	*C. sinensis* (L.) Osbeck	113288
A155		Sweet orange	Moro NC	2	*C. sinensis* (L.) Osbeck	113291
A156		Sweet orange	Parson Brown	1	*C. sinensis* (L.) Osbeck	117306
A157		Sweet orange	Pineapple	2	*C. sinensis* (L.) Osbeck	117517
A158		Sweet orange	Santa Catarina	1	*C. sinensis* (L.) Osbeck	113270
A159		Sweet orange	Seike navel	1	*C. sinensis* (L.) Osbeck	117312
A160		Sweet orange	Shamouti	1	*C. sinensis* (L.) Osbeck	115510
A161		Sweet orange	Tong Zi Gan	1	*C. sinensis* (L.) Osbeck	117522
A162	*	Sweet orange	Trovita	1	*C. sinensis* (L.) Osbeck	172154
A163		Sweet orange	Washington navel	1	*C. sinensis* (L.) Osbeck	115511
A164		Sweet orange	Valencia	1	*C. sinensis* (L.) Osbeck	117303
A165		Sweet orange	Wu Yue Cheng	1	*C. sinensis* (L.) Osbeck	117914
A166		Sweet orange	Xue Gan	1	*C. sinensis* (L.) Osbeck	117521
A167		Sweet orange	Yinzi Gan	1	*C. sinensis* (L.) Osbeck	117302
A168	*	Tachibana	(Stock strain)	2	*C. tachibana* (Makino) Tanaka	117405
A169		Tachibana	Anettaishijou	1	*C. tachibana* (Makino) Tanaka	117883
A170		Tachibana	Botanical garden	1	*C. tachibana* (Makino) Tanaka	117884
A171		Tachibana	Hananoiwaya	1	*C. tachibana* (Makino) Tanaka	168874
A172		Tachibana	Heda 1	1	*C. tachibana* (Makino) Tanaka	168872
A173		Tachibana	Heda 2	1	*C. tachibana* (Makino) Tanaka	168873
A174	*	Tachibana	Ishinami Minka	1	*C. tachibana* (Makino) Tanaka	117882
A175	*	Tachibana	Ishinami No.1	1	*C. tachibana* (Makino) Tanaka	117880
A176		Tachibana	Ishinami No.2	1	*C. tachibana* (Makino) Tanaka	117881
A177		Tachibana	Oodomari OP-2	1	*C. tachibana* (Makino) Tanaka	115536
A178		Tachibana	Okitsu	1	*C. tachibana* (Makino) Tanaka	168876
A179		Tachibana	Reizanji	1	*C. tachibana* (Makino) Tanaka	168875
A180		Tajima mikan	(Stock strain)	1	*C. tachibana* (Makino) Tanaka	113359
A181		Takumanatsukunenbo	(Stock strain)	1	*C*. spp	113355
A182		Tankan	(Stock strain)	1	*C. tankan* Hayata	171489
A183	*	Tankan	Taishun	1	*C. tankan* Hayata	115492
A184		Tankan	Tarumizu 1	1	*C. tankan* Hayata	113508
A185		Tankan	T-132	1	*C. tankan* Hayata	113507
A186	*	Temple	(Stock strain)	1	*C. temple* hort. ex Yu.Tanaka	113357
A187	*	Tengu	(Stock strain)	1	*C. tengu* hort. ex Tanaka	117360
A188	*	Tizon	(Stock strain)	1	*C. papillaris* Blanco	113354
A189		Toukan	(Stock strain)	1	*C*. spp	113356
A190		Tookunin	(Stock strain)	1	*C. nobilis* Lour. var. kunep Tanaka	204391
A191	*	Tosa buntan	(Stock strain)	1	*C. maxima* Merr.	117280
A192	*	Twukkuni	(Stock strain)	1	*C. nobilis* Lour. var. kunep Tanaka	204376
A193		Twukkunin	(Stock strain)	1	*C. nobilis* Lour. var. kunep Tanaka	204373
A194		Twukunihu	(Stock strain)	1	*C. nobilis* Lour. var. kunep Tanaka	204407
A195		Twuukuribu	(Stock strain)	1	*C. nobilis* Lour. var. kunep Tanaka	204397
A196	*	Uchimurasaki	(Stock strain)	1	*C. maxima* Merr.	116973
A197	*	Ujukitsu	(Stock strain)	1	*C. ujukitsu* hort. ex Tanaka	115519
A198	*	Unzoki	(Stock strain)	1	*C*. spp	113330
A199	*	USSR Tangelo	(Stock strain)	1	*C*. spp	113350
A200	*	Willowleaf mandarin	Mediterranean mandarin	1	*C. deliciosa* Ten.	117393
A201	*	Willowleaf mandarin	Willowleaf mandarin	1	*C. deliciosa* Ten.	117941
A202	*	Yamabuki	(Stock strain)	1	*C. yamabuki* hort. ex Yu.Tanaka	117364
A203	*	Yamamikan	(Stock strain)	4	*C. intermedia* hort. ex Tanaka	117359
A204	*	Yatsushiro	(Stock strain)	1	*C. yatsushiro* hort. ex Tanaka	117388
A205	*	Yuge hyoukan	(Stock strain)	3	*C. yuge-hyokan* hort. ex Yu.Tanaka	117507
A206	*	Youpi ju (Yuhikitsu)	(Stock strain)	1	*C. oleocarpa* hort. ex Tanaka	117401
A207	*	Yuukunibu	(Stock strain)	2	*C. yanbaruensis* Tanaka	113383
A208	*	Yuzu	(Stock strain)	2	*C. junos* Siebold ex Tanaka	117380

*) Accession ID: JP numbers in NIAS Genebank (http://www.gene.affrc.go.jp/index_en.php)

B: hybrid varieties

ID	Rep	Vaiety name	Seed parent	Pollen parent	RA	Accession ID	Breeder +)
B001	*	'Akemi'	'Kiyomi'	'Seminole'	1	NA	1
B002	*	'Aki Marine'	'Kiyomi'	*'Southern red'*	1	NA	2
B003	*	'Aki Tangor'	Satsuma mandarin	Sweet orange	1	NA	2
B004	*	'Allspice'	*'Imperial' grapefruit*	Willowleaf mandarin	1	115522	3
B005	*	'Amaka'	'Kiyomi'	'Encore'	1	NA	1
B006	*	'Ariake'	Sweet orange	Clementine	1	NA	1
B007	*	'Asumi'	'Okitsu-46'	'Harumi'	2	NA	1
B008	*	'Aurastar'	'H･FD-1'	Banpeiyu	1	NA	1
B009	*	'Awa Orange'	Hyuganatsu	Sweet orange	1	115757	4
B010	*	'Benibae'	'HF9'	'Encore'	1	NA	1
B011	*	'Benimadoka'	Mato buntan	Hirado buntan	1	NA	1
B012	*	'Chandler pummelo'	*Siamese Pink pummelo*	*Siamese Sweet pummelo*	1	113449	3
B013	*	'Ehimekashi 28'	'Nankou'	*'Amakusa'*	1	NA	5
B014	*	'Encore'	King mandarin	Willowleaf mandarin	1	117421	3
B015	*	'Fairchild'	Clementine	'Orland'	1	113367	6
B016	*	'Fortune'	Clementine	Dancy tangerine	1	115495	6
B017	*	'Harehime'	'E-647'	Satsuma mandarin	1	NA	1
B018	*	'Hareyaka'	'Encore'	Ponkan	1	NA	1
B019	*	'Haruhi'	'Okitsu-46'	'Awa Orange'	2	NA	1
B020	*	'Haruka'	Hyuganatsu	Natsudaidai	1	NA	7
B021	*	'Harumi'	'Kiyomi'	Ponkan	1	NA	1
B022	*	'Hayaka'	Satsuma mandarin	Ponkan	1	NA	1
B023	*	'Hayasaki'	Mato buntan	Hirado buntan	1	NA	1
B024	*	'Himekoharu'	'Kiyomi'	Oogonkan	1	NA	5
B025	*	'Hiroshimakaken 11'	'Kiyomi'	*'Southern red'*	1	NA	2
B026	*	'Honey'	King mandarin	Willowleaf mandarin	1	117930	3
B027	*	'Kanpei'	'Nishinokaori'	Ponkan	1	NA	5
B028	*	'Kara'	Satsuma mandarin	King mandarin	2	117422	3
B029	*	'Kincy mandarin'	King mandarin	Dancy tangerine	1	117423	3
B030	*	'Kinnow mandarin'	King mandarin	Willowleaf mandarin	1	113458	3
B031	*	'Kiyomi'	Satsuma mandarin	Sweet orange	1	NA	1
B032		'Kuchinotsu-41'	(Autotetraploid of Hyuganatsu)		1	NA	1
B033	*	'Lee'	Clementine	'Orland'	1	113385	6
B034	*	'May Pomelo'	Hassaku	Hirado buntan	1	NA	1
B035	*	'Mihaya'	'Tsunonozomi'	'No.1408'	1	NA	1
B036	*	'Mihocore'	Satsuma mandarin	'Encore'	1	NA	1
B037	*	'Mineola'	'Duncan' grapefruit	Dancy tangerine	1	113377	6
B038	*	'Nankou'	Satsuma mandarin	Clementine	1	NA	1
B039	*	'Nishinokaori'	'Kiyomi'	Sweet orange	1	NA	1
B040	*	'Nou 5 gou'	'Lee'	Kishu mandarin	1	NA	1
B041	*	'Nou 6 gou'	King mandarin	Kishu mandarin	1	NA	1
B042	*	'Nou 7 gou'	'H･FD-1'	Banpeiyu	1	NA	1
B043	*	'Nova'	Clementine	'Orland'	1	113360	6
B044	*	'Ooitakaken-4'	Satsuma mandarin	*'Amakusa'*	1	NA	8
B045	*	'Orland'	'Duncan' grapefruit	Dancy tangerine	1	113327	6
B046		'Oroblanco'	*Acid less pummelo*	*Grapefruit (Tetraploid)*	1	NA	3
B047	*	'Osceola'	Clementine	'Orland'	1	113329	6
B048	*	'Page'	'Mineola'	Clementine	1	113370	6
B049	*	'Pearl'	*'Imperial' grapefruit*	Willowleaf mandarin	1	113364	3
B050	*	'Pixie mandarin'	Kincy mandarin	(unknown; open pollination)	1	113366	3
B051	*	'Robinson'	Clementine	'Orland'	1	113386	6
B052	*	'Saga Mandarin'	Satsuma mandarin	'Fairchild'	1	115755	9
B053		'Sagakashi 34'	(Nucellar seedling of Shiranuhi)		1	NA	9
B054	*	'Seihou'	'Kiyomi'	'Mineola'	1	NA	1
B055	*	'Seinannohikari'	'KyOw21'	'Youkou'	1	NA	1
B056	*	'Seminole'	Grapefruit	Dancy tangerine	1	113348	6
B057	*	'Setomi'	'Kiyomi'	Ponkan	1	223670	10
B058	*	'Shiranuhi'	'Kiyomi'	Ponkan	1	NA	1
B059	*	'Southern Yellow'	'Tanikawa Buntan'	Kishu mandarin	1	NA	1
B060	*	'Summer Fresh'	Hassaku	Natsudaidai	1	NA	1
B061	*	'Sweet spring'	Satsuma mandarin	Hassaku	1	NA	1
B062	*	'Tamami'	'Kiyomi'	'Willking'	1	NA	1
B063	*	'Tanikawa Buntan'	Mato buntan?	Sanbokan?	2	117433	1
B064	*	'Tsunokagayaki'	'KyOw14'	'Encore'	1	NA	1
B065	*	'Tsunokaori'	'Kiyomi'	Satsuma mandarin	2	NA	1
B066	*	'Tsunonozomi'	'Kiyomi'	'Encore'	1	NA	1
B067	*	'Willking'	King mandarin	Willowleaf mandarin	1	117425	3
B068	*	'Yellow Pummelo'	Hassaku	Hirado buntan	1	NA	1
B069	*	'Youkou'	'Kiyomi'	Ponkan	1	NA	1
B070	*	'E-647'	'Kiyomi'	'Osceola'	1	NA	1
B071	*	'EnOw21'	'Encore'	Satsuma mandarin	1	NA	1
B072	*	'H･FD-1'	Hassaku	*Trifoliate orange*	1	NA	1
B073	*	'HF9'	Satsuma mandarin	Sweet orange	1	NA	1
B074	*	'KyOw14'	'Kiyomi'	Satsuma mandarin	1	NA	1
B075	*	'KyOw21'	'Kiyomi'	Satsuma mandarin	1	NA	1
B076	*	'No.1408'	'EnOw21'	*No.2681*	1	NA	1
B077	*	'Okitsu-46'	'Sweet spring'	Sweet orange	1	NA	1
B078	*	'Okitsu-56'	*'Okitsu-45'*	'Nou 5 gou'	1	NA	1

Italic : not evaluated in this study.

+) Breeders: 1 NARO, 2 Hiroshima Prefectural Agricultural Station, 3 UC Riverside, 4 Tokushima Prefectural Fruit Tree Station, 5 Ehime Research Institute of Agriculture, Forestry, and Fisheries Fruit Tree Research Center, 6 USDA, 7 Mr. Ishii Tokuo, 8 Oita Prefectural Fruit Tree Station, 9 Saga Prefectural Fruit Tree Station, 10 Yamaguchi Prefectural Fruit Tree Station

C: selected strains

ID	Rep	Line name	Seed parent	Pollen parent	RA	Accession ID	Breeder +)
C001	*	Strain 01	'Tsunonozomi'	U	1	NA	1
C002	*	Strain 02	'Harehime'	Clementine	1	NA	1
C003	*	Strain 03	'Harehime'	'Seinannohikari'	1	NA	1
C004	*	Strain 04	U	U	1	NA	1
C005	*	Strain 05	U	U	1	NA	1
C006	*	Strain 06	'Sweet spring'	Sweet orange	1	NA	1
C007	*	Strain 07	Andoukan	Hassaku	1	NA	1
C008	*	Strain 08	'Kiyomi'	U	1	NA	1
C009	*	Strain 09	'Kiyomi'	Sweet orange	1	NA	1
C010	*	Strain 10	'Kiyomi'	'Willking'	1	NA	1
C011	*	Strain 11	'Kiyomi'	'Willking'	2	NA	1
C012	*	Strain 12	'Kiyomi'	Line63	1	NA	1
C013	*	Strain 13	Satsuma mandarin	Hyuganatsu	1	NA	1
C014	*	Strain 14	'Kiyomi'	'E-647'	1	NA	1
C015	*	Strain 15	U	Satsuma mandarin	1	NA	1
C016	*	Strain 16	Mato buntan	Hirado buntan	1	NA	1
C017	*	Strain 17	Satsuma mandarin	Murcott	1	NA	1

C018	*	Strain 18	Satsuma mandarin	Clementine	1	NA	1
C019	*	Strain 19	'KyOw21'	'Encore'	1	NA	1
C020	*	Strain 20	'Encore'	Ponkan	1	NA	1
C021	*	Strain 21	U	'Encore'	1	NA	1
C022	*	Strain 22	'EnOw21'	'Youkou'	1	NA	1
C023	*	Strain 23	'KyOw21'	Dancy	1	NA	1
C024	*	Strain 24	'KyOw21'	King mandarin	1	NA	1
C025	*	Strain 25	Line41	Murcott	1	NA	1
C026	*	Strain 26	'KyOw21'	U	1	NA	1
C027	*	Strain 27	'KyOw14'	'Encore'	1	NA	1
C028	*	Strain 28	'KyOw21'	'Encore'	1	NA	1
C029	*	Strain 29	U	Murcott	1	NA	1
C030	*	Strain 30	'KyOw21'	'Robinson'	1	NA	1
C031	*	Strain 31	'Encore'	Satsuma mandarin	1	NA	1
C032	*	Strain 32	'KyOw21'	U	1	NA	1
C033	*	Strain 33	U	Line22	1	NA	1
C034	*	Strain 34	'Sweet spring'	'Ariake'	1	NA	1
C035	*	Strain 35	U	U	1	NA	1
C036	*	Strain 36	U	'Ariake'	1	NA	1
C037	*	Strain 37	Line30	U	1	NA	1
C038	*	Strain 38	U	Line22	1	NA	1
C039	*	Strain 39	'Tsunokagayaki'	'Ariake'	1	NA	1
C040	*	Strain 40	'Kiyomi'	'Encore'	2	NA	1
C041	*	Strain 41	'Kiyomi'	'Encore'	1	NA	1
C042	*	Strain 42	'KyOw21'	'Ariake'	1	NA	1
C043	*	Strain 43	'KyOw21'	Dancy	1	NA	1
C044	*	Strain 44	'Lee'	Satsuma mandarin	1	NA	1
C045	*	Strain 45	'Kiyomi'	'Robinson'	1	NA	1
C046	*	Strain 46	'Nishinokaori'	Iyo	1	NA	1
C047	*	Strain 47	'Nankou'	Line46	1	NA	1
C048	*	Strain 48	Satsuma mandarin	Sweet orange	1	NA	1
C049	*	Strain 49	Satsuma mandarin	Clementine	1	NA	1
C050	*	Strain 50	Satsuma mandarin	Clementine	1	NA	1
C051	*	Strain 51	Hyuganatsu	Sweet orange	1	NA	1
C052	*	Strain 52	Hyuganatsu	Sweet orange	1	NA	1
C053	*	Strain 53	Hyuganatsu	Sweet orange	1	NA	1
C054	*	Strain 54	Hassaku	Dancy	1	NA	1
C055	*	Strain 55	U	Clementine	1	NA	1
C056	*	Strain 56	Sweet orange	'Sweet spring'	1	NA	1
C057	*	Strain 57	'Sweet spring'	Hassaku	1	NA	1
C058	*	Strain 58	Iyo	U	1	NA	1
C059	*	Strain 59	'Sweet spring'	Hassaku	1	NA	1
C060	*	Strain 60	Hassaku	Dancy	1	NA	1
C061	*	Strain 61	Hassaku	Hirado buntan	1	NA	1
C062	*	Strain 62	Hassaku	Sweet orange	1	NA	1
C063	*	Strain 63	'Kiyomi'	'Willking'	1	NA	1
C064	*	Strain 64	Satsuma mandarin	Sweet orange	1	NA	1
C065	*	Strain 65	'Kiyomi'	'Willking'	1	NA	1
C066	*	Strain 66	'Kiyomi'	'Willking'	1	NA	1
C067	*	Strain 67	Line06	Satsuma mandarin	1	NA	1
C068	*	Strain 68	'Kiyomi'	'Okitsu-46'	1	NA	1
C069	*	Strain 69	'Okitsu-46'	'Harumi'	1	NA	1
C070	*	Strain 70	Line06	Line10	1	NA	1
C071	*	Strain 71	'Okitsu-46'	'Harumi'	2	NA	1
C072	*	Strain 72	'Kiyomi'	Pummelo whitetype	1	NA	1
C073	*	Strain 73	'Harehime'	'Okitsu-56'	1	NA	1
C074	*	Strain 74	'E-647'	Ponkan	1	NA	1
C075	*	Strain 75	U	U	1	NA	1
C076	*	Strain 76	U	U	1	NA	1
C077	*	Strain 77	'Harehime'	'Okitsu-56'	1	NA	1
C078	*	Strain 78	'Okitsu-46'	'Harumi'	1	NA	1
C079	*	Strain 79	'Kiyomi'	U	1	NA	1
C080	*	Strain 80	'Kiyomi'	U	1	NA	1
C081	*	Strain 81	'E-647'	U	1	NA	1
C082	*	Strain 82	Satsuma mandarin	U	1	NA	1
C083	*	Strain 83	'Harehime'	'Harumi'	1	NA	1
C084	*	Strain 84	'Harehime'	'Asumi'	1	NA	1
C085	*	Strain 85	'Kiyomi'	'Asumi'	1	NA	1

RA: Replicate of analysis; U: not used in this study.
