# Supplementary material for: Hybrid Origins of Citrus Varieties Inferred from DNA Marker Analysis of Nuclear and Organelle Genomes
Source: PLoS One. 2016 Nov 30;11(11):e0166969. doi: 10.1371/journal.pone.0166969 (PMC5130255; doi:10.1371/journal.pone.0166969)
Supplement: S2 Table — (PDF) [file pone.0166969.s005.pdf]

Table S2. DNA markers used in this study

A. Genomic DNA markers developed in this study

#	Marker	Cert.	Category	LG	Accession ID	SSR position	Source	Motif	Tag	Tagged forward primer	F Tm	tail	Tailed reverse primer	R Tm	Amp	Program
1	CTVR01	*	gSSR	LG7	AF506028	274544	Genomic sequence	(ctg)n	F9GAC	ctagtatcaggacgacTAGAGTTCCCAGACTGAGTTC	58.91	gtttctt	gtttcttCAAACACTGTAAACATGCAGC	59.30	305	56/32
2	CTVR04	**	gSSR	LG7	AF506028	274557	Genomic sequence	(tgctgt)n	F9GCC	ctagtattaggacgccTAGAGTTCCCAGACTGAGTTC	58.91	gtttctt	gtttcttCAAACACTGTAAACATGCAGC	59.30	305	56/32
3	CUBER403		gSSR	LG5	GA975130	98	Citrus unshiu BAC end sequences	(taat)n	F9GTC	ctagtatgaggacgtcACTTTTCTCCTTTTCATTGGG	57.02	gtttct	gtttctTAATCTTCGTGCTAATGTGAC	56.49	251	58/32
4	CUBER410	*	gSSR	LG5	GA977509	312	Citrus unshiu BAC end sequences	(aaac)n	F9TAC	ctagtatcaggactacCAACCACAGCTTTTATCAATC	56.16	gtttct	gtttctTGTTTTGTTTTTACTGCTTGG	56.4	258	54/32
5	CUBER601		gSSR	LG2	GA975859	111	Citrus unshiu BAC end sequences	(tggtaa)n	F9GTC	ctagtatgaggacgtcAAATGACTGTGATAATGGTGC	57.27	gtttctt	gtttcttCTAGCGAAGGTTTGTCTTTC	57.04	164	56/32
6	CUBER602	**	gSSR	LG1	GA976278	99	Citrus unshiu BAC end sequences	(ttcctc)n	F9TAC	ctagtatcaggactacCGCCATTTTCTTTTTCTTCTC	56.73	gtttctt	gtttcttGACTGAAAGTGCTTCAAAAAC	56.87	232	54/34
7	CUBER903		gSSR	LG7	GA976453	774	Citrus unshiu BAC end sequences	(ata)n	F9CCG	ctagtattaggacccgAACCTGCACAATCATAAGTAG	58.03	gttt	gtttGAAGATAATGAGAGCCAACTAT	57.43	175	56/32
8	CUBER918	*	gSSR	LG9	GA974879	627	Citrus unshiu BAC end sequences	(tcc)n	F9GTC	ctagtatgaggacgtcCAGGCTCGTGTTCCATGAT	62.34	gttt	gtttGAGTATACGCCGCCAGTTT	62.38	101	56/32
9	CUBER920	*	gSSR	LG4	GA977190	719	Citrus unshiu BAC end sequences	(atga)n	F9GCC	ctagtattaggacgccCATGACGATGATTCTGAAACTT	59.06	gttt	gtttCGGACATCAGCTTTCATTC	58.87	164	56/32
10	CUBER925		gSSR	--	GA975321	635	Citrus unshiu BAC end sequences	(tat)n	F9GAC	ctagtatcaggacgacGCAATGTATGTCCTTTATTTGA	57.06	gttt	gtttAGGTAACTTTAGGTGTGAGG	58.65	167	56/32
11	CUBER935	**	gSSR	LG6	GA976619	482	Citrus unshiu BAC end sequences	(tga)n	F9GAC	ctagtatcaggacgacGTCTTTGGTTCTTCACATGAATAA	59.61	gt	gtTTATGATTCCCGCCCAATAT	58.93	178	58/32
12	CUBER939	*	gSSR	LG1	GA975313	822	Citrus unshiu BAC end sequences	(tca)n	F9GCC	ctagtattaggacgccTAAGATTAGGAAGTTGACCGA	58.21	gttt	gtttGGCAGAGGATACTTGGATA	57.8	215	60/32
13	CUBER951	*	gSSR	LG1	GA976278	322	Citrus unshiu BAC end sequences	(ttc)n	F9AGG	ctagtattaggacaggTTTCCTCTTCCTTCTCCTC	57.87	gttt	gtttAGTATTCCTGACACTGATGA	57.74	161	56/32
14	GRW2011	*	gSSR	LG3	Scaffold3	8602126	Clementine v1.0	(TA)n(CT)n	F9GTC	ctagtatgaggacgtcTCAATCAGCCCTTTCAGTTT	59.83	gttt	gtttCGTGGATCTAAGTGGGAAGTAT	61.38	122	54/32
15	GRW2051		gSSR	LG3	Scaffold3	43996493	Clementine v1.0	(TA)n	F9TAC	ctagtatcaggactacATGTGACAACAAAGATTTGTCC	59.71	gttt	gtttATTTCTTAATTACAGTTAACAGGG	57.01	108	56/32
16	GRW3021		gSSR	LG5	BUZY1863.g1	426	NCBI Trace Archive	(TGT)n	F9TAC	CTAGTATCAGGACTACCATCCCGCATCCTCTTTCT	61.75	gttt	gtttGTGGCTGGCTTTGACATAA	60.59	117	56/32
17	GRW3011	*	gSSR	LG3	Scaffold3	8606684	Clementine v1.0	(TAT)n	F9GAC	ctagtatcaggacgacCACGGACACAGTTTCAACAC	62.02	gttt	gtttAAAGCTCTAGCACTCTCACC	61.58	94	58/32
18	GSR10S09	*	gSSR	LG5	BDGO01000073	2983	Shimizu, T. et al. unpublished	(aga)n	F9CCG	ctagtattaggacccgTGGAACAGACAAAATAACCTG	56.77	gttt	gtttAtTAAAACAAGCAGTGATCCG	57.57	196	54/32
19	GSR10S17	*	gSSR	LG5	BDGO01000144	3275	Shimizu, T. et al. unpublished	(attatc)n	F9GAC	ctagtatcaggacgacGTCATGCTATGGTTATCACAC	57.17	g	gTTTTATTGTGACGTGGAAACG	58.08	163	54/32
20	GSR10S29	*	gSSR	LG2	BDGO01000234	2691	Shimizu, T. et al. unpublished	(ttaa)n	F9GTC	ctagtatgaggacgtcCTTCCCCTGTTGTCTTAAATC	56.92	gtt	gttTATTATCTTTACCGTGGGAGC	57.67	139	54/32
21	GSR2101		gSSR	LG1	BUZY106502.b1	151	NCBI Trace Archive	(ta)n(ga)n	F9GAC	ctagtatcaggacgacAAATGAATTGTAAATAACGGAGAT	57.07	gttt	gtttGAGATTCTATAATTGAAAGCTCTC	57.07	113	54/34
22	GSR2103		gSSR	LG1	BUZY123497.b1	527	NCBI Trace Archive	(ga)n	F9TAC	ctagtatcaggactacAAGCCAATTCACACTTTGTC	59.26	gttt	gtttGACAAGTCATCAGTCTTTGG	58.48	221	56/32
23	GSR2108		gSSR	LG3	BUZY43752.b1	1001	NCBI Trace Archive	(tc)n	F9GTC	ctagtatgaggacgtcATCAATATTCTAAGAGCCTGCC	60.04	gttt	gtttAGCCTGGTTCTTGTATCCC	61.3	176	56/34
24	GSR2129		gSSR	LG3	BUZY33352.b1	639	NCBI Trace Archive	(at)n	F9CCG	ctagtattaggacccgCTGGTGGCTTGTTGTAATAGAG	61.03	gttt	gtttGGTTAGAGGCAAACATATTCC	58.73	157	54/34
25	GSR2131		gSSR	LG8	BUZY261862.g1	600	NCBI Trace Archive	(ac)n	F9GAC	ctagtatcaggacgacCTTGTTGAAGCACATATGCC	59.84	gttt	gtttGTTACTGTAGCTAGATAGGTGAG	59.28	156	60/32
26	GSR2150		gSSR	LG1	BUZY112333.g1	589	NCBI Trace Archive	(tc)n	F9GTC	ctagtatgaggacgtcGTTGATTCTTCCCTTTCCTC	58.16	gtt	gttTACTTGTCTTTCTTCTGCTACC	59.78	234	56/36
27	GSR3114		gSSR	LG7	BUZY25274.b1	204	NCBI Trace Archive	(atc)n	F9GTC	ctagtatgaggacgtcATCCGGCTCTTCTGATTATG	59.32	gttt	gtttAGTGTTAGCAAATAGTCGGT	59.03	155	56/32
28	GSR3121	*	gSSR	LG4	BUZY53309.g1	466	NCBI Trace Archive	(aag)n	F9TAC	ctagtatcaggactacCCAGTAATCAAGACATTGTAGG	58.52	gttt	gtttCGAAACTGCTTTAGTCTAAGAG	58.81	156	54/32
29	GSR3126		gSSR	LG7	BUZY170281.g1	793	NCBI Trace Archive	(aat)n	F9GTC	ctagtatgaggacgtcCGCCACATTGACAAACATT	59.61	gttt	gtttGTACACAAGATCACTCAACA	57.45	209	56/32
30	GSR3128	*	gSSR	--	BUZY72931.g1	514	NCBI Trace Archive	(gaa)n	F9TCC	ctagtatcaggactccCGGGTTGCTTCTTATTGTC	58.79	gttt	gtttATGAGAGGCTTCTAATCTTGG	59.27	127	56/32
31	GSR3136	*	gSSR	LG3	BUZY128287.b1	671	NCBI Trace Archive	(atc)n(gtc)n	F9AGG	ctagtattaggacaggGCCAGTACCATCTTCATGT	59.37	gttt	gtttCTTGGAGTGACGAATGGAGA	61.58	133	58/32
32	GSR3137		gSSR	LG8	BUZY232019.g1	765	NCBI Trace Archive	(aat)n	F9GAC	ctagtatcaggacgacGCCATCACAATTTGTATCCT	58.22	gtt	gttTAATCTGATAGTGGCATCCT	57.57	163	56/32
33	GSR3138	*	gSSR	LG5	BUZY63372.g1	120	NCBI Trace Archive	(aat)n	F9GTC	ctagtatgaggacgtcGCCTTTGCTAACTGGAAAGTC	61.82	gttt	gtttCATAAGCTTGCGTGTTGTGT	61.83	135	54/32
34	GSR3140	*	gSSR	LG5	BUZY59969.b1	213	NCBI Trace Archive	(aat)n	F9TCC	ctagtatcaggactccGGCGAGAAGGAGAATTAGTT	59.47	gttt	gtttGTGGTTGATAGATTGAGAGG	57.37	170	56/32
35	GSR3141		gSSR	LG8	BUZY111250.g1	389	NCBI Trace Archive	(att)n	F9CCG	ctagtattaggacccgGGGATATACTCGATTAAGTAGAC	57.25	gttt	gtttAATAGTAGGGATTCAGATTCAC	57.07	144	56/34
36	GSR5105		gSSR	--	BUZZ96761.g1	86	NCBI Trace Archive	(atctc)n	F9CCG	ctagtattaggacccgTAATGAGAGAAGCCACAAAAG	56.57	gtttctt	gtttcttACAATTAGGAAGAGAGGAAGG	57.14	116	60/32
37	GSR5112		gSSR	LG8	BUZY9077.b1	1002	NCBI Trace Archive	(actaa)n	F9GTC	ctagtatgaggacgtcCTCCTGCAATCACAAGATAAG	57.09	gtt	gttTCTTTGACTTTCTTTCCCAAC	56.98	226	56/34
38	GSR5114		gSSR	LG5	BUZY129906.g1	488	NCBI Trace Archive	(aacac)n	F9TAC	ctagtatcaggactacTAGTAACGGTGAGTGAAAAAG	56.27	gtttctt	gtttcttAACCTGTGAAAGTTAATGTGG	57.05	308	56/36
39	GSR5122		gSSR	LG3	BUZY155080.g1	1878	NCBI Trace Archive	(cgcca)n	F9CCG	ctagtattaggacccgCACACTGACCTATCTTTTGAC	56.96	gtttc	gtttcTTTCCAATTTCATCTCCCAAG	56.81	253	56/32
40	GSR5127		gSSR	LG3	BUZY8842.b1	463	NCBI Trace Archive	(caagc)n	F9GCC	ctagtattaggacgccACTTACCTTTCTTTCATGCTC	56.57	gtttctt	gtttcttACGAGATGTCACACTTTTTG	56.87	179	54/32
41	GSR5129		gSSR	LG3	BUZZ54533.g1	270	NCBI Trace Archive	(tgagc)n	F9AGG	ctagtattaggacaggGGCCTTGAATAAAAATTCGTG	56.79	gtttctt	gtttcttAAAACACTTACAAGATCCACC	56.77	260	56/34
42	GSR6101	*	gSSR	LG7	BUZY128935.g1	107	NCBI Trace Archive	(ccggca)n	F9GCC	ctagtattaggacgccAAAAACAGAGCAGAAGGAAC	57.04	gtttct	gtttctTGCAATCCCATAATAGCAAAG	57.04	248	60/32
43	GSR6122	*	gSSR	LG2	BUZY50477.b1	103	NCBI Trace Archive	(aaacga)n	F9GCC	ctagtattaggacgccCTAAAGAAGTGTGCTAGGTTG	57.15	gtttct	gtttctTGTAGTATCAAAAGACCCTCC	57.15	213	54/32
44	GSR6129	*	gSSR	LG6	BUZY22109.b1	229	NCBI Trace Archive	(gaaggt)n	F9AGG	ctagtattaggacaggGAAGAGAAATGGGAAAGTTGG	57.48	gtttctt	gtttcttAAGCTCAAAAGAAGAGTCAAG	56.79	227	56/30
45	GSR6133	*	gSSR	LG6	BUZY106766.g1	443	NCBI Trace Archive	(ctgggg)n	F9GCC	ctagtattaggacgccGTTTTGATCTTCTTGTTGCTG	57.22	gtttc	gtttcTTCTGGGCTTACTCTCAATC	57.22	256	54/32
46	GSR6135	*	gSSR	LG7	BUZY210038.g1	459	NCBI Trace Archive	(agggtt)n	F9AGG	ctagtattaggacaggTAGGGTTTTACGATTTCCAAG	56.96	gtttctt	gtttcttGAACAACAGTATGAGGTGAAG	56.96	178	56/32
47	NSX02		gSSR	LG3	scaffold_3	8740	Clementine haploid v.0.9	(TA)n(CA)n	F9GTC	ctagtatgaggacgtcACATCACATCGCCAATACCC	60.66	gtttctt	gtttcttACTCGTGGGACTCATGACTC	61.46	143	54/32
48	NSX23	*	gSSR	LG3	scaffold_3	5899745	Clementine haploid v.0.9	(TAA)n	F9CCG	ctagtattaggacccgAAGATCATGGCTTCCATTCAAATC	60.18	gtttctt	gtttcttGTTGTGTTATTACGTACTTTGCATTT	59.67	246	54/32
49	NSX32	*	gSSR	LG3	scaffold_3	8574561	Clementine haploid v.0.9	(TTTGGCA)n	F9GTC	ctagtatgaggacgtcCTTTGGTGGGAATTGTCAGAGA	60.74	gtttctt	gtttcttAAAGACTCGAAATGCTGACCTT	60.73	261	56/32
50	NSX34		gSSR	LG3	scaffold_3	8970241	Clementine haploid v.0.9	(TA)n+(ATC)n	F9GCC	ctagtattaggacgccTATCCCAGTCTGCAACAAATCTAAT	60.65	gtttctt	gtttcttTTTATATTGTGTCTTATGGACCCATTT	59.83	259	54/34
51	NSX38	*	gSSR	LG2	scaffold_1	13148619	Clementine haploid v.0.9	(AT)n	F9GTC	ctagtatgaggacgtcTAGTTCCCTCTCGTTTGATCTC	59.48	gtttctt	gtttcttGCTCATTAAACAACATGCCTC	58.32	244	56/32
52	NSX39	*	gSSR	LG2	scaffold_1	13086339	Clementine haploid v.0.9	(TGAA)n	F9TAC	ctagtatcaggactacGTGAATTTGAATCCAATCCAGAT	57.64	gtttctt	gtttcttGCCACTAAATTCTGCTACTAGAT	58.29	202	56/32
53	NSX43	*	gSSR	LG5	scaffold_1	12772252	Clementine haploid v.0.9	(TTCATGA)n	F9GAC	ctagtatcaggacgacTTCTGGATCAAACTAGCTTCGT	59.94	gtttctt	gtttcttAACTTTAACCCTACAATCAGCC	58.81	158	58/32
54	NSX48		gSSR	LG5	scaffold_1	11014805	Clementine haploid v.0.9	(CTATTCT)n	F9AGG	ctagtattaggacaggCATCGTCGTAACTAAATGAGGT	58.45	gtttctt	gtttcttTTCGTAAGGAGAGTTGAGAATG	58.04	128	56/32
55	NSX53		gSSR	LG5	scaffold_1	9577531	Clementine haploid v.0.9	(ATT)n(TTV)n	F9CCG	ctagtattaggacccgATTTCACAATTTCACTCCCATCA	59.10	gtttctt	gtttcttGCTGTATTTCCATTGACGTATTT	58.00	187	56/32
56	NSX67		gSSR	LG5	scaffold_1	6050494	Clementine haploid v.0.9	(TATT)n	F9GAC	ctagtatcaggacgacCACGAGTGTTCATCACTTTAGGA	60.82	gtttctt	gtttcttCGCTGTATACCAAGTAGCTTAAGA	60.48	173	54/32
57	NSX79		gSSR	LG5	scaffold_1	1989018	Clementine haploid v.0.9	(TATG)n	F9GAC	ctagtatcaggacgacTAGCGCGGGTGTAGTCTTT	61.45	gtttctt	gtttcttATCTATACCAAACTCTGATGGCAA	60.12	223	56/32
58	NSX82	*	gSSR	LG5	scaffold_1	11437282	Clementine haploid v.0.9	(TC)n	F9GCC	ctagtattaggacgccGTGTAGCTTCAGAGCATGG	58.38	gtttctt	gtttcttGGCAATCCAATACCTGCTT	58.26	262	58/32
59	NSX84	*	gSSR	LG5	scaffold_1	6046416	Clementine haploid v.0.9	(TGGTAG)n	F9AGG	ctagtattaggacaggTGAGAAATGAGAATCCGATGTG	58.70	gtttctt	gtttcttGAGAAGGAAATGTTAAGAAGCCAAG	60.63	174	56/32
60	NSX115	*	gSSR	LG7	scaffold_23	2031639	Clementine haploid v.0.9	(CTT)n	F9TAC	ctagtatcaggactacCAATATACAGAGGAGTAACGAGG	58.12	gtttctt	gtttcttTCAACTGATAACAGGCTAATTCTA	57.72	112	56/32
61	NSX119		gSSR	LG9	scaffold_27	158921	Clementine haploid v.0.9	(GT)n(AT)n	F9TAC	ctagtatcaggactacCGACTCATCTTATCTCAAACCCT	60.13	gtttctt	gtttcttAGACTACAGATCCCACGAC	58.27	176	56/32
62	NSX121	*	gSSR	LG9	scaffold_27	624113	Clementine haploid v.0.9	(AATA)n	F9CCG	ctagtattaggacccgCTGCTCTACTTCATCATCCGT	59.81	gtttctt	gtttcttATTAGCCATGTTCACAGCAC	59.01	211	56/32
63	NSX132	*	gSSR	LG8	scaffold_28	149059	Clementine haploid v.0.9	(CAATCAC)n,(TA)n	F9GCC	ctagtattaggacgccGATGTTGGTTGCTCATTCAATGG	61.19	gtttctt	gtttcttTGGCTGCATTGCTTTAAGTTAGAG	62.17	164	56/32
64	NSX137	*	gSSR	LG8	scaffold_28	1606493	Clementine haploid v.0.9	(GAGCAG)n	F9TAC	ctagtatcaggactacCCTCCAAGCATACTACCACC	60.01	gtttctt	gtttcttGGTTCTGATGCTGCTTTGTTA	59.33	104	56/32
65	NSX141	*	gSSR	LG9	scaffold_30	777074	Clementine haploid v.0.9	(AAC)n(AAT)n	F9GAC	ctagtatcaggacgacTTAAGCCAATTCCTGAGCCT	59.49	gtttctt	gtttcttTGGTCTGATGGATTCTGATTGT	59.67	301	56/32
66	NSX145	*	gSSR	LG4	scaffold_16	422006	Clementine haploid v.0.9	(TC)n	F9CCG	ctagtattaggacccgATCCCTAACAATTTCCCTCCT	58.63	gtttctt	gtttcttTGTGATTAGTAAATAAATCCAAGAGAC	57.93	128	56/32
67	NSX150	*	gSSR	LG7	scaffold_38	744154	Clementine haploid v.0.9	(CTGTGAGCT)n	F9GCC	ctagtattaggacgccGTAACATCACTTCTTGTCTCAC	57.23	gtttctt	gtttcttGTTCAATACTATCTTGGTCCACAG	59.40	173	54/30
68	NSX153	*	gSSR	LG7	scaffold_41	1092766	Clementine haploid v.0.9	(ATGATG)n	F9GAC	ctagtatcaggacgacACTGTGGCGTACCTTTGAG	60.10	gtttctt	gtttcttGCAATAGGAAGGCTGAGGA	59.25	142	58/32
69	NSX156	*	gSSR	LG6	scaffold_42	1003587	Clementine haploid v.0.9	(TTCCT)n(TAT)n(TCT)n	F9GCC	ctagtattaggacgccCAGGTAGGTGGCTTTAAGG	57.58	gtttctt	gtttcttGGAGTTTGACAGTTTGAACCA	59.12	216	56/32
70	NSX160	*	gSSR	LG2	scaffold_49	892879	Clementine haploid v.0.9	(CACCAG)n	F9GTC	ctagtatgaggacgtcCATCGTAATGAGGCAGTGG	58.46	gtttctt	gtttcttGTTATGCGGAGGTGGTGAG	60.62	169	56/32
71	NSX162	*	gSSR	LG8	scaffold_52	782149	Clementine haploid v.0.9	(cca)n	F9TAC	ctagtatcaggactacGTGGTGCAGACAATTTACAG	57.47	gtttctt	gtttcttCTATTACTTCCATCTGCCACC	58.36	293	56/34
72	NSX165	*	gSSR	LG3	scaffold_57	147000	Clementine haploid v.0.9	(AT)n,(AGA)n	F9GAC	ctagtatcaggacgacGTTGTTCTTGTATGAAGGGTACAG	59.82	gtttctt	gtttcttTTTGTTTGTCTGTTGGCTTGTC	60.59	289	56/32
73	NSX169	*	gSSR	LG5	scaffold_66	148208	Clementine haploid v.0.9	(aag)n	F9CCG	ctagtattaggacccgGACACGTAGCTCTATCTATATCTTC	58.27	gtttctt	gtttcttGCTGCTGTTGATTGACACAT	59.59	184	56/32
74	NSX170	*	gSSR	LG4	scaffold_66	1144777	Clementine haploid v.0.9	(caa)n	F9AGG	ctagtattaggacaggAGAGAATGATCCATCCTCGT	58.09	gtttctt	gtttcttGAAAGATTAGATGCGGAACCA	58.57	187	56/32
75	NSX175		gSSR	LG1	scaffold_64	685430	Clementine haploid v.0.9	(AAGA)n(AAT)n	F9CCG	ctagtattaggacccgGAGCTTACTAATAGGATACGCTG	58.51	gtttctt	gtttcttATTGTAAGGCGAATTGGATGG	59.18	214	56/32
76	NSX186		gSSR	LG2	scaffold_117	291624	Clementine haploid v.0.9	(CTAT)n(TC)n	F9GCC	ctagtattaggacgccACACTTTGAGAGTTTCTTCAGAC	59.06	gtttctt	gtttcttTTACCAAGACTTGTTTAAGGAATC	57.38	209	56/32
77	NSX187	*	gSSR	LG2	scaffold_117	543666	Clementine haploid v.0.9	(CA)n	F9CCG	ctagtattaggacccgGAATACCCAAATCGTCTTCCTC	59.03	gtttctt	gtttcttCAGGTGGTTCATGATGTTGTAG	59.49	218	58/32
78	SRPE02	*	gSSR	LG8	AB573149	25365	Nakano et al (2012)	(acaac)n	F9GTC	ctagtatgaggacgtcTATACATAACAAACCCGCTGC	59.14	gtttctt	gtttcttAAGAGTGTTACATTTCGACGG	59.02	146	54/32
79	SRPE03		gSSR	LG1	AB573149	41640	Nakano et al (2012)	(tca)n	F9CCG	ctagtattaggacccgTAGCCGCTTTCTAATTTTCCG	59.42	gtttctt	gtttcttAGGCCACTAATTTTTGTAGGG	58.62	127	56/32
80	SRPE05	*	gSSR	LG1	AB573149	206845	Nakano et al (2012)	(ttgaa)n	F9CCG	ctagtattaggacccgATTAAAACCGCGTGAAAGTTG	58.69	gtttctt	gtttcttCTAGATATGGCGTGGAAACTC	58.67	186	58/32
81	TSRA101	*	gSSR	LG1	Scaffold_1	3013751	Clementine v1.0	(CTTT)n	F9GAC	ctagtatcaggacgacCAATGATGAAAGGCAAGGTTCTG	60.88	gtttctt	gtttcttAATTGTCACGGCAGATTATCTC	59.03	166	56/32
82	TSRA103	*	gSSR	LG6	Scaffold_1	3278744	Clementine v1.0	(AAAG)n	F9GCC	ctagtattaggacgccTGGAGATGCTCTAACTTAACATGG	60.67	gtttctt	gtttcttTGTTGTCTACGTTAATCAGACGG	60.87	188	56/32
83	TSRA107		gSSR	LG4	Scaffold_6	2823329	Clementine v1.0	(TGAAGA)n	F9GAC	ctagtatcaggacgacGGATTTAGAGAAAGTAGAGCAACCA	60.87	gtttctt	gtttcttTTTGAGAACCCAGCACTATACC	60.27	200	56/32
84	TSRA108		gSSR	LG4	Scaffold_4	16259231	Clementine v1.0	(CTT)n	F9GTC	ctagtatgaggacgtcTCAACTTGTTCACGACTTTCAC	59.82	gtttctt	gtttcttCTCAGCAGATCCAGAAGGT	58.87	187	56/32
85	TSRA109		gSSR	LG4	Scaffold_4	19243690	Clementine v1.0	(ATT)n	F9GCC	ctagtattaggacgccGCAAGAGTCAAACCAATTAGAC	58.06	gtttctt	gtttcttCATCCATATAGAAACCGTCAAACC	60.00	179	56/32
86	TSRA110	*	gSSR	LG4	Scaffold_4	24967051	Clementine v1.0	(CTTTTTT)n	F9CCG	ctagtattaggacccgGTTACACTGTCAAAGCCACTG	59.88	gtttctt	gtttcttCGTGACAAATCTCCGACTCC	60.93	173	56/32
87	TSRA117	*	gSSR	--	Scaffold_5	26579999	Clementine v1.0	(TTC)n	F9GCC	ctagtattaggacgccTTTACCATGTTCAAGCAGATATCAC	59.94	gtttctt	gtttcttTTTCGAACATCAAACTCAATTTGG	59.22	164	56/32

88	TSGR222		gSSR	LG1	Scaffold_1		Clementine v1.0	(ttta)n	F9CCG	ctagtattaggacccgTTGAGACACTCCACTATAACATT	57.49	gtttctt	gtttcttTGGTGAAGCGGTTGTTAAA	57.97	196	58/32
89	TSGR264	*	gSSR	LG2	Scaffold_2		Clementine v1.0	(aat)n	F9GCC	ctagtattaggacgccTAACACCACCTTCAATTCCCA	59.72	gtttctt	gtttcttGGCAAACTATTAGACCTCCGT	60.01	140	58/32
90	TSGR265	**	gSSR	LG2	Scaffold_2		Clementine v1.0	(ta[gca])n(caa)n	F9GCC	ctagtattaggacgccAACAGTCCGACTAATTCATCC	57.94	gtttctt	gtttcttTATCTCCAAGACTCCAACCT	57.54	165	56/32
91	TSGR271	*	gSSR	--	Scaffold_9		Clementine v1.0	(ata)n	F9CCG	ctagtattaggacccgGCTTTAGAGCTCAGTCCCA	59.18	gtttctt	gtttcttACTGCCTGTTAATGAACCCT	59.42	202	56/32
92	TSGR282		gSSR	LG3	Scaffold_3		Clementine v1.0	(tttttc)n(tg)n	F9GCC	ctagtattaggacgccAGCAAATTAATTAAACACGGCT	57.70	gtttctt	gtttcttCTCTGAAATGTGCTCTGCC	58.98	251	56/32
93	NSX16		INDEL	--	scaffold_3	4098890	Clementine haploid v.0.9	CCATGTTTAACTAAAGAGAGAATCTGTGTCGATCAACCA/C	F9GCC	ctagtattaggacgccCTCCCGCAATTATACGAGAG	58.11	gtttctt	gtttcttAGTTGCCGGTAATTTCTTGG	58.93	114	60/32
94	NSX27		INDEL	LG3	scaffold_3	7642393	Clementine haploid v.0.9	AC/AAAACC	F9TAC	ctagtatcaggactacGAACTTTCAAATGACAGCCGA	59.61	gtttctt	gtttcttCACATGTTCATTCTAGCCATCA	58.95	322	56/32
95	NSX28		INDEL	LG3	scaffold_3	8000304	Clementine haploid v.0.9	A/ACT	F9GCC	ctagtattaggacgccTTGGAGAATTGTAAACAGACAAA	57.07	gtttctt	gtttcttTCGAGTTTCATGCATAAAGACA	58.56	234	54/32
96	NSX70	*	INDEL	--	scaffold_1	5092937	Clementine haploid v.0.9	A/ACAAGCAAT	F9GCC	ctagtattaggacgccAAAGACTAGTGGCAGTCCAG	59.50	gtttctt	gtttcttTACAATGATTTGAAGCCTGCTC	59.48	132	54/32
97	NSX87		INDEL	LG5	scaffold_37	1669	Clementine haploid v.0.9	AGTT/AGTTAGACAATGTT	F9TAC	ctagtatcaggactacGAGATCCTCCATAAGCATAACA	57.60	gtttctt	gtttcttGAGACAATGTAACCTTTACCGA	58.30	132	56/32
98	NSX94		INDEL	LG5	scaffold_37	1274730	Clementine haploid v.0.9	CAAGAAG/CAAG	F9GCC	ctagtattaggacgccTCAAGTCTCAACAAACCTTCGT	60.60	gtttctt	gtttcttGAATGTGGAAAGGCGTTCTTA	59.05	117	56/32
99	NSX118	*	INDEL	LG9	scaffold_27	27707	Clementine haploid v.0.9	TACGAGTTATTA/TA	F9GTC	ctagtatgaggacgtcCGATTCCTGCAAATGAGGTA	58.14	gtttctt	gtttcttGGTGGCTGGTGAGGTATGTA	61.48	142	58/32
100	NSX129		INDEL	LG9	scaffold_27	3226975	Clementine haploid v.0.9	GCA/GCACACATCACA	F9GAC	ctagtatcaggacgacGACTTTGAAATTACATTCCCAAGAA	58.53	gtttctt	gtttcttTCCAATTTACAGTTTCTTACATTACAA	58.19	210	56/32
101	NSX148		INDEL	--	scaffold_16	3943508	Clementine haploid v.0.9	TTAATACTGGTAAT/TT	F9GTC	ctagtatgaggacgtcAACGCTTGAATGCCTGCTC	61.81	gtttctt	gtttcttTTGTCGCAGTCGCATTTCTTT	61.90	149	54/32
102	NSX161	*	INDEL	LG8	scaffold_52	156369	Clementine haploid v.0.9	AATTGGATT/AATTGGATTTAAGGATTGGATT	F9GCC	ctagtattaggacgccAGAAATCCAAGTAATTGAGTGTTTAT	57.65	gtttctt	gtttcttATCCACAAATTACCCTACATCCT	59.47	101	56/32
103	NSX179		INDEL	LG7	scaffold_68	414820	Clementine haploid v.0.9	AAAGTGCAA/*	F9TAC	ctagtatcaggactacTTTCCAACACATATTCTCAACTCA	58.89	gtttctt	gtttcttCTTTGAAGAAGTACCGACAATATTA	57.44	251	54/34
104	TSA10A29		INDEL	LG1	EY715084	473	EY715084	C/TC/CCTCTC/CTCTCCCTCTC, TCTCCCTCTCA/A	F9TCC	ctagtatcaggactCCTCTTGTTCCACATTTCTT	58.00	gttt	gtttACATCAACCTGTAAGCAAG	57.61	222	54/34
105	SSR07A05	*	EST/cDNA	LG2	CX663436		EST/cDNA	(cac)n	F9GCC	ctagtattaggacgccTCTTTGAGCAATCTCATGTCTC	58.63	gtttctt	gtttcttCAGGATTAGGGCTATTGCTG	58.28	140	56/32
106	SSR07A07	*	EST/cDNA	LG4	CX663643		EST/cDNA	(gaa)n	F9CCG	ctagtattaggacccgTGCCTACTTAAGGAAGAATCAG	57.74	gtttctt	gtttcttGGAGGTTCTTTATCTTTCTTGG	56.90	176	58/34
107	SSR07A14	*	EST/cDNA	--	CX666503		EST/cDNA	(att)n	F9TAC	ctagtatcaggactacGCAAATATGTGGCTTAAGCCT	59.46	gtttctt	gtttcttCAGGTCTCTTCATCATCCAC	57.86	141	54/32
108	SSR07A17		EST/cDNA	LG2	CX667444		EST/cDNA	(atg)n	F9AGG	ctagtattaggacaggTGGCGTTCAATCATCATAACAG	59.55	gttt	gttTCCTCATCATTCTCCCATCG	59.14	223	56/32
109	SSR07A19	*	EST/cDNA	LG3	CX669071		EST/cDNA	(cat)n	F9GAC	ctagtatcaggacgacGTTTGTGTTTATACTTCCTGTG	55.78	gtttctt	gtttcttGAAGATCAGAAGAGTGATTGAG	56.48	129	56/34
110	SSR07A23	*	EST/cDNA	LG7	CX670335		EST/cDNA	(ca[ct])n	F9TAC	ctagtatcaggactacATGCCACCAAACCAAATATCC	59.38	gttt	gtttCTAATTTCCGACACTTTCCCTG	59.49	192	56/32
111	SSR07A24	*	EST/cDNA	LG9	CX670407		EST/cDNA	(aag)n	F9TAC	ctagtatcaggactacCTTACAAGCCTCTTCACTACAG	58.64	gtttctt	gtttcttAACATCCTACTACAGATCAGCA	58.86	168	54/34
112	SSR07A20	*	EST/cDNA	LG5	CX669338		EST/cDNA	(ttc)n(tac)n(gac)n	F9GTC	ctagtatgaggacgtcCCTAATGACTTCATGACCCT	59.37	gttt	gtttCGATACTCGCATATCTTGCC	58.83	201	58/32
113	SSR07B01	*	EST/cDNA	LG1	CX294454		EST/cDNA	(ct)n	F9GAC	ctagtatcaggacgacTCGTCGTAATTTCCCAACCT	62.45	gttt	gtttCTATCTATCTGTCTATAGCCGTC	60.28	137	54/32
114	SSR07B05		EST/cDNA	--	CK936720		EST/cDNA	(ctt)n	F9TCC	ctagtatcaggactccTGTCCCTTTCATCATCGTTA	58.73	gttt	gtttCCACCAGCATCACTATCAAA	60.05	177	58/32
115	SSR07B08	*	EST/cDNA	LG3	CB292573		EST/cDNA	(gcg)n	F9GAC	ctagtatcaggacgacTATTACGCATCAAGCATCACGA	62.53	gtttct	gtttcttCTAATCCGACATTGGTAGTGAG	60.31	192	54/30
116	SSR07B09		EST/cDNA	LG9	CB292707		EST/cDNA	(cgc)n	F9GTC	ctagtatgaggacgtcTTTAGCACGTGTTTGTTCTG	59.28	gtttct	gtttcttCCGTTGGATTATCAGATGGG	60.27	203	54/34
117	SSR07B14	*	EST/cDNA	LG7	CN183851		EST/cDNA	(ctt)n	F9GAC	ctagtatcaggacgacTTTCGCTCACTACTTGACTG	61.60	gttt	gtttGGCTTTATGTCCGATTCTG	61.36	215	56/32
118	SSR07B27	*	EST/cDNA	LG5	CX671829		EST/cDNA	(cct)n	F9GAC	ctagtatcaggacgacTACTTGGTAGACCAAGAACAG	59.07	gtttct	gtttcttGAAGGCAGAAACTTGTTCCT	60.49	191	54/32
119	SSR07B25		EST/cDNA	LG9	CX074011		EST/cDNA	(ga)n	F9GTC	ctagtatgaggacgtcAGGCTATCTCTAATCCATTGCT	60.86	gttt	gtttGCCATTGCTGTTCATTTACTC	60.19	211	54/32
120	SSR07B30		EST/cDNA	LG5	CK665183		EST/cDNA	(ta)n	F9GTC	ctagtatgaggacgtcCAGTAATGGAGAAGTTGTTCCT	60.04	gtttct	gtttctTGGCAAACAATGCTATTCCT	60.27	185	56/32
121	SSR08A02	*	EST/cDNA	--	DY267483		EST/cDNA	(ttta)n	F9GTC	ctagtatgaggacgtcTACCTATTTCTTCTTGTTCCC	57.16	gttt	gtttAACAGTGAAATTGTACGGA	57.69	150	54/32
122	SSR08A06	*	EST/cDNA	LG1	DY304961		EST/cDNA	(ttta)n	F9GTC	ctagtatgaggacgtcGTTATTTCAAGATTAGGTGTCAG	57.50	gtttctt	gtttcttATAGAGCCAACATCAATTCCC	60.34	126	58/32
123	SSR08A07		EST/cDNA	--	DY257192		EST/cDNA	(caga)n	F9TAC	ctagtatcaggactacAATATTCCAGCAGACAGACAG	59.99	gtttctt	gtttcttAATTTGCTTGTTCTCGGAGG	61.02	123	58/32
124	SSR08A09	*	EST/cDNA	LG2	CX663959		EST/cDNA	(cagg)n	F9GCC	ctagtattaggacgccCATCATTGCATCAGCATCAC	60.08	gtttctt	gtttcttAGTTCTACATCATAACCTGCC	59.71	116	56/32
125	SSR08A14		EST/cDNA	LG2	DN618700		EST/cDNA	(aaat)n	F9GAC	ctagtatcaggacgacTTCGGTCCAATCATAGCATC	60.01	gtttctt	gtttcTTGGGCTTTCCTTGTTCTTC	60.88	186	58/32
126	SSR08A15	*	EST/cDNA	LG5	CK665717		EST/cDNA	(aaat)n	F9TCC	ctagtatcaggactccATATTTACGCAAAGTGGAAGT	59.51	gttt	gtttATTATTCCCATCAATCTCAAATG	57.25	141	58/32
127	SSR08A16	*	EST/cDNA	LG5	DN134966		EST/cDNA	(acga)n	F9GTC	ctagtatgaggacgtcCATATTTACGCAAAGTGGAAGT	59.51	gtttctt	gtttctTATTATTCCCATCAATCTCAAATG	57.22	145	56/32
128	SSR08B02		EST/cDNA	LG6	DY300589		EST/cDNA	(aat)n	F9GAC	ctagtatcaggacgacTGCTGCTAGGACTACTCTC	61.13	gttt	gtttCATTTGAGCAAAGGGTGAAGG	62.11	126	56/32
129	SSR08B06	*	EST/cDNA	LG9	DY298942		EST/cDNA	(cct)n	F9CCG	ctagtattaggacccgGTAATCGTCCCGATCAAGGAG	62.36	gtttctt	gtttcttGCGGACTTGGTAAGTAACGA	62.19	121	60/32
130	SSR08B13		EST/cDNA	LG9	DY295447		EST/cDNA	(cct)n	F9GTC	ctagtatgaggacgtcGTAATCGTCCCGATCAAGGAG	62.51	gtttctt	gtttcttGCGGACTTGGTAAGTAACGA	62.29	121	56/32
131	SSR08B15	*	EST/cDNA	LG7	DY292389		EST/cDNA	(acc)n	F9TAC	ctagtatcaggactacCCCATACTTGAAACCAAACC	59.14	gtttctt	gtttcttAGCAGCATGACTTAATCCA	59.01	106	56/32
132	SSR08B20	*	EST/cDNA	LG9	DY291354		EST/cDNA	(att)n	F9GTC	ctagtatgaggacgtcGCTGTTACAACATTGTTTGG	61.07	gttt	gtttACAGTCCGCAGCATTACAC	62.95	152	56/32
133	SSR08B21	**	EST/cDNA	LG2	DY290555		EST/cDNA	(acc)n	F9GCC	ctagtattaggacgccATGCTACTCATCACCTATTCTT	58.95	gttt	gtttCTTGGCGGCTTTAGAATTG	59.51	130	60/32
134	SSR08B22		EST/cDNA	LG7	DY289533		EST/cDNA	(aat)n	F9TAC	ctagtatcaggactacTTTATCAATAACTCTGCTACCC	58.94	gttt	gtttACGGCCATATTCACTCTAAA	58.90	138	56/32
135	SSR08B25	**	EST/cDNA	LG9	DY289996		EST/cDNA	(att)n	F9AGG	ctagtattaggacaggCGTTGACTCTCAAGAGATCTG	60.19	gttt	gtttCTTCACAGTCCGCAGCATTA	62.84	123	56/32
136	SSR08B27	*	EST/cDNA	LG6	DY288475		EST/cDNA	(ctca)n(ctcg)n	F9AGG	ctagtattaggacaggCCAACTCATCATCGTCATCTC	60.59	gtttctt	gtttcttGCTTAATATTCATTACCTCGCAG	59.67	101	60/32
137	SSR08B28	*	EST/cDNA	LG2	DY286595		EST/cDNA	(aat)n	F9GAC	ctagtatcaggacgacACTCTCCCACTGTTTATGACTG	61.78	gtttctt	gtttcttAGTACAAGTTGCCTTTGCGT	62.95	181	58/32
138	SSR08B29	*	EST/cDNA	LG6	DY285888		EST/cDNA	(cag)n	F9GTC	ctagtatgaggacgtcCTCCTCAGCAAGAGATCAC	59.46	gtttctt	gtttcttGCGGGTACTGATAGTACTG	58.93	160	56/32
139	SSR08B31		EST/cDNA	LG4	DY283748		EST/cDNA	(ggt)n	F9TAC	ctagtatcaggactacCACTGAAATTATTCACAATCGAG	58.18	gtttctt	gtttcttATGTCGAAACAGTTGAAGTC	58.41	129	56/32
140	SSR08B32	*	EST/cDNA	LG6	DY281946		EST/cDNA	(aat)n	F9GAC	ctagtatcaggacgacATATTCATTACCTCGCAGTGT	59.28	gttt	gtttGCCAACTCATCATCGTCAT	60.14	94	56/30
141	SSR08B35	**	EST/cDNA	LG2	DY287625		EST/cDNA	(cgc)n	F9AGG	ctagtattaggacaggCCTCTTCCTTCTACTTCTA	57.84	gttt	gtttATCCGTAAACTTCTCTTCGT	58.74	170	54/32
142	SSR08B38		EST/cDNA	LG3	DY283862		EST/cDNA	(cat)n	F9TCC	ctagtatcaggactccACTCCTTTAAATTATTCTCTGCT	57.63	gttt	gtttCCGAACAAATACAATGAATCTG	58.03	115	56/32
143	SSR08B40	**	EST/cDNA	LG2	DY280605		EST/cDNA	(aat)n	F9AGG	ctagtattaggacaggACTCTCCCACTGTTTATGACTG	61.98	gtttctt	gtttcttGCCTTTGCGTTCAACTGATAA	61.97	171	56/32
144	SSR08B44		EST/cDNA	LG3	DY276228		EST/cDNA	(ctca)n(ctcg)n	F9TAC	ctagtatcaggactacACTCCTTTAAATTATTCTCTGCT	57.87	gtttctt	gtttcttCCGAACAAATACAATGAATCTG	58.22	118	54/34
145	SSR08B47		EST/cDNA	LG3	DY272433		EST/cDNA	(aat)n	F9CCG	ctagtattaggacccgACTCCTTTAAATTATTCTCTGCT	57.87	gttt	gtttGAATCCGAACAAATACAATGAA	57.54	119	56/32
146	SSR08B54	**	EST/cDNA	LG2	DY268149		EST/cDNA	(cgg)n	F9CCG	ctagtattaggacccgCACATCCTAACTCTCCCACT	60.74	gtttctt	gtttcttGCCTTTGCGTTCAACTGATA	61.37	180	58/32
147	SSR08B60	*	EST/cDNA	--	DY265343		EST/cDNA	(cgg)n	F9TAC	ctagtatcaggactacATTCATTGGCGGTGTATT	58.51	gttt	gtttCTCCCTCCAAATTAGATAACAG	58.17	133	56/32
148	SSR08B62	*	EST/cDNA	LG5	DY257536		EST/cDNA	(aaagtg)n(aagatg)n	F9TCC	ctagtatcaggactccTGACTCAAGTGATGAAGAGG	59.13	gttt	gtttACTGTCTTGGTCTTTGGCT	61.43	145	54/32
149	SSR08B64		EST/cDNA	LG4	CV716381		EST/cDNA	(ctt)n	F9GAC	ctagtatcaggacgacTTCAATGTCTTCTTCAACAAAG	58.71	gttt	gttTGATGGGAATAAGCTCTGT	57.98	147	54/32
150	SSR08B66	*	EST/cDNA	LG8	CV711633		EST/cDNA	(cga)n	F9GTC	ctagtatgaggacgtcAGAGACAAATCAACCGAC	58.47	gttt	gtttAAGCACAACAATCTTCCTC	57.96	111	58/32
151	SSR08B68	*	EST/cDNA	LG3	CV709642		EST/cDNA	(atggga)n	F9AGG	ctagtattaggacaggTTATGGAGGTATGAATCAG	57.76	gttt	gtttGGCTCATTCCCATATTCCC	59.95	125	58/32
152	SSR08B75	*	EST/cDNA	LG4	CX296800		EST/cDNA	(gttt)n	F9TCC	ctagtatcaggactccTTAGAGATGTGAGTGACTGA	58.59	gttt	gtttGATACAACCAACCGAGTTC	58.12	191	54/34
153	SSR08B78	*	EST/cDNA	LG7	CX639446		EST/cDNA	(aat)n	F9AGG	ctagtattaggacaggACGGCCATATTCACTCTAAA	58.80	gtttctt	gtttcctTTATCAATAACTCTGCTACC	58.71	142	56/32
154	SSR08B82		EST/cDNA	LG6	CX077953		EST/cDNA	(att)n	F9GAC	ctagtatcaggacgacTGAGCAAAGGGTGAAGGAG	61.83	gttt	gtttCAACCGTTCAAGAAAGCAT	58.93	169	60/32
155	SSR08B84	*	EST/cDNA	LG5	CX076164		EST/cDNA	(ctt)n	F9GCC	ctagtattaggacgccCTCTAACCACTCTCATATCCC	58.79	gtttctt	gtttcttGCTGTCAAGTCATTTGGAG	58.71	153	54/32
156	SSR08B85	*	EST/cDNA	LG7	CX072241		EST/cDNA	(ctg)n	F9CCG	ctagtattaggacccgAGAGATGGCTTTGAATAAACTGA	60.10	gtttctt	gtttcttAATGGAGGGAGTGGCTATG	61.07	191	60/32
157	SSR08B88	*	EST/cDNA	LG6	CX044303		EST/cDNA	(ggt)n	F9AGG	ctagtattaggacaggACTCGTCGTAGAATCATTAGTG	59.72	gtttctt	gtttcttGCCAACTCATCATCGTCAT	60.14	115	56/36
158	SSR08B90	*	EST/cDNA	LG8	CK934959		EST/cDNA	(aaag)n	F9GAC	ctagtatcaggacgacTGAACCAACTTACACGCAC	60.81	gtttctt	gtttcttACTGAGTGAATTGCGAACA	59.83	174	56/30
159	SSR08B92	*	EST/cDNA	LG8	CF509535		EST/cDNA	(aac)n(agc)n	F9GTC	ctagtatgaggacgtcCTAACCCAGATGCTCTAACTC	59.63	gtttctt	gtttcttATATCTGTCGAGATTTCCTCC	58.79	133	56/32
160	SSR08B95	*	EST/cDNA	LG4	BQ623755		EST/cDNA	(agg)n	F9AGG	ctagtattaggacaggTTAAGAGCAAGTCGTGG	60.66	gttt	gtttAAATCTTTGTTTCTGAGGACCC	60.84	167	56/32
161	SSR11A06	*	EST/cDNA	LG6	DN621103		EST/cDNA	(gcc)n	F9AGG	ctagtattaggacaggTCTCAATCCACAATCTCTCTC	57.08	gtttctt	gtttcttGAGAAAGTATCAGTTGTGTCG	57.08	259	56/32
162	SSR11B14		EST/cDNA	LG4	CX672695		EST/cDNA	(cag)n	F9CCG	ctagtattaggacccgTTCAAACCGCAAATATCGAG	57.61	gtttctt	gtttcttAATTGAGGCATTGATTGCTG	57.49	242	58/32
163	SSR11A27	*	EST/cDNA	LG7	FC889066		EST/cDNA	(gcagaa)n	F9GCC	ctagtattaggacgccTTCTTGTTTACGAGTTCATGC	57.34	gtttctt	gtttcttAATATGTTCGTTGACTTGACG	57.34	237	56/32
164	TSGR901	**	EST/cDNA	LG9	BQ624820		EST/cDNA	(gt)n	F9TAC	ctagtatcaggactacTTAGTGTTGTAGCATTCTCT	57.41	gttt	gtttGATGAATCGATCTTGCTTTAGT	58.60	152	56/32
165	TSGR904	*	EST/cDNA	LG3	CX070091		EST/cDNA	(gct)n	F9CCG	ctagtattaggacccgTGTTGTGTAAGATGCAAGTG	62.60	gttt	gtttCCTATGGGCACTTAGTCCTG	61.73	133	56/32
166	TSGR905	*	EST/cDNA	LG4	DR909865		EST/cDNA	(tg)n	F9TCC	ctagtatcaggactccACATTAGAAGAGGCATTTCC	59.01	gttt	gtttGTCAAAGCATCCCATCAAA	58.30	246	62/32
167	TSGR906		EST/cDNA	LG8	EY706514		EST/cDNA	(att)n	F9GAC	ctagtatcaggacgacATCTGCTTTAATTATGTTCTGT	57.24	gttt	gtttAGTGCATTAACATTCCACCA	59.90	102	58/32
168	TSR8EM15	*	EST/cDNA	LG7	DY287852		EST/cDNA	(cca)n	F9GAC	ctagtatcaggacgacAATCCGAGTTCCCAGAAGA	60.21	gtttctt	gtttcttGAAACACCTTGACCTATATGCT	60.17	279	56/32
169	TSR8EM24	*	EST/cDNA	LG3	EY700381		EST/cDNA	(cat)n	F9GTC	ctagtatgaggacgtcCCTTAATCGGAACAAATCATCGG	61.97	gtttctt	gtttcttAAGACACAGCCAGATCTTCC	61.88	156	56/32
170	TSR8EM28		EST/cDNA	LG3	EY804307		EST/cDNA	(atc)n	F9TAC	ctagtatcaggactacGCCAACTTGTGTAATCTTCT	58.09	gtttctt	gtttcttCAACAGAGAACCCGAAGAAT	59.98	144	54/32
171	TSRB07		EST/cDNA	LG1	DY281096		EST/cDNA	(agc)n	F9GCC	ctagtattaggacgccACCAGTACAGTTATTTAATCCC	57.55	gtttctt	gtttcttACTCGTTTATTGGTTGCTG	58.33	169	60/32
172	TSRB09	*	EST/cDNA	LG7	CD573990		EST/cDNA	(aca)n	F9GTC	ctagtatgaggacgtcATCTTCTTACATCTTCCGCAC	60.19	gtttctt	gtttcttCTCTCAGCTATACGATCCGA	60.34	137	56/32
173	TSRB11	*	EST/cDNA	LG4	EY654447		EST/cDNA	(aga)n	F9AGG	ctagtattaggacaggCAATGGCGGGAACAAAGTC	61.93	gtttctt	gtttcttCATCAGCTGAATCATTCCCA	60.13	238	54/32
174	TSRB29	*	EST/cDNA	LG4	EY720405		EST/cDNA	(att)n	F9GTC	ctagtatgaggacgtcGCAGTTTCAGGAATTTCAACAC	60.89	gtttctt	gtttcttGAATCCAAGACCAAGAGCC	60.36	176	56/34
175	TSRB38	*	EST/cDNA	LG7	EY768192		EST/cDNA	(aca)n	F9GAC	ctagtatcaggacgacGTTCCTCTTGTTTGACACTTACC	61.67	gtttctt	gtttcttCAGAACTTGGTGTTGGTGG	61.12	217	54/32
176	TSRB39	*	EST/cDNA	LG2	EY666329		EST/cDNA	(aag)n	F9TAC	ctagtatcaggactacGTTGGTAAAGGAGACACTG	57.64	gtttctt	gtttcttGGGTCCTCAATCATTATGG	57.20	255	56/34
177	TSRF101	*	EST/cDNA	LG1	DY267528		EST/cDNA	(caa)n	F9GAC	ctagtatcaggacgacCATCACCACCAAAACAACGAC	60.58	gttt	gtttCAATGGGTGTCCCACTATCTC	60.58	167	56/32
178	TSRF105	*	EST/cDNA	LG7	DC895459		EST/cDNA	(aca)n	F9CCG	ctagtattaggacccgACCAAAAGCAAAAGCCATTCC	61.43	gttt	gtttGGGCTGAGAGAAGTTGTAGTTG	61.43	270	56/30

179	TSRF116	*	EST/cDNA	LG3	DC895047	EST/cDNA	(aac)n(gac)n	F9GCC	ctagtattaggacgccAGGCCAAGAACTGACAAATCC	60.87	gttt	gtttATCCATTTGGTGCCTACCATC	60.87	174	56/30
180	TSRF120		EST/cDNA	LG2	CX295530	EST/cDNA	(aag)n	F9GTC	ctagtatgaggacgtcAGATCGAGAGAGGGGAGAAAC	60.65	gttt	gtttATTCCCTGTCGCAGTAATAGC	60.65	197	60/32
181	TSRF124	*	EST/cDNA	LG7	EY705148	EST/cDNA	(agg)n	F9AGG	ctagtattaggacaggATGAGGTTACAGCCAAACCAG	61.05	gttt	gtttCTGTACTTGGAGCTGAAGGTG	61.05	235	60/32
182	TSRF136	*	EST/cDNA	LG5	FC877062	EST/cDNA	(aca)n	F9AGG	ctagtattaggacaggTGTTACCACTCCTCCAACCAC	60.99	gttt	gtttGCGGGTGATATAAACAGGAGG	60.99	213	56/32
183	TSRF138	*	EST/cDNA	--	DY272315	EST/cDNA	(aca)n(ata)n	F9GTC	ctagtatgaggacgtcACGAGTGTTGAAACTGTGTCC	60.78	gttt	gtttATATCCCGCAATCAGACGATG	60.78	177	60/32
184	TSRF144	*	EST/cDNA	LG2	DN618162	EST/cDNA	(acc)n	F9TAC	ctagtatcaggactacACAGATGTAGCAGCTCGAAAG	60.99	gttt	gtttAAACTTCTCTTCGTGTTCCGC	60.99	222	60/32
185	TSRF157	*	EST/cDNA	LG7	DY262980	EST/cDNA	(agc)n	F9TAC	ctagtatcaggactacGTTGTGTGAGAGTTACAATGC	61.20	gtttctt	gtttcttCAAGGCTGGTGGTATCAGATG	61.20	293	56/32
186	TSRF160	*	EST/cDNA	--	EY683407	EST/cDNA	(agc)n	F9CCG	ctagtattaggacccgTCAGAAAGAACATCATGGCCG	61.05	gtttctt	gtttcttGAGCTGAACATTTTGCCAAGG	61.05	214	54/32
187	TSRF161	*	EST/cDNA	LG8	EY651421	EST/cDNA	(agc)n	F9GAC	ctagtatcaggacgacGCTTGAAATGGCAACAAGACC	60.44	gtttctt	gtttcttGGCAACAATCTTTCCCAAGTC	60.44	253	56/32
188	TSRF162	*	EST/cDNA	LG7	DY296684	EST/cDNA	(agcc)n	F9TAC	ctagtatcaggactacTTCAGTTTCAAAGGAGGCCAG	61.06	gtttctt	gtttcttCACTTTCCCCACTTCCTCATC	61.06	190	58/32
189	TSRF164	*	EST/cDNA	--	DY265747	EST/cDNA	(agccaa)n	F9GCC	ctagtattaggacgccTTAGATACACAAGGGCCATGC	61.12	gtttctt	gtttcttGGGACTTGTTCGGTTATCCAG	61.12	229	54/32
190	TSRF167	*	EST/cDNA	--	EY863111	EST/cDNA	(att)n	F9GCC	ctagtattaggacgccGCTGCTGAATGAGTAAATCACG	61.48	gtttct	gtttctTCGACGAAAAACCAAGCTCC	61.48	175	58/32
191	TSRF169	*	EST/cDNA	LG9	FC913104	EST/cDNA	(ata)n	F9GAC	ctagtatcaggacgacGTGTAACAGCGAGTTCATTG	60.84	gtttctt	gtttcttAGATTAAAAATGGGCGTTGCG	60.84	310	56/32
192	TSRF172		EST/cDNA	LG4	DY297851	EST/cDNA	(ata)n	F9AGG	ctagtattaggacaggCTGATAACGCTTCCAACAAC	60.91	gtttctt	gtttcttGCGGTGGCACAAGATAAATTC	60.91	268	56/32
193	TSRF184	*	EST/cDNA	LG1	EY884078	EST/cDNA	(atc)n	F9CCG	ctagtattaggacccgATGGGCTCTTATACCTGGGAG	61.04	gtttctt	gtttcttATCATCATCGTCATCAGCGAC	61.04	258	56/34
194	TSRF187	*	EST/cDNA	--	EY650146	EST/cDNA	(atc)n	F9GTC	ctagtatgaggacgtcTAACGGAGGAGAACAGGAG	61.12	gtttctt	gtttcttCAGTCGTTGATGGTTAGGACC	61.12	237	62/32
195	TSRF191	**	EST/cDNA	LG3	CF507794	EST/cDNA	(atg)n	F9GAC	ctagtatcaggacgacGTTTGCTTCTTTCACCACCAC	61.72	gtttctt	gtttcttAGACGCACATCAAATCGAAGG	61.72	187	54/32
196	TSRF192	*	EST/cDNA	LG5	CX672882	EST/cDNA	(atg)n	F9TAC	ctagtatcaggactacAGCGGAAGAAGCGAAGAATAG	61.19	gtttctt	gtttcttGCTCCTTATCCGTTCTCCAAC	61.19	179	56/32
197	TSRF194	*	EST/cDNA	--	CX637983	EST/cDNA	(atg)n	F9TAC	ctagtatcaggactacGATGGTGGATACGACTATGC	61.47	gtttctt	gtttcttAGTTTATTCATGCAGCCGGAG	61.47	172	58/34
198	TSRF195	*	EST/cDNA	LG3	FC927104	EST/cDNA	(atgaaa)n	F9AGG	ctagtattaggacaggATGGGAGTTGATGCTCAGATG	61.19	gtttctt	gtttcttCTCTTCCGATGTCCCACAATC	61.19	167	58/32
199	TSRF198	*	EST/cDNA	LG3	EY849742	EST/cDNA	(atgggg)n	F9GAC	ctagtatcaggacgacTGGCAATGCAACAACAACTTC	61.00	gtttctt	gtttcttAGAAAGGAGGAAGCAATGGTG	61.00	167	56/32
200	TSRF204	*	EST/cDNA	LG5	EY673751	EST/cDNA	(caa)n	F9GCC	ctagtattaggacgccTCATCCGTGTTTTCCAAGCTC	61.13	gtttctt	gtttcttGCTCTCAAACCCCAGATTCTC	61.13	207	56/30
201	TSRF208	*	EST/cDNA	LG7	EY776018	EST/cDNA	(caa)n	F9GTC	ctagtatgaggacgtcCTCCAAACCAAAACCCTTC	61.19	gtttctt	gtttcttGTGGGTATGAAGATTCCGGTG	61.19	214	62/32
202	TSRF211	*	EST/cDNA	LG7	EY879492	EST/cDNA	(caacag)n	F9GAC	ctagtatcaggacgacGAGTAGCAACAAAGCATGTC	60.93	gtttctt	gtttcttAAACCCCTTTTAGCACCAGAG	60.93	243	60/32
203	TSRF215	*	EST/cDNA	LG3	CF507794	EST/cDNA	(cac)n	F9AGG	ctagtattaggacaggTTTACATGGATGAAGAGGCGG	61.72	gtttctt	gtttcttAGACGCACATCAAATCGAAGG	61.72	247	60/32
204	TSRF218	*	EST/cDNA	LG1	CV714512	EST/cDNA	(cag)n	F9CCG	ctagtattaggacccgTCGTCACCATCTTTCAACGTG	61.41	gtttctt	gtttcttATTTGAAGAACGGGTATGGGC	61.41	204	56/32
205	TSRF206	**	EST/cDNA	LG8	EY651421	EST/cDNA	(caa)n	F9GAC	ctagtatcaggacgacATGAATAGAGGGACGTTGCTG	61.62	gtttc	gtttcTTGTTGTCCCATCATCAAGCC	61.62	267	56/32
206	TSRF225	*	EST/cDNA	LG4	EY741460	EST/cDNA	(cag)n	F9AGG	ctagtattaggacaggTTTCTTCCATCCGCAAACAAC	60.65	gtttct	gtttctTCGCAGTAAGCCATAATCAGG	60.65	151	54/32
207	TSRF229	*	EST/cDNA	--	FC913104	EST/cDNA	(cag)n	F9TAC	ctagtatcaggactacAAAGATTCCCACAACGTCTCC	61.10	gtttctt	gtttcttCAGCTTGTAACATGAACGCAC	61.10	289	56/32
208	TSRF230	*	EST/cDNA	LG8	CB293840	EST/cDNA	(cag)n	F9GTC	ctagtatgaggacgtcGTAAGATCCTCTCAACAGCC	60.91	gtttctt	gtttcttGAAAGCTGTGTTGTGTTCACC	60.91	230	60/32
209	TSRF234	**	EST/cDNA	LG8	CB293840	EST/cDNA	(cag)n	F9CCG	ctagtattaggacccgATAATGGGGGATGGCAGTTC	61.47	gtt	gttTCTTTGCTGCTGCTGATATGG	61.47	203	60/32
210	TSRF237	*	EST/cDNA	--	DC886823	EST/cDNA	(ctgcgg)n	F9GTC	ctagtatgaggacgtcACCCGAATACCCACAAGAAAC	61.21	gtttctt	gtttcttCTAATGGGCTTTGGGAGATGG	61.21	186	54/32
211	TSRF238		EST/cDNA	--	EY770405	EST/cDNA	(caggtt)n	F9GCC	ctagtattaggacgccATCGGCTTCCGTTGAAAAAG	60.85	gtttctt	gtttcttGCACTTACAGGCTCTTCTCTC	60.85	183	54/32
212	TSRN214	*	EST/cDNA	LG2	DY271862	EST/cDNA	(can)n	F9TCC	ctagtatcaggactccCGCCAACAAGAAGATAACAAGAC	60.62	gtttctt	gtttcttTCTCTTTAACCCTACCTTGCTG	59.94	145	60/32
213	TSRP06	*	EST/cDNA	LG6	DY281841	EST/cDNA	(gaa)n	F9GAC	ctagtatcaggacgacAACAGCACAAACACGCAAA	61.94	gtttctt	gtttcttCAAGTACATGAACACGCAC	59.40	208	60/32
214	TSRP07	*	EST/cDNA	LG2	EY812469	EST/cDNA	(ag)n	F9GTC	ctagtatgaggacgtcATCACCAACTTATATTTGCTCAT	58.29	gtttctt	gtttcttCGACCTATCATAGAAAGCACA	59.63	161	60/32

B. SSR markers used in this study

#	Marker	Cert.	Reference	LG	Accession ID	Tag	Tagged forward primer	tail	Tailed reverse primer	Amp	Program
215	CiBE0246	*	1)	LG5	ET089421	F9GCC	ctagtattaggacgccATTTGAGTTGTGTTGAGGTTG	gtttctt	gtttcttCGGTGACGAAGAGTATGATT	233	56/32
216	CiBE0447	*	1)	LG6	ET091388	F9CCG	ctagtattaggacccgCACAAAGAGAGTAACCCACAA	gtttctt	gtttcttCGTCAAGAAGAGAGAATGATG	326	56/32
217	CiBE1644	*	1)	LG3	ET097780	F9GCC	ctagtattaggacgccACAGAAGAGGAGCCATTATTT	gtttctt	gtttcttCAGAGAGAACCCGAAGAAG	383	56/32
218	CiBE2165	*	1)	LG7	ET102021	F9GTC	ctagtatgaggacgtcAATCCACTCTCAAACACCAG	gtttctt	gtttcttAACTGCCAAATAACTACCATTC	286	56/32
219	CiBE2227	*	1)	LG8	ET100673	F9TAC	ctagtatcaggactacAGAACCAAGTCACCTCACAG	gtttctt	gtttcttAGAGAAGGATAGGGATGGAA	176	54/32
220	CiBE4265	*	1)	LG2	ET106965	F9AGG	ctagtattaggacaggCACACTGCTAAACCGACAC	gtttctt	gtttcttATACTCAACACGACACGAGAA	254	56/32
221	CX0004	*	2)	LG5	CK933007	F9CCG	ctagtattaggacccgAAACCCCACTTCACAGCAAC	gttt	gtttGAAAGCGAGCCTTTGATGTC	276	56/30
222	CX0010		2)	LG1	BQ623170	F9TAC	ctagtatcaggactacAACCGAAGATGGAGGGAACT	gtttctt	gtttcttACATTCATGGCCACATCTCA	228	58/32
223	CX0035	*	2)	LG3	CD575772	F9GTC	ctagtatgaggacgtcCCATTAACGAGAAAACCAAACA	gtttctt	gtttcttCAAAAAGGGGTTGCAAAGAA	189	56/34
224	CX2004	*	2)	LG2	CF653229	F9CCG	ctagtattaggacccgAAACCGCCGAAACTTTTACA	gtttctt	gtttcttGGAATCCCAAAGAAAGTTGG	202	56/32
225	CX2018		2)	LG2	CF507198	F9GTC	ctagtatgaggacgtcAAGAGATCGAAAGAGCAAGAGA	gtttc	gtttcTTTGAGATTGATGTGAGCCG	179	56/32
226	CX2021		2)	LG2	CK739933	F9TAC	ctagtatcaggactacAAGGTCATGTCTTTAGCACTTTGA	gtttctt	gtttcttCAAGTTGCCAATTCAGGAGG	147	58/32
227	CX2040	*	2)	LG3	CK932713	F9CCG	ctagtattaggacccgACAGCCTCCCCACTTTCTTT	gtttctt	gtttcttGATTAGTTTTTGCTTCGCCG	112	56/32
228	CX3001	*	2)	LG3	CO913029	F9GAC	ctagtatcaggacgacAAAAACAATGAATGGAGGCG	gttt	gtttGAGACTCAACCCAAGACCCA	432	56/32
229	CX3040		2)	LG9	CN190783	F9TCC	ctagtatcaggactccAAGCGATGCAACAGATACCC	gtttctt	gtttcttCAGCAGCAACAAGTCCTTCA	308	60/32
230	CX4005	*	2)	LG3	CK938465	F9AGG	ctagtattaggacaggACACACCAAACTTTGCCCTC	gtttct	gtttctTGAAAATGGATGTGCGGTAA	372	54/34
231	CX4008		2)	LG4	CK933430	F9GAC	ctagtatcaggacgacACCTCACTCACCCACACTCC	gtttct	gtttctTGAGGCTAGCGACTGGTTTT	394	58/32
232	CX4040		2)	LG2	CF834831	F9TAC	ctagtatcaggactacCGAAAGAGGCAAGAATACCG	gtttctt	gtttcttGCACTTCTCATGACCCAAAAA	368	56/32
233	CX5022	**	2)	LG7	CD574077	F9TAC	ctagtatcaggactacCTCTCGATCCACTGCAAACA	gtttctt	gtttcttAGCCAGCCAAAGCAATCTAA	255	56/32
234	CX5039	*	2)	LG7	CF833361	F9AGG	ctagtattaggacaggTCGGCAATAATTTCATTTTATTCA	gtttct	gtttctTCGATGAATTCGGAGGAATC	443	56/32
235	CX6024	*	2)	LG6	C95505	F9AGG	ctagtattaggacaggCAGGCTGCTGCTTCTTCTTC	gtttct	gtttctTCTCGCGATTGTTGAGTTTG	203	56/32
236	CX6035	*	2)	LG7	CD573989	F9GTC	ctagtatgaggacgtcCTTGCCAGGGTCTCAGTCTC	gtttctt	gtttcttCTCGGCATCAGGTTTACCAT	258	56/32
237	CX6037	*	2)	LG7	DR909803	F9TAC	ctagtatcaggactacGAAGGGGACTATGGCAACAA	gtttct	gtttctTGGTCTTCACCCTCTCCATC	177	58/32
238	CX6F21	*	2)	LG1	DN621499	F9GAC	ctagtatcaggacgacCTACAAGTTCCCCAGTTATCCCG	gtttctt	gtttcttACTTGACCCGCTCTAGGAGTGAC	163	58/32
239	CX6F32		2)	LG5	CF506971	F9TAC	ctagtatcaggactacACCCTCTCTTAGTGGGCAGTCTTT	gttt	gtttACAAAGAAAGGGATTGTGACGGTA	166	56/32
240	F02	*	3)	LG5	BQ623425	F9TAC	ctagtatcaggactacAACAGTGTAGCATCGCACTTTCAC	gtttctt	gtttcttGATACAAGGGACTTGCCCATCTC	178	56/32
241	F03	**	3)	LG5	DR912164	F9GCC	ctagtattaggacgccCGAGGATGACTCAAGTGATGAAGA	gtttctt	gtttcttCTTGGTCTTTGGCTTTTTCTCAG	146	54/32
242	F16	**	3)	LG7	DR909803	F9GAC	ctagtatcaggacgacGTCTTCACCCTCTCCATCTTCATC	gtttctt	gtttcttGGGACTATGGCAACAATAACTCCA	172	52/34
243	F21		3)	LG1	DN621499	F9GTC	ctagtatgaggacgtcCTACAAGTTCCCCAGTTATCCCG	gtttctt	gtttcttACTTGACCCGCTCTAGGAGTGAC	163	52/34
244	F40	*	3)	LG7	CV716690	F9GCC	ctagtattaggacgccTGTTTGTTACAAGGCAAGGCAAG	gtttctt	gtttcttCTGCAGAGGAGCTTCCTTACTCAA	104	56/32
245	F50	*	3)	LG4	CN183579	F9GAC	ctagtatcaggacgacAGGACGATTTCAGGTTCAGACTTG	gtttctt	gtttcttGAGCAAAAATCGAAGAGATCAAGC	165	54/34
246	F79	*	3)	LG2	BQ624534	F9GCC	ctagtattaggacgccGTGGCTGCAAGACTTCTTGTGTAG	gtttctt	gtttcttATAGATGTGGCAAGCAGAGCAAG	176	58/32

Cert: certified markers by error check with trio sets. *) certified and used for genetic analysis. **) certified, but rejected from genetic analysis due to overlap to other certified marker. No symbol: not certified due to error on certain trio.
Category: gSSR - designed from genomic SSR, EST/cDNA - designed from public EST or cDNA sequence. INDEL: genomic indel.
LG: Linkage group of this marker on one or two cross population(s).
Source:
Tag: type of BS tag (Shimizu and Yano, 2012).
Tail: pig tail sequence (xxxx).
Amp: amplicon size with tag sequence.
Program: anneal temperature/PCR cycles
Reference: 1) Ollitrault, F. et al. (2010) Am. J. Bot. e124-e129.
2) Chen, C. et al. (2008) Tree Genet. Genom. 4: 1-10.
3) Chen, C. et al. (2006) Theor Appl Genet. 112:1248-1257.
