# Supplementary material for: Hybrid Origins of Citrus Varieties Inferred from DNA Marker Analysis of Nuclear and Organelle Genomes
Source: PLoS One. 2016 Nov 30;11(11):e0166969. doi: 10.1371/journal.pone.0166969 (PMC5130255; doi:10.1371/journal.pone.0166969)
Supplement: S3 Table — (PDF) [file pone.0166969.s006.pdf]

S3 Table. Genotypes of 373 plant samples obtained with 246 preliminary selected markers

ID	Rep	Sample name	Strain	#	1	2	3	4	5	6	7	8	9	10	11	12	13	14	15	16	17	18	19	20	21	22	23	24	25	26	27
				Certified marker	*	**		*		**		*	*		**	*	*	*			*	*	*	*							
					CTVR01	CTVR04	CUBER403	CUBER410	CUBER601	CUBER602	CUBER903	CUBER918	CUBER920	CUBER925	CUBER935	CUBER939	CUBER951	GRW2011	GRW2051	GRW3021	GRW3011	GSR10S09	GSR10S17	GSR10S29	GSR2101	GSR2103	GSR2108	GSR2129	GSR2131	GSR2150	GSR3114
				ND	0	0	11	0	0	0	0	0	2	1	0	0	1	0	0	3	0	0	0	0	0	0	2	0	1	2	0
A001	*	Andoukan		1	298/313	299/314	252/252	253/264	159/165	225/228	174/175	96/102	158/162	165/168	171/177	205/214	155/158	119/120	96/96	102/114	87/90	193/193	149/161	133/137	106/110	183/193	174/177	136/148	135/141	217/235	137/156
A002	*	Anseikan		4	286/298	287/299	252/252	272/272	159/171	219/228	175/175	99/105	158/158	165/168	171/180	205/214	149/158	120/120	106/106	114/114	90/90	193/193	161/161	137/137	101/110	197/217	160/173	140/148	129/135	217/235	138/174
A003	*	Asahikan		0	298/313	299/314	234/234	253/272	159/171	219/228	174/175	96/102	158/162	165/168	171/180	205/214	149/158	119/120	92/92	102/114	87/90	193/193	124/161	133/137	108/110	183/197	173/174	148/156	135/141	217/235	137/144
A004	*	Banpeiyu		3	298/298	299/299	nd.	272/272	159/159	228/228	175/175	96/96	158/158	168/168	171/171	205/213	158/158	120/120	92/93	114/114	90/93	193/193	161/161	137/137	108/108	185/213	177/177	158/158	135/135	217/217	144/174
A005	*	Ben Di Zao		0	307/313	308/314	234/247	253/260	159/165	219/231	146/174	99/102	162/162	165/165	177/180	209/214	149/161	119/119	96/100	114/114	87/87	193/193	124/149	133/133	110/157	209/209	170/174	136/148	135/141	216/217	135/148
A006	*	Bergamot		2	298/313	299/314	252/252	253/268	165/165	228/234	171/174	102/105	158/158	168/168	177/177	205/205	158/164	120/120	92/100	114/114	87/90	193/193	161/167	133/137	106/106	193/210	174/181	138/170	142/142	213/234	137/137
A007		Bendi Guangju	(Honchi Kokitsu)	0	298/313	299/314	234/252	253/272	159/165	219/231	146/175	102/105	162/162	165/165	177/180	209/214	149/161	119/120	92/100	102/114	87/90	193/193	124/161	133/137	110/157	183/209	174/177	148/148	129/141	218/235	137/174
A008	*	Binkitsu		0	298/307	299/308	252/252	253/272	165/171	225/228	174/175	99/105	158/162	165/168	171/177	205/214	155/158	119/120	98/100	114/114	87/90	193/193	124/149	133/133	106/110	189/209	170/177	136/148	129/131	218/223	135/174
A009	*	Clementine		0	301/313	302/314	252/252	253/272	159/165	231/231	174/174	102/105	162/162	165/165	171/177	209/213	161/161	119/120	100/100	114/114	87/87	191/193	124/149	133/133	135/157	183/209	177/185	142/158	135/152	235/235	148/148
A010		Clementine	A Peau Fin	0	301/313	302/314	252/252	253/272	159/165	231/231	174/174	102/105	162/162	165/165	171/177	209/213	161/161	119/120	100/100	114/114	87/87	191/193	124/149	133/133	135/157	183/209	177/185	142/158	135/152	235/235	148/148
A011		Clementine	Caffin	0	301/313	302/314	252/252	253/272	159/165	231/231	174/174	102/105	162/162	165/165	171/177	209/213	161/161	119/120	100/100	114/114	87/87	191/193	124/149	133/133	135/157	183/209	177/185	142/158	135/152	235/235	148/148
A012		Clementine	de Nules	0	301/313	302/314	252/252	253/272	159/165	231/231	174/174	102/105	162/162	165/165	171/177	209/213	161/161	119/120	100/100	114/114	87/87	191/193	124/149	133/133	135/157	183/209	177/185	142/158	135/152	235/235	148/148
A013	*	Cleopatra		0	301/313	302/314	252/252	253/253	130/130	228/231	174/174	102/102	162/170	165/165	174/177	209/213	158/161	120/120	96/100	114/114	87/87	193/193	149/149	133/133	116/157	189/207	177/187	148/156	142/152	216/229	137/148
A014	*	Cravo mandarin		0	313/313	314/314	252/252	253/253	165/171	231/231	174/175	102/102	162/162	165/165	171/177	209/214	161/161	119/120	96/100	114/114	87/87	191/193	124/149	133/133	110/135	189/209	174/185	142/148	135/152	235/235	148/148
A015	*	Dada		0	286/301	287/302	244/252	253/268	165/171	231/231	174/175	102/105	158/162	165/168	171/177	205/218	161/161	120/120	104/104	114/114	90/90	193/193	149/161	133/137	116/155	189/195	160/181	140/142	135/139	215/215	135/156
A016	*	Dancy		0	301/313	302/314	252/252	253/260	159/165	219/231	174/174	102/102	162/162	165/165	177/177	209/214	149/161	119/120	96/100	102/114	87/87	191/193	149/149	133/133	110/157	209/209	174/174	148/148	135/141	235/235	137/148
A017		Dancy	Obenimikan	0	301/313	302/314	252/252	253/260	159/165	219/231	174/174	102/102	162/162	165/165	177/177	209/214	149/161	119/120	96/100	102/114	87/87	191/193	149/149	133/133	110/157	209/209	174/174	148/148	135/141	235/235	137/148
A018	*	Egami buntan		5	298/304	299/305	nd.	272/272	159/159	231/231	174/175	96/96	nd.	168/168	171/171	205/205	161/161	120/120	106/106	114/114	90/90	193/193	161/161	137/137	106/106	185/217	173/177	148/158	129/135	217/217	174/174
A019	*	Fukure mikan		0	301/313	302/314	252/252	253/253	165/165	219/231	156/174	102/102	162/162	165/165	177/180	209/214	149/161	119/120	98/100	102/102	87/90	193/193	149/149	133/133	110/157	183/209	174/181	148/158	131/141	220/235	135/135
A020		Fukushukan		1	301/313	302/314	252/252	253/268	165/171	228/234	174/175	99/102	162/162	165/165	171/177	214/218	158/164	120/120	100/102	114/114	90/90	193/193	149/161	133/137	89/110	193/199	181/183	138/140	135/139	213/235	135/137
A021	*	Funadoko		0	286/313	287/314	234/247	253/253	159/165	228/231	174/174	96/99	162/162	165/168	171/180	205/214	158/161	119/120	92/100	102/114	87/90	193/193	124/149	133/133	106/110	209/217	170/177	136/158	129/141	217/235	138/148
A022	*	Genshokan		1	313/313	314/314	243/252	253/260	165/165	219/231	174/174	102/102	162/162	165/165	171/177	213/214	149/161	120/120	96/100	102/114	87/87	191/193	124/149	133/133	103/124	183/189	174/177	154/158	135/142	221/221	148/148
A023	*	Girimikan		1	301/313	302/314	249/252	253/260	159/165	231/234	174/175	99/102	158/162	165/168	177/180	214/214	161/164	120/120	92/102	114/114	90/90	193/193	137/149	133/133	89/110	189/197	158/162	152/162	141/142	220/220	135/135
A024	*	Grapefruit	Marsh	0	298/313	299/314	252/252	253/272	159/171	228/231	174/175	96/105	158/162	165/168	171/177	205/214	158/161	120/120	93/100	114/114	90/97	193/193	161/161	137/137	106/110	209/213	173/177	158/158	139/152	229/235	148/174
A025		Grapefruit	Red blush	0	298/313	299/314	252/252	253/272	159/171	228/231	174/175	96/105	158/162	165/168	171/177	205/214	158/161	120/120	93/100	114/114	90/97	193/193	161/161	137/137	106/110	209/213	173/177	158/158	139/152	229/235	148/174
A026		Grapefruit	Triumph	0	298/313	299/314	252/252	253/272	123/130	228/231	174/175	96/105	158/162	165/168	171/177	205/214	158/161	120/120	93/100	114/114	90/97	193/193	161/161	137/137	106/110	209/213	173/177	156/158	139/152	229/235	148/174
A027	*	Hanayu		2	301/304	302/305	239/239	253/253	165/171	225/234	174/175	99/102	162/162	165/165	171/177	196/214	155/164	119/120	96/98	114/114	90/100	193/193	137/149	133/133	89/127	193/199	181/185	142/162	131/133	235/235	135/144
A028	*	Hassaku		0	286/313	287/314	234/234	253/272	159/159	228/231	174/175	96/102	158/162	165/168	171/177	205/214	158/161	119/120	100/106	102/114	87/90	193/193	124/161	133/137	106/110	183/185	174/177	148/148	135/141	217/235	137/138
A029		Hebesu		0	298/304	299/305	234/239	253/253	159/165	231/231	174/177	99/105	162/162	165/165	171/180	196/214	161/161	119/119	96/100	102/114	87/100	193/193	124/149	133/133	110/125	183/191	174/185	142/148	133/141	235/235	144/174
A030	*	Henka mikan		0	313/326	314/327	234/244	253/253	165/171	219/231	174/175	99/102	162/162	165/165	171/177	196/209	149/161	120/120	96/100	102/114	87/90	193/193	124/137	133/133	125/157	183/191	174/177	148/154	129/133	235/235	135/137
A031	*	Hickson	(Limb sport of 'Ellendale')	0	313/326	314/327	243/243	253/253	165/171	219/231	171/174	102/102	162/162	165/165	177/177	209/213	149/161	120/120	100/100	114/114	87/90	191/193	124/149	133/133	110/135	183/209	174/177	148/158	135/152	218/218	135/148
A032	*	Hirado buntan		0	298/304	299/305	244/244	268/272	159/159	225/231	175/175	96/105	158/158	168/168	171/171	213/213	155/161	120/120	106/106	114/114	90/90	193/193	161/161	137/137	108/116	213/217	173/173	164/164	129/135	217/217	138/144
A033	*	Hiroshimanatsubuntan		0	298/313	299/314	244/249	253/260	159/165	228/231	133/175	96/102	158/158	165/168	171/177	209/218	158/161	120/120	100/106	114/114	87/90	193/193	124/161	133/137	108/110	195/210	174/177	148/168	135/135	216/217	137/144
A034		Houraikan		0	304/313	305/314	234/252	253/272	165/171	219/231	174/175	102/105	162/162	165/165	171/177	209/214	149/161	119/120	92/100	102/114	87/90	193/193	124/161	133/137	110/157	183/209	173/174	144/148	129/141	235/235	137/141
A035	*	Hyoukan		1	298/313	299/314	234/252	253/264	123/130	228/231	174/175	96/105	158/162	165/168	171/180	205/214	158/161	120/120	100/106	102/114	90/90	193/193	124/161	133/137	106/110	183/195	177/178	142/148	129/135	217/235	137/144
A036	*	Hyuganatsu		1	298/301	299/302	252/252	253/253	159/165	228/234	174/175	102/105	158/162	165/168	177/177	209/214	158/164	120/120	96/106	102/114	90/90	193/193	124/161	133/137	89/157	189/217	162/177	148/162	135/141	217/217	174/174
A037		Hyuganatsu	Ihara 1	1	298/301	299/302	252/252	253/253	159/165	228/234	174/175	102/105	158/162	165/168	177/177	209/214	158/164	120/120	96/106	102/114	90/90	193/193	124/161	133/137	89/157	189/217	162/177	148/162	135/141	217/217	174/174
A038		Hyuganatsu	Muroto Konatsu	1	298/301	299/302	252/252	253/253	159/165	228/234	174/175	102/105	158/162	165/168	177/177	209/214	158/164	120/120	96/106	102/114	90/90	193/193	124/161	133/137	89/157	189/217	162/177	148/162	135/141	217/217	174/174
A039		Hyuganatsu	Orange Hyuga	1	298/301	299/302	252/252	253/253	159/165	228/234	174/175	102/105	158/162	165/168	177/177	209/214	158/164	120/120	96/106	102/114	74/90	193/193	124/161	133/137	89/157	189/217	162/177	148/162	135/141	217/217	174/174
A040		Hyuganatsu	Shoukakukei Hyuganatsu	1	298/301	299/302	252/252	253/253	159/165	228/234	175/175	102/105	158/162	165/168	177/177	209/214	158/164	120/120	96/106	102/114	90/90	193/193	124/161	133/137	89/157	189/217	162/177	148/162	135/141	217/217	174/174
A041	*	Ichanchii		4	298/313	299/314	nd.	264/268	165/171	228/231	174/175	99/102	158/158	165/165	171/177	196/218	158/161	119/119	92/100	114/114	84/97	193/193	149/167	133/133	110/127	191/193	183/191	136/136	134/134	213/213	137/138
A042	*	Ichang lemon		0	298/304	299/305	239/252	253/272	122/123	225/231	175/177	99/105	158/162	165/168	171/177	196/205	155/161	119/120	92/98	114/114	100/100	193/193	149/161	133/137	106/125	191/197	173/185	142/146	129/142	214/214	135/144
A043		Iyo	Miyauchi Iyo	3	298/313	299/314	252/252	253/264	159/165	219/228	172/174	102/105	162/162	165/168	171/177	205/214	149/158	119/119	100/100	114/114	87/87	193/193	149/161	133/137	106/110	193/209	174/174	148/148	135/141	217/235	148/156
A044	*	Iyo	Ootani Iyo	0	298/313	299/314	252/252	253/264	159/165	219/228	172/174	102/105	162/162	165/168	171/177	205/214	149/158	119/119	100/100	114/114	87/87	193/193	149/161	133/137	106/110	193/209	174/174	148/148	135/141	217/235	148/156
A045	*	Jabara		3	298/304	299/305	239/252	253/272	159/165	219/231	175/177	99/102	162/162	165/165	171/180	196/209	149/161	119/120	96/100	114/114	90/100	193/193	149/161	133/137	125/157	191/209	177/185	142/148	129/133	235/235	144/174
A046	*	Jabbon		0	313/326	314/327	244/252	253/272	165/171	225/228	171/175	96/99	162/162	165/168	171/171	205/214	155/158	120/120	92/96	114/114	87/90	193/193	124/137	133/133	108/125	193/217	173/174	144/154	129/133	218/235	135/137
A047	*	Kabosu		0	304/313	305/314	244/252	253/272	159/165	231/231	174/177	99/105	162/162	165/165	177/180	214/214	161/161	119/119	98/100	114/114	87/100	193/193	137/161	133/137	110/125	183/193	174/185	142/148	133/141	218/218	137/144
A048	*	Kabuchi		0	298/301	299/302	234/234	253/253	159/165	219/228	172/174	99/105	162/162	165/165	177/180	209/214	149/158	119/120	92/102	102/102	87/90	193/193	124/149	133/133	89/157	199/209	162/174	148/156	131/141	218/218	135/174
A049	*	Kaikoukan		0	298/298	299/299	234/234	253/264	159/165	228/231	146/174	96/105	158/162	165/168	171/180	205/214	158/161	119/120	93/100	102/114	87/90	193/193	124/161	133/137	106/110	193/209	174/177	148/148	129/135	217/218	156/174
A050	*	Kawabata		1	298/301	299/302	252/252	253/272	159/165	231/231	174/174	99/105	158/162	165/168	171/177	205/214	161/161	119/120	96/100	102/102	87/90	193/193	124/149	133/133	108/110	183/217	174/178	148/148	129/141	235/235	135/174
A051	*	Kawachi bankan		2	286/304	287/305	252/252	264/272	159/165	219/228	175/175	96/105	158/162	165/168	171/177	209/209	149/158	120/120	100/106	114/114	90/90	193/193	124/161	133/137	106/157	195/209	173/177	144/148	135/141	217/235	137/138
A052	*	Keraji		0	301/313	302/314	234/234	253/253	159/165	219/231	174/175	99/102	162/162	165/165	177/180	209/214	149/161	119/120	92/100	102/114	87/90	193/193	124/149	133/133	110/157	183/199	174/177	148/148	129/141	235/235	135/137
A053	*	Kikudaidai		0	298/301	299/302	239/252	253/272	165/165	225/228	133/174	99/102	162/162	165/168	171/177	205/214	155/158	119/120	92/98	114/114	87/90	193/193	149/161	133/137	89/106	199/213	174/181	148/162	129/133	218/235	135/138
A054	*	King mandarin		0	301/313	302/314	234/249	253/253	159/165	228/231	174/175	99/105	162/162	165/165	171/177	213/214	158/161	120/120	92/102	114/114	87/90	193/193	124/149	133/133	103/110	183/209	177/178	142/144	129/148	235/235	135/138
A055	*	Kinkoji		1	298/298	299/299	234/234	253/272	165/171	228/231	146/156	96/105	158/162	165/168	171/177	205/214	158/161	119/120	100/106	102/102	87/93	193/193	124/161	133/137	106/110	203/209	173/174	148/158	135/141	235/235	144/174
A056		Kinukawa		2	298/313	299/314	252/252	253/264	123/128	225/228	172/174	96/102	158/162	165/168	171/177	205/214	155/158	119/120	93/96	102/114	87/90	193/193	149/161	133/137	106/110	183/193	174/177	136/148	135/141	217/235	137/156
A057		Kishu mandarin	Hira Kishu	0	313/320	314/320	234/252	253/253	165/165	219/225	174/174	102/102	162/162	165/165	177/180	209/214	149/155	119/120	96/100	102/102	87/87	193/193	124/149	133/133	110/157	183/207	170/174	136/148	141/144	235/235	135/137
A058		Kishu mandarin	Hisago Komikan	1	313/320	314/320	234/252	253/253	125/128	219/225	174/174	102/102	162/162	165/165	177/180	209/214	149/155	119/120	96/100	102/102	87/87	193/193	124/149	133/133	110/157	183/207	170/174	136/148	141/144	235/235	135/137
A059	*	Kishu mandarin	Kishu	0	313/320	314/320	234/252	253/253	165/165	219/225	174/174	102/102	162/162	165/165	177/180	209/214	149/155	119/120	96/100	102/102	87/87	193/193	124/149	133/133	110/157	183/207	170/174	136/148	141/144	235/235	135/137
A060		Kishu mandarin	Kishu mikan	0	313/320	314/320	234/252	253/253	165/165	219/225	174/174	102/102	162/162	165/165	177/180	209/214	149/155	119/120	96/100	102/102	87/87	193/193	124/149	133/133	110/157	183/207	170/174	136/148	141/144	235/235	135/137
A061		Kishu mandarin	Kishu mikan Ihara Ichijoji	0	313/320	314/320	234/252	253/253	125/128	219/225	174/174	102/102	162/162	165/165	177/180	209/214	149/155	119/120	96/100	102/102	87/87	193/193	124/149	133/133	110/157	183/207	170/174	136/148	141/144	235/235	135/137
A062		Kishu mandarin	Komikan Fukuyama (Kinkou	0	313/320	314/320	234/252	253/253	125/128	219/225	174/174	102/102	162/162	165/165	177/180	209/214	149/155	119/120	96/100	102/102	87/87	193/193	124/149	133/133	110/157	183/207	170/174	136/148	141/144	235/235	135/137
A063		Kishu mandarin	Komikan Kawachi	0	313/320	314/320	234/252	253/253	125/128	219/225	174/174	102/102	162/162	165/165	177/180	209/214	149/155	119/120	96/100	102/102	87/87	193/193	124/149	133/133	110/157	183/207	170/174	136/148	141/144	235/235	135/137
A064		Kishu mandarin	Komikan Tensui	0	313/320	314/320	234/252	253/253	125/128	219/225	174/174	102/102	162/162	165/165	177/180	209/214	149/155	119/120	96/100	102/102	87/87	193/193	124/149	133/133	110/157	183/207	170/174	136/148	141/144	235/235	135/137
A065		Kishu mandarin	Kouda mikan	0	313/320	314/320	234/252	253/253	125/128	219/225	174/174	102/102	162/162	165/165	177/180	209/214	149/155	119/120	96/100	102/102	87/87	193/193	124/149	133/133	110/157	183/207	170/174	136/148	141/144	235/235	135/137
A066		Kishu mandarin	Mukaku Kishu (seedless Kishu)	0	313/320	314/320	234/252	253/253	165/165	219/225	174/174	102/102	162/162	165/165	177/180	209/214	149/155	119/120	96/100	102/102	87/87	193/193	124/149	133/133	110/157	183/207	170/174	136/148	141/144	235/235	135/137
A067		Kishu mandarin	Nan feng mi ju	0	313/320	314/320	234/252	253/253	125/128	219/225	174/174	102/102	162/162	165/165	177/180	209/214	149/155	119/120	96/100	102/102	87/87	193/193	124/149	133/133	110/157	183/207	170/174	136/148	141/144	235/237	135/137
A068		Kishu mandarin	Ozaki Komikan	0	313/320	314/320	234/252	253/253	125/128	219/225	174/174	102/102	162/162	165/165	177/180	209/214	149/155	119/120	96/100	102/102	87/87	193/193	124/149	133/133	110/157	183/207	170/174	136/148	141/144	235/235	135/137
A069		Kishu mandarin	Sakurajima Komikan Matsuura	0	313/320	314/320	234/252	253/253	125/128	219/225	174/174	102/102	162/162	165/165	177/180	209/214	149/155	119/120	96/100	102/102	87/87	193/193	124/149	133/133	110/157	183/207	170/174	136/148	141/144	235/235	135/137
A070		Kishu mandarin	Sakurajima Komikan senbatsu 1gou	0	313/320	314/320	234/252	253/253	125/128	219/225	174/174	102/102	162/162	165/165	177/180	209/214	149/155	119/120	96/100	102/102	87/87	193/193	124/149	133/133	110/157	183/207	170/174	136/148	141/144	235/235	135/137
A071		Kishu mandarin	Sakurajima Komikan Shirahama	0	313/320	314/320	234/252	253/253	125/128	219/225	174/174	102/102	162/162	165/165	177/180	209/214	149/155	119/120	96/100	102/102	87/87	193/193	124/149	133/133	110/157	183/207	170/174	136/148	141/144	235/235	135/137
A072		Kishu mandarin	Taka Mkan	0	313/320	314/320	234/252	253/253	125/128	219/225	174/174	102/102	162/162	165/165	177/180	209/214	149/155	119/120	96/100	102/102	87/87	193/193	124/149	133/133	110/157	183/207	170/174	136/148	141/144	235/235	135/137
A073	*	Kizu		0	298/304	299/305	234/239	253/253	159/165	231/231	174/177	99/105	162/162	165/165	171/180	196/214	161/161	119/119	96/100	102/114	87/100	193/193	124/149	133/133	110/125	183/191	174/185	142/148	133/141	235/235	144/174
A074		Kobayashi mikan		0	298/304/313	305/314	234/244/252	248/253	165/171	225/231	174/175	102/105	158/162	165/168	171/177/180	209/214	161/161	119/120	92/93/100	102/102	87/90	193/193	149/161	133/137	106/110	183/193	173/174	148/148	129/141	220/235	137/156
A075	*	Kobeni mikan		0	307/313	308/314	243/252	253/260	165/165	219/231	172/174	102/105	162/162	165/165	174/180	209/214	149/161	120/120	96/98	102/114	87/87	193/193	137/137	133/133	110/155	183/195	174/185	142/154	141/142	223/235	137/137
A076	*	Koji		0	301/304	302/305	249/252	253/260	159/165	228/231	146/156	102/102	158/162	165/165	177/180	214/214	158/161	120/120	92/98	114/114	87/90	193/193	137/149	133/133	110/159	197/209	178/181	150/158	131/142	215/220	135/147
A077		Komikan 2009-130		0	301/304	302/305	249/252	253/260	130/130	228/231	146/156	102/102	158/162	165/165	177/180	214/214	158/161	120/120	92/98	114/114	87/90	193/193	137/149	133/133	110/157	197/209	178/181	150/158	131/142	215/220	135/147
A078		Konejime		0	298/307	299/308	240/252	253/272	165/171	228/228	174/177	102/105	162/162	165/168	171/174	205/214	158/158	120/120	96/100	114/114	87/90	191/193	149/161	133/133	101/110	207/209	170/178	140/148	133/133	218/218	140/144
A079	*	Kourai Tachibana		2	301/304	302/305	239/252	253/253	165/171	225/228	146/174	99/102	162/162	165/165	171/174	214/214	155/158	119/120	96/98	114/114	90/100	193/193	124/149	133/133	91/145	189/191	181/185	142/158	133/135	215/235	144/144
A080	*	Kotokan		0	304/313	305/314	244/252	253/268	165/171	219/228	174/175	102/105	158/162	165/168	171/177	205/213	149/158	120/120	92/100	114/114	87/97	191/193	149/161	133/137	103/106	183/209	170/170	142/142	135/144	229/229	137/144
A081	*	Kunenbo-A		0	298/313	299/314	234/252	253/272	159/165	219/231	146/175	102/105	162/162	165/165	177/180	209/214	149/161	119/120	92/100	102/114	87/90	193/193	124/161	133/137	110/157	183/209	174/177	148/148	129/141	218/235	137/174
A082	*	Kunenbo-B	Kagoshima 0007	0	301/313	302/314	249/249	253/253	127/127	228/228	174/174	99/102	158/162	168/168	177/177	205/214	158/158	120/120	100/104	114/114	90/90	193/193	124/149	133/133	89/110	193/197	181/181	138/160	135/141	216/216	135/137
A083		Kunenbo	Kagoshima 0027	2	298/313	299/314	234/252	253/272	128/130	219/231	174/175	102/105	162/162	165/165	177/180	209/214	149/161	119/120	92/100	102/114	87/90	193/193	124/161	133/137	110/157	183/209	174/177	148/148	129/141	218/235	137/174
A084		Kunenbo	Kunenbo Kamikoshikijima	1	298/313	299/314	234/252	253/272	128/130	219/231	174/175	102/105	162/162	165/165	177/180	209/214	149/161	119/120	92/100	102/114	87/90	193/193	124/161	133/137	110/157	183/209	174/177	148/148	129/141	218/235	137/174
A085	*	Lemon	Lisbon	2	292/313	292/314	252/252	253/268	165/165	228/234	171/174	93/102	158/158	165/165	177/177	218/218	158/164	119/120	92/100	114/114	84/90	193/193	161/167	133/137	110/110	185/193	181/183	136/138	142/142	213/234	137/141
A086	*	Lemonade		0	298/307	299/308	252/252	253/253	165/165	231/231	174/175	102/105	158/158	165/165	171/171	209/209	161/161	119/120	100/100	114/114	87/90	193/193	149/179	133/133	135/135	185/209	174/174	152/152	142/142	235/235	144/144
A087	*	Limonia		0	292/301	292/302	247/247	253/253	165/165	231/231	174/175	93/102	162/162	165/165	177/177	218/218	161/161	119/120	96/96	114/114	84/87	193/193	124/179	133/133	110/110	185/189	189/189	136/142	137/137	229/229	135/141
A088	*	Mato buntan		4	286/304	287/305	252/252	272/272	123/123	228/231	175/175	99/105	158/158	168/168	171/171	205/205	158/161	120/120	92/106	114/114	90/90	193/193	161/161	137/137	110/110	197/217	177/177	148/148	129/135	217/229	138/144
A089	*	Mexican lime		7	292/304	292/305	247/247	253/256	165/165	228/231	171/174	93/99	nd.	165/165	174/174	205/205	158/161	119/125	92/106	114/114	84/100	193/193	167/167	133/133	108/108	185/195	177/183	136/140	137/137	230/230	135/141
A090	*	Meyer lemon		0	292/307	292/308	252/252	253/253	165/165	231/231	171/174	93/105	162/162	165/165	180/180	213/213	161/161	119/119	96/100	114/114	84/87	193/193	149/179	133/133	120/120	185/209	174/183	136/152	135/135	235/242	141/141
A091	*	Mochiyu		2	313/326	314/327	234/239	253/253	159/165	219/231	174/175	99/102	162/162	165/165	177/177	196/209	149/161	119/119	98/100	114/114	87/100	193/193	124/149	133/133	125/157	193/209	174/185	142/148	129/133	235/235	135/137
A092	*	Murcott		0	301/301	302/302	249/252	253/253	159/159	231/231	174/174	102/105	158/162	165/165	177/177	214/214	161/161	120/120	100/102	102/114	87/97	193/193	149/149	133/133	103/110	183/209	174/178	144/148	139/148	235/235	135/137
A093		Myrttle leaf orange	Chinott	0	298/313	299/314	249/252	253/268	165/171	228/228	174/175	102/105	158/162	165/168	171/177	205/218	158/158	120/120	92/100	114/114	87/90	193/193	124/161	133/137	106/112	193/210	174/181	138/172	135/142	213/216	137/144
A094		Nansho daidai		0	313/326	314/327	234/244	253/253	165/171	219/231	174/177	99/102	162/162	165/165	171/177	196/209	149/161	120/120	96/100	102/114	87/90	193/193	124/137	133/133	125/157	183/191	174/177	148/154	129/133	235/235	135/137
A095	*	Naruto		1	286/313	287/314	252/252	253/272	159/165	219/231	175/175	96/102	158/162	165/168	171/177	205/214	149/161	119/120	100/106	102/114	87/97	193/193	149/161	133/137	106/110	197/207	173/174	148/158	129/141	217/235	137/138
A096		Natsudaidai		0	304/313	305/314	234/244	248/253	165/171	231/231	172/174	102/105	158/162	165/168	171/180	209/214	161/161	119/120	93/100	102/102	87/90	193/193	149/161	133/137	106/110	183/193	173/174	148/148	129/141	220/235	137/156
A097		Natsudaidai	Beniamanatsu	0	304/313	305/314	234/244	248/253	165/171	231/231	172/174	102/105	158/162	165/168	171/180	209/214	161/161	119/120	93/100	102/102	87/90	193/193	149/161	133/137	106/110	183/193	173/174	148/148	129/141	220/235	137/156
A098	*	Natsudaidai	Kawano Natsudaidai	0	304/313	305/314	234/244	248/253	165/171	231/231	172/174	102/105	158/162	165/168	171/180	209/214	161/161	119/120	93/100	102/102	87/90	193/193	149/161	133/137	106/110	183/193	173/174	148/148	129/141	220/235	137/156
A099		Natsudaidai	Tachibana orange	0	304/313	305/314	234/244	248/253	165/171	231/231	172/175	102/105	158/162	165/168	171/180	209/214	161/161	119/120	93/100	102/102	87/90	193/193	149/161	133/137	106/110	183/193	173/174	148/148	129/141	220/235	137/156
A100	*	Nidonari mikan		0	298/320	299/320	249/252	253/253	165/171	219/228	172/174	102/102	162/162	165/165	177/177	209/218	149/158	119/120	100/100	102/102	87/87	193/193	149/161	133/137	110/155	207/210	174/174	148/168	135/141	216/235	135/144
A101	*	Oogonkan		0	298/313	299/314	234/247	253/253	159/165	228/231	174/175	102/105	158/162	165/165	171/180	214/214	158/161	120/120	96/106	102/102	87/90	191/193	149/161	133/137	110/110	185/189	177/178	148/150	131/135	218/218	144/144
A102		Ootachibana		0	298/298	299/299	234/234	253/272	159/171	228/231	175/175	96/105	158/162	165/168	171/171	205/214	158/161	120/120	93/106	102/114	90/90	193/193	124/161	133/137	108/110	185/217	177/177	148/148	135/141	217/218	144/144

A103	*	Ootoukan		0	304/313	305/314	247/252	253/264	159/165	228/231	174/177	99/105	158/162	165/168	171/177	205/214	158/161	119/119	100/100	114/114	87/87	193/193	149/161	133/137	108/110	197/209	170/174	136/148	135/141	214/217	144/148
A104	*	Oukan		0	313/326	314/327	252/252	253/272	165/171	225/228	174/174	96/102	158/162	165/168	171/180	213/214	155/158	119/120	100/100	102/114	87/87	193/193	149/161	133/137	108/110	183/191	170/174	136/148	133/144	217/235	135/137
A105		Ponkan	Ihara ponkan	0	313/326	314/327	243/252	253/260	159/165	219/231	174/174	102/105	162/162	165/165	177/177	209/214	149/161	119/120	96/100	102/114	87/87	191/193	124/149	133/133	110/157	209/209	174/177	148/158	141/152	223/235	135/148
A106		Ponkan	Morita ponkan	0	313/326	314/327	243/252	253/260	159/165	219/231	174/174	102/105	162/162	165/165	177/177	209/214	149/161	119/120	96/100	102/114	87/87	191/193	124/149	133/133	110/157	209/209	174/177	148/158	141/152	223/235	135/148
A107	*	Ponkan	Oota ponkan	0	313/326	314/327	243/252	253/260	159/165	219/231	174/174	102/105	162/162	165/165	177/177	209/214	149/161	119/120	96/100	102/114	87/87	191/193	124/149	133/133	110/157	209/209	174/177	148/158	141/152	223/235	135/148
A108		Ponkan	Yoshida ponkan	0	313/326	314/327	243/252	253/260	159/165	219/231	174/174	102/105	162/162	165/165	177/177	209/214	149/161	119/120	96/100	102/114	87/87	191/193	124/149	133/133	110/157	209/209	174/177	148/158	141/152	223/235	135/148
A109	*	Ponkitsu		0	313/326	314/327	243/247	253/260	165/165	228/231	153/175	102/105	162/162	165/165	177/177	209/209	158/161	120/120	96/100	114/114	87/90	191/191	149/149	133/133	157/157	189/209	177/178	148/158	135/141	235/235	135/135
A110	*	Pummelo whitetype		3	286/298	287/299	252/252	264/268	171/171	228/231	174/175	96/105	158/158	168/168	171/171	205/213	158/161	120/120	93/106	114/114	90/90	193/193	161/161	137/137	108/108	217/217	173/177	148/164	129/135	217/224	144/144
A111	*	Rokugatsumikan		2	301/313	302/314	252/252	253/268	165/171	228/234	174/175	99/102	162/162	165/165	171/177	214/218	158/164	120/120	100/102	114/114	90/90	193/193	149/161	133/137	89/110	193/199	181/183	138/140	135/139	213/235	135/137
A112	*	Sanbokan		0	298/313	299/314	234/234	253/264	159/165	225/228	172/174	102/105	158/162	165/168	171/177	205/214	155/158	120/120	93/100	102/114	87/90	193/193	124/161	133/137	106/110	183/193	170/177	136/148	135/141	218/235	137/156
A113		Satsuma mandarin	Aoshima Unshu	0	298/313	299/314	252/252	253/253	165/165	225/231	174/175	102/102	162/162	165/165	177/180	214/214	155/161	120/120	92/96	102/102	87/90	193/193	149/161	133/137	110/110	183/209	170/177	136/148	129/141	218/235	137/174
A114		Satsuma mandarin	Dobashi Beni	0	298/313	299/314	252/252	253/253	165/165	225/231	174/175	102/102	162/162	165/165	177/180	214/214	155/161	120/120	92/96	102/102	87/90	193/193	149/161	133/137	110/110	183/209	170/177	136/148	129/141	218/235	137/174
A115		Satsuma mandarin	Haraguchi Wase	0	298/313	299/314	252/252	253/253	125/128	225/231	174/175	102/102	162/162	165/165	177/180	214/214	155/161	120/120	92/96	102/102	87/90	193/193	149/161	133/137	110/110	183/209	170/177	136/148	129/141	218/235	137/174
A116		Satsuma mandarin	Imamura Unshu	0	298/313	299/314	252/252	253/253	125/128	225/231	174/175	102/102	162/162	165/165	177/180	214/214	155/161	120/120	92/96	102/102	87/90	193/193	149/161	133/137	110/110	183/209	170/177	136/148	129/141	218/235	137/174
A117		Satsuma mandarin	Iwasaki Wase	0	298/313	299/314	252/252	253/253	125/128	225/231	174/175	102/102	162/162	165/165	177/180	214/214	155/161	120/120	92/96	102/102	87/90	193/193	149/161	133/137	110/110	183/209	170/177	136/148	129/141	218/235	137/174
A118		Satsuma mandarin	Juman Unshu	0	298/313	299/314	252/252	253/253	125/128	225/231	174/175	102/102	162/162	165/165	177/180	214/214	155/161	120/120	92/96	102/102	87/90	193/193	149/161	133/137	110/110	183/209	170/177	136/148	129/141	218/235	137/174
A119		Satsuma mandarin	Jutaro Unshu NC	0	298/313	299/314	252/252	253/253	165/165	225/231	174/174	102/102	162/162	165/165	177/180	214/214	155/161	120/120	92/96	102/102	87/90	193/193	149/161	133/137	110/110	183/209	170/177	136/148	129/141	218/235	137/174
A120		Satsuma mandarin	Kinokuni Unshu	0	298/313	299/314	252/252	253/253	125/128	225/231	174/175	102/102	162/162	165/165	177/180	214/214	155/161	120/120	?/96	102/102	87/90	193/193	149/161	133/137	110/110	183/209	170/177	136/148	129/141	218/235	137/174
A121		Satsuma mandarin	Kuno Unshu	0	298/313	299/314	252/252	253/253	125/128	225/231	174/175	102/102	162/162	165/165	177/180	214/214	155/161	120/120	92/96	102/102	87/90	193/193	149/161	133/137	110/110	183/209	170/177	136/148	129/141	218/235	137/174
A122		Satsuma mandarin	Miyagawa Wase	0	298/313	299/314	252/252	253/253	165/165	225/231	174/175	102/102	162/162	165/165	177/180	214/214	155/161	120/120	92/96	102/102	87/90	193/193	149/161	133/137	110/110	183/209	170/177	136/148	129/141	218/235	137/174
A123		Satsuma mandarin	Nagahashi Unshu NC	0	298/313	299/314	252/252	253/253	165/165	225/231	175/175	102/102	162/162	165/165	177/180	214/214	155/161	120/120	92/96	102/102	87/90	193/193	149/161	133/137	110/110	183/209	170/177	136/148	129/141	218/235	137/174
A124		Satsuma mandarin	Niu Unshu	0	298/313	299/314	252/252	253/253	125/128	225/231	174/175	102/102	162/162	165/165	177/180	214/214	155/161	120/120	92/96	102/102	87/90	193/193	149/161	133/137	110/110	183/209	170/177	136/148	129/141	218/235	137/174
A125	*	Satsuma mandarin	Okitsu Wase (NC)	0	298/313	299/314	252/252	253/253	165/165	225/231	174/175	102/102	162/162	165/165	177/180	214/214	155/161	120/120	92/96	102/102	87/90	193/193	149/161	133/137	110/110	183/209	170/177	136/148	129/141	218/235	137/174
A126		Satsuma mandarin	Original tree	0	298/313	299/314	252/252	253/253	165/165	225/231	174/175	102/102	162/162	165/165	177/180	214/214	155/161	120/120	92/96	102/102	87/90	193/193	149/161	133/137	110/110	183/209	170/177	136/148	129/141	218/235	137/174
A127		Satsuma mandarin	Otsu-4 (NC)	0	298/313	299/314	252/252	253/253	165/165	225/231	174/175	102/102	162/162	165/165	177/180	214/214	155/161	120/120	92/96	102/102	87/90	193/193	149/161	133/137	110/110	183/209	170/177	136/148	129/141	218/235	137/174
A128		Satsuma mandarin	Shirakawa Unshu	0	298/313	299/314	252/252	253/253	125/128	225/231	174/175	102/102	162/162	165/165	177/180	214/214	155/161	120/120	92/96	102/102	87/90	193/193	149/161	133/137	110/110	183/209	170/177	136/148	129/141	218/235	137/174
A129		Satsuma mandarin	Sugiyama Unshu	0	298/313	299/314	252/252	253/253	125/128	225/231	174/175	102/102	162/162	165/165	177/180	214/214	155/161	120/120	92/96	102/102	87/90	193/193	149/161	133/137	110/110	183/209	170/177	136/148	129/141	218/235	137/174
A130		Satsuma mandarin	Suruga Beni	0	298/313	299/314	252/252	253/253	165/165	225/231	174/175	102/102	162/162	165/165	177/180	214/214	155/161	120/120	92/96	102/102	87/90	193/193	149/161	133/137	110/110	183/209	170/177	136/148	129/141	218/235	137/174
A131		Satsuma mandarin	Ueno Wase	0	298/313	299/314	252/252	253/253	165/165	225/231	174/175	102/102	162/162	165/165	177/180	214/214	155/161	120/120	92/96	102/102	87/90	193/193	149/161	133/137	110/110	183/209	170/177	136/148	129/141	218/235	137/174
A132		Satsuma mandarin	Yamada Unshu NC	0	298/313	299/314	252/252	253/253	165/165	225/231	174/174	102/102	162/162	165/165	177/180	214/214	155/161	120/120	92/96	102/102	87/90	193/193	149/161	133/137	110/110	183/209	170/177	136/148	129/141	218/235	137/174
A133		Satsuma mandarin	Yamashita Beni	0	298/313	299/314	252/252	253/253	165/165	225/231	174/175	102/102	162/162	165/165	177/180	214/214	155/161	120/120	92/96	102/102	87/90	193/193	149/161	133/137	110/110	183/209	170/177	136/148	129/141	218/235	137/174
A134	*	Satsuma Kikoku		0	298/307	299/308	240/252	253/272	165/171	228/228	174/174	102/105	162/162	165/168	171/174	205/214	158/158	120/120	96/100	114/114	87/90	191/193	149/161	133/133	101/110	207/209	170/178	140/148	133/133	218/218	140/144
A135	*	Shiikuwasha		1	301/313	302/314	252/252	253/253	165/165	228/228	174/174	99/102	162/162	165/165	177/177	209/214	158/158	120/120	96/102	nd.	87/90	193/193	137/149	133/133	89/155	189/199	177/183	140/160	131/142	221/221	135/148
A136	*	Shiikuwasha_Ogimikugani	Oogimi Kuganii	0	301/313	302/314	252/252	253/253	165/165	228/228	174/174	99/102	162/162	165/165	177/177	214/218	158/158	120/120	100/102	102/102	87/90	193/193	137/149	133/133	89/110	199/209	181/185	142/148	141/142	221/221	135/137
A137	*	Shunkokan		1	286/320	287/320	252/252	253/272	165/171	219/231	146/175	102/102	158/162	165/165	171/177	209/214	149/161	119/120	100/106	102/114	87/97	193/193	149/161	133/137	110/110	183/217	173/174	148/158	135/144	218/235	135/138
A138	*	Sokitsu		0	307/313	308/314	234/243	253/260	165/165	219/231	174/175	102/102	162/162	165/165	174/177	209/214	149/161	120/120	96/98	102/114	87/87	193/193	124/137	133/133	110/157	183/195	170/185	136/142	141/141	223/237	137/137
A139		Sour orange	Bouquet de Fleurs	0	298/313	299/314	249/252	253/268	165/171	228/228	174/175	102/105	158/162	165/168	171/177	205/218	158/158	120/120	92/100	114/114	87/90	193/193	124/161	133/137	106/110	193/210	174/181	138/138	135/142	213/216	137/144
A140		Sour orange	Chaozhouchen	0	298/313	299/314	249/252	253/268	165/171	228/228	133/174	102/105	158/162	165/168	171/177	205/218	158/158	120/120	92/100	114/114	87/90	193/193	124/161	133/137	106/110	193/210	174/181	138/168	135/142	213/216	137/144
A141	*	Sour orange	Daidai	0	298/313	299/314	249/252	253/268	165/171	228/228	133/174	102/105	158/162	165/168	171/177	205/218	158/158	120/120	92/100	114/114	87/90	193/193	124/161	133/137	106/110	193/210	174/181	138/168	135/142	213/216	137/144
A142		Sour orange	Kaiseito	2	298/313	299/314	249/252	253/268	165/171	228/228	133/174	102/105	158/162	165/168	171/177	205/218	158/158	120/120	92/100	114/114	87/90	193/193	124/161	133/137	106/110	193/210	174/181	138/168	135/142	213/216	137/144
A143		Sour orange	Za daidai	0	298/313	299/314	249/252	253/268	165/171	228/228	174/174	102/105	158/162	165/168	171/177	205/218	158/158	120/120	92/100	114/114	87/90	193/193	124/161	133/137	106/110	193/210	174/181	138/168	135/142	213/216	137/144
A144	*	Sudachi		0	304/313	305/314	239/247	253/253	159/165	231/231	174/174	99/102	158/162	165/165	171/180	196/214	161/161	119/120	96/98	114/114	87/100	193/193	149/149	133/133	110/125	189/193	178/185	142/150	133/142	217/217	144/144
A145	*	Suisho buntan		3	298/298	299/299	nd.	272/272	159/171	225/231	175/175	96/105	158/162	165/168	171/171	213/214	155/161	120/120	93/106	102/114	90/90	193/193	161/161	137/137	110/116	185/213	173/177	142/148	129/141	218/218	144/174
A146	*	Sunki		0	301/313	302/314	252/252	253/260	165/165	228/231	174/175	102/102	162/162	165/165	177/177	209/218	158/161	119/120	96/100	114/114	87/87	193/193	124/149	133/133	103/157	189/209	174/185	142/148	141/141	216/216	137/137
A147	*	Suruga Yuko		0	304/313	305/314	252/252	253/253	165/165	225/228	174/175	102/102	162/162	165/165	177/180	209/214	155/158	120/120	96/98	102/102	87/87	193/193	124/137	133/133	157/157	183/209	170/178	136/150	131/141	215/235	135/147
A148		Sweet orange	Cadenera	0	298/313	299/314	252/252	253/272	159/171	231/231	174/175	102/105	158/162	165/165	171/177	209/214	161/161	119/120	100/100	114/114	87/90	191/193	149/161	133/137	110/157	209/217	174/177	148/158	135/152	229/235	148/156
A149		Sweet orange	Cara Cara	2	298/313	299/314	252/252	253/272	128/130	231/231	174/175	102/105	158/162	165/165	171/177	209/214	161/161	119/120	100/100	114/114	87/90	191/193	149/161	133/137	110/157	209/217	174/177	148/158	135/152	229/235	148/156
A150		Sweet orange	Crescent	0	298/313	299/314	252/252	253/272	159/171	231/231	174/175	102/105	158/162	165/165	171/177	209/214	161/161	119/120	100/100	114/114	87/90	191/193	149/161	133/137	110/157	209/217	174/177	148/158	135/152	229/235	148/156
A151		Sweet orange	Hamlin	0	298/313	299/314	252/252	253/272	159/171	231/231	174/175	102/105	158/162	165/165	171/177	209/214	161/161	119/120	100/100	114/114	87/90	191/193	149/161	133/137	110/157	209/217	174/177	148/158	135/152	229/235	148/156
A152		Sweet orange	Jin Cheng	0	298/313	299/314	252/252	253/272	159/171	231/231	174/175	102/105	158/162	165/165	171/177	209/214	161/161	119/120	100/100	114/114	87/90	191/193	149/161	133/137	110/157	209/217	174/177	148/158	135/152	229/235	148/156
A153		Sweet orange	Joppa	0	298/313	299/314	252/252	253/272	159/171	231/231	133/174	102/105	158/162	165/165	171/177	209/214	161/161	119/120	100/100	114/114	87/90	191/193	149/161	133/137	110/157	209/217	174/177	148/158	135/152	229/235	148/156
A154		Sweet orange	Mediterranean Sweet Orange	0	298/313	299/314	252/252	253/272	159/171	231/231	174/175	102/105	158/162	165/165	171/177	209/214	161/161	119/120	100/100	114/114	87/90	191/193	149/161	133/137	110/157	209/217	174/177	148/158	135/152	229/235	148/156
A155		Sweet orange	Moro NC	0	298/313	299/314	252/252	253/272	159/171	231/231	174/175	102/105	158/162	165/165	171/177	209/214	161/161	119/120	100/100	114/114	87/90	191/193	149/161	133/137	110/157	209/217	174/177	148/158	135/152	229/235	148/156
A156		Sweet orange	Parson Brown	0	298/313	299/314	252/252	253/272	159/171	231/231	174/175	102/105	158/162	165/165	171/177	209/214	161/161	119/120	100/100	114/114	87/90	191/193	149/161	133/137	110/157	209/217	174/177	148/158	135/152	229/235	148/156
A157		Sweet orange	Pineapple	0	298/313	299/314	252/252	253/272	159/171	231/231	174/175	102/105	158/162	165/165	171/177	209/214	161/161	119/120	100/100	114/114	87/90	191/193	149/161	133/137	110/157	209/217	174/177	148/158	135/152	229/235	148/156
A158		Sweet orange	Santa Catarina	0	298/313	299/314	252/252	253/272	159/171	231/231	174/175	102/105	158/162	165/165	171/177	209/214	161/161	119/120	100/100	114/114	87/90	191/193	149/161	133/137	110/157	209/217	174/177	148/158	135/152	229/235	148/156
A159		Sweet orange	Seike navel	0	298/313	299/314	252/252	253/272	159/171	231/231	174/175	102/105	158/162	165/165	171/177	209/214	161/161	119/120	100/100	114/114	87/90	191/193	149/161	133/137	110/157	209/217	174/177	148/158	135/152	229/235	148/156
A160		Sweet orange	Shamouti	0	298/313	299/314	252/252	253/272	159/171	231/231	174/174	102/105	158/162	165/165	171/177	209/214	161/161	119/120	100/100	114/114	87/90	191/193	149/161	133/137	110/157	209/217	174/177	148/158	135/152	229/235	148/156
A161		Sweet orange	Tong Zi Gan	0	298/313	299/314	252/252	253/272	159/171	231/231	174/175	102/105	158/162	165/165	171/177	209/214	161/161	119/120	100/100	114/114	87/90	191/193	149/161	133/137	110/157	209/217	174/177	148/158	135/152	229/235	148/156
A162	*	Sweet orange	Trovita	0	298/313	299/314	252/252	253/272	159/171	231/231	174/175	102/105	158/162	165/165	171/177	209/214	161/161	119/120	100/100	114/114	87/90	191/193	149/161	133/137	110/157	209/217	174/177	148/158	135/152	229/235	148/156
A163		Sweet orange	Washington navel	0	298/313	299/314	252/252	253/272	159/171	231/231	174/175	102/105	158/162	165/165	171/177	209/214	161/161	119/120	100/100	114/114	87/90	191/193	149/161	133/137	110/157	209/217	174/177	148/158	135/152	229/235	148/156
A164		Sweet orange	Valencia	0	298/313	299/314	252/252	253/272	159/171	231/231	174/175	102/105	158/162	165/165	171/177	209/214	161/161	119/120	100/100	114/114	87/90	191/193	149/161	133/137	110/157	209/217	174/177	148/158	135/152	229/235	148/156
A165		Sweet orange	Wu Yue Cheng	0	298/313	299/314	252/252	253/272	159/171	231/231	174/175	102/105	158/162	165/165	171/177	209/214	161/161	119/120	100/100	114/114	87/90	191/193	149/161	133/137	110/157	209/217	174/177	148/158	135/152	229/235	148/156
A166		Sweet orange	Xue Gan	0	298/313	299/314	252/252	253/272	159/171	231/231	174/175	102/105	158/162	165/165	171/177	209/214	161/161	119/120	100/100	114/114	87/90	191/193	149/161	133/137	110/157	209/217	174/177	148/158	135/152	229/235	148/156
A167		Sweet orange	Yinzi Gan	0	298/313	299/314	252/252	253/272	159/171	231/231	174/175	102/105	158/162	165/165	171/177	209/214	161/161	119/120	100/100	114/114	87/90	191/193	149/161	133/137	110/157	209/217	174/177	148/158	135/152	229/235	148/156
A168		Tachibana		3	301/301	302/302	nd.	253/253	165/165	228/234	156/174	99/102	162/162	165/165	174/177	214/214	158/164	120/120	96/98	114/114	87/90	193/193	137/137	133/133	89/155	189/199	178/181	148/162	131/135	nd.	135/135
A169		Tachibana	Anettaishijou	0	301/301	302/302	252/252	253/253	165/165	228/234	146/174	99/102	162/162	165/168	174/177	214/214	158/164	120/120	96/102	114/114	87/90	193/193	124/137	133/133	89/157	189/197	162/178	148/162	135/141	221/223	135/135
A170		Tachibana	Botanical garden	1	301/301	302/302	252/252	253/253	165/165	228/234	146/174	99/102	162/162	165/168	174/177	214/214	158/164	120/120	96/102	114/114	87/90	193/193	124/137	133/133	89/159	189/197	162/178	148/162	135/141	221/223	135/135
A171		Tachibana	Hananoiwaya	0	301/301	302/302	252/252	253/253	165/165	228/234	146/174	99/102	162/162	165/168	174/177	214/214	158/164	120/120	96/102	114/114	87/90	193/193	124/137	133/133	89/159	189/197	162/178	148/162	135/141	221/223	135/135
A172	*	Tachibana	Heda 1	2	301/301	302/302	nd.	253/253	165/165	228/234	171/174	99/102	162/162	165/165	174/177	214/214	158/164	120/120	96/98	114/114	87/90	193/193	137/137	133/133	89/157	189/199	178/181	148/162	131/135	221/223	135/135
A173		Tachibana	Heda 2	2	301/301	302/302	nd.	253/253	165/165	228/234	171/174	99/102	162/162	165/165	174/177	214/214	158/164	120/120	96/98	114/114	87/90	193/193	137/137	133/133	89/157	189/199	178/181	148/162	131/135	221/223	135/135
A174	*	Tachibana	Ishinami Minka	0	301/313	302/314	247/252	253/253	165/165	228/228	146/174	99/102	162/162	165/168	177/180	214/214	158/158	120/120	96/104	102/102	87/87	191/193	149/149	133/133	89/110	189/197	178/181	140/150	131/131	217/217	135/135
A175	*	Tachibana	Ishinami No.1	0	301/301	302/302	252/252	253/253	165/165	228/234	146/174	99/102	162/162	165/168	174/177	214/214	158/164	120/120	96/102	114/114	87/90	193/193	124/137	133/133	89/157	189/197	162/178	148/162	135/141	221/223	135/135
A176		Tachibana	Ishinami No.2	0	301/301	302/302	252/252	253/253	165/165	228/234	146/174	99/102	162/162	165/168	174/177	214/214	158/164	120/120	96/102	114/114	87/90	193/193	124/137	133/133	89/151	189/197	162/178	148/162	135/141	221/223	135/135
A177		Tachibana	Oodomari OP-2	0	301/301	302/302	252/252	253/253	165/165	228/234	146/174	99/102	162/162	165/168	174/177	214/214	158/164	120/120	96/102	114/114	87/90	193/193	124/137	133/133	89/159	189/197	162/178	148/164	135/141	221/223	135/135
A178		Tachibana	Okitsu	2	301/301	302/302	nd.	253/253	165/165	228/234	171/174	99/102	162/162	165/165	174/177	214/214	158/164	120/120	96/98	114/114	87/90	193/193	137/137	133/133	89/155	189/199	178/181	148/162	131/135	221/223	135/135
A179		Tachibana	Reizanji	0	301/301	302/302	252/252	253/253	165/165	228/234	146/174	99/102	162/162	165/168	174/177	214/214	158/164	120/120	96/102	114/114	87/90	193/193	124/137	133/133	89/159	189/197	162/178	148/162	135/141	221/223	135/135
A180		Tajima mikan		0	301/313	302/314	249/252	253/260	159/165	231/234	146/174	99/102	158/162	165/168	177/180	214/214	161/164	120/120	92/102	114/114	90/90	193/193	137/149	133/133	89/110	189/197	158/162	152/162	141/142	220/220	135/135
A181		Takumanatsukunenbo		0	298/313	299/314	244/249	253/260	159/165	228/231	133/175	96/102	158/158	165/168	171/177	209/218	158/161	120/120	100/106	114/114	87/90	193/193	124/161	133/137	108/110	195/210	174/177	148/168	135/135	216/217	137/144
A182		Tankan		0	313/326	314/327	243/252	253/253	128/130	231/231	174/174	102/105	162/162	165/165	171/177	209/214	161/161	120/120	100/100	114/114	87/90	191/191	149/149	133/133	110/157	209/209	177/178	148/158	135/135	229/229	135/148
A183	*	Tankan	Taishun	0	313/326	314/327	243/252	253/253	165/171	231/231	174/174	102/105	162/162	165/165	171/177	209/214	161/161	120/120	100/100	114/114	87/90	191/191	149/149	133/133	110/157	209/209	177/178	148/158	135/135	229/229	135/148
A184		Tankan	Tarumizu 1	0	313/326	314/327	243/252	253/253	165/171	231/231	174/174	102/105	162/162	165/165	171/177	209/214	161/161	120/120	100/100	114/114	87/90	191/191	149/149	133/133	110/157	209/209	177/178	148/158	135/135	229/229	135/148
A185		Tankan	T-132	0	313/326	314/327	243/252	253/253	165/171	231/231	174/174	102/105	162/162	165/165	171/177	209/214	161/161	120/120	100/100	114/114	87/90	191/191	149/149	133/133	110/157	209/209	177/178	148/158	135/135	229/229	135/148
A186	*	Temple		0	313/313	314/314	252/252	253/272	159/165	231/231	174/175	102/105	158/162	165/165	171/177	209/213	161/161	120/120	100/100	102/114	87/90	193/193	149/161	133/137	135/157	183/217	177/177	152/158	135/152	218/235	148/148
A187	*	Tengu		1	298/313	299/314	234/252	253/253	123/128	231/231	174/174	105/105	158/162	165/168	177/177	209/214	161/161	120/120	100/106	102/114	90/90	191/193	124/149	133/133	108/110	183/209	177/177	148/158	135/152	217/235	144/148
A188	*	Tizon		0	313/313	314/314	252/252	253/253	159/165	231/231	174/175	102/105	158/162	165/165	174/177	213/214	161/161	120/120	96/100	114/114	87/90	193/193	149/161	133/137	110/116	189/209	177/177	156/158	135/142	216/235	137/148
A189		Toukan		0	301/304	302/305	249/252	253/260	159/165	228/231	174/177	102/102	158/162	165/165	177/180	214/214	158/161	120/120	92/98	114/114	87/90	193/193	137/149	133/133	110/159	197/209	178/181	150/158	131/142	215/220	135/147
A190		Tookunin		2	298/313	299/314	234/252	253/272	128/130	219/231	174/175	102/105	162/162	165/165	177/180	209/214	149/161	119/120	92/100	102/114	87/90	193/193	124/161	133/137	110/157	183/209	174/177	148/148	129/141	218/235	137/174
A191	*	Tosa buntan		0	298/298	299/299	234/234	253/272	159/171	228/231	175/175	96/105	158/162	165/168	171/171	205/214	158/161	120/120	93/106	102/114	90/90	193/193	124/161	133/137	108/110	185/217	177/177	148/148	135/141	217/218	144/144
A192	*	Twukkuni		3	301/313	302/314	252/252	253/253	127/130	228/231	174/174	99/105	162/162	165/165	177/177	214/214	158/161	120/120	92/96	nd.	90/90	193/193	137/149	133/133	89/157	189/199	nd.	148/160	131/142	218/218	135/137
A193		Twukkunin		0	298/313	299/314	234/252	253/272	128/130	219/231	174/175	102/105	162/162	165/165	177/180	209/214	149/161	119/120	92/100	102/114	87/90	193/193	124/161	133/137	110/157	183/209	174/177	148/148	129/141	218/235	137/174
A194		Twukunihu		0	298/313	299/314	234/252	253/272	128/130	219/231	174/175	102/105	162/162	165/165	177/180	209/214	149/161	119/120	92/100	102/114	87/90	193/193	124/161	133/137	110/157	183/209	174/177	148/148	129/141	218/235	137/174
A195		Twuukuribu		0	298/313	299/314	234/252	253/272	128/130	219/231	174/175	102/105	162/162	165/165	177/180	209/214	149/161	119/120	92/100	102/114	87/90	193/193	124/161	133/137	110/157	183/209	174/177	148/148	129/141	218/235	137/174
A196	*	Uchimurasaki		3	286/298	287/299	252/252	264/272	159/171	228/231	175/175	96/105	158/158	168/168	171/171	205/209	158/161	120/120	93/106	114/114	90/90	193/193	161/161	137/137	108/116	195/213	177/177	148/158	129/129	214/217	138/174
A197	*	Ujukitsu		0	304/313	305/314	234/252	253/272	165/171	219/231	146/174	102/105	162/162	165/165	171/177	209/214	149/161	119/120	92/100	102/114	87/90	193/193	124/161	133/137	110/157	183/209	173/174	144/148	129/141	235/235	137/141
A198	*	Unzoki		0	298/301	299/302	234/251	253/253	165/165	228/231	171/174	96/102	162/162	165/165	177/180	214/214	158/161	120/120	92/102	102/102	90/90	193/193	124/149	133/133	87/110	197/209	177/181	148/156	129/141	218/235	135/174
A199	*	USSR Tangelo		2	298/313	299/314	252/252	253/272	159/165	228/231	174/175	102/105	162/162	165/168	171/177	214/214	158/161	120/120	96/106	102/114	90/90	193/193	149/161	133/137	108/110	183/217	170/173	136/164	135/141	217/218	137/174
A200	*	Willowleaf mandarin	Mediterranean mandarin	0	301/313	302/314	252/252	253/253	165/165	219/231	174/174	102/102	162/162	165/165	177/177	209/209	149/161	119/120	96/100	102/114	87/87	191/193	149/149	133/133	103/157	183/189	174/185	142/152	135/152	235/235	137/137
A201	*	Willowleaf mandarin	Willowleaf mandarin	0	301/313	302/314	252/252	253/253	165/165	231/231	174/174	102/102	162/162	165/165	171/177	209/213	161/161	119/120	96/100	102/102	87/87	193/193	124/149	133/133	103/135	183/189	174/185	142/152	135/152	221/221	148/148
A202	*	Yamabuki		1	298/313	299/314	252/252	253/264	159/165	225/228	175/177	102/105	158/162	165/168	171/180	205/209	155/158	120/120	96/96	102/114	90/90	193/193	149/161	133/137	106/157	183/209	177/181	148/158	135/142	217/235	135/174
A203	*	Yamamikan		0	298/313	299/314	244/252	253/260	159/171	228/231	174/174	99/105	158/162	165/168	171/177	205/214	158/161	120/120	100/106	114/114	90/90	191/193	149/161	133/137	106/110	185/209	174/177	148/148	135/152	217/235	144/148
A204	*	Yatsushiro		0	298/320	299/320	234/252	253/253	159/165	225/231	174/174	102/102	162/162	165/165	177/177	214/214	155/161	119/119	92/100	102/102	87/87	193/193	124/149	133/133	110/110	183/209	174/174	148/148	129/144	218/235	135/174
A205	*	Yuge hyoukan		2	304/313	305/314	234/234	272/272	159/165	219/228	174/175	96/102	158/162	165/168	171/171	205/209	149/158	119/120	93/100	114/114	87/90	193/193	124/161	133/137	106/157	183/195	174/177	148/148	129/141	217/235	137/174
A206	*	Youpi ju (Yuhikitsu)		1	313/313	314/314	234/252	253/253	165/165	228/231	174/174	102/102	162/162	165/165	171/177	209/209	158/161	119/120	96/98	114/114	87/87	191/193	149/149	133/133	103/151	189/213	174/185	142/148	144/152	235/235	137/137
A207	*	Yuukunibu		0	298/313	299/314	252/252	253/264	159/159	225/228	174/174	96/102	158/162	165/168	171/177	205/214	155/158	119/120	96/96	102/114	87/90	193/193	149/161	133/137	106/110	183/193	174/177	136/148	135/135	217/235	137/156
A208	*	Yuzu		0	304/326	305/327	239/244	253/253	165/171	225/231	174/177	99/99	162/162	165/165	171/177	196/214	155/161	119/120	96/98	114/114	87/100	193/193	137/149	133/133	125/125	191/193	174/185	142/154	133/142	235/235	135/144
B001	*	'Akemi'		2	298/301	299/302	252/252	253/253	159/165	231/231	174/175	102/105	162/162	165/165	177/177	209/214	161/161	120/120	92/92	102/114	87/90	193/193	149/161	133/137	110/110	183/213	174/177	148/148	129/141	229/235	137/174
B002	*	'Aki Marine'		0	298/313	299/314	252/252	253/260	159/159	231/231	174/174	102/105	162/162	165/165	177/177	214/214	161/161	120/120	92/96	102/114	90/90	191/193	149/149	133/133	103/110	209/209	177/177	142/148	129/152	229/235	148/156

B003	*	'Aki Tangor'	0	298/313	299/314	252/252	253/272	165/171	225/231	175/175	102/102	162/162	165/165	177/177	209/214	155/161	120/120	92/100	102/114	87/90	193/193	149/161	133/137	110/110	209/209	170/177	136/158	129/135	229/235	148/174
B004	*	'Allspice'	0	298/301	299/302	252/252	253/253	165/171	231/231	174/177	102/105	158/162	165/165	171/177	209/214	161/161	120/120	100/106	102/114	87/90	191/193	124/149	133/133	103/110	183/213	173/185	142/158	152/152	235/235	148/156
B005	*	'Amaka'	0	298/301	299/302	249/252	253/253	165/165	231/231	174/174	102/102	162/162	165/165	177/177	213/214	161/161	120/120	92/96	102/102	90/90	193/193	124/149	133/133	110/135	183/209	177/177	142/148	129/135	235/235	135/156
B006	*	'Ariake'	0	298/313	299/314	252/252	253/272	159/171	231/231	174/175	102/105	158/162	165/165	171/177	213/214	161/161	120/120	100/100	114/114	87/90	191/191	149/149	133/133	135/157	209/217	177/185	142/158	135/135	229/229	148/156
B007	*	'Asumi'	0	298/313	299/314	243/252	253/260	159/159	231/231	174/174	102/102	158/162	165/165	177/177	209/209	161/161	119/120	92/96	102/102	87/90	191/193	149/161	133/137	157/157	185/209	174/177	148/158	141/152	235/235	148/156
B008	*	'Aurastar'	6	298/304	299/305	nd.	253/272	155/159	228/231	175/175	96/96	158/158	165/168	171/174	205/214	158/161	120/120	92/93	114/114	90/103	193/193	119/161	133/137	108/110	185/213	170/177	136/158	135/141	217/217	126/144
B009	*	'Awa Orange'	0	298/313	299/314	252/252	253/253	159/165	228/231	174/175	102/102	158/162	165/165	177/177	209/209	158/161	119/120	96/100	114/114	87/90	193/193	124/161	133/137	157/157	209/217	174/177	148/148	135/141	217/235	156/174
B010	*	'Benibae'	0	301/313	302/314	249/252	253/253	159/159	231/231	174/175	99/105	162/162	165/165	171/177	209/213	161/161	119/120	96/96	114/114	87/90	191/193	124/149	133/133	110/135	183/217	174/177	148/152	135/152	229/235	135/137
B011	*	'Benimadoka'	2	286/298	287/299	252/252	272/272	159/171	225/228	175/175	96/105	158/158	168/168	171/171	205/213	155/158	120/120	92/106	114/114	90/90	193/193	161/161	137/137	110/116	197/217	173/177	148/164	129/135	217/217	138/144
B012	*	'Chandler pummelo'	3	298/298	299/299	252/252	264/272	159/171	228/228	175/175	96/105	158/158	168/168	171/171	205/205	158/158	120/120	106/106	114/114	90/90	193/193	161/161	137/137	108/108	213/217	173/177	148/164	129/135	217/224	144/144
B013	*	'Ehimekashi 28'	0	313/313	314/314	252/252	253/253	159/159	231/231	174/175	102/105	162/162	165/165	177/180	209/213	161/161	120/120	92/100	102/114	87/90	191/193	149/149	133/133	135/157	209/209	177/185	142/158	129/152	229/229	137/148
B014	*	'Encore'	0	301/313	302/314	249/252	253/253	159/165	231/231	174/174	99/102	162/162	165/165	171/177	213/213	161/161	119/120	92/96	114/114	87/90	193/193	124/149	133/133	103/135	183/189	174/177	142/152	135/148	235/235	135/135
B015	*	'Fairchild'	0	301/313	302/314	252/252	253/253	159/165	231/231	174/174	102/105	162/162	165/165	177/177	213/214	161/161	120/120	100/100	114/114	87/90	193/193	124/161	133/137	110/135	183/209	177/185	142/158	135/152	229/229	137/148
B016	*	'Fortune'	0	298/301	299/302	252/252	253/260	159/165	231/231	174/175	102/102	162/162	165/165	171/177	213/214	161/161	120/120	100/100	114/114	87/90	193/193	124/161	133/137	110/157	183/209	177/185	142/158	135/152	213/221	148/174
B017	*	'Harehime'	0	301/313	302/314	252/252	253/260	159/165	225/231	174/175	102/105	162/162	165/165	177/180	209/214	155/161	119/120	96/100	102/102	87/90	191/193	149/149	133/133	110/110	209/209	174/177	148/148	129/152	229/235	137/137
B018	*	'Hareyaka'	0	313/313	314/314	252/252	253/253	159/165	219/231	174/174	102/102	162/162	165/165	171/177	209/213	149/161	119/120	96/100	102/102	87/90	191/193	149/149	133/133	103/157	183/209	174/177	142/148	141/148	223/235	148/148
B019	*	'Haruhi'	0	286/298	287/299	252/252	253/253	159/165	231/231	175/175	102/102	158/162	165/165	177/177	209/214	161/161	119/120	92/100	102/114	87/90	193/193	124/161	133/137	110/157	185/217	174/177	148/148	141/141	217/235	138/156
B020	*	'Haruka'	0	301/313	302/314	234/252	253/253	165/171	231/234	133/175	102/102	158/162	165/168	171/177	214/214	161/164	120/120	96/100	102/114	90/90	193/193	124/149	133/133	89/110	183/217	162/173	148/162	135/141	220/220	137/137
B021	*	'Harumi'	0	298/326	299/327	243/252	253/260	159/165	219/231	174/175	102/105	162/162	165/165	177/177	209/209	149/161	119/120	92/96	102/102	87/90	191/193	149/149	133/133	157/157	209/209	174/177	148/158	141/152	223/235	135/156
B022	*	'Hayaka'	0	298/326	299/327	243/252	253/260	159/165	231/231	174/175	102/102	162/162	165/165	177/177	214/214	161/161	120/120	96/100	102/102	87/87	193/193	124/149	133/133	110/110	209/209	170/177	136/158	129/152	223/235	135/174
B023	*	'Hayasaki'	5	286/304	287/305	nd.	268/272	159/171	225/231	175/175	105/105	158/158	168/168	171/171	205/213	155/161	120/120	92/106	114/114	90/90	193/193	161/161	137/137	110/116	197/213	173/177	148/164	129/129	217/217	138/138
B024	*	'Himekoharu'	0	298/313	299/314	234/252	253/253	159/165	231/231	174/174	102/102	158/162	165/165	177/180	209/214	161/161	119/120	100/106	102/102	87/90	193/193	149/161	133/137	110/157	189/209	174/177	148/148	129/131	235/235	156/156
B025	*	'Hiroshimakaken 11'	0	298/313	299/314	252/252	253/260	159/159	231/231	174/174	102/105	162/162	165/165	177/177	209/214	161/161	120/120	92/96	102/114	90/90	191/193	149/149	133/133	103/157	183/209	177/177	142/148	129/152	235/235	148/156
B026	*	'Honey'	0	301/313	302/314	249/252	253/253	165/165	231/231	174/174	99/102	162/162	165/165	171/171	213/213	161/161	119/120	100/102	102/114	87/90	193/193	124/149	133/133	103/135	183/183	174/177	142/152	148/152	221/221	138/148
B027	*	'Kanpei'	3	298/326	299/327	243/252	253/260	159/165	219/231	174/175	102/102	158/162	165/165	177/177	209/214	149/161	120/120	92/96	102/114	87/90	193/193	124/149	133/133	110/157	209/209	177/177	148/158	129/141	223/235	148/174
B028	*	'Kara'	0	298/301	299/302	249/252	253/253	159/165	231/231	174/175	99/102	162/162	165/165	177/177	214/214	161/161	120/120	92/96	102/114	87/90	193/193	149/161	133/137	103/110	183/209	170/177	136/142	129/141	235/235	135/137
B029	*	'Kincy mandarin'	0	301/313	302/314	249/252	253/253	165/165	231/231	174/175	99/105	162/162	165/165	171/171	209/214	161/161	120/120	92/100	114/114	87/87	193/193	124/124	133/133	110/157	209/209	178/185	142/144	129/135	235/235	148/148
B030	*	'Kinnow mandarin'	0	301/301	302/302	249/252	253/253	159/165	231/231	174/175	102/105	162/162	165/165	177/177	213/213	161/161	120/120	100/102	102/114	87/90	193/193	149/149	133/133	103/135	183/183	177/185	142/142	135/148	221/221	135/148
B031	*	'Kiyomi'	0	298/298	299/299	252/252	253/272	159/165	231/231	174/174	102/105	162/162	165/165	177/177	209/214	161/161	119/120	92/100	102/114	87/90	193/193	149/161	133/137	110/157	183/209	174/177	148/148	129/152	229/235	156/174
B032		'Kuchinotsu-41'	1	298/301	299/302	252/252	253/253	159/165	228/234	174/175	102/105	158/162	165/168	177/177	209/214	158/164	120/120	96/106	102/114	90/90	193/193	124/161	133/137	89/157	189/217	162/177	148/162	135/141	217/217	174/174
B033	*	'Lee'	0	301/301	302/302	252/252	253/260	159/159	231/231	174/174	102/105	162/162	165/165	171/177	213/214	161/161	119/120	100/100	114/114	87/87	191/193	124/149	133/133	110/135	183/209	174/185	142/148	135/135	229/229	137/148
B034	*	May Pummelo'	0	286/298	287/299	234/234	253/268	159/159	225/228	175/175	96/102	158/158	165/168	171/171	213/214	155/158	119/120	100/106	102/114	87/90	193/193	124/161	133/137	110/116	185/217	173/174	148/164	129/141	217/217	138/144
B035	*	'Mihaya'	0	313/313	314/314	252/252	253/253	159/159	231/231	153/175	102/102	162/162	165/165	171/177	213/214	161/161	119/120	92/100	102/102	87/90	193/193	149/161	133/137	110/135	183/193	177/177	148/148	135/141	235/235	156/156
B036	*	'Mihocore'	0	298/301	299/302	249/252	253/253	159/165	231/231	174/175	99/102	162/162	165/165	171/177	213/214	161/161	119/120	92/96	102/114	87/90	193/193	149/161	133/137	103/110	183/209	174/177	148/152	129/135	218/218	135/174
B037	*	'Mineola'	0	298/313	299/314	252/252	253/260	130/130	219/231	174/175	102/105	162/162	165/165	177/177	214/214	149/161	119/120	96/100	102/114	87/90	191/193	149/161	133/137	110/110	209/213	174/177	148/158	139/141	235/235	148/174
B038	*	'Nankou'	2	298/313	299/314	252/252	253/272	159/165	225/231	174/175	102/102	162/162	165/165	171/177	213/214	155/161	120/120	92/100	102/114	87/90	193/193	124/149	133/133	110/135	209/209	177/185	142/148	129/152	235/235	148/174
B039	*	'Nishinokaori'	1	298/298	299/299	252/252	253/253	159/165	231/231	174/175	102/105	158/162	165/165	177/177	209/209	161/161	119/120	92/100	102/114	87/90	191/193	149/149	133/133	110/157	209/217	174/177	148/148	129/152	229/235	156/174
B040	*	'Nou 5 gou'	0	301/320	302/320	234/252	253/253	159/165	225/231	174/175	102/105	162/162	165/165	171/177	209/213	155/161	119/120	100/100	102/102	87/87	193/193	124/124	133/133	110/135	183/183	174/185	142/148	135/141	235/235	135/148
B041	*	'Nou 6 gou'	0	313/320	314/320	234/252	253/253	159/165	225/228	174/174	102/105	162/162	165/165	171/177	214/214	155/158	120/120	92/100	102/114	87/90	193/193	124/149	133/133	103/157	183/183	170/177	136/142	129/141	235/235	135/138
B042	*	'Nou 7 gou'	3	298/304	299/305	nd.	253/272	159/159	228/231	174/174	96/96	158/158	165/168	171/171	213/214	158/161	120/120	92/92	114/114	93/103	193/193	119/161	133/137	108/110	193/213	170/177	136/158	135/141	217/224	126/144
B043	*	'Nova'	0	298/301	299/302	252/252	260/272	159/165	231/231	174/175	102/105	162/162	165/165	171/177	209/214	161/161	119/119	100/100	114/114	87/87	191/191	149/149	133/133	110/157	183/213	174/177	148/158	135/152	213/221	148/174
B044	*	'Ooitakaken-4'	1	298/313	299/314	252/252	253/253	159/165	231/231	174/175	102/105	162/162	165/165	177/180	209/214	161/161	120/120	92/96	102/114	87/90	191/193	149/149	133/133	110/157	209/209	170/177	136/158	129/152	229/235	137/174
B045	*	'Orland'	0	298/301	299/302	252/252	253/260	159/159	231/231	174/175	102/105	162/162	165/165	171/177	214/214	161/161	119/120	100/100	114/114	87/90	191/193	149/161	133/137	110/110	209/213	174/177	148/158	135/152	229/229	137/174
B046		'Oroblanco'	0	298/313	299/314	252/252	253/268/272	171/171	228/231	174/175	96/105	158/162	165/168	171/177	205/214	158/161	120/120	93/100/106	114/114	90/97	193/193	161/161	137/137	106/110	209/213/217	173/177	158/164	135/152	217/229/235	144/148
B047	*	'Osceola'	0	301/313	302/314	252/252	253/260	159/159	231/231	174/174	102/105	162/162	165/165	177/177	213/214	161/161	120/120	100/100	114/114	87/90	191/191	149/149	133/133	110/135	209/209	177/185	142/158	152/152	229/235	137/148
B048	*	'Page'	0	313/313	314/314	252/252	253/272	159/159	231/231	174/174	102/105	162/162	165/165	171/177	209/214	161/161	119/120	100/100	114/114	87/90	191/191	149/149	133/133	110/157	209/209	177/177	158/158	139/152	235/235	148/174
B049	*	'Pearl'	1	298/301	299/302	252/252	253/264	159/165	231/231	174/175	96/102	158/162	165/165	171/177	209/214	161/161	120/120	100/100	nd.	87/90	193/193	124/161	133/137	103/110	183/213	173/185	142/158	129/135	235/235	148/156
B050	*	'Pixie mandarin'	0	301/301	302/302	249/249	253/253	159/165	231/231	174/174	102/105	162/162	165/165	171/171	214/214	161/161	120/120	96/96	114/114	87/87	193/193	124/149	133/133	110/157	209/209	174/178	144/148	129/141	235/235	135/137
B051	*	'Robinson'	0	301/313	302/314	252/252	253/260	159/159	231/231	174/174	102/102	162/162	165/165	177/177	213/214	161/161	119/119	100/100	114/114	87/87	191/193	124/149	133/133	110/135	209/209	174/177	148/158	135/152	221/221	137/148
B052	*	'Saga Mandarin'	0	298/301	299/302	252/252	253/253	159/165	231/231	174/175	102/102	162/162	165/165	177/180	213/214	161/161	120/120	92/100	102/102	87/90	193/193	124/149	133/133	110/135	209/209	177/185	142/148	129/135	229/235	137/174
B053		'Sagakashi 34'	0	298/313	299/314	243/252	253/260	159/159	231/231	174/174	102/102	162/162	165/165	177/177	209/214	161/161	120/120	92/96	102/102	87/90	193/193	124/149	133/133	110/157	209/209	177/177	148/158	129/152	223/235	148/174
B054	*	'Seihou'	0	298/298	299/299	252/252	253/253	159/165	231/231	174/175	102/105	162/162	165/165	177/177	209/214	161/161	119/120	92/96	102/114	87/90	193/193	149/161	133/137	110/157	209/213	174/177	148/158	139/152	235/235	174/174
B055	*	'Seinannohikari'	0	301/313	302/314	243/249	253/260	159/165	231/231	174/175	99/102	162/162	165/165	177/177	213/214	161/161	120/120	92/96	102/102	87/90	193/193	124/149	133/133	103/110	209/209	174/177	152/158	129/152	235/235	135/148
B056	*	'Seminole'	0	298/301	299/302	252/252	253/260	159/165	231/231	174/175	102/105	162/162	165/165	177/177	214/214	161/161	120/120	93/100	102/114	87/97	191/193	149/161	133/137	110/157	209/213	173/174	148/158	139/141	229/235	137/174
B057	*	'Setomi'	0	298/313	299/314	252/252	253/272	159/159	231/231	174/175	102/105	162/162	165/165	177/177	209/214	161/161	119/120	96/100	102/114	87/87	191/193	149/161	133/137	110/157	209/209	174/177	148/158	129/141	223/229	148/174
B058	*	'Shiranuhi'	0	298/313	299/314	243/252	253/260	159/159	231/231	174/174	102/102	162/162	165/165	177/177	209/214	161/161	120/120	92/96	102/102	87/90	193/193	124/149	133/133	110/157	209/209	177/177	148/158	129/152	223/235	148/174
B059	*	'Southern Yellow'	0	298/320	299/320	252/252	253/268	165/171	225/228	174/175	99/102	158/162	165/168	171/177	205/214	155/158	119/119	100/106	102/102	87/87	193/193	149/161	133/137	110/116	183/217	174/174	148/148	141/141	235/235	135/174
B060	*	'Summer Fresh'	2	286/304	287/305	234/244	248/253	159/171	231/231	174/175	96/105	158/162	165/168	171/180	209/214	161/161	120/120	93/106	102/102	90/90	193/193	124/161	133/137	106/110	183/185	173/177	148/148	141/141	235/235	138/156
B061	*	Sweet Spring'	1	286/313	287/314	234/252	253/253	159/165	225/231	174/175	102/102	158/162	nd.	171/177	214/214	155/161	119/120	92/100	102/114	87/90	193/193	124/149	133/133	110/110	183/185	174/177	148/148	141/141	235/235	137/138
B062	*	'Tamami'	0	298/301	299/302	249/252	253/253	165/165	231/231	174/174	102/102	162/162	165/165	177/177	213/214	161/161	119/119	92/100	102/114	87/87	193/193	149/149	133/133	103/110	183/183	174/185	142/148	135/152	229/229	135/156
B063	*	'Tanikawa Buntan'	0	298/298	299/299	252/252	268/272	159/171	228/231	175/175	99/105	158/162	165/168	171/177	205/214	158/161	119/120	96/106	102/114	87/90	193/193	124/161	133/137	110/116	217/217	174/177	148/148	129/141	217/235	174/174
B064	*	'Tsunokagayaki'	0	298/301	299/302	249/252	253/253	159/165	231/231	174/174	102/102	162/162	165/165	171/177	209/213	161/161	119/120	92/96	102/102	87/90	193/193	124/161	133/137	103/157	183/209	174/177	148/152	129/148	229/229	135/156
B065	*	'Tsunokaori'	5	298/313	299/314	252/252	253/272	159/165	231/231	174/174	102/105	162/162	165/165	177/180	209/214	161/161	119/120	92/100	102/114	87/90	193/193	149/161	133/137	110/157	183/209	174/177	148/148	141/152	218/229	137/156
B066	*	'Tsunonozomi'	0	298/313	299/314	249/252	253/253	159/159	231/231	174/174	102/105	162/162	165/165	171/177	209/213	161/161	119/120	92/96	102/102	87/90	193/193	124/149	133/133	135/157	183/209	174/177	148/152	129/135	229/229	156/156
B067	*	'Willking'	0	301/313	302/314	249/252	253/253	165/165	231/231	174/174	102/105	162/162	165/165	171/177	213/213	161/161	119/119	100/102	102/114	87/87	193/193	124/149	133/133	103/103	183/189	185/185	142/142	135/135	221/221	135/135
B068	*	'Yellow Pummelo'	2	298/313	299/314	234/244	253/272	159/159	231/231	174/175	102/105	158/162	165/168	171/171	213/214	161/161	119/120	100/106	102/114	87/90	193/193	124/161	133/137	108/110	183/213	173/174	148/164	129/141	217/235	137/144
B069	*	'Youkou'	0	298/313	299/314	243/252	253/260	159/159	231/231	174/174	102/102	162/162	165/165	177/177	209/214	161/161	120/120	92/96	102/102	87/90	193/193	124/149	133/133	110/157	209/209	177/177	148/158	129/152	235/235	148/156
B070	*	E-647	0	298/301	299/302	252/252	253/260	159/159	231/231	174/175	105/105	162/162	165/165	177/177	209/214	161/161	119/120	100/100	102/102	87/90	191/193	149/149	133/133	110/157	183/209	174/177	148/158	152/152	229/229	137/174
B071	*	EnOw21	0	301/313	302/314	249/252	253/253	159/165	231/231	174/175	99/102	162/162	165/165	171/177	213/214	161/161	119/120	92/96	102/102	87/90	193/193	124/161	133/137	103/110	183/209	174/177	148/152	129/135	235/235	135/137
B072	*	H･FD-1	2	286/304	287/305	234/234	253/272	155/159	231/231	174/174	96/102	158/158	165/174	171/174	214/214	161/161	120/120	92/106	114/114	90/103	193/193	119/124	133/133	110/110	185/193	170/177	136/148	137/141	217/224	126/138
B073	*	HF9	0	298/313	299/314	252/252	253/253	159/165	225/231	174/174	102/105	162/162	165/165	177/177	209/214	155/161	119/120	96/100	102/114	87/90	191/193	149/149	133/133	110/110	183/217	174/177	148/148	129/152	229/235	137/156
B074	*	KyOw14	0	298/313	299/314	252/252	253/272	159/165	225/231	174/175	102/102	162/162	165/165	177/180	209/214	155/161	119/120	92/100	102/114	87/90	193/193	149/161	133/137	110/157	209/209	174/177	148/148	129/129	229/235	137/156
B075	*	KyOw21	0	298/313	299/314	252/252	253/253	165/165	225/231	174/174	102/105	162/162	165/165	177/180	209/214	155/161	119/120	92/100	102/114	87/90	193/193	149/161	133/137	110/157	183/209	174/177	148/148	129/152	229/235	137/156
B076	*	No.1408	0	301/313	302/314	252/252	253/253	159/165	231/231	174/174	102/105	162/162	165/165	171/177	214/214	161/161	119/120	96/100	102/114	87/90	193/193	161/161	137/137	110/110	183/193	174/177	148/148	135/141	235/235	135/156
B077	*	Okitsu-46	0	286/313	287/314	252/252	253/253	159/171	231/231	174/175	102/102	158/162	165/165	177/177	209/214	161/161	119/120	92/100	102/114	87/90	191/193	124/161	133/137	110/157	185/217	174/177	148/148	141/152	229/235	138/148
B078	*	Okitsu-56	0	301/313	302/314	252/252	253/253	165/165	225/231	174/174	102/105	162/162	165/165	177/177	209/213	155/161	119/120	100/102	102/114	87/87	193/193	124/124	133/133	103/135	183/189	185/185	142/142	135/141	221/221	148/148
C001	*	Strain 01	0	298/301	299/302	252/252	253/253	159/159	231/231	174/174	102/102	162/162	165/165	177/177	209/214	161/161	119/120	92/100	102/102	87/90	193/193	149/149	133/133	110/157	183/209	174/177	148/152	129/152	221/221	135/156
C002	*	Strain 02	0	301/313	302/314	252/252	253/272	159/159	231/231	174/175	102/105	162/162	165/165	171/180	209/209	161/161	119/119	100/100	102/114	87/87	191/193	149/149	133/133	110/135	209/209	174/177	148/158	135/152	229/235	137/148
C003	*	Strain 03	0	301/301	302/302	249/252	253/260	159/159	225/231	174/174	99/105	162/162	165/165	177/180	209/213	155/161	120/120	96/100	102/102	87/90	191/193	149/149	133/133	103/110	209/209	177/177	148/158	129/152	235/235	135/137
C004	*	Strain 04	0	298/313	299/314	252/252	253/268	159/165	228/228	174/174	102/105	162/162	165/165	177/177	209/218	158/158	120/120	100/106	102/114	90/90	193/193	124/161	133/137	110/157	193/217	177/181	138/148	135/142	213/217	137/174
C005	*	Strain 05	0	298/301	299/302	252/252	253/253	159/165	231/231	174/175	102/105	162/162	165/165	171/180	214/214	161/161	119/120	96/100	114/114	87/90	191/193	149/161	133/137	110/110	183/217	174/177	148/148	129/141	229/235	135/174
C006	*	Strain 06	0	313/313	314/314	252/252	253/253	159/171	231/231	174/174	102/102	162/162	165/165	171/177	214/214	161/161	119/120	92/100	102/114	87/90	191/193	149/149	133/133	110/110	183/209	174/177	148/148	141/152	229/235	137/148
C007	*	Strain 07	2	313/313	314/314	252/252	253/272	159/165	225/228	174/175	96/102	162/162	165/168	171/177	205/214	155/158	120/120	93/106	102/114	90/90	193/193	161/161	133/137	106/110	183/183	177/177	148/148	135/141	217/235	137/137
C008	*	Strain 08	0	298/298	299/299	252/252	253/253	159/165	231/231	133/174	102/105	158/162	165/165	177/177	209/214	161/161	119/120	100/100	102/114	87/90	193/193	149/161	133/137	110/157	209/213	173/174	148/158	139/152	229/229	137/156
C009	*	Strain 09	0	298/313	299/314	252/252	253/253	159/159	231/231	174/174	102/102	158/162	165/165	177/177	209/214	161/161	119/120	92/100	102/114	87/90	193/193	161/161	137/137	110/157	209/209	174/177	148/148	129/152	229/229	148/174
C010	*	Strain 10	0	298/313	299/314	252/252	253/253	159/165	231/231	174/174	102/105	162/162	165/165	177/177	213/214	161/161	119/119	100/102	102/114	87/87	193/193	124/149	133/133	103/110	183/209	174/185	142/148	135/152	235/235	156/156
C011	*	Strain 11	0	298/301	299/302	249/252	253/253	159/165	231/231	174/175	102/105	162/162	165/165	177/177	209/213	161/161	119/120	92/100	102/114	87/90	193/193	149/149	133/133	103/157	183/189	177/185	142/148	129/135	235/235	135/174
C012	*	Strain 12	0	298/301	299/302	252/252	253/253	159/165	231/231	174/174	102/102	162/162	165/165	177/177	213/214	161/161	120/120	92/100	114/114	90/90	193/193	124/149	133/133	103/110	189/209	177/177	148/148	129/152	235/235	135/156
C013	*	Strain 13	0	298/313	299/314	252/252	253/253	159/165	228/231	174/175	102/105	162/162	165/165	177/177	209/214	158/161	120/120	96/106	102/114	87/90	193/193	124/161	133/137	110/157	183/217	170/177	136/148	135/141	217/235	137/174
C014	*	Strain 14	0	298/301	299/302	252/252	253/260	159/159	231/231	175/175	102/105	162/162	165/165	177/177	209/214	161/161	119/119	92/100	102/102	87/87	191/193	149/149	133/133	110/157	183/209	174/177	148/158	129/152	229/229	137/156
C015	*	Strain 15	0	301/313	302/314	249/252	253/253	159/165	231/231	174/174	102/102	162/162	165/165	177/180	214/214	161/161	120/120	92/96	102/114	87/90	193/193	149/149	133/133	103/110	183/183	170/177	142/148	129/152	235/235	135/137
C016	*	Strain 16	2	304/304	305/305	252/252	272/272	159/171	231/231	174/175	99/105	158/158	168/168	171/171	205/213	161/161	120/120	106/106	114/114	90/90	193/193	161/161	137/137	108/110	213/217	173/177	148/164	129/129	217/217	138/144
C017	*	Strain 17	0	298/301	299/302	249/252	253/253	159/165	225/231	174/174	102/105	158/162	165/165	177/177	214/214	155/161	120/120	92/100	102/102	90/97	193/193	149/149	133/137	110/110	209/209	174/177	148/148	129/139	235/235	137/174
C018	*	Strain 18	0	313/313	314/314	252/252	253/253	159/165	231/231	174/174	102/102	162/162	165/165	177/180	213/214	161/161	120/120	92/100	102/102	87/90	191/193	149/149	133/133	110/135	209/209	177/185	142/148	129/152	218/218	137/148
C019	*	Strain 19	0	301/313	302/314	249/252	253/253	165/165	225/231	174/175	102/105	162/162	165/165	177/180	213/214	155/161	119/120	92/92	102/102	87/90	193/193	149/161	133/137	110/135	183/183	174/177	148/152	135/152	235/235	135/137
C020	*	Strain 20	0	313/326	314/327	249/252	253/253	159/165	231/231	174/174	102/102	162/162	165/165	177/177	213/214	161/161	119/120	96/96	114/114	87/90	191/193	124/149	133/133	103/157	183/209	174/174	152/158	135/141	235/235	135/135
C021	*	Strain 21	0	298/313	299/314	252/252	253/253	159/159	225/231	175/175	99/105	162/162	165/165	171/177	209/213	155/161	119/120	92/100	102/102	87/90	193/193	149/149	133/133	110/135	189/217	174/177	148/152	148/152	235/235	156/156
C022	*	Strain 22	0	298/313	299/314	252/252	253/253	159/159	231/231	174/175	99/102	162/162	165/165	171/177	209/213	161/161	119/120	92/96	102/102	87/87	193/193	124/124	133/137	103/157	209/209	174/177	152/158	135/152	235/235	137/156
C023	*	Strain 23	0	301/313	302/314	252/252	253/260	159/165	219/231	174/175	102/102	162/162	165/165	177/177	209/209	149/161	119/120	92/100	102/102	87/90	191/193	149/161	133/137	157/157	183/209	174/177	148/148	135/152	229/235	137/137
C024	*	Strain 24	1	298/313	299/314	234/252	253/253	165/165	225/231	175/175	99/105	162/162	165/165	177/180	213/214	155/161	119/120	92/100	102/114	87/90	193/193	124/161	133/137	103/110	183/209	174/177	142/148	129/129	235/235	138/156
C025	*	Strain 25	0	301/313	302/314	252/252	253/253	159/165	231/231	174/174	99/102	162/162	165/165	171/177	209/214	161/161	119/120	92/100	102/114	87/87	193/193	149/149	133/133	103/110	183/189	174/178	144/148	135/148	229/229	137/137
C026	*	Strain 26	0	298/313	299/314	252/252	253/253	159/165	219/231	174/174	102/105	162/162	165/165	177/177	209/214	149/161	119/120	92/100	114/114	87/90	193/193	149/149	133/133	110/157	209/217	174/177	148/148	129/141	229/235	137/156
C027	*	Strain 27	0	313/313	314/314	249/252	253/253	159/165	231/231	174/174	99/102	162/162	165/165	177/180	209/213	161/161	119/120	92/96	114/114	87/90	193/193	124/149	133/133	135/157	189/209	174/177	148/152	129/135	235/235	137/137
C028	*	Strain 28	0	298/313	299/314	249/252	253/253	159/165	231/231	174/175	99/102	162/162	165/165	177/180	209/213	161/161	119/119	92/96	102/102	87/87	193/193	124/161	133/137	135/157	189/209	174/174	148/152	129/135	229/229	135/156
C029	*	Strain 29	0	301/313	302/314	252/252	253/253	159/159	231/231	174/175	99/102	158/162	165/165	177/177	213/214	161/161	120/120	92/100	114/114	90/97	193/193	149/149	133/133	103/110	183/209	174/177	142/148	135/148	229/229	135/135
C030	*	Strain 30	0	298/301	299/302	252/252	253/260	159/165	225/231	174/174	102/105	162/162	165/165	177/180	213/214	155/161	119/120	92/100	102/114	87/90	191/193	149/161	133/137	110/135	209/209	174/177	148/148	129/135	229/229	137/156
C031	*	Strain 31	0	301/313	302/314	249/252	253/253	159/165	231/231	174/175	99/102	162/162	165/165	177/180	213/214	161/161	119/120	92/96	102/114	87/87	193/193	124/149	133/133	103/110	183/183	170/174	136/152	129/148	235/235	135/137
C032	*	Strain 32	0	298/298	299/299	252/252	253/253	159/165	231/231	174/175	102/105	162/162	165/165	171/177	209/214	161/161	119/120	92/100	114/114	87/90	193/193	149/149	133/133	110/157	209/217	174/177	148/148	129/141	229/235	156/156

C033	*	Strain 33	0	298/298	299/299	252/252	253/260	159/165	231/231	174/174	102/105	162/162	165/165	171/177	209/209	161/161	120/120	92/92	102/102	87/90	193/193	124/161	133/137	157/157	209/213	177/177	158/158	139/152	229/235	156/174
C034	*	Strain 34	0	286/313	287/314	234/252	253/272	165/171	231/231	174/175	102/105	158/162	165/165	177/177	214/214	161/161	119/120	92/100	114/114	87/90	191/193	124/149	133/133	110/157	185/209	174/177	148/158	135/141	235/235	138/148
C035	*	Strain 35	0	298/313	299/314	252/252	253/253	159/165	231/231	174/175	102/105	162/162	165/165	177/177	209/214	161/161	120/120	92/100	102/114	90/90	191/193	149/149	133/133	110/157	209/209	177/177	148/148	129/152	229/235	137/156
C036	*	Strain 36	2	313/313	314/314	252/252	253/272	159/171	225/231	174/174	102/105	158/162	165/165	171/171	213/214	155/161	120/120	92/100	102/114	87/90	191/193	149/161	133/137	110/135	189/209	177/185	142/148	135/135	229/235	148/148
C037	*	Strain 37	0	301/313	302/314	252/252	253/260	159/165	231/231	174/174	102/102	162/162	165/165	171/177	213/214	161/161	119/120	92/96	114/114	87/90	191/193	149/161	133/137	110/135	183/209	174/177	148/148	129/141	229/235	137/156
C038	*	Strain 38	3	298/301	299/302	252/252	253/260	159/165	219/231	174/174	99/105	162/162	165/165	171/177	209/209	149/161	119/120	92/100	102/102	87/87	191/193	124/149	133/137	157/157	209/209	174/177	148/158	135/152	nd.	137/156
C039	*	Strain 39	0	298/301	299/302	249/252	253/272	165/171	231/231	174/175	102/105	162/162	165/165	171/177	213/214	161/161	120/120	92/100	114/114	87/90	191/193	149/161	133/137	103/157	183/217	177/185	142/148	135/148	229/229	135/156
C040	*	Strain 40	0	298/301	299/302	252/252	253/253	159/165	231/231	172/175	99/105	162/162	165/165	171/177	209/213	161/161	120/120	92/92	114/114	90/90	193/193	124/149	133/133	103/157	183/209	177/177	142/148	135/152	229/235	135/156
C041	*	Strain 41	0	298/301	299/302	252/252	253/272	165/165	231/231	174/175	99/102	162/162	165/165	171/177	213/214	161/161	119/119	96/100	114/114	87/87	193/193	149/161	133/137	103/110	183/209	174/174	148/152	148/152	235/235	135/156
C042	*	Strain 42	0	313/313	314/314	252/252	253/253	159/165	231/231	174/175	102/105	158/162	165/165	177/177	209/213	161/161	119/120	100/100	114/114	87/90	191/193	149/161	133/137	157/157	183/209	174/177	148/158	129/135	229/229	137/148
C043	*	Strain 43	0	298/313	299/314	252/252	253/253	159/165	219/225	174/174	102/105	162/162	165/165	177/180	209/214	149/155	120/120	92/96	114/114	87/90	193/193	149/149	133/133	110/157	209/209	174/177	148/148	129/135	235/235	148/156
C044	*	Strain 44	0	301/313	302/314	252/252	253/260	159/165	231/231	174/174	102/105	162/162	165/165	177/177	213/214	161/161	119/120	96/100	102/102	87/90	191/193	149/161	133/137	110/135	183/209	170/174	136/148	129/135	218/218	137/148
C045	*	Strain 45	0	298/301	299/302	252/252	253/272	159/165	231/231	174/174	102/105	162/162	165/165	177/177	214/214	161/161	119/120	92/100	114/114	87/90	191/193	149/149	133/137	110/110	183/209	177/177	148/158	152/152	235/235	148/174
C046	*	Strain 46	0	298/298	299/299	252/252	253/264	159/165	228/231	174/174	102/105	158/162	165/168	177/177	205/209	158/161	119/120	92/100	114/114	87/90	191/193	149/161	133/137	106/110	193/217	174/177	148/148	141/152	217/229	156/174
C047	*	Strain 47	0	298/313	299/314	252/252	253/253	159/165	228/231	174/174	102/105	158/162	165/168	171/177	205/213	158/161	120/120	92/100	102/114	87/90	193/193	149/161	133/137	106/135	209/217	177/185	142/148	129/141	217/235	148/174
C048	*	Strain 48	0	298/313	299/314	252/252	253/253	159/165	225/231	174/175	102/102	162/162	165/165	177/177	209/214	155/161	119/120	92/100	102/114	87/90	191/193	149/149	133/133	110/157	183/217	174/177	148/148	135/141	229/235	137/156
C049	*	Strain 49	1	298/313	299/314	252/252	253/253	165/165	231/231	133/174	102/102	162/162	165/165	171/180	213/214	161/161	120/120	92/100	102/102	87/90	193/193	124/161	133/137	110/135	183/209	177/177	148/158	129/152	218/218	148/174
C050	*	Strain 50	4	313/313	314/314	252/252	253/253	159/165	231/231	172/174	102/105	162/162	165/165	177/177	209/214	161/161	119/120	92/100	102/114	87/90	193/193	124/149	133/133	110/135	183/183	177/185	142/148	nd.	218/218	137/148
C051	*	Strain 51	0	301/313	302/314	252/252	253/272	159/165	228/231	174/175	102/102	158/162	165/165	177/177	209/209	158/161	120/120	100/106	114/114	90/90	193/193	124/161	133/137	157/157	209/217	162/177	158/162	141/152	217/229	156/156
C052	*	Strain 52	0	298/301	299/302	252/252	253/253	159/165	228/231	174/174	102/105	158/162	165/165	177/177	209/214	158/161	120/120	100/106	102/114	90/90	191/193	124/149	133/133	110/157	189/217	162/177	158/162	135/141	217/235	156/156
C053	*	Strain 53	0	301/313	302/314	252/252	253/272	159/159	228/231	174/175	102/105	158/158	165/165	177/177	209/214	158/161	119/120	100/106	114/114	87/90	193/193	124/161	133/137	157/157	209/217	174/177	148/148	141/152	217/235	148/148
C054	*	Strain 54	0	286/301	287/302	234/252	253/260	159/159	228/231	174/175	102/102	162/162	165/168	177/177	205/214	158/161	120/120	100/106	102/102	87/90	191/193	124/149	133/133	106/110	185/209	174/177	148/148	135/141	235/235	137/138
C055	*	Strain 55	0	298/313	299/314	252/252	253/253	159/159	231/231	172/174	102/105	158/162	165/165	171/177	209/214	161/161	119/119	100/100	114/114	87/87	193/193	124/149	133/133	110/135	183/213	174/177	148/158	135/141	235/235	137/148
C056	*	Strain 56	0	298/313	299/314	234/252	253/253	159/165	231/231	174/174	102/105	158/162	165/165	171/177	214/214	161/161	119/120	100/100	114/114	87/90	191/193	124/149	133/133	110/110	183/217	174/177	148/158	141/152	235/235	137/156
C057	*	Strain 57	0	286/313	287/314	234/252	253/253	159/165	231/231	174/175	96/102	162/162	165/165	177/177	214/214	161/161	119/120	92/106	102/114	87/90	193/193	124/149	133/133	110/110	183/185	174/177	148/148	135/141	235/235	137/138
C058	*	Strain 58	0	298/301	299/302	252/252	253/253	159/159	219/231	174/175	102/105	158/162	165/165	171/177	214/214	149/161	119/120	100/100	114/114	87/97	193/193	149/149	133/133	110/110	193/213	173/174	148/158	135/141	235/235	137/156
C059	*	Strain 59	0	286/313	287/314	234/234	253/253	159/159	231/231	174/175	96/102	158/158	165/165	171/177	214/214	161/161	119/120	100/106	102/114	87/90	193/193	124/124	133/133	110/110	183/185	174/177	148/148	135/141	235/235	137/138
C060	*	Strain 60	0	301/313	302/314	234/252	253/260	159/165	219/228	174/175	102/102	158/162	165/168	177/177	205/209	149/158	120/120	96/100	102/102	87/90	191/193	149/161	133/137	106/157	183/209	174/177	148/148	135/141	235/235	137/137
C061	*	Strain 61	2	286/298	287/299	234/244	253/272	159/159	231/231	174/175	102/105	158/158	165/168	171/177	213/214	161/161	120/120	106/106	102/114	90/90	193/193	161/161	137/137	108/110	185/217	173/177	148/164	129/141	217/235	138/144
C062	*	Strain 62	0	286/313	287/314	234/252	253/253	159/159	231/231	174/175	102/105	158/162	165/168	177/177	209/214	161/161	119/120	100/100	114/114	87/90	193/193	124/161	133/137	106/110	185/209	174/177	148/158	135/141	217/235	138/148
C063	*	Strain 63	0	298/301	299/302	252/252	253/272	165/165	231/231	174/175	102/105	162/162	165/165	177/177	209/213	161/161	119/120	92/102	114/114	87/90	193/193	124/161	133/137	103/157	189/209	177/185	142/148	129/135	235/235	135/156
C064	*	Strain 64	0	313/313	314/314	252/252	253/272	165/171	225/231	174/175	102/102	158/162	165/165	177/177	214/214	155/161	119/120	92/100	102/114	87/90	193/193	149/161	133/137	110/110	183/217	174/177	148/148	129/152	235/235	137/148
C065	*	Strain 65	0	298/313	299/314	252/252	253/253	159/165	231/231	174/175	102/105	162/162	165/165	171/177	209/213	161/161	119/119	100/102	102/114	87/87	193/193	124/149	133/133	103/157	183/189	174/185	142/148	135/152	229/229	156/156
C066	*	Strain 66	0	298/301	299/302	252/252	253/253	159/165	231/231	172/175	102/102	162/162	165/165	177/177	213/214	161/161	119/119	92/102	102/114	87/87	193/193	124/161	133/133	103/110	183/183	174/185	142/148	135/152	235/235	135/174
C067	*	Strain 67	3	313/313	314/314	252/252	253/253	159/165	231/231	175/175	102/102	162/162	165/165	177/180	214/214	161/161	119/120	92/100	102/114	87/90	193/193	149/161	133/137	110/110	183/209	174/177	148/148	129/141	218/229	137/148
C068	*	Strain 68	0	286/298	287/299	252/252	253/253	165/171	231/231	174/175	102/105	162/162	165/165	177/177	209/209	161/161	119/120	92/100	102/102	87/90	191/193	149/161	133/137	157/157	183/185	174/177	148/148	129/141	229/235	138/174
C069	*	Strain 69	0	313/326	314/327	252/252	253/253	159/171	231/231	175/175	102/102	162/162	165/165	177/177	209/214	161/161	119/120	96/100	102/102	87/90	191/193	149/161	133/137	110/157	209/217	174/177	148/158	152/152	235/235	135/148
C070	*	Strain 70	0	313/313	314/314	252/252	253/253	159/171	231/231	174/174	102/102	162/162	165/165	177/177	214/214	161/161	119/120	92/102	114/114	87/90	191/193	149/149	133/133	110/110	183/183	177/185	142/148	135/141	235/235	148/148
C071	*	Strain 71	2	298/313	299/314	243/252	253/260	128/130	231/231	174/175	102/105	158/162	165/165	177/177	209/214	161/161	119/120	92/96	102/114	87/90	191/191	149/161	133/137	110/157	209/217	nd.	148/158	141/141	235/235	138/156
C072	*	Strain 72	0	286/298	287/299	252/252	253/268	165/171	231/231	174/175	105/105	158/162	165/168	171/177	209/213	161/161	120/120	92/106	102/114	90/90	193/193	149/161	133/137	108/110	209/217	173/177	148/164	129/129	224/229	144/156
C073	*	Strain 73	0	301/313	302/314	252/252	253/260	159/165	225/231	174/174	102/105	162/162	165/165	177/177	209/209	155/161	119/120	100/102	102/114	87/87	191/193	124/149	133/133	110/135	183/209	174/185	142/148	129/141	229/229	137/137
C074	*	Strain 74	0	301/313	302/314	252/252	253/260	159/159	231/231	174/175	102/105	162/162	165/165	177/177	209/214	161/161	120/120	96/100	114/114	87/90	191/193	149/149	133/133	110/157	209/209	177/177	158/158	152/152	229/235	137/148
C075	*	Strain 75	2	286/304	287/305	252/252	253/264	155/171	231/231	174/174	96/105	158/158	165/168	171/177	213/214	161/161	119/120	93/106	102/114	87/90	193/193	149/161	133/137	108/110	183/217	177/177	148/148	129/141	217/224	126/144
C076	*	Strain 76	2	286/304	287/305	252/252	253/264	159/171	228/231	174/175	96/102	158/162	165/168	171/174	213/214	158/161	120/120	92/92	102/114	90/90	193/193	149/161	133/137	108/110	185/217	173/177	148/164	135/141	217/224	126/144
C077	*	Strain 77	0	301/301	302/302	252/252	253/253	159/165	231/231	175/175	105/105	162/162	165/165	177/180	213/214	161/161	120/120	100/102	102/114	87/90	193/193	124/149	133/133	110/135	183/209	177/185	142/148	129/141	229/229	137/148
C078	*	Strain 78	3	286/326	287/327	252/252	253/260	159/165	231/231	174/175	102/102	158/162	165/165	177/177	209/209	nd.	119/120	92/100	102/114	87/90	191/193	149/161	133/137	157/157	185/209	174/177	148/148	141/141	229/235	135/138
C079	*	Strain 79	0	298/304	299/305	252/252	253/253	165/171	228/231	174/174	102/105	162/162	165/168	177/177	205/209	158/161	119/120	100/100	102/114	87/90	193/193	119/149	133/133	110/157	183/213	174/177	148/158	139/152	222/229	126/174
C080	*	Strain 80	0	298/301	299/302	252/252	253/253	159/165	231/234	174/174	102/105	158/162	165/168	171/177	209/214	161/164	120/120	92/100	102/114	87/90	193/193	124/149	133/133	89/157	209/217	177/185	142/148	129/141	229/229	156/156
C081	*	Strain 81	0	298/313	299/314	252/252	253/260	159/159	231/231	174/174	102/105	162/162	165/165	177/180	209/214	161/161	119/120	100/100	114/114	87/90	191/191	149/149	133/133	110/157	209/209	174/177	148/158	152/152	229/229	137/174
C082	*	Strain 82	0	313/313	314/314	252/252	253/264	165/171	228/231	174/175	102/105	158/162	165/168	171/180	205/214	158/161	120/120	92/106	102/114	90/90	193/193	149/161	133/137	99/110	183/209	173/177	148/159	129/135	217/218	137/148
C083	*	Strain 83	2	298/313	299/314	252/252	253/253	159/165	219/231	174/175	102/105	162/162	165/165	177/180	209/209	149/161	119/120	96/100	102/102	87/90	193/193	149/149	133/133	110/157	209/209	174/177	148/148	129/141	223/229	137/156
C084	*	Strain 84	1	301/313	302/314	243/252	253/260	128/128	225/231	174/174	102/102	162/162	165/165	177/177	209/209	155/161	119/120	96/100	102/102	87/90	191/191	149/149	133/133	110/157	209/209	174/174	148/158	129/141	235/235	137/148
C085	*	Strain 85	0	298/313	299/314	243/252	260/272	159/165	231/231	174/174	102/102	162/162	165/165	177/177	209/214	161/161	119/119	92/96	102/114	87/87	191/193	149/149	133/133	110/157	209/209	174/174	148/158	129/141	235/235	148/156

28	29	30	31	32	33	34	35	36	37	38	39	40	41	42	43	44	45	46	47	48	49	50	51	52	53	54	55	56	57	58	59	60	61
*		*	*		*	*								*	*		*	*		*	*		*	*	*		*			*	*	*	
GSR3121	GSR3126	GSR3128	GSR3136	GSR3137	GSR3138	GSR3140	GSR3141	GSR5105	GSR5112	GSR5114	GSR5122	GSR5127	GSR5129	GSR6101	GSR6122	GSR6129	GSR6133	GSR6135	NSX02	NSX23	NSX32	NSX34	NSX38	NSX39	NSX43	NSX48	NSX53	NSX67	NSX79	NSX82	NSX84	NSX115	NSX119
0	3	0	0	2	2	0	2	0	1	2	0	13	0	1	0	0	0	0	0	0	1	0	0	2	0	0	17	1	2	0	0	0	0
149/152	181/181	125/125	117/123	161/161	137/162	168/177	92/104	111/111	211/221	303/316	239/243	179/179	259/259	210/243	208/213	227/227	256/262	170/176	145/172	246/247	248/252	255/255	239/249	205/205	131/167	125/125	186/186	180/184	218/230	263/263	174/178	107/109	168/170
143/144	214/214	125/125	117/123	144/144	164/164	177/177	104/104	111/111	221/227	303/303	238/243	175/175	254/254	240/243	208/208	221/227	274/280	176/181	144/150	250/256	252/252	255/255	239/247	201/205	131/131	126/126	nd.	nd.	218/218	261/263	174/174	107/107	168/168
149/152	184/184	125/125	104/117	147/161	132/162	168/177	79/79	111/111	211/221	303/316	238/239	175/175	259/264	210/237	195/208	221/227	274/280	176/181	145/156	247/253	248/252	255/255	233/249	205/205	131/160	139/139	192/192	187/187	218/226	261/261	174/174	107/107	168/170
143/149	184/184	125/125	104/123	144/147	162/162	177/177	92/125	111/111	221/221	303/303	238/238	175/175	254/264	240/249	208/208	221/227	280/280	176/176	144/156	253/259	240/248	255/255	239/239	205/205	131/131	126/126	nd.	173/173	214/218	261/263	174/174	107/107	174/174
152/152	181/181	125/125	117/117	144/156	132/132	168/180	83/104	111/111	211/211	311/316	239/249	175/179	259/259	210/210	208/213	227/227	268/274	170/177	144/145	245/246	252/259	250/255	245/247	201/201	153/160	125/139	192/192	184/187	222/222	261/263	174/178	107/109	170/170
144/152	181/181	125/125	117/126	144/144	151/164	177/177	101/101	111/111	211/211	298/303	239/239	175/179	259/259	210/210	176/195	221/227	274/274	170/170	147/152	243/246	252/252	256/284	235/239	201/205	131/167	126/140	nd.	173/180	218/232	261/263	174/174	107/107	173/173
152/152	181/184	125/125	117/117	144/161	132/155	168/177	104/104	111/111	211/227	303/316	239/243	175/175	259/259	210/243	208/208	227/227	268/274	170/176	145/172	245/247	252/252	250/255	239/249	205/205	131/160	139/139	192/192	184/187	226/230	261/263	174/174	107/109	170/174
144/152	181/181	125/125	117/117	144/153	132/132	168/168	83/83	111/111	211/227	316/316	239/249	175/179	259/259	210/243	208/213	221/227	280/286	170/183	144/144	245/246	252/252	255/255	247/251	201/201	160/167	125/139	186/192	180/184	222/230	261/263	174/174	109/109	170/172
152/152	181/181	125/125	113/131	144/147	132/134	168/168	83/144	111/111	211/211	316/316	249/249	175/175	259/259	210/210	208/208	221/227	256/256	170/176	142/147	245/245	259/262	250/257	245/247	201/201	160/167	125/139	186/186	172/187	222/222	261/261	174/174	109/109	172/174
152/152	181/181	125/125	113/131	144/147	132/134	168/168	83/144	111/111	211/211	316/316	249/249	175/175	259/259	210/210	208/208	221/227	256/256	170/176	142/147	245/245	259/262	250/257	245/247	201/201	160/167	125/139	186/186	172/187	222/222	261/261	174/174	109/109	172/174
152/152	181/181	125/125	113/131	144/147	132/134	168/168	83/144	111/111	211/211	316/316	249/249	175/175	259/259	210/210	208/208	221/227	256/256	170/176	142/147	245/245	259/262	250/257	245/247	201/201	160/167	125/139	186/186	172/187	222/222	261/261	174/174	109/109	172/174
152/152	181/181	125/125	113/131	144/147	132/134	168/168	83/144	111/111	211/211	316/316	249/249	175/175	259/259	210/210	208/208	221/227	256/256	170/176	142/147	245/245	259/262	250/257	245/247	201/201	160/167	125/139	186/186	172/187	222/222	261/261	174/174	109/109	172/174
152/152	181/181	125/125	113/126	153/161	132/132	168/171	83/83	111/111	211/211	316/316	249/249	170/175	259/259	210/210	208/208	227/227	268/268	170/170	147/147	246/246	252/259	269/269	249/253	201/201	167/167	125/125	186/192	187/187	226/230	261/261	174/174	107/109	170/170
144/152	181/208	125/125	113/117	147/161	132/134	168/168	104/144	111/111	211/211	316/316	249/249	175/175	259/259	210/210	208/208	221/227	256/256	170/176	142/147	245/246	259/262	255/257	245/247	201/201	131/160	125/139	186/186	184/187	222/230	261/261	174/178	107/109	172/174
144/152	181/190	125/125	110/113	143/144	137/164	168/177	83/89	111/111	211/221	303/316	238/244	175/175	249/249	210/243	208/208	227/227	262/274	170/176	167/172	245/274	252/252	251/255	233/257	201/205	131/167	125/125	186/186	172/187	222/226	261/263	174/174	107/107	170/172
152/152	181/181	125/125	117/117	156/161	134/137	168/180	83/104	111/111	211/211	316/316	239/249	175/175	254/259	210/210	208/208	227/227	268/268	170/170	134/144	245/245	252/259	250/255	247/249	201/201	153/167	125/125	186/186	184/187	205/230	261/263	174/178	109/109	170/170
152/152	181/181	125/125	117/117	156/161	134/137	168/180	83/104	111/111	211/211	316/316	239/249	175/175	254/259	210/210	208/208	227/227	268/268	170/170	134/144	245/245	252/259	250/255	247/249	201/201	153/167	125/125	186/186	184/187	205/230	261/263	174/178	109/109	170/170
144/149	184/184	125/125	110/117	144/144	162/162	177/177	79/79	111/111	221/221	303/303	243/243	175/175	259/259	240/240	207/208	221/227	274/274	176/176	172/172	247/250	240/252	272/274	233/233	205/205	131/131	126/126	nd.	180/180	214/218	261/261	174/174	107/107	174/174
149/152	181/181	125/125	117/123	153/161	132/137	168/168	83/83	111/111	211/211	316/316	234/239	175/175	249/259	210/210	213/213	227/227	274/280	170/183	142/145	245/246	252/253	250/269	249/251	201/205	160/167	125/125	186/186	184/187	222/230	263/263	174/178	107/109	170/170
152/152	181/181	125/125	117/123	143/153	132/164	162/177	79/83	111/111	211/221	303/305	239/244	175/175	249/259	210/210	208/213	221/227	274/280	170/183	147/167	245/246	240/253	263/288	235/251	201/205	131/160	132/132	nd.	173/180	218/222	261/263	174/174	107/107	170/173
152/152	181/214	125/125	104/117	144/156	132/132	168/180	83/83	106/111	211/221	311/316	239/249	175/179	259/259	210/243	208/208	227/227	262/274	170/176	144/145	245/247	248/252	250/255	239/247	201/205	131/153	125/139	192/192	187/187	222/226	261/263	174/174	107/109	170/174
152/152	181/181	125/125	113/126	159/159	132/132	162/168	83/83	111/111	211/211	304/316	249/249	170/175	259/259	210/210	208/208	221/221	268/286	170/170	147/147	245/246	252/259	255/255	245/249	201/201	160/167	125/139	186/192	184/184	222/230	261/261	178/178	109/109	170/170
149/152	181/181	125/125	110/123	143/144	132/132	162/168	83/104	111/111	211/211	305/316	244/249	nd.	249/259	210/210	208/213	227/227	268/274	170/176	147/167	245/253	248/253	251/257	245/251	201/201	160/167	125/132	192/192	180/180	218/222	261/263	174/174	107/109	172/181
143/152	181/184	125/125	117/131	144/161	155/162	177/177	89/144	111/111	211/221	303/316	238/249	175/175	254/259	210/240	207/208	221/227	256/262	170/181	147/172	245/253	248/252	250/255	239/239	205/205	131/131	126/140	186/186	173/184	214/230	261/263	174/178	107/109	168/172
143/152	181/184	125/125	117/131	144/161	155/162	177/177	89/144	111/111	211/221	303/316	238/249	175/175	254/259	210/240	207/208	221/227	256/262	170/181	147/172	245/253	248/252	250/255	239/239	205/205	131/131	126/140	186/186	173/184	214/230	261/263	174/178	107/109	168/172
143/152	181/184	125/125	117/131	144/161	155/162	177/177	89/144	111/111	211/221	303/316	238/249	175/175	254/259	210/240	207/208	221/227	256/262	170/181	147/172	245/253	248/252	250/256	239/239	205/205	131/131	126/140	186/186	173/184	214/230	261/263	174/178	107/109	168/172
144/158	181/181	122/125	110/123	143/161	126/132	162/174	83/98	111/111	211/211	306/313	244/249	179/179	249/249	211/231	213/233	227/227	268/280	170/176	154/173	245/245	253/261	251/274	235/249	201/202	131/160	132/132	192/192	180/188	222/226	261/263	174/174	107/107	170/174
143/152	181/214	125/125	117/117	144/161	132/164	168/177	104/104	106/106	221/227	303/316	238/239	175/175	254/259	210/243	195/208	221/227	268/280	170/176	144/145	245/250	248/252	250/255	239/239	205/205	131/160	139/139	192/192	187/187	218/226	261/261	174/174	107/109	168/170
152/158	181/181	122/125	110/117	144/159	126/132	168/174	98/104	111/111	211/211	313/316	239/244	175/175	254/259	231/243	208/208	221/227	268/274	170/176	145/154	245/247	252/252	250/250	249/251	201/205	131/160	139/139	192/192	187/188	226/226	261/263	174/174	107/109	174/174
144/152	181/181	125/125	117/117	144/161	132/132	165/168	98/98	111/111	211/227	311/316	243/249	175/179	249/249	210/210	208/208	221/227	268/268	170/176	144/172	245/245	252/261	250/274	235/249	202/205	160/160	132/139	192/192	187/188	226/226	261/261	174/174	107/109	174/174
152/152	181/181	125/125	117/126	153/156	132/134	168/180	83/83	111/111	211/211	304/304	239/249	170/175	254/259	210/211	208/213	227/227	256/268	170/170	134/142	245/245	252/259	250/255	247/255	201/201	153/160	125/139	192/192	184/184	230/230	261/261	178/178	109/109	170/170
143/149	187/187	125/125	117/123	144/144	162/162	177/177	92/92	111/111	221/221	303/303	238/243	175/175	254/254	243/249	208/208	221/221	280/280	181/181	150/156	247/250	240/248	255/255	239/239	205/205	131/131	126/126	192/192	180/180	214/218	261/261	174/174	107/107	172/172
144/144	214/214	125/125	123/126	144/147	134/164	177/180	79/104	106/106	221/221	303/336	238/238	179/179	254/254	210/237	195/195	221/227	262/274	176/176	144/146	247/250	248/252	255/255	239/247	201/205	131/167	125/125	192/192	180/184	205/214	261/261	174/178	107/107	168/170
152/152	181/184	125/125	110/117	144/147	132/162	168/177	92/104	111/111	211/221	303/316	238/239	175/179	259/264	210/237	208/208	221/227	256/280	170/176	144/145	245/256	252/252	250/255	239/253	201/205	131/160	139/139	192/192	180/187	218/226	261/261	174/174	107/107	170/170
143/152	181/214	125/125	117/117	144/144	132/164	168/177	79/79	111/111	221/227	303/316	238/239	175/175	259/264	210/237	195/208	221/227	274/280	170/175	145/172	245/256	248/252	250/256	239/249	205/205	131/160	139/139	192/192	180/187	218/226	261/261	174/174	107/109	168/174
144/144	181/214	125/125	123/123	143/147	132/162	168/177	83/83	111/111	211/227	303/316	234/238	nd.	249/254	210/243	208/213	221/227	268/268	176/181	142/144	246/250	252/252	255/255	239/251	201/205	131/167	125/125	186/186	187/187	226/226	261/263	174/174	107/109	168/170
144/144	181/214	125/125	123/123	143/147	132/162	168/177	83/83	111/111	211/227	303/316	234/238	nd.	249/254	210/243	208/213	221/227	268/268	176/181	142/144	246/250	252/252	255/255	239/251	201/205	131/167	125/125	186/186	187/187	226/226	261/263	174/174	107/109	168/170
144/144	181/214	125/125	123/123	143/147	132/162	168/177	83/83	111/111	211/227	303/316	234/238	nd.	249/254	210/243	208/213	221/227	268/268	176/181	142/144	246/250	252/252	255/255	239/251	201/205	131/167	125/125	186/186	187/187	226/226	261/263	174/174	107/109	168/170
144/144	181/214	125/125	123/123	143/147	132/162	168/177	83/83	111/111	211/227	303/316	234/238	nd.	249/254	210/243	208/213	221/227	268/268	176/181	142/144	246/250	252/252	255/255	239/251	201/205	131/167	125/125	186/186	187/187	226/226	261/263	174/174	107/109	168/170
144/144	181/214	125/125	123/123	143/147	132/162	168/177	83/83	111/111	211/227	303/316	234/238	nd.	249/254	210/243	208/213	221/227	268/268	176/181	142/144	246/250	252/252	255/255	239/251	201/205	131/167	125/125	186/186	187/187	226/226	261/263	174/174	107/109	168/170
144/167	181/181	119/122	113/117	189/189	126/151	177/184	101/114	111/117	211/211	298/311	239/244	175/175	254/259	210/231	176/176	221/221	268/274	170/176	140/152	244/246	249/252	256/267	239/243	201/201	125/131	133/140	195/195	173/175	209/218	261/263	174/174	107/107	170/172
146/158	181/184	122/125	110/110	144/159	126/164	174/177	98/104	111/111	211/221	303/313	243/249	175/175	249/249	231/249	214/214	221/221	274/286	170/176	144/146	247/247	259/261	274/274	239/251	201/205	131/131	126/140	189/189	188/188	214/226	261/263	174/174	107/107	172/172
152/152	181/181	125/125	117/117	147/156	137/162	168/177	104/104	111/111	211/221	303/316	238/249	175/175	254/259	210/243	208/208	227/227	262/268	170/176	144/144	245/247	252/252	250/250	239/249	201/205	131/167	125/125	186/186	180/184	218/230	263/263	174/178	107/109	170/170
152/152	181/181	125/125	117/117	147/156	137/162	168/177	104/104	111/111	211/221	303/316	238/249	175/175	254/259	210/243	208/208	227/227	262/268	170/176	144/144	245/247	252/252	250/250	239/249	201/205	131/167	125/125	186/186	180/184	218/230	263/263	174/174	107/109	170/170
152/158	181/181	122/125	110/117	144/161	126/155	174/177	83/83	111/111	211/227	303/313	239/249	175/175	249/259	231/243	208/233	227/227	268/274	170/176	144/145	245/245	252/252	255/255	249/251	201/205	131/131	140/140	189/189	184/188	226/230	263/263	174/178	107/109	170/172
144/158	181/214	122/125	110/117	144/161	132/132	168/174	83/104	111/111	211/211	303/311	238/244	179/179	254/254	210/210	208/208	227/227	268/280	170/176	144/154	245/256	252/262	258/258	239/251	201/205	131/160	132/139	192/192	184/184	222/230	261/261	174/174	107/107	172/172
152/158	181/181	125/125	110/117	161/161	132/155	165/177	98/104	111/111	211/211	303/311	239/244	175/175	254/259	210/231	208/208	221/227	274/286	170/170	145/154	245/247	252/261	250/274	235/239	202/205	131/160	132/140	192/192	184/187	222/230	261/263	174/174	107/109	174/174
152/152	181/184	125/125	117/123	143/161	132/132	162/168	83/104	111/111	211/211	316/316	243/244	175/175	249/249	211/243	208/213	227/227	274/280	176/183	167/172	245/247	252/253	250/251	239/251	201/205	160/160	132/139	192/192	180/187	222/226	261/261	174/174	107/107	174/181
149/152	184/184	125/125	117/123	147/161	132/162	168/177	92/92	111/111	221/227	303/316	238/243	175/175	254/254	243/249	195/208	221/227	262/274	176/176	144/172	245/247	248/252	250/255	239/239	205/205	131/160	139/139	192/192	180/187	218/226	261/263	174/174	107/107	168/170
144/152	181/214	125/125	117/117	144/144	132/132	168/168	104/104	111/111	211/227	305/316	238/239	175/175	254/259	210/243	208/213	221/227	268/280	170/176	144/145	245/246	248/252	250/255	239/251	201/205	160/167	125/139	192/192	180/184	222/226	261/261	174/174	107/109	174/174
143/152	184/184	125/125	110/117	144/147	132/162	168/177	92/92	111/111	211/221	303/303	238/239	175/179	259/259	210/243	208/208	221/227	256/280	170/176	144/145	245/250	248/252	255/255	233/253	201/205	131/160	139/139	192/192	180/180	214/218	261/263	174/174	107/107	168/168
152/152	181/181	125/125	117/117	144/161	132/132	162/168	83/104	111/111	211/227	316/316	239/243	175/175	259/259	210/211	208/213	227/227	268/280	176/183	145/172	245/247	252/252	250/255	239/251	201/205	160/160	132/139	192/192	187/187	226/230	261/261	174/174	107/109	170/181
143/158	184/184	122/125	117/123	144/161	126/164	174/177	98/104	111/111	211/221	303/313	243/244	175/175	249/259	211/243	208/213	221/227	280/280	176/176	172/173	245/247	252/253	250/251	239/249	201/205	131/131	126/140	189/189	173/188	222/230	261/263	174/174	107/107	172/174
152/152	181/184	125/125	117/117	144/153	132/134	168/168	83/83	111/111	211/227	316/316	249/249	170/179	254/259	210/211	208/208	221/227	274/286	169/176	144/144	246/247	252/259	253/255	239/245	201/205	160/167	125/139	192/192	184/184	230/234	261/261	178/178	107/107	172/174
143/152	184/184	125/125	110/117	144/161	132/162	168/177	91/104	111/111	211/221	303/316	238/239	175/175	259/264	237/243	208/214	221/227	268/274	170/175	144/145	245/250	248/252	250/255	233/249	205/205	131/160	139/139	192/192	180/187	214/226	261/263	174/174	107/107	172/174
149/152	181/181	125/125	117/123	161/161	137/162	168/177	92/104	111/111	211/221	303/316	239/243	179/179	259/259	210/243	208/213	227/227	256/262	170/176	145/172	246/247	248/252	256/256	239/249	205/205	131/167	125/125	186/186	180/184	218/230	263/263	174/178	107/109	168/170
152/152	181/181	125/125	117/117	144/161	132/137	168/168	83/104	111/111	211/211	316/316	239/239	175/179	259/259	210/211	208/213	227/227	256/274	170/170	145/145	245/246	252/252	250/250	249/253	201/205	160/167	125/139	186/192	184/187	226/230	261/263	174/178	107/109	170/170
152/152	181/181	125/125	117/117	144/161	132/137	168/168	83/104	111/111	211/211	316/316	239/239	175/179	259/259	210/211	208/213	227/227	256/274	170/170	145/145	245/246	252/252	250/250	249/253	201/205	160/167	125/139	186/192	184/187	226/230	261/263	174/178	107/109	170/170
152/152	181/181	125/125	117/117	144/161	132/137	168/168	83/104	111/111	211/211	316/316	239/239	175/179	259/259	210/211	208/213	227/227	256/274	170/170	145/145	245/246	252/252	250/250	249/253	201/205	160/167	125/139	186/192	184/187	226/230	261/263	174/178	107/109	170/170
152/152	181/181	125/125	117/117	144/161	132/137	168/168	83/104	111/111	211/211	316/316	239/239	175/179	259/259	210/211	208/213	227/227	256/274	170/170	145/145	245/246	252/252	250/250	249/253	201/205	160/167	125/139	186/192	184/187	226/230	261/263	174/178	107/109	170/170
152/152	181/181	125/125	117/117	144/161	132/137	168/168	83/104	111/111	211/211	316/316	239/239	175/179	259/259	210/211	208/213	227/227	256/274	170/170	145/145	245/246	252/252	250/250	249/253	201/205	160/167	125/139	186/192	184/187	226/230	261/263	174/178	107/109	170/170
152/152	181/181	125/125	117/117	144/161	132/137	168/168	83/104	111/111	211/211	316/316	239/239	175/179	259/259	210/211	208/213	227/227	256/274	170/170	145/145	245/246	252/252	250/250	249/253	201/205	160/167	125/139	186/192	184/187	226/230	261/263	174/178	107/109	170/170
152/152	181/181	125/125	117/117	144/161	132/137	168/168	83/104	111/111	211/211	316/316	239/239	175/179	259/259	210/211	208/213	227/227	256/274	170/170	145/145	245/246	252/252	250/250	249/253	201/205	160/167	125/139	186/192	184/187	226/230	261/263	174/178	107/109	170/170
152/152	181/181	125/125	117/117	144/161	132/137	168/168	83/104	111/111	211/211	316/316	239/239	175/179	259/259	210/211	208/213	227/227	256/274	170/170	145/145	245/246	252/252	250/250	249/253	201/205	160/167	125/139	186/192	184/187	226/230	261/263	174/178	107/109	170/170
152/152	181/181	125/125	117/117	144/161	132/137	168/168	83/104	111/111	211/211	316/316	239/239	175/179	259/259	210/211	208/213	227/227	256/274	170/170	145/145	245/246	252/252	250/250	249/253	201/205	160/167	125/139	186/192	184/187	226/230	261/263	174/178	107/109	170/170
152/152	181/181	125/125	117/117	144/161	132/137	168/168	83/104	111/111	211/211	316/316	239/239	175/179	259/259	210/211	208/213	227/227	256/274	170/170	145/145	245/246	252/252	250/250	249/253	201/205	160/167	125/139	186/192	184/187	226/230	261/263	174/178	107/109	170/170
152/152	181/181	125/125	117/117	144/161	132/137	168/168	83/104	111/111	211/211	316/316	239/239	175/179	259/259	210/211	208/213	227/227	256/274	170/170	145/145	245/246	252/252	250/250	249/253	201/205	160/167	125/139	186/192	184/187	226/230	261/263	174/178	107/109	170/170
152/152	181/181	125/125	117/117	144/161	132/137	168/168	83/104	111/111	211/211	316/316	239/239	175/179	259/259	210/211	208/213	227/227	256/274	170/170	145/145	245/246	252/252	250/250	249/253	201/205	160/167	125/139	186/192	184/187	226/230	261/263	174/178	107/109	170/170
152/152	181/181	125/125	117/117	144/161	132/137	168/168	83/104	111/111	211/211	316/316	239/239	175/179	259/259	210/211	208/213	227/227	256/274	170/170	145/145	245/246	252/252	250/250	249/253	201/205	160/167	125/139	186/192	184/187	226/230	261/263	174/178	107/109	170/170
152/152	174/181	125/125	117/117	144/161	132/137	168/168	83/104	111/111	211/211	316/316	239/239	175/179	259/259	210/211	208/213	227/227	256/274	170/170	145/145	245/246	252/252	250/250	249/253	201/205	160/167	125/139	186/192	184/187	226/230	261/263	174/178	107/109	170/170
152/152	181/181	125/125	117/117	144/161	132/137	168/168	83/104	111/111	211/211	316/316	239/239	175/179	259/259	210/211	208/213	227/227	256/274	170/170	145/145	245/246	252/252	250/250	249/253	201/205	160/167	125/139	186/192	184/187	226/230	261/263	174/178	107/109	170/170
152/152	181/181	125/125	117/117	144/161	132/137	168/168	83/104	111/111	211/211	316/316	239/239	175/179	259/259	210/211	208/213	227/227	256/274	170/170	145/145	245/246	252/252	250/250	249/253	201/205	160/167	125/139	186/192	184/187	226/230	261/263	174/178	107/109	170/170
152/158	181/181	122/125	110/117	144/159	126/132	168/174	98/104	111/111	211/211	313/316	239/243	175/175	254/259	231/243	208/208	221/227	268/274	170/176	145/154	245/247	252/252	250/250	249/251	201/205	131/160	139/139	192/192	187/188	226/226	261/263	174/174	107/109	174/174
143/152	181/181	125/125	110/117	144/161	137/162	168/180	83/89	111/111	211/221/227	303/316	238/239	175/179	254/259	210/243	208/208	221/227	274/280	170/176	144/145	245/250	240/252	250/255	225/249	205/205	131/167	125/125	186/192	173/184	226/230/234	261/263	174/178	107/109	168/170
152/152	181/181	125/125	110/113	159/161	132/132	165/165	83/104	111/111	211/211	304/316	239/239	175/179	259/259	210/210	208/213	227/227	256/274	169/177	144/145	246/246	252/252	268/269	247/253	201/201	153/167	160/160	186/192	184/184	222/226	261/261	178/178	109/109	170/172
144/149	181/181	125/125	123/138	144/153	132/132	162/168	83/83	111/111	211/211	316/316	234/249	175/175	249/259	210/249	208/213	227/227	274/280	170/183	142/144	245/253	252/253	269/269	247/251	201/201	160/167	125/125	186/192	180/187	218/222	261/263	174/174	107/109	170/172
144/149	181/181	125/125	123/138	144/153	132/132	162/168	83/83	111/111	211/211	316/316	234/249	175/175	249/259	210/249	208/213	227/227	274/280	170/183	142/144	245/253	252/252	269/269	247/251	201/201	160/167	125/125	186/192	180/187	218/222	261/263	174/174	109/109	170/172
144/152	181/187	125/125	117/123	147/159	132/162	171/177	83/92	111/111	211/221	307/316	238/239	170/175	259/259	210/240	208/213	221/227	274/280	170/175	142/144	246/247	252/252	255/260	237/239	205/205	131/153	132/132	192/192	173/187	218/222	261/261	172/174	107/109	170/170
144/144	181/181	122/125	110/123	153/159	126/132	168/174	83/83	111/111	211/211	313/316	234/249	nd.	249/249	210/231	213/233	227/227	256/268	170/183	142/144	245/245	252/252	269/269	235/245	201/202	131/167	125/125	186/186	187/188	226/226	261/263	174/174	107/109	170/174
149/152	181/185	125/125	113/117	144/144	134/164	177/180	83/104	106/106	211/221	303/316	238/239	175/179	259/259	210/237	195/208	221/227	268/280	170/175	145/168	245/253	252/259	253/255	241/249	201/205	131/153	125/125	186/186	180/184	218/230	261/261	174/178	107/109	170/173
152/152	181/184	125/125	117/117	144/161	132/155	168/177	104/104	111/111	211/227	303/316	239/243	175/175	259/259	210/243	208/208	227/227	268/274	170/176	145/172	245/247	252/252	250/255	239/249	205/205	131/160	139/139	192/192	184/187	226/230	261/263	174/174	107/109	170/174
144/152	181/181	125/125	117/123	143/153	132/134	162/180	79/83	111/111	211/221	316/336	239/244	175/175	249/259	210/211	208/213	227/227	274/274	170/176	147/167	245/246	240/253	256/263	235/253	201/205	160/167	125/132	192/192	180/184	205/222	261/261	174/178	107/107	170/181
152/152	181/184	125/125	117/117	144/161	132/155	168/177	104/104	111/111	211/227	303/316	239/243	175/175	259/259	210/243	208/208	227/227	268/274	170/176	145/172	245/247	nd.	250/256	239/249	205/205	131/160	139/139	192/192	184/187	nd.	261/263	174/174	107/109	170/174
152/152	181/184	125/125	117/117	144/161	132/155	168/177	104/104	111/111	211/227	303/316	239/243	175/175	259/259	210/243	208/208	227/227	268/274	170/176	145/172	245/247	252/252	250/256	239/249	205/205	131/160	139/139	192/192	184/187	226/230	261/263	174/174	107/109	170/174
141/144	181/181	119/125	98/117	144/144	151/164	177/184	101/101	111/111	211/211	298/303	239/239	175/175	259/259	210/231	176/208	221/221	274/274	170/170	147/152	243/246	252/252	256/284	239/247	201/201	125/167	126/140	nd.	173/180	218/232	261/263	174/174	107/107	170/170
141/144	214/214	125/125	113/113	159/159	137/151	168/177	83/101	111/111	211/211	298/316	238/249	175/179	254/259	210/249	208/213	221/227	262/268	176/176	138/144	245/247	240/252	255/258	239/255	201/205	125/160	125/125	186/186	180/187	222/222	261/263	174/174	107/107	170/173
141/152	181/181	125/125	98/120	153/153	132/151	171/177	83/101	111/111	211/211	298/316	239/249	164/164	259/259	210/231	175/208	221/227	268/274	170/170	138/147	243/245	252/252	256/281	239/245	201/201	125/167	125/125	186/186	180/184	218/218	261/261	174/178	107/109	170/173
144/149	214/214	125/125	123/123	144/144	162/164	177/177	92/116	111/111	221/221	303/303	238/243	nd.	254/254	243/249	208/208	221/227	280/280	175/181	144/172	247/250	248/252	256/256	233/241	205/205	131/131	126/126	nd.	180/180	214/214	261/263	174/174	107/107	168/172
141/144	188/188	119/127	98/138	nd.	151/164	174/184	101/101	111/111	211/221	298/302	238/239	179/179	259/264	231/231	175/206	221/221	268/274	178/178	138/177	243/253	256/256	260/281	239/248	201/201	125/139	126/140	nd.	180/180	nd.	261/269	174/174	107/107	173/173
141/152	181/181	125/125	98/113	144/144	134/151	177/180	83/101	111/111	211/227	298/316	239/249	179/179	259/264	210/231	176/208	221/227	268/268	176/176	138/147	243/245	252/252	255/284	239/249	201/201	125/160	125/160	186/186	180/184	230/230	261/263	174/178	107/109	172/172
144/152	181/184	122/125	110/117	161/161	126/132	168/174	98/98	111/111	211/227	313/316	239/244	175/175	254/259	210/210	208/233	227/227	268/286	170/176	145/154	245/247	252/261	250/274	239/251	201/205	131/160	139/139	192/192	187/187	222/230	261/263	174/174	107/107	170/174
152/152	181/181	125/125	117/117	144/153	134/137	168/168	83/89	111/111	211/221	316/316	239/249	170/175	254/254	210/210	208/208	221/227	256/274	169/170	134/144	245/246	252/252	250/255	245/247	201/201	167/167	125/125	186/192	184/184	230/234	261/263	178/178	107/109	170/172
144/152	181/214	125/125	117/126	144/153	134/164	177/180	79/79	111/111	211/221	303/336	238/239	175/179	254/259	210/237	195/208	221/227	274/274	170/176	146/147	246/246	240/252	256/263	235/247	201/205	131/167	125/125	192/192	173/184	205/218	261/263	174/178	107/107	170/173
144/152	181/181	125/125	117/117	144/161	132/132	165/168	98/98	111/111	211/227	311/316	243/249	175/179	249/249	210/210	208/208	221/227	268/268	170/176	144/172	245/245	252/261	250/274	235/249	202/205	160/160	132/139	192/192	187/188	226/226	261/261	174/174	107/109	174/174
143/152	181/181	125/125	117/117	144/161	137/162	168/177	83/92	111/111	211/221	303/316	238/239	175/179	254/259	210/243	195/213	221/227	256/280	170/176	145/156	245/247	248/252	250/255	233/253	201/205	131/167	125/125	186/186	184/184	218/230	263/263	174/178	107/109	170/170
143/152	181/181	125/125	110/117	144/161	137/162	168/180	83/89	111/111	211/221	303/316	238/239	175/179	254/259	210/243	208/208	221/227	274/280	170/176	144/145	245/250	240/252	250/255	225/249	205/205	131/167	125/125	192/192	173/184	230/234	261/263	174/178	107/109	168/170
143/152	181/181	125/125	110/117	144/161	137/162	168/180	83/89	111/111	211/221	303/316	238/239	175/179	254/259	210/243	208/208	221/227	274/280	170/176	144/145	245/250	240/252	250/255	225/249	205/205	131/167	125/125	192/192	173/184	230/234	261/263	174/178	107/109	168/170
143/152	181/181	125/125	110/117	144/161	137/162	168/180	83/89	111/111	211/221	303/316	238/239	175/179	254/259	210/243	208/208	221/227	274/280	170/176	144/145	245/250	240/252	250/255	225/249	205/205	131/167	125/125	192/192	173/184	230/234	261/263	174/178	107/109	168/170
143/152	181/181	125/125	110/117	144/161	137/162	168/180	83/89	111/111	211/221	303/316	238/239	175/179	254/259	210/243	208/208	221/227	274/280	170/176	144/145	245/250	240/252	250/255	225/249	205/205	131/167	125/125	192/192	173/184	230/234	261/263	174/178	107/109	168/170
143/152	181/181	125/125	110/117	144/161	137/162	168/180	83/89	111/111	211/221	303/316	238/239	175/179	254/259	210/243	208/208	221/227	274/280	170/176	144/145	245/250	240/252	250/255	225/249	205/205	131/167	125/125	192/192	173/184	230/234	261/263	174/178	107/109	168/170
152/152	181/214	125/125	117/126	153/161	137/164	168/177	79/83	111/111	211/221	316/336	239/239	175/179	259/259	211/237	208/213	227/227	256/274	170/170	145/147	245/246	240/252	250/263	235/249	205/205	131/167	125/125	186/192	184/184	205/230	263/263	178/178	107/107	170/170
144/144	181/214	125/125	123/138	143/147	132/164	168/177	83/104	111/111	211/211	311/316	239/249	175/175	259/259	210/249	208/208	227/227	262/274	170/170	145/147	246/250	252/252	255/269	239/251	201/205	131/167	125/125	192/192	180/187	222/226	261/261	174/174	107/109	170/174
149/152	214/214	125/125	117/117	144/144	132/162	168/177	89/91	106/111	211/221	303/316	238/238	175/175	254/254	240/249	208/208	221/221	262/280	176/181	144/146	256/259	240/252	255/255	233/241	205/205	131/131	139/139	192/192	187/187	218/226	261/263	174/174	107/107	168/168

143/152	181/181	125/125	117/117	144/161	132/162	168/177	92/104	111/111	221/227	303/311	239/249	175/179	259/259	210/249	213/214	221/227	262/268	170/176	144/145	245/245	248/252	250/255	239/245	201/205	131/160	125/125	192/192	180/187	218/222	263/267	174/174	107/109	170/174
149/152	181/181	125/125	110/117	144/161	137/162	168/177	104/104	111/111	211/211	303/316	239/243	179/179	259/259	210/210	208/213	227/227	274/280	170/176	145/172	245/245	252/252	250/250	249/249	205/205	131/167	125/125	186/186	184/184	218/230	261/263	174/178	107/109	168/170
152/152	181/181	125/125	117/126	156/161	132/134	168/180	83/104	111/111	211/211	304/316	239/249	170/175	254/259	210/211	208/213	221/227	268/268	170/170	134/144	245/245	252/259	250/255	247/249	201/205	153/160	125/139	186/192	184/187	205/230	261/261	174/178	109/109	170/172
152/152	181/181	125/125	117/126	156/161	132/134	168/180	83/104	111/111	211/211	304/316	239/249	170/175	254/259	210/211	208/213	221/227	268/268	170/170	134/144	245/245	252/259	250/255	247/249	201/205	153/160	125/139	186/192	184/187	205/230	261/261	174/178	109/109	170/172
152/152	181/181	125/125	117/126	156/161	132/134	168/180	83/104	111/111	211/211	304/316	239/249	170/175	254/259	210/211	208/213	221/227	268/268	170/170	134/144	245/245	252/259	250/255	247/249	201/205	153/160	125/139	186/192	184/187	205/230	261/261	174/178	109/109	170/172
152/152	181/181	125/125	117/126	156/161	132/134	168/180	83/104	111/111	211/211	304/316	239/249	170/175	254/259	210/211	208/213	221/227	268/268	170/170	134/144	245/245	252/259	250/255	247/249	201/205	153/160	125/139	186/192	184/187	205/230	261/261	174/178	109/109	170/172
152/152	181/181	125/125	123/131	144/156	132/132	162/162	83/83	111/111	211/211	304/316	249/249	170/175	259/259	210/211	208/208	227/227	256/268	170/170	144/147	245/246	252/252	256/256	245/249	201/205	167/167	125/125	186/192	184/184	230/230	261/261	178/178	109/109	170/172
144/144	187/187	125/125	117/117	144/144	nd.	177/177	92/104	111/111	221/221	303/303	238/243	175/175	254/254	249/249	209/209	221/227	280/280	181/181	144/156	256/259	240/248	255/272	239/239	205/205	131/131	126/126	192/192	172/180	214/218	263/263	174/174	107/107	168/168
152/152	181/181	125/125	117/123	143/153	132/164	162/177	79/83	111/111	211/221	303/305	239/244	175/175	249/259	210/210	208/213	221/227	274/280	170/183	147/167	245/246	240/253	263/288	235/251	201/205	131/160	132/132	nd.	173/180	218/222	261/263	174/174	107/107	170/173
149/152	181/181	125/125	117/123	144/147	132/162	168/177	104/104	111/111	211/227	303/316	238/239	179/179	254/259	210/243	208/213	221/227	256/262	170/176	144/145	245/247	248/252	250/255	239/253	201/205	131/160	139/139	192/192	180/187	218/226	261/263	174/174	107/109	168/170
152/152	181/184	125/125	117/117	144/161	137/155	168/177	83/104	111/111	211/227	303/316	239/239	175/179	259/259	210/243	208/208	227/227	256/274	170/176	145/145	246/247	252/252	250/255	249/249	205/205	131/167	125/125	186/186	184/187	226/230	263/263	174/178	107/109	170/170
152/152	181/184	125/125	117/117	144/161	137/155	168/177	83/104	111/111	211/227	303/316	239/239	175/179	259/259	210/243	208/208	227/227	256/274	170/176	145/145	246/247	252/252	250/255	249/249	205/205	131/167	125/125	186/186	184/187	226/230	263/263	174/178	107/109	170/170
152/152	181/184	125/125	117/117	144/161	137/155	168/177	83/104	111/111	211/227	303/316	239/239	175/179	259/259	210/243	208/208	227/227	256/274	170/176	145/145	246/247	252/252	250/256	249/249	205/205	131/167	125/125	186/186	184/187	226/230	263/263	174/178	107/109	170/170
152/152	181/184	125/125	117/117	144/161	137/155	168/177	83/104	111/111	211/227	303/316	239/239	175/179	259/259	210/243	208/208	227/227	256/274	170/176	145/145	246/247	252/252	250/256	249/249	205/205	131/167	125/125	186/186	184/187	226/230	263/263	174/178	107/109	170/170
152/152	181/184	125/125	117/117	144/161	137/155	168/177	83/104	111/111	211/227	303/316	239/239	175/179	259/259	210/243	208/208	227/227	256/274	170/176	145/145	246/247	252/252	250/256	249/249	205/205	131/167	125/125	186/186	184/187	226/230	263/263	174/178	107/109	170/170
152/152	181/184	125/125	117/117	144/161	137/155	168/177	83/104	111/111	211/227	303/316	239/239	175/179	259/259	210/243	208/208	227/227	256/274	170/176	145/145	246/247	252/252	250/256	249/249	205/205	131/167	125/125	186/186	184/187	226/230	263/263	174/178	107/109	170/170
152/152	181/184	125/125	117/117	144/161	137/155	168/177	83/104	111/111	211/227	303/316	239/239	175/179	259/259	210/243	208/208	227/227	256/274	170/176	145/145	246/247	252/252	250/255	249/249	205/205	131/167	125/125	186/186	184/187	226/230	263/263	174/178	107/109	170/170
152/152	181/184	125/125	117/117	144/161	137/155	168/177	83/104	111/111	211/227	303/316	239/239	175/179	259/259	210/243	208/208	227/227	256/274	170/176	145/145	246/247	252/252	250/256	249/249	205/205	131/167	125/125	186/186	184/187	226/230	263/263	174/178	107/109	170/170
152/152	181/184	125/125	117/117	144/161	137/155	168/177	83/104	111/111	211/227	303/316	239/239	175/179	259/259	210/243	208/208	227/227	256/274	170/176	145/145	246/247	252/252	250/256	249/249	205/205	131/167	125/125	186/186	184/187	226/230	263/263	174/178	107/109	170/170
152/152	181/184	125/125	117/117	144/161	137/155	168/177	83/104	111/111	211/227	303/316	239/239	175/179	259/259	210/243	208/208	227/227	256/274	170/176	145/145	246/247	252/252	250/255	249/249	205/205	131/167	125/125	186/186	184/187	226/230	263/263	174/178	107/109	170/170
152/152	181/184	125/125	117/117	144/161	137/155	168/177	83/104	111/111	211/227	303/316	239/239	175/179	259/259	210/243	208/208	227/227	256/274	170/176	145/145	246/247	252/252	250/255	249/249	205/205	131/167	125/125	186/186	184/187	226/230	263/263	174/178	107/109	170/170
152/152	181/184	125/125	117/117	144/161	137/155	168/177	83/104	111/111	211/227	303/316	239/239	175/179	259/259	210/243	208/208	227/227	256/274	170/176	145/145	246/247	252/252	250/255	249/249	205/205	131/167	125/125	186/186	184/187	226/230	263/263	174/178	107/109	170/170
152/152	181/184	125/125	117/117	144/161	137/155	168/177	83/104	111/111	211/227	303/316	239/239	175/179	259/259	210/243	208/208	227/227	256/274	170/176	145/145	246/247	252/252	250/255	249/249	205/205	131/167	125/125	186/186	184/187	226/230	263/263	174/178	107/109	170/170
152/152	181/184	125/125	117/117	144/161	137/155	168/177	83/104	111/111	211/227	303/316	239/239	175/179	259/259	210/243	208/208	227/227	256/274	170/176	145/145	246/247	252/252	250/255	249/249	205/205	131/167	125/125	186/186	184/187	226/230	263/263	174/178	107/109	170/170
152/152	181/184	125/125	117/117	144/161	137/155	168/177	83/104	111/111	211/227	303/316	239/239	175/179	259/259	210/243	208/208	227/227	256/274	170/176	145/145	246/247	252/252	250/255	249/249	205/205	131/167	125/125	186/186	184/187	226/230	263/263	174/178	107/109	170/170
152/152	181/184	125/125	117/117	144/161	137/155	168/177	83/104	111/111	211/227	303/316	239/239	175/179	259/259	210/243	208/208	227/227	256/274	170/176	145/145	246/247	252/252	250/256	249/249	205/205	131/167	125/125	186/186	184/187	226/230	263/263	174/178	107/109	170/170
152/152	181/184	125/125	117/117	144/161	137/155	168/177	83/104	111/111	211/227	303/316	239/239	175/179	259/259	210/243	208/208	227/227	256/274	170/176	145/145	246/247	252/252	250/256	249/249	205/205	131/167	125/125	186/186	184/187	226/230	263/263	174/178	107/109	170/170
152/152	181/184	125/125	117/117	144/161	137/155	168/177	83/104	111/111	211/227	303/316	239/239	175/179	259/259	210/243	208/208	227/227	256/274	170/176	145/145	246/247	252/252	250/255	249/249	205/205	131/167	125/125	186/186	184/187	226/230	263/263	174/178	107/109	170/170
152/152	181/184	125/125	117/117	144/161	137/155	168/177	83/104	111/111	211/227	303/316	239/239	175/179	259/259	210/243	208/208	227/227	256/274	170/176	145/145	246/247	252/252	250/255	249/249	205/205	131/167	125/125	186/186	184/187	226/230	263/263	174/178	107/109	170/170
152/152	181/184	125/125	117/117	144/161	137/155	168/177	83/104	111/111	211/227	303/316	239/239	175/179	259/259	210/243	208/208	227/227	256/274	170/176	145/145	246/247	252/252	250/255	249/249	205/205	131/167	125/125	186/186	184/187	226/230	263/263	174/178	107/109	170/170
152/152	181/184	125/125	117/117	144/161	137/155	168/177	83/104	111/111	211/227	303/316	239/239	175/179	259/259	210/243	208/208	227/227	256/274	170/176	145/145	246/247	252/252	250/255	249/249	205/205	131/167	125/125	186/186	184/187	226/230	263/263	174/178	107/109	170/170
144/152	181/187	125/125	117/123	147/159	132/162	171/177	83/92	111/111	211/221	307/316	238/239	170/175	259/259	210/240	208/213	221/227	274/280	170/175	142/144	246/247	252/252	255/260	237/239	205/205	131/153	132/132	192/192	173/187	218/222	261/261	172/174	107/109	170/170
152/152	181/181	125/125	123/126	143/156	132/132	162/171	83/83	111/111	211/211	316/316	244/249	179/179	249/259	210/210	208/213	227/227	274/274	170/176	144/167	245/246	252/253	251/255	249/257	201/201	160/167	125/132	186/186	180/187	222/222	261/261	174/174	107/107	170/181
152/152	181/181	125/125	113/117	143/153	132/132	162/171	83/83	111/111	211/211	316/316	244/249	175/175	249/259	210/211	208/213	227/227	268/268	170/183	144/173	245/245	252/253	250/252	233/251	201/205	160/167	125/132	186/192	180/187	222/230	261/261	174/174	107/107	170/181
143/152	181/181	125/125	110/117	144/147	137/162	168/177	104/104	111/111	211/221	303/316	238/239	175/175	259/264	211/243	208/208	221/227	256/280	170/170	144/145	245/256	248/252	250/255	235/249	205/205	131/167	125/125	186/186	184/184	218/230	263/263	174/178	107/109	170/174
152/152	181/181	125/125	113/117	161/161	132/132	165/168	83/104	111/111	211/211	304/316	239/239	179/179	259/259	210/210	208/213	227/227	256/256	170/177	144/145	246/246	252/252	250/268	247/249	201/205	153/160	139/160	192/192	184/187	222/226	261/261	174/178	109/109	170/172
144/152	181/214	125/125	117/126	144/153	134/164	177/180	79/79	111/111	211/221	303/336	238/239	175/179	254/259	210/237	195/208	221/227	274/274	170/176	146/147	246/247	240/252	256/263	235/247	201/205	131/167	125/125	192/192	173/184	205/218	261/263	174/178	107/107	170/173
144/152	181/214	125/125	117/126	144/153	134/164	177/180	79/79	111/111	211/221	303/336	238/239	175/179	254/259	210/237	195/208	221/227	274/274	170/176	146/147	246/247	240/252	256/263	235/247	201/205	131/167	125/125	192/192	173/184	205/218	261/263	174/178	107/107	170/173
144/152	181/214	125/125	117/126	144/153	134/164	177/180	79/79	111/111	211/221	303/336	238/239	175/179	254/259	210/237	195/208	221/227	274/274	170/176	146/147	246/247	240/252	256/263	235/247	201/205	131/167	125/125	192/192	173/184	205/218	261/263	174/178	107/107	170/173
144/152	181/214	125/125	117/126	144/153	134/164	177/180	79/79	111/111	211/221	303/336	238/239	175/179	254/259	210/237	195/208	221/227	274/274	170/176	146/147	246/247	240/252	256/263	235/247	201/205	131/167	125/125	192/192	173/184	205/218	261/263	174/178	107/107	170/173
144/152	181/214	125/125	117/126	144/153	134/164	177/180	79/79	111/111	211/221	303/336	238/239	175/179	254/259	210/237	195/208	221/227	274/274	170/176	146/147	246/247	240/252	256/263	235/247	201/205	131/167	125/125	192/192	173/184	205/218	261/263	174/178	107/107	170/173
144/149	181/181	122/125	110/138	144/159	126/132	168/174	83/98	111/111	211/211	311/313	244/249	175/175	254/259	210/231	208/233	221/227	268/274	170/170	144/154	246/247	252/261	269/274	247/251	201/201	131/167	125/125	192/192	187/188	222/226	263/263	174/174	107/109	172/174
144/149	214/214	125/125	117/117	144/144	162/162	177/177	89/104	111/111	211/221	303/303	238/238	175/175	254/254	240/249	208/208	221/221	280/280	181/181	146/156	250/259	240/240	255/255	233/239	205/205	131/131	126/126	nd.	173/173	214/226	261/263	174/174	107/107	168/172
152/152	181/181	125/125	113/117	156/159	137/137	168/168	83/83	111/111	211/211	316/316	249/249	175/179	259/259	210/210	208/208	227/227	256/268	170/170	142/144	245/245	252/259	253/269	249/251	201/201	160/167	125/125	186/186	184/187	230/230	261/263	174/178	109/109	170/170
144/152	181/181	125/125	117/123	144/153	132/132	162/168	83/104	111/111	211/211	316/316	234/239	179/179	249/259	211/249	208/213	227/227	256/274	170/170	142/145	245/246	252/253	269/269	251/253	201/201	160/160	125/139	186/186	184/187	222/230	261/261	174/178	109/109	170/170
144/152	181/208	125/125	117/131	144/161	134/155	168/177	104/144	111/111	211/221	303/316	249/249	175/175	259/259	210/243	208/208	221/227	256/262	170/176	142/147	245/245	252/262	250/257	239/247	201/205	131/167	125/125	186/186	172/184	222/230	261/263	174/178	107/109	170/172
144/152	181/208	125/125	117/131	144/161	134/155	168/177	104/144	111/111	211/221	303/316	249/249	175/175	259/259	210/243	208/208	221/227	256/262	170/176	142/147	245/245	252/262	250/257	239/247	201/205	131/167	125/125	186/186	172/184	222/230	261/263	174/178	107/109	170/172
144/152	181/208	125/125	117/131	144/161	134/155	168/177	104/144	111/111	211/221	303/316	249/249	175/175	259/259	210/243	208/208	221/227	256/262	170/176	142/147	245/245	252/262	250/257	239/247	201/205	131/167	125/125	186/186	172/184	222/230	261/263	174/178	107/109	170/172
144/152	181/208	125/125	117/131	144/161	134/155	168/177	104/144	111/111	211/221	303/316	249/249	175/175	259/259	210/243	208/208	221/227	256/262	170/176	142/147	245/245	252/262	250/257	239/247	201/205	131/167	125/125	186/186	172/184	222/230	261/263	174/178	107/109	170/172
144/152	181/208	125/125	117/131	144/161	134/155	168/177	104/144	111/111	211/221	303/316	249/249	175/175	259/259	210/243	208/208	221/227	256/262	170/176	142/147	245/245	252/262	250/257	239/247	201/205	131/167	125/125	186/186	172/184	222/230	261/263	174/178	107/109	170/172
144/152	181/208	125/125	117/131	144/161	134/155	168/177	104/144	111/111	211/221	303/316	249/249	175/175	259/259	210/243	208/208	221/227	256/262	170/176	142/147	245/245	252/262	250/257	239/247	201/205	131/167	125/125	186/186	172/184	222/230	261/263	174/178	107/109	170/172
144/152	181/208	125/125	117/131	144/161	134/155	168/177	104/144	111/111	211/221	303/316	249/249	175/175	259/259	210/243	208/208	221/227	256/262	170/176	142/147	245/245	252/262	250/257	239/247	201/205	131/167	125/125	186/186	172/184	222/230	261/263	174/178	107/109	170/172
144/152	181/208	125/125	117/131	144/161	134/155	168/177	104/144	111/111	211/221	303/316	249/249	175/175	259/259	210/243	208/208	221/227	256/262	170/176	142/147	245/245	252/262	250/257	239/247	201/205	131/167	125/125	186/186	172/184	222/230	261/263	174/178	107/109	170/172
144/152	181/208	125/125	117/131	144/161	134/155	168/177	104/144	111/111	211/221	303/316	249/249	175/175	259/259	210/243	208/208	221/227	256/262	170/176	142/147	245/245	252/262	250/257	239/247	201/205	131/167	125/125	186/186	172/184	222/230	261/263	174/178	107/109	170/172
144/152	181/208	125/125	117/131	144/161	134/155	168/177	104/147	111/111	211/221	303/316	249/249	175/175	259/259	210/243	208/208	221/227	256/262	170/176	142/147	245/245	252/262	250/257	239/247	201/205	131/167	125/125	186/186	172/184	222/230	261/263	174/178	107/109	170/172
144/152	181/208	125/125	117/131	144/161	134/155	168/177	104/144	111/111	211/221	303/316	249/249	175/175	259/259	210/243	208/208	221/227	256/262	170/176	142/147	245/245	252/262	250/257	239/247	201/205	131/167	125/125	186/186	172/184	222/230	261/263	174/178	107/109	170/172
144/152	181/208	125/125	117/131	144/161	134/155	168/177	104/144	111/111	211/221	303/316	249/249	175/175	259/259	210/243	208/208	221/227	256/262	170/176	142/147	245/245	252/262	250/257	239/247	201/205	131/167	125/125	186/186	172/184	222/230	261/263	174/178	107/109	170/172
144/152	181/208	125/125	117/131	144/161	134/155	168/177	104/144	111/111	211/221	303/316	249/249	175/175	259/259	210/243	208/208	221/227	256/262	170/176	142/147	245/245	252/262	250/257	239/247	201/205	131/167	125/125	186/186	172/184	222/230	261/263	174/178	107/109	170/172
144/152	181/208	125/125	117/131	144/161	134/155	168/177	104/144	111/111	211/221	303/316	249/249	175/175	259/259	210/243	208/208	221/227	256/262	170/176	142/147	245/245	252/262	250/257	239/247	201/205	131/167	125/125	186/186	172/184	222/230	261/263	174/178	107/109	170/172
144/152	181/208	125/125	117/131	144/161	134/155	168/177	104/144	111/111	211/221	303/316	249/249	175/175	259/259	210/243	208/208	221/227	256/262	170/176	142/147	245/245	252/262	250/257	239/247	201/205	131/167	125/125	186/186	172/184	222/230	261/263	174/178	107/109	170/172
144/152	181/208	125/125	117/131	144/161	134/155	168/177	104/144	111/111	211/221	303/316	249/249	175/175	259/259	210/243	208/208	221/227	256/262	170/176	142/147	245/245	252/262	250/257	239/247	201/205	131/167	125/125	186/186	172/184	222/230	261/263	174/178	107/109	170/172
144/152	181/208	125/125	117/131	144/161	134/155	168/177	104/144	111/111	211/221	303/316	249/249	175/175	259/259	210/243	208/208	221/227	256/262	170/176	142/147	245/245	252/262	250/257	239/247	201/205	131/167	125/125	186/186	172/184	222/230	261/263	174/178	107/109	170/172
144/152	181/208	125/125	117/131	144/161	134/155	168/177	104/144	111/111	211/221	303/316	249/249	175/175	259/259	210/243	208/208	221/227	256/262	170/176	142/147	245/245	252/262	250/257	239/247	201/205	131/167	125/125	186/186	172/184	222/230	261/263	174/178	107/109	170/172
144/152	181/208	125/125	117/131	144/161	134/155	168/177	104/147	111/111	211/221	303/316	249/249	175/175	259/259	210/243	208/208	221/227	256/262	170/176	142/147	245/245	252/262	250/257	239/247	201/205	131/167	125/125	186/186	172/184	222/230	261/263	174/178	107/109	170/172
144/152	181/208	125/125	117/131	144/161	134/155	168/177	104/147	111/111	211/221	303/316	249/249	175/175	259/259	210/243	208/208	221/227	256/262	170/176	142/147	245/245	252/262	250/257	239/247	201/205	131/167	125/125	186/186	172/184	222/230	261/263	174/178	107/109	170/172
144/152	181/181	125/125	123/129	143/153	132/132	162/162	83/83	111/111	211/211	306/306	244/244	175/175	249/249	210/211	213/213	227/227	256/280	176/183	161/173	245/246	252/253	251/256	245/249	201/201	160/160	132/132	192/192	180/180	222/222	261/261	174/174	107/109	170/181
144/152	181/181	125/125	123/138	143/153	132/132	162/168	83/83	111/111	211/211	305/316	234/244	175/175	249/249	210/210	213/213	227/227	256/268	176/183	142/167	245/246	252/253	251/256	245/251	201/201	160/167	125/132	186/186	180/187	222/226	261/261	174/174	107/109	170/181
144/152	181/181	125/125	123/138	143/153	nd.	162/168	83/83	111/111	211/211	305/316	234/244	175/175	249/249	210/210	213/213	227/227	256/268	176/183	142/167	245/246	252/253	251/256	245/251	201/201	160/167	125/132	186/186	180/187	222/226	261/261	174/174	107/109	170/181
144/152	181/181	125/125	123/138	143/153	132/132	162/168	83/83	111/111	211/211	305/316	234/244	175/175	249/249	210/210	213/213	227/227	256/268	176/183	142/167	245/246	252/253	251/256	245/251	201/201	160/167	125/132	186/186	180/187	222/226	261/261	174/174	107/109	170/181
144/152	181/181	125/125	123/129	143/153	132/132	162/162	83/83	111/111	211/211	306/306	244/244	175/175	249/249	210/211	213/213	227/227	256/280	176/183	173/173	245/246	252/253	251/256	245/249	201/201	160/160	132/132	192/192	180/180	222/222	261/261	174/174	107/109	170/181
144/152	181/181	125/125	123/129	143/153	132/132	162/162	83/83	111/111	211/211	306/306	244/244	175/175	249/249	210/211	213/213	227/227	256/280	176/183	173/173	245/246	252/253	251/256	245/249	201/201	160/160	132/132	192/192	180/180	222/222	261/261	174/174	107/109	170/181
144/152	181/181	125/125	135/138	143/144	132/132	162/168	83/83	111/111	211/211	306/311	244/249	175/179	249/259	210/210	208/213	227/227	274/280	170/176	147/165	245/246	252/253	251/269	245/251	201/201	160/167	125/132	192/192	180/187	222/222	261/261	174/174	107/109	170/181
144/152	181/181	125/125	123/138	143/153	132/132	162/168	83/83	111/111	211/211	305/316	234/244	175/175	249/249	210/210	213/213	227/227	256/268	176/183	142/167	245/246	252/253	251/256	245/251	201/201	160/167	125/132	186/186	180/187	222/226	261/261	174/174	107/109	170/181
144/152	181/181	125/125	123/138	143/153	132/132	162/168	83/83	111/111	211/211	305/316	234/244	175/175	249/249	210/210	213/213	227/227	256/268	176/183	142/167	245/246	252/253	251/256	245/251	201/201	160/167	125/132	186/186	180/187	222/226	261/261	174/174	107/109	170/181
144/152	181/181	125/125	123/138	143/153	132/132	162/168	83/83	111/111	211/211	305/316	234/244	175/175	249/249	210/210	213/213	227/227	256/268	176/183	142/167	245/246	252/253	251/256	245/251	201/201	160/167	125/132	186/186	180/187	222/226	261/261	174/174	107/109	170/181
144/152	181/181	125/125	123/129	143/153	132/132	162/162	83/83	111/111	211/211	306/306	244/244	175/175	249/249	210/211	213/213	227/227	256/280	176/183	161/173	245/246	252/253	251/256	245/249	201/201	160/160	132/132	192/192	180/180	222/222	261/261	174/174	107/109	170/181
144/152	181/181	125/125	123/138	143/153	132/132	162/168	83/83	111/111	211/211	305/316	234/244	175/175	249/249	210/210	213/213	227/227	256/268	176/183	142/167	245/246	252/253	251/256	245/251	201/201	160/167	125/132	186/186	180/187	222/226	261/261	174/174	107/109	170/181
149/152	181/181	125/125	110/123	143/144	132/132	162/168	83/104	111/111	211/211	305/316	244/249	165/175	249/259	210/210	208/213	227/227	268/274	170/176	147/167	245/253	248/253	251/257	245/251	201/201	160/167	125/132	192/192	180/180	218/222	261/263	174/174	107/109	172/181
144/144	214/214	125/125	123/126	144/147	134/164	177/180	79/104	106/106	221/221	303/336	238/238	179/179	254/254	210/237	195/195	221/227	262/274	176/176	144/146	247/250	248/252	255/255	239/247	201/205	131/167	125/125	192/192	180/184	205/214	261/261	174/178	107/107	168/170
152/152	181/181	125/125	123/131	144/159	132/134	162/168	83/144	111/111	211/221	304/316	249/249	170/175	259/259	210/211	208/208	221/227	256/286	170/170	142/147	245/245	252/262	256/257	245/247	201/201	167/167	125/125	186/192	184/184	222/230	261/261	178/178	109/109	170/172
152/152	181/181	125/125	123/131	144/159	132/134	162/168	83/144	111/111	211/221	304/316	249/249	170/175	259/259	210/211	208/208	221/227	256/286	170/170	142/147	245/245	252/262	257/257	245/247	201/201	167/167	125/125	186/192	184/184	222/230	261/261	178/178	109/109	170/172
152/152	181/181	125/125	123/131	144/159	132/134	162/168	83/144	111/111	211/221	304/316	249/249	170/175	259/259	210/211	208/208	221/227	256/286	170/170	142/147	245/245	252/262	257/257	245/247	201/201	167/167	125/125	186/192	184/184	222/230	261/261	178/178	109/109	170/172
152/152	181/181	125/125	123/131	144/159	132/134	162/168	83/144	111/111	211/221	304/316	249/249	170/175	259/259	210/211	208/208	221/227	256/286	170/170	142/147	245/245	252/262	257/257	245/247	201/201	167/167	125/125	186/192	184/184	222/230	261/261	178/178	109/109	170/172
144/152	181/181	125/125	113/131	144/153	137/155	168/177	83/144	111/111	211/221	303/316	239/249	175/175	259/259	210/210	208/208	221/227	262/268	170/170	142/144	245/245	259/262	257/257	247/249	201/205	131/160	125/125	186/186	172/187	222/222	263/263	174/174	109/109	170/172
144/152	181/184	125/125	117/131	144/144	132/134	168/168	104/104	111/111	211/221	316/316	238/249	175/175	254/259	210/249	208/208	221/227	256/268	170/170	142/144	245/250	252/252	250/256	233/247	201/205	160/167	125/139	186/192	184/187	226/230	261/261	174/178	107/109	168/170
144/152	181/181	125/125	126/131	161/161	132/155	171/177	83/104	111/111	211/221	303/316	249/249	170/175	259/259	210/210	208/208	227/227	256/268	170/170	147/147	245/246	259/262	257/269	239/249	201/205	131/167	125/125	192/192	184/187	226/230	261/263	174/178	107/109	170/172
144/149	181/181	125/125	123/138	144/153	132/132	162/168	83/83	111/111	211/211	316/316	234/249	175/175	249/259	210/249	208/213	227/227	274/280	170/183	142/144	245/253	252/253	269/269	247/251	201/201	160/167	125/125	186/192	180/187	218/222	261/263	174/174	107/109	170/172
152/152	181/184	125/125	117/117	144/161	132/155	168/177	104/104	111/111	211/227	303/316	239/243	175/175	259/259	210/243	208/208	227/227	268/274	170/176	145/172	245/247	252/252	250/256	239/249	205/205	131/160	139/139	192/192	184/187	226/230	261/263	174/174	107/109	170/174
149/152	214/214	125/125	117/117	144/144	132/162	168/177	89/91	106/111	211/221	303/316	238/238	175/175	254/254	240/249	208/208	221/221	262/280	176/181	144/146	256/259	240/252	255/255	233/241	205/205	131/131	139/139	192/192	187/187	218/226	261/263	174/174	107/107	168/168
152/152	181/181	125/125	117/123	143/153	132/137	162/168	83/83	111/111	211/211	316/316	243/249	175/175	259/259	210/210	208/208	227/227	268/274	170/176	144/172	246/247	253/253	251/256	233/257	201/205	160/167	125/132	186/186	187/187	222/230	261/263	174/174	107/107	174/181
152/152	181/184	125/125	117/117	144/161	132/155	168/177	104/104	111/111	211/227	303/316	239/243	175/175	259/259	210/243	208/208	227/227	268/274	170/176	145/172	245/247	252/252	250/256	239/249	205/205	131/160	139/139	192/192	184/187	226/230	261/263	174/174	107/109	170/174
152/152	181/184	125/125	117/117	144/161	132/155	168/177	104/104	111/111	211/227	303/316	239/243	175/175	259/259	210/243	208/208	227/227	268/274	170/176	145/172	245/247	252/252	250/256	239/249	205/205	131/160	139/139	192/192	184/187	226/230	261/263	174/174	107/109	170/174
152/152	181/184	125/125	117/117	144/161	132/155	168/177	104/104	111/111	211/227	303/316	239/243	175/175	259/259	210/243	208/208	227/227	268/274	170/176	145/172	245/247	252/252	250/256	239/249	205/205	131/160	139/139	192/192	184/187	226/230	261/263	174/174	107/109	170/174
143/143	185/185	125/125	104/117	144/147	162/162	177/177	89/92	111/111	221/221	303/303	238/238	175/175	254/254	240/243	208/208	221/221	280/280	176/176	144/156	250/256	248/252	255/255	233/233	205/205	131/131	140/140	192/192	180/180	218/239	267/267	174/174	107/107	168/168
152/152	181/184	125/125	110/117	144/147	132/162	168/177	92/104	111/111	211/221	303/316	238/239	175/179	259/264	210/237	208/208	221/227	256/280	170/176	144/145	245/256	252/252	250/255	239/253	201/205	131/160	139/139	192/192	180/187	218/226	261/261	174/174	107/107	170/170
152/152	181/184	125/125	113/117	143/144	132/132	162/168	83/104	111/111	211/227	306/316	243/244	175/175	249/249	210/243	208/213	221/227	274/280	176/176	167/172	245/247	252/252	252/255	239/251	201/205	160/160	132/139	192/192	180/187	222/226	261/263	174/174	107/107	170/181
144/152	184/187	125/125	117/117	144/147	155/164	168/177	83/119	111/111	211/221	303/303	238/239	175/179	259/259	210/240	208/208	221/227	256/280	175/176	144/145	246/250	252/252	250/255	233/249	205/205	131/167	126/140	nd.	187/200	218/226	261/263	174/174	107/107	168/170
152/152	181/181	125/125	113/113	147/156	134/137	168/168	83/104	111/111	211/211	316/316	249/249	175/175	259/259	210/210	208/208	221/227	256/268	170/170	142/147	245/246	259/259	250/255	245/249	201/205	153/160	125/125	186/186	184/184	222/230	261/263	178/178	109/109	170/174
152/152	181/181	125/125	113/113	147/159	132/132	168/168	83/104	111/111	211/211	316/316	249/249	175/175	259/259	210/210	208/208	227/227	256/256	170/170	142/147	245/246	259/259	250/255	245/249	201/205	153/160	125/139	186/186	184/187	222/230	261/263	178/178	109/109	170/174
144/149	181/184	125/125	123/123	144/147	137/162	168/177	83/92	111/111	211/221	303/316	239/243	nd.	254/259	210/243	195/208	221/227	256/262	170/176	145/172	246/247	248/252	255/255	239/249	205/205	131/160	125/125	186/186	180/184	218/230	263/263	174/178	107/109	168/170
149/152	181/214	125/125	117/117	144/161	134/164	168/177	91/144	106/106	211/221	303/316	238/249	175/175	259/259	210/249	208/208	221/227	256/280	170/176	142/144	245/256	252/252	250/255	233/247	205/205	131/167	125/125	186/186	180/184	214/230	261/261	174/178	107/109	168/172
152/152	181/184	125/125	117/117	144/144	132/137	168/168	104/104	111/111	211/227	316/316	239/243	175/175	259/259	211/243	208/213	221/227	256/268	170/176	145/172	245/247	252/252	250/255	239/249	205/205	160/167	125/139	186/192	184/187	226/230	261/263	174/174	107/109	170/174
149/152	181/214	125/125	117/117	144/147	132/162	168/177	91/92	111/111	211/221	303/316	238/243	175/175	264/264	210/240	195/208	221/221	280/280	176/176	144/150	245/253	248/252	250/255	233/253	205/205	131/160	139/139	192/192	180/180	214/218	261/261	174/174	107/107	168/170
152/152	181/181	125/125	113/117	147/153	132/134	168/195	83/83	111/111	211/211	316/316	249/249	175/175	259/259	210/210	208/208	227/227	268/268	170/170	142/144	246/246	252/259	250/255	245/249	201/205	153/167	125/125	186/192	184/184	222/230	261/261	178/178	109/109	170/170
149/152	181/181	125/125	117/123	161/161	137/162	168/177	92/92	111/111	221/221	303/316	239/239	179/179	249/259	210/243	208/213	227/227	256/262	170/176	145/145	246/247	248/252	255/255	239/249	205/205	131/167	125/125	186/186	180/184	218/230	263/263	174/178	107/109	168/170
144/158	181/181	122/125	110/117	159/161	126/132	165/174	83/98	111/111	211/211	311/313	244/249	179/179	249/254	210/231	233/233	221/227	268/286	170/176	144/154	245/247	252/261	274/274	235/251	201/202	131/160	132/140	192/192	188/188	222/226	261/263	174/174	107/107	172/174
152/152	181/184	125/125	117/117	156/161	137/155	168/177	83/104	111/111	nd.	316/316	239/249	175/175	259/259	210/243	208/208	221/227	256/268	170/170	145/147	247/253	248/252	255/255	239/249	201/205	131/167	125/125	186/186	184/184	230/230	263/263	178/178	107/109	170/172
152/152	181/181	125/125	117/117	156/161	134/137	168/177	89/104	111/111	211/227	316/316	239/249	175/179	259/259	210/243	208/208	221/227	256/268	170/176	145/147	246/247	252/252	250/255	239/249	205/205	131/167	125/125	186/186	184/187	230/230	261/263	174/178	109/109	170/172

152/152	181/208	125/125	117/131	144/144	137/155	168/177	104/144	111/111	211/227	303/316	239/249	175/179	259/259	210/243	208/208	221/227	256/256	170/176	142/145	245/247	252/262	255/257	239/249	205/205	131/167	125/125	186/186	172/184	222/230	263/263	174/178	107/109	170/170
144/152	181/214	125/125	113/117	144/147	132/134	168/168	104/134	111/111	211/211	316/316	238/249	175/175	254/259	210/243	208/208	227/227	256/280	170/181	142/172	245/256	243/259	250/255	239/245	201/205	131/160	125/139	186/186	184/187	230/230	261/261	174/178	107/109	170/172
152/152	181/181	125/125	117/117	147/161	132/137	168/168	83/104	111/111	211/227	316/316	239/249	175/179	259/259	210/243	208/208	221/227	256/274	169/176	145/147	246/247	252/259	255/255	245/249	201/205	160/167	125/125	186/192	184/184	230/234	261/263	178/178	107/109	170/174
144/152	181/208	125/125	113/131	144/147	134/134	168/168	83/144	111/111	211/221	316/316	249/249	175/175	259/259	210/243	208/208	227/227	256/256	170/176	142/147	245/245	262/262	257/257	245/247	201/201	167/167	125/125	186/186	172/184	222/230	261/261	174/178	107/109	170/172
144/152	181/181	125/125	117/126	144/161	132/134	177/180	83/144	111/111	211/221	304/316	239/239	170/175	254/259	210/243	208/208	221/227	256/268	170/170	134/145	245/247	252/259	255/255	247/249	201/205	131/153	125/139	186/186	184/187	205/230	261/263	174/178	107/109	170/170
138/143	nd.	125/125	91/123	144/161	151/162	177/177	125/125	111/111	221/227	303/303	238/238	nd.	254/264	237/249	208/208	221/221	274/280	170/176	144/156	244/253	240/258	255/288	233/239	201/205	131/131	126/126	nd.	173/181	218/218	263/263	174/176	107/107	174/174
144/152	181/214	125/125	117/123	147/161	132/155	168/177	83/104	111/111	211/221	303/316	234/249	175/175	249/259	243/243	208/213	227/227	256/268	170/176	142/147	245/246	252/262	257/257	239/251	201/205	131/167	125/125	186/186	184/187	226/230	261/263	174/178	107/109	170/170
152/152	181/208	125/125	113/117	144/161	132/134	168/168	83/104	111/111	211/211	316/316	249/249	175/179	259/259	210/210	208/208	227/227	256/256	170/170	142/147	246/246	252/259	250/255	245/249	201/205	153/167	125/125	186/192	184/184	230/234	261/261	178/178	107/109	170/174
143/149	187/187	125/125	123/123	144/144	162/164	177/177	116/116	111/111	221/221	303/303	238/243	nd.	254/254	243/249	208/208	221/221	280/280	175/181	150/172	247/247	248/252	255/255	233/239	205/205	131/131	126/126	nd.	180/180	214/218	261/261	174/174	107/107	172/172
143/144	187/190	125/125	117/117	144/144	162/162	177/177	104/104	111/111	221/221	303/303	238/243	175/175	254/254	240/249	208/209	227/227	280/280	181/181	144/156	253/256	248/252	255/272	239/239	205/205	131/131	126/126	nd.	180/180	214/218	263/263	174/174	107/107	168/168
152/152	181/181	125/125	113/117	144/144	134/137	168/180	83/104	111/111	211/227	316/316	239/249	175/175	259/259	210/210	208/208	221/227	256/274	170/176	145/147	245/247	252/252	250/255	239/249	205/205	131/153	125/125	186/186	184/184	226/230	261/263	178/178	109/109	172/174
152/152	181/181	125/125	113/117	144/147	132/132	168/168	83/83	111/111	211/211	316/316	249/249	175/179	254/259	210/210	208/208	221/227	256/274	169/170	144/147	246/247	259/259	253/255	245/245	201/201	153/160	125/125	186/192	184/184	230/234	261/263	178/178	107/109	174/174
152/152	181/184	125/125	113/131	144/161	132/155	168/177	83/89	111/111	211/211	316/316	249/249	175/175	259/259	210/210	208/208	221/227	256/268	170/181	142/147	245/245	252/259	250/250	239/245	201/205	131/160	139/139	186/186	184/187	222/230	261/263	174/178	107/109	170/174
152/152	181/181	125/125	113/131	144/156	132/155	168/177	83/144	111/111	211/211	316/316	249/249	175/175	259/259	210/240	208/208	221/227	256/268	170/176	134/142	245/245	252/259	250/250	239/245	201/205	131/160	139/139	186/186	187/187	205/222	261/263	174/174	109/109	170/174
152/152	181/184	125/125	117/117	161/161	134/137	168/177	83/104	111/111	221/227	316/316	239/239	175/175	259/259	210/210	208/208	227/227	256/274	170/176	145/145	246/247	252/252	250/250	245/249	201/205	131/167	125/125	186/186	184/187	230/230	261/263	174/178	107/109	170/172
152/152	181/181	125/125	117/117	144/156	132/134	168/180	83/83	111/111	211/211	316/316	249/249	175/179	254/259	210/210	208/213	221/227	256/268	169/170	144/144	245/246	252/259	250/255	245/247	201/201	153/160	125/125	186/186	184/184	230/230	261/263	178/178	107/109	170/174
144/152	181/214	125/125	117/123	144/147	132/155	168/177	83/83	111/111	211/227	303/316	239/249	175/175	259/259	243/243	208/213	227/227	256/268	170/176	145/147	245/247	252/262	255/257	249/251	201/205	131/167	139/139	186/186	184/184	230/230	261/263	178/178	107/107	170/170
144/152	181/214	125/125	110/123	144/147	132/137	168/168	89/89	111/111	211/227	316/316	234/239	179/179	249/259	210/210	208/208	221/221	268/280	170/181	142/145	245/246	240/252	255/255	225/251	201/205	131/167	125/125	186/192	184/187	226/230	261/263	174/178	107/109	170/170
152/152	181/181	125/125	117/126	161/161	134/137	168/180	83/104	111/111	221/221	304/316	239/249	170/175	254/259	211/243	208/208	221/227	256/268	170/170	134/142	245/247	252/259	255/255	239/247	201/205	153/167	125/125	186/186	184/187	205/230	261/263	174/178	109/109	170/172
152/152	181/181	125/125	117/117	161/161	132/137	168/168	83/104	111/111	211/227	304/316	239/239	175/179	254/259	211/243	208/213	227/227	256/268	170/170	134/145	245/246	252/252	250/250	247/249	201/205	160/167	125/139	186/192	184/187	205/230	261/263	174/178	107/109	170/170
144/149	nd.	125/125	123/123	144/144	162/162	177/177	116/116	111/111	221/221	303/303	238/243	nd.	254/254	243/243	208/208	221/227	280/280	181/181	150/172	247/247	248/252	255/255	233/239	205/205	131/131	126/126	nd.	180/180	214/214	261/263	174/174	107/107	172/172
144/152	181/181	125/125	117/123	143/161	137/164	168/177	83/104	111/111	211/227	316/316	239/249	175/175	259/259	210/243	208/208	227/227	256/274	170/176	142/145	245/250	252/252	250/255	239/251	201/205	131/167	125/125	186/192	184/187	226/230	261/263	174/178	109/109	170/170
152/152	181/181	125/125	117/117	144/161	134/137	168/177	89/104	111/111	211/227	316/316	249/249	175/179	259/259	210/243	208/208	221/227	256/268	169/170	142/144	246/247	252/259	253/255	249/249	205/205	131/167	125/125	186/186	184/187	230/230	261/263	174/178	109/109	170/172
152/152	181/181	125/125	113/117	153/159	132/134	168/168	83/104	111/111	211/211	316/316	249/249	175/179	254/259	210/211	208/208	221/227	256/286	169/170	142/144	245/246	252/259	250/255	245/245	201/201	160/167	125/139	186/192	184/184	230/234	261/261	178/178	107/109	170/174
144/152	181/181	125/125	117/126	156/161	132/137	168/168	83/144	111/111	211/211	304/316	239/249	170/175	254/259	210/243	208/208	221/227	256/268	170/176	144/147	245/247	252/259	255/255	247/249	201/205	160/167	125/139	186/192	184/187	205/230	261/263	174/178	109/109	170/170
152/152	181/184	125/125	117/117	144/144	134/155	168/177	83/104	111/111	211/227	303/316	239/249	179/179	259/259	210/210	208/208	221/227	256/274	169/176	144/145	246/247	252/259	250/253	239/249	205/205	167/167	125/125	192/192	184/187	226/234	261/263	174/178	107/107	170/172
152/152	181/181	125/125	113/117	144/147	132/132	168/168	83/83	111/111	211/227	316/316	249/249	175/179	259/259	210/210	208/208	221/221	256/274	170/176	144/147	245/247	259/259	250/253	245/249	201/201	160/167	139/139	186/192	184/187	222/234	261/261	174/178	109/109	172/174
152/152	181/181	125/125	113/117	147/153	132/134	168/168	83/104	111/111	211/211	316/316	249/249	175/179	254/259	210/210	208/208	221/221	256/274	169/170	142/144	245/246	259/259	250/253	245/245	201/201	153/167	125/125	186/192	184/184	222/234	261/263	178/178	107/109	172/174
152/152	181/181	125/125	117/117	161/161	137/155	168/177	104/104	111/111	221/227	303/316	239/249	175/175	259/259	243/243	208/208	221/227	256/256	170/176	142/145	245/247	252/252	250/255	239/249	205/205	131/167	125/125	186/186	172/184	222/230	263/263	174/178	109/109	170/172
144/144	181/214	125/125	123/123	143/147	132/162	168/177	83/83	111/111	211/227	303/316	234/238	nd.	249/254	210/243	208/213	221/227	268/268	176/181	142/144	246/250	252/252	255/255	239/251	201/205	131/167	125/125	186/186	187/187	226/226	261/263	174/174	107/109	168/170
152/152	181/181	125/125	113/117	144/156	132/134	168/180	89/144	111/111	211/211	316/316	249/249	175/175	259/259	210/210	208/208	221/227	256/268	170/170	134/142	245/245	252/259	250/250	245/247	201/201	153/160	125/139	186/186	187/187	205/222	261/261	174/174	109/109	170/172
143/149	181/187	125/125	117/123	144/161	132/162	177/177	92/92	106/111	221/227	303/316	238/243	175/175	254/254	243/249	208/208	221/221	280/280	170/181	144/150	245/247	248/252	250/255	239/239	205/205	131/131	139/139	192/192	180/187	214/226	261/261	174/174	107/107	168/172
152/152	181/181	125/125	117/117	147/156	137/155	168/177	104/104	111/111	211/211	303/316	239/249	175/175	259/259	210/210	208/208	227/227	256/268	169/176	144/147	245/247	252/259	255/255	245/249	201/205	160/167	125/125	186/186	184/187	226/230	263/263	174/178	107/107	170/174
152/152	181/181	125/125	113/117	144/147	132/155	168/177	104/104	111/111	211/227	303/316	239/249	175/175	259/259	210/243	208/208	227/227	256/256	169/170	145/147	246/247	252/259	255/255	245/249	201/205	153/167	125/125	186/186	184/187	226/234	263/263	174/178	107/107	170/174
152/152	181/184	125/125	117/131	144/156	134/155	177/180	83/89	111/111	211/221	316/316	249/249	175/175	259/259	210/240	208/208	221/227	256/268	170/181	144/147	245/245	252/252	250/250	239/249	201/205	131/153	125/125	186/186	184/187	205/230	261/263	174/178	107/109	170/172
152/152	181/184	125/125	113/117	144/161	132/137	168/168	83/104	111/111	211/227	316/316	239/249	175/175	259/259	210/243	208/208	221/227	256/256	176/176	142/145	245/247	252/262	255/257	245/249	201/205	131/167	125/139	186/186	172/184	222/226	261/263	174/178	107/109	170/174
144/152	181/181	125/125	117/131	161/161	134/137	168/168	104/144	111/111	211/227	316/316	239/249	175/175	259/259	243/243	208/208	227/227	256/256	170/176	145/147	245/247	252/262	255/257	247/249	201/205	131/167	125/125	186/186	184/184	230/230	261/263	178/178	109/109	170/170
152/152	181/181	125/125	117/117	144/161	132/132	168/168	83/144	111/111	211/211	316/316	239/249	175/175	259/259	210/211	208/213	221/227	256/256	170/170	134/134	245/245	252/259	250/250	245/253	201/201	160/160	139/139	186/192	184/187	222/230	261/261	174/178	109/109	170/172
152/152	181/181	125/125	117/117	144/161	132/137	168/168	83/104	111/111	211/227	316/316	239/249	179/179	259/259	211/211	208/208	221/227	256/286	169/170	144/145	245/247	252/259	250/253	245/249	201/205	160/167	125/139	186/192	184/184	230/230	261/263	178/178	107/109	170/172
138/143	181/181	125/125	91/104	144/144	151/162	177/177	92/92	111/111	221/227	303/303	238/238	175/175	254/254	237/249	208/213	221/227	280/280	170/176	144/144	244/259	248/258	255/288	233/239	201/205	131/131	126/126	nd.	173/181	214/214	263/263	174/176	107/109	168/174
152/152	181/181	125/125	117/131	144/161	134/134	168/180	83/89	111/111	211/211	316/316	249/249	175/175	259/259	210/240	208/208	221/227	256/268	170/176	134/147	245/245	252/262	250/257	245/247	201/201	153/167	125/125	186/186	172/187	205/222	261/261	174/174	107/109	170/172
152/152	181/184	125/125	117/117	144/161	134/137	168/180	104/144	111/111	211/227	316/316	239/239	175/179	259/259	210/243	208/208	221/227	256/274	170/176	145/145	246/247	252/252	250/255	239/249	nd.	167/167	125/125	186/186	184/184	226/230	261/263	178/178	107/109	170/172
152/152	181/184	125/125	117/131	156/161	134/155	177/180	83/89	111/111	211/211	316/316	249/249	175/175	259/259	210/240	208/208	227/227	262/268	170/181	134/147	245/245	252/252	250/250	239/247	201/205	131/153	125/125	186/186	184/187	205/230	261/263	174/178	107/109	170/172
143/144/152	181/184/187	125/125	117/131	144/161	155/162	177/177	89/104	111/111	211/221	303/316	238/243	175/175	254/254	210/240/249	208/209	221/227	256/262/280	170/181	144/172	245/253/256	240/248/252	250/255	239/239	205/205	131/131	126/140	186/186	172/173/184	[illegible]4/218/230/2[illegible]	261/263	174/178	107/109	168/172
152/152	181/181	125/125	113/117	144/156	134/134	168/177	83/89	111/111	211/211	316/316	249/249	175/175	259/259	210/210	208/208	227/227	256/268	170/176	147/147	245/245	252/259	250/250	245/247	201/201	131/167	125/125	186/186	187/187	222/230	261/261	174/174	109/109	170/172
152/152	181/181	125/125	117/131	144/147	134/134	168/180	83/144	111/111	211/221	316/316	249/249	175/175	259/259	210/240	208/208	221/227	256/256	170/176	142/147	245/245	252/262	250/257	239/245	201/205	153/167	125/125	186/186	172/184	222/230	261/261	174/178	109/109	172/174
144/152	181/214	125/125	113/117	147/161	132/162	168/177	83/89	111/111	211/221	303/316	238/249	175/175	254/259	210/243	208/208	227/227	256/280	170/181	147/172	245/245	243/259	250/255	239/249	205/205	131/160	139/139	186/186	173/187	214/222	261/263	174/174	107/109	170/172
152/152	181/181	125/125	117/117	144/156	132/134	168/168	83/83	111/111	211/227	316/316	249/249	175/179	259/259	210/210	208/208	221/221	274/286	170/170	144/144	245/245	259/259	255/255	249/249	201/201	160/167	125/139	192/192	184/184	234/234	261/261	178/178	109/109	170/172
152/152	181/181	125/125	117/131	144/156	132/134	168/180	83/89	111/111	211/211	316/316	249/249	175/175	259/259	210/210	208/208	227/227	256/268	170/176	142/147	245/245	252/262	250/257	247/247	201/201	153/167	125/139	186/186	187/187	205/222	261/261	174/174	109/109	170/174
152/152	181/184	125/125	113/117	144/161	132/137	168/168	89/104	111/111	211/227	316/316	239/249	175/175	259/259	210/243	208/208	227/227	268/274	170/176	145/147	245/247	252/259	250/255	239/249	205/205	160/167	125/139	186/186	184/187	222/230	261/263	174/178	107/109	170/174
152/152	181/181	125/125	117/126	156/161	132/137	168/168	83/104	111/111	211/227	304/316	249/249	170/175	259/259	210/243	208/213	221/227	256/268	170/176	142/144	245/247	252/259	255/255	239/247	201/205	160/167	125/139	186/192	184/187	205/230	261/263	174/178	109/109	170/172
152/152	181/184	125/125	117/117	144/161	137/155	168/177	89/104	111/111	221/221	316/316	249/249	175/175	259/259	240/243	208/208	221/227	256/268	176/181	142/144	245/247	252/252	250/255	249/249	201/205	131/167	125/125	186/186	184/184	230/230	263/263	178/178	107/109	170/172
152/152	181/184	125/125	113/126	161/161	132/137	168/168	83/104	111/111	211/227	304/316	239/239	170/175	254/259	210/210	208/208	227/227	256/268	170/176	145/145	246/247	252/259	255/255	239/249	205/205	160/167	125/139	192/192	187/187	205/226	261/263	174/174	107/109	170/174
152/152	181/184	125/125	117/117	156/161	134/155	177/180	83/89	111/111	211/221	316/316	239/249	175/175	254/259	210/240	208/208	227/227	256/268	170/181	134/147	245/253	248/252	250/255	239/249	201/205	131/153	125/125	186/186	184/187	205/230	261/263	174/178	107/109	170/172
152/152	181/181	125/125	117/126	156/161	134/155	177/180	104/104	111/111	211/227	303/316	238/249	170/175	254/259	210/243	208/213	221/227	256/268	170/176	134/142	245/245	252/259	250/255	239/247	201/205	131/153	125/125	186/186	172/184	205/222	261/263	174/178	109/109	170/172
152/152	181/181	125/125	117/126	156/161	132/137	168/168	83/104	111/111	211/227	304/316	249/249	170/175	259/259	210/243	208/213	221/227	256/268	170/176	142/144	245/247	252/259	255/255	239/247	201/205	160/167	125/139	186/192	184/187	205/230	261/263	174/178	109/109	170/172
149/152	181/214	125/125	117/117	144/161	137/162	168/177	83/92	106/111	211/227	303/316	238/239	175/175	259/259	211/243	208/213	221/227	256/280	170/176	146/146	245/250	248/252	250/255	239/253	201/205	131/160	125/125	186/186	172/184	226/230	263/263	174/178	107/109	170/172
143/152	181/214	125/125	110/117	144/144	132/162	168/180	83/83	106/111	211/227	303/316	238/239	175/179	254/254	243/243	195/208	221/221	274/280	170/176	144/145	250/250	240/248	255/255	225/239	205/205	131/160	139/139	192/192	173/187	226/234	261/261	174/174	107/109	168/168
143/152	181/214	125/125	117/117	144/144	132/137	168/168	83/104	111/111	211/227	316/316	239/239	175/175	259/259	210/243	208/208	221/227	256/280	170/176	145/145	245/247	252/252	250/255	239/249	205/205	160/167	125/139	186/192	184/187	226/230	261/263	174/178	107/109	170/170
152/152	181/181	125/125	113/117	147/161	134/137	168/168	83/104	111/111	211/221	316/316	239/249	175/175	259/259	210/243	208/208	221/227	256/286	169/170	142/145	245/247	252/259	250/255	239/245	201/205	131/160	125/125	186/192	184/184	230/234	261/263	178/178	107/109	170/170
149/152	214/214	125/125	117/123	144/144	132/162	168/177	92/92	106/106	221/227	303/316	238/238	175/175	254/254	240/243	208/208	221/221	268/280	176/176	144/146	246/250	248/252	255/255	239/251	201/205	131/160	139/139	192/192	172/184	214/226	261/263	174/174	107/107	172/172
152/152	181/181	125/125	113/117	144/161	132/155	168/177	104/104	111/111	211/227	316/316	239/249	175/175	259/259	210/243	208/208	221/227	256/256	169/176	145/147	246/247	252/259	255/255	245/249	201/205	131/160	125/125	186/192	184/187	226/230	261/263	174/178	107/109	170/174
152/152	181/181	125/125	117/117	144/161	137/155	168/177	104/104	111/111	221/227	303/316	239/249	175/175	259/259	210/243	208/208	221/227	256/274	170/170	142/145	245/247	252/252	250/255	249/249	205/205	131/167	125/125	186/186	172/184	222/230	263/263	174/178	109/109	170/172
152/152	181/181	125/125	113/117	147/161	132/137	168/168	104/104	111/111	211/227	316/316	239/249	175/175	259/259	210/243	208/208	227/227	256/256	169/170	145/147	246/247	252/259	255/255	245/249	201/205	160/167	125/125	186/192	184/184	230/230	261/263	178/178	107/109	172/174
152/152	181/181	125/125	113/113	147/153	132/134	168/168	83/83	111/111	211/211	316/316	249/249	175/175	259/259	210/210	208/208	227/227	256/286	169/169	142/142	245/246	259/259	250/253	239/245	201/205	160/160	125/139	186/192	184/184	230/234	261/261	178/178	107/107	170/170
143/152	181/181	125/125	117/117	144/161	132/162	168/177	104/104	111/111	221/227	303/316	238/239	175/175	254/259	210/249	208/208	221/221	280/280	170/181	144/156	245/250	240/252	250/255	239/239	205/205	131/160	139/139	192/192	187/187	218/226	261/261	174/174	107/109	170/172
152/152	181/181	125/125	117/126	161/161	132/137	168/168	104/104	111/111	211/227	304/316	239/239	170/175	254/259	210/243	208/208	227/227	256/268	170/170	134/145	245/247	252/259	250/255	239/247	201/205	160/167	125/139	186/192	184/187	205/230	261/263	174/178	109/109	170/170
152/152	181/181	125/125	117/117	156/161	134/137	168/177	83/104	111/111	211/221	316/316	239/249	175/175	259/259	210/243	208/208	221/227	256/256	170/176	145/147	245/247	252/252	250/250	239/245	201/205	131/167	125/125	186/186	184/187	230/230	261/263	174/178	109/109	170/172
152/152	181/184	125/125	113/117	144/161	132/155	168/177	83/104	111/111	211/227	303/316	239/249	175/175	254/259	210/210	208/208	227/227	256/256	169/176	144/145	246/247	252/259	255/255	245/249	201/205	131/160	125/125	192/192	184/187	226/230	261/263	174/178	107/107	170/174
138/143	181/181	125/125	91/117	161/161	132/151	177/177	nd.	106/111	210/227	316/316	238/244	175/175	249/254	237/243	208/213	221/227	274/280	170/170	131/144	244/250	248/258	255/288	233/239	201/205	131/131	139/139	192/192	181/181	218/218	261/263	174/176	107/109	168/168
152/152	181/208	125/125	117/117	161/161	134/137	168/168	83/104	111/111	211/227	316/316	239/249	175/179	259/259	210/243	208/208	227/227	256/256	170/170	142/145	245/246	252/252	250/250	247/249	201/205	167/167	125/125	186/186	184/184	230/230	261/263	178/178	107/109	170/172
152/152	181/181	125/125	117/117	144/161	137/155	168/177	104/104	111/111	227/227	303/316	239/249	175/175	259/259	210/243	208/208	221/227	256/274	170/176	142/145	245/247	252/252	250/255	249/249	205/205	131/167	125/125	186/186	172/187	222/226	263/263	174/174	109/109	170/170
152/152	181/181	125/125	117/117	161/161	137/155	168/177	104/104	111/111	221/227	303/316	239/249	175/175	259/259	210/243	208/208	227/227	256/274	170/170	142/145	245/247	252/252	250/255	239/249	205/205	131/167	125/125	186/186	184/187	230/230	263/263	174/178	109/109	170/172
152/152	181/181	125/125	117/117	144/156	155/162	177/177	83/104	111/111	211/211	303/316	249/249	175/175	254/259	210/210	208/208	227/227	256/268	169/176	144/144	245/246	252/259	250/255	245/249	201/205	131/160	126/140	186/186	184/187	226/230	263/263	174/178	107/107	170/170
144/152	181/208	125/125	117/117	144/161	132/134	168/177	83/144	111/111	211/227	316/316	239/249	175/175	259/259	210/243	208/208	227/227	256/256	170/176	142/145	245/247	252/252	250/255	247/249	201/205	131/167	125/139	186/186	184/184	230/230	261/263	178/178	107/107	170/170
152/152	181/181	125/125	113/117	161/161	132/132	168/168	83/83	111/111	211/211	316/316	249/249	175/175	259/259	210/210	208/213	221/227	256/256	170/170	134/142	245/246	252/259	250/253	239/245	201/205	160/160	139/139	186/186	184/187	230/230	261/261	174/178	107/109	170/170
152/152	181/181	125/125	113/117	153/161	137/137	168/168	89/89	111/111	221/227	316/316	239/249	175/175	254/259	210/243	208/208	227/227	256/274	169/169	144/147	245/247	252/259	255/255	245/249	201/205	167/167	125/125	186/186	184/184	230/230	263/263	178/178	107/107	170/174
152/152	181/184	125/125	117/131	144/161	134/137	168/168	104/144	111/111	211/221	316/316	239/249	175/175	259/259	210/210	208/208	221/227	256/274	170/176	145/147	245/247	252/262	250/257	247/249	201/205	167/167	125/125	186/186	172/184	222/230	261/263	174/178	107/109	170/174
152/152	181/181	125/125	117/126	161/161	134/137	168/177	83/83	111/111	221/227	316/316	239/239	170/175	254/259	210/210	208/208	227/227	268/274	170/170	145/145	246/247	252/259	250/255	239/249	205/205	131/167	125/125	186/192	187/187	226/230	261/263	174/174	109/109	170/172
141/144	181/214	125/125	117/123	147/147	132/164	168/177	83/83	111/111	211/227	303/316	238/239	175/175	254/259	210/243	208/213	221/227	268/274	170/181	144/147	246/250	252/252	255/255	239/251	201/201	167/167	125/125	186/186	173/187	218/226	261/263	174/174	107/107	168/170
152/152	181/181	125/125	117/117	144/161	134/155	177/177	104/104	111/111	211/227	303/316	249/249	175/175	254/259	210/243	208/208	227/227	256/274	170/176	144/147	245/246	252/259	250/255	245/249	201/205	131/160	125/125	186/186	184/184	230/230	261/263	178/178	107/109	170/172
152/152	181/214	125/125	117/117	144/161	134/137	168/168	83/144	111/111	211/227	316/316	239/249	175/175	259/259	210/210	208/208	221/227	256/262	170/176	145/147	245/247	252/252	250/255	239/247	201/205	167/167	125/125	186/186	184/184	230/230	261/263	178/178	107/109	170/170
152/152	181/181	125/125	117/123	144/161	137/164	177/177	104/104	111/111	211/221	303/316	238/243	175/175	254/254	210/210	208/213	227/227	262/268	170/170	144/145	247/250	248/248	255/255	239/249	205/205	131/131	125/125	186/186	184/184	218/230	261/263	174/178	109/109	168/170
143/152	181/181	125/125	117/117	144/161	137/155	168/177	89/104	111/111	221/221	316/316	239/249	175/175	254/259	210/243	208/208	227/227	256/268	170/170	134/142	245/245	248/252	250/255	239/249	205/205	131/167	125/125	186/186	184/184	230/230	263/263	178/178	109/109	170/172
144/152	181/181	125/125	117/117	161/161	155/155	177/177	104/104	111/111	211/227	303/303	239/249	175/175	259/259	210/243	208/208	227/227	256/256	170/176	142/145	245/247	252/262	255/257	247/249	201/205	131/131	140/140	192/192	184/184	230/230	263/263	178/178	109/109	170/172
152/152	181/181	125/125	113/117	147/161	132/137	168/168	83/104	111/111	211/221	316/316	249/249	175/175	259/259	210/243	208/208	227/227	256/256	169/170	142/142	245/246	252/259	250/253	239/249	205/205	160/167	125/139	186/186	184/184	230/230	261/263	178/178	107/109	170/172
152/152	181/181	125/125	113/117	147/161	134/137	168/168	83/104	111/111	211/227	316/316	249/249	175/175	259/259	210/243	208/208	227/227	256/256	169/176	142/142	245/247	252/259	250/255	239/249	205/205	160/167	125/125	186/192	184/184	230/234	261/263	178/178	107/109	170/172
152/152	181/181	125/125	117/117	161/161	132/137	168/168	83/104	111/111	227/227	316/316	239/249	175/175	259/259	210/243	208/208	227/227	256/256	169/170	142/142	245/247	252/252	250/255	239/239	201/205	160/167	125/139	186/186	184/184	230/234	261/263	178/178	107/109	170/170
144/152	181/181	125/125	117/123	144/147	132/155	168/177	83/104	111/111	211/211	303/316	234/239	179/179	249/259	210/243	208/213	227/227	256/268	170/176	142/145	246/250	252/252	250/255	239/249	205/205	131/167	125/125	186/186	187/187	226/226	261/263	174/174	109/109	168/170
152/152	181/181	125/125	117/117	156/161	134/137	168/177	83/104	111/111	221/221	316/316	239/249	175/175	259/259	210/243	208/208	227/227	256/256	170/176	145/147	245/247	252/252	250/250	239/245	201/205	131/167	125/125	186/186	184/187	230/230	261/263	174/178	109/109	170/172
152/152	181/181	125/125	117/117	144/156	134/137	168/177	83/83	111/111	211/227	316/316	239/249	175/175	259/259	210/210	208/208	227/227	256/274	169/170	144/145	246/247	252/259	250/255	249/249	205/205	131/167	125/125	186/192	184/184	230/234	261/263	178/178	107/109	170/170
144/149	214/214	125/125	117/123	144/144	162/164	177/177	92/92	111/111	221/221	303/303	238/243	175/175	254/254	243/249	208/208	221/221	280/280	175/181	144/156	250/250	240/248	255/255	239/241	205/205	131/131	126/126	nd.	180/180	214/218	261/261	174/174	107/107	168/172
152/152	181/181	125/125	117/117	153/161	134/155	168/168	83/104	111/111	221/227	303/316	239/239	175/175	254/259	210/243	208/208	227/227	256/274	170/170	134/145	245/247	252/252	250/255	245/249	201/205	167/167	125/125	192/192	184/187	226/234	261/263	174/178	109/109	170/172
152/152	181/184	125/125	113/117	144/161	134/137	168/168	83/104	111/111	211/227	316/316	239/249	175/175	259/259	210/210	208/208	227/227	256/274	176/176	145/147	245/247	252/259	250/255	247/249	201/205	167/167	125/125	186/186	184/187	222/226	261/263	174/178	107/109	170/174
152/152	181/181	125/125	117/117	147/161	132/155	168/177	83/104	111/111	211/227	303/316	239/249	175/179	259/259	210/210	208/208	221/227	256/274	169/170	144/145	247/247	252/259	253/255	239/245	201/205	131/153	125/125	192/192	184/187	230/234	263/263	174/178	107/109	172/174
152/152	181/181	125/125	113/126	147/156	132/134	168/180	104/104	111/111	211/211	316/316	239/249	170/175	254/259	210/211	208/213	221/227	268/274	170/170	134/147	245/246	259/259	255/255	245/247	201/201	153/160	125/125	186/192	184/184	230/234	261/261	178/178	107/109	170/174
152/152	181/208	125/125	113/117	144/161	132/137	168/168	83/83	111/111	211/211	316/316	249/249	175/179	259/259	210/243	208/208	227/227	256/256	169/170	142/144	245/246	252/259	250/253	245/247	201/201	160/167	125/125	186/186	184/184	230/230	263/263	178/178	107/107	172/174
152/152	181/184	125/125	117/126	144/161	132/155	168/168	83/104	111/111	211/211	303/316	239/249	170/175	254/254	210/243	208/208	227/227	256/256	170/176	134/144	246/247	252/252	250/255	239/245	201/205	160/160	139/139	186/186	184/187	226/230	261/261	174/178	107/109	170/174
152/152	181/181	125/125	117/117	161/161	134/155	168/180	83/104	111/111	211/221	303/321	239/249	175/175	259/259	210/210	208/208	227/227	256/268	170/170	134/145	245/247	252/252	250/255	239/247	201/205	153/167	125/125	186/186	187/187	205/230	261/263	174/174	109/109	170/170
152/152	181/184	125/125	117/117	144/161	132/155	168/177	104/104	111/111	221/227	nd.	239/249	175/179	259/259	211/243	208/208	221/227	274/274	170/176	144/145	245/247	252/259	250/253	245/249	201/205	131/160	139/139	192/192	184/187	230/230	261/263	174/178	107/109	172/174
152/152	181/181	125/125	113/117	147/153	137/137	168/168	83/83	111/111	211/211	316/316	239/239	170/175	254/259	210/210	208/208	221/221	256/256	169/169	134/145	245/247	252/259	255/255	247/249	201/205	167/167	125/125	186/186	184/184	230/234	263/263	178/178	107/109	170/172
152/152	181/208	125/125	117/117	161/161	137/137	168/168	104/104	111/111	211/227	316/316	249/249	175/175	259/259	210/243	208/208	227/227	256/256	170/176	142/147	245/247	252/252	250/255	239/249	205/205	167/167	125/125	186/186	184/184	230/230	263/263	178/178	107/109	170/170
152/152	181/181	125/125	117/117	144/144	132/137	168/168	83/104	111/111	211/227	316/316	239/249	175/179	259/259	210/210	208/208	221/227	274/274	169/176	145/147	246/247	252/259	255/255	245/249	201/205	160/167	125/125	186/192	172/184	222/234	261/263	174/178	109/109	170/174
152/152	181/181	125/125	113/117	147/161	132/155	168/177	83/104	111/111	211/221	303/316	239/249	175/175	254/259	210/243	208/208	221/227	274/274	169/170	144/145	246/247	252/259	255/255	245/249	201/205	131/160	125/125	192/192	184/187	230/234	261/263	174/178	109/109	170/174
152/152	181/181	125/125	113/117	147/153	132/137	168/168	83/83	111/111	211/211	316/316	239/239	175/175	254/259	210/210	208/208	221/227	256/256	169/170	134/145	245/247	252/252	250/255	247/249	201/205	167/167	125/125	186/186	184/184	234/234	263/263	178/178	107/109	170/174
152/152	181/181	125/125	117/117	156/161	134/155	177/180	83/104	111/111	211/221	303/316	239/249	175/175	259/259	210/243	208/208	227/227	256/274	170/176	145/147	245/247	252/252	250/255	247/249	201/205	131/153	125/125	186/186	187/187	205/230	261/263	174/174	109/109	170/174
152/152	181/181	125/125	113/117	144/161	132/137	168/177	104/104	111/111	211/227	316/316	239/249	175/179	259/259	210/210	208/208	221/227	274/274	169/176	145/147	246/247	252/259	250/255	245/249	201/205	131/160	125/125	186/192	184/184	230/234	261/263	178/178	107/109	170/174
152/152	181/208	125/125	117/117	156/161	137/137	168/168	104/144	111/111	211/227	316/316	238/249	175/175	259/259	243/243	208/208	227/227	256/262	170/176	142/144	245/247	252/252	250/255	247/249	201/205	167/167	125/125	186/186	184/184	230/230	263/263	178/178	107/109	170/170

152/152	181/184	125/125	126/131	144/147	132/155	168/177	83/89	111/111	211/221	316/316	239/249	170/175	254/259	240/243	208/208	227/227	256/256	170/181	134/147	247/247	252/252	250/255	245/249	201/201	131/160	139/139	186/186	184/187	205/230	261/263	174/178	107/109	170/170
143/152	181/214	125/125	117/131	144/147	132/134	168/168	83/83	111/111	211/227	316/316	239/249	175/175	259/259	210/243	208/208	227/227	256/256	170/176	145/147	245/247	252/262	255/257	247/249	201/205	160/167	125/139	186/192	172/187	226/230	261/261	174/174	107/109	170/172
152/152	181/181	125/125	117/117	147/161	134/137	168/180	104/104	111/111	211/227	316/316	249/249	175/175	259/259	210/243	208/208	221/227	256/256	170/176	142/147	245/247	252/252	250/255	239/249	205/205	153/167	125/125	186/186	184/184	230/230	261/263	178/178	109/109	170/172
144/152	181/181	125/125	113/117	147/147	134/155	168/177	83/83	111/111	211/211	303/316	249/249	175/175	254/259	210/210	208/208	227/227	256/256	169/170	142/144	245/247	252/262	255/257	245/247	201/201	131/167	125/125	186/186	172/187	222/226	261/263	174/174	107/109	170/172
152/152	181/181	125/125	117/117	156/161	134/162	177/180	83/104	111/111	211/221	316/316	239/249	175/175	254/259	210/210	208/208	227/227	256/256	169/170	144/147	246/247	252/259	255/255	245/249	201/205	131/153	125/125	186/186	184/187	205/230	261/263	174/178	107/109	170/174
152/152	181/181	125/125	117/126	144/156	134/155	168/168	83/104	111/111	211/221	nd.	239/239	170/175	254/259	210/243	208/208	227/227	256/256	170/170	134/145	245/247	252/252	250/250	239/239	205/205	160/167	125/125	186/186	187/187	205/226	261/261	174/174	109/109	170/174
152/152	181/181	125/125	113/117	147/161	134/155	168/177	104/144	111/111	211/221	316/316	239/249	175/175	259/259	210/243	208/208	221/227	256/256	169/170	145/147	245/247	252/262	255/257	247/249	201/205	131/167	125/125	186/192	184/184	230/230	261/263	178/178	107/107	172/174
152/152	181/181	125/125	117/117	144/161	132/137	168/168	83/104	111/111	211/221	316/316	249/249	175/179	259/259	210/243	208/208	227/227	256/256	169/176	142/147	247/247	252/259	253/255	239/245	201/205	160/167	125/125	186/186	172/184	222/234	261/263	174/178	107/109	172/174
152/152	181/181	125/125	113/117	147/161	132/155	168/177	83/104	111/111	211/221	303/316	249/249	175/175	259/259	210/243	208/208	227/227	256/256	169/176	142/147	245/246	252/259	250/255	245/249	201/205	131/153	125/125	186/186	172/184	222/230	263/263	174/178	107/109	170/174
144/152	181/181	125/125	117/131	147/161	134/155	168/177	83/104	111/111	211/227	303/316	249/249	175/175	259/259	210/210	208/208	227/227	256/256	170/170	142/147	245/245	252/262	250/257	239/247	201/205	131/167	125/125	186/186	184/187	230/230	261/263	174/178	109/109	170/172
152/152	181/181	125/125	117/117	161/161	137/137	168/168	83/104	111/111	211/227	316/316	239/249	175/175	254/259	210/243	208/208	227/227	268/274	170/170	134/142	245/247	252/252	250/255	247/249	201/205	167/167	125/125	186/186	184/184	230/230	263/263	178/178	109/109	170/172
152/152	181/181	125/125	117/117	156/161	134/155	177/180	83/89	111/111	211/227	303/316	239/249	175/179	259/259	210/210	208/208	221/227	256/268	170/170	142/145	245/246	252/259	250/250	247/249	201/205	131/160	125/125	186/186	187/187	222/230	261/263	174/174	109/109	170/172
152/152	181/181	125/125	117/117	144/161	134/155	168/180	89/104	111/111	211/227	303/316	249/249	175/175	259/259	210/243	208/208	221/227	256/268	170/176	142/147	245/247	252/252	250/255	247/249	201/205	153/167	125/125	186/186	172/187	205/222	261/263	174/174	109/109	172/174
144/152	181/181	125/125	117/117	156/161	134/162	168/177	104/144	111/111	211/211	303/316	239/249	175/175	259/259	243/243	208/208	227/227	256/268	170/176	144/145	245/247	252/252	250/255	247/249	201/201	131/167	125/125	186/186	180/184	218/230	261/263	174/178	107/109	170/170
144/152	181/184	125/125	113/117	156/161	137/162	168/177	104/104	111/111	211/227	316/316	239/249	175/175	259/259	210/243	208/208	221/227	256/256	170/176	144/145	245/247	252/252	250/255	245/249	201/201	131/167	125/125	186/186	184/184	226/230	263/263	178/178	107/109	170/170
152/152	181/208	125/125	117/117	144/144	134/137	168/168	104/104	111/111	211/221	303/316	239/249	175/175	259/259	210/243	208/208	227/227	256/256	176/176	145/147	245/247	252/252	250/255	247/249	201/205	131/167	125/125	186/186	184/187	226/230	261/263	174/178	107/109	170/170
152/152	181/184	125/125	117/131	147/161	132/155	168/177	104/144	111/111	211/227	303/316	239/249	175/175	259/259	210/243	208/208	227/227	256/274	170/176	145/147	245/247	252/262	255/257	247/249	201/205	131/160	139/139	186/186	187/187	222/230	261/263	174/174	107/109	170/174
152/152	181/181	125/125	113/117	144/147	132/137	168/168	83/104	111/111	211/211	316/316	239/249	175/175	259/259	nd.	208/208	227/227	256/256	170/170	142/145	245/247	252/262	255/257	245/249	201/205	167/167	125/139	186/186	184/187	222/230	261/263	174/178	109/109	170/174
144/152	181/214	125/125	123/131	143/161	132/155	177/177	104/104	111/111	211/211	303/316	238/249	175/175	254/259	210/243	208/213	221/221	256/268	170/181	144/147	245/250	252/262	255/257	247/251	201/201	131/131	125/125	186/186	172/187	222/226	261/263	174/174	107/109	168/170
144/144	181/208	125/125	123/131	143/161	132/134	168/168	83/104	111/111	211/221	303/316	238/249	175/175	254/259	210/243	208/208	221/227	256/268	176/176	144/147	245/250	252/252	250/255	239/247	201/205	167/167	125/125	186/186	184/187	222/226	261/261	174/178	107/109	168/170
144/144	181/214	125/125	117/123	147/161	132/155	168/177	83/144	111/111	211/211	303/316	238/249	175/175	254/259	210/210	208/213	227/227	256/268	170/181	142/144	245/250	252/252	250/255	239/247	201/205	131/167	125/125	186/186	172/187	222/226	261/263	174/174	107/109	168/172
152/152	181/214	125/125	117/117	156/161	132/134	168/180	83/104	111/111	211/221	316/316	239/249	175/175	259/259	210/243	208/208	227/227	268/268	170/176	144/145	245/250	248/252	255/255	239/247	201/205	153/160	125/139	186/192	187/187	205/226	261/261	174/174	107/109	168/170
143/152	181/181	125/125	117/131	144/144	132/137	168/168	83/89	111/111	211/211	316/316	249/249	175/175	259/259	210/240	208/208	227/227	256/268	170/170	147/147	245/245	252/259	250/250	239/245	201/205	160/167	125/139	186/186	184/187	222/230	261/263	174/178	109/109	170/172
143/152	181/208	125/125	117/131	144/161	132/134	168/168	104/144	111/111	211/227	316/316	239/249	175/175	259/259	210/243	208/208	227/227	256/262	170/176	142/145	245/245	252/262	250/257	247/249	201/205	160/167	125/139	186/192	184/187	226/230	261/261	174/178	107/109	170/172
152/152	181/214	125/125	117/117	144/144	132/137	168/168	104/104	111/111	221/227	316/316	238/239	175/175	254/259	210/243	208/208	221/227	256/268	170/176	144/145	247/250	248/252	255/255	239/249	205/205	131/167	125/139	186/192	184/187	226/230	261/263	174/178	107/109	168/170
143/152	181/181	125/125	117/117	147/156	137/137	168/168	104/104	111/111	211/221	316/316	238/239	175/175	254/254	210/243	208/208	227/227	262/268	170/176	134/144	245/247	252/252	250/250	239/239	205/205	167/167	125/125	186/186	184/184	230/230	263/263	178/178	107/109	170/170
143/152	181/214	125/125	117/117	144/161	132/132	168/177	83/83	111/111	211/221	316/316	238/239	175/175	254/259	210/243	208/208	221/227	256/280	170/170	144/145	245/250	248/252	250/255	239/239	205/205	131/160	139/139	192/192	187/187	226/226	261/261	174/174	107/109	168/170
143/152	181/181	125/125	117/117	161/161	134/164	168/180	83/104	106/111	211/227	316/316	239/239	175/175	254/259	210/210	208/208	227/227	268/268	170/170	134/145	245/245	252/259	250/255	239/247	201/205	153/160	125/125	186/192	187/187	205/226	261/261	174/174	109/109	170/170
143/149	187/214	125/125	117/117	144/161	162/164	177/177	nd.	111/111	221/227	303/303	238/243	175/175	254/254	243/249	208/208	221/227	268/280	170/181	144/150	250/250	240/248	255/255	239/239	205/205	131/131	126/126	192/192	187/187	218/226	261/261	174/174	107/107	170/172
143/152	181/214	125/125	117/131	144/161	132/155	168/177	144/144	106/106	211/227	303/316	239/249	175/175	259/259	210/243	195/208	221/227	256/268	170/176	145/147	245/245	252/262	250/257	239/239	205/205	131/160	139/139	192/192	184/184	218/230	261/263	174/178	107/109	170/172
152/152	181/181	125/125	113/117	153/161	132/155	168/177	83/104	111/111	211/227	303/316	239/249	175/175	259/259	210/243	208/208	227/227	256/256	169/176	142/145	246/247	252/259	253/255	239/249	201/205	131/160	139/139	186/186	172/184	222/234	261/263	174/178	107/109	170/172
144/152	181/208	125/125	117/117	161/161	137/155	168/177	83/144	111/111	211/227	303/316	239/249	175/175	259/259	210/210	208/208	227/227	256/256	170/176	142/145	245/247	252/252	250/255	247/249	201/205	131/167	125/125	186/186	172/184	226/230	263/263	174/178	107/109	170/170
152/152	181/181	125/125	113/117	153/161	132/137	168/168	83/104	111/111	211/221	316/316	249/249	175/175	259/259	210/243	208/208	227/227	256/286	169/170	142/142	245/246	252/259	250/253	239/239	205/205	160/167	125/139	186/186	184/184	230/230	261/263	178/178	107/109	170/170
152/152	181/181	125/125	113/117	153/161	132/137	168/177	83/104	111/111	211/227	316/316	239/249	175/175	259/259	210/243	208/208	227/227	256/256	169/170	142/145	246/247	252/259	253/255	239/245	201/205	131/160	125/139	186/186	184/184	230/234	261/263	178/178	107/109	170/170
152/152	181/181	125/125	117/117	144/161	137/155	168/177	83/83	111/111	227/227	303/316	239/239	175/175	259/259	210/210	208/208	227/227	256/274	170/170	145/145	245/247	252/252	250/255	239/249	205/205	131/167	125/125	186/186	184/184	230/230	263/263	178/178	109/109	170/170
152/152	181/181	125/125	117/117	144/161	134/137	168/177	104/144	111/111	211/227	316/316	239/239	175/175	259/259	243/243	208/208	227/227	256/256	170/170	145/145	245/247	252/252	250/255	239/249	205/205	131/167	125/125	186/186	184/184	230/230	263/263	178/178	107/109	170/172
152/152	181/181	125/125	117/126	161/161	134/137	168/177	104/144	111/111	211/221	316/316	239/249	170/175	254/259	210/211	208/208	221/227	256/268	170/170	134/142	245/245	252/259	250/255	247/249	201/201	131/167	125/125	186/186	184/184	230/230	263/263	178/178	107/109	170/172
152/152	181/214	125/125	113/117	144/147	134/137	168/168	83/83	111/111	211/227	316/316	239/249	175/175	259/259	210/210	208/208	227/227	256/256	169/176	142/145	246/247	252/259	253/255	247/249	201/205	167/167	125/125	186/186	184/184	230/230	261/263	178/178	107/107	170/170
144/152	181/208	125/125	117/126	144/161	134/134	177/180	83/144	111/111	211/227	304/316	249/249	170/175	259/259	243/243	208/208	221/227	256/268	170/176	142/142	245/247	253/259	255/255	247/249	201/205	131/153	125/125	186/186	184/187	230/230	261/263	174/178	107/109	170/170
144/152	181/187	125/125	117/117	144/161	137/162	168/177	104/104	111/111	221/227	303/316	239/243	175/175	254/259	243/249	208/209	221/227	256/280	170/181	144/145	247/256	248/252	255/272	239/249	205/205	131/167	125/125	186/186	172/184	218/230	263/263	174/178	107/109	172/172
152/152	181/181	125/125	117/117	161/161	132/134	168/177	83/104	111/111	211/227	316/316	239/249	175/175	259/259	210/210	208/208	221/227	256/256	170/170	142/145	246/247	252/259	250/253	245/249	201/205	131/160	125/139	186/186	184/187	230/230	261/261	174/178	109/109	170/172
152/152	181/181	125/125	117/126	156/161	134/137	168/180	83/83	111/111	211/221	316/316	249/249	170/175	254/259	210/210	208/213	221/227	256/268	170/176	144/147	245/245	252/259	250/255	239/247	201/205	153/167	125/125	186/186	184/187	230/230	261/263	174/178	109/109	170/172
138/144	181/187	125/125	117/117	144/161	137/162	168/177	104/104	111/111	221/227	303/316	238/239	175/175	254/259	237/249	208/209	221/227	256/280	176/181	145/156	247/259	248/248	255/272	239/239	nd.	131/167	125/125	186/186	180/184	214/230	263/263	174/178	107/109	168/168
144/152	181/187	125/125	117/117	144/161	137/162	168/177	92/92	111/111	221/227	303/316	239/243	175/175	254/259	237/249	208/209	221/221	274/280	170/181	144/145	247/259	240/252	250/255	239/239	205/205	131/167	125/125	186/186	180/184	214/230	263/263	174/178	107/109	168/172
152/152	181/181	125/125	117/117	161/161	132/137	168/168	83/104	111/111	211/227	316/316	239/249	175/175	259/259	210/210	208/208	221/227	256/274	170/176	134/145	246/247	252/252	250/250	245/245	201/201	160/167	125/139	186/186	184/187	230/230	261/263	174/178	109/109	170/172
144/152	181/181	125/125	117/117	nd.	134/137	168/177	83/83	111/111	211/227	316/316	239/249	175/175	259/259	211/243	208/208	221/227	256/256	170/170	142/145	245/247	252/252	250/255	247/249	201/205	131/167	125/125	186/186	184/187	205/230	263/263	174/178	107/109	170/170
138/152	181/181	125/125	117/131	161/161	137/151	168/168	104/144	111/111	221/221	316/316	249/249	175/175	259/259	237/243	208/208	221/227	256/256	170/170	142/142	245/245	252/262	250/257	247/249	201/205	139/167	125/125	186/186	181/184	222/230	263/265	178/178	107/109	170/172
144/152	181/214	125/125	117/117	161/161	132/137	168/168	104/144	111/111	211/227	316/316	239/249	175/175	259/259	210/243	208/208	221/227	256/256	176/181	134/145	245/247	252/259	250/255	239/251	201/205	160/167	125/139	186/186	184/184	230/230	261/263	178/178	107/109	168/172
152/152	181/181	125/125	117/117	144/156	134/137	168/180	104/104	111/111	211/221	316/316	249/249	175/175	259/259	210/243	208/208	227/227	256/274	170/170	147/147	245/247	252/252	250/250	239/249	205/205	153/167	125/125	186/186	184/187	222/230	261/263	174/178	109/109	170/172
143/152	181/181	125/125	117/123	161/161	137/162	168/177	91/104	111/111	221/227	303/316	238/239	175/175	254/259	210/210	195/208	221/227	262/274	170/181	145/150	247/259	248/252	255/255	239/249	205/205	131/167	125/125	186/186	180/187	218/226	261/263	174/174	107/109	170/170
152/152	nd.	125/125	117/117	161/161	137/137	168/168	83/83	111/111	211/227	316/316	239/239	175/175	254/259	210/243	208/208	221/227	268/274	170/170	134/145	245/247	252/252	250/255	247/249	201/205	153/167	125/125	186/186	184/184	230/230	263/263	178/178	109/109	170/172
152/152	181/184	125/125	117/126	144/161	134/134	177/180	104/144	111/111	211/227	304/316	239/239	170/175	254/259	210/210	208/208	227/227	256/268	170/176	145/145	245/247	252/259	250/255	245/247	201/201	131/153	125/125	186/186	184/187	205/230	261/261	174/178	107/109	170/170
152/152	181/181	125/125	117/126	144/161	134/137	168/180	83/104	111/111	211/221	303/304	239/239	170/175	254/259	210/243	208/208	221/227	256/256	170/170	134/142	245/247	252/259	255/255	247/249	201/205	153/167	125/125	186/186	172/187	205/222	261/263	174/174	109/109	170/170

62	63	64	65	66	67	68	69	70	71	72	73	74	75	76	77	78	79	80	81	82	83	84	85	86	87	88	89	90	91	92	93	94	95
*	*	*	*	*	*	*	*	*	*	*	*	*			*	*		*	*	*				*	*		*	**	*				
NSX121	NSX132	NSX137	NSX141	NSX145	NSX150	NSX153	NSX156	NSX160	NSX162	NSX165	NSX169	NSX170	NSX175	NSX186	NSX187	SRPE02	SRPE03	SRPE05	TSRA101	TSRA103	TSRA107	TSRA108	TSRA109	TSRA110	TSRA117	TSGR222	TSGR264	TSGR265	TSGR271	TSGR282	NSX16	NSX27	NSX28
0	0	1	2	2	1	0	0	0	2	0	0	0	0	0	0	0	0	0	1	0	2	1	0	1	4	1	0	0	0	3	0	0	1
212/223	154/161	99/99	280/298	128/128	163/172	141/141	207/219	162/168	293/293	278/300	162/182	171/177	187/211	208/209	216/216	142/142	121/126	175/186	169/183	174/188	184/198	185/188	176/176	152/160	160/164	197/213	113/137	163/177	202/208	250/250	111/111	315/321	209/232
218/223	161/161	99/105	280/280	128/129	172/172	144/144	207/216	168/168	260/293	278/278	162/162	174/183	187/202	208/213	216/216	142/142	121/126	175/186	169/183	174/188	184/198	185/188	176/179	145/161	164/179	197/209	113/167	165/177	202/210	244/244	73/111	322/322	213/213
212/223	154/154	99/99	280/298	128/138	163/172	141/141	207/216	162/168	293/296	278/300	162/171	177/183	187/202	208/218	216/216	130/142	121/126	175/186	169/183	174/188	184/198	185/185	176/179	145/161	161/179	197/209	113/113	180/186	202/208	250/250	111/111	321/322	209/232
218/218	161/161	99/99	280/280	128/138	172/172	141/144	207/207	162/168	260/296	278/278	162/162	174/177	199/199	213/222	216/216	142/142	121/121	175/175	169/169	174/178	184/184	185/188	176/176	145/145	164/167	209/209	113/113	177/177	208/210	244/244	73/111	319/321	209/209
208/208	161/161	99/99	298/301	128/128	172/172	141/141	216/219	162/168	293/296	282/300	171/171	171/183	187/208	208/233	214/216	142/142	126/126	186/186	183/183	188/188	198/198	182/185	179/179	152/161	169/169	164/197	113/131	180/186	202/202	250/251	111/111	315/315	232/232
208/218	154/154	99/99	280/298	128/138	163/172	141/141	216/216	162/162	293/293	285/295	150/162	177/183	187/199	211/218	216/216	130/142	121/121	175/175	169/169	174/185	196/198	185/185	179/179	145/153	157/163	209/209	84/113	163/183	202/210	251/251	111/111	315/319	227/232
212/218	154/161	99/105	280/298	128/128	163/172	138/141	207/216	162/168	293/293	278/300	162/171	183/183	187/208	208/209	216/216	130/142	126/126	186/186	165/183	184/188	184/198	185/185	179/179	152/161	161/170	164/197	113/137	163/186	202/202	250/250	111/111	315/322	232/232
210/220	154/161	99/105	280/298	128/128	163/172	138/147	207/219	156/168	293/293	278/282	162/165	171/183	187/202	208/233	214/216	142/142	121/126	175/177	183/183	174/188	184/192	182/185	179/179	152/161	169/170	197/209	113/134	177/180	202/202	250/251	111/111	315/322	232/232
210/210	161/161	99/99	280/298	128/138	172/172	141/141	204/216	168/168	293/293	285/297	162/182	183/183	208/211	208/208	216/216	130/142	121/126	175/186	165/183	184/192	174/198	185/188	179/179	145/175	160/163	164/197	113/137	163/186	202/202	250/251	111/111	315/318	230/232
210/210	161/161	99/99	280/298	128/138	172/172	141/141	204/216	168/168	293/293	285/297	162/182	183/183	208/211	208/208	216/216	130/142	121/126	175/186	165/183	184/192	174/198	185/188	179/179	145/175	160/163	164/197	113/137	163/186	202/202	250/251	111/111	315/318	230/232
210/210	161/161	99/99	280/298	128/138	172/172	141/141	204/216	168/168	293/293	285/297	162/182	183/183	208/211	208/208	216/216	130/142	121/126	175/186	165/183	184/192	174/198	185/188	179/179	145/175	160/163	164/197	113/137	163/186	202/202	250/251	111/111	315/318	230/232
210/210	161/161	99/99	280/298	128/138	172/172	141/141	204/216	168/168	293/293	285/297	162/182	183/183	208/211	208/208	216/216	130/142	121/126	175/186	165/183	184/192	174/198	185/188	179/179	145/175	160/163	164/197	113/137	163/186	202/202	250/251	111/111	315/318	230/232
208/212	154/154	99/99	298/298	128/128	163/163	141/141	216/216	162/168	293/293	286/286	179/179	171/183	208/223	208/208	216/216	130/142	126/126	186/186	165/183	184/192	198/198	185/188	179/179	161/161	160/160	164/197	134/134	180/180	202/202	250/251	111/111	316/316	232/232
208/210	154/161	99/99	298/298	128/138	163/172	141/141	204/204	168/168	293/293	285/297	182/182	183/183	187/208	208/208	216/216	142/142	126/126	186/186	183/183	188/204	174/198	185/188	179/179	152/161	160/160	164/197	113/134	172/180	202/202	250/251	111/111	316/318	230/232
208/218	154/161	99/99	280/298	128/128	163/172	141/144	207/219	156/162	293/293	278/286	162/182	183/183	187/199	210/218	216/216	130/142	121/126	175/186	165/165	174/184	184/192	185/185	176/179	145/161	160/164	164/209	113/128	165/180	202/208	256/256	111/111	317/322	230/232
208/220	161/161	99/99	298/298	128/128	163/172	141/141	204/216	168/168	293/293	300/300	179/182	174/177	208/241	208/208	216/216	142/142	126/126	186/186	165/183	177/184	198/198	173/185	176/179	152/161	160/160	164/197	113/130	180/186	202/202	250/252	111/111	315/316	230/232
208/220	161/161	99/99	298/298	128/128	163/172	141/141	204/216	168/168	293/293	300/300	179/182	174/177	208/241	208/208	216/216	142/142	126/126	186/186	165/183	177/184	198/198	173/185	176/179	152/161	160/160	164/197	113/130	180/186	202/202	250/252	111/111	315/316	230/232
218/218	154/161	99/99	280/280	128/128	172/172	144/144	207/207	162/162	293/296	278/278	162/162	177/183	199/199	218/218	216/216	141/142	121/121	175/175	169/169	174/174	184/184	188/188	176/176	161/161	170/173	213/213	113/113	177/180	208/208	244/244	111/111	319/322	209/213
212/220	154/154	99/99	298/298	128/138	163/172	141/147	219/219	156/168	293/293	300/300	165/171	177/183	187/208	208/210	216/216	130/142	126/126	186/186	183/183	188/192	192/198	182/185	179/179	152/160	160/162	197/197	128/137	163/165	202/202	251/255	111/111	315/317	230/232
212/218	154/161	99/105	298/298	128/128	163/163	141/141	207/219	156/162	293/293	275/292	162/165	171/177	187/208	210/218	216/216	142/142	97/126	180/186	183/183	188/211	192/192	185/195	176/179	152/161	163/163	164/197	113/128	165/183	202/202	251/256	111/111	315/317	230/232
208/218	161/161	99/99	280/301	128/129	172/172	141/144	207/216	162/168	293/293	278/282	171/171	183/183	187/211	208/218	216/216	142/142	121/126	175/186	169/183	174/188	198/198	185/185	179/179	152/161	161/169	197/213	113/113	186/186	202/202	250/250	111/111	315/321	209/232
208/208	161/161	99/99	298/298	128/128	172/172	141/141	216/216	162/168	293/293	278/300	171/182	174/183	223/238	208/208	216/216	142/142	126/126	186/186	165/183	184/192	198/198	173/185	176/176	152/161	160/169	197/197	134/137	163/172	202/202	250/250	111/111	315/316	232/232
210/220	154/154	99/105	298/298	138/138	163/163	141/141	216/219	156/168	293/293	278/300	162/165	171/177	187/205	208/210	216/216	142/142	97/126	180/186	183/183	177/188	192/198	185/195	179/185	152/161	169/170	164/197	113/128	165/186	202/202	251/256	111/111	317/321	211/230
210/223	161/161	99/99	280/280	128/128	172/172	141/144	204/207	162/162	293/293	278/300	162/182	174/183	187/220	218/218	216/216	142/142	121/126	175/186	169/183	174/188	174/184	188/188	176/176	145/161	160/173	197/213	113/113	177/180	202/210	250/250	111/111	315/321	209/232
210/223	161/161	99/99	280/280	128/128	172/172	141/144	204/207	162/162	293/293	278/300	162/182	174/183	187/220	218/218	216/216	142/142	121/126	175/186	169/183	174/188	174/184	188/188	176/176	145/161	160/173	197/213	113/113	177/180	202/210	250/250	111/111	315/321	209/232
210/223	161/161	99/99	280/280	128/128	172/172	141/144	204/207	162/162	293/293	278/300	162/182	174/183	187/220	218/218	216/216	142/142	121/126	175/186	169/183	174/188	174/184	188/188	176/176	145/161	160/173	197/213	113/113	177/180	202/210	250/250	111/111	315/321	209/232
220/220	154/168	99/105	280/298	128/128	163/163	141/141	219/219	156/156	293/293	290/300	153/165	171/180	187/187	210/225	216/216	111/142	97/126	180/180	nd.	185/200	184/192	179/182	176/176	nd.	174/174	164/164	113/128	163/165	202/202	250/256	111/111	317/321	230/230
212/218	161/161	99/105	280/298	128/128	172/172	138/144	207/207	162/168	293/296	278/300	162/171	174/183	187/202	209/213	216/216	142/142	121/126	175/186	169/183	174/188	184/184	185/188	176/179	145/161	161/179	197/213	113/113	177/186	202/210	250/250	111/111	315/319	209/232
218/218	154/168	99/99	280/280	128/128	163/172	138/141	216/219	156/168	293/293	300/316	153/171	180/183	175/187	208/216	216/216	130/142	126/126	175/186	174/183	185/188	192/198	179/185	176/179	145/161	161/174	197/197	113/128	163/186	202/202	250/250	111/111	315/315	232/232
218/218	161/168	99/105	280/298	128/128	163/163	141/141	207/219	156/168	293/293	278/290	153/171	171/183	175/208	208/216	216/216	142/142	126/126	175/186	165/174	184/185	184/192	185/188	176/179	161/161	161/174	164/164	113/137	163/163	202/202	250/250	111/111	315/321	227/232
208/210	154/154	99/99	298/298	128/128	163/172	141/147	204/216	168/168	293/293	300/300	182/182	183/183	208/211	208/208	216/216	130/142	126/126	186/186	165/183	177/184	174/198	185/185	179/179	152/161	160/160	164/197	113/134	172/186	202/202	251/252	111/111	315/316	230/232
218/218	154/161	99/99	280/280	128/138	172/172	141/141	207/207	162/168	293/293	278/278	162/162	174/177	202/202	218/222	216/216	142/142	121/121	175/175	169/169	174/174	184/184	185/185	176/176	145/161	173/179	209/209	113/113	177/177	210/210	244/244	73/111	319/319	209/213
208/223	161/161	99/99	280/298	128/138	163/172	141/141	207/207	162/168	293/293	278/285	162/171	183/183	181/208	208/218	216/216	142/142	121/126	175/186	169/169	174/174	184/184	185/185	176/179	145/161	160/173	197/209	113/167	165/177	202/210	244/244	111/111	315/319	209/232
212/218	154/161	99/99	280/298	128/128	163/172	138/141	207/219	162/168	293/296	278/300	162/171	171/183	187/208	208/218	216/216	130/142	126/126	186/186	165/183	184/188	184/198	185/188	176/176	145/160	161/179	164/197	113/137	163/177	202/202	250/250	73/111	315/322	209/232
218/223	161/161	99/105	280/280	128/128	163/172	141/144	207/216	162/168	293/293	278/300	162/171	174/183	187/202	208/218	216/216	142/142	121/126	175/186	169/183	174/188	184/198	185/188	176/179	145/161	161/164	197/209	113/137	163/177	202/210	250/250	111/111	315/319	209/232
218/220	154/161	105/105	280/298	128/128	163/172	138/141	207/219	156/162	293/293	278/300	171/171	171/183	202/205	209/210	216/216	142/142	97/121	175/180	165/183	177/184	184/192	182/185	176/185	152/161	161/162	164/164	113/128	165/180	202/202	255/255	73/111	315/319	213/213
218/220	154/161	105/105	280/298	128/128	163/172	138/141	207/219	156/162	293/293	278/300	171/171	171/183	202/205	209/210	216/216	142/142	97/121	175/180	165/183	177/184	184/192	182/185	176/185	152/161	161/162	164/164	113/128	165/180	202/202	255/255	73/111	315/319	213/213
218/220	154/161	105/105	280/298	128/128	163/172	138/141	207/219	156/162	293/293	278/300	171/171	171/183	202/205	209/210	216/216	142/142	97/121	175/180	165/183	177/184	184/192	182/185	176/185	152/161	161/162	164/164	113/128	165/180	202/202	255/255	73/111	315/319	213/213
218/220	154/161	105/105	280/298	128/128	163/172	138/141	207/219	156/162	293/293	278/300	171/171	171/183	202/205	209/210	216/216	142/142	97/121	175/180	165/183	177/184	184/192	182/185	176/185	152/161	161/162	164/164	113/128	165/180	202/202	255/255	73/111	315/319	213/213
218/220	154/161	105/105	280/298	128/128	163/172	138/141	207/219	156/162	293/293	278/300	171/171	171/183	202/205	209/210	216/216	142/142	97/121	175/180	165/183	177/184	184/192	182/185	176/185	152/161	161/162	164/164	113/128	165/180	202/202	255/255	73/111	315/319	213/213
208/208	153/154	99/99	280/298	128/138	163/163	141/141	216/222	156/162	293/293	285/301	162/165	183/183	175/208	211/217	216/216	142/142	126/126	175/186	187/187	185/193	160/198	170/185	176/176	145/153	163/163	197/197	84/124	163/163	202/202	nd.	111/111	315/316	209/232
218/226	154/168	99/99	280/280	128/138	163/172	138/141	207/219	156/162	293/293	278/290	153/162	180/183	187/202	209/216	216/216	142/142	121/126	175/180	174/174	174/185	192/192	179/185	176/179	145/161	174/179	209/209	113/128	163/180	202/208	250/250	73/111	319/321	209/227
212/220	161/161	99/99	280/298	128/128	172/172	141/141	204/207	162/168	293/293	300/300	162/182	177/183	211/241	208/218	216/216	142/142	121/126	175/186	169/183	174/177	184/198	185/185	176/179	145/161	160/164	197/213	113/130	177/180	202/208	250/250	111/111	315/315	232/232
212/220	161/161	99/99	280/298	128/128	172/172	141/141	204/207	162/168	293/293	300/300	162/182	177/183	211/241	208/218	216/216	142/142	121/126	175/186	169/183	174/177	184/198	185/185	176/179	145/161	160/164	197/213	113/130	177/180	202/208	250/250	111/111	315/315	232/232
212/226	161/168	99/105	298/298	128/128	163/172	138/141	216/219	156/168	293/293	278/316	153/162	180/183	175/208	208/216	216/216	142/142	126/126	175/186	165/174	184/185	184/198	179/185	176/179	161/161	170/174	164/164	113/128	163/186	202/202	250/250	111/111	315/315	232/232
218/218	154/168	99/99	280/280	128/128	163/172	141/141	207/219	162/162	293/293	278/316	162/174	180/183	187/220	218/225	216/216	130/142	121/126	175/180	165/174	184/185	184/184	179/188	176/176	145/161	161/170	197/197	113/128	163/177	202/210	244/244	111/111	315/322	232/232
218/218	154/168	99/99	280/280	128/128	163/163	141/141	216/219	156/162	293/293	290/300	162/174	180/183	187/187	209/216	216/216	130/142	126/126	175/186	174/183	185/188	192/198	179/185	176/179	145/152	161/170	197/197	113/128	163/186	202/202	250/250	111/111	315/321	227/232
212/218	154/154	99/105	280/298	128/128	163/172	138/141	216/222	156/162	293/293	285/300	165/171	171/183	187/208	209/210	216/216	130/142	97/126	180/186	165/183	184/184	192/198	185/198	176/179	152/161	161/161	164/164	113/128	165/186	202/202	256/256	111/111	316/322	230/232
212/223	161/161	99/105	280/280	128/128	172/172	138/141	207/216	162/162	293/293	278/300	162/171	177/183	187/211	209/218	216/216	142/142	121/126	175/186	169/183	174/188	184/198	185/185	176/179	145/152	161/164	197/213	113/113	177/186	202/208	244/244	111/111	315/321	209/232
218/218	154/161	99/105	280/298	128/128	163/172	138/141	207/219	156/162	293/293	300/300	165/171	183/183	181/187	209/210	216/216	130/142	97/126	180/186	169/183	174/188	184/192	185/188	176/179	152/176	161/161	197/209	113/128	165/186	202/202	250/250	73/111	315/321	209/232
218/218	154/161	99/99	280/280	128/129	163/172	141/144	207/207	162/168	293/293	278/278	162/162	171/174	202/208	213/218	216/216	130/142	121/126	175/186	165/169	174/184	184/184	188/188	176/176	145/160	173/179	164/209	113/137	163/180	202/210	250/250	73/111	315/322	232/232
212/212	154/161	99/105	298/298	128/128	163/163	141/141	207/222	156/162	293/293	278/300	162/171	171/183	187/208	209/210	216/216	130/142	126/126	186/186	165/165	184/184	184/192	185/198	176/179	152/152	161/170	164/197	113/128	165/186	202/202	250/250	111/111	322/322	232/232
218/218	161/168	99/99	280/298	128/129	163/172	138/141	207/219	156/162	293/293	300/300	162/165	174/180	187/211	210/218	216/216	142/142	121/126	175/180	169/183	174/200	184/184	179/188	176/176	145/161	169/170	164/213	113/128	165/180	202/202	256/256	111/111	317/322	230/232
210/210	154/161	99/105	280/298	128/128	163/172	141/147	204/207	162/168	293/293	282/285	182/182	171/171	187/216	208/208	216/216	142/142	126/126	186/186	165/183	184/192	184/198	188/192	164/179	145/152	160/161	197/197	113/119	180/186	202/202	250/252	111/111	315/316	230/231
218/218	154/161	99/99	280/280	128/138	172/172	138/138	207/207	162/168	293/293	278/300	162/171	174/183	187/202	208/218	216/216	130/142	121/126	175/186	169/183	174/188	184/184	185/188	176/179	145/161	161/173	197/213	113/137	163/180	202/210	250/250	73/111	315/319	213/232
212/223	154/161	99/99	280/298	128/128	163/172	141/141	207/219	162/168	293/293	278/300	162/182	171/177	187/211	208/209	216/216	142/142	121/126	175/186	169/183	174/188	184/198	185/188	176/176	152/160	160/164	197/213	113/137	163/177	202/208	250/250	111/111	315/321	209/232
208/212	154/154	99/99	298/298	128/128	163/172	141/147	216/219	156/168	293/293	300/300	171/182	171/183	187/208	208/233	214/216	130/142	126/126	186/186	165/183	184/188	198/198	185/188	179/179	160/161	160/161	164/197	137/137	163/180	202/202	250/251	111/111	315/315	232/232
208/212	154/154	99/99	298/298	128/128	163/172	141/147	216/219	156/168	293/293	300/300	171/182	171/183	187/208	208/233	214/216	130/142	126/126	186/186	165/183	184/188	198/198	185/188	179/179	160/161	160/161	164/197	137/137	163/180	202/202	250/251	111/111	315/315	232/232
208/212	154/154	99/99	298/298	128/128	163/172	141/147	216/219	156/168	293/293	300/300	171/182	171/183	187/208	208/233	214/216	130/142	126/126	186/186	165/183	184/188	198/198	185/188	179/179	160/161	160/161	164/197	137/137	163/180	202/202	250/251	111/111	315/315	232/232
208/212	154/154	99/99	298/298	128/128	163/172	141/147	216/219	156/168	293/293	300/300	171/182	171/183	187/208	208/233	214/216	130/142	126/126	186/186	165/183	184/188	198/198	185/188	179/179	160/161	160/161	164/197	137/137	163/180	202/202	250/251	111/111	315/315	232/232
208/212	154/154	99/99	298/298	128/128	163/172	141/147	216/219	156/168	293/293	300/300	171/182	171/183	187/208	208/233	214/216	130/142	126/126	186/186	165/183	184/188	198/198	185/188	179/179	160/161	160/161	164/197	137/137	163/180	202/202	250/251	111/111	315/315	232/232
208/212	154/154	99/99	298/298	128/128	163/172	141/147	216/219	156/168	293/293	300/300	171/182	171/183	187/208	208/233	214/216	130/142	126/126	186/186	165/183	184/188	198/198	185/188	179/179	160/161	160/161	164/197	137/137	163/180	202/202	250/251	111/111	315/315	232/232
208/212	154/154	99/99	298/298	128/128	163/172	141/147	216/219	156/168	293/293	300/300	171/182	171/183	187/208	208/233	214/216	130/142	126/126	186/186	165/183	184/188	198/198	185/188	179/179	160/161	160/161	164/197	137/137	163/180	202/202	250/251	111/111	315/315	232/232
208/212	154/154	99/99	298/298	128/128	163/172	141/147	216/219	156/168	293/293	300/300	171/182	171/183	187/208	208/233	214/216	130/142	126/126	186/186	165/183	184/188	198/198	185/188	179/179	160/161	160/161	164/197	137/137	163/180	202/202	250/251	111/111	315/315	232/232
208/212	154/154	99/99	298/298	128/128	163/172	141/147	216/219	156/168	293/293	300/300	171/182	171/183	187/208	208/233	214/216	130/142	126/126	186/186	165/183	184/188	198/198	185/188	179/179	160/161	160/161	164/197	137/137	163/180	202/202	250/251	111/111	315/315	232/232
208/212	154/154	99/99	298/298	128/128	163/172	141/147	216/219	156/168	293/293	300/300	171/182	171/183	187/208	208/233	214/216	130/142	126/126	186/186	165/183	184/188	198/198	185/188	179/179	160/161	160/161	164/197	137/137	163/180	202/202	250/251	111/111	315/315	232/232
208/212	154/154	99/99	298/298	128/128	163/172	141/147	216/219	156/168	293/293	300/300	171/182	171/183	187/208	208/233	214/216	130/142	126/126	186/186	165/183	184/188	198/198	185/188	179/179	160/161	160/161	164/197	137/137	163/180	202/202	250/251	111/111	315/315	232/232
208/212	154/154	99/99	298/298	128/128	163/172	141/147	216/219	156/168	293/293	300/300	171/182	171/183	187/208	208/233	214/216	130/142	126/126	186/186	165/183	184/188	198/198	185/188	179/179	160/161	160/161	164/197	137/137	163/180	202/202	250/251	111/111	315/315	232/232
208/212	154/154	99/99	298/298	128/128	163/172	141/147	216/219	156/168	293/293	300/300	171/182	171/183	187/208	208/233	214/216	130/142	126/126	186/186	165/183	184/188	198/198	185/188	179/179	160/161	160/161	164/197	137/137	163/180	202/202	250/251	111/111	315/315	232/232
208/212	154/154	99/99	298/298	128/128	163/172	141/147	216/219	156/168	293/293	300/300	171/182	171/183	187/208	208/233	214/216	130/142	126/126	186/186	165/183	184/188	198/198	185/188	179/179	160/161	160/161	164/197	137/137	163/180	202/202	250/251	111/111	315/315	232/232
208/212	154/154	99/99	298/298	128/128	163/172	141/147	216/219	156/168	293/293	300/300	171/182	171/183	187/208	208/233	214/216	130/142	126/126	186/186	165/183	184/188	198/198	185/188	179/179	160/161	160/161	164/197	137/137	163/180	202/202	250/251	111/111	315/315	232/232
208/212	154/154	99/99	298/298	128/128	163/172	141/147	216/219	156/168	293/293	300/300	171/182	171/183	187/208	208/233	214/216	130/142	126/126	186/186	165/183	184/188	198/198	185/188	179/179	160/161	160/161	164/197	137/137	163/180	202/202	250/251	111/111	315/315	232/232
218/218	154/168	99/99	280/280	128/128	163/172	138/141	216/219	156/168	293/293	300/316	153/171	180/183	175/187	208/216	216/216	130/142	126/126	175/186	174/183	185/188	192/198	179/185	176/179	145/161	161/174	197/197	113/128	163/186	202/202	250/250	111/111	315/315	232/232
212/223	154/161	99/99	280/298	128/129	163/172	141/141	207/216	162/168	260/293	278/300/303	162/171/182	171/174/183	181/187	208/222	216/216	142/142	121/126	175/186	169/183	174/188	184/198	185/188	176/179	145/152/161	160/173	197/213	113/137	163/180	202/210	250/250	73/111	315/321	209/232
208/210	154/161	99/99	280/298	128/128	163/163	141/141	216/216	162/168	293/293	285/286	171/182	171/171	187/208	208/220	216/216	130/142	126/126	177/186	165/183	184/188	198/198	185/192	179/179	152/160	161/169	164/197	131/137	180/180	202/202	250/251	111/111	315/315	232/232
210/220	154/154	99/99	298/298	128/138	163/172	141/147	204/219	156/168	293/293	286/300	162/165	171/177	187/187	208/210	216/216	130/142	126/126	186/186	183/183	188/192	174/192	182/185	179/179	152/161	162/170	164/197	113/128	165/186	202/202	251/255	111/111	317/321	211/230
210/220	154/154	99/99	298/298	128/138	163/172	141/147	204/219	156/168	293/293	286/300	162/165	171/177	187/187	208/210	216/216	130/142	126/126	186/186	183/183	188/192	174/192	182/185	179/179	152/161	162/170	164/197	113/128	165/186	202/202	251/255	111/111	317/321	211/230
208/218	154/161	99/99	280/298	128/128	172/172	138/141	207/216	162/168	293/293	278/278	162/179	171/183	187/187	208/222	216/216	130/142	121/126	175/186	169/169	174/192	184/198	185/188	176/179	145/161	161/163	164/164	134/134	180/183	202/208	251/251	111/111	315/322	212/212
220/220	154/168	99/99	298/298	128/128	163/172	141/141	216/219	156/162	293/293	290/300	153/171	171/171	187/187	216/221	216/216	130/142	126/126	180/186	169/183	177/196	184/198	182/188	176/176	152/161	162/174	164/164	113/131	163/163	202/202	250/256	111/111	315/317	230/230
212/218	154/161	99/99	280/298	128/128	172/172	141/147	207/216	162/168	293/293	285/285	162/182	171/177	184/223	208/213	216/216	142/142	121/126	175/186	169/183	174/192	184/198	188/188	179/179	145/161	160/164	197/209	113/137	163/186	202/208	250/250	111/111	316/322	209/230
212/218	154/161	99/105	280/298	128/128	163/172	138/141	207/216	162/168	293/293	278/300	162/171	183/183	187/208	208/209	216/216	130/142	126/126	186/186	165/183	184/188	184/198	185/185	179/179	152/161	161/170	164/197	113/137	163/186	202/202	250/250	111/111	315/322	232/232
208/212	154/161	99/105	298/298	128/138	163/163	141/141	216/219	156/162	293/293	275/285	156/171	171/183	187/199	210/218	216/216	142/142	97/126	180/186	183/183	177/188	192/198	185/195	179/182	145/152	160/160	164/197	113/128	165/183	202/202	251/256	111/111	317/322	209/231
212/218	154/161	99/105	280/298	128/128	163/172	138/141	207/216	162/168	293/293	278/300	162/171	183/183	187/208	208/209	216/216	130/142	126/126	186/186	165/183	184/188	184/198	185/185	179/179	152/161	161/170	164/197	113/137	163/186	202/202	250/250	111/111	315/322	232/232
212/218	154/161	99/105	280/298	128/128	163/172	138/141	207/216	162/168	293/293	278/300	162/171	183/183	187/208	208/209	216/216	130/142	126/126	186/186	165/183	184/188	184/198	185/185	179/179	152/161	161/170	164/197	113/137	163/186	202/202	250/250	111/111	315/322	232/232
202/208	154/154	99/99	280/298	129/138	163/172	141/141	216/216	162/162	293/293	285/295	150/162	177/183	187/208	211/218	216/216	130/142	126/126	175/186	183/183	185/188	196/198	182/185	179/179	153/161	157/163	197/197	84/167	163/165	202/202	251/251	111/111	315/319	227/232
202/220	154/154	99/99	280/298	127/128	163/172	141/141	216/216	156/168	293/293	285/300	150/182	177/183	181/208	208/211	216/216	130/142	126/126	175/186	165/165	184/192	196/198	182/185	176/176	145/161	157/160	164/164	84/134	163/172	202/202	250/250	111/111	316/322	213/230
202/220	154/154	99/99	280/298	127/128	163/163	141/153	216/216	162/168	293/293	292/295	150/179	171/177	181/263	208/211	216/216	130/142	126/126	175/186	183/183	188/193	174/196	179/182	179/179	161/176	157/160	197/197	84/151	163/183	202/202	251/251	111/111	315/320	228/228
218/223	161/161	99/99	280/280	128/138	172/172	141/144	207/207	162/162	293/293	278/278	162/162	177/183	199/211	218/222	216/216	142/142	121/121	175/175	169/169	174/174	184/184	185/185	176/176	145/145	163/173	209/213	113/113	180/180	208/210	244/244	73/111	319/322	209/213
202/212	154/154	99/99	280/280	127/127	154/163	141/153	216/216	156/168	293/293	295/295	150/168	177/192	181/187	211/216	216/216	142/142	121/121	175/175	221/221	170/185	196/196	182/188	173/173	145/168	157/169	nd.	84/122	163/183	202/202	249/249	111/111	318/319	228/228
202/210	154/161	99/105	280/280	127/128	163/163	141/141	216/216	156/162	293/293	285/295	150/182	171/177	181/196	209/211	216/216	142/142	126/126	175/186	183/183	192/192	196/198	182/185	179/179	153/161	157/160	197/197	84/137	163/163	202/202	250/250	111/111	316/320	230/230
212/212	161/168	99/105	280/298	128/128	163/163	141/141	207/219	162/162	293/293	290/300	162/174	171/183	175/208	209/225	216/216	111/142	126/126	175/186	165/174	184/185	184/192	185/188	176/179	152/161	161/170	164/164	113/128	163/186	202/202	250/250	111/111	315/321	227/232
210/220	154/161	99/99	280/298	128/128	163/172	141/141	204/207	168/168	293/293	285/300	179/182	171/174	187/187	208/208	216/216	142/142	126/126	186/186	165/183	184/192	174/184	188/192	164/176	152/161	160/161	197/197	113/119	180/186	202/202	252/252	111/111	315/315	231/232
208/218	154/161	99/99	280/298	128/138	163/172	141/141	207/216	162/168	293/293	275/285	162/171	177/183	199/208	208/218	216/216	130/142	121/126	175/186	169/183	174/188	198/198	185/185	179/179	145/161	160/163	197/209	113/167	165/183	202/210	251/251	111/111	315/322	209/232
218/218	161/168	99/105	280/298	128/128	163/163	141/141	207/219	156/168	293/293	278/290	153/171	171/183	175/208	208/216	216/216	142/142	126/126	175/186	165/174	184/185	184/192	185/188	176/179	161/161	161/174	164/164	113/137	163/163	202/202	250/250	111/111	315/321	227/232
212/218	154/161	99/99	280/298	128/129	163/172	141/144	207/219	156/162	293/293	278/300	162/182	171/174	187/202	218/233	214/216	142/142	121/126	175/186	165/169	174/184	184/198	188/188	176/176	145/160	160/179	197/213	113/137	177/180	202/210	250/250	111/111	315/321	209/232
212/223	154/161	99/99	280/298	128/129	163/172	141/141	207/216	162/168	260/293	300/303	162/182	171/174	181/187	208/222	216/216	142/142	121/126	175/186	169/183	174/188	198/198	188/188	176/179	145/161	160/173	197/213	113/137	163/180	202/210	250/250	73/111	315/321	209/232
212/223	154/161	99/99	280/298	128/129	163/172	141/141	207/216	162/168	260/293	300/303	162/182	171/174	181/187	208/222	216/216	142/142	121/126	175/186	169/183	174/188	198/198	188/188	176/179	145/161	160/173	197/213	113/137	163/180	202/210	250/250	73/111	315/321	209/232
212/223	154/161	99/99	280/298	128/129	163/172	141/141	207/216	162/168	260/293	300/303	162/182	171/174	181/187	208/222	216/216	142/142	121/126	175/186	169/183	174/188	198/198	188/188	176/179	145/161	160/173	197/213	113/137	163/180	202/210	250/250	73/111	315/321	209/232
212/223	154/161	99/99	280/298	128/129	163/172	141/141	207/216	162/168	260/293	300/303	162/182	171/174	181/187	208/222	216/216	142/142	121/126	175/186	169/183	174/188	198/198	188/188	176/179	145/161	160/173	197/213	113/137	163/180	202/210	250/250	73/111	315/321	209/232
208/212	154/161	99/99	298/298	128/128	172/172	141/147	216/219	162/168	293/293	285/300	171/182	177/183	208/208	208/218	216/216	142/142	126/126	186/186	165/169	184/188	198/198	185/185	179/179	161/161	160/160	164/197	113/137	180/183	202/202	251/251	111/111	315/315	209/232
208/218	154/154	99/99	280/298	128/128	163/172	141/141	198/207	156/162	293/293	278/286	171/179	171/183	187/205	210/218	216/216	130/142	126/126	186/186	165/183	184/188	184/210	185/188	176/176	152/152	161/161	197/197	113/164	165/186	202/210	250/251	73/111	315/319	213/213
218/223	154/161	99/99	280/280	128/138	172/172	141/141	207/207	162/162	293/296	278/278	162/171	177/183	187/202	213/218	216/216	130/142	121/126	175/186	169/183	174/188	184/184	185/185	176/179	145/161	161/179	197/209	113/113	180/180	202/210	244/244	73/111	322/322	209/213

208/218	161/161	99/105	280/301	128/128	172/172	141/141	207/216	162/162	293/296	278/282	162/171	174/183	187/202	209/233	214/216	142/142	121/126	175/186	169/183	174/188	198/198	185/188	176/179	145/152	169/179	197/209	113/131	180/186	202/210	250/250	111/111	315/321	209/232
208/223	154/161	99/105	280/298	128/138	163/163	141/141	207/216	168/168	293/293	300/300	162/182	177/183	187/202	208/233	214/216	130/142	121/126	175/186	169/183	174/188	198/198	185/185	179/179	152/161	160/179	197/209	137/137	163/180	202/210	250/250	111/111	315/315	232/232
208/210	154/161	99/99	280/298	128/128	163/172	141/147	204/216	162/168	293/293	285/300	179/182	174/183	208/241	208/221	216/216	130/142	126/126	186/186	165/183	177/184	198/198	173/185	176/179	161/175	160/160	164/197	113/113	183/186	202/202	250/252	111/111	315/316	230/232
208/210	154/161	99/99	280/298	128/128	163/172	141/147	204/216	162/168	293/293	285/300	179/182	174/183	208/241	208/221	216/216	130/142	126/126	186/186	165/183	177/184	198/198	173/185	176/179	161/175	160/160	164/197	113/113	183/186	202/202	250/252	111/111	315/316	230/232
208/210	154/161	99/99	280/298	128/128	163/172	141/147	204/216	162/168	293/293	285/300	179/182	174/183	208/241	208/221	216/216	130/142	126/126	186/186	165/183	177/184	198/198	173/185	176/179	161/175	160/160	164/197	113/113	183/186	202/202	250/252	111/111	315/316	230/232
208/210	154/161	99/99	280/298	128/128	163/172	141/147	204/216	162/168	293/293	285/300	179/182	174/183	208/241	208/221	216/216	130/142	126/126	186/186	165/183	177/184	198/198	173/185	176/179	161/175	160/160	164/197	113/113	183/186	202/202	250/252	111/111	315/316	230/232
208/210	161/161	99/99	280/298	128/128	163/172	141/147	207/216	168/168	293/293	278/278	182/182	183/183	208/208	208/208	216/216	142/142	126/126	186/186	165/183	184/188	184/198	185/185	176/179	152/152	160/160	164/197	130/137	163/180	202/202	250/250	111/111	315/315	231/232
218/218	154/161	99/99	280/280	128/138	172/172	138/141	207/207	162/162	293/296	278/278	162/162	183/183	202/202	218/218	216/216	142/142	121/121	175/175	169/169	174/174	184/184	185/185	176/176	145/145	164/179	209/209	113/113	177/180	210/210	nd.	73/111	319/322	209/213
212/218	154/161	99/105	298/298	128/128	163/163	141/141	207/219	156/162	293/293	275/292	162/165	171/177	187/208	210/218	216/216	142/142	97/126	180/186	183/183	188/211	192/192	185/195	176/179	152/161	163/163	164/197	113/128	165/183	202/202	251/256	111/111	315/317	230/232
208/223	154/161	99/105	280/298	128/128	172/172	141/141	216/219	156/162	293/293	278/300	162/171	177/183	187/211	218/233	214/216	130/142	126/126	186/186	169/183	174/188	198/198	185/185	176/179	145/161	161/164	197/213	113/137	163/177	202/208	250/250	111/111	315/321	209/232
208/212	154/161	99/105	298/298	128/128	163/172	138/141	207/219	168/168	293/293	278/300	171/182	183/183	187/187	208/208	216/216	142/142	126/126	186/186	183/183	188/188	184/198	185/185	179/179	152/160	160/161	197/197	137/137	163/163	202/202	250/251	111/111	315/322	232/232
208/212	154/161	99/105	298/298	128/128	163/172	138/141	207/219	168/168	293/293	278/300	171/182	183/183	187/187	208/208	216/216	142/142	126/126	186/186	183/183	188/188	184/198	185/185	179/179	152/160	160/161	197/197	137/137	163/163	202/202	250/251	111/111	315/322	232/232
208/212	154/161	99/105	298/298	128/128	163/172	138/141	207/219	168/168	293/293	278/300	171/182	183/183	187/187	208/208	216/216	142/142	126/126	186/186	183/183	188/188	184/198	185/185	179/179	152/160	160/161	197/197	137/137	163/163	202/202	250/251	111/111	315/322	232/232
208/212	154/161	99/105	298/298	128/128	163/172	138/141	207/219	168/168	293/293	278/300	171/182	183/183	187/187	208/208	216/216	142/142	126/126	186/186	183/183	188/188	184/198	185/185	179/179	152/160	160/161	197/197	137/137	163/163	202/202	250/251	111/111	315/322	232/232
208/212	154/161	99/105	298/298	128/128	163/172	138/141	207/219	168/168	293/293	278/300	171/182	183/183	187/187	208/208	216/216	142/142	126/126	186/186	183/183	188/188	184/198	185/185	179/179	152/160	160/161	197/197	137/137	163/163	202/202	250/251	111/111	315/322	232/232
208/212	154/161	99/105	298/298	128/128	163/172	138/141	207/219	168/168	293/293	278/300	171/182	183/183	187/187	208/208	216/216	142/142	126/126	186/186	183/183	188/188	184/198	185/185	179/179	152/160	160/161	197/197	137/137	163/163	202/202	250/251	111/111	315/322	232/232
208/212	154/161	99/105	298/298	128/128	163/172	138/141	207/219	168/168	293/293	278/300	171/182	183/183	187/187	208/208	216/216	142/142	126/126	186/186	183/183	188/188	184/198	185/185	179/179	152/160	160/161	197/197	137/137	163/163	202/202	250/251	111/111	315/322	232/232
208/212	154/161	99/105	298/298	128/128	163/172	138/141	207/219	168/168	293/293	278/300	171/182	183/183	187/187	208/208	216/216	142/142	126/126	186/186	183/183	188/188	184/198	185/185	179/179	152/160	160/161	197/197	137/137	163/163	202/202	250/251	111/111	315/322	232/232
208/212	154/161	99/105	298/298	128/128	163/172	138/141	207/219	168/168	293/293	278/300	171/182	183/183	187/187	208/208	216/216	142/142	126/126	186/186	183/183	188/188	184/198	185/185	179/179	152/160	160/161	197/197	137/137	163/163	202/202	250/251	111/111	315/322	232/232
208/212	154/161	99/105	298/298	128/128	163/172	138/141	207/219	168/168	293/293	278/300	171/182	183/183	187/187	208/208	216/216	142/142	126/126	186/186	183/183	188/188	184/198	185/185	179/179	152/160	160/161	197/197	137/137	163/163	202/202	250/251	111/111	315/322	232/232
208/212	154/161	99/105	298/298	128/128	163/172	138/141	207/219	168/168	293/293	278/300	171/182	183/183	187/187	208/208	216/216	142/142	126/126	186/186	183/183	188/188	184/198	185/185	179/179	152/160	160/161	197/197	137/137	163/163	202/202	250/251	111/111	315/322	232/232
208/212	154/161	99/105	298/298	128/128	163/172	138/141	207/219	168/168	293/293	278/300	171/182	183/183	187/187	208/208	216/216	142/142	126/126	186/186	183/183	188/188	184/198	185/185	179/179	152/160	160/161	197/197	137/137	163/163	202/202	250/251	111/111	315/322	232/232
208/212	154/161	99/105	298/298	128/128	163/172	138/141	207/219	168/168	293/293	278/300	171/182	183/183	187/187	208/208	216/216	142/142	126/126	186/186	183/183	188/188	184/198	185/185	179/179	152/160	160/161	197/197	137/137	163/163	202/202	250/251	111/111	315/322	232/232
208/212	154/161	99/105	298/298	128/128	163/172	138/141	207/219	168/168	293/293	278/300	171/182	183/183	187/187	208/208	216/216	142/142	126/126	186/186	183/183	188/188	184/198	185/185	179/179	152/160	160/161	197/197	137/137	163/163	202/202	250/251	111/111	315/322	232/232
208/212	154/161	99/105	298/298	128/128	163/172	138/141	207/219	168/168	293/293	278/300	171/182	183/183	187/187	208/208	216/216	142/142	126/126	186/186	183/183	188/188	184/198	185/185	179/179	152/160	160/161	197/197	137/137	163/163	202/202	250/251	111/111	315/322	232/232
208/212	154/161	99/105	298/298	128/128	163/172	138/141	207/219	168/168	293/293	278/300	171/182	183/183	187/187	208/208	216/216	142/142	126/126	186/186	183/183	188/188	184/198	185/185	179/179	152/160	160/161	197/197	137/137	163/163	202/202	250/251	111/111	315/322	232/232
208/212	154/161	99/105	298/298	128/128	163/172	138/141	207/219	168/168	293/293	278/300	171/182	183/183	187/187	208/208	216/216	142/142	126/126	186/186	183/183	188/188	184/198	185/185	179/179	152/160	160/161	197/197	137/137	163/163	202/202	250/251	111/111	315/322	232/232
208/212	154/161	99/105	298/298	128/128	163/172	138/141	207/219	168/168	293/293	278/300	171/182	183/183	187/187	208/208	216/216	142/142	126/126	186/186	183/183	188/188	184/198	185/185	179/179	152/160	160/161	197/197	137/137	163/163	202/202	250/251	111/111	315/322	232/232
208/212	154/161	99/105	298/298	128/128	163/172	138/141	207/219	168/168	293/293	278/300	171/182	183/183	187/187	208/208	216/216	142/142	126/126	186/186	183/183	188/188	184/198	185/185	179/179	152/160	160/161	197/197	137/137	163/163	202/202	250/251	111/111	315/322	232/232
208/212	154/161	99/105	298/298	128/128	163/172	138/141	207/219	168/168	293/293	278/300	171/182	183/183	187/187	208/208	216/216	142/142	126/126	186/186	183/183	188/188	184/198	185/185	179/179	152/160	160/161	197/197	137/137	163/163	202/202	250/251	111/111	315/322	232/232
208/212	154/161	99/105	298/298	128/128	163/172	138/141	207/219	168/168	293/293	278/300	171/182	183/183	187/187	208/208	216/216	142/142	126/126	186/186	183/183	188/188	184/198	185/185	179/179	152/160	160/161	197/197	137/137	163/163	202/202	250/251	111/111	315/322	232/232
208/212	154/161	99/105	298/298	128/128	163/172	138/141	207/219	168/168	293/293	278/300	171/182	183/183	187/187	208/208	216/216	142/142	126/126	186/186	183/183	188/188	184/198	185/185	179/179	152/160	160/161	197/197	137/137	163/163	202/202	250/251	111/111	315/322	232/232
208/218	154/161	99/99	280/298	128/128	172/172	138/141	207/216	162/168	293/293	278/278	162/179	171/183	187/187	208/222	216/216	130/142	121/126	175/186	169/169	174/192	184/198	185/188	176/179	145/161	161/163	164/209	134/134	180/183	202/208	251/251	111/111	315/322	212/212
208/210	154/154	99/105	298/298	128/128	163/172	141/141	216/222	156/168	293/293	278/290	165/182	171/183	187/208	208/210	216/216	130/142	126/126	180/186	165/183	177/184	192/198	185/195	176/179	152/161	160/160	164/164	128/137	163/165	202/202	250/256	111/111	315/316	230/230
208/212	154/154	99/105	298/298	128/128	163/163	141/141	216/219	156/168	293/293	290/300	165/171	171/183	187/187	208/214	216/216	130/142	97/126	180/186	183/183	184/188	192/198	185/195	176/179	152/161	160/161	164/197	128/134	163/165	202/202	250/256	111/111	315/316	230/232
212/218	154/161	99/99	298/298	128/129	172/172	144/147	207/219	162/162	293/293	278/300	162/182	174/183	187/208	209/209	216/216	130/142	126/126	186/186	183/183	188/188	198/198	185/195	176/179	152/161	160/164	197/197	113/137	163/177	202/202	250/250	111/111	315/319	209/232
210/212	154/161	99/99	298/298	128/128	163/163	141/141	216/219	156/162	293/293	285/300	171/182	171/171	187/208	220/233	214/216	142/142	126/126	177/186	165/183	184/188	198/198	185/188	179/179	152/161	161/169	164/197	131/137	163/180	202/202	251/251	111/111	315/315	232/232
208/218	154/161	99/99	280/298	128/138	163/172	141/141	207/216	162/168	293/293	275/285	162/171	177/183	199/208	208/218	216/216	130/142	121/126	175/186	169/183	174/188	198/198	185/185	179/179	145/161	160/163	197/209	113/167	165/183	202/210	251/251	111/111	315/322	209/232
208/218	154/161	99/99	280/298	128/138	163/172	141/141	207/216	162/168	293/293	275/285	162/171	177/183	199/208	208/218	216/216	130/142	121/126	175/186	169/183	174/188	198/198	185/185	179/179	145/161	160/163	197/209	113/167	165/183	202/210	251/251	111/111	315/322	209/232
208/218	154/161	99/99	280/298	128/138	163/172	141/141	207/216	162/168	293/293	275/285	162/171	177/183	199/208	208/218	216/216	130/142	121/126	175/186	169/183	174/188	198/198	185/185	179/179	145/161	160/163	197/209	113/167	165/183	202/210	251/251	111/111	315/322	209/232
208/218	154/161	99/99	280/298	128/138	163/172	141/141	207/216	162/168	293/293	275/285	162/171	177/183	199/208	208/218	216/216	130/142	121/126	175/186	169/183	174/188	198/198	185/185	179/179	145/161	160/163	197/209	113/167	165/183	202/210	251/251	111/111	315/322	209/232
208/218	154/161	99/99	280/298	128/138	163/172	141/141	207/216	162/168	293/293	275/285	162/171	177/183	199/208	208/218	216/216	130/142	121/126	175/186	169/183	174/188	198/198	185/185	179/179	145/161	160/163	197/209	113/167	165/183	202/210	251/251	111/111	315/322	209/232
210/210	154/168	99/99	280/298	128/138	163/163	141/141	216/219	162/168	293/293	286/290	153/171	171/177	175/187	208/225	216/216	130/142	126/126	175/186	174/183	185/188	184/198	185/188	176/179	145/161	169/174	197/197	113/128	163/186	202/202	251/251	111/111	315/321	227/227
218/218	154/161	99/99	280/280	138/138	172/172	141/144	207/207	162/168	293/293	278/278	162/171	177/183	187/202	218/222	216/216	130/142	121/126	175/186	169/183	174/188	184/184	185/185	176/179	161/161	161/173	197/209	113/113	177/180	210/210	243/244	73/73	319/322	209/213
212/220	154/154	99/99	298/298	128/128	163/172	141/141	216/216	168/168	293/293	285/285	179/182	177/183	187/208	208/208	216/216	130/142	126/126	186/186	183/183	184/192	198/198	185/185	179/179	161/161	160/160	164/197	128/134	172/180	202/202	250/252	111/111	315/315	232/232
212/220	154/154	99/99	298/298	128/128	172/172	147/147	204/219	156/156	293/293	300/300	165/182	171/171	187/208	210/233	214/216	130/142	126/126	186/186	165/183	184/192	174/198	182/188	179/179	152/161	160/162	164/164	128/137	165/180	202/202	250/255	111/111	315/317	230/232
208/210	161/161	99/99	280/298	128/138	172/172	141/141	204/207	162/168	293/296	297/300	162/182	183/183	187/208	208/218	216/216	142/142	121/126	175/186	183/183	188/192	174/184	185/188	176/179	145/161	160/163	197/197	113/113	180/186	202/202	250/251	111/111	315/318	232/232
208/210	161/161	99/99	nd.	nd.	172/172	141/141	204/207	162/168	293/296	297/300	162/182	183/183	187/208	208/218	216/216	142/142	121/126	175/186	183/183	188/192	174/184	185/188	176/179	145/161	160/163	197/197	113/113	180/186	202/202	250/251	111/111	315/318	232/232
208/210	161/161	99/99	280/298	128/138	172/172	141/141	204/207	162/168	293/296	297/300	162/182	183/183	187/208	208/218	216/216	142/142	121/126	175/186	183/183	188/192	174/184	185/188	176/179	145/161	160/163	197/197	113/113	180/186	202/202	250/251	111/111	315/318	232/232
208/210	161/161	99/99	280/298	128/138	172/172	141/141	204/207	162/168	293/296	297/300	162/182	183/183	187/208	208/218	216/216	142/142	121/126	175/186	183/183	188/192	174/184	185/188	176/179	145/161	160/163	197/197	113/113	180/186	202/202	250/251	111/111	315/318	232/232
208/210	161/161	99/99	280/298	128/138	172/172	141/141	204/207	162/168	293/296	297/300	162/182	183/183	187/208	208/218	216/216	142/142	121/126	175/186	183/183	188/192	174/184	185/188	176/179	145/161	160/163	197/197	113/113	180/186	202/202	250/251	111/111	315/318	232/232
208/210	161/161	99/99	280/298	128/138	172/172	141/141	204/207	162/168	293/296	297/300	162/182	183/183	187/208	208/218	216/216	142/142	121/126	175/186	183/183	188/192	174/184	185/188	176/179	145/161	160/163	197/197	113/113	180/186	202/202	250/251	111/111	315/318	232/232
208/210	161/161	99/99	280/298	128/138	172/172	141/141	204/207	162/168	293/296	297/300	162/182	183/183	187/208	208/218	216/216	142/142	121/126	175/186	183/183	188/192	174/184	185/188	176/179	145/161	160/163	197/197	113/113	180/186	202/202	250/251	111/111	315/318	232/232
208/210	161/161	99/99	280/298	128/138	172/172	141/141	204/207	162/168	293/296	297/300	162/182	183/183	187/208	208/218	216/216	142/142	121/126	175/186	183/183	192/192	174/184	185/188	176/179	145/161	160/163	197/197	113/113	180/186	202/202	250/251	111/111	315/318	232/232
208/210	161/161	99/99	280/298	128/138	172/172	141/141	204/207	162/168	293/296	297/300	162/182	183/183	187/208	208/218	216/216	142/142	121/126	175/186	183/183	188/192	174/184	185/188	176/179	145/161	160/163	197/197	113/113	180/186	202/202	250/251	111/111	315/318	232/232
208/210	161/161	99/99	280/298	128/138	172/172	141/141	204/207	162/168	293/296	297/300	162/182	183/183	187/208	208/218	216/216	142/142	121/126	175/186	183/183	188/192	174/184	185/188	176/179	145/161	160/163	197/197	113/113	180/186	202/202	250/251	111/111	315/318	232/232
208/210	161/161	99/99	280/298	128/138	172/172	141/141	204/207	162/168	293/296	297/300	162/182	183/183	187/208	208/218	216/216	142/142	121/126	175/186	183/183	188/192	174/184	185/188	176/179	145/161	160/163	197/197	113/113	180/186	202/202	250/251	111/111	315/318	232/232
208/210	161/161	99/99	280/298	128/138	172/172	141/141	204/207	162/168	293/296	297/300	162/182	183/183	187/208	208/218	216/216	142/142	121/126	175/186	183/183	188/192	174/184	185/188	176/179	145/161	160/163	197/197	113/113	180/186	202/202	250/251	111/111	315/318	232/232
208/210	161/161	99/99	280/298	128/138	172/172	141/141	204/207	162/168	293/296	297/300	162/182	183/183	187/208	208/218	216/216	142/142	121/126	175/186	183/183	188/192	174/184	185/188	176/179	145/161	160/163	197/197	113/113	180/186	202/202	250/251	111/111	315/318	232/232
208/210	161/161	99/99	280/298	128/138	172/172	141/141	204/207	162/168	293/296	297/300	162/182	183/183	187/208	208/218	216/216	142/142	121/126	175/186	183/183	188/192	174/184	185/188	176/179	145/161	160/163	197/197	113/113	180/186	202/202	250/251	111/111	315/318	232/232
208/210	161/161	99/99	280/298	128/138	172/172	141/141	204/207	162/168	293/296	297/300	162/182	183/183	187/208	208/218	216/216	142/142	121/126	175/186	183/183	188/192	174/184	185/188	176/179	145/161	160/163	197/197	113/113	180/186	202/202	250/251	111/111	315/318	232/232
208/210	161/161	99/99	280/298	128/138	172/172	141/141	204/207	162/168	293/296	297/300	162/182	183/183	187/208	208/218	216/216	142/142	121/126	175/186	183/183	188/192	174/184	185/188	176/179	145/161	160/163	197/197	113/113	180/186	202/202	250/251	111/111	315/318	232/232
208/210	161/161	99/99	280/298	128/138	172/172	141/141	204/207	162/168	293/296	297/300	162/182	183/183	187/208	208/218	216/216	142/142	121/126	175/186	183/183	188/192	174/184	185/188	176/179	145/161	160/163	197/197	113/113	180/186	202/202	250/251	111/111	315/318	232/232
208/210	161/161	99/99	280/298	128/138	172/172	141/141	204/207	162/168	293/296	297/300	162/182	183/183	187/208	208/218	216/216	142/142	121/126	175/186	183/183	188/192	174/184	185/188	176/179	145/161	160/163	197/197	113/113	180/186	202/202	250/251	111/111	315/318	232/232
208/210	161/161	99/99	280/298	128/138	172/172	141/141	204/207	162/168	293/296	297/300	162/182	183/183	187/208	208/218	216/216	142/142	121/126	175/186	183/183	188/192	174/184	185/188	176/179	145/161	160/163	197/197	113/113	180/186	202/202	250/251	111/111	315/318	232/232
208/210	161/161	99/99	280/298	128/138	172/172	141/141	204/207	162/168	293/296	297/300	162/182	183/183	187/208	208/218	216/216	142/142	121/126	175/186	183/183	188/192	174/184	185/188	176/179	145/161	160/163	197/197	113/113	180/186	202/202	250/251	111/111	315/318	232/232
208/210	161/161	99/99	280/298	128/138	172/172	141/141	204/207	162/168	293/296	297/300	162/182	183/183	187/208	208/218	216/216	142/142	121/126	175/186	183/183	188/192	174/184	185/188	176/179	145/161	160/163	197/197	113/113	180/186	202/202	250/251	111/111	315/318	232/232
208/220	154/154	99/105	298/298	128/128	163/172	141/141	216/219	156/162	293/293	286/300	165/165	171/171	187/187	210/221	216/216	142/142	97/126	180/186	183/183	192/200	192/198	182/195	176/176	152/152	nd.	164/164	128/134	163/165	202/202	256/256	111/111	315/317	230/230
220/220	154/154	99/105	298/298	128/128	163/163	141/141	216/219	156/162	293/293	286/300	165/171	171/171	187/205	210/221	216/216	130/142	97/126	180/186	183/183	177/192	192/198	182/195	185/185	152/176	162/162	164/164	128/134	163/165	202/202	255/256	111/111	315/317	230/230
220/220	154/154	99/105	298/298	128/128	163/163	141/141	216/219	156/162	293/293	286/300	165/171	171/171	187/205	210/221	216/216	130/142	97/126	180/186	183/183	177/192	192/198	182/195	185/185	152/176	162/162	164/164	128/134	163/165	202/202	255/256	111/111	315/317	230/230
220/220	154/154	99/105	298/298	128/128	163/163	141/141	216/219	156/162	293/293	286/300	165/171	171/171	187/205	210/221	216/216	130/142	97/126	180/186	183/183	177/192	192/198	182/195	185/185	152/176	162/162	164/164	128/134	163/165	202/202	255/256	111/111	315/317	230/230
208/220	154/154	99/105	298/298	128/128	163/172	141/141	216/219	156/162	293/293	286/300	165/165	171/171	187/187	210/221	216/216	142/142	97/126	180/186	183/183	192/200	192/198	182/195	176/176	152/152	nd.	164/164	128/134	163/165	202/202	256/256	111/111	315/317	230/230
208/220	154/154	99/105	298/298	128/128	163/172	141/141	216/219	156/162	293/293	286/300	165/165	171/171	187/187	210/221	216/216	142/142	97/126	180/186	183/183	192/200	192/198	182/195	176/176	152/152	nd.	164/164	128/134	163/165	202/202	256/256	111/111	315/317	230/230
208/212	154/154	99/105	298/298	128/128	163/163	141/141	198/219	156/168	293/293	286/286	165/179	171/171	187/205	208/210	216/216	130/142	126/126	186/186	183/183	177/188	192/210	188/195	176/176	152/152	161/161	164/197	128/164	165/165	202/202	251/256	111/111	315/318	230/230
220/220	154/154	99/105	298/298	128/128	163/163	141/141	216/219	156/162	293/293	286/300	165/171	171/171	187/205	210/221	216/216	130/142	97/126	180/186	183/183	177/192	192/198	182/195	185/185	152/176	162/162	164/164	128/134	163/165	202/202	255/256	111/111	315/317	230/230
220/220	154/154	99/105	298/298	128/128	163/163	141/141	216/219	156/162	293/293	286/300	165/171	171/171	187/205	210/221	216/216	130/142	97/126	180/186	183/183	177/192	192/198	182/195	185/185	152/176	162/162	164/164	128/134	163/165	202/202	255/256	111/111	315/317	230/230
220/220	154/154	99/105	298/298	128/128	163/163	141/141	216/219	156/162	293/293	286/300	165/171	171/171	187/205	210/221	216/216	130/142	97/126	180/186	183/183	177/192	192/198	182/195	185/185	152/176	162/162	164/164	128/134	163/165	202/202	255/256	111/111	315/317	230/230
208/220	154/154	99/105	298/298	128/128	163/172	141/141	216/219	156/162	293/293	286/300	165/165	171/171	187/187	210/221	216/216	142/142	97/126	180/186	183/183	192/200	192/198	182/195	176/176	152/152	nd.	164/164	128/134	163/165	202/202	256/256	111/111	315/317	230/230
220/220	154/154	99/105	298/298	128/128	163/163	141/141	216/219	156/162	293/293	286/300	165/171	171/171	187/205	210/221	216/216	130/142	97/126	180/186	183/183	177/192	192/198	182/195	185/185	152/176	162/162	164/164	128/134	163/165	202/202	255/256	111/111	315/317	230/230
210/220	154/154	99/105	298/298	138/138	163/163	141/141	216/219	156/168	293/293	278/300	162/165	171/177	187/205	208/210	216/216	142/142	97/126	180/186	183/183	177/188	192/198	185/195	179/185	152/161	169/170	164/197	113/128	165/186	202/202	251/256	111/111	317/321	211/230
208/223	161/161	99/99	280/298	128/138	163/172	141/141	207/207	162/168	293/293	278/285	162/171	183/183	181/208	208/218	216/216	142/142	121/126	175/186	169/169	174/174	184/184	185/185	179/185	145/161	160/173	197/209	113/167	165/177	202/210	244/244	111/111	315/319	209/232
208/210	161/161	99/99	280/298	128/128	163/172	141/147	207/216	168/168	293/296	278/297	162/182	174/183	208/238	208/208	216/216	142/142	121/126	175/186	183/183	177/188	184/198	173/185	176/179	152/161	160/163	197/197	113/137	163/180	202/202	250/251	111/111	315/318	232/232
208/210	161/161	99/99	280/298	128/128	163/172	141/147	207/216	168/168	293/296	278/297	162/182	174/183	208/238	208/208	216/216	142/142	121/126	175/186	183/183	177/188	184/198	173/185	176/179	152/161	160/163	197/197	113/137	163/180	202/202	250/251	111/111	315/318	232/232
208/210	161/161	99/99	280/298	128/128	163/172	141/147	207/216	168/168	293/296	278/297	162/182	174/183	208/238	208/208	216/216	142/142	121/126	175/186	183/183	177/188	184/198	173/185	176/179	152/161	160/163	197/197	113/137	163/180	202/202	250/251	111/111	315/318	232/232
208/210	161/161	99/99	280/298	128/128	163/172	141/147	207/216	168/168	293/296	278/297	162/182	174/183	208/238	208/208	216/216	142/142	121/126	175/186	183/183	177/188	184/198	173/185	176/179	152/161	160/163	197/197	113/137	163/180	202/202	250/251	111/111	315/318	232/232
208/210	161/161	99/99	280/298	128/138	172/172	141/141	207/216	168/168	293/293	297/300	162/171	183/183	208/211	208/208	216/216	142/142	121/126	175/186	165/183	184/192	184/198	185/188	176/176	145/175	160/163	164/197	113/134	172/186	202/202	251/251	111/111	316/318	230/232
210/223	161/161	99/99	280/280	128/128	172/172	141/141	204/207	162/168	293/293	278/300	171/182	183/183	181/187	208/209	216/216	142/142	121/126	175/186	169/183	174/188	174/184	185/188	176/179	145/161	160/161	197/209	113/113	180/186	202/210	250/250	73/111	315/319	213/232
210/212	154/161	99/99	280/298	128/138	163/172	141/141	204/216	162/162	293/296	286/297	179/182	183/183	187/223	208/218	216/216	130/142	126/126	186/186	183/183	188/192	174/198	185/188	179/179	161/161	160/160	197/197	113/134	180/186	202/202	250/250	111/111	315/316	232/232
210/220	154/154	99/99	298/298	128/138	163/172	141/147	204/219	156/168	293/293	286/300	162/165	171/177	187/187	208/210	216/216	130/142	126/126	180/186	183/183	188/192	174/192	182/185	179/179	152/161	162/170	164/197	113/128	165/186	202/202	251/255	111/111	317/321	211/230
212/218	154/161	99/105	280/298	128/128	163/172	138/141	207/216	162/168	293/293	278/300	162/171	183/183	187/208	208/209	216/216	130/142	126/126	186/186	165/183	184/188	184/198	185/185	179/179	152/161	161/170	164/197	113/137	163/186	202/202	250/250	111/111	315/322	232/232
218/223	154/161	99/99	280/280	128/138	172/172	141/141	207/207	162/162	293/296	278/278	162/171	177/183	187/202	213/218	216/216	130/142	121/126	175/186	169/183	174/188	184/184	185/185	176/179	145/161	161/179	197/209	113/113	180/180	202/210	244/244	73/111	322/322	209/213
210/218	154/154	99/105	280/298	128/128	163/163	141/141	207/222	168/168	nd.	278/290	165/171	171/183	187/187	208/208	216/216	130/142	126/126	180/186	165/183	177/184	184/192	185/185	179/179	161/161	160/161	164/164	128/134	163/165	202/202	250/250	111/111	316/322	230/232
212/218	154/161	99/105	280/298	128/128	163/172	138/141	207/216	162/168	293/293	278/300	162/171	183/183	187/208	208/209	216/216	130/142	126/126	186/186	165/183	184/188	184/198	185/185	179/179	152/161	161/170	164/197	113/137	163/186	202/202	250/250	111/111	315/322	232/232
212/218	154/161	99/105	280/298	128/128	163/172	138/141	207/216	162/168	293/293	278/300	162/171	183/183	187/208	208/209	216/216	130/142	126/126	186/186	165/183	184/188	184/198	185/185	179/179	152/161	161/170	164/197	113/137	163/186	202/202	250/250	111/111	315/322	232/232
212/218	154/161	99/105	280/298	128/128	163/172	138/141	207/216	162/168	293/293	278/300	162/171	183/183	187/208	208/209	216/216	130/142	126/126	186/186	165/183	184/188	184/198	185/185	179/179	152/161	161/170	164/197	113/137	163/186	202/202	250/250	111/111	315/322	232/232
218/223	161/161	99/99	280/280	128/129	172/172	144/144	207/207	162/162	260/293	278/278	162/162	174/174	181/211	218/218	216/216	142/142	121/121	175/175	169/169	174/174	nd.	188/188	176/176	145/145	179/179	209/209	113/113	180/180	208/210	nd.	73/111	319/322	213/213
212/218	154/161	99/99	280/298	128/128	163/172	138/141	207/219	162/168	293/296	278/300	162/171	171/183	187/208	208/218	216/216	130/142	126/126	186/186	165/183	184/188	184/198	185/188	176/176	145/160	161/179	164/197	113/137	163/177	202/202	250/250	73/111	315/322	209/232
210/212	154/161	99/105	298/298	128/128	163/172	138/141	216/219	156/162	293/293	278/285	165/171	171/183	187/205	209/210	216/216	142/142	126/126	180/186	183/183	188/200	192/198	185/195	179/185	152/161	160/161	164/197	113/128	165/186	202/202	256/256	111/111	317/322	230/232
208/223	154/161	99/105	280/298	128/128	163/172	141/141	207/219	162/168	293/293	300/303	162/171	183/183	187/202	208/218	216/216	142/142	121/126	175/186	169/183	174/188	184/198	185/188	176/179	145/160	161/179	197/209	113/137	163/177	202/208	250/250	111/111	315/319	213/232
210/220	154/161	99/99	298/298	128/138	163/172	141/141	204/216	162/168	293/293	285/300	171/179	171/171	208/223	208/208	216/216	142/142	126/126	186/186	165/183	184/204	174/198	185/188	179/179	152/175	169/169	164/164	134/137	163/172	202/202	251/251	111/111	315/316	230/230
208/210	154/161	99/99	298/298	128/128	163/172	141/141	204/216	168/168	293/293	285/285	171/182	171/183	208/211	208/208	216/216	130/142	126/126	186/186	165/183	184/204	198/198	185/188	179/179	152/175	160/160	164/164	134/137	163/172	202/202	251/251	111/111	315/316	230/230
220/223	154/161	99/99	280/298	128/128	163/172	138/141	207/219	162/168	293/293	278/300	162/182	171/177	208/211	208/218	216/216	130/142	121/126	175/186	165/169	174/184	184/192	182/185	176/176	145/161	160/164	164/213	113/137	163/177	202/208	250/250	111/111	315/321	209/232
210/223	161/161	99/99	280/280	128/138	172/172	141/141	204/207	162/168	293/296	278/300	162/182	177/183	187/202	208/218	216/216	142/142	121/126	175/186	169/183	174/188	174/184	185/185	176/179	145/161	160/173	197/209	113/113	180/186	202/210	251/251	111/111	315/322	213/232
208/218	154/161	99/105	298/298	128/128	172/172	138/147	207/219	162/168	293/293	300/300	171/182	183/186	187/187	208/209	216/216	130/142	126/126	186/186	183/183	188/188	184/198	185/185	179/179	161/161	160/161	197/197	113/137	163/186	202/202	250/250	111/111	315/322	232/232
212/218	154/154	99/99	280/298	128/128	163/172	141/144	207/219	162/162	260/293	278/300	162/172	171/177	202/208	218/218	216/216	130/142	121/126	175/186	169/183	174/188	184/198	188/188	176/176	145/145	179/179	164/209	113/137	163/180	202/210	244/244	111/111	315/322	232/232
220/220	154/154	99/99	298/298	128/128	163/172	141/141	216/216	168/168	293/293	300/300	179/182	171/177	208/208	208/208	216/216	130/142	126/126	186/186	165/183	184/204	174/198	188/188	179/179	152/161	160/169	164/164	130/137	163/180	202/202	251/251	111/111	315/316	nd.
212/223	161/161	99/99	280/298	128/128	163/172	141/141	207/219	162/168	293/293	278/300	162/182	171/177	187/211	208/209	216/216	142/142	121/126	175/186	169/183	174/188	184/198	185/188	176/176	160/160	160/164	197/213	113/137	163/177	202/208	250/250	111/111	315/321	209/232
226/226	154/168	99/99	280/298	128/128	163/163	141/141	219/219	156/162	293/293	290/316	153/174	171/180	175/187	216/225	216/216	130/142	126/126	175/180	169/174	185/196	184/192	179/188	176/176	145/161	161/174	164/164	113/128	163/163	202/202	250/250	111/111	315/321	227/227
210/212	161/161	99/105	280/298	128/128	163/172	138/141	204/204	162/168	293/293	278/278	182/182	183/183	187/208	208/218	216/216	142/142	121/126	175/186	183/183	188/188	174/174	173/188	176/176	145/160	160/160	197/197	113/130	180/186	202/202	250/250	111/111	321/322	209/232
210/212	161/161	99/105	280/298	128/128	172/172	141/141	216/219	162/168	293/293	278/300	182/182	171/183	187/208	208/218	216/216	142/142	121/126	175/186	183/183	188/192	198/198	188/188	176/179	152/160	160/160	197/197	113/137	163/186	202/202	250/250	111/111	315/322	232/232

208/208	161/161	99/105	298/298	128/128	172/172	138/141	204/219	162/168	293/293	297/300	162/182	183/183	187/208	208/218	216/216	142/142	121/126	175/186	183/183	188/188	174/198	185/185	179/179	160/161	160/163	197/197	113/137	163/180	202/202	250/251	111/111	318/322	232/232
208/218	154/161	99/99	280/298	128/128	163/172	141/141	207/216	162/168	293/293	278/285	171/182	183/183	187/208	208/220	216/216	142/142	126/126	186/186	169/183	174/204	184/198	185/188	176/179	145/175	160/160	164/197	113/134	172/180	202/210	251/251	111/111	315/319	213/230
210/212	161/161	99/105	298/298	128/128	163/172	141/141	204/204	168/168	293/293	278/282	171/182	171/183	187/211	208/208	216/216	130/142	126/126	186/186	183/183	184/188	174/198	188/188	176/179	145/175	160/161	164/197	134/137	163/172	202/202	250/251	111/111	316/322	230/232
208/210	161/161	99/99	280/298	138/138	172/172	141/141	204/216	168/168	293/296	285/297	162/182	183/183	187/211	208/208	216/216	130/142	121/126	175/186	165/183	184/192	174/198	185/188	176/179	161/175	160/163	164/197	113/113	186/186	202/202	250/251	111/111	318/318	232/232
208/208	161/161	99/99	298/298	128/128	172/172	141/141	204/204	168/168	293/296	278/285	179/182	183/183	187/208	208/208	216/216	142/142	121/126	175/186	165/183	184/192	174/198	185/188	176/179	160/160	160/160	164/197	113/137	163/186	202/202	250/252	111/111	316/322	230/232
218/218	161/161	99/105	280/280	128/128	163/172	141/141	207/221	156/168	293/296	278/279	162/162	171/174	187/199	209/213	216/216	142/142	121/126	175/186	169/183	178/188	184/184	185/185	176/177	145/145	164/179	197/209	113/113	177/180	202/208	244/244	111/111	316/321	209/228
208/220	154/161	99/105	298/298	128/128	172/172	138/141	207/219	156/168	293/296	300/300	171/182	183/183	202/208	208/210	216/216	142/142	121/126	175/186	165/183	184/188	184/192	185/185	176/179	145/161	160/162	164/197	113/128	165/186	202/202	250/255	111/111	315/318	232/232
210/210	161/161	99/99	280/298	128/128	163/172	141/141	207/216	168/168	293/293	285/300	182/182	171/183	208/211	208/208	216/216	130/142	121/126	175/186	183/183	184/188	184/198	185/188	179/179	145/161	160/161	164/197	113/119	180/186	202/202	251/251	111/111	315/316	230/232
218/223	154/161	99/99	280/280	128/138	172/172	141/144	207/207	162/162	293/293	278/278	162/162	174/177	199/202	218/222	216/216	142/142	121/121	175/175	169/169	174/174	184/184	185/185	176/176	145/145	163/179	209/209	113/113	177/180	210/210	244/244	111/111	319/322	209/209
218/218	154/161	99/99	280/280	128/138	172/172	141/141	207/207	162/168	293/293	278/278	162/162	174/183	202/211	218/222	216/216	142/142	121/121	175/175	169/169	174/189	184/184	185/188	176/176	145/161	164/164	209/209	113/113	177/180	210/210	244/244	73/111	319/321	209/209
210/210	161/161	99/105	280/298	138/138	163/172	141/141	204/207	162/168	293/293	285/300	171/182	177/183	208/211	208/218	216/216	142/142	121/126	175/186	183/183	192/192	174/184	185/185	176/179	145/152	160/161	197/197	113/137	163/177	202/202	250/251	111/111	315/322	232/232
210/210	154/161	99/99	298/298	128/128	163/172	141/141	204/216	168/168	293/293	282/285	171/182	171/183	211/216	208/208	216/216	130/142	126/126	186/186	183/183	184/192	198/198	185/188	179/179	145/175	160/161	164/197	119/134	172/180	202/202	251/252	111/111	316/316	230/230
210/210	161/161	99/99	280/298	128/138	163/172	141/141	204/204	162/168	293/293	285/300	182/182	183/183	187/211	208/218	216/216	142/142	126/126	186/186	183/183	177/192	174/198	173/185	176/179	145/145	160/160	197/197	113/137	163/177	202/202	250/251	111/111	315/315	230/232
208/210	161/161	99/99	298/298	128/138	172/172	141/144	204/204	162/168	293/293	285/300	179/182	174/183	187/211	208/218	216/216	142/142	126/126	186/186	165/183	188/192	174/198	173/185	176/179	145/145	160/160	197/197	113/137	163/177	202/202	250/251	111/111	315/315	230/232
210/212	161/161	99/105	280/298	128/138	163/163	141/141	207/219	168/168	293/296	278/300	182/182	183/183	187/208	208/208	216/216	142/142	121/126	175/186	183/183	188/188	184/198	185/188	176/179	160/160	160/160	197/197	113/137	163/186	202/202	250/251	111/111	315/315	232/232
208/210	154/161	99/99	298/298	128/128	163/172	141/141	216/216	168/168	293/293	282/300	171/179	171/174	208/216	208/208	216/216	130/142	126/126	186/186	165/183	184/192	198/198	185/188	179/179	145/161	160/160	164/197	113/134	172/186	202/202	250/252	111/111	315/316	230/232
208/220	154/161	105/105	298/298	128/128	172/172	141/144	204/219	156/168	293/293	278/300	171/182	183/183	187/208	208/210	216/216	142/142	126/126	186/186	183/183	188/188	174/192	185/185	176/179	160/161	160/160	197/197	128/137	163/165	202/202	250/250	111/111	318/322	232/232
212/220	154/161	99/105	298/298	128/128	163/163	141/141	216/219	156/162	293/293	300/303	171/182	171/183	187/205	209/222	216/216	142/142	97/126	180/186	183/183	177/188	192/198	185/188	176/179	145/161	160/162	164/197	113/128	165/180	202/202	250/255	111/111	315/315	232/232
208/210	161/161	99/99	280/298	128/128	163/172	141/147	204/204	162/168	293/296	278/285	179/182	183/183	208/208	208/218	216/216	142/142	121/126	175/186	165/183	184/192	174/198	185/185	176/179	160/175	160/160	164/197	113/137	163/186	202/202	251/252	111/111	316/322	230/232
208/208	154/161	99/105	298/298	128/128	163/172	138/147	204/219	168/168	293/293	300/300	179/182	174/183	187/241	208/208	216/216	130/142	126/126	186/186	183/183	177/188	198/198	173/185	176/179	152/161	160/160	197/197	113/137	163/186	202/202	251/252	111/111	315/315	232/232
218/223	154/161	99/99	280/280	128/138	172/172	141/144	207/207	162/162	293/293	278/278	162/162	177/183	202/211	218/222	216/216	142/142	121/121	175/175	169/169	174/174	184/184	185/185	176/176	145/161	173/173	209/213	113/113	177/180	210/210	244/244	111/111	319/322	209/209
208/212	154/161	99/105	298/298	128/128	163/172	141/141	198/219	156/162	293/293	278/300	171/182	183/183	187/208	210/218	216/216	130/142	126/126	186/186	165/183	184/188	198/210	185/188	176/176	152/160	160/161	197/197	137/164	163/165	202/202	250/251	73/111	315/319	213/232
208/210	161/161	99/105	280/298	128/128	172/172	141/141	204/216	168/168	293/293	278/282	182/182	174/183	187/208	208/208	216/216	142/142	121/126	175/186	183/183	188/192	174/198	173/188	176/179	145/152	160/160	197/197	137/137	163/163	202/202	251/251	111/111	315/322	232/232
208/210	154/154	99/99	298/298	128/128	163/163	141/147	207/216	168/168	293/293	285/285	171/182	171/183	211/216	208/208	216/216	142/142	126/126	186/186	165/183	184/204	184/198	188/192	164/179	152/152	160/161	164/197	119/134	172/180	202/202	251/252	111/111	315/315	230/231
208/212	161/161	99/99	298/298	128/138	172/172	138/141	204/216	168/168	293/293	278/285	179/182	183/183	208/241	208/208	216/216	130/142	121/126	175/186	183/183	177/192	174/198	185/188	176/179	160/161	160/160	197/197	113/137	163/186	202/202	250/252	111/111	316/322	230/232
210/212	154/161	99/105	298/298	128/128	163/172	141/141	204/219	162/168	293/293	282/300	171/182	171/183	187/187	208/208	216/216	142/142	126/126	186/186	183/183	188/192	198/198	185/188	179/179	152/160	161/161	197/197	113/137	163/186	202/202	250/251	111/111	315/316	230/232
210/210	161/161	99/105	280/298	128/138	172/172	141/141	204/204	168/168	293/293	282/285	182/182	171/183	208/235	208/208	216/216	130/142	121/126	175/186	183/183	177/192	174/198	185/188	179/179	145/152	160/161	197/197	130/137	163/180	202/202	250/250	111/111	315/316	230/230
210/210	154/161	99/99	280/298	128/128	163/172	141/141	204/216	168/168	293/293	282/285	182/182	171/183	211/216	208/208	216/216	142/142	126/126	186/186	165/165	184/184	198/198	188/192	164/179	152/175	160/161	164/197	113/134	172/186	202/202	251/252	111/111	315/316	230/230
210/212	161/161	99/105	280/298	128/128	172/172	138/141	204/219	162/168	293/296	278/300	162/182	183/183	187/208	208/218	216/216	142/142	121/126	175/186	183/183	188/192	174/198	185/188	176/179	145/160	160/163	197/197	113/137	163/186	202/202	250/251	111/111	315/322	232/232
218/220	154/161	105/105	280/298	128/128	163/172	138/141	207/219	156/162	293/293	278/300	171/171	171/183	202/205	209/210	216/216	142/142	97/121	175/180	165/183	177/184	184/192	182/185	176/185	152/161	161/162	164/164	113/128	165/180	202/202	255/255	73/111	315/319	213/213
208/210	161/161	99/99	280/298	128/138	163/172	141/141	204/216	168/168	293/293	285/300	179/182	183/183	208/235	208/208	216/216	130/142	121/126	175/186	183/183	177/192	198/198	185/188	176/179	145/175	160/160	197/197	113/113	177/186	202/202	250/251	111/111	315/315	230/232
218/218	154/161	99/105	280/298	128/128	172/172	141/144	207/207	168/168	293/293	278/300	162/171	174/177	202/202	213/222	216/216	142/142	121/121	175/175	169/183	174/188	184/184	185/188	176/176	145/161	161/173	197/209	113/113	177/177	202/210	244/244	111/111	315/319	209/232
210/212	161/161	99/99	298/298	128/128	163/172	141/141	204/219	168/168	293/293	278/285	171/182	171/183	187/211	208/208	216/216	142/142	126/126	186/186	183/183	184/188	198/198	185/185	176/179	160/160	160/161	164/197	119/137	163/180	202/202	250/250	111/111	316/322	230/232
208/210	154/161	99/105	298/298	128/128	163/172	138/141	216/219	168/168	293/293	278/285	171/182	183/183	187/216	208/208	216/216	142/142	126/126	186/186	183/183	188/192	198/198	185/185	179/179	160/175	161/161	197/197	119/137	163/180	202/202	250/251	111/111	316/322	230/232
210/220	161/161	99/99	280/298	128/128	172/172	141/144	204/216	162/168	293/293	300/300	179/182	177/183	187/235	208/218	216/216	142/142	126/126	186/186	183/183	177/188	174/198	185/188	176/179	145/152	160/160	197/197	113/130	177/180	202/202	250/250	111/111	315/316	232/232
210/212	161/161	99/105	298/298	128/138	172/172	138/141	204/219	168/168	293/293	278/285	162/171	183/183	187/211	208/208	216/216	142/142	126/126	186/186	183/183	188/192	174/198	nd.	179/179	152/175	161/163	197/197	113/137	163/186	202/202	251/251	111/111	318/322	232/232
210/212	161/161	99/105	280/298	128/138	172/172	138/141	204/204	168/168	293/293	278/297	182/182	183/183	208/208	208/208	216/216	142/142	121/121	175/175	183/183	188/192	174/174	185/188	176/179	145/160	160/160	197/197	113/137	163/186	202/202	250/250	111/111	318/322	232/232
210/212	154/161	99/99	280/298	128/128	172/172	141/147	216/219	156/168	293/293	300/300	182/182	171/183	187/208	208/233	214/216	130/142	121/126	175/186	183/183	188/192	198/198	188/188	176/179	145/161	160/160	197/197	113/137	163/186	202/202	250/250	111/111	315/315	230/232
208/210	154/161	99/105	280/298	128/128	163/172	147/147	207/219	168/168	293/293	282/300	182/182	171/171	187/187	208/208	216/216	142/142	126/126	186/186	165/183	184/192	184/198	185/192	164/179	152/161	160/160	164/197	119/137	180/180	202/202	250/251	111/111	315/316	230/232
218/218	161/161	99/105	280/280	128/128	163/172	141/141	207/207	156/162	293/296	278/279	156/162	171/174	187/187	203/222	216/216	142/142	121/128	175/175	169/183	174/188	184/184	185/185	176/177	145/145	167/172	197/197	113/113	177/180	202/208	244/244	73/73	316/319	209/228
208/210	161/161	99/99	280/298	128/138	172/172	141/144	204/216	168/168	293/293	297/300	162/179	183/183	208/235	208/208	216/216	130/142	121/126	175/186	183/183	188/192	198/198	173/185	176/179	145/175	160/163	197/197	113/113	177/186	202/202	250/250	111/111	315/318	232/232
208/210	161/161	99/105	280/298	128/128	163/172	138/141	207/216	162/168	293/293	278/300	171/182	183/183	187/208	208/218	216/216	142/142	121/126	175/186	183/183	188/192	184/198	185/185	176/179	145/152	160/161	197/197	113/137	163/177	202/202	250/251	111/111	315/322	232/232
208/210	161/161	99/99	280/298	128/128	163/172	141/144	204/207	162/168	293/293	300/300	179/182	174/183	187/235	208/218	216/216	142/142	126/126	186/186	183/183	177/188	184/198	173/188	176/176	145/161	160/160	197/197	113/113	177/186	202/202	250/250	111/111	315/315	232/232
210/218/223	161/161	99/99	280/280	128/138	172/172	141/144	204/207	162/162	293/293	278/300	162/182	174/183	187/202/220	218/218	216/216	142/142	121/126	175/186	169/183	174/188	174/184	185/188	176/176	145/161	160/173/179	197/209/213	113/113	180/180	202/210	244/244	111/111	315/319/321	209/232
208/210	161/161	99/99	280/298	128/138	163/172	141/141	204/216	168/168	293/293	285/300	182/182	174/183	187/211	208/208	216/216	130/142	126/126	186/186	183/183	188/192	198/198	173/185	176/179	145/175	160/160	164/197	113/137	163/177	202/202	250/250	111/111	315/315	230/232
210/210	161/161	99/99	280/298	128/128	172/172	141/144	204/216	162/168	293/293	297/300	162/182	177/183	211/235	208/218	216/216	142/142	126/126	186/186	183/183	177/192	174/198	185/185	176/179	145/152	160/163	197/197	113/137	163/177	202/202	250/251	111/111	315/318	232/232
208/210	154/161	99/99	280/298	128/128	163/172	141/141	204/207	162/168	293/293	278/285	162/182	183/183	187/208	208/218	216/216	142/142	126/126	186/186	183/183	188/204	184/198	185/188	176/179	145/175	160/173	164/197	113/134	172/180	202/210	251/251	111/111	315/319	213/230
208/210	161/161	99/105	280/298	128/128	163/172	141/141	207/216	162/168	293/293	300/300	182/182	171/171	235/235	208/208	216/216	142/142	126/126	186/186	165/183	177/184	184/198	188/192	164/179	152/152	161/161	164/197	130/130	180/180	202/202	250/250	111/111	316/316	230/230
208/210	161/161	99/99	298/298	128/138	163/172	141/141	204/216	162/168	293/293	297/300	179/182	183/183	187/211	208/218	216/216	130/142	126/126	186/186	165/183	184/188	198/198	173/185	176/179	161/175	160/160	164/197	113/113	186/186	202/202	250/251	111/111	315/318	232/232
210/212	161/161	99/105	280/298	128/138	163/163	138/141	204/207	162/168	293/293	278/285	182/182	183/183	187/211	208/218	216/216	142/142	126/126	186/186	183/183	177/188	184/198	185/185	179/179	145/152	160/160	197/197	113/137	163/177	202/202	250/250	111/111	315/322	230/232
208/210	154/161	99/105	298/298	128/128	172/172	138/141	204/219	162/168	293/293	278/285	179/182	183/183	208/241	208/218	216/216	130/142	126/126	186/186	183/183	177/192	198/198	185/188	176/176	160/161	160/160	197/197	113/113	186/186	202/202	250/251	111/111	316/322	230/232
210/220	161/161	99/99	280/298	128/128	172/172	138/144	204/219	168/168	293/296	278/300	182/182	183/183	187/208	208/208	216/216	142/142	126/126	186/186	183/183	188/192	174/198	185/188	176/179	145/160	160/160	197/197	130/137	163/180	202/202	250/251	111/111	316/322	232/232
210/212	161/161	99/105	298/298	128/128	172/172	141/141	207/216	168/168	293/293	285/285	171/182	174/183	216/241	208/208	216/216	130/142	126/126	186/186	183/183	188/192	184/198	173/185	176/179	145/161	160/161	197/197	113/137	163/186	202/202	250/250	111/111	315/316	230/232
208/210	161/161	99/99	280/298	128/128	163/172	141/144	204/216	162/168	293/293	278/300	179/182	174/183	187/235	208/218	216/216	142/142	126/126	186/186	165/183	184/188	174/198	173/188	176/176	145/161	160/160	164/197	113/130	177/180	202/202	250/252	111/111	315/321	209/232
210/212	161/161	99/105	280/298	128/128	163/172	138/141	216/219	162/162	293/293	285/300	162/179	183/183	208/241	218/220	216/216	142/142	121/126	175/186	183/183	177/188	198/198	185/185	179/179	160/175	160/163	197/197	113/113	186/186	202/202	251/252	111/111	315/316	230/232
208/210	154/161	99/105	298/298	128/128	172/172	138/141	204/219	162/168	293/293	278/285	179/182	183/183	208/241	208/218	216/216	130/142	126/126	186/186	183/183	177/192	198/198	185/188	176/176	160/161	160/160	197/197	113/113	186/186	202/202	250/251	111/111	316/322	230/232
212/218	154/161	99/105	298/298	128/138	172/172	138/147	207/219	156/156	293/293	300/300	171/182	177/183	187/202	210/233	214/216	142/142	121/126	175/186	169/183	174/188	184/198	185/185	176/176	152/160	160/161	197/213	113/137	180/180	202/202	250/250	111/111	315/319	209/232
218/223	154/161	99/105	280/280	128/129	172/172	141/144	207/216	162/168	293/293	278/303	162/171	174/183	187/187	213/222	216/216	142/142	126/126	186/186	169/183	174/188	184/198	185/188	176/179	145/161	161/173	197/213	113/113	180/186	202/210	244/244	73/111	319/321	209/209
212/212	154/161	99/105	298/298	128/128	163/172	141/144	207/219	162/168	293/293	278/300	162/182	174/183	187/187	208/209	216/216	142/142	126/126	186/186	183/183	188/188	184/198	185/188	176/179	145/160	160/161	197/197	113/137	163/186	202/202	250/251	111/111	315/322	232/232
208/212	161/161	99/99	298/298	128/128	172/172	141/141	216/219	168/168	293/296	278/285	182/182	183/183	187/208	208/208	216/216	130/142	121/126	175/186	165/183	184/188	198/198	185/192	164/179	152/160	160/161	197/197	134/137	163/172	202/202	250/251	111/111	315/322	230/232
218/218	161/161	99/105	280/298	128/138	172/172	138/144	207/207	156/162	293/293	278/300	162/171	177/183	187/202	210/218	216/216	142/142	121/126	175/186	169/183	174/188	184/184	185/185	176/179	145/152	161/173	197/213	113/128	165/180	202/202	244/244	73/111	319/321	209/213
210/212	154/161	99/105	298/298	128/128	172/172	141/141	204/216	168/168	293/293	278/285	171/171	171/183	208/216	208/208	216/216	142/142	121/126	175/186	183/183	192/192	174/198	185/188	179/179	145/145	160/161	197/197	113/134	172/186	202/202	250/251	111/111	316/322	230/232
210/212	161/161	99/105	280/298	128/128	nd.	141/141	207/219	168/168	293/296	278/300	162/182	183/183	187/208	208/208	216/216	142/142	121/126	175/186	183/183	188/192	184/198	185/188	176/179	145/160	160/163	197/197	113/137	163/186	202/202	250/251	111/111	315/322	232/232
210/210	161/161	99/105	280/298	128/128	163/172	141/141	216/219	168/168	293/293	278/285	171/182	171/183	208/211	208/208	216/216	142/142	121/126	175/186	183/183	184/192	198/198	185/188	179/179	160/175	160/160	164/197	113/119	180/186	202/202	250/251	111/111	316/322	230/232
208/208	161/161	99/99	280/298	128/128	172/172	141/141	216/216	162/168	293/293	285/285	171/182	171/183	208/216	208/208	216/216	130/142	126/126	186/186	165/165	184/184	198/198	192/192	164/179	152/152	160/161	197/197	134/134	172/172	202/202	251/251	111/111	315/315	230/231
212/218	154/161	99/105	280/298	128/138	172/172	138/141	207/207	162/168	293/293	278/300	162/171	174/183	187/202	209/222	216/216	142/142	121/126	175/186	169/183	174/188	184/184	185/185	176/179	145/161	161/179	197/209	113/113	177/186	202/210	250/250	73/111	315/319	213/232
208/212	161/161	99/105	298/298	128/128	172/172	141/141	204/216	168/168	293/293	285/300	179/182	174/183	208/241	208/208	216/216	142/142	121/126	175/186	183/183	177/192	174/198	173/185	176/179	145/161	160/160	197/197	113/113	186/186	202/202	250/252	111/111	315/316	230/232
208/210	161/161	99/99	280/280	128/138	163/172	138/141	204/219	162/168	293/296	300/300	182/182	183/183	187/208	208/218	216/216	130/142	121/126	175/186	183/183	188/188	198/198	185/188	176/179	160/175	160/160	197/197	113/137	163/186	202/202	250/250	111/111	315/315	232/232
210/212	161/161	99/105	298/298	128/128	163/172	141/141	207/216	168/168	293/293	278/285	171/171	171/183	187/216	208/208	216/216	130/142	126/126	186/186	183/183	188/192	184/198	185/188	179/179	145/152	160/161	197/197	119/137	163/180	202/202	252/252	111/111	316/322	230/232
218/218	154/161	105/105	280/280	128/128	163/172	141/144	207/221	156/162	293/293	278/279	156/162	171/174	187/199	203/209	216/216	142/142	126/126	175/186	183/183	185/188	184/184	188/188	176/177	143/145	172/179	197/207	113/113	180/186	202/210	244/249	111/111	316/319	209/227
210/212	161/161	99/105	280/298	128/128	163/172	141/141	207/219	168/168	293/293	300/300	182/182	183/183	187/208	208/208	216/216	142/142	121/126	175/186	183/183	188/188	184/198	185/185	179/179	160/161	160/160	197/197	113/137	163/186	202/202	250/251	111/111	315/315	232/232
208/212	161/161	105/105	298/298	128/128	163/172	141/141	204/207	168/168	293/293	278/300	162/171	183/183	187/208	208/208	216/216	142/142	121/126	175/186	183/183	188/192	174/184	185/185	176/179	145/160	161/163	197/197	113/137	163/186	202/202	250/251	111/111	315/322	232/232
210/212	161/161	99/105	280/298	128/128	163/172	141/141	207/219	162/168	293/296	278/300	182/182	183/183	187/208	208/218	216/216	142/142	121/126	175/186	183/183	188/192	184/198	185/188	179/179	160/160	160/160	197/197	137/137	163/163	202/202	250/251	111/111	315/322	232/232
212/212	161/161	99/99	280/298	128/128	172/172	141/141	204/216	168/168	293/293	285/300	171/182	183/183	187/187	208/208	216/216	130/142	126/126	186/186	183/183	188/192	198/198	185/188	176/179	152/160	160/161	197/197	119/137	163/180	202/208	250/252	111/111	315/316	230/232
208/212	161/161	99/105	298/298	128/128	172/172	141/144	204/207	168/168	293/293	278/300	182/182	183/183	187/208	208/208	216/216	142/142	121/126	175/186	183/183	188/192	174/184	185/188	176/179	160/161	160/160	197/197	113/137	163/180	202/202	250/251	111/111	315/322	232/232
208/212	154/161	99/99	298/298	128/128	172/172	141/141	204/219	168/168	293/293	285/300	171/182	171/171	187/208	208/208	216/216	130/142	126/126	186/186	165/183	184/192	174/198	188/192	179/179	152/161	160/160	197/197	134/137	163/172	202/202	250/251	111/111	315/315	231/232
210/220	161/161	99/105	298/298	128/128	172/172	141/141	204/219	168/168	293/296	278/285	179/182	171/171	187/208	208/208	216/216	142/142	126/126	186/186	165/183	184/192	174/198	188/188	164/179	152/175	160/160	197/197	113/119	180/186	202/202	250/252	111/111	322/322	230/232
210/212	161/161	99/99	280/298	128/138	163/172	141/141	207/216	162/168	293/296	297/300	162/182	183/183	208/208	208/208	216/216	130/142	121/121	175/175	165/183	184/188	184/198	185/185	179/179	160/175	160/163	164/197	113/137	163/186	202/202	250/250	111/111	315/318	232/232
210/212	161/161	99/105	280/298	128/128	163/172	141/141	207/216	168/168	293/296	285/300	171/182	183/183	187/216	208/208	216/216	130/142	126/126	186/186	183/183	188/192	184/198	185/188	176/179	160/161	160/161	197/197	113/137	163/186	202/202	250/250	111/111	315/316	230/232
208/218	154/161	99/105	280/298	128/129	172/172	138/141	216/219	156/162	293/293	278/285	162/171	177/183	202/208	210/211	216/216	130/142	121/126	175/186	165/165	184/185	192/198	182/182	185/185	152/153	161/163	164/197	84/128	163/165	202/202	249/249	73/111	315/319	213/232
210/212	161/161	99/105	280/298	128/128	172/172	138/141	204/219	168/168	293/293	285/300	182/182	183/183	187/187	208/208	216/216	142/142	121/126	175/186	183/183	188/192	198/198	185/185	176/179	145/160	160/160	197/197	119/137	163/180	202/202	250/252	111/111	315/316	230/232
208/212	161/161	99/105	298/298	128/128	163/172	141/141	204/219	162/168	293/293	278/300	182/182	183/183	187/208	208/208	216/216	142/142	121/126	175/186	183/183	188/188	174/198	185/188	179/179	145/160	160/160	197/197	113/113	186/186	202/202	250/251	111/111	315/322	232/232
212/223	154/161	99/105	280/298	128/128	163/172	138/141	207/207	162/168	293/293	278/278	162/182	171/183	187/202	208/209	216/216	142/142	121/126	175/186	169/183	174/188	184/184	185/188	176/176	145/152	160/179	197/213	113/137	163/186	202/208	244/244	111/111	319/321	209/209
210/220	161/161	99/99	280/298	128/128	163/172	141/141	204/204	162/168	293/296	278/300	182/182	174/183	187/208	208/218	216/216	142/142	121/126	175/186	183/183	188/192	174/198	185/188	176/176	160/161	160/160	197/197	113/137	163/177	202/202	251/252	111/111	315/321	209/232
208/212	161/161	99/105	298/298	128/138	172/172	138/141	204/219	168/168	293/293	278/297	182/182	183/183	187/208	208/208	216/216	142/142	121/126	175/186	183/183	188/192	174/198	185/188	176/179	145/160	160/160	197/197	113/137	163/186	202/202	250/251	111/111	318/322	232/232
208/210	161/161	99/99	280/298	128/128	172/172	141/141	216/219	162/168	293/296	285/300	171/182	183/183	187/216	208/208	216/216	130/142	126/126	186/186	165/183	184/192	198/198	188/192	164/176	145/152	160/160	197/197	113/134	172/186	202/202	251/251	111/111	315/315	231/232
208/212	161/161	99/105	280/298	128/128	172/172	138/141	204/216	162/168	293/293	278/285	182/182	183/183	208/216	208/208	216/216	142/142	126/126	186/186	165/183	184/192	174/198	185/192	164/179	145/152	160/161	197/197	134/137	163/172	202/202	251/251	111/111	315/322	230/232
208/212	161/161	105/105	298/298	128/128	172/172	141/141	204/216	162/168	293/293	278/300	182/182	183/183	187/208	208/218	216/216	142/142	126/126	186/186	165/183	184/188	174/198	188/188	176/179	152/160	160/161	197/197	113/134	172/186	202/202	250/251	111/111	315/322	232/232
208/218	154/154	99/105	280/298	128/128	163/172	138/141	219/219	162/168	293/293	278/300	171/171	171/183	187/202	208/209	216/216	142/142	121/126	175/186	165/183	184/188	192/198	182/185	179/185	152/152	161/162	164/197	113/137	163/180	202/202	251/255	111/111	315/319	213/232
208/212	161/161	99/99	280/298	128/138	163/172	141/141	204/204	162/168	296/296	300/300	182/182	183/183	187/208	208/218	216/216	130/142	121/121	175/175	183/183	188/188	174/198	185/185	179/179	160/175	160/160	197/197	113/113	186/186	202/202	250/250	111/111	315/315	232/232
208/208	161/161	99/105	298/298	128/138	163/172	141/141	207/219	168/168	293/293	278/300	182/182	174/183	187/187	208/208	216/216	142/142	126/126	186/186	183/183	188/192	184/198	185/188	179/179	152/152	160/161	197/197	137/137	163/163	202/202	251/251	111/111	315/322	232/232
218/218	154/161	99/99	280/280	128/138	172/172	141/141	207/207	162/162	293/293	278/278	162/162	177/183	202/211	218/218	216/216	142/142	121/121	175/175	169/169	174/174	184/184	185/185	176/176	145/161	163/179	209/213	113/113	177/180	210/210	244/244	73/73	319/319	213/213
208/210	161/161	99/105	280/298	128/128	163/172	138/141	204/219	168/168	293/293	278/300	171/179	174/183	187/187	208/208	216/216	142/142	126/126	186/186	165/183	184/188	174/198	185/188	176/179	152/160	161/161	197/197	119/137	163/180	202/202	250/252	111/111	315/322	232/232
208/210	161/161	99/105	298/298	128/128	163/172	141/141	204/207	168/168	293/293	278/285	171/182	183/183	187/211	208/208	216/216	130/142	126/126	186/186	165/183	184/188	174/184	185/188	179/179	152/175	160/161	164/197	113/137	163/186	202/202	250/250	111/111	315/322	230/232
210/210	154/161	99/105	280/298	128/128	163/163	141/141	207/216	168/168	293/293	278/282	182/182	183/183	187/211	208/208	216/216	142/142	126/126	186/186	183/183	184/188	184/198	185/185	179/179	145/160	160/161	164/197	134/137	163/172	202/202	250/251	111/111	316/322	230/232
208/210	154/161	99/99	298/298	128/128	163/172	141/141	204/216	168/168	293/293	285/285	182/182	171/174	216/241	208/208	216/216	142/142	126/126	186/186	165/183	184/192	198/198	173/188	176/179	161/175	160/161	164/197	113/134	172/186	202/202	251/252	111/111	316/316	230/230
210/210	154/161	99/99	280/298	128/128	172/172	141/141	204/219	168/168	293/293	285/300	171/182	183/183	208/211	208/208	216/216	142/142	126/126	186/186	183/183	188/192	198/198	185/188	179/179	145/161	160/160	164/197	113/119	180/186	202/202	251/251	111/111	315/316	230/232
210/212	161/161	99/99	298/298	128/128	163/172	141/141	216/216	168/168	293/293	278/285	171/182	183/183	208/216	208/208	216/216	130/142	121/126	175/186	183/183	192/192	198/198	185/185	176/179	152/161	160/161	197/197	113/119	180/186	202/202	252/252	111/111	315/322	232/232
212/220	161/161	99/99	298/298	128/128	163/163	141/141	207/216	162/168	293/296	278/300	179/182	174/183	208/208	208/218	216/216	142/142	121/126	175/186	183/183	177/192	184/198	173/188	176/179	160/161	160/160	164/197	113/137	163/186	202/202	250/250	111/111	315/322	232/232
210/210	161/161	99/105	280/298	128/128	172/172	141/141	204/219	168/168	293/296	282/300	182/182	171/183	187/216	208/208	216/216	142/142	126/126	186/186	165/183	184/192	198/198	185/192	164/179	152/160	160/160	197/197	119/137	163/180	202/202	250/250	111/111	315/316	230/232
210/220	154/154	99/99	298/298	128/128	163/163	141/141	204/219	168/168	293/293	285/285	179/182	171/171	187/208	208/208	216/216	142/142	121/126	175/186	165/183	188/192	174/198	188/192	164/179	145/161	160/161	197/197	113/137	163/186	202/202	250/252	111/111	315/322	232/232
212/212	161/161	99/105	280/298	128/128	163/172	141/141	204/219	162/168	293/293	278/300	182/182	183/183	208/241	208/218	216/216	142/142	121/126	175/186	183/183	188/192	174/198	185/188	179/179	145/160	160/160	197/197	113/137	163/186	202/208	250/251	111/111	315/322	232/232
210/212	161/161	99/105	298/298	128/128	163/172	141/141	204/207	168/168	293/293	278/285	162/182	183/183	208/216	208/208	216/216	130/142	126/126	186/186	183/183	192/192	184/198	185/185	176/179	145/145	161/163	164/197	113/119	180/186	202/202	250/251	111/111	316/322	230/232
210/212	161/161	99/99	298/298	128/128	172/172	141/141	207/216	168/168	293/296	285/300	182/182	183/183	208/216	208/208	216/216	142/142	121/126	175/186	165/183	184/192	184/198	185/185	179/179	160/175	160/161	164/197	119/137	163/186	202/202	250/252	111/111	316/322	230/232
210/220	154/154	99/99	298/298	128/128	163/172	141/141	204/219	168/168	293/293	285/300	182/182	174/183	187/208	208/208	216/216	142/142	121/126	175/186	165/183	184/192	174/198	188/188	176/179	145/161	161/161	197/197	113/137	163/186	202/202	250/252	111/111	315/322	232/232
210/212	161/161	99/99	280/298	128/128	172/172	141/141	207/216	168/168	293/296	278/300	179/182	183/183	187/211	208/208	216/216	142/142	126/126	186/186	183/183	184/188	184/198	173/188	176/179	160/161	160/160	164/197	113/137	163/186	202/202	250/250	111/111	315/322	232/232
210/212	154/161	99/105	298/298	128/128	163/163	141/141	204/207	168/168	293/293	285/300	182/182	171/183	187/211	208/208	216/216	142/142	126/126	186/186	183/183	184/188	184/198	185/188	179/179	152/175	160/161	197/197	119/137	163/180	202/202	250/251	111/111	315/316	230/232
212/212	161/161	99/105	280/298	128/128	172/172	141/141	207/219	168/168	293/293	278/300	182/182	183/183	208/241	208/208	216/216	142/142	121/121	175/175	169/183	177/188	184/198	185/185	179/179	160/161	160/160	197/197	113/137	163/186	202/208	251/251	111/111	315/322	232/232

210/212	161/161	99/99	280/298	128/128	172/172	141/144	204/216	162/168	293/293	285/300	171/179	183/183	208/208	208/218	216/216	130/142	121/121	175/175	183/183	192/192	174/198	185/188	176/176	145/152	160/160	197/197	119/130	180/180	202/202	251/252	111/111	315/322	232/232
210/212	161/161	99/105	280/298	128/138	172/172	141/144	204/219	168/168	293/293	297/300	162/182	174/183	187/187	208/208	216/216	130/142	121/126	175/186	183/183	188/192	174/198	185/188	176/179	145/161	160/161	197/197	113/137	163/186	202/202	251/251	111/111	318/322	232/232
210/212	161/161	99/105	280/298	128/138	163/172	141/141	216/219	162/168	293/293	278/300	182/182	177/183	208/235	208/218	216/216	111/142	121/126	175/186	183/183	177/188	198/198	185/185	179/179	145/145	160/160	197/197	113/137	163/177	202/202	250/251	111/111	315/322	232/232
208/210	154/161	99/99	280/298	128/138	163/172	141/141	204/216	168/168	293/293	278/285	162/171	171/183	187/187	208/208	216/216	130/142	121/126	175/186	165/183	184/188	198/198	188/188	176/179	152/161	161/163	164/197	113/119	180/186	202/202	251/252	111/111	316/318	232/232
210/212	161/161	99/99	298/298	128/128	172/172	141/141	204/216	168/168	293/296	278/285	179/182	183/183	187/211	208/208	216/216	142/142	126/126	186/186	165/183	184/188	198/198	173/188	176/179	152/161	160/160	164/197	119/137	163/180	202/202	250/252	111/111	316/322	230/232
210/212	161/161	99/99	280/298	128/128	172/172	141/141	216/219	162/168	293/296	285/300	171/179	177/183	208/208	208/218	216/216	142/142	121/126	175/186	165/183	184/192	198/198	185/185	176/179	160/161	160/161	164/197	113/137	163/186	202/202	250/252	111/111	315/315	232/232
210/210	154/161	99/99	280/298	128/138	172/172	141/141	216/216	168/168	293/296	278/285	171/182	171/183	187/208	208/208	216/216	142/142	121/126	175/186	183/183	192/192	198/198	185/188	179/179	145/175	160/160	197/197	113/134	172/186	202/202	250/250	111/111	318/322	232/232
210/210	161/161	99/99	280/298	128/128	172/172	141/141	216/219	162/168	293/296	278/282	162/182	171/183	208/216	208/218	216/216	130/142	121/126	175/186	183/183	192/192	198/198	185/188	179/179	145/175	161/163	197/197	113/134	172/186	202/202	251/251	111/111	316/322	230/232
210/212	161/161	99/99	298/298	128/128	172/172	141/141	204/219	162/168	293/296	285/300	162/171	183/183	187/216	208/218	216/216	130/142	126/126	186/186	183/183	192/192	198/198	185/188	176/179	160/175	160/163	197/197	134/137	163/172	202/202	251/251	111/111	315/316	230/232
208/210	161/161	99/105	280/298	128/138	163/172	141/141	204/219	162/168	293/293	297/300	182/182	183/183	208/211	208/218	216/216	130/142	121/126	175/186	183/183	188/192	174/198	185/188	176/179	160/175	160/160	197/197	113/137	163/186	202/202	250/251	111/111	315/318	232/232
210/220	161/161	99/105	280/298	128/128	163/172	141/141	204/207	168/168	293/293	278/300	182/182	177/183	187/208	208/208	216/216	142/142	126/126	186/186	165/183	184/188	184/198	185/188	179/179	160/161	160/160	164/197	113/137	163/186	202/202	250/252	111/111	316/322	230/232
210/212	161/161	99/105	280/298	128/138	163/163	141/141	204/207	168/168	293/293	285/300	182/182	183/183	187/208	208/208	216/216	142/142	121/126	175/186	183/183	188/192	184/198	185/185	179/179	152/175	160/160	197/197	113/137	163/177	202/202	250/251	111/111	315/315	230/232
210/210	161/161	99/105	280/298	128/128	172/172	138/141	204/204	168/168	293/293	278/300	162/179	183/183	187/211	208/208	216/216	142/142	126/126	186/186	183/183	188/192	174/198	173/188	176/176	145/161	160/163	197/197	113/137	163/186	202/202	250/251	111/111	315/322	232/232
210/220	161/161	99/99	280/298	128/138	172/172	138/141	204/204	168/168	293/293	278/300	162/182	183/183	208/211	208/208	216/216	142/142	121/121	175/175	169/183	174/192	174/198	185/188	176/179	145/145	160/164	197/213	113/130	180/186	202/208	250/250	111/111	315/322	232/232
210/212	161/161	99/105	280/298	128/138	172/172	138/141	204/204	168/168	293/293	285/300	171/182	183/183	211/211	208/208	216/216	142/142	121/126	175/186	169/183	174/192	174/198	185/188	176/179	145/175	160/161	197/213	113/130	180/186	202/202	250/251	111/111	315/322	232/232
208/208	154/161	99/99	298/298	128/128	163/172	141/141	204/219	168/168	293/296	278/300	171/182	183/183	187/208	208/208	216/216	142/142	121/126	175/186	183/183	188/192	174/198	185/185	179/179	145/160	160/161	197/197	113/137	163/186	202/202	250/250	111/111	315/322	232/232
208/210	161/161	99/105	298/298	128/138	172/172	138/141	204/207	168/168	293/293	278/297	182/182	183/183	187/211	208/208	216/216	142/142	121/126	175/186	165/183	184/188	174/184	185/185	179/179	160/175	160/160	164/197	113/137	163/186	202/202	250/250	111/111	318/322	232/232
210/212	154/161	99/99	280/298	128/138	163/172	141/141	204/219	168/168	293/293	278/297	182/182	183/183	187/208	208/208	216/216	130/142	126/126	186/186	183/183	188/192	174/198	185/188	179/179	152/175	160/160	197/197	137/137	163/163	202/202	250/251	111/111	318/322	232/232
208/218	154/161	99/105	298/298	128/128	163/172	141/141	204/219	156/168	293/293	297/300	162/171	171/183	202/208	208/210	216/216	142/142	121/121	175/175	165/183	184/192	174/192	182/188	176/185	152/161	162/163	164/197	113/128	165/186	202/202	250/250	73/111	318/319	213/232
208/218	154/161	99/105	280/298	128/138	163/172	141/141	204/207	162/168	293/296	278/297	162/171	171/183	187/202	208/209	216/216	142/142	121/126	175/186	165/183	184/188	174/184	182/188	176/185	145/161	161/163	164/197	113/128	165/186	202/202	250/250	73/111	315/319	213/232
210/218	154/161	99/105	280/298	128/138	163/172	141/141	204/219	156/168	293/293	278/300	162/171	183/183	187/202	208/210	216/216	142/142	121/126	175/186	165/183	184/192	174/192	185/188	176/176	145/161	162/163	164/197	113/113	180/186	202/202	251/251	73/111	315/319	213/232
218/220	161/161	99/99	298/298	128/128	163/172	141/144	207/216	168/168	293/296	278/300	171/179	177/183	202/235	208/213	216/216	142/142	126/126	186/186	169/183	174/177	184/198	185/185	176/179	145/161	160/161	197/213	113/113	177/186	202/202	250/250	111/111	315/319	209/232
210/220	161/161	99/99	280/298	128/128	163/172	141/141	204/204	162/168	293/293	285/300	182/182	174/183	208/235	208/218	216/216	130/142	121/126	175/186	165/183	184/192	174/198	188/188	176/179	145/161	160/160	164/197	113/137	163/177	202/202	250/250	111/111	315/315	230/232
210/212	161/161	99/105	280/298	128/128	163/172	141/141	207/219	168/168	293/293	297/300	162/182	174/183	187/187	208/208	216/216	142/142	126/126	186/186	183/183	188/192	184/198	185/188	176/179	145/160	160/161	197/197	113/137	163/186	202/202	250/251	111/111	315/318	232/232
212/218	161/161	99/105	280/298	128/128	172/172	138/144	207/219	168/168	293/296	278/278	171/182	183/183	187/187	208/213	216/216	142/142	126/126	186/186	183/183	188/188	184/198	185/185	179/179	145/145	160/161	197/197	113/137	163/186	202/210	251/251	111/111	319/322	209/232
212/220	161/161	99/99	280/298	128/128	163/172	141/141	204/207	162/162	293/293	300/300	179/182	174/177	187/241	208/218	216/216	142/142	126/126	186/186	183/183	177/188	174/184	185/188	176/179	145/161	160/160	197/197	113/113	177/177	202/208	252/252	111/111	315/315	232/232
212/218	154/161	99/99	280/298	128/128	163/172	141/144	207/219	162/168	293/296	278/300	162/171	174/183	187/187	208/209	216/216	142/142	126/126	186/186	183/183	188/188	184/198	188/188	176/176	145/161	161/161	197/197	113/113	177/186	202/202	251/251	111/111	315/319	209/232
212/220	161/161	99/99	298/298	128/128	163/172	138/141	204/207	168/168	293/293	278/300	171/179	174/174	202/208	208/213	216/216	142/142	126/126	186/186	169/183	174/177	184/198	173/185	176/179	161/161	160/161	164/213	113/113	186/186	202/202	250/252	111/111	315/316	230/232
212/218	154/161	99/105	280/298	128/128	172/172	141/144	207/207	162/162	293/293	278/278	162/171	174/177	187/202	209/218	216/216	142/142	121/126	175/186	169/183	174/188	184/184	185/188	176/176	145/145	161/179	197/209	113/113	177/186	202/210	244/244	73/111	319/319	209/213
210/212	161/161	99/105	280/298	128/128	172/172	141/144	204/207	168/168	293/293	297/300	162/182	174/183	187/208	208/213	216/216	142/142	121/126	175/186	183/183	188/188	174/184	185/188	176/176	145/161	160/179	197/213	113/113	180/186	202/202	250/250	111/111	315/318	232/232
208/210	161/161	99/105	280/298	128/128	172/172	141/141	204/216	168/168	293/293	278/285	162/182	183/183	208/208	208/208	216/216	142/142	126/126	186/186	165/183	184/188	174/198	188/192	164/176	152/160	161/163	197/197	134/137	163/172	202/202	250/251	111/111	315/322	231/232
208/208	161/161	99/105	298/298	128/138	163/172	141/141	204/219	168/168	293/293	278/300	171/182	183/183	187/187	208/208	216/216	142/142	126/126	186/186	183/183	188/192	174/198	185/188	176/179	152/161	160/161	197/197	113/137	163/186	202/202	250/251	111/111	318/322	232/232
208/210	161/161	99/99	280/298	128/128	172/172	141/141	204/216	162/162	293/296	285/300	171/182	171/183	208/208	208/218	216/216	130/142	121/126	175/186	165/183	184/192	174/198	188/192	176/179	145/152	160/160	197/197	113/134	172/186	202/202	251/251	111/111	315/315	231/232
208/212	161/161	99/105	298/298	128/128	172/172	138/141	204/216	162/168	293/293	278/285	182/182	171/183	187/216	208/218	216/216	130/142	126/126	186/186	165/183	184/188	174/198	188/192	164/176	145/152	160/161	197/197	113/134	172/186	202/202	250/251	111/111	315/322	231/232
208/212	161/161	nd.	nd.	nd.	163/172	141/141	207/219	168/168	293/293	300/300	182/182	183/183	187/208	208/208	216/216	142/142	121/126	175/186	183/183	188/188	184/198	185/185	179/179	145/160	160/160	197/197	113/137	163/186	202/202	250/251	111/111	315/322	232/232
210/212	161/161	99/105	280/298	128/128	172/172	138/144	204/204	162/168	293/293	278/300	182/182	183/183	187/208	208/218	216/216	142/142	121/126	175/186	183/183	188/192	174/174	185/188	176/179	145/161	160/160	197/197	137/137	163/163	202/202	250/250	111/111	315/322	232/232
210/212	161/161	99/99	298/298	128/128	163/172	141/147	204/204	168/168	293/296	285/300	182/182	183/183	187/208	208/208	216/216	142/142	121/126	175/186	183/183	188/192	174/198	185/185	179/179	160/175	160/160	197/197	113/137	163/186	202/202	251/252	111/111	315/316	230/232
208/208	161/161	99/105	298/298	128/128	172/172	141/141	219/219	168/168	293/293	278/285	171/182	183/183	187/187	208/208	216/216	130/142	126/126	186/186	183/183	188/192	198/198	185/192	164/179	145/160	160/160	197/197	113/113	186/186	202/202	251/251	111/111	315/322	231/232
208/212	161/161	99/105	280/298	128/128	172/172	141/144	204/207	168/168	nd.	278/285	182/182	183/183	187/208	208/208	216/216	142/142	121/126	175/186	183/183	192/192	184/198	185/188	176/179	161/175	160/160	197/197	113/137	163/186	202/202	251/251	111/111	316/322	230/232
210/218	161/161	99/105	280/280	128/138	172/172	141/141	207/219	162/168	293/296	278/278	162/182	183/183	202/208	208/218	216/216	142/142	121/121	175/175	169/183	174/188	198/198	185/185	176/179	145/145	160/179	197/209	113/137	163/180	202/210	250/250	111/111	319/322	209/232
208/210	154/161	99/105	280/298	128/138	163/172	141/141	204/219	168/168	293/293	278/300	171/182	171/183	187/208	208/208	216/216	130/142	121/126	175/186	183/183	188/192	174/198	188/188	176/179	160/161	160/160	197/197	113/134	172/186	202/202	250/251	111/111	315/315	231/232
208/210	154/161	99/99	280/298	128/128	163/172	141/141	204/219	162/162	293/296	285/300	182/182	183/183	208/241	218/221	216/216	130/142	126/126	186/186	183/183	177/188	198/198	185/188	176/179	160/161	160/160	197/197	113/113	186/186	202/202	250/252	111/111	315/316	230/232
218/218	161/161	99/105	280/280	138/138	163/172	138/141	204/207	162/168	293/296	278/278	162/182	171/183	187/202	208/218	216/216	142/142	121/128	175/175	169/183	174/192	174/174	185/185	176/177	145/145	160/164	197/209	113/113	180/180	202/210	250/250	73/111	319/319	209/209
212/218	154/161	99/105	280/298	128/138	163/172	138/141	207/221	162/168	293/293	278/278	162/182	183/183	199/202	208/218	216/216	142/142	121/128	175/175	169/183	174/185	nd.	185/185	176/176	145/145	160/164	207/209	113/113	180/186	202/210	250/250	73/111	322/322	213/232
208/210	154/161	99/105	280/298	128/138	163/172	141/141	204/207	168/168	293/293	278/300	182/182	171/183	208/208	208/208	216/216	142/142	121/126	175/186	165/183	184/188	174/184	188/188	176/179	152/160	160/160	197/197	113/137	163/186	202/202	250/251	111/111	315/315	232/232
208/212	161/161	99/105	298/298	128/128	163/172	144/147	204/207	168/168	293/293	278/300	179/182	183/183	208/208	208/208	216/216	142/142	121/121	175/175	183/183	192/192	184/198	185/188	176/179	160/160	160/160	197/197	113/137	163/186	202/202	250/251	111/111	315/322	232/232
208/210	161/161	99/99	280/298	128/128	163/172	138/141	204/219	168/168	293/296	297/300	162/182	171/183	208/220	208/208	216/216	142/142	121/127	175/175	183/183	188/192	174/198	185/185	177/179	145/145	160/163	197/197	113/137	163/180	202/202	251/251	111/111	315/318	232/232
210/218	161/161	99/105	280/298	128/128	163/172	141/141	219/219	156/162	293/293	278/300	182/182	183/183	205/208	210/218	216/216	130/142	97/121	175/180	183/183	177/192	192/198	185/188	176/176	160/161	160/160	164/197	113/128	165/186	202/202	250/250	111/111	315/322	230/232
208/212	161/161	99/99	280/298	138/138	163/172	138/141	204/216	162/168	293/296	300/300	162/182	183/183	187/208	208/218	216/216	142/142	121/126	175/186	183/183	188/192	198/198	185/185	176/179	152/160	160/163	197/197	113/137	163/186	202/202	250/250	111/111	315/315	232/232
212/218	161/161	99/105	280/298	128/128	163/172	141/141	207/207	162/168	293/293	278/278	162/171	174/183	187/211	208/218	216/216	142/142	121/126	175/186	169/183	174/188	184/184	185/188	176/179	152/161	161/164	197/209	113/137	163/180	202/202	251/251	111/111	322/322	209/232
208/210	161/161	99/105	280/298	128/128	163/172	141/141	204/207	168/168	293/293	278/300	182/182	183/183	208/208	208/208	216/216	142/142	121/126	175/186	183/183	188/192	174/184	185/185	179/179	160/175	160/160	197/197	113/137	163/186	202/202	251/252	111/111	315/316	230/232
208/212	161/161	99/105	298/298	128/138	163/172	141/141	204/207	168/168	293/293	285/300	182/182	183/183	187/208	208/208	216/216	142/142	121/126	175/186	165/183	184/188	184/198	185/188	176/179	160/160	160/160	164/197	113/113	186/186	202/202	250/252	111/111	315/316	230/232
208/212	161/161	99/99	298/298	128/128	172/172	141/141	204/219	168/168	293/296	278/285	162/179	183/183	187/208	208/208	216/216	142/142	126/126	186/186	183/183	188/192	174/198	185/185	179/179	145/160	160/163	164/197	113/137	163/186	202/202	250/252	111/111	316/322	230/232

96	97	98	99	100	101	102	103	104	105	106	107	108	109	110	111	112	113	114	115	116	117	118	119	120	121	122	123	124	125	126	127	128	129
*			*			*			*	*	*		*	*	*	*	*		*		*	*			*	*		*		*	*		*
NSX70	NSX87	NSX94	NSX118	NSX129	NSX148	NSX161	NSX179	TSA10A29	SSR07A05	SSR07A07	SSR07A14	SSR07A17	SSR07A19	SSR07A23	SSR07A24	SSR07A20	SSR07B01	SSR07B05	SSR07B08	SSR07B09	SSR07B14	SSR07B27	SSR07B25	SSR07B30	SSR08A02	SSR08A06	SSR08A07	SSR08A09	SSR08A14	SSR08A15	SSR08A16	SSR08B02	SSR08B06
0	18	1	0	0	0	0	0	5	1	0	0	0	2	0	0	0	1	0	1	0	0	1	1	0	0	0	0	0	0	0	0	3	0
129/129	128/128	114/116	128/138	216/216	149/149	113/113	250/250	216/216	134/140	171/174	139/145	231/231	127/132	164/182	147/156	195/201	130/135	164/164	190/193	204/204	205/205	191/191	199/211	180/184	151/151	124/128	106/114	94/102	171/179	132/135	138/141	119/122	115/121
129/129	nd.	114/114	128/128	216/216	149/149	113/113	259/259	212/213	134/142	177/180	139/145	219/231	127/127	164/164	156/156	201/201	130/135	176/176	190/193	204/210	205/211	191/191	194/194	184/184	151/151	120/124	114/118	94/102	171/179	132/132	138/138	119/119	115/115
129/137	128/128	115/123	128/138	207/216	149/149	113/113	225/250	216/216	134/140	171/174	139/145	219/231	127/132	164/164	147/156	198/200	130/135	164/176	190/193	204/210	208/211	191/197	199/211	180/184	151/151	120/124	106/122	102/114	171/179	132/135	138/141	119/131	115/121
129/129	nd.	114/114	128/128	216/216	137/149	113/113	259/259	213/213	134/134	174/177	145/145	219/219	127/127	164/164	156/156	201/201	130/130	164/164	190/190	210/211	211/211	191/191	194/194	184/184	151/151	124/124	114/118	102/102	171/171	132/138	138/144	131/131	115/115
129/129	128/138	114/116	128/128	207/216	149/149	99/113	250/259	211/216	131/131	171/171	139/139	231/231	132/132	182/182	147/153	195/198	131/135	164/176	193/193	205/205	205/211	185/191	203/205	178/186	151/151	120/120	106/106	94/102	179/179	135/135	141/141	119/119	115/121
129/129	nd.	135/135	128/128	207/216	149/149	113/113	259/259	213/213	134/142	160/171	139/145	216/219	127/127	182/182	147/156	195/200	130/131	176/176	190/190	204/210	205/205	185/197	194/194	186/195	151/155	124/124	118/122	94/106	171/179	132/132	138/138	116/122	118/121
129/137	128/128	114/123	128/138	207/215	149/149	113/113	250/259	216/216	134/140	171/171	139/139	231/231	127/132	164/182	147/153	198/200	133/135	164/176	190/193	204/204	205/211	176/191	199/199	180/186	151/151	120/120	106/114	94/102	171/179	135/138	141/144	119/122	115/121
129/129	128/128	114/126	128/138	216/216	137/149	113/113	259/259	216/221	131/131	171/180	139/139	231/231	127/132	182/182	153/156	198/198	130/135	164/164	187/190	204/204	205/217	185/191	198/209	186/186	151/151	120/124	106/106	94/102	179/179	135/138	141/144	119/122	115/121
129/129	128/128	114/114	138/138	207/216	149/149	99/99	250/250	211/221	131/140	171/171	139/139	231/231	132/132	182/183	147/153	198/200	131/133	176/176	190/193	204/205	205/211	185/188	205/209	184/186	151/151	120/128	106/106	94/102	179/179	135/138	141/144	122/131	121/121
129/129	128/128	114/114	138/138	207/216	149/149	99/99	250/250	211/221	131/140	171/171	139/139	231/231	132/132	182/183	147/153	198/200	131/133	176/176	190/193	204/205	205/211	185/188	205/209	184/186	151/151	120/128	106/106	94/102	179/179	135/138	141/144	122/131	121/121
129/129	128/128	114/114	138/138	207/216	149/149	99/99	250/250	211/221	131/140	171/171	139/139	231/231	132/132	182/183	147/153	198/200	131/133	176/176	190/193	204/205	205/211	185/188	205/209	184/186	151/151	120/128	106/106	94/102	179/179	135/138	141/144	122/131	121/121
129/129	128/128	114/114	138/138	207/216	149/149	99/99	250/250	211/221	131/140	171/171	139/139	231/231	132/132	182/183	147/153	198/200	131/133	176/176	190/193	204/205	205/211	185/188	205/209	184/186	151/151	120/128	106/106	94/102	179/179	135/138	141/144	122/131	121/121
129/129	128/128	114/114	128/128	207/207	149/149	99/113	250/250	211/216	131/140	171/171	139/139	228/231	132/132	182/182	147/147	192/198	131/131	176/176	193/193	204/204	205/205	185/191	201/209	180/189	151/151	120/128	106/106	94/94	179/179	135/135	141/141	122/122	121/121
129/129	128/138	114/116	138/138	207/216	149/149	99/113	250/250	211/221	137/140	165/171	139/139	231/231	132/132	182/183	147/147	198/200	133/135	176/176	190/193	204/205	205/211	182/188	205/209	186/186	151/151	120/120	106/118	94/114	171/179	135/135	141/141	119/119	121/121
129/129	128/128	114/114	128/128	216/216	149/149	113/113	259/259	211/211	128/134	171/180	139/145	219/220	127/127	164/182	147/156	195/200	130/131	164/164	187/190	204/210	205/208	185/191	194/203	184/184	151/151	124/128	110/118	94/102	171/179	135/136	141/142	122/122	115/121
129/129	128/138	114/116	128/128	207/207	149/149	99/99	250/250	216/221	131/131	171/171	139/139	231/231	132/132	182/182	147/147	195/195	131/135	176/176	190/193	204/204	205/205	191/191	199/205	180/186	151/151	120/128	106/106	94/102	179/179	135/135	141/141	122/131	115/121
129/129	128/138	114/116	128/128	207/207	149/149	99/99	250/250	216/221	131/131	171/171	139/139	231/231	132/132	182/182	147/147	195/195	131/135	176/176	190/193	204/204	205/205	191/191	199/205	180/186	151/151	120/128	106/106	94/102	179/179	135/135	141/141	122/131	115/121
129/129	nd.	114/114	128/128	215/215	149/149	113/113	259/259	212/213	134/137	174/180	145/145	219/219	127/127	164/164	156/156	204/204	130/130	164/164	190/190	210/210	205/205	188/188	194/194	184/184	151/151	124/124	114/114	102/114	171/171	132/132	138/138	119/119	112/115
129/129	128/128	123/126	128/138	207/207	149/149	113/113	250/250	216/216	131/134	171/188	139/139	208/231	127/132	182/182	147/147	198/198	131/135	164/164	187/193	204/205	205/214	185/191	198/199	186/196	151/151	120/120	106/110	94/94	179/179	135/135	141/141	119/122	115/121
129/129	128/128	123/135	128/128	207/216	149/149	113/113	259/259	216/216	128/142	165/171	139/139	219/231	127/127	182/182	147/156	198/200	131/131	164/164	187/190	203/204	211/214	185/197	194/201	186/186	151/151	120/128	110/118	94/106	171/179	132/135	138/141	122/131	115/115
129/137	128/138	116/123	128/128	207/216	149/149	99/113	250/259	211/211	131/134	171/171	139/139	219/231	127/132	164/182	153/156	195/200	130/131	164/176	193/193	204/205	205/211	191/191	203/203	180/186	151/151	120/124	106/114	102/102	171/179	135/135	141/141	122/131	115/115
129/129	128/138	116/123	128/128	207/216	149/149	99/99	250/250	221/221	131/140	171/171	139/139	231/231	127/132	182/182	147/147	195/198	131/133	176/176	190/199	204/204	205/205	185/191	199/199	180/186	151/151	128/128	106/106	94/94	179/179	135/135	141/141	122/122	121/121
129/129	128/128	115/132	128/138	207/216	149/149	113/113	250/259	216/221	131/134	165/188	139/139	220/231	121/127	182/182	147/156	195/198	131/133	167/176	187/190	204/204	205/214	185/191	198/209	178/186	151/151	120/128	106/110	94/102	179/179	132/135	138/141	122/131	115/121
129/129	138/138	116/131	128/138	216/216	137/149	99/113	250/259	221/221	134/137	171/177	139/145	219/219	127/127	164/182	153/156	200/201	130/135	176/176	190/193	204/210	205/208	188/188	209/211	184/186	151/151	120/124	118/118	102/114	171/171	132/135	138/141	119/122	115/121
129/129	138/138	116/131	128/138	216/216	137/149	99/113	250/259	221/221	134/137	171/177	139/145	219/219	127/127	164/182	153/156	200/201	130/135	164/176	190/193	204/210	205/208	188/188	209/211	184/186	151/151	120/124	118/118	102/114	171/171	132/135	138/141	119/122	115/121
129/129	138/138	116/131	128/138	216/216	137/149	99/113	250/259	221/221	134/137	171/177	139/145	220/220	127/127	164/182	153/156	200/201	130/135	164/176	190/193	204/210	205/208	188/188	209/211	184/186	151/151	120/124	118/118	102/114	171/171	132/135	138/141	119/122	115/121
129/129	128/128	114/123	128/128	207/216	149/149	113/113	259/259	211/215	128/131	162/171	139/139	220/231	121/127	182/184	147/147	195/195	130/131	162/164	187/190	204/210	205/211	185/188	190/203	186/196	151/151	128/128	106/110	94/110	175/179	132/135	138/141	119/131	118/121
129/137	128/128	114/123	128/138	207/216	149/149	113/113	250/259	216/216	134/134	171/177	139/145	219/231	127/132	173/182	147/156	198/201	130/135	164/164	190/193	204/210	211/211	191/191	194/199	180/184	151/151	120/124	114/114	102/102	171/171	132/135	138/141	122/131	115/121
129/137	128/128	123/123	128/128	215/215	149/149	113/113	259/259	211/211	131/140	171/171	139/139	231/231	127/132	182/193	153/153	195/198	130/135	162/176	190/190	204/219	205/208	188/191	203/203	180/184	151/151	120/120	106/106	94/102	171/179	132/135	138/141	116/119	112/115
129/137	128/128	123/123	128/128	215/215	149/149	113/113	250/259	215/215	131/140	168/171	139/139	231/231	127/127	182/193	147/147	195/198	130/133	164/176	190/193	204/219	205/208	191/191	190/190	180/184	151/151	120/120	106/106	94/110	171/179	132/135	138/141	116/119	115/118
129/129	138/138	116/116	128/128	207/216	149/149	113/113	250/259	221/221	131/140	171/171	139/139	217/231	127/132	182/182	147/147	195/195	131/135	176/176	190/193	204/204	205/205	188/191	194/199	180/186	151/151	120/128	106/106	94/102	179/179	135/138	141/144	119/122	115/121
129/129	128/128	114/115	128/128	216/216	149/149	113/113	259/259	214/214	137/137	174/177	145/145	219/219	127/127	164/164	156/156	201/204	130/130	164/164	190/190	210/210	205/208	191/191	211/211	184/184	151/151	124/124	118/122	102/102	171/171	132/132	138/138	131/131	115/115
129/129	128/128	114/123	128/138	207/216	149/149	113/113	225/259	216/216	131/134	160/180	139/145	219/231	127/127	173/182	147/156	195/204	130/130	164/164	190/190	204/210	205/211	185/188	207/211	184/184	151/151	120/124	106/118	94/102	171/179	132/135	138/141	122/131	115/121
129/137	128/128	114/123	128/138	207/216	149/149	113/113	250/259	216/216	134/140	171/171	139/139	231/231	127/132	164/182	147/153	198/201	133/135	164/164	190/193	204/205	205/211	185/188	194/199	184/184	151/151	120/120	106/118	94/102	171/179	132/135	138/141	122/122	115/121
129/137	128/128	114/123	128/128	215/216	149/149	113/113	225/250	nd.	134/140	171/177	139/145	220/231	127/127	164/182	153/156	198/201	130/135	164/176	190/190	204/210	205/208	191/191	194/211	184/186	151/151	120/124	106/114	94/102	171/179	132/135	138/141	119/119	115/115
129/137	128/128	116/123	128/128	207/215	149/149	113/113	250/259	211/211	134/134	171/180	139/139	231/231	121/127	164/182	147/153	195/201	131/135	167/167	187/190	204/204	211/214	182/191	203/211	178/178	151/151	120/128	110/114	94/114	171/179	135/135	141/141	119/122	115/121
129/137	128/128	116/123	128/128	207/215	149/149	113/113	250/259	211/211	134/134	171/180	139/139	231/231	121/127	164/182	147/153	195/201	131/135	167/167	187/190	204/204	211/214	182/191	203/211	178/178	151/151	120/128	110/114	94/114	171/179	135/135	141/141	119/122	115/121
129/137	128/128	116/123	128/128	207/215	149/149	113/113	250/259	211/211	134/134	171/180	139/139	231/231	121/127	164/182	147/153	195/201	131/135	167/167	187/190	204/204	211/214	182/191	203/211	178/178	151/151	120/128	110/114	94/114	171/179	135/135	141/141	119/122	115/121
129/137	128/128	116/123	128/128	207/215	149/149	113/113	250/259	211/211	134/134	171/180	139/139	231/231	121/127	164/182	147/153	195/201	131/135	167/167	187/190	204/204	211/214	182/191	203/211	178/178	151/151	120/128	110/114	94/114	171/179	135/135	141/141	119/122	115/121
129/137	128/128	116/123	128/128	207/215	149/149	113/113	250/259	211/211	134/134	171/180	139/139	231/231	121/127	164/182	147/153	195/201	131/135	164/167	187/190	204/204	211/214	182/191	203/211	178/178	151/151	120/128	110/114	94/114	171/179	135/135	141/141	119/122	115/121
129/129	nd.	116/135	128/138	207/216	149/149	113/113	259/259	210/216	131/134	160/160	139/139	216/225	127/127	182/193	147/147	195/195	130/131	162/176	187/190	204/204	205/208	185/194	190/207	182/186	151/151	120/120	106/122	94/106	171/179	132/133	138/139	119/131	121/121
129/129	138/138	114/123	128/138	216/216	149/149	113/113	259/259	211/211	131/134	160/160	139/145	220/231	127/127	164/184	156/156	195/201	130/130	164/176	190/190	210/210	211/211	188/191	194/203	184/186	151/151	124/124	106/114	94/102	171/171	132/136	138/142	116/119	112/115
129/129	138/138	114/116	128/138	207/215	149/149	99/113	250/250	216/216	nd.	171/171	139/145	231/231	132/132	164/182	147/153	195/201	130/131	176/176	190/193	204/211	205/205	191/191	199/205	180/184	151/151	124/128	106/118	94/102	171/179	132/135	138/141	119/131	115/121
129/129	138/138	114/116	128/138	207/215	149/149	99/113	250/250	216/216	131/134	171/171	139/145	231/231	132/132	164/182	147/153	195/201	130/131	176/176	190/193	204/211	205/205	191/191	199/205	180/184	151/151	124/128	106/118	94/102	171/179	132/135	138/141	119/131	115/121
129/129	nd.	114/123	138/138	207/216	149/149	113/113	259/259	211/216	131/140	171/171	139/139	231/231	127/127	182/193	147/156	195/198	131/133	162/176	190/190	204/219	205/208	188/191	199/203	184/186	151/151	120/120	106/106	94/102	171/179	132/138	138/144	116/119	118/121
129/129	128/128	114/116	128/138	216/216	149/149	113/113	250/259	211/211	131/134	162/180	139/145	219/231	127/127	164/184	156/156	195/198	130/133	162/164	190/190	210/219	205/211	188/191	194/203	186/186	151/151	124/128	106/118	94/102	171/175	135/138	141/144	119/131	112/115
129/129	128/128	114/116	128/128	215/215	149/149	113/113	250/259	215/215	131/140	171/171	139/139	231/231	127/132	164/184	153/153	195/200	130/135	176/176	190/190	204/219	211/211	176/191	190/190	186/186	151/151	120/128	106/114	94/102	171/171	135/138	141/144	116/119	115/118
129/137	128/128	115/123	128/138	215/215	149/149	113/113	259/259	216/216	134/134	165/171	139/139	231/231	127/132	164/182	153/156	198/198	131/135	164/176	187/190	204/204	205/205	185/191	199/203	180/196	151/151	120/128	110/114	94/102	171/179	135/135	141/141	122/122	115/121
129/137	128/128	114/123	128/138	215/215	149/149	113/113	259/259	216/216	134/134	171/174	139/145	219/231	127/132	164/164	153/156	198/201	130/135	164/176	190/193	204/211	205/211	191/191	199/211	180/184	151/151	120/124	114/118	102/102	171/171	132/135	138/141	110/110	115/121
129/129	128/128	123/126	128/128	215/215	149/149	113/113	250/259	212/214	134/134	171/180	139/139	231/231	127/132	164/182	153/156	195/198	130/135	164/164	190/193	204/204	205/205	182/191	211/211	180/186	151/151	120/124	110/114	94/102	171/179	135/135	141/141	122/122	115/115
129/129	nd.	114/114	128/128	216/216	149/149	113/113	259/259	213/214	134/134	171/177	139/145	219/231	127/127	164/182	153/156	198/201	130/135	164/164	190/193	204/210	205/205	185/194	194/211	184/184	151/151	120/124	114/118	94/114	171/171	132/132	138/138	122/122	115/115
137/137	128/128	114/123	128/138	207/216	149/149	113/113	250/259	216/216	134/134	165/171	139/139	231/231	127/132	164/182	147/156	198/198	133/135	164/164	190/190	204/204	205/211	185/191	199/203	180/186	151/151	120/120	110/114	94/102	171/179	135/138	141/144	122/131	115/115
129/129	128/128	114/114	128/128	207/216	149/149	113/113	259/259	212/215	131/134	162/177	139/139	220/220	121/132	164/182	147/147	195/204	130/131	162/164	190/190	204/210	205/214	185/191	190/194	186/186	151/151	124/128	110/122	94/114	171/179	135/138	141/144	122/122	115/118
129/129	128/138	116/123	138/138	207/216	149/149	113/113	250/250	216/221	131/131	171/171	139/139	222/231	127/135	164/182	147/153	198/198	131/133	176/176	190/193	204/204	205/211	185/191	207/209	186/186	151/151	120/128	106/106	94/102	179/179	135/135	141/141	116/122	115/121
129/137	128/128	123/135	128/128	215/215	149/149	113/113	259/259	nd.	134/140	171/177	139/145	219/231	127/132	164/182	153/156	198/201	130/135	164/164	190/190	204/210	205/208	191/191	194/194	180/184	151/151	120/124	106/114	94/114	171/179	135/135	141/141	119/119	115/115
129/129	128/128	114/116	128/138	216/216	149/149	113/113	250/250	216/216	134/140	171/174	139/145	231/231	127/132	164/182	147/156	195/201	130/135	164/164	190/193	204/204	205/205	191/191	199/211	180/184	151/151	124/128	106/114	94/102	171/179	132/135	138/141	122/131	115/121
129/137	128/128	116/123	128/138	207/216	149/149	113/113	250/259	216/216	131/140	171/171	139/139	208/231	127/132	182/182	147/147	195/198	133/135	164/176	193/193	204/205	205/205	185/191	199/203	180/186	151/151	120/128	106/106	94/94	179/179	135/135	141/141	119/122	115/121
129/137	128/128	116/123	128/138	207/216	149/149	113/113	250/259	216/216	131/140	171/171	139/139	208/231	127/132	182/182	147/147	195/198	133/135	164/176	193/193	204/205	205/205	185/191	199/203	180/186	151/151	120/128	106/106	94/94	179/179	135/135	141/141	119/122	115/121
129/137	128/128	116/123	128/138	207/216	149/149	113/113	250/259	216/216	131/140	171/171	139/139	208/231	127/132	182/182	147/147	195/198	133/135	164/176	193/193	204/205	205/205	185/191	199/203	180/186	151/151	120/128	106/106	94/94	179/179	135/135	141/141	119/122	115/121
129/137	128/128	116/123	128/138	207/216	149/149	113/113	250/259	216/216	131/140	171/171	139/139	208/231	127/132	182/182	147/147	195/198	133/135	164/176	193/193	204/205	205/205	185/191	199/203	180/186	151/151	120/128	106/106	94/94	179/179	135/135	141/141	119/122	115/121
129/137	128/128	116/123	128/138	207/216	149/149	113/113	250/259	216/216	131/140	171/171	139/139	208/231	127/132	182/182	147/147	195/198	133/135	164/176	193/193	204/205	205/205	185/191	199/203	180/186	151/151	120/128	106/106	94/94	179/179	135/135	141/141	119/122	115/121
129/137	128/128	116/123	128/138	207/216	149/149	113/113	250/259	216/216	131/140	171/171	139/139	208/231	127/132	182/182	147/147	195/198	133/135	164/176	193/193	204/205	205/205	185/191	199/203	180/186	151/151	120/128	106/106	94/94	179/179	135/135	141/141	119/122	115/121
129/137	128/128	116/123	128/138	207/216	149/149	113/113	250/259	216/216	131/140	171/171	139/139	208/231	127/132	182/182	147/147	195/198	133/135	164/176	193/193	204/205	205/205	185/191	199/203	180/186	151/151	120/128	106/106	94/94	179/179	135/135	141/141	119/122	115/121
129/137	128/128	116/123	128/138	207/216	149/149	113/113	250/259	216/216	131/140	171/171	139/139	208/231	127/132	182/182	147/147	195/198	133/135	164/176	193/193	204/205	205/205	185/191	199/203	180/186	151/151	120/128	106/106	94/94	179/179	135/135	141/141	119/122	115/121
129/137	128/128	116/123	128/138	207/216	149/149	113/113	250/259	216/216	131/140	171/171	139/139	208/231	127/132	182/182	147/147	195/198	133/135	164/176	193/193	204/205	205/205	185/191	199/203	180/186	151/151	120/128	106/106	94/94	179/179	135/135	141/141	119/122	115/121
129/137	128/128	116/123	128/138	207/216	149/149	113/113	250/259	216/216	131/140	171/171	139/139	208/231	127/132	182/182	147/147	195/198	133/135	164/176	193/193	204/205	205/205	185/191	199/203	180/186	151/151	120/128	106/106	94/94	179/179	135/135	141/141	119/122	115/121
129/137	128/128	116/123	128/138	207/216	149/149	113/113	250/259	216/216	131/140	171/171	139/139	208/231	127/132	182/182	147/147	195/198	133/135	164/176	193/193	204/205	205/205	185/191	199/203	180/186	151/151	120/128	106/106	94/94	179/179	135/135	141/141	119/122	115/121
129/137	128/128	116/123	128/138	207/216	149/149	113/113	250/259	216/216	131/140	171/171	139/139	208/231	127/132	182/182	147/147	195/198	133/135	164/176	193/193	204/205	205/205	185/191	199/203	180/186	151/151	120/128	106/106	94/94	179/179	135/135	141/141	119/122	115/121
129/137	128/128	116/123	128/138	207/216	149/149	113/113	250/259	216/216	131/140	171/171	139/139	208/231	127/132	182/182	147/147	195/198	133/135	164/176	193/193	204/205	205/205	185/191	199/203	180/186	151/151	120/128	106/106	94/94	179/179	135/135	141/141	119/122	115/121
129/137	128/128	116/123	128/138	207/216	149/149	113/113	250/259	216/216	131/140	171/171	139/139	208/231	127/132	182/182	147/147	195/198	133/135	164/176	193/193	204/205	205/205	185/191	199/203	180/186	151/151	120/128	106/106	94/94	179/179	135/135	141/141	119/122	115/121
129/137	128/128	116/123	128/138	207/216	149/149	113/113	250/259	216/216	131/140	171/171	139/139	208/231	127/132	182/182	147/147	195/198	133/135	164/176	193/193	204/205	205/205	185/191	199/203	180/186	151/151	120/128	106/106	94/94	179/179	135/135	141/141	119/122	115/121
129/137	128/128	116/123	128/138	207/216	149/149	113/113	250/259	216/216	131/140	171/171	139/139	208/231	127/132	182/182	147/147	195/198	133/135	164/176	193/193	204/205	205/205	185/191	199/203	180/186	151/151	120/128	106/106	94/94	179/179	135/135	141/141	119/122	115/121
129/137	128/128	116/123	128/138	207/216	149/149	113/113	250/259	216/216	131/140	171/171	139/139	208/231	127/132	182/182	147/147	195/198	133/135	164/176	193/193	204/205	205/205	185/191	199/203	180/186	151/151	120/128	106/106	94/94	179/179	135/135	141/141	119/122	115/121
129/137	128/128	123/123	128/128	215/215	149/149	113/113	259/259	211/211	131/140	171/171	139/139	231/231	127/132	182/193	153/153	195/198	130/135	162/176	190/190	204/219	205/208	188/191	203/203	180/184	151/151	120/120	106/106	94/102	171/179	132/135	138/141	116/119	112/115
129/129	128/128	116/123/131	128/138	207/216	149/149	113/113	250/259	216/216	131/137	171/177	139/145	208/219/231	127/132	164/182	147/156	195/198/200	130/135	164/176	190/193	204/210	205/205	176/191	199/211	180/184	151/151	120/124	106/114/118	94/114	171/179	132/135	138/141	122/131	115/121
129/129	128/138	116/116	128/138	207/216	149/149	99/113	250/259	216/221	131/140	171/171	139/139	231/231	127/132	182/182	147/153	195/198	133/135	176/176	190/193	204/204	205/211	185/191	199/209	186/186	151/151	120/120	106/106	94/94	179/183	135/135	141/141	119/119	121/121
129/129	128/128	126/132	128/138	207/207	149/149	113/113	250/250	216/221	131/134	171/188	139/139	231/231	127/132	182/182	147/147	198/198	131/135	164/176	187/193	204/204	205/214	185/191	198/209	186/186	151/151	120/128	106/110	94/102	179/179	132/135	138/141	119/119	115/121
129/129	128/128	126/132	128/138	207/207	149/149	113/113	250/250	216/221	131/134	171/188	139/139	231/231	127/132	182/182	147/147	198/198	131/135	164/176	187/193	204/204	205/214	185/191	198/209	186/186	151/151	120/128	106/110	94/102	179/179	132/135	138/141	119/119	115/121
129/129	128/128	114/123	128/128	216/216	149/149	113/113	250/250	216/216	134/134	171/177	139/145	219/220	127/132	164/182	147/156	198/201	130/133	164/176	190/190	205/210	205/214	185/191	205/205	180/184	151/151	124/128	106/122	94/106	171/179	132/135	138/141	128/128	112/121
129/129	128/128	116/123	128/128	207/216	149/149	113/113	250/259	211/215	131/131	168/171	139/139	231/231	127/127	182/184	147/156	195/198	131/131	162/176	187/190	204/219	205/211	185/188	190/203	178/186	151/151	128/128	106/106	94/110	171/183	132/135	138/141	119/119	118/121
129/129	138/138	116/131	128/138	207/216	149/149	113/113	250/259	216/216	137/140	171/186	139/145	219/231	127/127	164/182	147/153	195/201	130/131	164/176	190/193	204/210	205/211	191/194	194/199	186/186	151/151	124/128	106/122	94/106	171/179	132/135	138/141	116/122	112/121
129/137	128/128	114/123	128/138	207/215	149/149	113/113	250/259	216/216	134/140	171/171	139/139	231/231	127/132	164/182	147/153	198/200	133/135	164/176	190/193	204/204	205/211	176/191	199/199	180/186	151/151	120/120	106/114	94/102	171/179	135/138	141/144	119/122	115/121
129/129	128/128	123/126	128/138	207/216	149/149	113/113	259/259	216/216	128/142	160/165	139/139	231/234	127/127	182/182	147/156	195/198	130/131	167/176	187/190	204/204	205/214	185/185	201/207	186/186	151/151	124/128	110/118	94/106	171/179	135/135	141/141	119/122	115/121
129/137	128/128	114/123	128/138	207/215	149/149	113/113	250/259	216/216	134/140	171/171	139/139	231/231	127/132	164/182	147/153	198/200	133/135	164/176	190/193	204/204	205/211	176/191	194/199	180/186	151/151	120/120	106/114	94/102	171/179	135/138	141/144	119/122	115/121
129/137	128/128	114/123	128/138	207/215	149/149	113/113	250/259	216/216	134/140	171/171	139/139	231/231	127/132	164/182	147/153	198/200	133/135	164/176	190/193	204/204	205/211	176/191	194/199	180/186	151/151	120/120	106/114	94/102	171/179	135/138	141/144	119/122	115/121
129/129	nd.	135/135	128/138	207/218	149/149	113/113	259/259	213/216	134/142	160/160	139/145	216/231	127/127	182/182	143/147	192/195	131/131	176/176	187/190	204/210	205/217	179/185	194/207	186/195	151/155	120/124	118/122	94/94	171/179	132/132	138/138	119/119	118/121
129/129	128/128	114/114	128/128	207/216	137/149	113/113	259/259	211/213	134/140	168/168	139/145	217/220	127/127	164/164	147/153	192/195	131/133	176/176	187/190	205/210	205/205	179/185	194/205	186/189	151/155	120/124	106/122	94/94	175/179	132/135	138/141	122/122	118/121
129/137	128/128	114/114	128/128	207/218	149/154	113/113	259/259	213/216	131/134	171/171	139/145	217/217	127/132	182/182	142/142	192/195	131/131	176/176	187/193	204/218	214/217	179/185	194/199	189/191	151/155	124/128	106/122	94/94	179/179	132/135	138/141	119/122	118/124
129/129	nd.	114/135	128/128	216/216	137/149	113/113	259/259	nd.	134/137	160/168	145/145	220/220	127/127	164/164	156/156	201/201	130/130	164/164	190/193	210/210	205/211	182/191	194/217	184/184	151/151	124/124	114/118	102/114	171/171	132/132	138/138	119/131	115/115
129/132	nd.	nd.	128/128	216/216	154/156	113/113	259/259	213/213	134/134	162/167	145/151	216/221	127/127	191/191	143/147	192/198	130/131	164/164	187/196	210/217	208/217	179/185	194/198	184/189	151/155	124/124	106/122	94/94	179/187	132/132	138/138	119/119	115/118
129/129	138/138	116/116	128/138	218/218	137/154	113/113	259/259	213/221	134/134	171/171	139/145	216/220	127/127	164/164	142/153	192/195	131/131	176/176	187/190	204/210	211/217	179/191	194/209	186/189	151/155	120/124	114/122	94/94	171/175	132/135	138/141	119/122	115/118
129/137	128/128	114/116	128/138	207/216	149/149	113/113	250/259	215/216	131/134	168/171	139/139	231/231	127/132	164/184	147/147	195/198	130/133	164/176	190/190	204/219	211/211	176/188	190/199	186/186	151/151	120/120	106/114	94/102	171/171	135/138	141/144	122/131	118/121
129/129	128/138	114/116	128/138	207/216	149/149	113/113	250/250	216/221	131/131	171/171	139/139	231/231	132/135	182/182	147/153	195/198	131/135	176/176	190/193	204/204	205/205	185/191	205/209	180/186	151/151	120/120	106/106	94/102	179/179	135/135	141/141	122/122	115/121
129/129	128/128	123/135	128/138	207/216	149/149	113/113	259/259	216/216	131/142	160/171	139/145	219/231	127/127	182/183	147/156	195/200	130/131	164/176	190/190	204/210	205/211	185/197	194/207	186/186	151/151	120/124	106/118	94/106	171/179	132/135	138/141	122/122	115/121
129/137	128/128	123/123	128/128	215/215	149/149	113/113	250/259	215/215	131/140	168/171	139/139	231/231	127/127	182/193	147/147	195/198	130/133	164/176	190/193	204/219	205/208	191/191	190/190	180/184	151/151	120/120	106/106	94/110	171/179	132/135	138/141	116/122	115/118
129/129	128/128	114/116	128/138	207/216	149/149	113/113	250/259	216/216	131/134	171/177	139/145	208/219	127/132	164/182	147/156	195/201	130/133	164/164	190/193	205/210	205/205	188/191	194/203	180/184	151/151	124/128	106/114	94/102	171/171	132/135	138/141	nd.	115/115
129/129	128/128	116/131	128/138	207/216	149/149	113/113	250/259	216/216	131/137	171/177	139/145	208/219	127/132	164/182	147/156	195/198	130/135	176/176	190/193	204/210	205/205	191/191	199/211	180/184	151/151	120/124	106/118	94/114	171/179	132/135	138/141	122/122	115/121
129/129	128/128	116/131	128/138	207/216	149/149	113/113	250/259	216/216	131/137	171/177	139/145	208/219	127/132	164/182	147/156	195/198	130/135	176/176	190/193	204/210	205/205	191/191	199/211	180/184	151/151	120/124	106/118	94/114	171/179	132/135	138/141	122/122	115/121
129/129	128/128	116/131	128/138	207/216	149/149	113/113	250/259	216/216	131/137	171/177	139/145	208/219	127/132	164/182	147/156	195/198	130/135	176/176	190/193	204/210	205/205	191/191	199/211	180/184	151/151	120/124	106/118	94/114	171/179	132/135	138/141	122/122	115/121
129/129	128/128	116/131	128/138	207/216	149/149	113/113	250/259	216/216	131/137	171/177	139/145	208/219	127/132	164/182	147/156	195/198	130/135	164/176	190/193	204/210	205/205	191/191	199/211	180/184	151/151	120/124	106/118	94/114	171/179	132/135	138/141	119/131	115/121
129/129	128/128	116/123	138/138	207/207	149/149	113/113	259/259	216/216	140/142	171/171	139/139	208/219	127/132	182/182	147/147	195/200	130/133	164/176	190/193	204/205	205/205	191/197	199/207	180/186	151/151	120/120	106/118	94/106	171/179	135/135	141/141	122/131	121/121
129/137	128/128	114/123	128/128	207/215	149/149	113/113	250/259	211/211	128/134	171/180	139/145	231/231	127/132	182/182	147/153	195/204	130/131	164/164	187/190	204/210	205/214	185/194	205/205	180/189	151/151	120/120	110/114	94/102	171/179	135/135	141/141	119/131	115/121
129/137	128/128	115/123	128/128	216/216	137/149	113/113	259/259	212/213	134/137	168/171	139/145	219/219	127/127	164/164	156/156	200/201	130/135	164/164	190/190	204/210	205/211	176/191	194/201	180/184	151/151	120/124	114/126	110/114	171/171	132/135	138/141	131/131	115/115

129/129	138/138	116/131	128/128	207/215	137/149	113/113	250/259	211/211	131/134	171/177	139/145	219/231	132/132	164/182	153/153	198/201	130/135	164/176	190/193	205/210	205/211	185/191	194/205	180/186	151/151	120/124	106/114	94/102	171/171	132/135	138/141	128/128	115/121
129/129	128/128	115/116	128/128	216/216	149/149	113/113	250/259	216/216	131/140	171/174	139/145	208/231	127/132	164/182	147/156	195/200	130/135	164/176	193/193	205/210	205/205	191/197	203/217	180/184	151/151	124/128	106/106	94/94	179/179	132/135	138/141	122/122	115/115
129/129	128/138	114/116	128/138	207/216	149/149	99/113	250/259	221/221	131/131	171/171	139/139	208/231	132/132	182/182	147/153	195/198	131/135	176/176	190/193	204/205	205/217	188/191	199/209	180/186	151/151	120/128	106/106	102/102	175/179	135/135	141/141	122/131	121/121
129/129	128/138	114/116	128/138	207/216	149/149	99/113	250/259	221/221	131/131	171/171	139/139	208/231	132/132	182/182	147/153	195/198	131/135	176/176	190/193	204/205	205/217	188/191	199/209	180/186	151/151	120/128	106/106	102/102	175/179	135/135	141/141	122/131	121/121
129/129	128/138	114/116	128/138	207/216	149/149	99/113	250/259	221/221	131/131	171/171	139/139	208/231	132/132	182/182	147/153	195/198	131/135	176/176	190/193	204/205	205/217	188/191	199/209	180/186	151/151	120/128	106/106	102/102	175/179	135/135	141/141	122/131	121/121
129/129	128/138	114/116	128/138	207/216	149/149	99/113	250/259	221/221	131/131	171/171	139/139	208/231	132/132	182/182	147/153	195/198	131/135	176/176	190/193	204/205	205/217	188/191	199/209	180/186	151/151	120/128	106/106	102/102	175/179	135/135	141/141	122/131	121/121
129/129	138/138	116/116	128/138	216/216	149/149	99/99	250/259	216/221	140/140	171/171	139/139	231/231	127/132	182/182	147/153	195/195	131/133	152/176	190/193	204/204	205/205	191/191	199/205	186/186	151/151	120/120	106/106	94/94	179/179	135/135	141/141	122/122	115/121
129/129	nd.	114/114	128/128	216/216	137/149	113/113	259/259	212/212	137/137	180/180	145/145	219/219	127/127	164/164	156/156	204/204	130/130	164/164	190/190	210/210	205/214	191/191	199/211	184/184	151/151	124/124	114/118	102/114	171/171	132/132	138/138	119/122	115/115
129/129	128/128	123/135	128/128	207/216	149/149	113/113	259/259	216/216	128/142	165/171	139/139	219/231	127/127	182/182	147/156	198/200	131/131	164/164	187/190	203/204	211/214	185/197	194/201	186/186	151/151	120/128	110/118	94/106	171/179	132/135	138/141	119/131	115/115
129/137	128/128	114/116	128/128	215/215	149/149	113/113	250/250	216/216	131/134	171/174	139/145	208/231	127/127	164/182	147/153	198/201	130/135	164/176	193/193	205/211	205/205	185/191	203/211	180/184	151/151	124/128	106/114	94/102	171/171	132/135	138/141	122/131	115/115
129/137	128/128	116/123	128/138	207/216	149/149	113/113	250/259	216/216	134/140	171/171	139/139	231/231	127/127	164/182	147/147	195/200	135/135	164/164	190/193	204/205	205/211	176/191	199/203	180/180	151/151	120/128	106/114	94/94	179/179	135/135	141/141	119/119	115/121
129/137	128/128	116/123	128/138	207/216	149/149	113/113	250/259	216/216	134/140	171/171	139/139	231/231	127/127	164/182	147/147	195/200	135/135	164/164	190/193	204/205	205/211	176/191	199/203	180/180	151/151	120/128	106/114	94/94	179/179	135/135	141/141	119/119	115/121
129/137	128/128	116/123	128/138	207/216	149/149	113/113	250/259	216/216	134/140	171/171	139/139	231/231	127/127	164/182	147/147	195/200	135/135	164/164	190/193	204/205	205/211	176/191	199/203	180/180	151/151	120/128	106/114	94/94	179/179	135/135	141/141	119/119	115/121
129/137	128/128	116/123	128/138	207/216	149/149	113/113	250/259	216/216	134/140	171/171	139/139	231/231	127/127	164/182	147/147	195/200	135/135	164/164	190/193	204/205	205/211	176/191	199/203	180/180	151/151	120/128	106/114	94/94	179/179	135/135	141/141	119/119	115/121
129/137	128/128	116/123	128/138	207/216	149/149	113/113	250/259	216/216	134/140	171/171	139/139	231/231	127/127	164/182	147/147	195/200	135/135	164/164	190/193	204/205	205/211	176/191	199/203	180/180	151/151	120/128	106/114	94/94	179/179	135/135	141/141	119/119	115/121
129/137	128/128	116/123	128/138	207/216	149/149	113/113	250/259	216/216	134/140	171/171	139/139	231/231	127/127	164/182	147/147	195/200	135/135	164/164	190/193	204/205	205/211	176/191	199/203	180/180	151/151	120/128	106/114	94/94	179/179	135/135	141/141	119/119	115/121
129/137	128/128	116/123	128/138	207/216	149/149	113/113	250/259	216/216	134/140	171/171	139/139	231/231	127/127	164/182	147/147	195/200	135/135	164/164	190/193	204/205	205/211	176/191	199/203	180/180	151/151	120/128	106/114	94/94	179/179	135/135	141/141	122/131	115/121
129/137	128/128	116/123	128/138	207/216	149/149	113/113	250/259	216/216	134/140	171/171	139/139	231/231	127/127	164/182	147/147	195/200	135/135	164/164	190/193	204/205	205/211	176/191	199/203	180/180	151/151	120/128	106/114	94/94	179/179	135/135	141/141	119/119	115/121
129/137	128/128	116/123	128/138	207/216	149/149	113/113	250/259	216/216	134/140	171/171	139/139	231/231	127/127	164/182	147/147	195/200	135/135	164/164	190/193	204/205	205/211	176/191	199/203	180/180	151/151	120/128	106/114	94/94	179/179	135/135	141/141	119/119	115/121
129/137	128/128	116/123	128/138	207/216	149/149	113/113	250/259	216/216	134/140	171/171	139/139	231/231	127/127	164/182	147/147	195/200	135/135	164/164	190/193	204/205	205/211	176/191	199/203	180/180	151/151	120/128	106/114	94/94	179/179	135/135	141/141	119/119	115/121
129/137	128/128	116/123	128/138	207/216	149/149	113/113	250/259	216/216	134/140	171/171	139/139	231/231	127/127	164/182	147/147	195/200	135/135	164/164	190/193	204/205	205/211	176/191	199/203	180/180	151/151	120/128	106/114	94/94	179/179	135/135	141/141	119/119	115/121
129/137	128/128	116/123	128/138	207/216	149/149	113/113	250/259	216/216	134/140	171/171	139/139	231/231	127/127	164/182	147/147	195/200	135/135	164/164	190/193	204/205	205/211	176/191	199/203	180/180	151/151	120/128	106/114	94/94	179/179	135/135	141/141	119/119	115/121
129/137	128/128	116/123	128/138	207/216	149/149	113/113	250/259	216/216	134/140	171/171	139/139	231/231	127/127	164/182	147/147	195/200	135/135	164/164	190/193	204/205	205/211	176/191	199/203	180/180	151/151	120/128	106/114	94/94	179/179	135/135	141/141	119/119	115/121
129/137	128/128	116/123	128/138	207/216	149/149	113/113	250/259	216/216	134/140	171/171	139/139	231/231	127/127	164/182	147/147	195/200	135/135	164/164	190/193	204/205	205/211	176/191	199/203	180/180	151/151	120/128	106/114	94/94	179/179	135/135	141/141	119/119	115/121
129/137	128/128	116/123	128/138	207/216	149/149	113/113	250/259	216/216	134/140	171/171	139/139	231/231	127/127	164/182	147/147	195/200	135/135	164/164	190/193	204/205	205/211	176/191	199/203	180/180	151/151	120/128	106/114	94/94	179/179	135/135	141/141	119/119	115/121
129/137	128/128	116/123	128/138	207/216	149/149	113/113	250/259	216/216	134/140	171/171	139/139	231/231	127/127	164/182	147/147	195/200	135/135	164/164	190/193	204/205	205/211	176/191	199/203	180/180	151/151	120/128	106/114	94/94	179/179	135/135	141/141	119/119	115/121
129/137	128/128	116/123	128/138	207/216	149/149	113/113	250/259	216/216	134/140	171/171	139/139	231/231	127/127	164/182	147/147	195/200	135/135	164/164	190/193	204/205	205/211	176/191	199/203	180/180	151/151	120/128	106/114	94/94	179/179	135/135	141/141	119/119	115/121
129/137	128/128	116/123	128/138	207/216	149/149	113/113	250/259	216/216	134/140	171/171	139/139	231/231	127/127	164/182	147/147	195/200	135/135	164/164	190/193	204/205	205/211	176/191	199/203	180/180	151/151	120/128	106/114	94/94	179/179	135/135	141/141	119/119	115/121
129/137	128/128	116/123	128/138	207/216	149/149	113/113	250/259	216/216	134/140	171/171	139/139	231/231	127/127	164/182	147/147	195/200	135/135	164/164	190/193	204/205	205/211	176/191	199/203	180/180	151/151	120/128	106/114	94/94	179/179	135/135	141/141	119/119	115/121
129/137	128/128	116/123	128/138	207/216	149/149	113/113	250/259	216/216	134/140	171/171	139/139	231/231	127/127	164/182	147/147	195/200	135/135	164/164	190/193	204/205	205/211	176/191	199/203	180/180	151/151	120/128	106/114	94/94	179/179	135/135	141/141	119/131	115/121
129/137	128/128	116/123	128/138	207/216	149/149	113/113	250/259	216/216	134/140	171/171	139/139	231/231	127/127	164/182	147/147	195/200	135/135	164/164	190/193	204/205	205/211	176/191	199/203	180/180	151/151	120/128	106/114	94/94	179/179	135/135	141/141	119/119	115/121
129/129	128/128	114/123	128/128	216/216	149/149	113/113	250/250	216/216	134/134	171/177	139/145	219/220	127/132	164/182	147/156	198/201	130/133	164/176	190/190	205/210	205/214	185/191	205/205	180/184	151/151	124/128	106/122	94/106	171/179	132/135	138/141	119/122	112/121
129/129	128/128	114/126	128/128	216/216	149/149	113/113	250/259	211/216	128/131	165/171	139/139	231/231	127/132	182/182	147/167	195/198	131/133	164/176	187/193	204/204	205/214	185/185	201/203	186/186	151/151	116/120	106/110	94/94	179/179	135/135	141/141	119/122	115/121
129/137	128/138	114/123	128/128	216/216	149/149	113/113	250/259	211/216	128/131	165/171	139/139	231/231	127/132	182/182	147/156	195/198	131/131	164/176	187/193	204/204	205/217	185/185	201/203	186/186	151/151	128/128	106/110	94/94	171/179	135/135	141/141	119/122	115/115
129/129	128/128	114/116	128/138	207/207	137/149	113/113	259/259	nd.	134/134	171/177	139/139	231/231	127/132	182/182	147/156	195/201	130/135	164/164	190/193	204/205	205/205	185/188	194/194	180/180	151/151	120/120	114/114	94/102	171/171	132/135	138/141	119/119	115/115
129/137	128/138	116/123	138/138	207/207	149/149	99/113	250/259	216/221	131/131	171/171	139/139	208/231	127/132	182/182	147/147	198/198	133/135	164/176	193/193	204/205	205/211	185/191	199/209	186/186	151/151	120/120	106/106	94/94	183/183	135/135	141/141	119/119	121/121
129/129	128/128	123/135	128/138	207/216	149/149	113/113	259/259	216/216	131/142	160/171	139/145	219/231	127/127	182/183	147/156	195/200	130/131	164/188	190/190	204/210	205/211	185/197	194/207	186/186	151/151	120/124	106/118	94/106	171/179	132/135	138/141	122/131	115/121
129/129	128/128	123/135	128/138	207/216	149/149	113/113	259/259	216/216	131/142	160/171	139/145	219/231	127/127	182/183	147/156	195/200	130/131	164/176	190/190	204/210	205/211	185/197	194/207	186/186	151/151	120/124	106/118	94/106	171/179	132/135	138/141	122/122	115/121
129/129	128/128	123/135	128/138	207/216	149/149	113/113	259/259	216/216	131/142	160/171	139/145	219/231	127/127	182/183	147/156	195/200	130/131	176/176	190/190	204/210	205/211	185/197	194/207	186/186	151/151	120/124	106/118	94/106	171/179	132/135	138/141	122/122	115/121
129/129	128/128	123/135	128/138	207/216	149/149	113/113	259/259	216/216	131/142	160/171	139/145	219/231	127/127	182/183	147/156	195/200	130/131	164/176	190/190	204/210	205/211	nd.	194/207	186/186	151/151	120/124	106/118	94/106	171/179	132/135	138/141	122/122	115/121
129/129	128/128	123/135	128/138	207/216	149/149	113/113	259/259	216/216	131/142	160/171	139/145	219/231	127/127	182/183	147/156	195/200	130/131	164/176	190/190	204/210	205/211	185/197	194/207	186/186	151/151	120/124	106/118	94/106	171/179	132/135	138/141	116/119	115/121
129/129	128/128	114/123	128/138	207/216	149/149	113/113	259/259	215/221	131/131	168/188	139/139	220/231	127/132	182/184	147/147	195/198	130/135	162/176	190/193	204/219	205/211	188/191	190/209	178/184	151/151	120/120	106/106	94/102	175/179	132/135	138/141	116/119	118/121
129/129	128/128	114/123	128/128	216/216	149/149	113/113	259/259	212/214	137/137	168/180	145/145	219/219	127/127	164/164	156/156	201/201	130/130	164/164	190/190	210/210	205/211	191/191	194/211	180/184	151/151	120/124	114/118	102/114	171/171	132/135	138/141	131/131	115/115
129/137	128/138	114/116	128/138	207/207	149/149	113/113	250/259	216/216	131/131	171/171	139/139	231/231	132/132	182/182	147/147	195/195	131/133	176/176	190/193	204/204	205/205	185/191	205/205	186/186	151/151	120/128	106/106	94/94	179/179	135/135	141/141	122/131	115/121
129/129	128/128	116/126	128/138	207/207	149/149	113/113	250/250	216/216	131/134	171/171	139/139	231/231	127/132	182/182	147/147	198/198	131/133	164/176	193/193	205/205	205/205	185/185	198/199	180/196	151/151	120/128	106/110	94/94	179/179	135/135	141/141	122/131	115/121
129/129	138/138	116/116	128/138	216/216	149/149	99/113	250/259	221/221	137/140	165/171	139/139	219/231	127/132	182/183	147/153	198/200	131/135	152/176	190/193	204/204	205/211	188/191	194/209	184/186	151/151	120/120	106/118	102/114	171/179	135/138	141/144	116/122	115/121
129/129	138/138	116/116	128/138	216/216	149/149	99/113	250/259	221/221	137/140	165/171	139/139	220/231	127/132	182/183	147/153	198/200	131/135	152/176	190/193	204/204	205/211	188/191	194/209	184/186	151/151	120/120	106/118	102/114	171/179	135/138	141/144	116/122	115/121
129/129	138/138	116/116	128/138	216/216	149/149	99/113	250/259	221/221	137/140	165/171	139/139	219/231	127/132	182/183	147/153	198/200	131/135	152/176	190/193	204/204	205/211	188/191	194/209	184/186	151/151	120/120	106/118	102/114	171/179	135/138	141/144	116/122	115/121
129/129	138/138	116/116	128/138	216/216	149/149	99/113	250/259	221/221	137/140	165/171	139/139	219/231	127/132	182/183	147/153	198/200	131/135	152/176	190/193	204/204	205/211	188/191	194/209	184/186	151/151	120/120	106/118	102/114	171/179	135/138	141/144	116/122	115/121
129/129	138/138	116/116	128/138	216/216	149/149	99/113	250/259	221/221	137/140	165/171	139/139	219/231	127/132	182/183	147/153	198/200	131/135	152/176	190/193	204/204	205/211	188/191	194/209	184/186	151/151	120/120	106/118	102/114	171/179	135/138	141/144	116/122	115/121
129/129	138/138	116/116	128/138	216/216	149/149	99/113	250/259	221/221	137/140	165/171	139/139	219/231	127/132	182/183	147/153	198/200	131/135	152/176	190/193	204/204	205/211	188/191	194/209	184/186	151/151	120/120	106/118	102/114	171/179	135/138	141/144	122/122	115/121
129/129	138/138	116/116	128/138	216/216	149/149	99/113	250/259	221/221	137/140	165/171	139/139	219/231	127/132	182/183	147/153	198/200	131/135	152/176	190/193	204/204	205/211	188/191	194/209	184/186	151/151	120/120	106/118	102/114	171/179	135/138	141/144	116/122	115/121
129/129	138/138	116/116	128/138	216/216	149/149	99/113	250/259	221/221	137/140	165/171	139/139	219/231	127/132	182/183	147/153	198/200	131/135	152/176	190/193	204/204	205/211	188/191	194/209	184/186	151/151	120/120	106/118	102/114	171/179	135/138	141/144	116/122	115/121
129/129	138/138	116/116	128/138	216/216	149/149	99/113	250/259	221/221	137/140	165/171	139/139	219/231	127/132	182/183	147/153	198/200	131/135	152/176	190/193	204/204	205/211	188/191	194/209	184/186	151/151	120/120	106/118	102/114	171/179	135/138	141/144	119/122	115/121
129/129	138/138	116/116	128/138	216/216	149/149	99/113	250/259	221/221	137/140	165/171	139/139	219/231	127/132	182/183	147/153	198/200	131/135	152/176	190/193	204/204	205/211	188/191	194/209	184/186	151/151	120/120	106/118	102/114	171/179	135/138	141/144	116/122	115/121
129/129	138/138	116/116	128/138	216/216	149/149	99/113	250/259	221/221	137/140	165/171	139/139	219/231	127/132	182/183	147/153	198/200	131/135	176/176	190/193	204/204	205/211	188/191	194/209	184/186	151/151	120/120	106/118	102/114	171/179	135/138	141/144	116/122	115/121
129/129	138/138	116/116	128/138	216/216	149/149	99/113	250/259	221/221	137/140	165/171	139/139	219/231	127/132	182/183	147/153	198/200	131/135	152/176	190/193	204/204	205/211	188/191	194/209	184/186	151/151	120/120	106/118	102/114	171/179	135/138	141/144	116/122	115/121
129/129	138/138	116/116	128/138	216/216	149/149	99/113	250/259	221/221	137/140	165/171	139/139	219/231	127/132	182/183	147/153	198/200	131/135	152/176	190/193	204/204	205/211	188/191	194/209	184/186	151/151	120/120	106/118	102/114	171/179	135/138	141/144	122/131	115/121
129/129	138/138	116/116	128/138	216/216	149/149	99/113	250/259	221/221	137/140	165/171	139/139	219/231	127/132	182/183	147/153	198/200	131/135	152/176	190/193	204/204	205/211	188/191	194/209	184/186	151/151	120/120	106/118	102/114	171/179	135/138	141/144	116/122	115/121
129/129	138/138	116/116	128/138	216/216	149/149	99/113	250/259	221/221	137/140	165/171	139/139	219/231	127/132	182/183	147/153	198/200	131/135	176/176	190/193	204/204	205/211	188/191	194/209	184/186	151/151	120/120	106/118	102/114	171/179	135/138	141/144	116/122	115/121
129/129	138/138	116/116	128/138	216/216	149/149	99/113	250/259	221/221	137/140	165/171	139/139	219/231	127/132	182/183	147/153	198/200	131/135	176/176	190/193	204/204	205/211	188/191	194/209	184/186	151/151	120/120	106/118	102/114	171/179	135/138	141/144	116/122	115/121
129/129	138/138	116/116	128/138	216/216	149/149	99/113	250/259	221/221	137/140	165/171	139/139	219/231	127/132	182/183	147/153	198/200	131/135	176/176	190/193	204/204	205/211	188/191	194/209	184/186	151/151	120/120	106/118	102/114	171/179	135/138	141/144	116/122	115/121
129/129	138/138	116/116	128/138	216/216	149/149	99/113	250/259	221/221	137/140	165/171	139/139	219/231	127/132	182/183	147/153	198/200	131/135	152/176	190/193	204/204	205/211	188/191	194/209	184/186	151/151	120/120	106/118	102/114	171/179	135/138	141/144	116/122	115/121
129/129	138/138	116/116	128/138	216/216	149/149	99/113	250/259	221/221	137/140	165/171	139/139	219/231	127/132	182/183	147/153	198/200	131/135	152/176	190/193	204/204	205/211	188/191	194/209	184/186	151/151	120/120	106/118	102/114	171/179	135/138	141/144	116/122	115/121
129/129	138/138	116/116	128/138	216/216	149/149	99/113	250/259	221/221	137/140	165/171	139/139	219/231	127/132	182/183	147/153	198/200	131/135	152/176	190/193	204/204	205/211	188/191	194/209	184/186	151/151	120/120	106/118	102/114	171/179	135/138	141/144	122/131	115/121
129/129	128/128	114/126	128/128	207/216	149/149	113/113	250/259	211/216	128/131	165/171	139/139	231/231	121/132	182/182	147/156	195/198	131/131	164/176	187/193	204/204	205/205	185/185	198/203	196/196	151/151	128/128	106/110	94/94	179/183	135/135	141/141	119/122	115/121
129/129	128/128	115/116	128/128	207/216	149/149	113/113	250/259	211/216	131/134	165/171	139/139	231/231	121/132	182/182	147/156	195/195	131/131	167/176	187/193	204/204	205/214	182/185	198/203	178/186	151/151	128/128	106/110	94/94	179/183	135/135	141/141	119/122	115/121
129/129	128/128	115/116	128/128	207/216	149/149	113/113	250/259	211/216	131/134	165/171	139/139	231/231	121/132	182/182	147/156	195/195	131/131	167/176	187/193	204/204	205/214	182/185	198/203	178/186	151/151	128/128	106/110	94/94	179/183	135/135	141/141	119/122	115/121
129/129	128/128	115/116	128/128	207/216	149/149	113/113	250/259	211/216	131/134	165/171	139/139	231/231	121/132	182/182	147/156	195/195	131/131	167/176	187/193	204/204	205/214	182/185	198/203	178/186	151/151	128/128	106/110	94/94	179/183	135/135	141/141	119/122	115/121
129/129	128/128	114/126	128/128	207/216	149/149	113/113	250/259	211/216	128/131	165/171	139/139	231/231	121/132	182/182	147/156	195/198	131/131	164/176	187/193	204/204	205/205	185/185	198/203	196/196	151/151	128/128	106/110	94/94	179/183	135/135	141/141	119/122	115/121
129/129	128/128	114/126	128/128	207/216	149/149	113/113	250/259	211/216	128/131	165/171	139/139	231/231	121/132	182/182	147/156	195/198	131/131	164/176	187/193	204/204	205/205	185/185	198/203	196/196	151/151	128/128	106/110	94/94	179/183	135/135	141/141	119/122	115/121
129/129	128/128	114/126	128/128	207/216	149/149	113/113	250/259	211/216	128/131	165/171	139/139	220/231	127/132	182/182	147/156	195/195	131/135	164/164	187/193	204/204	205/214	185/185	201/205	189/196	151/151	120/128	106/110	94/94	179/179	135/135	141/141	119/119	115/121
129/129	128/128	115/116	128/128	207/216	149/149	113/113	250/259	211/216	131/134	165/171	139/139	231/231	121/132	182/182	147/156	195/195	131/131	167/176	187/193	204/204	205/214	182/185	198/203	178/186	151/151	128/128	106/110	94/94	179/183	135/135	141/141	119/122	115/121
129/129	128/128	115/116	128/128	207/216	149/149	113/113	250/259	211/216	131/134	165/171	139/139	231/231	121/132	182/182	147/156	195/195	131/131	167/176	187/193	204/204	205/214	182/185	198/203	178/186	151/151	128/128	106/110	94/94	179/183	135/135	141/141	119/122	115/121
129/129	128/128	115/116	128/128	207/216	149/149	113/113	250/259	211/216	131/134	165/171	139/139	231/231	121/132	182/182	147/156	195/195	131/131	167/176	187/193	204/204	205/214	182/185	198/203	178/186	151/151	128/128	106/110	94/94	179/183	135/135	141/141	119/122	115/121
129/129	128/128	114/126	128/128	207/216	149/149	113/113	250/259	211/216	128/131	165/171	139/139	231/231	121/132	182/182	147/156	195/198	131/131	164/176	187/193	204/204	205/205	185/185	198/203	196/196	151/151	128/128	106/110	94/94	179/183	135/135	141/141	119/122	115/121
129/129	128/128	115/116	128/128	207/216	149/149	113/113	250/259	211/216	131/134	165/171	139/139	231/231	121/132	182/182	147/156	195/195	131/131	167/176	187/193	204/204	205/214	182/185	198/203	178/186	151/151	128/128	106/110	94/94	179/183	135/135	141/141	119/122	115/121
129/129	128/128	115/132	128/138	207/216	149/149	113/113	250/259	216/221	131/134	165/188	139/139	220/231	121/127	182/182	147/156	195/198	131/133	167/176	187/190	204/204	205/214	185/191	198/209	178/186	151/151	120/128	106/110	94/102	179/179	132/135	138/141	110/110	115/121
129/129	128/128	114/123	128/138	207/216	149/149	113/113	225/259	216/216	131/134	160/180	139/145	219/231	127/127	173/182	147/156	195/204	130/130	164/164	190/190	204/210	205/211	185/188	207/211	184/184	151/151	120/124	106/118	94/102	171/179	132/135	138/141	122/131	115/121
129/129	138/138	116/116	128/138	207/216	149/149	99/113	250/259	216/221	140/140	171/171	139/139	231/231	127/132	182/182	147/153	195/198	131/135	152/176	190/193	204/204	205/205	191/191	205/209	184/186	151/151	120/128	106/106	94/114	179/179	135/138	141/144	122/131	115/121
129/129	138/138	116/116	128/138	207/216	149/149	99/113	250/259	216/221	140/140	171/171	139/139	231/231	127/132	182/182	147/153	195/198	131/135	176/176	190/193	204/204	205/205	191/191	205/209	184/186	151/151	120/128	106/106	94/114	179/179	135/138	141/144	122/131	115/121
129/129	138/138	116/116	128/138	207/216	149/149	99/113	250/259	216/221	140/140	171/171	139/139	231/231	127/132	182/182	147/153	195/198	131/135	152/176	190/193	204/204	205/205	191/191	205/209	184/186	151/151	120/128	106/106	94/114	179/179	135/138	141/144	122/131	115/121
129/129	138/138	116/116	128/138	207/216	149/149	99/113	250/259	216/221	140/140	171/171	139/139	231/231	127/132	182/182	147/153	195/198	131/135	152/176	190/193	204/204	205/205	191/191	205/209	184/186	151/151	120/128	106/106	94/114	179/179	135/138	141/144	122/131	115/121
129/129	128/128	123/123	128/138	216/216	149/149	99/99	250/250	221/221	131/137	165/171	139/139	231/231	127/132	182/182	147/153	195/200	131/133	176/176	193/193	204/204	205/205	188/188	199/209	180/184	151/151	120/128	106/106	94/102	179/179	135/138	141/144	116/122	121/121
129/137	128/138	116/123	128/128	216/216	149/149	99/113	250/259	nd.	134/140	171/180	139/145	231/231	127/127	164/182	153/153	198/198	130/135	164/176	190/193	204/210	205/211	191/191	194/211	180/186	151/151	120/124	106/114	102/114	171/179	135/135	141/141	122/131	115/115
129/129	128/138	114/116	128/138	207/216	149/149	99/113	250/250	216/221	131/137	165/171	139/139	219/228	127/132	182/182	147/153	198/200	131/135	176/176	193/193	204/204	205/205	185/188	209/209	186/189	151/151	120/128	106/118	94/102	171/179	135/135	141/141	119/122	121/121
129/129	128/138	126/132	128/138	207/207	149/149	113/113	250/250	216/221	131/134	171/188	139/139	231/231	127/132	182/182	147/147	198/198	131/135	164/176	187/193	204/204	205/214	185/191	198/209	186/186	151/151	120/128	106/110	94/102	179/179	132/135	138/141	119/131	115/121
129/137	128/128	114/123	128/138	207/215	149/149	113/113	250/259	216/216	134/140	171/171	139/139	231/231	127/132	164/182	147/153	198/200	133/135	164/176	190/193	204/204	205/211	176/191	194/199	180/186	151/151	120/120	106/114	94/102	171/179	135/138	141/144	119/122	115/121
129/137	128/128	115/123	128/128	216/216	137/149	113/113	259/259	212/213	134/137	168/171	139/145	219/219	127/127	164/164	156/156	200/201	130/135	164/176	190/190	204/210	205/211	176/191	194/201	180/184	151/151	120/124	114/126	110/114	171/171	132/135	138/141	131/131	115/115
129/137	128/138	123/126	128/128	215/216	149/149	113/113	250/259	216/216	131/131	171/171	139/139	231/231	127/127	182/182	153/167	195/198	131/133	164/164	187/190	204/204	205/214	176/185	194/201	186/186	151/151	116/120	106/106	94/94	171/179	135/135	141/141	122/122	115/115
129/137	128/128	114/123	128/138	207/215	149/149	113/113	250/259	216/216	134/140	171/171	139/139	231/231	127/132	164/182	147/153	198/200	133/135	164/176	190/193	204/204	205/211	176/191	194/199	180/186	151/151	120/120	106/114	94/102	171/179	135/138	141/144	119/122	115/121
129/137	128/128	114/123	128/138	207/215	149/149	113/113	250/259	216/216	134/140	171/171	139/139	231/231	127/132	164/182	147/153	198/200	133/135	164/176	190/193	204/204	205/211	176/191	194/199	180/186	151/151	120/120	106/114	94/102	171/179	135/138	141/144	119/122	115/121
129/137	128/128	114/123	128/138	207/215	149/149	113/113	250/259	216/216	134/140	171/171	139/139	231/231	127/132	164/182	147/153	198/200	133/135	164/176	190/193	204/204	205/211	176/191	194/199	180/186	151/151	120/120	106/114	94/102	171/179	135/138	141/144	119/122	115/121
129/129	nd.	115/131	128/128	216/216	137/149	113/113	259/259	212/212	134/134	177/177	145/145	219/219	127/127	164/164	156/156	201/201	130/130	164/164	190/190	210/211	205/208	191/191	194/194	184/184	151/151	124/124	114/114	114/114	171/171	132/132	138/138	119/119	115/115
129/137	128/128	114/123	128/138	207/216	149/149	113/113	250/259	216/216	134/140	171/171	139/139	231/231	127/132	164/182	147/153	198/201	133/135	164/164	190/193	204/205	205/211	185/188	194/199	184/184	151/151	120/120	106/118	94/102	171/179	132/135	138/141	119/119	115/121
129/137	128/128	115/123	128/138	207/216	149/149	113/113	259/259	216/216	128/140	165/171	139/139	231/231	127/132	164/182	147/156	198/198	131/135	167/176	187/193	204/204	205/211	185/191	199/201	180/196	151/151	120/128	110/114	94/102	171/179	135/135	141/141	119/122	115/121
129/137	128/128	115/123	128/128	216/216	149/149	113/113	250/250	216/216	137/140	168/171	139/145	219/231	127/127	164/164	147/156	195/201	130/135	164/164	190/190	205/211	211/214	191/191	194/203	180/184	151/151	120/124	106/114	94/102	171/179	132/135	138/141	119/122	115/121
129/129	128/128	114/123	128/138	207/216	149/149	99/113	250/250	211/216	131/140	171/171	139/139	208/231	127/132	182/182	147/147	195/195	131/135	176/176	199/199	205/205	205/205	185/185	205/205	180/189	151/151	120/128	106/106	94/94	179/179	135/135	141/141	122/122	121/121
129/129	128/128	114/123	128/138	207/216	149/149	99/113	250/250	211/221	131/140	171/171	139/139	231/231	127/132	182/182	147/147	195/198	133/133	176/176	190/193	205/205	205/205	182/185	199/205	180/186	151/151	120/128	106/106	94/94	179/179	135/135	141/141	122/131	121/121
129/129	128/128	114/116	128/128	207/215	149/149	113/113	250/259	216/216	131/134	171/174	139/145	219/231	127/132	164/182	147/153	198/201	130/133	164/164	193/193	204/211	205/211	191/191	198/211	184/184	151/151	120/124	106/118	94/102	171/179	132/135	138/141	116/119	115/115
129/129	138/138	114/116	128/138	216/216	149/149	99/113	250/259	221/221	134/140	168/171	139/145	219/231	127/132	164/182	153/156	198/204	130/135	164/176	190/190	204/210	205/208	191/194	209/211	184/184	151/151	120/124	106/114	102/114	171/179	132/135	138/141	116/116	115/121
129/137	128/128	116/123	128/128	207/216	149/149	113/113	259/259	216/216	131/134	171/171	139/139	208/231	132/132	164/182	147/147	195/198	135/135	164/164	193/193	204/205	205/205	185/191	203/203	180/180	151/151	120/128	106/114	94/102	171/179	135/135	141/141	116/131	115/115
129/129	nd.	114/114	128/128	207/216	149/149	113/113	250/259	216/216	134/134	171/174	139/145	219/231	127/132	164/173	147/156	198/201	130/135	164/164	190/193	205/210	205/211	185/191	199/211	184/184	151/151	120/124	114/118	94/114	171/171	132/132	138/138	nd.	115/121
129/137	128/128	114/114	128/128	207/207	149/149	113/113	250/250	216/216	131/140	171/171	139/139	220/231	132/132	182/182	147/147	195/195	131/133	176/176	193/199	205/205	205/205	185/185	205/207	186/189	151/151	120/120	106/106	94/94	179/179	135/135	141/141	122/122	121/121
129/129	128/128	114/116	128/138	216/216	149/149	113/113	250/250	216/216	134/140	171/174	139/145	231/231	127/132	164/182	147/156	195/201	130/130	164/164	193/193	204/204	205/205	191/191	199/211	180/184	151/151	124/128	106/114	94/102	171/179	132/135	138/141	122/131	115/115
129/129	128/128	116/123	128/138	216/216	149/149	113/113	259/259	211/215	131/131	168/168	139/139	220/231	127/127	184/193	156/156	195/195	130/131	162/176	190/190	210/219	208/211	188/191	190/203	184/184	151/151	128/128	106/106	94/110	171/175	132/135	138/141	116/119	112/118
129/129	128/138	116/116	138/138	207/216	149/149	99/113	250/259	216/221	131/140	171/171	139/139	231/231	127/132	182/182	147/153	195/200	135/135	176/176	190/193	204/204	205/205	188/191	199/209	180/186	151/151	120/120	106/118	94/102	171/179	135/135	141/141	122/122	121/121
129/129	128/128	116/123	138/138	216/216	149/149	99/113	250/259	216/216	131/140	171/171	139/139	219/231	127/127	182/183	147/153	195/200	130/135	164/176	190/193	204/205	205/211	188/191	199/199	184/186	151/151	120/120	106/118	94/102	171/179	135/135	141/141	116/119	115/121

129/129	128/128	116/116	128/128	216/216	149/149	99/113	250/259	216/216	134/137	171/171	139/139	219/231	127/127	182/183	147/147	195/200	135/135	164/176	190/190	204/204	205/211	188/191	194/203	180/184	151/151	120/120	114/118	94/114	171/179	135/138	141/144	131/131	115/115
129/129	128/138	116/123	128/128	216/216	149/149	99/113	250/259	212/221	131/137	168/171	139/145	219/231	127/132	164/182	147/153	198/200	130/133	164/176	190/190	205/210	205/205	182/188	194/199	180/186	151/151	120/120	106/118	94/114	171/179	135/135	141/141	116/119	115/121
129/129	128/128	114/116	138/138	207/207	149/149	99/113	250/259	211/216	131/134	171/171	139/139	231/231	127/127	182/183	147/147	195/198	133/135	176/176	190/193	204/205	205/211	191/191	199/199	180/186	151/151	120/128	106/114	94/94	179/179	135/135	141/141	116/119	121/121
129/129	138/138	116/116	128/138	216/216	149/149	99/113	250/259	221/221	131/140	165/171	139/139	231/231	127/132	182/183	147/153	198/200	131/133	176/176	193/193	204/204	205/211	188/191	194/205	184/186	151/151	120/128	106/106	102/102	179/179	135/138	141/144	122/131	115/121
129/129	128/128	114/116	128/128	207/216	149/149	99/113	250/259	221/221	131/140	165/171	139/139	231/231	127/132	182/182	147/147	195/200	131/135	176/176	193/193	204/204	205/205	188/191	194/199	180/180	151/151	120/120	106/106	94/102	179/179	135/135	141/141	119/122	115/121
129/129	nd.	114/114	128/128	216/216	137/137	113/113	259/259	211/211	134/134	177/177	145/145	219/231	127/129	164/188	156/156	198/201	130/131	150/164	190/190	211/211	199/211	179/191	194/194	184/184	151/155	120/124	114/114	94/102	171/171	132/132	138/138	nd.	115/?
129/129	128/138	116/116	128/128	207/216	149/149	113/113	250/259	211/211	134/140	171/180	139/139	231/231	127/132	182/182	147/147	195/200	135/135	167/176	190/193	204/204	205/214	182/188	194/203	178/186	151/151	120/120	106/110	94/102	179/179	135/135	141/141	119/122	115/121
129/129	128/138	116/123	128/138	207/216	149/149	99/99	250/250	211/216	131/140	171/171	139/139	231/231	127/127	182/182	147/153	195/198	131/135	176/176	190/193	204/205	205/205	185/191	199/203	186/186	151/151	120/128	106/114	94/102	179/179	135/135	141/141	119/122	115/121
129/129	128/128	115/135	128/128	216/216	149/149	113/113	259/259	213/214	137/137	168/177	145/145	219/219	127/127	164/164	156/156	201/201	130/130	164/164	190/190	210/210	208/211	182/191	194/211	184/184	151/151	124/124	118/122	102/102	171/171	132/132	138/138	119/131	115/115
129/129	nd.	114/135	128/128	216/216	149/149	113/113	259/259	212/212	137/137	177/180	145/145	219/219	127/127	164/164	156/156	200/204	130/130	164/164	190/190	210/210	214/220	191/197	194/211	184/184	151/151	124/124	114/118	102/114	171/171	132/138	138/144	131/131	115/115
129/129	128/138	116/123	138/138	207/216	149/149	99/113	250/250	216/221	134/140	171/171	139/139	219/231	127/132	164/182	147/153	195/200	131/135	164/176	190/193	204/205	205/211	176/191	199/209	180/180	151/151	120/128	106/118	94/102	171/179	135/135	141/141	119/122	121/121
129/129	128/128	114/123	138/138	207/207	149/149	99/113	250/250	211/216	131/131	171/171	139/139	231/231	127/127	182/182	147/147	195/198	131/133	176/176	190/193	204/205	205/205	185/191	199/207	186/186	151/151	128/128	106/106	94/94	179/179	135/135	141/141	116/122	121/121
129/129	128/138	114/116	128/138	207/216	149/149	99/99	250/250	211/216	134/140	171/171	139/139	231/231	127/132	164/182	147/153	198/200	131/135	176/176	190/193	204/205	208/211	185/188	205/205	186/186	151/151	120/128	106/118	94/102	171/179	135/135	141/141	116/119	115/121
129/129	128/138	114/114	128/138	207/207	149/149	99/99	250/259	211/221	131/131	171/171	139/139	231/231	127/132	182/183	147/147	198/200	130/133	176/176	190/193	204/205	205/205	185/188	209/209	186/186	151/151	120/128	106/118	94/102	171/179	135/135	141/141	119/119	121/121
129/129	128/128	116/116	138/138	207/216	149/149	113/113	250/250	216/216	134/140	171/171	139/139	231/231	127/132	164/182	147/153	195/200	130/135	164/164	190/193	204/204	205/211	188/191	194/199	180/180	151/151	120/128	106/114	94/102	179/179	135/135	141/141	119/122	115/121
129/129	128/128	114/114	128/138	207/207	149/149	99/113	250/250	211/221	131/131	171/171	139/139	208/231	127/132	182/182	147/147	195/198	133/135	176/176	190/190	204/205	205/217	191/191	207/209	180/186	151/151	120/128	106/106	94/102	179/179	135/135	141/141	122/122	121/121
129/129	128/128	116/116	128/128	207/216	149/149	113/113	259/259	211/211	134/140	171/180	139/139	231/231	127/132	182/182	147/147	195/200	135/135	167/176	193/193	204/204	205/214	188/188	194/203	178/178	151/151	120/120	106/110	94/94	179/179	135/135	141/141	119/122	115/121
129/129	128/128	116/116	128/138	207/207	149/149	113/113	250/250	211/216	134/137	171/180	139/139	219/231	121/127	164/182	147/147	195/198	131/135	167/176	187/193	204/204	205/211	182/191	199/203	178/180	151/151	120/128	114/118	94/114	171/171	135/135	141/141	122/131	121/121
129/129	128/128	114/116	128/138	207/216	149/149	99/113	259/259	221/221	131/140	171/171	139/139	231/231	127/132	182/182	147/153	195/195	135/135	176/176	190/193	204/204	205/205	191/191	194/199	180/180	151/151	120/120	106/118	94/102	171/179	135/135	141/141	119/119	115/121
129/129	128/128	114/116	128/128	207/216	149/149	113/113	259/259	216/221	131/134	171/171	139/139	208/231	127/132	182/182	147/147	195/198	135/135	164/176	193/193	204/205	205/217	191/191	199/203	180/180	151/151	120/128	106/114	94/102	179/179	135/135	141/141	119/131	115/121
129/129	nd.	114/115	128/128	216/216	137/149	113/113	259/259	213/214	134/137	160/174	145/145	219/219	127/127	164/164	156/156	201/201	130/130	164/164	190/190	210/210	205/205	191/191	211/217	184/184	151/151	124/124	118/122	102/102	171/171	132/132	138/138	131/131	115/115
129/137	128/128	116/123	128/138	207/207	149/149	113/113	250/259	211/216	128/140	171/180	139/139	231/231	127/132	182/183	147/147	195/204	130/135	164/164	187/193	204/204	211/214	191/194	199/205	180/180	151/151	120/120	110/118	94/94	171/179	135/135	141/141	119/119	121/121
129/129	128/138	116/116	128/138	207/216	149/149	99/113	250/259	216/216	131/134	171/171	139/139	219/231	127/127	182/182	147/153	195/200	131/135	176/176	190/193	204/205	205/205	188/191	194/205	180/184	151/151	120/120	106/114	94/94	179/179	135/135	141/141	122/122	121/121
129/129	128/128	123/123	128/138	207/207	149/149	113/113	250/250	216/221	131/140	171/171	139/139	222/231	127/127	182/182	147/147	198/198	133/133	164/176	190/193	204/205	205/205	182/185	205/207	180/186	151/151	128/128	106/106	94/94	179/179	135/135	141/141	116/122	121/121
129/129	128/138	114/116	128/138	207/207	149/149	99/99	259/259	216/221	131/134	165/171	139/139	231/231	127/132	182/183	147/147	195/198	nd.	176/176	190/193	204/205	211/217	191/191	nd.	180/186	151/151	120/128	106/114	94/102	179/179	135/135	141/141	119/119	121/121
129/137	128/128	123/123	138/138	216/216	149/149	113/113	250/259	216/221	131/140	171/171	139/139	222/231	127/127	164/182	147/147	195/198	131/135	164/176	190/193	204/205	205/211	185/191	199/209	180/186	151/151	120/120	106/106	94/102	179/179	135/135	141/141	116/119	115/121
129/129	128/128	114/123	138/138	207/216	149/149	99/113	250/250	221/221	131/131	171/171	139/139	231/231	132/135	164/182	147/153	195/198	131/131	176/176	190/193	204/205	205/211	185/191	199/209	186/186	151/151	120/128	106/106	94/94	179/179	135/135	141/141	116/122	121/121
129/129	128/128	114/123	138/138	207/216	149/149	99/113	250/250	211/216	131/140	171/171	139/139	231/231	127/132	182/182	147/153	198/198	133/133	176/176	190/193	204/205	205/205	182/185	205/207	180/186	151/151	128/128	106/106	94/102	179/179	135/135	141/141	119/122	121/121
129/129	128/128	116/116	138/138	207/216	149/149	113/113	259/259	216/216	134/140	171/171	139/139	231/231	127/132	182/183	147/153	195/200	135/135	164/176	190/193	204/204	205/211	188/191	194/199	180/184	151/151	120/120	114/118	94/102	171/179	135/138	141/144	119/122	115/121
129/137	128/128	116/123	128/128	207/215	149/149	113/113	250/259	211/211	134/134	171/180	139/139	231/231	121/127	164/182	147/153	195/201	131/135	167/167	187/190	204/204	211/214	182/191	203/211	178/178	151/151	120/128	110/114	94/114	171/179	135/135	141/141	119/122	115/121
129/129	128/138	114/114	128/138	207/216	149/149	99/99	250/250	216/221	131/131	171/171	139/139	231/231	132/132	182/182	147/153	195/200	131/135	176/176	190/193	204/205	205/205	188/191	205/209	186/186	151/151	120/128	106/106	102/102	179/179	135/135	141/141	122/131	115/121
129/137	128/128	114/123	128/128	216/216	149/149	113/113	259/259	212/214	134/137	174/177	139/145	219/231	127/132	164/182	147/156	201/201	130/130	164/164	190/193	204/210	208/211	191/191	194/211	180/184	151/151	120/124	114/118	102/102	171/171	132/135	138/141	131/131	115/115
129/137	128/128	116/123	138/138	207/207	149/149	99/99	250/250	211/216	131/134	171/171	139/139	231/231	127/127	164/182	147/147	195/198	135/135	164/176	190/193	204/204	205/205	191/191	199/199	180/180	151/151	120/128	106/114	94/94	179/179	135/135	141/141	122/122	121/121
129/137	128/128	123/123	128/138	207/207	149/149	113/113	250/259	216/216	131/134	171/171	139/139	231/231	127/127	182/182	147/147	195/195	131/135	164/176	190/193	204/205	205/205	191/191	203/207	180/186	151/151	120/128	106/114	94/94	179/179	135/135	141/141	119/122	115/121
129/129	128/138	114/116	128/138	207/216	149/149	99/113	250/259	216/221	131/134	171/171	139/139	220/231	127/132	164/182	147/153	195/200	131/135	176/176	190/193	204/204	205/208	188/191	205/209	180/186	151/151	120/128	106/118	94/102	171/179	135/135	141/141	119/122	115/121
129/129	128/128	123/123	138/138	207/216	149/149	99/113	250/259	211/216	140/140	171/171	139/139	231/231	127/132	164/183	147/147	200/200	131/135	164/176	190/193	205/205	211/211	176/188	199/205	180/184	151/151	120/128	106/106	94/94	179/179	135/138	141/144	119/131	121/121
129/129	128/138	116/116	128/138	207/216	149/149	99/113	259/259	216/216	134/140	165/171	139/139	231/231	127/132	182/183	147/153	195/200	135/135	176/176	193/193	204/204	205/211	188/191	194/199	180/186	151/151	120/120	106/114	94/102	179/179	135/135	141/141	119/122	115/121
129/129	128/128	114/116	138/138	207/216	149/149	99/113	250/259	216/221	131/131	171/171	139/139	208/231	132/132	182/182	147/153	198/200	131/135	164/176	193/193	205/205	205/205	185/188	203/209	180/186	151/151	120/120	106/106	94/102	179/179	135/135	141/141	119/122	115/121
129/129	128/138	116/116	128/138	207/216	149/149	113/113	250/259	216/221	131/140	171/171	139/139	231/231	127/127	182/182	147/153	195/198	131/133	164/164	190/193	204/204	205/205	191/191	203/209	180/186	151/151	120/128	106/106	94/94	179/179	135/135	141/141	119/122	115/115
129/129	nd.	115/115	128/128	216/216	137/137	113/113	259/259	216/216	128/134	177/177	145/145	219/231	127/129	164/182	156/156	201/201	130/131	164/164	190/196	211/211	211/211	191/191	194/199	184/184	151/151	120/124	118/118	94/102	171/171	132/138	138/144	122/131	115/121
129/129	138/138	114/114	128/138	207/216	149/149	99/99	250/259	211/216	131/131	171/171	139/139	219/231	132/132	182/183	147/153	195/200	130/131	176/176	190/190	204/204	205/211	188/191	205/205	184/184	151/151	120/128	106/106	102/102	179/179	135/138	141/144	119/122	115/121
129/129	128/138	116/123	128/138	216/216	149/149	99/113	250/259	216/221	134/134	171/171	139/139	219/231	127/127	164/182	147/153	195/195	135/135	164/176	193/193	204/205	205/205	191/191	203/209	180/180	151/151	120/120	114/118	94/102	171/179	135/135	141/141	119/119	115/121
129/129	138/138	114/116	128/138	207/216	149/149	99/99	250/259	216/221	131/134	171/171	139/139	219/231	127/132	164/182	147/153	195/200	130/135	164/176	190/193	204/204	205/208	188/191	205/209	184/184	151/151	120/128	106/118	102/102	171/179	135/135	141/141	119/122	115/121
129/129	138/138	114/116/131	128/138	216/216	137/149	99/113	250/259	212/221	137/137	171/177/180	139/145	219/219	127/127	164/182	153/156	200/201/204	130/135	176/176	190/193	204/210	205/208/214	188/191	211/211	184/186	151/151	120/124	114/118	114/114	171/171	132/135	138/141	119/122	115/115
129/129	128/138	114/116	128/138	207/216	149/149	99/99	250/250	216/221	131/140	171/171	139/139	219/231	127/132	182/183	147/153	200/200	130/131	176/176	190/193	204/204	205/211	188/188	205/209	184/186	151/151	120/128	106/106	94/102	179/179	135/135	141/141	122/122	115/121
129/129	138/138	116/116	138/138	207/216	149/149	99/113	250/250	211/221	134/140	171/171	139/139	219/231	127/132	182/183	147/153	195/200	131/131	176/176	190/193	204/205	205/211	188/191	205/209	180/184	151/151	128/128	106/118	94/102	171/179	135/138	141/144	122/131	121/121
129/129	128/128	114/131	128/138	216/216	149/149	113/113	250/259	221/221	137/140	168/171	139/145	219/231	127/132	164/182	147/153	198/201	133/135	164/176	190/193	205/210	205/220	182/191	199/209	180/184	151/151	120/120	106/118	94/94	171/179	132/135	138/141	122/122	121/121
129/129	128/128	123/123	128/138	207/216	149/149	99/113	250/250	216/221	131/131	171/171	139/139	231/231	132/135	182/182	147/153	198/198	131/135	164/176	193/193	204/204	205/205	191/191	199/207	186/186	151/151	128/128	106/106	94/94	179/179	135/135	141/141	116/119	121/121
129/129	128/138	114/114	128/138	207/207	149/149	99/99	250/250	211/216	131/134	171/171	139/139	219/231	132/132	182/183	147/147	195/200	130/133	176/176	190/193	204/205	205/211	188/191	205/205	186/186	151/151	120/128	106/118	102/102	171/179	135/135	141/141	122/131	115/121
129/129	128/128	114/116	128/138	207/207	149/149	99/113	250/259	211/216	134/140	171/171	139/139	231/231	127/132	164/182	147/147	195/198	135/135	164/176	190/193	204/204	211/211	185/191	203/205	180/186	151/151	120/128	106/118	94/102	171/179	135/135	141/141	119/131	115/121
129/129	128/128	114/116	128/138	207/207	149/149	113/113	250/259	221/221	131/134	171/171	139/139	208/231	127/132	182/183	147/147	195/198	131/135	164/176	193/193	204/204	211/217	188/191	194/199	180/180	151/151	120/128	106/118	102/102	171/179	135/135	141/141	119/131	115/121
129/129	128/138	116/116	128/138	207/216	149/149	113/113	259/259	216/216	134/134	171/171	139/139	219/231	127/132	164/183	147/153	195/200	135/135	164/176	190/193	204/204	205/205	188/191	194/205	180/186	151/151	120/120	114/118	94/94	179/179	135/135	141/141	122/122	115/115
129/137	128/128	116/123	138/138	207/207	149/149	99/113	250/250	216/216	134/140	171/171	139/139	231/231	127/132	164/182	147/147	195/198	135/135	176/176	193/193	204/205	211/217	191/191	194/207	180/180	151/151	128/128	106/114	94/102	179/179	135/135	141/141	119/119	115/121
129/129	128/138	114/116	128/138	207/216	149/149	99/113	250/259	221/221	131/137	171/171	139/139	219/231	127/132	164/182	147/153	195/200	135/135	176/176	190/193	204/204	205/208	188/191	199/209	180/186	151/151	120/128	106/118	94/102	171/179	135/135	141/141	122/122	121/121
129/129	128/128	114/114	138/138	207/216	149/149	99/113	250/259	216/221	131/140	171/171	139/139	208/231	132/132	182/183	147/153	195/200	131/135	164/176	190/193	204/205	211/217	188/191	199/209	180/184	151/151	120/128	106/118	102/102	171/175	135/138	141/144	119/122	121/121
129/129	128/128	114/116	128/138	207/207	149/149	113/113	250/259	221/221	131/134	171/171	139/139	208/231	127/132	182/183	147/147	195/198	131/135	164/176	193/193	204/204	211/217	188/191	194/199	180/180	151/151	120/128	106/118	102/102	171/179	135/135	141/141	119/131	115/121
129/129	128/128	116/123	128/138	207/216	149/149	113/113	259/259	216/216	131/134	168/171	139/139	208/219	132/132	164/182	147/156	198/200	130/135	164/164	193/193	204/205	205/205	185/188	194/203	180/180	151/151	124/128	106/114	94/114	171/171	135/135	141/141	119/119	115/115
129/137	128/128	123/131	128/128	216/216	149/149	113/113	259/259	212/214	131/134	171/177	139/145	208/219	127/127	173/182	156/156	198/198	130/135	176/176	190/193	204/210	205/211	191/191	194/211	180/184	151/151	120/120	114/118	102/114	171/171	132/135	138/141	122/131	115/115
129/137	128/128	114/116	138/138	207/207	149/149	113/113	250/259	216/216	134/140	171/177	139/139	231/231	127/132	173/182	147/147	195/198	135/135	164/164	190/193	204/205	205/211	191/191	199/199	180/184	151/151	120/128	106/114	94/102	171/179	132/135	138/141	119/131	121/121
129/129	128/128	116/123	128/138	207/216	149/149	99/113	250/259	221/221	131/134	171/171	139/139	231/231	127/132	182/182	147/147	198/200	133/135	164/176	190/193	204/205	205/205	188/191	194/199	180/186	151/151	120/120	106/114	94/94	179/179	135/135	141/141	116/119	115/121
129/129	128/128	114/123	128/128	216/216	149/149	113/113	259/259	213/214	134/134	168/171	139/139	219/231	127/132	164/164	156/156	198/200	130/135	164/164	190/193	204/204	205/208	188/191	194/194	180/180	151/151	120/124	114/118	94/114	171/171	132/135	138/141	119/131	115/115
129/137	128/128	114/123	138/138	207/207	149/149	113/113	250/259	211/216	131/134	171/171	139/139	231/231	127/127	182/183	147/147	198/200	131/135	176/176	193/193	204/205	205/211	191/191	199/199	180/186	151/151	120/128	106/114	94/102	179/179	135/135	141/141	122/122	121/121
129/129	128/128	116/116	138/138	207/216	149/149	113/113	250/259	216/216	134/140	171/171	139/139	231/231	127/132	182/182	147/153	195/195	135/135	164/164	nd.	204/204	205/205	188/191	194/199	180/184	151/151	120/120	114/114	94/102	179/179	135/138	141/144	119/119	115/121
129/129	128/128	114/116	138/138	207/216	149/149	99/113	250/259	211/211	131/134	171/171	139/139	231/231	127/127	182/182	147/153	195/198	131/135	164/176	190/190	204/205	205/205	191/191	194/199	180/186	151/151	120/128	106/114	94/102	179/179	135/135	141/141	119/122	115/121
129/129	128/128	123/123	128/128	216/216	149/149	99/99	250/250	221/221	131/131	171/171	139/139	222/231	127/127	182/182	147/153	198/198	133/133	176/176	190/193	204/205	205/205	182/191	199/199	180/186	151/151	120/128	106/106	94/94	179/179	135/135	141/141	116/116	121/121
129/137	128/128	115/123	128/138	207/216	149/149	113/113	250/259	216/216	134/137	171/177	139/145	219/231	127/132	164/182	147/156	198/204	130/135	164/164	190/190	204/210	205/211	191/191	199/211	180/184	151/151	120/124	114/118	102/102	171/171	132/135	138/141	131/131	115/121
129/129	128/128	114/116	128/138	207/207	149/149	99/113	250/259	216/221	131/134	171/171	139/139	231/231	127/132	182/182	147/147	195/195	131/135	176/176	190/193	204/205	205/217	191/191	194/209	180/186	151/151	120/128	106/114	102/102	179/179	135/135	141/141	119/122	115/121
129/129	128/138	116/116	128/138	216/216	149/149	99/113	250/259	216/216	140/140	171/171	139/139	231/231	127/132	182/183	153/153	195/200	130/135	164/176	193/193	204/204	205/211	188/191	194/205	180/186	151/151	120/120	106/118	94/102	171/179	135/135	141/141	119/122	115/115
129/137	128/128	114/123	138/138	207/207	149/149	99/113	250/250	216/216	131/140	171/171	139/139	231/231	127/127	164/182	147/147	198/200	131/135	164/176	193/193	204/205	205/211	176/191	199/207	180/186	151/151	120/128	106/106	94/94	179/179	135/135	141/141	122/122	121/121
129/129	nd.	114/115	128/128	216/216	149/149	113/113	259/259	211/216	128/134	177/177	145/145	231/231	127/129	182/188	156/156	198/201	131/135	150/164	190/196	210/210	199/211	179/191	199/199	184/184	151/155	120/124	114/114	94/102	171/171	132/132	138/138	119/119	121/121
129/129	128/138	116/116	128/138	207/216	149/149	99/113	250/259	216/216	140/140	171/171	139/139	231/231	127/132	182/182	147/153	195/198	135/135	152/164	190/193	204/205	205/205	191/191	194/203	180/186	151/151	120/128	106/114	94/102	179/179	135/135	141/141	122/122	115/115
129/137	128/128	123/123	128/138	207/216	149/149	113/113	250/259	216/216	134/134	171/171	139/139	231/231	127/132	182/183	147/147	195/200	135/135	164/176	190/193	204/205	205/211	191/191	199/203	180/184	151/151	120/128	114/114	94/102	179/179	135/138	141/144	119/122	115/121
129/129	128/128	116/116	138/138	207/216	149/149	113/113	250/259	216/216	134/140	171/171	139/139	231/231	127/132	164/182	147/153	195/200	135/135	164/164	193/193	204/205	205/211	176/191	194/199	180/184	151/151	120/128	114/118	94/94	171/179	135/138	141/144	119/122	115/121
129/137	128/138	116/123	138/138	207/215	149/149	99/99	250/250	216/216	131/134	171/171	139/145	231/231	127/132	164/182	147/153	198/201	131/135	176/176	190/193	204/211	205/205	191/191	199/199	180/184	151/151	120/120	106/114	94/94	179/179	135/135	141/141	119/122	121/121
129/129	128/128	116/116	128/138	207/216	149/149	99/113	250/259	216/216	140/140	165/171	139/139	231/231	127/132	182/183	147/147	195/200	131/135	164/176	193/193	204/204	205/211	188/191	194/199	180/184	151/151	120/120	106/106	94/114	179/179	135/138	141/144	119/131	115/121
129/129	128/128	116/123	128/138	207/216	149/149	99/113	250/250	216/221	131/131	171/171	139/139	208/231	127/132	182/182	147/147	198/200	131/133	164/176	190/193	204/205	205/205	188/188	199/203	186/186	151/151	120/120	106/106	94/94	179/179	135/135	141/141	116/119	115/121
129/129	128/128	114/116	128/138	207/207	149/149	113/113	250/259	211/216	131/131	171/171	139/139	231/231	127/127	182/182	147/147	195/195	135/135	164/176	190/190	204/205	205/205	191/191	199/205	180/180	151/151	120/120	106/106	94/102	179/179	135/135	141/141	119/119	115/121
129/129	128/128	116/116	138/138	207/216	149/149	99/113	250/250	211/216	134/140	171/171	139/139	231/231	132/132	164/182	147/153	195/200	130/133	164/176	193/193	204/205	205/211	188/191	199/205	180/184	151/151	120/120	106/114	94/102	179/179	135/138	141/144	122/131	121/121
129/137	128/128	116/123	138/138	207/216	149/149	113/113	250/250	216/216	134/140	171/171	139/139	231/231	127/132	182/182	147/153	195/200	130/135	164/176	190/193	204/204	205/217	188/191	194/194	180/180	151/151	120/128	114/114	94/102	179/179	135/135	141/141	119/122	115/115
129/137	128/128	123/135	128/138	207/215	149/149	113/113	259/259	216/216	134/134	180/180	139/139	231/231	127/127	164/182	147/153	195/195	131/135	167/176	187/187	204/204	205/211	182/185	207/211	180/186	151/151	120/120	110/122	94/94	179/179	132/135	138/141	122/131	115/121
129/129	138/138	116/116	138/138	207/216	149/149	99/113	250/259	216/221	131/140	171/171	139/139	231/231	127/132	164/182	147/153	198/200	135/135	164/176	193/193	204/204	205/205	188/191	199/209	184/186	151/151	120/128	106/106	94/94	179/179	135/135	141/141	119/122	121/121
129/129	128/138	116/116	128/138	207/216	149/149	99/113	250/250	216/216	140/140	171/171	139/139	231/231	127/132	173/182	147/147	195/200	135/135	152/164	193/193	204/205	205/211	188/191	194/199	180/186	151/151	120/120	106/106	102/102	179/179	135/135	141/141	116/119	115/121
129/129	128/128	114/116	128/138	207/216	149/149	113/113	250/250	216/216	134/140	171/171	139/145	231/231	nd.	173/182	147/156	201/201	130/135	164/164	193/193	204/204	205/211	191/191	199/211	180/184	151/151	124/128	106/114	94/102	171/179	132/135	138/141	119/119	115/121
129/129	128/138	116/116	128/138	207/216	137/149	113/113	259/259	216/216	134/134	171/177	139/139	231/231	127/127	182/182	147/153	195/200	135/135	176/176	190/193	204/204	205/205	188/188	194/205	180/186	151/151	120/120	114/118	94/102	171/179	135/135	141/141	122/131	115/115
129/129	128/138	116/116	138/138	207/216	149/149	99/113	250/259	216/221	134/140	165/171	139/139	231/231	127/132	182/183	147/147	200/200	131/135	164/176	193/193	204/204	205/211	188/188	199/209	180/186	151/151	120/120	106/114	94/102	179/179	135/135	141/141	119/122	121/121
129/129	128/128	116/123	128/138	216/216	149/149	99/113	250/259	221/221	131/134	171/171	139/139	231/231	127/132	182/182	147/153	195/198	133/135	164/176	190/193	204/204	205/205	182/191	194/199	180/186	151/151	120/128	106/114	94/102	179/179	135/135	141/141	116/119	115/121
129/129	128/128	116/123	128/138	207/216	149/149	99/113	250/259	221/221	131/134	171/171	139/139	231/231	127/127	182/183	147/153	198/200	133/135	176/176	190/190	204/204	205/211	188/191	194/199	180/186	151/151	120/128	106/114	94/94	179/179	135/135	141/141	116/122	115/121
129/129	128/128	123/123	128/138	207/216	149/149	113/113	250/259	216/221	131/140	171/171	139/139	222/231	127/127	182/182	147/147	195/198	133/135	176/176	190/193	204/205	205/205	182/191	194/199	184/186	151/151	120/120	106/118	94/102	171/179	135/138	141/144	116/119	115/121
129/137	128/128	116/123	128/128	215/215	149/149	113/113	250/259	216/216	134/140	171/171	139/139	231/231	127/127	182/182	147/153	195/200	135/135	164/167	190/193	204/205	205/214	176/182	203/211	178/180	151/151	120/120	106/114	94/114	171/179	135/135	141/141	122/122	115/115
129/129	128/138	116/116	128/138	207/216	149/149	113/113	250/259	216/216	140/140	171/171	139/139	231/231	127/132	182/183	147/153	195/200	130/135	176/176	190/193	204/204	205/211	188/191	194/205	180/186	151/151	120/120	106/118	102/102	171/179	135/135	141/141	119/122	115/115
129/129	128/128	116/123	128/128	207/216	149/149	99/113	250/250	216/216	131/140	171/171	139/139	219/231	127/127	182/182	147/147	195/200	131/135	164/164	193/193	204/205	205/205	188/191	203/205	180/184	151/151	120/120	106/106	94/94	179/179	135/135	141/141	122/122	115/121
129/129	128/128	115/135	128/128	216/216	137/149	113/113	259/259	213/214	134/137	160/174	145/145	219/219	127/127	164/164	156/156	201/201	130/130	164/164	190/193	210/210	205/211	182/191	211/217	184/184	151/151	124/124	114/122	102/114	171/171	132/132	138/138	119/119	115/115
129/137	128/128	114/123	128/138	216/216	149/149	113/113	250/259	216/221	131/134	171/171	139/139	231/231	127/132	182/182	147/153	195/198	135/135	164/176	190/193	204/205	205/205	185/191	203/209	180/180	151/151	120/128	106/114	94/94	179/179	135/135	141/141	122/122	115/121
129/129	128/128	114/123	128/138	207/216	149/149	99/113	250/250	211/216	140/140	171/171	139/139	231/231	127/132	164/183	147/147	195/200	133/135	164/176	193/193	204/205	211/211	188/191	203/205	180/186	151/151	120/128	106/106	94/102	179/179	135/135	141/141	122/131	115/121
129/129	128/128	114/116	138/138	207/216	149/149	113/113	250/250	211/211	131/134	171/171	139/139	231/231	127/127	164/182	147/153	195/200	133/135	164/176	190/193	204/204	205/211	185/191	194/199	184/186	151/151	128/128	106/114	94/94	179/179	135/135	141/141	119/122	115/121
129/129	128/138	116/123	128/138	207/207	149/149	99/113	250/259	211/221	131/131	171/171	139/139	208/231	127/132	182/182	147/147	195/198	131/135	176/176	190/193	204/205	205/217	191/191	199/199	186/186	151/151	128/128	106/106	94/102	179/179	135/135	141/141	119/122	121/121
129/129	128/128	114/116	138/138	207/216	149/149	99/113	250/259	211/211	131/140	171/171	139/139	231/231	127/127	182/182	147/153	195/198	131/135	164/176	190/193	204/205	205/205	191/191	194/199	180/186	151/151	120/128	106/106	94/102	179/179	135/135	141/141	119/119	115/121
129/137	128/128	116/123	138/138	207/207	149/149	99/99	250/259	216/216	131/134	171/171	139/139	231/231	127/132	164/182	147/147	195/198	131/135	176/176	190/193	205/205	205/211	191/191	194/207	180/180	151/151	120/128	106/114	94/102	179/179	135/135	141/141	119/122	115/121
129/129	128/128	114/116	128/138	207/207	149/149	99/113	250/250	216/216	131/140	171/171	139/139	231/231	127/132	182/182	147/147	195/195	131/135	164/176	193/193	204/205	205/205	191/191	199/205	180/186	151/151	120/120	106/118	94/102	171/179	135/135	141/141	119/122	115/121
129/129	128/138	116/116	138/138	207/216	149/149	113/113	250/259	221/221	131/134	171/171	139/139	222/231	127/132	164/182	147/153	198/200	133/135	164/176	193/193	204/204	205/211	176/191	194/209	180/186	151/151	128/128	106/114	94/94	179/179	135/135	141/141	122/131	115/121
129/129	128/128	114/123	128/138	207/207	149/149	113/113	250/250	221/221	131/134	171/171	139/139	231/231	132/135	182/182	147/147	195/195	131/135	164/176	190/193	204/204	205/205	191/191	194/209	180/186	151/151	120/120	106/114	94/102	179/179	135/135	141/141	119/119	115/121
129/129	138/138	116/116	138/138	207/215	149/149	99/113	250/259	216/216	134/134	171/171	139/145	231/231	127/132	182/183	147/153	195/195	135/135	164/176	190/193	205/211	205/211	191/191	199/199	184/184	151/151	120/128	114/118	94/102	171/179	135/138	141/144	116/119	121/121
129/129	128/128	114/114	128/138	207/207	149/149	99/113	250/250	216/216	131/134	171/171	139/139	231/231	127/127	182/183	147/147	195/198	131/135	164/176	193/193	204/204	205/211	191/191	199/203	184/186	151/151	120/128	106/114	94/102	179/179	135/138	141/144	116/122	115/121
129/129	128/128	116/123	138/138	207/207	149/149	99/113	250/259	211/216	131/134	171/171	139/139	231/231	127/132	164/182	147/147	198/200	133/135	164/176	193/193	205/205	205/211	185/191	199/199	180/186	151/151	120/128	106/114	94/94	179/179	135/135	141/141	119/122	121/121
129/129	128/138	116/123	128/138	207/207	149/149	113/113	250/250	216/216	131/134	171/171	139/139	231/231	127/132	182/182	147/147	195/195	131/135	164/176	193/193	204/204	205/205	191/191	194/205	186/186	151/151	120/128	106/114	94/102	179/179	135/135	141/141	119/119	115/115
129/129	128/138	114/116	138/138	207/216	149/149	99/113	250/259	211/216	131/134	171/171	139/139	231/231	127/132	182/183	147/153	195/200	130/135	164/176	190/193	205/205	205/211	176/191	199/205	180/186	151/151	128/128	106/114	94/102	179/179	135/135	141/141	119/122	121/121
129/129	128/128	116/123	138/138	207/207	149/149	113/113	250/250	216/216	131/140	171/171	139/139	231/231	127/127	164/182	147/147	198/200	133/135	164/176	190/190	204/205	205/211	176/191	199/207	180/186	151/151	120/128	106/106	94/94	179/179	135/135	141/141	122/122	121/121
129/129	138/138	116/116	138/138	207/215	149/149	99/113	259/259	216/216	134/137	171/171	139/145	219/231	127/132	182/183	147/153	195/195	130/135	164/164	193/193	205/211	205/211	191/191	199/199	184/184	151/151	120/128	106/114	94/102	179/179	135/138	141/144	119/122	121/121

129/129	128/128	114/123	128/138	207/216	149/149	99/113	259/259	216/216	131/134	171/171	139/139	231/231	127/132	164/182	147/153	195/200	135/135	176/176	193/193	204/205	205/208	188/191	194/205	180/186	151/151	120/120	106/118	94/94	171/179	135/135	141/141	116/122	115/115
129/137	138/138	114/116	138/138	207/216	149/149	99/113	250/259	216/221	140/140	171/177	139/139	231/231	127/132	173/182	147/153	195/200	131/135	164/176	193/193	204/204	205/211	191/191	199/205	184/184	151/151	120/120	106/106	94/102	179/179	132/135	138/141	119/122	121/121
129/129	128/138	116/116	138/138	207/216	149/149	99/113	250/259	216/216	134/134	171/171	139/139	219/231	127/127	182/183	147/153	195/195	131/135	164/176	190/193	204/204	205/211	188/191	194/199	180/180	151/151	120/128	114/118	94/102	171/179	135/135	141/141	116/119	115/121
129/137	128/128	123/123	128/138	216/216	149/149	99/113	250/250	216/221	131/131	165/171	139/139	231/231	nd.	182/183	147/153	198/200	133/135	176/176	193/193	204/204	205/211	188/191	203/205	180/184	151/151	120/128	106/106	94/102	179/179	135/138	141/144	116/119	115/121
129/129	138/138	114/116	138/138	207/207	149/149	99/113	250/250	211/216	131/134	171/171	139/139	231/231	127/132	182/182	147/147	195/201	130/131	176/176	190/193	204/205	205/205	191/191	199/205	184/186	151/151	120/128	106/114	94/94	179/179	135/135	141/141	119/122	121/121
129/137	128/128	114/123	128/138	207/216	149/149	99/113	250/259	216/216	131/140	171/171	139/139	231/231	132/132	164/182	147/153	195/198	131/135	164/176	190/193	204/205	205/211	176/191	205/207	180/184	151/151	120/120	106/118	94/102	171/179	135/135	141/141	119/122	115/121
129/129	128/138	114/116	138/138	207/216	149/149	113/113	250/259	216/221	134/140	171/171	139/139	231/231	127/132	182/182	147/153	200/200	131/131	176/176	193/193	204/205	205/205	188/191	199/205	184/186	151/151	120/128	106/114	94/102	179/179	135/135	141/141	119/119	121/121
129/129	128/128	123/123	138/138	207/216	149/149	99/113	250/259	216/216	131/140	171/171	139/139	231/231	127/127	182/183	147/153	195/198	131/135	164/176	190/193	204/204	205/211	191/191	194/207	184/186	151/151	120/128	106/118	94/102	171/179	135/138	141/144	119/119	115/121
129/129	128/128	114/114	138/138	207/207	149/149	113/113	250/259	211/216	131/140	171/171	139/139	231/231	127/132	182/183	147/147	195/200	131/135	164/176	193/193	204/204	205/211	185/188	194/199	180/186	151/151	120/128	106/118	94/94	171/179	135/138	141/144	119/122	115/121
129/129	138/138	116/116	128/138	216/216	149/149	99/113	250/250	213/213	140/140	165/171	139/139	231/231	127/132	182/182	147/153	200/200	131/135	164/176	193/193	204/204	205/205	176/188	194/194	184/186	151/151	120/128	106/118	94/102	171/179	135/138	141/144	119/122	115/115
129/129	138/138	116/116	128/138	207/216	149/149	99/113	250/259	216/216	131/134	171/171	139/139	231/231	127/132	182/182	147/153	195/195	135/135	164/176	190/193	204/204	205/205	191/191	194/205	184/184	151/151	120/128	106/114	94/102	179/179	135/138	141/144	119/122	115/115
129/137	128/128	114/116	138/138	207/216	149/149	99/113	250/250	216/221	131/134	171/171	139/139	231/231	127/132	182/182	147/153	200/200	131/135	164/176	190/193	204/205	205/205	176/188	199/209	180/186	151/151	120/120	106/114	94/102	179/179	135/135	141/141	119/119	121/121
129/129	138/138	114/114	138/138	207/216	149/149	99/113	250/259	211/211	131/134	171/171	139/139	231/231	127/132	182/183	147/153	195/195	130/135	176/176	190/193	204/205	205/211	191/191	194/205	184/184	151/151	120/128	106/114	94/102	179/179	135/138	141/144	119/122	115/121
129/129	138/138	114/116	128/128	207/216	149/149	99/99	259/259	216/216	131/140	165/171	139/145	231/231	127/132	164/182	147/153	195/200	130/135	176/176	193/193	204/211	205/205	188/191	194/205	184/186	151/151	120/124	106/106	94/102	179/179	132/135	138/141	119/122	115/115
129/129	128/138	116/123	128/138	216/216	149/149	99/113	250/259	216/216	131/140	165/171	139/139	231/231	127/132	164/182	147/153	195/200	130/131	176/176	190/193	204/205	205/211	176/191	194/199	180/184	151/151	124/128	106/106	94/94	179/179	135/135	141/141	119/122	115/121
129/137	128/138	116/123	128/128	216/216	149/149	113/113	250/259	216/216	140/140	171/171	139/139	231/231	127/132	164/183	147/147	195/200	135/135	164/176	190/193	204/205	211/211	188/191	194/203	180/186	151/151	120/128	106/106	94/102	179/179	135/135	141/141	119/122	115/115
129/137	128/128	114/116	128/138	207/216	149/149	99/113	250/259	211/216	140/140	171/171	139/139	231/231	127/132	164/182	147/147	198/200	133/135	164/176	193/193	205/205	205/205	176/185	203/205	180/186	151/151	120/128	106/106	94/102	179/179	135/135	141/141	122/122	115/121
129/129	128/128	116/116	138/138	207/216	149/149	99/113	250/250	211/216	140/140	171/171	139/139	231/231	127/132	164/182	147/153	195/200	131/135	164/176	190/190	204/204	205/211	188/191	199/205	180/184	151/151	120/120	106/106	94/94	179/179	135/138	141/144	119/119	121/121
129/129	128/128	116/116	128/128	215/215	149/149	99/113	250/250	213/214	134/140	171/171	139/139	231/231	121/127	164/182	147/147	200/201	131/135	167/176	187/193	204/204	205/211	188/191	194/211	178/184	151/151	120/120	106/110	94/102	179/179	135/138	141/144	119/119	115/115
129/137	128/138	116/123	128/128	215/215	149/149	113/113	250/259	213/214	134/140	165/171	139/139	231/231	121/127	182/183	147/153	195/198	135/135	164/176	187/193	204/204	211/214	182/191	194/211	180/186	151/151	120/120	106/114	94/102	171/179	135/135	141/141	122/122	115/115
129/129	128/128	116/116	128/138	215/215	149/149	99/113	250/250	221/221	134/140	165/180	139/139	231/231	127/132	164/182	147/153	195/200	131/131	167/176	190/193	204/204	205/211	182/188	209/211	178/186	151/151	120/120	106/110	102/114	179/179	135/138	141/144	119/119	115/121
129/137	128/128	114/123	128/128	207/207	149/149	99/113	250/259	216/216	131/134	171/171	139/139	231/231	127/132	173/182	147/147	195/201	130/131	164/176	190/193	204/204	205/211	191/191	194/205	180/184	151/151	124/128	106/114	102/102	171/179	135/135	141/141	119/122	115/115
129/129	128/138	114/116	128/138	207/216	137/149	99/99	250/259	216/221	131/131	171/177	139/139	231/231	132/132	182/182	147/153	198/200	131/135	176/176	193/193	204/204	205/205	185/188	205/209	186/186	151/151	120/128	106/118	94/102	171/179	135/135	141/141	119/122	115/121
129/137	138/138	114/116	138/138	207/216	149/149	99/113	250/259	216/221	140/140	171/177	139/139	231/231	127/132	182/183	147/153	198/198	131/135	152/164	190/193	204/205	205/211	191/191	199/209	184/184	151/151	120/120	106/106	94/102	179/179	132/135	138/141	119/122	121/121
129/137	128/128	116/123	128/138	207/216	149/149	113/113	250/259	216/216	134/140	171/171	139/145	231/231	127/132	173/182	147/156	195/201	135/135	164/164	190/193	204/210	211/211	191/191	194/199	180/184	151/151	120/120	106/114	94/102	171/179	135/135	141/141	122/122	121/121
129/129	128/128	114/116	128/138	207/215	137/149	99/113	250/250	216/216	131/134	171/177	139/145	231/231	127/132	164/182	147/153	195/195	131/135	164/176	190/190	204/211	205/205	191/191	199/205	184/186	151/151	120/128	106/118	102/102	171/179	132/135	138/141	122/131	115/121
137/137	128/128	114/123	128/138	207/216	149/149	113/113	250/259	216/216	134/140	171/177	139/139	231/231	127/132	182/182	147/156	198/201	135/135	164/164	190/190	204/204	205/211	191/191	194/199	180/184	151/151	120/120	106/114	102/102	171/179	132/135	138/141	122/122	115/121
129/137	128/128	114/123	128/138	207/207	149/149	99/113	250/250	216/216	131/134	171/177	139/139	231/231	127/132	182/182	147/147	195/198	130/131	164/176	193/193	204/204	205/211	191/191	199/205	180/180	151/151	120/124	106/114	102/102	171/179	135/135	141/141	119/122	115/121
129/137	128/128	115/123	128/138	207/216	149/149	113/113	259/259	216/216	134/137	174/177	139/145	219/231	127/127	164/182	147/156	201/204	130/135	164/164	190/193	204/210	205/211	191/191	199/211	180/184	151/151	120/124	114/122	102/102	171/171	132/135	138/141	122/122	115/121
129/129	138/138	114/116	138/138	207/216	149/149	99/113	250/259	216/216	134/140	171/177	139/139	219/231	127/132	173/182	147/153	198/200	135/135	164/176	193/193	204/204	211/211	188/191	194/199	184/184	151/151	120/120	106/114	102/114	171/179	132/135	138/141	122/131	115/121
129/129	128/128	123/123	128/138	216/216	149/149	99/113	250/259	221/221	131/134	171/171	139/139	222/231	127/127	182/183	147/153	198/200	133/135	176/176	190/193	205/205	205/211	182/188	194/199	184/186	151/151	120/120	106/114	94/94	179/179	135/138	141/144	119/122	115/121
129/129	128/138	116/123	128/128	216/216	149/149	99/113	250/250	216/216	140/140	165/171	139/139	231/231	127/132	164/183	147/147	195/200	131/135	164/176	190/190	204/205	211/211	188/191	194/203	180/186	151/151	120/128	106/106	94/102	179/179	135/135	141/141	119/119	115/121
129/129	128/128	116/123	128/138	216/216	149/149	99/113	250/259	216/221	131/140	171/171	139/139	231/231	127/132	182/182	147/153	195/198	133/135	176/176	190/193	204/204	205/205	182/188	199/199	180/186	151/151	120/120	106/118	94/102	171/179	135/135	141/141	119/122	121/121
129/129	128/128	116/123	128/138	207/216	149/149	99/113	250/259	221/221	131/140	171/171	139/139	231/231	127/132	182/182	147/147	198/200	133/135	176/176	193/193	204/205	205/205	182/188	194/199	180/186	151/151	120/128	106/118	94/102	171/179	135/135	141/141	122/131	115/121
129/129	128/138	116/116	128/138	207/216	149/149	113/113	250/250	216/216	134/140	171/171	139/139	231/231	127/132	182/182	147/147	195/200	135/135	164/164	190/193	205/205	205/205	176/191	199/203	180/186	151/151	120/120	106/114	94/102	179/179	135/135	141/141	122/131	115/121
129/129	128/128	116/116	138/138	207/216	149/149	99/113	259/259	216/216	140/140	171/171	139/139	231/231	127/132	182/182	147/153	195/200	131/135	176/176	190/193	204/204	205/205	188/191	194/199	180/180	151/151	120/120	106/118	94/94	171/179	135/135	141/141	122/131	115/121
129/129	128/128	116/116	138/138	207/207	149/149	99/113	250/259	216/216	131/140	171/171	139/139	231/231	127/132	182/182	147/147	195/200	135/135	176/176	193/193	204/204	205/205	188/191	194/199	180/180	151/151	120/120	106/106	94/102	179/179	135/135	141/141	119/122	115/121
129/129	128/138	116/123	128/128	216/216	149/149	99/113	250/250	221/221	131/140	171/171	139/139	231/231	127/132	173/182	147/147	195/200	135/135	164/164	190/193	204/204	205/211	188/191	194/199	186/186	151/151	120/120	106/114	102/102	179/179	135/135	141/141	116/122	115/121
129/129	128/128	116/116	128/138	207/207	149/149	99/113	250/259	216/221	131/140	165/171	139/139	231/231	127/132	182/183	147/153	195/200	131/135	164/176	193/193	204/204	205/211	188/191	199/199	180/184	151/151	120/120	106/106	94/102	179/179	135/138	141/144	119/122	121/121
129/129	128/128	114/116	128/138	216/216	137/149	113/113	259/259	212/212	134/137	171/180	139/145	219/231	127/127	164/182	153/156	195/204	130/135	164/164	190/190	204/210	205/214	191/191	194/211	180/184	151/151	120/124	114/114	94/114	171/179	132/135	138/141	119/119	115/115
129/129	128/128	116/123	128/138	207/216	149/149	113/113	250/250	221/221	131/134	171/171	139/139	231/231	132/132	182/182	147/153	200/200	130/131	164/176	193/193	204/205	205/205	188/188	194/199	180/186	151/151	120/120	106/114	94/102	179/179	135/135	141/141	119/119	115/121
129/129	138/138	116/116	128/138	207/216	149/149	113/113	250/250	221/221	131/140	171/171	139/139	208/231	127/132	182/183	147/153	195/195	131/135	164/176	190/193	204/204	211/217	191/191	194/199	186/186	151/151	120/128	106/118	102/102	171/175	135/135	141/141	119/122	115/121
129/129	128/128	114/116	128/128	216/216	149/149	113/113	259/259	212/216	134/137	180/180	145/145	219/231	127/127	164/183	156/156	195/204	130/135	164/176	190/190	210/210	211/214	191/191	199/211	180/184	151/151	120/124	114/114	94/114	171/179	132/135	138/141	119/122	115/115
129/129	128/128	114/116	128/138	207/216	137/149	113/113	259/259	212/212	134/137	171/180	145/145	219/231	127/127	164/182	147/156	195/204	130/135	150/164	190/190	210/210	211/214	191/191	194/199	180/184	151/151	120/124	114/114	102/114	171/179	132/135	138/141	122/122	115/115
129/129	128/128	116/116	128/138	216/216	149/149	113/113	250/250	221/221	131/140	171/171	139/139	231/231	127/132	164/182	147/153	195/198	133/135	164/176	190/193	204/204	205/211	188/191	194/199	180/186	151/151	120/120	106/106	94/102	179/179	135/135	141/141	119/122	115/121
129/129	128/128	114/114	128/138	207/207	149/149	99/113	259/259	221/221	131/140	165/171	139/139	231/231	127/132	182/182	147/147	195/200	131/135	164/176	190/193	204/204	205/205	188/191	194/199	180/184	151/151	120/120	106/106	94/102	179/179	135/138	141/144	119/122	115/121
129/129	128/128	116/116	128/138	216/216	149/149	113/113	259/259	213/213	134/140	171/171	139/139	231/231	127/132	182/182	147/153	195/201	135/135	164/176	190/190	204/204	205/205	188/191	194/194	180/184	151/151	120/124	106/114	94/114	179/179	135/138	141/144	119/122	115/115
129/129	128/128	116/116	128/138	207/215	149/149	99/113	250/259	213/214	134/140	171/180	139/139	231/231	127/132	164/183	147/153	195/200	131/135	164/167	187/190	204/204	211/211	188/191	194/211	180/184	151/151	120/128	110/118	94/102	171/179	135/135	141/141	119/122	115/115
129/129	138/138	116/116	138/138	207/216	149/149	99/113	250/259	216/216	134/140	171/171	139/139	219/231	127/132	182/182	147/153	195/195	130/135	176/176	190/193	204/204	205/205	191/191	194/199	184/186	151/151	120/120	114/118	94/102	171/179	135/138	141/144	116/119	115/121
129/137	128/128	114/123	128/138	207/216	137/149	113/113	250/250	216/216	134/134	171/177	139/139	220/231	127/127	164/182	147/153	195/201	130/135	164/164	190/193	204/205	205/205	188/191	199/211	180/184	151/151	120/124	114/118	94/110	171/179	132/135	138/141	119/119	115/121
129/129	128/128	116/116	128/138	207/216	149/149	99/113	250/259	221/221	131/134	171/171	139/139	231/231	127/132	182/182	147/153	195/195	135/135	164/176	193/193	204/204	205/205	191/191	194/199	180/180	151/151	120/120	106/114	94/102	179/179	135/135	141/141	119/119	115/121
129/129	128/128	116/116	128/138	207/207	149/149	99/113	250/250	221/221	140/140	171/171	139/139	231/231	132/132	164/182	147/147	195/200	130/135	164/176	193/193	204/204	205/211	188/191	194/199	180/180	151/151	120/120	106/106	102/102	179/179	135/135	141/141	119/122	115/121
129/129	128/128	114/114	128/138	207/207	149/149	99/113	250/259	216/221	140/140	171/171	139/139	231/231	132/132	182/182	147/147	195/195	135/135	164/176	193/193	204/204	205/205	191/191	199/199	180/184	151/151	120/120	106/114	94/102	179/179	135/138	141/144	119/122	121/121

130	131	132	133	134	135	136	137	138	139	140	141	142	143	144	145	146	147	148	149	150	151	152	153	154	155	156	157	158	159	160	161	162	163
	*	*	**		**	*	*	*		*	**		**			**	*	*		*	*	*	*		*	*	*	*	*	*	*		*
SSR08B13	SSR08B15	SSR08B20	SSR08B21	SSR08B22	SSR08B25	SSR08B27	SSR08B28	SSR08B29	SSR08B31	SSR08B32	SSR08B35	SSR08B38	SSR08B40	SSR08B44	SSR08B47	SSR08B54	SSR08B60	SSR08B62	SSR08B64	SSR08B66	SSR08B68	SSR08B75	SSR08B78	SSR08B82	SSR08B84	SSR08B85	SSR08B88	SSR08B90	SSR08B92	SSR08B95	SSR11A06	SSR11B14	SSR11A27
0	0	0	0	0	0	0	0	0	0	0	0	0	0	0	1	1	0	1	0	0	0	0	0	0	0	0	1	0	0	0	1	0	0
109/112	89/95	146/151	117/126	133/139	115/120	96/108	184/184	165/168	117/126	92/105	149/164	92/95	174/174	94/97	99/102	182/182	125/129	262/269	133/142	96/96	110/110	176/185	131/137	168/177	145/151	185/191	265/277	170/170	130/130	152/164	255/255	230/235	222/233
112/112	89/89	146/153	115/117	133/133	115/122	96/108	180/184	159/165	117/117	92/105	146/149	92/92	170/174	94/94	99/99	178/182	125/125	262/274	136/142	96/96	110/110	185/185	131/131	165/165	145/145	179/179	265/277	170/170	135/135	161/161	240/255	235/238	233/233
112/118	89/95	143/151	117/117	133/133	112/120	96/99	180/180	159/165	117/123	92/96	149/149	92/92	170/170	94/94	99/99	178/178	125/129	262/274	133/142	96/96	110/110	180/185	131/131	165/177	145/145	185/191	265/268	170/170	127/130	161/161	240/255	230/235	222/233
112/112	89/89	146/146	117/117	133/133	115/115	96/99	180/184	162/165	117/120	92/96	149/149	92/92	170/174	94/94	99/99	178/182	125/125	274/280	133/142	96/113	110/110	185/185	131/131	162/177	145/145	179/185	265/268	170/170	127/127	152/152	255/255	238/238	233/233
112/118	95/95	148/150	115/117	133/133	117/119	96/96	180/180	159/168	117/126	92/92	149/152	89/95	170/170	91/97	96/102	178/178	125/129	262/269	142/142	96/113	110/110	176/185	131/131	165/168	145/145	191/191	265/265	170/170	138/138	164/164	240/255	235/244	222/233
115/118	89/89	145/146	115/117	133/133	114/115	96/96	175/175	159/159	120/126	82/92	143/146	89/92	165/165	91/94	96/99	173/173	118/129	269/274	136/145	96/96	110/116	176/189	131/131	162/162	145/145	179/191	255/265	166/170	132/132	152/158	255/255	230/230	233/233
112/118	89/95	151/153	117/126	133/136	120/122	96/105	180/184	159/171	117/126	92/102	149/164	89/92	170/174	91/94	96/99	178/182	125/129	262/274	142/142	96/108	110/123	176/185	131/134	165/168	145/145	191/194	265/274	170/170	130/135	161/164	240/255	235/235	222/233
112/112	95/101	150/153	117/123	136/139	119/122	102/105	179/184	159/171	117/117	99/102	149/161	89/95	169/174	91/97	96/102	177/182	125/129	262/269	136/142	96/108	110/123	185/185	134/137	162/165	145/151	191/194	271/274	170/170	135/138	161/164	255/255	230/235	222/233
118/118	95/101	153/153	117/126	136/136	122/122	96/96	180/180	159/159	117/126	92/92	149/164	89/113	170/170	91/115	96/119	178/178	129/129	269/274	142/142	96/113	110/110	176/193	134/134	168/177	151/151	191/191	265/265	166/170	132/138	164/164	255/255	235/235	233/233
118/118	95/101	153/153	117/126	136/136	122/122	96/96	180/180	159/159	117/126	92/92	149/164	89/113	170/170	91/115	96/119	178/178	129/129	269/274	142/142	96/113	110/110	176/193	134/134	168/177	151/151	191/191	265/265	166/170	132/138	164/164	255/255	235/235	233/233
118/118	95/101	153/153	117/126	136/136	122/122	96/96	180/180	159/159	117/126	92/92	149/164	89/113	170/170	91/115	96/119	178/178	129/129	269/274	142/142	96/113	110/110	176/193	134/134	168/177	151/151	191/191	265/265	166/170	132/138	164/164	255/255	235/235	233/233
118/118	95/101	153/153	117/126	136/136	122/122	96/96	180/180	159/159	117/126	92/92	149/164	89/113	170/170	91/115	96/119	178/178	129/129	269/274	142/142	96/113	110/110	176/193	134/134	168/177	151/151	191/191	265/265	166/170	132/138	164/164	255/255	235/235	233/233
118/118	89/98	153/153	120/120	133/139	122/122	96/96	180/184	159/159	114/117	92/92	158/164	89/95	170/174	91/97	96/102	178/182	118/133	262/262	142/142	96/96	110/110	193/193	131/137	168/168	145/145	200/200	265/265	166/166	138/138	164/164	255/255	235/235	233/233
112/112	95/101	151/153	109/117	133/136	120/122	96/96	184/184	159/159	126/126	92/92	146/149	89/110	174/174	91/112	96/117	182/182	129/129	263/274	142/142	96/113	110/110	176/176	131/134	168/177	151/151	191/200	265/265	166/170	127/138	152/164	255/255	235/235	233/233
112/118	89/95	143/155	120/120	133/133	112/124	102/108	184/207	159/165	117/126	99/105	152/155	92/98	174/197	94/100	99/105	182/206	125/133	257/274	133/142	96/113	110/110	176/180	131/131	168/177	145/151	185/191	271/277	166/170	127/138	161/164	255/255	230/235	216/233
112/118	95/95	151/153	115/117	136/139	120/122	96/96	180/184	159/159	117/117	92/92	149/152	89/95	170/174	91/97	96/102	178/182	129/129	262/262	130/142	96/113	110/110	193/193	134/137	168/177	151/151	185/191	265/265	170/170	138/138	164/164	255/255	235/235	222/233
112/118	95/95	151/153	115/117	136/139	120/122	96/96	180/184	159/159	117/117	92/92	149/152	89/95	170/174	91/97	96/102	178/182	129/129	262/262	130/142	96/113	110/110	193/193	134/137	168/177	151/151	185/191	265/265	170/170	138/138	164/164	255/255	235/235	222/233
112/112	89/89	143/143	115/120	133/133	112/112	99/99	175/180	165/165	117/117	96/96	146/152	92/92	165/170	94/94	99/99	173/178	125/125	274/274	133/136	96/108	110/123	185/185	131/131	177/177	145/145	179/179	269/269	170/170	127/135	152/152	255/255	238/238	233/233
112/118	95/101	148/153	117/126	133/139	117/122	96/102	183/184	159/168	117/117	92/99	155/164	95/98	173/174	97/100	102/105	181/182	125/129	262/269	136/142	96/96	110/110	185/185	131/137	165/168	145/151	185/191	265/271	166/170	130/138	152/164	255/255	235/235	222/233
112/118	95/98	145/150	115/115	133/133	114/119	96/102	175/183	159/168	111/126	92/99	146/155	89/98	165/173	91/100	96/105	173/181	129/129	257/274	133/145	96/113	110/110	172/185	131/131	156/162	145/145	191/200	265/271	166/170	138/138	152/161	255/255	235/235	216/233
118/118	89/95	148/151	115/117	133/133	117/120	96/96	175/180	159/165	117/126	92/92	146/149	89/89	165/170	91/91	96/96	173/178	125/125	269/280	142/142	96/113	110/110	176/185	131/131	162/165	145/145	179/191	265/265	170/170	135/138	164/164	240/255	235/244	233/233
118/118	95/101	151/151	109/126	136/139	120/120	96/99	180/184	159/159	126/126	92/96	146/164	89/89	170/174	91/91	96/96	178/182	129/129	262/263	130/142	96/113	110/110	176/185	134/137	168/177	145/145	185/191	265/268	170/170	138/138	164/164	255/255	235/235	233/233
112/112	95/101	150/153	117/117	133/136	119/122	96/99	126/180	159/162	117/120	92/96	149/155	92/98	116/170	94/100	99/105	124/178	125/129	257/262	133/136	96/96	110/110	185/185	131/134	165/165	145/145	191/191	265/268	166/166	132/138	152/158	240/255	230/235	216/222
112/118	89/101	146/153	117/117	133/136	115/122	96/99	184/184	159/162	117/126	92/96	149/149	89/92	174/174	91/94	96/99	182/182	125/129	274/274	142/142	96/113	110/110	176/185	131/134	165/168	145/151	179/191	265/268	166/170	127/135	161/164	255/255	230/238	233/233
112/118	89/101	146/153	117/117	133/136	115/122	96/99	184/184	159/162	117/126	92/96	149/149	89/92	174/174	91/94	96/99	182/182	125/129	274/274	142/142	96/113	110/110	176/185	131/134	165/168	145/151	179/191	265/268	166/170	127/135	161/164	255/255	230/238	233/233
112/118	89/101	146/153	117/117	133/136	115/122	96/99	184/184	159/162	117/126	92/96	149/149	89/92	174/174	91/94	96/99	182/182	125/129	274/274	142/142	96/113	110/110	176/185	131/134	165/168	145/151	179/191	265/268	166/170	127/135	161/164	255/255	230/238	233/233
112/112	95/95	145/153	120/126	133/133	114/122	102/102	126/167	156/159	123/126	82/99	158/164	89/95	116/157	91/97	96/102	124/124	125/129	257/269	133/142	96/96	110/110	176/176	131/131	162/168	145/145	177/183	257/271	166/166	135/138	149/164	255/258	235/235	216/233
112/118	89/89	146/151	117/117	133/136	115/120	105/108	180/184	165/171	117/126	102/105	149/149	89/92	170/174	91/94	96/99	178/182	125/129	262/274	142/142	96/96	110/110	176/185	131/134	168/177	145/145	179/191	274/277	170/170	130/135	161/161	255/255	235/238	233/233
109/112	89/98	143/153	115/117	136/142	112/122	96/96	179/180	159/159	123/126	92/92	149/152	89/92	169/170	91/94	96/99	177/178	125/125	262/269	133/142	96/96	110/110	176/176	134/140	162/165	145/145	177/194	265/265	166/170	135/141	149/161	240/258	235/235	233/233
112/118	95/95	143/153	120/126	136/142	112/122	96/105	167/184	159/171	123/126	92/102	158/164	89/95	157/174	91/97	96/102	182/182	125/129	262/269	133/142	96/108	110/110	176/176	134/140	162/165	145/145	191/191	265/274	166/170	135/135	152/161	240/258	235/235	222/233
115/121	95/95	150/150	109/117	136/136	119/119	96/96	184/184	159/159	117/126	92/92	146/149	89/95	174/174	91/97	96/102	182/182	129/129	262/263	142/142	96/113	110/110	185/189	134/134	168/168	145/145	185/200	265/265	166/170	138/138	164/164	255/255	235/235	222/233
112/112	89/89	143/146	115/117	133/133	112/115	96/108	180/184	162/171	117/117	92/105	146/149	92/92	170/174	94/94	99/99	178/182	125/125	274/280	133/142	96/113	110/123	185/189	131/131	162/177	145/145	185/185	265/277	170/170	127/132	152/161	255/255	230/238	233/233
112/118	89/98	145/146	115/117	133/133	114/115	96/96	180/184	165/168	117/123	92/92	149/152	92/92	170/174	94/94	99/99	178/182	125/125	269/274	136/136	96/113	110/110	185/189	131/131	168/177	145/145	185/191	265/265	170/170	127/132	161/161	255/255	235/238	233/233
112/118	89/95	148/153	117/126	133/136	117/122	96/96	184/184	168/168	117/117	92/92	149/164	89/92	174/174	91/94	96/99	182/182	125/129	262/274	142/142	96/113	110/123	185/185	131/134	162/168	145/145	179/191	265/265	170/170	127/138	152/161	255/255	230/235	222/233
112/112	89/95	146/153	117/126	133/136	115/122	96/102	175/184	159/171	117/126	92/99	149/164	89/92	165/174	91/94	96/99	173/182	125/125	262/274	142/142	96/108	110/110	176/189	131/134	165/165	145/145	179/191	265/273	170/170	135/135	161/161	240/255	235/238	222/233
112/118	89/95	153/153	117/120	133/139	122/122	96/105	126/175	159/171	117/126	92/102	152/155	89/92	116/165	91/94	96/99	124/173	129/129	257/274	136/142	96/96	110/110	176/185	131/137	165/168	145/145	191/194	265/274	166/170	127/138	152/161	255/255	230/235	216/233
112/118	89/95	153/153	117/120	133/139	122/122	96/105	126/175	159/171	117/126	92/102	152/155	89/92	116/165	91/94	96/99	124/173	129/129	257/274	136/142	96/96	110/110	176/185	131/137	165/168	145/145	191/194	265/274	166/170	127/138	152/161	255/255	230/235	216/233
112/118	89/95	153/153	117/120	133/139	122/122	96/105	126/175	159/171	117/126	92/102	152/155	89/92	116/165	91/94	96/99	124/173	129/129	257/274	136/142	96/96	110/110	176/185	131/137	165/168	145/145	191/194	265/274	166/170	127/138	152/161	255/255	230/235	216/233
112/118	89/95	153/153	117/120	133/139	122/122	96/105	126/175	159/171	117/126	92/102	152/155	89/92	116/165	91/94	96/99	124/173	129/129	257/274	136/142	96/96	110/110	176/185	131/137	165/168	145/145	191/194	265/274	166/170	127/138	152/161	255/255	230/235	216/233
112/112	89/95	153/153	117/120	133/139	122/122	96/105	126/175	159/171	117/126	92/102	152/155	89/92	116/165	91/94	96/99	124/173	129/129	257/274	136/142	96/96	110/110	176/185	131/137	165/168	145/145	191/194	265/274	166/170	127/138	152/161	255/255	230/235	216/233
112/118	95/104	143/145	115/117	133/142	112/114	96/99	167/167	159/159	114/123	92/96	143/152	92/92	157/157	94/94	99/99	nd.	118/118	269/269	133/136	96/99	110/110	189/200	131/140	162/174	145/145	179/185	265/268	166/174	124/135	149/158	255/255	227/235	233/233
109/112	89/95	145/146	115/117	133/133	114/115	96/105	177/179	159/162	117/117	92/102	149/152	92/92	167/169	94/94	99/99	175/177	125/125	269/274	133/133	96/108	110/110	176/180	131/131	159/165	145/145	177/179	265/276	170/170	135/141	152/161	255/255	230/235	233/233
112/118	89/95	146/151	115/117	133/136	115/120	96/108	184/184	159/165	117/117	92/105	149/152	89/92	174/174	91/94	nd.	182/182	125/129	262/280	142/142	96/96	110/110	185/193	131/134	165/177	145/151	185/191	265/277	170/170	127/138	161/164	nd.	235/235	233/233
112/118	89/95	146/151	115/117	133/136	115/120	96/108	184/184	159/165	117/117	92/105	149/152	89/92	174/174	91/94	96/99	182/182	125/129	262/280	142/142	96/96	110/110	185/193	131/134	165/177	145/151	185/191	265/277	170/170	127/138	161/164	240/255	235/235	233/233
115/118	89/98	143/151	115/117	136/142	112/120	96/96	179/180	156/159	117/117	82/92	149/152	89/95	169/170	91/97	96/102	177/178	125/125	262/269	133/142	96/108	110/123	185/189	134/140	162/165	145/145	177/194	257/265	170/170	135/135	152/161	240/258	235/235	233/233
115/118	89/95	143/146	115/117	133/133	112/115	96/96	179/184	156/168	117/123	82/92	149/152	92/95	169/174	94/97	99/102	177/182	125/125	262/269	133/136	96/108	110/123	176/180	131/131	165/168	145/145	191/191	257/265	166/170	135/135	152/161	255/255	230/235	233/233
112/115	95/98	143/153	115/117	133/136	112/122	96/96	179/180	159/159	117/117	92/92	149/152	89/92	169/170	91/94	96/99	177/178	125/125	269/274	133/142	96/96	110/110	185/189	131/134	162/165	145/145	177/191	265/265	166/170	130/135	149/164	240/258	235/235	222/233
112/118	89/95	150/153	117/117	133/133	119/122	96/102	180/195	159/159	111/117	92/99	149/155	92/98	170/185	94/100	99/105	178/194	125/129	257/262	133/142	96/96	110/110	185/185	131/131	165/165	138/145	194/200	265/271	166/170	130/138	152/161	240/255	235/235	216/233
112/112	89/89	146/153	117/126	133/133	115/122	96/108	180/184	159/165	117/117	92/105	149/164	92/92	170/174	94/94	99/99	178/182	125/125	262/280	133/142	96/108	110/110	185/185	131/131	165/177	145/145	185/194	265/277	170/170	127/130	161/164	240/255	230/235	233/233
112/118	89/89	150/151	117/126	133/136	119/120	99/105	180/195	159/171	117/117	96/102	149/164	89/92	170/185	91/94	96/99	178/194	125/129	262/262	142/142	96/108	110/123	185/185	131/134	168/177	145/145	191/194	268/274	170/170	135/135	152/164	255/255	235/238	216/233
112/112	89/95	146/153	120/126	133/133	115/122	96/96	175/184	156/168	117/117	92/92	152/164	89/92	165/174	91/94	96/99	173/182	125/129	274/274	142/142	96/96	110/123	185/185	131/131	162/168	145/145	179/191	265/265	170/170	135/138	152/161	255/255	235/238	222/233
109/118	95/95	150/151	117/117	133/136	119/120	102/105	180/195	159/171	117/126	99/102	149/155	89/92	170/185	91/94	96/99	178/194	129/129	257/262	133/142	96/108	110/123	176/185	131/134	165/165	145/145	191/200	271/274	170/170	130/135	152/164	240/255	235/235	216/222
112/118	89/95	145/151	115/126	133/133	114/120	96/96	126/175	156/165	117/123	82/92	146/164	89/92	116/165	91/94	96/99	124/173	125/129	257/280	133/142	96/119	110/110	176/185	131/131	165/168	145/145	183/185	257/265	166/170	135/135	149/164	255/255	235/235	216/233
112/118	95/95	151/153	117/120	133/133	120/122	99/105	180/184	159/171	126/126	96/102	149/158	92/95	170/174	94/97	99/102	178/182	125/129	262/269	130/130	96/108	110/110	172/176	131/131	162/168	145/145	179/185	268/274	166/170	135/138	161/161	255/255	235/235	233/233
112/118	89/89	146/153	120/126	133/133	115/122	102/105	180/184	171/171	117/126	99/102	152/164	89/92	170/174	91/94	96/99	178/182	125/129	262/274	142/142	96/96	110/110	176/185	131/131	165/168	145/145	179/194	273/274	170/170	130/135	161/164	255/255	235/238	233/233
112/118	89/95	146/151	117/126	133/139	115/120	96/108	184/184	165/168	117/126	92/105	149/164	92/95	174/174	94/97	99/102	182/182	125/129	262/269	133/142	96/96	110/110	176/185	131/137	168/177	145/151	185/191	265/277	170/170	130/130	152/164	255/255	230/235	222/233
112/118	95/95	148/151	126/126	136/139	117/120	96/96	180/184	159/168	117/126	92/92	164/164	89/95	170/174	91/97	96/102	178/182	125/129	262/269	142/142	96/96	110/110	176/185	134/137	165/168	145/151	185/191	265/265	170/170	130/138	152/161	240/255	235/235	222/222
112/118	95/95	148/151	126/126	136/139	117/120	96/96	180/184	159/168	117/126	92/92	164/164	89/95	170/174	91/97	96/102	178/182	125/129	262/269	142/142	96/96	110/110	176/185	134/137	165/168	145/151	185/191	265/265	170/170	130/138	152/161	240/255	235/235	222/222
112/118	95/95	148/151	126/126	136/139	117/120	96/96	180/184	159/168	117/126	92/92	164/164	89/95	170/174	91/97	96/102	178/182	125/129	262/269	142/142	96/96	110/110	176/185	134/137	165/168	145/151	185/191	265/265	170/170	130/138	152/161	240/255	235/235	222/222
112/118	95/95	148/151	126/126	136/139	117/120	96/96	180/184	159/168	117/126	92/92	164/164	89/95	170/174	91/97	96/102	178/182	125/129	262/269	142/142	96/96	110/110	176/185	134/137	165/168	145/151	185/191	265/265	170/170	130/138	152/161	240/255	235/235	222/222
118/118	95/95	148/151	126/126	136/139	117/120	96/96	180/184	159/168	117/126	92/92	164/164	89/95	170/174	91/97	96/102	178/182	125/129	262/269	142/142	96/96	110/110	176/185	134/137	165/168	145/151	185/191	265/265	170/170	130/138	152/161	240/255	235/235	222/222
112/118	95/95	148/151	126/126	136/139	117/120	96/96	180/184	159/168	117/126	92/92	164/164	89/95	170/174	91/97	96/102	178/182	125/129	262/269	142/142	96/96	110/110	176/185	134/137	165/168	145/151	185/191	265/265	170/170	130/138	152/161	240/255	235/235	222/222
112/118	95/95	148/151	126/126	136/139	117/120	96/96	180/184	159/168	117/126	92/92	164/164	89/95	170/174	91/97	96/102	178/182	125/129	262/269	142/142	96/96	110/110	176/185	134/137	165/168	145/151	185/191	265/265	170/170	130/138	152/161	240/255	235/235	222/222
112/118	95/95	148/151	126/126	136/139	117/120	96/96	180/184	159/168	117/126	92/92	164/164	89/95	170/174	91/97	96/102	178/182	125/129	262/269	142/142	96/96	110/110	176/185	134/137	165/168	145/151	185/191	265/265	170/170	130/138	152/161	240/255	235/235	222/222
112/118	95/95	148/151	126/126	136/139	117/120	96/96	180/184	159/168	117/126	92/92	164/164	89/95	170/174	91/97	96/102	178/182	125/129	262/269	142/142	96/96	110/110	176/185	134/137	165/168	145/151	185/191	265/265	170/170	130/138	152/161	240/255	235/235	222/222
112/118	95/95	148/151	126/126	136/139	117/120	96/96	180/184	159/168	117/126	92/92	164/164	89/95	170/174	91/97	96/102	178/182	125/129	262/269	142/142	96/96	110/110	176/185	134/137	165/168	145/151	185/191	265/265	170/170	130/138	152/161	240/255	235/235	222/222
112/118	95/95	148/151	126/126	136/139	117/120	96/96	180/184	159/168	117/126	92/92	164/164	89/95	170/174	91/97	96/102	178/182	125/129	262/269	142/142	96/96	110/110	176/185	134/137	165/168	145/151	185/191	265/265	170/170	130/138	152/161	240/255	235/235	222/222
112/118	95/95	148/151	126/126	136/139	117/120	96/96	180/184	159/168	117/126	92/92	164/164	89/95	170/174	91/97	96/102	178/182	125/129	262/269	142/142	96/96	110/110	176/185	134/137	165/168	145/151	185/191	265/265	170/170	130/138	152/161	240/255	235/235	222/222
112/118	95/95	148/151	126/126	136/139	117/120	96/96	180/184	159/168	117/126	92/92	164/164	89/95	170/174	91/97	96/102	178/182	125/129	262/269	142/142	96/96	110/110	176/185	134/137	165/168	145/151	185/191	265/265	170/170	130/138	152/161	240/255	235/235	222/222
112/118	95/95	148/151	126/126	136/139	117/120	96/96	180/184	159/168	117/126	92/92	164/164	89/95	170/174	91/97	96/102	178/182	125/129	262/269	142/142	96/96	110/110	176/185	134/137	165/168	145/151	185/191	265/265	170/170	130/138	152/161	240/255	235/235	222/222
112/118	95/95	148/151	126/126	136/139	117/120	96/96	180/184	159/168	117/126	92/92	164/164	89/95	170/174	91/97	96/102	178/182	125/129	262/269	142/142	96/96	110/110	176/185	134/137	165/168	145/151	185/191	265/265	170/170	130/138	152/161	240/255	235/235	222/222
112/118	95/95	148/151	126/126	136/139	117/120	96/96	180/184	159/168	117/126	92/92	164/164	89/95	170/174	91/97	96/102	178/182	125/129	262/269	142/142	96/96	110/110	176/185	134/137	165/168	145/151	185/191	265/265	170/170	130/138	152/161	240/255	235/235	222/222
112/118	95/95	148/151	126/126	136/139	117/120	96/96	180/184	159/168	117/126	92/92	164/164	89/95	170/174	91/97	96/102	178/182	125/129	262/269	142/142	96/96	110/110	176/185	134/137	165/168	145/151	185/191	265/265	170/170	130/138	152/161	240/255	235/235	222/222
109/112	89/98	143/153	115/117	136/142	112/122	96/96	179/180	159/159	123/126	92/92	149/152	89/92	169/170	91/94	96/99	177/178	125/125	262/269	133/142	96/96	110/110	176/176	134/140	162/165	145/145	177/194	265/265	166/170	135/141	149/161	240/258	235/235	233/233
112/112	89/95	146/153	117/126	133/139	115/122	96/96	164/184	159/165	117/126	92/92	149/164	89/92	153/174	91/94	96/99/102	162/182	125/129	262/274	142/142	96/108	110/110	176/185	131/137	162/165/168	145/151	185/191	265/265	170/170	132/135	161/161	240/255	235/238	222/233
118/118	95/101	151/153	115/123	133/139	120/122	96/96	180/180	159/162	111/129	92/92	152/161	95/95	170/170	97/97	102/102	178/178	125/129	262/262	148/148	96/96	110/110	189/193	131/137	165/168	145/151	191/191	265/265	166/170	138/138	152/164	240/255	235/244	222/222
112/118	98/101	153/153	117/117	133/136	122/122	96/102	180/183	159/159	117/123	92/99	149/155	92/98	170/173	94/100	99/105	178/181	125/129	257/262	136/142	96/96	110/110	185/189	131/134	165/165	145/145	185/191	265/271	166/166	132/138	164/164	240/255	235/244	233/233
112/118	98/101	153/153	117/117	133/136	122/122	96/102	180/183	159/159	117/123	92/99	149/155	92/98	170/173	94/100	99/105	178/181	125/129	257/262	136/142	96/96	110/110	185/189	131/134	165/165	145/145	185/191	265/271	166/166	132/138	164/164	240/255	235/244	233/233
118/118	89/95	143/153	117/126	133/133	112/122	99/99	184/184	162/168	117/129	96/96	149/164	89/92	174/174	91/94	96/99	182/182	118/129	262/274	136/148	96/113	110/110	193/193	131/131	168/177	145/148	185/191	268/268	166/170	132/138	161/164	255/255	230/235	233/233
112/112	98/101	143/153	117/120	133/139	112/122	99/99	167/183	156/162	123/126	82/96	155/158	95/98	157/173	97/100	102/105	181/181	125/129	257/269	133/142	96/96	110/110	176/176	131/137	162/168	145/145	177/191	257/268	166/170	138/141	152/164	255/258	235/235	233/233
112/118	89/95	146/151	115/126	133/139	115/120	96/108	180/184	159/165	114/117	92/105	146/164	92/113	170/174	94/115	99/119	178/182	125/129	262/274	133/142	96/113	110/116	180/185	131/137	168/177	145/145	185/191	265/277	170/170	132/138	161/164	255/255	235/238	222/233
112/118	89/95	151/153	117/126	133/136	120/122	96/105	180/184	159/171	117/126	92/102	149/164	89/92	170/174	91/94	96/99	178/182	125/129	262/274	142/142	96/108	110/123	176/185	131/134	165/168	145/145	191/194	265/274	170/170	130/135	161/164	240/255	235/235	222/233
112/118	95/98	145/150	115/115	133/133	114/119	96/102	175/195	159/159	111/123	92/99	146/155	89/98	165/185	91/100	96/105	173/194	118/129	257/269	133/136	96/113	110/110	176/189	131/131	165/168	145/145	191/191	265/271	166/170	138/138	152/164	255/255	230/235	216/233
112/118	89/95	151/153	117/126	133/136	120/122	96/105	180/184	159/171	117/126	92/102	149/164	89/92	170/174	91/94	96/99	178/182	125/129	262/274	142/142	96/108	110/123	176/185	131/134	165/168	145/145	191/194	265/274	170/170	130/135	161/164	240/255	235/235	222/233
112/118	89/95	151/153	117/126	133/136	120/122	96/105	180/184	159/171	117/126	92/102	149/164	89/92	170/174	91/94	96/99	178/182	125/129	262/274	142/142	96/108	110/123	176/185	131/134	165/168	145/145	191/194	265/274	170/170	130/135	161/164	240/255	235/235	222/233
115/118	89/95	139/145	115/117	133/133	107/114	96/96	180/180	159/159	120/123	82/92	143/152	89/92	170/170	91/94	96/99	178/178	118/129	269/269	136/139	96/96	110/116	176/189	131/131	162/165	145/145	179/179	255/265	166/170	124/132	158/161	255/255	227/230	233/233
112/118	89/95	139/153	109/117	133/133	107/122	96/96	184/184	159/159	126/129	82/92	143/146	92/113	174/174	94/115	99/119	182/182	125/133	269/269	136/139	96/96	110/110	185/193	131/131	162/177	145/151	185/185	255/265	166/166	124/138	161/164	255/255	227/238	233/233
112/118	95/98	139/153	117/120	133/133	107/122	96/96	184/184	159/159	120/129	82/92	143/158	92/95	174/174	94/97	99/102	182/182	118/133	263/269	139/142	96/96	110/116	176/176	131/131	162/168	145/145	179/191	255/265	166/166	124/138	158/161	255/255	227/235	233/233
112/112	89/89	146/146	117/117	133/133	115/115	96/96	176/180	165/168	117/123	92/92	149/149	92/92	166/170	94/94	99/99	174/178	125/125	274/274	133/136	108/108	110/110	185/185	131/131	165/177	145/145	179/185	265/265	170/170	127/135	161/161	255/255	230/230	222/233
112/115	95/95	137/139	117/120	133/136	106/107	96/96	179/179	159/159	114/120	82/92	143/146	92/92	169/169	94/94	99/99	177/177	133/133	269/269	133/139	96/113	110/116	178/182	131/134	162/165	145/145	179/179	255/265	166/166	124/143	158/161	255/255	227/235	227/233
112/115	95/95	139/153	117/126	133/133	107/122	96/96	184/184	159/159	120/129	82/92	143/164	92/113	174/174	94/115	99/119	182/182	129/133	269/269	130/139	96/113	110/116	176/185	131/131	162/168	145/151	179/185	255/265	166/170	124/135	158/164	255/255	227/235	233/233
112/112	95/95	143/151	115/117	133/133	112/120	96/105	179/180	159/171	123/126	92/102	149/152	89/92	169/170	91/94	96/99	177/178	125/129	269/274	133/142	96/108	110/110	176/176	131/131	165/165	145/145	191/191	265/274	166/170	130/135	152/164	240/255	235/235	222/233
112/118	95/95	151/153	117/117	133/139	120/122	96/105	180/180	159/171	117/126	92/102	149/149	95/95	170/170	97/97	102/102	178/178	129/129	262/262	130/130	96/113	110/110	176/193	131/137	168/168	145/151	179/185	265/274	166/170	135/138	161/164	255/255	235/235	222/233
112/112	89/98	145/146	115/115	133/133	114/115	96/96	175/180	159/168	123/126	92/92	146/152	89/92	165/170	91/94	96/99	173/178	118/118	269/274	136/145	96/113	110/110	172/189	131/131	162/168	145/145	179/191	265/265	170/170	132/138	152/161	255/255	230/235	233/233
115/118	95/95	143/153	120/126	136/142	112/122	96/105	167/184	159/171	123/126	92/102	158/164	89/95	157/174	91/97	96/102	182/182	125/129	262/269	133/142	96/108	110/110	176/176	134/140	162/165	145/145	191/191	265/274	166/170	135/135	152/161	240/258	235/235	222/233
112/112	89/95	146/148	117/126	133/139	115/117	96/108	180/184	165/168	117/117	92/105	149/164	89/92	170/174	91/94	96/99	178/182	125/129	262/274	142/142	96/108	110/110	185/185	131/137	162/168	145/151	179/191	265/277	170/170	127/130	152/161	255/255	235/238	222/233
112/118	89/95	146/153	117/126	133/139	115/122	96/96	164/184	159/165	117/117	92/92	149/164	89/92	153/174	91/94	96/99	162/182	125/125	262/274	142/142	96/108	110/110	185/185	131/137	162/168	145/145	185/191	265/265	170/170	132/135	161/161	255/255	235/238	222/233
112/118	89/95	146/153	117/126	133/139	115/122	96/96	164/184	159/165	117/117	92/92	149/164	89/92	153/174	91/94	96/99	162/182	125/125	262/274	142/142	96/108	110/110	185/185	131/137	162/168	145/145	185/191	265/265	170/170	132/135	161/161	255/255	235/238	222/233
112/118	89/95	146/153	117/126	133/139	115/122	96/96	164/184	159/165	117/117	92/92	149/164	89/92	153/174	91/94	96/99	162/182	125/125	262/274	142/142	96/108	110/110	185/185	131/137	162/168	145/145	185/191	265/265	170/170	132/135	161/161	255/255	235/238	222/233
112/118	89/95	146/153	117/126	133/139	115/122	96/96	164/184	159/165	117/117	92/92	149/164	89/92	153/174	91/94	96/99	162/182	125/125	262/274	142/142	96/108	110/110	185/185	131/137	162/168	145/145	185/191	265/265	170/170	132/135	161/161	255/255	235/238	222/233
112/118	89/95	145/148	115/126	133/136	114/117	96/96	175/180	159/168	126/126	92/92	146/164	89/89	165/170	91/91	96/96	173/178	118/129	262/274	142/145	96/113	110/110	172/176	131/134	168/168	145/151	179/185	265/265	170/170	130/138	161/161	255/255	235/235	222/233
112/118	95/101	146/152	115/117	133/133	115/121	88/108	180/180	159/165	111/117	82/105	149/152	89/92	170/170	91/94	96/99	178/178	125/125	262/274	130/136	96/96	110/110	185/189	131/131	165/177	145/145	185/191	255/277	166/170	127/138	164/164	240/255	235/238	222/233
112/112	89/89	146/153	117/120	133/133	115/122	102/108	175/180	156/171	117/123	99/105	149/152	92/92	165/170	94/94	99/99	173/178	125/125	274/274	133/142	96/96	110/110	185/185	131/131	177/177	145/145	185/185	273/277	170/170	127/135	161/161	255/255	230/235	233/233

118/118	89/95	146/148	115/117	133/133	115/117	96/96	180/180	159/165	120/126	92/92	149/152	89/89	170/170	91/91	96/96	178/178	125/129	269/274	142/142	108/113	110/110	176/185	131/131	165/168	145/145	185/191	265/265	170/170	130/138	161/164	255/255	238/244	233/233
118/118	89/95	146/148	117/126	133/139	115/117	96/108	180/180	159/165	123/126	92/105	149/164	89/89	170/170	91/91	96/96	178/178	125/129	262/274	133/142	96/96	110/110	176/180	131/137	165/177	145/151	191/191	265/277	166/170	130/138	161/161	240/255	235/235	222/233
118/118	95/95	150/151	117/123	136/136	119/120	96/96	180/184	159/159	117/126	92/92	149/161	89/95	170/174	91/97	96/102	178/182	129/129	262/263	130/142	96/96	110/110	176/193	134/134	168/177	145/151	185/191	265/265	166/170	138/138	164/164	255/255	235/235	233/233
118/118	95/95	150/151	117/123	136/136	119/120	96/96	180/184	159/159	117/126	92/92	149/161	89/95	170/174	91/97	96/102	178/182	129/129	262/263	130/142	96/96	110/110	176/193	134/134	168/177	145/151	185/191	265/265	166/170	138/138	164/164	255/255	235/235	233/233
118/118	95/95	150/151	117/123	136/136	119/120	96/96	180/184	159/159	117/126	92/92	149/161	89/95	170/174	91/97	96/102	178/182	129/129	262/263	130/142	96/96	110/110	176/193	134/134	168/177	145/151	185/191	265/265	166/170	138/138	164/164	255/255	235/235	233/233
118/118	95/95	150/151	117/123	136/136	119/120	96/96	180/184	159/159	117/126	92/92	149/161	89/95	170/174	91/97	96/102	178/182	129/129	262/263	130/142	96/96	110/110	176/193	134/134	168/177	145/151	185/191	265/265	166/170	138/138	164/164	255/255	235/235	233/233
118/118	95/101	151/153	109/115	136/139	120/122	99/102	184/184	159/168	126/126	96/99	146/152	89/89	174/174	91/91	96/96	182/182	118/125	262/262	142/142	96/113	110/110	176/185	134/137	168/168	145/145	185/191	268/273	170/170	127/138	164/164	255/255	235/235	233/233
112/118	89/89	146/146	117/117	133/133	115/115	96/105	184/184	156/165	117/123	92/102	149/149	92/92	174/174	94/94	99/99	182/182	125/125	280/280	136/136	96/108	110/110	185/185	131/131	162/162	145/145	179/185	265/274	170/170	135/135	152/161	255/255	238/238	233/233
112/118	95/98	145/150	115/115	133/133	114/119	96/102	175/183	159/168	111/126	92/99	146/155	89/98	165/173	91/100	96/105	173/181	129/129	257/274	133/145	96/113	110/110	172/185	131/131	156/162	145/145	191/200	265/271	166/170	138/138	152/161	255/255	235/235	216/233
112/112	89/95	146/148	117/126	133/139	115/117	96/96	184/184	159/168	117/117	92/92	149/164	89/92	174/174	91/94	96/99	182/182	125/129	269/280	133/142	96/96	110/110	185/185	131/137	168/177	145/145	185/191	265/265	170/170	127/138	161/161	255/255	230/235	222/233
112/118	89/95	148/151	126/126	133/139	117/120	96/105	184/184	168/171	126/126	92/102	164/164	92/95	174/174	94/97	99/102	182/182	129/129	262/274	142/142	96/108	110/123	176/176	131/137	165/165	145/151	191/194	265/274	170/170	130/138	152/164	240/240	235/235	222/233
112/118	89/95	148/151	126/126	133/139	117/120	96/105	184/184	168/171	126/126	92/102	164/164	92/95	174/174	94/97	99/102	182/182	129/129	262/274	142/142	96/108	110/123	176/176	131/137	165/165	145/151	191/194	265/274	170/170	130/138	152/164	240/240	235/235	222/233
112/118	89/95	148/151	126/126	133/139	117/120	96/105	184/184	168/171	126/126	92/102	164/164	92/95	174/174	94/97	99/102	182/182	129/129	262/274	142/142	96/108	110/123	176/176	131/137	165/165	145/151	191/194	265/274	170/170	130/138	152/164	240/240	235/235	222/233
112/118	89/95	148/151	126/126	133/139	117/120	96/105	184/184	168/171	126/126	92/102	164/164	92/95	174/174	94/97	99/102	182/182	129/129	262/274	142/142	96/108	110/123	176/176	131/137	165/165	145/151	191/194	265/274	170/170	130/138	152/164	240/240	235/235	222/233
112/118	89/95	148/151	126/126	133/139	117/120	96/105	184/184	168/171	126/126	92/102	164/164	92/95	174/174	94/97	99/102	182/182	129/129	262/274	142/142	96/108	110/123	176/176	131/137	165/165	145/151	191/194	265/274	170/170	130/138	152/164	240/240	235/235	222/233
112/118	89/95	148/151	126/126	133/139	117/120	96/105	184/184	168/171	126/126	92/102	164/164	92/95	174/174	94/97	99/102	182/182	129/129	262/274	142/142	96/108	110/123	176/176	131/137	165/165	145/151	191/194	265/274	170/170	130/138	152/164	240/240	235/235	222/233
112/118	89/95	148/151	126/126	133/139	117/120	96/105	184/184	168/171	126/126	92/102	164/164	92/95	174/174	94/97	99/102	182/182	129/129	262/274	142/142	96/108	110/123	176/176	131/137	165/165	145/151	191/194	265/274	170/170	130/138	152/164	240/240	235/235	222/233
112/118	89/95	148/151	126/126	133/139	117/120	96/105	184/184	168/171	126/126	92/102	164/164	92/95	174/174	94/97	99/102	182/182	129/129	262/274	142/142	96/108	110/123	176/176	131/137	165/165	145/151	191/194	265/274	170/170	130/138	152/164	240/240	235/235	222/233
112/118	89/95	148/151	126/126	133/139	117/120	96/105	184/184	168/171	126/126	92/102	164/164	92/95	174/174	94/97	99/102	182/182	129/129	262/274	142/142	96/108	110/123	176/176	131/137	165/165	145/151	191/194	265/274	170/170	130/138	152/164	240/240	235/235	222/233
112/118	89/95	148/151	126/126	133/139	117/120	96/105	184/184	168/171	126/126	92/102	164/164	92/95	174/174	94/97	99/102	182/182	129/129	262/274	142/142	96/108	110/123	176/176	131/137	165/165	145/151	191/194	265/274	170/170	130/138	152/164	240/240	235/235	222/233
112/112	89/95	148/151	126/126	133/139	117/120	96/105	184/184	168/171	126/126	92/102	164/164	92/95	174/174	94/97	99/102	182/182	129/129	262/274	142/142	96/108	110/123	176/176	131/137	165/165	145/151	191/194	265/274	170/170	130/138	152/164	240/240	235/235	222/233
112/118	89/95	148/151	126/126	133/139	117/120	96/105	184/184	168/171	126/126	92/102	164/164	92/95	174/174	94/97	99/102	182/182	129/129	262/274	142/142	96/108	110/123	176/176	131/137	165/165	145/151	191/194	265/274	170/170	130/138	152/164	240/240	235/235	222/233
112/118	89/95	148/151	126/126	133/139	117/120	96/105	184/184	168/171	126/126	92/102	164/164	92/95	174/174	94/97	99/102	182/182	129/129	262/274	142/142	96/108	110/123	176/176	131/137	165/165	145/151	191/194	265/274	170/170	130/138	152/164	240/240	235/235	222/233
112/118	89/95	148/151	126/126	133/139	117/120	96/105	184/184	168/171	126/126	92/102	164/164	92/95	174/174	94/97	99/102	182/182	129/129	262/274	142/142	96/108	110/123	176/176	131/137	165/165	145/151	191/194	265/274	170/170	130/138	152/164	240/240	235/235	222/233
112/118	89/95	148/151	126/126	133/139	117/120	96/105	184/184	168/171	126/126	92/102	164/164	92/95	174/174	94/97	99/102	182/182	129/129	262/274	142/142	96/108	110/123	176/176	131/137	165/165	145/151	191/194	265/274	170/170	130/138	152/164	240/240	235/235	222/233
112/118	89/95	148/151	126/126	133/139	117/120	96/105	184/184	168/171	126/126	92/102	164/164	92/95	174/174	94/97	99/102	182/182	129/129	262/274	142/142	96/108	110/123	176/176	131/137	165/165	145/151	191/194	265/274	170/170	130/138	152/164	240/240	235/235	222/233
112/118	89/95	148/151	126/126	133/139	117/120	96/105	184/184	168/171	126/126	92/102	164/164	92/95	174/174	94/97	99/102	182/182	129/129	262/274	142/142	96/108	110/123	176/176	131/137	165/165	145/151	191/194	265/274	170/170	130/138	152/164	240/240	235/235	222/233
112/118	89/95	148/151	126/126	133/139	117/120	96/105	184/184	168/171	126/126	92/102	164/164	92/95	174/174	94/97	99/102	182/182	129/129	262/274	142/142	96/108	110/123	176/176	131/137	165/165	145/151	191/194	265/274	170/170	130/138	152/164	240/240	235/235	222/233
112/118	89/95	148/151	126/126	133/139	117/120	96/105	184/184	168/171	126/126	92/102	164/164	92/95	174/174	94/97	99/102	182/182	129/129	262/274	142/142	96/108	110/123	176/176	131/137	165/165	145/151	191/194	265/274	170/170	130/138	152/164	240/240	235/235	222/233
118/118	89/95	148/151	126/126	133/139	117/120	96/105	184/184	168/171	126/126	92/102	164/164	92/95	174/174	94/97	99/102	182/182	129/129	262/274	142/142	96/108	110/123	176/176	131/137	165/165	145/151	191/194	265/274	170/170	130/138	152/164	240/240	235/235	222/233
112/118	89/95	148/151	126/126	133/139	117/120	96/105	184/184	168/171	126/126	92/102	164/164	92/95	174/174	94/97	99/102	182/182	129/129	262/274	142/142	96/108	110/123	176/176	131/137	165/165	145/151	191/194	265/274	170/170	130/138	152/164	240/240	235/235	222/233
112/118	89/95	143/153	117/126	133/133	112/122	99/99	184/184	162/168	117/129	96/96	149/164	89/92	174/174	91/94	96/99	182/182	118/129	262/274	136/148	96/113	110/110	193/193	131/131	168/177	145/148	185/191	268/268	166/170	132/138	161/164	255/255	230/235	233/233
112/118	95/95	150/155	126/126	133/133	119/124	96/102	184/187	159/159	111/126	92/99	155/164	89/98	174/177	91/100	96/105	182/185	129/133	257/269	133/142	96/96	110/110	176/193	131/131	165/168	138/151	191/200	265/271	166/170	138/138	152/164	255/255	235/235	216/233
112/112	95/95	150/155	126/126	133/133	119/124	96/102	184/187	159/159	111/126	92/99	155/164	89/98	174/177	91/100	96/105	182/185	129/129	257/269	133/142	96/96	110/110	176/176	131/131	165/168	145/145	191/200	265/271	166/166	138/138	152/164	255/255	235/235	216/222
112/118	89/95	148/151	117/126	133/139	117/120	96/102	180/184	165/168	123/126	92/99	149/164	89/92	170/174	91/94	96/99	178/182	125/129	269/274	142/142	96/96	110/110	176/180	131/137	162/168	145/151	179/185	265/271	170/170	130/130	161/164	255/255	235/238	222/233
112/118	95/101	148/151	123/126	133/139	117/120	96/96	180/184	159/168	117/129	92/92	161/164	95/95	170/174	97/97	102/102	178/182	129/129	262/269	142/148	96/96	110/110	185/193	131/137	168/168	145/145	191/191	265/265	170/170	130/138	161/164	255/255	235/235	222/222
109/118	89/98	145/146	115/115	133/133	114/115	96/96	175/180	159/168	123/126	92/92	146/152	89/92	165/170	91/94	96/99	173/178	118/118	269/274	136/145	96/113	110/110	172/189	131/131	162/168	145/145	179/191	265/265	170/170	132/138	152/161	255/255	230/235	233/233
112/118	89/98	145/146	115/115	133/133	114/115	96/96	175/180	159/168	123/126	92/92	146/152	89/92	165/170	91/94	96/99	173/178	118/118	269/274	136/145	96/113	110/110	172/189	131/131	162/168	145/145	179/191	265/265	170/170	132/138	152/161	255/255	230/235	233/233
112/118	89/98	145/146	115/115	133/133	114/115	96/96	175/180	159/168	123/126	92/92	146/152	89/92	165/170	91/94	96/99	173/178	118/118	269/274	136/145	96/113	110/110	172/189	131/131	162/168	145/145	179/191	265/265	170/170	132/138	152/161	255/255	230/235	233/233
112/118	89/98	145/146	115/115	133/133	114/115	96/96	175/180	159/168	123/126	92/92	146/152	89/92	165/170	91/94	96/99	173/178	118/118	nd.	136/145	96/113	110/110	172/189	131/131	162/168	145/145	179/191	265/265	170/170	132/138	152/161	255/255	230/235	233/233
112/118	89/98	145/146	115/115	133/133	114/115	96/96	175/180	159/168	123/126	92/92	146/152	89/92	165/170	91/94	96/99	173/178	118/118	269/274	136/145	96/113	110/110	172/189	131/131	162/168	145/145	179/191	265/265	170/170	132/138	152/161	255/255	230/235	233/233
115/118	98/101	143/153	115/117	133/133	112/122	99/99	179/180	156/162	117/123	82/96	149/152	89/92	169/170	91/94	96/99	177/178	125/125	262/269	133/136	96/96	110/110	176/185	131/131	162/165	145/145	177/191	257/268	166/166	135/141	149/164	240/258	230/235	222/233
112/112	89/89	146/146	115/120	133/133	115/115	102/108	175/184	162/171	117/123	99/105	146/152	92/92	165/174	94/94	99/99	173/182	125/125	274/280	136/136	96/113	110/123	185/185	131/131	162/177	145/145	179/185	273/277	170/170	127/135	152/161	255/255	230/235	233/233
118/118	98/101	150/153	109/120	136/139	119/122	96/96	184/184	159/159	126/126	92/92	146/158	89/95	174/174	91/97	96/102	182/182	129/129	262/269	142/142	96/96	110/116	176/185	134/137	168/168	145/151	191/191	265/265	170/170	132/138	164/164	255/255	235/235	222/233
109/118	95/98	148/153	117/126	136/139	117/122	96/96	180/183	159/168	117/123	92/92	155/164	95/98	170/173	97/100	102/105	178/181	125/129	257/269	142/142	96/96	110/110	185/189	134/137	165/168	145/151	185/185	265/265	166/170	138/138	161/164	240/255	235/235	222/233
112/118	89/101	151/153	117/117	133/136	120/122	96/102	180/184	159/168	117/126	92/99	149/149	89/113	170/174	91/115	96/119	178/182	125/129	262/274	142/142	96/113	110/110	176/193	131/134	162/168	145/151	185/191	265/273	166/170	127/132	161/164	255/255	230/235	233/233
112/118	89/101	151/153	117/117	133/136	120/122	96/102	180/184	159/168	117/126	92/99	149/149	89/113	170/174	91/115	96/119	178/182	125/129	262/274	142/142	96/113	110/110	176/193	131/134	162/168	145/151	185/191	265/273	166/170	127/132	161/164	255/255	230/235	233/233
112/118	89/101	151/153	117/117	133/136	120/122	96/102	180/184	159/168	117/126	92/99	149/149	89/110	170/174	91/112	96/117	178/182	125/129	262/274	142/142	96/113	110/110	176/193	131/134	162/168	145/151	185/191	265/273	166/170	127/132	161/164	255/255	230/235	233/233
112/118	89/101	151/153	117/117	133/136	120/122	96/102	180/184	159/168	117/126	92/99	149/149	89/113	170/174	91/115	96/119	178/182	125/129	262/274	142/142	96/113	110/110	176/193	131/134	162/168	145/151	185/191	265/273	166/170	127/132	161/164	255/255	230/235	233/233
112/118	89/101	151/153	117/117	133/136	120/122	96/102	180/184	159/168	117/126	92/99	149/149	89/89	170/174	91/91	96/96	178/182	125/129	262/274	142/142	96/113	110/110	176/193	131/134	162/168	145/151	185/191	265/273	166/170	127/132	161/164	255/255	230/235	233/233
112/118	89/101	151/153	117/117	133/136	120/122	96/102	180/184	159/168	117/126	92/99	149/149	89/113	170/174	91/115	96/119	178/182	125/129	262/274	142/142	96/113	110/110	176/193	131/134	162/168	145/151	185/191	265/273	166/170	127/132	161/164	255/255	230/235	233/233
112/118	89/101	151/153	117/117	133/136	120/122	96/102	180/184	159/168	117/126	92/99	149/149	89/113	170/174	91/115	96/119	178/182	125/129	262/274	142/142	96/113	110/110	176/193	131/134	162/168	145/151	185/191	265/273	166/170	127/132	161/164	255/255	230/235	233/233
112/118	89/101	151/153	117/117	133/136	120/122	96/102	180/184	159/168	117/126	92/99	149/149	89/113	170/174	91/115	96/119	178/182	125/129	262/274	142/142	96/113	110/110	176/193	131/134	162/168	145/151	185/191	265/273	166/170	127/132	161/164	255/255	230/235	233/233
112/112	89/101	151/153	117/117	133/136	120/122	96/102	180/184	159/168	117/126	92/99	149/149	89/113	170/174	91/115	96/119	178/182	125/129	262/274	142/142	96/113	110/110	176/193	131/134	162/168	145/151	185/191	265/273	166/170	127/132	161/164	255/255	230/235	233/233
112/118	89/101	151/153	117/117	133/136	120/122	96/102	180/184	159/168	117/126	92/99	149/149	89/113	170/174	91/115	96/119	178/182	125/129	262/274	142/142	96/113	110/110	176/193	131/134	162/168	145/151	185/191	265/273	166/170	127/132	161/164	255/255	230/235	233/233
112/118	89/101	151/153	117/117	133/136	120/122	96/102	180/184	159/168	117/126	92/99	149/149	89/113	170/174	91/115	96/119	178/182	125/129	262/274	142/142	96/113	110/110	176/193	131/134	162/168	145/151	185/191	265/273	166/170	127/132	161/164	255/255	230/235	233/233
112/118	89/101	151/153	117/117	133/136	120/122	96/102	180/184	159/168	117/126	92/99	149/149	89/113	170/174	91/115	96/119	178/182	125/129	262/274	142/142	96/113	110/110	176/193	131/134	162/168	145/151	185/191	265/273	166/170	127/132	161/164	255/255	230/235	233/233
118/118	89/101	151/153	117/117	133/136	120/122	96/102	180/184	159/168	117/126	92/99	149/149	89/113	170/174	91/115	96/119	178/182	125/129	262/274	142/142	96/113	110/110	176/193	131/134	162/168	145/151	185/191	265/273	166/170	127/132	161/164	255/255	230/235	233/233
112/118	89/101	151/153	117/117	133/136	120/122	96/102	180/184	159/168	117/126	92/99	149/149	89/89	170/174	91/91	96/96	178/182	125/129	262/274	142/142	96/113	110/110	176/193	131/134	162/168	145/151	185/191	265/273	166/170	127/132	161/164	255/255	230/235	233/233
112/118	89/101	151/153	117/117	133/136	120/122	96/102	180/184	159/168	117/126	92/99	149/149	89/113	170/174	91/115	96/119	178/182	125/129	262/274	142/142	96/113	110/110	176/193	131/134	162/168	145/151	185/191	265/273	166/170	127/132	161/164	255/255	230/235	233/233
112/118	89/101	151/153	117/117	133/136	120/122	96/102	180/184	159/168	117/126	92/99	149/149	89/113	170/174	91/115	96/119	178/182	125/129	262/274	142/142	96/113	110/110	176/193	131/134	162/168	145/151	185/191	265/273	166/170	127/132	161/164	255/255	230/235	233/233
112/118	89/101	151/153	117/117	133/136	120/122	96/102	180/184	159/168	117/126	92/99	149/149	89/113	170/174	91/115	96/119	178/182	125/129	262/274	142/142	96/113	110/110	176/193	131/134	162/168	145/151	185/191	265/273	166/170	127/132	161/164	255/255	230/235	233/233
112/118	89/101	151/153	117/117	133/136	120/122	96/102	180/184	159/168	117/126	92/99	149/149	89/113	170/174	91/115	96/119	178/182	125/129	262/274	142/142	96/113	110/110	176/193	131/134	162/168	145/151	185/191	265/273	166/170	127/132	161/164	255/255	230/235	233/233
112/118	89/101	151/153	117/117	133/136	120/122	96/102	180/184	159/168	117/126	92/99	149/149	89/116	170/174	91/91	96/122	178/182	125/129	262/274	142/142	96/113	110/110	176/193	131/134	162/168	145/151	185/191	265/273	166/170	127/132	161/164	255/255	230/235	233/233
112/112	89/101	151/153	117/117	133/136	120/122	96/102	180/184	159/168	117/126	92/99	149/149	89/113	170/174	91/115	96/119	178/182	125/129	262/274	142/142	96/113	110/110	176/193	131/134	162/168	145/151	185/191	265/273	166/170	127/132	161/164	255/255	230/235	233/233
112/118	95/101	150/153	126/126	133/139	119/122	99/102	126/184	159/162	123/126	96/99	164/164	89/98	116/174	91/100	96/105	124/182	129/129	257/257	133/142	96/96	110/110	176/189	131/137	165/168	145/145	183/191	268/271	166/170	138/138	164/164	255/255	235/235	216/233
112/118	95/95	150/153	117/126	133/139	119/122	96/99	126/184	159/162	120/126	92/96	155/164	89/98	116/174	91/100	96/105	124/182	129/129	257/262	133/142	96/96	110/110	176/185	131/137	165/168	145/145	191/191	265/268	166/170	138/138	152/152	255/255	235/235	216/216
112/118	95/95	150/153	117/126	133/139	119/122	96/99	126/184	159/162	120/126	92/96	155/164	89/98	116/174	91/100	96/105	124/182	129/129	257/262	133/142	96/96	110/110	176/185	131/137	165/168	145/145	191/191	265/268	166/170	138/138	152/152	255/255	235/235	216/216
112/118	95/95	150/153	117/126	133/139	119/122	96/99	126/184	159/162	120/126	92/96	155/164	89/98	116/174	91/100	96/105	124/182	129/129	257/262	133/142	96/96	110/110	176/185	131/137	165/168	145/145	191/191	265/268	166/170	138/138	152/152	255/255	235/235	216/216
112/118	95/101	150/153	126/126	133/139	119/122	99/102	126/184	159/162	123/126	96/99	164/164	89/98	116/174	91/100	96/105	124/182	129/129	257/257	133/142	96/96	110/110	176/189	131/137	165/168	145/145	183/191	268/271	166/170	138/138	164/164	255/255	235/235	216/233
112/118	95/101	150/153	126/126	133/139	119/122	99/102	126/184	159/162	123/126	96/99	164/164	89/98	116/174	91/100	96/105	124/182	129/129	257/257	133/142	96/96	110/110	176/189	131/137	165/168	145/145	183/191	268/271	166/170	138/138	164/164	255/255	235/235	216/233
112/118	95/101	150/152	115/117	133/133	119/121	88/102	126/180	159/159	111/120	82/99	152/155	89/98	116/170	91/100	96/105	124/178	125/129	257/262	130/133	96/96	110/110	185/189	131/131	165/165	145/145	191/200	255/271	166/166	138/138	164/164	240/255	235/235	216/222
112/118	95/95	150/153	117/126	133/139	119/122	96/99	126/184	159/162	120/126	92/96	155/164	89/98	116/174	91/100	96/105	124/182	129/129	257/262	133/142	96/96	110/110	176/185	131/137	165/168	145/145	191/191	265/268	166/170	138/138	152/152	255/255	235/235	216/216
112/118	95/95	150/153	117/126	133/139	119/122	96/99	126/184	159/162	120/126	92/96	155/164	89/98	116/174	91/100	96/105	124/182	129/129	257/262	133/142	96/96	110/110	176/185	131/137	165/168	145/145	191/191	265/268	166/170	138/138	152/152	255/255	235/235	216/216
112/118	95/95	150/153	117/126	133/139	119/122	96/99	126/184	159/162	120/126	92/96	155/164	89/98	116/174	91/100	96/105	124/182	129/129	257/262	133/142	96/96	110/110	176/185	131/137	165/168	145/145	191/191	265/268	166/170	138/138	152/152	255/255	235/235	216/216
112/118	95/101	150/153	126/126	133/139	119/122	99/102	126/184	159/162	123/126	96/99	164/164	89/98	116/174	91/100	96/105	124/182	129/129	257/257	133/142	96/96	110/110	176/189	131/137	165/168	145/145	183/191	268/271	166/170	138/138	164/164	255/255	235/235	216/233
112/118	95/95	150/153	117/126	133/139	119/122	96/99	126/184	159/162	120/126	92/96	155/164	89/98	116/174	91/100	96/105	124/182	129/129	257/262	133/142	96/96	110/110	176/185	131/137	165/168	145/145	191/191	265/268	166/170	138/138	152/152	255/255	235/235	216/216
112/112	95/101	150/153	117/117	133/136	119/122	96/99	126/180	159/162	117/120	92/96	149/155	92/98	116/170	94/100	99/105	124/178	125/129	257/262	133/136	96/96	110/110	185/185	131/134	165/165	145/145	191/191	265/268	166/166	132/138	152/158	240/255	230/235	216/222
112/118	89/98	145/146	115/117	133/133	114/115	96/96	180/184	165/168	117/123	92/92	149/152	92/92	170/174	94/94	99/99	178/182	125/125	269/274	136/136	96/113	110/110	185/189	131/131	168/177	145/145	185/191	265/265	170/170	127/132	161/161	255/255	235/238	233/233
112/118	95/101	151/153	109/117	136/139	120/122	99/102	184/184	159/168	117/126	96/99	146/149	89/89	174/174	91/91	96/96	182/182	125/129	262/262	130/142	113/113	110/110	176/193	134/137	168/177	145/151	185/191	268/273	170/170	127/138	152/164	255/255	235/235	233/233
112/118	95/101	151/153	109/117	136/139	120/122	99/102	184/184	159/168	117/126	96/99	146/149	89/89	174/174	91/91	96/96	182/182	125/129	262/262	130/142	113/113	110/110	176/193	134/137	168/177	145/151	185/191	268/273	170/170	127/138	152/164	255/255	235/235	233/233
112/118	95/101	151/153	109/117	136/139	120/122	99/102	184/184	159/168	117/126	96/99	146/149	89/89	174/174	91/91	96/96	182/182	125/129	262/262	130/142	113/113	110/110	176/193	134/137	168/177	145/151	185/191	268/273	170/170	127/138	152/164	255/255	235/235	233/233
112/118	95/101	151/153	109/117	136/139	120/122	99/102	184/184	159/168	117/126	96/99	146/149	89/89	174/174	91/91	96/96	182/182	125/129	262/262	130/142	113/113	110/110	176/193	134/137	168/177	145/151	185/191	268/273	170/170	127/138	152/164	255/255	235/235	233/233
118/118	95/101	150/153	109/117	136/136	119/122	96/102	180/184	159/168	117/126	92/99	146/149	89/113	170/174	91/115	96/119	178/182	125/129	263/274	142/142	96/113	110/110	185/193	134/134	162/168	145/151	185/191	265/273	166/170	132/138	161/164	255/255	230/235	222/233
112/112	89/101	146/151	117/120	133/136	115/120	96/105	175/180	159/171	117/126	92/102	149/152	89/92	165/170	91/94	96/99	173/178	129/129	262/274	136/142	96/96	110/110	176/185	131/134	168/177	145/151	185/191	265/274	170/170	132/135	161/164	255/255	235/238	233/233
112/118	89/89	153/153	117/120	136/139	122/122	96/96	180/184	159/159	117/117	92/92	149/164	89/95	170/174	91/97	96/102	178/182	118/129	262/274	142/142	96/113	110/110	193/193	134/137	168/168	145/145	185/200	265/265	166/170	127/138	164/164	255/255	235/235	233/233
112/112	98/101	153/153	117/117	133/136	122/122	96/102	180/183	159/159	117/123	92/99	149/155	92/98	170/173	94/100	99/105	178/181	125/129	257/262	136/142	96/96	110/110	185/189	131/134	165/165	145/145	185/191	265/271	166/166	132/138	164/164	240/255	235/244	233/233
112/118	89/95	151/153	117/126	133/136	120/122	96/105	180/184	159/171	117/126	92/102	149/164	89/92	170/174	91/94	96/99	178/182	125/129	262/274	142/142	96/108	110/123	176/185	131/134	165/168	145/145	191/194	265/274	170/170	130/135	161/164	240/255	235/235	222/233
112/112	89/89	146/153	117/120	133/133	115/122	102/108	175/180	156/171	117/123	99/105	149/152	92/92	165/170	94/94	99/99	173/178	125/125	274/274	133/142	96/96	110/110	185/185	131/131	177/177	145/145	185/185	273/277	170/170	127/135	161/161	255/255	230/235	233/233
112/112	95/95	150/153	126/126	133/133	119/122	102/105	184/187	159/171	111/126	99/102	155/164	89/92	174/177	91/94	96/99	182/185	129/129	257/274	142/148	96/96	110/123	176/193	131/131	168/168	138/145	191/200	271/274	166/166	138/138	164/164	255/255	235/235	216/222
112/118	89/95	151/153	117/126	133/136	120/122	96/105	180/184	159/171	117/126	92/102	149/164	89/92	170/174	91/94	96/99	178/182	125/129	262/274	142/142	96/108	110/123	176/185	131/134	165/168	145/145	191/194	265/274	170/170	130/135	161/164	240/255	235/235	222/233
112/118	89/95	151/153	117/126	133/136	120/122	96/105	180/184	159/171	117/126	92/102	149/164	89/92	170/174	91/94	96/99	178/182	125/129	262/274	142/142	96/108	110/123	176/185	131/134	165/168	145/145	191/194	265/274	170/170	130/135	161/164	240/255	235/235	222/233
112/118	89/95	151/153	117/126	133/136	120/122	96/105	180/184	159/171	117/126	92/102	149/164	89/92	170/174	91/94	96/99	178/182	125/129	262/274	142/142	96/108	110/123	176/185	131/134	165/168	145/145	191/194	265/274	170/170	130/135	161/164	240/255	235/235	222/233
112/112	89/89	146/146	117/120	133/133	115/115	96/96	175/180	156/165	117/120	92/92	149/152	92/92	165/170	94/94	99/99	173/178	125/125	274/274	142/142	108/113	110/110	185/185	131/131	165/165	145/145	179/179	265/265	170/170	127/135	161/161	255/255	238/238	233/233
112/112	89/95	148/153	117/126	133/136	117/122	96/96	184/184	168/168	117/117	92/92	149/164	89/92	174/174	91/94	96/99	182/182	125/129	262/274	142/142	96/113	110/123	185/185	131/134	162/168	145/145	179/191	265/265	170/170	127/138	152/161	255/255	230/235	222/233
112/118	89/95	150/153	117/117	133/133	119/122	96/102	180/187	159/159	111/117	92/99	149/155	92/98	170/177	94/100	99/105	178/185	125/129	257/262	133/142	96/108	110/123	185/185	131/131	165/165	138/145	194/200	265/271	166/170	130/138	161/164	240/255	235/235	216/233
112/118	89/95	146/148	115/126	133/133	115/117	96/99	180/184	162/168	120/126	92/96	146/164	92/95	170/174	94/97	99/102	178/182	125/129	262/280	133/142	96/113	110/110	176/185	131/131	165/177	145/145	185/191	265/268	170/170	135/138	161/164	240/255	235/238	222/233
118/118	95/101	150/153	109/126	133/136	119/122	96/96	180/184	159/159	117/117	92/92	146/164	89/104	170/174	91/106	96/111	178/182	129/133	263/269	142/142	96/113	110/110	185/193	131/134	168/168	151/151	185/191	265/265	166/170	138/138	164/164	255/255	235/235	233/233
118/118	95/95	150/153	109/126	133/136	119/122	96/96	180/184	159/159	111/126	92/92	146/164	89/89	170/174	91/91	96/96	178/182	129/129	263/269	142/142	96/113	110/110	176/189	131/134	168/177	151/151	191/200	265/265	166/170	138/138	152/164	255/255	235/235	233/233
109/112	89/101	146/153	117/126	133/139	115/122	96/108	184/184	159/165	117/123	92/105	149/164	92/95	174/174	94/97	99/102	182/182	125/129	262/262	133/142	96/96	110/110	185/189	131/137	165/177	145/151	191/194	265/277	166/170	127/138	161/164	240/255	230/235	233/233
118/118	89/101	146/153	117/120	133/136	115/122	96/108	175/180	159/165	123/126	92/105	149/152	89/92	165/170	91/94	96/99	173/178	125/129	262/274	133/142	96/96	110/110	176/185	131/134	165/168	145/151	185/191	265/277	166/170	127/135	161/164	255/255	230/235	233/233
118/118	89/95	148/151	117/126	133/139	117/120	96/105	180/184	168/171	117/126	92/102	149/164	89/92	170/174	91/94	96/99	178/182	129/129	262/269	142/142	96/108	110/123	176/185	131/137	165/168	145/151	185/194	265/274	170/170	130/135	161/161	240/255	235/235	222/233
112/118	89/95	146/148	117/126	133/136	115/117	96/102	175/184	168/171	117/117	92/99	149/164	89/92	165/174	91/94	96/99	173/182	125/125	262/274	133/142	96/108	110/110	185/185	131/134	162/162	145/145	179/191	265/273	170/170	132/138	161/161	255/255	230/238	222/233
112/118	89/95	153/153	120/126	133/133	122/122	96/99	180/184	159/159	117/126	92/96	158/164	101/104	170/174	103/106	108/111	178/182	129/133	262/269	142/152	96/96	110/110	193/193	131/131	168/177	148/151	191/200	265/268	166/166	138/138	164/164	255/255	235/235	233/233
118/118	95/95	146/151	117/117	133/139	115/120	96/108	184/184	165/168	117/126	92/105	149/149	92/95	174/174	94/97	99/102	182/182	125/129	262/269	133/142	96/96	110/110	176/185	131/137	168/177	145/151	185/191	265/277	170/170	130/130	152/152	255/255	230/235	222/222
109/115	95/98	143/145	115/120	133/142	112/114	96/96	167/179	156/159	117/123	82/92	152/158	92/95	157/169	94/97	99/102	177/177	125/125	269/269	133/133	96/96	110/110	176/189	131/140	162/165	145/145	177/191	257/265	166/170	135/141	149/152	255/258	235/235	233/233
118/118	89/89	153/153	115/117	133/139	122/122	96/96	180/184	159/159	126/126	92/92	149/152	92/92	170/174	94/94	99/99	178/182	129/129	262/262	142/142	108/113	110/123	176/176	131/137	168/168	151/151	185/194	265/265	170/170	127/132	152/164	255/255	230/235	233/233
112/118	95/101	148/153	117/117	136/139	117/122	96/96	180/180	159/168	126/126	92/92	149/149	89/92	170/170	91/94	96/99	178/178	129/129	262/274	130/142	96/108	110/123	172/176	134/137	162/165	151/151	179/185	265/265	166/170	132/138	152/161	240/255	230/235	233/233

112/112	89/101	151/151	117/126	133/139	120/120	96/96	184/184	159/168	126/126	92/92	149/164	92/113	174/174	94/115	99/119	182/182	129/129	262/274	142/142	108/113	110/110	176/176	131/137	165/168	145/151	191/194	265/265	170/170	127/138	152/164	240/255	235/235	233/233
112/115	89/95	146/150	109/117	133/136	115/119	96/108	184/184	159/162	114/126	92/105	146/149	89/92	174/174	91/94	96/99	182/182	125/129	263/274	133/142	96/96	110/110	176/180	131/134	162/168	151/151	185/200	265/277	166/166	135/138	161/164	255/255	235/238	233/233
118/118	95/101	151/153	109/126	133/136	120/122	96/99	184/184	159/159	126/126	92/96	146/164	89/92	174/174	91/94	96/99	182/182	129/129	262/262	130/142	108/113	110/123	176/176	131/134	162/165	145/151	185/200	265/268	170/170	138/138	161/164	240/255	230/235	233/233
112/118	101/101	151/153	117/117	133/136	120/122	96/96	180/180	159/159	117/117	92/92	149/149	89/89	170/170	91/91	96/96	178/178	129/129	262/274	142/142	113/113	110/110	193/193	131/134	168/177	151/151	185/191	265/265	170/170	132/138	164/164	255/255	230/235	233/233
112/118	101/101	151/151	117/117	136/139	120/120	96/96	180/184	159/159	126/126	92/92	149/149	92/95	170/174	94/97	99/102	178/182	129/129	262/262	142/142	96/113	110/123	176/176	134/137	165/168	145/151	185/191	265/265	166/170	138/138	152/152	240/255	235/235	233/233
112/118	89/101	145/146	112/117	133/133	114/115	85/99	170/184	159/162	120/120	79/96	149/149	92/92	160/174	94/94	99/99	168/182	125/125	269/280	133/142	96/96	110/123	185/189	131/131	162/162	145/145	184/185	252/268	170/170	127/130	152/161	255/255	233/238	227/233
112/118	89/89	151/153	117/117	136/139	120/122	96/102	126/180	159/159	117/126	92/99	149/155	89/89	116/170	91/91	96/96	124/178	129/129	257/274	136/142	96/113	110/110	176/185	134/137	165/168	145/145	185/194	265/273	166/170	127/127	161/161	255/255	230/235	233/233
112/118	95/95	153/153	117/117	133/136	122/122	96/102	180/180	159/168	126/126	92/99	149/149	89/89	170/170	91/91	96/96	178/178	129/129	262/269	130/142	96/113	110/110	176/176	131/134	165/168	145/151	179/191	265/273	166/170	130/135	161/164	240/255	235/235	222/233
112/112	89/89	143/146	117/117	133/133	112/115	96/108	180/184	162/165	117/117	92/105	149/149	92/92	170/174	94/94	99/99	178/182	125/125	274/274	133/142	108/113	110/123	185/185	131/131	165/177	145/151	179/185	265/277	170/170	127/127	161/161	255/255	230/238	222/233
112/112	89/89	143/146	115/117	133/133	112/115	96/105	180/184	165/171	117/123	92/102	146/149	92/92	170/174	94/94	99/99	178/182	125/125	274/280	136/142	96/113	110/110	185/185	131/131	162/177	145/145	179/179	265/274	170/170	135/135	152/161	255/255	235/235	233/233
112/112	95/95	153/153	117/117	136/136	122/122	96/105	180/184	159/171	117/117	92/102	149/149	89/95	170/174	91/97	96/102	178/182	129/129	274/274	142/142	96/108	110/123	193/193	134/134	165/165	151/151	191/191	265/274	166/170	130/132	161/164	240/255	230/235	222/233
118/118	95/95	151/153	109/117	133/133	120/122	96/99	180/184	159/159	126/126	92/96	146/149	89/95	170/174	91/97	96/102	178/182	129/129	262/269	130/142	96/113	110/110	176/176	131/131	162/168	145/151	179/200	265/268	166/170	135/138	161/164	255/255	235/235	233/233
118/118	95/101	153/153	117/126	133/136	122/122	96/96	180/184	159/159	117/126	92/92	149/164	89/89	170/174	91/91	96/96	178/182	129/129	269/274	142/142	96/113	110/110	176/193	131/134	168/177	151/151	185/191	265/265	166/170	132/135	161/164	255/255	235/235	233/233
118/118	89/95	151/153	117/126	136/139	120/122	96/96	180/184	159/159	117/126	92/92	149/164	89/89	170/174	91/91	96/96	178/182	129/129	269/274	130/142	96/113	110/110	176/193	134/137	168/177	151/151	179/191	265/265	166/170	132/138	161/164	255/255	235/235	233/233
112/118	95/95	151/153	117/126	133/139	120/122	96/105	180/184	168/171	117/126	92/102	149/164	92/95	170/174	94/97	99/102	178/182	129/129	262/274	142/142	108/113	110/123	176/193	131/137	165/168	151/151	185/191	265/274	166/170	130/138	152/152	240/255	235/235	222/233
118/118	95/95	151/153	109/117	133/136	120/122	96/96	180/184	159/159	117/126	92/92	146/149	89/89	170/174	91/91	96/96	178/182	129/129	262/262	130/130	96/96	110/110	176/193	131/134	168/168	151/151	191/200	265/265	166/170	135/138	161/164	255/255	235/235	233/233
112/118	89/89	151/153	117/117	139/139	120/122	96/96	126/184	159/159	117/126	92/92	149/155	89/92	116/174	91/94	96/99	124/182	129/129	257/274	136/142	96/96	110/123	176/185	137/137	165/168	145/151	179/185	265/265	166/170	127/138	152/161	255/255	230/235	233/233
112/118	95/95	153/153	117/117	133/139	122/122	96/96	126/164	159/159	117/117	92/92	149/155	89/89	116/153	91/91	96/96	124/162	125/129	257/274	136/142	96/96	110/110	185/185	131/137	162/168	145/145	191/191	265/265	170/170	132/138	161/161	255/255	230/235	216/222
118/118	95/101	151/153	117/126	136/139	120/122	96/96	180/184	159/159	126/126	92/92	149/164	92/95	170/174	94/97	99/102	178/182	129/129	262/274	142/142	96/113	110/123	176/176	134/137	165/168	145/151	185/185	265/265	166/170	132/138	152/164	240/255	235/235	233/233
112/118	89/95	148/151	117/126	136/139	117/120	96/96	180/184	159/168	117/126	92/92	149/164	89/95	170/174	91/97	96/102	178/182	129/129	262/262	130/142	96/108	110/110	176/193	134/137	165/177	145/151	185/194	265/265	166/170	130/138	164/164	240/255	235/235	233/233
112/112	89/89	146/146	115/117	133/133	115/115	96/108	180/180	162/165	117/123	92/105	146/149	92/92	170/170	94/94	99/99	178/178	125/125	274/274	136/136	108/113	110/123	185/189	131/131	177/177	145/145	179/185	265/277	170/170	127/127	152/161	255/255	230/230	222/233
118/118	89/101	151/152	117/126	133/136	120/121	88/96	180/180	159/168	117/126	82/92	149/164	92/113	170/174	94/115	99/119	178/182	125/129	274/274	136/142	96/108	110/110	176/185	131/134	165/165	145/151	185/191	255/265	166/170	132/138	152/164	240/240	230/238	222/233
112/118	95/101	148/153	117/117	133/139	117/122	96/96	180/180	159/159	117/126	92/92	149/149	92/95	170/170	94/97	99/102	178/178	129/129	262/274	130/142	96/108	110/123	176/193	131/137	168/168	151/151	185/191	265/265	166/170	138/138	161/161	255/255	230/235	233/233
118/118	95/95	150/151	109/120	133/136	119/120	96/105	184/184	159/171	126/126	92/102	146/158	89/95	174/174	91/97	96/102	182/182	125/129	263/269	130/142	96/96	110/110	172/176	131/134	162/168	145/151	185/200	265/274	166/166	138/138	152/161	255/255	235/235	233/233
112/112	89/95	151/153	117/123	136/136	120/122	96/96	180/184	159/159	117/117	92/92	149/161	92/95	170/174	94/97	99/102	178/182	129/129	262/262	142/142	96/108	110/123	193/193	134/134	168/168	145/151	191/194	265/265	170/170	138/138	152/164	255/255	230/235	233/233
112/118	95/95	148/153	117/126	133/133	117/122	96/99	180/184	159/168	126/126	92/96	149/164	92/95	170/174	94/97	99/102	178/182	129/129	262/269	130/142	96/108	110/110	172/176	131/131	162/165	145/145	179/191	265/268	170/170	135/138	152/161	240/255	235/235	222/233
112/118	95/95	151/153	115/126	136/139	120/122	96/99	180/184	159/159	117/126	92/96	152/164	89/92	170/174	91/94	96/99	178/182	129/129	262/269	130/142	108/113	110/110	193/193	134/137	162/177	145/151	191/191	265/268	170/170	135/138	164/164	255/255	235/235	233/233
112/118	95/95	150/153	109/117	133/136	119/122	96/99	180/184	159/159	126/126	92/96	146/149	89/95	170/174	91/97	96/102	178/182	129/129	262/263	130/142	96/113	110/110	176/176	131/134	168/168	145/151	179/200	265/268	166/170	135/138	161/164	255/255	235/235	233/233
112/118	89/101	151/153	117/126	136/139	120/122	96/96	180/184	159/168	126/126	92/92	149/164	92/113	170/174	94/115	99/119	178/182	129/129	262/274	142/142	108/113	110/123	176/176	134/137	165/168	145/151	185/194	265/265	166/170	132/138	152/161	240/255	230/235	233/233
112/118	89/95	153/153	117/120	133/139	122/122	96/105	126/175	159/171	117/126	92/102	152/155	89/92	116/165	91/94	96/99	124/173	129/129	257/274	136/142	96/96	110/110	176/185	131/137	165/168	145/145	191/194	265/274	166/170	127/138	152/161	255/255	230/235	216/233
112/118	95/95	151/153	117/117	136/139	120/122	96/96	180/184	159/159	117/126	92/92	149/149	89/89	170/174	91/91	96/96	178/182	129/129	262/274	142/142	113/113	110/110	176/193	134/137	168/177	151/151	185/191	265/265	170/170	132/138	161/164	255/255	235/238	233/233
112/112	89/89	143/151	117/117	133/136	112/120	105/108	184/184	162/171	117/117	102/105	149/149	89/92	174/174	91/94	96/99	182/182	125/125	262/274	133/142	96/113	110/123	185/189	131/134	162/177	145/145	179/185	274/277	170/170	130/132	152/161	255/255	230/238	233/233
112/118	95/101	151/153	117/126	133/133	120/122	96/96	180/184	159/168	117/126	92/92	149/164	89/92	170/174	91/94	96/99	178/182	129/129	262/262	130/142	96/113	110/123	176/185	131/131	165/165	145/151	185/200	265/265	170/170	138/138	152/152	240/240	235/235	233/233
112/118	89/95	151/153	117/126	133/139	120/122	96/96	180/184	159/168	126/126	92/92	149/164	89/92	170/174	91/94	96/99	178/182	129/129	262/262	142/142	108/113	110/123	176/176	131/137	165/168	145/151	179/194	265/265	170/170	138/138	152/164	240/255	235/235	233/233
112/118	89/95	153/153	115/117	133/136	122/122	96/96	184/184	159/159	117/126	92/92	149/152	89/95	174/174	91/97	96/102	182/182	129/129	262/274	142/142	96/113	110/110	176/193	131/134	165/168	151/151	179/191	265/265	170/170	135/138	161/164	255/255	230/235	233/233
118/118	95/101	148/153	117/126	133/136	117/122	96/96	180/184	159/168	117/126	92/92	149/164	95/113	170/174	97/115	102/119	178/182	129/129	274/274	142/142	96/108	110/123	176/193	131/134	165/177	151/151	191/194	265/265	166/170	130/132	164/164	240/255	235/235	233/233
112/118	89/89	151/153	117/126	136/136	120/122	96/96	180/184	159/159	117/126	92/92	149/164	92/113	170/174	94/115	99/119	178/182	129/129	262/274	142/142	96/108	110/123	176/193	134/134	165/168	145/151	185/194	265/265	166/170	127/138	152/161	240/255	230/235	233/233
112/118	95/95	148/153	117/126	136/139	117/122	96/96	180/184	159/168	117/126	92/92	149/164	89/89	170/174	91/91	96/96	178/182	129/129	269/274	142/142	96/113	110/110	176/185	134/137	168/177	145/151	185/191	265/265	170/170	132/138	161/161	255/255	235/238	222/233
112/118	95/95	151/151	117/126	133/139	120/120	96/105	180/180	168/171	117/126	92/102	149/164	89/92	170/170	91/94	96/99	178/178	125/129	262/269	130/142	96/108	110/110	172/185	131/137	162/168	145/151	185/185	265/274	170/170	130/135	161/161	255/255	235/235	222/233
112/118	89/101	145/146	117/117	133/136	114/115	96/108	180/180	165/165	120/120	92/105	149/149	92/92	170/174	94/94	99/99	178/182	125/125	274/274	133/142	96/96	110/123	185/189	131/134	162/168	145/145	184/185	265/277	170/170	127/127	152/161	255/255	233/238	227/233
112/118	89/101	151/153	117/117	136/139	120/122	96/96	180/184	159/159	117/117	92/92	149/149	89/89	170/174	91/91	96/96	178/182	129/129	262/274	142/142	96/113	110/110	193/193	134/137	168/177	151/151	179/191	265/265	166/170	132/135	164/164	255/255	235/235	233/233
112/118	89/89	148/151	117/126	133/136	117/120	96/105	184/184	159/171	126/126	92/102	149/164	92/95	174/174	94/97	99/102	182/182	129/129	262/274	142/142	96/108	110/123	176/176	131/134	165/165	151/151	191/194	265/274	166/170	130/138	161/164	240/255	230/235	222/233
112/118	89/95	151/153	117/117	133/139	120/122	96/99	180/184	159/162	117/126	92/96	149/149	89/89	170/174	91/91	96/96	178/182	125/129	262/274	130/142	96/113	110/110	176/193	131/137	165/168	151/151	179/185	265/268	166/170	135/138	161/164	255/255	235/235	233/233
112/112	89/101	146/153	117/117	133/136	115/122	96/99/105	184/184	159/162/165	117/123/126	92/96/102	149/149	92/92	174/174	94/94	99/99	182/182	125/129	274/280	136/142	96/96	110/110	176/185	131/134	162/165/168	145/151	179/191	265/268/274	166/170	127/135	152/161/164	255/255	230/238	233/233
112/118	95/101	151/153	117/117	136/139	120/122	96/96	180/184	159/159	117/117	92/92	149/149	89/89	170/174	91/91	96/96	178/182	129/129	274/274	130/142	96/96	110/110	193/193	134/137	168/168	145/151	185/191	265/265	166/166	132/138	161/164	255/255	235/235	233/233
118/118	89/101	153/153	117/117	136/136	122/122	96/96	180/184	159/159	117/117	92/92	149/149	89/89	170/174	91/91	96/96	178/182	129/129	262/274	142/142	96/113	110/110	193/193	134/134	165/168	151/151	179/191	265/265	166/170	135/138	164/164	255/255	235/235	233/233
118/118	89/95	146/150	109/120	133/136	115/119	96/108	175/184	159/162	114/126	92/105	146/152	89/89	165/174	91/91	96/96	173/182	125/129	263/274	133/142	113/113	110/110	176/180	131/134	162/168	145/151	185/200	265/277	170/170	127/138	161/164	255/255	235/235	233/233
112/118	95/95	151/153	115/117	139/139	120/122	96/105	184/184	159/171	126/126	92/102	149/152	92/95	174/174	94/97	99/102	182/182	125/129	262/262	130/130	96/108	110/110	172/176	137/137	162/162	145/145	179/185	265/274	170/170	135/138	161/161	255/255	235/235	222/233
112/118	95/101	151/153	117/117	136/139	120/122	96/96	180/180	159/159	117/126	92/92	149/149	89/89	170/170	91/91	96/96	178/178	129/129	262/274	142/142	96/113	110/110	176/193	134/137	168/168	151/151	185/191	265/265	166/170	138/138	164/164	255/255	235/235	233/233
112/112	95/95	151/153	117/126	133/136	120/122	96/105	184/184	159/171	117/126	92/102	149/164	89/92	174/174	91/94	96/99	182/182	129/129	262/269	142/142	108/113	110/123	176/193	131/134	165/168	151/151	185/194	265/274	166/170	130/138	164/164	240/255	235/235	222/233
112/118	89/95	151/151	117/117	136/136	120/120	96/96	180/180	159/168	126/126	92/92	149/149	89/92	170/170	91/94	96/99	178/178	129/129	262/263	142/142	96/108	110/123	176/176	134/134	165/177	145/151	191/194	265/265	170/170	132/138	152/164	240/255	230/235	233/233
112/112	89/89	153/153	115/117	133/136	122/122	96/96	180/184	159/168	126/126	92/92	149/152	92/95	170/174	94/97	99/102	178/182	129/129	262/274	142/142	113/113	110/123	176/176	131/134	168/168	151/151	179/194	265/265	166/170	132/135	152/161	255/255	230/235	233/233
112/118	95/95	150/151	117/126	133/136	119/120	96/105	184/184	159/171	117/126	92/102	149/164	89/92	170/174	91/94	96/99	178/182	129/129	262/262	130/142	96/108	110/110	176/193	131/134	165/165	145/145	179/191	265/274	166/170	138/138	164/164	240/240	235/235	233/233
118/118	89/95	151/153	115/117	133/136	120/122	96/96	184/184	159/159	117/126	92/92	149/152	89/92	174/174	91/94	96/99	182/182	129/129	262/274	130/142	113/113	110/110	176/193	131/134	168/168	151/151	179/185	265/265	170/170	127/138	161/164	255/255	230/235	222/233
112/118	89/95	150/151	117/117	136/136	119/120	96/96	180/180	159/168	126/126	92/92	149/149	95/113	170/170	97/115	102/119	178/178	129/129	262/274	142/142	96/108	110/110	176/176	134/134	165/177	145/151	191/194	265/265	170/170	132/138	152/164	240/255	235/235	233/233
112/118	89/95	151/151	117/117	136/136	120/120	96/96	180/180	159/168	126/126	92/92	149/149	89/92	170/170	91/94	96/99	178/178	129/129	262/263	142/142	96/108	110/123	176/176	134/134	165/177	145/151	191/194	265/265	166/170	132/138	152/164	240/255	230/235	233/233
112/112	89/95	148/150	120/126	133/139	117/119	96/108	175/180	165/168	117/123	92/105	152/164	89/92	165/170	91/94	96/99	173/178	125/129	262/274	133/142	96/96	110/123	185/185	131/137	165/165	145/151	185/194	265/277	170/170	130/135	152/161	240/255	230/235	222/233
112/112	89/89	146/151	117/117	133/139	115/120	96/108	164/180	159/165	117/126	92/105	149/149	92/92	153/170	94/94	99/99	162/178	125/125	262/274	142/142	96/96	110/110	176/185	131/137	168/177	145/145	179/185	265/277	170/170	132/135	161/161	255/255	235/238	233/233
118/118	89/95	148/151	117/126	133/139	117/120	96/105	180/184	168/171	117/126	92/102	149/164	89/92	170/174	91/94	96/99	178/182	125/129	262/274	142/142	96/96	110/123	176/185	131/137	165/177	145/151	179/191	265/274	170/170	135/138	152/161	240/255	235/238	222/233
112/118	95/101	151/153	109/126	133/139	120/122	96/96	184/184	159/168	126/126	92/92	146/164	89/92	174/174	91/94	96/99	182/182	125/129	263/274	142/142	113/113	110/123	176/176	131/137	162/165	145/151	179/185	265/265	166/170	138/138	152/152	240/255	235/235	233/233
112/112	89/89	150/153	117/120	133/133	119/122	105/108	175/180	165/171	117/123	102/105	149/152	92/92	165/170	94/94	99/99	173/178	125/129	262/274	133/142	96/96	110/123	185/185	131/131	165/177	145/145	179/194	274/277	170/170	135/135	161/164	255/255	235/235	233/233
118/118	95/101	151/153	109/117	133/136	120/122	96/96	180/184	159/159	126/126	92/92	146/149	89/92	170/174	91/94	96/99	178/182	129/129	262/274	130/142	96/108	110/123	176/176	131/134	168/168	145/151	179/185	265/265	166/170	135/138	161/161	255/255	235/235	233/233
112/118	95/101	151/153	117/126	139/139	120/122	96/105	180/184	168/171	126/126	92/102	149/164	92/113	170/174	94/115	99/119	178/182	129/129	262/274	142/142	96/113	110/123	176/176	137/137	165/165	145/151	185/191	265/274	166/170	138/138	152/161	240/240	230/235	222/233
112/118	95/101	153/153	117/117	133/139	122/122	96/96	180/180	159/168	126/126	92/92	149/149	89/92	170/170	91/94	96/99	178/178	129/129	262/262	130/142	108/113	110/123	176/176	131/137	165/168	145/151	185/200	265/265	170/170	138/138	152/164	240/255	235/235	233/233
118/118	95/95	153/153	109/109	133/133	122/122	96/96	184/184	159/159	126/126	92/92	146/146	89/95	174/174	91/97	96/102	182/182	125/129	262/263	130/142	113/113	110/110	172/176	131/131	162/162	145/151	179/191	265/265	170/170	138/138	152/161	255/255	235/235	233/233
112/118	89/89	146/151	115/117	133/136	115/120	108/108	180/180	162/165	117/126	105/105	146/149	89/92	170/170	91/94	96/99	178/178	125/125	262/274	142/142	96/113	110/110	176/185	131/134	177/177	145/145	185/191	277/277	170/170	127/135	161/161	255/255	235/238	233/233
112/118	95/101	150/153	117/117	136/139	119/122	96/96	180/180	159/159	117/126	92/92	149/149	92/95	170/170	94/97	99/102	178/178	129/129	262/262	142/142	96/108	110/110	176/193	134/137	165/168	145/151	185/191	265/265	166/170	132/138	161/164	240/255	235/235	233/233
118/118	89/95	153/153	117/117	136/139	122/122	96/96	180/180	159/168	117/126	92/92	149/164	92/92	170/174	94/94	99/99	178/178	129/129	262/262	142/142	96/113	110/110	176/193	134/137	165/168	151/151	185/194	265/265	166/166	132/138	152/164	240/255	235/235	233/233
112/118	95/95	151/153	117/126	133/133	120/122	96/105	180/184	159/171	126/126	92/102	149/164	89/92	170/174	91/94	96/99	178/182	129/129	262/274	130/142	108/113	110/123	176/176	131/131	165/168	145/151	179/191	265/274	170/170	135/138	161/164	240/255	235/235	222/233
112/118	89/101	145/146	112/117	133/136	114/115	85/108	170/180	159/165	117/117	79/105	149/149	92/92	160/174	94/94	99/99	168/178	125/125	269/274	133/142	96/111	110/123	185/189	131/134	168/168	145/145	179/184	252/277	170/174	127/130	161/161	255/255	233/238	227/233
112/118	89/95	148/153	117/126	136/139	117/122	96/102	180/184	168/168	126/126	92/99	149/164	89/95	170/174	91/97	96/102	178/182	129/129	262/262	142/142	96/108	110/110	176/176	134/137	165/168	151/151	185/191	265/273	166/170	127/130	152/164	240/255	235/235	222/233
112/112	95/101	148/151	117/126	136/139	117/120	96/105	180/184	159/171	126/126	92/102	149/164	92/113	170/174	94/115	99/119	178/182	129/129	262/274	142/142	108/108	110/123	176/176	134/137	165/168	145/151	185/191	265/274	170/170	130/138	152/161	240/255	230/235	222/233
112/118	89/101	148/153	126/126	136/139	117/122	96/105	184/184	168/171	126/126	92/102	164/164	92/113	174/174	94/115	99/119	182/182	129/129	262/274	142/142	108/113	110/123	176/176	134/137	165/165	145/151	185/191	265/274	166/170	132/138	152/152	240/240	235/235	222/233
118/118	95/101	146/151	117/126	133/133	115/120	96/96	180/184	159/159	117/126	92/92	149/164	89/89	170/174	91/91	96/96	178/182	129/129	262/280	142/142	96/113	110/110	176/185	131/131	165/165	145/151	179/185	265/265	170/170	135/138	152/164	240/240	235/235	233/233
118/118	89/101	151/151	117/117	133/139	120/120	96/105	184/184	159/171	126/126	92/102	149/149	89/92	174/174	91/94	96/99	182/182	129/129	262/274	142/142	96/96	110/123	176/176	131/137	165/168	151/151	179/191	265/274	166/170	127/138	152/164	240/255	235/235	233/233
118/118	95/95	148/153	109/126	136/139	117/122	96/96	184/184	159/168	117/126	92/92	146/164	89/113	174/174	91/115	96/119	182/182	129/129	274/274	130/142	96/113	110/110	176/185	134/137	165/168	151/151	191/191	265/265	170/170	132/138	152/161	240/255	235/235	233/233
112/118	95/101	151/153	117/117	133/133	120/122	96/96	180/180	159/168	126/126	92/92	149/149	92/95	170/170	94/97	99/102	178/178	129/129	262/262	130/130	108/113	110/123	176/176	131/131	165/165	151/151	179/185	265/265	166/170	138/138	161/164	240/240	235/235	233/233
118/118	95/101	153/153	117/117	133/136	122/122	96/105	180/180	159/171	117/126	92/102	149/149	92/113	170/170	94/115	99/119	178/178	129/129	274/274	142/142	113/113	110/110	176/193	131/134	168/177	151/151	191/191	265/274	166/170	132/138	152/164	255/255	235/235	222/233
112/118	95/95	151/153	117/117	136/139	120/122	96/105	180/180	159/171	117/126	92/102	149/149	89/92	170/170	91/94	96/99	178/178	129/129	262/274	142/142	108/113	110/110	176/193	134/137	165/168	145/151	179/185	265/274	166/170	138/138	152/164	240/255	235/235	222/233
112/118	89/89	145/153	117/117	133/133	114/122	96/96	126/126	159/159	117/120	92/92	143/155	89/92	116/116	91/94	96/99	124/124	118/129	257/269	136/139	96/96	110/110	176/185	131/131	165/165	145/145	179/194	265/265	170/170	124/127	152/158	255/255	227/235	233/233
112/118	89/95	151/151	117/126	133/136	120/120	96/96	180/184	159/168	117/126	92/92	149/164	89/113	170/174	91/115	96/119	178/182	129/129	262/274	142/142	96/108	110/110	176/185	131/134	165/165	145/151	179/194	265/265	170/170	130/135	152/161	240/240	235/235	233/233
112/118	95/101	148/151	117/117	133/136	117/120	96/96	180/180	159/168	126/126	92/92	149/149	89/92	170/170	91/94	96/99	178/178	125/129	262/274	142/142	96/96	110/123	176/176	131/134	162/165	151/151	191/191	265/265	170/170	132/135	152/161	240/255	235/235	222/233
112/112	89/95	146/151	117/126	136/139	115/120	108/108	180/184	165/165	117/126	105/105	149/164	92/92	170/174	94/94	99/99	178/182	125/129	262/274	142/142	96/96	110/110	176/185	134/137	168/177	145/151	191/191	277/277	170/170	130/135	161/164	255/255	235/238	222/233
112/118	95/101	151/153	117/126	136/139	120/122	96/96	184/184	159/159	117/126	92/92	149/164	95/113	174/174	97/115	102/119	182/182	129/129	274/274	142/142	113/113	110/110	176/185	134/137	165/168	151/151	185/185	265/265	170/170	132/135	152/164	240/255	230/238	222/233
112/118	89/89	151/151	117/117	136/136	120/120	96/96	180/180	159/168	117/126	92/92	149/149	92/113	170/174	94/115	99/119	178/178	129/129	274/274	142/142	96/108	110/123	176/176	131/134	165/168	145/145	191/194	265/265	166/170	127/132	152/161	240/255	230/235	233/233
112/118	95/101	153/153	109/117	133/139	122/122	96/96	180/184	159/168	126/126	92/92	146/149	95/113	170/174	97/115	102/119	178/182	129/129	262/262	142/142	113/113	110/110	176/176	131/137	162/165	151/151	185/191	265/265	166/170	132/138	152/161	240/255	235/235	233/233
112/118	89/95	151/153	109/117	133/136	120/122	96/96	180/184	159/159	126/126	92/92	146/149	89/92	170/174	91/94	96/99	178/182	125/129	263/274	142/142	108/113	110/123	176/176	131/134	162/165	145/151	179/194	265/265	170/170	138/138	161/161	240/255	235/235	233/233
112/118	89/95	151/153	109/117	133/136	120/122	96/96	180/184	159/159	126/126	92/92	146/149	92/113	170/174	94/115	99/119	178/182	129/129	262/263	142/142	108/113	110/123	176/176	131/134	162/165	151/151	179/185	265/265	166/170	132/138	152/152	240/255	230/235	233/233
112/112	89/95	148/153	120/126	139/139	117/122	96/96	175/184	159/168	126/126	92/92	152/164	89/95	165/174	91/97	96/102	173/182	129/129	257/274	142/142	96/96	110/110	176/176	137/137	165/165	145/145	191/194	265/265	170/170	127/138	152/152	240/255	235/235	222/233
112/118	95/101	151/153	117/117	136/139	120/122	96/96	180/180	159/159	117/126	92/92	149/149	92/92	170/170	94/94	99/99	178/178	129/129	262/274	142/142	108/113	110/110	176/193	134/137	165/168	151/151	185/185	265/265	166/170	138/138	152/164	240/255	235/235	233/233
118/118	95/95	148/151	117/126	133/139	117/120	96/105	180/184	159/171	117/126	92/102	149/164	92/95	170/174	94/97	99/102	178/182	129/129	262/274	130/142	96/108	110/123	176/193	131/137	162/165	145/151	179/191	265/274	166/170	138/138	161/164	240/255	235/235	222/233
112/112	89/89	143/146	115/117	133/133	112/115	96/108	176/180	162/165	117/123	92/105	146/149	92/92	166/170	94/94	99/99	174/178	125/125	274/274	133/136	108/113	110/110	185/185	131/131	162/165	145/145	185/185	265/277	170/170	127/127	152/161	255/255	230/230	233/233
112/118	89/95	148/153	117/126	139/139	117/122	96/96	180/184	159/168	117/126	92/92	149/164	92/95	170/174	94/97	99/102	178/182	129/129	262/262	130/142	108/113	110/123	176/193	137/137	165/168	145/145	185/194	265/265	170/170	130/138	152/161	240/255	235/235	222/233
112/118	95/101	151/153	117/126	133/136	120/122	96/105	180/184	159/171	126/126	92/102	149/164	89/92	170/174	91/94	96/99	178/182	129/129	262/274	142/142	96/108	110/123	176/176	131/134	165/168	151/151	191/191	265/274	166/170	130/132	164/164	240/255	235/235	222/233
115/118	89/95	151/153	109/126	133/139	120/122	96/105	184/184	159/171	126/126	92/102	146/164	89/92	174/174	91/94	96/99	182/182	129/129	262/269	142/142	113/113	110/123	176/176	131/137	162/165	145/151	179/191	265/274	166/170	132/138	152/161	240/255	235/235	222/233
112/118	95/95	151/153	109/117	133/136	120/122	96/99	180/184	159/159	126/126	92/96	146/149	89/95	170/174	91/97	96/102	178/182	129/129	262/269	130/130	96/113	110/110	176/176	131/134	162/168	145/151	185/200	265/268	170/170	138/138	164/164	255/255	235/235	233/233
112/112	89/95	153/153	117/117	133/136	122/122	96/99	180/180	159/168	126/126	92/96	149/149	89/89	170/170	91/91	96/96	178/178	129/129	262/262	142/142	96/96	110/110	176/176	131/134	165/168	145/151	179/185	265/268	166/166	127/135	161/164	240/255	235/235	233/233
112/118	95/101	150/153	117/117	133/139	119/122	96/96	180/180	159/159	126/126	92/92	149/149	89/92	170/170	91/94	96/99	178/178	129/129	262/262	142/142	96/113	110/123	176/176	131/137	165/165	145/151	185/191	265/265	166/170	132/135	164/164	240/240	235/235	222/233
112/118	89/95	148/151	117/126	136/139	117/120	96/105	180/184	159/171	117/126	92/102	149/164	89/92	170/174	91/94	96/99	178/182	129/129	262/262	130/142	113/113	110/123	176/193	134/137	165/168	145/151	185/191	265/274	166/170	138/138	152/164	240/255	235/235	222/222
112/118	89/95	153/153	120/126	133/139	122/122	96/99	184/184	159/168	126/126	92/96	158/164	92/113	174/174	94/115	99/119	182/182	129/129	269/274	130/142	108/108	110/110	176/176	131/137	165/168	145/145	185/185	265/268	170/170	138/138	152/161	240/255	235/235	222/233
112/118	95/95	151/151	117/126	133/133	120/120	96/96	180/184	159/168	126/126	92/92	149/164	92/95	170/174	94/97	99/102	178/182	129/129	262/262	130/130	96/113	110/110	176/176	131/131	162/168	151/151	185/200	265/265	166/170	135/138	161/164	255/255	235/235	222/233
112/118	89/89	146/148	117/126	133/136	115/117	96/96	180/184	159/168	117/126	92/92	149/164	92/113	170/174	94/115	99/119	178/182	129/129	262/274	142/142	96/108	110/123	176/185	131/134	165/168	151/151	185/191	265/265	170/170	127/132	152/161	240/255	235/235	222/233
118/118	95/101	151/151	117/117	133/136	120/120	99/105	180/180	159/171	126/126	96/102	149/149	89/92	170/170	91/94	96/99	178/178	129/129	262/274	142/142	108/113	110/123	176/176	131/134	162/165	145/151	191/200	268/274	170/170	135/138	161/161	240/255	230/235	233/233
112/118	95/101	148/153	117/126	133/139	117/122	96/105	180/184	159/171	126/126	92/102	149/164	89/92	170/174	91/94	96/99	178/182	129/129	262/262	142/142	108/113	110/110	176/176	131/137	162/165	145/145	179/185	265/274	166/170	138/138	152/164	240/255	235/235	233/233
112/118	95/95	151/151	117/117	133/136	120/120	96/96	180/180	159/168	117/126	92/92	149/164	92/95	170/170	94/97	99/102	178/178	129/129	262/262	130/142	96/113	110/123	176/193	131/134	165/168	151/151	179/200	265/265	166/170	135/138	161/164	240/255	235/235	233/233
112/118	101/101	148/153	117/126	136/136	117/122	96/105	180/184	159/171	126/126	92/102	149/164	89/92	170/174	91/94	96/99	178/182	129/129	262/274	142/142	108/113	110/123	176/176	134/134	165/168	151/151	185/185	265/274	170/170	138/138	152/164	240/255	235/235	233/233
118/118	95/95	148/151	117/126	133/133	117/120	99/105	180/184	159/171	126/126	96/102	149/164	89/92	170/174	91/94	96/99	178/182	129/129	269/274	130/142	96/108	110/110	176/176	131/131	162/165	145/151	191/200	268/274	166/170	130/138	164/164	240/255	235/235	222/233
112/118	89/101	146/148	117/126	133/139	115/117	96/108	180/184	165/168	117/126	92/105	149/164	89/92	170/174	91/94	96/99	178/182	125/129	262/262	142/142	96/108	110/123	176/185	131/137	165/165	151/151	185/185	265/277	170/170	138/138	152/164	240/240	235/235	233/233

112/118	89/101	150/153	115/117	133/139	119/122	96/96	180/184	159/159	126/126	92/92	149/152	92/92	170/174	94/94	99/99	178/182	129/129	262/274	142/142	96/113	110/110	176/176	131/137	165/165	151/151	179/185	265/265	166/170	135/138	161/164	240/240	230/235	233/233
118/118	89/101	151/153	117/126	133/136	120/122	96/96	180/184	159/168	117/117	92/92	149/164	89/92	170/174	91/94	96/99	178/182	129/129	262/274	142/142	96/113	110/110	185/193	131/134	165/168	145/151	179/191	265/265	170/170	138/138	161/164	240/255	235/238	233/233
112/118	95/101	151/153	117/126	136/136	120/122	96/96	184/184	159/168	117/126	92/92	149/164	89/92	174/174	91/94	96/99	182/182	129/129	274/274	142/142	96/108	110/123	176/193	134/134	165/165	151/151	185/191	265/265	166/170	130/132	161/164	240/255	235/235	222/233
118/118	95/101	151/153	117/117	133/136	120/122	96/99	180/180	159/159	117/126	92/96	149/149	89/95	170/170	91/97	96/102	178/178	129/129	262/274	130/142	113/113	110/123	176/193	131/134	168/177	145/151	191/200	265/268	170/170	138/138	164/164	255/255	230/235	233/233
112/112	101/101	148/151	117/126	133/136	117/120	96/96	180/184	159/159	126/126	92/92	149/164	89/92	170/174	91/94	96/99	178/182	129/129	262/280	142/142	96/108	110/123	176/176	131/134	165/168	151/151	185/185	265/265	170/170	138/138	164/164	240/255	235/235	233/233
118/118	95/101	153/153	117/126	139/139	122/122	96/96	180/184	159/168	117/126	92/92	149/164	92/113	170/174	94/115	99/119	178/182	129/129	262/274	142/142	113/113	110/110	176/193	137/137	165/165	145/151	185/185	nd.	166/170	135/138	152/164	240/240	235/235	233/233
118/118	95/101	153/153	109/117	133/136	122/122	96/96	180/184	159/159	117/126	92/92	146/149	89/92	170/174	91/94	96/99	178/182	129/129	274/274	130/142	96/113	110/123	176/193	131/134	168/177	145/151	179/185	265/265	170/170	138/138	161/164	255/255	235/235	233/233
112/112	95/101	151/151	109/117	133/136	120/120	96/96	180/184	159/168	126/126	92/92	146/149	92/95	170/174	94/97	99/102	178/182	129/129	262/269	130/142	113/113	110/123	176/176	131/134	165/168	151/151	179/185	265/265	166/170	132/135	161/164	240/255	235/235	233/233
118/118	95/101	151/151	109/126	133/136	120/120	96/99	184/184	159/168	126/126	92/96	146/164	89/113	174/174	91/115	96/119	182/182	129/129	269/274	142/142	96/113	110/110	176/176	131/134	165/168	145/151	179/185	265/268	166/166	132/138	152/164	240/255	230/235	233/233
112/118	89/101	151/153	117/126	136/139	120/122	96/96	180/184	159/168	117/126	92/92	149/164	89/113	170/174	91/115	96/119	178/182	129/129	274/274	142/142	108/113	110/110	176/193	134/137	165/168	145/151	191/191	265/265	170/170	138/138	152/164	240/255	230/235	222/233
112/118	95/101	151/153	117/126	136/136	120/122	96/105	180/184	159/171	117/126	92/102	149/164	92/95	170/174	94/97	99/102	178/182	129/129	262/274	142/142	108/113	110/123	176/193	134/134	165/168	151/151	185/185	265/274	170/170	138/138	152/164	240/255	235/235	222/233
112/118	95/95	148/153	117/126	139/139	117/122	96/105	184/184	159/171	117/126	92/102	149/164	89/92	174/174	91/94	96/99	182/182	129/129	274/274	142/142	108/113	110/110	176/193	137/137	165/168	145/151	191/191	265/274	170/170	130/132	164/164	240/255	235/235	222/233
118/118	89/101	153/153	117/126	136/139	122/122	96/96	180/184	159/159	126/126	92/92	149/164	89/92	170/174	91/94	96/99	178/182	129/129	262/262	142/142	96/113	110/123	176/176	134/137	165/168	145/151	191/194	265/265	166/166	132/138	161/164	240/255	230/235	233/233
118/118	89/89	146/153	115/117	133/136	115/122	96/96	180/184	159/159	117/117	92/92	149/152	89/92	170/174	91/94	96/99	178/182	129/129	262/274	142/142	96/96	110/123	185/193	131/134	165/165	145/151	185/194	265/265	166/170	138/138	161/161	240/240	230/235	233/233
112/112	89/101	153/153	115/117	133/136	122/122	96/96	180/184	159/159	117/126	92/92	149/152	89/95	170/174	91/97	96/102	178/182	129/129	262/274	142/142	96/108	110/110	176/185	131/134	165/177	151/151	191/194	265/265	170/170	130/138	161/164	240/255	230/235	233/233
115/118	89/95	148/151	117/126	133/133	117/120	96/96	180/184	159/168	126/126	92/92	149/164	89/92	170/174	91/94	96/99	178/182	129/129	262/274	142/142	96/113	110/123	176/176	131/131	165/168	145/151	185/191	265/265	170/170	130/132	152/161	240/255	235/235	222/233
118/118	89/101	148/153	126/126	133/136	117/122	96/105	180/184	159/171	117/126	92/102	164/164	92/113	170/174	94/115	99/119	178/182	129/129	269/274	142/142	96/108	110/123	176/193	131/134	165/177	145/151	191/194	265/274	166/170	130/138	152/164	240/255	235/235	233/233
118/118	95/101	151/153	126/126	136/139	120/122	96/96	180/184	159/168	117/126	92/92	164/164	89/92	170/174	91/94	96/99	178/182	129/129	269/274	142/142	96/113	110/123	176/193	134/137	165/168	151/151	191/191	265/265	170/170	130/138	164/164	240/255	235/235	222/233
112/118	89/95	151/153	117/117	133/136	120/122	96/96	126/180	159/159	126/126	92/92	149/155	89/92	116/170	91/94	96/99	124/178	129/129	274/274	142/142	96/96	110/110	176/176	131/134	168/168	145/145	185/191	265/265	166/166	127/138	152/164	255/255	230/235	216/233
118/118	89/95	151/153	117/117	133/139	120/122	96/105	126/180	159/171	117/126	92/102	149/155	89/92	116/170	91/94	96/99	124/178	129/129	257/262	142/142	96/113	110/110	176/193	131/137	168/168	145/151	185/191	265/274	166/170	127/138	161/161	255/255	230/235	216/233
112/118	89/95	151/153	117/120	133/136	120/122	96/96	175/180	159/159	117/117	92/92	149/152	89/92	165/170	91/94	96/99	173/178	129/129	257/274	136/142	96/96	110/110	185/193	131/134	165/168	145/145	185/191	265/265	166/166	127/127	161/161	255/255	230/230	216/233
112/112	89/95	151/151	117/117	133/139	120/120	96/105	180/180	159/171	117/126	92/102	149/149	89/92	170/170	91/94	96/99	178/178	129/129	262/274	142/142	96/96	110/110	176/193	131/137	168/168	145/151	179/185	265/274	170/170	130/138	161/164	255/255	235/235	222/233
112/118	95/101	151/153	117/117	136/136	120/122	96/96	180/184	159/159	117/126	92/92	149/149	95/113	170/174	97/115	102/119	178/182	129/129	269/274	142/142	96/113	110/110	176/185	134/134	168/177	151/151	185/191	265/265	170/170	132/135	164/164	255/255	235/238	222/233
118/118	89/95	148/153	117/126	133/139	117/122	96/102	180/184	168/168	117/126	92/99	149/164	89/113	170/174	91/115	96/119	178/182	125/129	274/274	142/142	96/96	110/110	176/185	131/137	165/168	145/151	185/191	265/273	166/170	127/135	152/161	240/255	235/235	222/233
112/112	89/89	146/151	117/126	133/136	115/120	96/105	180/184	168/171	117/126	92/102	149/164	92/92	170/174	94/94	99/99	178/182	129/129	274/274	142/142	96/96	110/123	176/185	131/134	165/177	145/151	179/191	265/274	170/170	135/135	161/161	240/255	235/235	233/233
112/118	89/95	146/151	117/117	133/136	115/120	96/108	184/184	159/165	117/117	92/105	149/149	92/95	174/174	94/97	99/102	182/182	125/129	262/274	142/142	96/96	110/110	185/193	131/134	168/177	151/151	185/185	265/277	170/170	138/138	161/164	255/255	235/238	222/233
112/112	89/95	151/151	117/117	136/139	120/120	96/108	180/184	165/168	117/126	92/105	149/149	89/92	170/174	91/94	96/99	178/182	125/129	274/274	142/142	96/96	110/110	176/185	134/137	165/177	145/145	179/191	265/277	170/170	130/135	161/161	240/255	238/238	222/233
112/118	89/95	151/153	117/117	136/139	120/122	96/108	180/180	159/171	117/117	92/105	149/149	89/95	170/170	91/97	96/102	178/178	129/129	262/262	130/142	96/113	110/110	185/193	134/137	168/177	145/151	185/191	265/277	170/170	130/138	161/164	255/255	235/235	222/233
112/112	89/89	146/151	115/117	133/133	115/120	105/108	180/180	162/171	117/117	102/105	146/149	92/92	170/170	94/94	99/99	178/178	125/129	274/280	133/142	96/113	110/110	185/189	131/131	168/177	145/145	179/185	274/277	170/170	127/130	161/161	255/255	230/238	233/233
112/118	89/89	151/153	117/117	133/136	120/122	96/105	180/180	159/171	117/126	92/102	149/149	89/89	170/170	91/91	96/96	178/178	129/129	262/274	142/142	96/113	110/110	176/185	131/134	168/177	145/145	179/191	265/274	170/170	130/132	161/161	255/255	230/238	233/233
118/118	95/101	153/153	109/126	133/136	122/122	96/96	184/184	159/159	126/126	92/92	146/164	92/95	174/174	94/97	99/102	182/182	129/129	263/274	142/142	108/113	110/123	176/176	131/134	162/165	145/151	179/185	265/265	170/170	132/138	152/161	240/255	230/235	233/233
112/112	89/95	148/151	117/126	133/139	117/120	96/96	184/184	159/168	117/126	92/92	149/164	92/113	174/174	94/115	99/119	182/182	129/129	262/274	142/142	96/108	110/123	176/193	131/137	165/168	145/151	191/191	265/265	166/170	127/138	164/164	240/255	230/235	222/233
118/118	95/101	153/153	109/117	133/139	122/122	96/96	180/184	159/159	126/126	92/92	146/149	95/113	170/174	97/115	102/119	178/182	129/129	263/274	130/142	113/113	110/110	176/176	131/137	162/165	151/151	185/191	265/265	166/170	132/138	152/161	240/255	230/235	233/233
112/118	89/95	151/153	109/117	133/139	120/122	96/96	180/184	159/159	126/126	92/92	146/149	92/95	170/174	94/97	99/102	178/182	129/129	263/274	130/142	113/113	110/123	172/176	131/137	162/168	151/151	179/194	265/265	166/170	132/138	161/161	255/255	230/235	233/233
112/118	95/101	148/148	117/126	136/139	117/117	96/105	180/184	168/171	126/126	92/102	149/164	89/92	170/174	91/94	96/99	178/182	129/129	262/274	142/142	96/108	110/110	176/176	134/137	165/165	145/151	191/191	265/274	170/170	130/135	152/161	240/240	235/235	222/233
112/118	89/89	151/153	117/126	139/139	120/122	96/96	184/184	159/159	126/126	92/92	149/164	89/92	174/174	91/94	96/99	182/182	129/129	274/274	142/142	96/108	110/123	176/176	137/137	165/168	151/151	179/194	265/265	170/170	132/138	161/164	240/255	230/235	233/233
112/118	89/95	151/151	117/117	136/139	120/120	96/96	180/184	159/159	126/126	92/92	149/149	89/95	170/174	91/97	96/102	178/182	129/129	262/274	142/142	96/113	110/110	176/176	134/137	168/168	151/151	185/191	265/265	166/166	127/138	152/164	240/255	235/235	233/233
112/118	101/101	151/153	117/117	133/133	120/122	96/96	180/180	168/168	126/126	92/92	149/149	92/95	170/170	94/97	99/102	178/178	129/129	262/274	142/142	96/113	110/123	176/176	131/131	162/165	151/151	191/191	265/265	170/170	135/138	152/161	240/255	235/235	233/233
118/118	89/95	151/153	117/117	133/139	120/122	96/105	180/184	159/171	126/126	92/102	149/149	92/95	170/174	94/97	99/102	178/182	129/129	262/262	142/142	96/96	110/123	176/176	131/137	165/168	145/151	179/185	265/274	170/170	138/138	164/164	240/255	235/235	233/233
112/118	89/101	146/153	117/126	133/139	115/122	96/96	184/184	156/168	123/126	92/92	149/164	92/92	174/174	94/94	99/99	182/182	125/129	262/280	136/142	108/108	110/123	176/185	131/137	162/165	145/151	179/185	265/265	170/170	135/138	161/161	240/255	235/238	233/233
112/118	95/95	148/153	109/117	136/139	117/122	96/96	180/184	159/168	117/126	92/92	146/149	92/113	170/174	94/115	99/119	178/182	129/129	274/274	130/142	96/108	110/123	176/193	134/137	168/168	151/151	185/191	265/265	170/170	130/132	152/161	255/255	235/235	233/233
112/118	95/95	151/153	117/117	136/136	120/122	96/96	180/180	159/168	126/126	92/92	149/149	95/95	170/170	97/97	102/102	178/178	129/129	262/262	142/142	96/113	110/110	176/176	134/134	168/177	151/151	185/191	265/265	166/166	132/138	152/164	255/255	235/235	233/233
112/118	89/101	145/146	112/117	133/136	114/115	96/96	170/184	156/159	117/117	92/92	149/149	92/92	160/174	94/94	99/99	168/182	125/129	274/280	133/136	96/108	110/110	185/189	131/134	162/168	145/151	184/185	265/265	170/170	130/135	161/161	255/255	233/238	227/233
112/118	89/101	145/146	117/117	133/136	114/115	85/96	180/184	156/159	123/126	79/92	149/149	92/92	170/174	94/94	99/99	178/182	125/125	274/280	136/142	96/113	110/110	176/185	131/134	162/162	145/151	184/185	252/265	170/170	135/138	161/161	255/255	230/238	227/233
112/118	95/95	151/153	117/126	136/139	120/122	96/105	180/184	159/171	117/117	92/102	149/164	92/113	170/174	94/115	99/119	178/182	129/129	262/274	142/142	96/108	110/123	185/193	134/137	165/168	151/151	185/191	265/274	170/170	130/132	152/152	240/255	235/235	233/233
112/118	89/95	151/151	117/126	136/139	120/120	96/105	184/184	159/171	126/126	92/102	149/164	89/92	174/174	91/94	96/99	182/182	129/129	274/274	142/142	96/96	110/123	176/176	134/137	165/165	151/151	179/185	265/274	170/170	132/138	152/152	240/240	235/235	233/233
118/118	89/101	151/153	117/126	136/139	120/122	96/96	184/184	159/168	126/126	92/92	149/164	113/113	174/174	115/115	119/119	182/182	129/129	262/262	133/142	113/113	110/110	176/176	134/137	165/165	145/151	184/194	265/265	166/170	127/138	161/161	240/255	233/235	227/233
112/112	95/101	151/153	117/117	133/136	120/122	96/96	126/180	159/168	117/126	92/92	149/155	89/92	116/170	91/94	96/99	124/178	129/129	262/274	136/142	96/108	110/123	176/185	131/134	165/177	151/151	185/191	265/265	170/170	132/138	152/161	240/255	230/230	216/233
112/118	89/95	151/153	117/117	136/139	120/122	96/96	180/180	159/159	117/117	92/92	149/149	89/92	170/170	91/94	96/99	178/178	129/129	262/262	142/142	96/96	110/110	176/193	134/137	165/168	151/151	191/194	265/265	166/170	130/138	152/164	240/255	235/235	233/233
112/118	95/101	148/153	120/126	133/139	117/122	99/105	184/184	162/171	117/126	96/102	152/164	92/92	174/174	94/94	99/99	182/182	125/129	262/274	142/142	108/113	110/123	176/180	131/137	165/165	145/151	191/191	268/274	170/170	127/130	164/164	240/255	235/235	222/233
112/118	95/101	151/153	117/126	139/139	120/122	96/105	180/184	159/171	126/126	92/102	149/164	92/95	170/174	94/97	99/102	178/182	129/129	262/274	142/142	96/108	110/123	176/176	137/137	165/168	151/151	185/191	265/274	170/170	130/138	152/164	240/255	235/235	233/233
112/118	95/101	151/151	117/117	133/141	120/120	96/105	180/184	159/171	117/126	92/102	149/149	92/95	170/174	94/97	99/102	178/182	129/129	262/274	142/142	96/108	110/110	176/193	131/134	165/168	145/151	185/191	265/274	170/170	130/138	152/152	240/255	235/235	233/233
112/112	101/101	151/151	117/126	139/139	120/120	96/96	180/184	159/168	126/126	92/92	149/164	92/95	170/174	94/97	99/102	178/182	129/129	262/262	142/142	96/108	110/123	176/176	137/137	168/168	145/145	185/191	265/265	170/170	138/138	152/161	255/255	230/235	233/233

164	165	166	167	168	169	170	171	172	173	174	175	176	177	178	179	180	181	182	183	184	185	186	187	188	189	190	191	192	193	194	195	196	197
**	*	*		*	*			*	*	*	*	*	*	*	*		*	*	*	*	*	*	*	*	*	*	*		*	*	**	*	*
TSGR901	TSGR904	TSGR905	TSGR906	TSR8EM15	TSR8EM24	TSR8EM28	TSRB07	TSRB09	TSRB11	TSRB29	TSRB38	TSRB39	TSRF101	TSRF105	TSRF116	TSRF120	TSRF124	TSRF136	TSRF138	TSRF144	TSRF157	TSRF160	TSRF161	TSRF162	TSRF164	TSRF167	TSRF169	TSRF172	TSRF184	TSRF187	TSRF191	TSRF192	TSRF194
0	1	0	0	1	0	9	1	0	0	0	3	2	0	0	0	1	2	1	0	0	0	0	1	0	0	0	0	1	1	0	1	0	0
151/153	129/131	242/242	103/103	278/281	146/156	138/138	170/170	130/136	225/228	162/165	206/211	364/370	152/161	259/265	174/174	196/196	436/442	203/206	178/180	220/234	288/290	211/211	246/249	182/186	225/230	171/171	302/304	264/273	253/255	230/234	178/183	175/175	172/178
153/153	129/129	241/242	103/103	278/281	146/146	nd.	170/170	130/130	228/228	162/162	206/206	364/370	152/161	259/259	174/174	195/195	442/442	206/206	180/180	217/220	290/290	211/211	246/246	182/182	230/230	171/171	299/304	259/264	253/255	230/230	178/178	175/178	172/181
151/153	129/131	241/242	87/103	278/281	146/156	138/141	170/170	130/136	225/234	162/162	206/206	364/367	152/161	259/259	174/174	195/196	442/442	203/214	178/180	220/220	288/290	211/211	249/254	182/182	225/230	171/171	299/304	264/273	253/255	230/234	178/183	175/175	172/181
153/153	129/131	242/242	103/103	281/281	146/146	138/138	161/164	130/130	225/228	162/162	206/211	379/379	152/152	259/265	174/174	195/195	442/442	203/203	178/180	220/220	288/290	211/211	246/246	182/182	230/230	171/171	299/302	259/259	253/253	230/230	178/178	175/175	178/178
151/151	131/131	241/241	100/103	278/278	156/156	138/141	164/170	136/136	228/234	162/165	211/217	364/396	158/161	265/271	171/171	195/195	436/438	206/214	178/178	220/223	288/288	211/211	249/249	186/186	225/225	171/171	299/304	261/273	255/255	234/234	183/183	175/175	172/172
151/153	131/131	242/243	100/100	278/278	146/156	138/141	161/161	130/136	225/228	162/165	211/211	370/376	154/158	265/265	174/174	194/195	438/438	197/206	178/178	214/217	288/288	211/211	246/246	186/186	224/225	171/174	304/307	259/273	253/255	230/234	178/183	178/180	178/178
151/153	129/131	241/242	103/103	278/281	146/156	138/141	164/170	130/136	234/234	162/165	206/211	364/367	154/161	259/265	174/174	195/196	436/442	206/214	178/178	220/234	288/290	211/211	249/254	182/186	225/230	171/171	304/304	261/273	255/255	230/234	178/183	171/175	172/172
151/153	129/131	241/242	100/100	278/278	146/156	138/138	170/170	130/133	228/228	162/162	211/211	364/396	154/161	265/265	171/174	195/196	436/436	206/214	178/178	220/232	288/290	202/211	246/254	186/186	225/225	171/171	304/307	264/273	253/255	234/234	178/183	175/175	172/178
144/153	131/131	241/241	100/100	278/278	146/156	138/141	153/164	136/136	234/234	165/177	206/211	364/396	161/161	259/265	171/171	195/196	436/436	206/214	178/178	220/234	288/288	211/211	249/249	186/186	225/225	171/171	304/304	259/261	253/255	234/234	183/183	175/175	172/172
144/153	131/131	241/241	100/100	278/278	146/156	138/141	153/164	136/136	234/234	165/177	206/211	364/396	161/161	259/265	171/171	195/196	436/436	206/214	178/178	220/234	288/288	211/211	249/249	186/186	225/225	171/171	304/304	259/261	253/255	234/234	183/183	175/175	172/172
144/153	131/131	241/241	100/100	278/278	146/156	138/141	153/164	136/136	234/234	165/177	206/211	364/396	161/161	259/265	171/171	195/196	436/436	206/214	178/178	220/234	288/288	211/211	249/249	186/186	225/225	171/171	304/304	259/261	253/255	234/234	183/183	175/175	172/172
144/153	131/131	241/241	100/100	278/278	146/156	138/141	153/164	136/136	234/234	165/177	206/211	364/396	161/161	259/265	171/171	195/196	436/436	206/214	178/178	220/234	288/288	211/211	249/249	186/186	225/225	171/171	304/304	259/261	253/255	234/234	183/183	175/175	172/172
151/151	131/131	241/241	100/100	278/278	156/156	141/141	164/170	136/136	228/234	165/165	211/211	364/364	161/161	265/265	171/171	196/196	436/438	203/206	178/178	229/234	288/288	211/211	246/249	186/186	225/225	171/171	304/304	256/261	255/255	234/234	183/183	175/175	172/172
144/151	129/131	241/242	100/103	278/278	156/156	141/141	153/170	136/136	234/234	165/165	206/211	364/396	161/161	259/265	171/174	195/195	436/442	206/214	178/178	217/220	288/288	211/211	246/249	182/186	225/225	171/171	304/304	259/261	255/255	234/234	183/183	175/175	172/172
144/153	131/131	242/242	100/100	278/278	146/156	138/138	164/164	130/133	225/234	162/165	206/208	364/381	154/161	259/262	171/174	195/196	436/442	206/206	178/180	223/226	288/290	211/211	246/246	182/186	218/225	171/171	299/304	261/264	253/255	230/230	178/183	175/175	172/178
144/151	129/131	241/242	100/103	278/278	156/156	141/141	153/164	136/136	228/228	165/165	211/214	364/396	161/161	265/268	171/174	195/196	436/436	206/206	178/178	220/223	288/288	211/211	249/249	186/186	225/225	171/171	304/304	273/273	255/255	234/234	183/183	175/175	172/172
144/151	129/131	241/242	100/103	278/278	156/156	141/141	153/164	136/136	228/228	165/165	211/214	364/396	161/161	265/268	171/174	195/196	436/436	206/206	178/178	220/223	288/288	211/211	249/249	186/186	225/225	171/171	304/304	273/273	255/255	234/234	183/183	175/175	172/172
153/153	129/129	242/242	103/103	278/281	146/146	nd.	164/164	130/130	225/228	162/165	206/211	379/381	152/152	259/265	174/174	195/195	442/442	203/203	180/180	217/223	290/290	211/211	246/246	182/182	230/230	171/171	299/299	264/264	253/253	230/230	178/178	175/175	178/178
151/151	131/131	242/242	100/103	278/278	156/156	138/141	153/170	133/133	228/234	162/162	208/211	364/364	154/161	262/265	171/174	196/196	436/436	206/206	178/178	226/234	288/288	211/211	246/249	186/186	225/225	171/171	299/304	259/273	255/255	230/234	183/183	168/175	172/172
151/151	131/131	241/242	100/103	278/278	146/159	138/138	164/164	133/136	228/228	162/165	208/211	364/370	154/161	262/265	171/174	195/196	436/438	206/206	178/178	217/226	288/288	202/211	246/246	186/186	225/225	171/171	304/304	267/273	255/255	230/234	178/183	171/178	172/172
151/153	131/131	241/242	100/100	278/281	146/156	138/138	170/170	130/136	234/234	162/162	206/211	381/396	158/161	259/265	171/174	195/195	436/442	206/214	178/180	217/220	288/290	211/211	249/254	182/186	225/225	171/171	299/304	259/261	253/255	234/234	178/183	175/175	172/178
151/151	129/131	241/242	100/100	278/278	156/156	141/141	153/170	136/136	228/234	165/165	211/214	364/364	161/161	265/268	171/174	196/196	436/436	203/214	178/178	217/234	288/288	211/211	249/249	186/186	225/225	171/171	304/304	261/261	255/255	234/234	183/183	168/175	172/172
144/151	129/131	242/242	100/103	278/278	146/156	138/138	164/170	133/136	228/228	162/162	208/211	364/364	154/161	262/265	171/174	195/196	436/436	206/206	178/178	220/226	288/288	202/211	246/249	186/186	225/225	171/171	304/304	259/267	255/255	230/234	178/183	175/175	172/172
153/153	129/131	241/242	100/103	278/281	146/146	138/138	170/170	130/136	228/234	162/165	206/211	367/370	154/161	259/265	174/174	195/195	436/442	203/206	178/180	220/220	288/290	211/211	246/249	182/186	225/230	171/171	299/304	261/264	253/255	230/234	178/178	175/175	172/178
153/153	129/131	241/242	100/103	278/281	146/146	138/138	170/170	130/136	228/234	162/165	206/211	367/370	154/161	259/265	174/174	195/195	436/442	203/206	178/180	220/220	288/290	211/211	246/249	182/186	225/230	171/171	299/304	261/264	253/255	230/234	178/178	175/175	172/178
153/153	129/131	241/242	100/103	278/281	146/146	138/138	170/170	130/136	228/234	162/165	206/211	367/370	154/161	259/265	174/174	195/195	436/442	203/206	178/180	220/220	288/290	211/211	246/249	182/186	225/230	171/171	299/304	261/264	253/255	230/234	178/178	175/175	172/178
151/151	131/131	242/242	100/100	278/278	146/159	138/138	164/164	122/133	228/234	165/168	208/211	364/376	161/161	262/265	171/171	194/196	436/438	206/206	178/178	229/234	288/288	202/211	246/246	186/186	225/225	171/171	304/304	247/261	255/255	230/230	175/183	168/175	172/172
151/153	129/131	242/242	103/103	278/281	146/156	141/141	170/170	130/130	228/234	162/165	206/211	367/370	152/161	259/265	174/174	195/195	436/442	206/214	178/180	220/220	288/290	211/211	246/254	182/186	225/230	171/171	302/304	261/264	253/255	230/234	178/183	175/175	172/178
151/153	129/131	241/242	103/103	278/278	146/156	138/141	170/170	122/130	228/234	165/168	211/211	364/367	154/161	265/265	171/174	195/195	436/436	206/214	178/178	220/223	288/290	211/211	246/249	186/189	225/225	171/171	299/304	261/270	255/259	230/234	175/183	168/175	172/172
151/151	131/131	241/242	100/103	278/278	146/156	138/138	164/164	133/136	228/234	165/168	211/211	364/376	154/161	265/265	171/174	194/196	436/436	194/214	178/178	229/234	288/288	211/211	246/254	186/189	225/230	171/171	299/304	261/270	255/259	230/234	178/183	171/175	172/172
151/151	129/129	241/241	100/100	278/278	156/156	141/141	153/153	133/136	234/234	165/165	211/211	364/396	161/161	265/265	174/174	195/196	436/436	206/214	178/180	217/220	288/288	211/211	246/246	186/186	225/225	171/171	304/304	261/273	255/255	234/234	178/183	168/168	172/172
153/157	129/129	242/242	103/103	278/281	146/146	138/138	156/161	130/130	225/228	162/162	206/206	370/381	152/152	259/259	174/174	195/195	442/442	203/203	180/180	217/220	288/290	211/211	246/249	182/182	230/230	171/171	299/299	259/264	253/253	230/230	178/178	175/175	178/184
151/153	129/131	241/242	103/103	278/278	146/156	141/141	161/164	130/136	225/228	162/162	206/206	364/370	152/158	259/259	174/174	195/195	442/442	206/206	178/180	220/223	288/290	211/211	246/246	182/182	230/230	171/171	299/304	259/264	253/255	230/230	178/183	175/175	172/181
151/153	129/131	242/242	103/103	278/281	146/156	141/141	164/170	130/136	228/234	162/162	206/211	364/370	154/161	259/265	174/174	196/196	436/442	208/214	178/178	220/234	288/290	211/211	246/246	182/186	218/225	171/171	299/304	273/273	255/255	230/234	178/183	175/175	172/172
153/153	129/129	241/242	103/103	278/281	146/146	138/138	170/170	130/136	228/234	162/165	211/211	364/379	152/161	265/265	174/174	195/196	436/442	208/214	178/180	220/234	288/290	211/211	246/254	182/186	218/225	171/171	299/304	261/264	253/255	230/234	178/178	175/178	172/181
151/153	129/131	241/242	100/100	278/281	146/156	138/138	164/164	130/133	228/234	162/165	206/211	364/367	152/161	259/265	171/174	195/196	436/442	203/203	178/180	223/226	288/290	211/211	246/254	182/186	225/230	171/171	304/304	261/264	253/255	230/230	178/183	168/175	172/172
151/153	129/131	241/242	100/100	278/281	146/156	138/138	164/164	130/133	228/234	162/165	206/211	364/367	152/161	259/265	171/174	195/196	436/442	203/203	178/180	223/226	288/290	211/211	246/254	182/186	225/230	171/171	304/304	261/264	253/255	230/230	178/183	168/175	172/172
151/153	129/131	241/242	100/100	278/281	146/156	138/138	164/164	130/133	228/234	162/165	206/211	364/367	152/161	259/265	171/174	195/196	436/442	203/203	178/180	223/226	288/290	211/211	246/254	182/186	225/230	171/171	304/304	261/264	253/255	230/230	178/183	168/175	172/172
151/153	129/131	241/242	100/100	278/281	146/156	138/138	164/164	130/133	228/234	162/165	206/211	364/367	152/161	259/265	171/174	195/196	436/442	203/203	178/180	223/226	288/290	211/211	246/254	182/186	225/230	171/171	304/304	261/264	253/255	230/230	178/183	168/175	172/172
151/153	129/131	241/242	100/100	278/281	146/156	138/138	164/164	130/133	228/234	162/165	206/211	364/367	152/161	259/265	171/174	195/196	436/442	203/203	178/180	223/226	288/290	211/211	246/254	182/186	225/230	171/171	304/304	261/264	253/255	230/230	178/183	168/175	172/172
151/151	131/131	242/243	100/108	278/278	146/146	138/138	156/161	130/133	225/228	162/165	206/211	373/376	154/154	259/265	171/171	194/195	436/438	197/203	178/180	214/223	288/288	211/211	246/246	186/186	224/225	171/171	299/304	259/266	255/255	230/230	171/175	168/180	172/172
151/153	129/131	242/242	87/87	278/278	146/146	138/138	161/161	122/130	228/228	162/168	208/211	364/367	154/154	262/265	171/174	195/195	438/442	206/206	178/178	220/223	288/290	211/211	246/246	182/186	225/230	171/171	304/304	247/264	253/255	230/234	175/178	166/169	172/178
151/153	129/131	241/242	100/100	278/281	156/156	141/141	153/153	130/136	228/234	162/165	206/211	364/370	152/161	259/265	174/174	196/196	436/442	203/206	178/178	220/223	288/290	211/211	246/249	182/186	225/230	171/171	302/304	273/273	253/255	230/234	183/183	175/175	172/178
151/153	129/131	241/242	100/100	278/281	156/156	141/141	153/153	130/136	228/234	162/165	206/211	364/370	152/161	259/265	174/174	196/196	436/442	203/206	178/178	220/223	288/290	211/211	246/249	182/186	225/230	171/171	302/304	273/273	253/255	230/234	183/183	175/175	172/178
151/151	129/131	241/242	100/103	278/278	146/146	138/138	164/164	122/130	228/234	162/162	nd.	364/364	161/161	265/265	171/174	196/196	436/436	206/206	178/178	220/223	288/290	211/211	246/254	186/189	225/225	171/171	299/304	247/273	255/259	234/234	175/178	168/171	172/172
151/153	129/131	242/242	103/103	278/281	146/156	138/138	164/164	133/136	228/228	162/168	206/211	364/370	154/161	259/265	171/174	195/195	438/442	194/214	178/178	220/223	288/288	211/211	246/249	182/186	225/230	171/171	299/299	264/270	253/255	230/230	178/183	171/171	172/172
151/153	131/131	242/242	103/103	278/278	146/156	138/141	170/170	122/136	228/234	162/162	211/211	364/367	154/154	265/265	171/174	195/195	436/438	194/206	178/178	220/223	288/288	211/211	246/249	186/186	225/225	171/171	299/304	247/273	255/259	230/234	175/183	171/171	172/172
151/153	129/131	241/242	100/103	278/281	156/156	138/141	164/164	130/133	228/234	162/162	206/208	364/367	154/161	259/262	171/174	195/196	436/442	206/214	178/178	220/226	288/290	202/211	246/249	182/186	225/230	171/171	304/304	267/273	255/255	230/230	183/183	175/175	172/172
153/153	129/131	242/242	103/103	281/281	146/156	138/141	170/170	130/130	225/234	162/162	206/206	367/370	152/154	259/259	174/174	195/195	442/442	203/214	178/180	220/234	290/290	211/211	246/254	182/182	230/230	171/171	302/304	264/273	253/255	230/230	178/183	175/175	172/178
151/153	129/131	242/242	103/103	278/278	156/156	141/141	170/170	130/133	228/234	162/162	206/211	364/367	161/161	259/265	174/174	195/196	436/442	206/214	178/178	220/234	288/290	202/211	249/254	182/186	218/225	171/171	304/304	264/273	255/255	230/234	183/183	175/175	172/181
153/153	129/131	242/242	103/103	278/278	146/146	nd.	164/164	130/136	228/228	162/162	206/211	364/379	152/161	259/265	174/174	195/196	442/442	203/214	178/180	223/234	288/290	211/211	246/246	182/182	225/230	171/171	299/304	259/273	253/255	230/234	178/178	175/175	172/184
151/151	129/129	242/242	103/103	278/281	146/156	138/141	164/164	133/136	228/234	162/165	206/208	364/367	161/161	259/262	174/174	195/196	436/442	206/214	178/178	220/226	288/288	211/211	249/254	182/186	225/230	171/171	304/304	261/273	255/255	230/234	178/183	175/175	172/172
151/151	129/131	242/242	103/103	278/278	156/159	138/141	164/164	130/133	228/228	162/168	206/208	364/379	152/161	259/262	171/174	195/196	436/442	206/208	178/178	217/234	288/290	211/211	246/246	182/186	225/230	171/171	304/304	247/259	253/255	230/230	183/183	168/178	172/178
151/153	129/131	242/242	100/100	278/281	156/162	138/138	164/164	133/136	228/228	165/165	206/211	364/367	161/161	259/265	171/174	195/196	436/442	206/214	178/178	220/229	288/288	211/211	246/254	182/186	225/225	171/171	304/304	273/273	255/255	234/234	178/183	175/175	172/172
153/153	131/131	242/242	103/103	278/281	146/156	141/141	170/170	130/130	228/234	162/165	206/211	364/379	154/161	259/265	174/174	195/196	436/442	203/214	178/178	223/234	290/290	211/211	249/254	182/186	225/230	171/171	299/304	259/261	253/255	230/234	178/183	175/175	172/178
151/153	129/131	242/242	103/103	278/281	146/156	138/138	170/170	130/136	225/228	162/165	206/211	364/370	152/161	259/265	174/174	196/196	nd.	203/206	178/180	220/234	288/290	211/211	246/249	182/186	225/230	171/171	302/304	264/273	253/255	230/234	178/183	175/175	172/178
151/151	131/131	241/242	103/103	278/278	156/156	138/141	164/170	133/136	228/234	162/165	211/211	364/364	161/161	265/265	171/174	195/196	436/436	206/214	178/178	234/234	288/288	211/211	246/249	186/186	225/225	171/171	299/304	261/273	255/255	234/234	183/183	175/175	172/172
151/151	131/131	241/242	103/103	278/278	156/156	138/141	164/170	133/136	228/234	162/165	211/211	nd.	161/161	265/265	171/174	195/196	436/436	206/214	178/178	234/234	288/288	211/211	246/249	186/186	225/225	171/171	299/304	261/273	255/255	234/234	183/183	175/175	172/172
151/151	131/131	241/242	103/103	278/278	156/156	138/141	164/170	133/136	228/234	162/165	211/211	364/364	161/161	265/265	171/174	195/196	436/436	206/214	178/178	234/234	288/288	211/211	246/249	186/186	225/225	171/171	299/304	261/273	255/255	234/234	183/183	175/175	172/172
151/151	131/131	241/242	103/103	278/278	156/156	138/141	164/170	133/136	228/234	162/165	211/211	364/364	161/161	265/265	171/174	195/196	436/436	206/214	178/178	234/234	288/288	211/211	246/249	186/186	225/225	171/171	299/304	261/273	255/255	234/234	183/183	175/175	172/172
151/151	131/131	241/242	103/103	278/278	156/156	138/141	164/170	133/136	228/234	162/165	211/211	364/364	161/161	265/265	171/174	195/196	436/436	206/214	178/178	234/234	288/288	211/211	246/249	186/186	225/225	171/171	299/304	261/273	255/255	234/234	183/183	175/175	172/172
151/151	131/131	241/242	103/103	278/278	156/156	138/141	164/170	133/136	228/234	162/165	211/211	364/364	161/161	265/265	171/174	195/196	436/436	206/214	178/178	234/234	288/288	211/211	246/249	186/186	225/225	171/171	299/304	261/273	255/255	234/234	183/183	175/175	172/172
151/151	131/131	241/242	103/103	278/278	156/156	138/141	164/170	133/136	228/234	162/165	211/211	364/364	161/161	265/265	171/174	195/196	436/436	206/214	178/178	234/234	288/288	211/211	246/249	186/186	225/225	171/171	299/304	261/273	255/255	234/234	183/183	175/175	172/172
151/151	131/131	241/242	103/103	278/278	156/156	138/141	164/170	133/136	228/234	162/165	211/211	364/364	161/161	265/265	171/174	195/196	436/436	206/214	178/178	234/234	288/288	211/211	246/249	186/186	225/225	171/171	299/304	261/273	255/255	234/234	183/183	175/175	172/172
151/151	131/131	241/242	103/103	278/278	156/156	138/141	164/170	133/136	228/234	162/165	211/211	364/364	161/161	265/265	171/174	195/196	436/436	206/214	178/178	234/234	288/288	211/211	246/249	186/186	225/225	171/171	299/304	261/273	255/255	234/234	183/183	175/175	172/172
151/151	131/131	241/242	103/103	278/278	156/156	138/141	164/170	133/136	228/234	162/165	211/211	364/364	161/161	265/265	171/174	195/196	436/436	206/214	178/178	234/234	288/288	211/211	246/249	186/186	225/225	171/171	299/304	261/273	255/255	234/234	183/183	175/175	172/172
151/151	131/131	241/242	103/103	278/278	156/156	138/141	164/170	133/136	228/234	162/165	211/211	364/364	161/161	265/265	171/174	195/196	436/436	206/214	178/178	234/234	288/288	211/211	246/249	186/186	225/225	171/171	299/304	261/273	255/255	234/234	183/183	175/175	172/172
151/151	131/131	241/242	103/103	278/278	156/156	138/141	164/170	133/136	228/234	162/165	211/211	364/364	161/161	265/265	171/174	195/196	436/436	206/214	178/178	234/234	288/288	211/211	246/249	186/186	225/225	171/171	299/304	261/273	255/255	234/234	183/183	175/175	172/172
151/151	131/131	241/242	103/103	278/278	156/156	138/141	164/170	133/136	228/234	162/165	211/211	364/364	161/161	265/265	171/174	195/196	436/436	206/214	178/178	234/234	288/288	211/211	246/249	186/186	225/225	171/171	299/304	261/273	255/255	234/234	183/183	175/175	172/172
151/151	131/131	241/242	103/103	278/278	156/156	138/141	164/170	133/136	228/234	162/165	211/211	364/364	161/161	265/265	171/174	195/196	436/436	206/214	178/178	234/234	288/288	211/211	246/249	186/186	225/225	171/171	299/304	261/273	255/255	234/234	183/183	175/175	172/172
151/151	131/131	241/242	103/103	278/278	156/156	138/141	164/170	133/136	228/234	162/165	211/211	364/364	161/161	265/265	171/174	195/196	436/436	206/214	178/178	234/234	288/288	211/211	246/249	186/186	225/225	171/171	299/304	261/273	255/255	234/234	183/183	175/175	172/172
151/151	131/131	241/242	103/103	278/278	156/156	138/141	164/170	133/136	228/234	162/165	211/211	364/364	161/161	265/265	171/174	195/196	436/436	206/214	178/178	234/234	288/288	211/211	246/249	186/186	225/225	171/171	299/304	261/273	255/255	234/234	183/183	175/175	172/172
151/151	131/131	241/242	103/103	278/278	156/156	138/141	164/170	133/136	228/234	162/165	211/211	364/364	161/161	265/265	171/174	195/196	436/436	206/214	178/178	234/234	288/288	211/211	246/249	186/186	225/225	171/171	299/304	261/273	255/255	234/234	183/183	175/175	172/172
151/153	129/131	241/242	103/103	278/278	146/156	138/141	170/170	122/130	228/234	165/168	211/211	364/367	154/161	265/265	171/174	195/195	436/436	206/214	178/178	220/223	288/290	211/211	246/249	186/189	225/225	171/171	299/304	261/270	255/259	230/234	175/183	168/175	172/172
151/153	129/131	241/242	103/103	278/281	146/156	138/141	170/170	130/136	228/234	162/165	206/211	364/367	154/161	259/265	171/174	195/196	436/442	206/206	178/180	220/234	288/290	211/211	246/249/254	182/186	225/230	171/171	299/304	259/261/273	253/255	230/234	178/183	171/175	172/178
144/153	131/131	241/242	100/103	278/278	156/156	141/141	164/170	136/136	228/228	165/165	211/217	364/396	161/161	265/271	171/171	195/195	436/436	206/206	178/178	223/232	288/288	211/211	246/249	186/186	225/225	171/171	304/304	273/273	255/255	234/234	183/183	168/175	172/172
144/151	131/131	241/242	100/100	278/278	156/156	138/141	153/170	122/133	228/234	162/162	208/211	364/396	154/161	262/265	171/171	195/196	436/436	206/206	178/178	220/226	288/288	211/211	246/246	186/186	225/225	171/171	304/304	259/261	255/255	230/234	183/183	168/175	172/172
144/151	131/131	241/242	100/100	278/278	156/156	138/141	153/170	122/133	228/234	162/162	208/211	364/396	154/161	262/265	171/171	195/196	436/436	206/206	178/178	220/226	288/288	211/211	246/246	186/186	225/225	171/171	304/304	259/259	255/255	230/234	183/183	168/175	172/172
151/153	131/131	241/242	100/103	278/278	146/156	138/141	158/158	130/136	225/228	162/165	211/211	364/376	158/161	265/265	171/174	195/196	436/442	203/206	178/178	220/234	288/290	211/211	246/246	182/186	225/230	171/171	299/304	256/265	253/255	230/234	178/183	168/178	172/178
151/151	131/131	242/242	100/100	278/278	146/156	138/138	164/167	122/136	228/234	165/168	211/211	364/376	161/161	265/265	171/171	194/195	436/438	203/206	178/178	226/229	288/288	211/211	246/246	186/186	225/225	171/171	299/304	261/270	255/255	230/234	175/183	168/168	172/172
151/153	131/131	241/242	103/103	278/281	146/156	141/141	156/156	122/133	228/228	162/162	206/211	364/370	154/161	259/265	171/174	195/196	436/442	206/206	178/178	217/234	288/288	211/211	246/249	182/186	225/230	171/171	299/304	264/273	253/255	230/234	178/183	168/171	172/178
151/153	129/131	241/242	103/103	278/281	146/156	138/141	164/170	130/136	234/234	162/165	206/211	364/367	154/161	259/265	174/174	195/196	436/442	206/214	178/178	220/234	288/290	211/211	249/254	182/186	225/230	171/171	304/304	261/273	255/255	230/234	178/183	171/175	172/172
151/151	131/131	242/242	100/103	278/278	146/156	138/138	164/164	133/136	225/228	162/162	206/211	364/370	158/161	259/265	171/171	195/196	436/438	206/206	178/178	217/226	288/288	202/211	246/246	186/186	225/225	171/171	304/304	259/267	255/255	230/234	178/183	175/175	172/172
151/153	129/131	241/242	103/103	278/281	146/156	138/141	164/170	130/136	234/234	162/165	206/211	364/367	154/161	259/265	174/174	195/196	436/442	206/214	178/178	220/234	288/290	211/211	249/254	182/186	225/230	171/171	304/304	261/273	255/255	230/234	178/183	171/175	172/172
151/153	129/131	241/242	103/103	278/281	146/156	138/141	164/170	130/136	234/234	162/165	206/211	nd.	154/161	259/265	174/174	195/196	436/442	206/214	178/178	220/234	288/290	211/211	249/254	182/186	225/230	171/171	304/304	261/273	255/255	230/234	178/183	171/175	172/172
138/151	131/131	241/243	100/100	278/281	146/146	138/138	164/164	130/133	225/228	162/165	197/211	364/376	154/158	251/265	171/174	194/195	436/438	197/206	178/180	214/223	288/293	211/211	246/246	177/186	224/225	171/174	304/304	259/266	255/255	230/234	171/178	178/180	172/172
151/153	131/131	242/243	100/100	278/281	156/156	141/141	153/153	130/136	225/228	162/165	197/211	364/376	158/161	251/265	174/174	194/196	436/442	197/206	178/180	214/217	288/290	211/211	246/246	177/182	225/230	171/174	296/304	261/266	255/255	230/230	178/183	175/180	172/172
138/151	131/131	241/243	100/100	278/281	146/156	138/141	164/164	133/133	228/228	165/165	197/211	364/376	158/161	251/265	171/171	194/196	436/436	197/206	178/180	214/229	288/293	211/211	246/246	177/186	224/225	171/174	296/307	266/273	255/255	230/234	171/183	175/180	172/172
153/153	129/129	242/242	103/103	278/281	146/146	138/138	164/164	122/130	225/225	162/162	208/211	370/379	152/154	262/265	174/174	195/195	442/442	203/206	180/180	220/220	290/290	211/211	246/246	182/182	218/230	171/171	299/307	259/264	253/253	230/230	178/178	175/184	178/178
138/138	131/131	243/243	100/108	278/281	146/146	138/138	161/161	133/136	228/228	146/165	197/211	376/376	158/158	251/265	171/171	194/196	436/436	194/197	178/180	214/217	288/293	211/211	246/246	177/181	224/230	174/174	296/296	251/266	253/255	230/230	171/178	163/180	172/172
138/153	129/131	241/243	103/108	281/281	146/156	138/141	164/164	133/136	228/228	162/165	197/206	364/376	158/164	251/259	171/174	194/196	436/442	197/206	178/180	214/234	288/293	211/211	246/254	177/182	224/225	171/174	296/304	266/273	255/255	230/234	171/183	175/180	172/172
151/151	129/131	242/242	100/103	278/281	146/156	138/141	164/164	133/136	228/234	165/168	206/211	364/367	154/161	259/265	171/174	195/195	438/442	206/214	178/178	220/223	288/288	211/211	246/254	182/186	225/225	171/171	299/304	261/270	255/259	230/234	175/183	168/175	172/172
151/153	129/131	241/242	100/103	278/278	156/162	141/141	164/170	136/136	228/228	165/165	211/214	364/396	161/161	265/268	174/174	195/196	436/436	206/206	178/178	220/220	288/288	211/211	246/246	186/186	225/225	171/171	304/304	273/273	255/255	234/234	178/183	175/175	172/172
151/153	131/131	241/242	100/103	278/278	146/156	138/141	164/164	130/136	225/228	162/165	206/211	364/370	154/158	259/265	171/174	195/195	438/442	206/206	178/178	217/223	288/290	211/211	246/246	182/186	225/230	171/171	304/307	259/273	253/255	230/234	178/183	175/178	172/178
151/151	131/131	241/242	100/103	278/278	146/156	138/138	164/164	133/136	228/234	165/168	211/211	364/376	154/161	265/265	171/174	194/196	436/436	194/214	178/178	229/234	288/288	211/211	246/254	186/189	225/230	171/171	299/304	261/270	255/259	230/234	178/183	171/175	172/172
151/153	129/131	242/242	103/103	278/281	146/156	138/138	164/164	130/136	228/228	162/162	206/211	364/379	152/161	259/265	174/174	195/195	436/442	203/206	178/178	220/234	288/290	211/211	246/246	182/186	225/230	171/171	299/299	259/273	253/255	230/234	178/183	175/175	172/178
151/153	129/131	241/242	103/103	278/281	146/156	138/141	170/170	130/136	228/228	162/162	206/211	364/367	154/161	259/265	174/174	195/196	436/442	206/206	178/180	220/234	288/290	211/211	246/249	182/186	225/230	171/171	299/304	259/273	253/255	230/234	178/183	171/175	172/178
151/153	129/131	241/242	103/103	278/281	146/156	138/141	170/170	130/136	228/228	162/162	206/211	364/367	154/161	259/265	174/174	195/196	436/442	206/206	178/180	220/234	288/290	211/211	246/249	182/186	225/230	171/171	299/304	259/273	253/255	230/234	178/183	171/175	172/178
151/153	129/131	241/242	103/103	278/281	146/156	138/141	170/170	130/136	228/228	162/162	206/211	364/367	154/161	259/265	174/174	195/196	436/442	206/206	178/180	220/234	288/290	211/211	246/249	182/186	225/230	171/171	299/304	259/273	253/255	230/234	178/183	171/175	172/178
151/153	129/131	241/242	103/103	278/281	146/156	138/141	170/170	130/136	228/228	162/162	206/211	364/367	154/161	259/265	174/174	195/196	436/442	206/206	178/180	220/234	288/290	211/211	246/249	182/186	225/230	171/171	299/304	259/273	253/255	230/234	178/183	171/175	172/178
151/151	131/131	241/241	103/103	278/278	156/156	141/141	164/164	130/133	228/234	165/165	211/211	364/370	158/161	265/265	171/174	195/195	436/438	206/206	178/178	217/234	288/290	211/211	246/249	186/186	225/225	171/171	299/304	261/273	255/255	234/234	183/183	175/175	172/172
144/153	129/131	241/242	100/103	278/278	146/156	138/141	170/170	122/136	228/228	162/162	211/211	364/367	154/161	265/265	171/174	195/195	436/442	203/206	178/178	220/223	288/288	202/211	246/249	182/186	225/225	171/171	299/304	256/264	255/255	234/234	178/183	175/175	172/172
153/153	129/129	242/242	103/103	278/281	146/146	138/138	170/170	122/130	225/234	162/162	206/206	379/385	152/154	259/259	174/174	195/195	442/442	203/214	178/180	220/223	288/288	211/211	246/249	182/182	218/230	171/171	299/304	259/273	253/255	230/230	178/178	175/175	172/181

151/153	129/131	241/242	103/103	278/281	156/156	138/141	164/164	122/136	228/234	162/165	206/211	364/367	154/158	259/265	171/174	195/195	436/442	203/206	178/178	220/223	288/290	211/211	249/254	182/186	225/225	171/171	299/299	259/261	253/255	234/234	183/183	175/175	172/178
151/153	131/131	241/241	103/103	278/281	146/156	138/138	170/170	133/136	225/234	162/162	206/211	364/364	152/161	259/265	171/174	196/196	436/442	203/206	178/178	220/234	288/288	211/211	246/254	182/186	225/230	171/171	299/299	261/264	253/255	230/234	183/183	175/175	172/181
151/153	129/131	241/241	100/103	278/278	156/156	141/141	153/164	133/136	228/234	165/165	211/211	364/396	161/161	265/265	171/174	195/195	436/436	206/214	178/178	220/232	288/288	211/211	246/249	186/186	225/225	171/171	304/304	261/273	255/255	234/234	183/183	168/175	172/172
151/153	129/131	241/241	100/103	278/278	156/156	141/141	153/164	133/136	228/234	165/165	211/211	364/396	161/161	265/265	171/174	195/195	436/436	206/214	178/178	220/232	288/288	211/211	246/249	186/186	225/225	171/171	304/304	261/273	255/255	234/234	183/183	168/175	172/172
151/153	129/131	241/241	100/103	278/278	156/156	141/141	153/164	133/136	228/234	165/165	211/211	364/396	161/161	265/265	171/174	195/195	436/436	206/214	178/178	220/232	288/288	211/211	246/249	186/186	225/225	171/171	304/304	261/273	255/255	234/234	183/183	168/175	172/172
151/153	129/131	241/241	100/103	278/278	156/156	141/141	153/164	133/136	228/234	165/165	211/211	364/396	161/161	265/265	171/174	195/195	436/436	206/214	178/178	220/232	288/288	211/211	246/249	186/186	225/225	171/171	304/304	261/273	255/255	234/234	183/183	168/175	172/172
151/153	131/131	241/242	100/100	278/278	146/156	138/141	164/164	133/136	234/234	165/165	211/214	364/364	161/161	265/268	171/171	196/196	436/436	203/203	178/180	217/223	288/288	211/211	249/249	186/186	225/225	171/171	304/304	261/261	255/255	234/234	178/183	168/175	172/172
153/153	129/129	242/242	103/103	281/281	146/146	138/138	161/161	130/130	228/228	162/162	206/206	370/379	152/152	259/259	174/174	195/195	442/442	203/203	180/180	220/220	288/290	211/211	246/249	182/182	230/230	171/171	299/299	259/264	253/253	230/230	178/178	175/175	178/178
151/151	131/131	241/242	100/103	278/278	146/159	138/138	164/164	133/136	228/228	162/165	208/211	364/370	154/161	262/265	171/174	195/196	436/438	206/206	178/178	217/226	288/288	202/211	246/246	186/186	225/225	171/171	304/304	267/273	255/255	230/234	178/183	171/178	172/172
151/153	131/131	241/242	103/103	278/281	146/156	138/138	170/170	130/136	225/234	162/162	206/211	364/370	154/161	259/265	171/174	195/195	436/442	203/214	178/180	220/234	288/290	211/211	246/254	182/186	225/230	171/171	299/302	264/273	255/255	230/234	178/183	175/175	172/178
151/151	129/131	241/242	103/103	278/281	146/156	138/138	170/170	130/136	234/234	165/165	206/211	364/364	154/161	259/265	171/174	196/196	436/442	206/206	178/178	234/234	288/290	211/211	249/254	182/186	225/225	171/171	299/304	261/261	255/255	234/234	178/183	171/175	172/172
151/151	129/131	241/242	103/103	278/281	146/156	138/138	170/170	130/136	234/234	165/165	206/211	364/364	154/161	259/265	171/174	196/196	436/442	206/206	178/178	234/234	288/290	211/211	249/254	182/186	225/225	171/171	299/304	261/261	255/255	234/234	178/183	171/175	172/172
151/151	129/131	241/242	103/103	278/281	146/156	138/138	170/170	130/136	234/234	165/165	206/211	364/364	154/161	259/265	171/174	196/196	436/442	206/206	178/178	234/234	288/290	211/211	249/254	182/186	225/225	171/171	299/304	261/261	255/255	234/234	178/183	171/175	172/172
151/151	129/131	241/242	103/103	278/281	146/156	138/138	170/170	130/136	234/234	165/165	206/211	364/364	154/161	259/265	171/174	196/196	436/442	206/206	178/178	234/234	288/290	211/211	249/254	182/186	225/225	171/171	299/304	261/261	255/255	234/234	178/183	171/175	172/172
151/151	129/131	241/242	103/103	278/281	146/156	138/138	170/170	130/136	234/234	165/165	206/211	364/364	154/161	259/265	171/174	196/196	436/442	206/206	178/178	234/234	288/290	211/211	249/254	182/186	225/225	171/171	299/304	261/261	255/255	234/234	178/183	171/175	172/172
151/151	129/131	241/242	103/103	278/281	146/156	138/138	170/170	130/136	234/234	165/165	206/211	364/364	154/161	259/265	171/174	196/196	436/442	206/206	178/178	234/234	288/290	211/211	249/254	182/186	225/225	171/171	299/304	261/261	255/255	234/234	178/183	171/175	172/172
151/151	129/131	241/242	103/103	278/281	146/156	138/138	170/170	130/136	234/234	165/165	206/211	364/364	154/161	259/265	171/174	196/196	436/442	206/206	178/178	234/234	288/290	211/211	249/254	182/186	225/225	171/171	299/304	261/261	255/255	234/234	178/183	171/175	172/172
151/151	129/131	241/242	103/103	278/281	146/156	138/138	170/170	130/136	234/234	165/165	206/211	364/364	154/161	259/265	171/174	196/196	436/442	206/206	178/178	234/234	288/290	211/211	249/254	182/186	225/225	171/171	299/304	261/261	255/255	234/234	178/183	171/175	172/172
151/151	129/131	241/242	103/103	278/281	146/156	138/138	170/170	130/136	234/234	165/165	206/211	364/364	154/161	259/265	171/174	196/196	436/442	206/206	178/178	234/234	288/290	211/211	249/254	182/186	225/225	171/171	299/304	261/261	255/255	234/234	178/183	171/175	172/172
151/151	129/131	241/242	103/103	278/281	146/156	138/138	170/170	130/136	234/234	165/165	206/211	364/364	154/161	259/265	171/174	196/196	436/442	206/206	178/178	234/234	288/290	211/211	249/254	182/186	225/225	171/171	299/304	261/261	255/255	234/234	178/183	171/175	172/172
151/151	129/131	241/242	103/103	278/281	146/156	138/138	170/170	130/136	234/234	165/165	206/211	364/364	154/161	259/265	171/174	196/196	436/442	206/206	178/178	234/234	288/290	211/211	249/254	182/186	225/225	171/171	299/304	261/261	255/255	234/234	178/183	171/175	172/172
151/151	129/131	241/242	103/103	278/281	146/156	138/138	170/170	130/136	234/234	165/165	206/211	364/364	154/161	259/265	171/174	196/196	436/442	206/206	178/178	234/234	288/290	211/211	249/254	182/186	225/225	171/171	299/304	261/261	255/255	234/234	178/183	171/175	172/172
151/151	129/131	241/242	103/103	278/281	146/156	138/138	170/170	130/136	234/234	165/165	206/211	364/364	154/161	259/265	171/174	196/196	436/442	206/206	178/178	234/234	288/290	211/211	249/254	182/186	225/225	171/171	299/304	261/261	255/255	234/234	178/183	171/175	172/172
151/151	129/131	241/242	103/103	278/281	146/156	138/138	170/170	130/136	234/234	165/165	206/211	364/364	154/161	259/265	171/174	196/196	436/442	206/206	178/178	234/234	288/290	211/211	249/254	182/186	225/225	171/171	299/304	261/261	255/255	234/234	178/183	171/175	172/172
151/151	129/131	241/242	103/103	278/281	146/156	138/138	170/170	130/136	234/234	165/165	206/211	364/364	154/161	259/265	171/174	196/196	436/442	206/206	178/178	234/234	288/290	211/211	249/254	182/186	225/225	171/171	299/304	261/261	255/255	234/234	178/183	171/175	172/172
151/151	129/131	241/242	103/103	278/281	146/156	138/138	170/170	130/136	234/234	165/165	206/211	364/364	154/161	259/265	171/174	196/196	436/442	206/206	178/178	234/234	288/290	211/211	249/254	182/186	225/225	171/171	299/304	261/261	255/255	234/234	178/183	171/175	172/172
151/151	129/131	241/242	103/103	278/281	146/156	138/138	170/170	130/136	234/234	165/165	206/211	364/364	154/161	259/265	171/174	196/196	436/442	206/206	178/178	234/234	288/290	211/211	249/254	182/186	225/225	171/171	299/304	261/261	255/255	234/234	178/183	171/175	172/172
151/151	129/131	241/242	103/103	278/281	146/156	138/138	170/170	130/136	234/234	165/165	206/211	364/364	154/161	259/265	171/174	196/196	436/442	206/206	178/178	234/234	288/290	211/211	249/254	182/186	225/225	171/171	299/304	261/261	255/255	234/234	178/183	171/175	172/172
151/151	129/131	241/242	103/103	278/281	146/156	138/138	170/170	130/136	234/234	165/165	206/211	364/364	154/161	259/265	171/174	196/196	436/442	206/206	178/178	234/234	288/290	211/211	249/254	182/186	225/225	171/171	299/304	261/261	255/255	234/234	178/183	171/175	172/172
151/151	129/131	241/242	103/103	278/281	146/156	138/138	170/170	130/136	234/234	165/165	206/211	364/364	154/161	259/265	171/174	196/196	436/442	206/206	178/178	234/234	288/290	211/211	249/254	182/186	225/225	171/171	299/304	261/261	255/255	234/234	178/183	171/175	172/172
151/151	129/131	241/242	103/103	278/281	146/156	138/138	170/170	130/136	234/234	165/165	206/211	364/364	154/161	259/265	171/174	196/196	436/442	206/206	178/178	234/234	288/290	211/211	249/254	182/186	225/225	171/171	299/304	261/261	255/255	234/234	178/183	171/175	172/172
151/153	131/131	241/242	100/103	278/278	146/156	138/141	158/158	130/136	225/228	162/165	211/211	364/376	158/161	265/265	171/174	195/196	436/442	203/206	178/178	220/234	288/290	211/211	246/246	182/186	225/230	171/171	299/304	256/265	253/255	230/234	178/183	168/178	172/178
151/151	131/131	241/242	100/100	278/278	156/156	138/141	164/164	130/136	228/234	162/165	208/211	364/364	161/161	262/265	171/171	196/196	436/438	203/206	178/178	226/234	288/288	202/211	246/246	186/186	225/225	171/171	304/304	261/267	255/255	230/234	183/183	175/175	172/172
144/151	131/131	241/242	100/100	278/278	156/156	138/141	164/170	133/136	228/234	162/165	208/211	364/364	161/161	262/265	171/174	195/196	436/438	203/206	178/178	226/234	288/288	202/211	246/246	186/186	225/225	171/171	304/307	261/267	255/255	230/234	183/183	175/175	161/172
151/151	129/131	241/241	103/103	278/278	146/156	141/141	170/170	130/133	228/234	162/165	211/211	364/370	154/161	265/265	174/174	195/196	436/442	203/206	178/178	220/234	288/290	211/211	246/246	182/186	225/230	171/171	299/304	259/261	255/255	230/234	178/183	175/175	172/172
144/151	131/131	241/242	103/103	278/278	156/156	138/141	164/170	136/136	228/228	162/165	211/217	364/396	161/161	265/271	171/171	195/196	436/436	206/214	178/178	232/234	288/288	211/211	249/249	186/186	225/225	171/171	299/304	273/273	255/255	234/234	183/183	168/175	172/172
151/153	131/131	241/242	100/103	278/278	146/156	138/141	164/164	130/136	225/228	162/165	206/211	364/370	154/158	259/265	171/174	195/195	438/442	206/206	178/178	217/223	288/290	211/211	246/246	182/186	225/230	171/171	304/307	259/273	253/255	230/234	178/183	175/178	172/178
151/153	131/131	241/242	100/103	278/278	146/156	138/141	164/164	130/136	225/228	162/165	206/211	364/370	154/158	259/265	171/174	195/195	438/442	206/206	178/178	217/223	288/290	211/211	246/246	182/186	225/230	171/171	304/307	259/273	253/255	230/234	178/183	175/178	172/178
151/153	131/131	241/242	100/103	278/278	146/156	138/141	164/164	130/136	225/228	162/165	206/211	364/370	154/158	259/265	171/174	195/195	438/442	206/206	178/178	217/223	288/290	211/211	246/246	182/186	225/230	171/171	304/307	259/273	253/255	230/234	178/183	175/178	172/178
151/153	131/131	241/242	100/103	278/278	146/156	138/141	164/164	130/136	225/228	162/165	206/211	364/370	154/158	259/265	171/174	195/195	438/442	206/206	178/178	217/223	288/290	211/211	246/246	182/186	225/230	171/171	304/307	259/273	253/255	230/234	178/183	175/178	172/178
151/153	131/131	241/242	100/103	278/278	146/156	138/141	164/164	130/136	225/228	162/165	206/211	364/370	154/158	259/265	171/174	195/195	438/442	206/206	178/178	217/223	288/290	211/211	246/246	182/186	225/230	171/171	304/307	259/273	253/255	230/234	178/183	175/178	172/178
144/151	131/131	241/242	100/100	278/278	146/156	138/141	170/170	122/136	228/228	162/168	211/211	364/396	154/158	265/265	171/171	195/195	436/438	206/206	178/178	220/223	288/288	211/211	246/246	186/186	225/225	171/171	299/304	259/270	255/259	230/234	175/183	168/175	172/172
153/153	129/129	242/242	103/103	278/281	146/146	nd.	170/170	130/130	225/228	162/162	206/206	370/379	152/154	259/259	174/174	195/195	442/442	203/203	180/180	217/223	288/290	211/211	249/254	182/182	230/230	171/171	299/299	259/259	253/255	230/230	178/178	175/175	172/184
144/153	131/131	241/241	100/100	278/278	156/156	141/141	164/164	136/136	228/234	165/165	211/214	364/364	161/161	265/268	171/174	195/196	436/436	206/206	178/178	217/229	288/288	211/211	246/246	186/186	225/225	171/171	304/304	261/261	255/255	234/234	183/183	168/175	172/172
151/151	131/131	241/242	100/103	278/278	156/156	138/138	153/164	122/133	228/234	162/162	211/211	364/364	161/161	265/265	171/174	195/196	436/436	206/214	178/178	226/234	288/288	211/211	246/246	186/186	225/225	171/171	299/304	261/273	255/255	230/234	183/183	168/175	172/172
151/153	131/131	241/242	100/103	278/278	146/156	138/141	164/170	130/136	225/234	165/177	206/211	367/396	154/161	259/265	171/174	195/195	436/442	206/206	178/180	220/220	288/290	211/211	249/249	182/186	225/225	171/171	304/304	259/261	253/255	234/234	178/183	175/178	172/172
151/153	131/131	241/242	100/103	278/278	146/156	138/141	164/170	130/136	225/234	165/177	206/211	367/396	154/161	259/265	171/174	195/195	436/442	206/206	178/180	220/220	288/290	211/211	249/249	182/186	225/225	171/171	304/304	259/261	253/255	234/234	178/183	175/178	172/172
151/153	131/131	241/242	100/103	278/278	146/156	138/141	164/170	130/136	225/234	165/177	206/211	367/396	154/161	259/265	171/174	195/195	436/442	206/206	178/180	220/220	288/290	211/211	249/249	182/186	225/225	171/171	304/304	259/261	253/255	234/234	178/183	175/178	172/172
151/153	131/131	241/242	100/103	278/278	146/156	138/141	164/170	130/136	225/234	165/177	206/211	367/396	154/161	259/265	171/174	195/195	436/442	206/206	178/180	220/220	288/290	211/211	249/249	182/186	225/225	171/171	304/304	259/261	253/255	234/234	178/183	175/178	172/172
151/153	131/131	241/242	100/103	278/278	146/156	138/141	164/170	130/136	225/234	165/177	206/211	367/396	154/161	259/265	171/174	195/195	436/442	206/206	178/180	220/220	288/290	211/211	249/249	182/186	225/225	171/171	304/304	259/261	253/255	234/234	178/183	175/178	172/172
151/153	131/131	241/242	100/103	278/278	146/156	138/141	164/170	130/136	225/234	165/177	206/211	367/396	154/161	259/265	171/174	195/195	436/442	206/206	178/180	220/220	288/290	211/211	249/249	182/186	225/225	171/171	304/304	259/261	253/255	234/234	178/183	175/178	172/172
151/153	131/131	241/242	100/103	278/278	146/156	138/141	164/170	130/136	225/234	165/177	206/211	367/396	154/161	259/265	171/174	195/195	436/442	206/206	178/180	220/220	288/290	211/211	249/249	182/186	225/225	171/171	304/304	259/261	253/255	234/234	178/183	175/178	172/172
151/153	131/131	241/242	100/103	278/278	146/156	138/141	164/164	130/136	225/234	165/177	206/211	367/396	154/161	259/265	171/174	195/195	436/442	206/206	178/180	220/220	288/290	211/211	249/249	182/186	225/225	171/171	304/304	259/261	253/255	234/234	178/183	175/178	172/172
151/153	131/131	241/242	100/103	278/278	146/156	138/141	164/170	130/136	225/234	165/177	206/211	367/396	154/161	259/265	171/174	195/195	436/442	206/206	178/180	220/220	288/290	211/211	249/249	182/186	225/225	171/171	304/304	259/261	253/255	234/234	178/183	175/178	172/172
151/153	131/131	241/242	100/103	278/278	146/156	138/141	164/170	130/136	225/234	165/177	206/211	367/396	154/161	259/265	171/174	195/195	436/442	206/206	178/180	220/220	288/290	211/211	249/249	182/186	225/225	171/171	304/304	259/261	253/255	234/234	178/183	175/178	172/172
151/153	131/131	241/242	100/103	278/278	146/156	138/141	164/170	130/136	225/234	165/177	206/211	367/396	154/161	259/265	171/174	195/195	436/442	206/206	178/180	220/220	288/290	211/211	249/249	182/186	225/225	171/171	304/304	259/261	253/255	234/234	178/183	175/178	172/172
151/153	131/131	241/242	100/103	278/278	146/156	138/141	164/170	130/136	225/234	165/177	206/211	367/396	154/161	259/265	171/174	195/195	436/442	206/206	178/180	220/220	288/290	211/211	249/249	182/186	225/225	171/171	304/304	259/261	253/255	234/234	178/183	175/178	172/172
151/153	131/131	241/242	100/103	278/278	146/156	138/141	164/170	130/136	225/234	165/177	206/211	367/396	154/161	259/265	171/174	195/195	436/442	206/206	178/180	220/220	288/290	211/211	249/249	182/186	225/225	171/171	304/304	259/261	253/255	234/234	178/183	175/178	172/172
151/153	131/131	241/242	100/103	278/278	146/156	138/141	164/170	130/136	225/234	165/177	206/211	367/396	154/161	259/265	171/174	195/195	436/442	206/206	178/180	220/220	288/290	211/211	249/249	182/186	225/225	171/171	304/304	259/261	253/255	234/234	178/183	175/178	172/172
151/153	131/131	241/242	100/103	278/278	146/156	138/141	164/170	130/136	225/234	165/177	206/211	367/396	154/161	259/265	171/174	195/195	436/442	206/206	178/180	220/220	288/290	211/211	249/249	182/186	225/225	171/171	304/304	259/261	253/255	234/234	178/183	175/178	172/172
151/153	131/131	241/242	100/103	278/278	146/156	138/141	164/170	130/136	225/234	165/177	206/211	367/396	154/161	259/265	171/174	195/195	436/442	206/206	178/180	220/220	288/290	211/211	249/249	182/186	225/225	171/171	304/304	259/261	253/255	234/234	178/183	175/178	172/172
151/153	131/131	241/242	100/103	278/278	146/156	138/141	164/170	130/136	225/234	165/177	206/211	367/396	154/161	259/265	171/174	195/195	436/442	206/206	178/180	220/220	288/290	211/211	249/249	182/186	225/225	171/171	304/304	259/261	253/255	234/234	178/183	175/178	172/172
151/153	131/131	241/242	100/103	278/278	146/156	138/141	164/170	130/136	225/234	165/177	206/211	367/396	154/161	259/265	171/174	195/195	436/442	206/206	178/180	220/220	288/290	211/211	249/249	182/186	225/225	171/171	304/304	259/261	253/255	234/234	178/183	175/178	172/172
151/153	131/131	241/242	100/103	278/278	146/156	138/141	164/170	130/136	225/234	165/177	206/211	367/396	154/161	259/265	171/174	195/195	436/442	206/206	178/180	220/220	288/290	211/211	249/249	182/186	225/225	171/171	304/304	259/261	253/255	234/234	178/183	175/178	172/172
151/153	131/131	241/242	100/103	278/278	146/156	138/141	164/170	130/136	225/234	165/177	206/211	367/396	154/161	259/265	171/174	195/195	436/442	206/206	178/180	220/220	288/290	211/211	249/249	182/186	225/225	171/171	304/304	259/261	253/255	234/234	178/183	175/178	172/172
151/153	131/131	241/242	100/103	278/278	146/156	138/141	164/170	130/136	225/234	165/177	206/211	367/396	154/161	259/265	171/174	195/195	436/442	206/206	178/180	220/220	288/290	211/211	249/249	182/186	225/225	171/171	304/304	259/261	253/255	234/234	178/183	175/178	172/172
151/151	131/131	242/242	100/100	278/278	156/159	138/141	153/164	133/136	228/234	162/165	208/211	364/364	161/161	262/265	171/171	195/196	436/436	206/206	178/178	234/234	288/288	202/202	246/246	186/186	225/225	171/171	304/307	261/267	255/255	230/230	183/183	171/175	172/172
151/151	131/131	242/242	100/100	278/278	156/156	138/141	153/164	133/133	228/234	162/165	208/211	364/364	161/161	262/265	171/171	195/196	436/436	203/206	178/178	226/234	288/288	202/211	246/246	186/186	225/225	171/171	304/304	261/267	255/255	230/230	183/183	168/175	172/172
151/151	131/131	242/242	100/100	278/278	156/156	138/141	153/164	133/133	228/234	162/165	208/211	364/364	161/161	262/265	171/171	195/196	436/436	203/206	178/178	226/234	288/288	202/211	246/246	186/186	225/225	171/171	304/304	261/267	255/255	230/230	183/183	168/175	172/172
151/151	131/131	242/242	100/100	278/278	156/156	138/141	153/164	133/133	228/234	162/165	208/211	364/364	161/161	262/265	171/171	195/196	436/436	203/206	178/178	226/234	288/288	202/211	246/246	186/186	225/225	171/171	304/304	261/267	255/255	230/230	183/183	168/175	172/172
151/151	131/131	242/242	100/100	278/278	156/159	138/141	153/164	133/136	228/234	162/165	208/211	364/364	161/161	262/265	171/171	195/196	436/436	206/206	178/178	234/234	288/288	202/202	246/246	186/186	225/225	171/171	304/307	261/267	255/255	230/230	183/183	171/175	172/172
151/151	131/131	242/242	100/100	278/278	156/159	138/141	153/164	133/136	228/234	162/165	208/211	364/364	161/161	262/265	171/171	195/196	436/436	206/206	178/178	234/234	288/288	202/202	246/246	186/186	225/225	171/171	304/307	261/267	255/255	230/230	183/183	171/175	172/172
144/151	131/131	242/242	100/100	278/278	156/156	138/141	164/170	133/136	228/228	162/165	208/211	364/364	158/161	262/265	171/171	195/195	436/436	203/206	178/178	223/226	288/288	202/211	246/246	186/186	225/225	171/171	304/307	256/267	255/255	230/234	183/183	175/175	172/172
151/151	131/131	242/242	100/100	278/278	156/156	138/141	153/164	133/133	228/234	162/165	208/211	364/364	161/161	262/265	171/171	195/196	436/436	203/206	178/178	226/234	288/288	202/211	246/246	186/186	225/225	171/171	304/304	261/267	255/255	230/230	183/183	168/175	172/172
151/151	131/131	242/242	100/100	278/278	156/156	138/141	153/164	133/133	228/234	162/165	208/211	364/364	161/161	262/265	171/171	195/196	436/436	203/206	178/178	226/234	288/288	202/211	246/246	186/186	225/225	171/171	304/304	261/267	255/255	230/230	183/183	168/175	172/172
151/151	131/131	242/242	100/100	278/278	156/156	138/141	153/164	133/133	228/234	162/165	208/211	364/364	161/161	262/265	171/171	195/196	436/436	203/206	178/178	226/234	288/288	202/211	246/246	186/186	225/225	171/171	304/304	261/267	255/255	230/230	183/183	168/175	172/172
151/151	131/131	242/242	100/100	278/278	156/159	138/141	153/164	133/136	228/234	162/165	208/211	364/364	161/161	262/265	171/171	195/196	436/436	206/206	178/178	234/234	288/288	202/202	246/246	186/186	225/225	171/171	304/307	261/267	255/255	230/230	183/183	171/175	172/172
151/151	131/131	242/242	100/100	278/278	156/156	138/141	153/164	133/133	228/234	162/165	208/211	364/364	161/161	262/265	171/171	195/196	436/436	203/206	178/178	226/234	288/288	202/211	246/246	186/186	225/225	171/171	304/304	261/267	255/255	230/230	183/183	168/175	172/172
144/151	129/131	242/242	100/103	278/278	146/156	138/138	164/170	133/136	228/228	162/162	208/211	364/364	154/161	262/265	171/174	195/196	436/436	206/206	178/178	220/226	288/288	202/211	246/249	186/186	225/225	171/171	304/304	259/267	255/255	230/234	178/183	175/175	172/172
151/153	129/131	241/242	103/103	278/278	146/156	141/141	161/164	130/136	225/228	162/162	206/206	364/370	152/158	259/259	174/174	195/195	442/442	206/206	178/180	220/223	288/290	211/211	246/246	182/182	230/230	171/171	299/304	259/264	253/255	230/230	178/183	175/175	172/181
151/153	129/131	241/241	100/103	278/278	146/156	138/141	153/164	133/136	228/234	165/165	211/214	364/367	154/161	265/268	171/174	195/195	436/436	203/206	178/180	217/220	288/288	211/211	249/249	186/186	225/225	171/171	304/304	261/273	253/255	234/234	178/183	168/175	172/172
151/153	129/131	241/241	100/103	278/278	146/156	138/141	153/164	133/136	228/234	165/165	211/214	364/367	154/161	265/268	171/174	195/195	436/436	203/206	178/180	217/220	288/288	211/211	249/249	186/186	225/225	171/171	304/304	261/273	253/255	234/234	178/183	168/175	172/172
151/153	129/131	241/241	100/103	278/278	146/156	138/141	153/164	133/136	228/234	165/165	211/214	364/367	154/161	265/268	171/174	195/195	436/436	203/206	178/180	217/220	288/288	211/211	249/249	186/186	225/225	171/171	304/304	261/273	253/255	234/234	178/183	168/175	172/172
151/153	129/131	241/241	100/103	278/278	146/156	138/141	153/164	133/136	228/234	165/165	211/214	364/367	154/161	265/268	171/174	195/195	436/436	203/206	178/180	217/220	288/288	211/211	249/249	186/186	225/225	171/171	304/304	261/273	253/255	234/234	178/183	168/175	172/172
151/153	129/131	241/242	100/100	278/278	146/156	138/141	153/164	136/136	225/234	165/177	211/211	364/396	161/161	265/265	171/174	195/195	436/436	206/206	178/180	217/220	288/288	211/211	249/249	186/186	225/225	171/171	304/304	259/273	253/255	234/234	178/183	175/178	172/172
153/153	129/131	241/242	103/103	278/281	146/146	138/138	170/170	122/136	228/234	162/165	206/211	379/396	152/161	259/265	171/174	195/195	436/442	206/214	180/180	220/223	288/288	211/211	246/249	182/186	225/230	171/171	299/304	261/264	253/255	230/234	178/178	175/175	172/181
151/153	131/131	241/241	100/103	278/278	146/156	138/141	170/170	130/136	225/234	165/177	211/211	364/367	161/161	265/265	171/174	195/196	436/438	206/206	178/180	220/234	288/288	211/211	249/249	186/186	225/225	171/171	304/304	259/261	255/255	234/234	178/183	175/178	172/172
144/151	131/131	241/242	100/100	278/278	156/156	138/141	153/170	122/133	228/234	162/162	208/211	364/396	154/161	262/265	171/171	195/196	436/436	206/206	178/178	220/226	288/288	211/211	246/246	186/186	225/225	171/171	304/304	259/261	255/255	230/234	183/183	168/175	172/172
151/153	129/131	241/242	103/103	278/281	146/156	138/141	164/170	130/136	234/234	162/165	206/211	364/367	154/161	259/265	174/174	195/196	436/442	206/214	178/178	220/234	288/290	211/211	249/254	182/186	225/230	171/171	304/304	261/273	255/255	230/234	nd.	171/175	172/172
153/153	129/129	242/242	103/103	278/281	146/146	138/138	170/170	122/130	225/234	162/162	206/206	379/385	152/154	259/259	174/174	195/195	442/442	203/214	178/180	220/223	288/288	211/211	246/249	182/182	218/230	171/171	299/304	259/273	253/255	230/230	178/178	175/175	172/181
151/153	129/131	241/241	100/100	278/278	146/156	138/138	164/164	130/136	228/234	162/165	208/208	364/364	154/161	262/262	171/174	195/196	436/436	206/206	178/178	226/234	288/288	211/211	246/246	186/186	225/230	171/171	304/304	261/261	255/255	230/234	178/183	175/175	172/172
151/153	129/131	241/242	103/103	278/281	146/156	138/141	164/170	130/136	234/234	162/165	206/211	364/367	154/161	259/265	174/174	195/196	436/442	206/214	178/178	220/234	288/290	211/211	249/254	182/186	225/230	171/171	304/304	261/273	255/255	230/234	178/183	171/175	172/172
151/153	129/131	241/242	103/103	278/281	146/156	138/141	164/170	130/136	234/234	162/165	206/211	364/367	154/161	259/265	174/174	195/196	436/442	206/214	178/178	220/234	288/290	211/211	249/254	182/186	225/230	171/171	304/304	261/273	255/255	230/234	178/183	171/175	172/172
151/153	129/131	241/242	103/103	278/281	146/156	138/141	164/170	130/136	234/234	162/165	206/211	364/367	154/161	259/265	174/174	195/196	436/442	206/214	178/178	220/234	288/290	211/211	249/254	182/186	225/230	171/171	304/304	261/273	255/255	230/234	178/183	171/175	172/172
153/153	129/129	242/242	103/103	281/281	146/146	138/138	161/161	130/130	228/228	162/162	206/206	379/379	152/154	259/259	174/174	195/195	442/442	203/203	180/180	220/223	290/290	211/211	246/254	182/182	230/230	171/171	299/302	259/259	253/253	230/230	178/178	175/175	178/181
151/153	129/131	242/242	103/103	278/281	146/156	141/141	164/170	130/136	228/234	162/162	206/211	364/370	154/161	259/265	174/174	196/196	436/442	208/214	178/178	220/234	288/290	211/211	246/246	182/186	218/225	171/171	299/304	273/273	255/255	230/234	178/183	175/175	172/172
151/151	129/131	241/242	100/100	278/281	146/156	138/138	164/170	130/133	228/234	162/162	206/208	364/367	154/161	259/262	171/174	195/196	436/442	206/214	178/178	220/226	288/290	202/211	246/254	182/186	225/230	171/171	304/307	267/273	255/255	230/230	178/183	175/175	172/172
151/153	129/129	242/242	87/103	281/281	146/156	138/138	170/170	122/136	225/234	162/165	206/211	364/379	152/154	259/265	174/174	195/196	442/442	206/208	178/180	217/234	288/290	211/211	246/249	182/182	225/230	171/171	299/302	259/261	253/255	230/234	178/178	171/175	172/178
151/151	131/131	241/241	100/103	278/278	156/156	141/141	153/164	136/136	228/228	165/165	211/211	364/364	161/161	265/265	171/171	196/196	436/436	206/206	178/178	217/234	288/288	211/211	246/249	186/186	225/225	171/171	304/304	259/273	255/255	234/234	183/183	175/175	172/172
144/151	129/131	241/242	100/103	278/278	156/156	141/141	153/153	136/136	228/234	165/165	211/211	364/364	161/161	265/265	171/174	196/196	436/436	206/214	178/178	217/234	288/288	211/211	246/249	186/186	225/225	171/171	304/304	259/261	255/255	234/234	183/183	175/175	172/172
151/153	131/131	242/242	100/103	278/281	146/156	138/138	164/164	130/133	225/234	162/162	206/211	364/370	152/161	259/265	171/174	196/196	436/442	203/206	178/180	220/234	288/290	211/211	246/246	182/186	225/230	171/171	302/304	261/264	253/255	230/234	178/183	175/175	172/178
153/153	129/131	241/242	100/103	278/281	146/156	141/141	170/170	122/136	225/234	162/165	206/206	379/396	152/161	259/259	174/174	195/195	442/442	206/206	180/180	220/223	288/288	211/211	246/249	182/186	225/230	171/171	299/304	259/261	253/255	230/234	178/178	175/175	172/181
151/151	131/131	241/241	103/103	278/281	156/156	141/141	170/170	130/133	234/234	162/165	206/211	364/367	154/161	259/265	171/174	195/196	436/442	206/214	178/178	220/234	288/290	211/211	246/254	182/186	225/230	171/171	299/304	261/273	255/255	230/234	183/183	175/175	172/172
151/153	129/131	242/242	103/103	278/281	146/156	141/141	164/164	130/136	225/228	162/162	206/211	364/379	152/161	259/265	174/174	195/196	436/442	203/214	178/180	220/234	288/290	211/211	246/246	182/186	218/230	171/171	299/304	264/273	253/255	230/230	178/183	175/175	172/181
151/151	131/131	241/241	100/100	278/278	156/156	141/141	153/164	136/136	228/228	165/165	211/211	364/364	161/161	265/265	171/174	196/196	436/436	206/206	178/178	229/234	288/288	211/211	246/246	186/186	225/225	171/171	304/304	270/273	255/255	234/234	183/183	175/175	172/172
151/153	129/131	242/242	103/103	278/281	146/156	138/138	170/170	130/136	225/228	162/165	206/211	364/370	152/161	259/265	174/174	196/196	436/442	203/206	178/180	220/220	288/290	211/211	246/246	182/186	225/225	171/171	302/304	264/273	253/255	234/234	178/183	175/175	172/178
151/151	131/131	242/242	100/100	278/278	146/156	138/138	167/167	122/133	228/228	162/168	211/211	364/376	154/161	265/265	171/171	194/194	436/438	194/206	178/178	223/229	288/288	211/211	246/246	186/189	225/225	171/171	299/304	247/270	255/259	230/234	175/183	168/171	172/172
151/153	129/131	241/242	100/103	278/278	146/156	138/141	170/170	130/136	234/234	165/165	211/211	364/367	154/161	265/265	174/174	195/196	436/436	206/206	178/178	220/223	288/290	211/211	249/254	182/186	225/225	171/171	304/304	261/261	nd.	234/234	178/183	175/175	172/172
151/153	129/131	242/242	103/103	278/278	146/156	138/138	164/170	130/136	228/234	165/165	206/214	364/367	154/161	259/268	174/174	195/196	436/442	206/206	178/178	220/220	288/290	211/211	249/254	186/186	225/225	171/171	299/304	261/273	253/255	234/234	178/178	175/175	172/172

151/151	131/131	241/241	100/100	278/278	146/156	138/138	170/170	130/136	234/234	165/177	206/211	364/367	154/161	259/265	171/174	195/196	436/442	206/206	178/180	220/234	288/290	211/211	249/254	182/186	225/225	171/171	304/304	261/261	253/255	234/234	178/183	175/178	172/172
151/153	129/131	241/242	100/103	278/281	146/156	141/141	153/170	130/136	228/234	162/165	206/211	364/367	161/161	259/265	171/174	195/195	436/442	206/214	178/178	217/220	288/290	211/211	246/249	182/186	225/230	171/171	299/304	259/261	255/255	230/234	178/183	175/175	172/172
144/151	129/131	241/242	100/100	278/278	146/156	138/138	153/170	130/136	228/234	165/165	206/211	364/364	161/161	259/265	171/174	196/196	436/442	206/214	178/178	217/234	288/290	211/211	249/254	186/186	225/225	171/171	304/304	261/273	255/255	234/234	178/178	175/175	172/172
151/153	131/131	241/241	100/103	278/278	146/156	138/141	153/164	130/136	225/234	177/177	206/211	364/396	154/161	259/265	171/171	195/196	436/442	206/206	178/180	220/220	288/290	211/211	249/249	182/186	225/225	171/171	304/304	259/259	253/255	234/234	178/183	175/175	172/172
151/151	131/131	241/241	100/100	278/278	146/156	138/141	164/164	130/136	225/234	165/165	211/211	364/396	161/161	265/265	171/174	195/196	436/436	206/206	178/178	220/220	288/290	211/211	249/249	186/186	225/225	171/171	304/304	261/261	253/255	234/234	178/183	175/175	172/172
153/153	131/131	242/244	103/103	278/281	146/153	138/147	170/170	130/136	228/228	159/162	200/206	364/379	152/152	254/259	171/174	195/195	421/442	197/203	172/180	202/220	288/293	211/211	246/254	182/186	230/230	170/171	302/304	253/259	253/255	230/230	178/178	168/175	172/178
151/151	129/131	241/242	100/103	278/278	156/156	138/141	164/170	130/130	228/234	162/165	211/211	364/367	152/161	265/265	171/174	195/196	436/436	203/206	178/180	220/226	290/290	211/211	246/249	186/186	225/225	171/171	304/304	261/264	253/255	230/234	178/183	168/178	172/172
151/153	129/129	241/242	100/103	278/278	156/156	138/141	153/170	136/136	228/234	165/165	211/211	364/364	154/161	265/265	174/174	196/196	436/436	206/214	178/178	220/220	288/288	211/211	249/249	186/186	225/225	171/171	304/304	261/261	253/255	234/234	178/183	175/175	172/172
153/153	129/129	242/242	103/103	281/281	146/146	138/138	156/164	130/130	225/228	162/162	206/211	379/381	152/152	259/265	174/174	195/195	442/442	203/206	180/180	220/220	288/290	211/211	246/246	182/182	218/230	171/171	299/307	259/264	253/253	230/230	178/178	175/184	178/184
153/153	129/131	242/242	103/103	281/281	146/146	nd.	161/164	130/130	228/228	162/162	206/206	370/370	152/152	259/259	174/174	195/195	442/442	203/203	180/180	217/220	288/290	211/211	249/254	182/182	230/230	171/171	299/299	259/264	253/253	230/230	178/178	175/175	178/178
144/153	131/131	242/242	100/100	278/281	156/156	141/141	164/164	136/136	228/234	177/177	206/214	364/370	154/161	259/268	171/171	196/196	436/436	206/206	178/180	220/220	288/288	211/211	249/254	186/186	225/225	171/171	304/304	259/259	253/255	234/234	178/183	175/175	172/172
144/151	129/129	241/242	100/100	278/278	156/156	138/141	153/164	136/136	228/234	165/165	211/211	364/364	161/161	265/265	174/174	196/196	436/436	206/214	178/178	217/220	288/288	211/211	249/254	186/186	225/225	171/171	304/304	261/273	255/255	234/234	178/183	175/175	172/172
144/153	131/131	241/241	100/103	278/281	146/156	138/141	164/170	136/136	234/234	165/177	206/211	364/370	154/161	259/265	171/174	196/196	436/442	206/214	178/180	220/234	288/288	211/211	249/249	182/186	225/225	171/171	304/304	259/261	255/255	234/234	178/183	175/175	172/172
144/151	129/131	241/241	100/100	278/278	146/156	138/141	164/170	130/136	228/234	165/177	206/214	364/370	161/161	259/268	171/174	196/196	436/436	206/214	178/180	220/234	288/290	211/211	249/249	186/186	225/225	171/171	304/304	259/261	255/255	234/234	178/183	175/175	172/172
151/153	129/131	241/241	103/103	278/281	146/156	138/141	170/170	136/136	234/234	165/177	206/214	364/364	154/161	259/268	171/174	196/196	436/442	206/206	178/178	220/234	288/288	211/211	249/254	182/186	225/225	171/171	304/304	259/261	253/255	234/234	178/183	175/175	172/172
144/151	129/129	241/242	100/100	278/278	156/156	138/141	164/164	136/136	228/228	165/165	211/211	364/396	161/161	265/265	174/174	195/196	436/436	206/206	178/178	217/220	288/288	211/211	246/254	186/186	225/225	171/171	304/304	273/273	255/255	234/234	178/183	175/175	172/172
151/151	131/131	241/242	100/100	278/278	146/156	138/138	170/170	130/130	228/234	162/165	211/211	364/364	152/161	265/265	171/174	196/196	436/436	206/214	178/178	220/226	290/290	211/211	246/254	186/186	225/225	171/171	304/304	261/264	255/255	234/234	178/183	175/178	172/172
151/151	131/131	241/241	103/103	278/281	146/156	138/138	164/170	133/136	228/228	162/162	206/211	364/367	154/161	259/265	171/174	195/196	436/442	203/206	178/180	220/226	288/288	211/211	249/254	182/186	225/225	171/171	304/304	264/273	255/255	230/234	178/183	168/175	172/172
151/153	129/131	241/241	100/100	278/278	146/156	138/141	164/164	130/133	234/234	165/165	211/211	364/396	161/161	265/265	171/174	195/196	436/436	206/206	178/178	220/234	288/290	211/211	249/249	186/186	225/225	171/171	304/304	261/261	253/255	234/234	178/183	175/175	172/172
151/151	129/131	241/241	100/100	278/278	156/156	138/141	153/170	130/133	228/234	165/165	211/211	364/396	161/161	265/265	171/174	195/196	436/436	206/214	178/178	220/234	288/290	211/211	246/254	186/186	225/225	171/171	299/304	261/273	255/255	234/234	183/183	168/175	172/172
153/157	129/129	242/242	103/103	278/278	146/146	138/138	156/164	130/130	225/225	162/162	206/208	379/381	152/152	259/262	174/174	195/195	442/442	203/203	180/180	217/220	290/290	211/211	246/246	182/182	218/230	171/171	299/307	259/264	253/253	230/230	178/178	175/175	178/184
144/151	131/131	241/242	100/100	278/278	146/156	138/141	164/170	130/136	228/234	162/165	206/211	364/367	161/161	259/265	171/171	195/195	436/442	206/206	178/178	220/234	288/290	211/211	246/254	186/186	225/225	171/171	304/304	261/264	255/255	234/234	178/183	175/175	172/172
151/153	129/131	242/242	103/103	278/278	146/156	138/138	164/164	130/136	228/234	165/177	211/211	364/364	161/161	265/265	174/174	196/196	436/436	206/206	178/178	220/220	288/290	211/211	249/254	186/186	225/225	171/171	299/304	259/261	253/255	234/234	178/178	175/175	172/172
144/151	131/131	242/242	100/103	278/278	156/156	138/141	153/164	133/136	228/234	165/165	211/211	364/364	161/161	265/265	171/174	196/196	436/436	206/214	178/178	217/229	288/288	211/211	246/246	186/186	225/225	171/171	304/304	261/273	255/255	234/234	178/183	175/175	172/172
151/151	129/131	241/241	100/100	278/278	146/156	nd.	153/164	130/136	225/234	165/177	206/211	364/396	161/161	259/265	174/174	195/196	436/442	206/214	178/178	220/232	288/290	211/211	246/249	186/186	225/225	171/171	304/304	259/273	253/255	234/234	178/183	168/175	172/172
151/151	129/131	241/242	100/100	278/281	156/156	138/138	164/170	136/136	228/234	165/165	206/211	364/367	161/161	259/265	171/174	195/196	436/442	206/206	178/178	220/234	288/288	211/211	246/254	182/186	225/225	171/171	299/304	261/273	255/255	234/234	178/183	171/175	172/172
151/153	129/131	241/242	100/100	278/278	156/156	138/141	153/164	136/136	228/234	165/177	206/211	364/364	161/161	259/265	171/174	196/196	436/436	214/214	178/178	223/234	288/288	211/211	249/254	186/186	225/225	171/171	304/304	259/273	253/255	234/234	183/183	175/175	172/172
151/153	129/131	242/242	100/103	278/278	156/156	138/141	153/164	136/136	228/234	165/165	211/211	364/367	161/161	265/265	174/174	195/195	436/436	206/206	178/178	217/220	288/288	211/211	246/249	186/186	225/225	171/171	304/304	261/273	255/255	234/234	178/183	175/175	172/172
151/153	129/131	241/242	103/103	278/278	146/156	138/141	164/170	130/130	234/234	165/165	206/211	364/367	154/161	259/265	171/174	195/196	436/442	206/206	178/178	220/234	290/290	211/211	249/254	186/186	225/225	171/171	304/304	261/261	253/255	234/234	178/183	175/178	172/172
151/153	129/131	241/242	100/100	278/281	146/156	138/138	164/164	130/133	228/234	162/165	206/211	364/367	152/161	259/265	171/174	195/196	436/442	203/203	178/180	223/226	288/290	211/211	246/254	182/186	225/230	171/171	304/304	261/264	253/255	230/230	178/183	168/175	172/172
151/153	129/131	241/242	100/103	278/278	156/156	141/141	153/164	136/136	234/234	165/165	211/214	396/396	154/161	265/268	171/174	195/195	436/436	206/214	178/178	220/220	288/288	211/211	249/249	186/186	225/225	171/171	304/304	259/261	253/255	234/234	183/183	175/175	172/172
151/153	129/129	242/242	103/103	278/281	146/156	138/141	170/170	130/130	225/228	162/162	206/211	370/381	152/152	259/265	174/174	195/195	436/442	203/214	178/180	220/220	288/290	211/211	249/254	182/186	230/230	171/171	299/304	264/264	253/253	230/230	178/183	175/175	172/184
144/151	129/129	241/241	100/100	278/281	146/146	138/138	153/170	130/136	228/234	162/165	206/211	364/364	161/161	259/265	174/174	196/196	436/442	206/206	178/178	220/234	288/288	211/211	249/249	182/186	225/225	171/171	304/304	261/273	255/255	234/234	178/183	171/175	172/172
151/151	129/129	241/242	103/103	278/278	146/156	138/141	164/170	130/136	234/234	165/165	211/211	364/364	154/161	265/265	174/174	196/196	436/436	206/206	178/178	220/234	288/290	211/211	254/254	186/186	225/225	171/171	304/304	261/261	255/255	234/234	178/183	171/175	172/172
144/153	129/131	242/242	100/103	278/281	146/156	138/141	153/170	130/136	228/234	165/165	206/214	364/370	161/161	259/268	171/174	196/196	436/442	206/206	178/180	220/223	288/290	211/211	246/249	182/186	225/225	171/171	304/304	261/273	255/255	234/234	178/183	175/175	172/172
144/151	129/131	241/242	100/100	278/281	146/156	138/141	164/170	130/136	234/234	165/177	206/206	364/364	161/161	259/259	171/174	196/196	436/442	nd.	178/178	220/234	288/290	211/211	249/254	182/186	225/225	171/171	299/304	259/261	255/255	234/234	178/183	175/175	172/172
151/153	131/131	241/242	100/100	nd.	146/146	138/138	164/170	130/130	225/234	165/177	206/211	364/396	154/161	259/265	171/174	195/196	436/442	206/206	178/178	220/234	290/290	211/211	249/254	186/186	225/225	171/171	304/304	259/261	253/253	234/234	178/183	175/175	172/172
151/153	131/131	241/242	100/103	278/278	156/156	141/141	164/170	133/136	228/234	162/165	211/211	364/396	161/161	265/265	171/174	195/195	436/436	214/214	178/178	220/234	288/288	211/211	249/249	186/186	225/225	171/171	299/304	261/273	253/255	234/234	183/183	175/175	172/172
151/153	129/131	241/242	100/103	278/278	156/156	138/138	164/164	133/133	228/228	162/165	211/211	364/364	161/161	265/265	174/174	195/196	436/436	206/214	178/178	220/234	288/288	211/211	249/254	186/186	225/225	171/171	304/304	273/273	255/255	234/234	178/183	175/175	172/172
153/153	131/131	242/242	103/103	278/281	146/153	138/147	170/170	130/136	228/228	159/162	206/211	364/379	152/154	259/265	171/174	195/195	436/442	197/203	172/178	220/220	288/293	211/211	246/254	182/186	230/230	171/171	302/304	253/259	253/255	230/230	178/178	168/175	172/178
151/153	129/131	241/241	100/103	278/278	146/156	138/141	164/170	130/136	234/234	165/177	206/214	396/396	161/161	259/268	171/174	195/195	436/436	206/206	178/178	220/220	288/290	211/211	249/249	186/186	225/225	171/171	304/304	259/261	253/255	234/234	183/183	175/175	172/172
151/153	131/131	242/242	100/100	278/281	156/156	138/141	164/170	130/136	234/234	165/165	206/214	364/370	154/161	259/268	171/171	196/196	436/442	206/206	178/178	220/234	288/290	211/211	249/254	182/186	225/225	171/171	299/304	261/261	253/255	234/234	178/183	175/175	172/172
151/153	129/131	241/242	100/103	278/281	146/156	138/141	153/170	130/136	228/234	165/165	206/214	370/396	154/161	259/268	174/174	195/195	436/442	206/206	178/180	220/220	288/290	211/211	249/249	182/186	225/225	171/171	304/304	261/273	255/255	234/234	178/183	175/175	172/172
153/153	129/131	241/242	100/103	278/281	146/146	138/138	170/170	130/136	228/234	162/165	206/211	367/370	152/154/161	259/265	174/174	195/195	436/442	203/206	178/180	220/220	288/290	211/211	246/249	182/186	230/230	171/171	299/304	259/261/264	253/255	230/230	178/178	175/175	172/178
151/153	131/131	241/242	100/103	278/278	156/156	141/141	164/170	136/136	228/234	165/177	206/214	364/396	154/161	259/268	171/174	195/196	436/436	206/206	178/180	220/220	288/288	211/211	249/249	186/186	225/225	171/171	304/304	259/273	255/255	234/234	178/183	175/175	172/172
144/153	129/131	241/242	100/100	278/278	146/156	138/141	153/164	130/136	228/234	165/177	206/214	364/370	161/161	259/268	171/174	196/196	436/436	206/206	178/180	220/220	288/288	211/211	249/249	186/186	225/225	171/171	304/304	259/273	255/255	234/234	178/183	175/175	172/172
151/153	129/131	241/242	103/103	278/281	146/156	141/141	153/170	130/136	228/234	162/165	206/211	364/367	161/161	259/265	174/174	195/196	436/442	203/214	178/178	217/223	288/290	211/211	246/254	182/186	225/230	171/171	299/304	259/261	255/255	230/234	178/183	175/175	172/172
151/153	131/131	242/242	103/103	278/278	156/156	138/141	153/164	136/136	228/228	165/165	211/214	364/364	161/161	265/268	171/174	196/196	436/436	206/214	178/178	220/223	288/288	211/211	249/254	186/186	225/225	171/171	304/304	273/273	255/255	234/234	183/183	175/175	172/172
144/151	129/131	241/241	100/103	278/278	146/156	138/141	153/170	136/136	234/234	165/177	206/214	396/396	161/161	259/268	171/174	195/195	436/436	206/214	178/178	220/220	288/288	211/211	249/249	186/186	225/225	171/171	304/304	259/261	255/255	234/234	183/183	175/175	172/172
144/151	131/131	241/242	103/103	278/281	146/156	138/141	164/170	130/136	234/234	165/177	206/211	364/370	154/161	259/265	171/171	196/196	436/442	206/214	178/178	220/234	288/290	211/211	249/254	182/186	225/225	171/171	304/304	259/261	255/255	234/234	178/183	175/175	172/172
151/151	131/131	241/241	100/100	278/278	146/156	138/141	153/164	130/136	234/234	165/165	206/211	367/396	161/161	259/265	171/174	195/195	436/442	206/214	178/178	220/220	288/290	211/211	246/254	186/186	225/225	171/171	304/304	261/261	255/255	234/234	178/183	168/175	172/172
144/153	129/131	241/242	103/103	278/281	156/156	141/141	164/170	130/130	234/234	165/165	206/206	364/364	161/161	259/259	171/174	196/196	442/442	206/206	178/180	220/223	290/290	211/211	246/249	182/186	225/225	171/171	304/304	261/261	255/255	234/234	178/183	175/175	172/172
151/151	129/131	241/242	100/103	278/281	156/156	141/141	164/170	136/136	228/234	165/165	206/211	364/367	161/161	259/265	171/174	195/196	436/442	206/214	178/178	220/234	288/288	211/211	249/254	182/186	225/225	171/171	304/304	261/273	255/255	234/234	178/183	168/175	172/172
151/153	129/131	241/242	100/103	278/281	146/156	141/141	164/170	130/136	228/234	165/165	206/211	364/367	154/161	259/265	174/174	195/196	436/442	206/206	178/178	220/223	288/290	211/211	246/249	182/186	225/225	171/171	304/304	261/273	255/255	234/234	178/183	175/175	172/172
151/153	129/131	241/241	103/103	278/278	156/156	141/141	153/170	130/136	234/234	165/165	206/211	367/396	161/161	259/265	171/174	195/195	436/442	206/206	178/178	220/220	288/290	211/211	249/254	186/186	225/225	171/171	304/304	261/261	253/255	234/234	183/183	175/178	172/172
151/151	131/131	241/241	100/100	278/278	146/156	138/141	153/164	130/136	234/234	165/165	206/211	367/396	161/161	259/265	171/174	195/195	436/442	206/214	178/178	220/220	288/290	211/211	246/254	186/186	225/225	171/171	304/304	261/261	255/255	234/234	178/183	168/175	172/172
151/151	131/131	242/242	103/103	278/278	156/156	141/141	170/170	130/133	225/234	162/162	206/211	364/379	161/161	259/265	171/174	195/195	436/442	203/206	178/178	223/234	288/290	211/211	246/249	182/186	225/230	171/171	299/304	259/273	253/255	230/234	183/183	175/175	172/178
153/153	129/131	242/242	103/103	278/281	146/146	138/138	170/170	130/130	228/234	162/165	nd.	367/370	161/161	259/265	174/174	195/195	436/442	206/214	180/180	220/220	290/290	211/211	249/254	182/186	225/230	171/171	299/304	259/261	255/255	230/234	178/178	171/175	172/178
151/151	129/131	242/242	103/103	278/281	146/156	138/141	170/170	130/136	228/234	162/165	206/211	364/367	161/161	259/265	174/174	195/196	436/442	206/214	178/178	220/234	288/290	211/211	249/254	182/186	225/225	171/171	299/304	261/264	255/255	234/234	178/183	175/175	172/172
151/151	129/131	241/242	100/103	278/278	156/156	141/141	164/170	130/136	234/234	165/165	211/211	364/367	154/161	265/265	171/174	195/195	436/436	206/206	178/178	217/234	288/290	211/211	249/249	186/186	225/225	171/171	304/304	261/261	253/255	234/234	183/183	175/175	172/172
151/153	129/131	242/242	103/103	278/281	146/156	138/141	170/170	130/130	225/234	162/162	206/206	364/379	152/161	259/259	174/174	195/196	442/442	203/214	178/180	220/223	290/290	211/211	246/254	182/182	218/230	171/171	304/304	259/273	253/255	230/230	178/183	175/175	172/178
144/151	129/131	242/242	103/103	278/278	146/156	138/141	164/164	130/136	228/234	165/165	206/211	364/364	154/161	259/265	171/174	196/196	436/442	206/214	178/178	217/220	288/290	211/211	254/254	186/186	225/225	171/171	304/304	261/273	253/255	234/234	178/183	175/175	172/172
151/153	129/131	241/242	103/103	278/278	146/156	138/141	164/170	130/136	234/234	165/165	211/211	364/364	154/154	265/265	171/174	196/196	nd.	206/206	178/178	220/234	288/290	211/211	249/254	186/186	225/225	171/171	304/304	261/261	253/255	234/234	178/183	175/178	172/172
144/153	129/129	241/241	103/103	278/278	146/156	138/141	153/164	130/136	228/234	165/165	211/211	364/367	154/161	265/265	174/174	195/195	436/436	206/214	178/178	220/220	288/290	211/211	249/254	186/186	225/225	171/171	304/304	261/261	253/255	234/234	178/183	175/175	172/172
151/153	129/129	242/242	100/100	278/278	156/156	141/141	164/164	136/136	228/234	165/165	211/211	364/367	161/161	265/265	174/174	195/195	436/436	206/214	178/178	217/217	288/288	211/211	246/249	186/186	225/225	171/171	304/304	261/273	255/255	234/234	183/183	175/175	172/172
151/157	129/129	242/242	103/103	278/278	146/156	141/141	170/170	130/130	228/234	162/165	nd.	367/370	152/161	259/265	174/174	195/195	436/442	203/214	178/180	217/220	288/288	211/211	246/254	182/186	225/230	171/171	299/304	259/261	253/255	230/230	178/183	175/175	172/178
151/151	129/131	241/242	103/103	278/278	146/156	138/141	153/164	130/136	228/234	165/165	211/211	367/396	161/161	265/265	171/174	195/195	436/436	206/214	178/178	220/220	288/290	211/211	249/249	186/186	225/225	171/171	304/304	261/273	253/255	234/234	178/183	168/175	172/172
153/153	131/131	241/241	100/103	278/278	156/156	141/141	170/170	130/136	234/234	165/177	206/214	364/367	154/154	259/268	171/174	195/196	436/442	206/206	178/180	220/220	288/290	211/211	249/249	186/186	225/225	171/171	304/304	259/261	253/255	234/234	178/183	175/175	172/172
151/151	129/129	242/242	100/100	278/281	146/156	138/141	164/170	136/136	228/234	165/165	206/211	364/364	161/161	259/265	174/174	196/196	436/442	206/214	178/178	220/234	288/288	211/211	249/254	182/186	225/225	171/171	304/304	261/273	255/255	234/234	178/183	171/175	172/172
136/153	129/131	242/244	103/103	278/278	146/153	147/147	170/170	130/136	228/228	159/162	200/211	364/367	152/154	254/265	171/174	195/195	421/436	197/214	172/180	202/220	290/293	211/211	246/254	186/186	219/230	170/171	302/304	253/264	255/255	230/230	178/178	168/175	169/172
151/153	129/131	241/241	103/103	278/278	156/156	138/141	170/170	130/136	234/234	165/165	211/211	364/396	154/161	265/265	171/174	195/196	436/436	206/206	178/178	220/234	288/290	211/211	249/254	186/186	225/225	171/171	299/304	261/261	253/255	234/234	178/183	175/175	172/172
151/151	129/131	242/242	103/103	278/278	146/156	138/141	164/170	130/136	234/234	165/165	206/211	364/364	154/161	259/265	171/174	196/196	436/442	206/206	178/178	220/234	288/290	211/211	254/254	186/186	225/225	171/171	299/304	261/261	253/255	234/234	178/183	175/178	172/172
151/153	129/131	241/241	103/103	278/278	146/156	138/141	164/170	130/136	234/234	165/165	211/211	364/367	154/161	265/265	171/174	195/196	436/442	206/206	178/178	234/234	288/290	211/211	249/254	186/186	225/225	171/171	299/304	261/261	253/255	234/234	178/183	171/175	172/172
151/153	129/129	241/242	100/100	278/281	146/156	138/141	164/170	130/136	234/234	162/165	206/211	364/364	161/161	259/265	174/174	196/196	436/442	203/206	178/178	220/234	288/288	211/211	249/249	182/186	225/225	171/171	302/304	261/273	255/255	234/234	178/183	171/175	172/172
151/151	131/131	241/242	100/100	278/278	146/156	138/141	164/170	130/136	225/234	165/165	206/211	364/396	154/161	259/265	174/174	195/196	436/442	206/214	178/178	220/220	288/290	211/211	249/254	182/186	225/225	171/171	304/304	261/261	253/255	234/234	178/183	175/175	172/172
151/151	129/131	241/242	100/103	278/278	156/156	141/141	164/164	136/136	228/228	165/165	211/211	367/396	161/161	265/265	171/174	195/195	436/436	214/214	178/178	217/234	288/288	211/211	249/249	186/186	225/225	171/171	299/304	261/273	255/255	234/234	183/183	175/175	172/172
144/151	129/131	241/242	103/103	278/278	146/156	138/141	164/170	130/136	228/228	165/165	211/211	364/367	161/161	265/265	174/174	195/196	436/436	206/206	178/178	220/220	288/290	211/211	249/254	186/186	225/225	171/171	304/304	261/273	255/255	234/234	178/183	175/175	172/172
144/153	131/131	241/241	100/100	278/281	146/156	138/141	153/170	136/136	234/234	165/177	206/211	364/396	154/161	259/265	171/171	195/196	436/442	206/206	178/178	220/220	288/288	211/211	249/249	182/186	225/225	171/171	304/304	259/261	253/253	234/234	183/183	175/175	172/172
151/153	129/131	241/241	100/103	278/278	146/156	138/141	164/170	136/136	234/234	165/177	211/214	364/367	161/161	265/268	171/174	195/196	436/436	206/206	178/178	220/220	288/288	211/211	249/254	186/186	225/225	171/171	304/304	259/261	255/255	234/234	178/183	175/175	172/172
151/153	131/131	242/243	100/100	278/281	146/146	138/138	164/164	130/130	228/228	162/165	206/211	364/376	152/154	259/265	174/174	194/196	438/442	203/206	178/180	214/226	288/290	211/211	246/254	182/186	225/230	171/171	304/304	264/266	253/255	230/234	178/178	168/178	172/172
151/153	129/129	241/242	100/100	278/281	146/156	138/141	164/170	130/136	234/234	162/177	206/211	364/364	154/161	259/265	174/174	196/196	436/442	206/206	178/178	220/234	288/290	211/211	249/254	182/186	225/225	171/171	304/304	261/273	253/255	234/234	178/183	171/175	172/172
151/151	131/131	242/242	100/103	278/281	146/156	138/141	170/170	136/136	234/234	165/165	206/211	367/396	154/161	259/265	171/174	195/195	436/442	206/206	178/178	220/220	288/288	211/211	249/254	182/186	225/225	171/171	299/304	261/261	253/255	234/234	178/183	175/175	172/172
151/153	129/131	242/242	103/103	278/278	146/146	138/138	170/170	130/136	228/234	162/165	211/211	364/367	152/161	265/265	174/174	195/196	436/436	206/206	180/180	220/234	288/288	211/211	249/254	186/186	230/230	171/171	302/304	264/273	253/255	230/234	178/178	175/175	172/178
151/153	129/131	241/242	103/103	278/278	146/156	141/141	164/170	130/136	228/234	162/165	211/211	364/370	154/161	265/265	171/174	196/196	436/436	206/206	178/178	220/234	288/290	211/211	246/249	186/186	225/225	171/171	304/304	261/264	253/255	234/234	178/183	175/175	172/172
151/151	131/131	241/241	100/100	278/278	146/156	138/141	164/170	130/136	225/234	165/177	206/211	364/396	154/154	259/265	171/174	195/196	436/442	206/206	178/178	220/220	288/290	211/211	249/254	186/186	225/225	171/171	304/304	259/261	253/255	234/234	178/183	178/178	172/172
151/153	129/131	242/242	100/100	278/278	156/156	141/141	164/164	130/136	234/234	165/165	211/211	364/367	161/161	265/265	171/174	195/196	436/436	206/214	178/178	217/220	288/290	211/211	249/249	186/186	225/225	171/171	304/304	261/261	255/255	234/234	183/183	175/175	172/172
151/153	129/129	241/242	100/103	278/278	146/156	138/141	164/164	130/136	234/234	165/165	206/211	364/367	161/161	259/265	171/174	195/196	436/442	206/206	178/178	217/220	288/290	211/211	246/254	186/186	225/225	171/171	304/304	261/261	255/255	234/234	178/183	175/175	172/172
151/151	129/131	241/242	100/100	278/278	146/146	138/138	164/170	130/136	234/234	165/165	211/211	367/367	161/161	265/265	171/174	195/195	436/436	206/214	178/178	217/220	288/290	211/211	254/254	186/186	225/225	171/171	304/304	261/261	255/255	234/234	178/178	175/175	172/172
151/153	129/131	241/242	100/100	278/278	146/156	138/138	164/170	130/136	234/234	165/165	211/211	364/367	152/161	265/265	171/174	195/196	436/436	203/206	178/180	223/234	288/290	211/211	246/249	186/186	225/225	171/171	299/304	261/261	253/255	230/234	178/183	168/171	172/172
151/153	129/131	241/241	100/100	278/278	156/156	141/141	170/170	130/136	234/234	165/165	206/214	364/367	154/154	259/268	171/174	195/196	436/442	206/206	178/178	220/220	288/290	211/211	249/249	186/186	225/225	171/171	304/304	259/261	253/253	234/234	183/183	175/175	172/172
151/151	129/131	242/242	103/103	278/278	146/156	138/141	164/170	136/136	228/234	165/177	211/211	364/364	161/161	265/265	171/174	196/196	436/436	206/206	178/178	220/234	288/288	211/211	249/254	186/186	225/225	171/171	299/304	259/261	255/255	234/234	178/183	175/175	172/172
153/157	129/129	242/242	103/103	278/281	146/146	138/138	nd.	122/130	225/225	162/162	206/211	370/370	152/152	259/265	174/174	195/195	442/442	203/206	180/180	217/220	290/290	211/211	246/246	182/182	230/230	171/171	299/307	259/264	253/253	230/230	178/178	175/184	178/178
151/153	129/131	241/242	103/103	278/278	146/156	138/141	170/170	130/136	228/234	165/165	211/214	364/364	161/161	265/268	174/174	196/196	436/436	206/206	178/178	220/234	288/290	211/211	246/254	186/186	225/225	171/171	299/304	261/273	255/255	234/234	178/178	171/175	172/172
144/151	129/131	241/242	100/100	278/281	146/156	138/141	153/170	136/136	234/234	165/165	206/206	364/396	154/161	259/259	171/174	195/196	436/442	206/206	178/178	220/234	288/288	211/211	249/254	182/186	225/225	171/171	304/304	261/261	255/255	234/234	178/183	175/175	172/172
144/153	129/131	241/242	100/103	278/278	146/156	138/138	153/170	136/136	234/234	165/165	211/211	364/367	161/161	265/265	171/174	195/195	436/436	206/214	178/178	217/234	288/288	211/211	254/254	186/186	225/225	171/171	304/304	261/261	255/255	234/234	178/183	171/175	172/172
144/151	129/131	241/241	103/103	278/278	156/156	141/141	164/164	133/136	228/228	165/165	211/211	364/396	161/161	265/265	171/174	195/195	436/436	206/214	178/178	217/220	288/288	211/211	249/254	186/186	225/225	171/171	304/304	261/273	255/255	234/234	178/183	175/175	172/172
151/153	129/129	241/242	103/103	278/278	156/156	138/141	164/170	130/136	234/234	165/165	211/211	364/396	161/161	265/265	174/174	195/196	436/436	206/206	178/178	220/220	288/290	211/211	249/254	186/186	225/225	171/171	304/304	261/261	255/255	234/234	178/183	175/175	172/172
151/151	129/129	241/242	100/103	278/281	146/156	138/141	164/164	130/136	234/234	165/165	206/211	364/367	161/161	259/265	171/174	195/195	436/442	206/214	178/178	220/220	288/290	211/211	249/249	182/186	225/225	171/171	304/304	261/261	253/255	234/234	183/183	171/175	172/172
144/151	131/131	241/241	100/100	278/278	146/156	138/141	164/164	136/136	228/234	165/165	211/214	367/396	154/161	265/268	171/171	195/195	436/442	206/206	178/178	220/234	288/288	211/211	249/249	186/186	225/225	171/171	299/304	261/273	253/255	234/234	178/183	171/175	172/172
151/153	131/131	241/242	103/103	278/281	156/156	138/141	164/164	130/136	228/234	165/165	206/211	364/364	161/161	259/265	171/174	196/196	436/442	206/214	178/178	229/234	288/290	211/211	249/254	182/186	225/225	171/171	304/304	261/273	255/255	234/234	178/183	171/175	172/172
151/151	129/131	241/242	100/100	278/278	156/162	141/141	164/170	136/136	228/228	165/165	211/211	364/396	154/161	265/265	171/174	195/196	436/436	206/206	178/178	220/234	288/288	211/211	246/254	186/186	225/225	171/171	304/304	273/273	253/255	234/234	183/183	175/175	172/172
151/153	131/131	241/242	100/100	278/278	146/156	138/141	164/170	130/136	234/234	162/165	206/211	364/396	154/161	259/265	174/174	195/196	442/442	206/206	178/178	220/234	288/290	211/211	249/254	182/186	225/225	171/171	299/302	261/273	253/255	234/234	178/183	175/175	172/172
151/151	129/131	242/242	100/100	278/278	146/156	138/138	164/164	136/136	234/234	165/165	206/211	364/364	161/161	259/265	171/174	196/196	436/442	206/214	178/178	220/220	288/288	211/211	249/254	186/186	225/225	171/171	304/304	261/261	255/255	234/234	178/183	175/175	172/172
151/151	129/131	241/241	100/103	278/278	156/156	141/141	153/164	130/136	234/234	165/165	211/211	364/364	154/161	265/265	171/174	196/196	436/436	206/214	178/178	220/234	288/290	211/211	249/249	186/186	225/225	171/171	299/304	261/261	253/255	234/234	183/183	171/175	172/172
151/151	131/131	241/242	100/100	278/278	156/156	141/141	164/170	136/136	228/234	165/165	206/211	364/396	154/161	259/265	174/174	195/196	436/442	206/206	178/178	220/220	288/288	211/211	246/254	186/186	225/225	171/171	304/304	261/273	253/255	234/234	178/178	175/175	172/172
144/153	129/131	241/241	100/100	278/278	146/156	138/141	153/170	130/136	234/234	165/165	206/211	364/396	154/161	259/265	171/174	195/196	436/442	206/206	178/178	220/234	288/290	211/211	249/249	186/186	225/225	171/171	299/304	261/261	255/255	234/234	178/183	171/175	172/172
151/151	129/129	241/242	103/103	278/281	156/156	138/141	164/170	136/136	228/234	165/165	206/211	364/364	161/161	259/265	174/174	196/196	436/442	206/214	178/178	220/234	288/288	211/211	254/254	182/186	225/225	171/171	304/304	261/273	255/255	234/234	183/183	175/175	172/172
151/153	131/131	241/242	100/100	278/278	146/156	138/141	153/170	130/130	234/234	162/165	206/211	364/396	154/161	259/265	171/174	195/196	436/442	206/206	178/178	220/234	290/290	211/211	249/254	182/186	225/230	171/171	299/302	261/273	253/253	230/234	178/183	175/175	172/172

151/153	129/131	241/242	100/103	278/281	146/156	138/141	164/164	130/130	234/234	165/165	206/211	364/364	154/161	259/265	171/174	196/196	436/442	206/214	178/180	220/223	290/290	211/211	246/249	182/186	225/225	171/171	304/304	261/261	253/253	234/234	178/183	175/175	172/172
151/153	131/131	241/242	100/100	278/281	146/156	138/141	164/170	130/136	228/234	162/177	206/211	364/396	161/161	259/265	171/174	195/196	436/442	206/214	178/180	220/234	288/290	211/211	249/254	182/186	225/225	171/171	304/304	259/264	253/255	234/234	178/183	175/175	172/172
151/153	129/131	242/242	103/103	278/278	146/156	138/141	153/170	130/136	228/234	165/177	206/214	364/370	154/161	259/268	171/174	196/196	442/442	206/206	178/178	220/234	288/290	211/211	254/254	186/186	225/225	171/171	304/304	259/261	253/255	234/234	178/178	175/175	172/172
151/153	131/131	241/242	100/100	278/278	146/156	138/141	153/170	136/136	225/228	165/177	211/211	364/396	154/161	265/265	171/171	195/196	436/436	206/206	178/178	220/220	288/288	211/211	249/254	186/186	225/225	171/171	304/304	259/273	253/255	234/234	178/183	171/175	172/172
144/151	129/129	241/242	100/100	278/278	146/156	138/141	153/170	130/136	234/234	165/165	211/211	364/364	154/161	265/265	171/174	196/196	436/442	203/206	178/178	220/234	288/288	211/211	249/249	186/186	225/225	171/171	299/304	261/261	255/255	234/234	178/183	175/175	172/172
151/153	129/129	241/241	100/103	278/278	156/156	141/141	164/164	130/136	228/234	165/165	211/214	367/367	154/161	265/268	171/174	195/195	436/436	206/206	178/178	220/234	288/290	211/211	249/249	186/186	225/225	171/171	304/304	261/273	253/255	234/234	183/183	171/175	172/172
144/153	129/131	241/242	103/103	278/278	146/156	138/141	164/164	130/136	228/234	165/177	211/211	364/396	154/161	265/265	171/174	195/196	436/436	206/206	178/178	217/220	288/290	211/211	249/254	186/186	225/225	171/171	304/304	259/273	253/255	234/234	178/183	175/175	172/172
151/153	129/131	241/242	100/103	278/278	146/156	138/138	164/164	130/136	228/234	165/165	206/211	364/367	154/161	259/265	174/174	195/195	436/442	206/206	178/178	217/220	288/290	211/211	249/249	186/186	225/225	171/171	304/304	261/273	253/255	234/234	178/178	175/175	172/172
151/151	129/131	241/241	100/103	278/278	156/156	141/141	164/164	130/136	234/234	165/165	206/211	364/364	161/161	259/265	171/174	196/196	436/442	206/206	178/178	217/234	288/290	211/211	249/249	186/186	225/225	171/171	304/304	261/261	255/255	234/234	183/183	175/178	172/172
151/153	129/131	241/241	100/103	278/278	146/156	138/141	164/170	136/136	225/234	165/177	211/211	364/367	154/161	265/265	171/174	195/196	436/436	206/206	178/180	220/234	288/288	211/211	249/254	186/186	225/225	171/171	304/304	259/261	253/255	234/234	178/183	171/175	172/172
144/153	129/129	241/241	100/100	278/278	146/156	138/141	164/170	130/136	228/234	165/165	211/211	364/396	161/161	265/265	174/174	195/196	436/442	206/206	178/178	220/234	288/290	211/211	249/254	186/186	225/225	171/171	304/304	261/273	255/255	234/234	178/183	175/175	172/172
151/153	129/131	241/241	103/103	278/278	156/156	138/141	164/170	136/136	234/234	165/165	211/214	364/396	154/161	265/268	174/174	195/196	436/436	206/206	178/178	220/234	288/288	211/211	249/254	186/186	225/225	171/171	299/304	259/261	253/255	234/234	178/183	171/175	172/172
144/153	129/131	241/242	103/103	278/278	146/156	138/141	164/170	130/136	234/234	165/165	206/214	364/396	161/161	259/268	171/174	195/196	436/442	206/206	178/178	220/234	288/290	211/211	249/254	186/186	225/225	171/171	304/304	261/261	255/255	234/234	178/183	175/178	172/172
151/153	131/131	242/242	100/100	278/281	146/156	138/141	170/170	130/130	225/234	162/177	206/211	364/396	152/154	259/265	174/174	195/196	436/442	203/206	178/178	220/223	290/290	211/211	249/249	182/186	225/225	171/171	302/304	259/273	253/253	234/234	178/183	175/175	172/178
151/153	131/131	241/242	100/100	278/281	156/156	141/141	164/164	130/136	225/234	162/165	206/211	364/364	152/161	259/265	171/174	196/196	436/442	203/206	178/178	220/223	288/290	211/211	249/254	182/186	225/225	171/171	304/304	259/261	253/255	234/234	178/183	175/175	172/178
151/151	131/131	241/242	103/103	278/281	146/156	138/141	164/170	130/136	234/234	165/165	206/206	364/396	154/161	259/259	174/174	195/196	442/442	206/206	178/178	220/234	288/290	211/211	249/249	182/182	225/225	171/171	299/304	261/261	253/255	234/234	178/183	171/175	172/172
144/151	129/131	241/242	100/100	278/281	146/146	138/138	153/170	130/136	234/234	165/177	206/211	364/396	154/161	259/265	171/174	195/196	436/442	206/214	178/178	234/234	288/290	211/211	249/254	182/186	225/225	171/171	299/304	259/261	253/255	234/234	178/183	171/175	172/172
151/153	129/131	241/242	100/103	278/278	146/156	138/141	164/170	136/136	234/234	165/177	211/211	364/364	154/161	265/265	171/174	nd.	436/436	206/214	178/178	234/234	288/288	211/211	249/249	186/186	225/225	171/171	304/304	259/261	253/255	234/234	178/183	175/175	172/172
151/151	131/131	241/242	103/103	278/281	146/156	138/138	164/164	130/133	234/234	165/165	206/211	364/396	152/154	259/265	171/171	195/196	436/442	203/206	178/180	220/226	288/290	211/211	249/254	182/186	225/230	171/171	304/304	261/261	253/253	230/234	178/183	168/178	172/172
151/153	131/131	241/242	100/103	278/278	146/156	138/138	164/170	130/133	225/234	165/177	206/211	367/396	152/161	259/265	171/171	195/195	436/442	203/206	178/180	220/226	288/290	211/211	246/249	182/186	225/230	171/171	304/304	259/261	253/255	230/234	178/183	175/175	172/172
151/153	129/131	241/242	100/100	278/281	146/156	138/141	164/164	130/133	225/228	162/177	206/211	367/396	152/161	259/265	171/174	195/195	436/442	203/206	178/180	220/223	288/288	211/211	246/249	182/186	225/230	171/171	304/304	259/264	253/255	230/234	178/183	168/178	172/172
151/151	129/131	241/242	100/103	278/281	146/156	141/141	153/153	130/136	228/234	165/165	206/214	370/396	161/161	259/268	174/174	195/195	436/442	206/214	178/180	220/220	288/290	211/211	246/249	182/186	225/225	171/171	304/304	261/273	255/255	234/234	178/183	175/175	172/178
151/153	131/131	241/241	100/103	278/278	146/156	138/141	164/164	136/136	228/234	162/165	211/211	364/370	161/161	265/265	171/171	196/196	436/436	206/214	178/178	220/220	288/290	211/211	249/249	186/186	225/225	171/171	304/304	261/264	253/255	234/234	183/183	175/175	172/172
151/153	131/131	242/242	100/100	278/278	146/156	138/141	164/170	130/136	228/234	162/165	206/211	364/396	161/161	259/265	174/174	195/196	436/442	206/214	178/180	220/234	288/290	211/211	249/254	182/186	225/225	171/171	299/304	261/264	255/255	234/234	178/183	175/175	172/172
151/153	129/131	242/242	103/103	278/281	146/156	138/141	170/170	130/130	234/234	162/165	206/211	364/370	161/161	259/265	174/174	195/196	436/442	206/214	178/178	220/234	288/290	211/211	246/254	182/186	225/230	171/171	302/304	261/261	255/255	230/234	178/183	175/175	172/172
151/153	129/129	241/242	103/103	278/281	146/156	141/141	153/170	130/136	228/228	162/165	206/211	370/370	161/161	259/265	174/174	195/195	436/442	206/206	178/178	220/220	288/290	211/211	246/249	182/186	225/230	171/171	302/304	264/273	255/255	230/234	178/183	175/175	172/172
151/153	129/129	242/242	103/103	278/278	146/156	141/141	170/170	130/136	228/228	162/165	211/211	367/367	161/161	265/265	174/174	195/195	436/436	214/214	178/180	220/220	288/290	211/211	249/254	186/186	225/230	171/171	304/304	261/264	255/255	230/234	178/183	175/175	172/172
144/151	131/131	241/242	100/103	278/278	146/156	141/141	153/153	130/136	228/228	162/165	211/214	370/396	161/161	265/268	171/174	195/195	436/436	206/206	178/180	220/220	288/288	211/211	246/249	186/186	225/225	171/171	304/304	264/273	255/255	234/234	178/183	175/175	172/178
151/157	129/129	242/242	103/103	278/278	146/146	nd.	170/170	130/130	225/228	162/162	206/211	367/370	152/161	259/265	174/174	195/195	436/442	203/206	180/180	217/220	288/290	211/211	246/254	182/182	230/230	171/171	299/304	264/264	253/255	230/230	178/178	175/175	172/178
151/153	131/131	242/242	100/100	278/281	146/156	138/141	170/170	130/136	228/234	162/165	206/211	367/370	152/161	259/265	174/174	195/195	436/442	206/214	178/180	220/220	288/290	211/211	249/254	182/186	225/225	171/171	304/304	261/264	253/255	234/234	178/183	175/178	172/178
151/153	129/131	242/242	100/103	278/278	146/156	138/141	164/170	130/136	234/234	165/165	206/211	364/367	161/161	259/265	171/174	195/196	436/442	206/214	178/178	217/234	288/290	211/211	249/254	186/186	225/225	171/171	304/304	261/261	255/255	234/234	178/183	175/178	172/172
151/151	129/131	241/242	100/103	278/278	146/156	138/141	164/170	136/136	225/234	165/177	206/211	364/396	161/161	259/265	171/174	195/196	436/442	206/206	178/178	220/234	288/288	211/211	249/254	182/186	225/225	171/171	299/304	259/261	255/255	234/234	178/183	175/178	172/172
151/153	129/129	242/242	100/103	278/278	156/156	141/141	164/164	130/136	228/234	165/165	211/211	367/367	154/161	265/265	174/174	195/195	436/436	206/214	178/178	217/220	288/290	211/211	249/249	186/186	225/225	171/171	304/304	261/261	253/255	234/234	183/183	175/175	172/172
151/151	129/131	242/242	100/100	278/278	156/156	141/141	164/170	130/136	228/234	165/165	211/211	364/367	161/161	265/265	171/174	195/195	436/436	206/214	178/178	217/220	288/290	211/211	249/254	186/186	225/225	171/171	304/304	261/273	255/255	234/234	183/183	175/175	172/172
151/151	129/131	241/242	103/103	278/278	146/156	138/141	170/170	136/136	234/234	165/165	211/211	364/367	154/154	265/265	171/174	195/196	436/436	206/206	178/178	220/234	288/288	211/211	254/254	186/186	225/225	171/171	299/299	261/261	253/255	234/234	178/183	171/175	172/172
151/153	129/131	242/242	100/100	278/278	146/156	138/141	164/170	130/130	234/234	165/165	211/211	364/367	154/161	265/265	174/174	195/196	436/436	206/206	178/178	220/234	290/290	211/211	249/254	186/186	225/225	171/171	304/304	261/261	253/255	234/234	178/183	175/175	172/172
151/151	131/131	241/242	100/100	278/278	146/156	138/141	164/170	133/136	234/234	165/165	211/211	364/396	161/161	265/265	171/174	195/196	436/436	206/206	178/178	220/220	288/288	211/211	249/249	186/186	225/225	171/171	304/304	261/261	253/255	234/234	178/183	175/175	172/172
151/151	129/131	242/242	100/103	278/281	146/156	138/141	164/170	130/136	234/234	165/165	206/211	364/396	161/161	259/265	174/174	195/196	436/442	206/206	178/178	220/220	288/288	211/211	249/254	182/186	225/225	171/171	304/304	261/261	255/255	234/234	178/183	175/175	172/172
151/153	131/131	241/241	100/100	278/278	146/156	138/141	164/164	130/130	225/234	165/165	206/211	364/396	161/161	259/265	171/174	195/196	436/442	206/206	178/178	220/220	290/290	211/211	249/254	182/186	225/225	171/171	304/304	261/261	253/255	234/234	178/183	175/175	172/172
153/153	129/129	242/242	103/103	278/281	146/146	138/138	170/170	130/130	228/234	162/165	206/211	364/370	152/154	259/265	174/174	195/196	436/442	203/206	178/180	220/234	290/290	211/211	246/254	182/186	225/230	171/171	299/304	259/261	253/253	230/234	178/178	175/175	172/178
151/153	131/131	241/241	100/100	278/278	146/156	138/141	164/170	136/136	228/234	165/177	211/214	364/396	161/161	265/268	171/171	195/196	436/436	206/214	178/178	217/220	288/288	211/211	249/254	186/186	225/225	171/171	299/304	259/261	253/255	234/234	183/183	175/175	172/172
151/153	129/131	241/241	100/100	278/278	156/156	141/141	153/170	136/136	234/234	165/165	206/211	367/396	154/161	259/265	174/174	195/195	436/442	206/206	178/180	220/220	288/288	211/211	246/249	186/186	225/225	171/171	304/304	261/261	253/255	234/234	178/183	175/175	172/172
153/153	129/129	242/242	103/103	278/281	146/146	nd.	164/164	130/136	228/228	159/162	206/206	367/370	152/154	259/259	171/174	195/195	442/442	203/206	178/180	202/220	290/293	211/211	246/254	182/186	225/230	171/171	299/304	253/259	253/255	230/234	178/183	175/175	172/178
151/153	129/129	242/242	103/103	278/281	146/146	nd.	173/173	130/136	228/234	162/165	206/211	367/370	152/154	259/265	174/174	195/195	436/442	203/206	180/180	220/220	290/293	211/211	249/254	182/186	225/230	170/171	299/304	259/261	253/255	230/234	178/178	175/175	169/178
151/153	129/131	241/241	103/103	278/281	146/156	138/141	164/170	136/136	228/234	165/177	206/211	364/396	154/161	259/265	171/174	195/196	436/436	206/214	178/178	220/234	288/288	211/211	249/254	186/186	225/225	171/171	304/304	259/273	253/255	234/234	178/183	175/175	172/172
151/151	nd.	241/242	100/100	278/278	146/156	138/141	164/164	130/133	225/234	165/165	211/211	364/396	154/161	265/265	174/174	195/196	436/436	206/206	178/178	220/234	288/290	211/211	249/254	186/186	225/225	171/171	304/304	261/261	253/253	234/234	178/183	175/175	172/172
151/153	129/129	242/242	100/100	278/278	146/156	138/141	164/170	130/136	228/234	165/165	211/211	364/396	154/154	265/265	174/174	195/196	436/436	197/206	178/180	220/234	290/293	211/211	249/249	186/186	225/225	171/171	304/304	253/261	253/253	234/234	178/183	168/175	172/172
151/153	129/131	241/242	100/100	278/281	146/156	138/141	164/164	130/133	228/234	162/165	206/206	364/367	154/161	259/259	171/174	195/196	442/442	206/214	178/178	220/226	288/290	211/211	249/254	182/186	225/225	171/171	304/304	261/264	253/255	234/234	178/183	175/175	172/172
151/153	129/131	241/242	103/103	278/278	156/156	141/141	164/170	130/136	234/234	165/177	211/214	364/364	154/154	265/268	171/174	196/196	436/442	206/206	178/180	220/220	288/290	211/211	249/254	186/186	225/225	171/171	304/304	259/259	253/255	234/234	178/183	175/175	172/172
151/153	129/131	241/242	103/103	278/278	146/146	138/138	170/170	136/136	228/234	162/165	206/211	364/367	152/154	259/265	171/174	195/196	436/442	203/206	178/180	223/234	288/288	211/211	246/254	182/186	225/230	171/171	299/304	261/261	253/255	230/234	178/178	175/175	172/181
151/153	129/131	241/241	100/100	278/278	146/156	138/141	164/170	130/136	234/234	165/165	211/214	364/364	154/161	265/268	171/174	196/196	436/436	206/206	178/178	220/234	288/290	211/211	nd.	186/186	225/225	171/171	304/304	261/261	253/255	234/234	178/183	175/175	172/172
151/151	131/131	241/241	100/100	278/281	156/156	141/141	164/170	136/136	234/234	165/177	206/211	364/396	161/161	259/265	171/174	195/196	436/442	206/206	178/178	220/220	288/288	211/211	249/254	182/186	225/225	171/171	304/304	nd.	253/255	234/234	178/183	175/175	172/172
151/151	131/131	241/242	100/100	278/278	156/156	141/141	164/170	130/136	234/234	165/165	211/211	364/396	161/161	265/265	171/171	195/196	436/436	206/206	178/178	220/234	288/290	211/211	249/249	186/186	225/225	171/171	304/304	261/261	255/255	234/234	183/183	175/178	172/172

198	199	200	201	202	203	204	205	206	207	208	209	210	211	212	213	214	215	216	217	218	219	220	221	222	223	224	225	226	227	228	229	230	231
*	*	*	*	*	*	*	**	*	*	*	**	*		*	*	*	*	*	*	*	*	*	*		*	*			*	*		*	
TSRF195	TSRF198	TSRF204	TSRF208	TSRF211	TSRF215	TSRF218	TSRF206	TSRF225	TSRF229	TSRF230	TSRF234	TSRF237	TSRF238	TSRN214	TSRP06	TSRP07	CiBE0246	CiBE0447	CiBE1644	CiBE2165	CiBE2227	CiBE4265	CX0004	CX0010	CX0035	CX2004	CX2018	CX2021	CX2040	CX3001	CX3040	CX4005	CX4008
1	0	0	0	2	0	0	0	0	0	1	1	3	0	0	0	0	0	0	1	0	1	0	0	0	2	0	0	0	0	0	1	0	0
163/163	165/165	203/206	206/212	229/234	238/243	196/202	259/262	147/149	281/283	212/224	184/195	177/183	177/177	143/143	202/202	159/159	233/236	309/328	366/378	281/298	160/170	241/249	261/273	216/228	175/184	196/206	174/181	149/149	106/110	417/426	452/455	368/368	382/390
163/163	165/165	203/203	206/210	234/234	238/238	196/202	259/259	147/147	278/283	224/224	195/195	177/183	177/177	134/143	202/204	159/159	236/236	312/325	366/378	281/281	160/160	241/249	261/273	219/219	184/184	196/199	168/177	147/151	106/110	420/420	464/464	368/368	386/390
163/169	165/165	203/206	206/212	229/234	238/243	196/202	262/268	147/149	278/283	212/224	184/195	177/177	177/177	143/143	202/204	159/159	233/236	312/325	366/378	281/281	160/170	241/249	273/273	216/219	175/184	199/206	174/175	149/151	100/110	420/426	452/455	368/372	390/390
163/163	165/165	203/203	206/212	234/234	238/238	202/202	259/259	147/147	278/281	224/224	195/195	183/183	177/177	143/143	204/207	159/187	236/236	309/312	366/366	281/281	160/160	249/249	261/273	216/216	184/184	199/199	185/185	147/151	100/106	426/426	452/464	368/368	390/390
163/169	165/165	203/206	206/206	229/229	243/243	196/196	262/262	149/149	278/283	212/224	184/195	183/183	177/177	143/143	202/202	159/187	233/233	325/328	366/381	285/298	160/170	249/253	273/273	228/228	175/175	202/206	170/174	145/147	110/114	417/423	455/458	368/372	382/390
163/169	165/165	203/203	206/212	229/234	238/243	202/202	259/259	147/147	283/286	212/212	184/184	177/183	177/177	132/143	204/204	159/159	236/236	316/325	366/387	285/285	162/176	242/253	261/273	216/219	184/184	199/217	141/157	139/147	102/102	417/430	463/463	368/368	390/390
163/169	165/165	203/206	206/212	229/234	238/243	196/196	262/268	149/149	283/283	212/224	184/195	177/183	177/177	143/143	202/202	159/187	233/236	325/328	366/378	281/298	160/170	249/249	273/273	219/228	175/184	196/206	174/181	147/149	110/118	420/423	455/464	368/372	382/390
163/163	165/165	203/206	206/212	223/229	238/243	196/202	259/268	147/149	283/286	212/224	184/195	183/183	177/177	132/143	202/204	159/187	233/233	312/319	366/381	287/298	160/167	249/253	273/273	216/228	175/184	202/206	170/174	145/147	118/126	420/423	455/464	368/368	386/390
163/163	165/165	203/203	206/206	229/229	243/243	196/196	262/262	149/149	283/283	212/212	184/184	183/183	177/177	140/143	202/204	159/187	233/233	309/328	366/384	287/287	160/170	249/249	273/276	219/219	175/175	202/206	174/174	145/147	110/112	417/417	457/464	368/368	382/390
163/163	165/165	203/203	206/206	229/229	243/243	196/196	262/262	149/149	283/283	212/212	184/184	183/183	177/177	140/143	202/204	159/187	233/233	309/328	366/384	287/287	160/170	249/249	273/276	219/219	175/175	202/206	174/174	145/147	110/112	417/417	457/464	368/368	382/390
163/163	165/165	203/203	206/206	229/229	243/243	196/196	262/262	149/149	283/283	212/212	184/184	183/183	177/177	140/143	202/204	159/187	233/233	309/328	366/384	287/287	160/170	249/249	273/276	219/219	175/175	202/206	174/174	145/147	110/112	417/417	457/464	368/368	382/390
163/163	165/165	203/203	206/206	229/229	243/243	196/196	262/262	149/149	283/283	212/212	184/184	183/183	177/177	140/143	202/204	159/187	233/233	309/328	366/384	287/287	160/170	249/249	273/276	219/219	175/175	202/206	174/174	145/147	110/112	417/417	457/464	368/368	382/390
169/169	165/165	203/206	206/206	229/229	243/243	196/196	259/262	149/149	283/283	212/212	184/184	183/183	177/177	143/143	202/202	159/159	233/233	309/322	366/378	298/298	164/170	249/249	273/276	219/219	175/175	206/206	174/176	147/147	108/110	417/426	455/455	368/368	382/382
163/169	165/165	203/206	206/206	229/229	243/243	196/196	259/262	149/149	283/283	212/224	184/195	183/183	177/177	140/143	202/204	159/187	233/233	309/328	366/384	281/287	160/170	249/249	273/276	228/228	175/175	199/206	157/174	145/147	110/112	417/423	455/457	368/372	382/382
158/163	165/165	203/206	206/212	229/234	238/243	196/202	259/259	147/149	278/283	212/224	184/195	177/183	177/177	134/143	202/204	159/159	233/236	316/319	378/378	281/285	160/170	241/257	273/273	216/228	175/184	199/212	157/174	147/149	108/112	420/420	457/463	368/368	382/390
169/169	146/165	203/206	206/206	229/229	243/243	196/196	262/262	149/149	283/283	212/212	184/184	183/183	177/177	140/143	202/204	159/187	233/233	309/309	366/366	287/294	160/170	249/249	273/276	219/228	175/175	206/208	168/174	145/147	110/112	423/423	455/455	372/372	382/382
169/169	146/165	203/206	206/206	229/229	243/243	196/196	262/262	149/149	283/283	212/212	184/184	183/183	177/177	140/143	202/204	159/187	233/233	309/309	366/366	287/294	160/170	249/249	273/276	219/228	175/175	206/208	168/174	145/147	110/112	423/423	455/455	372/372	382/382
163/163	165/165	203/203	212/212	234/234	238/238	202/202	259/259	147/147	278/278	224/224	195/195	177/177	177/177	143/143	204/204	187/187	236/236	309/309	366/378	281/285	160/160	230/249	273/273	226/226	184/184	199/203	175/175	149/149	100/118	417/426	452/452	368/368	382/390
158/169	165/165	203/206	206/212	229/229	243/243	196/202	259/262	149/149	278/283	212/212	184/184	171/183	177/177	125/143	202/202	159/159	233/233	319/325	378/378	298/298	167/170	249/253	273/273	219/228	175/175	202/212	174/175	149/150	110/126	423/426	457/458	368/372	390/390
158/163	165/165	203/209	206/206	229/229	238/243	196/196	259/259	149/149	283/283	212/218	184/190	171/183	177/177	143/146	202/204	159/159	233/236	309/319	366/366	285/298	160/170	249/253	261/273	219/219	175/184	199/212	157/174	147/150	102/108	417/420	455/455	368/368	382/382
163/163	165/165	203/206	206/212	229/234	238/243	196/202	262/268	149/149	278/283	212/224	184/195	183/183	177/177	143/143	202/204	187/187	233/233	312/325	366/366	281/298	160/170	241/249	273/273	216/228	175/184	199/206	174/175	145/147	110/110	420/423	452/458	368/368	390/402
169/169	165/165	206/206	206/206	229/229	243/243	196/196	262/262	149/149	283/283	212/212	184/184	183/183	177/177	140/143	202/204	159/159	233/233	309/309	366/378	285/294	160/170	249/249	273/276	219/228	175/175	206/214	168/174	147/147	112/114	423/423	455/455	368/368	382/382
163/169	165/165	206/209	206/206	229/229	238/243	196/196	259/262	147/149	283/283	212/224	184/195	171/183	177/177	125/143	202/202	159/159	233/233	319/325	366/369	287/298	164/167	249/249	273/279	219/228	175/184	206/214	170/174	149/151	100/126	423/426	455/457	368/368	382/390
163/163	165/165	203/206	206/206	229/234	238/238	196/202	259/262	147/147	278/283	212/224	184/195	177/183	177/177	143/143	202/204	159/187	236/236	309/328	366/378	285/287	160/170	249/249	273/273	216/228	184/184	199/199	157/168	147/149	106/110	423/426	452/464	368/368	382/390
163/163	165/165	203/206	206/206	229/234	238/238	196/202	259/262	147/147	278/283	212/224	184/195	177/183	177/177	143/143	202/204	159/187	236/236	309/328	366/378	285/287	160/170	249/249	273/273	216/228	184/184	199/199	157/168	147/149	106/110	423/426	452/464	368/368	382/390
163/163	165/165	203/206	206/206	229/234	238/238	196/202	259/262	147/147	278/283	212/224	184/195	177/183	177/177	143/143	202/204	159/187	236/236	309/328	366/378	285/287	160/170	249/249	273/273	216/228	184/184	199/199	157/168	147/149	106/110	423/426	452/464	368/368	382/390
163/169	165/165	200/206	206/206	229/229	235/243	196/196	259/259	149/149	283/283	212/215	184/187	171/171	177/177	140/140	202/205	159/159	230/233	319/334	369/369	285/298	165/167	237/249	273/276	219/231	175/184	204/214	170/179	145/151	116/126	416/426	452/457	368/369	382/382
163/169	165/165	203/206	206/206	229/234	238/243	196/202	259/268	147/149	281/283	224/224	195/195	177/183	177/177	143/143	202/204	187/187	233/236	312/328	378/378	281/298	160/170	241/249	273/273	219/228	175/184	196/199	177/181	147/147	100/110	423/426	452/455	368/372	382/402
163/169	165/165	200/206	206/212	229/229	235/243	193/196	259/262	149/149	278/283	212/215	184/187	177/183	177/177	134/143	202/205	159/187	230/233	325/334	369/378	289/298	165/170	249/249	273/276	228/229	175/184	201/206	154/174	147/149	116/118	423/423	452/464	369/372	382/382
163/163	165/165	200/206	206/212	229/229	238/243	193/196	259/268	149/149	278/283	215/224	187/195	171/183	177/177	134/143	202/205	159/159	233/233	328/334	366/381	289/298	160/165	237/249	273/273	219/229	175/184	201/206	154/174	145/149	110/116	416/423	452/455	368/368	382/382
169/169	146/165	206/206	206/206	229/229	238/243	196/196	259/259	149/149	283/283	212/212	184/184	183/183	177/177	143/143	202/202	159/187	233/233	328/328	366/378	287/287	160/170	249/249	273/276	219/228	175/184	202/206	168/174	145/149	110/112	417/423	455/457	368/372	382/382
163/163	165/165	203/206	212/212	234/234	238/238	202/202	259/262	147/147	278/278	224/224	195/195	177/183	177/177	143/143	204/204	159/187	236/236	309/309	366/366	281/281	160/160	230/241	273/273	216/219	184/184	203/203	157/185	147/147	100/100	417/426	452/452	368/368	390/390
163/169	165/165	203/203	212/212	234/234	238/243	196/202	259/259	147/149	278/283	218/224	190/195	177/183	177/177	143/143	202/204	159/187	233/236	309/325	366/387	281/323	160/160	241/253	273/273	216/219	184/184	199/202	168/168	147/151	100/102	417/426	452/452	368/368	390/402
163/169	165/165	203/206	206/206	229/234	238/243	196/202	259/259	149/149	278/283	212/224	184/195	177/183	177/177	143/143	202/207	159/159	233/236	303/328	366/378	281/298	160/170	241/249	273/273	219/228	175/184	199/206	168/174	147/149	104/114	420/423	458/464	368/372	390/390
163/163	165/165	203/206	206/206	229/229	238/238	196/202	259/268	147/149	278/283	224/224	195/195	177/183	177/177	143/143	202/204	159/159	233/236	312/325	366/366	281/298	160/160	249/249	273/273	219/228	184/184	199/206	174/176	147/149	118/118	423/426	464/468	368/368	382/402
163/169	165/165	203/206	206/212	229/234	238/243	196/202	259/268	147/149	283/283	212/224	184/195	171/183	177/177	125/143	202/202	159/187	233/236	319/328	366/369	281/298	160/167	249/253	261/276	219/219	175/184	196/214	170/181	147/151	118/126	417/423	457/464	368/368	382/402
163/169	165/165	203/206	206/212	229/234	238/243	196/202	259/268	147/149	283/283	212/224	184/195	171/183	177/177	125/143	202/202	159/187	233/236	319/328	366/369	281/298	160/167	249/253	261/276	219/219	175/184	196/214	170/181	147/151	118/126	417/423	457/464	368/368	382/402
163/169	165/165	203/206	206/212	229/234	238/243	196/202	259/268	147/149	283/283	212/224	184/195	171/183	177/177	125/143	202/202	159/187	233/236	319/328	366/369	281/298	160/167	249/253	261/276	219/219	175/184	196/214	170/181	147/151	118/126	417/423	457/464	368/368	382/402
163/169	165/165	203/206	206/212	229/234	238/243	196/202	259/268	147/149	283/283	212/224	184/195	171/183	177/177	125/143	202/202	159/187	233/236	319/328	366/369	281/298	160/167	249/253	261/276	219/219	175/184	196/214	170/181	147/151	118/126	417/423	457/464	368/368	382/402
163/169	165/165	203/206	206/212	229/234	238/243	196/202	259/268	147/149	283/283	212/224	184/195	171/183	177/177	125/143	202/202	159/187	233/236	319/328	366/369	281/298	160/167	249/253	261/276	219/219	175/184	196/214	170/181	147/151	118/126	417/423	457/464	368/368	382/402
163/169	165/165	203/206	206/215	229/229	232/235	196/196	259/259	147/147	278/283	212/218	184/190	177/177	177/177	132/134	204/205	159/159	230/230	316/325	369/381	285/289	160/162	242/249	273/276	219/225	184/184	201/217	141/148	139/145	102/106	417/420	454/454	368/369	346/346
163/169	165/165	200/203	212/212	229/229	235/238	193/202	259/259	147/149	283/283	215/224	187/195	171/177	177/177	134/143	202/204	159/187	230/242	309/319	366/369	283/289	160/170	249/249	273/276	216/231	184/184	199/201	154/187	145/151	102/116	416/426	450/452	368/369	382/390
169/169	165/165	203/206	206/212	229/234	243/243	196/202	259/262	149/149	281/283	212/224	184/195	177/183	177/177	143/143	202/204	159/159	233/236	309/309	366/378	281/287	160/170	241/249	261/273	216/219	175/175	196/206	168/174	147/149	110/112	423/423	455/464	372/372	382/390
169/169	165/165	203/206	206/212	229/234	243/243	196/202	259/262	149/149	281/283	212/224	184/195	177/183	177/177	143/143	202/204	159/159	233/236	309/309	366/378	281/287	160/170	241/249	261/273	216/219	175/175	196/206	168/174	147/149	110/112	423/423	455/464	372/372	382/390
163/169	165/165	200/203	212/212	229/234	235/238	196/196	259/268	149/149	278/283	215/224	187/195	nd.	177/177	140/143	202/205	159/159	230/236	325/334	366/369	289/298	160/165	249/249	273/276	219/229	175/184	201/206	154/174	145/149	108/118	423/423	455/459	368/369	382/390
163/163	165/165	203/206	206/206	229/229	238/243	196/202	259/262	147/149	278/278	212/215	184/187	171/177	177/177	134/143	202/202	159/159	233/233	303/334	366/381	281/285	160/165	241/249	273/273	216/231	175/184	199/201	168/179	147/149	106/108	420/423	452/452	368/368	382/390
163/169	165/165	203/206	206/212	229/229	235/243	193/196	259/262	149/149	278/283	212/215	184/187	177/183	177/177	134/143	202/205	159/187	233/236	319/325	369/378	285/298	165/170	249/249	273/273	228/231	175/184	201/206	154/181	145/147	110/116	416/423	452/464	369/372	382/390
158/169	165/165	206/209	206/212	229/234	243/243	196/196	259/262	149/149	283/283	212/212	184/184	177/183	177/177	137/143	202/202	159/187	233/233	319/325	378/378	281/294	167/170	249/253	273/273	219/219	175/175	196/212	170/181	147/149	102/118	420/420	459/464	368/372	382/390
163/169	165/165	203/206	212/212	234/234	238/243	196/202	259/268	147/149	281/283	224/224	195/195	177/177	177/177	143/143	202/204	159/187	233/236	309/325	366/378	281/281	160/160	241/249	261/273	216/228	175/184	196/199	168/181	147/149	106/110	423/426	452/464	368/372	390/402
169/169	165/165	203/206	206/212	229/234	243/243	196/196	262/268	147/149	283/283	212/224	184/195	183/183	177/177	125/143	202/204	159/187	233/233	312/328	378/378	281/298	160/170	249/249	273/273	216/228	175/175	196/212	172/181	147/151	110/118	423/426	455/464	372/372	390/390
163/163	165/165	203/203	206/212	234/234	238/238	196/202	259/259	147/149	278/283	212/224	184/195	183/183	177/177	143/143	202/204	159/187	233/236	309/328	366/378	281/281	160/170	241/249	261/273	219/219	184/184	199/199	168/175	147/149	100/114	423/426	452/464	368/368	390/402
163/169	165/165	206/206	206/206	229/229	238/243	196/196	262/268	149/149	283/283	212/224	184/195	177/183	177/177	137/143	202/202	159/187	233/233	319/325	366/378	294/298	160/170	249/253	273/273	219/228	175/184	196/212	170/181	147/149	118/118	420/420	455/459	368/372	382/390
169/169	165/165	200/203	206/212	229/234	243/243	196/202	259/259	147/149	283/283	215/224	187/195	171/177	177/177	140/143	202/204	159/187	230/236	312/319	369/378	285/298	160/165	241/249	273/276	216/219	175/175	199/204	170/175	147/151	100/116	420/426	452/455	368/372	382/402
163/169	165/165	206/206	206/206	229/229	238/243	196/196	259/268	149/149	283/283	212/224	184/195	183/183	177/177	140/143	202/204	159/187	233/233	312/325	366/387	281/287	160/170	249/249	273/273	219/228	175/184	206/206	168/170	147/147	114/118	423/426	464/464	368/368	382/382
163/169	165/165	203/206	212/212	234/234	238/243	196/202	262/268	147/149	278/283	212/224	184/195	183/183	177/177	143/143	202/204	159/187	233/236	312/328	366/378	281/281	160/170	241/249	261/273	219/228	175/184	199/206	174/175	149/149	110/118	417/423	452/464	368/372	382/390
163/163	165/165	203/206	206/212	nd.	238/243	196/202	259/262	147/149	281/283	212/224	184/195	177/183	177/177	143/143	202/202	159/159	233/236	309/328	366/378	281/298	160/170	241/249	261/273	216/228	175/184	196/206	174/181	149/149	106/110	417/426	452/455	368/368	382/390
163/169	165/165	206/206	206/206	229/229	243/243	196/196	259/262	149/149	278/283	212/212	184/184	183/183	177/177	143/143	202/202	159/159	233/233	325/328	378/394	298/298	164/170	249/249	273/273	219/228	175/175	202/206	170/174	147/149	110/114	417/423	455/458	368/372	382/390
163/169	165/165	206/206	206/206	229/229	243/243	196/196	259/262	149/149	278/283	212/212	184/184	183/183	177/177	143/143	202/202	159/159	233/233	325/328	378/394	298/298	164/170	249/249	273/273	219/228	175/175	202/206	170/174	147/149	110/114	417/423	455/458	368/372	382/390
163/169	165/165	206/206	206/206	229/229	243/243	196/196	259/262	149/149	278/283	212/212	184/184	183/183	177/177	143/143	202/202	159/159	233/233	325/328	378/394	298/298	164/170	249/249	273/273	219/228	175/175	202/206	170/174	147/149	110/114	417/423	455/458	368/372	382/390
163/169	165/165	206/206	206/206	229/229	243/243	196/196	259/262	149/149	278/283	212/212	184/184	183/183	177/177	143/143	202/202	159/159	233/233	325/328	378/394	298/298	164/170	249/249	273/273	219/228	175/175	202/206	170/174	147/149	110/114	417/423	455/458	368/372	382/390
163/169	165/165	206/206	206/206	229/229	243/243	196/196	259/262	149/149	278/283	212/212	184/184	183/183	177/177	143/143	202/202	159/159	233/233	325/328	378/394	298/298	164/170	249/249	273/273	219/228	175/175	202/206	170/174	147/149	110/114	417/423	455/458	368/372	382/390
163/169	165/165	206/206	206/206	229/229	243/243	196/196	259/262	149/149	278/283	212/212	184/184	183/183	177/177	143/143	202/202	159/159	233/233	325/328	378/394	298/298	164/170	249/249	273/273	219/228	175/175	202/206	170/174	147/149	110/114	417/423	455/458	368/372	382/390
163/169	165/165	206/206	206/206	229/229	243/243	196/196	259/262	149/149	278/283	212/212	184/184	183/183	177/177	143/143	202/202	159/159	233/233	325/328	378/394	298/298	164/170	249/249	273/273	219/228	175/175	202/206	170/174	147/149	110/114	417/423	455/458	368/372	382/390
163/169	165/165	206/206	206/206	229/229	243/243	196/196	259/262	149/149	278/283	212/212	184/184	183/183	177/177	143/143	202/202	159/159	233/233	325/328	378/394	298/298	164/170	249/249	273/273	219/228	175/175	202/206	170/174	147/149	110/114	417/423	455/458	368/372	382/390
163/169	165/165	206/206	206/206	229/229	243/243	196/196	259/262	149/149	278/283	212/212	184/184	183/183	177/177	143/143	202/202	159/159	233/233	325/328	378/394	298/298	164/170	249/249	273/273	219/228	175/175	202/206	170/174	147/149	110/114	417/423	455/458	368/372	382/390
163/169	165/165	206/206	206/206	229/229	243/243	196/196	259/262	149/149	278/283	212/212	184/184	183/183	177/177	143/143	202/202	159/159	233/233	325/328	378/394	298/298	164/170	249/249	273/273	219/228	175/175	202/206	170/174	147/149	110/114	417/423	455/458	368/372	382/390
163/169	165/165	206/206	206/206	229/229	243/243	196/196	259/262	149/149	278/283	212/212	184/184	183/183	177/177	143/143	202/202	159/159	233/233	325/328	378/394	298/298	164/170	249/249	273/273	219/228	175/175	202/206	170/174	147/149	110/114	417/423	455/458	368/372	382/390
163/169	165/165	206/206	206/206	229/229	243/243	196/196	259/262	149/149	278/283	212/212	184/184	183/183	177/177	143/143	202/202	159/159	233/233	325/328	378/394	298/298	164/170	249/249	273/273	219/228	175/175	202/206	170/174	147/149	110/114	417/423	455/458	368/372	382/390
163/169	165/165	206/206	206/206	229/229	243/243	196/196	259/262	149/149	278/283	212/212	184/184	183/183	177/177	143/143	202/202	159/159	233/233	325/328	378/394	298/298	164/170	249/249	273/273	219/228	175/175	202/206	170/174	147/149	110/114	417/423	455/458	368/372	382/390
163/169	165/165	206/206	206/206	229/229	243/243	196/196	259/262	149/149	278/283	212/212	184/184	183/183	177/177	143/143	202/202	159/159	233/233	325/328	378/394	298/298	164/170	249/249	273/273	219/228	175/175	202/206	170/174	147/149	110/114	417/423	455/458	368/372	382/390
163/169	165/165	206/206	206/206	229/229	243/243	196/196	259/262	149/149	278/283	212/212	184/184	183/183	177/177	143/143	202/202	159/159	233/233	325/328	378/394	298/298	164/170	249/249	273/273	219/228	175/175	202/206	170/174	147/149	110/114	417/423	455/458	368/372	382/390
163/169	165/165	206/206	206/206	229/229	243/243	196/196	259/262	149/149	278/283	212/212	184/184	183/183	177/177	143/143	202/202	159/159	233/233	325/328	378/394	298/298	164/170	249/249	273/273	219/228	175/175	202/206	170/174	147/149	110/114	417/423	455/458	368/372	382/390
163/169	165/165	206/206	206/206	229/229	243/243	196/196	259/262	149/149	278/283	212/212	184/184	183/183	177/177	143/143	202/202	159/159	233/233	325/328	378/394	298/298	164/170	249/249	273/273	219/228	175/175	202/206	170/174	147/149	110/114	417/423	455/458	368/372	382/390
163/169	165/165	200/206	206/212	229/229	235/243	193/196	259/262	149/149	278/283	212/215	184/187	177/183	177/177	134/143	202/205	159/187	230/233	325/334	369/378	289/298	165/170	249/249	273/276	228/229	175/184	201/206	154/174	147/149	116/118	423/423	452/464	369/372	382/382
163/169	165/165	203/206	206/212	229/234	238/243	196/202	259/262	147/149	278/283	212/224	184/195	183/183	177/177	143/143	202/204	159/187	233/242	309/325	366/378/394	281/298	160/170	249/249	273/273	216/228	175/184	199/202	174/185	147/149	100/110/118	423/426	458/468	368/372	382/390
169/169	165/165	206/206	206/206	229/229	243/243	196/196	259/262	149/149	283/283	212/224	184/195	183/183	177/177	143/143	202/202	159/187	233/233	325/328	394/403	298/298	164/170	253/253	273/273	228/228	175/175	206/206	168/174	145/149	108/114	423/423	457/464	368/368	382/382
158/169	165/165	203/206	206/212	229/234	243/243	196/202	259/259	147/149	283/283	212/212	184/184	171/183	177/177	125/140	202/202	159/187	233/233	319/325	394/394	287/298	164/167	249/253	273/279	221/228	175/175	206/212	174/175	145/150	114/126	423/426	457/457	368/368	382/390
158/169	165/165	203/206	206/212	229/234	243/243	196/202	259/259	147/149	283/283	212/212	184/184	171/183	177/177	125/140	202/202	159/187	233/233	319/325	394/394	287/298	164/167	249/253	273/279	221/228	175/175	206/212	174/176	145/150	114/126	423/426	457/457	368/368	382/390
163/169	165/165	203/206	206/206	229/234	238/243	196/202	259/259	147/149	278/283	212/224	184/195	177/183	177/177	143/143	202/204	159/187	233/236	316/334	366/384	298/347	160/170	249/249	273/273	216/219	175/184	199/206	181/181	145/151	95/106	420/423	457/462	368/368	382/390
nd.	165/165	200/203	206/212	229/229	235/243	196/196	259/259	149/149	278/283	212/212	184/184	171/171	177/177	125/140	202/205	159/159	230/233	325/334	369/369	285/298	164/165	237/253	276/276	228/231	175/184	201/208	154/162	145/147	116/126	423/426	457/459	368/369	382/382
163/169	165/165	206/206	206/212	229/234	238/243	196/202	259/262	147/149	278/283	212/224	184/195	183/183	177/177	140/143	202/204	159/159	233/236	309/309	366/394	281/298	160/164	249/249	273/276	216/228	175/184	199/206	174/187	147/147	112/114	420/423	458/464	368/368	390/402
163/169	165/165	203/206	206/212	229/234	238/243	196/196	262/268	149/149	283/283	212/224	184/195	177/183	177/177	143/143	202/202	159/187	233/236	325/328	366/378	281/298	160/170	249/249	273/273	219/228	175/184	196/206	174/181	147/149	110/118	420/423	455/464	368/372	382/390
163/169	165/165	203/206	206/206	229/229	238/243	196/196	259/259	147/149	283/283	212/218	184/190	171/183	177/177	134/143	202/202	159/159	233/233	319/325	387/387	285/298	160/167	245/249	273/273	216/219	175/175	199/206	157/172	147/147	114/126	420/426	459/459	368/368	382/390
163/169	165/165	203/206	206/212	229/234	238/243	196/196	262/268	149/149	283/283	212/224	184/195	177/183	177/177	143/143	202/202	159/187	233/236	325/328	366/378	281/298	160/170	249/249	273/273	219/228	175/184	196/206	174/181	147/149	110/118	420/423	455/464	368/372	382/390
163/169	165/165	203/206	206/212	229/234	238/243	196/196	262/268	149/149	283/283	212/224	184/195	177/183	177/177	143/143	202/202	159/187	233/236	325/328	366/378	281/298	160/170	249/249	273/273	219/228	175/184	196/206	174/181	147/149	110/118	420/423	455/464	368/372	382/390
163/163	165/165	203/203	206/206	229/240	232/238	196/196	259/259	147/149	283/283	212/212	184/184	177/183	177/177	132/143	204/206	159/159	230/236	316/325	366/369	285/298	162/176	242/253	261/273	219/219	184/184	199/217	141/157	139/147	102/103	417/430	473/473	368/373	390/390
169/169	165/165	203/203	210/212	234/240	238/243	196/196	259/259	147/149	275/283	212/212	184/184	177/183	177/177	143/143	204/205	159/159	230/233	303/303	366/384	281/281	162/170	242/249	273/273	219/219	184/184	203/206	145/168	139/149	103/106	420/423	457/464	368/368	382/386
163/169	165/165	203/203	206/210	229/240	232/243	196/196	259/259	149/149	275/286	212/212	184/184	183/183	177/177	132/143	202/204	159/159	230/233	316/325	372/372	298/298	162/170	242/249	273/273	219/228	175/184	203/206	145/174	139/147	103/128	417/430	455/476	368/373	382/382
163/163	165/165	203/203	210/212	234/234	238/238	202/202	259/259	147/147	278/286	224/224	195/195	177/183	177/177	134/134	204/204	187/187	236/236	309/312	366/366	281/281	160/160	241/241	261/273	219/226	184/184	199/203	176/185	149/151	100/112	417/426	464/468	368/368	390/402
162/163	165/165	203/203	206/210	229/240	232/238	196/196	259/259	149/149	275/275	212/212	184/184	171/183	177/177	132/140	205/205	159/159	230/230	316/319	369/387	298/298	151/162	242/246	256/273	217/219	184/184	201/203	141/146	139/145	103/116	426/433	457/473	369/373	382/382
163/169	165/165	203/206	206/210	229/240	232/243	196/196	259/268	149/149	275/283	212/224	184/195	177/183	177/177	132/143	202/205	159/159	230/233	316/328	372/384	287/298	160/162	242/249	273/276	219/228	184/184	196/203	145/181	139/147	103/118	423/430	464/476	368/373	386/386
163/169	165/165	200/206	206/206	229/229	235/243	193/196	259/268	149/149	278/283	215/224	187/195	177/177	177/177	140/143	202/202	159/187	230/233	319/328	369/378	281/285	160/165	249/249	273/276	219/229	175/184	196/201	154/181	147/147	116/118	416/423	452/455	369/372	382/382
169/169	146/165	206/206	206/206	229/229	238/243	196/196	259/259	147/149	283/283	212/224	184/195	183/183	177/177	143/143	202/202	159/187	233/233	325/328	366/378	287/294	160/170	249/249	273/273	228/228	184/184	206/206	168/174	145/147	110/118	423/426	455/464	368/372	382/382
163/169	165/165	203/203	206/212	229/234	238/243	196/202	259/259	147/149	283/286	212/218	184/190	183/183	177/177	143/143	202/204	159/159	233/236	309/325	366/387	285/323	160/176	249/253	261/273	216/219	175/184	199/202	157/168	147/151	102/114	417/426	463/463	368/368	382/390
163/163	165/165	200/206	206/212	229/229	238/243	193/196	259/268	149/149	278/283	215/224	187/195	171/183	177/177	134/143	202/205	159/159	233/233	328/334	366/381	289/298	160/165	237/249	273/273	219/229	175/184	201/206	154/174	145/149	110/116	416/423	452/455	368/368	382/382
163/163	165/165	203/206	206/212	229/234	238/243	196/202	259/259	147/149	278/278	212/224	184/195	177/183	177/177	143/143	202/204	159/187	233/242	309/328	366/378	281/298	160/170	249/249	261/273	216/219	175/184	199/202	170/175	147/149	100/110	417/426	452/458	368/368	390/402
163/169	165/165	203/206	206/212	229/234	238/243	196/202	259/262	147/149	278/283	212/224	184/195	183/183	177/177	143/143	202/204	159/187	233/242	309/325	366/378	281/298	160/170	249/249	273/273	216/228	175/184	199/202	174/185	147/149	100/110	423/426	458/468	368/372	390/390
163/169	165/165	203/206	206/212	229/234	238/243	196/202	259/262	147/149	278/283	212/224	184/195	183/183	177/177	143/143	202/204	159/187	233/242	309/325	366/378	281/298	160/170	249/249	273/273	216/228	175/184	199/202	174/185	147/149	100/110	423/426	458/468	368/372	390/390
163/169	165/165	203/206	206/212	229/234	238/243	196/202	259/262	147/149	278/283	212/224	184/195	183/183	177/177	143/143	202/204	159/187	233/242	309/325	366/378	281/298	160/170	249/249	273/273	216/228	175/184	199/202	174/185	147/149	100/110	423/426	458/468	368/372	390/390
163/169	165/165	203/206	206/212	229/234	238/243	196/202	259/262	147/149	278/283	212/224	184/195	183/183	177/177	143/143	202/204	159/187	233/242	309/325	366/378	281/298	160/170	249/249	273/273	216/228	175/184	199/202	174/185	147/149	100/110	423/426	458/468	368/372	390/390
169/169	165/165	203/206	206/212	229/234	243/243	196/196	259/262	149/149	278/283	212/218	184/190	183/183	177/177	143/143	202/202	159/159	233/236	325/328	378/387	298/323	160/170	249/249	261/273	219/219	175/175	199/206	157/174	147/149	110/114	417/423	458/458	368/372	382/382
163/169	165/165	206/209	206/206	229/234	238/243	196/196	259/262	147/149	278/283	212/212	184/184	183/183	177/177	143/143	202/204	159/187	233/236	309/325	366/394	281/298	160/164	249/253	273/276	219/228	175/184	199/212	172/175	147/151	118/126	417/423	457/464	368/368	382/402
163/163	165/165	203/206	206/212	234/234	238/238	196/202	259/262	147/149	278/283	212/224	184/195	177/177	177/177	134/143	204/204	187/187	233/236	309/312	366/366	281/281	160/170	230/241	261/273	219/228	184/184	199/199	175/183	149/149	100/112	420/426	452/463	368/368	390/390

163/169	165/165	203/203	206/212	229/234	243/243	196/202	262/268	147/149	278/278	224/224	195/195	183/183	177/177	143/143	202/204	159/187	233/242	312/328	378/381	281/298	160/160	249/253	273/279	216/228	175/175	199/202	170/181	147/147	114/118	423/426	458/464	368/372	382/390
163/169	165/165	203/206	206/206	229/229	243/243	196/202	259/268	147/149	278/278	212/224	184/195	177/183	177/177	143/143	202/204	159/159	233/236	312/325	378/394	281/298	164/165	237/249	273/273	219/228	175/175	202/206	170/174	149/149	110/114	423/423	452/458	368/369	390/390
169/169	146/165	203/206	206/206	229/229	243/243	196/196	259/262	149/149	283/283	212/212	184/184	183/183	177/177	140/143	202/204	159/187	233/233	309/328	366/378	287/287	170/170	249/253	273/276	219/228	175/175	206/208	162/174	145/149	110/112	423/423	455/464	368/372	382/382
169/169	146/165	203/206	206/206	229/229	243/243	196/196	259/262	149/149	283/283	212/212	184/184	183/183	177/177	140/143	202/204	159/187	233/233	309/328	366/378	287/287	170/170	249/253	273/276	219/228	175/175	206/208	162/174	145/149	110/112	423/423	455/464	368/372	382/382
169/169	146/165	203/206	206/206	229/229	243/243	196/196	259/262	149/149	283/283	212/212	184/184	183/183	177/177	140/143	202/204	159/187	233/233	309/328	366/378	287/287	170/170	249/253	273/276	219/228	175/175	206/208	162/174	145/149	110/112	423/423	455/464	368/372	382/382
169/169	146/165	203/206	206/206	229/229	243/243	196/196	259/262	149/149	283/283	212/212	184/184	183/183	177/177	140/143	202/204	159/187	233/233	309/328	366/378	287/287	170/170	249/253	273/276	219/228	175/175	206/208	162/174	145/149	110/112	423/423	455/464	368/372	382/382
163/169	165/165	203/206	206/206	229/229	238/243	196/196	262/262	149/149	283/283	212/212	184/184	183/183	177/177	143/143	202/202	159/159	233/233	309/328	366/394	287/294	160/170	249/249	276/276	219/219	175/184	206/206	168/168	138/147	110/116	423/423	455/464	368/368	382/382
163/163	165/165	203/203	206/212	234/234	238/238	202/202	259/262	147/147	278/278	224/224	195/195	183/183	177/177	149/149	204/204	187/187	236/236	309/309	366/366	281/281	160/160	241/241	261/261	216/216	184/184	196/196	168/175	147/151	100/100	426/426	452/452	368/368	390/390
158/163	165/165	203/209	206/206	229/229	238/243	196/196	259/259	149/149	283/283	212/218	184/190	171/183	177/177	143/146	202/204	159/159	233/236	309/319	366/366	285/298	160/170	249/253	261/273	219/219	175/184	199/212	157/174	147/150	102/108	417/420	455/455	368/368	382/382
163/163	165/165	203/206	206/212	229/234	238/243	196/196	259/268	147/149	278/281	212/224	184/195	177/183	177/177	143/143	202/204	159/159	233/236	309/328	366/394	281/298	160/170	241/249	261/273	216/228	175/184	196/202	170/181	147/149	110/114	423/426	458/464	368/368	390/402
163/163	165/165	206/206	206/212	229/234	238/243	196/196	262/268	149/149	278/283	212/224	184/195	183/183	177/177	143/143	202/202	159/159	233/236	325/328	366/394	281/298	160/170	249/249	273/273	228/228	175/184	196/206	174/174	149/149	110/118	417/420	455/458	368/368	382/382
163/163	165/165	206/206	206/212	229/234	238/243	196/196	262/268	149/149	278/283	212/224	184/195	183/183	177/177	129/131	202/202	159/159	233/236	325/328	366/394	281/298	160/170	249/249	273/273	228/228	175/184	196/206	174/174	149/149	110/118	417/420	455/458	368/368	382/382
163/163	165/165	206/206	206/212	229/234	238/243	196/196	262/268	149/149	278/283	212/224	184/195	183/183	177/177	143/143	202/202	159/159	233/236	325/328	366/394	281/298	160/170	249/249	273/273	228/228	175/184	196/206	174/174	149/149	110/118	417/420	455/458	368/368	382/382
163/163	165/165	206/206	206/212	229/234	238/243	196/196	262/268	149/149	278/283	212/224	184/195	183/183	177/177	143/143	202/202	159/159	233/236	325/328	366/394	281/298	160/170	249/249	273/273	228/228	175/184	196/206	174/174	149/149	110/118	417/420	455/458	368/368	382/382
163/163	165/165	206/206	206/212	229/234	238/243	196/196	262/268	149/149	278/283	212/224	184/195	183/183	177/177	143/143	202/202	159/159	233/236	325/328	366/394	281/298	160/170	249/249	273/273	228/228	175/184	196/206	174/174	149/149	110/118	417/420	455/458	368/368	382/382
163/163	165/165	206/206	206/212	229/234	238/243	196/196	262/268	149/149	278/283	212/224	184/195	183/183	177/177	143/143	202/202	159/159	233/236	325/328	366/394	281/298	160/170	249/249	273/273	228/228	175/184	196/206	174/174	149/149	110/118	417/420	455/458	368/368	382/382
163/163	165/165	206/206	206/212	229/234	238/243	196/196	262/268	149/149	278/283	212/224	184/195	183/183	177/177	143/143	202/202	159/159	233/236	325/328	366/394	281/298	160/170	249/249	273/273	228/228	175/184	196/206	174/174	149/149	110/118	417/420	455/458	368/368	382/382
163/163	165/165	206/206	206/212	229/234	238/243	196/196	262/268	149/149	278/283	212/224	184/195	183/183	177/177	143/143	202/202	159/159	233/236	325/328	366/394	281/298	160/170	249/249	273/273	228/228	175/184	196/206	174/174	149/149	110/118	417/420	455/458	368/368	382/382
163/163	165/165	206/206	206/212	229/234	238/243	196/196	262/268	149/149	278/283	212/224	184/195	183/183	177/177	143/143	202/202	159/159	233/236	325/328	366/394	281/298	160/170	249/249	273/273	228/228	175/184	196/206	174/174	149/149	110/118	417/420	455/458	368/368	382/382
163/163	165/165	206/206	206/212	229/234	238/243	196/196	262/268	149/149	278/283	212/224	184/195	183/183	177/177	143/143	202/202	159/159	233/236	325/328	366/394	281/298	160/170	249/249	273/273	228/228	175/184	196/206	174/174	149/149	110/118	417/420	455/458	368/368	382/382
163/163	165/165	206/206	206/212	229/234	238/243	196/196	262/268	149/149	278/283	212/224	184/195	183/183	177/177	143/143	202/202	159/159	233/236	325/328	366/394	281/298	160/170	249/249	273/273	228/228	175/184	196/206	174/174	149/149	110/118	417/420	455/458	368/368	382/382
163/163	165/165	206/206	206/212	229/234	238/243	196/196	262/268	149/149	278/283	212/224	184/195	183/183	177/177	143/143	202/202	159/159	233/236	325/328	366/394	281/298	160/170	249/249	273/273	228/228	175/184	196/206	174/174	149/149	110/118	417/420	455/458	368/368	382/382
163/163	165/165	206/206	206/212	229/234	238/243	196/196	262/268	149/149	278/283	212/224	184/195	183/183	177/177	143/143	202/202	159/159	233/236	325/328	366/394	281/298	160/170	249/249	273/273	228/228	175/184	196/206	174/174	149/149	110/118	417/420	455/458	368/368	382/382
163/163	165/165	206/206	206/212	229/234	238/243	196/196	262/268	149/149	278/283	212/224	184/195	183/183	177/177	143/143	202/202	159/159	233/236	325/328	366/394	281/298	160/170	249/249	273/273	228/228	175/184	196/206	174/174	149/149	110/118	417/420	455/458	368/368	382/382
163/163	165/165	206/206	206/212	229/234	238/243	196/196	262/268	149/149	278/283	212/224	184/195	183/183	177/177	143/143	202/202	159/159	233/236	325/328	366/394	281/298	160/170	249/249	273/273	228/228	175/184	196/206	174/174	149/149	110/118	417/420	455/458	368/368	382/382
163/163	165/165	206/206	206/212	229/234	238/243	196/196	262/268	149/149	278/283	212/224	184/195	183/183	177/177	143/143	202/202	159/159	233/236	325/328	366/394	281/298	160/170	249/249	273/273	228/228	175/184	196/206	174/174	149/149	110/118	417/420	455/458	368/368	382/382
163/163	165/165	206/206	206/212	229/234	238/243	196/196	262/268	149/149	278/283	212/224	184/195	183/183	177/177	143/143	202/202	159/159	233/236	325/328	366/394	281/298	160/170	249/249	273/273	228/228	175/184	196/206	174/174	149/149	110/118	417/420	455/458	368/368	382/382
163/163	165/165	206/206	206/212	229/234	238/243	196/196	262/268	149/149	278/283	212/224	184/195	183/183	177/177	143/143	202/202	159/159	233/236	325/328	366/394	281/298	160/170	249/249	273/273	228/228	175/184	196/206	174/174	149/149	110/118	417/420	455/458	368/368	382/382
163/163	165/165	206/206	206/212	229/234	238/243	196/196	262/268	149/149	278/283	212/224	184/195	183/183	177/177	143/143	202/202	159/159	233/236	325/328	366/394	281/298	160/170	249/249	273/273	228/228	175/184	196/206	174/174	149/149	110/118	417/420	455/458	368/368	382/382
163/163	165/165	206/206	206/212	229/234	238/243	196/196	262/268	149/149	278/283	212/224	184/195	183/183	177/177	143/143	202/202	159/159	233/236	325/328	366/394	281/298	160/170	249/249	273/273	228/228	175/184	196/206	174/174	149/149	110/118	417/420	455/458	368/368	382/382
163/163	165/165	206/206	206/212	229/234	238/243	196/196	262/268	149/149	278/283	212/224	184/195	183/183	177/177	143/143	202/202	159/159	233/236	325/328	366/394	281/298	160/170	249/249	273/273	228/228	175/184	196/206	174/174	149/149	110/118	417/420	455/458	368/368	382/382
163/169	165/165	203/206	206/206	229/234	238/243	196/202	259/259	147/149	278/283	212/224	184/195	177/183	177/177	143/143	202/204	159/187	233/236	316/334	366/384	298/347	160/170	249/249	273/273	216/219	175/184	199/206	181/181	145/151	95/106	420/423	457/462	368/368	382/390
158/169	165/165	206/209	206/206	229/229	243/243	196/196	259/259	149/149	283/283	212/212	184/184	171/183	177/177	143/146	202/202	159/159	233/233	316/319	378/378	285/294	164/167	249/253	273/276	219/219	175/175	206/212	172/174	147/149	106/128	420/423	455/455	368/368	382/382
169/169	165/165	203/206	206/206	229/229	243/243	196/196	259/259	149/149	283/286	212/212	184/184	183/183	177/177	143/146	202/202	159/159	233/233	309/319	378/378	294/298	164/167	245/249	273/276	219/228	175/175	206/212	170/174	138/150	106/128	420/423	455/457	368/372	382/382
163/169	165/165	203/206	206/212	229/234	238/243	196/196	259/259	147/149	278/283	212/224	184/195	177/183	177/177	143/143	202/204	159/187	233/236	309/328	366/378	281/298	160/164	241/249	261/273	219/228	175/184	196/196	181/181	147/149	100/110	423/426	455/455	368/372	382/390
163/169	165/165	206/206	206/206	229/229	243/243	196/196	262/262	149/149	278/283	212/224	184/195	183/183	177/177	143/143	202/204	159/187	233/233	328/328	394/403	298/298	170/170	249/253	273/273	219/228	175/175	202/206	170/174	145/149	110/114	417/423	457/458	368/368	382/390
163/169	165/165	203/203	206/212	229/234	238/243	196/202	259/259	147/149	283/286	212/218	184/190	183/183	177/177	143/143	202/204	159/159	233/236	309/325	366/387	285/323	160/176	249/253	261/273	216/219	175/184	199/202	157/168	147/151	102/114	417/426	463/463	368/368	382/390
163/169	165/165	203/203	206/212	229/234	238/243	196/202	259/259	147/149	283/286	212/218	184/190	183/183	177/177	143/143	202/204	159/159	233/236	309/325	366/387	285/323	160/176	249/253	261/273	216/219	175/184	199/202	157/168	147/151	102/114	417/426	463/463	368/368	382/390
163/169	165/165	203/203	206/212	229/234	238/243	196/202	259/259	147/149	283/286	212/218	184/190	183/183	177/177	143/143	202/204	159/159	233/236	309/325	366/387	285/323	160/176	249/253	261/273	216/219	175/184	199/202	157/168	147/151	102/114	417/426	463/463	368/368	382/390
163/169	165/165	203/203	206/212	229/234	238/243	196/202	259/259	147/149	283/286	212/218	184/190	183/183	177/177	143/143	202/204	159/159	233/236	309/325	366/387	285/323	160/176	249/253	261/273	216/219	175/184	199/202	157/168	147/151	102/114	417/426	463/463	368/368	382/390
163/169	165/165	203/203	206/212	229/234	238/243	196/202	259/259	147/149	283/286	212/218	184/190	183/183	177/177	143/143	202/204	159/159	233/236	309/325	366/387	285/323	160/176	249/253	261/273	216/219	175/184	199/202	157/168	147/151	102/114	417/426	463/463	368/368	382/390
163/169	165/165	200/203	206/212	229/229	235/243	193/196	259/259	147/149	278/283	212/215	184/187	177/183	177/177	140/140	202/205	159/187	230/233	325/334	369/394	285/298	164/165	249/249	273/276	228/229	175/184	204/206	174/179	145/147	114/116	416/423	452/457	368/369	382/390
163/163	165/165	203/203	206/206	234/234	238/238	196/202	262/268	147/149	278/278	212/224	184/195	177/183	177/177	143/143	204/204	187/187	236/236	312/312	366/366	281/281	160/170	241/249	261/273	219/228	184/184	199/203	175/185	147/149	100/102	417/426	452/463	368/368	390/390
169/169	165/165	206/206	206/206	229/229	243/243	196/196	259/259	149/149	283/283	212/212	184/184	183/183	177/177	143/143	202/202	159/159	233/233	309/328	378/394	287/294	170/170	249/249	273/276	219/219	175/175	206/206	168/174	147/147	116/128	423/426	457/457	368/372	382/382
158/163	165/165	203/206	206/212	229/234	243/243	196/196	259/259	149/149	278/283	212/212	184/184	171/183	177/177	125/143	202/202	159/159	233/233	325/328	394/394	287/298	167/170	249/253	273/279	221/228	175/175	202/212	170/175	147/150	110/114	417/426	457/458	368/368	382/390
163/169	165/165	203/206	206/212	229/234	238/243	196/202	262/262	147/149	283/283	212/224	184/195	183/183	177/177	143/143	202/204	187/187	233/236	319/328	366/366	281/287	160/170	249/249	273/276	219/228	175/184	199/206	157/174	145/147	110/112	417/423	455/464	368/372	382/390
163/169	165/165	203/206	206/212	229/234	238/243	196/202	262/262	147/149	283/283	212/224	184/195	183/183	177/177	143/143	202/204	187/187	233/236	319/328	366/366	281/287	160/170	249/249	273/276	219/228	175/184	199/206	157/174	145/147	110/112	417/423	455/464	368/372	382/390
163/169	165/165	203/206	206/212	229/234	238/243	196/202	262/262	147/149	283/283	212/224	184/195	183/183	177/177	143/143	202/204	187/187	233/236	319/328	366/366	281/287	160/170	249/249	273/276	219/228	175/184	199/206	157/174	145/147	110/112	417/423	455/464	368/372	382/390
163/169	165/165	203/206	206/212	229/234	238/243	196/202	262/262	147/149	283/283	212/224	184/195	183/183	177/177	143/143	202/204	187/187	233/236	319/328	366/366	281/287	160/170	249/249	273/276	219/228	175/184	199/206	157/174	145/147	110/112	417/423	455/464	368/372	382/390
163/169	165/165	203/206	206/212	229/234	238/243	196/202	262/262	147/149	283/283	212/224	184/195	183/183	177/177	143/143	202/204	187/187	233/236	319/328	366/366	281/287	160/170	249/249	273/276	219/228	175/184	199/206	157/174	145/147	110/112	417/423	455/464	368/372	382/390
163/169	165/165	203/206	206/212	229/234	238/243	196/202	262/262	147/149	283/283	212/224	184/195	183/183	177/177	143/143	202/204	187/187	233/236	319/328	366/366	281/287	160/170	249/249	273/276	219/228	175/184	199/206	157/174	145/147	110/112	417/423	455/464	368/372	382/390
163/169	165/165	203/206	206/212	229/234	238/243	196/202	262/262	147/149	283/283	212/224	184/195	183/183	177/177	143/143	202/204	187/187	233/236	319/328	366/366	281/287	160/170	249/249	273/276	219/228	175/184	199/206	157/174	145/147	110/112	417/423	455/464	368/372	382/390
163/169	165/165	203/206	206/212	229/234	238/243	196/202	262/262	147/149	283/283	212/224	184/195	183/183	177/177	143/143	202/204	187/187	233/236	319/328	366/366	281/287	160/170	249/249	273/276	219/228	175/184	199/206	157/174	145/147	110/112	417/423	455/464	368/372	382/390
163/169	165/165	203/206	206/212	229/234	238/243	196/202	262/262	147/149	283/283	212/224	184/195	183/183	177/177	143/143	202/204	187/187	233/236	319/328	366/366	281/287	160/170	249/249	273/276	219/228	175/184	199/206	157/174	145/147	110/112	417/423	455/464	368/372	382/390
163/169	165/165	203/206	206/212	229/234	238/243	196/202	262/262	147/149	283/283	212/224	184/195	183/183	177/177	143/143	202/204	187/187	233/236	319/328	366/366	281/287	160/170	249/249	273/276	219/228	175/184	199/206	157/174	145/147	110/112	417/423	455/464	368/372	382/390
163/169	165/165	203/206	206/212	229/234	238/243	196/202	262/262	147/149	283/283	212/224	184/195	183/183	177/177	143/143	202/204	187/187	233/236	319/328	366/366	281/287	160/170	249/249	273/276	219/228	175/184	199/206	157/174	145/147	110/112	417/423	455/464	368/372	382/390
163/169	165/165	203/206	206/212	229/234	238/243	196/202	262/262	147/149	283/283	212/224	184/195	183/183	177/177	143/143	202/204	187/187	233/236	319/328	366/366	281/287	160/170	249/249	273/276	219/228	175/184	199/206	157/174	145/147	110/112	417/423	455/464	368/372	382/390
163/169	165/165	203/206	206/212	229/234	238/243	196/202	262/262	147/149	283/283	212/224	184/195	183/183	177/177	143/143	202/204	187/187	233/236	319/328	366/366	281/287	160/170	249/249	273/276	219/228	175/184	199/206	157/174	145/147	110/112	417/423	455/464	368/372	382/390
163/169	165/165	203/206	206/212	229/234	238/243	196/202	262/262	147/149	283/283	212/224	184/195	183/183	177/177	143/143	202/204	187/187	233/236	319/328	366/366	281/287	160/170	249/249	273/276	219/228	175/184	199/206	157/174	145/147	110/112	417/423	455/464	368/372	382/390
163/169	165/165	203/206	206/212	229/234	238/243	196/202	262/262	147/149	283/283	212/224	184/195	183/183	177/177	143/143	202/204	187/187	233/236	319/328	366/366	281/287	160/170	249/249	273/276	219/228	175/184	199/206	157/174	145/147	110/112	417/423	455/464	368/372	382/390
163/169	165/165	203/206	206/212	229/234	238/243	196/202	262/262	147/149	283/283	212/224	184/195	183/183	177/177	143/143	202/204	187/187	233/236	319/328	366/366	281/287	160/170	249/249	273/276	219/228	175/184	199/206	157/174	145/147	110/112	417/423	455/464	368/372	382/390
163/169	165/165	203/206	206/212	229/234	238/243	196/202	262/262	147/149	283/283	212/224	184/195	183/183	177/177	143/143	202/204	187/187	233/236	319/328	366/366	281/287	160/170	249/249	273/276	219/228	175/184	199/206	157/174	145/147	110/112	417/423	455/464	368/372	382/390
163/169	165/165	203/206	206/212	229/234	238/243	196/202	262/262	147/149	283/283	212/224	184/195	183/183	177/177	143/143	202/204	187/187	233/236	319/328	366/366	281/287	160/170	249/249	273/276	219/228	175/184	199/206	157/174	145/147	110/112	417/423	455/464	368/372	382/390
163/169	165/165	203/206	206/212	229/234	238/243	196/202	262/262	147/149	283/283	212/224	184/195	183/183	177/177	143/143	202/204	187/187	233/236	319/328	366/366	281/287	160/170	249/249	273/276	219/228	175/184	199/206	157/174	145/147	110/112	417/423	455/464	368/372	382/390
163/169	165/165	203/206	206/212	229/234	238/243	196/202	262/262	147/149	283/283	212/224	184/195	183/183	177/177	143/143	202/204	159/187	233/236	319/328	366/366	281/287	160/170	249/249	273/276	219/228	175/184	199/206	157/174	145/147	110/112	417/423	455/464	368/372	382/390
169/169	165/165	206/206	206/206	229/229	243/243	196/196	259/259	149/149	283/286	212/212	184/184	171/171	177/177	140/146	202/202	159/159	233/233	319/325	369/394	298/298	164/167	249/253	273/279	219/221	175/175	208/214	162/170	147/151	126/128	423/426	455/457	368/368	382/382
169/169	165/165	203/209	206/206	229/229	243/243	196/196	259/259	149/149	283/283	212/212	184/184	171/171	177/177	125/140	202/202	159/159	233/233	319/325	369/394	298/298	164/167	249/253	276/279	219/221	175/175	208/214	162/170	147/151	126/128	423/426	455/457	368/368	382/382
169/169	165/165	203/209	206/206	229/229	243/243	196/196	259/259	149/149	283/283	212/212	184/184	171/171	177/177	125/140	202/202	159/159	233/233	319/325	369/394	298/298	164/167	249/253	276/279	219/221	175/175	208/214	162/170	147/151	126/128	423/426	455/457	368/368	382/382
169/169	165/165	203/209	206/206	229/229	243/243	196/196	259/259	149/149	283/283	212/212	184/184	171/171	177/177	125/140	202/202	159/159	233/233	319/325	369/394	298/298	164/167	249/253	276/279	219/221	175/175	208/214	162/170	147/151	126/128	423/426	455/457	368/368	382/382
169/169	165/165	206/206	206/206	229/229	243/243	196/196	259/259	149/149	283/286	212/212	184/184	171/171	177/177	140/146	202/202	159/159	233/233	319/325	369/394	298/298	164/167	249/253	273/279	219/221	175/175	208/214	162/170	147/151	126/128	423/426	455/457	368/368	382/382
169/169	165/165	206/206	206/206	229/229	243/243	196/196	259/259	149/149	283/286	212/212	184/184	171/171	177/177	140/146	202/202	159/159	233/233	319/325	369/394	298/298	164/167	249/253	273/279	219/221	175/175	208/214	162/170	147/151	126/128	423/426	455/457	368/368	382/382
169/169	165/165	203/209	206/206	229/229	243/243	196/196	259/259	149/149	283/286	212/212	184/184	171/183	177/177	125/143	202/202	159/159	233/233	319/325	394/394	294/298	164/167	253/253	273/276	219/221	175/175	206/212	172/174	149/151	114/126	420/423	455/457	368/368	382/382
169/169	165/165	203/209	206/206	229/229	243/243	196/196	259/259	149/149	283/283	212/212	184/184	171/171	177/177	125/140	202/202	159/159	233/233	319/325	369/394	298/298	164/167	249/253	276/279	219/221	175/175	208/214	162/170	147/151	126/128	423/426	455/457	368/368	382/382
169/169	165/165	203/209	206/206	229/229	243/243	196/196	259/259	149/149	283/283	212/212	184/184	171/171	177/177	125/140	202/202	159/159	233/233	319/325	369/394	298/298	164/167	249/253	276/279	219/221	175/175	208/214	162/170	147/151	126/128	423/426	455/457	368/368	382/382
169/169	165/165	203/209	206/206	229/229	243/243	196/196	259/259	149/149	283/283	212/212	184/184	171/171	177/177	125/140	202/202	159/159	233/233	319/325	369/394	298/298	164/167	249/253	276/279	219/221	175/175	208/214	162/170	147/151	126/128	423/426	455/457	368/368	382/382
169/169	165/165	206/206	206/206	229/229	243/243	196/196	259/259	149/149	283/286	212/212	184/184	171/171	177/177	140/146	202/202	159/159	233/233	319/325	369/394	298/298	164/167	249/253	273/279	219/221	175/175	208/214	162/170	147/151	126/128	423/426	455/457	368/368	382/382
169/169	165/165	203/209	206/206	229/229	243/243	196/196	259/259	149/149	283/283	212/212	184/184	171/171	177/177	125/140	202/202	159/159	233/233	319/325	369/394	298/298	164/167	249/253	276/279	219/221	175/175	208/214	162/170	147/151	126/128	423/426	455/457	368/368	382/382
163/169	165/165	206/209	206/206	229/229	238/243	196/196	259/262	147/149	283/283	212/224	184/195	171/183	177/177	125/143	202/202	159/159	233/233	319/325	366/369	287/298	164/167	249/249	273/279	219/228	175/184	206/214	170/174	149/151	100/126	423/426	455/457	368/368	382/390
163/169	165/165	203/203	212/212	234/234	238/243	196/202	259/259	147/149	278/283	218/224	190/195	177/183	177/177	143/143	202/204	159/187	233/236	309/325	366/387	281/323	160/160	241/253	273/273	216/219	184/184	199/202	168/168	147/151	100/102	417/426	452/452	368/368	390/402
163/169	165/165	206/206	206/206	229/229	238/243	196/202	262/262	149/149	283/283	212/224	184/195	183/183	177/177	143/143	202/204	159/187	233/233	309/328	366/378	287/294	160/160	249/249	276/276	219/219	175/184	206/206	168/174	145/147	110/112	417/423	455/464	368/368	382/382
163/169	165/165	206/206	206/206	229/229	238/243	196/202	262/262	149/149	283/283	212/224	184/195	183/183	177/177	143/143	202/204	159/187	233/233	309/328	366/378	287/294	160/160	249/249	276/276	219/219	175/184	206/206	168/174	145/147	110/112	417/423	455/464	368/368	382/382
163/169	165/165	206/206	206/206	229/229	238/243	196/202	262/262	149/149	283/283	212/224	184/195	183/183	177/177	143/143	202/204	159/187	233/233	309/328	366/378	287/294	160/160	249/249	276/276	219/219	175/184	206/206	168/174	145/147	110/112	417/423	455/464	368/368	382/382
163/169	165/165	206/206	206/206	229/229	238/243	196/202	262/262	149/149	283/283	212/224	184/195	183/183	177/177	143/143	202/204	159/187	233/233	309/328	366/378	287/294	160/160	249/249	276/276	219/219	175/184	206/206	168/174	145/147	110/112	417/423	455/464	368/368	382/382
163/169	165/165	203/203	206/212	229/234	238/243	196/196	262/262	147/149	283/283	212/224	184/195	183/183	177/177	143/143	202/204	159/187	233/236	319/325	366/378	287/287	160/170	249/249	273/273	219/219	175/184	199/202	174/174	145/147	110/110	417/417	455/464	368/368	382/390
163/169	165/165	206/206	206/206	229/234	238/238	196/202	259/262	147/149	278/283	224/224	195/195	183/183	177/177	143/143	202/204	187/187	233/233	328/328	366/378	281/287	160/170	241/249	273/276	216/228	184/184	196/206	174/181	145/149	110/118	417/423	452/464	368/368	382/402
163/169	165/165	203/206	206/206	229/229	238/243	196/196	262/262	147/149	283/283	212/224	184/195	183/183	177/177	143/143	202/202	159/187	233/236	309/328	366/378	287/298	160/164	249/249	273/273	219/228	175/184	199/206	157/175	147/147	108/110	423/426	455/464	368/368	382/390
158/169	165/165	203/206	206/212	229/234	243/243	196/202	259/259	147/149	283/283	212/212	184/184	171/183	177/177	125/140	202/202	159/187	233/233	319/325	394/394	287/298	164/167	249/253	273/279	221/228	175/175	206/212	174/175	145/150	114/126	423/426	457/457	368/368	382/390
163/169	165/165	203/206	206/212	229/234	238/243	196/196	262/268	149/149	283/283	212/224	184/195	177/183	177/177	143/143	202/202	159/187	233/236	325/328	366/378	281/298	nd.	249/249	273/273	219/228	175/184	196/206	174/181	147/149	110/118	420/423	455/464	368/372	382/390
163/163	165/165	203/206	206/212	234/234	238/238	196/202	259/262	147/149	278/283	212/224	184/195	177/177	177/177	134/143	204/204	187/187	233/236	309/312	366/366	281/281	160/170	230/241	261/273	219/228	184/184	199/199	175/183	149/149	100/112	420/426	452/463	368/368	390/390
158/163	165/165	203/206	206/206	229/229	238/243	196/196	259/259	149/149	283/283	212/212	184/184	177/183	177/177	143/143	202/202	159/159	233/233	309/316	366/366	294/298	164/167	249/253	273/273	219/228	175/184	206/206	174/174	138/149	106/118	420/423	455/464	368/368	382/382
163/169	165/165	203/206	206/212	229/234	238/243	196/196	262/268	149/149	283/283	212/224	184/195	177/183	177/177	143/143	202/202	159/187	233/236	325/328	366/378	281/298	160/170	249/249	273/273	219/228	175/184	196/206	174/181	147/149	110/118	420/423	455/464	368/372	382/390
163/169	165/165	203/206	206/212	229/234	238/243	196/196	262/268	149/149	283/283	212/224	184/195	177/183	177/177	143/143	202/202	159/187	233/236	325/328	366/378	281/298	160/170	249/249	273/273	219/228	175/184	196/206	174/181	147/149	110/118	420/423	455/464	368/372	382/390
163/169	165/165	203/206	206/212	229/234	238/243	196/196	262/268	149/149	283/283	212/224	184/195	177/183	177/177	143/143	202/202	159/187	233/236	325/328	366/378	281/298	160/170	249/249	273/273	219/228	175/184	196/206	174/181	147/149	110/118	420/423	455/464	368/372	382/390
163/163	165/165	203/203	212/212	234/234	238/238	202/202	259/268	147/147	278/281	224/224	195/195	183/183	177/177	143/143	204/204	187/187	236/242	312/312	366/378	281/281	160/160	241/249	279/279	216/216	184/184	199/199	175/175	147/149	100/106	420/426	452/468	368/368	390/402
163/169	165/165	203/206	206/206	229/234	238/243	196/202	259/259	149/149	278/283	212/224	184/195	177/183	177/177	143/143	202/207	159/159	233/236	303/328	366/378	281/298	160/170	241/249	273/273	219/228	175/184	199/206	168/174	147/149	104/114	420/423	458/464	368/372	390/390
158/163	165/165	206/206	206/212	229/234	238/243	196/196	259/268	149/149	283/286	212/224	184/195	171/177	177/177	134/143	202/202	159/187	233/233	319/325	366/369	281/294	160/167	249/253	273/273	228/233	175/184	206/212	174/181	147/150	106/110	420/420	455/455	368/368	382/390
163/163	165/165	203/206	206/206	229/234	238/238	196/202	259/262	147/149	278/281	212/224	184/195	183/183	177/177	143/143	202/204	159/187	233/236	309/328	366/366	281/281	160/170	241/249	273/273	216/228	184/184	203/206	174/175	149/149	100/118	417/417	nd.	368/368	382/390
163/169	165/165	206/206	206/206	229/229	243/243	196/196	259/262	149/149	283/283	212/224	184/195	183/183	177/177	140/143	202/202	159/159	233/233	325/328	384/384	287/298	160/170	249/249	273/276	228/228	175/175	202/206	174/174	147/149	118/118	417/423	455/457	368/368	382/382
163/169	165/165	203/206	206/206	229/229	243/243	196/196	259/262	149/149	283/283	212/224	184/195	183/183	177/177	140/140	202/204	159/159	233/233	309/328	384/384	287/298	160/170	249/249	273/273	219/228	175/175	202/206	174/174	147/149	110/112	417/423	455/457	368/368	382/382
158/163	165/165	203/206	206/212	229/234	238/243	196/202	259/259	147/149	281/283	212/224	184/195	177/183	177/177	140/143	202/204	159/159	233/236	309/325	366/366	281/298	160/164	241/249	261/273	216/228	175/184	199/206	168/174	149/149	110/114	417/426	457/464	368/368	382/402
163/169	165/165	203/206	206/206	229/234	238/238	196/202	259/262	147/149	278/283	212/224	184/195	177/183	177/177	134/143	202/204	187/187	233/236	312/328	366/378	281/287	160/170	249/249	273/276	219/228	184/184	199/206	174/175	145/149	106/112	420/423	464/464	368/372	382/390
169/169	165/165	206/206	206/212	229/234	243/243	196/196	259/268	149/149	278/283	212/224	184/195	177/183	177/177	143/143	202/202	159/187	233/233	328/328	378/378	281/298	160/164	249/249	273/273	228/228	175/175	196/202	174/181	147/149	110/114	420/423	455/458	372/372	382/390
163/169	165/165	203/203	206/212	229/234	238/243	196/202	259/259	147/149	278/283	212/224	184/195	183/183	177/177	143/143	204/207	159/187	233/236	303/309	366/378	281/298	160/170	241/249	273/273	219/219	175/184	199/199	168/175	147/149	100/114	423/426	452/458	368/372	390/402
163/169	165/165	206/206	206/206	229/229	243/243	196/196	259/259	149/149	283/283	212/212	184/184	183/183	177/177	140/140	202/204	159/159	233/233	309/328	378/384	298/302	170/170	249/257	273/276	219/228	175/175	206/206	174/174	149/149	118/128	423/436	457/457	368/372	382/382
163/163	165/165	203/206	206/212	229/234	238/243	196/202	259/259	147/149	281/283	224/224	195/195	177/183	177/177	143/143	202/202	159/159	233/236	309/328	366/378	281/298	160/160	241/241	261/273	216/228	175/184	196/206	174/181	149/149	110/110	417/426	452/455	368/368	382/390
163/169	165/165	200/206	206/212	229/229	235/243	193/196	259/259	149/149	278/283	212/215	184/187	171/177	177/177	134/140	202/205	159/159	230/233	319/334	369/381	285/289	165/170	237/249	273/276	229/231	175/184	201/204	154/179	145/147	108/116	416/423	452/459	368/369	382/382
163/169	165/165	206/206	206/212	234/234	238/243	196/202	262/268	147/149	283/283	212/224	184/195	183/183	177/177	143/143	202/202	159/187	233/236	309/328	366/366	285/298	160/160	249/249	273/273	219/228	175/184	206/208	157/174	147/147	110/118	420/426	455/464	368/372	382/382
163/169	165/165	203/206	206/206	229/229	238/238	196/202	262/268	147/149	278/283	212/224	184/195	183/183	177/177	143/143	202/204	159/187	233/236	309/328	366/366	287/294	160/170	249/249	273/276	219/228	184/184	206/206	157/174	147/149	110/118	417/420	458/464	368/368	382/382

163/163	165/165	203/206	206/212	229/234	238/243	196/202	262/268	149/149	283/283	212/224	184/195	183/183	177/177	143/143	202/202	159/187	233/236	328/328	366/394	281/298	160/160	249/249	273/273	219/228	175/184	196/199	157/174	147/149	110/112	417/420	455/458	368/368	382/390
163/163	165/165	203/206	206/206	229/234	238/243	196/196	259/262	147/149	278/283	212/224	184/195	177/183	177/177	140/143	202/204	159/187	233/233	309/328	366/384	281/287	170/170	249/249	273/276	228/228	175/184	199/202	174/175	147/147	100/112	417/417	452/455	368/368	382/390
163/163	165/165	206/206	206/206	229/229	238/238	196/196	262/268	147/149	283/283	212/224	184/195	183/183	177/177	140/143	202/204	159/159	233/233	325/328	366/366	287/287	160/160	249/249	273/273	219/228	184/184	196/202	174/174	147/149	110/118	420/423	455/457	368/368	382/382
163/163	165/165	203/206	206/212	229/234	238/243	196/202	262/262	147/149	283/283	212/224	184/195	183/183	177/177	140/143	202/204	159/187	233/233	309/328	366/384	281/287	160/160	249/249	276/276	219/228	175/184	202/206	174/174	145/147	110/110	417/417	455/464	368/368	390/390
163/169	146/165	203/206	206/212	229/234	238/243	196/196	262/262	147/149	283/283	212/212	184/184	183/183	177/177	140/143	202/202	159/187	233/236	328/328	366/366	281/287	160/170	249/249	273/276	219/228	175/184	206/206	174/174	145/149	110/110	420/423	455/455	368/368	382/382
163/163	165/165	200/203	204/212	223/234	238/238	196/202	259/268	147/152	281/283	215/224	187/195	177/183	177/177	143/143	204/207	159/161	218/236	309/325	362/366	281/283	160/170	241/249	261/267	216/228	184/195	196/199	181/185	147/154	100/106	416/426	452/452	368/368	390/395
169/169	165/165	203/206	206/212	229/234	238/243	196/202	259/262	147/149	283/283	212/224	184/195	171/183	177/177	125/143	202/202	159/187	233/236	319/328	366/366	281/287	160/167	249/253	273/276	219/219	175/184	206/214	170/174	145/151	110/118	417/423	455/457	368/372	382/402
163/169	165/165	206/206	206/212	229/234	238/243	196/202	262/262	149/149	283/283	212/224	184/195	183/183	177/177	140/143	202/202	159/159	233/233	309/328	366/384	281/298	160/170	249/249	273/276	219/219	175/184	202/206	174/174	147/149	110/118	417/423	464/464	368/368	382/382
163/163	165/165	203/203	210/212	234/234	238/238	202/202	259/259	147/147	278/286	224/224	195/195	177/177	177/177	134/143	204/204	159/187	236/236	309/312	366/366	281/281	160/160	241/241	273/273	219/226	184/184	203/203	157/185	147/149	100/112	426/426	452/468	368/368	390/402
163/163	165/165	203/203	206/206	234/234	238/238	202/202	262/268	147/147	278/278	224/224	195/195	177/183	177/177	143/149	204/204	187/187	236/242	309/312	366/366	281/281	160/160	230/241	261/261	216/216	184/184	196/203	175/185	147/151	100/100	426/426	452/452	368/368	390/402
163/169	165/165	203/206	206/212	229/234	238/243	196/202	262/268	149/149	283/283	212/224	184/195	183/183	177/177	143/143	202/204	159/159	233/233	325/328	366/384	287/287	160/170	249/249	273/276	219/219	175/175	199/206	168/174	149/149	110/112	420/423	457/464	368/372	390/390
163/169	165/165	206/206	206/206	229/229	238/243	196/196	262/268	149/149	283/283	212/224	184/195	183/183	177/177	140/143	202/204	159/159	233/233	309/325	366/384	287/298	160/170	249/249	273/273	219/228	175/184	202/206	170/174	147/147	110/118	423/423	457/457	368/368	382/382
163/163	165/165	203/206	206/206	229/229	238/243	196/196	262/262	149/149	283/283	212/212	184/184	183/183	177/177	143/143	202/204	159/159	233/236	309/328	366/384	285/287	160/170	249/249	273/273	219/228	175/175	199/206	168/174	147/149	110/112	417/423	457/464	368/368	382/390
163/163	146/165	203/203	206/206	229/234	238/243	196/196	262/262	149/149	283/283	212/212	184/184	183/183	177/177	140/143	204/204	159/159	233/236	309/309	366/384	287/294	160/170	249/249	273/273	219/219	175/184	202/206	168/174	147/149	110/112	417/423	455/457	368/368	382/390
163/169	165/165	203/206	206/212	229/234	238/243	196/202	262/268	147/149	283/283	224/224	195/195	183/183	177/177	143/143	202/202	159/159	233/236	325/328	366/366	281/294	160/160	249/249	273/276	219/228	175/184	196/206	174/174	147/149	110/118	417/423	455/464	368/372	382/390
163/169	165/165	206/206	206/206	229/229	238/243	196/196	259/268	149/149	283/283	212/224	184/195	183/183	177/177	140/143	202/202	159/187	233/233	309/328	366/366	287/287	170/170	249/249	273/276	228/228	175/184	206/206	170/174	145/147	112/118	423/423	455/457	368/372	382/382
163/169	165/165	206/206	206/212	234/234	238/243	196/196	259/268	147/149	283/283	212/224	184/195	183/183	177/177	125/143	202/202	159/159	233/236	319/328	366/366	281/281	167/170	249/253	273/276	219/228	175/184	206/214	170/174	149/151	110/110	417/420	455/455	368/368	382/402
163/169	165/165	203/206	206/206	229/229	238/243	196/202	262/268	147/149	283/283	212/224	184/195	171/183	177/177	143/143	202/204	159/187	233/233	309/328	366/369	281/298	160/170	249/253	273/276	219/228	175/184	196/199	181/185	147/151	110/126	423/423	455/458	368/368	390/402
163/169	146/165	203/206	206/206	229/234	238/243	196/196	262/268	149/149	283/283	212/224	184/195	183/183	177/177	140/143	202/202	159/187	233/233	328/328	366/366	287/298	160/170	249/249	273/273	219/228	175/184	206/206	157/174	145/147	110/118	420/423	455/464	368/368	382/382
163/169	146/165	203/206	206/212	229/234	243/243	196/196	259/268	149/149	278/283	212/224	184/195	183/183	177/177	143/143	202/204	159/187	233/233	309/328	378/394	281/287	160/170	249/249	273/273	219/228	175/175	196/206	174/174	145/149	110/110	417/423	455/458	368/372	382/382
163/163	165/165	203/206	210/212	234/234	238/238	202/202	259/259	147/147	278/286	224/224	195/195	177/177	177/177	134/143	204/204	159/187	236/236	309/309	366/366	281/281	160/160	230/241	261/273	219/219	184/184	199/203	157/185	147/149	100/112	426/426	468/468	368/368	390/390
163/169	165/165	206/206	206/206	229/229	238/243	196/196	259/268	147/147	283/283	212/224	184/195	183/183	177/177	143/143	202/202	159/187	233/236	325/328	366/366	287/298	160/164	249/249	273/273	219/228	175/184	206/212	157/172	147/151	110/126	417/423	455/457	368/372	382/402
163/163	165/165	203/206	206/206	229/229	238/238	196/196	262/268	147/149	278/283	212/224	184/195	183/183	177/177	143/143	202/202	159/159	233/236	309/328	366/366	294/298	160/170	249/249	273/276	219/228	184/184	196/206	174/174	149/149	110/118	417/420	455/464	368/368	382/390
163/169	165/165	206/206	206/206	229/229	238/243	196/196	259/259	149/149	283/283	212/224	184/195	183/183	177/177	140/143	202/204	159/159	233/233	309/312	366/384	287/287	170/170	249/249	273/273	219/228	175/184	206/206	170/174	147/147	112/114	417/426	457/457	368/368	382/382
163/169	146/165	203/206	206/206	229/229	238/243	196/196	259/262	147/149	283/283	212/212	184/184	183/183	177/177	140/143	202/202	159/187	233/233	328/328	366/378	287/287	160/170	249/253	273/273	219/228	175/184	196/206	174/174	145/149	110/112	420/423	455/455	368/368	382/390
163/163	165/165	206/206	206/212	229/234	238/243	196/196	259/268	149/149	278/283	212/224	184/195	183/183	177/177	140/143	202/204	159/187	233/236	325/328	366/394	281/287	160/170	249/249	273/276	228/228	175/184	206/206	168/174	147/149	110/118	417/423	458/458	368/368	382/382
163/163	165/165	203/206	206/206	229/229	243/243	196/196	262/268	149/149	283/283	212/224	184/195	183/183	177/177	140/143	204/204	159/159	233/233	309/312	384/387	287/294	160/160	249/249	273/273	219/219	175/184	202/208	174/174	147/147	110/118	417/423	464/464	368/368	382/390
163/163	165/165	206/206	206/206	229/229	238/243	196/196	259/262	149/149	283/283	212/224	184/195	183/183	177/177	140/143	202/202	159/187	233/233	325/328	366/384	287/287	160/170	249/249	273/273	219/228	175/184	206/206	170/174	147/147	112/114	417/423	455/464	368/368	382/382
163/169	165/165	203/206	206/212	229/234	238/243	196/202	262/268	147/149	283/283	224/224	195/195	183/183	177/177	143/143	202/202	159/187	233/236	328/328	366/366	287/298	160/160	249/249	273/273	219/228	175/184	196/206	157/174	147/149	110/118	420/423	455/464	368/372	382/382
163/169	165/165	203/206	206/212	229/234	238/243	196/202	259/268	147/149	283/283	212/224	184/195	171/183	177/177	125/143	202/202	159/187	233/236	319/328	366/369	281/298	160/167	249/253	261/276	219/219	175/184	196/214	170/181	147/151	118/126	417/423	457/464	368/368	382/402
163/169	146/165	203/203	206/206	229/229	243/243	196/196	262/262	147/149	283/283	212/212	184/184	183/183	177/177	143/143	202/204	187/187	233/233	309/309	366/384	287/294	160/160	249/249	273/276	219/219	175/175	202/206	174/174	145/147	110/112	417/423	455/464	368/372	382/382
163/169	165/165	206/206	206/212	234/234	238/243	202/202	262/268	147/147	278/283	224/224	195/195	177/183	177/177	143/143	204/204	159/187	236/236	309/312	366/378	281/281	160/170	241/241	273/273	219/219	184/184	199/203	177/185	147/147	100/110	423/426	452/455	368/372	390/402
163/163	165/165	206/206	206/212	229/234	238/243	196/196	262/262	149/149	283/283	212/224	184/195	183/183	177/177	140/143	202/202	159/159	233/236	309/328	366/384	281/287	160/170	249/249	273/273	219/228	175/184	196/202	174/174	147/149	110/118	420/423	455/457	368/368	382/390
163/169	165/165	206/206	206/212	229/234	238/243	196/196	268/268	149/149	283/283	224/224	195/195	183/183	177/177	143/143	202/202	159/159	233/236	309/328	366/384	281/287	160/160	249/249	273/273	228/228	175/184	196/206	174/174	147/149	110/118	420/423	455/455	368/368	382/382
163/169	165/165	203/206	206/206	229/234	238/243	196/196	259/262	147/149	283/283	212/224	184/195	183/183	177/177	143/143	202/204	159/159	233/236	309/328	366/366	285/287	160/170	249/249	273/276	228/228	175/184	199/208	168/168	145/149	106/110	423/423	464/464	368/372	382/382
163/163	165/165	203/206	206/212	229/234	238/243	196/196	262/268	149/149	278/283	212/224	184/195	183/183	177/177	143/143	202/204	159/159	233/233	309/328	366/384	281/287	160/170	249/249	273/273	219/228	175/184	206/206	174/174	147/149	112/118	417/420	457/458	368/368	382/390
163/163	165/165	206/206	206/212	229/234	238/243	196/202	262/268	147/147	283/283	212/224	184/195	183/183	177/177	143/143	202/202	159/187	233/233	328/328	366/366	287/287	160/170	249/249	273/276	219/228	175/184	196/206	174/174	145/149	110/110	417/420	455/464	368/368	382/390
169/169	146/165	203/206	206/206	229/229	243/243	196/196	262/262	147/149	278/283	212/212	184/184	183/183	177/177	143/143	202/204	159/187	233/233	309/328	378/384	287/298	160/170	249/249	273/273	219/228	175/175	202/202	170/174	147/147	110/110	417/423	458/464	372/372	382/390
163/163	165/165	206/206	206/206	229/229	238/243	196/196	262/268	149/149	283/283	212/212	184/184	183/183	177/177	143/143	202/204	159/159	233/233	312/328	366/378	287/298	160/170	249/249	273/273	219/228	175/184	206/206	170/174	147/149	110/118	423/423	458/464	368/368	382/390
163/163	165/165	200/203	204/206	223/234	238/238	196/202	259/268	147/152	281/283	224/224	195/195	177/183	177/177	137/143	202/207	159/161	236/236	312/312	362/366	281/298	160/170	249/249	261/267	216/228	184/195	187/199	158/185	151/154	106/112	416/426	452/464	368/368	390/395
163/169	146/165	203/203	206/206	229/234	243/243	196/196	262/262	147/149	283/283	212/212	184/184	183/183	177/177	143/143	202/204	187/187	233/233	309/309	366/366	285/287	160/170	249/249	276/276	219/219	175/175	202/206	174/174	145/147	112/112	417/423	455/464	368/372	382/390
163/169	165/165	203/206	206/212	229/234	238/243	196/202	262/268	149/149	278/283	212/224	184/195	183/183	177/177	140/143	202/204	159/159	233/236	325/328	366/394	281/287	160/170	249/249	273/276	219/228	175/175	196/199	168/174	149/149	110/110	417/420	458/464	368/372	382/382
163/169	146/165	203/206	206/206	229/234	238/243	196/196	262/262	147/149	283/283	212/212	184/184	183/183	177/177	143/143	202/204	159/187	233/236	309/309	366/366	285/294	160/170	249/249	273/276	219/228	175/184	199/206	168/174	145/149	110/112	423/423	455/464	368/372	382/382
163/163	165/165	203/206	206/206	229/234	238/238	196/202	259/262	147/147	278/283	212/224	184/195	177/183	177/177	143/149	202/204	187/187	236/236	309/328	366/378	281/285/287	160/170	241/249	261/273	216/228	184/184	196/199	157/175	147/151	100/106/110	423/426	452/464	368/368	382/390
163/169	165/165	203/203	206/206	229/229	238/243	196/196	262/262	149/149	283/283	212/212	184/184	183/183	177/177	140/143	202/202	159/187	233/236	309/328	366/384	287/294	170/170	249/249	276/276	219/228	175/184	206/206	174/174	145/147	110/112	417/423	455/464	368/372	382/390
163/169	165/165	203/203	206/206	229/229	238/243	196/196	262/268	149/149	283/283	212/212	184/184	183/183	177/177	140/143	202/204	159/159	233/233	309/328	366/366	287/287	160/170	249/249	276/276	219/228	175/184	199/206	168/174	147/149	106/110	417/423	457/464	368/372	382/390
163/163	165/165	203/203	206/206	229/234	238/243	196/196	259/268	147/149	278/283	224/224	195/195	183/183	177/177	140/143	202/204	159/187	233/236	309/328	366/384	281/287	160/160	241/249	261/273	228/228	175/184	199/206	157/174	147/149	100/110	417/417	452/455	368/368	382/390
163/169	165/165	206/206	206/206	229/229	243/243	196/196	262/268	149/149	283/283	212/224	184/195	183/183	177/177	143/143	204/204	159/159	233/233	312/312	366/387	294/294	160/170	249/249	273/273	219/219	175/184	206/208	174/174	147/147	110/114	423/423	455/464	368/372	382/382
163/169	165/165	203/203	206/206	229/229	243/243	196/196	262/262	149/149	283/283	212/212	184/184	183/183	177/177	143/143	202/202	187/187	233/233	309/328	366/366	287/294	160/170	249/249	273/276	219/228	175/175	199/202	168/174	145/145	110/112	417/423	455/457	368/372	382/390
163/163	165/165	203/206	206/212	229/234	238/243	196/196	262/268	149/149	283/283	212/224	184/195	183/183	177/177	143/143	202/202	159/159	233/233	309/325	366/384	281/287	160/160	249/249	273/273	219/228	175/184	199/206	168/174	149/149	110/112	417/420	455/457	368/368	382/390
163/169	165/165	203/206	206/212	229/234	238/243	196/196	259/268	147/149	283/283	212/224	184/195	183/183	177/177	143/143	202/204	187/187	233/236	309/328	366/378	287/287	160/170	249/249	273/273	219/219	175/184	196/206	157/174	145/147	110/110	420/423	455/455	368/368	382/382
163/169	165/165	206/206	206/206	229/234	238/243	196/196	259/262	147/149	283/283	224/224	195/195	183/183	177/177	143/143	202/202	159/159	233/236	309/328	366/366	285/287	160/160	249/249	273/273	219/228	175/184	196/199	168/174	145/149	106/110	420/423	464/464	372/372	382/382
169/169	165/165	203/206	206/212	229/234	238/243	196/196	262/268	149/149	283/283	212/212	184/184	183/183	177/177	140/143	202/202	159/187	233/233	328/328	366/378	281/287	160/170	249/249	273/273	219/228	175/184	196/206	174/174	149/149	110/110	423/423	455/455	368/368	382/382
163/169	146/165	203/206	206/206	229/234	238/243	196/196	259/262	147/149	283/283	212/224	184/195	183/183	177/177	143/143	202/202	159/159	233/236	309/328	366/366	285/287	160/170	249/249	273/276	219/228	175/184	199/208	157/174	147/147	110/112	423/426	455/464	368/372	382/382
169/169	146/165	203/206	206/206	229/229	243/243	196/202	262/268	149/149	283/283	212/224	184/195	183/183	177/177	143/143	202/204	187/187	233/236	309/328	366/378	287/287	160/170	249/249	273/276	219/219	175/175	206/208	157/162	145/147	110/112	423/423	455/464	368/372	382/382
163/169	165/165	203/206	206/212	229/234	238/243	196/196	259/268	147/149	283/283	212/224	184/195	183/183	177/177	143/143	202/204	187/187	233/233	309/328	366/378	287/287	160/170	249/249	273/273	219/219	175/184	196/206	157/174	145/147	110/110	420/423	455/455	368/368	382/382
169/169	165/165	203/206	206/206	229/229	243/243	196/202	259/262	147/149	278/283	212/224	184/195	177/183	177/177	134/143	202/204	159/187	233/236	312/328	378/394	281/298	170/170	241/249	261/273	219/228	175/175	199/202	170/181	147/151	110/114	417/423	455/458	372/372	390/390
163/163	165/165	203/206	206/206	229/234	238/238	196/196	262/268	147/149	278/283	212/224	184/195	nd.	177/177	143/143	202/204	187/187	233/242	312/325	366/378	281/298	170/170	249/249	273/273	228/228	184/184	199/202	177/185	147/147	100/110	426/426	452/468	368/368	382/390
163/169	165/165	206/206	206/206	229/234	238/243	196/196	262/268	147/149	278/283	212/224	184/195	183/183	177/177	143/143	202/204	159/187	233/233	312/328	366/378	281/298	170/170	249/249	273/273	228/228	175/184	196/206	174/181	147/149	110/118	420/423	455/455	368/372	382/402
163/169	165/165	206/206	206/206	229/229	243/243	196/202	262/262	149/149	283/283	212/224	184/195	183/183	177/177	140/143	202/202	159/159	233/233	328/328	366/384	287/298	160/160	249/249	273/273	219/228	175/175	196/202	174/174	147/147	110/118	417/420	455/457	368/372	382/382
163/169	165/165	203/203	206/212	229/234	238/243	196/202	259/268	147/149	283/283	224/224	195/195	177/183	177/177	134/143	204/204	159/187	233/236	312/328	366/378	281/281	160/170	241/249	261/273	219/228	175/184	196/199	168/181	149/151	110/118	417/426	455/462	368/372	390/390
163/169	165/165	206/206	206/206	229/229	238/243	196/202	268/268	149/149	283/283	224/224	195/195	183/183	177/177	140/143	202/202	159/159	233/236	309/328	366/384	287/287	160/170	249/249	273/273	219/228	175/175	196/202	174/174	147/149	110/110	420/423	455/457	368/368	382/382
163/169	165/165	203/206	206/206	nd.	238/243	196/202	262/268	147/149	283/283	nd.	195/195	183/183	177/177	143/143	202/202	159/159	233/236	325/328	366/366	298/298	160/170	249/249	273/273	219/228	175/184	196/206	174/174	149/149	110/118	420/423	455/464	368/372	382/382
163/169	165/165	206/206	206/206	229/229	238/243	196/202	262/268	149/149	283/283	224/224	195/195	183/183	177/177	143/143	202/202	159/187	233/233	309/328	366/384	287/298	160/160	249/249	273/273	219/219	175/184	196/202	174/174	147/149	118/118	420/423	457/464	368/372	382/382
163/163	165/165	203/206	206/206	229/229	243/243	196/196	259/262	149/149	283/283	212/212	184/184	183/183	177/177	140/140	204/204	159/187	233/233	312/328	384/384	287/287	160/160	249/249	273/273	219/228	175/175	202/206	168/174	147/147	110/118	417/426	457/464	368/368	382/382
163/169	165/165	203/206	206/212	229/234	238/243	196/202	259/268	147/149	278/283	224/224	195/195	nd.	177/177	143/143	204/204	187/187	233/236	309/312	366/378	281/298	160/170	230/249	273/273	216/228	175/184	196/203	181/185	147/147	100/100	417/423	455/455	368/372	382/390
169/169	146/165	203/206	206/206	229/229	238/243	196/202	262/262	149/149	283/283	212/224	184/195	183/183	177/177	140/143	202/202	187/187	233/236	328/328	366/378	287/298	160/170	249/249	273/273	219/228	175/184	196/206	174/174	145/149	110/118	423/423	455/455	368/368	382/382
169/169	165/165	203/206	206/212	229/234	238/243	196/202	262/262	147/149	283/283	212/224	184/195	183/183	177/177	143/143	202/202	159/187	233/236	328/328	366/366	287/294	160/170	249/249	273/276	219/219	175/184	206/206	157/174	147/147	110/110	417/423	464/464	372/372	382/390
163/169	165/165	206/206	206/212	229/234	238/243	196/196	262/268	149/149	283/283	212/224	184/195	183/183	177/177	140/143	202/202	159/159	233/236	309/328	366/384	281/287	160/160	249/249	273/273	228/228	175/184	202/206	174/174	147/149	110/110	420/423	455/455	368/368	382/382
163/163	158/165	200/203	204/206	223/234	238/238	196/196	259/268	147/152	281/283	215/224	187/195	177/177	177/177	137/143	202/204	161/187	218/236	312/325	362/378	283/298	170/172	241/249	267/273	222/228	184/195	187/196	158/181	147/154	100/112	416/426	452/463	368/368	395/402
163/169	165/165	206/206	206/212	229/234	238/243	196/202	262/268	149/149	278/283	224/224	195/195	183/183	177/177	143/143	202/202	159/187	233/233	328/328	366/366	281/298	160/170	249/249	273/276	219/228	175/184	206/206	174/174	145/149	110/118	417/423	455/464	368/372	382/382
163/169	165/165	203/206	206/212	229/234	238/243	196/202	268/268	149/149	278/283	224/224	195/195	183/183	177/177	143/143	202/202	159/159	233/236	325/328	366/366	287/298	160/160	249/249	273/273	219/228	175/175	196/196	174/174	149/149	110/118	420/423	455/458	368/372	382/382
163/169	165/165	206/206	206/206	229/229	238/243	196/202	262/268	147/149	278/283	224/224	195/195	183/183	177/177	143/143	202/202	159/187	233/236	325/328	366/366	287/298	160/160	249/249	273/273	219/228	175/184	196/206	157/174	147/149	110/110	420/423	455/464	368/372	382/382
163/169	165/165	206/206	206/212	229/234	238/243	196/196	262/262	149/149	281/283	212/212	184/184	183/183	177/177	140/143	202/202	159/159	236/236	309/309	366/378	281/287	160/170	249/249	261/273	228/228	175/184	196/202	174/174	147/149	110/112	423/423	455/464	368/372	382/390
163/169	165/165	206/206	206/212	234/234	238/243	196/202	262/268	147/149	283/283	212/224	184/195	183/183	177/177	143/143	202/202	159/187	233/236	328/328	366/366	281/281	170/170	249/249	273/273	219/228	175/184	206/206	174/174	145/149	110/110	420/423	455/455	368/372	382/382
163/169	146/165	203/203	206/206	229/229	243/243	196/196	262/268	149/149	278/283	212/212	184/184	183/183	177/177	140/143	202/202	187/187	233/233	328/328	384/384	287/298	160/170	249/249	273/273	219/228	175/175	202/202	168/174	147/147	110/118	423/426	458/464	368/372	382/382
163/169	165/165	206/206	206/206	229/229	238/243	196/196	262/268	149/149	283/283	224/224	195/195	183/183	177/177	143/143	202/202	159/187	233/233	325/328	366/384	287/287	160/160	249/249	273/273	219/228	175/184	202/206	168/174	147/147	118/118	420/420	455/457	368/368	382/382
163/169	165/165	203/206	206/212	229/234	243/243	196/202	262/262	149/149	283/283	212/224	184/195	183/183	177/177	143/143	202/204	159/187	233/233	309/325	366/366	281/287	160/160	249/249	273/276	219/219	175/175	196/206	174/174	145/149	110/110	417/423	457/464	368/372	382/390
163/169	165/165	203/206	206/212	229/234	238/243	196/196	262/268	147/149	283/283	212/224	184/195	183/183	177/177	140/143	202/202	159/187	233/236	325/328	366/378	287/294	160/160	249/249	273/273	228/228	175/184	196/206	174/174	149/149	110/118	423/423	464/464	368/372	382/390
163/163	165/165	203/203	206/212	229/234	238/238	196/202	259/268	149/149	283/283	212/224	184/195	183/183	177/177	143/143	202/206	159/159	233/236	316/319	366/366	281/285	160/176	242/249	261/276	219/219	184/184	214/217	141/170	139/151	103/126	417/417	464/464	368/368	402/402
163/169	165/165	206/206	206/212	229/234	238/243	196/202	262/268	149/149	283/283	212/224	184/195	183/183	177/177	140/143	202/202	159/159	233/236	309/325	366/366	281/287	160/170	249/249	273/276	219/228	175/184	202/206	174/174	147/149	110/118	423/423	455/464	368/372	390/390
163/169	165/165	206/206	206/206	229/229	238/243	196/202	262/268	149/149	278/283	212/224	184/195	183/183	177/177	143/143	202/204	187/187	233/233	319/328	366/366	281/287	170/170	249/249	273/276	219/228	175/184	206/206	174/174	145/147	110/110	420/423	455/455	368/372	382/382
163/163	165/165	203/206	206/206	229/229	238/238	196/202	262/268	149/149	281/283	212/224	184/195	177/177	177/177	143/143	202/202	159/187	236/236	309/328	366/378	298/298	160/170	249/249	261/273	219/228	nd.	196/206	174/181	147/149	110/110	426/426	452/455	368/368	382/402
163/169	146/165	206/206	206/206	229/234	238/243	196/202	259/262	147/149	283/283	224/224	195/195	183/183	177/177	143/143	202/202	159/159	233/236	309/328	366/366	287/298	160/160	249/249	273/273	219/228	175/184	196/199	168/174	149/149	110/118	423/426	455/464	368/372	382/390
163/169	165/165	206/206	206/206	229/229	238/243	196/202	262/268	147/149	283/283	212/224	184/195	183/183	177/177	143/143	202/202	159/187	236/236	328/328	366/366	287/298	160/170	249/249	273/273	219/228	175/184	196/206	174/174	145/149	110/110	417/420	455/464	368/372	382/390
163/169	165/165	203/206	206/206	229/229	243/243	196/196	262/262	147/149	283/283	212/224	184/195	183/183	177/177	140/143	202/204	159/187	233/233	328/328	366/384	287/287	160/160	249/249	273/273	228/228	175/175	196/202	174/174	147/149	110/118	423/426	457/464	368/372	382/382
163/163	165/165	206/206	206/212	229/234	238/243	196/196	259/268	149/149	283/283	212/224	184/195	183/183	177/177	140/143	202/204	159/187	233/233	328/328	366/366	287/298	160/160	249/249	273/273	219/228	175/175	196/206	168/174	147/149	118/118	417/420	455/464	368/368	382/382
163/163	165/165	206/206	206/206	229/229	238/238	196/196	268/268	147/147	283/283	212/224	184/195	183/183	177/177	140/143	202/204	187/187	233/233	328/328	366/366	281/287	160/170	249/249	273/273	219/228	175/184	206/206	157/174	147/147	110/118	420/423	455/457	368/368	382/382
163/163	165/165	203/206	206/212	229/234	238/243	196/202	259/262	149/149	278/283	212/212	184/184	171/183	177/177	125/143	202/202	159/187	233/236	319/328	366/394	298/298	160/170	249/249	273/276	219/228	175/184	196/206	174/181	147/149	110/118	417/417	458/464	368/368	382/382
169/169	165/165	203/206	206/212	229/234	243/243	202/202	262/262	149/149	283/283	212/224	184/195	183/183	177/177	143/143	202/202	159/187	233/236	328/328	366/366	287/294	160/160	249/249	273/276	219/228	175/175	206/206	157/174	147/147	110/118	417/423	455/464	372/372	382/382
163/169	165/165	206/206	206/206	229/229	238/243	196/196	262/268	149/149	278/283	212/212	184/184	183/183	177/177	143/143	202/204	159/159	233/236	325/325	366/394	287/298	160/160	249/249	273/273	228/228	175/184	206/206	174/174	149/149	110/110	417/420	455/458	368/372	382/390
163/163	165/165	203/203	212/212	234/234	238/238	202/202	259/259	147/147	278/286	224/224	195/195	183/183	177/177	134/143	204/204	187/187	236/236	309/312	366/366	281/281	160/160	230/241	273/273	216/219	184/184	199/203	157/175	147/151	100/100	417/417	464/464	368/368	390/390
163/169	146/165	206/206	206/212	229/234	238/238	196/196	259/268	147/149	278/283	224/224	195/195	183/183	177/177	143/143	202/202	159/159	233/233	325/328	366/378	294/298	160/160	249/249	273/273	228/228	184/184	196/206	174/174	145/149	110/118	420/423	458/464	368/372	382/382
163/163	165/165	203/206	206/206	229/229	238/243	196/196	262/268	149/149	283/283	212/224	184/195	183/183	177/177	143/143	202/202	159/187	233/233	325/328	366/384	281/287	160/170	249/249	273/276	219/228	175/184	206/206	174/174	145/149	110/110	417/420	457/458	368/368	382/382
163/163	165/165	206/206	206/206	229/229	238/243	196/196	268/268	149/149	283/283	224/224	195/195	183/183	177/177	140/143	202/204	159/187	233/236	325/325	366/384	287/298	160/160	249/249	273/273	228/228	175/175	196/202	174/174	147/147	110/118	420/423	457/464	368/368	382/382
169/169	146/165	206/206	206/206	229/229	238/243	196/196	262/268	149/149	283/283	212/224	184/195	183/183	177/177	140/143	202/204	159/187	233/233	325/328	366/366	287/287	160/170	249/249	273/276	219/219	175/184	202/206	174/174	145/147	110/118	423/423	455/457	368/368	382/382
163/169	165/165	206/206	206/212	229/234	238/243	196/196	262/268	149/149	283/283	224/224	195/195	183/183	177/177	143/143	202/202	159/187	233/233	309/328	366/384	281/287	170/170	249/249	273/273	219/228	175/184	206/206	170/174	145/147	110/118	423/423	464/464	368/368	382/382
163/169	146/165	206/206	206/212	229/234	243/243	196/202	262/262	149/149	283/283	212/212	184/184	183/183	177/177	140/143	202/202	159/187	233/233	309/328	378/384	281/298	160/170	249/249	273/273	219/228	175/175	196/202	174/174	147/149	110/118	420/423	455/455	368/368	382/382
163/169	165/165	203/206	206/206	229/229	238/243	196/202	262/262	147/149	278/283	212/224	184/195	183/183	177/177	143/143	202/202	187/187	233/236	309/328	366/366	287/294	160/160	249/249	273/276	219/228	175/175	206/206	157/174	145/147	110/110	420/423	455/455	368/372	382/382
163/169	165/165	206/206	206/206	229/229	238/243	196/196	262/268	149/149	283/283	224/224	195/195	183/183	177/177	143/143	202/202	159/159	233/236	325/325	366/384	281/298	160/160	249/249	273/273	228/228	175/184	196/206	170/174	145/149	110/114	423/423	464/464	368/372	382/382
169/169	146/165	206/206	206/206	229/229	243/243	196/202	259/268	149/149	283/283	212/224	184/195	183/183	177/177	143/143	202/204	159/187	233/233	309/328	366/366	287/298	160/170	249/249	273/273	219/228	175/184	196/206	174/174	145/149	110/118	420/423	455/455	368/368	382/382
163/169	165/165	206/206	206/212	229/234	238/243	196/202	262/268	147/149	278/281	212/224	184/195	183/183	177/177	143/143	202/202	159/187	233/233	328/328	366/366	281/287	160/170	249/249	273/273	219/219	184/184	196/196	168/174	149/149	110/112	420/423	455/464	368/372	382/390
163/163	165/165	206/206	206/212	229/234	238/243	196/196	262/268	149/149	283/283	212/224	184/195	183/183	177/177	143/143	202/204	159/159	233/233	325/325	366/384	287/298	160/160	249/249	273/273	219/228	175/175	196/202	174/174	147/149	110/110	420/423	455/455	368/368	382/382
169/169	165/165	206/206	206/206	229/229	243/243	196/202	262/262	149/149	278/283	212/224	184/195	183/183	177/177	143/143	202/204	159/159	233/236	325/325	366/384	298/298	160/160	249/249	273/273	219/228	175/175	196/202	174/174	145/149	110/110	420/423	455/455	368/372	382/382
169/169	146/165	206/206	206/206	229/229	238/238	196/202	259/268	147/149	283/283	212/224	184/195	183/183	177/177	140/143	202/202	159/187	233/233	328/328	366/378	287/287	160/160	249/249	273/273	219/228	184/184	196/206	168/174	145/149	110/110	420/423	455/455	368/372	382/382
163/169	165/165	203/206	206/206	229/229	238/243	196/196	262/262	147/149	278/283	212/224	184/195	183/183	177/177	143/143	202/202	159/187	233/236	325/328	366/366	287/298	160/160	249/249	273/276	219/228	175/184	196/202	174/174	145/149	110/112	420/423	457/464	368/372	382/382
163/169	165/165	206/206	206/206	229/229	243/243	196/196	268/268	149/149	283/283	224/224	195/195	183/183	177/177	143/143	202/204	159/159	233/236	325/325	384/394	287/298	160/170	249/249	273/273	219/228	175/184	206/206	174/174	147/149	118/118	417/423	455/455	368/368	382/382
163/169	165/165	206/206	206/212	229/234	238/243	202/202	262/268	149/149	278/281	212/224	184/195	183/183	177/177	143/143	202/202	159/187	233/233	309/328	366/378	281/298	160/170	249/249	273/273	219/219	175/175	196/199	174/174	145/149	110/110	420/423	455/464	368/372	382/390

163/169	146/165	203/206	206/206	229/234	238/243	196/202	259/262	147/149	283/283	212/224	184/195	183/183	177/177	143/143	202/202	159/159	233/236	309/328	366/384	285/298	160/170	249/249	273/273	219/219	175/175	199/202	168/174	147/149	110/110	420/423	455/464	368/368	382/382
163/169	165/165	203/206	206/206	229/234	238/243	196/196	262/268	147/149	283/283	212/224	184/195	183/183	177/177	143/143	202/204	159/187	233/233	328/328	366/378	281/287	160/170	249/249	273/276	228/228	175/184	206/206	174/174	145/149	110/110	417/420	455/464	368/372	390/402
163/169	165/165	203/206	206/212	229/234	238/238	196/202	268/268	149/149	283/283	224/224	195/195	183/183	177/177	143/143	202/204	159/159	233/233	328/328	366/366	287/298	160/170	249/249	273/276	219/228	175/184	196/199	168/174	149/149	110/118	420/423	455/464	368/372	382/390
163/163	165/165	203/206	206/206	229/229	238/243	196/202	262/268	147/149	283/283	212/224	184/195	183/183	177/177	140/143	202/204	159/187	233/236	309/309	366/384	287/287	160/160	249/249	273/276	228/228	nd.	202/206	170/174	145/147	110/110	417/423	458/464	368/368	382/390
163/169	165/165	203/206	206/206	229/229	238/243	196/196	262/262	149/149	278/283	212/224	184/195	183/183	177/177	140/143	202/202	159/159	233/236	309/328	366/378	287/298	160/170	249/249	261/276	219/228	184/184	196/202	174/174	147/149	110/112	420/423	455/457	368/372	382/382
169/169	146/165	203/206	206/206	229/229	243/243	196/196	262/262	149/149	283/283	212/212	184/184	183/183	177/177	143/143	202/202	187/187	233/233	309/328	366/378	294/298	160/160	249/249	273/276	219/228	175/175	202/206	157/174	147/149	110/112	423/423	464/464	368/372	382/382
163/163	165/165	203/206	206/212	229/234	238/243	196/202	262/268	149/149	283/283	224/224	195/195	183/183	177/177	140/143	202/204	159/187	233/236	309/328	366/384	281/287	160/170	249/249	273/276	219/219	175/175	196/206	174/174	145/149	110/110	417/420	457/464	368/368	382/390
163/163	165/165	206/206	206/206	229/229	238/238	196/202	262/262	149/149	283/283	212/224	184/195	183/183	177/177	143/143	202/202	159/187	233/233	309/328	366/366	287/287	160/160	249/249	273/273	219/228	184/184	202/206	157/174	147/147	110/118	420/423	464/464	368/368	382/382
169/169	165/165	203/206	206/206	229/229	243/243	196/196	262/262	147/149	283/283	212/224	184/195	183/183	177/177	140/143	202/202	159/159	233/236	309/328	366/384	287/287	160/170	249/249	273/273	228/228	175/175	202/206	157/174	147/149	110/110	423/423	455/455	368/372	382/382
163/169	165/165	206/206	206/206	229/229	238/243	196/202	262/268	147/149	283/283	212/224	184/195	183/183	177/177	140/143	202/202	159/187	233/236	328/328	366/366	287/298	160/160	249/249	273/276	219/219	175/184	206/206	157/174	145/147	110/110	417/423	455/464	368/372	382/390
163/169	146/165	206/206	206/206	229/229	238/243	196/196	262/268	147/149	283/283	212/224	184/195	183/183	177/177	143/143	202/202	159/187	233/233	309/325	366/366	287/287	160/160	249/249	273/273	228/228	175/184	196/206	174/174	145/149	110/112	420/423	464/464	368/372	382/382
163/169	165/165	203/206	206/206	229/229	238/243	196/196	262/268	149/149	278/283	212/224	184/195	183/183	177/177	143/143	202/202	159/187	233/236	309/328	366/366	294/298	160/160	249/249	273/276	219/228	175/184	196/206	174/174	145/149	110/118	417/417	458/464	368/372	382/382
163/169	165/165	203/203	206/212	229/234	238/243	196/196	262/268	147/149	283/283	212/224	184/195	183/183	177/177	143/143	202/202	159/187	233/233	309/328	366/366	287/294	160/170	249/249	273/276	219/228	175/184	196/202	174/174	145/149	110/118	420/423	457/464	368/372	382/382
163/169	165/165	203/206	212/212	234/234	238/243	202/202	262/262	147/149	281/283	212/212	184/184	183/183	177/177	143/143	202/202	159/187	233/236	309/328	366/378	281/287	170/170	249/249	261/276	216/219	175/184	206/206	174/174	145/147	110/110	420/423	455/464	368/372	390/390
163/169	165/165	206/206	206/212	229/234	238/243	196/202	262/268	147/149	283/283	212/224	184/195	183/183	177/177	143/143	202/204	159/159	233/236	309/328	366/384	281/287	160/170	249/249	261/273	216/219	175/184	206/206	174/174	147/149	110/112	420/423	458/464	368/372	382/390
163/169	165/165	206/206	206/212	229/234	238/243	196/202	262/262	149/149	278/283	212/224	184/195	183/183	177/177	143/143	202/202	159/187	233/233	328/328	366/366	281/298	160/170	249/249	273/276	219/228	184/184	206/206	174/174	145/149	110/112	420/423	455/458	368/372	382/382
163/163	165/165	203/206	206/212	229/234	238/243	196/196	262/268	149/149	278/283	212/224	184/195	183/183	177/177	140/143	202/202	159/187	233/236	309/325	nd.	281/287	160/170	249/249	273/273	219/228	175/184	202/206	174/174	145/149	110/110	417/420	457/458	368/368	382/390
163/163	165/165	203/206	206/206	229/229	238/243	196/196	262/262	149/149	283/283	212/212	nd.	183/183	177/177	143/143	202/202	159/159	233/233	328/328	366/366	287/298	160/170	249/249	273/273	219/228	175/184	206/206	174/174	147/149	112/118	417/420	455/464	368/368	382/390
163/169	165/165	203/203	206/212	229/234	238/243	202/202	262/268	147/149	283/283	224/224	195/195	183/183	177/177	125/143	202/202	159/187	236/236	328/328	366/369	281/287	167/170	249/253	273/276	219/219	175/184	206/214	170/174	145/151	110/126	417/417	455/457	368/368	382/382
163/169	165/165	206/206	206/212	229/234	238/243	196/202	259/262	147/149	283/283	212/224	184/195	183/183	177/177	143/143	202/202	187/187	233/233	328/328	366/369	281/298	160/167	249/253	276/276	219/228	175/184	196/206	174/181	145/147	110/126	417/423	455/464	368/368	382/390
163/169	165/165	203/203	206/212	229/234	238/243	196/202	259/262	147/147	283/283	212/212	184/184	183/183	177/177	125/143	202/202	187/187	233/236	319/328	366/366	281/287	167/170	249/249	273/276	219/228	175/184	206/214	170/174	145/147	110/118	417/423	455/464	368/372	390/402
163/169	165/165	203/206	206/206	229/234	238/243	196/196	259/262	149/149	283/283	212/224	184/195	183/183	177/177	143/143	202/202	187/187	233/233	309/328	366/378	281/294	160/170	249/249	273/276	219/219	175/184	196/208	168/181	145/147	110/112	423/426	455/455	368/372	382/382
163/169	165/165	203/206	206/206	229/234	243/243	196/196	262/262	147/149	283/283	212/212	184/184	183/183	177/177	143/143	202/204	159/159	233/233	309/309	366/366	287/287	160/170	249/249	273/273	219/219	175/175	202/206	168/174	147/149	110/110	417/423	455/464	368/372	382/390
163/169	165/165	206/206	206/212	229/234	238/243	196/196	262/268	147/149	278/283	212/224	184/195	183/183	177/177	143/143	202/202	159/187	233/233	319/328	366/378	281/298	170/170	249/249	273/276	228/228	175/184	206/206	174/174	145/149	110/112	417/423	455/464	368/372	382/402
163/169	165/165	206/206	206/206	229/234	238/243	196/196	259/268	149/149	281/283	224/224	195/195	177/183	177/177	143/143	202/204	159/187	233/233	328/328	366/378	281/298	160/170	249/249	273/273	228/228	175/184	196/206	174/181	147/149	110/118	420/426	452/455	368/372	382/402
163/169	146/165	206/206	206/212	234/234	238/243	196/196	259/262	147/149	281/283	212/224	184/195	177/183	177/177	143/143	202/204	159/159	233/233	309/309	366/378	281/287	160/170	241/249	273/273	219/228	175/184	196/206	168/174	149/149	106/112	423/423	455/464	368/372	382/390
163/169	165/165	206/206	206/206	229/234	238/243	196/196	262/268	147/147	283/283	212/224	184/195	177/183	177/177	143/143	202/204	187/187	233/233	312/328	378/378	281/298	160/170	241/249	273/273	228/228	175/184	196/206	174/181	147/147	100/118	423/426	452/455	368/372	382/402
163/169	146/165	203/206	206/206	229/229	238/243	196/196	259/262	147/149	283/283	212/224	184/195	183/183	177/177	140/143	202/204	187/187	233/233	309/328	366/378	294/298	160/170	249/249	273/276	219/228	175/184	199/206	174/177	145/147	110/110	423/423	455/455	368/372	382/402
163/163	165/165	203/206	206/212	234/234	238/238	196/202	259/268	147/147	278/283	224/224	195/195	177/177	177/177	143/143	202/204	187/187	236/236	309/328	366/378	281/281	160/170	230/249	273/273	216/228	184/184	196/203	157/181	147/147	100/110	417/426	455/455	368/368	390/402
163/169	165/165	206/206	206/206	229/234	238/243	196/202	262/268	147/149	283/283	212/224	184/195	183/183	177/177	143/143	202/204	187/187	233/236	328/328	366/378	281/287	160/170	249/249	273/273	219/228	175/184	199/206	174/177	145/147	110/110	417/423	455/464	368/372	382/402
163/163	165/165	203/206	206/206	229/229	238/243	196/196	262/268	147/149	283/283	212/224	184/195	183/183	177/177	140/143	202/204	159/187	233/236	328/328	366/384	287/287	160/160	249/249	273/273	219/219	175/175	196/206	174/174	147/149	110/118	420/426	457/464	368/368	382/382
163/169	165/165	203/206	206/212	229/234	238/243	196/196	262/268	147/149	278/283	212/224	184/195	183/183	177/177	143/143	202/202	159/187	233/236	328/328	366/366	281/298	160/170	249/249	273/273	228/228	175/184	206/206	174/174	145/149	112/118	417/420	455/458	368/372	382/390
163/169	165/165	203/206	206/212	229/234	243/243	196/202	262/262	147/149	283/283	212/224	184/195	183/183	177/177	140/143	202/204	187/187	233/233	312/328	366/384	287/298	160/160	249/249	273/273	219/219	175/175	202/206	157/168	147/147	110/118	423/426	457/464	368/372	382/382
163/169	165/165	203/206	206/212	229/234	243/243	196/196	262/268	147/149	283/283	212/224	184/195	183/183	177/177	140/143	202/204	159/187	233/236	328/328	366/384	287/298	160/160	249/249	273/273	228/228	175/175	202/206	157/174	147/147	110/110	420/426	455/457	368/372	382/382
163/169	165/165	206/206	206/206	229/229	238/243	196/202	268/268	149/149	278/278	224/224	195/195	183/183	177/177	143/143	202/202	159/187	233/236	325/328	366/366	287/298	160/170	249/249	273/273	219/228	175/184	196/206	174/174	147/149	110/118	420/423	455/458	368/372	382/382
163/169	165/165	206/206	206/212	234/234	238/243	196/202	262/268	147/149	283/283	212/224	184/195	183/183	177/177	143/143	202/202	159/187	233/236	328/328	366/366	281/298	160/170	249/249	273/273	219/228	175/184	206/206	157/174	147/149	110/118	420/423	455/464	368/372	382/382
163/169	146/165	206/206	206/212	229/234	238/243	196/196	262/262	149/149	283/283	212/224	184/195	183/183	177/177	140/143	202/202	159/187	233/236	328/328	366/366	281/287	160/170	249/249	273/273	219/228	175/184	206/206	174/174	145/145	110/110	423/423	455/455	368/368	382/382
163/163	165/165	206/206	206/206	229/229	238/243	196/196	262/268	149/149	283/283	212/224	184/195	183/183	177/177	140/143	202/204	159/187	233/233	328/328	366/366	281/287	160/170	249/249	273/276	228/228	175/184	202/206	174/174	145/149	110/118	420/426	455/457	368/368	382/382
163/169	165/165	203/206	206/212	229/234	238/243	196/196	262/268	147/149	283/283	212/212	184/184	183/183	177/177	140/143	202/202	159/187	233/236	328/328	366/366	281/298	170/170	249/249	273/276	219/228	175/184	206/206	174/174	145/149	110/110	420/423	455/464	368/368	382/382
163/163	165/165	203/206	206/206	229/234	238/238	202/202	259/268	147/149	278/283	224/224	195/195	183/183	177/177	143/149	202/204	159/187	233/236	309/328	366/366	281/287	160/160	241/249	261/273	216/219	184/184	196/196	174/175	149/151	100/118	420/426	452/464	368/368	382/390
163/169	165/165	203/203	206/206	229/229	243/243	196/202	262/268	147/149	278/283	212/224	184/195	183/183	177/177	143/143	202/202	159/187	233/236	328/328	366/384	294/298	160/170	249/249	273/276	219/228	175/175	196/202	168/174	147/149	110/110	423/426	458/464	368/372	382/390
169/169	165/165	203/206	206/206	229/229	238/243	196/196	259/262	147/149	283/283	212/212	184/184	183/183	177/177	143/143	202/204	187/187	233/233	309/328	366/378	287/287	160/170	249/249	273/276	219/219	175/184	206/208	157/162	145/147	110/112	417/423	455/464	368/372	382/382
163/163	165/165	203/206	212/212	234/234	238/243	196/202	259/268	147/152	278/283	224/224	195/195	183/183	177/177	143/149	202/204	187/187	233/236	309/328	366/366	281/287	160/170	241/241	261/273	216/228	175/184	196/196	174/175	147/151	100/118	426/426	452/452	368/368	390/395
163/163	165/165	203/206	206/206	234/234	238/238	196/202	262/268	147/149	278/283	224/224	195/195	183/183	177/177	143/149	204/204	187/187	233/236	309/325	366/378	281/287	160/170	241/249	261/273	216/222	184/184	196/196	174/175	147/151	100/100	420/426	452/452	368/368	382/390
163/169	146/165	203/206	206/206	229/229	238/243	196/202	262/268	147/149	283/283	212/224	184/195	183/183	177/177	140/143	202/202	159/187	233/233	325/328	366/384	294/298	160/170	249/249	273/273	219/219	175/184	202/206	168/174	147/147	110/118	417/423	464/464	368/372	382/390
163/169	165/165	206/206	206/206	229/234	238/243	196/202	262/268	147/149	283/283	212/224	184/195	183/183	177/177	140/143	202/202	159/187	233/236	328/328	366/366	281/287	170/170	249/249	273/273	219/219	175/184	206/206	174/174	145/149	110/118	420/423	455/455	368/372	382/382
163/169	165/165	200/206	204/212	229/234	238/243	196/202	262/262	149/152	283/283	212/224	184/195	183/183	177/177	143/143	202/204	159/187	230/233	328/328	366/366	287/298	160/160	249/249	267/273	216/219	184/184	196/206	174/174	145/149	106/118	417/423	455/464	368/372	382/382
163/169	146/165	203/206	206/212	229/234	238/243	196/202	262/268	147/147	283/283	212/224	184/195	183/183	177/177	143/143	202/204	159/187	233/233	309/328	366/384	281/287	160/170	249/249	273/273	219/219	175/184	206/214	157/170	147/151	118/126	417/420	455/464	368/372	382/390
169/169	165/165	203/203	206/212	229/234	238/243	196/202	262/268	149/149	283/283	224/224	195/195	183/183	177/177	140/143	202/202	159/159	233/233	325/328	366/366	294/298	170/170	249/249	273/276	219/219	175/184	196/206	157/174	147/149	106/110	423/423	455/464	372/372	382/390
163/163	165/165	203/206	206/212	229/234	238/238	196/202	259/268	147/149	278/283	224/224	195/195	177/183	177/177	143/143	202/204	159/187	233/236	309/325	366/366	298/314	160/160	230/249	273/273	219/228	184/184	196/199	168/174	149/149	106/110	420/420	455/464	368/368	382/390
163/169	146/165	206/206	206/212	229/234	238/243	196/202	262/268	149/149	283/283	212/224	184/195	183/183	177/177	143/143	202/202	159/159	233/233	325/328	366/366	294/298	160/170	249/249	273/273	219/228	175/184	196/206	174/174	145/149	110/118	423/423	455/464	368/372	382/382
169/169	146/165	203/206	206/206	229/229	238/243	196/196	262/268	147/149	283/283	212/224	184/195	183/183	177/177	143/143	202/202	159/187	233/236	328/328	366/366	281/287	160/170	249/249	276/276	219/228	175/184	206/206	174/174	145/147	110/110	423/423	455/455	368/372	382/390
169/169	146/165	203/203	206/206	229/229	243/243	196/196	262/262	149/149	283/283	212/224	184/195	183/183	177/177	143/143	202/202	159/187	233/233	328/328	366/366	287/298	160/170	249/249	273/276	219/228	175/175	206/206	174/174	145/149	110/110	420/423	455/455	368/372	382/382

232	233	234	235	236	237	238	239	240	241	242	243	244	245	246
	**	*	*	*	*	*		*	**	**		*	*	*
CX4040	CX5022	CX5039	CX6024	CX6035	CX6037	CX6F21	CX6F32	F02	F03	F16	F21	F40	F50	F79
0	0	0	0	0	0	0	0	0	0	1	4	0	0	0
368/368	233/237	432/437	198/198	246/252	165/171	146/152	157/157	168/168	266/272	163/169	nd.	80/99	156/156	172/172
368/369	237/237	432/432	192/198	252/252	171/171	146/152	157/157	168/170	266/278	169/169	149/155	80/90	156/156	172/172
368/369	237/237	432/432	192/198	246/252	165/171	152/152	157/157	168/168	266/278	163/169	155/155	99/99	156/156	172/172
369/369	237/237	432/432	192/192	252/252	171/171	146/152	157/157	168/168	278/284	169/169	149/155	80/80	156/156	172/172
368/370	233/233	437/442	198/198	246/246	165/165	146/152	157/164	168/168	266/272	163/163	149/155	90/99	156/161	172/172
365/369	233/233	437/437	192/198	246/252	165/165	146/152	157/157	154/168	272/278	163/163	149/155	95/95	161/161	172/180
368/368	233/237	432/437	198/198	246/252	165/171	146/152	157/157	168/168	266/278	163/169	149/155	90/99	156/161	172/172
368/368	233/233	437/442	192/198	240/246	165/171	146/152	157/164	168/168	266/272	163/169	149/155	85/99	156/156	172/172
368/368	233/233	437/437	192/198	246/246	165/165	146/152	157/164	168/172	272/278	163/163	149/155	99/99	156/161	172/172
368/368	233/233	437/437	192/198	246/246	165/165	146/152	157/164	168/172	272/278	163/163	149/155	99/99	156/161	172/172
368/368	233/233	437/437	192/198	246/246	165/165	146/152	157/164	168/172	272/278	163/163	149/155	99/99	156/161	172/172
368/368	233/233	437/437	192/198	246/246	165/165	146/152	157/164	168/172	272/278	163/163	149/155	99/99	156/161	172/172
369/371	233/233	437/437	198/198	246/246	165/165	146/152	157/157	168/168	266/266	163/163	149/155	90/99	161/161	172/172
366/370	233/237	432/437	192/198	246/246	165/165	152/152	157/164	168/172	267/278	163/163	155/155	99/99	161/161	172/172
369/375	233/237	432/437	192/198	246/252	153/177	146/152	157/157	168/168	261/278	151/175	149/155	90/90	156/161	172/172
370/371	233/233	437/442	198/198	246/246	165/165	146/152	157/164	168/172	266/266	163/163	149/155	99/99	161/161	172/172
370/371	233/233	437/442	198/198	246/246	165/165	146/152	157/164	168/172	266/266	163/163	149/155	99/99	161/161	172/172
369/370	237/237	432/432	192/192	252/252	171/171	146/146	157/157	168/168	278/278	169/169	149/149	90/90	156/156	172/172
368/370	233/233	437/442	181/198	246/246	165/165	146/152	157/164	168/168	266/272	163/163	149/155	90/99	156/156	172/172
369/375	233/233	437/446	181/192	246/246	153/165	146/152	157/157	168/175	261/278	151/163	149/155	99/104	161/161	172/172
369/370	233/237	432/442	192/198	246/252	165/171	146/152	157/164	168/168	272/284	163/169	149/155	80/90	156/161	172/172
368/368	233/233	437/442	192/192	246/246	165/165	146/152	157/157	168/168	266/267	163/163	nd.	90/99	161/161	172/172
370/370	233/233	437/442	198/198	246/246	153/165	146/152	157/164	168/175	261/266	151/163	149/155	80/99	156/161	172/172
366/366	233/237	432/437	192/198	246/252	165/171	152/152	157/157	168/168	278/278	163/169	155/155	90/99	156/161	172/172
366/366	233/237	432/437	192/198	246/252	165/171	152/152	157/157	168/168	278/278	163/169	155/155	90/99	156/161	172/172
367/367	233/237	432/437	192/198	246/252	165/171	152/152	157/157	168/168	278/278	163/169	155/155	90/99	156/161	172/172
370/371	233/233	437/442	192/198	246/246	153/177	140/146	157/157	175/175	261/272	151/175	143/149	80/80	161/161	172/172
368/369	233/237	432/437	192/198	246/252	171/171	152/152	157/157	168/170	266/278	169/169	155/155	90/90	156/161	172/172
368/369	232/233	432/437	192/198	246/246	171/177	140/152	157/157	168/175	266/272	169/175	143/155	80/90	161/161	169/172
368/369	232/233	432/437	192/198	246/246	165/165	140/146	157/157	168/168	266/272	163/163	143/149	95/99	161/161	169/172
368/370	233/233	437/437	198/198	246/246	165/165	146/152	157/164	168/172	266/267	163/163	149/155	99/99	161/161	172/172
366/369	237/237	432/432	192/192	252/252	171/171	146/146	157/157	168/168	278/284	169/169	149/149	90/99	156/156	172/172
368/369	237/237	432/432	192/198	252/252	165/171	146/152	157/157	168/170	272/278	163/169	149/155	80/99	156/156	172/172
368/370	233/237	432/437	192/198	246/252	165/171	146/152	157/157	168/168	266/278	163/169	149/155	90/99	156/156	172/172
369/371	233/237	432/437	192/198	246/246	165/171	146/152	157/157	168/168	266/278	163/169	149/155	99/99	156/161	172/172
368/370	233/237	432/442	192/192	246/252	153/171	146/146	157/157	168/168	261/278	151/169	149/149	80/90	156/161	172/172
368/370	233/237	432/442	192/192	246/252	153/171	146/146	157/157	168/168	261/278	151/169	149/149	80/90	156/161	172/172
368/370	233/237	432/442	192/192	246/252	153/171	146/146	157/157	168/168	261/278	151/169	149/149	80/90	156/161	172/172
368/370	233/237	432/442	192/192	246/252	153/171	146/146	157/157	168/168	261/278	151/169	149/149	80/90	156/161	172/172
368/370	233/237	432/442	192/192	246/252	153/171	146/146	157/157	168/168	261/278	151/169	149/149	80/90	156/161	172/172
365/365	232/233	437/437	192/198	246/246	171/171	140/152	157/164	154/196	272/272	169/169	143/155	80/95	156/156	169/180
370/370	233/237	432/437	192/192	246/246	165/178	140/146	157/157	168/175	272/278	163/175	143/149	95/99	156/156	169/172
368/370	233/237	432/437	198/198	246/252	165/171	146/152	157/157	168/168	266/284	163/169	149/155	80/99	156/161	172/172
368/370	233/237	432/437	198/198	246/252	165/171	146/152	157/157	168/168	266/284	163/169	149/155	80/99	156/161	172/172
368/369	232/233	432/437	192/198	246/252	171/177	140/146	157/157	168/175	266/272	169/175	143/149	80/90	156/156	169/172
369/370	233/237	432/437	192/198	246/246	165/171	140/146	157/157	168/168	266/272	163/169	143/149	90/95	156/161	172/172
368/369	233/233	437/437	192/198	246/246	165/177	140/152	157/157	168/168	272/278	163/175	143/155	80/99	156/156	169/172
368/375	232/237	432/442	192/198	246/252	153/171	146/146	157/157	168/175	261/266	151/169	149/149	80/90	156/161	172/172
368/369	237/237	432/432	192/198	252/252	171/171	146/152	157/157	168/168	266/284	169/169	149/155	80/90	156/156	172/172
368/370	233/237	432/437	192/198	246/252	171/171	146/152	157/157	168/175	266/266	169/169	149/155	90/90	156/156	172/172
369/370	237/237	432/432	192/198	252/252	165/171	146/152	157/157	168/168	278/278	163/169	149/155	80/99	156/156	172/172
368/375	232/233	437/442	192/198	246/246	153/165	146/152	157/157	168/175	261/266	151/163	149/155	80/99	156/161	172/172
369/371	233/237	432/442	192/192	246/252	153/171	146/146	157/157	168/175	261/284	151/169	149/149	80/90	156/161	172/172
370/370	233/237	432/437	192/198	246/246	165/165	152/152	157/157	168/168	266/272	163/163	155/155	99/104	161/167	172/172
368/369	237/237	432/432	192/198	252/252	171/171	152/152	157/157	168/168	266/278	169/169	155/155	90/90	156/161	172/172
369/369	233/237	432/437	198/198	246/252	165/171	146/152	157/157	168/168	266/272	163/169	149/155	80/99	156/156	172/172
368/368	233/233	437/437	198/198	246/246	165/165	146/152	157/157	168/168	266/272	163/163	149/155	99/99	156/161	172/172
369/369	233/233	437/437	198/198	246/246	165/165	146/152	157/157	168/168	266/272	163/163	149/155	99/99	156/161	172/172
368/368	233/233	437/437	198/198	246/246	165/165	146/152	157/157	168/168	266/272	163/163	149/155	99/99	156/161	172/172
368/368	233/233	437/437	198/198	246/246	165/165	146/152	157/157	168/168	266/272	163/163	149/155	99/99	156/161	172/172
369/369	233/233	437/437	198/198	246/246	165/165	146/152	157/157	168/168	266/272	163/163	149/155	99/99	156/161	172/172
369/369	233/233	437/437	198/198	246/246	165/165	146/152	157/157	168/168	266/272	163/163	149/155	99/99	156/161	172/172
369/369	233/233	437/437	198/198	246/246	165/165	146/152	157/157	168/168	266/272	163/163	149/155	99/99	156/161	172/172
369/369	233/233	437/437	198/198	246/246	165/165	146/152	157/157	168/168	266/272	163/163	149/155	99/99	156/161	172/172
369/369	233/233	437/437	198/198	246/246	165/165	146/152	157/157	168/168	266/272	163/163	149/155	99/99	156/161	172/172
368/368	233/233	437/437	198/198	246/246	165/165	146/152	157/157	168/168	266/272	163/163	149/155	99/99	156/161	172/172
369/369	233/233	437/437	198/198	246/246	165/165	146/152	157/157	168/168	266/272	163/163	149/155	99/99	156/161	172/172
369/369	233/233	437/437	198/198	246/246	165/165	146/152	157/157	168/168	266/272	163/163	149/155	99/99	156/161	172/172
369/369	233/233	437/437	198/198	246/246	165/165	146/152	157/157	168/168	266/272	163/163	149/155	99/99	156/161	172/172
369/369	233/233	437/437	198/198	246/246	165/165	146/152	157/157	168/168	266/272	163/163	149/155	99/99	156/161	172/172
369/369	233/233	437/437	198/198	246/246	165/165	146/152	157/157	168/168	266/272	163/163	149/155	99/99	156/161	172/172
369/369	233/233	437/437	198/198	246/246	165/165	146/152	157/157	168/168	266/272	163/163	149/155	99/99	156/161	172/172
368/369	232/233	432/437	192/198	246/246	171/177	140/152	157/157	168/175	266/272	169/175	143/155	80/90	161/161	169/172
368/370	233/237	432/437	192/198	246/252	165/171	146/152	157/157	168/168	266/278	163/169	149/155	80/99	156/161	172/172
368/370	233/233	437/437	198/198	246/246	165/165	146/152	157/157	168/168	266/266	163/163	149/155	99/99	161/161	172/172
370/370	233/233	437/442	181/198	246/252	165/178	146/152	157/164	168/175	261/266	163/175	149/155	85/90	156/161	172/172
371/371	233/233	437/442	181/198	246/252	165/178	146/152	157/164	168/175	261/266	163/175	149/155	85/90	156/161	172/172
369/371	233/237	432/442	192/198	246/252	165/165	146/146	157/157	168/170	266/278	163/163	149/149	90/90	156/161	172/172
369/369	233/233	437/442	192/198	246/246	165/177	146/146	157/157	168/175	261/272	163/175	149/149	80/90	161/161	169/174
366/368	233/237	432/437	192/198	246/252	165/171	146/152	157/164	170/172	266/278	163/169	149/155	90/99	156/156	172/172
368/368	233/237	432/437	198/198	246/252	165/171	146/152	157/157	168/168	266/278	163/169	149/155	90/99	156/161	172/172
369/371	233/233	437/442	192/198	246/246	153/165	146/152	157/157	168/175	261/272	151/163	149/155	99/99	156/161	172/172
369/369	233/237	432/437	198/198	246/252	165/171	146/152	157/157	168/168	266/278	163/169	149/155	90/99	156/161	172/172
369/369	233/237	432/437	198/198	246/252	165/171	146/152	157/157	168/168	266/278	163/169	149/155	90/99	156/161	172/172
365/369	233/246	417/437	192/198	246/258	165/171	146/152	157/157	154/168	272/272	163/169	149/155	95/95	156/156	172/180
369/370	237/237	422/432	192/198	252/258	165/171	152/158	157/157	154/168	272/272	163/169	155/161	95/99	161/161	172/180
365/370	233/246	422/437	198/198	246/258	165/171	152/158	157/157	154/168	267/272	163/169	155/161	95/99	161/161	172/176
370/371	237/237	432/432	192/192	252/252	171/171	146/152	157/164	168/168	278/278	169/169	149/155	90/90	156/156	172/172
365/383	246/246	422/442	192/198	246/258	171/171	146/158	157/157	154/182	272/272	169/169	149/161	85/95	161/161	172/180
365/369	233/246	422/437	192/198	246/258	165/171	152/158	157/164	154/172	272/272	163/169	155/161	95/99	161/161	172/180
368/369	233/237	432/437	192/198	246/246	165/165	140/146	157/157	168/175	272/278	nd.	nd.	95/99	161/161	172/172
370/370	233/233	437/442	192/198	246/246	165/165	152/152	157/157	168/168	266/266	163/163	155/155	99/99	161/167	172/172
368/369	233/237	432/437	192/198	246/252	165/165	152/152	157/157	168/168	272/278	163/163	155/155	95/99	156/161	172/172
368/369	232/233	432/437	192/198	246/246	165/165	140/146	157/157	168/168	266/272	163/163	143/149	95/99	161/161	169/172
368/370	233/237	432/437	192/198	246/252	165/171	152/152	157/157	168/168	266/278	163/169	155/155	80/99	156/156	172/172
368/370	233/237	432/437	192/198	246/252	165/171	146/152	157/157	168/168	266/278	163/169	149/155	80/99	156/156	172/172
368/370	233/237	432/437	192/198	246/252	165/171	146/152	157/157	168/168	266/278	163/169	149/155	80/99	156/156	172/172
368/370	233/237	432/437	192/198	246/252	165/171	146/152	157/157	168/168	266/278	163/169	149/155	80/99	156/156	172/172
368/370	233/237	432/437	192/198	246/252	165/171	146/152	157/157	168/168	266/278	163/169	149/155	80/99	156/156	172/172
368/369	233/237	432/437	198/198	246/252	165/165	146/152	157/157	168/168	266/278	163/163	149/155	95/99	161/161	172/172
369/370	233/237	432/442	198/198	246/252	165/171	152/152	157/157	168/170	266/278	163/169	155/155	99/99	156/161	172/172
366/369	237/237	432/432	192/192	252/252	171/171	146/152	157/157	168/168	278/278	169/169	149/155	80/90	156/156	172/176

368/369	233/237	432/442	192/198	246/252	165/171	152/152	157/164	168/168	272/278	163/169	155/155	90/99	156/161	172/172
368/368	233/237	432/437	192/198	246/246	165/171	146/152	157/157	168/168	266/278	163/169	149/155	90/99	156/161	172/172
370/371	233/233	437/437	192/198	246/246	165/171	146/152	157/164	168/172	266/267	163/169	149/155	85/99	161/161	172/174
370/371	233/233	437/437	192/198	246/246	165/171	146/152	157/164	168/172	266/267	163/169	149/155	85/99	161/161	172/174
370/371	233/233	437/437	192/198	246/246	165/171	146/152	157/164	168/172	266/267	163/169	149/155	85/99	161/161	172/174
370/371	233/233	437/437	192/198	246/246	165/171	146/152	157/164	168/172	266/267	163/169	149/155	85/99	161/161	172/174
368/368	233/233	437/442	198/198	246/246	165/171	146/146	157/157	168/168	266/266	163/169	149/149	85/90	161/161	172/172
369/369	237/237	432/432	192/192	252/252	171/171	146/152	157/157	168/168	284/284	169/169	149/155	90/90	156/156	172/172
369/375	233/233	437/446	181/192	246/246	153/165	146/152	157/157	168/175	261/278	151/163	nd.	99/104	161/161	172/172
368/368	233/237	432/437	192/198	246/252	165/171	146/152	157/157	168/168	272/284	163/169	149/155	80/99	156/156	172/172
368/368	233/237	432/437	198/198	246/252	165/171	152/152	157/157	168/168	266/278	163/169	155/155	90/99	161/161	172/172
368/368	233/237	432/437	198/198	246/252	165/171	152/152	157/157	168/168	266/278	163/169	155/155	90/99	161/161	172/172
369/369	233/237	432/437	198/198	246/252	165/171	152/152	157/157	168/168	266/278	163/169	155/155	90/99	161/161	172/172
369/369	233/237	432/437	198/198	246/252	165/171	152/152	157/157	168/168	266/278	163/169	155/155	90/99	161/161	172/172
369/369	233/237	432/437	198/198	246/252	165/171	152/152	157/157	168/168	266/278	163/169	155/155	90/99	161/161	172/172
369/369	233/237	432/437	198/198	246/252	165/171	152/152	157/157	168/168	266/278	163/169	155/155	90/99	161/161	172/172
368/368	233/237	432/437	198/198	246/252	165/171	152/152	157/157	168/168	266/278	163/169	155/155	90/99	161/161	172/172
369/369	233/237	432/437	198/198	246/252	165/171	152/152	157/157	168/168	266/278	163/169	155/155	90/99	161/161	172/172
369/369	233/237	432/437	198/198	246/252	165/171	152/152	157/157	168/168	266/278	163/169	155/155	90/99	161/161	172/172
368/368	233/237	432/437	198/198	246/252	165/171	152/152	157/157	168/168	266/278	163/169	155/155	90/99	161/161	172/172
368/368	233/237	432/437	198/198	246/252	165/171	152/152	157/157	168/168	266/278	163/169	155/155	90/99	161/161	172/172
369/369	233/237	432/437	198/198	246/252	165/171	152/152	157/157	168/168	266/278	163/169	155/155	90/99	161/161	172/172
368/368	233/237	432/437	198/198	246/252	165/171	152/152	157/157	168/168	266/278	163/169	155/155	90/99	161/161	172/172
368/368	233/237	432/437	198/198	246/252	165/171	152/152	157/157	168/168	266/278	163/169	155/155	90/99	161/161	172/172
368/368	233/237	432/437	198/198	246/252	165/171	152/152	157/157	168/168	266/278	163/169	155/155	90/99	161/161	172/172
369/369	233/237	432/437	198/198	246/252	165/171	152/152	157/157	168/168	266/278	163/169	155/155	90/99	161/161	172/172
369/369	233/237	432/437	198/198	246/252	165/171	152/152	157/157	168/168	266/278	163/169	155/155	90/99	161/161	172/172
368/368	233/237	432/437	198/198	246/252	165/171	152/152	157/157	168/168	266/278	163/169	155/155	90/99	161/161	172/172
368/368	233/237	432/437	198/198	246/252	165/171	152/152	157/157	168/168	266/278	163/169	155/155	90/99	161/161	172/172
368/368	233/237	432/437	198/198	246/252	165/171	152/152	157/157	168/168	266/278	163/169	155/155	90/99	161/161	172/172
368/368	233/237	432/437	198/198	246/252	165/171	152/152	157/157	168/168	266/278	163/169	155/155	90/99	161/161	172/172
369/371	233/237	432/442	192/198	246/252	165/165	146/146	157/157	168/170	266/278	163/163	149/149	90/90	156/161	172/172
368/375	233/233	437/442	192/198	246/246	153/165	146/146	157/157	168/175	261/272	151/163	149/149	90/99	161/161	172/172
370/370	233/233	437/446	192/198	246/246	153/165	146/152	157/157	168/175	261/272	151/163	149/155	80/99	161/161	172/172
368/368	233/237	432/437	198/198	246/252	165/171	146/152	157/157	168/168	272/278	163/169	149/155	80/99	156/161	172/172
368/370	233/233	437/437	198/198	246/246	165/165	146/152	157/157	168/168	266/272	163/163	149/155	99/99	156/161	172/172
368/369	233/237	432/437	192/198	246/252	165/165	152/152	157/157	168/168	272/278	163/163	155/155	95/99	156/161	172/172
368/369	233/237	432/437	192/198	246/252	165/165	152/152	157/157	168/168	272/278	163/163	155/155	95/99	156/161	172/172
368/369	233/237	432/437	192/198	246/252	165/165	152/152	157/157	168/168	272/278	163/163	155/155	95/99	156/161	172/172
368/369	233/237	432/437	192/198	246/252	165/165	152/152	157/157	168/168	272/278	163/163	155/155	95/99	156/161	172/172
368/369	233/237	432/437	192/198	246/252	165/165	152/152	157/157	168/168	272/278	163/163	155/155	95/99	156/161	172/172
370/371	233/233	437/442	192/198	246/246	165/177	140/152	157/164	168/175	266/272	163/175	143/155	80/99	156/161	172/172
366/369	237/237	432/432	192/192	252/252	171/171	146/152	157/157	168/168	278/284	169/169	149/155	80/90	156/156	172/172
370/370	233/233	437/442	198/198	246/246	165/165	146/152	157/157	168/168	266/272	163/163	149/155	90/99	161/161	172/172
368/370	233/233	437/437	198/198	246/252	165/178	146/146	157/157	168/175	261/272	163/175	149/149	85/99	156/161	172/172
366/368	233/237	432/437	192/198	246/252	165/177	146/152	157/164	168/172	266/278	163/175	149/155	90/99	156/161	172/172
367/369	233/237	432/437	192/198	246/252	165/178	146/152	157/164	168/172	266/278	163/175	149/155	90/99	156/161	172/172
366/368	233/237	432/437	192/198	246/252	165/177	146/152	157/164	168/172	266/278	163/175	149/155	90/99	156/161	172/172
366/368	233/237	432/437	192/198	246/252	165/177	146/152	157/164	168/172	266/278	163/175	149/155	90/99	156/161	172/172
366/368	233/237	432/437	192/198	246/252	165/177	146/152	157/164	168/172	266/278	163/175	149/155	90/99	156/161	172/172
366/368	233/237	432/437	192/198	246/252	165/177	146/152	157/164	168/172	266/278	163/175	149/155	90/99	156/161	172/172
366/368	233/237	432/437	192/198	246/252	165/177	146/152	157/164	168/172	266/278	163/175	149/155	90/99	156/161	172/172
366/368	233/237	432/437	192/198	246/252	165/177	146/152	157/164	168/172	266/278	163/175	149/155	90/99	156/161	172/172
366/368	233/237	432/437	192/198	246/252	165/177	146/152	157/164	168/172	266/278	163/175	149/155	90/99	156/161	172/172
366/368	233/237	432/437	192/198	246/252	165/177	146/152	157/164	168/172	266/278	163/175	149/155	90/99	156/161	172/172
368/368	233/237	432/437	192/198	246/252	165/177	146/152	157/164	168/172	266/278	163/175	149/155	90/99	156/161	172/172
366/368	233/237	432/437	192/198	246/252	165/177	146/152	157/164	168/172	266/278	163/175	149/155	90/99	156/161	172/172
366/368	233/237	432/437	192/198	246/252	165/177	146/152	157/164	168/172	266/278	163/175	149/155	90/99	156/161	172/172
366/368	233/237	432/437	192/198	246/252	165/177	146/152	157/164	168/172	266/278	163/175	149/155	90/99	156/161	172/172
366/368	233/237	432/437	192/198	246/252	165/177	146/152	157/164	168/172	266/278	163/175	149/155	90/99	156/161	172/172
366/368	233/237	432/437	192/198	246/252	165/177	146/152	157/164	168/172	266/278	163/175	149/155	90/99	156/161	172/172
366/368	233/237	432/437	192/198	246/252	165/177	146/152	157/164	168/172	266/278	163/175	149/155	90/99	156/161	172/172
366/368	233/237	432/437	192/198	246/252	165/177	146/152	157/164	168/172	266/278	163/175	149/155	90/99	156/161	172/172
366/368	233/237	432/437	192/198	246/252	165/177	146/152	157/164	168/172	266/278	163/175	149/155	90/99	156/161	172/172
366/368	233/237	432/437	192/198	246/252	165/177	146/152	157/164	168/172	266/278	163/175	149/155	90/99	156/161	172/172
370/371	233/233	442/442	192/198	246/246	153/165	146/146	157/157	175/175	261/261	151/163	149/149	80/90	161/161	172/174
370/371	233/233	442/442	192/198	246/246	153/153	146/146	157/157	168/175	261/266	151/151	149/149	80/80	161/161	172/174
370/371	233/233	442/442	192/198	246/246	153/153	146/146	157/157	168/175	261/266	151/151	149/149	80/80	161/161	172/174
370/371	233/233	442/442	192/198	246/246	153/153	146/146	157/157	168/175	261/266	151/151	149/149	80/80	161/161	172/174
370/371	233/233	442/442	192/198	246/246	153/165	146/146	157/157	175/175	261/261	151/163	149/149	80/90	161/161	172/174
370/371	233/233	442/442	192/198	246/246	153/165	146/146	157/157	175/175	261/261	151/163	149/149	80/90	161/161	172/174
370/370	233/233	442/442	181/198	246/246	153/165	146/152	157/157	168/175	261/266	151/163	149/155	90/99	161/161	172/172
370/371	233/233	442/442	192/198	246/246	153/153	146/146	157/157	168/175	261/266	151/151	149/149	80/80	161/161	172/174
370/371	233/233	442/442	192/198	246/246	153/153	146/146	157/157	168/175	261/266	151/151	149/149	80/80	161/161	172/174
370/371	233/233	442/442	192/198	246/246	153/153	146/146	157/157	168/175	261/266	151/151	149/149	80/80	161/161	172/174
370/371	233/233	442/442	192/198	246/246	153/165	146/146	157/157	175/175	261/261	151/163	149/149	80/90	161/161	172/174
370/371	233/233	442/442	192/198	246/246	153/153	146/146	157/157	168/175	261/266	151/151	149/149	80/80	161/161	172/174
370/370	233/233	437/442	198/198	246/246	153/165	146/152	157/164	168/175	261/266	151/163	149/155	80/99	156/161	172/172
368/369	237/237	432/432	192/198	252/252	165/171	146/152	157/157	168/170	272/278	163/169	149/155	80/99	156/156	172/172
369/369	233/233	437/442	192/198	246/246	165/171	146/152	157/164	168/172	266/266	163/169	149/155	85/99	161/161	172/172
368/368	233/233	437/442	192/198	246/246	165/171	146/152	157/164	168/172	266/266	163/169	149/155	85/99	161/161	172/172
368/368	233/233	437/442	192/198	246/246	165/171	146/152	157/164	168/172	266/266	163/169	149/155	85/99	161/161	172/172
368/368	233/233	437/442	192/198	246/246	165/171	146/152	157/164	168/172	266/266	163/169	149/155	85/99	161/161	172/172
366/368	233/233	437/437	198/198	246/252	165/165	146/152	157/157	168/168	267/278	163/163	149/155	99/99	156/161	172/172
369/369	233/237	432/437	192/198	246/252	165/171	146/152	157/164	168/172	266/278	163/169	149/155	99/99	156/161	172/172
366/370	233/233	437/437	198/198	246/246	165/177	152/152	157/157	168/168	266/278	163/175	155/155	90/90	156/161	172/172
370/370	233/233	437/442	181/198	246/252	165/178	146/152	157/164	168/175	261/266	163/175	149/155	85/90	156/161	172/172
369/369	233/237	432/437	198/198	246/252	165/171	146/152	157/157	168/168	266/278	163/169	149/155	90/99	156/161	172/172
366/369	237/237	432/432	192/192	252/252	171/171	146/152	157/157	168/168	278/278	169/169	149/155	80/90	156/156	172/176
369/371	233/233	437/442	198/198	246/246	153/165	146/146	157/157	168/175	261/278	151/163	149/149	90/99	161/161	172/172
369/369	233/237	432/437	198/198	246/252	165/171	146/152	157/157	168/168	266/278	163/169	149/155	90/99	156/161	172/172
369/369	233/237	432/437	198/198	246/252	165/171	146/152	157/157	168/168	266/278	163/169	149/155	90/99	156/161	172/172
369/369	233/237	432/437	198/198	246/252	165/171	146/152	157/157	168/168	266/278	163/169	149/155	90/99	156/161	172/172
370/370	237/237	432/432	192/192	252/252	171/171	146/152	157/157	168/168	278/278	169/169	149/155	80/80	156/156	172/172
368/370	233/237	432/437	192/198	246/252	165/171	146/152	157/157	168/168	266/278	163/169	149/155	90/99	156/156	172/172
368/370	232/237	432/442	181/198	246/252	153/171	146/152	157/157	168/175	261/266	151/169	149/155	80/90	156/161	172/172
366/368	237/237	432/432	192/198	246/252	165/171	152/152	157/157	168/168	266/284	163/169	155/155	90/99	156/161	172/172
368/370	233/233	437/442	198/198	246/246	165/165	146/152	157/164	168/172	267/272	163/163	149/155	99/99	156/161	172/172
368/370	233/233	437/442	192/198	246/246	165/165	152/152	157/164	168/168	267/272	163/163	155/155	99/99	161/161	172/172
369/370	233/237	432/437	192/198	246/252	165/171	146/146	157/157	168/168	266/266	163/169	149/149	90/90	156/161	172/172
368/369	233/237	432/437	192/198	246/252	165/171	152/152	157/164	170/172	266/278	163/169	155/155	99/99	156/161	172/172
368/368	233/237	432/437	198/198	246/252	165/171	152/152	157/157	168/168	266/272	163/169	155/155	90/99	156/161	172/172
369/370	233/237	432/437	192/198	246/252	165/171	146/152	157/157	168/170	266/278	163/169	149/155	90/99	156/156	172/172
370/371	233/233	442/442	198/198	246/246	165/165	146/152	157/164	168/172	266/272	163/163	149/155	99/99	161/161	172/172
368/368	233/237	432/437	198/198	246/252	165/165	146/152	157/157	168/168	266/272	163/163	149/155	99/99	156/156	172/172
369/371	232/233	432/437	192/192	246/246	165/177	140/146	157/157	168/175	272/272	163/175	143/149	80/95	156/161	169/172
368/371	233/237	432/437	192/198	252/252	171/171	152/152	157/157	168/168	266/266	169/169	155/155	90/90	161/161	172/172
366/368	233/233	437/442	198/198	246/246	165/165	152/152	157/164	168/172	266/278	163/163	155/155	99/99	161/161	172/172

366/368	233/237	432/437	192/198	246/252	165/171	146/152	157/157	168/168	266/278	163/169	149/155	90/99	161/161	172/172
368/369	233/237	432/437	192/198	246/252	165/165	152/152	157/164	168/172	267/278	163/163	155/155	95/99	156/161	172/172
368/368	233/233	437/437	192/198	246/246	165/165	152/152	157/157	168/168	266/266	163/163	155/155	99/99	161/167	172/172
368/368	233/237	432/437	198/198	246/252	165/165	152/152	164/164	172/172	266/278	163/163	155/155	99/99	156/156	172/172
368/370	233/233	437/437	192/198	246/252	165/165	146/146	164/164	168/172	266/266	163/163	149/149	99/99	161/161	172/172
368/369	237/251	432/432	192/198	240/252	171/171	152/152	157/158	168/175	272/284	169/169	155/155	80/95	156/156	172/172
368/370	233/233	437/442	192/198	246/252	171/177	146/146	157/157	168/168	261/278	169/175	149/149	90/90	156/161	172/172
368/368	233/233	437/442	192/198	246/252	165/165	152/152	157/164	168/172	266/272	163/163	155/155	99/99	161/161	172/172
366/369	237/237	432/432	192/192	252/252	171/171	146/152	157/164	168/168	278/278	169/169	149/155	90/99	156/156	172/172
369/369	237/237	432/432	192/192	252/252	171/171	146/152	157/157	168/168	278/284	169/169	149/155	90/90	156/156	172/172
366/368	233/233	437/437	192/198	246/252	165/165	146/152	157/164	168/172	278/278	163/163	149/155	99/99	156/156	172/172
368/370	233/233	437/442	192/192	246/246	165/165	152/152	157/164	168/168	266/272	163/163	155/155	99/99	161/167	172/172
368/371	233/237	432/437	192/198	246/246	165/165	152/152	157/157	168/168	272/278	163/163	155/155	99/99	156/161	172/172
368/371	233/233	437/442	192/198	246/252	165/171	146/152	157/157	168/168	272/278	163/169	149/155	90/99	156/161	172/172
368/368	233/237	432/442	198/198	246/252	165/165	146/152	157/164	168/172	266/278	163/163	149/155	99/99	156/161	172/172
370/371	233/233	437/437	192/198	246/246	165/165	146/152	164/164	168/172	266/266	163/163	149/155	99/99	161/167	172/172
368/370	233/233	437/442	192/198	252/252	171/171	146/152	157/157	168/168	261/278	169/169	149/155	90/90	156/161	172/172
368/370	233/237	432/437	192/192	246/246	153/165	146/152	157/157	168/168	261/278	151/163	149/155	80/99	156/156	172/172
368/370	233/233	437/437	192/198	246/246	165/165	146/146	157/164	168/172	266/278	163/163	149/149	99/99	161/161	172/172
368/370	233/233	437/437	198/198	246/252	171/171	152/152	157/157	168/168	266/266	169/169	155/155	85/90	161/161	172/172
366/370	237/237	432/432	192/192	252/252	171/171	146/146	157/157	168/168	278/278	169/169	149/149	90/90	156/156	172/172
368/370	233/233	437/442	198/198	246/246	165/171	146/152	157/157	168/170	278/278	163/169	149/155	90/99	156/161	172/172
366/368	233/233	437/442	192/198	246/246	165/165	146/152	157/164	168/172	266/278	163/163	149/155	99/99	156/161	172/172
370/370	233/233	437/437	192/198	246/246	165/165	152/152	157/157	168/168	267/272	163/163	155/155	99/104	161/161	172/172
368/370	233/233	437/437	192/198	246/246	165/171	146/152	157/157	168/168	266/266	163/169	149/155	90/99	156/161	172/172
368/370	233/237	432/437	198/198	246/252	165/165	152/152	157/157	168/168	266/272	163/163	155/155	99/99	161/161	172/172
368/371	233/233	437/442	192/192	246/246	165/165	146/152	157/157	168/168	266/272	163/163	149/155	99/99	156/167	172/172
370/370	233/233	437/437	192/192	246/246	165/165	152/152	157/164	168/168	266/267	163/163	155/155	99/99	161/167	172/172
368/368	233/233	437/437	192/198	246/252	165/171	146/152	157/157	168/168	266/278	163/169	149/155	90/99	161/161	172/172
368/370	233/237	432/442	192/192	246/252	153/171	146/146	157/157	168/168	261/278	151/169	149/149	80/90	156/161	172/172
368/371	233/233	437/442	192/198	246/246	165/165	152/152	157/164	168/172	266/278	163/163	155/155	99/99	156/161	172/172
369/369	233/237	432/437	192/192	252/252	171/171	146/152	157/157	168/168	266/278	169/169	149/155	90/99	156/156	172/172
368/368	233/237	432/437	198/198	246/252	165/165	152/152	157/157	168/168	266/266	163/163	155/155	99/99	156/161	172/172
368/370	233/233	437/437	192/198	246/252	165/171	152/152	157/164	168/168	266/266	163/169	155/155	90/99	161/161	172/172
367/372	233/237	432/437	192/198	246/252	165/171	152/152	157/164	168/172	266/278	163/169	155/155	90/99	161/161	172/172
368/368	233/237	432/437	192/198	246/252	165/165	152/152	157/157	168/168	278/278	163/163	155/155	99/99	156/161	172/172
368/368	233/233	437/437	198/198	246/252	171/177	146/152	157/164	168/172	266/278	169/175	149/155	90/90	156/161	172/172
368/371	233/233	437/437	192/198	246/246	165/165	152/152	157/157	168/168	272/278	163/163	155/155	99/99	156/161	172/172
368/370	233/233	437/437	192/198	246/246	165/165	146/152	157/157	168/168	266/272	163/163	149/155	99/104	156/161	172/172
369/369	233/237	432/437	192/198	240/252	171/171	146/152	157/158	168/175	278/278	169/169	149/155	80/95	156/156	172/176
368/371	233/233	437/442	192/198	246/252	165/171	146/152	164/164	172/172	266/278	163/169	149/155	90/99	156/161	172/172
366/368	233/237	432/437	192/198	246/252	165/171	146/152	157/164	168/172	266/278	163/169	149/155	90/99	161/161	172/172
366/371	233/237	432/442	198/198	246/252	165/171	152/152	157/164	168/172	266/278	163/169	155/155	90/99	161/161	172/172
366/369	233/237	432/437	192/198	246/252	165/171	152/152	157/157	168/168	278/284	163/169	155/155	90/99	156/161	172/172
366/368	233/233	437/442	198/198	246/246	165/165	152/152	164/164	172/172	278/278	163/163	155/155	99/99	156/161	172/172
366/368	233/233	437/437	192/198	246/246	165/171	146/152	164/164	172/172	266/278	163/169	149/155	90/99	156/161	172/172
366/370	233/237	432/437	192/198	246/252	165/165	152/152	157/157	168/168	267/278	163/163	155/155	95/99	156/161	172/172
370/371	233/233	442/442	192/192	246/246	165/165	146/152	157/157	168/168	266/266	163/163	149/155	99/99	161/167	172/172
366/368	233/233	437/442	198/198	246/246	165/165	152/152	157/164	168/172	266/278	163/163	155/155	99/99	156/161	172/172
368/371	233/237	432/437	198/198	246/252	165/165	152/152	157/157	168/168	266/272	163/163	155/155	99/99	156/161	172/172
368/371	233/233	437/437	192/198	246/252	165/171	146/152	157/157	168/168	266/267	163/169	149/155	90/99	161/161	172/172
366/368	233/237	432/437	192/198	246/252	171/171	146/152	157/157	168/168	266/278	169/169	149/155	90/90	161/161	172/172
368/368	233/237	432/437	198/198	246/252	165/165	152/152	157/157	168/168	266/266	163/163	155/155	99/99	161/167	172/172
366/371	233/237	432/437	198/198	246/252	165/171	146/152	157/164	168/172	266/278	163/169	149/155	90/99	161/161	172/172
368/371	233/233	437/437	192/192	246/246	171/171	146/152	157/164	168/172	266/278	169/169	149/155	85/90	161/161	172/174
368/371	233/233	437/437	192/198	246/252	165/171	146/152	157/157	168/168	266/267	163/169	149/155	90/99	161/161	172/172
368/369	233/237	432/437	192/198	246/246	165/171	152/152	157/157	168/168	266/278	163/169	155/155	90/99	156/156	172/172
368/369	233/237	432/437	192/192	246/252	171/171	146/152	157/157	168/168	266/278	169/169	149/155	80/90	156/161	172/172
368/368	233/237	432/437	192/198	246/252	165/171	152/152	157/157	168/168	266/278	163/169	155/155	90/99	156/161	172/172
368/368	233/233	437/437	192/198	246/246	165/165	152/152	157/157	168/168	267/278	163/163	155/155	99/104	161/161	172/172
368/369	237/237	432/432	192/192	246/252	171/171	152/152	157/157	168/168	266/278	169/169	155/155	90/90	156/156	172/172
368/368	233/233	437/437	192/192	246/246	165/165	146/152	157/157	168/168	266/278	163/163	149/155	99/99	161/167	172/172
368/368	233/233	437/437	192/198	246/246	165/165	146/152	157/157	168/168	266/278	163/163	149/155	99/99	161/161	172/172
368/368	233/233	437/437	192/198	246/246	165/165	146/152	157/157	168/168	266/266	163/163	149/155	99/99	161/161	172/172
368/370	233/233	437/437	198/198	246/246	165/165	152/152	157/157	168/168	266/267	163/163	155/155	99/104	161/161	172/172
368/369	233/237	432/437	192/192	246/252	171/171	146/152	157/157	168/168	266/278	169/169	149/155	90/90	156/161	172/172
368/370	233/233	437/437	198/198	246/246	165/165	146/152	157/157	168/168	266/266	163/163	149/155	99/99	161/161	172/172
368/368	233/233	437/442	192/198	246/252	165/171	146/152	157/164	168/172	266/278	163/169	149/155	90/99	156/161	172/172
368/368	233/237	432/437	192/198	246/252	165/165	152/152	157/157	168/168	266/278	163/163	155/155	99/99	161/167	172/172
368/369	233/251	432/437	198/198	240/252	171/171	152/158	157/158	168/175	272/278	169/169	155/161	90/95	156/156	172/176
368/368	233/233	437/437	198/198	246/252	165/177	152/152	157/164	168/172	266/266	163/175	155/155	90/99	161/161	172/172
368/368	233/233	437/437	192/198	246/252	165/165	146/152	157/157	168/168	266/278	163/163	149/155	99/99	161/161	172/172
368/368	233/233	437/437	198/198	246/246	165/171	146/152	157/157	168/168	266/278	163/169	149/155	90/99	161/161	172/172
368/368	233/237	432/437	198/198	246/252	165/165	152/152	157/157	168/168	266/284	163/163	155/155	99/99	156/161	172/172
368/368	233/237	432/437	198/198	252/252	165/171	146/152	157/164	168/168	266/278	163/169	149/155	90/99	161/161	172/172
368/371	233/233	437/437	192/198	246/246	165/165	152/152	157/157	168/168	278/278	163/163	155/155	99/99	156/161	172/172
368/370	233/233	437/437	192/198	246/246	165/165	146/152	157/157	168/168	266/266	163/163	149/155	99/99	161/167	172/172
368/368	233/237	432/437	192/198	246/252	165/165	146/146	157/164	168/172	278/278	163/163	149/149	99/99	156/161	172/172
368/368	233/233	437/442	198/198	246/252	165/165	146/152	157/164	168/172	266/278	163/163	149/155	99/99	156/167	172/172
369/370	233/237	432/437	192/198	246/252	165/171	146/152	157/157	168/168	261/272	163/169	149/155	90/95	156/156	172/180
368/368	233/237	432/437	198/198	246/252	165/171	152/152	157/164	168/172	266/278	163/169	155/155	90/99	156/161	172/172
368/368	233/237	432/437	192/192	246/246	165/165	152/152	157/164	168/172	266/278	163/163	155/155	99/99	161/161	172/172
368/368	233/233	437/437	198/198	246/246	165/171	152/152	157/157	168/170	266/278	163/169	155/155	90/99	156/156	172/172
366/368	233/233	437/437	198/198	246/252	165/165	146/152	157/157	168/168	278/278	163/163	149/155	99/99	156/161	172/172
368/368	233/233	437/437	198/198	246/246	171/177	146/152	157/157	168/168	278/278	169/175	149/155	90/90	156/161	172/172
368/368	233/233	437/437	198/198	246/246	165/165	152/152	157/157	168/168	266/266	163/163	155/155	99/99	161/161	172/172
368/370	233/233	437/437	198/198	246/252	165/171	146/152	157/157	168/168	267/278	163/169	149/155	90/104	161/161	172/172
368/370	233/233	437/437	198/198	246/246	165/171	152/152	157/157	168/168	266/267	163/169	155/155	90/104	161/161	172/172
368/368	233/233	437/442	192/198	246/252	165/171	146/152	157/157	168/168	261/278	163/169	149/155	90/99	161/161	172/172
368/368	233/233	437/442	198/198	246/252	165/165	146/152	157/164	168/172	266/278	163/163	149/155	99/99	156/161	172/172
366/368	233/233	437/437	198/198	246/246	165/165	152/152	157/157	168/168	266/278	163/163	155/155	99/99	156/161	172/172
366/370	237/237	432/432	192/192	252/252	171/171	146/146	157/164	168/168	278/278	169/169	149/149	90/90	156/156	172/172
368/370	233/233	437/442	198/198	246/252	165/171	152/152	157/157	168/168	266/266	163/169	155/155	90/99	161/161	172/172
368/368	233/237	432/437	198/198	246/246	165/165	146/152	157/164	168/172	266/278	163/163	149/155	99/99	161/161	172/172
368/368	233/233	437/437	192/198	246/246	165/171	152/152	157/157	168/168	266/272	163/169	155/155	90/99	161/161	172/172
368/370	233/233	437/437	192/198	246/246	165/165	152/152	157/164	168/172	266/272	163/163	155/155	99/99	161/167	172/172
368/370	233/233	437/437	192/198	246/252	165/177	152/152	157/164	168/168	266/266	163/175	155/155	90/99	161/161	172/172
368/368	233/237	432/437	198/198	246/252	165/165	146/152	157/157	168/168	266/266	163/163	149/155	99/99	161/161	172/172
368/370	233/233	437/442	198/198	246/246	165/171	146/146	157/164	168/172	266/266	163/169	149/149	90/99	161/161	172/172
368/370	233/237	432/437	192/198	246/246	165/171	152/152	157/157	168/168	272/278	163/169	155/155	90/99	161/167	172/172
368/370	233/233	437/442	192/192	246/246	165/165	152/152	157/157	168/168	266/266	163/163	155/155	99/99	167/167	172/172
368/370	233/237	432/437	198/198	246/252	171/177	146/152	157/157	168/168	266/278	169/175	149/155	90/90	156/161	172/172
368/368	233/233	437/442	192/198	246/252	165/165	146/152	157/157	168/168	266/278	163/163	149/155	99/99	161/161	172/172
368/368	233/233	437/442	192/198	246/246	165/165	146/152	157/157	168/168	266/266	163/163	149/155	99/99	161/161	172/172
368/370	233/233	437/437	192/198	246/246	165/165	152/152	157/164	168/168	266/266	163/163	155/155	99/99	161/161	172/172
368/368	233/233	437/437	198/198	246/246	165/165	152/152	157/164	168/172	266/278	163/163	155/155	99/99	161/161	172/172
368/370	233/233	437/437	192/198	246/246	165/165	152/152	157/157	168/168	272/278	163/163	155/155	99/99	161/167	172/172
366/368	233/237	432/437	198/198	246/252	165/177	146/152	157/157	168/168	266/266	163/175	149/155	90/99	156/161	172/172

368/371	233/237	432/437	198/198	246/252	165/171	146/146	157/157	168/168	266/278	163/169	149/149	90/99	161/161	172/172
368/368	233/237	432/437	198/198	246/252	165/171	152/152	157/164	168/172	266/278	163/169	155/155	90/99	156/156	172/172
366/368	233/233	437/437	192/198	246/252	165/165	146/152	157/164	168/172	278/278	163/163	149/155	99/99	156/161	172/172
368/370	233/233	437/437	192/198	246/246	165/165	152/152	157/164	168/172	266/278	163/163	155/155	99/99	156/167	172/172
368/368	233/233	437/437	198/198	246/246	165/165	152/152	157/164	168/172	266/284	163/163	155/155	99/99	161/161	172/172
368/368	233/233	437/442	198/198	246/246	165/165	146/146	157/164	168/172	266/278	163/163	149/149	99/99	161/161	172/172
368/368	233/233	437/437	192/198	246/252	165/165	152/152	157/164	168/172	278/278	163/163	155/155	99/99	156/167	172/172
368/368	233/233	437/437	192/198	246/246	165/165	146/152	157/157	168/168	266/272	163/163	149/155	99/99	161/167	172/172
368/368	233/233	437/437	192/198	246/246	165/165	152/152	157/164	168/168	272/278	163/163	155/155	99/99	161/161	172/172
368/368	233/233	437/437	198/198	246/246	165/171	146/152	157/164	168/172	278/278	163/169	149/155	90/99	156/161	172/172
368/370	233/233	437/437	198/198	246/246	165/165	146/152	157/157	168/168	266/278	163/163	149/155	99/99	161/161	172/172
368/371	233/233	437/442	192/198	246/246	165/165	152/152	157/164	168/172	278/278	163/163	155/155	99/99	156/161	172/172
368/368	233/233	437/442	192/198	246/252	165/171	152/152	157/164	168/172	266/266	163/169	155/155	90/99	161/161	172/172
368/370	233/237	432/437	198/198	252/252	171/171	146/152	157/164	168/172	266/278	169/169	149/155	80/90	156/156	172/172
368/368	233/237	432/437	192/198	246/252	165/171	146/152	157/157	168/168	266/278	163/169	149/155	90/99	156/161	172/172
368/368	237/237	432/432	198/198	246/252	165/177	146/152	157/164	168/172	266/278	163/175	149/155	90/99	161/161	172/172
368/368	233/237	432/437	198/198	246/252	165/171	152/152	157/157	168/168	272/278	163/169	155/155	90/99	156/161	172/172
368/368	233/233	437/437	198/198	246/246	165/165	152/152	157/157	168/168	272/278	163/163	155/155	99/99	156/161	172/172
368/370	233/237	432/437	192/192	246/252	153/177	146/146	157/157	168/168	278/278	151/175	149/149	80/90	161/161	172/172
368/368	233/237	432/442	192/198	246/252	153/177	146/152	157/164	168/172	261/266	151/175	149/155	80/90	156/161	172/172
368/370	233/237	432/437	192/198	246/252	153/177	146/152	157/157	168/168	261/278	151/175	149/155	80/90	156/156	172/172
368/371	233/237	432/442	198/198	246/252	165/171	152/152	157/164	168/172	266/278	163/169	155/155	90/99	161/161	172/172
368/370	233/233	437/437	198/198	246/252	165/165	146/152	157/157	168/168	272/278	163/163	149/155	99/99	156/161	172/172
368/368	233/237	432/437	198/198	246/252	165/177	152/152	157/164	168/172	278/278	163/175	155/155	90/99	156/161	172/172
368/368	233/237	432/437	192/198	246/252	171/171	152/152	157/157	168/168	278/278	169/169	155/155	90/90	161/161	172/172
368/370	233/237	432/437	198/198	252/252	165/171	152/152	157/157	168/168	266/278	163/169	155/155	80/99	156/161	172/172
368/368	233/237	432/437	192/198	246/252	165/171	152/152	157/157	168/168	278/278	163/169	155/155	90/99	156/161	172/172
368/370	233/233	437/442	198/198	246/246	165/171	146/152	157/164	170/172	266/266	163/169	149/155	90/99	156/161	172/172
366/368	237/237	432/432	192/198	252/252	171/171	146/152	157/157	168/170	278/284	169/169	149/155	90/99	156/156	172/172
368/369	233/237	432/437	192/198	246/252	171/177	146/152	157/157	168/168	266/278	169/175	149/155	90/90	156/161	172/172
368/370	233/233	437/437	198/198	246/246	165/165	146/152	157/157	168/168	267/278	163/163	149/155	99/104	161/161	172/172
368/368	233/237	432/437	198/198	246/252	165/177	152/152	157/157	168/168	266/278	163/175	155/155	90/99	156/161	172/172
368/368	233/233	437/437	198/198	246/252	165/165	146/152	157/157	168/168	267/278	163/163	149/155	99/99	161/161	172/172
368/368	233/233	437/437	198/198	246/252	165/171	152/152	157/157	168/168	267/278	163/169	155/155	90/104	161/161	172/172
368/368	233/233	437/437	192/198	246/246	165/165	152/152	157/157	168/168	266/278	163/163	155/155	99/99	161/161	172/172
368/368	233/233	437/437	198/198	252/252	171/171	146/146	157/164	168/168	278/278	169/169	149/149	90/90	161/161	172/172
368/370	233/233	437/437	192/198	246/252	165/171	146/152	157/164	168/168	266/278	163/169	149/155	90/99	161/161	172/172
368/368	233/237	432/437	192/198	246/246	165/165	152/152	157/164	168/172	266/278	163/163	155/155	99/99	161/161	172/172
369/371	233/237	432/437	192/198	246/252	165/171	146/152	164/164	168/172	266/266	163/169	149/155	90/99	161/161	172/172
368/369	233/237	432/437	192/198	246/252	165/171	146/152	157/157	168/168	266/284	163/169	149/155	90/99	156/161	172/172
368/368	233/233	437/442	192/198	246/246	165/165	146/152	157/164	168/172	278/278	163/163	149/155	99/99	156/161	172/172
368/371	233/233	437/437	192/198	246/246	165/165	146/152	157/164	168/172	266/266	163/163	149/155	99/99	161/161	172/174
368/369	233/237	432/437	192/192	252/252	171/171	146/152	157/157	168/168	278/284	169/169	149/155	90/95	156/156	172/172
368/369	233/237	432/437	192/198	252/252	171/171	146/152	157/157	168/168	278/284	169/169	149/155	90/95	156/161	172/172
368/368	233/233	437/442	192/198	246/246	165/165	152/152	157/157	168/168	266/278	163/163	155/155	99/99	156/156	172/172
368/370	233/233	437/437	192/198	246/252	165/171	146/146	157/164	168/168	278/278	163/169	149/149	90/99	161/161	172/172
368/368	233/233	437/437	192/198	246/252	171/171	146/152	157/158	168/175	266/266	169/169	149/155	90/95	161/161	172/172
368/368	233/237	432/437	192/198	246/252	153/165	146/146	157/157	168/168	266/278	151/163	149/149	80/99	156/161	172/172
368/368	233/233	437/442	198/198	246/252	165/165	146/152	157/164	168/172	266/266	163/163	149/155	99/99	156/156	172/172
368/369	233/237	432/437	192/198	246/252	165/165	152/152	157/157	168/168	266/278	163/163	155/155	99/99	156/161	172/172
368/370	233/233	437/442	192/198	246/252	165/165	146/146	157/157	168/168	266/278	163/163	149/149	99/99	161/161	172/172
369/369	233/237	432/437	198/198	246/246	165/165	146/146	164/164	172/172	266/278	163/163	149/149	99/99	156/161	172/172
368/368	233/233	437/437	192/198	246/246	165/165	146/152	157/164	168/172	266/266	163/163	149/155	99/99	161/161	172/172
