# Supplementary material for: Hybrid Origins of Citrus Varieties Inferred from DNA Marker Analysis of Nuclear and Organelle Genomes
Source: PLoS One. 2016 Nov 30;11(11):e0166969. doi: 10.1371/journal.pone.0166969 (PMC5130255; doi:10.1371/journal.pone.0166969)
Supplement: S4 Table — (PDF) [file pone.0166969.s007.pdf]

S4 Table. Summary of DNA marker inconsistency for known parent-offspring trios

#	Marker name	Synthetic varieties		Selected lines		Selection
		Inconsisted trios	ND in data set	Inconsisted trios	ND in data set	
1	CTVR01	0	0	0	0	*
2	CTVR04	0	0	0	0	**
3	CUBER403	5	5	2	0	
4	CUBER410	0	0	0	0	*
5	CUBER601	7	0	2	0	
6	CUBER602	0	0	0	0	**
7	CUBER903	8	0	17	0	
8	CUBER918	0	0	0	0	*
9	CUBER920	0	0	0	0	*
10	CUBER925	2	2	5	5	
11	CUBER935	0	0	0	0	**
12	CUBER939	0	0	0	0	*
13	CUBER951	0	0	1	1	*
14	GRW2011	0	0	0	0	*
15	GRW2051	1	0	1	0	
16	GRW3021	9	0	8	0	
17	GRW3011	0	0	0	0	*
18	GSR10S09	0	0	0	0	*
19	GSR10S17	0	0	0	0	*
20	GSR10S29	0	0	0	0	*
21	GSR2101	0	0	0	0	
22	GSR2103	0	0	0	0	
23	GSR2108	0	0	0	0	
24	GSR2129	0	0	0	0	
25	GSR2131	0	0	1	1	
26	GSR2150	16	0	16	0	
27	GSR3114	4	0	10	0	
28	GSR3121	0	0	0	0	*
29	GSR3126	5	2	2	1	
30	GSR3128	0	0	0	0	*
31	GSR3136	0	0	0	0	*
32	GSR3137	1	0	1	1	
33	GSR3138	0	0	1	1	*
34	GSR3140	0	0	0	0	*
35	GSR3141	10	2	7	1	
36	GSR5105	2	0	6	0	
37	GSR5112	2	1	2	0	
38	GSR5114	2	0	2	1	
39	GSR5122	1	0	0	0	
40	GSR5127	4	6	6	5	
41	GSR5129	1	0	1	0	
42	GSR6101	0	0	1	1	*
43	GSR6122	0	0	0	0	*
44	GSR6129	0	0	0	0	*
45	GSR6133	0	0	0	0	*
46	GSR6135	0	0	0	0	*
47	NSX02	2	0	0	0	
48	NSX23	0	0	0	0	*
49	NSX32	0	0	0	0	*
50	NSX34	3	0	1	0	

51	NSX38	0	0	0	0	*
52	NSX39	0	0	0	0	*
53	NSX43	0	0	0	0	*
54	NSX48	12	0	11	0	
55	NSX53	7	8	10	2	
56	NSX67	1	0	3	0	
57	NSX79	1	0	0	0	
58	NSX82	0	0	0	0	*
59	NSX84	0	0	0	0	*
60	NSX115	0	0	0	0	*
61	NSX119	1	0	1	0	
62	NSX121	0	0	0	0	*
63	NSX132	0	0	0	0	*
64	NSX137	0	0	1	1	*
65	NSX141	0	0	1	1	*
66	NSX145	0	0	1	1	*
67	NSX150	1	1	0	0	*
68	NSX153	0	0	0	0	*
69	NSX156	0	0	0	0	*
70	NSX160	0	0	0	0	*
71	NSX162	0	0	0	0	*
72	NSX165	0	0	0	0	*
73	NSX169	0	0	0	0	*
74	NSX170	0	0	0	0	*
75	NSX175	4	0	1	0	
76	NSX186	1	0	0	0	
77	NSX187	0	0	0	0	*
78	SRPE02	0	0	0	0	*
79	SRPE03	1	0	0	0	
80	SRPE05	0	0	0	0	*
81	TSRA101	0	0	0	0	*
82	TSRA103	0	0	0	0	*
83	TSRA107	0	0	1	0	
84	TSRA108	3	1	1	1	
85	TSRA109	1	0	0	0	
86	TSRA110	0	0	0	0	*
87	TSRA117	0	0	0	0	*
88	TSGR222	1	0	0	0	
89	TSGR264	0	0	0	0	*
90	TSGR265	0	0	0	0	**
91	TSGR271	0	0	0	0	*
92	TSGR282	6	0	8	1	
93	NSX16	1	0	0	0	
94	NSX27	0	0	0	0	
95	NSX28	3	0	0	0	
96	NSX70	0	0	0	0	*
97	NSX87	10	9	10	2	
98	NSX94	6	0	10	0	
99	NSX118	0	0	0	0	*
100	NSX129	0	0	4	0	
101	NSX148	2	0	0	0	
102	NSX161	0	0	0	0	*
103	NSX179	0	0	1	0	
104	TSA10A29	20	2	28	1	
105	SSR07A05	0	0	0	0	*
106	SSR07A07	0	0	0	0	*
107	SSR07A14	0	0	0	0	*

108	SSR07A17	5	0	1	0	
109	SSR07A19	0	0	1	1	*
110	SSR07A23	0	0	0	0	*
111	SSR07A24	0	0	0	0	*
112	SSR07A20	0	0	0	0	*
113	SSR07B01	1	1	0	0	*
114	SSR07B05	6	0	3	0	
115	SSR07B08	1	1	0	0	*
116	SSR07B09	2	0	1	0	
117	SSR07B14	0	0	0	0	*
118	SSR07B27	0	0	0	0	*
119	SSR07B25	2	1	0	0	
120	SSR07B30	5	0	3	0	
121	SSR08A02	0	0	0	0	*
122	SSR08A06	0	0	0	0	*
123	SSR08A07	1	0	0	0	
124	SSR08A09	0	0	0	0	*
125	SSR08A14	1	0	0	0	
126	SSR08A15	0	0	0	0	*
127	SSR08A16	0	0	0	0	*
128	SSR08B02	14	1	31	0	
129	SSR08B06	0	0	0	0	*
130	SSR08B13	4	0	13	0	
131	SSR08B15	0	0	0	0	*
132	SSR08B20	0	0	0	0	*
133	SSR08B21	0	0	0	0	**
134	SSR08B22	0	0	1	0	
135	SSR08B25	0	0	0	0	**
136	SSR08B27	0	0	0	0	*
137	SSR08B28	0	0	0	0	*
138	SSR08B29	0	0	0	0	*
139	SSR08B31	2	0	0	0	
140	SSR08B32	0	0	0	0	*
141	SSR08B35	0	0	0	0	**
142	SSR08B38	1	0	1	0	
143	SSR08B40	0	0	0	0	**
144	SSR08B44	1	0	1	0	
145	SSR08B47	1	0	1	0	
146	SSR08B54	0	0	0	0	**
147	SSR08B60	0	0	0	0	*
148	SSR08B62	0	0	0	0	*
149	SSR08B64	1	0	0	0	
150	SSR08B66	0	0	0	0	*
151	SSR08B68	0	0	0	0	*
152	SSR08B75	0	0	0	0	*
153	SSR08B78	0	0	0	0	*
154	SSR08B82	1	0	0	0	
155	SSR08B84	0	0	0	0	*
156	SSR08B85	0	0	0	0	*
157	SSR08B88	0	0	0	0	*
158	SSR08B90	0	0	0	0	*
159	SSR08B92	0	0	0	0	*
160	SSR08B95	0	0	0	0	*
161	SSR11A06	0	0	0	0	*
162	SSR11B14	3	0	0	0	
163	SSR11A27	0	0	0	0	*
164	TSGR901	0	0	0	0	**

165	TSGR904	0	0	1	1	*
166	TSGR905	0	0	0	0	*
167	TSGR906	15	0	26	0	
168	TSR8EM15	2	2	1	1	*
169	TSR8EM24	0	0	0	0	*
170	TSR8EM28	4	1	2	1	
171	TSRB07	4	0	7	1	
172	TSRB09	0	0	0	0	*
173	TSRB11	0	0	0	0	*
174	TSRB29	0	0	0	0	*
175	TSRB38	2	2	0	0	*
176	TSRB39	0	0	0	0	*
177	TSRF101	0	0	0	0	*
178	TSRF105	0	0	0	0	*
179	TSRF116	0	0	0	0	*
180	TSRF120	5	0	5	1	
181	TSRF124	1	1	0	0	*
182	TSRF136	1	1	1	1	*
183	TSRF138	0	0	0	0	*
184	TSRF144	0	0	0	0	*
185	TSRF157	0	0	0	0	*
186	TSRF160	0	0	0	0	*
187	TSRF161	0	0	1	1	*
188	TSRF162	0	0	0	0	*
189	TSRF164	0	0	0	0	*
190	TSRF167	0	0	0	0	*
191	TSRF169	0	0	0	0	*
192	TSRF172	0	0	1	1	
193	TSRF184	1	1	0	0	*
194	TSRF187	0	0	0	0	*
195	TSRF191	0	0	0	0	**
196	TSRF192	0	0	0	0	*
197	TSRF194	0	0	0	0	*
198	TSRF195	0	0	0	0	*
199	TSRF198	0	0	0	0	*
200	TSRF204	0	0	0	0	*
201	TSRF208	0	0	0	0	*
202	TSRF211	1	1	0	0	*
203	TSRF215	0	0	0	0	*
204	TSRF218	0	0	0	0	*
205	TSRF206	0	0	0	0	**
206	TSRF225	0	0	0	0	*
207	TSRF229	0	0	0	0	*
208	TSRF230	1	1	0	0	*
209	TSRF234	0	0	1	1	**
210	TSRF237	2	2	0	0	*
211	TSRF238	0	0	0	0	
212	TSRN214	0	0	0	0	*
213	TSRP06	0	0	0	0	*
214	TSRP07	0	0	0	0	*
215	CiBE0246	0	0	0	0	*
216	CiBE0447	0	0	0	0	*
217	CiBE1644	0	0	1	1	*
218	CiBE2165	0	0	0	0	*
219	CiBE2227	0	0	0	0	*
220	CiBE4265	0	0	0	0	*
221	CX0004	0	0	0	0	*

222	CX0010	0	0	0	0	
223	CX0035	0	0	1	1	*
224	CX2004	0	0	0	0	*
225	CX2018	0	0	1	0	
226	CX2021	0	0	0	0	
227	CX2040	0	0	0	0	*
228	CX3001	0	0	0	0	*
229	CX3040	9	0	10	0	
230	CX4005	0	0	0	0	*
231	CX4008	0	0	0	0	
232	CX4040	6	0	1	0	
233	CX5022	0	0	0	0	**
234	CX5039	0	0	0	0	*
235	CX6024	0	0	0	0	*
236	CX6035	0	0	0	0	*
237	CX6037	0	0	0	0	*
238	CX6F21	0	0	0	0	*
239	CX6F32	0	0	0	0	
240	F02	0	0	0	0	*
241	F03	0	0	0	0	**
242	F16	0	0	0	0	**
243	F21	0	0	1	1	
244	F40	0	0	0	0	*
245	F50	0	0	0	0	*
246	F79	0	0	0	0	*
