# Supplementary material for: Hybrid Origins of Citrus Varieties Inferred from DNA Marker Analysis of Nuclear and Organelle Genomes
Source: PLoS One. 2016 Nov 30;11(11):e0166969. doi: 10.1371/journal.pone.0166969 (PMC5130255; doi:10.1371/journal.pone.0166969)
Supplement: S5 Table — (PDF) [file pone.0166969.s008.pdf]

S5 Table. Summary of matched genotypes between a pair of samples by the 169 validated markers

ID	Rep	Variety name	*H*	Matched (<5)	ND	A001	A002	A003	A004	A005	A006	A007	A008	A009	A010	A011	A012	A013	A014	A015	A016	A017	A018	A019	A020	A021	A022	A023	A024	A025	A026	A027	A028	A029	A030	A031	A032	A033	A034	A035	A036	A037	A038
				Matched(<5)		2	1	1	1	1	1	8	1	4	4	4	4	1	1	1	2	2	1	1	2	1	1	2	3	3	3	1	1	2	2	1	1	2	2	1	6	6	6
					Variety name	Andoukan	Anseikan	Asahikan	Banpeiyu	Ben Di Zao	Bergamot	Bendi Guangju	Binkitsu	Clementine	Clementine	Clementine	Clementine	Cleopatra	Cravo mandarin	Dada	Dancy	Dancy	Egami buntan	Fukure mikan	Fukushukan	Funadoko	Genshokan	Girimikan	Grapefruit	Grapefruit	Grapefruit	Hanayu	Hassaku	Hebesu	Henka mikan	Hickson	Hirado buntan	Hiroshimanatsubuntan	Houraikan	Hyoukan	Hyuganatsu	Hyuganatsu	Hyuganatsu
					ND	0	0	0	0	0	0	0	0	0	0	0	0	0	0	0	0	0	1	0	0	0	0	0	0	0	0	2	0	0	0	0	0	0	0	0	0	0	0
A001	*	Andoukan	0.769	2	0	0	126	85	134	132	129	87	109	134	134	134	134	134	128	98	137	137	137	120	131	105	139	121	87	87	87	145	84	135	127	132	129	105	85	96	105	105	105
A002	*	Anseikan	0.462	1	0	126	0	107	91	145	126	137	136	142	142	142	142	145	148	126	142	142	97	145	132	115	141	142	112	112	112	145	114	135	139	146	87	98	130	95	139	139	139
A003	*	Asahikan	0.716	1	0	85	107	0	126	134	129	95	120	138	138	138	138	145	137	114	145	145	133	130	138	102	141	128	100	100	100	145	80	132	132	140	129	94	87	91	118	118	118
A004	*	Banpeiyu	0.272	1	0	134	91	126	0	143	128	139	142	140	140	140	140	140	145	135	140	140	77	148	140	126	134	142	112	112	112	149	120	144	146	145	64	108	135	124	138	138	138
A005	*	Ben Di Zao	0.467	1	0	132	145	134	143	0	143	110	114	99	99	99	99	91	100	136	81	81	148	100	119	105	91	111	131	131	131	126	136	124	119	98	143	131	115	136	129	129	129
A006	*	Bergamot	0.550	1	0	129	126	129	128	143	0	135	135	130	130	130	130	127	143	119	139	139	125	134	123	135	137	138	134	134	134	140	136	134	133	128	130	111	130	134	132	132	132
A007		Bendi Guangju	0.751	8	0	87	137	95	139	110	135	0	109	118	118	118	118	127	114	114	119	119	143	124	128	97	125	120	111	111	111	137	93	119	118	116	138	122	60	100	94	94	94
A008	*	Binkitsu	0.722	1	0	109	136	120	142	114	135	109	0	122	122	122	122	135	125	116	123	123	145	115	123	110	125	106	122	122	122	139	120	128	117	124	144	126	114	124	113	113	113
A009	*	Clementine	0.426	4	0	134	142	138	140	99	130	118	122	0	0	0	0	92	73	130	83	83	146	121	124	117	90	122	132	132	132	129	136	124	125	87	146	128	119	138	136	136	136
A010		Clementine	0.426	4	0	134	142	138	140	99	130	118	122	0	0	0	0	92	73	130	83	83	146	121	124	117	90	122	132	132	132	129	136	124	125	87	146	128	119	138	136	136	136
A011		Clementine	0.426	4	0	134	142	138	140	99	130	118	122	0	0	0	0	92	73	130	83	83	146	121	124	117	90	122	132	132	132	129	136	124	125	87	146	128	119	138	136	136	136
A012		Clementine	0.426	4	0	134	142	138	140	99	130	118	122	0	0	0	0	92	73	130	83	83	146	121	124	117	90	122	132	132	132	129	136	124	125	87	146	128	119	138	136	136	136
A013	*	Cleopatra	0.266	1	0	134	145	145	140	91	127	127	135	92	92	92	92	0	99	139	78	78	143	100	118	121	73	112	136	136	136	120	141	125	118	86	136	135	128	136	131	131	131
A014	*	Cravo mandarin	0.479	1	0	128	148	137	145	100	143	114	125	73	73	73	73	99	0	132	81	81	150	111	121	113	93	123	121	121	121	130	132	119	125	89	143	134	112	139	126	126	126
A015	*	Dada	0.734	1	0	98	126	114	135	136	119	114	116	130	130	130	130	139	132	0	140	140	135	130	113	107	141	128	111	111	111	133	115	128	127	137	135	112	105	116	98	98	98
A016	*	Dancy	0.367	2	0	137	142	145	140	81	139	119	123	83	83	83	83	78	81	140	0	0	154	105	117	122	66	117	141	141	141	128	143	128	128	71	142	130	128	141	133	133	133
A017		Dancy	0.367	2	0	137	142	145	140	81	139	119	123	83	83	83	83	78	81	140	0	0	154	105	117	122	66	117	141	141	141	128	143	128	128	71	142	130	128	141	133	133	133
A018	*	Egami buntan	0.231	1	1	137	97	133	77	148	125	143	145	146	146	146	146	143	150	135	154	154	0	151	144	138	145	152	117	117	117	151	130	145	146	147	69	118	133	128	141	141	141
A019	*	Fukure mikan	0.550	1	0	120	145	130	148	100	134	124	115	121	121	121	121	100	111	130	105	105	151	0	115	130	105	102	136	136	136	126	141	124	117	112	145	137	116	138	131	131	131
A020		Fukushukan	0.627	2	0	131	132	138	140	119	123	128	123	124	124	124	124	118	121	113	117	117	144	115	0	133	116	96	139	139	139	109	136	132	117	118	141	130	123	128	115	115	115
A021	*	Funadoko	0.633	1	0	105	115	102	126	105	135	97	110	117	117	117	117	121	113	107	122	122	138	130	133	0	123	132	104	104	104	140	100	120	126	123	129	111	100	103	111	111	111
A022	*	Genshokan	0.385	1	0	139	141	141	134	91	137	125	125	90	90	90	90	73	93	141	66	66	145	105	116	123	0	111	132	132	132	127	138	129	122	87	137	135	128	138	134	134	134
A023	*	Girimikan	0.627	2	0	121	142	128	142	111	138	120	106	122	122	122	122	112	123	128	117	117	152	102	96	132	111	0	128	128	128	114	130	126	120	122	147	133	128	134	121	121	121
A024	*	Grapefruit	0.669	3	0	87	112	100	112	131	134	111	122	132	132	132	132	136	121	111	141	141	117	136	139	104	132	128	0	0	0	151	85	132	132	141	119	111	107	83	122	122	122
A025		Grapefruit	0.669	3	0	87	112	100	112	131	134	111	122	132	132	132	132	136	121	111	141	141	117	136	139	104	132	128	0	0	0	151	85	132	132	141	119	111	107	83	122	122	122
A026		Grapefruit	0.669	3	0	87	112	100	112	131	134	111	122	132	132	132	132	136	121	111	141	141	117	136	139	104	132	128	0	0	0	151	85	132	132	141	119	111	107	83	122	122	122
A027	*	Hanayu	0.574	1	2	145	145	145	149	126	140	137	139	129	129	129	129	120	130	133	128	128	151	126	109	140	127	114	151	151	151	0	141	118	113	127	152	142	132	146	138	138	138
A028	*	Hassaku	0.704	1	0	84	114	80	120	136	136	93	120	136	136	136	136	141	132	115	143	143	130	141	136	100	138	130	85	85	85	141	0	133	128	143	126	104	90	82	110	110	110
A029		Hebesu	0.698	2	0	135	135	132	144	124	134	119	128	124	124	124	124	125	119	128	128	128	145	124	132	120	129	126	132	132	132	118	133	0	93	125	141	128	118	126	127	127	127
A030	*	Henka mikan	0.621	2	0	127	139	132	146	119	133	118	117	125	125	125	125	118	125	127	128	128	146	117	117	126	122	120	132	132	132	113	128	93	0	120	143	136	117	118	122	122	122
A031	*	Hickson	0.385	1	0	132	146	140	145	98	128	116	124	87	87	87	87	86	89	137	71	71	147	112	118	123	87	122	141	141	141	127	143	125	120	0	146	134	118	140	131	131	131
A032	*	Hirado buntan	0.314	1	0	129	87	129	64	143	130	138	144	146	146	146	146	136	143	135	142	142	69	145	141	129	137	147	119	119	119	152	126	141	143	146	0	103	134	121	140	140	140
A033	*	Hiroshimanatsubuntan	0.615	2	0	105	98	94	108	131	111	122	126	128	128	128	128	135	134	112	130	130	118	137	130	111	135	133	111	111	111	142	104	128	136	134	103	0	106	105	129	129	129
A034		Houraikan	0.704	2	0	85	130	87	135	115	130	60	114	119	119	119	119	128	112	105	128	128	133	116	123	100	128	128	107	107	107	132	90	118	117	118	134	106	0	97	105	105	105
A035	*	Hyoukan	0.669	1	0	96	95	91	124	136	134	100	124	138	138	138	138	136	139	116	141	141	128	138	128	103	138	134	83	83	83	146	82	126	118	140	121	105	97	0	118	118	118
A036	*	Hyuganatsu	0.728	6	0	105	139	118	138	129	132	94	113	136	136	136	136	131	126	98	133	133	141	131	115	111	134	121	122	122	122	138	110	127	122	131	140	129	105	118	0	0	0
A037		Hyuganatsu	0.728	6	0	105	139	118	138	129	132	94	113	136	136	136	136	131	126	98	133	133	141	131	115	111	134	121	122	122	122	138	110	127	122	131	140	129	105	118	0	0	0
A038		Hyuganatsu	0.728	6	0	105	139	118	138	129	132	94	113	136	136	136	136	131	126	98	133	133	141	131	115	111	134	121	122	122	122	138	110	127	122	131	140	129	105	118	0	0	0
A039		Hyuganatsu	0.734	6	0	105	140	118	138	129	132	94	113	136	136	136	136	131	126	99	133	133	142	131	116	111	134	122	122	122	122	138	110	127	122	131	141	129	105	119	1	1	1
A040		Hyuganatsu	0.728	6	0	105	139	118	138	129	132	94	113	136	136	136	136	131	126	98	133	133	141	131	115	111	134	121	122	122	122	138	110	127	122	131	140	129	105	118	0	0	0
A041	*	Ichanchii	0.586	1	1	140	137	138	132	130	126	140	143	130	130	130	130	128	130	131	139	139	140	135	124	136	131	130	132	132	132	123	140	132	129	136	137	126	137	139	141	141	141
A042	*	Ichang lemon	0.686	1	0	127	131	132	129	145	131	129	134	137	137	137	137	147	135	120	136	136	133	140	126	137	140	130	130	130	130	132	130	114	134	144	126	115	129	131	124	124	124
A043		Iyo	0.675	2	2	57	121	103	139	116	131	93	127	125	125	125	125	124	116	102	115	115	142	126	136	102	124	133	88	88	88	144	97	126	133	136	134	113	95	108	116	116	116
A044	*	Iyo	0.686	2	0	57	120	102	139	115	131	92	127	125	125	125	125	124	116	102	115	115	142	125	136	100	124	131	88	88	88	144	97	126	133	136	134	112	95	107	116	116	116
A045	*	Jabara	0.680	1	2	137	134	136	143	128	139	123	128	131	131	131	131	133	131	134	128	128	142	128	130	130	127	126	139	139	139	127	136	75	97	133	139	128	120	129	132	132	132
A046	*	Jabbon	0.663	1	0	110	128	125	136	129	129	115	114	126	126	126	126	130	128	113	136	136	126	127	127	115	135	121	122	122	122	129	122	109	105	130	123	118	106	119	123	123	123
A047	*	Kabosu	0.639	1	0	133	138	130	140	118	125	116	128	117	117	117	117	117	127	126	126	126	143	121	124	125	132	122	135	135	135	121	133	65	107	122	137	126	117	128	131	131	131
A048	*	Kabuchi	0.657	1	0	127	138	123	147	120	141	104	115	128	128	128	128	120	121	121	123	123	149	99	119	116	125	107	133	133	133	131	126	120	130	122	143	138	112	135	98	98	98
A049	*	Kaikoukan	0.609	1	0	97	86	81	102	142	140	111	120	141	141	141	141	144	145	124	143	143	115	139	138	108	142	133	107	107	107	143	77	135	137	144	107	97	110	92	124	124	124
A050	*	Kawabata	0.615	1	0	105	132	117	133	124	139	94	95	116	116	116	116	124	107	111	121	121	133	117	127	108	122	118	118	118	118	133	100	123	126	126	137	130	100	112	105	105	105
A051	*	Kawachi bankan	0.592	1	0	101	95	97	101	137	124	117	119	131	131	131	131	137	133	109	139	139	108	137	130	108	140	135	96	96	96	142	109	131	133	136	110	100	95	92	119	119	119
A052	*	Keraji	0.609	2	0	121	142	125	148	106	141	83	116	123	123	123	123	120	115	125	123	123	143	115	112	117	119	96	133	133	133	124	116	125	116	109	142	135	102	124	116	116	116
A053	*	Kikudaidai	0.722	1	0	119	133	129	135	136	135	124	122	132	132	132	132	139	129	111	133	133	142	129	122	121	131	133	130	130	130	128	126	129	131	134	139	128	124	133	102	102	102
A054	*	King mandarin	0.574	1	0	121	134	127	134	108	138	99	107	101	101	101	101	105	92	123	101	101	140	117	116	109	100	115	121	121	121	126	112	124	113	103	137	121	106	123	111	111	111
A055	*	Kinkoji	0.663	1	0	102	106	86	113	129	132	98	122	134	134	134	134	134	129	112	142	142	120	136	134	101	134	137	93	93	93	141	73	128	132	143	115	110	98	90	114	114	114
A056		Kinukawa	0.757	2	2	2	126	86	134	132	130	89	109	134	134	134	134	134	129	100	137	137	137	120	131	107	139	121	89	89	89	145	86	135	127	132	129	105	87	97	107	107	107

A057		Kishu mandarin	0.462	16	0	129	146	135	148	77	134	110	114	109	109	109	109	78	104	135	90	90	152	79	125	116	90	111	136	136	136	129	135	114	117	89	148	136	117	134	128	128	128
A058		Kishu mandarin	0.462	16	1	129	146	135	148	77	134	110	114	109	109	109	109	79	104	135	90	90	152	80	125	116	91	112	136	136	136	129	135	114	117	89	148	136	117	134	128	128	128
A059	*	Kishu mandarin	0.462	16	0	129	146	135	148	77	134	110	114	109	109	109	109	78	104	135	90	90	152	79	125	116	90	111	136	136	136	129	135	114	117	89	148	136	117	134	128	128	128
A060		Kishu mandarin	0.462	16	0	129	146	135	148	77	134	110	114	109	109	109	109	78	104	135	90	90	152	79	125	116	90	111	136	136	136	129	135	114	117	89	148	136	117	134	128	128	128
A061		Kishu mandarin	0.462	16	0	129	146	135	148	77	134	110	114	109	109	109	109	78	104	135	90	90	152	79	125	116	90	111	136	136	136	129	135	114	117	89	148	136	117	134	128	128	128
A062		Kishu mandarin	0.462	16	0	129	146	135	148	77	134	110	114	109	109	109	109	78	104	135	90	90	152	79	125	116	90	111	136	136	136	129	135	114	117	89	148	136	117	134	128	128	128
A063		Kishu mandarin	0.462	16	0	129	146	135	148	77	134	110	114	109	109	109	109	78	104	135	90	90	152	79	125	116	90	111	136	136	136	129	135	114	117	89	148	136	117	134	128	128	128
A064		Kishu mandarin	0.462	16	0	129	146	135	148	77	134	110	114	109	109	109	109	78	104	135	90	90	152	79	125	116	90	111	136	136	136	129	135	114	117	89	148	136	117	134	128	128	128
A065		Kishu mandarin	0.462	16	0	129	146	135	148	77	134	110	114	109	109	109	109	78	104	135	90	90	152	79	125	116	90	111	136	136	136	129	135	114	117	89	148	136	117	134	128	128	128
A066		Kishu mandarin	0.462	16	0	129	146	135	148	77	134	110	114	109	109	109	109	78	104	135	90	90	152	79	125	116	90	111	136	136	136	129	135	114	117	89	148	136	117	134	128	128	128
A067		Kishu mandarin	0.462	16	0	129	146	135	148	77	134	110	114	109	109	109	109	78	104	135	90	90	152	79	125	116	90	111	136	136	136	129	135	114	117	89	148	136	117	134	128	128	128
A068		Kishu mandarin	0.462	16	0	129	146	135	148	77	134	110	114	109	109	109	109	78	104	135	90	90	152	79	125	116	90	111	136	136	136	129	135	114	117	89	148	136	117	134	128	128	128
A069		Kishu mandarin	0.462	16	0	129	146	135	148	77	134	110	114	109	109	109	109	78	104	135	90	90	152	79	125	116	90	111	136	136	136	129	135	114	117	89	148	136	117	134	128	128	128
A070		Kishu mandarin	0.462	16	0	129	146	135	148	77	134	110	114	109	109	109	109	78	104	135	90	90	152	79	125	116	90	111	136	136	136	129	135	114	117	89	148	136	117	134	128	128	128
A071		Kishu mandarin	0.462	16	0	129	146	135	148	77	134	110	114	109	109	109	109	78	104	135	90	90	152	79	125	116	90	111	136	136	136	129	135	114	117	89	148	136	117	134	128	128	128
A072		Kishu mandarin	0.462	16	0	129	146	135	148	77	134	110	114	109	109	109	109	78	104	135	90	90	152	79	125	116	90	111	136	136	136	129	135	114	117	89	148	136	117	134	128	128	128
A073	*	Kizu	0.698	2	0	135	135	132	144	124	134	119	128	124	124	124	124	125	119	128	128	128	145	124	132	120	129	126	132	132	132	118	133	0	93	125	141	128	118	126	127	127	127
A075	*	Kobeni mikan	0.396	1	0	131	148	138	150	81	137	113	124	94	94	94	94	77	96	135	89	89	151	102	123	125	87	111	137	137	137	127	148	128	122	97	146	138	120	144	131	131	131
A076	*	Koji	0.609	3	0	130	141	130	143	112	131	123	112	119	119	119	119	105	124	129	112	112	150	79	112	127	112	75	130	130	130	121	133	128	131	111	147	128	129	132	122	122	122
A077		Komikan 2009-130	0.598	3	0	131	140	130	143	113	130	123	110	118	118	118	118	106	125	128	111	111	150	81	112	128	111	76	131	131	131	121	134	128	132	110	147	128	128	133	122	122	122
A078		Konejime	0.680	2	0	98	130	106	134	129	117	107	118	123	123	123	123	131	122	97	138	138	134	131	124	107	132	133	103	103	103	142	104	123	119	126	133	112	97	105	113	113	113
A079	*	Kourai Tachibana	0.586	1	1	134	143	140	149	120	128	136	133	128	128	128	128	117	130	132	129	129	149	117	115	133	126	118	142	142	142	82	142	103	110	121	151	139	131	139	133	133	133
A080	*	Kotokan	0.799	1	0	85	118	100	136	136	130	102	121	127	127	127	127	135	123	102	133	133	143	129	123	108	124	125	105	105	105	141	103	133	125	139	132	107	93	109	116	116	116
A081	*	Kunenbo-A	0.751	8	0	87	137	95	139	110	135	0	109	118	118	118	118	127	114	114	119	119	143	124	128	97	125	120	111	111	111	137	93	119	118	116	138	122	60	100	94	94	94
A082	*	Kunenbo-B	0.568	1	0	126	138	134	138	109	124	133	120	117	117	117	117	111	113	117	119	119	141	105	76	126	119	88	138	138	138	112	134	136	121	115	138	125	125	133	121	121	121
A083		Kunenbo	0.751	8	1	87	138	95	139	110	136	1	110	118	118	118	118	127	114	115	119	119	143	124	128	97	125	120	111	111	111	137	93	120	118	116	138	122	61	100	95	95	95
A084		Kunenbo	0.746	8	1	87	137	96	139	110	135	1	109	118	118	118	118	127	114	114	119	119	143	124	128	97	125	120	111	111	111	137	93	120	118	116	138	122	60	100	95	95	95
A085	*	Lemon	0.633	1	0	136	131	133	142	142	84	139	139	135	135	135	135	133	144	132	141	141	139	137	128	141	140	139	140	140	140	136	144	142	147	138	141	130	135	138	137	137	137
A086	*	Lemonade	0.598	1	0	120	133	120	134	143	106	128	133	132	132	132	132	129	135	120	142	142	132	137	135	129	137	138	128	128	128	133	132	133	134	128	135	118	121	130	132	132	132
A087	*	Limonia	0.621	1	0	135	146	135	150	138	119	136	143	134	134	134	134	123	133	141	137	137	148	132	135	132	134	136	142	142	142	135	138	129	133	127	152	136	127	142	143	143	143
A088	*	Mato buntan	0.308	1	0	136	85	129	68	144	123	145	145	142	142	142	142	140	148	136	145	145	70	144	137	129	145	144	114	114	114	146	129	145	146	144	79	110	135	120	142	142	142
A089	*	Mexican lime	0.533	1	1	156	140	154	142	150	126	154	154	145	145	145	145	137	150	146	147	147	140	147	144	146	144	146	149	149	149	144	150	147	144	142	142	145	148	147	149	149	149
A090	*	Meyer lemon	0.669	1	0	129	144	140	146	139	130	135	135	139	139	139	139	137	125	136	140	140	148	141	131	138	139	134	128	128	128	139	136	131	127	139	146	133	130	135	135	135	135
A091	*	Mochiyu	0.698	1	1	134	145	133	147	116	137	106	128	124	124	124	124	132	120	132	126	126	146	122	124	125	127	119	137	137	137	108	128	87	83	121	150	136	117	131	119	119	119
A092	*	Murcott	0.432	1	0	126	141	124	136	94	132	104	106	101	101	101	101	101	95	123	79	79	142	112	119	128	98	109	121	121	121	130	131	129	120	92	144	129	116	134	114	114	114
A093		Myrttle leaf orange	0.722	6	0	100	124	101	128	133	89	114	122	122	122	122	122	134	125	98	139	139	128	132	112	105	138	132	110	110	110	137	104	129	125	136	135	80	103	111	116	116	116
A094		Nansho daidai	0.621	2	0	127	139	132	146	119	133	118	117	125	125	125	125	118	125	127	128	128	146	117	117	126	122	120	132	132	132	113	128	93	0	120	143	136	117	118	122	122	122
A095	*	Naruto	0.781	1	0	59	121	97	129	139	131	90	107	130	130	130	130	141	129	102	135	135	134	130	131	104	139	129	93	93	93	147	85	135	127	138	129	104	82	100	110	110	110
A096		Natsudaidai	0.769	4	0	71	114	81	130	129	131	87	111	128	128	128	128	138	119	102	129	129	135	124	135	93	139	125	90	90	90	148	87	119	126	133	134	97	82	91	109	109	109
A097		Natsudaidai	0.769	4	0	71	114	81	130	129	131	87	111	128	128	128	128	138	119	102	129	129	135	124	135	93	139	125	90	90	90	148	87	119	126	133	134	97	82	91	109	109	109
A098	*	Natsudaidai	0.769	4	0	71	114	81	130	129	131	87	111	128	128	128	128	138	119	102	129	129	135	124	135	93	139	125	90	90	90	148	87	119	126	133	134	97	82	91	109	109	109
A099		Natsudaidai	0.769	4	0	71	114	81	130	129	131	87	111	128	128	128	128	138	119	102	129	129	135	124	135	93	139	125	90	90	90	148	87	119	126	133	134	97	82	91	109	109	109
A100	*	Nidonari mikan	0.521	1	0	114	141	125	139	97	131	112	130	121	121	121	121	94	103	122	103	103	141	103	118	113	102	132	132	132	132	134	131	129	125	102	141	133	107	128	129	129	129
A101	*	Oogonkan	0.639	1	0	116	134	108	146	117	122	106	119	123	123	123	123	114	120	104	118	118	150	122	124	109	124	115	125	125	125	137	119	119	128	115	142	110	108	115	103	103	103
A102		Ootachibana	0.515	2	0	119	89	93	97	139	132	134	136	143	143	143	143	139	146	119	147	147	96	139	138	116	144	138	103	103	103	148	111	133	139	150	94	106	123	101	128	128	128
A103	*	Ootoukan	0.686	1	0	105	121	102	134	108	134	111	117	121	121	121	121	130	122	118	126	126	135	135	136	96	130	135	98	98	98	141	88	125	136	143	137	106	107	99	116	116	116
A104	*	Oukan	0.680	1	0	83	123	94	147	118	132	106	119	118	118	118	118	126	126	109	133	133	145	118	129	112	134	121	113	113	113	135	105	133	123	136	139	125	108	99	118	118	118
A105		Ponkan	0.521	4	0	136	149	141	148	98	133	122	118	86	86	86	86	86	89	143	66	66	154	108	126	126	83	130	135	135	135	132	137	119	128	67	147	133	120	144	127	127	127
A106		Ponkan	0.521	4	0	136	149	141	148	98	133	122	118	86	86	86	86	86	89	143	66	66	154	108	126	126	83	130	135	135	135	132	137	119	128	67	147	133	120	144	127	127	127
A107	*	Ponkan	0.521	4	0	136	149	141	148	98	133	122	118	86	86	86	86	86	89	143	66	66	154	108	126	126	83	130	135	135	135	132	137	119	128	67	147	133	120	144	127	127	127
A108		Ponkan	0.521	4	0	136	149	141	148	98	133	122	118	86	86	86	86	86	89	143	66	66	154	108	126	126	83	130	135	135	135	132	137	119	128	67	147	133	120	144	127	127	127
A109	*	Ponkitsu	0.391	1	0	122	143	133	141	107	133	109	122	104	104	104	104	94	104	131	84	84	144	112	122	111	82	118	130	130	130	129	132	131	122	84	142	126	113	135	122	122	122
A110	*	Pummelo whitetype	0.183	1	1	135	90	129	62	147	129	143	140	146	146	146	146	143	145	138	148	148	69	144	141	133	145	143	116	116	116	153	127	146	139	147	60	115	137	122	137	137	137
A111	*	Rokugatsumikan	0.627	2	0	131	132	138	140	119	123	128	123	124	124	124	124	118	121	113	117	117	144	115	0	133	116	96	139	139	139	109	136	132	117	118	141	130	123	128	115	115	115
A112	*	Sanbokan	0.740	1	0	63	123	88	132	127	125	90	108	129	129	129	129	132	127	98	139	139	138	127	119	95	136	120	99	99	99	140	92	126	120	134	136	107	77	88	101	101	101
A113		Satsuma mandarin	0.544	21	0	91	140	115	143	120	141	68	118	133	133	133	133	120	108	113	120	120	144	114	123	99	119	123	113	113	113	139	120	130	112	116	141	128	91	115	104	104	104
A114		Satsuma mandarin	0.550	21	0	92	140	116	144	121	141	69	118	133	133	133	133	121	108	113	120	120	145	114	123	100	119	123	114	114	114	139	121	130	112	117	142	129	92	116	104	104	104
A115		Satsuma mandarin	0.544	21	0	91	140	115	143	120	141	68	118	133	133	133	133	120	108	113	120	120	144	114	123	99	119	123	113	113	113	139	120	130	112	116	141	128	91	115	104	104	104
A116		Satsuma mandarin	0.544	21	0	91	140	115	143	120	141	68	118	133	133	133	133	120	108	113	120	120	144	114	123	99	119	123	113	113	113	139	120	130	112	116	141	128	91	115	104	104	104
A117		Satsuma mandarin	0.544	21	0	91	140	115	143	120	141	68	118	133	133	133	133	120	108	113	120	120	144	114	123	99	119	123	113	113	113	139	120	130	112	116	141	128	91	115	104	104	104
A118		Satsuma mandarin	0.544	21	0	91	140	115	143	120	141	68	118	133	133	133	133	120	108	113	120	120	144	114	123	99	119	123	113	113	113	139	120	130	112	116	141	128	91	115	104	104	104
A119		Satsuma mandarin	0.544	21	0	91	140	115	143	120	141	68	118	133	133	133	133	120	108	113	120	120	144	114	123	99	119	123	113	113	113	139	120	130	112	116	141	128	91	115	104	104	104
A120		Satsuma mandarin	0.544	21	0	91	140	115	143	120	141	68	118	133	133	133	133	120	108	113	120	120	144	114	123	99	119	123	113	113	113	139	120	130	112	116	141	128	91	115	104	104	104
A121		Satsuma mandarin	0.544	21	0	91	140	115	143	120	141	68	118	133	133	133	133	120	108	113	120	120	144	114	123	99	119	123	113	113	113	139	120	130	112	116	141	128	91	115	104	104	104
A122		Satsuma mandarin	0.544	21	0	91	140	115	143	120	141	68	118	133	133	133	133	120	108	113	120	120	144	114	123	99	119	123	113	113	113	139	120	130	112	116	141	128	91	115	104	104	104
A123		Satsuma mandarin	0.544	21	0	91	140	115	143	120	141	68	118	133	133	133	133	120	108	113	120	120	144	114	123	99	119	123	113	113	113	139	120	130	112	116	141	128	91	115	104	104	104
A124		Satsuma mandarin	0.544	21	0	91	140	115	143	120	141	68	118	133	133	133	133	120	108	113	120	120	144	114	123	99	119	123	113	113	113	139	120	130	112	116	141	128	91	115	104	104	104
A125	*	Satsuma mandarin	0.544	21	0	91	140	115	143	120	141	68	118	133	133	133	133	120	108	113	120	120	144	114	123	99	119	123	113	113	113	139	120	130	112	116	141	128	91	115	104	104	104
A126		Satsuma mandarin	0.544	21	0	91	140	115	143	120	141	68	118	133	133	133	133	120	108	113	120	120	144	114	123	99	119	123	113	113	113	139	120	130	112	116	141	128	91	115	104	104	104
A127		Satsuma mandarin	0.544	21	0	91	140	115	143	120	141	68	118	133	133	133	133	120	108	113	120	120	144	114	123	99	119	123	113	113	113	139	120	130	112	116	141	128	91	115	104	104	104
A128		Satsuma mandarin	0.544	21	0	91	140	115	143	120	141	68	118	133	133	133	133	120	108	113	120	120	144	114	123	99	119	123	113	113	113	139	120	130	112	116	141	128	91	115	104	104	104
A129		Satsuma mandarin	0.544	21	0	91	140	115	143	120	141	68	118	133	133	133	133	120	108	113	120	120	144	114	123	99	119	123	113	113	113	139	120	130	112	116	141	128	91	115	104	104	104
A130		Satsuma mandarin	0.544	21	0	91	140	115	143	120	141	68	118	133	133	133	133	120	108	113	120	120	144	114	123	99	119	123	113	113	113	139	120	130	112	116	141	128	91	115	104	104	104
A131		Satsuma mandarin	0.544	21	0	91	140	115	143	120	141	68	118	133	133	133	133	120	108	113	120	120	144	114	123	99	119	123	113	113	113	139	120	130	112	116	141	128	91	115	104	104	104
A132		Satsuma mandarin	0.544	21	0	91	140	115	143	120	141	68	118	133	133	133	133	120	108	113	120	120	144	114	123	99	119	123	113	113	113	139	120	130	112	116	141	128	91	115	104	104	104

A133		Satsuma mandarin	0.544	21	0	91	140	115	143	120	141	68	118	133	133	133	133	120	108	113	120	120	144	114	123	99	119	123	113	113	113	139	120	130	112	116	141	128	91	115	104	104	104
A134	*	Satsuma Kikoku	0.680	2	0	98	130	106	134	129	117	107	118	123	123	123	123	131	122	97	138	138	134	131	124	107	132	133	103	103	103	142	104	123	119	126	133	112	97	105	113	113	113
A135	*	Shiikuwasha	0.509	1	0	138	146	142	147	106	127	129	126	112	112	112	112	91	111	133	104	104	145	87	92	133	98	98	143	143	143	105	143	130	119	103	147	140	128	139	126	126	126
A136	*	Shiikuwasha_Ogimikugani	0.503	1	0	140	144	140	145	111	128	133	125	121	121	121	121	93	115	136	108	108	148	95	89	128	105	100	148	148	148	107	140	128	119	107	148	141	129	137	133	133	133
A137	*	Shunkokan	0.686	1	0	76	132	107	133	118	129	85	114	126	126	126	126	121	120	107	123	123	141	111	119	117	123	121	101	101	101	137	98	126	122	120	139	113	80	105	112	112	112
A138	*	Sokitsu	0.432	1	0	136	144	143	146	67	140	116	117	101	101	101	101	87	96	137	89	89	149	100	120	115	88	116	139	139	139	132	142	127	120	100	144	132	121	139	125	125	125
A139		Sour orange	0.728	6	0	99	124	101	128	133	89	114	122	122	122	122	122	135	125	98	139	139	128	132	112	105	138	132	110	110	110	137	104	129	125	136	135	80	103	111	116	116	116
A140		Sour orange	0.728	6	0	99	124	101	128	133	89	114	122	122	122	122	122	135	125	98	139	139	128	132	112	105	138	132	110	110	110	137	104	129	125	136	135	80	103	111	116	116	116
A141	*	Sour orange	0.728	6	0	99	124	101	128	133	89	114	122	122	122	122	122	135	125	98	139	139	128	132	112	105	138	132	110	110	110	137	104	129	125	136	135	80	103	111	116	116	116
A142		Sour orange	0.716	6	2	99	124	101	128	133	91	114	122	123	123	123	123	135	125	98	139	139	128	132	113	105	138	132	110	110	110	137	104	129	125	136	135	81	103	111	116	116	116
A143		Sour orange	0.728	6	0	99	124	101	128	133	89	114	122	122	122	122	122	135	125	98	139	139	128	132	112	105	138	132	110	110	110	137	104	129	125	136	135	80	103	111	116	116	116
A144	*	Sudachi	0.680	1	0	127	136	127	146	116	131	129	126	122	122	122	122	125	128	131	126	126	148	122	126	132	133	115	138	138	138	103	134	77	112	128	147	124	128	131	136	136	136
A145	*	Suisho buntan	0.391	1	0	123	90	115	74	142	134	134	135	142	142	142	142	141	148	125	147	147	76	147	137	118	141	140	111	111	111	148	122	143	143	147	66	110	125	111	136	136	136
A146	*	Sunki	0.349	2	0	130	149	141	148	90	129	123	120	92	92	92	92	72	94	137	68	68	148	86	121	126	77	108	136	136	136	123	142	127	124	81	150	137	120	141	136	136	136
A147	*	Suruga Yuko	0.491	1	0	131	146	139	151	99	132	124	117	117	117	117	117	94	118	132	105	105	149	79	120	124	100	112	138	138	138	128	145	128	123	100	147	138	122	142	127	127	127
A148		Sweet orange	0.740	20	0	103	134	102	131	118	133	90	117	101	101	101	101	133	94	101	115	115	147	122	124	101	124	128	98	98	98	140	97	129	131	123	140	110	101	121	110	110	110
A149		Sweet orange	0.728	20	2	104	134	104	132	118	135	91	118	103	103	103	103	133	95	102	115	115	147	123	124	101	124	128	98	98	98	141	98	129	132	123	141	112	102	121	111	111	111
A150		Sweet orange	0.740	20	0	103	134	102	131	118	133	90	117	101	101	101	101	133	94	101	115	115	147	122	124	101	124	128	98	98	98	140	97	129	131	123	140	110	101	121	110	110	110
A151		Sweet orange	0.740	20	0	103	134	102	131	118	133	90	117	101	101	101	101	133	94	101	115	115	147	122	124	101	124	128	98	98	98	140	97	129	131	123	140	110	101	121	110	110	110
A152		Sweet orange	0.740	20	0	103	134	102	131	118	133	90	117	101	101	101	101	133	94	101	115	115	147	122	124	101	124	128	98	98	98	140	97	129	131	123	140	110	101	121	110	110	110
A153		Sweet orange	0.740	20	0	103	134	102	131	118	133	90	117	101	101	101	101	133	94	101	115	115	147	122	124	101	124	128	98	98	98	140	97	129	131	123	140	110	101	121	110	110	110
A154		Sweet orange	0.740	20	0	103	134	102	131	118	133	90	117	101	101	101	101	133	94	101	115	115	147	122	124	101	124	128	98	98	98	140	97	129	131	123	140	110	101	121	110	110	110
A155		Sweet orange	0.734	20	0	103	134	102	131	118	133	90	117	101	101	101	101	133	94	101	115	115	147	123	124	101	124	128	98	98	98	140	97	129	131	123	140	110	101	121	110	110	110
A156		Sweet orange	0.740	20	0	103	134	102	131	118	133	90	117	101	101	101	101	133	94	101	115	115	147	122	124	101	124	128	98	98	98	140	97	129	131	123	140	110	101	121	110	110	110
A157		Sweet orange	0.740	20	0	103	134	102	131	118	133	90	117	101	101	101	101	133	94	101	115	115	147	122	124	101	124	128	98	98	98	140	97	129	131	123	140	110	101	121	110	110	110
A158		Sweet orange	0.740	20	0	103	134	102	131	118	133	90	117	101	101	101	101	133	94	101	115	115	147	122	124	101	124	128	98	98	98	140	97	129	131	123	140	110	101	121	110	110	110
A159		Sweet orange	0.740	20	0	103	134	102	131	118	133	90	117	101	101	101	101	133	94	101	115	115	147	122	124	101	124	128	98	98	98	140	97	129	131	123	140	110	101	121	110	110	110
A160		Sweet orange	0.740	20	0	103	134	102	131	118	133	90	117	101	101	101	101	133	94	101	115	115	147	122	124	101	124	128	98	98	98	140	97	129	131	123	140	110	101	121	110	110	110
A161		Sweet orange	0.740	20	0	103	134	102	131	118	133	90	117	101	101	101	101	133	94	101	115	115	147	122	124	101	124	128	98	98	98	140	97	129	131	123	140	110	101	121	110	110	110
A162	*	Sweet orange	0.740	20	0	103	134	102	131	118	133	90	117	101	101	101	101	133	94	101	115	115	147	122	124	101	124	128	98	98	98	140	97	129	131	123	140	110	101	121	110	110	110
A163		Sweet orange	0.740	20	0	103	134	102	131	118	133	90	117	101	101	101	101	133	94	101	115	115	147	122	124	101	124	128	98	98	98	140	97	129	131	123	140	110	101	121	110	110	110
A164		Sweet orange	0.740	20	0	103	134	102	131	118	133	90	117	101	101	101	101	133	94	101	115	115	147	122	124	101	124	128	98	98	98	140	97	129	131	123	140	110	101	121	110	110	110
A165		Sweet orange	0.740	20	0	103	134	102	131	118	133	90	117	101	101	101	101	133	94	101	115	115	147	122	124	101	124	128	98	98	98	140	97	129	131	123	140	110	101	121	110	110	110
A166		Sweet orange	0.740	20	0	103	134	102	131	118	133	90	117	101	101	101	101	133	94	101	115	115	147	122	124	101	124	128	98	98	98	140	97	129	131	123	140	110	101	121	110	110	110
A167		Sweet orange	0.746	20	0	103	134	102	130	117	133	89	116	100	100	100	100	133	93	101	114	114	148	122	124	102	124	128	97	97	97	140	98	128	131	122	139	109	101	121	109	109	109
A168		Tachibana	0.408	4	1	141	145	149	149	114	127	141	129	124	124	124	124	100	121	135	116	116	146	100	103	138	104	101	146	146	146	112	143	134	120	111	148	142	139	140	128	128	128
A169		Tachibana	0.426	7	0	141	148	146	145	112	130	138	132	119	119	119	119	95	120	137	116	116	146	91	102	139	100	92	142	142	142	107	143	134	120	110	149	144	139	142	128	128	128
A170		Tachibana	0.426	7	1	141	148	146	145	113	130	138	133	119	119	119	119	96	120	137	116	116	146	91	102	140	101	93	142	142	142	107	143	134	121	110	149	144	139	142	128	128	128
A171		Tachibana	0.426	7	0	141	148	146	145	112	130	138	132	119	119	119	119	95	120	137	116	116	146	91	102	139	100	92	142	142	142	107	143	134	120	110	149	144	139	142	128	128	128
A172	*	Tachibana-A	0.408	4	1	141	145	149	149	114	127	141	129	124	124	124	124	100	121	135	116	116	146	100	103	138	104	101	146	146	146	112	143	134	120	111	148	142	139	140	128	128	128
A173		Tachibana	0.408	4	1	141	145	149	149	114	127	141	129	124	124	124	124	100	121	135	116	116	146	100	103	138	104	101	146	146	146	112	143	134	120	111	148	142	139	140	128	128	128
A174	*	Tachibana-C	0.467	1	0	139	144	144	147	105	130	133	127	115	115	115	115	90	116	136	105	105	148	92	100	133	102	79	148	148	148	114	144	133	123	107	147	144	135	142	136	136	136
A175	*	Tachibana-B	0.426	7	0	141	148	146	145	112	130	138	132	119	119	119	119	95	120	137	116	116	146	91	102	139	100	92	142	142	142	107	143	134	120	110	149	144	139	142	128	128	128
A176		Tachibana	0.426	7	0	141	148	146	145	112	130	138	132	119	119	119	119	95	120	137	116	116	146	91	102	139	100	92	142	142	142	107	143	134	120	110	149	144	139	142	128	128	128
A177		Tachibana	0.426	7	0	141	148	146	145	112	130	138	132	119	119	119	119	95	120	137	116	116	146	91	102	139	100	92	142	142	142	107	143	134	120	110	149	144	139	142	128	128	128
A178		Tachibana	0.408	4	1	141	145	149	149	114	127	141	129	124	124	124	124	100	121	135	116	116	146	100	103	138	104	101	146	146	146	112	143	134	120	111	148	142	139	140	128	128	128
A179		Tachibana	0.426	7	0	141	148	146	145	112	130	138	132	119	119	119	119	95	120	137	116	116	146	91	102	139	100	92	142	142	142	107	143	134	120	110	149	144	139	142	128	128	128
A180		Tajima mikan	0.627	2	0	121	142	128	142	111	138	120	106	122	122	122	122	112	123	128	117	117	152	102	96	132	111	0	128	128	128	114	130	126	120	122	147	133	128	134	121	121	121
A181		Takumanatsukunenbo	0.615	2	0	105	98	94	108	131	111	122	126	128	128	128	128	135	134	112	130	130	118	137	130	111	135	133	111	111	111	142	104	128	136	134	103	0	106	105	129	129	129
A182		Tankan	0.550	4	0	116	137	126	141	111	138	114	108	95	95	95	95	111	94	127	94	94	145	118	124	116	96	116	119	119	119	133	126	125	119	86	146	124	111	132	120	120	120
A183	*	Tankan	0.550	4	0	116	137	126	141	111	138	114	108	95	95	95	95	111	94	127	94	94	145	118	124	116	96	116	119	119	119	133	126	125	119	86	146	124	111	132	120	120	120
A184		Tankan	0.550	4	0	116	137	126	141	111	138	114	108	95	95	95	95	111	94	127	94	94	145	118	124	116	96	116	119	119	119	133	126	125	119	86	146	124	111	132	120	120	120
A185		Tankan	0.550	4	0	116	137	126	141	111	138	114	108	95	95	95	95	111	94	127	94	94	145	118	124	116	96	116	119	119	119	133	126	125	119	86	146	124	111	132	120	120	120
A186	*	Temple	0.568	1	0	115	133	115	134	118	122	105	105	82	82	82	82	112	102	109	104	104	144	121	117	114	101	114	122	122	122	138	120	131	124	98	139	114	117	125	117	117	117
A187	*	Tengu	0.633	1	0	102	110	109	129	124	136	110	127	119	119	119	119	130	105	116	124	124	125	133	139	98	120	135	85	85	85	148	96	124	127	130	129	116	115	93	110	110	110
A188	*	Tizon	0.521	1	0	119	142	122	139	103	121	105	121	99	99	99	99	90	97	115	102	102	141	117	118	116	108	115	129	129	129	133	126	126	131	99	140	119	112	129	112	112	112
A189		Toukan	0.609	3	0	130	141	130	143	112	131	123	112	119	119	119	119	105	124	129	112	112	150	79	112	127	112	75	130	130	130	121	133	128	131	111	147	128	129	132	122	122	122
A190		Tookunin	0.740	8	2	89	137	97	139	111	136	2	110	119	119	119	119	127	115	116	120	120	143	124	130	99	126	121	112	112	112	137	95	119	119	118	138	123	62	100	95	95	95
A191	*	Tosa buntan	0.515	2	0	119	89	93	97	139	132	134	136	143	143	143	143	139	146	119	147	147	96	139	138	116	144	138	103	103	103	148	111	133	139	150	94	106	123	101	128	128	128
A192	*	Twukkuni	0.491	1	1	133	138	137	143	114	126	115	119	118	118	118	118	98	129	122	108	108	141	107	97	123	110	94	139	139	139	117	134	125	115	112	138	135	117	134	117	117	117
A193		Twukkunin	0.751	8	0	87	137	95	139	110	135	0	109	118	118	118	118	127	114	114	119	119	143	124	128	97	125	120	111	111	111	137	93	119	118	116	138	122	60	100	94	94	94
A194		Twukunihu	0.751	8	0	87	137	95	139	110	135	0	109	118	118	118	118	127	114	114	119	119	143	124	128	97	125	120	111	111	111	137	93	119	118	116	138	122	60	100	94	94	94
A195		Twuukuribu	0.751	8	0	87	137	95	139	110	135	0	109	118	118	118	118	127	114	114	119	119	143	124	128	97	125	120	111	111	111	137	93	119	118	116	138	122	60	100	94	94	94
A196	*	Uchimurasaki	0.219	1	0	140	80	131	65	142	133	150	145	144	144	144	144	138	150	141	144	144	69	150	145	124	142	147	121	121	121	152	128	146	146	146	73	109	139	120	143	143	143
A197	*	Ujukitsu	0.704	2	0	85	130	87	135	115	130	60	114	119	119	119	119	128	112	105	128	128	133	116	123	100	128	128	107	107	107	132	90	118	117	118	134	106	0	97	105	105	105
A198	*	Unzoki	0.686	1	0	125	136	128	143	119	141	103	103	137	137	137	137	129	126	110	133	133	143	115	111	110	130	99	129	129	129	136	127	125	122	127	142	129	114	128	93	93	93
A199	*	USSR Tangelo	0.680	1	0	88	114	86	111	131	129	99	115	132	132	132	132	137	134	101	136	136	126	144	127	108	132	128	91	91	91	142	94	136	125	142	114	100	102	91	111	111	111
A200	*	Willowleaf mandarin	0.385	1	0	135	147	145	143	90	133	122	123	86	86	86	86	73	84	137	68	68	154	96	117	122	70	107	139	139	139	126	145	128	130	81	143	135	126	144	134	134	134
A201	*	Willowleaf mandarin	0.414	1	0	132	144	136	143	94	131	121	112	82	82	82	82	74	65	131	72	72	147	100	113	118	70	111	125	125	125	125	135	114	119	86	142	132	117	138	128	128	128
A202	*	Yamabuki	0.746	1	0	58	120	98	134	140	128	100	120	131	131	131	131	137	129	87	145	145	136	134	126	103	146	129	96	96	96	142	97	131	127	141	130	111	91	91	101	101	101
A203	*	Yamamikan	0.746	1	0	92	107	87	137	130	133	113	129	132	132	132	132	138	119	109	134	134	131	134	139	110	133	129	72	72	72	149	91	126	135	144	127	101	106	90	122	122	122
A204	*	Yatsushiro	0.562	1	0	108	143	132	141	98	147	78	117	121	121	121	121	110	115	121	114	114	142	109	133	101	115	126	121	121	121	142	120	123	118	118	137	140	106	116	110	110	110
A205	*	Yuge hyoukan	0.710	1	0	85	113	79	120	137	126	95	117	134	134	134	134	144	134	108	145	145	126	128	137	107	143	130	105	105	105	145	85	137	137	133	132	100	76	90	113	113	113
A206	*	Youpi ju (Yuhikitsu)	0.325	1	0	139	143	140	143	104	128	128	134	95	95	95	95	64	90	137	75	75	150	101	116	126	72	117	145	145	145	115	141	125	119	85	148	137	129	141	135	135	135
A207	*	Yuukunibu	0.633	1	0	23	123	94	122	130	126	102	115	133	133	133	133	131	131	102	134	134	131	125	130	106	135	126	96	96	96	141	85	138	128	128	127	104	98	99	111	111	111

A208	*	Yuzu	0.645	1	0	141	143	137	144	135	131	136	140	132	132	132	132	130	135	130	140	140	144	130	120	136	137	128	142	142	142	92	139	93	107	134	145	136	127	138	139	139	139
B001	*	Akemi'	0.467	1	1	112	132	119	130	115	128	93	116	114	114	114	114	109	100	118	103	103	137	127	128	106	116	130	126	126	126	141	113	111	124	108	131	108	105	124	107	107	107
B002	*	'Aki Marine'	0.515	1	0	116	130	115	132	101	132	102	117	95	95	95	95	115	99	120	99	99	141	123	125	113	112	117	110	110	110	135	118	117	124	114	136	118	106	113	119	119	119
B003	*	'Aki Tangor'	0.562	1	0	97	132	121	131	115	134	84	120	107	107	107	107	119	103	112	123	123	141	123	122	92	121	131	106	106	106	138	117	124	121	113	135	117	98	108	109	109	109
B004	*	'Allspice'	0.669	1	0	97	135	118	142	122	130	101	120	112	112	112	112	120	77	108	120	120	142	125	135	103	120	125	99	99	99	138	110	121	121	116	133	121	102	118	106	106	106
B005	*	'Amaka'	0.456	1	0	131	134	133	133	112	138	109	115	100	100	100	100	102	84	130	98	98	139	118	123	110	87	113	117	117	117	132	134	126	122	107	139	134	118	123	123	123	123
B006	*	'Ariake'	0.485	1	0	110	141	111	134	114	136	106	117	74	74	74	74	114	92	113	109	109	143	131	138	103	119	124	112	112	112	143	112	132	131	110	142	119	112	132	121	121	121
B007	*	'Asumi'	0.456	1	0	123	142	126	137	109	120	115	120	97	97	97	97	97	98	119	81	81	147	123	124	110	96	127	128	128	128	137	126	120	127	90	143	120	124	136	122	122	122
B008	*	'Aurastar'	0.663	1	0	131	114	132	121	150	136	145	139	149	149	149	149	149	152	135	151	151	121	149	142	129	148	148	126	126	126	148	122	142	141	153	118	126	136	124	140	140	140
B009	*	'Awa Orange'	0.639	1	0	109	136	119	140	118	130	105	113	131	131	131	131	119	112	111	114	114	144	107	114	116	117	109	118	118	118	134	111	119	124	111	140	125	118	120	91	91	91
B010	*	'Benibae'	0.479	1	0	120	140	118	146	96	127	111	110	83	83	83	83	99	92	121	92	92	150	105	120	114	96	115	122	122	122	130	125	116	113	89	149	131	114	129	120	120	120
B011	*	'Benimadoka'	0.314	1	0	133	86	130	58	148	129	139	140	146	146	146	146	143	150	136	147	147	72	146	142	135	141	148	118	118	118	151	129	150	146	147	55	114	135	122	141	141	141
B012	*	'Chandler pummelo'	0.284	1	0	135	87	127	59	143	130	137	142	145	145	145	145	140	146	135	141	141	77	146	138	129	137	142	121	121	121	149	124	146	142	147	52	109	138	121	139	139	139
B013	*	'Ehimekashi 28'	0.515	1	0	122	135	120	138	109	133	97	120	91	91	91	91	109	101	122	101	101	146	118	128	107	109	122	125	125	125	133	126	133	120	107	144	131	111	128	119	119	119
B014	*	'Encore'	0.432	1	0	131	140	130	137	97	131	115	109	91	91	91	91	89	78	127	87	87	142	107	113	115	78	108	126	126	126	122	135	120	113	91	137	133	123	138	130	130	130
B015	*	'Fairchild'	0.509	1	0	111	139	122	133	105	129	95	128	69	69	69	69	99	81	114	90	90	144	112	115	104	101	122	114	114	114	136	116	126	123	99	138	114	96	116	111	111	111
B016	*	'Fortune'	0.544	1	0	118	137	127	130	113	128	101	120	79	79	79	79	104	98	120	85	85	143	122	119	107	90	122	120	120	120	133	120	126	128	98	137	114	99	123	120	120	120
B017	*	'Harehime'	0.615	1	0	106	132	119	140	111	136	79	124	102	102	102	102	117	105	119	98	98	151	115	127	109	120	115	134	134	134	137	115	132	124	114	142	126	103	122	108	108	108
B018	*	'Hareyaka'	0.438	1	0	133	140	138	143	97	130	112	112	90	90	90	90	84	71	137	70	70	151	111	117	120	87	114	135	135	135	126	132	119	121	69	142	130	119	135	128	128	128
B019	*	'Haruhi'	0.485	1	0	121	133	130	136	113	126	110	110	125	125	125	125	113	111	111	115	115	136	105	113	113	112	111	134	134	134	129	121	123	123	111	137	130	117	130	100	100	100
B020	*	'Haruka'	0.586	1	0	113	135	127	138	111	140	115	116	115	115	115	115	107	103	115	115	115	141	106	104	111	113	88	119	119	119	124	120	124	118	118	138	125	113	127	93	93	93
B021	*	'Harumi'	0.521	1	0	124	140	131	141	103	130	99	112	86	86	86	86	101	92	130	72	72	152	119	128	109	95	121	133	133	133	134	128	117	124	83	143	122	106	134	124	124	124
B022	*	'Hayaka'	0.485	1	0	127	139	136	141	91	137	105	108	106	106	106	106	96	96	125	85	85	147	102	124	110	97	119	130	130	130	135	130	115	125	92	143	133	118	128	119	119	119
B023	*	'Hayasaki'	0.290	1	0	136	88	130	69	143	125	146	141	145	145	145	145	146	147	131	144	144	68	147	138	133	145	148	123	123	123	149	135	146	144	144	53	118	139	125	139	139	139
B024	*	'Himekoharu'	0.627	1	0	106	144	123	143	111	134	83	114	116	116	116	116	112	106	115	111	111	146	105	120	118	114	114	121	121	121	126	117	127	127	115	144	124	105	121	107	107	107
B025	*	'Hiroshimakaken 11'	0.444	1	0	119	130	119	131	104	125	113	118	91	91	91	91	106	100	123	94	94	139	113	125	114	104	118	114	114	114	136	123	125	125	105	136	118	115	115	124	124	124
B026	*	'Honey'	0.391	1	0	130	143	135	141	109	131	121	115	100	100	100	100	87	88	131	101	101	140	104	112	122	90	108	130	130	130	118	132	125	106	86	142	136	116	136	127	127	127
B027	*	'Kanpei'	0.562	1	1	117	138	130	136	110	131	99	107	90	90	90	90	100	96	126	88	88	145	111	128	108	100	119	123	123	123	138	124	123	129	92	137	118	111	132	120	120	120
B028	*	'Kara'	0.562	1	0	109	133	118	131	98	137	87	118	116	116	116	116	111	99	118	102	102	140	113	115	109	101	121	119	119	119	131	119	122	103	111	137	126	99	113	106	106	106
B029	*	'Kincy mandarin'	0.438	1	0	131	137	134	131	98	140	116	114	84	84	84	84	91	88	133	88	88	141	109	124	113	82	111	128	128	128	129	135	127	118	113	136	133	125	136	126	126	126
B030	*	'Kinnow mandarin'	0.391	1	0	126	142	135	134	104	131	113	112	94	94	94	94	92	82	127	92	92	143	112	121	120	85	111	123	123	123	127	134	121	119	83	132	133	120	139	121	121	121
B031	*	'Kiyomi'	0.586	1	0	107	129	115	131	118	133	71	109	99	99	99	99	126	109	118	113	113	140	124	126	106	122	127	123	123	123	138	108	119	127	116	137	117	90	118	113	113	113
B032		'Kuchinotsu-41'	0.728	6	0	105	139	118	138	129	132	94	113	136	136	136	136	131	126	98	133	133	141	131	115	111	134	121	122	122	122	138	110	127	122	131	140	129	105	118	0	0	0
B033	*	'Lee'	0.420	1	0	140	141	127	137	98	132	120	120	68	68	68	68	98	78	134	62	62	145	118	130	120	84	122	133	133	133	134	122	125	132	86	143	123	125	139	134	134	134
B034	*	May Pummelo'	0.538	1	0	105	106	98	89	143	130	108	132	143	143	143	143	146	136	123	146	146	105	142	138	111	138	141	102	102	102	148	84	137	133	135	83	108	99	108	126	126	126
B035	*	'Mihaya'	0.456	1	0	109	138	131	136	108	136	93	131	103	103	103	103	104	93	121	95	95	138	117	121	105	95	126	122	122	122	136	123	134	124	109	137	128	103	125	119	119	119
B036	*	'Mihocore'	0.467	1	0	112	134	127	138	117	133	90	119	116	116	116	116	104	106	124	103	103	140	112	116	110	107	122	131	131	131	129	124	125	121	104	135	120	105	125	120	120	120
B037	*	'Mineola'	0.609	1	0	101	138	116	138	110	133	95	122	108	108	108	108	114	94	113	92	92	148	123	125	97	111	120	97	97	97	135	111	121	134	103	139	113	87	119	111	111	111
B038	*	'Nankou'	0.562	1	1	118	133	120	136	116	137	91	116	96	96	96	96	114	91	123	111	111	144	116	127	96	109	125	111	111	111	143	120	126	122	115	136	119	104	122	122	122	122
B039	*	'Nishinokaori'	0.509	1	1	121	133	133	131	120	133	95	118	95	95	95	95	120	99	121	106	106	141	125	130	102	123	124	129	129	129	140	127	126	133	110	138	130	112	136	119	119	119
B040	*	'Nou 5 gou'	0.444	1	0	128	145	132	146	83	135	120	109	80	80	80	80	94	96	129	80	80	148	104	127	118	96	128	126	126	126	135	129	125	119	92	142	123	115	126	130	130	130
B041	*	'Nou 6 gou'	0.432	1	0	115	134	128	138	102	128	98	101	96	96	96	96	96	107	128	97	97	141	107	110	114	89	112	128	128	128	128	125	123	105	92	141	130	112	121	119	119	119
B042	*	'Nou 7 gou'	0.621	1	0	117	112	136	107	147	138	131	135	145	145	145	145	144	144	129	144	144	116	146	140	126	143	141	113	113	113	148	107	140	140	149	113	125	127	115	129	129	129
B043	*	'Nova'	0.479	1	0	136	141	128	132	107	132	112	123	74	74	74	74	108	97	126	87	87	146	125	132	108	104	126	120	120	120	136	118	126	139	103	140	120	115	136	130	130	130
B044	*	'Ooitakaken-4'	0.598	1	1	104	132	119	143	112	134	78	116	113	113	113	113	122	100	113	112	112	148	118	131	96	121	128	113	113	113	137	118	125	117	117	146	126	91	110	113	113	113
B045	*	'Orland'	0.615	1	0	111	139	111	129	120	139	93	124	113	113	113	113	118	103	115	92	92	150	125	127	102	105	122	99	99	99	137	104	126	138	108	137	116	101	124	112	112	112
B047	*	'Osceola'	0.414	1	0	129	143	129	139	102	130	110	126	76	76	76	76	92	88	127	75	75	147	109	123	123	94	110	131	131	131	127	126	124	127	87	145	122	117	139	125	125	125
B048	*	'Page'	0.479	1	0	126	136	125	134	110	132	110	123	70	70	70	70	105	91	127	91	91	150	120	120	107	95	124	119	119	119	128	121	125	128	98	144	118	103	130	131	131	131
B049	*	'Pearl'	0.680	1	0	91	130	106	131	129	130	95	119	119	119	119	119	131	100	107	131	131	133	132	129	115	127	128	100	100	100	138	95	129	119	131	132	114	94	102	105	105	105
B050	*	'Pixie mandarin'	0.296	1	0	132	144	134	141	93	136	110	116	100	100	100	100	94	93	136	80	80	142	110	124	118	86	116	138	138	138	124	135	130	116	93	140	135	119	133	127	127	127
B051	*	'Robinson'	0.379	1	0	140	151	131	139	92	137	118	134	63	63	63	63	89	74	137	63	63	149	110	126	115	85	114	136	136	136	131	132	125	133	80	145	131	123	145	137	137	137
B052	*	'Saga Mandarin'	0.550	1	0	102	143	125	138	104	145	77	115	105	105	105	105	111	90	123	108	108	144	114	129	94	115	112	117	117	117	136	124	128	115	107	141	133	101	119	114	114	114
B053		'Sagakashi 34'	0.515	2	0	116	145	119	138	104	130	94	106	101	101	101	101	108	99	120	110	110	140	112	120	93	104	116	119	119	119	132	121	121	126	94	143	123	107	132	112	112	112
B054	*	'Seihou'	0.438	1	0	118	121	124	127	119	135	101	129	116	116	116	116	112	101	121	107	107	133	122	125	111	114	128	125	125	125	139	106	122	132	111	128	119	101	127	112	112	112
B055	*	'Seinannohikari'	0.497	1	0	118	138	129	138	106	139	84	127	100	100	100	100	95	98	128	93	93	144	113	123	102	89	118	122	122	122	131	119	120	113	101	141	139	113	121	113	113	113
B056	*	'Seminole'	0.609	1	0	100	140	120	138	112	137	85	126	113	113	113	113	111	93	115	92	92	147	128	128	104	114	118	101	101	101	140	115	119	136	102	140	117	88	114	107	107	107
B057	*	'Setomi'	0.533	1	0	124	139	124	140	110	139	106	125	87	87	87	87	115	96	130	97	97	146	129	126	110	112	130	124	124	124	136	123	126	139	101	151	130	117	131	123	123	123
B058	*	'Shiranuhi'	0.515	2	0	116	145	119	138	104	130	94	106	101	101	101	101	108	99	120	110	110	140	112	120	93	104	116	119	119	119	132	121	121	126	94	143	123	107	132	112	112	112
B059	*	'Southern Yellow'	0.686	1	0	89	127	114	142	116	144	116	115	131	131	131	131	126	115	115	130	130	142	115	126	111	132	128	113	113	113	138	109	134	127	143	135	127	106	111	119	119	119
B060	*	'Summer Fresh'	0.574	1	2	106	99	101	114	131	142	106	138	139	139	139	139	135	129	121	142	142	115	138	135	105	133	132	93	93	93	144	82	131	132	142	112	124	110	84	119	119	119
B061	*	Sweet Spring'	0.669	1	0	92	134	107	136	114	144	68	107	119	119	119	119	123	108	119	123	123	143	121	132	94	118	118	104	104	104	131	90	122	116	123	135	124	81	102	108	108	108
B062	*	'Tamami'	0.444	1	0	132	141	129	144	101	136	113	120	100	100	100	100	97	82	131	94	94	145	104	123	114	91	122	124	124	124	132	134	113	123	99	149	131	114	137	129	129	129
B063	*	'Tanikawa Buntan'	0.609	1	0	105	98	88	110	142	131	112	117	137	137	137	137	142	138	117	142	142	115	137	129	113	137	129	119	119	119	135	79	129	131	140	110	105	108	114	109	109	109
B064	*	'Tsunokagayaki'	0.497	1	0	120	131	130	132	113	134	101	116	94	94	94	94	103	83	123	100	100	139	115	116	105	103	119	127	127	127	130	113	121	120	104	135	120	105	121	112	112	112
B065	*	'Tsunokaori'	0.521	1	5	107	136	114	141	116	128	78	108	103	103	103	103	118	112	118	104	104	147	117	119	119	112	124	130	130	130	135	116	125	119	107	142	120	99	115	117	117	117
B066	*	'Tsunonozomi'	0.438	1	0	121	136	125	136	99	127	110	107	86	86	86	86	98	100	126	100	100	142	113	124	107	105	113	126	126	126	130	126	115	118	98	139	120	117	122	120	120	120
B067	*	'Willking'	0.237	1	0	130	143	141	137	93	126	125	123	85	85	85	85	76	93	133	90	90	135	107	120	121	85	125	129	129	129	125	136	121	120	90	129	132	117	136	131	131	131
B068	*	'Yellow Pummelo'	0.621	1	2	94	117	88	119	129	131	97	126	132	132	132	132	142	130	108	144	144	119	136	137	104	134	134	98	98	98	144	58	132	128	137	113	107	96	90	109	109	109
B069	*	'Youkou'	0.432	1	0	122	137	124	140	100	131	111	106	95	95	95	95	96	100	126	79	79	148	108	121	102	89	109	118	118	118	132	125	118	120	86	145	125	124	129	122	122	122
B070	*	E-647	0.503	1	0	118	138	114	135	103	137	102	120	95	95	95	95	116	102	116	101	101	151	121	132	96	120	127	123	123	123	140	123	120	141	108	146	121	107	127	115	115	115
B071	*	EnOw21	0.550	1	0	119	135	120	140	110	135	79	127	107	107	107	107	106	95	123	108	108	142	118	120	104	108	125	130	130	130	124	120	131	116	113	141	131	99	120	112	112	112
B072	*	H･FD-1	0.704	1	0	142	129	142	136	148	139	146	139	147	147	147	147	148	145	140	144	144	134	148	140	144	146	142	136	136	136	152	138	140	144	146	135	135	146	134	139	139	139
B073	*	HF9	0.497	1	0	113	136	123	140	109	131	93	112	107	107	107	107	108	104	125	90	90	154	108	124	106	111	124	130	130	130	136	124	120	120	97	142	126	109	122	119	119	119
B074	*	KyOw14	0.556	1	0	105	129	111	133	119	131	75	109	108	108	108	108	120	114	117	108	108	145	109	126	106	115	123	126	126	126	136	108	126	115	115	135	118	92	109	114	114	114
B075	*	KyOw21	0.527	1	0	105	141	115	141	114	133	81	109	119	119	119	119	121	119	119	116	116	148	116	126	111	122	124	125	125	125	136	121	126	120	107	141	119	98	115	116	116	116

B076	*	No.1408	0.497	1	0	104	134	125	134	112	127	96	137	98	98	98	98	110	99	124	100	100	138	114	129	106	100	134	121	121	121	138	114	129	130	108	138	129	104	131	125	125	125
B077	*	Okitsu-46	0.533	1	0	109	136	116	134	121	136	85	113	117	117	117	117	123	96	113	120	120	145	123	130	99	125	125	113	113	113	137	111	127	124	108	142	124	102	123	107	107	107
B078	*	Okitsu-56	0.373	1	0	135	147	134	147	87	135	121	123	89	89	89	89	86	85	141	80	80	144	99	120	117	79	117	126	126	126	134	135	126	120	90	145	134	114	138	133	133	133
C001	*	Strain 01	0.325	1	0	132	135	126	131	101	131	108	115	100	100	100	100	98	98	131	85	85	138	114	120	115	103	116	133	133	133	127	129	121	123	93	137	123	117	131	127	127	127
C002	*	Strain 02	0.509	1	0	127	144	126	138	103	138	99	130	70	70	70	70	112	87	122	103	103	144	120	135	111	121	129	120	120	120	140	130	126	128	118	146	136	111	133	118	118	118
C003	*	Strain 03	0.556	1	0	120	139	113	139	106	131	88	117	99	99	99	99	112	109	127	91	91	153	119	125	119	106	112	139	139	139	134	130	126	125	100	147	134	118	132	117	117	117
C004	*	Strain 04	0.592	1	0	109	123	115	123	131	118	103	127	127	127	127	127	132	134	114	139	139	129	135	124	112	139	131	112	112	112	132	111	129	128	135	132	117	104	111	102	102	102
C005	*	Strain 05	0.580	1	0	108	134	121	141	118	140	78	125	107	107	107	107	117	101	119	113	113	147	127	125	93	110	128	120	120	120	138	111	122	131	113	144	127	98	121	109	109	109
C006	*	Strain 06	0.426	1	0	124	138	119	142	104	140	105	121	102	102	102	102	111	82	124	104	104	147	111	124	92	112	117	120	120	120	129	113	118	115	108	147	130	103	129	124	124	124
C007	*	Strain 07	0.533	1	2	78	114	108	124	119	133	97	112	122	122	122	122	121	132	121	121	121	129	125	126	123	123	122	101	101	101	136	102	133	130	125	123	114	108	105	127	127	127
C008	*	Strain 08	0.462	1	0	97	138	118	136	113	125	98	114	108	108	108	108	114	105	117	102	102	141	114	125	115	115	115	115	115	115	138	110	124	123	102	140	120	103	109	113	113	113
C009	*	Strain 09	0.420	1	0	123	137	124	134	107	135	102	121	99	99	99	99	116	101	124	114	114	138	119	125	104	120	121	121	121	121	135	124	126	129	106	138	127	117	130	120	120	120
C010	*	Strain 10	0.438	1	0	127	144	128	139	91	136	104	119	86	86	86	86	88	81	131	85	85	146	109	130	113	89	115	129	129	129	133	128	117	131	90	139	127	118	134	122	122	122
C011	*	Strain 11	0.521	1	0	119	135	129	133	111	136	86	111	99	99	99	99	102	92	126	98	98	141	122	127	95	103	120	124	124	124	133	124	125	128	104	135	119	110	127	115	115	115
C012	*	Strain 12	0.391	1	0	130	131	138	130	112	137	99	112	100	100	100	100	100	92	130	95	95	137	122	122	108	94	114	122	122	122	132	132	117	122	87	130	128	118	124	124	124	124
C013	*	Strain 13	0.598	1	0	101	133	109	137	118	118	87	112	121	121	121	121	112	122	112	116	116	148	111	120	99	120	120	120	120	120	132	119	112	112	111	139	117	97	104	90	90	90
C014	*	Strain 14	0.402	1	0	126	143	123	132	98	132	102	127	93	93	93	93	108	90	125	86	86	149	116	134	109	109	127	133	133	133	139	130	121	141	105	140	129	116	138	120	120	120
C015	*	Strain 15	0.426	1	0	126	139	128	141	98	130	102	122	107	107	107	107	91	106	124	91	91	146	98	115	115	94	110	130	130	130	128	126	126	109	96	142	132	116	121	125	125	125
C016	*	Strain 16	0.284	1	0	135	83	132	72	147	127	145	145	139	139	139	139	144	141	134	149	149	62	146	139	131	142	148	121	121	121	148	133	144	142	145	54	116	136	129	140	140	140
C017	*	Strain 17	0.527	1	0	119	131	123	137	112	134	89	116	125	125	125	125	107	126	131	100	100	144	112	133	116	104	121	126	126	126	138	122	126	127	110	136	122	99	117	113	113	113
C018	*	Strain 18	0.521	1	0	123	139	133	143	107	143	82	122	98	98	98	98	99	81	127	85	85	149	114	122	109	100	112	129	129	129	134	132	124	118	96	144	131	111	127	121	121	121
C019	*	Strain 19	0.497	1	0	124	139	132	144	112	134	93	112	103	103	103	103	109	96	129	105	105	146	114	113	114	102	118	130	130	130	128	132	126	108	108	139	129	109	123	117	117	117
C020	*	Strain 20	0.467	1	0	131	144	134	140	98	139	116	110	91	91	91	91	95	74	138	73	73	150	118	119	116	79	114	132	132	132	131	133	121	120	64	142	135	126	140	132	132	132
C021	*	Strain 21	0.402	1	0	121	137	116	132	105	131	105	115	98	98	98	98	98	98	128	100	100	142	116	124	112	102	116	119	119	119	128	126	125	124	99	134	127	120	133	123	123	123
C022	*	Strain 22	0.473	1	0	112	145	123	144	106	135	96	124	88	88	88	88	106	89	121	91	91	149	113	132	100	102	129	122	122	122	139	126	120	123	100	147	127	109	129	115	115	115
C023	*	Strain 23	0.527	1	0	118	138	128	145	98	138	99	121	109	109	109	109	100	114	127	82	82	151	108	122	109	99	118	131	131	131	133	127	130	122	107	144	127	109	124	124	124	124
C024	*	Strain 24	0.527	1	0	112	137	115	136	105	140	83	127	115	115	115	115	108	102	132	114	114	143	119	120	102	105	124	120	120	120	123	113	125	118	113	139	125	94	114	110	110	110
C025	*	Strain 25	0.379	1	0	124	139	134	139	99	140	114	110	92	92	92	92	93	89	127	73	73	147	100	112	114	93	112	126	126	126	126	132	120	113	95	138	126	118	129	127	127	127
C026	*	Strain 26	0.556	1	0	98	133	114	131	109	133	70	114	104	104	104	104	122	106	117	108	108	143	123	135	97	124	127	119	119	119	142	107	128	125	111	135	115	95	113	117	117	117
C027	*	Strain 27	0.450	1	0	125	136	122	134	102	133	101	106	92	92	92	92	108	92	125	101	101	141	110	121	104	98	110	125	125	125	127	126	129	120	105	144	128	114	131	129	129	129
C028	*	Strain 28	0.473	1	0	128	133	124	138	98	137	103	123	98	98	98	98	94	97	127	101	101	145	105	123	109	92	126	129	129	129	133	130	121	122	109	145	124	112	119	128	128	128
C029	*	Strain 29	0.379	1	0	129	135	128	137	104	133	118	119	93	93	93	93	95	94	128	90	90	141	109	120	114	101	116	116	116	116	132	128	123	122	93	140	130	119	124	122	122	122
C030	*	Strain 30	0.568	1	0	119	135	126	139	104	139	82	120	96	96	96	96	114	101	125	96	96	149	123	128	108	107	121	132	132	132	133	121	126	127	107	140	126	100	127	119	119	119
C031	*	Strain 31	0.450	1	0	129	134	134	137	103	138	104	128	96	96	96	96	94	86	132	100	100	137	112	110	116	95	120	129	129	129	121	127	127	117	105	138	134	115	118	123	123	123
C032	*	Strain 32	0.544	1	0	97	131	127	132	114	138	80	119	106	106	106	106	118	108	119	115	115	138	124	134	101	122	128	116	116	116	140	111	129	124	122	134	129	103	128	120	120	120
C033	*	Strain 33	0.533	1	0	110	138	123	130	111	137	91	122	112	112	112	112	107	98	112	110	110	141	125	129	94	113	123	108	108	108	141	114	119	134	116	133	116	99	125	111	111	111
C034	*	Strain 34	0.651	1	0	109	134	112	138	116	142	85	118	94	94	94	94	119	94	117	113	113	148	130	134	88	120	125	107	107	107	138	97	121	132	114	140	120	96	114	118	118	118
C035	*	Strain 35	0.509	1	0	116	133	123	135	112	129	97	117	92	92	92	92	118	97	120	96	96	141	118	126	111	117	120	121	121	121	132	125	122	126	104	140	124	103	118	119	119	119
C036	*	Strain 36	0.562	1	2	127	141	113	139	113	127	104	109	85	85	85	85	113	94	122	104	104	148	129	123	121	111	117	126	126	126	138	127	130	127	106	140	125	117	128	120	120	120
C037	*	Strain 37	0.473	1	0	122	139	133	145	99	133	105	127	94	94	94	94	92	86	132	78	78	146	115	119	107	91	116	130	130	130	135	130	127	125	95	144	127	110	131	133	133	133
C038	*	Strain 38	0.473	1	1	128	140	135	144	98	142	105	127	82	82	82	82	97	102	139	84	84	148	112	133	121	87	128	134	134	134	134	127	123	127	98	142	133	113	131	128	128	128
C039	*	Strain 39	0.521	1	0	121	132	124	132	119	124	110	110	86	86	86	86	107	93	116	100	100	139	127	118	109	102	130	123	123	123	141	122	122	129	105	133	117	110	133	116	116	116
C040		Strain 40	0.491	1	0	121	126	133	127	96	136	104	108	83	83	83	83	105	96	123	103	103	136	118	126	104	105	119	113	113	113	134	126	115	120	101	130	119	111	123	120	120	120
C041	*	Strain 41	0.467	1	0	122	147	128	140	99	142	111	129	90	90	90	90	102	87	125	103	103	148	110	121	119	85	118	123	123	123	128	129	131	133	102	148	132	113	134	129	129	129
C042	*	Strain 42	0.556	1	0	115	141	123	142	112	131	98	113	96	96	96	96	110	104	121	99	99	154	119	123	115	114	115	133	133	133	136	130	126	131	96	147	118	117	126	120	120	120
C043	*	Strain 43	0.473	1	0	117	137	127	140	107	132	78	111	100	100	100	100	102	108	133	89	89	148	117	126	117	107	114	135	135	135	132	128	131	122	86	138	126	107	118	122	122	122
C044	*	Strain 44	0.509	1	0	122	140	124	145	101	135	99	120	92	92	92	92	114	96	131	92	92	151	113	120	115	108	118	129	129	129	132	129	126	116	99	149	128	108	124	124	124	124
C045	*	Strain 45	0.568	1	0	120	134	124	139	116	135	81	107	88	88	88	88	115	99	122	93	93	151	130	126	103	113	119	123	123	123	130	120	119	132	104	143	122	102	131	115	115	115
C046	*	Strain 46	0.574	1	0	101	123	123	125	123	128	107	127	116	116	116	116	122	117	118	113	113	135	138	141	110	129	133	118	118	118	147	103	127	142	120	129	116	112	122	123	123	123
C047	*	Strain 47	0.669	1	0	96	127	108	135	119	128	84	115	109	109	109	109	116	100	106	119	119	147	131	131	87	116	128	103	103	103	142	100	128	130	126	142	110	101	113	104	104	104
C048	*	Strain 48	0.527	1	0	112	126	113	133	106	134	88	122	112	112	112	112	117	107	122	96	96	144	115	127	96	114	132	129	129	129	139	126	121	125	104	130	114	98	123	119	119	119
C049	*	Strain 49	0.645	1	1	111	135	118	137	121	140	70	118	111	111	111	111	121	102	116	117	117	144	123	129	90	121	126	107	107	107	145	116	121	121	119	139	123	101	116	105	105	105
C050	*	Strain 50	0.396	1	2	129	145	132	141	101	127	100	111	83	83	83	83	99	89	127	90	90	147	108	123	106	99	119	137	137	137	134	137	120	123	93	139	126	114	136	128	128	128
C051	*	Strain 51	0.592	1	0	117	137	128	131	127	131	102	123	122	122	122	122	115	112	117	121	121	134	130	122	113	125	112	119	119	119	131	120	127	128	115	133	130	117	123	94	94	94
C052	*	Strain 52	0.698	1	0	109	137	118	138	126	135	104	121	118	118	118	118	125	109	107	130	130	146	127	128	105	131	116	121	121	121	140	113	128	123	125	141	119	110	120	79	79	79
C053	*	Strain 53	0.692	1	0	101	132	109	136	129	123	90	123	116	116	116	116	120	110	112	118	118	142	119	124	104	130	119	116	116	116	136	106	123	131	121	140	111	101	122	91	91	91
C054	*	Strain 54	0.574	1	0	106	136	107	135	111	144	95	126	118	118	118	118	108	101	123	106	106	145	123	134	88	109	125	107	107	107	136	99	129	127	102	139	128	105	116	110	110	110
C055	*	Strain 55	0.432	1	0	117	142	122	137	88	123	108	114	75	75	75	75	88	97	119	77	77	147	111	128	113	90	122	118	118	118	132	118	125	132	93	140	116	113	123	121	121	121
C056	*	Strain 56	0.651	1	0	94	141	116	142	112	141	87	120	104	104	104	104	122	100	116	118	118	147	114	135	105	123	122	108	108	108	132	105	123	120	112	145	124	95	115	110	110	110
C057	*	Strain 57	0.556	1	0	94	124	116	127	121	136	78	123	117	117	117	117	131	109	124	123	123	128	131	134	105	123	131	112	112	112	140	72	125	121	123	131	121	95	112	115	115	115
C058	*	Strain 58	0.544	1	0	81	132	104	133	123	138	92	121	133	133	133	133	127	114	115	114	114	138	121	135	110	128	117	97	97	97	138	93	131	127	119	127	121	97	115	117	117	117
C059	*	Strain 59	0.521	1	0	94	131	92	129	118	131	85	114	131	131	131	131	124	120	117	126	126	133	126	127	102	119	116	103	103	103	132	70	125	120	122	126	113	88	103	118	118	118
C060	*	Strain 60	0.538	1	0	115	138	115	142	108	140	112	116	113	113	113	113	106	106	122	82	82	150	114	121	104	99	109	117	117	117	135	119	136	129	103	142	125	119	124	123	123	123
C061	*	Strain 61	0.515	1	0	110	86	103	90	136	132	125	125	128	128	128	128	141	135	117	143	143	96	142	135	114	140	141	93	93	93	143	83	140	129	142	82	101	113	105	131	131	131
C062	*	Strain 62	0.621	1	0	102	132	96	129	116	136	87	122	116	116	116	116	135	109	110	130	130	135	136	129	94	130	128	92	92	92	136	73	128	129	123	140	120	98	102	102	102	102
C063	*	Strain 63	0.538	1	0	113	140	127	138	110	135	86	116	88	88	88	88	109	86	124	103	103	144	123	122	108	106	119	122	122	122	132	123	127	128	102	141	122	102	128	115	115	115
C064	*	Strain 64	0.574	1	0	104	143	118	143	104	136	87	119	113	113	113	113	118	93	112	106	106	151	112	124	101	118	118	121	121	121	140	119	127	124	109	143	127	98	119	108	108	108
C065	*	Strain 65	0.479	1	0	122	138	126	138	97	129	108	114	87	87	87	87	92	88	125	97	97	142	111	135	110	94	123	123	123	123	137	122	117	126	97	138	127	114	132	123	123	123
C066	*	Strain 66	0.509	1	0	127	144	126	140	105	138	97	117	105	105	105	105	91	87	128	93	93	147	115	129	110	99	124	130	130	130	134	126	118	135	95	140	128	111	134	116	116	116
C067	*	Strain 67	0.396	1	3	115	137	123	141	111	133	89	113	107	107	107	107	111	103	126	97	97	146	110	122	119	103	121	137	137	137	133	121	122	111	99	140	126	111	120	124	124	124
C068	*	Strain 68	0.391	1	0	110	136	123	136	115	132	89	116	110	110	110	110	115	104	122	107	107	134	124	132	108	117	124	127	127	127	139	115	122	128	101	136	125	106	128	118	118	118
C069	*	Strain 69	0.432	1	0	121	141	123	139	107	129	106	119	99	99	99	99	100	92	120	85	85	150	117	122	105	95	123	130	130	130	134	127	119	121	84	147	123	112	133	122	122	122
C070	*	Strain 70	0.379	1	0	129	137	123	134	104	138	104	127	91	91	91	91	106	76	128	88	88	142	121	121	98	99	122	125	125	125	123	123	125	124	93	139	131	108	136	130	130	130
C071	*	Strain 71	0.580	1	1	113	135	124	134	122	131	88	117	105	105	105	105	115	103	117	108	108	147	131	130	103	121	127	122	122	122	137	115	126	128	116	145	121	113	128	112	112	112
C072	*	Strain 72	0.621	1	0	89	110	118	115	132	136	118	126	129	129	129	129	140	127	117	144	144	118	140	135	113	143	139	85	85	85	150	99	142	132	145	118	114	108	84	124	124	124

C073	*	Strain 73	0.521	1	0	129	139	126	144	93	130	113	115	83	83	83	83	100	89	129	85	85	145	107	127	116	99	121	133	133	133	132	126	125	128	97	139	119	109	131	127	127	127
C074	*	Strain 74	0.450	1	0	121	145	123	137	103	128	116	126	83	83	83	83	97	84	126	76	76	142	112	119	114	105	116	124	124	124	131	122	125	129	77	145	124	113	141	123	123	123
C075	*	Strain 75	0.580	1	1	105	115	118	112	138	139	130	126	140	140	140	140	143	134	122	139	139	120	139	133	119	144	141	108	108	108	150	103	139	130	144	115	111	119	105	127	127	127
C076	*	Strain 76	0.645	1	0	98	115	113	111	140	133	117	133	142	142	142	142	140	136	117	143	143	119	140	134	117	140	139	94	94	94	150	93	146	139	148	111	109	119	97	124	124	124
C077	*	Strain 77	0.574	1	0	115	135	119	139	101	138	81	101	90	90	90	90	115	90	127	96	96	143	113	130	113	104	113	127	127	127	133	118	120	113	103	140	125	100	126	111	111	111
C078	*	Strain 78	0.497	1	2	112	143	122	143	124	131	94	106	115	115	115	115	120	106	122	107	107	144	123	128	120	116	122	122	122	122	139	125	126	123	104	144	126	107	125	115	115	115
C079	*	Strain 79	0.533	1	0	120	138	129	135	121	127	114	118	110	110	110	110	121	111	133	113	113	141	125	132	118	124	131	126	126	126	135	126	119	130	102	140	124	109	125	121	121	121
C080	*	Strain 80	0.669	1	0	117	137	112	141	115	137	91	104	115	115	115	115	127	101	111	116	116	146	124	124	101	122	108	117	117	117	139	113	122	128	120	140	123	105	124	94	94	94
C081	*	Strain 81	0.479	1	0	109	136	116	135	110	131	95	118	96	96	96	96	114	107	125	84	84	149	115	134	106	111	123	128	128	128	139	125	122	134	103	148	125	116	130	124	124	124
C082	*	Strain 82	0.669	1	0	91	115	101	128	127	134	103	117	129	129	129	129	134	124	112	132	132	131	130	130	109	132	130	88	88	88	144	87	131	115	138	128	112	104	78	106	106	106
C083	*	Strain 83	0.497	1	1	112	137	118	142	110	130	78	107	108	108	108	108	114	105	125	91	91	149	107	129	105	102	117	130	130	130	134	121	123	116	105	143	122	104	126	117	117	117
C084	*	Strain 84	0.462	1	0	122	142	126	138	106	147	97	130	102	102	102	102	101	90	130	91	91	150	117	129	103	106	117	124	124	124	136	117	137	127	101	140	130	120	128	122	122	122
C085	*	Strain 85	0.349	1	0	136	139	142	138	94	133	109	123	85	85	85	85	86	92	140	71	71	145	117	121	117	89	128	138	138	138	130	140	123	129	86	142	129	118	132	139	139	139

H: Observed heterozygosity of individuals estimated with 169 DNA markers

6	6	1	1	2	2	1	1	1	1	1	1	1	2	1	1	1	2	16	16	16	16	16	16	16	16	16	16	16	16	16	16	16	16	2	1	3	3	2	1	1	8	1	8	8	1	1	1	1	1	1	1
Hyuganatsu	Hyuganatsu	Ichanchii	Ichang lemon	Iyo	Iyo	Jabara	Jabbon	Kabosu	Kabuchi	Kaikoukan	Kawabata	Kawachi bankan	Keraji	Kikudaidai	King mandarin	Kinkoji	Kinukawa	Kishu mandarin	Kishu mandarin	Kishu mandarin	Kishu mandarin	Kishu mandarin	Kishu mandarin	Kishu mandarin	Kishu mandarin	Kishu mandarin	Kishu mandarin	Kishu mandarin	Kishu mandarin	Kishu mandarin	Kishu mandarin	Kishu mandarin	Kishu mandarin	Kizu	Kobeni mikan	Koji	Komikan 2009-130	Konejime	Kourai Tachibana	Kotokan	Kunenbo-A	Kunenbo-B	Kunenbo	Kunenbo	Lemon	Lemonade	Limonia	Mato buntan	Mexican lime	Meyer lemon	Mochiyu
A039	A040	A041	A042	A043	A044	A045	A046	A047	A048	A049	A050	A051	A052	A053	A054	A055	A056	A057	A058	A059	A060	A061	A062	A063	A064	A065	A066	A067	A068	A069	A070	A071	A072	A073	A075	A076	A077	A078	A079	A080	A081	A082	A083	A084	A085	A086	A087	A088	A089	A090	A091
0	0	1	0	2	0	2	0	0	0	0	0	0	0	0	0	0	2	0	1	0	0	0	0	0	0	0	0	0	0	0	0	0	0	0	0	0	0	0	1	0	0	0	1	1	0	0	0	0	1	0	1
105	105	140	127	57	57	137	110	133	127	97	105	101	121	119	121	102	2	129	129	129	129	129	129	129	129	129	129	129	129	129	129	129	129	135	131	130	131	98	134	85	87	126	87	87	136	120	135	136	156	129	134
140	139	137	131	121	120	134	128	138	138	86	132	95	142	133	134	106	126	146	146	146	146	146	146	146	146	146	146	146	146	146	146	146	146	135	148	141	140	130	143	118	137	138	138	137	131	133	146	85	140	144	145
118	118	138	132	103	102	136	125	130	123	81	117	97	125	129	127	86	86	135	135	135	135	135	135	135	135	135	135	135	135	135	135	135	135	132	138	130	130	106	140	100	95	134	95	96	133	120	135	129	154	140	133
138	138	132	129	139	139	143	136	140	147	102	133	101	148	135	134	113	134	148	148	148	148	148	148	148	148	148	148	148	148	148	148	148	148	144	150	143	143	134	149	136	139	138	139	139	142	134	150	68	142	146	147
129	129	130	145	116	115	128	129	118	120	142	124	137	106	136	108	129	132	77	77	77	77	77	77	77	77	77	77	77	77	77	77	77	77	124	81	112	113	129	120	136	110	109	110	110	142	143	138	144	150	139	116
132	132	126	131	131	131	139	129	125	141	140	139	124	141	135	138	132	130	134	134	134	134	134	134	134	134	134	134	134	134	134	134	134	134	134	137	131	130	117	128	130	135	124	136	135	84	106	119	123	126	130	137
94	94	140	129	93	92	123	115	116	104	111	94	117	83	124	99	98	89	110	110	110	110	110	110	110	110	110	110	110	110	110	110	110	110	119	113	123	123	107	136	102	0	133	1	1	139	128	136	145	154	135	106
113	113	143	134	127	127	128	114	128	115	120	95	119	116	122	107	122	109	114	114	114	114	114	114	114	114	114	114	114	114	114	114	114	114	128	124	112	110	118	133	121	109	120	110	109	139	133	143	145	154	135	128
136	136	130	137	125	125	131	126	117	128	141	116	131	123	132	101	134	134	109	109	109	109	109	109	109	109	109	109	109	109	109	109	109	109	124	94	119	118	123	128	127	118	117	118	118	135	132	134	142	145	139	124
136	136	130	137	125	125	131	126	117	128	141	116	131	123	132	101	134	134	109	109	109	109	109	109	109	109	109	109	109	109	109	109	109	109	124	94	119	118	123	128	127	118	117	118	118	135	132	134	142	145	139	124
136	136	130	137	125	125	131	126	117	128	141	116	131	123	132	101	134	134	109	109	109	109	109	109	109	109	109	109	109	109	109	109	109	109	124	94	119	118	123	128	127	118	117	118	118	135	132	134	142	145	139	124
136	136	130	137	125	125	131	126	117	128	141	116	131	123	132	101	134	134	109	109	109	109	109	109	109	109	109	109	109	109	109	109	109	109	124	94	119	118	123	128	127	118	117	118	118	135	132	134	142	145	139	124
131	131	128	147	124	124	133	130	117	120	144	124	137	120	139	105	134	134	78	79	78	78	78	78	78	78	78	78	78	78	78	78	78	78	125	77	105	106	131	117	135	127	111	127	127	133	129	123	140	137	137	132
126	126	130	135	116	116	131	128	127	121	145	107	133	115	129	92	129	129	104	104	104	104	104	104	104	104	104	104	104	104	104	104	104	104	119	96	124	125	122	130	123	114	113	114	114	144	135	133	148	150	125	120
99	98	131	120	102	102	134	113	126	121	124	111	109	125	111	123	112	100	135	135	135	135	135	135	135	135	135	135	135	135	135	135	135	135	128	135	129	128	97	132	102	114	117	115	114	132	120	141	136	146	136	132
133	133	139	136	115	115	128	136	126	123	143	121	139	123	133	101	142	137	90	90	90	90	90	90	90	90	90	90	90	90	90	90	90	90	128	89	112	111	138	129	133	119	119	119	119	141	142	137	145	147	140	126
133	133	139	136	115	115	128	136	126	123	143	121	139	123	133	101	142	137	90	90	90	90	90	90	90	90	90	90	90	90	90	90	90	90	128	89	112	111	138	129	133	119	119	119	119	141	142	137	145	147	140	126
142	141	140	133	142	142	142	126	143	149	115	133	108	143	142	140	120	137	152	152	152	152	152	152	152	152	152	152	152	152	152	152	152	152	145	151	150	150	134	149	143	143	141	143	143	139	132	148	70	140	148	146
131	131	135	140	126	125	128	127	121	99	139	117	137	115	129	117	136	120	79	80	79	79	79	79	79	79	79	79	79	79	79	79	79	79	124	102	79	81	131	117	129	124	105	124	124	137	137	132	144	147	141	122
116	115	124	126	136	136	130	127	124	119	138	127	130	112	122	116	134	131	125	125	125	125	125	125	125	125	125	125	125	125	125	125	125	125	132	123	112	112	124	115	123	128	76	128	128	128	135	135	137	144	131	124
111	111	136	137	102	100	130	115	125	116	108	108	108	117	121	109	101	107	116	116	116	116	116	116	116	116	116	116	116	116	116	116	116	116	120	125	127	128	107	133	108	97	126	97	97	141	129	132	129	146	138	125
134	134	131	140	124	124	127	135	132	125	142	122	140	119	131	100	134	139	90	91	90	90	90	90	90	90	90	90	90	90	90	90	90	90	129	87	112	111	132	126	124	125	119	125	125	140	137	134	145	144	139	127
122	121	130	130	133	131	126	121	122	107	133	118	135	96	133	115	137	121	111	112	111	111	111	111	111	111	111	111	111	111	111	111	111	111	126	111	75	76	133	118	125	120	88	120	120	139	138	136	144	146	134	119
122	122	132	130	88	88	139	122	135	133	107	118	96	133	130	121	93	89	136	136	136	136	136	136	136	136	136	136	136	136	136	136	136	136	132	137	130	131	103	142	105	111	138	111	111	140	128	142	114	149	128	137
122	122	132	130	88	88	139	122	135	133	107	118	96	133	130	121	93	89	136	136	136	136	136	136	136	136	136	136	136	136	136	136	136	136	132	137	130	131	103	142	105	111	138	111	111	140	128	142	114	149	128	137
122	122	132	130	88	88	139	122	135	133	107	118	96	133	130	121	93	89	136	136	136	136	136	136	136	136	136	136	136	136	136	136	136	136	132	137	130	131	103	142	105	111	138	111	111	140	128	142	114	149	128	137
138	138	123	132	144	144	127	129	121	131	143	133	142	124	128	126	141	145	129	129	129	129	129	129	129	129	129	129	129	129	129	129	129	129	118	127	121	121	142	82	141	137	112	137	137	136	133	135	146	144	139	108
110	110	140	130	97	97	136	122	133	126	77	100	109	116	126	112	73	86	135	135	135	135	135	135	135	135	135	135	135	135	135	135	135	135	133	148	133	134	104	142	103	93	134	93	93	144	132	138	129	150	136	128
127	127	132	114	126	126	75	109	65	120	135	123	131	125	129	124	128	135	114	114	114	114	114	114	114	114	114	114	114	114	114	114	114	114	0	128	128	128	123	103	133	119	136	120	120	142	133	129	145	147	131	87
122	122	129	134	133	133	97	105	107	130	137	126	133	116	131	113	132	127	117	117	117	117	117	117	117	117	117	117	117	117	117	117	117	117	93	122	131	132	119	110	125	118	121	118	118	147	134	133	146	144	127	83
131	131	136	144	136	136	133	130	122	122	144	126	136	109	134	103	143	132	89	89	89	89	89	89	89	89	89	89	89	89	89	89	89	89	125	97	111	110	126	121	139	116	115	116	116	138	128	127	144	142	139	121
141	140	137	126	134	134	139	123	137	143	107	137	110	142	139	137	115	129	148	148	148	148	148	148	148	148	148	148	148	148	148	148	148	148	141	146	147	147	133	151	132	138	138	138	138	141	135	152	79	142	146	150
129	129	126	115	113	112	128	118	126	138	97	130	100	135	128	121	110	105	136	136	136	136	136	136	136	136	136	136	136	136	136	136	136	136	128	138	128	128	112	139	107	122	125	122	122	130	118	136	110	145	133	136
105	105	137	129	95	95	120	106	117	112	110	100	95	102	124	106	98	87	117	117	117	117	117	117	117	117	117	117	117	117	117	117	117	117	118	120	129	128	97	131	93	60	125	61	60	135	121	127	135	148	130	117
119	118	139	131	108	107	129	119	128	135	92	112	92	124	133	123	90	97	134	134	134	134	134	134	134	134	134	134	134	134	134	134	134	134	126	144	132	133	105	139	109	100	133	100	100	138	130	142	120	147	135	131
1	0	141	124	116	116	132	123	131	98	124	105	119	116	102	111	114	107	128	128	128	128	128	128	128	128	128	128	128	128	128	128	128	128	127	131	122	122	113	133	116	94	121	95	95	137	132	143	142	149	135	119
1	0	141	124	116	116	132	123	131	98	124	105	119	116	102	111	114	107	128	128	128	128	128	128	128	128	128	128	128	128	128	128	128	128	127	131	122	122	113	133	116	94	121	95	95	137	132	143	142	149	135	119
1	0	141	124	116	116	132	123	131	98	124	105	119	116	102	111	114	107	128	128	128	128	128	128	128	128	128	128	128	128	128	128	128	128	127	131	122	122	113	133	116	94	121	95	95	137	132	143	142	149	135	119
0	1	141	124	116	116	132	123	131	98	124	105	120	116	102	111	114	107	128	128	128	128	128	128	128	128	128	128	128	128	128	128	128	128	127	131	122	122	113	133	116	94	122	95	95	137	132	143	143	149	135	119
1	0	141	124	116	116	132	123	131	98	124	105	119	116	102	111	114	107	128	128	128	128	128	128	128	128	128	128	128	128	128	128	128	128	127	131	122	122	113	133	116	94	121	95	95	137	132	143	142	149	135	119
141	141	0	129	141	140	131	137	128	144	141	139	137	139	135	136	135	140	138	138	138	138	138	138	138	138	138	138	138	138	138	138	138	138	132	138	132	132	142	125	138	140	118	140	140	118	127	136	137	130	127	128
124	124	129	0	128	127	105	102	119	134	132	122	123	135	104	127	127	127	145	145	145	145	145	145	145	145	145	145	145	145	145	145	145	145	114	148	132	132	129	136	126	129	132	129	130	133	140	148	131	147	145	114
116	116	141	128	0	2	132	119	129	120	113	113	112	126	119	121	114	59	121	121	121	121	121	121	121	121	121	121	121	121	121	121	121	121	126	122	130	130	107	139	88	93	137	94	93	141	122	137	143	156	131	133
116	116	140	127	2	0	132	118	129	119	112	113	112	125	118	121	114	59	120	120	120	120	120	120	120	120	120	120	120	120	120	120	120	120	126	121	128	128	107	139	88	92	137	93	92	141	122	136	143	156	131	131
132	132	131	105	132	132	0	116	109	134	136	125	137	126	118	125	140	138	122	123	122	122	122	122	122	122	122	122	122	122	122	122	122	122	75	137	136	136	135	118	130	123	135	124	123	139	144	143	147	147	137	100
123	123	137	102	119	118	116	0	111	124	130	122	119	116	111	113	122	110	131	131	131	131	131	131	131	131	131	131	131	131	131	131	131	131	109	129	133	133	120	130	116	115	117	115	115	133	129	140	137	145	137	94
131	131	128	119	129	129	109	111	0	127	134	125	126	119	129	126	125	133	117	117	117	117	117	117	117	117	117	117	117	117	117	117	117	117	65	126	124	125	125	106	130	116	125	116	117	136	139	134	139	149	133	94
98	98	144	134	120	119	134	124	127	0	133	88	135	82	105	111	134	129	104	104	104	104	104	104	104	104	104	104	104	104	104	104	104	104	120	119	95	96	124	129	134	104	114	104	105	137	127	141	149	149	141	119
124	124	141	132	113	112	136	130	134	133	0	119	102	130	131	125	78	97	141	141	141	141	141	141	141	141	141	141	141	141	141	141	141	141	135	149	137	137	120	144	117	111	137	111	111	143	132	146	111	149	137	133
105	105	139	122	113	113	125	122	125	88	119	0	110	94	109	99	107	107	121	121	121	121	121	121	121	121	121	121	121	121	121	121	121	121	123	122	118	119	117	131	120	94	113	94	95	144	135	143	138	152	139	123
120	119	137	123	112	112	137	119	126	135	102	110	0	136	122	126	78	101	138	138	138	138	138	138	138	138	138	138	138	138	138	138	138	138	131	138	136	136	120	140	113	117	135	117	117	128	124	139	101	142	140	139
116	116	139	135	126	125	126	116	119	82	130	94	136	0	127	100	117	122	106	106	106	106	106	106	106	106	106	106	106	106	106	106	106	106	125	114	113	113	123	122	132	83	105	84	84	143	138	140	147	150	132	101
102	102	135	104	119	118	118	111	129	105	131	109	122	127	0	127	122	121	136	136	136	136	136	136	136	136	136	136	136	136	136	136	136	136	129	139	124	125	115	129	123	124	126	124	124	137	133	145	140	146	143	121
111	111	136	127	121	121	125	113	126	111	125	99	126	100	127	0	119	122	103	103	103	103	103	103	103	103	103	103	103	103	103	103	103	103	124	105	127	127	119	129	112	99	110	99	100	144	135	135	138	146	130	111
114	114	135	127	114	114	140	122	125	134	78	107	78	117	122	119	0	103	129	129	129	129	129	129	129	129	129	129	129	129	129	129	129	129	128	142	135	135	103	139	107	98	133	98	98	141	129	138	123	147	143	130
107	107	140	127	59	59	138	110	133	129	97	107	101	122	121	122	103	0	129	129	129	129	129	129	129	129	129	129	129	129	129	129	129	129	135	131	131	132	100	134	87	89	126	89	89	136	121	135	136	156	130	134

128	128	138	145	121	120	122	131	117	104	141	121	138	106	136	103	129	129	0	1	0	0	0	0	0	0	0	0	0	0	0	0	0	0	114	84	108	108	130	118	133	110	113	111	110	139	135	130	151	146	140	122
128	128	138	145	121	120	123	131	117	104	141	121	138	106	136	103	129	129	1	0	1	1	1	1	1	1	1	1	1	1	1	1	1	1	114	84	108	108	130	118	133	110	113	111	109	139	135	130	151	146	140	122
128	128	138	145	121	120	122	131	117	104	141	121	138	106	136	103	129	129	0	1	0	0	0	0	0	0	0	0	0	0	0	0	0	0	114	84	108	108	130	118	133	110	113	111	110	139	135	130	151	146	140	122
128	128	138	145	121	120	122	131	117	104	141	121	138	106	136	103	129	129	0	1	0	0	0	0	0	0	0	0	0	0	0	0	0	0	114	84	108	108	130	118	133	110	113	111	110	139	135	130	151	146	140	122
128	128	138	145	121	120	122	131	117	104	141	121	138	106	136	103	129	129	0	1	0	0	0	0	0	0	0	0	0	0	0	0	0	0	114	84	108	108	130	118	133	110	113	111	110	139	135	130	151	146	140	122
128	128	138	145	121	120	122	131	117	104	141	121	138	106	136	103	129	129	0	1	0	0	0	0	0	0	0	0	0	0	0	0	0	0	114	84	108	108	130	118	133	110	113	111	110	139	135	130	151	146	140	122
128	128	138	145	121	120	122	131	117	104	141	121	138	106	136	103	129	129	0	1	0	0	0	0	0	0	0	0	0	0	0	0	0	0	114	84	108	108	130	118	133	110	113	111	110	139	135	130	151	146	140	122
128	128	138	145	121	120	122	131	117	104	141	121	138	106	136	103	129	129	0	1	0	0	0	0	0	0	0	0	0	0	0	0	0	0	114	84	108	108	130	118	133	110	113	111	110	139	135	130	151	146	140	122
128	128	138	145	121	120	122	131	117	104	141	121	138	106	136	103	129	129	0	1	0	0	0	0	0	0	0	0	0	0	0	0	0	0	114	84	108	108	130	118	133	110	113	111	110	139	135	130	151	146	140	122
128	128	138	145	121	120	122	131	117	104	141	121	138	106	136	103	129	129	0	1	0	0	0	0	0	0	0	0	0	0	0	0	0	0	114	84	108	108	130	118	133	110	113	111	110	139	135	130	151	146	140	122
128	128	138	145	121	120	122	131	117	104	141	121	138	106	136	103	129	129	0	1	0	0	0	0	0	0	0	0	0	0	0	0	0	0	114	84	108	108	130	118	133	110	113	111	110	139	135	130	151	146	140	122
128	128	138	145	121	120	122	131	117	104	141	121	138	106	136	103	129	129	0	1	0	0	0	0	0	0	0	0	0	0	0	0	0	0	114	84	108	108	130	118	133	110	113	111	110	139	135	130	151	146	140	122
128	128	138	145	121	120	122	131	117	104	141	121	138	106	136	103	129	129	0	1	0	0	0	0	0	0	0	0	0	0	0	0	0	0	114	84	108	108	130	118	133	110	113	111	110	139	135	130	151	146	140	122
128	128	138	145	121	120	122	131	117	104	141	121	138	106	136	103	129	129	0	1	0	0	0	0	0	0	0	0	0	0	0	0	0	0	114	84	108	108	130	118	133	110	113	111	110	139	135	130	151	146	140	122
128	128	138	145	121	120	122	131	117	104	141	121	138	106	136	103	129	129	0	1	0	0	0	0	0	0	0	0	0	0	0	0	0	0	114	84	108	108	130	118	133	110	113	111	110	139	135	130	151	146	140	122
128	128	138	145	121	120	122	131	117	104	141	121	138	106	136	103	129	129	0	1	0	0	0	0	0	0	0	0	0	0	0	0	0	0	114	84	108	108	130	118	133	110	113	111	110	139	135	130	151	146	140	122
127	127	132	114	126	126	75	109	65	120	135	123	131	125	129	124	128	135	114	114	114	114	114	114	114	114	114	114	114	114	114	114	114	114	0	128	128	128	123	103	133	119	136	120	120	142	133	129	145	147	131	87
131	131	138	148	122	121	137	129	126	119	149	122	138	114	139	105	142	131	84	84	84	84	84	84	84	84	84	84	84	84	84	84	84	84	128	0	114	112	130	126	132	113	116	114	113	138	136	131	147	148	138	126
122	122	132	132	130	128	136	133	124	95	137	118	136	113	124	127	135	131	108	108	108	108	108	108	108	108	108	108	108	108	108	108	108	108	128	114	0	2	127	114	129	123	107	123	123	135	131	132	140	141	145	132
122	122	132	132	130	128	136	133	125	96	137	119	136	113	125	127	135	132	108	108	108	108	108	108	108	108	108	108	108	108	108	108	108	108	128	112	2	0	127	114	130	123	107	124	123	134	131	132	140	141	145	132
113	113	142	129	107	107	135	120	125	124	120	117	120	123	115	119	103	100	130	130	130	130	130	130	130	130	130	130	130	130	130	130	130	130	123	130	127	127	0	127	92	107	119	108	107	139	115	128	142	153	129	133
133	133	125	136	139	139	118	130	106	129	144	131	140	122	129	129	139	134	118	118	118	118	118	118	118	118	118	118	118	118	118	118	118	118	103	126	114	114	127	0	139	136	114	137	136	130	130	127	148	143	133	113
116	116	138	126	88	88	130	116	130	134	117	120	113	132	123	112	107	87	133	133	133	133	133	133	133	133	133	133	133	133	133	133	133	133	133	132	129	130	92	139	0	102	126	102	102	140	134	135	142	150	132	132
94	94	140	129	93	92	123	115	116	104	111	94	117	83	124	99	98	89	110	110	110	110	110	110	110	110	110	110	110	110	110	110	110	110	119	113	123	123	107	136	102	0	133	1	1	139	128	136	145	154	135	106
122	121	118	132	137	137	135	117	125	114	137	113	135	105	126	110	133	126	113	113	113	113	113	113	113	113	113	113	113	113	113	113	113	113	136	116	107	107	119	114	126	133	0	133	133	129	135	135	143	144	132	118
95	95	140	129	94	93	124	115	116	104	111	94	117	84	124	99	98	89	111	111	111	111	111	111	111	111	111	111	111	111	111	111	111	111	120	114	123	124	108	137	102	1	133	0	2	140	128	137	145	154	136	106
95	95	140	130	93	92	123	115	117	105	111	95	117	84	124	100	98	89	110	109	110	110	110	110	110	110	110	110	110	110	110	110	110	110	120	113	123	123	107	136	102	1	133	2	0	139	128	136	145	154	135	107
137	137	118	133	141	141	139	133	136	137	143	144	128	143	137	144	141	136	139	139	139	139	139	139	139	139	139	139	139	139	139	139	139	139	142	138	135	134	139	130	140	139	129	140	139	0	98	95	137	113	106	142
132	132	127	140	122	122	144	129	139	127	132	135	124	138	133	135	129	121	135	135	135	135	135	135	135	135	135	135	135	135	135	135	135	135	133	136	131	131	115	130	134	128	135	128	128	98	0	92	134	120	95	144
143	143	136	148	137	136	143	140	134	141	146	143	139	140	145	135	138	135	130	130	130	130	130	130	130	130	130	130	130	130	130	130	130	130	129	131	132	132	128	127	135	136	135	137	136	95	92	0	151	117	85	139
143	142	137	131	143	143	147	137	139	149	111	138	101	147	140	138	123	136	151	151	151	151	151	151	151	151	151	151	151	151	151	151	151	151	145	147	140	140	142	148	142	145	143	145	145	137	134	151	0	137	150	150
149	149	130	147	156	156	147	145	149	149	149	152	142	150	146	146	147	156	146	146	146	146	146	146	146	146	146	146	146	146	146	146	146	146	147	148	141	141	153	143	150	154	144	154	154	113	120	117	137	0	127	151
135	135	127	145	131	131	137	137	133	141	137	139	140	132	143	130	143	130	140	140	140	140	140	140	140	140	140	140	140	140	140	140	140	140	131	138	145	145	129	133	132	135	132	136	135	106	95	85	150	127	0	135
119	119	128	114	133	131	100	94	94	119	133	123	139	101	121	111	130	134	122	122	122	122	122	122	122	122	122	122	122	122	122	122	122	122	87	126	132	132	133	113	132	106	118	106	107	142	144	139	150	151	135	0
114	114	135	133	125	125	136	130	128	123	137	111	126	113	136	80	132	126	101	101	101	101	101	101	101	101	101	101	101	101	101	101	101	101	129	83	115	115	124	126	126	104	112	105	104	136	126	138	140	148	133	125
116	116	127	114	106	106	132	114	130	136	117	125	107	134	122	120	106	101	131	131	131	131	131	131	131	131	131	131	131	131	131	131	131	131	129	133	126	126	96	134	93	114	110	114	114	128	119	134	134	148	136	130
122	122	129	134	133	133	97	105	107	130	137	126	133	116	131	113	132	127	117	117	117	117	117	117	117	117	117	117	117	117	117	117	117	117	93	122	131	132	119	110	125	118	121	118	118	147	134	133	146	144	127	83
110	110	143	115	84	83	130	113	132	128	107	100	84	121	115	115	98	61	136	136	136	136	136	136	136	136	136	136	136	136	136	136	136	136	135	140	134	135	101	139	87	90	131	90	90	141	128	138	132	155	136	129
109	109	141	121	92	92	128	116	118	127	99	100	87	125	123	117	93	73	129	129	129	129	129	129	129	129	129	129	129	129	129	129	129	129	119	131	130	131	98	138	82	87	133	87	88	139	123	134	135	154	132	130
109	109	141	121	92	92	128	116	118	127	99	100	87	125	123	117	93	73	129	129	129	129	129	129	129	129	129	129	129	129	129	129	129	129	119	131	130	131	98	138	82	87	133	87	88	139	123	134	135	154	132	130
109	109	141	121	92	92	128	116	118	127	99	100	87	125	123	117	93	73	129	129	129	129	129	129	129	129	129	129	129	129	129	129	129	129	119	131	130	131	98	138	82	87	133	87	88	139	123	134	135	154	132	130
109	109	141	121	92	92	128	116	118	127	99	100	87	125	123	117	93	73	129	129	129	129	129	129	129	129	129	129	129	129	129	129	129	129	119	131	130	131	98	138	82	87	133	87	88	139	123	134	135	154	132	130
129	129	133	140	107	107	123	138	121	119	138	121	126	131	126	117	125	115	85	85	85	85	85	85	85	85	85	85	85	85	85	85	85	85	129	102	125	125	118	129	115	112	115	112	112	139	133	137	139	149	141	130
103	103	138	132	114	113	128	118	118	107	127	121	127	121	129	114	119	118	110	110	110	110	110	110	110	110	110	110	110	110	110	110	110	110	119	116	114	114	108	126	108	106	122	107	107	134	132	133	146	152	142	122
129	128	135	127	125	125	142	122	128	146	86	129	90	140	137	137	90	119	146	146	146	146	146	146	146	146	146	146	146	146	146	146	146	146	133	145	138	138	130	139	127	134	139	134	134	136	129	146	84	144	145	142
116	116	142	124	91	90	130	120	122	123	104	107	110	130	118	112	99	107	126	126	126	126	126	126	126	126	126	126	126	126	126	126	126	126	125	135	125	126	113	139	107	111	138	111	112	138	132	143	132	153	139	127
118	118	142	133	94	93	135	113	125	127	114	113	118	124	127	124	109	84	113	114	113	113	113	113	113	113	113	113	113	113	113	113	113	113	133	120	130	130	106	127	102	106	123	107	106	134	130	137	144	156	131	122
127	127	140	142	121	121	130	129	124	113	143	119	138	118	134	101	138	136	90	90	90	90	90	90	90	90	90	90	90	90	90	90	90	90	119	87	117	116	133	126	127	122	120	122	122	135	133	132	152	150	134	127
127	127	140	142	121	121	130	129	124	113	143	119	138	118	134	101	138	136	90	90	90	90	90	90	90	90	90	90	90	90	90	90	90	90	119	87	117	116	133	126	127	122	120	122	122	135	133	132	152	150	134	127
127	127	140	142	121	121	130	129	124	113	143	119	138	118	134	101	138	136	90	90	90	90	90	90	90	90	90	90	90	90	90	90	90	90	119	87	117	116	133	126	127	122	120	122	122	135	133	132	152	150	134	127
127	127	140	142	121	121	130	129	124	113	143	119	138	118	134	101	138	136	90	90	90	90	90	90	90	90	90	90	90	90	90	90	90	90	119	87	117	116	133	126	127	122	120	122	122	135	133	132	152	150	134	127
122	122	132	142	121	121	133	124	132	128	136	127	137	119	144	98	136	122	98	99	98	98	98	98	98	98	98	98	98	98	98	98	98	98	131	92	115	113	123	126	126	109	114	110	109	138	129	125	145	143	134	131
138	137	138	130	138	138	142	131	143	146	109	132	109	146	136	138	120	135	150	150	150	150	150	150	150	150	150	150	150	150	150	150	150	150	146	149	144	144	138	151	139	143	143	143	143	145	138	150	64	142	146	149
116	115	124	126	136	136	130	127	124	119	138	127	130	112	122	116	134	131	125	125	125	125	125	125	125	125	125	125	125	125	125	125	125	125	132	123	112	112	124	115	123	128	76	128	128	128	135	135	137	144	131	124
101	101	138	130	87	86	134	113	124	122	96	100	92	120	120	116	98	65	127	127	127	127	127	127	127	127	127	127	127	127	127	127	127	127	126	134	127	128	98	132	91	90	126	90	90	133	115	133	131	152	130	130
104	104	140	135	103	103	120	122	132	121	135	113	125	113	136	103	122	93	99	100	99	99	99	99	99	99	99	99	99	99	99	99	99	99	130	116	130	130	118	129	107	68	125	69	68	145	133	133	146	153	129	120
104	104	140	135	104	104	120	122	132	121	136	113	126	113	136	103	123	94	100	101	100	100	100	100	100	100	100	100	100	100	100	100	100	100	130	117	130	130	119	129	107	69	125	70	69	145	134	133	146	153	129	120
104	104	140	135	103	103	120	122	132	121	135	113	125	113	136	103	122	93	99	100	99	99	99	99	99	99	99	99	99	99	99	99	99	99	130	116	130	130	118	129	107	68	125	69	68	145	133	133	146	153	129	120
104	104	140	135	103	103	120	122	132	121	135	113	125	113	136	103	122	93	99	100	99	99	99	99	99	99	99	99	99	99	99	99	99	99	130	116	130	130	118	129	107	68	125	69	68	145	133	133	146	153	129	120
104	104	140	135	103	103	120	122	132	121	135	113	125	113	136	103	122	93	99	100	99	99	99	99	99	99	99	99	99	99	99	99	99	99	130	116	130	130	118	129	107	68	125	69	68	145	133	133	146	153	129	120
104	104	140	135	103	103	120	122	132	121	135	113	125	113	136	103	122	93	99	100	99	99	99	99	99	99	99	99	99	99	99	99	99	99	130	116	130	130	118	129	107	68	125	69	68	145	133	133	146	153	129	120
104	104	140	135	103	103	120	122	132	121	135	113	125	113	136	103	122	93	99	100	99	99	99	99	99	99	99	99	99	99	99	99	99	99	130	116	130	130	118	129	107	68	125	69	68	145	133	133	146	153	129	120
104	104	140	135	103	103	120	122	132	121	135	113	125	113	136	103	122	93	99	100	99	99	99	99	99	99	99	99	99	99	99	99	99	99	130	116	130	130	118	129	107	68	125	69	68	145	133	133	146	153	129	120
104	104	140	135	103	103	120	122	132	121	135	113	125	113	136	103	122	93	99	100	99	99	99	99	99	99	99	99	99	99	99	99	99	99	130	116	130	130	118	129	107	68	125	69	68	145	133	133	146	153	129	120
104	104	140	135	103	103	120	122	132	121	135	113	125	113	136	103	122	93	99	100	99	99	99	99	99	99	99	99	99	99	99	99	99	99	130	116	130	130	118	129	107	68	125	69	68	145	133	133	146	153	129	120
104	104	140	135	103	103	120	122	132	121	135	113	125	113	136	103	122	93	99	100	99	99	99	99	99	99	99	99	99	99	99	99	99	99	130	116	130	130	118	129	107	68	125	69	68	145	133	133	146	153	129	120
104	104	140	135	103	103	120	122	132	121	135	113	125	113	136	103	122	93	99	100	99	99	99	99	99	99	99	99	99	99	99	99	99	99	130	116	130	130	118	129	107	68	125	69	68	145	133	133	146	153	129	120
104	104	140	135	103	103	120	122	132	121	135	113	125	113	136	103	122	93	99	100	99	99	99	99	99	99	99	99	99	99	99	99	99	99	130	116	130	130	118	129	107	68	125	69	68	145	133	133	146	153	129	120
104	104	140	135	103	103	120	122	132	121	135	113	125	113	136	103	122	93	99	100	99	99	99	99	99	99	99	99	99	99	99	99	99	99	130	116	130	130	118	129	107	68	125	69	68	145	133	133	146	153	129	120
104	104	140	135	103	103	120	122	132	121	135	113	125	113	136	103	122	93	99	100	99	99	99	99	99	99	99	99	99	99	99	99	99	99	130	116	130	130	118	129	107	68	125	69	68	145	133	133	146	153	129	120
104	104	140	135	103	103	120	122	132	121	135	113	125	113	136	103	122	93	99	100	99	99	99	99	99	99	99	99	99	99	99	99	99	99	130	116	130	130	118	129	107	68	125	69	68	145	133	133	146	153	129	120
104	104	140	135	103	103	120	122	132	121	135	113	125	113	136	103	122	93	99	100	99	99	99	99	99	99	99	99	99	99	99	99	99	99	130	116	130	130	118	129	107	68	125	69	68	145	133	133	146	153	129	120
104	104	140	135	103	103	120	122	132	121	135	113	125	113	136	103	122	93	99	100	99	99	99	99	99	99	99	99	99	99	99	99	99	99	130	116	130	130	118	129	107	68	125	69	68	145	133	133	146	153	129	120
104	104	140	135	103	103	120	122	132	121	135	113	125	113	136	103	122	93	99	100	99	99	99	99	99	99	99	99	99	99	99	99	99	99	130	116	130	130	118	129	107	68	125	69	68	145	133	133	146	153	129	120
104	104	140	135	103	103	120	122	132	121	135	113	125	113	136	103	122	93	99	100	99	99	99	99	99	99	99	99	99	99	99	99	99	99	130	116	130	130	118	129	107	68	125	69	68	145	133	133	146	153	129	120

104	104	140	135	103	103	120	122	132	121	135	113	125	113	136	103	122	93	99	100	99	99	99	99	99	99	99	99	99	99	99	99	99	99	130	116	130	130	118	129	107	68	125	69	68	145	133	133	146	153	129	120
113	113	142	129	107	107	135	120	125	124	120	117	120	123	115	119	103	100	130	130	130	130	130	130	130	130	130	130	130	130	130	130	130	130	123	130	127	127	0	127	92	107	119	108	107	139	115	128	142	153	129	133
126	126	130	140	137	137	133	129	132	95	149	116	141	108	130	116	142	138	101	102	101	101	101	101	101	101	101	101	101	101	101	101	101	101	130	104	94	95	127	108	145	129	80	129	129	129	125	132	146	138	137	121
133	133	133	142	136	136	133	131	128	104	145	115	140	102	134	119	136	140	96	97	96	96	96	96	96	96	96	96	96	96	96	96	96	96	128	111	101	102	130	107	142	133	85	133	133	131	130	127	145	140	135	118
112	112	137	127	96	96	128	125	121	120	112	107	102	113	121	122	97	78	113	113	113	113	113	113	113	113	113	113	113	113	113	113	113	113	126	129	116	117	105	125	100	85	126	85	85	135	129	134	135	155	132	126
125	125	133	147	128	128	131	130	127	120	144	129	137	109	140	106	131	136	67	67	67	67	67	67	67	67	67	67	67	67	67	67	67	67	127	59	115	113	130	126	130	116	116	117	116	148	142	134	147	149	140	126
116	116	127	114	106	106	132	114	130	136	117	125	107	134	122	119	106	100	131	131	131	131	131	131	131	131	131	131	131	131	131	131	131	131	129	134	126	126	95	134	93	114	110	114	114	128	119	134	134	148	136	130
116	116	127	114	106	106	132	114	130	136	117	125	107	134	122	119	106	100	131	131	131	131	131	131	131	131	131	131	131	131	131	131	131	131	129	134	126	126	95	134	93	114	110	114	114	128	119	134	134	148	136	130
116	116	127	114	106	106	132	114	130	136	117	125	107	134	122	119	106	100	131	131	131	131	131	131	131	131	131	131	131	131	131	131	131	131	129	134	126	126	95	134	93	114	110	114	114	128	119	134	134	148	136	130
116	116	127	115	106	106	132	114	131	136	117	125	107	134	122	119	106	100	131	131	131	131	131	131	131	131	131	131	131	131	131	131	131	131	129	134	126	126	95	134	93	114	110	114	114	128	119	134	134	148	136	131
116	116	127	114	106	106	132	114	130	136	117	125	107	134	122	119	106	100	131	131	131	131	131	131	131	131	131	131	131	131	131	131	131	131	129	134	126	126	95	134	93	114	110	114	114	128	119	134	134	148	136	130
136	136	122	121	134	134	108	122	79	137	137	132	134	125	128	129	130	127	124	124	124	124	124	124	124	124	124	124	124	124	124	124	124	124	77	125	110	111	122	86	124	129	120	129	129	133	132	126	144	148	134	101
137	136	142	130	130	130	143	126	142	147	100	127	93	135	137	136	102	123	149	149	149	149	149	149	149	149	149	149	149	149	149	149	149	149	143	146	142	142	130	146	133	134	141	134	134	136	131	149	76	145	144	150
136	136	134	143	124	124	130	132	126	117	143	125	136	119	136	103	139	130	75	76	75	75	75	75	75	75	75	75	75	75	75	75	75	75	127	88	102	101	137	119	136	123	113	123	123	127	124	121	142	139	135	129
127	127	137	148	126	124	131	134	117	102	148	123	140	123	128	125	142	132	77	78	77	77	77	77	77	77	77	77	77	77	77	77	77	77	128	99	84	84	126	113	133	124	111	124	124	140	133	131	146	146	139	128
110	110	134	127	98	98	131	126	127	129	122	116	126	128	123	110	110	105	128	128	128	128	128	128	128	128	128	128	128	128	128	128	128	128	129	125	121	122	106	139	94	90	124	90	90	142	132	142	141	155	137	130
111	111	136	127	99	99	131	126	128	130	122	117	126	128	124	111	111	106	128	128	128	128	128	128	128	128	128	128	128	128	128	128	128	128	129	126	121	122	107	140	95	91	125	91	91	143	133	143	142	155	137	131
110	110	134	127	98	98	131	126	127	129	122	116	126	128	123	110	110	105	128	128	128	128	128	128	128	128	128	128	128	128	128	128	128	128	129	125	121	122	106	139	94	90	124	90	90	142	132	142	141	155	137	130
110	110	134	127	98	98	131	126	127	129	122	116	126	128	123	110	110	105	128	128	128	128	128	128	128	128	128	128	128	128	128	128	128	128	129	125	121	122	106	139	94	90	124	90	90	142	132	142	141	155	137	130
110	110	134	127	98	98	131	126	127	129	122	116	126	128	123	110	110	105	128	128	128	128	128	128	128	128	128	128	128	128	128	128	128	128	129	125	121	122	106	139	94	90	124	90	90	142	132	142	141	155	137	130
110	110	134	127	98	98	131	126	127	129	122	116	126	128	123	110	110	105	128	128	128	128	128	128	128	128	128	128	128	128	128	128	128	128	129	125	121	122	106	139	94	90	124	90	90	142	132	142	141	155	137	130
110	110	134	127	98	98	131	126	127	129	122	116	126	128	123	110	110	105	128	128	128	128	128	128	128	128	128	128	128	128	128	128	128	128	129	125	121	122	106	139	94	90	124	90	90	142	132	142	141	155	137	130
110	110	134	127	98	98	131	126	127	129	122	116	126	128	123	110	110	105	128	128	128	128	128	128	128	128	128	128	128	128	128	128	128	128	129	125	122	123	106	139	94	90	124	90	90	142	132	142	141	155	136	130
110	110	134	127	98	98	131	126	127	129	122	116	126	128	123	110	110	105	128	128	128	128	128	128	128	128	128	128	128	128	128	128	128	128	129	125	121	122	106	139	94	90	124	90	90	142	132	142	141	155	137	130
110	110	134	127	98	98	131	126	127	129	122	116	126	128	123	110	110	105	128	128	128	128	128	128	128	128	128	128	128	128	128	128	128	128	129	125	121	122	106	139	94	90	124	90	90	142	132	142	141	155	137	130
110	110	134	127	98	98	131	126	127	129	122	116	126	128	123	110	110	105	128	128	128	128	128	128	128	128	128	128	128	128	128	128	128	128	129	125	121	122	106	139	94	90	124	90	90	142	132	142	141	155	137	130
110	110	134	127	98	98	131	126	127	129	122	116	126	128	123	110	110	105	128	128	128	128	128	128	128	128	128	128	128	128	128	128	128	128	129	125	121	122	106	139	94	90	124	90	90	142	132	142	141	155	137	130
110	110	134	127	98	98	131	126	127	129	122	116	126	128	123	110	110	105	128	128	128	128	128	128	128	128	128	128	128	128	128	128	128	128	129	125	121	122	106	139	94	90	124	90	90	142	132	142	141	155	137	130
110	110	134	127	98	98	131	126	127	129	122	116	126	128	123	110	110	105	128	128	128	128	128	128	128	128	128	128	128	128	128	128	128	128	129	125	121	122	106	139	94	90	124	90	90	142	132	142	141	155	137	130
110	110	134	127	98	98	131	126	127	129	122	116	126	128	123	110	110	105	128	128	128	128	128	128	128	128	128	128	128	128	128	128	128	128	129	125	121	122	106	139	94	90	124	90	90	142	132	142	141	155	137	130
110	110	134	127	98	98	131	126	127	129	122	116	126	128	123	110	110	105	128	128	128	128	128	128	128	128	128	128	128	128	128	128	128	128	129	125	121	122	106	139	94	90	124	90	90	142	132	142	141	155	137	130
110	110	134	127	98	98	131	126	127	129	122	116	126	128	123	110	110	105	128	128	128	128	128	128	128	128	128	128	128	128	128	128	128	128	129	125	121	122	106	139	94	90	124	90	90	142	132	142	141	155	137	130
110	110	134	127	98	98	131	126	127	129	122	116	126	128	123	110	110	105	128	128	128	128	128	128	128	128	128	128	128	128	128	128	128	128	129	125	121	122	106	139	94	90	124	90	90	142	132	142	141	155	137	130
110	110	134	127	98	98	131	126	127	129	122	116	126	128	123	110	110	105	128	128	128	128	128	128	128	128	128	128	128	128	128	128	128	128	129	125	121	122	106	139	94	90	124	90	90	142	132	142	141	155	137	130
110	110	134	127	98	98	131	126	127	129	122	116	126	128	123	110	110	105	128	128	128	128	128	128	128	128	128	128	128	128	128	128	128	128	129	125	121	122	106	139	94	90	124	90	90	142	132	142	141	155	137	130
109	109	134	126	98	98	131	126	126	128	121	115	125	127	122	109	109	105	128	128	128	128	128	128	128	128	128	128	128	128	128	128	128	128	128	124	120	121	105	139	94	89	124	89	89	142	132	142	142	155	137	129
128	128	132	143	142	142	131	132	130	112	148	123	144	113	130	121	147	141	105	106	105	105	105	105	105	105	105	105	105	105	105	105	105	105	134	108	106	108	138	104	145	141	98	141	141	133	132	132	146	138	137	131
128	128	127	142	142	141	135	130	128	107	149	117	146	110	129	122	145	141	109	110	109	109	109	109	109	109	109	109	109	109	109	109	109	109	134	105	96	98	134	101	148	138	100	138	138	132	132	128	146	139	135	126
128	128	127	142	142	141	135	131	128	108	149	118	146	111	129	122	145	141	109	110	109	109	109	109	109	109	109	109	109	109	109	109	109	109	134	106	97	99	134	101	148	138	100	138	138	132	132	128	146	139	135	126
128	128	127	142	142	141	135	130	128	107	149	117	146	110	129	122	145	141	109	110	109	109	109	109	109	109	109	109	109	109	109	109	109	109	134	105	96	98	134	101	148	138	100	138	138	132	132	128	146	139	135	126
128	128	132	143	142	142	131	132	130	112	148	123	144	113	130	121	147	141	105	106	105	105	105	105	105	105	105	105	105	105	105	105	105	105	134	108	106	108	138	104	145	141	98	141	141	133	132	132	146	138	137	131
128	128	132	143	142	142	131	132	130	112	148	123	144	113	130	121	147	141	105	106	105	105	105	105	105	105	105	105	105	105	105	105	105	105	134	108	106	108	138	104	145	141	98	141	141	133	132	132	146	138	137	131
136	136	133	143	136	135	136	132	127	110	149	121	142	106	136	122	144	139	103	104	103	103	103	103	103	103	103	103	103	103	103	103	103	103	133	105	85	87	129	117	142	133	90	133	133	137	130	127	146	139	138	125
128	128	127	142	142	141	135	130	128	107	149	117	146	110	129	122	145	141	109	110	109	109	109	109	109	109	109	109	109	109	109	109	109	109	134	105	96	98	134	101	148	138	100	138	138	132	132	128	146	139	135	126
128	128	127	142	142	141	135	130	128	107	149	117	146	110	129	122	145	141	109	110	109	109	109	109	109	109	109	109	109	109	109	109	109	109	134	105	96	98	134	101	148	138	100	138	138	132	132	128	146	139	135	126
128	128	127	142	142	141	135	130	128	107	149	117	146	110	129	122	145	141	109	110	109	109	109	109	109	109	109	109	109	109	109	109	109	109	134	105	96	98	134	101	148	138	100	138	138	132	132	128	146	139	135	126
128	128	132	143	142	142	131	132	130	112	148	123	144	113	130	121	147	141	105	106	105	105	105	105	105	105	105	105	105	105	105	105	105	105	134	108	106	108	138	104	145	141	98	141	141	133	132	132	146	138	137	131
128	128	127	142	142	141	135	130	128	107	149	117	146	110	129	122	145	141	109	110	109	109	109	109	109	109	109	109	109	109	109	109	109	109	134	105	96	98	134	101	148	138	100	138	138	132	132	128	146	139	135	126
122	121	130	130	133	131	126	121	122	107	133	118	135	96	133	115	137	121	111	112	111	111	111	111	111	111	111	111	111	111	111	111	111	111	126	111	75	76	133	118	125	120	88	120	120	139	138	136	144	146	134	119
129	129	126	115	113	112	128	118	126	138	97	130	100	135	128	121	110	105	136	136	136	136	136	136	136	136	136	136	136	136	136	136	136	136	128	138	128	128	112	139	107	122	125	122	122	130	118	136	110	145	133	136
120	120	141	139	115	115	137	123	126	128	131	124	128	119	137	99	129	116	111	111	111	111	111	111	111	111	111	111	111	111	111	111	111	111	125	101	121	120	121	131	118	114	123	114	115	143	130	130	147	152	130	123
120	120	141	139	115	115	137	123	126	128	131	124	128	119	137	99	129	116	111	111	111	111	111	111	111	111	111	111	111	111	111	111	111	111	125	101	121	120	121	131	118	114	123	114	115	143	130	130	147	152	130	123
120	120	141	139	115	115	137	123	126	128	131	124	128	119	137	99	129	116	111	111	111	111	111	111	111	111	111	111	111	111	111	111	111	111	125	101	121	120	121	131	118	114	123	114	115	143	130	130	147	152	130	123
120	120	141	139	115	115	137	123	126	128	131	124	128	119	137	99	129	116	111	111	111	111	111	111	111	111	111	111	111	111	111	111	111	111	125	101	121	120	121	131	118	114	123	114	115	143	130	130	147	152	130	123
117	117	138	133	115	115	130	129	116	126	123	121	124	127	128	109	121	116	114	114	114	114	114	114	114	114	114	114	114	114	114	114	114	114	131	108	119	118	112	139	111	105	122	105	105	142	128	140	143	152	141	131
111	110	139	131	93	93	137	120	133	131	123	110	109	122	135	111	101	104	118	118	118	118	118	118	118	118	118	118	118	118	118	118	118	118	124	131	136	136	114	139	101	110	127	111	110	142	132	137	130	151	133	126
112	112	133	134	128	128	138	125	111	129	130	118	129	122	138	104	121	119	105	105	105	105	105	105	105	105	105	105	105	105	105	105	105	105	126	108	112	113	112	124	121	105	108	105	106	133	131	133	144	153	135	131
122	122	132	132	130	128	136	133	124	95	137	118	136	113	124	127	135	131	108	108	108	108	108	108	108	108	108	108	108	108	108	108	108	108	128	114	0	2	127	114	129	123	107	123	123	135	131	132	140	141	145	132
95	95	140	130	94	93	123	116	116	104	112	95	118	85	124	101	100	91	110	110	110	110	110	110	110	110	110	110	110	110	110	110	110	110	119	113	123	123	109	136	103	2	134	3	3	139	129	136	145	154	135	106
129	128	135	127	125	125	142	122	128	146	86	129	90	140	137	137	90	119	146	146	146	146	146	146	146	146	146	146	146	146	146	146	146	146	133	145	138	138	130	139	127	134	139	134	134	136	129	146	84	144	145	142
118	117	133	137	135	135	133	121	128	107	142	124	134	101	137	107	130	133	108	109	108	108	108	108	108	108	108	108	108	108	108	108	108	108	125	115	114	114	126	119	139	115	100	115	115	132	124	128	141	140	136	120
94	94	140	129	93	92	123	115	116	104	111	94	117	83	124	99	98	89	110	110	110	110	110	110	110	110	110	110	110	110	110	110	110	110	119	113	123	123	107	136	102	0	133	1	1	139	128	136	145	154	135	106
94	94	140	129	93	92	123	115	116	104	111	94	117	83	124	99	98	89	110	110	110	110	110	110	110	110	110	110	110	110	110	110	110	110	119	113	123	123	107	136	102	0	133	1	1	139	128	136	145	154	135	106
94	94	140	129	93	92	123	115	116	104	111	94	117	83	124	99	98	89	110	110	110	110	110	110	110	110	110	110	110	110	110	110	110	110	119	113	123	123	107	136	102	0	133	1	1	139	128	136	145	154	135	106
144	143	140	133	142	142	142	134	142	152	101	141	101	148	138	138	118	140	150	150	150	150	150	150	150	150	150	150	150	150	150	150	150	150	146	147	148	148	145	152	146	150	145	150	150	142	140	152	61	137	147	149
105	105	137	129	95	95	120	106	117	112	110	100	95	102	124	106	98	87	117	117	117	117	117	117	117	117	117	117	117	117	117	117	117	117	118	120	129	128	97	131	93	60	125	61	60	135	121	127	135	148	130	117
94	93	143	135	127	126	124	119	132	52	127	104	134	84	115	103	132	127	116	116	116	116	116	116	116	116	116	116	116	116	116	116	116	116	125	125	100	99	123	128	126	103	104	104	104	144	137	144	143	150	134	117
112	111	135	127	99	98	140	121	130	135	97	118	93	129	121	118	92	89	135	135	135	135	135	135	135	135	135	135	135	135	135	135	135	135	136	138	133	132	107	138	100	99	132	100	99	138	124	141	124	150	134	132
134	134	132	139	125	125	130	133	125	121	148	127	138	116	133	101	136	135	79	80	79	79	79	79	79	79	79	79	79	79	79	79	79	79	128	82	111	110	132	125	127	122	106	122	122	139	136	132	148	147	135	129
128	128	135	139	121	121	128	126	114	113	141	109	132	111	133	93	130	132	74	75	74	74	74	74	74	74	74	74	74	74	74	74	74	74	114	85	116	115	127	122	128	121	107	121	121	137	132	129	147	147	128	124
102	101	140	129	79	77	135	112	131	131	100	110	101	133	118	121	100	60	141	141	141	141	141	141	141	141	141	141	141	141	141	141	141	141	131	135	127	128	91	130	86	100	130	100	100	135	119	131	132	152	129	134
123	122	138	127	83	83	137	121	129	137	107	123	110	130	131	122	95	93	134	134	134	134	134	134	134	134	134	134	134	134	134	134	134	134	126	137	131	131	106	141	92	113	132	114	113	142	134	142	132	154	131	135
110	110	142	138	104	102	127	126	128	106	136	98	129	105	125	102	119	110	86	86	86	86	86	86	86	86	86	86	86	86	86	86	86	86	123	117	122	122	118	132	116	78	127	79	79	144	134	136	148	155	131	110
113	113	139	126	112	112	137	116	130	121	94	102	79	121	121	119	98	87	137	137	137	137	137	137	137	137	137	137	137	137	137	137	137	137	137	142	129	129	106	140	96	95	132	95	95	131	124	138	125	145	142	131
135	135	127	137	131	131	129	133	130	119	144	128	136	123	131	102	133	139	82	83	82	82	82	82	82	82	82	82	82	82	82	82	82	82	125	95	111	112	132	119	132	128	116	128	128	133	129	121	149	140	136	131
111	111	138	128	70	70	135	117	138	131	101	115	112	126	120	122	109	25	124	124	124	124	124	124	124	124	124	124	124	124	124	124	124	124	138	133	128	129	104	133	97	102	126	102	102	138	122	136	126	153	135	134

139	139	112	115	138	138	116	113	98	137	142	140	137	129	121	133	134	141	131	131	131	131	131	131	131	131	131	131	131	131	131	131	131	131	93	135	128	128	132	95	132	136	123	136	136	133	130	130	145	142	131	107
107	107	143	131	109	109	117	126	117	122	119	100	118	118	128	99	114	112	116	116	116	116	116	116	116	116	116	116	116	116	116	116	116	116	111	109	130	131	118	136	117	93	126	93	94	143	133	133	136	153	137	126
120	119	135	134	112	111	124	129	118	134	129	119	116	121	139	103	119	117	116	116	116	116	116	116	116	116	116	116	116	116	116	116	116	116	117	103	130	128	114	131	120	102	122	103	103	145	137	140	134	156	132	123
109	109	137	133	102	101	122	121	122	132	123	114	118	118	125	117	118	99	124	124	124	124	124	124	124	124	124	124	124	124	124	124	124	124	124	119	129	130	109	135	109	84	128	84	85	144	132	135	137	152	130	124
106	106	139	131	97	97	135	114	129	113	134	102	118	116	127	96	113	99	119	119	119	119	119	119	119	119	119	119	119	119	119	119	119	119	121	121	129	130	96	134	100	101	123	101	102	143	120	134	148	154	131	119
124	123	134	141	122	120	127	126	128	124	140	105	127	113	136	89	133	132	94	95	94	94	94	94	94	94	94	94	94	94	94	94	94	94	126	98	124	125	126	128	128	109	115	109	109	140	129	134	141	153	130	126
121	121	136	137	114	114	137	124	128	132	133	115	127	129	134	109	127	112	127	127	127	127	127	127	127	127	127	127	127	127	127	127	127	127	132	112	119	120	112	142	112	106	127	106	106	142	132	139	141	153	142	135
122	122	138	139	126	125	123	137	117	127	131	122	132	128	131	115	130	124	106	106	106	106	106	106	106	106	106	106	106	106	106	106	106	106	120	113	119	120	117	130	117	115	120	115	115	142	132	136	146	151	140	130
140	140	143	126	132	132	132	130	142	145	121	138	120	146	133	137	127	131	153	153	153	153	153	153	153	153	153	153	153	153	153	153	153	153	142	152	146	146	136	151	139	145	146	145	145	140	142	152	124	139	144	142
91	91	134	133	116	116	125	129	123	111	119	108	130	115	117	118	114	110	112	112	112	112	112	112	112	112	112	112	112	112	112	112	112	112	119	123	105	106	115	129	126	105	110	105	106	142	136	144	141	148	137	125
120	120	142	141	118	117	126	125	121	110	140	114	135	110	136	90	131	121	94	95	94	94	94	94	94	94	94	94	94	94	94	94	94	94	116	99	113	114	118	126	115	111	115	111	111	142	131	136	146	151	136	121
142	141	136	131	143	143	147	129	143	149	111	134	103	144	139	137	122	133	152	152	152	152	152	152	152	152	152	152	152	152	152	152	152	152	150	150	146	146	139	149	134	139	137	139	139	141	138	152	49	141	150	148
140	139	140	128	138	138	140	126	143	147	108	133	109	142	138	137	113	135	147	147	147	147	147	147	147	147	147	147	147	147	147	147	147	147	146	147	147	147	136	150	139	137	137	137	137	142	138	153	77	142	145	145
119	119	138	143	116	115	132	130	124	123	126	118	130	117	136	100	123	123	113	113	113	113	113	113	113	113	113	113	113	113	113	113	113	113	133	104	121	119	121	138	114	97	122	98	97	144	132	139	140	151	135	124
130	130	131	140	129	129	124	127	125	125	138	114	135	106	136	76	125	131	87	88	87	87	87	87	87	87	87	87	87	87	87	87	87	87	120	94	120	121	121	118	129	115	112	115	115	139	124	130	145	149	129	127
111	111	134	136	112	112	137	118	118	120	127	118	126	120	134	86	115	112	106	106	106	106	106	106	106	106	106	106	106	106	106	106	106	106	126	111	121	122	116	132	113	95	110	95	95	133	123	129	140	146	131	123
120	120	138	137	119	119	128	125	121	121	127	120	119	127	131	102	124	119	108	108	108	108	108	108	108	108	108	108	108	108	108	108	108	108	126	116	119	118	121	131	120	101	122	101	101	135	127	133	136	147	136	132
108	108	144	135	115	114	130	125	129	119	122	117	138	113	131	103	119	108	110	111	110	110	110	110	110	110	110	110	110	110	110	110	110	110	132	116	126	126	117	140	111	79	122	80	79	147	142	143	146	157	138	120
128	128	135	138	131	131	124	130	120	124	135	119	135	105	134	88	127	133	90	90	90	90	90	90	90	90	90	90	90	90	90	90	90	90	119	82	115	116	124	124	127	112	117	112	112	138	131	135	148	149	133	123
100	100	133	142	131	131	125	131	122	119	121	117	125	111	130	114	114	121	100	101	100	100	100	100	100	100	100	100	100	100	100	100	100	100	123	113	113	113	124	127	131	110	109	110	110	134	127	135	134	146	132	129
94	93	129	126	122	122	134	117	121	120	131	105	119	98	127	99	119	114	112	112	112	112	112	112	112	112	112	112	112	112	112	112	112	112	124	114	110	111	124	125	110	115	95	115	116	137	138	138	132	147	130	113
124	124	138	138	126	125	124	130	121	123	130	120	134	120	134	96	130	124	104	104	104	104	104	104	104	104	104	104	104	104	104	104	104	104	117	92	127	126	115	132	118	99	122	99	99	142	134	137	150	153	135	122
119	119	138	142	119	117	121	136	117	113	131	100	132	120	128	103	126	128	74	74	74	74	74	74	74	74	74	74	74	74	74	74	74	74	115	101	107	107	122	122	135	105	121	106	105	144	140	132	149	153	132	121
140	139	136	126	139	139	143	133	138	148	110	136	102	145	135	143	120	136	152	152	152	152	152	152	152	152	152	152	152	152	152	152	152	152	146	149	143	143	138	149	139	146	140	146	146	138	137	154	53	143	150	148
107	107	133	136	120	120	130	130	124	118	124	108	127	102	133	107	117	107	90	90	90	90	90	90	90	90	90	90	90	90	90	90	90	90	127	103	116	114	121	126	121	83	110	84	84	138	132	135	150	154	130	119
125	124	128	137	116	115	127	128	123	135	135	121	119	123	141	112	125	119	111	112	111	111	111	111	111	111	111	111	111	111	111	111	111	111	125	102	127	126	118	129	123	113	114	113	113	136	132	139	131	150	130	130
127	127	129	136	129	129	126	128	120	122	140	114	136	104	136	80	133	130	85	86	85	85	85	85	85	85	85	85	85	85	85	85	85	85	125	97	117	118	120	118	124	121	106	121	121	138	122	120	142	140	125	121
120	120	133	134	121	120	130	116	125	123	126	109	121	112	134	106	122	118	103	103	103	103	103	103	103	103	103	103	103	103	103	103	103	103	123	105	116	115	125	134	120	99	108	99	99	139	131	138	142	153	140	121
106	106	137	135	116	115	112	123	124	115	123	108	122	111	126	74	116	111	98	98	98	98	98	98	98	98	98	98	98	98	98	98	98	98	122	107	125	125	118	130	108	87	122	87	88	146	137	135	140	152	132	108
126	126	131	140	119	119	130	122	126	122	138	113	133	116	139	84	127	131	94	95	94	94	94	94	94	94	94	94	94	94	94	94	94	94	127	93	123	122	129	132	126	116	112	116	116	141	136	136	139	150	132	127
121	121	133	137	124	124	133	126	117	119	139	116	136	111	139	80	131	126	95	95	95	95	95	95	95	95	95	95	95	95	95	95	95	95	121	89	122	123	123	127	126	113	116	113	114	142	128	136	145	149	135	125
113	113	143	136	110	109	120	121	116	124	107	114	127	119	127	108	113	109	118	118	118	118	118	118	118	118	118	118	118	118	118	118	118	118	119	114	126	124	113	139	108	71	129	72	72	143	133	135	141	157	132	125
1	0	141	124	116	116	132	123	131	98	124	105	119	116	102	111	114	107	128	128	128	128	128	128	128	128	128	128	128	128	128	128	128	128	127	131	122	122	113	133	116	94	121	95	95	137	132	143	142	149	135	119
134	134	135	136	123	123	135	132	127	118	134	122	136	129	134	103	128	140	97	97	97	97	97	97	97	97	97	97	97	97	97	97	97	97	125	103	117	116	126	133	123	120	122	120	120	136	129	134	141	149	139	134
126	126	138	133	121	121	137	127	135	132	96	113	111	119	130	124	96	106	144	144	144	144	144	144	144	144	144	144	144	144	144	144	144	144	137	148	144	144	118	143	119	108	138	108	108	143	128	142	100	147	141	137
119	119	141	137	109	108	124	123	133	123	133	112	126	113	130	91	120	111	96	97	96	96	96	96	96	96	96	96	96	96	96	96	96	96	134	108	128	128	125	135	118	93	122	93	93	141	129	137	141	150	135	127
120	120	137	138	118	116	119	130	126	118	122	115	127	119	132	106	126	113	98	99	98	98	98	98	98	98	98	98	98	98	98	98	98	98	125	106	124	124	125	128	117	90	117	90	90	142	129	131	142	150	133	130
111	111	137	127	90	89	117	122	121	122	125	117	124	118	125	102	119	103	118	118	118	118	118	118	118	118	118	118	118	118	118	118	118	118	121	115	125	125	108	136	103	95	124	96	95	133	126	126	141	150	125	126
122	122	138	139	114	113	122	124	124	125	125	110	126	115	130	97	123	120	108	109	108	108	108	108	108	108	108	108	108	108	108	108	108	108	126	120	124	125	114	136	120	91	130	91	91	148	137	140	141	152	133	125
119	119	139	137	123	122	130	128	132	122	122	113	132	121	137	109	119	123	119	119	119	119	119	119	119	119	119	119	119	119	119	119	119	119	126	113	121	120	123	143	118	95	121	95	95	145	138	146	139	156	143	129
130	130	138	139	117	117	130	134	118	122	139	114	133	121	135	102	123	128	75	75	75	75	75	75	75	75	75	75	75	75	75	75	75	75	125	100	118	117	114	129	122	120	120	120	120	143	131	137	149	148	137	130
119	119	137	134	119	119	123	121	124	118	131	103	127	108	133	78	127	115	84	85	84	84	84	84	84	84	84	84	84	84	84	84	84	84	123	93	121	122	123	120	123	98	115	98	98	137	125	137	139	147	134	121
129	129	141	125	122	122	129	129	136	141	117	123	122	137	131	126	124	118	146	146	146	146	146	146	146	146	146	146	146	146	146	146	146	146	140	151	141	142	137	147	122	131	141	131	131	146	144	152	114	144	145	140
130	130	138	137	119	119	132	129	125	123	135	122	131	128	127	116	131	137	113	113	113	113	113	113	113	113	113	113	113	113	113	113	113	113	126	114	120	121	120	134	122	112	126	112	112	137	133	133	139	148	135	138
113	113	136	137	99	98	125	126	129	119	125	103	126	115	132	102	122	106	115	115	115	115	115	115	115	115	115	115	115	115	115	115	115	115	125	117	128	128	108	136	105	78	127	79	78	146	129	133	148	150	127	121
112	112	139	130	103	102	130	130	132	116	125	117	130	126	122	100	115	113	118	118	118	118	118	118	118	118	118	118	118	118	118	118	118	118	126	117	124	124	111	135	104	93	129	94	93	137	127	131	144	154	134	133
125	125	134	141	125	125	136	127	122	119	136	131	134	120	142	100	127	129	100	100	100	100	100	100	100	100	100	100	100	100	100	100	100	100	124	94	108	107	125	127	126	110	116	110	110	136	125	130	141	146	141	131
131	131	135	137	123	123	127	123	122	128	132	127	130	119	134	101	127	126	111	111	111	111	111	111	111	111	111	111	111	111	111	111	111	111	125	111	125	124	123	129	116	110	119	110	110	136	131	125	138	147	132	133
105	105	136	129	95	95	132	119	131	125	111	110	104	120	127	106	104	93	127	127	127	127	127	127	127	127	127	127	127	127	127	127	127	127	129	124	127	128	106	133	102	95	131	95	96	136	121	137	136	152	133	122
127	127	135	132	117	117	130	128	123	122	141	109	138	112	138	79	137	132	93	94	93	93	93	93	93	93	93	93	93	93	93	93	93	93	130	86	126	125	131	127	131	110	118	110	110	140	134	132	141	148	132	121
137	137	134	139	129	128	136	127	127	121	145	128	140	120	137	105	138	140	96	96	96	96	96	96	96	96	96	96	96	96	96	96	96	96	125	92	114	113	132	127	140	118	113	118	118	137	141	135	141	149	137	127
114	114	138	142	106	105	130	123	129	114	131	103	133	108	135	89	124	104	104	104	104	104	104	104	104	104	104	104	104	104	104	104	104	104	128	113	115	116	117	138	121	77	111	77	77	150	135	133	144	151	132	119
112	112	135	139	120	118	125	122	124	118	128	104	123	111	131	100	128	118	101	101	101	101	101	101	101	101	101	101	101	101	101	101	101	101	121	102	113	112	116	129	120	94	111	94	94	136	129	131	143	155	128	121
112	112	141	135	112	112	120	123	125	122	113	113	129	117	135	105	119	119	107	108	107	107	107	107	107	107	107	107	107	107	107	107	107	107	122	112	130	130	123	138	116	101	129	102	101	135	129	131	134	147	134	129
113	113	142	139	114	114	125	122	126	114	129	99	140	103	132	83	120	120	102	102	102	102	102	102	102	102	102	102	102	102	102	102	102	102	120	107	114	115	122	127	125	84	118	84	85	145	137	136	148	152	139	107
107	107	140	130	90	90	126	120	121	118	133	112	124	120	129	103	120	102	115	115	115	115	115	115	115	115	115	115	115	115	115	115	115	115	119	109	125	126	114	140	99	85	126	85	86	140	128	133	139	154	131	125
123	123	138	130	108	107	123	136	125	129	132	111	130	125	125	118	132	125	116	116	116	116	116	116	116	116	116	116	116	116	116	116	116	116	126	106	132	131	125	138	124	106	132	106	106	142	137	144	142	157	138	131
112	112	135	139	120	118	125	122	124	118	128	104	123	111	131	100	128	118	101	101	101	101	101	101	101	101	101	101	101	101	101	101	101	101	121	102	113	112	116	129	120	94	111	94	94	136	129	131	143	155	128	121
119	119	138	129	94	92	131	124	126	126	120	100	101	126	113	124	98	90	115	115	115	115	115	115	115	115	115	115	115	115	115	115	115	115	134	127	135	136	119	133	101	116	124	116	116	144	140	149	139	158	142	128
120	119	138	129	113	112	133	126	137	136	103	112	105	121	134	121	92	108	137	137	137	137	137	137	137	137	137	137	137	137	137	137	137	137	131	138	134	135	117	139	108	106	136	106	106	143	137	141	111	149	136	136
108	108	141	132	109	108	123	123	129	119	116	95	111	100	129	93	98	94	98	98	98	98	98	98	98	98	98	98	98	98	98	98	98	98	122	126	125	125	113	127	104	68	125	69	69	145	129	134	145	156	132	112
129	129	135	140	115	113	127	130	118	120	137	118	131	123	128	100	134	132	89	89	89	89	89	89	89	89	89	89	89	89	89	89	89	89	113	91	123	124	125	129	129	113	124	113	114	144	133	130	147	153	130	121
109	109	136	122	117	117	132	123	126	124	71	104	100	124	114	120	79	106	147	147	147	147	147	147	147	147	147	147	147	147	147	147	147	147	129	148	132	132	124	140	121	112	132	112	112	137	128	142	108	144	140	132
112	112	135	134	121	120	125	121	117	123	124	118	123	116	128	89	119	121	101	102	101	101	101	101	101	101	101	101	101	101	101	101	101	101	121	114	127	128	123	133	118	101	116	101	101	143	133	135	138	152	130	120
117	117	140	134	114	114	122	125	115	130	122	120	133	119	133	112	124	105	107	108	107	107	107	107	107	107	107	107	107	107	107	107	107	107	125	110	133	131	112	137	119	78	125	79	78	143	133	137	149	155	136	124
120	120	138	137	121	119	124	124	119	120	131	113	131	107	137	95	132	121	96	96	96	96	96	96	96	96	96	96	96	96	96	96	96	96	115	97	119	120	124	125	128	110	115	110	111	142	130	136	139	153	138	118
131	131	135	139	116	116	138	126	117	111	144	111	130	114	137	90	133	130	87	87	87	87	87	87	87	87	87	87	87	87	87	87	87	87	121	88	121	121	127	124	136	125	115	125	126	139	122	125	134	145	132	126
109	109	135	129	105	105	132	123	129	134	89	113	115	118	125	124	82	96	134	134	134	134	134	134	134	134	134	134	134	134	134	134	134	134	132	142	132	133	105	141	107	97	133	97	97	143	131	141	124	151	136	134
122	122	138	141	112	110	130	126	120	120	129	121	136	116	135	104	133	122	94	94	94	94	94	94	94	94	94	94	94	94	94	94	94	94	118	92	114	113	122	131	131	111	114	111	111	140	137	137	141	155	135	120
115	115	141	134	115	114	132	131	122	123	129	113	120	121	128	120	121	120	117	117	117	117	117	117	117	117	117	117	117	117	117	117	117	117	120	111	117	115	118	138	121	102	127	103	103	137	130	137	142	154	137	135
112	112	140	139	123	122	125	123	125	120	123	112	126	101	133	81	114	121	103	104	103	103	103	103	103	103	103	103	103	103	103	103	103	103	131	116	131	131	124	129	113	79	118	79	79	143	133	135	143	152	137	111
139	139	145	133	147	147	137	139	135	147	137	132	129	147	139	137	145	142	150	150	150	150	150	150	150	150	150	150	150	150	150	150	150	150	140	149	142	143	144	147	146	146	147	146	147	145	143	143	130	148	139	144
119	119	141	135	114	113	125	132	122	117	126	109	134	120	133	107	127	114	98	98	98	98	98	98	98	98	98	98	98	98	98	98	98	98	120	108	122	122	120	128	119	93	125	94	93	147	139	139	149	157	141	119
114	114	136	135	110	109	118	124	117	122	111	118	124	115	129	115	117	107	97	98	97	97	97	97	97	97	97	97	97	97	97	97	97	97	126	119	124	122	117	132	116	75	127	76	75	148	139	142	141	153	137	116
116	116	142	136	110	110	130	125	116	128	127	130	132	116	132	113	127	106	104	104	104	104	104	104	104	104	104	104	104	104	104	104	104	104	126	115	132	130	114	131	118	81	130	82	82	144	130	134	147	158	133	121

125	125	140	141	97	96	133	126	132	121	122	120	131	114	137	97	115	106	101	102	101	101	101	101	101	101	101	101	101	101	101	101	101	101	129	113	133	133	120	134	115	96	128	96	96	142	127	130	137	151	135	125
107	107	143	135	110	109	134	125	131	125	119	117	118	108	131	112	113	110	118	118	118	118	118	118	118	118	118	118	118	118	118	118	118	118	127	119	125	124	120	135	116	85	121	86	85	143	131	135	142	158	139	116
133	133	137	142	124	123	126	131	124	115	142	121	133	112	135	99	134	135	75	75	75	75	75	75	75	75	75	75	75	75	75	75	75	75	126	91	118	119	123	123	129	121	114	121	121	143	132	127	146	149	132	124
127	127	137	137	128	128	125	125	123	116	129	118	134	106	136	91	135	132	97	97	97	97	97	97	97	97	97	97	97	97	97	97	97	97	121	92	124	124	128	128	136	108	112	108	109	140	132	137	138	152	136	123
118	118	138	138	112	112	134	125	124	125	142	116	134	120	126	109	128	129	121	121	121	121	121	121	121	121	121	121	121	121	121	121	121	121	126	106	123	124	122	138	118	99	127	99	99	147	140	143	141	154	141	117
117	117	142	137	130	129	132	134	123	121	133	117	136	115	138	102	125	121	111	111	111	111	111	111	111	111	111	111	111	111	111	111	111	111	126	101	116	115	125	135	126	88	122	88	89	142	141	143	142	157	141	121
103	102	127	131	111	111	130	124	132	124	119	122	106	124	127	122	116	110	133	133	133	133	133	133	133	133	133	133	133	133	133	133	133	133	129	127	130	129	114	133	115	103	125	104	103	117	114	127	125	136	123	129
109	109	145	132	101	101	119	126	127	119	126	108	127	116	126	103	113	110	109	110	109	109	109	109	109	109	109	109	109	109	109	109	109	109	122	120	131	132	116	142	105	78	132	78	78	146	132	138	145	155	135	127
124	124	137	137	121	120	126	120	121	127	131	120	130	110	132	98	120	125	101	101	101	101	101	101	101	101	101	101	101	101	101	101	101	101	118	106	130	130	123	128	116	105	122	106	105	143	139	136	141	154	135	110
128	127	141	128	98	98	135	126	124	134	110	121	112	124	128	119	109	78	116	116	116	116	116	116	116	116	116	116	116	116	116	116	116	116	133	121	132	131	109	133	110	97	126	97	98	142	137	142	130	156	136	129
113	113	143	138	110	109	127	127	119	122	119	119	121	122	132	115	120	98	103	103	103	103	103	103	103	103	103	103	103	103	103	103	103	103	124	105	122	121	114	133	119	98	119	98	98	138	129	137	138	154	135	130
120	120	134	132	125	124	125	126	126	128	129	116	127	111	133	113	122	124	113	113	113	113	113	113	113	113	113	113	113	113	113	113	113	113	126	111	123	122	127	139	130	102	115	102	102	143	141	143	134	157	144	125
122	122	136	137	114	112	135	131	116	116	133	106	134	121	134	91	132	127	83	83	83	83	83	83	83	83	83	83	83	83	83	83	83	83	117	87	117	118	123	126	132	104	118	104	105	144	133	134	145	157	138	123
115	115	139	137	110	108	126	128	124	114	128	109	129	115	128	86	131	121	102	102	102	102	102	102	102	102	102	102	102	102	102	102	102	102	125	99	121	122	125	133	116	86	120	86	87	141	125	132	140	152	129	128
125	124	135	140	121	120	124	124	121	124	137	117	126	112	134	93	141	130	91	91	91	91	91	91	91	91	91	91	91	91	91	91	91	91	117	99	124	124	130	126	129	99	119	100	99	138	135	133	134	151	133	123
90	90	136	136	111	110	123	128	116	117	123	122	105	124	127	120	116	102	109	109	109	109	109	109	109	109	109	109	109	109	109	109	109	109	112	114	120	118	113	123	116	87	124	88	88	136	128	131	136	142	138	120
120	120	140	138	108	107	136	137	123	117	139	111	131	124	128	114	133	128	108	108	108	108	108	108	108	108	108	108	108	108	108	108	108	108	121	92	120	118	126	136	132	102	123	103	103	141	136	140	138	157	138	130
125	125	134	139	129	128	124	128	118	129	129	124	136	109	140	97	123	126	83	84	83	83	83	83	83	83	83	83	83	83	83	83	83	83	126	106	122	123	123	118	129	102	115	102	102	146	136	135	147	150	133	120
141	140	135	127	137	137	145	125	139	146	114	132	108	146	139	136	122	135	153	153	153	153	153	153	153	153	153	153	153	153	153	153	153	153	144	147	146	146	137	146	135	145	136	145	145	138	132	147	47	140	148	148
113	113	144	136	119	117	122	133	128	116	121	113	122	122	135	112	130	120	89	90	89	89	89	89	89	89	89	89	89	89	89	89	89	89	126	105	123	121	132	134	122	89	126	90	89	142	135	134	140	153	139	128
121	121	144	137	124	123	133	120	125	119	131	114	140	105	140	89	131	124	99	99	99	99	99	99	99	99	99	99	99	99	99	99	99	99	124	97	119	120	123	135	121	82	122	82	82	147	140	141	151	157	135	110
117	117	140	133	124	122	123	123	120	123	133	121	130	110	130	85	138	124	105	105	105	105	105	105	105	105	105	105	105	105	105	105	105	105	126	101	129	130	121	127	114	93	119	93	94	140	131	129	144	152	130	121
132	132	134	139	132	132	127	129	127	126	139	113	141	110	137	81	130	131	92	92	92	92	92	92	92	92	92	92	92	92	92	92	92	92	121	95	121	122	126	127	127	116	115	116	116	143	135	133	152	148	131	125
123	123	132	136	113	112	127	133	124	115	135	112	135	119	138	95	130	122	105	105	105	105	105	105	105	105	105	105	105	105	105	105	105	105	125	96	111	111	126	127	128	105	120	105	105	141	125	137	139	149	142	135
115	115	144	140	109	108	127	126	125	109	143	108	130	115	128	101	134	114	100	100	100	100	100	100	100	100	100	100	100	100	100	100	100	100	120	101	120	120	126	131	121	96	126	97	97	140	139	129	144	152	137	119
124	124	137	140	119	118	130	129	124	132	127	130	134	115	136	120	124	119	103	103	103	103	103	103	103	103	103	103	103	103	103	103	103	103	130	102	122	120	122	130	118	99	119	100	99	142	140	140	152	157	140	117
110	110	135	130	108	106	125	124	120	114	127	117	131	108	133	91	122	113	102	103	102	102	102	102	102	102	102	102	102	102	102	102	102	102	125	115	132	133	125	132	116	83	122	83	83	140	125	132	140	153	137	117
127	127	135	132	119	118	127	126	120	124	131	119	128	117	126	91	128	124	96	96	96	96	96	96	96	96	96	96	96	96	96	96	96	96	120	95	118	119	122	130	120	114	117	114	114	144	138	134	144	149	131	118
117	117	142	136	98	97	124	121	128	125	118	108	120	105	134	107	115	99	111	111	111	111	111	111	111	111	111	111	111	111	111	111	111	111	128	113	126	126	110	143	110	70	131	71	70	147	135	141	140	155	138	124
129	129	138	142	120	118	126	121	126	120	134	104	132	101	132	88	133	127	100	101	100	100	100	100	100	100	100	100	100	100	100	100	100	100	129	107	117	116	128	123	125	101	114	101	101	139	133	137	141	154	131	121
128	128	133	140	116	114	129	134	119	129	126	120	127	122	132	107	124	128	93	94	93	93	93	93	93	93	93	93	93	93	93	93	93	93	121	101	128	127	120	126	124	103	128	103	103	146	135	136	146	153	138	120
122	122	134	138	121	119	128	125	121	121	136	115	124	111	133	98	124	130	100	100	100	100	100	100	100	100	100	100	100	100	100	100	100	100	123	97	114	114	127	133	124	118	116	119	118	139	131	139	142	149	135	119
119	119	135	133	119	117	128	129	124	130	124	119	133	118	135	107	121	120	108	108	108	108	108	108	108	108	108	108	108	108	108	108	108	108	126	103	131	129	117	132	115	82	128	83	82	149	138	144	149	159	140	118
123	123	134	138	124	123	126	129	118	118	129	112	134	99	135	76	123	130	86	87	86	86	86	86	86	86	86	86	86	86	86	86	86	86	127	102	129	130	126	126	126	104	113	104	104	143	133	135	142	151	130	111
120	120	144	134	99	99	131	122	127	123	115	103	132	105	132	106	112	99	114	114	114	114	114	114	114	114	114	114	114	114	114	114	114	114	129	115	131	131	117	136	105	80	129	81	80	151	137	141	145	158	137	124
111	111	138	137	104	103	133	115	120	121	126	122	123	127	133	105	112	112	115	116	115	115	115	115	115	115	115	115	115	115	115	115	115	115	119	108	128	128	113	140	107	91	126	92	91	136	126	130	140	152	130	126
118	118	140	134	107	106	129	126	128	123	119	104	125	113	135	102	108	111	117	117	117	117	117	117	117	117	117	117	117	117	117	117	117	117	121	119	123	124	109	140	106	85	127	85	85	142	127	132	147	153	135	124
120	119	140	138	110	109	125	127	122	123	124	117	121	124	133	108	131	117	115	115	115	115	115	115	115	115	115	115	115	115	115	115	115	115	122	111	126	124	116	135	123	97	120	98	97	144	134	138	133	155	130	127
120	120	132	132	128	128	136	122	123	133	134	129	133	119	136	114	122	127	119	119	119	119	119	119	119	119	119	119	119	119	119	119	119	119	130	104	116	117	115	133	118	104	123	104	104	138	134	146	145	152	146	129
133	133	134	140	116	114	128	133	121	133	130	126	134	117	140	105	127	123	92	93	92	92	92	92	92	92	92	92	92	92	92	92	92	92	127	109	125	126	121	129	120	105	124	105	105	147	139	137	147	152	134	128
128	128	144	141	113	113	129	137	127	116	132	122	140	124	133	110	133	128	98	98	98	98	98	98	98	98	98	98	98	98	98	98	98	98	123	95	126	124	129	131	131	105	128	106	105	144	137	142	146	154	145	127
116	116	137	133	114	114	124	131	119	125	130	117	121	130	124	106	119	122	115	115	115	115	115	115	115	115	115	115	115	115	115	115	115	115	122	112	121	121	115	140	116	110	124	110	110	142	130	140	139	150	134	134
121	120	140	138	111	110	130	118	116	121	134	113	119	115	137	90	129	122	98	98	98	98	98	98	98	98	98	98	98	98	98	98	98	98	115	102	124	125	123	130	120	104	119	104	105	144	134	139	135	154	136	122
129	129	131	138	110	109	127	131	118	126	141	116	135	119	126	108	133	123	102	103	102	102	102	102	102	102	102	102	102	102	102	102	102	102	131	105	121	122	123	129	127	111	121	111	111	145	131	136	151	153	131	125
120	120	142	134	126	125	134	126	119	130	125	123	126	117	134	115	125	115	108	108	108	108	108	108	108	108	108	108	108	108	108	108	108	108	126	108	122	121	126	137	116	98	121	98	99	138	136	138	144	159	138	119
122	122	141	141	116	114	132	130	125	124	126	114	135	99	140	97	129	118	96	96	96	96	96	96	96	96	96	96	96	96	96	96	96	96	131	94	126	124	124	134	127	78	121	79	78	147	136	141	145	158	143	121
124	124	135	137	121	119	129	133	122	129	127	120	132	115	139	107	122	122	113	113	113	113	113	113	113	113	113	113	113	113	113	113	113	113	126	108	127	126	123	138	126	99	122	99	99	149	135	136	145	155	135	113
115	115	142	135	118	116	121	129	121	122	121	105	128	114	124	107	123	122	119	119	119	119	119	119	119	119	119	119	119	119	119	119	119	119	119	112	119	117	116	134	115	81	130	82	81	141	128	134	146	153	132	128
123	123	141	131	99	99	135	129	126	136	97	119	123	128	136	121	99	102	127	127	127	127	127	127	127	127	127	127	127	127	127	127	127	127	127	132	130	130	117	137	111	107	135	108	107	141	129	138	128	149	145	134
104	104	133	129	82	81	125	125	127	123	113	109	119	119	123	100	104	98	116	117	116	116	116	116	116	116	116	116	116	116	116	116	116	116	128	119	125	125	106	143	93	84	126	85	84	138	126	140	138	149	137	131
119	119	142	137	117	116	127	133	125	124	123	121	119	115	134	116	119	113	98	98	98	98	98	98	98	98	98	98	98	98	98	98	98	98	121	117	124	124	118	132	112	88	132	89	88	147	134	135	139	151	136	123
105	105	139	134	104	103	123	121	123	115	125	112	123	115	133	100	116	113	107	107	107	107	107	107	107	107	107	107	107	107	107	107	107	107	121	122	125	126	124	141	117	70	130	70	70	148	138	142	147	154	138	111
128	128	137	138	129	128	131	117	115	122	139	126	135	111	136	102	132	129	87	88	87	87	87	87	87	87	87	87	87	87	87	87	87	87	120	98	122	121	126	129	129	100	119	100	100	141	134	134	145	151	138	121
95	94	135	131	126	126	129	127	127	119	133	116	122	111	119	116	122	119	133	133	133	133	133	133	133	133	133	133	133	133	133	133	133	133	127	124	109	110	124	131	124	102	121	102	102	137	130	136	125	141	143	124
80	79	140	137	115	115	138	127	138	108	135	109	125	111	125	107	119	111	124	124	124	124	124	124	124	124	124	124	124	124	124	124	124	124	128	119	121	121	110	135	109	104	112	105	104	143	133	139	142	151	137	128
91	91	130	129	107	107	128	118	120	114	121	117	123	114	117	108	116	103	123	123	123	123	123	123	123	123	123	123	123	123	123	123	123	123	123	122	121	121	120	131	110	90	124	91	90	127	123	136	132	143	139	121
110	110	142	138	104	103	135	127	139	118	123	113	127	111	129	104	117	108	113	113	113	113	113	113	113	113	113	113	113	113	113	113	113	113	129	116	128	129	119	135	115	95	128	95	95	144	136	136	135	151	139	125
121	121	134	135	109	109	135	131	119	115	137	114	128	124	132	104	121	118	82	82	82	82	82	82	82	82	82	82	82	82	82	82	82	82	125	102	113	112	121	127	121	108	114	108	108	137	128	132	142	148	140	132
110	110	137	138	108	107	130	129	129	116	125	103	123	111	133	100	114	96	108	108	108	108	108	108	108	108	108	108	108	108	108	108	108	108	123	119	119	120	117	126	107	87	123	87	87	149	131	138	149	158	135	119
115	115	142	133	103	102	129	125	130	120	108	98	127	106	136	99	106	96	113	113	113	113	113	113	113	113	113	113	113	113	113	113	113	113	125	130	138	139	118	136	117	78	129	78	78	146	132	132	133	153	129	116
117	117	142	131	69	68	138	119	133	120	117	116	118	111	130	111	110	82	120	120	120	120	120	120	120	120	120	120	120	120	120	120	120	120	131	122	128	128	110	136	99	92	128	93	92	140	117	131	139	155	133	132
118	118	139	134	112	111	135	124	126	120	110	106	112	100	128	106	102	95	115	115	115	115	115	115	115	115	115	115	115	115	115	115	115	115	125	124	123	124	113	129	112	85	128	85	85	137	124	128	131	152	133	132
123	123	140	134	112	111	134	128	136	129	123	124	129	115	133	110	124	115	106	106	106	106	106	106	106	106	106	106	106	106	106	106	106	106	136	107	121	120	124	137	106	112	115	112	112	137	142	141	145	154	142	134
132	131	130	135	117	117	136	130	137	138	93	117	101	135	134	122	106	111	145	145	145	145	145	145	145	145	145	145	145	145	145	145	145	145	140	141	141	141	120	142	115	125	132	125	125	138	132	146	96	149	136	137
102	102	142	130	104	104	130	130	130	133	111	110	120	114	129	107	98	104	128	128	128	128	128	128	128	128	128	128	128	128	128	128	128	128	128	129	132	133	108	140	107	87	127	87	87	139	127	135	134	153	137	125
115	115	135	133	109	107	130	121	118	126	128	110	125	112	125	99	130	114	110	110	110	110	110	110	110	110	110	110	110	110	110	110	110	110	127	101	127	128	121	134	120	86	121	86	87	140	127	134	140	154	129	123
108	108	139	129	112	111	119	122	118	123	129	109	128	113	128	111	118	106	108	108	108	108	108	108	108	108	108	108	108	108	108	108	108	108	127	117	117	117	114	130	117	87	120	88	87	147	139	141	148	158	136	118
123	123	137	142	108	107	134	133	118	117	129	108	130	124	133	106	127	123	90	90	90	90	90	90	90	90	90	90	90	90	90	90	90	90	117	93	114	115	121	130	125	108	123	108	108	145	132	137	141	156	140	126
116	116	141	139	115	115	127	133	116	111	131	107	126	118	126	95	126	128	89	89	89	89	89	89	89	89	89	89	89	89	89	89	89	89	118	103	117	118	124	131	129	97	121	97	98	142	135	132	147	155	133	127
124	124	139	132	121	121	125	125	112	128	121	126	135	117	135	107	123	115	95	95	95	95	95	95	95	95	95	95	95	95	95	95	95	95	122	106	128	126	122	130	126	89	123	90	90	147	138	138	146	155	137	114
118	118	145	138	110	109	128	133	121	127	112	111	126	116	135	108	112	110	114	114	114	114	114	114	114	114	114	114	114	114	114	114	114	114	122	108	129	129	112	135	122	89	129	90	90	146	130	129	137	154	134	132
122	122	142	142	120	119	118	133	116	126	134	127	133	119	136	103	129	122	99	99	99	99	99	99	99	99	99	99	99	99	99	99	99	99	119	106	117	118	121	128	113	106	122	106	106	141	134	130	146	150	136	129
130	130	139	137	127	126	129	120	129	125	132	113	128	105	135	87	123	130	110	110	110	110	110	110	110	110	110	110	110	110	110	110	110	110	125	101	129	129	125	129	124	104	117	104	104	141	136	136	137	153	137	115
112	112	138	131	116	115	124	126	126	126	123	112	132	118	131	109	124	115	125	125	125	125	125	125	125	125	125	125	125	125	125	125	125	125	126	117	124	125	123	137	101	88	125	88	88	140	132	137	144	156	136	119
125	124	142	134	98	97	138	127	138	144	105	114	96	132	130	127	100	91	138	138	138	138	138	138	138	138	138	138	138	138	138	138	138	138	142	143	138	139	117	144	111	118	134	118	118	140	134	142	112	154	126	137

127	127	135	135	121	120	129	127	125	126	132	116	121	109	136	103	120	129	91	91	91	91	91	91	91	91	91	91	91	91	91	91	91	91	125	103	119	118	120	132	121	113	112	113	113	141	136	136	147	151	138	122
123	123	134	139	124	124	138	125	126	123	132	116	130	120	137	102	130	122	104	104	104	104	104	104	104	104	104	104	104	104	104	104	104	104	125	93	119	118	124	132	123	116	114	116	116	135	126	135	143	149	136	128
127	127	147	128	114	114	134	131	133	148	106	124	106	136	130	125	110	105	141	141	141	141	141	141	141	141	141	141	141	141	141	141	141	141	139	147	145	146	125	144	120	130	145	130	130	146	138	144	109	148	140	142
125	124	139	131	107	107	141	131	143	142	112	119	109	128	136	122	103	99	139	139	139	139	139	139	139	139	139	139	139	139	139	139	139	139	146	148	137	138	119	144	115	117	132	117	117	143	139	152	109	146	141	142
111	111	136	133	122	121	126	117	123	121	122	105	131	100	139	81	113	115	95	95	95	95	95	95	95	95	95	95	95	95	95	95	95	95	120	100	122	120	117	135	116	81	113	82	81	144	137	140	147	154	133	110
115	115	145	137	116	116	121	131	125	126	125	115	128	114	130	117	128	113	115	115	115	115	115	115	115	115	115	115	115	115	115	115	115	115	126	121	121	121	117	136	118	94	125	95	94	142	135	135	146	158	137	126
121	121	140	135	125	124	130	135	124	131	126	118	130	123	130	117	126	121	115	115	115	115	115	115	115	115	115	115	115	115	115	115	115	115	119	114	124	125	120	133	132	114	132	114	114	142	128	132	134	147	132	128
94	94	143	129	114	113	130	120	122	101	128	93	125	97	124	99	118	118	112	112	112	112	112	112	112	112	112	112	112	112	112	112	112	112	122	121	118	119	116	140	119	91	123	91	92	146	136	146	145	157	140	122
124	124	139	140	107	106	127	136	123	120	132	118	132	124	127	118	138	111	108	109	108	108	108	108	108	108	108	108	108	108	108	108	108	108	122	99	121	119	128	138	128	95	131	96	95	136	130	136	141	154	140	133
107	106	137	131	100	99	130	127	131	123	100	111	101	130	128	116	103	93	129	129	129	129	129	129	129	129	129	129	129	129	129	129	129	129	131	130	127	128	107	135	106	103	133	103	104	142	130	146	128	151	132	125
117	117	140	137	110	108	118	129	129	118	124	112	134	108	135	98	124	113	104	105	104	104	104	104	104	104	104	104	104	104	104	104	104	104	123	102	125	123	119	134	121	78	124	79	78	146	136	135	153	154	137	119
122	122	138	133	114	113	133	126	136	124	130	119	141	108	136	97	122	123	107	107	107	107	107	107	107	107	107	107	107	107	107	107	107	107	137	107	125	126	124	136	121	97	117	97	97	147	139	146	148	156	141	121
139	139	131	139	121	121	127	135	123	132	138	118	132	119	134	116	139	136	97	97	97	97	97	97	97	97	97	97	97	97	97	97	97	97	123	96	119	118	129	132	132	109	125	109	109	142	138	138	145	150	139	127

1	6	2	1	4	4	4	4	1	1	2	1	1	4	4	4	4	1	1	2	1	21	21	21	21	21	21	21	21	21	21	21	21	21	21	21	21	21	21	21	21	21	2	1	1	1	1	6	6	6	6	6
Murcott	Myrttle leaf orange	Nansho daidai	Naruto	Natsudaidai	Natsudaidai	Natsudaidai	Natsudaidai	Nidonari mikan	Oogonkan	Ootachibana	Ootoukan	Oukan	Ponkan	Ponkan	Ponkan	Ponkan	Ponkitsu	Pummelo whitetype	Rokugatsumikan	Sanbokan	Satsuma mandarin	Satsuma mandarin	Satsuma mandarin	Satsuma mandarin	Satsuma mandarin	Satsuma mandarin	Satsuma mandarin	Satsuma mandarin	Satsuma mandarin	Satsuma mandarin	Satsuma mandarin	Satsuma mandarin	Satsuma mandarin	Satsuma mandarin	Satsuma mandarin	Satsuma mandarin	Satsuma mandarin	Satsuma mandarin	Satsuma mandarin	Satsuma mandarin	Satsuma mandarin	Satsuma Kikoku	Shiikuwasha	Shiikuwasha_Ogimikuga	Shunkokan	Sokitsu	Sour orange	Sour orange	Sour orange	Sour orange	Sour orange
A092	A093	A094	A095	A096	A097	A098	A099	A100	A101	A102	A103	A104	A105	A106	A107	A108	A109	A110	A111	A112	A113	A114	A115	A116	A117	A118	A119	A120	A121	A122	A123	A124	A125	A126	A127	A128	A129	A130	A131	A132	A133	A134	A135	A136	A137	A138	A139	A140	A141	A142	A143
0	0	0	0	0	0	0	0	0	0	0	0	0	0	0	0	0	0	1	0	0	0	0	0	0	0	0	0	0	0	0	0	0	0	0	0	0	0	0	0	0	0	0	0	0	0	0	0	0	0	2	0
126	100	127	59	71	71	71	71	114	116	119	105	83	136	136	136	136	122	135	131	63	91	92	91	91	91	91	91	91	91	91	91	91	91	91	91	91	91	91	91	91	91	98	138	140	76	136	99	99	99	99	99
141	124	139	121	114	114	114	114	141	134	89	121	123	149	149	149	149	143	90	132	123	140	140	140	140	140	140	140	140	140	140	140	140	140	140	140	140	140	140	140	140	140	130	146	144	132	144	124	124	124	124	124
124	101	132	97	81	81	81	81	125	108	93	102	94	141	141	141	141	133	129	138	88	115	116	115	115	115	115	115	115	115	115	115	115	115	115	115	115	115	115	115	115	115	106	142	140	107	143	101	101	101	101	101
136	128	146	129	130	130	130	130	139	146	97	134	147	148	148	148	148	141	62	140	132	143	144	143	143	143	143	143	143	143	143	143	143	143	143	143	143	143	143	143	143	143	134	147	145	133	146	128	128	128	128	128
94	133	119	139	129	129	129	129	97	117	139	108	118	98	98	98	98	107	147	119	127	120	121	120	120	120	120	120	120	120	120	120	120	120	120	120	120	120	120	120	120	120	129	106	111	118	67	133	133	133	133	133
132	89	133	131	131	131	131	131	131	122	132	134	132	133	133	133	133	133	129	123	125	141	141	141	141	141	141	141	141	141	141	141	141	141	141	141	141	141	141	141	141	141	117	127	128	129	140	89	89	89	91	89
104	114	118	90	87	87	87	87	112	106	134	111	106	122	122	122	122	109	143	128	90	68	69	68	68	68	68	68	68	68	68	68	68	68	68	68	68	68	68	68	68	68	107	129	133	85	116	114	114	114	114	114
106	122	117	107	111	111	111	111	130	119	136	117	119	118	118	118	118	122	140	123	108	118	118	118	118	118	118	118	118	118	118	118	118	118	118	118	118	118	118	118	118	118	118	126	125	114	117	122	122	122	122	122
101	122	125	130	128	128	128	128	121	123	143	121	118	86	86	86	86	104	146	124	129	133	133	133	133	133	133	133	133	133	133	133	133	133	133	133	133	133	133	133	133	133	123	112	121	126	101	122	122	122	123	122
101	122	125	130	128	128	128	128	121	123	143	121	118	86	86	86	86	104	146	124	129	133	133	133	133	133	133	133	133	133	133	133	133	133	133	133	133	133	133	133	133	133	123	112	121	126	101	122	122	122	123	122
101	122	125	130	128	128	128	128	121	123	143	121	118	86	86	86	86	104	146	124	129	133	133	133	133	133	133	133	133	133	133	133	133	133	133	133	133	133	133	133	133	133	123	112	121	126	101	122	122	122	123	122
101	122	125	130	128	128	128	128	121	123	143	121	118	86	86	86	86	104	146	124	129	133	133	133	133	133	133	133	133	133	133	133	133	133	133	133	133	133	133	133	133	133	123	112	121	126	101	122	122	122	123	122
101	134	118	141	138	138	138	138	94	114	139	130	126	86	86	86	86	94	143	118	132	120	121	120	120	120	120	120	120	120	120	120	120	120	120	120	120	120	120	120	120	120	131	91	93	121	87	135	135	135	135	135
95	125	125	129	119	119	119	119	103	120	146	122	126	89	89	89	89	104	145	121	127	108	108	108	108	108	108	108	108	108	108	108	108	108	108	108	108	108	108	108	108	108	122	111	115	120	96	125	125	125	125	125
123	98	127	102	102	102	102	102	122	104	119	118	109	143	143	143	143	131	138	113	98	113	113	113	113	113	113	113	113	113	113	113	113	113	113	113	113	113	113	113	113	113	97	133	136	107	137	98	98	98	98	98
79	139	128	135	129	129	129	129	103	118	147	126	133	66	66	66	66	84	148	117	139	120	120	120	120	120	120	120	120	120	120	120	120	120	120	120	120	120	120	120	120	120	138	104	108	123	89	139	139	139	139	139
79	139	128	135	129	129	129	129	103	118	147	126	133	66	66	66	66	84	148	117	139	120	120	120	120	120	120	120	120	120	120	120	120	120	120	120	120	120	120	120	120	120	138	104	108	123	89	139	139	139	139	139
142	128	146	134	135	135	135	135	141	150	96	135	145	154	154	154	154	144	69	144	138	144	145	144	144	144	144	144	144	144	144	144	144	144	144	144	144	144	144	144	144	144	134	145	148	141	149	128	128	128	128	128
112	132	117	130	124	124	124	124	103	122	139	135	118	108	108	108	108	112	144	115	127	114	114	114	114	114	114	114	114	114	114	114	114	114	114	114	114	114	114	114	114	114	131	87	95	111	100	132	132	132	132	132
119	112	117	131	135	135	135	135	118	124	138	136	129	126	126	126	126	122	141	0	119	123	123	123	123	123	123	123	123	123	123	123	123	123	123	123	123	123	123	123	123	123	124	92	89	119	120	112	112	112	113	112
128	105	126	104	93	93	93	93	113	109	116	96	112	126	126	126	126	111	133	133	95	99	100	99	99	99	99	99	99	99	99	99	99	99	99	99	99	99	99	99	99	99	107	133	128	117	115	105	105	105	105	105
98	138	122	139	139	139	139	139	102	124	144	130	134	83	83	83	83	82	145	116	136	119	119	119	119	119	119	119	119	119	119	119	119	119	119	119	119	119	119	119	119	119	132	98	105	123	88	138	138	138	138	138
109	132	120	129	125	125	125	125	132	115	138	135	121	130	130	130	130	118	143	96	120	123	123	123	123	123	123	123	123	123	123	123	123	123	123	123	123	123	123	123	123	123	133	98	100	121	116	132	132	132	132	132
121	110	132	93	90	90	90	90	132	125	103	98	113	135	135	135	135	130	116	139	99	113	114	113	113	113	113	113	113	113	113	113	113	113	113	113	113	113	113	113	113	113	103	143	148	101	139	110	110	110	110	110
121	110	132	93	90	90	90	90	132	125	103	98	113	135	135	135	135	130	116	139	99	113	114	113	113	113	113	113	113	113	113	113	113	113	113	113	113	113	113	113	113	113	103	143	148	101	139	110	110	110	110	110
121	110	132	93	90	90	90	90	132	125	103	98	113	135	135	135	135	130	116	139	99	113	114	113	113	113	113	113	113	113	113	113	113	113	113	113	113	113	113	113	113	113	103	143	148	101	139	110	110	110	110	110
130	137	113	147	148	148	148	148	134	137	148	141	135	132	132	132	132	129	153	109	140	139	139	139	139	139	139	139	139	139	139	139	139	139	139	139	139	139	139	139	139	139	142	105	107	137	132	137	137	137	137	137
131	104	128	85	87	87	87	87	131	119	111	88	105	137	137	137	137	132	127	136	92	120	121	120	120	120	120	120	120	120	120	120	120	120	120	120	120	120	120	120	120	120	104	143	140	98	142	104	104	104	104	104
129	129	93	135	119	119	119	119	129	119	133	125	133	119	119	119	119	131	146	132	126	130	130	130	130	130	130	130	130	130	130	130	130	130	130	130	130	130	130	130	130	130	123	130	128	126	127	129	129	129	129	129
120	125	0	127	126	126	126	126	125	128	139	136	123	128	128	128	128	122	139	117	120	112	112	112	112	112	112	112	112	112	112	112	112	112	112	112	112	112	112	112	112	112	119	119	119	122	120	125	125	125	125	125
92	136	120	138	133	133	133	133	102	115	150	143	136	67	67	67	67	84	147	118	134	116	117	116	116	116	116	116	116	116	116	116	116	116	116	116	116	116	116	116	116	116	126	103	107	120	100	136	136	136	136	136
144	135	143	129	134	134	134	134	141	142	94	137	139	147	147	147	147	142	60	141	136	141	142	141	141	141	141	141	141	141	141	141	141	141	141	141	141	141	141	141	141	141	133	147	148	139	144	135	135	135	135	135
129	80	136	104	97	97	97	97	133	110	106	106	125	133	133	133	133	126	115	130	107	128	129	128	128	128	128	128	128	128	128	128	128	128	128	128	128	128	128	128	128	128	112	140	141	113	132	80	80	80	81	80
116	103	117	82	82	82	82	82	107	108	123	107	108	120	120	120	120	113	137	123	77	91	92	91	91	91	91	91	91	91	91	91	91	91	91	91	91	91	91	91	91	91	97	128	129	80	121	103	103	103	103	103
134	111	118	100	91	91	91	91	128	115	101	99	99	144	144	144	144	135	122	128	88	115	116	115	115	115	115	115	115	115	115	115	115	115	115	115	115	115	115	115	115	115	105	139	137	105	139	111	111	111	111	111
114	116	122	110	109	109	109	109	129	103	128	116	118	127	127	127	127	122	137	115	101	104	104	104	104	104	104	104	104	104	104	104	104	104	104	104	104	104	104	104	104	104	113	126	133	112	125	116	116	116	116	116
114	116	122	110	109	109	109	109	129	103	128	116	118	127	127	127	127	122	137	115	101	104	104	104	104	104	104	104	104	104	104	104	104	104	104	104	104	104	104	104	104	104	113	126	133	112	125	116	116	116	116	116
114	116	122	110	109	109	109	109	129	103	128	116	118	127	127	127	127	122	137	115	101	104	104	104	104	104	104	104	104	104	104	104	104	104	104	104	104	104	104	104	104	104	113	126	133	112	125	116	116	116	116	116
114	116	122	110	109	109	109	109	129	103	129	116	118	127	127	127	127	122	138	116	101	104	104	104	104	104	104	104	104	104	104	104	104	104	104	104	104	104	104	104	104	104	113	126	133	112	125	116	116	116	116	116
114	116	122	110	109	109	109	109	129	103	128	116	118	127	127	127	127	122	137	115	101	104	104	104	104	104	104	104	104	104	104	104	104	104	104	104	104	104	104	104	104	104	113	126	133	112	125	116	116	116	116	116
135	127	129	143	141	141	141	141	133	138	135	142	142	140	140	140	140	132	138	124	138	140	140	140	140	140	140	140	140	140	140	140	140	140	140	140	140	140	140	140	140	140	142	130	133	137	133	127	127	127	127	127
133	114	134	115	121	121	121	121	140	132	127	124	133	142	142	142	142	142	130	126	130	135	135	135	135	135	135	135	135	135	135	135	135	135	135	135	135	135	135	135	135	135	129	140	142	127	147	114	114	114	115	114
125	106	133	84	92	92	92	92	107	114	125	91	94	121	121	121	121	121	138	136	87	103	104	103	103	103	103	103	103	103	103	103	103	103	103	103	103	103	103	103	103	103	107	137	136	96	128	106	106	106	106	106
125	106	133	83	92	92	92	92	107	113	125	90	93	121	121	121	121	121	138	136	86	103	104	103	103	103	103	103	103	103	103	103	103	103	103	103	103	103	103	103	103	103	107	137	136	96	128	106	106	106	106	106
136	132	97	130	128	128	128	128	123	128	142	130	135	130	130	130	130	133	142	130	134	120	120	120	120	120	120	120	120	120	120	120	120	120	120	120	120	120	120	120	120	120	135	133	133	128	131	132	132	132	132	132
130	114	105	113	116	116	116	116	138	118	122	120	113	129	129	129	129	124	131	127	113	122	122	122	122	122	122	122	122	122	122	122	122	122	122	122	122	122	122	122	122	122	120	129	131	125	130	114	114	114	114	114
128	130	107	132	118	118	118	118	121	118	128	122	125	124	124	124	124	132	143	124	124	132	132	132	132	132	132	132	132	132	132	132	132	132	132	132	132	132	132	132	132	132	125	132	128	121	127	130	130	130	131	130
123	136	130	128	127	127	127	127	119	107	146	123	127	113	113	113	113	128	146	119	122	121	121	121	121	121	121	121	121	121	121	121	121	121	121	121	121	121	121	121	121	121	124	95	104	120	120	136	136	136	136	136
137	117	137	107	99	99	99	99	138	127	86	104	114	143	143	143	143	136	109	138	96	135	136	135	135	135	135	135	135	135	135	135	135	135	135	135	135	135	135	135	135	135	120	149	145	112	144	117	117	117	117	117
111	125	126	100	100	100	100	100	121	121	129	107	113	119	119	119	119	127	132	127	100	113	113	113	113	113	113	113	113	113	113	113	113	113	113	113	113	113	113	113	113	113	117	116	115	107	129	125	125	125	125	125
126	107	133	84	87	87	87	87	126	127	90	110	118	138	138	138	138	137	109	130	92	125	126	125	125	125	125	125	125	125	125	125	125	125	125	125	125	125	125	125	125	125	120	141	140	102	137	107	107	107	107	107
113	134	116	121	125	125	125	125	131	121	140	130	124	118	118	118	118	119	146	112	120	113	113	113	113	113	113	113	113	113	113	113	113	113	113	113	113	113	113	113	113	113	123	108	102	113	109	134	134	134	134	134
136	122	131	115	123	123	123	123	126	129	137	118	127	134	134	134	134	144	136	122	120	136	136	136	136	136	136	136	136	136	136	136	136	136	136	136	136	136	136	136	136	136	115	130	134	121	140	122	122	122	122	122
80	120	113	115	117	117	117	117	117	114	137	112	124	101	101	101	101	98	138	116	116	103	103	103	103	103	103	103	103	103	103	103	103	103	103	103	103	103	103	103	103	103	119	116	119	122	106	119	119	119	119	119
132	106	132	98	93	93	93	93	125	119	90	99	109	138	138	138	138	136	120	134	98	122	123	122	122	122	122	122	122	122	122	122	122	122	122	122	122	122	122	122	122	122	103	142	136	97	131	106	106	106	106	106
126	101	127	61	73	73	73	73	115	118	119	107	84	136	136	136	136	122	135	131	65	93	94	93	93	93	93	93	93	93	93	93	93	93	93	93	93	93	93	93	93	93	100	138	140	78	136	100	100	100	100	100

101	131	117	136	129	129	129	129	85	110	146	126	113	90	90	90	90	98	150	125	127	99	100	99	99	99	99	99	99	99	99	99	99	99	99	99	99	99	99	99	99	99	130	101	96	113	67	131	131	131	131	131
101	131	117	136	129	129	129	129	85	110	146	126	114	90	90	90	90	99	150	125	127	100	101	100	100	100	100	100	100	100	100	100	100	100	100	100	100	100	100	100	100	100	130	102	97	113	67	131	131	131	131	131
101	131	117	136	129	129	129	129	85	110	146	126	113	90	90	90	90	98	150	125	127	99	100	99	99	99	99	99	99	99	99	99	99	99	99	99	99	99	99	99	99	99	130	101	96	113	67	131	131	131	131	131
101	131	117	136	129	129	129	129	85	110	146	126	113	90	90	90	90	98	150	125	127	99	100	99	99	99	99	99	99	99	99	99	99	99	99	99	99	99	99	99	99	99	130	101	96	113	67	131	131	131	131	131
101	131	117	136	129	129	129	129	85	110	146	126	113	90	90	90	90	98	150	125	127	99	100	99	99	99	99	99	99	99	99	99	99	99	99	99	99	99	99	99	99	99	130	101	96	113	67	131	131	131	131	131
101	131	117	136	129	129	129	129	85	110	146	126	113	90	90	90	90	98	150	125	127	99	100	99	99	99	99	99	99	99	99	99	99	99	99	99	99	99	99	99	99	99	130	101	96	113	67	131	131	131	131	131
101	131	117	136	129	129	129	129	85	110	146	126	113	90	90	90	90	98	150	125	127	99	100	99	99	99	99	99	99	99	99	99	99	99	99	99	99	99	99	99	99	99	130	101	96	113	67	131	131	131	131	131
101	131	117	136	129	129	129	129	85	110	146	126	113	90	90	90	90	98	150	125	127	99	100	99	99	99	99	99	99	99	99	99	99	99	99	99	99	99	99	99	99	99	130	101	96	113	67	131	131	131	131	131
101	131	117	136	129	129	129	129	85	110	146	126	113	90	90	90	90	98	150	125	127	99	100	99	99	99	99	99	99	99	99	99	99	99	99	99	99	99	99	99	99	99	130	101	96	113	67	131	131	131	131	131
101	131	117	136	129	129	129	129	85	110	146	126	113	90	90	90	90	98	150	125	127	99	100	99	99	99	99	99	99	99	99	99	99	99	99	99	99	99	99	99	99	99	130	101	96	113	67	131	131	131	131	131
101	131	117	136	129	129	129	129	85	110	146	126	113	90	90	90	90	98	150	125	127	99	100	99	99	99	99	99	99	99	99	99	99	99	99	99	99	99	99	99	99	99	130	101	96	113	67	131	131	131	131	131
101	131	117	136	129	129	129	129	85	110	146	126	113	90	90	90	90	98	150	125	127	99	100	99	99	99	99	99	99	99	99	99	99	99	99	99	99	99	99	99	99	99	130	101	96	113	67	131	131	131	131	131
101	131	117	136	129	129	129	129	85	110	146	126	113	90	90	90	90	98	150	125	127	99	100	99	99	99	99	99	99	99	99	99	99	99	99	99	99	99	99	99	99	99	130	101	96	113	67	131	131	131	131	131
101	131	117	136	129	129	129	129	85	110	146	126	113	90	90	90	90	98	150	125	127	99	100	99	99	99	99	99	99	99	99	99	99	99	99	99	99	99	99	99	99	99	130	101	96	113	67	131	131	131	131	131
101	131	117	136	129	129	129	129	85	110	146	126	113	90	90	90	90	98	150	125	127	99	100	99	99	99	99	99	99	99	99	99	99	99	99	99	99	99	99	99	99	99	130	101	96	113	67	131	131	131	131	131
101	131	117	136	129	129	129	129	85	110	146	126	113	90	90	90	90	98	150	125	127	99	100	99	99	99	99	99	99	99	99	99	99	99	99	99	99	99	99	99	99	99	130	101	96	113	67	131	131	131	131	131
129	129	93	135	119	119	119	119	129	119	133	125	133	119	119	119	119	131	146	132	126	130	130	130	130	130	130	130	130	130	130	130	130	130	130	130	130	130	130	130	130	130	123	130	128	126	127	129	129	129	129	129
83	133	122	140	131	131	131	131	102	116	145	135	120	87	87	87	87	92	149	123	134	116	117	116	116	116	116	116	116	116	116	116	116	116	116	116	116	116	116	116	116	116	130	104	111	129	59	134	134	134	134	134
115	126	131	134	130	130	130	130	125	114	138	125	130	117	117	117	117	115	144	112	127	130	130	130	130	130	130	130	130	130	130	130	130	130	130	130	130	130	130	130	130	130	127	94	101	116	115	126	126	126	126	126
115	126	132	135	131	131	131	131	125	114	138	126	130	116	116	116	116	113	144	112	128	130	130	130	130	130	130	130	130	130	130	130	130	130	130	130	130	130	130	130	130	130	127	95	102	117	113	126	126	126	126	126
124	96	119	101	98	98	98	98	118	108	130	113	106	133	133	133	133	123	138	124	98	118	119	118	118	118	118	118	118	118	118	118	118	118	118	118	118	118	118	118	118	118	0	127	130	105	130	95	95	95	95	95
126	134	110	139	138	138	138	138	129	126	139	139	127	126	126	126	126	126	151	115	132	129	129	129	129	129	129	129	129	129	129	129	129	129	129	129	129	129	129	129	129	129	127	108	107	125	126	134	134	134	134	134
126	93	125	87	82	82	82	82	115	108	127	107	102	127	127	127	127	126	139	123	91	107	107	107	107	107	107	107	107	107	107	107	107	107	107	107	107	107	107	107	107	107	92	145	142	100	130	93	93	93	93	93
104	114	118	90	87	87	87	87	112	106	134	111	106	122	122	122	122	109	143	128	90	68	69	68	68	68	68	68	68	68	68	68	68	68	68	68	68	68	68	68	68	68	107	129	133	85	116	114	114	114	114	114
112	110	121	131	133	133	133	133	115	122	139	138	123	120	120	120	120	114	143	76	126	125	125	125	125	125	125	125	125	125	125	125	125	125	125	125	125	125	125	125	125	125	119	80	85	126	116	110	110	110	110	110
105	114	118	90	87	87	87	87	112	107	134	111	107	122	122	122	122	110	143	128	90	69	70	69	69	69	69	69	69	69	69	69	69	69	69	69	69	69	69	69	69	69	108	129	133	85	117	114	114	114	114	114
104	114	118	90	88	88	88	88	112	107	134	112	106	122	122	122	122	109	143	128	90	68	69	68	68	68	68	68	68	68	68	68	68	68	68	68	68	68	68	68	68	68	107	129	133	85	116	114	114	114	114	114
136	128	147	141	139	139	139	139	139	134	136	138	134	135	135	135	135	138	145	128	133	145	145	145	145	145	145	145	145	145	145	145	145	145	145	145	145	145	145	145	145	145	139	129	131	135	148	128	128	128	128	128
126	119	134	128	123	123	123	123	133	132	129	132	130	133	133	133	133	129	138	135	115	133	134	133	133	133	133	133	133	133	133	133	133	133	133	133	133	133	133	133	133	133	115	125	130	129	142	119	119	119	119	119
138	134	133	138	134	134	134	134	137	133	146	143	137	132	132	132	132	125	150	135	133	133	133	133	133	133	133	133	133	133	133	133	133	133	133	133	133	133	133	133	133	133	128	132	127	134	134	134	134	134	134	134
140	134	146	132	135	135	135	135	139	146	84	132	144	152	152	152	152	145	64	137	131	146	146	146	146	146	146	146	146	146	146	146	146	146	146	146	146	146	146	146	146	146	142	146	145	135	147	134	134	134	134	134
148	148	144	155	154	154	154	154	149	152	144	153	156	150	150	150	150	143	142	144	152	153	153	153	153	153	153	153	153	153	153	153	153	153	153	153	153	153	153	153	153	153	153	138	140	155	149	148	148	148	148	148
133	136	127	136	132	132	132	132	141	142	145	139	131	134	134	134	134	134	146	131	130	129	129	129	129	129	129	129	129	129	129	129	129	129	129	129	129	129	129	129	129	129	129	137	135	132	140	136	136	136	136	136
125	130	83	129	130	130	130	130	130	122	142	127	122	127	127	127	127	131	149	124	130	120	120	120	120	120	120	120	120	120	120	120	120	120	120	120	120	120	120	120	120	120	133	121	118	126	126	130	130	130	131	130
0	127	120	119	114	114	114	114	110	113	143	127	127	100	100	100	100	90	139	119	127	104	105	104	104	104	104	104	104	104	104	104	104	104	104	104	104	104	104	104	104	104	124	112	120	114	103	127	127	127	127	127
127	0	125	102	92	92	92	92	113	116	118	100	113	136	136	136	136	127	138	112	95	118	119	118	118	118	118	118	118	118	118	118	118	118	118	118	118	118	118	118	118	118	96	132	133	114	131	1	1	1	3	1
120	125	0	127	126	126	126	126	125	128	139	136	123	128	128	128	128	122	139	117	120	112	112	112	112	112	112	112	112	112	112	112	112	112	112	112	112	112	112	112	112	112	119	119	119	122	120	125	125	125	125	125
119	102	127	0	68	68	68	68	122	119	126	96	83	133	133	133	133	135	133	131	79	104	105	104	104	104	104	104	104	104	104	104	104	104	104	104	104	104	104	104	104	104	101	146	146	69	136	102	102	102	102	102
114	92	126	68	0	0	0	0	115	108	114	93	89	129	129	129	129	125	135	135	83	102	103	102	102	102	102	102	102	102	102	102	102	102	102	102	102	102	102	102	102	102	98	146	145	86	133	92	92	92	92	92
114	92	126	68	0	0	0	0	115	108	114	93	89	129	129	129	129	125	135	135	83	102	103	102	102	102	102	102	102	102	102	102	102	102	102	102	102	102	102	102	102	102	98	146	145	86	133	92	92	92	92	92
114	92	126	68	0	0	0	0	115	108	114	93	89	129	129	129	129	125	135	135	83	102	103	102	102	102	102	102	102	102	102	102	102	102	102	102	102	102	102	102	102	102	98	146	145	86	133	92	92	92	92	92
114	92	126	68	0	0	0	0	115	108	114	93	89	129	129	129	129	125	135	135	83	102	103	102	102	102	102	102	102	102	102	102	102	102	102	102	102	102	102	102	102	102	98	146	145	86	133	92	92	92	92	92
110	113	125	122	115	115	115	115	0	118	133	125	119	105	105	105	105	111	140	118	118	95	96	95	95	95	95	95	95	95	95	95	95	95	95	95	95	95	95	95	95	95	118	120	116	98	101	113	113	113	113	113
113	116	128	119	108	108	108	108	118	0	131	106	115	119	119	119	119	107	143	124	109	106	107	106	106	106	106	106	106	106	106	106	106	106	106	106	106	106	106	106	106	106	108	119	122	109	119	116	116	116	116	116
143	118	139	126	114	114	114	114	133	131	0	122	122	151	151	151	151	146	90	138	109	138	138	138	138	138	138	138	138	138	138	138	138	138	138	138	138	138	138	138	138	138	130	149	145	129	144	118	118	118	118	118
127	100	136	96	93	93	93	93	125	106	122	0	99	130	130	130	130	136	133	136	93	119	120	119	119	119	119	119	119	119	119	119	119	119	119	119	119	119	119	119	119	119	113	137	136	109	129	100	100	100	101	100
127	113	123	83	89	89	89	89	119	115	122	99	0	130	130	130	130	132	148	129	83	113	114	113	113	113	113	113	113	113	113	113	113	113	113	113	113	113	113	113	113	113	106	130	127	100	122	113	113	113	113	113
100	136	128	133	129	129	129	129	105	119	151	130	130	0	0	0	0	95	152	126	136	132	132	132	132	132	132	132	132	132	132	132	132	132	132	132	132	132	132	132	132	132	133	107	107	119	95	136	136	136	136	136
100	136	128	133	129	129	129	129	105	119	151	130	130	0	0	0	0	95	152	126	136	132	132	132	132	132	132	132	132	132	132	132	132	132	132	132	132	132	132	132	132	132	133	107	107	119	95	136	136	136	136	136
100	136	128	133	129	129	129	129	105	119	151	130	130	0	0	0	0	95	152	126	136	132	132	132	132	132	132	132	132	132	132	132	132	132	132	132	132	132	132	132	132	132	133	107	107	119	95	136	136	136	136	136
100	136	128	133	129	129	129	129	105	119	151	130	130	0	0	0	0	95	152	126	136	132	132	132	132	132	132	132	132	132	132	132	132	132	132	132	132	132	132	132	132	132	133	107	107	119	95	136	136	136	136	136
90	127	122	135	125	125	125	125	111	107	146	136	132	95	95	95	95	0	141	122	123	108	109	108	108	108	108	108	108	108	108	108	108	108	108	108	108	108	108	108	108	108	123	107	115	129	101	127	127	127	127	127
139	138	139	133	135	135	135	135	140	143	90	133	148	152	152	152	152	141	0	141	136	137	137	137	137	137	137	137	137	137	137	137	137	137	137	137	137	137	137	137	137	137	138	150	149	140	149	138	138	138	138	138
119	112	117	131	135	135	135	135	118	124	138	136	129	126	126	126	126	122	141	0	119	123	123	123	123	123	123	123	123	123	123	123	123	123	123	123	123	123	123	123	123	123	124	92	89	119	120	112	112	112	113	112
127	95	120	79	83	83	83	83	118	109	109	93	83	136	136	136	136	123	136	119	0	102	103	102	102	102	102	102	102	102	102	102	102	102	102	102	102	102	102	102	102	102	98	138	132	92	128	95	95	95	95	95
104	118	112	104	102	102	102	102	95	106	138	119	113	132	132	132	132	108	137	123	102	0	1	0	0	0	0	0	0	0	0	0	0	0	0	0	0	0	0	0	0	0	118	126	126	100	114	117	117	117	117	117
105	119	112	105	103	103	103	103	96	107	138	120	114	132	132	132	132	109	137	123	103	1	0	1	1	1	1	1	1	1	1	1	1	1	1	1	1	1	1	1	1	1	119	126	126	101	115	118	118	118	118	118
104	118	112	104	102	102	102	102	95	106	138	119	113	132	132	132	132	108	137	123	102	0	1	0	0	0	0	0	0	0	0	0	0	0	0	0	0	0	0	0	0	0	118	126	126	100	114	117	117	117	117	117
104	118	112	104	102	102	102	102	95	106	138	119	113	132	132	132	132	108	137	123	102	0	1	0	0	0	0	0	0	0	0	0	0	0	0	0	0	0	0	0	0	0	118	126	126	100	114	117	117	117	117	117
104	118	112	104	102	102	102	102	95	106	138	119	113	132	132	132	132	108	137	123	102	0	1	0	0	0	0	0	0	0	0	0	0	0	0	0	0	0	0	0	0	0	118	126	126	100	114	117	117	117	117	117
104	118	112	104	102	102	102	102	95	106	138	119	113	132	132	132	132	108	137	123	102	0	1	0	0	0	0	0	0	0	0	0	0	0	0	0	0	0	0	0	0	0	118	126	126	100	114	117	117	117	117	117
104	118	112	104	102	102	102	102	95	106	138	119	113	132	132	132	132	108	137	123	102	0	1	0	0	0	0	0	0	0	0	0	0	0	0	0	0	0	0	0	0	0	118	126	126	100	114	117	117	117	117	117
104	118	112	104	102	102	102	102	95	106	138	119	113	132	132	132	132	108	137	123	102	0	1	0	0	0	0	0	0	0	0	0	0	0	0	0	0	0	0	0	0	0	118	126	126	100	114	117	117	117	117	117
104	118	112	104	102	102	102	102	95	106	138	119	113	132	132	132	132	108	137	123	102	0	1	0	0	0	0	0	0	0	0	0	0	0	0	0	0	0	0	0	0	0	118	126	126	100	114	117	117	117	117	117
104	118	112	104	102	102	102	102	95	106	138	119	113	132	132	132	132	108	137	123	102	0	1	0	0	0	0	0	0	0	0	0	0	0	0	0	0	0	0	0	0	0	118	126	126	100	114	117	117	117	117	117
104	118	112	104	102	102	102	102	95	106	138	119	113	132	132	132	132	108	137	123	102	0	1	0	0	0	0	0	0	0	0	0	0	0	0	0	0	0	0	0	0	0	118	126	126	100	114	117	117	117	117	117
104	118	112	104	102	102	102	102	95	106	138	119	113	132	132	132	132	108	137	123	102	0	1	0	0	0	0	0	0	0	0	0	0	0	0	0	0	0	0	0	0	0	118	126	126	100	114	117	117	117	117	117
104	118	112	104	102	102	102	102	95	106	138	119	113	132	132	132	132	108	137	123	102	0	1	0	0	0	0	0	0	0	0	0	0	0	0	0	0	0	0	0	0	0	118	126	126	100	114	117	117	117	117	117
104	118	112	104	102	102	102	102	95	106	138	119	113	132	132	132	132	108	137	123	102	0	1	0	0	0	0	0	0	0	0	0	0	0	0	0	0	0	0	0	0	0	118	126	126	100	114	117	117	117	117	117
104	118	112	104	102	102	102	102	95	106	138	119	113	132	132	132	132	108	137	123	102	0	1	0	0	0	0	0	0	0	0	0	0	0	0	0	0	0	0	0	0	0	118	126	126	100	114	117	117	117	117	117
104	118	112	104	102	102	102	102	95	106	138	119	113	132	132	132	132	108	137	123	102	0	1	0	0	0	0	0	0	0	0	0	0	0	0	0	0	0	0	0	0	0	118	126	126	100	114	117	117	117	117	117
104	118	112	104	102	102	102	102	95	106	138	119	113	132	132	132	132	108	137	123	102	0	1	0	0	0	0	0	0	0	0	0	0	0	0	0	0	0	0	0	0	0	118	126	126	100	114	117	117	117	117	117
104	118	112	104	102	102	102	102	95	106	138	119	113	132	132	132	132	108	137	123	102	0	1	0	0	0	0	0	0	0	0	0	0	0	0	0	0	0	0	0	0	0	118	126	126	100	114	117	117	117	117	117
104	118	112	104	102	102	102	102	95	106	138	119	113	132	132	132	132	108	137	123	102	0	1	0	0	0	0	0	0	0	0	0	0	0	0	0	0	0	0	0	0	0	118	126	126	100	114	117	117	117	117	117
104	118	112	104	102	102	102	102	95	106	138	119	113	132	132	132	132	108	137	123	102	0	1	0	0	0	0	0	0	0	0	0	0	0	0	0	0	0	0	0	0	0	118	126	126	100	114	117	117	117	117	117
104	118	112	104	102	102	102	102	95	106	138	119	113	132	132	132	132	108	137	123	102	0	1	0	0	0	0	0	0	0	0	0	0	0	0	0	0	0	0	0	0	0	118	126	126	100	114	117	117	117	117	117

104	118	112	104	102	102	102	102	95	106	138	119	113	132	132	132	132	108	137	123	102	0	1	0	0	0	0	0	0	0	0	0	0	0	0	0	0	0	0	0	0	0	118	126	126	100	114	117	117	117	117	117
124	96	119	101	98	98	98	98	118	108	130	113	106	133	133	133	133	123	138	124	98	118	119	118	118	118	118	118	118	118	118	118	118	118	118	118	118	118	118	118	118	118	0	127	130	105	130	95	95	95	95	95
112	132	119	146	146	146	146	146	120	119	149	137	130	107	107	107	107	107	150	92	138	126	126	126	126	126	126	126	126	126	126	126	126	126	126	126	126	126	126	126	126	126	127	0	44	132	111	132	132	132	132	132
120	133	119	146	145	145	145	145	116	122	145	136	127	107	107	107	107	115	149	89	132	126	126	126	126	126	126	126	126	126	126	126	126	126	126	126	126	126	126	126	126	126	130	44	0	130	107	133	133	133	133	133
114	114	122	69	86	86	86	86	98	109	129	109	100	119	119	119	119	129	140	119	92	100	101	100	100	100	100	100	100	100	100	100	100	100	100	100	100	100	100	100	100	100	105	132	130	0	118	114	114	114	115	114
103	131	120	136	133	133	133	133	101	119	144	129	122	95	95	95	95	101	149	120	128	114	115	114	114	114	114	114	114	114	114	114	114	114	114	114	114	114	114	114	114	114	130	111	107	118	0	132	132	132	132	132
127	1	125	102	92	92	92	92	113	116	118	100	113	136	136	136	136	127	138	112	95	117	118	117	117	117	117	117	117	117	117	117	117	117	117	117	117	117	117	117	117	117	95	132	133	114	132	0	0	0	2	0
127	1	125	102	92	92	92	92	113	116	118	100	113	136	136	136	136	127	138	112	95	117	118	117	117	117	117	117	117	117	117	117	117	117	117	117	117	117	117	117	117	117	95	132	133	114	132	0	0	0	2	0
127	1	125	102	92	92	92	92	113	116	118	100	113	136	136	136	136	127	138	112	95	117	118	117	117	117	117	117	117	117	117	117	117	117	117	117	117	117	117	117	117	117	95	132	133	114	132	0	0	0	2	0
127	3	125	102	92	92	92	92	113	116	118	101	113	136	136	136	136	127	138	113	95	117	118	117	117	117	117	117	117	117	117	117	117	117	117	117	117	117	117	117	117	117	95	132	133	115	132	2	2	2	0	2
127	1	125	102	92	92	92	92	113	116	118	100	113	136	136	136	136	127	138	112	95	117	118	117	117	117	117	117	117	117	117	117	117	117	117	117	117	117	117	117	117	117	95	132	133	114	132	0	0	0	2	0
118	125	112	128	118	118	118	118	129	120	138	129	128	130	130	130	130	130	149	126	134	137	137	137	137	137	137	137	137	137	137	137	137	137	137	137	137	137	137	137	137	137	122	126	125	117	124	124	124	124	124	124
139	119	143	123	126	126	126	126	139	145	69	129	130	153	153	153	153	142	71	137	123	137	138	137	137	137	137	137	137	137	137	137	137	137	137	137	137	137	137	137	137	137	130	149	148	133	147	119	119	119	119	119
88	131	124	142	134	134	134	134	95	125	146	130	124	86	86	86	86	84	149	121	131	106	107	106	106	106	106	106	106	106	106	106	106	106	106	106	106	106	106	106	106	106	137	99	99	121	90	131	131	131	131	131
117	131	123	136	136	136	136	136	100	118	148	131	123	104	104	104	104	109	153	120	124	117	117	117	117	117	117	117	117	117	117	117	117	117	117	117	117	117	117	117	117	117	126	89	103	115	93	131	131	131	131	131
108	99	131	103	92	92	92	92	116	113	131	111	113	115	115	115	115	113	140	124	106	108	109	108	108	108	108	108	108	108	108	108	108	108	108	108	108	108	108	108	108	108	106	135	139	102	128	99	99	99	99	99
109	101	132	104	93	93	93	93	116	114	132	111	115	116	116	116	116	114	141	124	107	108	109	108	108	108	108	108	108	108	108	108	108	108	108	108	108	108	108	108	108	108	107	135	139	102	128	101	101	101	101	101
108	99	131	103	92	92	92	92	116	113	131	111	113	115	115	115	115	113	140	124	106	108	109	108	108	108	108	108	108	108	108	108	108	108	108	108	108	108	108	108	108	108	106	135	139	102	128	99	99	99	99	99
108	99	131	103	92	92	92	92	116	113	131	111	113	115	115	115	115	113	140	124	106	108	109	108	108	108	108	108	108	108	108	108	108	108	108	108	108	108	108	108	108	108	106	135	139	102	128	99	99	99	99	99
108	99	131	103	92	92	92	92	116	113	131	111	113	115	115	115	115	113	140	124	106	108	109	108	108	108	108	108	108	108	108	108	108	108	108	108	108	108	108	108	108	108	106	135	139	102	128	99	99	99	99	99
108	99	131	103	92	92	92	92	116	113	131	111	113	115	115	115	115	113	140	124	106	108	109	108	108	108	108	108	108	108	108	108	108	108	108	108	108	108	108	108	108	108	106	135	139	102	128	99	99	99	99	99
108	99	131	103	92	92	92	92	116	113	131	111	113	115	115	115	115	113	140	124	106	108	109	108	108	108	108	108	108	108	108	108	108	108	108	108	108	108	108	108	108	108	106	135	139	102	128	99	99	99	99	99
108	99	131	103	92	92	92	92	116	113	131	111	113	115	115	115	115	113	140	124	106	108	109	108	108	108	108	108	108	108	108	108	108	108	108	108	108	108	108	108	108	108	106	135	139	102	128	99	99	99	99	99
108	99	131	103	92	92	92	92	116	113	131	111	113	115	115	115	115	113	140	124	106	108	109	108	108	108	108	108	108	108	108	108	108	108	108	108	108	108	108	108	108	108	106	135	139	102	128	99	99	99	99	99
108	99	131	103	92	92	92	92	116	113	131	111	113	115	115	115	115	113	140	124	106	108	109	108	108	108	108	108	108	108	108	108	108	108	108	108	108	108	108	108	108	108	106	135	139	102	128	99	99	99	99	99
108	99	131	103	92	92	92	92	116	113	131	111	113	115	115	115	115	113	140	124	106	108	109	108	108	108	108	108	108	108	108	108	108	108	108	108	108	108	108	108	108	108	106	135	139	102	128	99	99	99	99	99
108	99	131	103	92	92	92	92	116	113	131	111	113	115	115	115	115	113	140	124	106	108	109	108	108	108	108	108	108	108	108	108	108	108	108	108	108	108	108	108	108	108	106	135	139	102	128	99	99	99	99	99
108	99	131	103	92	92	92	92	116	113	131	111	113	115	115	115	115	113	140	124	106	108	109	108	108	108	108	108	108	108	108	108	108	108	108	108	108	108	108	108	108	108	106	135	139	102	128	99	99	99	99	99
108	99	131	103	92	92	92	92	116	113	131	111	113	115	115	115	115	113	140	124	106	108	109	108	108	108	108	108	108	108	108	108	108	108	108	108	108	108	108	108	108	108	106	135	139	102	128	99	99	99	99	99
108	99	131	103	92	92	92	92	116	113	131	111	113	115	115	115	115	113	140	124	106	108	109	108	108	108	108	108	108	108	108	108	108	108	108	108	108	108	108	108	108	108	106	135	139	102	128	99	99	99	99	99
108	99	131	103	92	92	92	92	116	113	131	111	113	115	115	115	115	113	140	124	106	108	109	108	108	108	108	108	108	108	108	108	108	108	108	108	108	108	108	108	108	108	106	135	139	102	128	99	99	99	99	99
108	99	131	103	92	92	92	92	116	113	131	111	113	115	115	115	115	113	140	124	106	108	109	108	108	108	108	108	108	108	108	108	108	108	108	108	108	108	108	108	108	108	106	135	139	102	128	99	99	99	99	99
108	99	131	103	92	92	92	92	116	113	131	111	113	115	115	115	115	113	140	124	106	108	109	108	108	108	108	108	108	108	108	108	108	108	108	108	108	108	108	108	108	108	106	135	139	102	128	99	99	99	99	99
108	99	131	103	92	92	92	92	116	113	131	111	113	115	115	115	115	113	140	124	106	108	109	108	108	108	108	108	108	108	108	108	108	108	108	108	108	108	108	108	108	108	106	135	139	102	128	99	99	99	99	99
108	99	131	103	92	92	92	92	116	113	131	111	113	115	115	115	115	113	140	124	106	108	109	108	108	108	108	108	108	108	108	108	108	108	108	108	108	108	108	108	108	108	106	135	139	102	128	99	99	99	99	99
107	99	131	102	91	91	91	91	116	112	132	110	113	114	114	114	114	113	141	124	106	108	109	108	108	108	108	108	108	108	108	108	108	108	108	108	108	108	108	108	108	108	105	135	139	101	127	99	99	99	99	99
112	137	120	145	144	144	144	144	125	125	149	140	136	116	116	116	116	106	151	103	139	122	122	122	122	122	122	122	122	122	122	122	122	122	122	122	122	122	122	122	122	122	138	79	79	137	109	137	137	137	137	137
114	140	120	146	149	149	149	149	127	124	149	138	132	115	115	115	115	112	151	102	137	130	130	130	130	130	130	130	130	130	130	130	130	130	130	130	130	130	130	130	130	130	134	74	78	136	106	140	140	140	140	140
114	140	121	146	149	149	149	149	127	124	149	138	132	115	115	115	115	113	150	102	137	130	130	130	130	130	130	130	130	130	130	130	130	130	130	130	130	130	130	130	130	130	134	75	79	136	107	140	140	140	140	140
114	140	120	146	149	149	149	149	127	124	149	138	132	115	115	115	115	112	151	102	137	130	130	130	130	130	130	130	130	130	130	130	130	130	130	130	130	130	130	130	130	130	134	74	78	136	106	140	140	140	140	140
112	137	120	145	144	144	144	144	125	125	149	140	136	116	116	116	116	106	151	103	139	122	122	122	122	122	122	122	122	122	122	122	122	122	122	122	122	122	122	122	122	122	138	79	79	137	109	137	137	137	137	137
112	137	120	145	144	144	144	144	125	125	149	140	136	116	116	116	116	106	151	103	139	122	122	122	122	122	122	122	122	122	122	122	122	122	122	122	122	122	122	122	122	122	138	79	79	137	109	137	137	137	137	137
121	137	123	146	149	149	149	149	123	114	149	138	125	116	116	116	116	113	149	100	139	128	128	128	128	128	128	128	128	128	128	128	128	128	128	128	128	128	128	128	128	128	129	68	66	136	111	137	137	137	137	137
114	140	120	146	149	149	149	149	127	124	149	138	132	115	115	115	115	112	151	102	137	130	130	130	130	130	130	130	130	130	130	130	130	130	130	130	130	130	130	130	130	130	134	74	78	136	106	140	140	140	140	140
114	140	120	146	149	149	149	149	127	124	149	138	132	115	115	115	115	112	151	102	137	130	130	130	130	130	130	130	130	130	130	130	130	130	130	130	130	130	130	130	130	130	134	74	78	136	106	140	140	140	140	140
114	140	120	146	149	149	149	149	127	124	149	138	132	115	115	115	115	112	151	102	137	130	130	130	130	130	130	130	130	130	130	130	130	130	130	130	130	130	130	130	130	130	134	74	78	136	106	140	140	140	140	140
112	137	120	145	144	144	144	144	125	125	149	140	136	116	116	116	116	106	151	103	139	122	122	122	122	122	122	122	122	122	122	122	122	122	122	122	122	122	122	122	122	122	138	79	79	137	109	137	137	137	137	137
114	140	120	146	149	149	149	149	127	124	149	138	132	115	115	115	115	112	151	102	137	130	130	130	130	130	130	130	130	130	130	130	130	130	130	130	130	130	130	130	130	130	134	74	78	136	106	140	140	140	140	140
109	132	120	129	125	125	125	125	132	115	138	135	121	130	130	130	130	118	143	96	120	123	123	123	123	123	123	123	123	123	123	123	123	123	123	123	123	123	123	123	123	123	133	98	100	121	116	132	132	132	132	132
129	80	136	104	97	97	97	97	133	110	106	106	125	133	133	133	133	126	115	130	107	128	129	128	128	128	128	128	128	128	128	128	128	128	128	128	128	128	128	128	128	128	112	140	141	113	132	80	80	80	81	80
89	128	119	123	109	109	109	109	123	120	146	125	126	89	89	89	89	60	144	124	125	116	117	116	116	116	116	116	116	116	116	116	116	116	116	116	116	116	116	116	116	116	121	120	125	116	114	128	128	128	128	128
89	128	119	123	109	109	109	109	123	120	146	125	126	89	89	89	89	60	144	124	125	116	117	116	116	116	116	116	116	116	116	116	116	116	116	116	116	116	116	116	116	116	121	120	125	116	114	128	128	128	128	128
89	128	119	123	109	109	109	109	123	120	146	125	126	89	89	89	89	60	144	124	125	116	117	116	116	116	116	116	116	116	116	116	116	116	116	116	116	116	116	116	116	116	121	120	125	116	114	128	128	128	128	128
89	128	119	123	109	109	109	109	123	120	146	125	126	89	89	89	89	60	144	124	125	116	117	116	116	116	116	116	116	116	116	116	116	116	116	116	116	116	116	116	116	116	121	120	125	116	114	128	128	128	128	128
97	108	124	113	109	109	109	109	115	110	134	117	121	110	110	110	110	100	143	117	113	116	117	116	116	116	116	116	116	116	116	116	116	116	116	116	116	116	116	116	116	116	112	130	135	103	106	108	108	108	108	108
115	111	127	116	100	100	100	100	124	105	110	102	116	120	120	120	120	127	126	139	111	107	108	107	107	107	107	107	107	107	107	107	107	107	107	107	107	107	107	107	107	107	114	135	130	119	120	111	111	111	111	111
96	114	131	119	107	107	107	107	108	104	134	130	117	101	101	101	101	95	144	118	114	114	115	114	114	114	114	114	114	114	114	114	114	114	114	114	114	114	114	114	114	114	112	121	126	105	105	114	114	114	114	114
115	126	131	134	130	130	130	130	125	114	138	125	130	117	117	117	117	115	144	112	127	130	130	130	130	130	130	130	130	130	130	130	130	130	130	130	130	130	130	130	130	130	127	94	101	116	115	126	126	126	126	126
106	115	119	92	89	89	89	89	113	107	135	111	106	122	122	122	122	111	143	130	92	70	71	70	70	70	70	70	70	70	70	70	70	70	70	70	70	70	70	70	70	70	109	129	133	86	116	115	115	115	115	115
143	118	139	126	114	114	114	114	133	131	0	122	122	151	151	151	151	146	90	138	109	138	138	138	138	138	138	138	138	138	138	138	138	138	138	138	138	138	138	138	138	138	130	149	145	129	144	118	118	118	118	118
105	130	115	139	139	139	139	139	127	115	139	139	128	113	113	113	113	105	145	97	126	113	114	113	113	113	113	113	113	113	113	113	113	113	113	113	113	113	113	113	113	113	126	73	78	129	118	129	129	129	129	129
104	114	118	90	87	87	87	87	112	106	134	111	106	122	122	122	122	109	143	128	90	68	69	68	68	68	68	68	68	68	68	68	68	68	68	68	68	68	68	68	68	68	107	129	133	85	116	114	114	114	114	114
104	114	118	90	87	87	87	87	112	106	134	111	106	122	122	122	122	109	143	128	90	68	69	68	68	68	68	68	68	68	68	68	68	68	68	68	68	68	68	68	68	68	107	129	133	85	116	114	114	114	114	114
104	114	118	90	87	87	87	87	112	106	134	111	106	122	122	122	122	109	143	128	90	68	69	68	68	68	68	68	68	68	68	68	68	68	68	68	68	68	68	68	68	68	107	129	133	85	116	114	114	114	114	114
146	137	146	140	133	133	133	133	139	143	82	131	148	152	152	152	152	142	61	145	132	149	150	149	149	149	149	149	149	149	149	149	149	149	149	149	149	149	149	149	149	149	145	151	149	140	146	137	137	137	137	137
116	103	117	82	82	82	82	82	107	108	123	107	108	120	120	120	120	113	137	123	77	91	92	91	91	91	91	91	91	91	91	91	91	91	91	91	91	91	91	91	91	91	97	128	129	80	121	103	103	103	103	103
121	131	122	125	127	127	127	127	127	112	139	127	130	132	132	132	132	130	140	111	117	100	100	100	100	100	100	100	100	100	100	100	100	100	100	100	100	100	100	100	100	100	123	112	108	120	115	131	131	131	131	131
123	102	125	95	95	95	95	95	129	112	97	101	106	139	139	139	139	129	119	127	90	113	114	113	113	113	113	113	113	113	113	113	113	113	113	113	113	113	113	113	113	113	107	143	142	108	136	102	102	102	102	102
92	133	130	137	132	132	132	132	89	118	145	140	128	82	82	82	82	91	147	117	140	119	119	119	119	119	119	119	119	119	119	119	119	119	119	119	119	119	119	119	119	119	132	102	113	122	81	133	133	133	133	133
89	127	119	129	124	124	124	124	93	120	143	130	124	75	75	75	75	95	148	113	131	112	112	112	112	112	112	112	112	112	112	112	112	112	112	112	112	112	112	112	112	112	127	96	103	117	85	127	127	127	127	127
129	97	127	76	90	90	90	90	126	112	119	108	85	138	138	138	138	130	133	126	67	106	106	106	106	106	106	106	106	106	106	106	106	106	106	106	106	106	106	106	106	106	91	140	143	90	143	96	96	96	96	96
125	106	135	99	92	92	92	92	126	106	92	98	100	132	132	132	132	132	130	139	110	120	120	120	120	120	120	120	120	120	120	120	120	120	120	120	120	120	120	120	120	120	106	143	144	107	137	106	106	106	106	106
113	132	118	109	113	113	113	113	97	121	142	115	102	120	120	120	120	130	145	133	100	75	76	75	75	75	75	75	75	75	75	75	75	75	75	75	75	75	75	75	75	75	118	122	119	92	102	132	132	132	132	132
127	105	137	81	76	76	76	76	124	115	109	100	103	141	141	141	141	133	130	137	90	111	112	111	111	111	111	111	111	111	111	111	111	111	111	111	111	111	111	111	111	111	106	140	139	98	140	105	105	105	105	105
102	136	119	141	143	143	143	143	104	111	146	134	126	87	87	87	87	94	149	116	138	122	122	122	122	122	122	122	122	122	122	122	122	122	122	122	122	122	122	122	122	122	132	97	97	118	92	137	137	137	137	137
122	100	128	71	90	90	90	90	117	119	125	105	94	139	139	139	139	124	129	130	77	101	102	101	101	101	101	101	101	101	101	101	101	101	101	101	101	101	101	101	101	101	104	137	138	89	132	99	99	99	99	99

137	127	107	138	135	135	135	135	135	133	139	144	135	134	134	134	134	137	146	120	134	137	137	137	137	137	137	137	137	137	137	137	137	137	137	137	137	137	137	137	137	137	132	128	131	129	134	127	127	127	127	127
95	117	124	107	104	104	104	104	104	112	134	110	130	108	108	108	108	101	135	128	117	88	89	88	88	88	88	88	88	88	88	88	88	88	88	88	88	88	88	88	88	88	118	128	130	104	115	117	117	117	117	117
90	116	124	118	110	110	110	110	107	108	131	118	122	111	111	111	111	104	136	125	120	95	96	95	95	95	95	95	95	95	95	95	95	95	95	95	95	95	95	95	95	95	114	128	132	119	111	115	115	115	115	115
125	108	121	103	95	95	95	95	103	119	133	113	110	124	124	124	124	112	134	122	96	67	68	67	67	67	67	67	67	67	67	67	67	67	67	67	67	67	67	67	67	67	109	131	133	100	118	108	108	108	108	108
111	106	121	101	97	97	97	97	119	103	134	114	115	114	114	114	114	114	142	135	101	108	108	108	108	108	108	108	108	108	108	108	108	108	108	108	108	108	108	108	108	108	96	126	129	107	119	106	106	106	106	106
88	128	122	131	130	130	130	130	113	126	140	124	131	108	108	108	108	103	138	123	123	89	89	89	89	89	89	89	89	89	89	89	89	89	89	89	89	89	89	89	89	89	126	114	112	128	105	127	127	127	127	127
104	106	131	112	104	104	104	104	126	114	134	118	119	116	116	116	116	105	140	138	110	112	112	112	112	112	112	112	112	112	112	112	112	112	112	112	112	112	112	112	112	112	112	131	140	119	121	106	106	106	106	106
97	117	127	122	118	118	118	118	104	112	143	127	131	95	95	95	95	100	145	124	121	112	112	112	112	112	112	112	112	112	112	112	112	112	112	112	112	112	112	112	112	112	117	121	127	114	112	117	117	117	117	117
147	130	141	130	134	134	134	134	150	140	123	131	141	156	156	156	156	146	118	142	128	142	143	142	142	142	142	142	142	142	142	142	142	142	142	142	142	142	142	142	142	142	136	145	147	148	150	130	130	130	130	130
110	118	124	123	116	116	116	116	106	107	129	116	125	120	120	120	120	111	136	114	120	110	110	110	110	110	110	110	110	110	110	110	110	110	110	110	110	110	110	110	110	110	115	112	119	110	121	118	118	118	118	118
78	121	113	124	121	121	121	121	110	108	144	125	127	101	101	101	101	94	149	120	127	107	107	107	107	107	107	107	107	107	107	107	107	107	107	107	107	107	107	107	107	107	118	111	113	119	105	122	122	122	122	122
141	138	146	128	137	137	137	137	144	147	93	138	139	151	151	151	151	149	61	142	135	142	142	142	142	142	142	142	142	142	142	142	142	142	142	142	142	142	142	142	142	142	139	147	147	135	145	138	138	138	138	138
140	134	142	129	138	138	138	138	140	144	92	133	142	148	148	148	148	145	50	138	136	140	140	140	140	140	140	140	140	140	140	140	140	140	140	140	140	140	140	140	140	140	136	145	144	137	143	134	134	134	134	134
90	128	120	125	115	115	115	115	110	127	138	129	128	107	107	107	107	114	144	128	124	103	104	103	103	103	103	103	103	103	103	103	103	103	103	103	103	103	103	103	103	103	121	124	125	126	110	128	128	128	128	128
74	128	113	131	128	128	128	128	110	122	141	135	131	97	97	97	97	99	145	113	132	104	104	104	104	104	104	104	104	104	104	104	104	104	104	104	104	104	104	104	104	104	121	104	107	117	92	127	127	127	127	127
97	110	123	114	107	107	107	107	106	110	140	118	116	98	98	98	98	94	145	115	104	103	104	103	103	103	103	103	103	103	103	103	103	103	103	103	103	103	103	103	103	103	116	118	122	109	108	110	110	110	111	110
102	116	128	117	117	117	117	117	104	122	141	119	130	93	93	93	93	95	140	119	111	110	110	110	110	110	110	110	110	110	110	110	110	110	110	110	110	110	110	110	110	110	121	119	126	104	107	116	116	116	117	116
100	122	124	111	107	107	107	107	111	109	141	113	108	117	117	117	117	109	144	127	119	80	81	80	80	80	80	80	80	80	80	80	80	80	80	80	80	80	80	80	80	80	117	128	130	109	118	121	121	121	121	121
73	130	121	129	124	124	124	124	109	120	143	137	134	75	75	75	75	98	145	117	129	120	120	120	120	120	120	120	120	120	120	120	120	120	120	120	120	120	120	120	120	120	124	106	113	115	88	130	130	130	130	130
108	117	123	130	126	126	126	126	103	117	134	129	122	117	117	117	117	109	131	113	115	102	102	102	102	102	102	102	102	102	102	102	102	102	102	102	102	102	102	102	102	102	124	109	118	111	109	117	117	117	117	117
112	116	118	112	101	101	101	101	120	116	127	121	111	117	117	117	117	118	142	104	102	107	108	107	107	107	107	107	107	107	107	107	107	107	107	107	107	107	107	107	107	107	124	115	114	110	109	116	116	116	116	116
88	125	124	118	115	115	115	115	107	118	149	136	132	79	79	79	79	81	150	128	131	113	113	113	113	113	113	113	113	113	113	113	113	113	113	113	113	113	113	113	113	113	115	120	126	116	104	125	125	125	125	125
96	132	125	123	119	119	119	119	91	117	144	121	118	84	84	84	84	103	146	124	120	97	98	97	97	97	97	97	97	97	97	97	97	97	97	97	97	97	97	97	97	97	122	114	115	100	91	132	132	132	132	132
137	136	144	130	138	138	138	138	141	142	92	138	140	153	153	153	153	144	66	138	135	145	145	145	145	145	145	145	145	145	145	145	145	145	145	145	145	145	145	145	145	145	138	147	147	135	150	136	136	136	136	136
103	120	127	115	108	108	108	108	106	100	142	126	111	117	117	117	117	107	150	120	108	86	87	86	86	86	86	86	86	86	86	86	86	86	86	86	86	86	86	86	86	86	121	112	115	100	100	120	120	120	120	120
87	122	125	121	113	113	113	113	107	117	129	123	118	96	96	96	96	101	134	125	120	96	97	96	96	96	96	96	96	96	96	96	96	96	96	96	96	96	96	96	96	96	118	119	123	118	109	121	121	121	121	121
86	128	106	129	132	132	132	132	106	123	145	137	129	93	93	93	93	92	139	112	131	105	105	105	105	105	105	105	105	105	105	105	105	105	105	105	105	105	105	105	105	105	120	102	101	120	103	128	128	128	128	128
104	119	129	113	110	110	110	110	118	126	140	125	128	80	80	80	80	101	141	128	122	106	106	106	106	106	106	106	106	106	106	106	106	106	106	106	106	106	106	106	106	106	125	116	121	108	105	119	119	119	119	119
92	115	103	110	103	103	103	103	96	105	132	119	124	119	119	119	119	116	141	115	107	72	72	72	72	72	72	72	72	72	72	72	72	72	72	72	72	72	72	72	72	72	118	122	122	106	101	114	114	114	114	114
92	131	118	131	129	129	129	129	117	132	135	123	122	100	100	100	100	100	143	124	131	110	110	110	110	110	110	110	110	110	110	110	110	110	110	110	110	110	110	110	110	110	129	106	107	130	93	131	131	131	131	131
66	133	119	126	124	124	124	124	104	116	139	138	136	93	93	93	93	97	143	121	133	110	110	110	110	110	110	110	110	110	110	110	110	110	110	110	110	110	110	110	110	110	123	113	117	122	99	133	133	133	133	133
108	112	127	103	96	96	96	96	107	118	139	106	115	112	112	112	112	109	139	126	116	87	88	87	87	87	87	87	87	87	87	87	87	87	87	87	87	87	87	87	87	87	113	133	134	105	119	112	112	112	112	112
114	116	122	110	109	109	109	109	129	103	128	116	118	127	127	127	127	122	137	115	101	104	104	104	104	104	104	104	104	104	104	104	104	104	104	104	104	104	104	104	104	104	113	126	133	112	125	116	116	116	116	116
81	123	132	132	124	124	124	124	115	118	147	119	127	70	70	70	70	92	143	130	137	126	127	126	126	126	126	126	126	126	126	126	126	126	126	126	126	126	126	126	126	126	126	114	119	127	100	123	123	123	123	123
132	121	133	111	103	103	103	103	130	131	103	117	118	148	148	148	148	134	95	138	103	117	118	117	117	117	117	117	117	117	117	117	117	117	117	117	117	117	117	117	117	117	118	149	144	120	143	121	121	121	121	121
103	118	124	111	112	112	112	112	100	120	137	124	125	118	118	118	118	99	143	121	113	79	79	79	79	79	79	79	79	79	79	79	79	79	79	79	79	79	79	79	79	79	125	122	121	109	99	118	118	118	118	118
103	121	121	115	111	111	111	111	99	119	145	122	127	110	110	110	110	95	139	116	112	73	74	73	73	73	73	73	73	73	73	73	73	73	73	73	73	73	73	73	73	73	125	118	119	104	107	120	120	120	120	120
102	111	134	104	101	101	101	101	100	103	138	110	120	93	93	93	93	98	142	125	102	91	92	91	91	91	91	91	91	91	91	91	91	91	91	91	91	91	91	91	91	91	108	129	130	106	124	111	111	111	111	111
114	116	122	110	112	112	112	112	111	120	138	123	124	119	119	119	119	111	142	127	107	84	85	84	84	84	84	84	84	84	84	84	84	84	84	84	84	84	84	84	84	84	114	132	130	107	107	116	116	116	116	116
106	121	133	116	114	114	114	114	119	119	141	128	126	113	113	113	113	108	139	130	118	102	103	102	102	102	102	102	102	102	102	102	102	102	102	102	102	102	102	102	102	102	123	129	133	123	115	121	121	121	121	121
95	121	119	122	122	122	122	122	97	119	144	117	119	81	81	81	81	100	151	127	118	126	127	126	126	126	126	126	126	126	126	126	126	126	126	126	126	126	126	126	126	126	114	115	110	111	86	121	121	121	122	121
73	125	105	115	116	116	116	116	113	114	140	126	117	97	97	97	97	89	136	110	113	97	98	97	97	97	97	97	97	97	97	97	97	97	97	97	97	97	97	97	97	97	123	107	113	113	104	125	125	125	125	125
143	129	140	116	124	124	124	124	145	137	120	124	128	148	148	148	148	144	113	140	120	134	134	134	134	134	134	134	134	134	134	134	134	134	134	134	134	134	134	134	134	134	137	145	147	141	144	129	129	129	129	129
103	117	139	126	119	119	119	119	120	123	144	117	126	92	92	92	92	102	140	132	131	122	123	122	122	122	122	122	122	122	122	122	122	122	122	122	122	122	122	122	122	122	120	123	129	124	118	117	117	117	117	117
100	122	117	112	98	98	98	98	109	116	143	119	118	117	117	117	117	104	139	131	105	70	70	70	70	70	70	70	70	70	70	70	70	70	70	70	70	70	70	70	70	70	108	127	130	112	123	121	121	121	121	121
95	121	138	108	102	102	102	102	111	104	145	115	122	96	96	96	96	95	146	127	116	98	99	98	98	98	98	98	98	98	98	98	98	98	98	98	98	98	98	98	98	98	111	127	134	105	126	121	121	121	121	121
87	119	127	135	125	125	125	125	113	109	144	127	133	91	91	91	91	79	148	123	126	117	118	117	117	117	117	117	117	117	117	117	117	117	117	117	117	117	117	117	117	117	125	109	113	119	97	119	119	119	119	119
106	119	128	122	116	116	116	116	113	120	147	126	130	88	88	88	88	96	145	120	123	120	120	120	120	120	120	120	120	120	120	120	120	120	120	120	120	120	120	120	120	120	123	122	121	116	109	119	119	119	119	119
114	100	119	95	96	96	96	96	117	111	127	104	109	129	129	129	129	120	137	129	94	107	107	107	107	107	107	107	107	107	107	107	107	107	107	107	107	107	107	107	107	107	106	132	135	96	126	100	100	100	100	100
71	134	116	136	127	127	127	127	108	120	138	128	121	98	98	98	98	83	135	124	126	107	108	107	107	107	107	107	107	107	107	107	107	107	107	107	107	107	107	107	107	107	131	111	114	131	95	134	134	134	134	134
92	128	133	139	132	132	132	132	106	113	147	123	129	79	79	79	79	88	145	126	142	118	119	118	118	118	118	118	118	118	118	118	118	118	118	118	118	118	118	118	118	118	132	105	113	132	98	128	128	128	128	128
100	121	115	116	109	109	109	109	109	121	147	125	124	125	125	125	125	105	139	129	108	74	75	74	74	74	74	74	74	74	74	74	74	74	74	74	74	74	74	74	74	74	117	122	126	111	110	121	121	121	121	121
101	113	126	117	110	110	110	110	107	121	144	123	119	88	88	88	88	103	140	120	104	88	89	88	88	88	88	88	88	88	88	88	88	88	88	88	88	88	88	88	88	88	116	114	120	112	110	113	113	113	113	113
103	123	132	114	108	108	108	108	99	111	130	118	124	111	111	111	111	98	129	125	117	92	93	92	92	92	92	92	92	92	92	92	92	92	92	92	92	92	92	92	92	92	123	127	126	111	119	123	123	123	123	123
95	127	113	123	114	114	114	114	112	115	142	122	127	104	104	104	104	104	145	123	117	88	88	88	88	88	88	88	88	88	88	88	88	88	88	88	88	88	88	88	88	88	122	111	111	116	106	126	126	126	126	126
95	118	136	105	95	95	95	95	99	101	140	111	123	95	95	95	95	95	141	128	103	87	88	87	87	87	87	87	87	87	87	87	87	87	87	87	87	87	87	87	87	87	114	126	130	103	119	118	118	118	118	118
108	121	139	122	116	116	116	116	106	132	143	111	116	78	78	78	78	115	143	126	126	106	107	106	106	106	106	106	106	106	106	106	106	106	106	106	106	106	106	106	106	106	125	130	126	129	116	121	121	121	121	121
101	113	126	117	110	110	110	110	107	121	144	123	119	88	88	88	88	103	140	120	104	88	89	88	88	88	88	88	88	88	88	88	88	88	88	88	88	88	88	88	88	88	116	114	120	112	110	113	113	113	113	113
130	116	127	82	103	103	103	103	105	125	119	101	79	129	129	129	129	143	140	126	90	110	110	110	110	110	110	110	110	110	110	110	110	110	110	110	110	110	110	110	110	110	119	132	129	97	124	116	116	116	116	116
127	118	132	105	88	88	88	88	129	124	99	106	109	138	138	138	138	138	114	135	103	117	118	117	117	117	117	117	117	117	117	117	117	117	117	117	117	117	117	117	117	117	117	142	140	106	129	118	118	118	118	118
102	119	116	81	89	89	89	89	114	111	139	105	109	120	120	120	120	127	143	132	104	68	69	68	68	68	68	68	68	68	68	68	68	68	68	68	68	68	68	68	68	68	113	130	131	77	113	119	119	119	119	119
91	128	123	131	122	122	122	122	100	126	147	117	126	103	103	103	103	99	145	123	126	103	103	103	103	103	103	103	103	103	103	103	103	103	103	103	103	103	103	103	103	103	125	117	116	123	99	128	128	128	128	128
133	113	131	105	111	111	111	111	131	125	83	103	120	145	145	145	145	133	114	129	100	131	131	131	131	131	131	131	131	131	131	131	131	131	131	131	131	131	131	131	131	131	124	145	144	115	147	113	113	113	113	113
103	120	120	119	117	117	117	117	106	124	136	121	123	108	108	108	108	115	139	116	115	94	94	94	94	94	94	94	94	94	94	94	94	94	94	94	94	94	94	94	94	94	123	123	118	119	102	119	119	119	119	119
94	119	119	107	105	105	105	105	105	115	143	125	115	114	114	114	114	103	142	119	120	82	83	82	82	82	82	82	82	82	82	82	82	82	82	82	82	82	82	82	82	82	112	128	128	107	118	119	119	119	119	119
86	121	118	128	118	118	118	118	112	116	146	120	130	104	104	104	104	100	140	124	122	104	104	104	104	104	104	104	104	104	104	104	104	104	104	104	104	104	104	104	104	104	124	118	119	126	106	120	120	120	120	120
97	129	120	134	133	133	133	133	100	119	137	124	134	85	85	85	85	97	134	120	130	119	119	119	119	119	119	119	119	119	119	119	119	119	119	119	119	119	119	119	119	119	127	105	108	129	100	129	129	129	129	129
129	103	128	105	86	86	86	86	123	112	93	100	106	142	142	142	142	131	120	137	100	117	118	117	117	117	117	117	117	117	117	117	117	117	117	117	117	117	117	117	117	117	105	147	142	106	139	103	103	103	103	103
86	124	120	130	118	118	118	118	106	121	143	128	131	84	84	84	84	90	144	121	128	101	101	101	101	101	101	101	101	101	101	101	101	101	101	101	101	101	101	101	101	101	122	115	117	121	97	124	124	124	124	124
104	116	141	116	107	107	107	107	109	118	134	118	119	109	109	109	109	102	140	132	119	107	108	107	107	107	107	107	107	107	107	107	107	107	107	107	107	107	107	107	107	107	118	127	130	116	116	116	116	116	116	116
102	119	116	117	116	116	116	116	114	118	136	123	116	121	121	121	121	113	145	120	114	80	80	80	80	80	80	80	80	80	80	80	80	80	80	80	80	80	80	80	80	80	124	119	117	114	104	118	118	118	118	118
138	141	144	139	138	138	138	138	146	139	136	143	145	144	144	144	144	148	135	140	137	145	145	145	145	145	145	145	145	145	145	145	145	145	145	145	145	145	145	145	145	145	144	149	149	144	146	141	141	141	142	141
93	129	120	113	109	109	109	109	105	108	148	122	128	102	102	102	102	94	147	124	123	88	89	88	88	88	88	88	88	88	88	88	88	88	88	88	88	88	88	88	88	88	120	120	121	107	112	129	129	129	129	129
109	115	115	105	103	103	103	103	98	117	137	117	117	124	124	124	124	115	136	126	117	64	65	64	64	64	64	64	64	64	64	64	64	64	64	64	64	64	64	64	64	64	117	130	127	100	113	115	115	115	115	115
109	124	120	119	106	106	106	106	106	109	142	118	115	116	116	116	116	106	139	126	123	60	61	60	60	60	60	60	60	60	60	60	60	60	60	60	60	60	60	60	60	60	114	128	130	113	119	124	124	124	124	124

104	115	130	113	110	110	110	110	106	113	141	119	124	113	113	113	113	97	139	129	110	95	95	95	95	95	95	95	95	95	95	95	95	95	95	95	95	95	95	95	95	95	120	123	120	111	107	115	115	115	115	115
104	118	124	110	106	106	106	106	112	117	134	121	114	125	125	125	125	109	135	130	113	82	83	82	82	82	82	82	82	82	82	82	82	82	82	82	82	82	82	82	82	82	120	127	131	109	117	118	118	118	118	118
88	126	120	138	128	128	128	128	91	118	146	125	130	88	88	88	88	94	144	120	132	111	111	111	111	111	111	111	111	111	111	111	111	111	111	111	111	111	111	111	111	111	123	105	107	120	81	126	126	126	126	126
71	127	123	134	122	122	122	122	104	116	147	120	137	107	107	107	107	93	134	120	130	102	103	102	102	102	102	102	102	102	102	102	102	102	102	102	102	102	102	102	102	102	128	114	118	121	98	127	127	127	127	127
96	120	128	121	114	114	114	114	117	116	141	117	112	113	113	113	113	115	141	135	125	114	115	114	114	114	114	114	114	114	114	114	114	114	114	114	114	114	114	114	114	114	122	127	133	123	109	120	120	120	120	120
78	129	125	128	123	123	123	123	108	105	138	128	120	105	105	105	105	100	140	125	125	91	91	91	91	91	91	91	91	91	91	91	91	91	91	91	91	91	91	91	91	91	125	121	120	115	115	128	128	128	128	128
128	100	128	117	112	112	112	112	124	126	114	117	119	132	132	132	132	127	126	124	95	116	117	116	116	116	116	116	116	116	116	116	116	116	116	116	116	116	116	116	116	116	114	126	128	121	129	100	100	100	100	100
111	117	131	103	95	95	95	95	105	116	143	113	110	115	115	115	115	103	144	125	109	79	79	79	79	79	79	79	79	79	79	79	79	79	79	79	79	79	79	79	79	79	116	131	135	114	117	117	117	117	117	117
95	120	115	118	113	113	113	113	110	111	142	120	114	118	118	118	118	108	146	124	122	87	88	87	87	87	87	87	87	87	87	87	87	87	87	87	87	87	87	87	87	87	123	125	127	118	107	120	120	120	120	120
119	123	130	96	105	105	105	105	121	129	120	118	104	121	121	121	121	120	131	126	109	115	116	115	115	115	115	115	115	115	115	115	115	115	115	115	115	115	115	115	115	115	109	129	134	109	119	123	123	123	123	123
94	114	123	113	98	98	98	98	98	107	134	112	122	109	109	109	109	101	137	125	112	100	101	100	100	100	100	100	100	100	100	100	100	100	100	100	100	100	100	100	100	100	114	127	128	97	119	114	114	114	114	114
105	120	129	124	117	117	117	117	112	124	139	122	122	119	119	119	119	114	134	125	124	99	100	99	99	99	99	99	99	99	99	99	99	99	99	99	99	99	99	99	99	99	127	128	130	121	107	120	120	120	120	120
84	127	131	129	118	118	118	118	101	115	145	119	128	85	85	85	85	96	146	130	123	113	113	113	113	113	113	113	113	113	113	113	113	113	113	113	113	113	113	113	113	113	123	115	118	119	93	127	127	127	127	127
100	123	128	120	113	113	113	113	108	123	143	120	128	102	102	102	102	101	141	127	112	89	89	89	89	89	89	89	89	89	89	89	89	89	89	89	89	89	89	89	89	89	125	122	123	120	110	123	123	123	123	123
91	133	122	128	127	127	127	127	109	121	141	128	135	101	101	101	101	101	136	122	126	97	97	97	97	97	97	97	97	97	97	97	97	97	97	97	97	97	97	97	97	97	130	118	120	118	105	133	133	133	133	133
117	114	112	117	104	104	104	104	103	94	128	122	123	113	113	113	113	99	138	120	100	89	89	89	89	89	89	89	89	89	89	89	89	89	89	89	89	89	89	89	89	89	113	115	117	117	116	114	114	114	114	114
95	122	141	126	117	117	117	117	102	114	139	118	120	98	98	98	98	102	139	134	130	107	108	107	107	107	107	107	107	107	107	107	107	107	107	107	107	107	107	107	107	107	126	121	123	122	107	122	122	122	122	122
82	129	109	123	122	122	122	122	103	106	144	133	114	110	110	110	110	102	145	115	128	73	74	73	73	73	73	73	73	73	73	73	73	73	73	73	73	73	73	73	73	73	123	113	115	104	101	128	128	128	128	128
141	133	142	132	134	134	134	134	145	145	90	133	141	153	153	153	153	144	66	139	136	142	142	142	142	142	142	142	142	142	142	142	142	142	142	142	142	142	142	142	142	142	137	145	145	142	146	133	133	133	133	133
84	124	127	117	109	109	109	109	105	111	144	117	126	107	107	107	107	90	143	133	114	82	83	82	82	82	82	82	82	82	82	82	82	82	82	82	82	82	82	82	82	82	132	121	122	112	106	124	124	124	124	124
86	133	118	120	119	119	119	119	111	108	146	138	121	108	108	108	108	98	148	122	121	82	83	82	82	82	82	82	82	82	82	82	82	82	82	82	82	82	82	82	82	82	123	119	119	117	101	133	133	133	133	133
90	126	108	122	122	122	122	122	113	112	147	118	120	107	107	107	107	100	137	113	119	80	80	80	80	80	80	80	80	80	80	80	80	80	80	80	80	80	80	80	80	80	121	119	117	123	108	126	126	126	126	126
75	136	120	127	128	128	128	128	106	119	150	138	135	69	69	69	69	94	148	119	138	111	111	111	111	111	111	111	111	111	111	111	111	111	111	111	111	111	111	111	111	111	126	112	111	120	96	136	136	136	136	136
78	124	124	127	123	123	123	123	102	117	144	121	135	105	105	105	105	95	135	124	123	95	96	95	95	95	95	95	95	95	95	95	95	95	95	95	95	95	95	95	95	95	126	116	121	118	108	124	124	124	124	124
97	120	123	120	109	109	109	109	107	112	146	112	120	109	109	109	109	107	149	132	119	84	84	84	84	84	84	84	84	84	84	84	84	84	84	84	84	84	84	84	84	84	126	120	121	122	103	119	119	119	119	119
99	136	122	121	115	115	115	115	110	117	142	128	118	103	103	103	103	90	148	122	130	103	104	103	103	103	103	103	103	103	103	103	103	103	103	103	103	103	103	103	103	103	122	117	120	104	101	136	136	136	136	136
106	114	118	117	111	111	111	111	112	115	142	103	110	117	117	117	117	99	140	120	101	78	79	78	78	78	78	78	78	78	78	78	78	78	78	78	78	78	78	78	78	78	125	117	120	118	113	114	114	114	115	114
72	127	113	118	116	116	116	116	105	121	142	125	126	100	100	100	100	104	146	112	125	110	111	110	110	110	110	110	110	110	110	110	110	110	110	110	110	110	110	110	110	110	122	114	115	113	94	127	127	127	127	127
108	112	125	99	95	95	95	95	108	118	139	113	118	121	121	121	121	110	138	135	104	92	93	92	92	92	92	92	92	92	92	92	92	92	92	92	92	92	92	92	92	92	110	133	135	107	110	112	112	112	112	112
90	123	120	124	121	121	121	121	116	122	142	122	123	112	112	112	112	109	140	121	122	91	92	91	91	91	91	91	91	91	91	91	91	91	91	91	91	91	91	91	91	91	128	116	120	122	112	122	122	122	122	122
102	125	122	128	118	118	118	118	105	127	137	119	118	106	106	106	106	110	142	123	123	97	98	97	97	97	97	97	97	97	97	97	97	97	97	97	97	97	97	97	97	97	120	121	115	116	91	124	124	124	124	124
64	127	122	125	119	119	119	119	110	122	140	123	123	104	104	104	104	106	142	120	127	114	114	114	114	114	114	114	114	114	114	114	114	114	114	114	114	114	114	114	114	114	127	113	116	124	101	127	127	127	127	127
100	125	127	116	112	112	112	112	105	109	140	124	123	104	104	104	104	102	145	128	119	86	87	86	86	86	86	86	86	86	86	86	86	86	86	86	86	86	86	86	86	86	117	129	126	112	104	125	125	125	125	125
94	127	117	124	124	124	124	124	108	121	141	120	112	108	108	108	108	111	143	110	124	94	95	94	94	94	94	94	94	94	94	94	94	94	94	94	94	94	94	94	94	94	126	110	110	117	94	126	126	126	127	126
111	120	124	101	104	104	104	104	119	125	141	113	111	126	126	126	126	110	142	134	101	98	99	98	98	98	98	98	98	98	98	98	98	98	98	98	98	98	98	98	98	98	117	131	132	105	115	120	120	120	120	120
106	117	134	111	105	105	105	105	109	109	132	116	113	108	108	108	108	100	144	129	107	92	93	92	92	92	92	92	92	92	92	92	92	92	92	92	92	92	92	92	92	92	113	127	130	112	113	117	117	117	117	117
109	114	132	97	96	96	96	96	118	112	137	110	112	109	109	109	109	109	151	134	100	105	106	105	105	105	105	105	105	105	105	105	105	105	105	105	105	105	105	105	105	105	109	132	129	103	114	114	114	114	114	114
101	114	126	123	110	110	110	110	111	121	137	123	130	105	105	105	105	107	137	126	111	106	107	106	106	106	106	106	106	106	106	106	106	106	106	106	106	106	106	106	106	106	116	125	129	121	117	114	114	114	114	114
91	116	127	123	122	122	122	122	128	106	132	128	118	109	109	109	109	113	145	123	125	113	113	113	113	113	113	113	113	113	113	113	113	113	113	113	113	113	113	113	113	113	115	122	128	126	111	116	116	116	116	116
104	131	125	123	117	117	117	117	101	114	141	131	125	99	99	99	99	96	147	119	125	97	97	97	97	97	97	97	97	97	97	97	97	97	97	97	97	97	97	97	97	97	121	118	115	106	92	130	130	130	130	130
99	134	127	128	125	125	125	125	102	122	144	126	129	83	83	83	83	99	149	133	132	115	116	115	115	115	115	115	115	115	115	115	115	115	115	115	115	115	115	115	115	115	129	118	118	124	99	134	134	134	134	134
103	114	129	113	115	115	115	115	102	115	134	118	133	101	101	101	101	114	139	118	118	106	106	106	106	106	106	106	106	106	106	106	106	106	106	106	106	106	106	106	106	106	115	126	126	113	111	114	114	114	114	114
96	121	120	123	114	114	114	114	120	127	134	118	129	108	108	108	108	114	137	126	124	110	111	110	110	110	110	110	110	110	110	110	110	110	110	110	110	110	110	110	110	110	123	124	125	124	101	121	121	121	121	121
105	120	133	129	127	127	127	127	97	129	141	121	117	108	108	108	108	106	145	121	125	98	98	98	98	98	98	98	98	98	98	98	98	98	98	98	98	98	98	98	98	98	123	119	116	116	105	120	120	120	121	120
105	122	131	119	107	107	107	107	117	111	138	125	111	99	99	99	99	104	152	123	119	114	114	114	114	114	114	114	114	114	114	114	114	114	114	114	114	114	114	114	114	114	126	127	128	107	113	122	122	122	122	122
81	131	122	117	117	117	117	117	108	111	146	133	128	100	100	100	100	92	142	126	120	94	95	94	94	94	94	94	94	94	94	94	94	94	94	94	94	94	94	94	94	94	124	119	122	109	104	131	131	131	131	131
84	131	116	123	116	116	116	116	110	115	140	127	123	102	102	102	102	96	144	120	122	103	104	103	103	103	103	103	103	103	103	103	103	103	103	103	103	103	103	103	103	103	123	124	121	114	104	131	131	131	131	131
96	119	132	113	108	108	108	108	117	118	146	115	132	104	104	104	104	97	147	126	110	93	94	93	93	93	93	93	93	93	93	93	93	93	93	93	93	93	93	93	93	93	116	127	128	111	119	119	119	119	119	119
122	120	142	104	103	103	103	103	123	113	118	113	106	123	123	123	123	119	133	141	108	116	117	116	116	116	116	116	116	116	116	116	116	116	116	116	116	116	116	116	116	116	117	140	138	111	130	120	120	120	120	120
111	99	130	95	96	96	96	96	109	108	130	91	101	123	123	123	123	110	139	131	98	83	84	83	83	83	83	83	83	83	83	83	83	83	83	83	83	83	83	83	83	83	106	128	129	106	118	99	99	99	99	99
107	123	125	108	102	102	102	102	102	115	130	126	125	117	117	117	117	111	137	127	116	76	77	76	76	76	76	76	76	76	76	76	76	76	76	76	76	76	76	76	76	76	118	129	125	106	102	123	123	123	123	123
111	120	121	111	108	108	108	108	112	110	136	116	120	124	124	124	124	114	142	129	109	66	66	66	66	66	66	66	66	66	66	66	66	66	66	66	66	66	66	66	66	66	124	128	131	111	111	120	120	120	121	120
95	117	123	127	123	123	123	123	109	115	143	136	124	99	99	99	99	85	148	123	126	101	102	101	101	101	101	101	101	101	101	101	101	101	101	101	101	101	101	101	101	101	126	120	122	120	92	117	117	117	118	117
121	117	128	123	117	117	117	117	124	121	128	114	129	131	131	131	131	118	132	122	110	111	111	111	111	111	111	111	111	111	111	111	111	111	111	111	111	111	111	111	111	111	124	119	126	119	123	117	117	117	117	117
102	113	123	116	106	106	106	106	127	104	128	119	119	122	122	122	122	111	134	128	108	112	113	112	112	112	112	112	112	112	112	112	112	112	112	112	112	112	112	112	112	112	110	123	128	118	122	113	113	113	113	113
118	105	131	114	98	98	98	98	115	111	120	112	114	120	120	120	120	114	135	124	106	112	112	112	112	112	112	112	112	112	112	112	112	112	112	112	112	112	112	112	112	112	120	125	126	118	127	105	105	105	105	105
97	131	127	109	106	106	106	106	109	105	131	111	125	105	105	105	105	101	133	134	109	88	89	88	88	88	88	88	88	88	88	88	88	88	88	88	88	88	88	88	88	88	119	122	121	108	114	131	131	131	131	131
92	111	132	124	112	112	112	112	102	111	143	112	124	85	85	85	85	94	146	128	118	125	126	125	125	125	125	125	125	125	125	125	125	125	125	125	125	125	125	125	125	125	121	117	120	105	95	111	111	111	112	111
105	119	120	99	99	99	99	99	119	104	141	112	117	112	112	112	112	114	152	135	104	97	98	97	97	97	97	97	97	97	97	97	97	97	97	97	97	97	97	97	97	97	117	128	127	91	110	119	119	119	119	119
105	122	121	94	101	101	101	101	118	121	135	118	115	123	123	123	123	117	132	134	109	88	89	88	88	88	88	88	88	88	88	88	88	88	88	88	88	88	88	88	88	88	118	135	131	99	125	122	122	122	122	122
99	115	127	90	91	91	91	91	121	114	131	113	107	123	123	123	123	112	130	135	98	100	101	100	100	100	100	100	100	100	100	100	100	100	100	100	100	100	100	100	100	100	110	135	139	96	127	115	115	115	115	115
106	117	120	91	98	98	98	98	122	114	129	112	118	121	121	121	121	114	132	127	104	104	105	104	104	104	104	104	104	104	104	104	104	104	104	104	104	104	104	104	104	104	113	129	132	87	123	117	117	117	117	117
88	127	129	121	113	113	113	113	113	107	137	121	122	100	100	100	100	90	142	121	121	109	109	109	109	109	109	109	109	109	109	109	109	109	109	109	109	109	109	109	109	109	124	119	114	114	106	127	127	127	127	127
130	117	129	111	109	109	109	109	136	131	92	111	120	143	143	143	143	137	94	135	114	130	131	130	130	130	130	130	130	130	130	130	130	130	130	130	130	130	130	130	130	130	120	144	142	124	143	117	117	117	117	117
102	107	129	99	91	91	91	91	118	122	129	103	107	128	128	128	128	118	133	129	106	104	105	104	104	104	104	104	104	104	104	104	104	104	104	104	104	104	104	104	104	104	108	138	138	108	126	107	107	107	107	107
103	118	128	121	116	116	116	116	111	121	139	120	124	104	104	104	104	100	141	122	113	92	92	92	92	92	92	92	92	92	92	92	92	92	92	92	92	92	92	92	92	92	121	127	127	120	107	117	117	117	117	117
102	115	124	103	100	100	100	100	102	102	139	115	104	123	123	123	123	112	145	124	105	66	67	66	66	66	66	66	66	66	66	66	66	66	66	66	66	66	66	66	66	66	114	128	130	94	106	115	115	115	115	115
96	125	126	125	114	114	114	114	102	120	136	116	127	94	94	94	94	109	140	135	125	119	119	119	119	119	119	119	119	119	119	119	119	119	119	119	119	119	119	119	119	119	121	120	123	114	100	125	125	125	125	125
103	131	135	126	121	121	121	121	96	118	143	112	126	95	95	95	95	110	149	129	121	101	101	101	101	101	101	101	101	101	101	101	101	101	101	101	101	101	101	101	101	101	124	118	117	109	101	130	130	130	130	130
89	126	111	109	110	110	110	110	96	112	144	126	117	117	117	117	117	101	140	122	135	75	76	75	75	75	75	75	75	75	75	75	75	75	75	75	75	75	75	75	75	75	122	125	125	107	108	126	126	126	126	126
97	127	128	117	107	107	107	107	103	116	133	123	125	114	114	114	114	94	133	132	122	90	91	90	90	90	90	90	90	90	90	90	90	90	90	90	90	90	90	90	90	90	112	130	132	110	116	127	127	127	127	127
96	126	121	119	112	112	112	112	93	113	144	125	122	92	92	92	92	89	144	122	125	95	95	95	95	95	95	95	95	95	95	95	95	95	95	95	95	95	95	95	95	95	121	121	123	109	103	126	126	126	126	126
88	124	124	124	122	122	122	122	109	114	139	125	123	108	108	108	108	104	142	121	126	90	90	90	90	90	90	90	90	90	90	90	90	90	90	90	90	90	90	90	90	90	125	117	121	125	107	123	123	123	123	123
103	114	128	110	110	110	110	110	116	115	143	118	116	104	104	104	104	105	141	130	111	98	98	98	98	98	98	98	98	98	98	98	98	98	98	98	98	98	98	98	98	98	123	130	130	108	124	114	114	114	114	114
137	111	132	99	93	93	93	93	127	129	107	103	103	143	143	143	143	138	109	135	96	117	117	117	117	117	117	117	117	117	117	117	117	117	117	117	117	117	117	117	117	117	117	147	147	98	142	111	111	111	111	111

96	120	128	124	121	121	121	121	110	120	137	118	121	89	89	89	89	100	147	127	124	116	117	116	116	116	116	116	116	116	116	116	116	116	116	116	116	116	116	116	116	116	120	114	117	117	94	119	119	119	119	119
82	118	129	129	109	109	109	109	111	116	138	126	129	74	74	74	74	89	139	119	122	118	119	118	118	118	118	118	118	118	118	118	118	118	118	118	118	118	118	118	118	118	124	114	114	125	113	118	118	118	118	118
141	117	130	108	104	104	104	104	129	134	104	120	120	145	145	145	145	142	104	133	111	126	126	126	126	126	126	126	126	126	126	126	126	126	126	126	126	126	126	126	126	126	125	148	147	116	147	117	117	117	117	117
131	111	139	103	104	104	104	104	129	132	102	116	110	142	142	142	142	139	106	134	118	121	121	121	121	121	121	121	121	121	121	121	121	121	121	121	121	121	121	121	121	121	119	146	145	110	143	111	111	111	111	111
73	123	113	109	105	105	105	105	119	119	139	123	114	104	104	104	104	101	144	130	123	94	94	94	94	94	94	94	94	94	94	94	94	94	94	94	94	94	94	94	94	94	117	118	124	120	106	122	122	122	122	122
97	127	123	113	112	112	112	112	110	118	143	123	124	113	113	113	113	104	139	128	120	88	88	88	88	88	88	88	88	88	88	88	88	88	88	88	88	88	88	88	88	88	117	129	132	97	119	127	127	127	127	127
106	129	130	122	112	112	112	112	118	124	139	130	132	110	110	110	110	99	133	132	123	120	121	120	120	120	120	120	120	120	120	120	120	120	120	120	120	120	120	120	120	120	120	133	132	118	121	129	129	129	129	129
107	113	128	109	105	105	105	105	121	110	140	114	127	115	115	115	115	112	143	124	108	103	104	103	103	103	103	103	103	103	103	103	103	103	103	103	103	103	103	103	103	103	116	129	129	113	124	113	113	113	113	113
95	123	134	128	110	110	110	110	110	121	144	122	130	108	108	108	108	96	138	134	127	102	102	102	102	102	102	102	102	102	102	102	102	102	102	102	102	102	102	102	102	102	128	126	129	125	119	123	123	123	123	123
119	107	115	98	94	94	94	94	124	110	115	97	99	138	138	138	138	128	132	130	95	109	110	109	109	109	109	109	109	109	109	109	109	109	109	109	109	109	109	109	109	109	107	139	140	101	131	107	107	107	107	107
84	122	116	109	110	110	110	110	108	118	148	126	126	107	107	107	107	85	143	129	118	84	85	84	84	84	84	84	84	84	84	84	84	84	84	84	84	84	84	84	84	84	119	123	126	114	111	122	122	122	122	122
92	125	127	121	120	120	120	120	107	121	143	125	116	105	105	105	105	98	145	129	130	93	94	93	93	93	93	93	93	93	93	93	93	93	93	93	93	93	93	93	93	93	124	120	126	120	104	125	125	125	125	125
97	129	129	132	122	122	122	122	102	132	148	127	141	89	89	89	89	97	143	121	125	115	116	115	115	115	115	115	115	115	115	115	115	115	115	115	115	115	115	115	115	115	129	119	117	120	91	129	129	129	129	129

1	1	2	1	20	20	20	20	20	20	20	20	20	20	20	20	20	20	20	20	20	20	20	20	4	7	7	7	4	4	1	7	7	7	4	7	2	2	4	4	4	4	1	1	1	3	8	2	1	8	8	8
Sudachi	Suisho buntan	Sunki	Suruga Yuko	Sweet orange	Sweet orange	Sweet orange	Sweet orange	Sweet orange	Sweet orange	Sweet orange	Sweet orange	Sweet orange	Sweet orange	Sweet orange	Sweet orange	Sweet orange	Sweet orange	Sweet orange	Sweet orange	Sweet orange	Sweet orange	Sweet orange	Sweet orange	Tachibana	Tachibana	Tachibana	Tachibana	Tachibana-A	Tachibana	Tachibana-C	Tachibana-B	Tachibana	Tachibana	Tachibana	Tachibana	Tajima mikan	Takumanatsukunenbo	Tankan	Tankan	Tankan	Tankan	Temple	Tengu	Tizon	Toukan	Tookunin	Tosa buntan	Twukkuni	Twukkunin	Twukunihu	Twuukuribu
A144	A145	A146	A147	A148	A149	A150	A151	A152	A153	A154	A155	A156	A157	A158	A159	A160	A161	A162	A163	A164	A165	A166	A167	A168	A169	A170	A171	A172	A173	A174	A175	A176	A177	A178	A179	A180	A181	A182	A183	A184	A185	A186	A187	A188	A189	A190	A191	A192	A193	A194	A195
0	0	0	0	0	2	0	0	0	0	0	0	0	0	0	0	0	0	0	0	0	0	0	0	1	0	1	0	1	1	0	0	0	0	1	0	0	0	0	0	0	0	0	0	0	0	2	0	1	0	0	0
127	123	130	131	103	104	103	103	103	103	103	103	103	103	103	103	103	103	103	103	103	103	103	103	141	141	141	141	141	141	139	141	141	141	141	141	121	105	116	116	116	116	115	102	119	130	89	119	133	87	87	87
136	90	149	146	134	134	134	134	134	134	134	134	134	134	134	134	134	134	134	134	134	134	134	134	145	148	148	148	145	145	144	148	148	148	145	148	142	98	137	137	137	137	133	110	142	141	137	89	138	137	137	137
127	115	141	139	102	104	102	102	102	102	102	102	102	102	102	102	102	102	102	102	102	102	102	102	149	146	146	146	149	149	144	146	146	146	149	146	128	94	126	126	126	126	115	109	122	130	97	93	137	95	95	95
146	74	148	151	131	132	131	131	131	131	131	131	131	131	131	131	131	131	131	131	131	131	131	130	149	145	145	145	149	149	147	145	145	145	149	145	142	108	141	141	141	141	134	129	139	143	139	97	143	139	139	139
116	142	90	99	118	118	118	118	118	118	118	118	118	118	118	118	118	118	118	118	118	118	118	117	114	112	113	112	114	114	105	112	112	112	114	112	111	131	111	111	111	111	118	124	103	112	111	139	114	110	110	110
131	134	129	132	133	135	133	133	133	133	133	133	133	133	133	133	133	133	133	133	133	133	133	133	127	130	130	130	127	127	130	130	130	130	127	130	138	111	138	138	138	138	122	136	121	131	136	132	126	135	135	135
129	134	123	124	90	91	90	90	90	90	90	90	90	90	90	90	90	90	90	90	90	90	90	89	141	138	138	138	141	141	133	138	138	138	141	138	120	122	114	114	114	114	105	110	105	123	2	134	115	0	0	0
126	135	120	117	117	118	117	117	117	117	117	117	117	117	117	117	117	117	117	117	117	117	117	116	129	132	133	132	129	129	127	132	132	132	129	132	106	126	108	108	108	108	105	127	121	112	110	136	119	109	109	109
122	142	92	117	101	103	101	101	101	101	101	101	101	101	101	101	101	101	101	101	101	101	101	100	124	119	119	119	124	124	115	119	119	119	124	119	122	128	95	95	95	95	82	119	99	119	119	143	118	118	118	118
122	142	92	117	101	103	101	101	101	101	101	101	101	101	101	101	101	101	101	101	101	101	101	100	124	119	119	119	124	124	115	119	119	119	124	119	122	128	95	95	95	95	82	119	99	119	119	143	118	118	118	118
122	142	92	117	101	103	101	101	101	101	101	101	101	101	101	101	101	101	101	101	101	101	101	100	124	119	119	119	124	124	115	119	119	119	124	119	122	128	95	95	95	95	82	119	99	119	119	143	118	118	118	118
122	142	92	117	101	103	101	101	101	101	101	101	101	101	101	101	101	101	101	101	101	101	101	100	124	119	119	119	124	124	115	119	119	119	124	119	122	128	95	95	95	95	82	119	99	119	119	143	118	118	118	118
125	141	72	94	133	133	133	133	133	133	133	133	133	133	133	133	133	133	133	133	133	133	133	133	100	95	96	95	100	100	90	95	95	95	100	95	112	135	111	111	111	111	112	130	90	105	127	139	98	127	127	127
128	148	94	118	94	95	94	94	94	94	94	94	94	94	94	94	94	94	94	94	94	94	94	93	121	120	120	120	121	121	116	120	120	120	121	120	123	134	94	94	94	94	102	105	97	124	115	146	129	114	114	114
131	125	137	132	101	102	101	101	101	101	101	101	101	101	101	101	101	101	101	101	101	101	101	101	135	137	137	137	135	135	136	137	137	137	135	137	128	112	127	127	127	127	109	116	115	129	116	119	122	114	114	114
126	147	68	105	115	115	115	115	115	115	115	115	115	115	115	115	115	115	115	115	115	115	115	114	116	116	116	116	116	116	105	116	116	116	116	116	117	130	94	94	94	94	104	124	102	112	120	147	108	119	119	119
126	147	68	105	115	115	115	115	115	115	115	115	115	115	115	115	115	115	115	115	115	115	115	114	116	116	116	116	116	116	105	116	116	116	116	116	117	130	94	94	94	94	104	124	102	112	120	147	108	119	119	119
148	76	148	149	147	147	147	147	147	147	147	147	147	147	147	147	147	147	147	147	147	147	147	148	146	146	146	146	146	146	148	146	146	146	146	146	152	118	145	145	145	145	144	125	141	150	143	96	141	143	143	143
122	147	86	79	122	123	122	122	122	122	122	123	122	122	122	122	122	122	122	122	122	122	122	122	100	91	91	91	100	100	92	91	91	91	100	91	102	137	118	118	118	118	121	133	117	79	124	139	107	124	124	124
126	137	121	120	124	124	124	124	124	124	124	124	124	124	124	124	124	124	124	124	124	124	124	124	103	102	102	102	103	103	100	102	102	102	103	102	96	130	124	124	124	124	117	139	118	112	130	138	97	128	128	128
132	118	126	124	101	101	101	101	101	101	101	101	101	101	101	101	101	101	101	101	101	101	101	102	138	139	140	139	138	138	133	139	139	139	138	139	132	111	116	116	116	116	114	98	116	127	99	116	123	97	97	97
133	141	77	100	124	124	124	124	124	124	124	124	124	124	124	124	124	124	124	124	124	124	124	124	104	100	101	100	104	104	102	100	100	100	104	100	111	135	96	96	96	96	101	120	108	112	126	144	110	125	125	125
115	140	108	112	128	128	128	128	128	128	128	128	128	128	128	128	128	128	128	128	128	128	128	128	101	92	93	92	101	101	79	92	92	92	101	92	0	133	116	116	116	116	114	135	115	75	121	138	94	120	120	120
138	111	136	138	98	98	98	98	98	98	98	98	98	98	98	98	98	98	98	98	98	98	98	97	146	142	142	142	146	146	148	142	142	142	146	142	128	111	119	119	119	119	122	85	129	130	112	103	139	111	111	111
138	111	136	138	98	98	98	98	98	98	98	98	98	98	98	98	98	98	98	98	98	98	98	97	146	142	142	142	146	146	148	142	142	142	146	142	128	111	119	119	119	119	122	85	129	130	112	103	139	111	111	111
138	111	136	138	98	98	98	98	98	98	98	98	98	98	98	98	98	98	98	98	98	98	98	97	146	142	142	142	146	146	148	142	142	142	146	142	128	111	119	119	119	119	122	85	129	130	112	103	139	111	111	111
103	148	123	128	140	141	140	140	140	140	140	140	140	140	140	140	140	140	140	140	140	140	140	140	112	107	107	107	112	112	114	107	107	107	112	107	114	142	133	133	133	133	138	148	133	121	137	148	117	137	137	137
134	122	142	145	97	98	97	97	97	97	97	97	97	97	97	97	97	97	97	97	97	97	97	98	143	143	143	143	143	143	144	143	143	143	143	143	130	104	126	126	126	126	120	96	126	133	95	111	134	93	93	93
77	143	127	128	129	129	129	129	129	129	129	129	129	129	129	129	129	129	129	129	129	129	129	128	134	134	134	134	134	134	133	134	134	134	134	134	126	128	125	125	125	125	131	124	126	128	119	133	125	119	119	119
112	143	124	123	131	132	131	131	131	131	131	131	131	131	131	131	131	131	131	131	131	131	131	131	120	120	121	120	120	120	123	120	120	120	120	120	120	136	119	119	119	119	124	127	131	131	119	139	115	118	118	118
128	147	81	100	123	123	123	123	123	123	123	123	123	123	123	123	123	123	123	123	123	123	123	122	111	110	110	110	111	111	107	110	110	110	111	110	122	134	86	86	86	86	98	130	99	111	118	150	112	116	116	116
147	66	150	147	140	141	140	140	140	140	140	140	140	140	140	140	140	140	140	140	140	140	140	139	148	149	149	149	148	148	147	149	149	149	148	149	147	103	146	146	146	146	139	129	140	147	138	94	138	138	138	138
124	110	137	138	110	112	110	110	110	110	110	110	110	110	110	110	110	110	110	110	110	110	110	109	142	144	144	144	142	142	144	144	144	144	142	144	133	0	124	124	124	124	114	116	119	128	123	106	135	122	122	122
128	125	120	122	101	102	101	101	101	101	101	101	101	101	101	101	101	101	101	101	101	101	101	101	139	139	139	139	139	139	135	139	139	139	139	139	128	106	111	111	111	111	117	115	112	129	62	123	117	60	60	60
131	111	141	142	121	121	121	121	121	121	121	121	121	121	121	121	121	121	121	121	121	121	121	121	140	142	142	142	140	140	142	142	142	142	140	142	134	105	132	132	132	132	125	93	129	132	100	101	134	100	100	100
136	136	136	127	110	111	110	110	110	110	110	110	110	110	110	110	110	110	110	110	110	110	110	109	128	128	128	128	128	128	136	128	128	128	128	128	121	129	120	120	120	120	117	110	112	122	95	128	117	94	94	94
136	136	136	127	110	111	110	110	110	110	110	110	110	110	110	110	110	110	110	110	110	110	110	109	128	128	128	128	128	128	136	128	128	128	128	128	121	129	120	120	120	120	117	110	112	122	95	128	117	94	94	94
136	136	136	127	110	111	110	110	110	110	110	110	110	110	110	110	110	110	110	110	110	110	110	109	128	128	128	128	128	128	136	128	128	128	128	128	121	129	120	120	120	120	117	110	112	122	95	128	117	94	94	94
136	137	136	127	110	111	110	110	110	110	110	110	110	110	110	110	110	110	110	110	110	110	110	109	128	128	128	128	128	128	136	128	128	128	128	128	122	129	120	120	120	120	117	111	112	122	95	129	118	94	94	94
136	136	136	127	110	111	110	110	110	110	110	110	110	110	110	110	110	110	110	110	110	110	110	109	128	128	128	128	128	128	136	128	128	128	128	128	121	129	120	120	120	120	117	110	112	122	95	128	117	94	94	94
122	142	134	137	134	136	134	134	134	134	134	134	134	134	134	134	134	134	134	134	134	134	134	134	132	127	127	127	132	132	133	127	127	127	132	127	130	126	141	141	141	141	138	139	133	132	140	135	133	140	140	140
121	130	143	148	127	127	127	127	127	127	127	127	127	127	127	127	127	127	127	127	127	127	127	126	143	142	142	142	143	143	143	142	142	142	143	142	130	115	139	139	139	139	133	131	134	132	130	127	137	129	129	129
134	130	124	126	98	99	98	98	98	98	98	98	98	98	98	98	98	98	98	98	98	98	98	98	142	142	142	142	142	142	136	142	142	142	142	142	133	113	115	115	115	115	115	93	128	130	94	125	135	93	93	93
134	130	124	124	98	99	98	98	98	98	98	98	98	98	98	98	98	98	98	98	98	98	98	98	142	141	141	141	142	142	135	141	141	141	142	141	131	112	115	115	115	115	115	93	128	128	93	125	135	92	92	92
108	143	130	131	131	131	131	131	131	131	131	131	131	131	131	131	131	131	131	131	131	131	131	131	131	135	135	135	131	131	136	135	135	135	131	135	126	128	137	137	137	137	130	137	138	136	123	142	133	123	123	123
122	126	132	134	126	126	126	126	126	126	126	126	126	126	126	126	126	126	126	126	126	126	126	126	132	130	131	130	132	132	132	130	130	130	132	130	121	118	123	123	123	123	129	120	125	133	116	122	121	115	115	115
79	142	126	117	127	128	127	127	127	127	127	127	127	127	127	127	127	127	127	127	127	127	127	126	130	128	128	128	130	130	127	128	128	128	130	128	122	126	126	126	126	126	116	133	111	124	116	128	128	116	116	116
137	147	117	102	129	130	129	129	129	129	129	129	129	129	129	129	129	129	129	129	129	129	129	128	112	107	108	107	112	112	110	107	107	107	112	107	107	138	128	128	128	128	126	131	129	95	104	146	107	104	104	104
137	100	143	148	122	122	122	122	122	122	122	122	122	122	122	122	122	122	122	122	122	122	122	121	148	149	149	149	148	148	149	149	149	149	148	149	133	97	131	131	131	131	123	123	130	137	112	86	142	111	111	111
132	127	125	123	116	117	116	116	116	116	116	116	116	116	116	116	116	116	116	116	116	116	116	115	123	117	118	117	123	123	121	117	117	117	123	117	118	130	124	124	124	124	121	110	118	118	95	129	124	94	94	94
134	93	136	140	126	126	126	126	126	126	126	126	126	126	126	126	126	126	126	126	126	126	126	125	144	146	146	146	144	144	142	146	146	146	144	146	135	100	128	128	128	128	124	109	129	136	118	90	134	117	117	117
125	135	119	123	128	128	128	128	128	128	128	128	128	128	128	128	128	128	128	128	128	128	128	127	113	110	111	110	113	113	106	110	110	110	113	110	96	135	119	119	119	119	127	122	122	113	85	140	101	83	83	83
128	137	136	128	123	124	123	123	123	123	123	123	123	123	123	123	123	123	123	123	123	123	123	122	130	129	129	129	130	130	136	129	129	129	130	129	133	128	137	137	137	137	128	135	138	124	124	137	137	124	124	124
129	136	103	125	110	111	110	110	110	110	110	110	110	110	110	110	110	110	110	110	110	110	110	109	121	122	122	122	121	121	122	122	122	122	121	122	115	121	99	99	99	99	109	111	104	127	101	137	107	99	99	99
130	102	139	142	110	111	110	110	110	110	110	110	110	110	110	110	110	110	110	110	110	110	110	109	147	145	145	145	147	147	144	145	145	145	147	145	137	110	129	129	129	129	121	101	121	135	100	90	130	98	98	98
127	123	130	132	105	106	105	105	105	105	105	105	105	105	105	105	105	105	105	105	105	105	105	105	141	141	141	141	141	141	139	141	141	141	141	141	121	105	116	116	116	116	116	104	119	131	91	119	133	89	89	89

124	149	75	77	128	128	128	128	128	128	128	128	128	128	128	128	128	128	128	128	128	128	128	128	105	109	109	109	105	105	103	109	109	109	105	109	111	136	111	111	111	111	114	118	105	108	110	146	108	110	110	110
124	149	76	78	128	128	128	128	128	128	128	128	128	128	128	128	128	128	128	128	128	128	128	128	106	110	110	110	106	106	104	110	110	110	106	110	112	136	111	111	111	111	114	118	105	108	110	146	109	110	110	110
124	149	75	77	128	128	128	128	128	128	128	128	128	128	128	128	128	128	128	128	128	128	128	128	105	109	109	109	105	105	103	109	109	109	105	109	111	136	111	111	111	111	114	118	105	108	110	146	108	110	110	110
124	149	75	77	128	128	128	128	128	128	128	128	128	128	128	128	128	128	128	128	128	128	128	128	105	109	109	109	105	105	103	109	109	109	105	109	111	136	111	111	111	111	114	118	105	108	110	146	108	110	110	110
124	149	75	77	128	128	128	128	128	128	128	128	128	128	128	128	128	128	128	128	128	128	128	128	105	109	109	109	105	105	103	109	109	109	105	109	111	136	111	111	111	111	114	118	105	108	110	146	108	110	110	110
124	149	75	77	128	128	128	128	128	128	128	128	128	128	128	128	128	128	128	128	128	128	128	128	105	109	109	109	105	105	103	109	109	109	105	109	111	136	111	111	111	111	114	118	105	108	110	146	108	110	110	110
124	149	75	77	128	128	128	128	128	128	128	128	128	128	128	128	128	128	128	128	128	128	128	128	105	109	109	109	105	105	103	109	109	109	105	109	111	136	111	111	111	111	114	118	105	108	110	146	108	110	110	110
124	149	75	77	128	128	128	128	128	128	128	128	128	128	128	128	128	128	128	128	128	128	128	128	105	109	109	109	105	105	103	109	109	109	105	109	111	136	111	111	111	111	114	118	105	108	110	146	108	110	110	110
124	149	75	77	128	128	128	128	128	128	128	128	128	128	128	128	128	128	128	128	128	128	128	128	105	109	109	109	105	105	103	109	109	109	105	109	111	136	111	111	111	111	114	118	105	108	110	146	108	110	110	110
124	149	75	77	128	128	128	128	128	128	128	128	128	128	128	128	128	128	128	128	128	128	128	128	105	109	109	109	105	105	103	109	109	109	105	109	111	136	111	111	111	111	114	118	105	108	110	146	108	110	110	110
124	149	75	77	128	128	128	128	128	128	128	128	128	128	128	128	128	128	128	128	128	128	128	128	105	109	109	109	105	105	103	109	109	109	105	109	111	136	111	111	111	111	114	118	105	108	110	146	108	110	110	110
124	149	75	77	128	128	128	128	128	128	128	128	128	128	128	128	128	128	128	128	128	128	128	128	105	109	109	109	105	105	103	109	109	109	105	109	111	136	111	111	111	111	114	118	105	108	110	146	108	110	110	110
124	149	75	77	128	128	128	128	128	128	128	128	128	128	128	128	128	128	128	128	128	128	128	128	105	109	109	109	105	105	103	109	109	109	105	109	111	136	111	111	111	111	114	118	105	108	110	146	108	110	110	110
124	149	75	77	128	128	128	128	128	128	128	128	128	128	128	128	128	128	128	128	128	128	128	128	105	109	109	109	105	105	103	109	109	109	105	109	111	136	111	111	111	111	114	118	105	108	110	146	108	110	110	110
124	149	75	77	128	128	128	128	128	128	128	128	128	128	128	128	128	128	128	128	128	128	128	128	105	109	109	109	105	105	103	109	109	109	105	109	111	136	111	111	111	111	114	118	105	108	110	146	108	110	110	110
124	149	75	77	128	128	128	128	128	128	128	128	128	128	128	128	128	128	128	128	128	128	128	128	105	109	109	109	105	105	103	109	109	109	105	109	111	136	111	111	111	111	114	118	105	108	110	146	108	110	110	110
77	143	127	128	129	129	129	129	129	129	129	129	129	129	129	129	129	129	129	129	129	129	129	128	134	134	134	134	134	134	133	134	134	134	134	134	126	128	125	125	125	125	131	124	126	128	119	133	125	119	119	119
125	146	88	99	125	126	125	125	125	125	125	125	125	125	125	125	125	125	125	125	125	125	125	124	108	105	106	105	108	108	105	105	105	105	108	105	111	138	101	101	101	101	108	131	108	114	113	145	115	113	113	113
110	142	102	84	121	121	121	121	121	121	121	122	121	121	121	121	121	121	121	121	121	121	121	120	106	96	97	96	106	106	85	96	96	96	106	96	75	128	121	121	121	121	119	136	112	0	123	138	114	123	123	123
111	142	101	84	122	122	122	122	122	122	122	123	122	122	122	122	122	122	122	122	122	122	122	121	108	98	99	98	108	108	87	98	98	98	108	98	76	128	120	120	120	120	118	136	113	2	123	138	114	123	123	123
122	130	137	126	106	107	106	106	106	106	106	106	106	106	106	106	106	106	106	106	106	106	106	105	138	134	134	134	138	138	129	134	134	134	138	134	133	112	121	121	121	121	112	114	112	127	109	130	126	107	107	107
86	146	119	113	139	140	139	139	139	139	139	139	139	139	139	139	139	139	139	139	139	139	139	139	104	101	101	101	104	104	117	101	101	101	104	101	118	139	131	131	131	131	139	139	124	114	136	139	119	136	136	136
124	133	136	133	94	95	94	94	94	94	94	94	94	94	94	94	94	94	94	94	94	94	94	94	145	148	148	148	145	145	142	148	148	148	145	148	125	107	118	118	118	118	111	101	121	129	103	127	139	102	102	102
129	134	123	124	90	91	90	90	90	90	90	90	90	90	90	90	90	90	90	90	90	90	90	89	141	138	138	138	141	141	133	138	138	138	141	138	120	122	114	114	114	114	105	110	105	123	2	134	115	0	0	0
120	141	113	111	124	125	124	124	124	124	124	124	124	124	124	124	124	124	124	124	124	124	124	124	98	100	100	100	98	98	90	100	100	100	98	100	88	125	123	123	123	123	122	127	108	107	134	139	100	133	133	133
129	134	123	124	90	91	90	90	90	90	90	90	90	90	90	90	90	90	90	90	90	90	90	89	141	138	138	138	141	141	133	138	138	138	141	138	120	122	114	114	114	114	105	111	105	123	3	134	115	1	1	1
129	134	123	124	90	91	90	90	90	90	90	90	90	90	90	90	90	90	90	90	90	90	90	89	141	138	138	138	141	141	133	138	138	138	141	138	120	122	115	115	115	115	105	110	106	123	3	134	115	1	1	1
133	136	127	140	142	143	142	142	142	142	142	142	142	142	142	142	142	142	142	142	142	142	142	142	133	132	132	132	133	133	137	132	132	132	133	132	139	130	143	143	143	143	142	142	133	135	139	136	132	139	139	139
132	131	124	133	132	133	132	132	132	132	132	132	132	132	132	132	132	132	132	132	132	132	132	132	132	132	132	132	132	132	130	132	132	132	132	132	138	118	130	130	130	130	128	132	131	131	129	129	124	128	128	128
126	149	121	131	142	143	142	142	142	142	142	142	142	142	142	142	142	142	142	142	142	142	142	142	132	128	128	128	132	132	127	128	128	128	132	128	136	136	130	130	130	130	140	137	133	132	136	146	128	136	136	136
144	76	142	146	141	142	141	141	141	141	141	141	141	141	141	141	141	141	141	141	141	141	141	142	146	146	146	146	146	146	146	146	146	146	146	146	144	110	147	147	147	147	143	130	144	140	145	84	141	145	145	145
148	145	139	146	155	155	155	155	155	155	155	155	155	155	155	155	155	155	155	155	155	155	155	155	138	139	139	139	138	138	139	139	139	139	138	139	146	145	152	152	152	152	152	151	153	141	154	144	140	154	154	154
134	144	135	139	137	137	137	137	137	137	137	136	137	137	137	137	137	137	137	137	137	137	137	137	137	135	135	135	137	137	138	135	135	135	137	135	134	133	130	130	130	130	141	133	135	145	135	145	136	135	135	135
101	150	129	128	130	131	130	130	130	130	130	130	130	130	130	130	130	130	130	130	130	130	130	129	131	126	126	126	131	131	125	126	126	126	131	126	119	136	123	123	123	123	131	126	131	132	106	142	120	106	106	106
118	139	88	117	108	109	108	108	108	108	108	108	108	108	108	108	108	108	108	108	108	108	108	107	112	114	114	114	112	112	121	114	114	114	112	114	109	129	89	89	89	89	97	115	96	115	106	143	105	104	104	104
125	119	131	131	99	101	99	99	99	99	99	99	99	99	99	99	99	99	99	99	99	99	99	99	137	140	140	140	137	137	137	140	140	140	137	140	132	80	128	128	128	128	108	111	114	126	115	118	130	114	114	114
112	143	124	123	131	132	131	131	131	131	131	131	131	131	131	131	131	131	131	131	131	131	131	131	120	120	121	120	120	120	123	120	120	120	120	120	120	136	119	119	119	119	124	127	131	131	119	139	115	118	118	118
128	123	142	136	103	104	103	103	103	103	103	103	103	103	103	103	103	103	103	103	103	103	103	102	145	146	146	146	145	145	146	146	146	146	145	146	129	104	123	123	123	123	113	116	119	134	92	126	139	90	90	90
118	126	134	136	92	93	92	92	92	92	92	92	92	92	92	92	92	92	92	92	92	92	92	91	144	149	149	149	144	144	149	149	149	149	144	149	125	97	109	109	109	109	109	100	107	130	89	114	139	87	87	87
118	126	134	136	92	93	92	92	92	92	92	92	92	92	92	92	92	92	92	92	92	92	92	91	144	149	149	149	144	144	149	149	149	149	144	149	125	97	109	109	109	109	109	100	107	130	89	114	139	87	87	87
118	126	134	136	92	93	92	92	92	92	92	92	92	92	92	92	92	92	92	92	92	92	92	91	144	149	149	149	144	144	149	149	149	149	144	149	125	97	109	109	109	109	109	100	107	130	89	114	139	87	87	87
118	126	134	136	92	93	92	92	92	92	92	92	92	92	92	92	92	92	92	92	92	92	92	91	144	149	149	149	144	144	149	149	149	149	144	149	125	97	109	109	109	109	109	100	107	130	89	114	139	87	87	87
129	139	95	100	116	116	116	116	116	116	116	116	116	116	116	116	116	116	116	116	116	116	116	116	125	127	127	127	125	125	123	127	127	127	125	127	132	133	123	123	123	123	115	124	108	125	113	133	127	112	112	112
120	145	125	118	113	114	113	113	113	113	113	113	113	113	113	113	113	113	113	113	113	113	113	112	125	124	124	124	125	125	114	124	124	124	125	124	115	110	120	120	120	120	110	105	104	114	107	131	115	106	106	106
138	69	146	148	131	132	131	131	131	131	131	131	131	131	131	131	131	131	131	131	131	131	131	132	149	149	149	149	149	149	149	149	149	149	149	149	138	106	146	146	146	146	134	110	134	138	135	0	139	134	134	134
129	129	130	131	111	111	111	111	111	111	111	111	111	111	111	111	111	111	111	111	111	111	111	110	140	138	138	138	140	140	138	138	138	138	140	138	135	106	125	125	125	125	117	102	130	125	111	122	139	111	111	111
128	130	124	123	113	115	113	113	113	113	113	113	113	113	113	113	113	113	113	113	113	113	113	113	136	132	132	132	136	136	125	132	132	132	136	132	121	125	126	126	126	126	121	116	117	130	106	122	128	106	106	106
130	153	86	104	115	116	115	115	115	115	115	115	115	115	115	115	115	115	115	115	115	115	115	114	116	115	115	115	116	116	116	115	115	115	116	115	130	133	89	89	89	89	110	120	101	117	122	151	113	122	122	122
130	153	86	104	115	116	115	115	115	115	115	115	115	115	115	115	115	115	115	115	115	115	115	114	116	115	115	115	116	116	116	115	115	115	116	115	130	133	89	89	89	89	110	120	101	117	122	151	113	122	122	122
130	153	86	104	115	116	115	115	115	115	115	115	115	115	115	115	115	115	115	115	115	115	115	114	116	115	115	115	116	116	116	115	115	115	116	115	130	133	89	89	89	89	110	120	101	117	122	151	113	122	122	122
130	153	86	104	115	116	115	115	115	115	115	115	115	115	115	115	115	115	115	115	115	115	115	114	116	115	115	115	116	116	116	115	115	115	116	115	130	133	89	89	89	89	110	120	101	117	122	151	113	122	122	122
130	142	84	109	113	114	113	113	113	113	113	113	113	113	113	113	113	113	113	113	113	113	113	113	106	112	113	112	106	106	113	112	112	112	106	112	118	126	60	60	60	60	100	127	95	115	111	146	105	109	109	109
149	71	149	153	140	141	140	140	140	140	140	140	140	140	140	140	140	140	140	140	140	140	140	141	151	151	150	151	151	151	149	151	151	151	151	151	143	115	144	144	144	144	143	126	144	144	143	90	145	143	143	143
126	137	121	120	124	124	124	124	124	124	124	124	124	124	124	124	124	124	124	124	124	124	124	124	103	102	102	102	103	103	100	102	102	102	103	102	96	130	124	124	124	124	117	139	118	112	130	138	97	128	128	128
134	123	131	124	106	107	106	106	106	106	106	106	106	106	106	106	106	106	106	106	106	106	106	106	139	137	137	137	139	139	139	137	137	137	139	137	120	107	125	125	125	125	113	111	114	127	92	109	126	90	90	90
137	137	106	117	108	108	108	108	108	108	108	108	108	108	108	108	108	108	108	108	108	108	108	108	122	130	130	130	122	122	128	130	130	130	122	130	123	128	116	116	116	116	116	107	114	130	70	138	113	68	68	68
137	138	107	117	109	109	109	109	109	109	109	109	109	109	109	109	109	109	109	109	109	109	109	109	122	130	130	130	122	122	128	130	130	130	122	130	123	129	117	117	117	117	117	108	115	130	71	138	114	69	69	69
137	137	106	117	108	108	108	108	108	108	108	108	108	108	108	108	108	108	108	108	108	108	108	108	122	130	130	130	122	122	128	130	130	130	122	130	123	128	116	116	116	116	116	107	114	130	70	138	113	68	68	68
137	137	106	117	108	108	108	108	108	108	108	108	108	108	108	108	108	108	108	108	108	108	108	108	122	130	130	130	122	122	128	130	130	130	122	130	123	128	116	116	116	116	116	107	114	130	70	138	113	68	68	68
137	137	106	117	108	108	108	108	108	108	108	108	108	108	108	108	108	108	108	108	108	108	108	108	122	130	130	130	122	122	128	130	130	130	122	130	123	128	116	116	116	116	116	107	114	130	70	138	113	68	68	68
137	137	106	117	108	108	108	108	108	108	108	108	108	108	108	108	108	108	108	108	108	108	108	108	122	130	130	130	122	122	128	130	130	130	122	130	123	128	116	116	116	116	116	107	114	130	70	138	113	68	68	68
137	137	106	117	108	108	108	108	108	108	108	108	108	108	108	108	108	108	108	108	108	108	108	108	122	130	130	130	122	122	128	130	130	130	122	130	123	128	116	116	116	116	116	107	114	130	70	138	113	68	68	68
137	137	106	117	108	108	108	108	108	108	108	108	108	108	108	108	108	108	108	108	108	108	108	108	122	130	130	130	122	122	128	130	130	130	122	130	123	128	116	116	116	116	116	107	114	130	70	138	113	68	68	68
137	137	106	117	108	108	108	108	108	108	108	108	108	108	108	108	108	108	108	108	108	108	108	108	122	130	130	130	122	122	128	130	130	130	122	130	123	128	116	116	116	116	116	107	114	130	70	138	113	68	68	68
137	137	106	117	108	108	108	108	108	108	108	108	108	108	108	108	108	108	108	108	108	108	108	108	122	130	130	130	122	122	128	130	130	130	122	130	123	128	116	116	116	116	116	107	114	130	70	138	113	68	68	68
137	137	106	117	108	108	108	108	108	108	108	108	108	108	108	108	108	108	108	108	108	108	108	108	122	130	130	130	122	122	128	130	130	130	122	130	123	128	116	116	116	116	116	107	114	130	70	138	113	68	68	68
137	137	106	117	108	108	108	108	108	108	108	108	108	108	108	108	108	108	108	108	108	108	108	108	122	130	130	130	122	122	128	130	130	130	122	130	123	128	116	116	116	116	116	107	114	130	70	138	113	68	68	68
137	137	106	117	108	108	108	108	108	108	108	108	108	108	108	108	108	108	108	108	108	108	108	108	122	130	130	130	122	122	128	130	130	130	122	130	123	128	116	116	116	116	116	107	114	130	70	138	113	68	68	68
137	137	106	117	108	108	108	108	108	108	108	108	108	108	108	108	108	108	108	108	108	108	108	108	122	130	130	130	122	122	128	130	130	130	122	130	123	128	116	116	116	116	116	107	114	130	70	138	113	68	68	68
137	137	106	117	108	108	108	108	108	108	108	108	108	108	108	108	108	108	108	108	108	108	108	108	122	130	130	130	122	122	128	130	130	130	122	130	123	128	116	116	116	116	116	107	114	130	70	138	113	68	68	68
137	137	106	117	108	108	108	108	108	108	108	108	108	108	108	108	108	108	108	108	108	108	108	108	122	130	130	130	122	122	128	130	130	130	122	130	123	128	116	116	116	116	116	107	114	130	70	138	113	68	68	68
137	137	106	117	108	108	108	108	108	108	108	108	108	108	108	108	108	108	108	108	108	108	108	108	122	130	130	130	122	122	128	130	130	130	122	130	123	128	116	116	116	116	116	107	114	130	70	138	113	68	68	68
137	137	106	117	108	108	108	108	108	108	108	108	108	108	108	108	108	108	108	108	108	108	108	108	122	130	130	130	122	122	128	130	130	130	122	130	123	128	116	116	116	116	116	107	114	130	70	138	113	68	68	68
137	137	106	117	108	108	108	108	108	108	108	108	108	108	108	108	108	108	108	108	108	108	108	108	122	130	130	130	122	122	128	130	130	130	122	130	123	128	116	116	116	116	116	107	114	130	70	138	113	68	68	68
137	137	106	117	108	108	108	108	108	108	108	108	108	108	108	108	108	108	108	108	108	108	108	108	122	130	130	130	122	122	128	130	130	130	122	130	123	128	116	116	116	116	116	107	114	130	70	138	113	68	68	68
137	137	106	117	108	108	108	108	108	108	108	108	108	108	108	108	108	108	108	108	108	108	108	108	122	130	130	130	122	122	128	130	130	130	122	130	123	128	116	116	116	116	116	107	114	130	70	138	113	68	68	68

137	137	106	117	108	108	108	108	108	108	108	108	108	108	108	108	108	108	108	108	108	108	108	108	122	130	130	130	122	122	128	130	130	130	122	130	123	128	116	116	116	116	116	107	114	130	70	138	113	68	68	68
122	130	137	126	106	107	106	106	106	106	106	106	106	106	106	106	106	106	106	106	106	106	106	105	138	134	134	134	138	138	129	134	134	134	138	134	133	112	121	121	121	121	112	114	112	127	109	130	126	107	107	107
126	149	99	89	135	135	135	135	135	135	135	135	135	135	135	135	135	135	135	135	135	135	135	135	79	74	75	74	79	79	68	74	74	74	79	74	98	140	120	120	120	120	130	135	121	94	129	149	73	129	129	129
125	148	99	103	139	139	139	139	139	139	139	139	139	139	139	139	139	139	139	139	139	139	139	139	79	78	79	78	79	79	66	78	78	78	79	78	100	141	125	125	125	125	135	130	126	101	133	145	78	133	133	133
117	133	121	115	102	102	102	102	102	102	102	102	102	102	102	102	102	102	102	102	102	102	102	101	137	136	136	136	137	137	136	136	136	136	137	136	121	113	116	116	116	116	103	119	105	116	86	129	129	85	85	85
124	147	90	93	128	128	128	128	128	128	128	128	128	128	128	128	128	128	128	128	128	128	128	127	109	106	107	106	109	109	111	106	106	106	109	106	116	132	114	114	114	114	106	120	105	115	116	144	118	116	116	116
124	119	131	131	99	101	99	99	99	99	99	99	99	99	99	99	99	99	99	99	99	99	99	99	137	140	140	140	137	137	137	140	140	140	137	140	132	80	128	128	128	128	108	111	114	126	115	118	129	114	114	114
124	119	131	131	99	101	99	99	99	99	99	99	99	99	99	99	99	99	99	99	99	99	99	99	137	140	140	140	137	137	137	140	140	140	137	140	132	80	128	128	128	128	108	111	114	126	115	118	129	114	114	114
124	119	131	131	99	101	99	99	99	99	99	99	99	99	99	99	99	99	99	99	99	99	99	99	137	140	140	140	137	137	137	140	140	140	137	140	132	80	128	128	128	128	108	111	114	126	115	118	129	114	114	114
124	119	131	131	99	101	99	99	99	99	99	99	99	99	99	99	99	99	99	99	99	99	99	99	137	140	140	140	137	137	137	140	140	140	137	140	132	81	128	128	128	128	108	111	114	126	115	118	129	114	114	114
124	119	131	131	99	101	99	99	99	99	99	99	99	99	99	99	99	99	99	99	99	99	99	99	137	140	140	140	137	137	137	140	140	140	137	140	132	80	128	128	128	128	108	111	114	126	115	118	129	114	114	114
0	144	125	123	122	125	122	122	122	122	122	122	122	122	122	122	122	122	122	122	122	122	122	121	131	128	128	128	131	131	124	128	128	128	131	128	115	124	128	128	128	128	122	131	120	110	129	138	128	129	129	129
144	0	147	152	137	137	137	137	137	137	137	137	137	137	137	137	137	137	137	137	137	137	137	138	150	150	150	150	150	150	149	150	150	150	150	150	140	110	145	145	145	145	139	121	139	142	135	69	140	134	134	134
125	147	0	94	132	132	132	132	132	132	132	132	132	132	132	132	132	132	132	132	132	132	132	132	103	97	97	97	103	103	102	97	97	97	103	97	108	137	101	101	101	101	114	131	102	102	123	146	90	123	123	123
123	152	94	0	134	133	134	134	134	134	134	134	134	134	134	134	134	134	134	134	134	134	134	134	102	96	97	96	102	102	94	96	96	96	102	96	112	138	121	121	121	121	116	132	114	84	124	148	110	124	124	124
122	137	132	134	0	3	0	0	0	0	0	1	0	0	0	0	0	0	0	0	0	0	0	1	144	144	144	144	144	144	141	144	144	144	144	144	128	110	90	90	90	90	75	95	85	121	92	131	135	90	90	90
125	137	132	133	3	0	3	3	3	3	3	4	3	3	3	3	3	3	3	3	3	3	3	4	144	144	144	144	144	144	141	144	144	144	144	144	128	112	91	91	91	91	77	95	88	121	93	132	136	91	91	91
122	137	132	134	0	3	0	0	0	0	0	1	0	0	0	0	0	0	0	0	0	0	0	1	144	144	144	144	144	144	141	144	144	144	144	144	128	110	90	90	90	90	75	95	85	121	92	131	135	90	90	90
122	137	132	134	0	3	0	0	0	0	0	1	0	0	0	0	0	0	0	0	0	0	0	1	144	144	144	144	144	144	141	144	144	144	144	144	128	110	90	90	90	90	75	95	85	121	92	131	135	90	90	90
122	137	132	134	0	3	0	0	0	0	0	1	0	0	0	0	0	0	0	0	0	0	0	1	144	144	144	144	144	144	141	144	144	144	144	144	128	110	90	90	90	90	75	95	85	121	92	131	135	90	90	90
122	137	132	134	0	3	0	0	0	0	0	1	0	0	0	0	0	0	0	0	0	0	0	1	144	144	144	144	144	144	141	144	144	144	144	144	128	110	90	90	90	90	75	95	85	121	92	131	135	90	90	90
122	137	132	134	0	3	0	0	0	0	0	1	0	0	0	0	0	0	0	0	0	0	0	1	144	144	144	144	144	144	141	144	144	144	144	144	128	110	90	90	90	90	75	95	85	121	92	131	135	90	90	90
122	137	132	134	1	4	1	1	1	1	1	0	1	1	1	1	1	1	1	1	1	1	1	2	144	144	144	144	144	144	141	144	144	144	144	144	128	110	90	90	90	90	75	95	86	122	92	131	135	90	90	90
122	137	132	134	0	3	0	0	0	0	0	1	0	0	0	0	0	0	0	0	0	0	0	1	144	144	144	144	144	144	141	144	144	144	144	144	128	110	90	90	90	90	75	95	85	121	92	131	135	90	90	90
122	137	132	134	0	3	0	0	0	0	0	1	0	0	0	0	0	0	0	0	0	0	0	1	144	144	144	144	144	144	141	144	144	144	144	144	128	110	90	90	90	90	75	95	85	121	92	131	135	90	90	90
122	137	132	134	0	3	0	0	0	0	0	1	0	0	0	0	0	0	0	0	0	0	0	1	144	144	144	144	144	144	141	144	144	144	144	144	128	110	90	90	90	90	75	95	85	121	92	131	135	90	90	90
122	137	132	134	0	3	0	0	0	0	0	1	0	0	0	0	0	0	0	0	0	0	0	1	144	144	144	144	144	144	141	144	144	144	144	144	128	110	90	90	90	90	75	95	85	121	92	131	135	90	90	90
122	137	132	134	0	3	0	0	0	0	0	1	0	0	0	0	0	0	0	0	0	0	0	1	144	144	144	144	144	144	141	144	144	144	144	144	128	110	90	90	90	90	75	95	85	121	92	131	135	90	90	90
122	137	132	134	0	3	0	0	0	0	0	1	0	0	0	0	0	0	0	0	0	0	0	1	144	144	144	144	144	144	141	144	144	144	144	144	128	110	90	90	90	90	75	95	85	121	92	131	135	90	90	90
122	137	132	134	0	3	0	0	0	0	0	1	0	0	0	0	0	0	0	0	0	0	0	1	144	144	144	144	144	144	141	144	144	144	144	144	128	110	90	90	90	90	75	95	85	121	92	131	135	90	90	90
122	137	132	134	0	3	0	0	0	0	0	1	0	0	0	0	0	0	0	0	0	0	0	1	144	144	144	144	144	144	141	144	144	144	144	144	128	110	90	90	90	90	75	95	85	121	92	131	135	90	90	90
122	137	132	134	0	3	0	0	0	0	0	1	0	0	0	0	0	0	0	0	0	0	0	1	144	144	144	144	144	144	141	144	144	144	144	144	128	110	90	90	90	90	75	95	85	121	92	131	135	90	90	90
122	137	132	134	0	3	0	0	0	0	0	1	0	0	0	0	0	0	0	0	0	0	0	1	144	144	144	144	144	144	141	144	144	144	144	144	128	110	90	90	90	90	75	95	85	121	92	131	135	90	90	90
122	137	132	134	0	3	0	0	0	0	0	1	0	0	0	0	0	0	0	0	0	0	0	1	144	144	144	144	144	144	141	144	144	144	144	144	128	110	90	90	90	90	75	95	85	121	92	131	135	90	90	90
121	138	132	134	1	4	1	1	1	1	1	2	1	1	1	1	1	1	1	1	1	1	1	0	144	144	144	144	144	144	141	144	144	144	144	144	128	109	89	89	89	89	74	96	84	120	91	132	135	89	89	89
131	150	103	102	144	144	144	144	144	144	144	144	144	144	144	144	144	144	144	144	144	144	144	144	0	45	46	45	0	0	77	45	45	45	0	45	101	142	122	122	122	122	132	139	122	106	141	149	101	141	141	141
128	150	97	96	144	144	144	144	144	144	144	144	144	144	144	144	144	144	144	144	144	144	144	144	45	0	1	0	45	45	72	0	0	0	45	0	92	144	127	127	127	127	133	138	121	96	138	149	94	138	138	138
128	150	97	97	144	144	144	144	144	144	144	144	144	144	144	144	144	144	144	144	144	144	144	144	46	1	0	1	46	46	73	1	1	1	46	1	93	144	127	127	127	127	133	138	121	97	138	149	94	138	138	138
128	150	97	96	144	144	144	144	144	144	144	144	144	144	144	144	144	144	144	144	144	144	144	144	45	0	1	0	45	45	72	0	0	0	45	0	92	144	127	127	127	127	133	138	121	96	138	149	94	138	138	138
131	150	103	102	144	144	144	144	144	144	144	144	144	144	144	144	144	144	144	144	144	144	144	144	0	45	46	45	0	0	77	45	45	45	0	45	101	142	122	122	122	122	132	139	122	106	141	149	101	141	141	141
131	150	103	102	144	144	144	144	144	144	144	144	144	144	144	144	144	144	144	144	144	144	144	144	0	45	46	45	0	0	77	45	45	45	0	45	101	142	122	122	122	122	132	139	122	106	141	149	101	141	141	141
124	149	102	94	141	141	141	141	141	141	141	141	141	141	141	141	141	141	141	141	141	141	141	141	77	72	73	72	77	77	0	72	72	72	77	72	79	144	124	124	124	124	136	137	124	85	133	149	95	133	133	133
128	150	97	96	144	144	144	144	144	144	144	144	144	144	144	144	144	144	144	144	144	144	144	144	45	0	1	0	45	45	72	0	0	0	45	0	92	144	127	127	127	127	133	138	121	96	138	149	94	138	138	138
128	150	97	96	144	144	144	144	144	144	144	144	144	144	144	144	144	144	144	144	144	144	144	144	45	0	1	0	45	45	72	0	0	0	45	0	92	144	127	127	127	127	133	138	121	96	138	149	94	138	138	138
128	150	97	96	144	144	144	144	144	144	144	144	144	144	144	144	144	144	144	144	144	144	144	144	45	0	1	0	45	45	72	0	0	0	45	0	92	144	127	127	127	127	133	138	121	96	138	149	94	138	138	138
131	150	103	102	144	144	144	144	144	144	144	144	144	144	144	144	144	144	144	144	144	144	144	144	0	45	46	45	0	0	77	45	45	45	0	45	101	142	122	122	122	122	132	139	122	106	141	149	101	141	141	141
128	150	97	96	144	144	144	144	144	144	144	144	144	144	144	144	144	144	144	144	144	144	144	144	45	0	1	0	45	45	72	0	0	0	45	0	92	144	127	127	127	127	133	138	121	96	138	149	94	138	138	138
115	140	108	112	128	128	128	128	128	128	128	128	128	128	128	128	128	128	128	128	128	128	128	128	101	92	93	92	101	101	79	92	92	92	101	92	0	133	116	116	116	116	114	135	115	75	121	138	94	120	120	120
124	110	137	138	110	112	110	110	110	110	110	110	110	110	110	110	110	110	110	110	110	110	110	109	142	144	144	144	142	142	144	144	144	144	142	144	133	0	124	124	124	124	114	116	119	128	123	106	135	122	122	122
128	145	101	121	90	91	90	90	90	90	90	90	90	90	90	90	90	90	90	90	90	90	90	89	122	127	127	127	122	122	124	127	127	127	122	127	116	124	0	0	0	0	89	117	100	121	115	146	117	114	114	114
128	145	101	121	90	91	90	90	90	90	90	90	90	90	90	90	90	90	90	90	90	90	90	89	122	127	127	127	122	122	124	127	127	127	122	127	116	124	0	0	0	0	89	117	100	121	115	146	117	114	114	114
128	145	101	121	90	91	90	90	90	90	90	90	90	90	90	90	90	90	90	90	90	90	90	89	122	127	127	127	122	122	124	127	127	127	122	127	116	124	0	0	0	0	89	117	100	121	115	146	117	114	114	114
128	145	101	121	90	91	90	90	90	90	90	90	90	90	90	90	90	90	90	90	90	90	90	89	122	127	127	127	122	122	124	127	127	127	122	127	116	124	0	0	0	0	89	117	100	121	115	146	117	114	114	114
122	139	114	116	75	77	75	75	75	75	75	75	75	75	75	75	75	75	75	75	75	75	75	74	132	133	133	133	132	132	136	133	133	133	132	133	114	114	89	89	89	89	0	123	76	119	107	134	124	105	105	105
131	121	131	132	95	95	95	95	95	95	95	95	95	95	95	95	95	95	95	95	95	95	95	96	139	138	138	138	139	139	137	138	138	138	139	138	135	116	117	117	117	117	123	0	128	136	111	110	128	110	110	110
120	139	102	114	85	88	85	85	85	85	85	86	85	85	85	85	85	85	85	85	85	85	85	84	122	121	121	121	122	122	124	121	121	121	122	121	115	119	100	100	100	100	76	128	0	112	106	134	116	105	105	105
110	142	102	84	121	121	121	121	121	121	121	122	121	121	121	121	121	121	121	121	121	121	121	120	106	96	97	96	106	106	85	96	96	96	106	96	75	128	121	121	121	121	119	136	112	0	123	138	114	123	123	123
129	135	123	124	92	93	92	92	92	92	92	92	92	92	92	92	92	92	92	92	92	92	92	91	141	138	138	138	141	141	133	138	138	138	141	138	121	123	115	115	115	115	107	111	106	123	0	135	116	2	2	2
138	69	146	148	131	132	131	131	131	131	131	131	131	131	131	131	131	131	131	131	131	131	131	132	149	149	149	149	149	149	149	149	149	149	149	149	138	106	146	146	146	146	134	110	134	138	135	0	139	134	134	134
128	140	90	110	135	136	135	135	135	135	135	135	135	135	135	135	135	135	135	135	135	135	135	135	101	94	94	94	101	101	95	94	94	94	101	94	94	135	117	117	117	117	124	128	116	114	116	139	0	115	115	115
129	134	123	124	90	91	90	90	90	90	90	90	90	90	90	90	90	90	90	90	90	90	90	89	141	138	138	138	141	141	133	138	138	138	141	138	120	122	114	114	114	114	105	110	105	123	2	134	115	0	0	0
129	134	123	124	90	91	90	90	90	90	90	90	90	90	90	90	90	90	90	90	90	90	90	89	141	138	138	138	141	141	133	138	138	138	141	138	120	122	114	114	114	114	105	110	105	123	2	134	115	0	0	0
129	134	123	124	90	91	90	90	90	90	90	90	90	90	90	90	90	90	90	90	90	90	90	89	141	138	138	138	141	141	133	138	138	138	141	138	120	122	114	114	114	114	105	110	105	123	2	134	115	0	0	0
151	78	147	151	148	148	148	148	148	148	148	148	148	148	148	148	148	148	148	148	148	148	148	149	151	151	151	151	151	151	150	151	151	151	151	151	147	109	145	145	145	145	144	123	142	148	150	82	145	150	150	150
128	125	120	122	101	102	101	101	101	101	101	101	101	101	101	101	101	101	101	101	101	101	101	101	139	139	139	139	139	139	135	139	139	139	139	139	128	106	111	111	111	111	117	115	112	129	62	123	117	60	60	60
141	140	124	114	128	128	128	128	128	128	128	128	128	128	128	128	128	128	128	128	128	128	128	127	113	115	116	115	113	113	111	115	115	115	113	115	99	129	126	126	126	126	124	126	127	100	104	139	108	103	103	103
136	106	140	145	108	109	108	108	108	108	108	108	108	108	108	108	108	108	108	108	108	108	108	107	140	140	140	140	140	140	140	140	140	140	140	140	128	100	128	128	128	128	111	105	121	133	100	97	131	99	99	99
126	145	80	99	116	117	116	116	116	116	116	116	116	116	116	116	116	116	116	116	116	116	116	116	107	110	110	110	107	107	101	110	110	110	107	110	107	135	106	106	106	106	101	124	90	111	123	145	111	122	122	122
124	144	76	98	115	115	115	115	115	115	115	115	115	115	115	115	115	115	115	115	115	115	115	115	110	108	109	108	110	110	110	108	108	108	110	108	111	132	98	98	98	98	96	119	89	116	122	143	103	121	121	121
128	122	143	124	102	103	102	102	102	102	102	102	102	102	102	102	102	102	102	102	102	102	102	102	143	140	140	140	143	143	140	140	140	140	143	140	129	111	122	122	122	122	119	108	118	127	101	119	131	100	100	100
127	119	143	139	88	89	88	88	88	88	88	88	88	88	88	88	88	88	88	88	88	88	88	89	147	149	149	149	147	147	144	149	149	149	147	149	129	101	125	125	125	125	116	62	121	131	114	92	139	113	113	113
134	137	100	108	129	129	129	129	129	129	129	129	129	129	129	129	129	129	129	129	129	129	129	128	127	123	123	123	127	127	120	123	123	123	127	123	126	140	132	132	132	132	133	108	117	122	78	142	113	78	78	78
130	116	137	139	108	109	108	108	108	108	108	108	108	108	108	108	108	108	108	108	108	108	108	107	144	143	143	143	144	144	145	143	143	143	144	143	130	100	124	124	124	124	117	113	124	129	97	109	139	95	95	95
122	150	70	101	129	129	129	129	129	129	129	129	129	129	129	129	129	129	129	129	129	129	129	129	105	102	102	102	105	105	96	102	102	102	105	102	117	137	110	110	110	110	114	129	104	111	128	146	99	128	128	128
129	126	127	131	102	103	102	102	102	102	102	102	102	102	102	102	102	102	102	102	102	102	102	102	139	138	138	138	139	139	138	138	138	138	139	138	126	104	119	119	119	119	111	107	121	128	103	125	131	102	102	102

90	142	134	126	133	135	133	133	133	133	133	133	133	133	133	133	133	133	133	133	133	133	133	133	131	128	128	128	131	131	129	128	128	128	131	128	128	136	139	139	139	139	138	143	132	128	136	139	129	136	136	136
122	132	115	133	93	94	93	93	93	93	93	93	93	93	93	93	93	93	93	93	93	93	93	92	132	134	134	134	132	132	138	134	134	134	132	134	130	108	97	97	97	97	98	119	84	130	94	134	121	93	93	93
122	126	110	127	93	94	93	93	93	93	93	94	93	93	93	93	93	93	93	93	93	93	93	92	130	132	132	132	130	130	129	132	132	132	130	132	117	118	99	99	99	99	110	104	102	130	103	131	114	102	102	102
136	132	118	126	77	77	77	77	77	77	77	77	77	77	77	77	77	77	77	77	77	77	77	76	135	134	134	134	135	135	138	134	134	134	135	134	131	117	95	95	95	95	104	111	105	129	85	133	125	84	84	84
131	139	124	123	100	101	100	100	100	100	100	100	100	100	100	100	100	100	100	100	100	100	100	99	129	133	133	133	129	129	130	133	133	133	129	133	125	121	106	106	106	106	103	91	104	129	102	134	125	101	101	101
131	131	97	118	116	116	116	116	116	116	116	116	116	116	116	116	116	116	116	116	116	116	116	116	121	118	118	118	121	121	120	118	118	118	121	118	113	134	101	101	101	101	114	107	108	124	109	140	105	109	109	109
130	137	119	127	68	69	68	68	68	68	68	68	68	68	68	68	68	68	68	68	68	68	68	67	138	137	137	137	138	138	134	137	137	137	138	137	124	119	90	90	90	90	87	112	91	119	107	134	128	106	106	106
125	145	104	111	82	82	82	82	82	82	82	82	82	82	82	82	82	82	82	82	82	82	82	81	126	125	125	125	126	126	127	125	125	125	126	125	127	120	105	105	105	105	84	116	85	119	117	143	118	115	115	115
147	118	149	153	140	140	140	140	140	140	140	140	140	140	140	140	140	140	140	140	140	140	140	140	148	148	148	148	148	148	147	148	148	148	148	148	148	126	146	146	146	146	145	133	146	146	146	123	141	145	145	145
125	140	120	111	94	94	94	94	94	94	94	94	94	94	94	94	94	94	94	94	94	94	94	93	124	119	119	119	124	124	120	119	119	119	124	119	109	125	117	117	117	117	108	113	102	105	106	129	116	105	105	105
120	145	95	112	95	96	95	95	95	95	95	95	95	95	95	95	95	95	95	95	95	95	95	95	121	120	120	120	121	121	119	120	120	120	121	120	115	131	88	88	88	88	91	111	102	113	113	144	110	111	111	111
149	80	148	148	142	143	142	142	142	142	142	142	142	142	142	142	142	142	142	142	142	142	142	141	148	148	148	148	148	148	147	148	148	148	148	148	148	114	149	149	149	149	141	135	144	146	139	93	143	139	139	139
145	65	151	152	141	142	141	141	141	141	141	141	141	141	141	141	141	141	141	141	141	141	141	142	147	148	148	148	147	147	143	148	148	148	147	148	142	109	146	146	146	146	138	126	141	147	137	92	143	137	137	137
130	137	113	124	96	97	96	96	96	96	96	95	96	96	96	96	96	96	96	96	96	96	96	96	130	127	127	127	130	130	123	127	127	127	130	127	122	131	102	102	102	102	96	111	105	121	99	138	121	97	97	97
119	137	84	111	116	116	116	116	116	116	116	116	116	116	116	116	116	116	116	116	116	116	116	116	114	109	110	109	114	114	115	109	109	109	114	109	108	133	98	98	98	98	97	121	97	120	117	141	104	115	115	115
132	142	98	122	85	87	85	85	85	85	85	85	85	85	85	85	85	85	85	85	85	85	85	85	126	123	123	123	126	126	124	123	123	123	126	123	122	114	99	99	99	99	91	110	74	121	97	140	116	95	95	95
133	138	99	114	94	95	94	94	94	94	94	95	94	94	94	94	94	94	94	94	94	94	94	94	130	128	128	128	130	130	130	128	128	128	130	128	122	114	97	97	97	97	87	124	87	119	103	141	116	101	101	101
127	142	113	127	85	87	85	85	85	85	85	85	85	85	85	85	85	85	85	85	85	85	85	85	136	132	132	132	136	136	128	132	132	132	136	132	115	126	108	108	108	108	102	107	102	126	80	141	112	79	79	79
118	143	89	111	111	111	111	111	111	111	111	111	111	111	111	111	111	111	111	111	111	111	111	110	120	117	117	117	120	120	113	117	117	117	120	117	114	130	92	92	92	92	94	126	92	115	113	143	113	112	112	112
128	132	106	104	105	105	105	105	105	105	105	105	105	105	105	105	105	105	105	105	105	105	105	105	115	113	113	113	115	115	121	113	113	113	115	113	111	130	116	116	116	116	106	120	95	113	111	134	111	110	110	110
124	130	110	119	110	110	110	110	110	110	110	110	110	110	110	110	110	110	110	110	110	110	110	109	118	115	115	115	118	118	111	115	115	115	118	115	88	125	110	110	110	110	119	114	100	110	117	127	108	115	115	115
127	144	97	115	95	96	95	95	95	95	95	95	95	95	95	95	95	95	95	95	95	95	95	94	128	128	128	128	128	128	128	128	128	128	128	128	121	122	85	85	85	85	93	122	85	127	101	149	113	99	99	99
130	143	88	90	121	121	121	121	121	121	121	121	121	121	121	121	121	121	121	121	121	121	121	120	119	115	115	115	119	119	116	115	115	115	119	115	119	133	104	104	104	104	108	123	94	107	106	144	116	105	105	105
144	75	147	146	148	149	148	148	148	148	148	148	148	148	148	148	148	148	148	148	148	148	148	147	146	148	148	148	146	146	147	148	148	148	146	148	148	118	146	146	146	146	142	135	143	143	146	92	140	146	146	146
122	146	105	113	93	93	93	93	93	93	93	93	93	93	93	93	93	93	93	93	93	93	93	92	123	121	121	121	123	123	119	121	121	121	123	121	114	124	113	113	113	113	102	113	91	116	84	142	112	83	83	83
124	128	104	117	92	94	92	92	92	92	92	93	92	92	92	92	92	92	92	92	92	92	92	92	122	124	124	124	122	122	123	124	124	124	122	124	118	118	100	100	100	100	100	108	95	127	114	129	110	113	113	113
121	143	80	104	123	123	123	123	123	123	123	123	123	123	123	123	123	123	123	123	123	123	123	123	105	104	104	104	105	105	105	104	104	104	105	104	108	136	91	91	91	91	107	116	107	117	122	145	101	121	121	121
128	138	100	116	101	102	101	101	101	101	101	101	101	101	101	101	101	101	101	101	101	101	101	100	128	121	121	121	128	128	126	121	121	121	128	121	119	118	102	102	102	102	97	110	91	116	101	140	114	99	99	99
125	138	105	116	107	107	107	107	107	107	107	108	107	107	107	107	107	107	107	107	107	107	107	106	124	123	123	123	124	124	128	123	123	123	124	123	121	126	113	113	113	113	107	113	105	125	89	132	116	87	87	87
127	135	87	114	112	114	112	112	112	112	112	112	112	112	112	112	112	112	112	112	112	112	112	112	118	109	110	109	118	118	113	109	109	109	118	109	111	133	96	96	96	96	104	115	104	123	116	135	103	116	116	116
128	139	97	116	116	117	116	116	116	116	116	116	116	116	116	116	116	116	116	116	116	116	116	115	112	108	108	108	112	112	121	108	108	108	112	108	111	133	93	93	93	93	88	120	87	122	115	139	107	113	113	113
126	137	117	124	74	75	74	74	74	74	74	75	74	74	74	74	74	74	74	74	74	74	74	73	140	139	139	139	140	140	140	139	139	139	140	139	127	117	95	95	95	95	92	113	96	126	72	139	123	71	71	71
136	136	136	127	110	111	110	110	110	110	110	110	110	110	110	110	110	110	110	110	110	110	110	109	128	128	128	128	128	128	136	128	128	128	128	128	121	129	120	120	120	120	117	110	112	122	95	128	117	94	94	94
124	144	81	117	92	94	92	92	92	92	92	92	92	92	92	92	92	92	92	92	92	92	92	93	124	118	118	118	124	124	120	118	118	118	124	118	122	123	81	81	81	81	93	113	96	117	120	147	119	120	120	120
143	90	140	148	123	124	123	123	123	123	123	123	123	123	123	123	123	123	123	123	123	123	123	122	147	149	149	149	147	147	148	149	149	149	147	149	141	108	135	135	135	135	134	117	135	144	110	103	138	108	108	108
137	138	97	116	105	105	105	105	105	105	105	105	105	105	105	105	105	105	105	105	105	105	105	105	124	124	124	124	124	124	129	124	124	124	124	124	126	128	108	108	108	108	97	113	92	128	95	137	111	93	93	93
131	143	98	109	104	104	104	104	104	104	104	105	104	104	104	104	104	104	104	104	104	104	104	104	118	119	119	119	118	118	122	119	119	119	118	119	122	120	100	100	100	100	104	124	96	124	91	145	110	90	90	90
134	137	103	128	81	82	81	81	81	81	81	81	81	81	81	81	81	81	81	81	81	81	81	81	135	133	133	133	135	135	131	133	133	133	135	133	120	113	100	100	100	100	108	102	92	125	97	138	123	95	95	95
136	135	113	115	94	95	94	94	94	94	94	95	94	94	94	94	94	94	94	94	94	94	94	94	132	135	135	135	132	132	132	135	135	135	132	135	125	119	111	111	111	111	102	118	102	124	93	138	122	91	91	91
127	135	120	124	82	84	82	82	82	82	82	83	82	82	82	82	82	82	82	82	82	82	82	81	135	135	135	135	135	135	131	135	135	135	135	135	124	130	106	106	106	106	95	113	88	121	97	141	124	95	95	95
120	143	91	94	112	113	112	112	112	112	112	113	112	112	112	112	112	112	112	112	112	112	112	111	117	116	117	116	117	117	119	116	116	116	117	116	128	123	100	100	100	100	98	122	93	118	121	144	123	120	120	120
128	137	92	103	122	123	122	122	122	122	122	122	122	122	122	122	122	122	122	122	122	122	122	122	112	111	111	111	112	112	115	111	111	111	112	111	112	130	96	96	96	96	99	117	103	121	100	140	100	98	98	98
145	114	147	150	126	126	126	126	126	126	126	126	126	126	126	126	126	126	126	126	126	126	126	126	143	143	143	143	143	143	144	143	143	143	143	143	141	125	143	143	143	143	141	122	142	141	132	120	144	131	131	131
128	137	105	122	78	80	78	78	78	78	78	79	78	78	78	78	78	78	78	78	78	78	78	79	130	128	128	128	130	130	126	128	128	128	130	128	126	120	91	91	91	91	93	115	100	120	113	144	122	112	112	112
131	138	121	123	91	92	91	91	91	91	91	92	91	91	91	91	91	91	91	91	91	91	91	91	130	131	131	131	130	130	126	131	131	131	130	131	128	126	100	100	100	100	116	99	115	128	80	143	125	78	78	78
135	142	116	129	69	70	69	69	69	69	69	69	69	69	69	69	69	69	69	69	69	69	69	68	134	131	131	131	134	134	133	131	131	131	134	131	122	116	98	98	98	98	99	113	96	124	95	145	122	93	93	93
121	147	82	115	94	96	94	94	94	94	94	95	94	94	94	94	94	94	94	94	94	94	94	93	122	119	119	119	122	122	114	119	119	119	122	119	110	122	83	83	83	83	87	126	82	108	111	144	107	110	110	110
126	144	107	123	78	80	78	78	78	78	78	78	78	78	78	78	78	78	78	78	78	78	78	78	126	126	126	126	126	126	128	126	126	126	126	126	124	118	88	88	88	88	88	116	88	125	112	147	119	110	110	110
131	127	128	131	99	100	99	99	99	99	99	99	99	99	99	99	99	99	99	99	99	99	99	98	134	137	137	137	134	134	138	137	137	137	134	137	128	114	116	116	116	116	103	106	108	127	96	127	132	95	95	95
128	135	86	110	121	122	121	121	121	121	121	121	121	121	121	121	121	121	121	121	121	121	121	121	110	109	109	109	110	110	107	109	109	109	110	109	116	135	87	87	87	87	101	119	106	126	111	138	110	110	110	110
127	142	79	108	98	99	98	98	98	98	98	98	98	98	98	98	98	98	98	98	98	98	98	99	119	116	116	116	119	119	110	116	116	116	119	116	114	131	98	98	98	98	102	115	95	114	119	147	110	118	118	118
128	140	102	118	100	101	100	100	100	100	100	100	100	100	100	100	100	100	100	100	100	100	100	100	130	127	127	127	130	130	123	127	127	127	130	127	112	133	110	110	110	110	115	112	102	115	78	147	113	77	77	77
131	139	96	105	92	92	92	92	92	92	92	92	92	92	92	92	92	92	92	92	92	92	92	93	129	122	122	122	129	129	124	122	122	122	129	122	116	123	103	103	103	103	103	113	98	113	96	144	115	94	94	94
131	133	108	125	94	95	94	94	94	94	94	95	94	94	94	94	94	94	94	94	94	94	94	94	132	132	132	132	132	132	135	132	132	132	132	132	128	119	106	106	106	106	102	109	92	130	102	130	120	101	101	101
132	143	97	114	112	112	112	112	112	112	112	113	112	112	112	112	112	112	112	112	112	112	112	111	120	112	112	112	120	120	119	112	112	112	120	112	118	139	112	112	112	112	107	103	100	114	86	142	104	84	84	84
132	139	104	127	87	88	87	87	87	87	87	87	87	87	87	87	87	87	87	87	87	87	87	87	136	136	136	136	136	136	132	136	136	136	136	136	118	117	97	97	97	97	107	105	91	125	86	140	117	85	85	85
140	142	106	130	83	84	83	83	83	83	83	83	83	83	83	83	83	83	83	83	83	83	83	84	134	133	133	133	134	134	130	133	133	133	134	133	130	130	98	98	98	98	106	119	106	132	107	143	131	106	106	106
131	139	96	105	92	92	92	92	92	92	92	92	92	92	92	92	92	92	92	92	92	92	92	93	129	122	122	122	129	129	124	122	122	122	129	122	116	123	103	103	103	103	103	113	98	113	96	144	115	94	94	94
136	130	129	121	119	120	119	119	119	119	119	119	119	119	119	119	119	119	119	119	119	119	119	118	132	132	132	132	132	132	129	132	132	132	132	132	128	127	134	134	134	134	127	110	127	135	116	119	137	116	116	116
135	100	141	138	120	120	120	120	120	120	120	120	120	120	120	120	120	120	120	120	120	120	120	121	139	140	140	140	139	139	141	140	140	140	139	140	132	124	137	137	137	137	129	89	130	134	106	99	137	106	106	106
126	129	115	127	106	106	106	106	106	106	106	106	106	106	106	106	106	106	106	106	106	106	106	105	130	132	132	132	130	130	131	132	132	132	130	132	118	124	114	114	114	114	117	99	110	125	69	139	124	68	68	68
126	142	93	107	105	105	105	105	105	105	105	105	105	105	105	105	105	105	105	105	105	105	105	105	121	119	119	119	121	121	120	119	119	119	121	119	122	131	90	90	90	90	104	123	103	123	113	147	116	113	113	113
132	97	141	145	115	117	115	115	115	115	115	115	115	115	115	115	115	115	115	115	115	115	115	114	145	144	144	144	145	145	148	144	144	144	145	144	129	105	131	131	131	131	113	117	124	132	114	83	129	112	112	112
134	136	101	123	101	101	101	101	101	101	101	100	101	101	101	101	101	101	101	101	101	101	101	101	124	125	125	125	124	124	125	125	125	125	124	125	119	120	104	104	104	104	97	114	96	127	103	136	111	101	101	101
125	140	112	124	82	83	82	82	82	82	82	83	82	82	82	82	82	82	82	82	82	82	82	82	130	130	130	130	130	130	133	130	130	130	130	130	124	120	97	97	97	97	85	120	95	133	80	143	118	78	78	78
128	138	95	121	109	110	109	109	109	109	109	109	109	109	109	109	109	109	109	109	109	109	109	108	118	121	121	121	118	118	123	121	121	121	118	121	113	120	86	86	86	86	105	117	95	119	111	146	107	110	110	110
131	136	85	102	130	131	130	130	130	130	130	130	130	130	130	130	130	130	130	130	130	130	130	129	115	112	112	112	115	115	108	112	112	112	115	112	125	132	101	101	101	101	108	121	99	121	125	137	104	125	125	125
128	109	143	144	102	104	102	102	102	102	102	102	102	102	102	102	102	102	102	102	102	102	102	103	144	144	144	144	144	144	145	144	144	144	144	144	134	107	126	126	126	126	114	95	122	132	99	93	141	97	97	97
132	141	84	104	97	97	97	97	97	97	97	97	97	97	97	97	97	97	97	97	97	97	97	98	120	114	114	114	120	120	120	114	114	114	120	114	109	125	88	88	88	88	94	109	92	114	113	143	110	111	111	111
122	137	112	124	77	78	77	77	77	77	77	77	77	77	77	77	77	77	77	77	77	77	77	76	142	140	140	140	142	142	134	140	140	140	142	140	127	121	95	95	95	95	103	110	93	117	104	134	123	102	102	102
136	139	103	122	107	107	107	107	107	107	107	108	107	107	107	107	107	107	107	107	107	107	107	107	125	123	123	123	125	125	125	123	123	123	125	123	125	131	115	115	115	115	107	116	98	131	80	136	110	79	79	79
146	133	149	148	143	143	143	143	143	143	143	143	143	143	143	143	143	143	143	143	143	143	143	143	145	145	145	145	145	145	145	145	145	145	145	145	142	135	144	144	144	144	143	138	139	142	146	136	145	146	146	146
122	143	107	112	95	97	95	95	95	95	95	95	95	95	95	95	95	95	95	95	95	95	95	94	126	130	130	130	126	126	125	130	130	130	126	130	124	126	90	90	90	90	99	112	97	122	95	148	113	93	93	93
129	138	108	117	92	92	92	92	92	92	92	93	92	92	92	92	92	92	92	92	92	92	92	92	131	134	134	134	131	131	130	134	134	134	131	134	123	118	107	107	107	107	97	112	113	124	76	137	122	75	75	75
127	139	107	120	97	98	97	97	97	97	97	98	97	97	97	97	97	97	97	97	97	97	97	96	128	135	135	135	128	128	133	135	135	135	128	135	124	119	96	96	96	96	100	116	96	132	82	142	117	81	81	81

135	138	105	119	104	105	104	104	104	104	104	105	104	104	104	104	104	104	104	104	104	104	104	104	129	127	127	127	129	129	126	127	127	127	129	127	134	129	114	114	114	114	103	111	97	133	98	141	118	96	96	96
131	132	117	132	78	78	78	78	78	78	78	79	78	78	78	78	78	78	78	78	78	78	78	77	136	136	136	136	136	136	132	136	136	136	136	136	125	124	101	101	101	101	105	108	106	125	86	134	124	85	85	85
122	143	78	87	122	122	122	122	122	122	122	122	122	122	122	122	122	122	122	122	122	122	122	123	115	111	112	111	115	115	109	111	111	111	115	111	117	134	111	111	111	111	107	120	101	118	122	146	117	121	121	121
124	139	94	109	113	113	113	113	113	113	113	113	113	113	113	113	113	113	113	113	113	113	113	112	118	121	121	121	118	118	120	121	121	121	118	121	116	123	98	98	98	98	103	125	101	124	109	147	107	108	108	108
125	148	114	131	82	84	82	82	82	82	82	82	82	82	82	82	82	82	82	82	82	82	82	81	137	135	135	135	137	137	133	135	135	135	137	135	129	136	101	101	101	101	97	109	109	123	99	141	124	99	99	99
124	143	102	118	103	105	103	103	103	103	103	104	103	103	103	103	103	103	103	103	103	103	103	102	128	125	125	125	128	128	123	125	125	125	128	125	112	134	96	96	96	96	94	120	93	116	89	138	104	88	88	88
135	118	128	133	115	116	115	115	115	115	115	115	115	115	115	115	115	115	115	115	115	115	115	115	138	131	131	131	138	138	132	131	131	131	138	131	131	117	127	127	127	127	126	119	127	130	103	114	128	103	103	103
135	139	118	123	81	82	81	81	81	81	81	82	81	81	81	81	81	81	81	81	81	81	81	81	132	134	134	134	132	132	133	134	134	134	132	134	128	127	108	108	108	108	105	111	96	131	80	143	123	78	78	78
126	138	106	124	88	88	88	88	88	88	88	88	88	88	88	88	88	88	88	88	88	88	88	89	126	129	129	129	126	126	121	129	129	129	126	129	117	130	101	101	101	101	110	106	110	130	106	142	120	105	105	105
132	115	121	121	125	126	125	125	125	125	125	125	125	125	125	125	125	125	125	125	125	125	125	124	132	131	131	131	132	132	132	131	131	131	132	131	122	114	113	113	113	113	112	115	108	132	98	120	122	97	97	97
122	139	99	117	97	98	97	97	97	97	97	98	97	97	97	97	97	97	97	97	97	97	97	97	129	130	130	130	129	129	135	130	130	130	129	130	115	120	94	94	94	94	90	114	87	122	99	134	118	98	98	98
125	129	115	125	88	89	88	88	88	88	88	89	88	88	88	88	88	88	88	88	88	88	88	87	131	132	132	132	131	131	132	132	132	132	131	132	121	127	109	109	109	109	96	120	91	123	104	139	123	102	102	102
128	142	92	107	106	107	106	106	106	106	106	106	106	106	106	106	106	106	106	106	106	106	106	105	120	117	117	117	120	120	119	117	117	117	120	117	115	127	93	93	93	93	90	121	81	117	104	145	115	104	104	104
134	139	104	113	100	101	100	100	100	100	100	100	100	100	100	100	100	100	100	100	100	100	100	99	129	128	128	128	129	129	124	128	128	128	129	128	120	119	99	99	99	99	99	118	99	121	87	143	115	86	86	86
131	133	102	115	114	114	114	114	114	114	114	114	114	114	114	114	114	114	114	114	114	114	114	115	124	124	124	124	124	124	125	124	124	124	124	124	114	128	103	103	103	103	102	114	101	124	99	141	109	99	99	99
127	137	115	108	112	113	112	112	112	112	112	112	112	112	112	112	112	112	112	112	112	112	112	111	124	123	123	123	124	124	119	123	123	123	124	123	120	117	108	108	108	108	111	112	112	120	89	128	105	87	87	87
127	143	101	116	88	90	88	88	88	88	88	88	88	88	88	88	88	88	88	88	88	88	88	87	134	133	133	133	134	134	124	133	133	133	134	133	127	129	100	100	100	100	109	118	99	120	102	139	118	102	102	102
125	143	95	105	111	112	111	111	111	111	111	112	111	111	111	111	111	111	111	111	111	111	111	111	109	112	112	112	109	109	112	112	112	112	109	112	110	132	108	108	108	108	97	120	92	122	104	144	100	102	102	102
142	81	147	147	140	141	140	140	140	140	140	140	140	140	140	140	140	140	140	140	140	140	140	141	145	145	145	145	145	145	146	145	145	145	145	145	148	116	143	143	143	143	139	125	141	146	145	90	141	145	145	145
132	141	96	105	118	119	118	118	118	118	118	118	118	118	118	118	118	118	118	118	118	118	118	118	123	129	129	129	123	123	125	129	129	129	123	129	121	122	102	102	102	102	108	120	105	123	89	144	109	89	89	89
129	140	101	117	103	103	103	103	103	103	103	103	103	103	103	103	103	103	103	103	103	103	103	102	126	124	124	124	126	126	117	124	124	124	126	124	112	131	102	102	102	102	99	111	95	119	84	146	108	82	82	82
126	143	98	120	119	120	119	119	119	119	119	119	119	119	119	119	119	119	119	119	119	119	119	118	119	117	117	117	119	119	120	117	117	117	119	117	118	129	104	104	104	104	106	123	103	129	94	147	110	93	93	93
119	144	97	114	113	113	113	113	113	113	113	113	113	113	113	113	113	113	113	113	113	113	113	112	116	116	116	116	116	116	115	116	116	116	116	116	114	135	85	85	85	85	97	117	96	121	118	150	114	116	116	116
122	138	100	112	103	105	103	103	103	103	103	104	103	103	103	103	103	103	103	103	103	103	103	102	121	119	119	119	121	121	120	119	119	119	121	119	116	127	99	99	99	99	105	118	97	111	106	144	110	105	105	105
133	144	96	113	101	101	101	101	101	101	101	100	101	101	101	101	101	101	101	101	101	101	101	100	121	124	124	124	121	121	124	124	124	124	121	124	129	127	108	108	108	108	106	106	107	120	97	146	120	96	96	96
125	143	93	119	97	97	97	97	97	97	97	97	97	97	97	97	97	97	97	97	97	97	97	98	125	123	123	123	125	125	120	123	123	123	125	123	118	127	88	88	88	88	107	120	96	122	100	142	110	99	99	99
135	140	105	117	104	105	104	104	104	104	104	104	104	104	104	104	104	104	104	104	104	104	104	104	122	119	119	119	122	122	126	119	119	119	122	119	124	125	111	111	111	111	117	119	106	132	84	142	113	83	83	83
116	139	87	110	110	110	110	110	110	110	110	111	110	110	110	110	110	110	110	110	110	110	110	109	118	114	114	114	118	118	114	114	114	114	118	114	112	126	95	95	95	95	97	120	98	118	115	142	110	114	114	114
128	131	117	130	87	88	87	87	87	87	87	88	87	87	87	87	87	87	87	87	87	87	87	86	138	139	139	139	138	138	135	139	139	139	138	139	127	115	108	108	108	108	107	106	104	126	72	139	127	70	70	70
131	138	97	116	103	103	103	103	103	103	103	102	103	103	103	103	103	103	103	103	103	103	103	103	122	121	121	121	122	122	119	121	121	121	122	121	110	128	99	99	99	99	100	113	105	117	102	142	110	101	101	101
129	140	99	111	108	108	108	108	108	108	108	108	108	108	108	108	108	108	108	108	108	108	108	108	120	120	120	120	120	120	119	120	120	120	120	120	126	124	99	99	99	99	102	117	102	128	103	137	118	103	103	103
122	135	97	115	112	112	112	112	112	112	112	112	112	112	112	112	112	112	112	112	112	112	112	111	122	120	120	120	122	122	119	120	120	120	122	120	116	130	101	101	101	101	100	112	104	114	119	140	114	118	118	118
130	141	112	121	94	95	94	94	94	94	94	94	94	94	94	94	94	94	94	94	94	94	94	93	134	135	135	135	134	134	131	135	135	135	134	135	121	126	104	104	104	104	99	118	90	131	83	140	113	82	82	82
129	141	96	116	118	118	118	118	118	118	118	118	118	118	118	118	118	118	118	118	118	118	118	118	118	115	115	115	118	118	114	115	115	115	118	115	120	134	117	117	117	117	111	115	106	129	105	141	107	104	104	104
128	138	122	128	97	99	97	97	97	97	97	97	97	97	97	97	97	97	97	97	97	97	97	96	138	138	138	138	138	138	131	138	138	138	138	138	128	129	105	105	105	105	103	110	109	131	82	141	123	80	80	80
135	134	113	121	92	93	92	92	92	92	92	91	92	92	92	92	92	92	92	92	92	92	92	92	132	129	129	129	132	132	133	129	129	129	132	129	123	116	111	111	111	111	99	109	91	128	93	132	119	91	91	91
128	134	122	128	74	76	74	74	74	74	74	75	74	74	74	74	74	74	74	74	74	74	74	73	141	136	136	136	141	141	134	136	136	136	141	136	125	120	101	101	101	101	95	96	96	123	87	137	123	85	85	85
127	136	110	121	91	93	91	91	91	91	91	91	91	91	91	91	91	91	91	91	91	91	91	91	132	131	131	131	132	132	126	131	131	131	132	131	120	124	95	95	95	95	105	106	108	126	98	137	116	97	97	97
122	131	116	131	86	88	86	86	86	86	86	86	86	86	86	86	86	86	86	86	86	86	86	85	128	126	126	126	128	128	127	126	126	126	128	126	117	125	99	99	99	99	88	126	89	116	105	132	119	104	104	104
132	144	96	109	106	106	106	106	106	106	106	106	106	106	106	106	106	106	106	106	106	106	106	106	127	127	127	127	127	127	118	127	127	127	127	127	116	127	104	104	104	104	104	124	83	125	107	141	111	105	105	105
133	147	91	116	102	103	102	102	102	102	102	102	102	102	102	102	102	102	102	102	102	102	102	103	122	122	122	122	122	122	123	122	122	122	122	122	128	133	95	95	95	95	111	122	104	126	105	144	111	105	105	105
129	136	113	121	88	90	88	88	88	88	88	87	88	88	88	88	88	88	88	88	88	88	88	87	132	134	134	134	132	132	134	134	134	134	132	134	130	117	98	98	98	98	79	118	90	121	112	134	118	110	110	110
126	127	105	126	95	96	95	95	95	95	95	94	95	95	95	95	95	95	95	95	95	95	95	94	128	128	128	128	128	128	128	128	128	128	128	128	119	119	88	88	88	88	96	110	98	124	104	134	110	104	104	104
132	141	96	115	99	99	99	99	99	99	99	98	99	99	99	99	99	99	99	99	99	99	99	99	121	117	117	117	121	121	118	117	117	117	121	117	118	132	107	107	107	107	98	132	96	121	112	141	123	111	111	111
121	142	105	119	75	77	75	75	75	75	75	76	75	75	75	75	75	75	75	75	75	75	75	74	132	131	131	131	132	132	128	131	131	131	132	131	115	118	82	82	82	82	76	120	71	122	99	138	118	98	98	98
127	138	99	119	107	108	107	107	107	107	107	107	107	107	107	107	107	107	107	107	107	107	107	106	126	127	127	127	126	126	125	127	127	127	126	127	114	126	90	90	90	90	93	121	95	126	79	146	108	78	78	78
122	145	95	126	103	105	103	103	103	103	103	104	103	103	103	103	103	103	103	103	103	103	103	102	129	126	126	126	129	129	124	126	126	126	129	126	118	128	93	93	93	93	96	118	94	127	100	140	109	99	99	99
124	140	107	125	84	85	84	84	84	84	84	85	84	84	84	84	84	84	84	84	84	84	84	83	136	132	132	132	136	136	128	132	132	132	136	132	119	122	95	95	95	95	93	116	96	119	83	146	118	81	81	81
133	128	125	137	96	98	96	96	96	96	96	96	96	96	96	96	96	96	96	96	96	96	96	95	141	142	142	142	141	141	137	142	142	142	141	142	133	116	126	126	126	126	107	112	102	130	108	118	132	107	107	107
128	135	119	129	75	77	75	75	75	75	75	75	75	75	75	75	75	75	75	75	75	75	75	75	135	133	133	133	135	135	130	133	133	133	135	133	128	110	109	109	109	109	98	94	99	125	86	130	128	84	84	84
126	128	107	119	92	93	92	92	92	92	92	93	92	92	92	92	92	92	92	92	92	92	92	91	133	137	137	137	133	133	130	137	137	137	133	137	132	114	107	107	107	107	113	109	105	124	90	130	119	88	88	88
133	139	124	117	91	92	91	91	91	91	91	91	91	91	91	91	91	91	91	91	91	91	91	90	133	139	139	139	133	133	136	139	139	139	133	139	126	123	113	113	113	113	98	106	104	125	72	136	119	70	70	70
126	140	93	110	99	101	99	99	99	99	99	100	99	99	99	99	99	99	99	99	99	99	99	99	124	127	127	127	124	124	125	127	127	127	124	127	119	126	93	93	93	93	89	126	81	122	102	143	103	100	100	100
129	132	126	126	109	109	109	109	109	109	109	109	109	109	109	109	109	109	109	109	109	109	109	108	130	130	130	130	130	130	125	130	130	130	130	130	112	130	124	124	124	124	118	117	120	109	103	128	124	102	102	102
131	137	131	135	88	90	88	88	88	88	88	88	88	88	88	88	88	88	88	88	88	88	88	89	129	129	129	129	129	129	130	129	129	129	129	129	116	119	107	107	107	107	112	90	113	121	105	128	118	104	104	104
127	138	122	130	85	87	85	85	85	85	85	85	85	85	85	85	85	85	85	85	85	85	85	86	136	135	135	135	136	136	132	135	135	135	136	135	119	111	118	118	118	118	106	99	111	121	91	120	120	90	90	90
142	136	108	125	101	101	101	101	101	101	101	101	101	101	101	101	101	101	101	101	101	101	101	102	130	129	129	129	130	130	132	129	129	129	130	129	125	128	106	106	106	106	120	94	115	128	97	131	113	95	95	95
124	139	84	105	93	94	93	93	93	93	93	93	93	93	93	93	93	93	93	93	93	93	93	93	122	120	120	120	122	122	120	120	120	120	122	120	122	116	101	101	101	101	83	110	87	113	109	143	114	108	108	108
121	143	118	122	74	76	74	74	74	74	74	75	74	74	74	74	74	74	74	74	74	74	74	73	131	131	131	131	131	131	127	131	131	131	131	131	122	124	102	102	102	102	100	101	99	119	88	141	125	87	87	87
134	136	119	134	111	112	111	111	111	111	111	111	111	111	111	111	111	111	111	111	111	111	111	110	134	133	133	133	134	134	134	133	133	133	134	133	131	121	118	118	118	118	122	93	113	138	80	135	117	78	78	78
129	133	120	128	102	103	102	102	102	102	102	102	102	102	102	102	102	102	102	102	102	102	102	102	136	139	139	139	136	136	131	139	139	139	136	139	117	121	114	114	114	114	115	104	113	128	94	131	129	92	92	92
122	127	123	127	102	103	102	102	102	102	102	102	102	102	102	102	102	102	102	102	102	102	102	103	127	128	129	128	127	127	126	128	128	128	127	128	116	113	110	110	110	110	109	117	109	123	87	129	125	85	85	85
130	139	98	120	100	100	100	100	100	100	100	100	100	100	100	100	100	100	100	100	100	100	100	101	129	129	129	129	129	129	123	129	129	129	129	129	109	125	98	98	98	98	100	110	101	121	114	137	113	112	112	112
139	86	145	143	125	126	125	125	125	125	125	125	125	125	125	125	125	125	125	125	125	125	125	126	145	145	145	145	145	145	141	145	145	145	145	145	141	101	128	128	128	128	129	107	138	141	126	92	141	125	125	125
131	126	129	142	67	68	67	67	67	67	67	67	67	67	67	67	67	67	67	67	67	67	67	68	139	137	137	137	139	139	143	137	137	137	139	137	128	120	105	105	105	105	104	93	101	132	89	129	135	87	87	87
135	137	101	125	97	98	97	97	97	97	97	97	97	97	97	97	97	97	97	97	97	97	97	96	131	130	130	130	131	131	131	130	130	130	131	130	119	122	101	101	101	101	94	124	94	127	87	139	116	86	86	86
125	142	107	114	87	89	87	87	87	87	87	88	87	87	87	87	87	87	87	87	87	87	87	86	128	129	129	129	128	128	129	129	129	129	128	129	118	127	118	118	118	118	90	116	84	117	89	139	120	87	87	87
130	138	100	111	100	101	100	100	100	100	100	100	100	100	100	100	100	100	100	100	100	100	100	101	127	125	125	125	127	127	125	125	125	125	127	125	123	127	97	97	97	97	88	120	91	114	108	136	120	108	108	108
133	141	100	104	113	113	113	113	113	113	113	113	113	113	113	113	113	113	113	113	113	113	113	112	125	122	122	122	125	125	124	122	122	122	125	122	124	128	108	108	108	108	96	128	93	117	97	143	116	97	97	97
120	143	100	116	110	108	110	110	110	110	110	110	110	110	110	110	110	110	110	110	110	110	110	109	122	126	126	126	122	122	127	126	126	126	122	126	121	126	100	100	100	100	91	120	92	128	91	144	120	89	89	89
127	131	108	125	98	99	98	98	98	98	98	99	98	98	98	98	98	98	98	98	98	98	98	97	131	132	132	132	131	131	130	132	132	132	131	132	124	125	96	96	96	96	101	124	92	129	91	133	118	89	89	89
121	148	102	114	89	89	89	89	89	89	89	90	89	89	89	89	89	89	89	89	89	89	89	88	124	128	128	128	124	124	126	128	128	128	124	128	123	123	86	86	86	86	88	120	85	117	108	144	123	106	106	106
129	133	103	128	100	100	100	100	100	100	100	101	100	100	100	100	100	100	100	100	100	100	100	99	125	122	122	122	125	125	121	122	122	122	125	122	122	131	107	107	107	107	115	110	105	129	106	139	112	104	104	104
128	141	119	132	73	74	73	73	73	73	73	72	73	73	73	73	73	73	73	73	73	73	73	72	134	134	134	134	134	134	130	134	134	134	134	134	127	121	102	102	102	102	96	107	100	124	89	143	119	88	88	88
138	116	141	139	115	116	115	115	115	115	115	115	115	115	115	115	115	115	115	115	115	115	115	114	147	145	145	145	147	147	144	145	145	145	147	145	139	114	126	126	126	126	127	94	127	138	118	107	141	118	118	118

122	134	89	114	107	109	107	107	107	107	107	108	107	107	107	107	107	107	107	107	107	107	107	106	123	122	122	122	123	123	121	122	122	122	123	122	121	119	97	97	97	97	93	119	98	119	114	137	115	113	113	113
132	142	93	123	88	89	88	88	88	88	88	88	88	88	88	88	88	88	88	88	88	88	88	89	128	122	122	122	128	128	116	122	122	122	128	122	116	124	85	85	85	85	102	113	96	119	118	138	112	116	116	116
143	109	146	146	115	115	115	115	115	115	115	115	115	115	115	115	115	115	115	115	115	115	115	116	150	152	152	152	150	150	148	152	152	152	150	152	141	111	131	131	131	131	132	114	132	145	132	104	143	130	130	130
140	102	146	147	112	114	112	112	112	112	112	112	112	112	112	112	112	112	112	112	112	112	112	113	146	146	146	146	146	146	146	146	146	146	146	146	139	109	140	140	140	140	130	99	128	137	118	102	142	117	117	117
121	135	106	125	100	102	100	100	100	100	100	100	100	100	100	100	100	100	100	100	100	100	100	99	128	124	124	124	128	128	129	124	124	124	128	124	113	125	94	94	94	94	100	107	97	122	83	139	105	81	81	81
124	141	107	128	97	97	97	97	97	97	97	96	97	97	97	97	97	97	97	97	97	97	97	96	130	131	131	131	130	130	132	131	131	131	130	131	122	126	102	102	102	102	99	118	101	121	95	143	125	94	94	94
130	142	112	121	96	97	96	96	96	96	96	97	96	96	96	96	96	96	96	96	96	96	96	95	135	133	133	133	135	135	139	133	133	133	135	133	131	124	79	79	79	79	100	128	104	124	115	139	124	114	114	114
132	135	119	128	94	95	94	94	94	94	94	94	94	94	94	94	94	94	94	94	94	94	94	93	133	132	132	132	133	133	131	132	132	132	133	132	108	123	105	105	105	105	106	107	104	118	93	140	118	91	91	91
130	141	106	118	89	90	89	89	89	89	89	90	89	89	89	89	89	89	89	89	89	89	89	89	135	135	135	135	135	135	129	135	135	135	135	135	123	125	89	89	89	89	106	120	106	121	96	144	124	95	95	95
129	124	127	131	112	113	112	112	112	112	112	112	112	112	112	112	112	112	112	112	112	112	112	111	140	142	142	142	140	140	140	142	142	142	140	142	130	112	118	118	118	118	110	103	122	127	103	115	126	103	103	103
129	142	102	122	95	96	95	95	95	95	95	96	95	95	95	95	95	95	95	95	95	95	95	95	126	127	127	127	126	126	128	127	127	127	126	127	117	122	87	87	87	87	96	114	102	125	80	148	109	78	78	78
131	141	101	126	94	95	94	94	94	94	94	94	94	94	94	94	94	94	94	94	94	94	94	93	128	126	126	126	128	128	126	126	126	126	128	126	117	130	110	110	110	110	106	108	105	125	99	143	118	97	97	97
127	141	94	112	101	101	101	101	101	101	101	102	101	101	101	101	101	101	101	101	101	101	101	100	122	120	120	120	122	122	119	120	120	120	122	120	128	129	96	96	96	96	96	126	98	119	110	148	121	109	109	109

1	2	1	1	1	1	1	1	1	1	1	1	1	1	1	1	1	1	1	1	1	1	1	1	1	1	1	1	1	1	1	1	1	1	1	1	1	1	1	1	1	1	1	1	6	1	1	1	1	1	1	1
Uchimurasaki	Ujukitsu	Unzoki	USSR Tangelo	Willowleaf mandarin	Willowleaf mandarin	Yamabuki	Yamamikan	Yatsushiro	Yuge hyoukan	Youpi ju (Yuhikitsu)	Yuukunibu	Yuzu	'Akemi'	'Aki Marine'	'Aki Tangor'	'Allspice'	'Amaka'	'Ariake'	'Asumi'	'Aurastar'	'Awa Orange'	'Benibae'	'Benimadoka'	'Chandler pummelo'	'Ehimekashi 28'	'Encore'	'Fairchild'	'Fortune'	'Harehime'	'Hareyaka'	'Haruhi'	'Haruka'	'Harumi'	'Hayaka'	'Hayasaki'	'Himekoharu'	'Hiroshimakaken 11'	'Honey'	'Kanpei'	'Kara'	'Kincy mandarin'	'Kinnow mandarin'	'Kiyomi'	'Kuchinotsu-41'	'Lee'	May Pummelo'	'Mihaya'	'Mihocore'	'Mineola'	'Nankou'	'Nishinokaori'
A196	A197	A198	A199	A200	A201	A202	A203	A204	A205	A206	A207	A208	B001	B002	B003	B004	B005	B006	B007	B008	B009	B010	B011	B012	B013	B014	B015	B016	B017	B018	B019	B020	B021	B022	B023	B024	B025	B026	B027	B028	B029	B030	B031	B032	B033	B034	B035	B036	B037	B038	B039
0	0	0	0	0	0	0	0	0	0	0	0	0	1	0	0	0	0	0	0	0	0	0	0	0	0	0	0	0	0	0	0	0	0	0	0	0	0	0	1	0	0	0	0	0	0	0	0	0	0	1	1
140	85	125	88	135	132	58	92	108	85	139	23	141	112	116	97	97	131	110	123	131	109	120	133	135	122	131	111	118	106	133	121	113	124	127	136	106	119	130	117	109	131	126	107	105	140	105	109	112	101	118	121
80	130	136	114	147	144	120	107	143	113	143	123	143	132	130	132	135	134	141	142	114	136	140	86	87	135	140	139	137	132	140	133	135	140	139	88	144	130	143	138	133	137	142	129	139	141	106	138	134	138	133	133
131	87	128	86	145	136	98	87	132	79	140	94	137	119	115	121	118	133	111	126	132	119	118	130	127	120	130	122	127	119	138	130	127	131	136	130	123	119	135	130	118	134	135	115	118	127	98	131	127	116	120	133
65	135	143	111	143	143	134	137	141	120	143	122	144	130	132	131	142	133	134	137	121	140	146	58	59	138	137	133	130	140	143	136	138	141	141	69	143	131	141	136	131	131	134	131	138	137	89	136	138	138	136	131
142	115	119	131	90	94	140	130	98	137	104	130	135	115	101	115	122	112	114	109	150	118	96	148	143	109	97	105	113	111	97	113	111	103	91	143	111	104	109	110	98	98	104	118	129	98	143	108	117	110	116	120
133	130	141	129	133	131	128	133	147	126	128	126	131	128	132	134	130	138	136	120	136	130	127	129	130	133	131	129	128	136	130	126	140	130	137	125	134	125	131	131	137	140	131	133	132	132	130	136	133	133	137	133
150	60	103	99	122	121	100	113	78	95	128	102	136	93	102	84	101	109	106	115	145	105	111	139	137	97	115	95	101	79	112	110	115	99	105	146	83	113	121	99	87	116	113	71	94	120	108	93	90	95	91	95
145	114	103	115	123	112	120	129	117	117	134	115	140	116	117	120	120	115	117	120	139	113	110	140	142	120	109	128	120	124	112	110	116	112	108	141	114	118	115	107	118	114	112	109	113	120	132	131	119	122	116	118
144	119	137	132	86	82	131	132	121	134	95	133	132	114	95	107	112	100	74	97	149	131	83	146	145	91	91	69	79	102	90	125	115	86	106	145	116	91	100	90	116	84	94	99	136	68	143	103	116	108	96	95
144	119	137	132	86	82	131	132	121	134	95	133	132	114	95	107	112	100	74	97	149	131	83	146	145	91	91	69	79	102	90	125	115	86	106	145	116	91	100	90	116	84	94	99	136	68	143	103	116	108	96	95
144	119	137	132	86	82	131	132	121	134	95	133	132	114	95	107	112	100	74	97	149	131	83	146	145	91	91	69	79	102	90	125	115	86	106	145	116	91	100	90	116	84	94	99	136	68	143	103	116	108	96	95
144	119	137	132	86	82	131	132	121	134	95	133	132	114	95	107	112	100	74	97	149	131	83	146	145	91	91	69	79	102	90	125	115	86	106	145	116	91	100	90	116	84	94	99	136	68	143	103	116	108	96	95
138	128	129	137	73	74	137	138	110	144	64	131	130	109	115	119	120	102	114	97	149	119	99	143	140	109	89	99	104	117	84	113	107	101	96	146	112	106	87	100	111	91	92	126	131	98	146	104	104	114	114	120
150	112	126	134	84	65	129	119	115	134	90	131	135	100	99	103	77	84	92	98	152	112	92	150	146	101	78	81	98	105	71	111	103	92	96	147	106	100	88	96	99	88	82	109	126	78	136	93	106	94	91	99
141	105	110	101	137	131	87	109	121	108	137	102	130	118	120	112	108	130	113	119	135	111	121	136	135	122	127	114	120	119	137	111	115	130	125	131	115	123	131	126	118	133	127	118	98	134	123	121	124	113	123	121
144	128	133	136	68	72	145	134	114	145	75	134	140	103	99	123	120	98	109	81	151	114	92	147	141	101	87	90	85	98	70	115	115	72	85	144	111	94	101	88	102	88	92	113	133	62	146	95	103	92	111	106
144	128	133	136	68	72	145	134	114	145	75	134	140	103	99	123	120	98	109	81	151	114	92	147	141	101	87	90	85	98	70	115	115	72	85	144	111	94	101	88	102	88	92	113	133	62	146	95	103	92	111	106
69	133	143	126	154	147	136	131	142	126	150	131	144	137	141	141	142	139	143	147	121	144	150	72	77	146	142	144	143	151	151	136	141	152	147	68	146	139	140	145	140	141	143	140	141	145	105	138	140	148	144	141
150	116	115	144	96	100	134	134	109	128	101	125	130	127	123	123	125	118	131	123	149	107	105	146	146	118	107	112	122	115	111	105	106	119	102	147	105	113	104	111	113	109	112	124	131	118	142	117	112	123	116	125
145	123	111	127	117	113	126	139	133	137	116	130	120	128	125	122	135	123	138	124	142	114	120	142	138	128	113	115	119	127	117	113	104	128	124	138	120	125	112	128	115	124	121	126	115	130	138	121	116	125	127	130
124	100	110	108	122	118	103	110	101	107	126	106	136	106	113	92	103	110	103	110	129	116	114	135	129	107	115	104	107	109	120	113	111	109	110	133	118	114	122	108	109	113	120	106	111	120	111	105	110	97	96	102
142	128	130	132	70	70	146	133	115	143	72	135	137	116	112	121	120	87	119	96	148	117	96	141	137	109	78	101	90	120	87	112	113	95	97	145	114	104	90	100	101	82	85	122	134	84	138	95	107	111	109	123
147	128	99	128	107	111	129	129	126	130	117	126	128	130	117	131	125	113	124	127	148	109	115	148	142	122	108	122	122	115	114	111	88	121	119	148	114	118	108	119	121	111	111	127	121	122	141	126	122	120	125	124
121	107	129	91	139	125	96	72	121	105	145	96	142	126	110	106	99	117	112	128	126	118	122	118	121	125	126	114	120	134	135	134	119	133	130	123	121	114	130	123	119	128	123	123	122	133	102	122	131	97	111	129
121	107	129	91	139	125	96	72	121	105	145	96	142	126	110	106	99	117	112	128	126	118	122	118	121	125	126	114	120	134	135	134	119	133	130	123	121	114	130	123	119	128	123	123	122	133	102	122	131	97	111	129
121	107	129	91	139	125	96	72	121	105	145	96	142	126	110	106	99	117	112	128	126	118	122	118	121	125	126	114	120	134	135	134	119	133	130	123	121	114	130	123	119	128	123	123	122	133	102	122	131	97	111	129
152	132	136	142	126	125	142	149	142	145	115	141	92	141	135	138	138	132	143	137	148	134	130	151	149	133	122	136	133	137	126	129	124	134	135	149	126	136	118	138	131	129	127	138	138	134	148	136	129	135	143	140
128	90	127	94	145	135	97	91	120	85	141	85	139	113	118	117	110	134	112	126	122	111	125	129	124	126	135	116	120	115	132	121	120	128	130	135	117	123	132	124	119	135	134	108	110	122	84	123	124	111	120	127
146	118	125	136	128	114	131	126	123	137	125	138	93	111	117	124	121	126	132	120	142	119	116	150	146	133	120	126	126	132	119	123	124	117	115	146	127	125	125	123	122	127	121	119	127	125	137	134	125	121	126	126
146	117	122	125	130	119	127	135	118	137	119	128	107	124	124	121	121	122	131	127	141	124	113	146	142	120	113	123	128	124	121	123	118	124	125	144	127	125	106	129	103	118	119	127	122	132	133	124	121	134	122	133
146	118	127	142	81	86	141	144	118	133	85	128	134	108	114	113	116	107	110	90	153	111	89	147	147	107	91	99	98	114	69	111	118	83	92	144	115	105	86	92	111	113	83	116	131	86	135	109	104	103	115	110
73	134	142	114	143	142	130	127	137	132	148	127	145	131	136	135	133	139	142	143	118	140	149	55	52	144	137	138	137	142	142	137	138	143	143	53	144	136	142	137	137	136	132	137	140	143	83	137	135	139	136	138
109	106	129	100	135	132	111	101	140	100	137	104	136	108	118	117	121	134	119	120	126	125	131	114	109	131	133	114	114	126	130	130	125	122	133	118	124	118	136	118	126	133	133	117	129	123	108	128	120	113	119	130
139	0	114	102	126	117	91	106	106	76	129	98	127	105	106	98	102	118	112	124	136	118	114	135	138	111	123	96	99	103	119	117	113	106	118	139	105	115	116	111	99	125	120	90	105	125	99	103	105	87	104	112
120	97	128	91	144	138	91	90	116	90	141	99	138	124	113	108	118	123	132	136	124	120	129	122	121	128	138	116	123	122	135	130	127	134	128	125	121	115	136	132	113	136	139	118	118	139	108	125	125	119	122	136
143	105	93	111	134	128	101	122	110	113	135	111	139	107	119	109	106	123	121	122	140	91	120	141	139	119	130	111	120	108	128	100	93	124	119	139	107	124	127	120	106	126	121	113	0	134	126	119	120	111	122	119
143	105	93	111	134	128	101	122	110	113	135	111	139	107	119	109	106	123	121	122	140	91	120	141	139	119	130	111	120	108	128	100	93	124	119	139	107	124	127	120	106	126	121	113	0	134	126	119	120	111	122	119
143	105	93	111	134	128	101	122	110	113	135	111	139	107	119	109	106	123	121	122	140	91	120	141	139	119	130	111	120	108	128	100	93	124	119	139	107	124	127	120	106	126	121	113	0	134	126	119	120	111	122	119
144	105	94	112	134	128	102	123	110	113	135	111	139	107	120	109	106	124	121	122	140	91	120	142	140	119	130	111	120	108	128	100	94	124	119	140	107	125	127	120	106	126	121	113	1	134	126	119	120	111	122	119
143	105	93	111	134	128	101	122	110	113	135	111	139	107	119	109	106	123	121	122	140	91	120	141	139	119	130	111	120	108	128	100	93	124	119	139	107	124	127	120	106	126	121	113	0	134	126	119	120	111	122	119
140	137	143	135	132	135	140	138	142	139	127	138	112	143	135	137	139	134	136	138	143	134	142	136	140	138	131	134	138	144	135	133	129	138	138	136	133	128	129	133	137	131	133	143	141	135	138	141	137	137	138	139
133	129	135	127	139	139	129	127	138	126	137	128	115	131	134	133	131	141	137	139	126	133	141	131	128	143	140	136	137	135	138	142	126	138	142	126	136	137	136	134	135	140	137	136	124	136	133	137	138	127	139	137
142	95	127	99	125	121	79	83	104	112	131	70	138	109	112	102	97	122	114	126	132	116	118	143	138	116	129	112	119	115	131	131	122	126	119	139	120	116	129	121	116	119	124	110	116	123	121	109	118	90	114	123
142	95	126	98	125	121	77	83	102	112	131	70	138	109	111	101	97	120	114	125	132	116	117	143	138	115	129	112	119	114	131	131	122	125	117	139	120	115	129	120	115	119	124	109	116	123	121	108	116	89	113	122
142	120	124	140	130	128	135	137	127	137	129	135	116	117	124	122	135	127	137	123	132	125	126	147	140	132	124	137	128	130	124	125	134	124	121	143	130	127	126	130	112	130	133	120	132	135	137	124	119	117	122	130
134	106	119	121	133	126	112	121	126	116	133	117	113	126	129	121	114	126	124	137	130	129	125	129	126	130	127	118	125	125	130	131	117	130	136	133	130	128	128	116	123	122	126	121	123	132	127	123	130	122	124	128
142	117	132	130	125	114	131	129	128	130	130	138	98	117	118	122	129	128	128	117	142	123	121	143	143	124	125	118	121	129	120	122	121	121	117	138	124	123	120	125	124	126	117	116	131	127	135	133	126	121	124	132
152	112	52	135	121	113	131	137	106	121	119	131	137	122	134	132	113	124	132	127	145	111	110	149	147	123	125	120	121	119	124	119	120	123	113	148	118	135	122	123	115	122	119	124	98	118	132	123	118	122	125	122
101	110	127	97	148	141	100	107	136	94	144	101	142	119	129	123	134	140	133	131	121	119	140	111	108	126	138	127	127	122	135	121	131	130	131	110	124	135	140	126	123	138	139	107	124	134	96	133	122	125	125	122
141	100	104	118	127	109	110	123	98	102	128	115	140	100	119	114	102	105	115	122	138	108	114	134	133	118	114	118	120	117	119	117	105	120	100	136	108	121	114	109	108	113	116	114	105	122	113	112	115	117	110	113
101	95	134	93	138	132	101	110	129	79	136	112	137	118	116	118	118	127	127	132	120	130	135	103	109	130	135	126	119	138	135	125	119	134	132	102	127	119	136	121	122	133	136	127	119	136	111	126	127	124	126	132
148	102	84	129	116	111	133	130	105	121	123	126	129	118	121	118	116	113	129	128	146	115	110	144	142	117	106	120	127	113	105	111	98	120	120	145	102	123	104	112	111	116	111	119	116	129	119	113	119	118	115	121
138	124	115	121	133	133	118	131	125	121	131	120	121	128	139	125	127	136	134	131	133	117	136	139	138	136	136	134	131	131	134	130	127	134	128	135	133	141	136	134	126	139	139	127	102	134	130	130	132	125	130	137
138	106	103	118	101	93	121	122	102	119	102	122	133	99	103	117	96	89	109	115	137	118	90	137	137	100	76	86	102	103	88	114	99	96	103	143	107	112	80	106	74	84	80	108	111	103	124	91	106	102	97	109
118	98	132	92	136	130	100	95	119	98	133	109	134	114	119	118	113	133	127	130	127	114	131	122	113	123	125	115	124	119	127	114	119	130	126	120	117	125	133	122	116	127	131	113	114	128	96	120	126	119	123	119
140	87	127	89	135	132	60	93	110	87	139	25	141	112	117	99	99	132	112	124	131	110	121	133	135	123	131	112	119	108	133	121	114	124	128	136	107	119	130	118	111	131	126	109	107	140	106	111	113	103	120	123

150	117	116	135	79	74	141	134	86	137	82	124	131	116	116	124	119	94	127	106	153	112	94	152	147	113	87	106	108	110	90	100	112	104	74	152	90	111	85	103	98	94	95	118	128	97	144	96	98	118	108	119
150	117	116	135	80	75	141	134	86	137	83	124	131	116	116	124	119	95	127	106	153	112	95	152	147	113	88	106	108	111	90	101	112	104	74	152	90	112	86	103	98	95	95	118	128	97	144	97	99	118	109	119
150	117	116	135	79	74	141	134	86	137	82	124	131	116	116	124	119	94	127	106	153	112	94	152	147	113	87	106	108	110	90	100	112	104	74	152	90	111	85	103	98	94	95	118	128	97	144	96	98	118	108	119
150	117	116	135	79	74	141	134	86	137	82	124	131	116	116	124	119	94	127	106	153	112	94	152	147	113	87	106	108	110	90	100	112	104	74	152	90	111	85	103	98	94	95	118	128	97	144	96	98	118	108	119
150	117	116	135	79	74	141	134	86	137	82	124	131	116	116	124	119	94	127	106	153	112	94	152	147	113	87	106	108	110	90	100	112	104	74	152	90	111	85	103	98	94	95	118	128	97	144	96	98	118	108	119
150	117	116	135	79	74	141	134	86	137	82	124	131	116	116	124	119	94	127	106	153	112	94	152	147	113	87	106	108	110	90	100	112	104	74	152	90	111	85	103	98	94	95	118	128	97	144	96	98	118	108	119
150	117	116	135	79	74	141	134	86	137	82	124	131	116	116	124	119	94	127	106	153	112	94	152	147	113	87	106	108	110	90	100	112	104	74	152	90	111	85	103	98	94	95	118	128	97	144	96	98	118	108	119
150	117	116	135	79	74	141	134	86	137	82	124	131	116	116	124	119	94	127	106	153	112	94	152	147	113	87	106	108	110	90	100	112	104	74	152	90	111	85	103	98	94	95	118	128	97	144	96	98	118	108	119
150	117	116	135	79	74	141	134	86	137	82	124	131	116	116	124	119	94	127	106	153	112	94	152	147	113	87	106	108	110	90	100	112	104	74	152	90	111	85	103	98	94	95	118	128	97	144	96	98	118	108	119
150	117	116	135	79	74	141	134	86	137	82	124	131	116	116	124	119	94	127	106	153	112	94	152	147	113	87	106	108	110	90	100	112	104	74	152	90	111	85	103	98	94	95	118	128	97	144	96	98	118	108	119
150	117	116	135	79	74	141	134	86	137	82	124	131	116	116	124	119	94	127	106	153	112	94	152	147	113	87	106	108	110	90	100	112	104	74	152	90	111	85	103	98	94	95	118	128	97	144	96	98	118	108	119
150	117	116	135	79	74	141	134	86	137	82	124	131	116	116	124	119	94	127	106	153	112	94	152	147	113	87	106	108	110	90	100	112	104	74	152	90	111	85	103	98	94	95	118	128	97	144	96	98	118	108	119
150	117	116	135	79	74	141	134	86	137	82	124	131	116	116	124	119	94	127	106	153	112	94	152	147	113	87	106	108	110	90	100	112	104	74	152	90	111	85	103	98	94	95	118	128	97	144	96	98	118	108	119
150	117	116	135	79	74	141	134	86	137	82	124	131	116	116	124	119	94	127	106	153	112	94	152	147	113	87	106	108	110	90	100	112	104	74	152	90	111	85	103	98	94	95	118	128	97	144	96	98	118	108	119
150	117	116	135	79	74	141	134	86	137	82	124	131	116	116	124	119	94	127	106	153	112	94	152	147	113	87	106	108	110	90	100	112	104	74	152	90	111	85	103	98	94	95	118	128	97	144	96	98	118	108	119
150	117	116	135	79	74	141	134	86	137	82	124	131	116	116	124	119	94	127	106	153	112	94	152	147	113	87	106	108	110	90	100	112	104	74	152	90	111	85	103	98	94	95	118	128	97	144	96	98	118	108	119
146	118	125	136	128	114	131	126	123	137	125	138	93	111	117	124	121	126	132	120	142	119	116	150	146	133	120	126	126	132	119	123	124	117	115	146	127	125	125	123	122	127	121	119	127	125	137	134	125	121	126	126
147	120	125	138	82	85	135	137	117	142	95	133	135	109	103	119	121	98	112	113	152	123	99	150	147	104	94	111	116	116	82	113	114	92	101	149	103	102	97	105	107	93	89	114	131	103	148	108	106	115	120	113
148	129	100	133	111	116	127	131	122	129	111	128	128	130	130	129	129	124	119	119	146	105	113	146	147	121	120	121	119	126	115	113	110	127	107	143	116	127	117	116	125	123	122	126	122	117	144	128	124	125	124	121
148	128	99	132	110	115	128	131	122	129	112	129	128	131	128	130	130	125	120	120	146	106	114	146	147	119	121	122	118	126	116	113	111	126	107	143	114	126	118	115	125	122	123	124	122	116	144	128	124	125	125	120
145	97	123	107	132	127	91	106	118	106	132	104	132	118	114	109	96	126	112	117	136	115	118	139	136	121	121	116	121	117	124	124	124	115	122	138	121	118	120	125	118	129	123	113	113	126	118	125	125	108	114	123
152	131	128	138	125	122	130	141	132	140	119	133	95	136	131	135	134	128	142	130	151	129	126	149	150	138	118	132	131	140	124	127	125	132	122	149	126	129	118	134	130	132	127	139	133	133	143	135	128	136	136	143
146	93	126	100	127	128	86	92	116	96	132	97	132	117	120	109	100	128	112	117	139	126	115	134	139	114	129	113	120	111	127	131	110	118	135	139	121	123	124	120	108	126	126	108	116	123	119	118	117	103	120	118
150	60	103	99	122	121	100	113	78	95	128	102	136	93	102	84	101	109	106	115	145	105	111	139	137	97	115	95	101	79	112	110	115	99	105	146	83	113	121	99	87	116	113	71	94	120	108	93	90	95	91	95
145	125	104	132	106	107	130	132	127	132	116	126	123	126	122	128	123	115	127	120	146	110	115	137	137	122	112	110	122	122	117	109	95	122	121	140	110	114	106	108	122	112	116	129	121	122	138	122	117	124	130	121
150	61	104	100	122	121	100	114	79	95	128	102	136	93	103	84	101	109	106	115	145	105	111	139	137	98	115	95	101	80	112	110	115	99	106	146	84	113	121	99	87	116	113	72	95	120	108	93	90	96	91	95
150	60	104	99	122	121	100	113	79	95	128	102	136	94	103	85	102	109	106	115	145	106	111	139	137	97	115	95	101	79	112	110	116	99	105	146	84	113	121	99	88	116	114	72	95	120	108	93	90	95	91	95
142	135	144	138	139	137	135	142	144	131	133	138	133	143	145	144	143	140	142	142	140	142	142	141	142	144	139	133	135	147	138	134	137	142	144	138	138	136	138	139	146	141	142	143	137	136	143	141	142	133	148	145
140	121	137	124	136	132	119	134	134	124	129	122	130	133	137	132	120	129	132	132	142	136	131	138	138	132	124	123	127	142	131	127	138	134	140	137	132	132	122	131	137	136	128	133	132	129	128	129	129	126	137	138
152	127	144	141	132	129	131	142	136	138	121	136	130	133	140	135	134	134	139	136	152	144	136	152	153	139	130	129	133	143	135	135	138	137	132	154	135	139	120	138	135	136	136	135	143	134	142	137	131	126	140	146
61	135	143	124	148	147	132	132	148	125	149	126	145	136	134	137	148	141	141	146	124	141	146	49	77	140	145	140	136	146	148	134	132	150	149	53	150	131	142	142	140	139	145	141	142	141	100	141	142	141	141	139
137	148	150	150	147	147	152	154	155	145	140	153	142	153	156	152	154	153	153	151	139	148	151	141	142	151	149	146	147	157	149	146	147	153	153	143	154	150	140	153	152	150	149	157	149	149	147	150	150	150	152	156
147	130	134	134	135	128	129	131	131	142	136	135	131	137	132	130	131	130	142	140	144	137	136	150	145	135	129	131	136	138	133	132	130	135	132	150	130	130	125	140	132	132	135	132	135	139	141	135	133	125	133	143
149	117	117	132	129	124	134	135	110	131	131	134	107	126	123	124	119	126	135	130	142	125	121	148	145	124	127	123	132	120	123	129	113	122	121	148	119	130	121	121	108	127	125	125	119	134	137	127	130	126	125	129
146	116	121	123	92	89	129	125	113	127	102	122	137	95	90	125	111	88	104	97	147	110	78	141	140	90	74	97	102	100	73	108	112	88	96	137	103	87	86	104	92	92	66	108	114	81	132	103	103	102	114	106
137	103	131	102	133	127	97	106	132	105	136	100	127	117	116	108	106	128	106	117	130	118	121	138	134	128	128	110	116	122	130	117	116	125	132	136	120	122	128	119	115	131	133	112	116	123	121	118	121	111	116	121
146	117	122	125	130	119	127	135	118	137	119	128	107	124	124	121	121	122	131	127	141	124	113	146	142	120	113	123	128	124	121	123	118	124	125	144	127	125	106	129	103	118	119	127	122	132	133	124	121	134	122	133
140	82	125	95	137	129	76	99	109	81	141	71	138	107	118	103	101	131	112	122	130	123	124	128	129	125	131	114	117	111	129	130	112	118	123	130	115	121	129	113	110	131	126	103	110	132	111	111	115	104	110	116
133	82	127	95	132	124	90	92	113	76	143	90	135	104	110	95	97	130	104	118	134	116	121	137	138	115	128	107	117	107	124	126	101	115	119	138	108	113	132	110	103	129	124	96	109	124	103	112	111	101	112	114
133	82	127	95	132	124	90	92	113	76	143	90	135	104	110	95	97	130	104	118	134	116	121	137	138	115	128	107	117	107	124	126	101	115	119	138	108	113	132	110	103	129	124	96	109	124	103	112	111	101	112	114
133	82	127	95	132	124	90	92	113	76	143	90	135	104	110	95	97	130	104	118	134	116	121	137	138	115	128	107	117	107	124	126	101	115	119	138	108	113	132	110	103	129	124	96	109	124	103	112	111	101	112	114
133	82	127	95	132	124	90	92	113	76	143	90	135	104	110	95	97	130	104	118	134	116	121	137	138	115	128	107	117	107	124	126	101	115	119	138	108	113	132	110	103	129	124	96	109	124	103	112	111	101	112	114
139	107	127	129	89	93	126	126	97	124	104	117	135	104	107	103	119	113	126	104	150	106	110	144	140	110	110	106	104	111	109	103	120	107	91	141	106	107	106	118	96	117	104	107	129	115	130	100	99	100	111	119
143	108	112	112	118	120	112	106	121	115	111	119	133	112	108	119	103	126	114	112	140	107	108	147	144	127	122	110	122	109	120	117	116	118	117	142	100	117	123	126	105	132	116	118	103	118	131	120	119	103	120	119
82	123	139	97	145	143	119	92	142	109	146	125	139	134	131	133	134	140	134	143	123	129	144	93	92	138	141	140	141	141	143	134	127	149	144	92	142	129	145	140	132	135	139	139	128	147	103	137	145	138	138	141
131	107	127	101	140	130	108	98	115	100	134	105	144	110	118	113	114	124	118	127	131	116	125	138	133	129	135	118	119	113	137	129	121	136	121	138	126	123	137	125	119	123	138	106	116	119	117	124	122	110	123	128
148	108	130	106	128	124	85	100	102	103	126	94	135	130	122	110	115	131	119	131	141	125	127	139	142	128	131	116	130	108	134	122	111	132	118	140	111	118	129	128	124	122	136	115	118	127	118	125	127	120	124	126
152	120	132	139	82	75	138	132	120	141	87	139	134	108	111	124	114	108	116	95	156	120	101	151	148	107	97	98	93	117	75	117	117	79	84	153	117	96	93	80	119	100	93	112	127	70	148	118	110	93	119	113
152	120	132	139	82	75	138	132	120	141	87	139	134	108	111	124	114	108	116	95	156	120	101	151	148	107	97	98	93	117	75	117	117	79	84	153	117	96	93	80	119	100	93	112	127	70	148	118	110	93	119	113
152	120	132	139	82	75	138	132	120	141	87	139	134	108	111	124	114	108	116	95	156	120	101	151	148	107	97	98	93	117	75	117	117	79	84	153	117	96	93	80	119	100	93	112	127	70	148	118	110	93	119	113
152	120	132	139	82	75	138	132	120	141	87	139	134	108	111	124	114	108	116	95	156	120	101	151	148	107	97	98	93	117	75	117	117	79	84	153	117	96	93	80	119	100	93	112	127	70	148	118	110	93	119	113
142	113	130	129	91	95	130	132	130	133	94	124	137	101	104	112	114	103	105	100	146	111	94	149	145	114	99	94	95	109	98	109	118	81	103	144	107	101	92	101	116	100	97	109	122	92	134	99	95	98	111	108
61	137	140	119	147	148	133	130	145	130	149	129	146	135	136	134	142	138	140	145	118	136	149	61	50	144	145	145	140	144	145	131	142	150	146	66	150	134	139	141	141	143	143	139	137	143	95	143	139	142	142	139
145	123	111	127	117	113	126	139	133	137	116	130	120	128	125	122	135	123	138	124	142	114	120	142	138	128	113	115	119	127	117	113	104	128	124	138	120	125	112	128	115	124	121	126	115	130	138	121	116	125	127	130
132	77	117	90	140	131	67	110	100	90	138	77	134	117	120	96	101	123	110	121	128	120	127	135	136	124	132	104	111	119	129	115	102	131	120	135	108	120	131	122	107	131	133	116	101	137	103	113	112	102	107	118
149	91	100	113	119	112	106	120	75	111	122	101	137	88	95	67	108	89	112	112	142	110	107	142	140	103	104	103	110	80	120	102	107	113	97	145	86	96	105	106	72	110	110	87	104	126	117	79	73	91	84	102
150	92	100	114	119	112	106	120	76	112	122	102	137	89	96	68	108	89	112	112	143	110	107	142	140	104	104	104	110	81	120	102	108	113	98	145	87	97	105	106	72	110	110	88	104	127	118	79	74	92	85	103
149	91	100	113	119	112	106	120	75	111	122	101	137	88	95	67	108	89	112	112	142	110	107	142	140	103	104	103	110	80	120	102	107	113	97	145	86	96	105	106	72	110	110	87	104	126	117	79	73	91	84	102
149	91	100	113	119	112	106	120	75	111	122	101	137	88	95	67	108	89	112	112	142	110	107	142	140	103	104	103	110	80	120	102	107	113	97	145	86	96	105	106	72	110	110	87	104	126	117	79	73	91	84	102
149	91	100	113	119	112	106	120	75	111	122	101	137	88	95	67	108	89	112	112	142	110	107	142	140	103	104	103	110	80	120	102	107	113	97	145	86	96	105	106	72	110	110	87	104	126	117	79	73	91	84	102
149	91	100	113	119	112	106	120	75	111	122	101	137	88	95	67	108	89	112	112	142	110	107	142	140	103	104	103	110	80	120	102	107	113	97	145	86	96	105	106	72	110	110	87	104	126	117	79	73	91	84	102
149	91	100	113	119	112	106	120	75	111	122	101	137	88	95	67	108	89	112	112	142	110	107	142	140	103	104	103	110	80	120	102	107	113	97	145	86	96	105	106	72	110	110	87	104	126	117	79	73	91	84	102
149	91	100	113	119	112	106	120	75	111	122	101	137	88	95	67	108	89	112	112	142	110	107	142	140	103	104	103	110	80	120	102	107	113	97	145	86	96	105	106	72	110	110	87	104	126	117	79	73	91	84	102
149	91	100	113	119	112	106	120	75	111	122	101	137	88	95	67	108	89	112	112	142	110	107	142	140	103	104	103	110	80	120	102	107	113	97	145	86	96	105	106	72	110	110	87	104	126	117	79	73	91	84	102
149	91	100	113	119	112	106	120	75	111	122	101	137	88	95	67	108	89	112	112	142	110	107	142	140	103	104	103	110	80	120	102	107	113	97	145	86	96	105	106	72	110	110	87	104	126	117	79	73	91	84	102
149	91	100	113	119	112	106	120	75	111	122	101	137	88	95	67	108	89	112	112	142	110	107	142	140	103	104	103	110	80	120	102	107	113	97	145	86	96	105	106	72	110	110	87	104	126	117	79	73	91	84	102
149	91	100	113	119	112	106	120	75	111	122	101	137	88	95	67	108	89	112	112	142	110	107	142	140	103	104	103	110	80	120	102	107	113	97	145	86	96	105	106	72	110	110	87	104	126	117	79	73	91	84	102
149	91	100	113	119	112	106	120	75	111	122	101	137	88	95	67	108	89	112	112	142	110	107	142	140	103	104	103	110	80	120	102	107	113	97	145	86	96	105	106	72	110	110	87	104	126	117	79	73	91	84	102
149	91	100	113	119	112	106	120	75	111	122	101	137	88	95	67	108	89	112	112	142	110	107	142	140	103	104	103	110	80	120	102	107	113	97	145	86	96	105	106	72	110	110	87	104	126	117	79	73	91	84	102
149	91	100	113	119	112	106	120	75	111	122	101	137	88	95	67	108	89	112	112	142	110	107	142	140	103	104	103	110	80	120	102	107	113	97	145	86	96	105	106	72	110	110	87	104	126	117	79	73	91	84	102
149	91	100	113	119	112	106	120	75	111	122	101	137	88	95	67	108	89	112	112	142	110	107	142	140	103	104	103	110	80	120	102	107	113	97	145	86	96	105	106	72	110	110	87	104	126	117	79	73	91	84	102
149	91	100	113	119	112	106	120	75	111	122	101	137	88	95	67	108	89	112	112	142	110	107	142	140	103	104	103	110	80	120	102	107	113	97	145	86	96	105	106	72	110	110	87	104	126	117	79	73	91	84	102
149	91	100	113	119	112	106	120	75	111	122	101	137	88	95	67	108	89	112	112	142	110	107	142	140	103	104	103	110	80	120	102	107	113	97	145	86	96	105	106	72	110	110	87	104	126	117	79	73	91	84	102
149	91	100	113	119	112	106	120	75	111	122	101	137	88	95	67	108	89	112	112	142	110	107	142	140	103	104	103	110	80	120	102	107	113	97	145	86	96	105	106	72	110	110	87	104	126	117	79	73	91	84	102
149	91	100	113	119	112	106	120	75	111	122	101	137	88	95	67	108	89	112	112	142	110	107	142	140	103	104	103	110	80	120	102	107	113	97	145	86	96	105	106	72	110	110	87	104	126	117	79	73	91	84	102

149	91	100	113	119	112	106	120	75	111	122	101	137	88	95	67	108	89	112	112	142	110	107	142	140	103	104	103	110	80	120	102	107	113	97	145	86	96	105	106	72	110	110	87	104	126	117	79	73	91	84	102
145	97	123	107	132	127	91	106	118	106	132	104	132	118	114	109	96	126	112	117	136	115	118	139	136	121	121	116	121	117	124	124	124	115	122	138	121	118	120	125	118	129	123	113	113	126	118	125	125	108	114	123
151	128	112	143	102	96	140	143	122	140	97	137	128	128	128	131	126	114	131	121	145	112	111	147	145	124	104	118	119	128	106	109	115	120	114	147	112	119	102	116	122	106	113	133	126	114	149	122	118	129	132	129
149	129	108	142	113	103	143	144	119	139	97	138	131	130	132	133	129	112	140	127	147	119	113	147	144	125	107	122	126	130	113	118	114	126	115	147	115	123	101	121	122	107	117	134	133	119	144	121	119	130	130	133
140	80	120	108	122	117	90	107	92	98	118	89	129	104	119	100	107	128	119	114	148	110	119	135	137	126	117	109	104	109	115	111	110	116	100	135	100	118	120	108	106	130	122	105	112	127	120	109	104	106	107	123
146	121	115	136	81	85	143	137	102	140	92	132	134	115	111	118	119	105	121	112	150	121	105	145	143	110	92	108	107	118	88	109	109	104	91	150	100	109	103	105	101	93	99	119	125	100	143	99	107	124	107	115
137	103	131	102	133	127	96	106	132	105	137	99	127	117	115	108	106	127	106	117	130	118	122	138	134	128	127	110	116	121	130	117	116	125	132	136	120	121	128	119	114	131	133	112	116	123	121	118	120	111	116	121
137	103	131	102	133	127	96	106	132	105	137	99	127	117	115	108	106	127	106	117	130	118	122	138	134	128	127	110	116	121	130	117	116	125	132	136	120	121	128	119	114	131	133	112	116	123	121	118	120	111	116	121
137	103	131	102	133	127	96	106	132	105	137	99	127	117	115	108	106	127	106	117	130	118	122	138	134	128	127	110	116	121	130	117	116	125	132	136	120	121	128	119	114	131	133	112	116	123	121	118	120	111	116	121
137	103	131	102	133	127	96	106	132	105	137	99	127	117	115	108	106	127	106	117	130	118	122	138	134	128	127	111	117	121	130	117	116	125	132	136	120	121	128	119	114	131	133	112	116	123	121	118	120	111	116	121
137	103	131	102	133	127	96	106	132	105	137	99	127	117	115	108	106	127	106	117	130	118	122	138	134	128	127	110	116	121	130	117	116	125	132	136	120	121	128	119	114	131	133	112	116	123	121	118	120	111	116	121
151	128	141	136	126	124	128	127	134	130	122	129	90	122	122	136	131	131	130	125	147	125	120	149	145	130	119	132	133	127	118	128	124	127	130	144	122	124	121	128	125	127	128	126	136	124	143	137	131	134	136	127
78	125	140	106	145	144	122	119	137	116	150	126	142	132	126	132	139	131	137	145	118	140	145	80	65	137	137	142	138	142	143	132	130	144	143	75	146	128	143	138	138	135	139	137	136	144	90	138	143	137	135	135
147	120	124	140	80	76	143	143	100	137	70	127	134	115	110	118	124	97	119	104	149	120	95	148	151	113	84	98	99	113	89	106	110	97	88	147	105	104	80	100	105	87	97	117	136	81	140	97	98	103	113	120
151	122	114	145	99	98	124	139	108	139	101	131	126	133	127	126	123	118	127	111	153	111	112	148	152	124	111	122	114	127	111	104	119	115	90	146	113	117	104	116	116	114	116	124	127	117	148	116	109	128	115	124
148	101	128	108	116	115	102	88	129	108	129	102	133	93	93	77	100	116	68	82	140	94	95	142	141	96	116	85	94	85	111	105	110	95	121	148	93	92	123	101	107	112	116	74	110	92	123	105	104	81	94	82
148	102	128	109	117	115	103	89	129	109	129	103	135	94	94	77	101	116	69	82	140	94	96	143	142	97	116	87	95	87	111	105	110	96	121	149	93	94	123	102	107	114	117	75	111	94	124	105	104	82	95	84
148	101	128	108	116	115	102	88	129	108	129	102	133	93	93	77	100	116	68	82	140	94	95	142	141	96	116	85	94	85	111	105	110	95	121	148	93	92	123	101	107	112	116	74	110	92	123	105	104	81	94	82
148	101	128	108	116	115	102	88	129	108	129	102	133	93	93	77	100	116	68	82	140	94	95	142	141	96	116	85	94	85	111	105	110	95	121	148	93	92	123	101	107	112	116	74	110	92	123	105	104	81	94	82
148	101	128	108	116	115	102	88	129	108	129	102	133	93	93	77	100	116	68	82	140	94	95	142	141	96	116	85	94	85	111	105	110	95	121	148	93	92	123	101	107	112	116	74	110	92	123	105	104	81	94	82
148	101	128	108	116	115	102	88	129	108	129	102	133	93	93	77	100	116	68	82	140	94	95	142	141	96	116	85	94	85	111	105	110	95	121	148	93	92	123	101	107	112	116	74	110	92	123	105	104	81	94	82
148	101	128	108	116	115	102	88	129	108	129	102	133	93	93	77	100	116	68	82	140	94	95	142	141	96	116	85	94	85	111	105	110	95	121	148	93	92	123	101	107	112	116	74	110	92	123	105	104	81	94	82
148	101	128	108	116	115	102	88	129	108	129	102	133	93	94	77	100	116	68	82	140	94	95	142	141	95	116	85	95	85	111	105	110	95	121	148	93	93	123	101	108	112	116	75	110	92	123	105	105	81	95	83
148	101	128	108	116	115	102	88	129	108	129	102	133	93	93	77	100	116	68	82	140	94	95	142	141	96	116	85	94	85	111	105	110	95	121	148	93	92	123	101	107	112	116	74	110	92	123	105	104	81	94	82
148	101	128	108	116	115	102	88	129	108	129	102	133	93	93	77	100	116	68	82	140	94	95	142	141	96	116	85	94	85	111	105	110	95	121	148	93	92	123	101	107	112	116	74	110	92	123	105	104	81	94	82
148	101	128	108	116	115	102	88	129	108	129	102	133	93	93	77	100	116	68	82	140	94	95	142	141	96	116	85	94	85	111	105	110	95	121	148	93	92	123	101	107	112	116	74	110	92	123	105	104	81	94	82
148	101	128	108	116	115	102	88	129	108	129	102	133	93	93	77	100	116	68	82	140	94	95	142	141	96	116	85	94	85	111	105	110	95	121	148	93	92	123	101	107	112	116	74	110	92	123	105	104	81	94	82
148	101	128	108	116	115	102	88	129	108	129	102	133	93	93	77	100	116	68	82	140	94	95	142	141	96	116	85	94	85	111	105	110	95	121	148	93	92	123	101	107	112	116	74	110	92	123	105	104	81	94	82
148	101	128	108	116	115	102	88	129	108	129	102	133	93	93	77	100	116	68	82	140	94	95	142	141	96	116	85	94	85	111	105	110	95	121	148	93	92	123	101	107	112	116	74	110	92	123	105	104	81	94	82
148	101	128	108	116	115	102	88	129	108	129	102	133	93	93	77	100	116	68	82	140	94	95	142	141	96	116	85	94	85	111	105	110	95	121	148	93	92	123	101	107	112	116	74	110	92	123	105	104	81	94	82
148	101	128	108	116	115	102	88	129	108	129	102	133	93	93	77	100	116	68	82	140	94	95	142	141	96	116	85	94	85	111	105	110	95	121	148	93	92	123	101	107	112	116	74	110	92	123	105	104	81	94	82
148	101	128	108	116	115	102	88	129	108	129	102	133	93	93	77	100	116	68	82	140	94	95	142	141	96	116	85	94	85	111	105	110	95	121	148	93	92	123	101	107	112	116	74	110	92	123	105	104	81	94	82
148	101	128	108	116	115	102	88	129	108	129	102	133	93	93	77	100	116	68	82	140	94	95	142	141	96	116	85	94	85	111	105	110	95	121	148	93	92	123	101	107	112	116	74	110	92	123	105	104	81	94	82
148	101	128	108	116	115	102	88	129	108	129	102	133	93	93	77	100	116	68	82	140	94	95	142	141	96	116	85	94	85	111	105	110	95	121	148	93	92	123	101	107	112	116	74	110	92	123	105	104	81	94	82
149	101	127	107	116	115	102	89	128	107	129	102	133	92	92	76	99	116	67	81	140	93	95	141	142	96	116	85	94	85	110	105	109	94	120	147	92	92	123	100	106	112	115	73	109	93	122	105	104	81	94	81
151	139	113	140	107	110	143	147	127	144	105	139	131	132	130	135	129	121	138	126	148	124	121	148	147	130	114	126	130	136	120	115	118	128	119	146	123	122	105	128	124	118	112	140	128	124	147	124	118	135	132	135
151	139	115	140	110	108	140	149	123	143	102	138	128	134	132	134	133	118	137	125	148	119	120	148	148	127	109	123	128	132	117	113	115	128	115	148	121	124	104	121	123	109	108	139	128	118	149	124	119	133	135	135
151	139	116	140	110	109	140	149	123	143	102	138	128	134	132	134	133	118	137	125	148	119	120	148	148	127	110	123	128	132	117	113	115	128	115	148	121	124	104	121	123	110	108	139	128	118	149	124	119	133	135	135
151	139	115	140	110	108	140	149	123	143	102	138	128	134	132	134	133	118	137	125	148	119	120	148	148	127	109	123	128	132	117	113	115	128	115	148	121	124	104	121	123	109	108	139	128	118	149	124	119	133	135	135
151	139	113	140	107	110	143	147	127	144	105	139	131	132	130	135	129	121	138	126	148	124	121	148	147	130	114	126	130	136	120	115	118	128	119	146	123	122	105	128	124	118	112	140	128	124	147	124	118	135	132	135
151	139	113	140	107	110	143	147	127	144	105	139	131	132	130	135	129	121	138	126	148	124	121	148	147	130	114	126	130	136	120	115	118	128	119	146	123	122	105	128	124	118	112	140	128	124	147	124	118	135	132	135
150	135	111	140	101	110	140	144	120	145	96	138	129	138	129	138	130	120	134	127	147	120	119	147	143	123	115	124	130	128	113	121	111	128	116	147	119	123	105	126	128	113	121	140	136	120	148	129	122	131	132	131
151	139	115	140	110	108	140	149	123	143	102	138	128	134	132	134	133	118	137	125	148	119	120	148	148	127	109	123	128	132	117	113	115	128	115	148	121	124	104	121	123	109	108	139	128	118	149	124	119	133	135	135
151	139	115	140	110	108	140	149	123	143	102	138	128	134	132	134	133	118	137	125	148	119	120	148	148	127	109	123	128	132	117	113	115	128	115	148	121	124	104	121	123	109	108	139	128	118	149	124	119	133	135	135
151	139	115	140	110	108	140	149	123	143	102	138	128	134	132	134	133	118	137	125	148	119	120	148	148	127	109	123	128	132	117	113	115	128	115	148	121	124	104	121	123	109	108	139	128	118	149	124	119	133	135	135
151	139	113	140	107	110	143	147	127	144	105	139	131	132	130	135	129	121	138	126	148	124	121	148	147	130	114	126	130	136	120	115	118	128	119	146	123	122	105	128	124	118	112	140	128	124	147	124	118	135	132	135
151	139	115	140	110	108	140	149	123	143	102	138	128	134	132	134	133	118	137	125	148	119	120	148	148	127	109	123	128	132	117	113	115	128	115	148	121	124	104	121	123	109	108	139	128	118	149	124	119	133	135	135
147	128	99	128	107	111	129	129	126	130	117	126	128	130	117	131	125	113	124	127	148	109	115	148	142	122	108	122	122	115	114	111	88	121	119	148	114	118	108	119	121	111	111	127	121	122	141	126	122	120	125	124
109	106	129	100	135	132	111	101	140	100	137	104	136	108	118	117	121	134	119	120	126	125	131	114	109	131	133	114	114	126	130	130	125	122	133	118	124	118	136	118	126	133	133	117	129	123	108	128	120	113	119	130
145	111	126	128	106	98	122	125	132	124	110	119	139	97	99	95	106	101	90	105	146	117	88	149	146	102	98	99	97	108	92	116	110	85	104	146	113	100	91	102	113	96	93	95	120	81	135	108	100	100	111	106
145	111	126	128	106	98	122	125	132	124	110	119	139	97	99	95	106	101	90	105	146	117	88	149	146	102	98	99	97	108	92	116	110	85	104	146	113	100	91	102	113	96	93	95	120	81	135	108	100	100	111	106
145	111	126	128	106	98	122	125	132	124	110	119	139	97	99	95	106	101	90	105	146	117	88	149	146	102	98	99	97	108	92	116	110	85	104	146	113	100	91	102	113	96	93	95	120	81	135	108	100	100	111	106
145	111	126	128	106	98	122	125	132	124	110	119	139	97	99	95	106	101	90	105	146	117	88	149	146	102	98	99	97	108	92	116	110	85	104	146	113	100	91	102	113	96	93	95	120	81	135	108	100	100	111	106
144	117	124	111	101	96	119	116	133	117	114	111	138	98	110	104	103	114	87	84	145	108	91	141	138	96	97	91	87	102	94	106	119	93	108	142	102	100	107	97	107	104	88	92	117	93	134	97	104	108	102	95
123	115	126	105	124	119	108	62	108	113	129	107	143	119	104	111	91	107	112	116	133	113	111	135	126	111	121	110	124	107	126	120	114	122	123	135	113	108	116	110	113	115	120	113	110	113	117	113	124	102	118	113
142	112	127	121	90	89	118	121	117	124	104	121	132	84	102	105	104	108	91	85	146	102	102	144	141	105	97	74	87	102	92	95	100	85	94	143	91	95	107	91	105	104	87	96	112	96	135	92	96	92	102	88
148	129	100	133	111	116	127	131	122	129	111	128	128	130	130	129	129	124	119	119	146	105	113	146	147	121	120	121	119	126	115	113	110	127	107	143	116	127	117	116	125	123	122	126	122	117	144	128	124	125	124	121
150	62	104	100	123	122	101	114	78	97	128	103	136	94	103	85	102	109	107	117	146	106	113	139	137	99	117	97	103	80	113	111	117	101	106	146	84	114	122	101	89	116	115	72	95	120	110	95	91	97	93	97
82	123	139	97	145	143	119	92	142	109	146	125	139	134	131	133	134	140	134	143	123	129	144	93	92	138	141	140	141	141	143	134	127	149	144	92	142	129	145	140	132	135	139	139	128	147	103	137	145	138	138	141
145	117	108	131	111	103	131	139	113	139	99	131	129	121	114	125	125	105	128	118	141	116	110	143	143	121	104	116	116	112	113	111	108	113	116	140	112	110	101	114	116	103	107	123	117	119	138	111	110	123	122	124
150	60	103	99	122	121	100	113	78	95	128	102	136	93	102	84	101	109	106	115	145	105	111	139	137	97	115	95	101	79	112	110	115	99	105	146	83	113	121	99	87	116	113	71	94	120	108	93	90	95	91	95
150	60	103	99	122	121	100	113	78	95	128	102	136	93	102	84	101	109	106	115	145	105	111	139	137	97	115	95	101	79	112	110	115	99	105	146	83	113	121	99	87	116	113	71	94	120	108	93	90	95	91	95
150	60	103	99	122	121	100	113	78	95	128	102	136	93	102	84	101	109	106	115	145	105	111	139	137	97	115	95	101	79	112	110	115	99	105	146	83	113	121	99	87	116	113	71	94	120	108	93	90	95	91	95
0	139	143	121	147	148	141	126	149	122	148	135	147	133	140	136	149	142	142	149	116	145	151	68	71	148	146	142	139	149	144	134	137	150	145	72	149	136	146	143	144	140	144	140	143	144	105	141	139	142	140	140
139	0	114	102	126	117	91	106	106	76	129	98	127	105	106	98	102	118	112	124	136	118	114	135	138	111	123	96	99	103	119	117	113	106	118	139	105	115	116	111	99	125	120	90	105	125	99	103	105	87	104	112
143	114	0	126	126	123	124	136	101	123	128	131	137	119	120	122	111	112	131	127	142	113	111	140	143	118	119	123	122	117	124	113	104	125	110	143	110	127	122	120	100	123	118	124	93	132	131	113	108	121	110	116
121	102	126	0	137	130	97	96	118	106	138	103	143	119	109	112	109	120	122	124	126	117	131	118	120	124	128	117	122	124	134	131	122	129	125	123	113	118	141	122	106	131	123	113	111	134	118	119	119	113	123	126
147	126	126	137	0	43	145	130	114	145	69	136	129	107	105	124	116	93	111	92	153	113	86	149	141	96	77	89	94	103	70	104	109	79	95	149	98	88	87	100	107	91	74	124	134	86	147	95	102	100	102	101
148	117	123	130	43	0	139	129	112	139	78	134	130	98	101	119	106	75	108	95	151	109	75	149	144	98	56	83	85	110	69	101	103	84	80	147	97	90	67	93	96	76	67	113	128	75	144	88	90	97	88	111
141	91	124	97	145	139	0	98	109	99	143	65	134	116	118	92	102	120	114	119	132	116	127	132	135	121	133	111	121	117	134	125	118	124	125	128	119	125	127	122	115	133	133	108	101	142	117	120	122	109	119	122
126	106	136	96	130	129	98	0	131	110	139	101	140	121	108	118	97	130	112	118	131	115	124	138	130	121	135	117	127	113	128	132	126	125	134	135	121	112	137	126	127	133	132	116	122	124	125	126	134	100	117	129
149	106	101	118	114	112	109	131	0	122	114	118	137	110	115	93	111	104	126	118	146	113	114	140	140	109	112	108	115	101	117	111	109	114	70	144	100	124	117	104	83	111	118	104	110	124	128	91	100	112	97	107
122	76	123	106	145	139	99	110	122	0	141	97	139	120	124	113	117	137	117	133	129	121	122	120	135	130	139	121	117	115	136	130	121	127	130	133	115	130	134	123	116	135	135	112	113	134	94	124	125	110	124	126
148	129	128	138	69	78	143	139	114	141	0	133	129	114	120	130	118	102	122	98	149	121	90	148	145	107	84	108	112	115	78	115	117	98	98	150	110	111	84	115	106	94	92	131	135	92	146	101	101	115	117	121
135	98	131	103	136	134	65	101	118	97	133	0	139	112	114	100	104	127	101	112	131	115	116	124	131	117	130	111	123	101	130	120	116	127	132	131	116	114	126	122	114	126	127	108	111	131	100	108	117	109	119	123

147	127	137	143	129	130	134	140	137	139	129	139	0	139	134	135	140	141	139	138	149	137	131	147	145	138	128	133	138	136	126	133	127	135	138	142	135	132	127	139	138	137	134	139	139	134	140	138	134	139	136	139
133	105	119	119	107	98	116	121	110	120	114	112	139	0	80	78	106	89	103	86	139	100	93	135	130	96	98	88	99	86	95	90	111	83	97	134	103	83	109	99	93	102	89	67	107	98	121	89	77	81	94	85
140	106	120	109	105	101	118	108	115	124	120	114	134	80	0	91	109	87	91	86	141	113	92	139	136	82	105	98	102	78	106	110	111	77	97	134	103	42	113	104	83	99	98	73	119	90	127	106	105	83	89	82
136	98	122	112	124	119	92	118	93	113	130	100	135	78	91	0	114	102	90	91	136	105	115	137	138	107	120	97	98	93	121	100	102	99	106	137	99	99	118	94	92	108	119	56	109	123	121	93	85	90	78	88
149	102	111	109	116	106	102	97	111	117	118	104	140	106	109	114	0	100	96	108	141	114	104	141	139	116	104	96	108	115	105	116	107	110	114	140	110	109	101	113	101	114	96	123	106	111	124	105	115	92	99	110
142	118	112	120	93	75	120	130	104	137	102	127	141	89	87	102	100	0	107	100	144	120	91	140	138	100	66	96	96	104	87	105	107	96	94	139	98	84	80	85	84	74	81	101	123	100	134	72	84	102	85	103
142	112	131	122	111	108	114	112	126	117	122	101	139	103	91	90	96	107	0	85	142	115	94	141	145	103	111	93	102	99	106	119	108	97	116	142	112	96	117	100	115	103	107	97	121	88	127	102	110	97	95	88
149	124	127	124	92	95	119	118	118	133	98	112	138	86	86	91	108	100	85	0	144	91	89	146	148	99	102	94	86	93	87	91	120	52	85	146	102	84	104	73	102	114	97	86	122	89	137	89	85	88	96	82
116	136	142	126	153	151	132	131	146	129	149	131	149	139	141	136	141	144	142	144	0	142	148	123	124	143	152	141	147	145	151	141	137	149	146	125	148	144	150	147	139	148	149	141	140	150	128	143	147	145	144	147
145	118	113	117	113	109	116	115	113	121	121	115	137	100	113	105	114	120	115	91	142	0	106	144	142	118	114	116	106	106	108	63	101	103	104	139	99	111	119	109	112	119	111	94	91	121	132	113	106	107	118	96
151	114	111	131	86	75	127	124	114	122	90	116	131	93	92	115	104	91	94	89	148	106	0	149	150	80	65	89	101	93	83	110	112	86	100	146	112	92	81	113	93	83	75	107	120	77	137	85	86	101	104	101
68	135	140	118	149	149	132	138	140	120	148	124	147	135	139	137	141	140	141	146	123	144	149	0	68	143	144	142	138	146	148	134	135	151	147	44	142	136	144	139	138	135	141	138	141	144	84	140	137	148	143	139
71	138	143	120	141	144	135	130	140	135	145	131	145	130	136	138	139	138	145	148	124	142	150	68	0	143	137	141	135	140	140	137	137	148	143	71	146	133	141	143	138	134	136	136	139	143	101	140	140	143	141	135
148	111	118	124	96	98	121	121	109	130	107	117	138	96	82	107	116	100	103	99	143	118	80	143	143	0	93	83	100	74	93	118	110	94	109	145	108	90	103	110	95	89	88	93	119	91	131	102	110	90	95	87
146	123	119	128	77	56	133	135	112	139	84	130	128	98	105	120	104	66	111	102	152	114	65	144	137	93	0	93	93	107	63	100	106	92	98	141	99	96	62	102	81	62	46	112	130	85	137	80	86	107	95	111
142	96	123	117	89	83	111	117	108	121	108	111	133	88	98	97	96	96	93	94	141	116	89	142	141	83	93	0	56	83	97	106	98	86	100	146	105	92	103	93	87	93	88	98	111	77	128	79	96	75	81	94
139	99	122	122	94	85	121	127	115	117	112	123	138	99	102	98	108	96	102	86	147	106	101	138	135	100	93	56	0	102	93	98	120	82	93	141	97	93	102	77	99	96	98	95	120	79	135	85	84	82	88	98
149	103	117	124	103	110	117	113	101	115	115	101	136	86	78	93	115	104	99	93	145	106	93	146	140	74	107	83	102	0	104	110	111	85	105	145	102	84	119	106	93	92	106	67	108	92	129	92	95	91	87	74
144	119	124	134	70	69	134	128	117	136	78	130	126	95	106	121	105	87	106	87	151	108	83	148	140	93	63	97	93	104	0	100	108	75	88	144	99	94	82	92	100	97	66	113	128	82	140	90	94	95	102	99
134	117	113	131	104	101	125	132	111	130	115	120	133	90	110	100	116	105	119	91	141	63	110	134	137	118	100	106	98	110	100	0	108	105	97	133	89	102	103	99	109	114	110	92	100	117	120	97	90	110	109	85
137	113	104	122	109	103	118	126	109	121	117	116	127	111	111	102	107	107	108	120	137	101	112	135	137	110	106	98	120	111	108	108	0	123	112	140	107	116	109	104	106	108	114	121	93	121	123	110	117	104	108	113
150	106	125	129	79	84	124	125	114	127	98	127	135	83	77	99	110	96	97	52	149	103	86	151	148	94	92	86	82	85	75	105	123	0	85	147	105	79	98	75	102	99	83	75	124	79	139	92	86	87	96	79
145	118	110	125	95	80	125	134	70	130	98	132	138	97	97	106	114	94	116	85	146	104	100	147	143	109	98	100	93	105	88	97	112	85	0	148	103	103	104	85	85	104	98	97	119	99	139	93	87	99	87	98
72	139	143	123	149	147	128	135	144	133	150	131	142	134	134	137	140	139	142	146	125	139	146	44	71	145	141	146	141	145	144	133	140	147	148	0	148	133	142	143	141	142	140	140	139	147	99	144	140	144	142	139
149	105	110	113	98	97	119	121	100	115	110	116	135	103	103	99	110	98	112	102	148	99	112	142	146	108	99	105	97	102	99	89	107	105	103	148	0	107	106	97	92	108	102	92	107	119	130	91	92	109	104	98
136	115	127	118	88	90	125	112	124	130	111	114	132	83	42	99	109	84	96	84	144	111	92	136	133	90	96	92	93	84	94	102	116	79	103	133	107	0	105	93	105	89	93	95	124	82	131	100	94	86	85	86
146	116	122	141	87	67	127	137	117	134	84	126	127	109	113	118	101	80	117	104	150	119	81	144	141	103	62	103	102	119	82	103	109	98	104	142	106	105	0	109	99	92	68	120	127	101	134	95	92	108	105	119
143	111	120	122	100	93	122	126	104	123	115	122	139	99	104	94	113	85	100	73	147	109	113	139	143	110	102	93	77	106	92	99	104	75	85	143	97	93	109	0	114	94	102	91	120	94	133	92	89	97	93	86
144	99	100	106	107	96	115	127	83	116	106	114	138	93	83	92	101	84	115	102	139	112	93	138	138	95	81	87	99	93	100	109	106	102	85	141	92	105	99	114	0	91	84	89	106	116	127	67	82	98	83	103
140	125	123	131	91	76	133	133	111	135	94	126	137	102	99	108	114	74	103	114	148	119	83	135	134	89	62	93	96	92	97	114	108	99	104	142	108	89	92	94	91	0	73	100	126	83	134	92	105	115	98	101
144	120	118	123	74	67	133	132	118	135	92	127	134	89	98	119	96	81	107	97	149	111	75	141	136	88	46	88	98	106	66	110	114	83	98	140	102	93	68	102	84	73	0	109	121	93	134	93	99	101	101	102
140	90	124	113	124	113	108	116	104	112	131	108	139	67	73	56	123	101	97	86	141	94	107	138	136	93	112	98	95	67	113	92	121	75	97	140	92	95	120	91	89	100	109	0	113	101	120	94	74	94	90	63
143	105	93	111	134	128	101	122	110	113	135	111	139	107	119	109	106	123	121	122	140	91	120	141	139	119	130	111	120	108	128	100	93	124	119	139	107	124	127	120	106	126	121	113	0	134	126	119	120	111	122	119
144	125	132	134	86	75	142	124	124	134	92	131	134	98	90	123	111	100	88	89	150	121	77	144	143	91	85	77	79	92	82	117	121	79	99	147	119	82	101	94	116	83	93	101	134	0	139	107	102	95	109	102
105	99	131	118	147	144	117	125	128	94	146	100	140	121	127	121	124	134	127	137	128	132	137	84	101	131	137	128	135	129	140	120	123	139	139	99	130	131	134	133	127	134	134	120	126	139	0	126	126	121	126	126
141	103	113	119	95	88	120	126	91	124	101	108	138	89	106	93	105	72	102	89	143	113	85	140	140	102	80	79	85	92	90	97	110	92	93	144	91	100	95	92	67	92	93	94	119	107	126	0	60	99	77	100
139	105	108	119	102	90	122	134	100	125	101	117	134	77	105	85	115	84	110	85	147	106	86	137	140	110	86	96	84	95	94	90	117	86	87	140	92	94	92	89	82	105	99	74	120	102	126	60	0	99	84	98
142	87	121	113	100	97	109	100	112	110	115	109	139	81	83	90	92	102	97	88	145	107	101	148	143	90	107	75	82	91	95	110	104	87	99	144	109	86	108	97	98	115	101	94	111	95	121	99	99	0	94	100
140	104	110	123	102	88	119	117	97	124	117	119	136	94	89	78	99	85	95	96	144	118	104	143	141	95	95	81	88	87	102	109	108	96	87	142	104	85	105	93	83	98	101	90	122	109	126	77	84	94	0	88
140	112	116	126	101	111	122	129	107	126	121	123	139	85	82	88	110	103	88	82	147	96	101	139	135	87	111	94	98	74	99	85	113	79	98	139	98	86	119	86	103	101	102	63	119	102	126	100	98	100	88	0
149	115	131	125	100	81	131	130	99	131	102	127	132	105	92	118	115	109	109	94	151	114	93	147	146	109	94	88	96	108	95	114	118	84	84	145	112	93	102	104	98	89	95	100	130	76	143	105	105	111	99	105
138	112	118	124	95	84	125	134	100	126	94	116	132	99	105	113	111	86	116	109	146	118	82	140	135	102	75	95	98	99	85	109	105	90	97	136	105	88	75	99	94	90	83	111	119	100	129	95	90	111	96	107
114	127	137	123	147	146	121	122	134	126	145	113	145	134	137	130	131	136	132	136	61	138	143	109	116	133	149	128	140	135	141	138	129	143	139	116	137	140	145	139	130	141	144	131	129	144	110	134	141	137	132	138
143	115	133	128	103	99	132	121	122	125	108	134	136	103	95	101	119	110	79	96	148	120	102	139	140	103	107	95	71	97	101	115	129	91	104	146	115	89	120	93	125	95	110	89	130	54	135	116	105	89	105	97
149	91	114	118	110	114	98	112	94	112	121	109	133	88	76	81	98	99	98	102	142	119	93	149	145	63	107	97	105	76	107	117	119	88	99	146	103	87	104	114	89	113	105	87	113	109	123	106	99	82	90	85
149	101	118	113	113	105	119	106	116	114	111	112	141	91	88	107	95	108	96	90	147	114	97	142	141	94	102	82	76	83	100	115	111	90	99	145	103	98	117	103	98	115	102	94	112	80	120	97	94	51	108	99
144	117	125	132	87	86	133	129	123	131	95	122	134	96	81	114	110	103	79	94	148	114	82	143	144	83	89	64	68	86	80	106	115	84	100	149	100	84	97	93	102	84	83	98	125	55	138	95	99	96	101	90
146	103	131	130	97	91	126	119	131	126	102	125	134	97	93	97	113	106	88	97	149	117	97	142	142	76	95	77	69	97	91	108	119	87	111	148	105	89	105	90	112	90	90	83	131	84	133	104	104	72	94	90
136	94	116	104	129	119	97	103	118	103	131	92	138	105	116	113	55	106	102	119	140	121	118	133	133	123	112	98	105	120	118	114	115	123	122	135	106	113	113	115	97	119	107	113	105	118	113	102	106	105	104	124
135	119	125	132	88	84	133	137	111	132	93	128	133	99	106	122	113	87	116	116	145	128	93	141	136	95	79	105	108	109	87	114	107	104	100	141	110	95	73	114	104	75	78	118	127	85	128	105	107	105	112	110
145	123	129	142	76	76	144	127	116	139	90	134	135	111	85	114	115	96	90	92	151	115	87	147	143	102	94	78	74	93	86	109	118	84	94	147	107	81	102	94	117	92	100	110	137	48	140	98	108	93	105	100
146	101	102	127	108	100	112	126	79	125	122	102	138	88	100	80	91	77	92	105	144	114	94	142	141	80	93	73	87	76	104	105	104	100	94	143	101	101	95	93	79	87	102	92	114	106	126	78	84	92	63	82
143	107	106	124	106	92	116	127	89	123	117	118	135	97	87	78	108	85	91	76	143	109	97	141	147	100	103	95	87	108	98	86	100	86	69	143	90	89	101	60	96	106	106	81	112	99	129	95	77	88	82	91
128	101	126	127	105	97	125	113	113	120	110	115	140	63	99	91	111	105	110	94	141	105	103	137	127	104	104	83	97	86	101	87	118	93	105	135	104	93	104	106	100	108	99	65	112	101	119	87	83	71	91	86
147	113	112	130	103	90	120	128	81	127	109	114	137	101	101	99	104	83	104	93	147	110	92	145	138	93	81	83	93	85	93	110	103	101	75	147	103	107	90	87	65	90	88	93	113	106	126	75	93	102	80	90
141	88	117	119	102	99	112	107	109	107	117	105	141	72	86	91	88	96	93	96	148	110	101	142	139	100	111	86	83	87	93	106	107	89	100	142	99	88	113	96	97	110	100	88	107	92	120	93	96	41	105	94
145	117	133	128	107	103	129	128	121	129	115	124	141	90	75	85	124	101	96	94	144	119	103	147	147	95	114	103	108	106	110	119	115	87	97	147	108	84	117	96	110	102	110	82	123	85	132	110	104	88	102	99
143	107	106	124	106	92	116	127	89	123	117	118	135	97	87	78	108	85	91	76	143	109	97	141	147	100	103	95	87	108	98	86	100	86	69	143	90	89	101	60	96	106	106	81	112	99	129	95	77	88	82	91
143	106	129	107	132	125	93	100	86	110	129	101	143	123	119	111	107	124	127	126	136	117	135	132	139	131	133	123	128	125	136	127	113	130	114	132	118	124	125	119	116	124	133	121	119	131	123	126	123	118	127	130
115	110	129	109	144	137	108	101	119	114	143	109	140	127	118	119	105	123	128	142	133	124	138	109	112	133	133	126	127	135	141	120	119	145	127	113	130	114	130	134	117	127	131	127	119	136	97	123	132	125	125	130
148	81	108	100	121	110	104	109	63	101	122	101	133	96	100	90	98	92	116	113	145	115	114	139	136	100	101	107	103	89	108	107	100	107	88	140	89	112	104	90	81	112	108	85	108	124	110	88	95	95	83	102
148	114	121	137	83	75	130	128	108	137	95	130	131	87	89	98	106	66	100	83	148	108	85	151	150	102	84	97	97	106	90	102	119	76	94	148	103	89	81	96	107	94	89	94	129	87	140	93	79	106	96	101
100	108	124	105	142	138	115	97	139	95	142	105	136	113	126	124	127	138	124	127	120	111	130	116	110	127	134	127	122	116	137	116	119	131	137	112	128	129	136	127	129	132	129	112	109	132	90	128	124	115	126	120
139	105	109	124	96	83	122	131	107	124	103	113	133	93	87	98	108	67	101	88	144	110	90	135	136	95	75	83	82	94	82	95	105	90	99	138	94	87	92	84	89	90	80	85	112	99	121	73	67	100	88	95
150	99	131	113	111	103	118	118	116	119	116	106	138	76	81	84	124	96	105	81	140	104	100	145	137	91	99	98	104	63	108	99	128	73	104	146	92	80	107	101	97	100	99	44	117	100	127	93	82	102	92	89
138	117	112	126	97	86	126	134	111	134	104	111	133	75	77	94	105	70	93	81	143	115	60	138	140	87	76	93	103	96	84	104	101	77	93	136	105	79	88	88	104	92	82	89	120	92	128	81	64	101	93	99
135	117	125	131	84	67	136	137	115	141	88	126	133	103	104	118	105	89	112	110	148	123	83	138	136	108	77	95	101	121	94	113	111	104	99	136	116	102	69	105	105	83	71	116	131	87	136	96	100	106	117	117
120	96	130	101	139	138	107	96	127	89	146	97	136	117	119	114	114	139	111	130	136	112	125	121	114	129	133	115	122	115	132	119	119	137	129	115	117	119	133	130	118	128	128	111	109	128	76	122	123	114	124	124
143	124	111	132	85	80	128	124	113	134	109	118	135	94	80	97	112	80	91	69	145	101	79	146	143	99	88	91	83	104	87	97	104	67	72	144	99	70	96	68	101	89	88	95	122	82	134	91	87	94	89	97
146	107	123	123	105	106	118	116	107	116	119	117	139	86	69	77	111	112	87	79	144	99	92	147	147	89	118	93	81	79	103	104	117	77	99	145	97	80	120	92	107	110	112	74	115	92	137	108	101	82	99	75
146	99	107	119	101	88	120	130	97	116	104	115	135	95	105	96	105	75	101	100	145	119	78	139	140	88	71	83	93	81	95	102	106	102	103	144	99	104	90	107	63	85	91	95	112	105	123	52	63	99	81	100
133	146	138	142	146	142	141	145	143	141	147	143	151	139	140	146	139	137	146	144	83	145	146	131	137	146	145	140	141	152	144	138	137	148	133	127	143	140	145	145	137	148	141	147	139	146	138	148	148	144	141	150
150	109	117	121	95	98	123	128	92	115	104	115	131	75	80	92	111	101	105	81	145	101	76	147	143	88	100	101	104	68	88	115	121	71	75	145	100	87	109	97	93	106	89	81	119	98	130	102	85	96	87	75
144	92	119	109	115	110	116	121	96	111	119	102	134	92	88	74	129	97	109	91	140	112	107	138	136	89	113	102	103	71	112	107	127	89	96	139	91	97	112	98	88	108	114	55	114	107	119	91	79	105	85	98
147	98	122	118	117	118	115	118	105	115	124	99	134	80	79	79	119	101	108	96	143	110	111	143	138	98	115	109	115	67	117	106	121	91	107	144	88	89	115	108	101	108	108	54	116	110	120	104	91	99	104	98

140	104	125	120	101	95	116	126	103	119	107	105	136	97	111	110	103	100	106	102	144	122	92	140	140	110	90	79	95	97	101	110	115	99	106	142	100	105	109	101	83	99	98	102	125	98	120	52	80	98	91	106
146	102	117	121	118	117	114	115	105	107	122	107	136	80	86	67	109	100	91	78	143	106	102	138	140	99	115	108	108	88	108	82	109	103	110	144	89	97	110	91	104	117	116	74	107	115	109	105	92	95	99	73
145	114	127	139	74	63	139	130	108	136	91	133	128	108	103	114	109	92	117	94	153	112	91	150	143	110	83	93	95	116	84	107	117	85	87	146	110	101	88	104	98	93	93	109	133	81	138	104	100	106	98	117
134	117	109	136	84	87	139	136	119	136	96	121	130	82	89	99	108	74	101	80	141	109	84	136	135	101	80	102	90	99	75	91	115	78	96	135	96	83	89	90	99	98	82	88	127	96	126	83	67	107	92	95
148	111	132	133	100	102	118	118	108	128	109	119	134	103	94	99	109	109	77	96	145	119	91	144	148	76	111	85	116	76	106	126	112	95	108	141	111	99	115	116	106	101	109	97	118	93	136	107	125	101	94	87
147	118	119	130	99	103	128	126	110	128	109	114	139	98	78	99	116	97	100	92	152	108	82	145	143	79	97	103	99	59	95	108	118	83	103	140	102	80	107	102	92	90	94	85	117	85	132	100	99	104	100	90
125	104	119	102	133	133	109	121	120	119	138	116	132	118	117	108	126	126	113	129	137	114	132	130	129	125	135	116	124	130	130	115	114	131	131	134	119	113	135	127	116	126	132	118	102	131	121	119	116	113	121	121
147	98	118	117	101	106	111	114	98	110	114	113	135	70	87	81	101	96	92	92	143	111	86	145	143	95	98	90	93	76	99	103	112	92	100	149	92	92	111	101	84	99	100	75	109	103	120	68	72	81	84	74
148	103	118	128	98	99	125	111	104	122	102	117	125	89	70	93	103	95	89	90	140	119	88	146	147	82	99	92	119	82	88	102	97	87	99	144	103	87	101	112	91	107	101	90	124	93	120	95	106	94	88	82
132	108	128	98	122	118	108	110	114	114	127	90	141	115	102	112	123	123	125	127	129	121	118	125	124	111	120	119	119	118	117	129	121	109	108	125	112	109	126	121	113	115	110	105	127	124	119	118	114	120	124	115
137	103	125	118	102	101	110	123	117	116	116	95	137	72	84	91	108	100	104	86	144	96	93	142	135	98	104	92	89	82	102	90	123	77	101	142	91	84	108	100	100	106	100	67	113	94	130	92	86	92	108	84
140	117	120	126	107	112	123	130	114	127	121	118	139	87	81	81	121	98	95	78	140	99	107	134	137	96	112	104	103	96	105	85	106	99	99	136	87	85	114	74	106	106	109	78	120	114	122	102	99	109	87	55
145	118	127	128	85	72	126	130	98	135	96	124	136	83	83	108	99	77	90	84	152	111	84	146	139	94	80	84	94	104	76	110	112	74	78	144	103	86	85	94	100	91	73	92	122	76	141	96	86	92	94	101
136	110	104	121	92	93	121	135	94	128	108	121	138	85	94	84	93	73	89	92	148	117	94	135	138	96	88	95	92	101	88	101	115	83	95	139	93	94	94	88	90	91	84	77	115	103	130	86	70	93	90	76
136	118	113	124	92	83	133	128	100	138	102	133	136	86	89	101	106	66	104	90	145	111	95	134	128	112	82	99	89	109	84	96	114	83	88	134	96	94	88	89	95	97	77	89	124	100	140	93	77	103	90	94
141	97	118	104	121	117	109	124	110	112	121	107	132	94	92	83	111	120	119	102	140	79	107	141	141	111	122	111	101	109	120	95	116	102	107	142	106	97	121	107	98	116	112	87	90	120	128	113	95	105	110	108
141	116	122	133	93	97	129	124	107	129	109	120	137	88	65	94	110	99	86	89	151	106	93	142	141	94	114	93	91	73	102	108	125	75	97	140	101	73	122	103	106	94	105	82	120	78	136	103	102	94	104	78
148	116	118	126	88	87	128	126	97	128	91	123	131	95	83	101	117	86	120	90	149	117	90	144	136	85	75	86	93	74	91	98	114	92	83	144	93	69	83	107	77	93	80	98	125	97	133	90	86	106	85	91
73	136	143	123	149	145	129	131	148	126	148	130	145	135	138	137	136	135	139	149	124	140	145	46	64	144	138	139	140	147	147	134	133	152	147	48	144	137	137	144	134	130	136	138	140	143	96	137	138	144	144	138
138	99	114	117	104	99	130	128	94	119	112	123	140	87	93	107	118	89	117	103	143	117	102	138	139	110	109	111	97	92	106	105	127	95	76	138	92	90	115	97	82	105	105	84	113	105	126	86	65	100	93	100
149	111	109	134	89	87	131	123	96	125	98	123	137	98	89	97	100	83	91	87	146	116	90	149	141	74	88	82	98	71	79	110	104	75	92	150	90	94	97	99	82	93	85	96	121	95	133	81	92	98	65	76
148	109	119	120	106	92	124	134	105	130	110	126	133	94	104	101	112	76	122	106	145	111	90	141	139	100	82	89	100	89	102	105	116	96	108	138	102	103	84	109	86	95	89	85	117	103	130	93	73	99	107	112
151	126	124	134	75	69	138	134	114	138	85	130	135	98	108	119	104	73	104	86	150	108	80	149	139	98	52	97	93	110	50	107	109	76	91	147	106	98	70	96	93	84	58	113	132	82	138	93	98	101	102	103
141	120	116	122	101	92	127	131	110	131	98	118	130	84	92	101	102	89	97	90	148	110	63	138	138	95	78	102	103	104	77	105	115	93	92	134	110	93	86	103	94	99	77	95	123	98	122	90	65	96	88	100
150	109	105	126	102	92	119	126	95	126	105	107	141	95	96	98	110	87	91	82	144	115	77	144	153	102	98	88	102	98	104	111	108	96	92	149	103	103	113	92	90	100	106	97	115	98	127	71	83	104	94	107
151	109	129	123	89	100	126	117	102	124	103	121	134	97	86	103	123	110	115	75	148	112	96	150	143	95	109	96	97	83	94	108	118	69	88	148	95	97	108	94	101	114	109	88	124	92	140	97	93	93	110	98
146	94	123	120	117	105	114	126	101	116	106	111	132	95	104	90	110	84	112	105	140	113	101	143	142	108	96	87	100	89	105	105	109	105	109	143	96	105	99	108	88	96	101	90	110	116	117	90	85	86	97	108
140	118	119	125	81	73	130	131	106	131	89	127	128	92	96	105	116	93	111	87	141	111	76	143	139	98	73	90	97	97	79	105	103	79	80	143	103	95	86	97	85	86	80	93	127	85	140	87	80	106	96	94
139	95	120	102	108	115	109	115	93	106	127	103	139	72	81	77	110	102	90	102	139	114	104	138	135	89	114	98	111	80	104	108	117	89	100	141	96	95	124	96	94	109	106	66	117	111	114	91	97	98	82	70
142	114	111	128	99	83	124	130	101	125	105	116	136	102	93	91	111	64	95	95	145	121	78	140	144	87	83	92	103	96	94	106	109	95	93	141	105	91	92	97	86	89	97	89	129	96	128	81	84	102	82	97
146	112	127	129	97	84	123	127	98	125	99	125	134	93	93	98	121	81	109	92	146	112	94	145	141	95	89	90	95	96	100	109	122	93	91	148	102	89	95	102	93	86	101	99	128	94	140	87	76	112	92	113
140	119	119	124	88	86	124	122	107	132	105	120	136	97	88	105	116	83	96	81	140	114	77	141	142	91	87	92	105	106	78	106	107	89	85	138	107	90	91	88	95	101	82	98	122	93	130	95	92	107	102	92
149	100	124	125	105	103	126	120	102	119	111	120	134	88	77	93	108	86	100	77	147	117	106	144	141	89	106	95	97	70	100	113	124	69	98	145	89	92	107	89	91	105	97	75	119	96	129	89	89	86	93	87
144	115	113	126	87	75	128	133	100	131	92	127	133	107	103	112	116	75	125	109	146	125	84	139	141	94	69	81	99	90	91	111	105	106	91	142	95	107	82	109	75	84	83	105	123	100	134	78	81	113	89	110
140	103	119	111	115	114	107	116	92	112	121	104	135	88	104	92	103	107	101	109	141	122	111	137	137	100	115	103	114	88	109	111	112	104	100	139	99	112	121	103	106	109	109	81	120	120	112	93	99	112	93	75
136	99	123	116	104	92	109	110	106	117	121	111	139	81	95	88	100	106	93	97	150	109	101	140	137	101	102	77	88	92	109	101	110	93	101	141	106	97	117	100	104	105	99	79	111	97	122	93	93	69	96	85
146	96	120	115	114	110	107	111	101	107	119	112	140	98	92	82	99	97	82	89	144	115	98	145	142	97	106	98	99	91	107	110	107	92	93	149	98	101	114	89	97	100	107	79	118	100	114	93	100	83	80	70
138	103	116	116	102	105	111	117	114	125	119	115	132	92	60	91	112	86	101	94	141	119	91	140	141	62	108	89	92	70	101	114	121	79	100	137	105	71	116	100	100	98	104	74	119	92	136	103	94	85	87	77
150	117	129	118	104	103	126	121	134	129	108	120	134	106	95	104	113	96	71	95	145	113	89	143	144	99	96	101	110	99	90	112	117	96	120	138	112	89	108	104	111	93	92	103	120	86	131	106	105	109	104	93
145	110	128	131	82	72	134	121	108	129	91	123	135	92	91	106	110	81	101	74	148	115	87	149	147	102	82	84	89	89	80	106	115	74	87	147	97	85	93	94	95	100	92	95	133	92	133	68	71	90	79	100
146	113	126	132	91	94	137	126	111	136	96	127	140	105	89	106	120	111	105	88	152	122	89	150	145	98	106	96	97	99	99	126	125	71	98	151	109	98	114	102	104	97	107	95	128	76	139	100	99	97	110	102
140	110	120	120	100	90	126	118	123	131	107	114	137	80	88	99	96	92	91	75	139	107	86	137	134	80	87	82	87	89	87	99	115	85	97	133	112	80	103	96	98	95	81	86	116	91	130	88	78	90	80	84
136	111	113	117	102	89	124	120	108	129	114	118	137	86	73	94	101	63	87	95	143	121	77	133	131	95	86	88	99	101	86	117	110	85	100	135	106	85	107	93	94	86	81	82	120	95	136	89	89	106	89	95
151	113	129	124	92	83	125	127	113	132	99	119	143	102	100	89	109	71	96	91	147	115	95	150	146	112	95	89	95	110	106	107	119	97	96	152	103	107	96	106	92	98	97	89	129	100	138	87	84	98	91	117
147	117	132	117	95	99	118	122	115	121	111	117	137	85	93	85	118	109	96	84	149	104	93	144	143	92	106	92	93	81	91	101	110	75	99	146	85	92	117	90	99	97	99	72	120	90	139	99	87	97	99	72
145	107	120	125	93	94	127	131	104	124	104	117	140	81	88	95	115	83	100	92	147	119	98	140	136	85	94	98	99	79	85	109	120	70	89	141	90	86	98	84	103	98	87	83	122	100	126	89	85	96	92	83
149	108	132	121	96	97	128	124	114	126	110	121	136	84	80	103	112	98	115	92	150	114	86	148	144	82	97	87	100	75	93	116	118	81	97	143	104	74	99	104	88	92	92	89	124	82	131	96	93	94	91	94
148	102	116	117	106	102	116	121	104	117	115	128	138	74	74	84	110	85	101	78	145	108	94	147	144	86	101	92	81	84	92	102	117	69	86	148	95	92	115	86	94	102	97	57	115	95	127	90	74	73	81	76
123	112	136	117	122	130	104	103	120	116	126	96	141	92	107	110	115	133	100	90	138	115	123	130	131	120	132	102	118	100	116	106	123	106	119	132	114	110	131	103	124	128	120	92	123	117	107	107	119	99	114	85
140	101	115	103	114	107	97	95	110	108	118	100	139	75	97	82	101	101	86	95	136	101	99	142	141	87	106	89	100	90	110	103	104	106	116	147	96	99	111	96	101	100	108	83	104	101	119	92	92	80	80	80
139	98	113	111	103	109	126	118	83	115	110	119	133	93	88	81	117	109	105	80	141	109	95	137	135	95	109	106	110	80	100	106	114	82	85	139	94	100	118	98	90	122	108	79	119	110	122	87	85	98	81	82
149	101	107	117	115	105	111	115	89	118	129	113	139	94	98	82	103	99	97	91	141	105	106	140	139	101	107	84	82	89	110	104	119	100	93	145	94	98	111	90	85	103	106	94	105	116	123	85	87	101	69	81
147	114	121	129	87	78	134	138	113	137	101	127	130	91	92	101	108	89	99	91	148	117	87	143	141	93	85	78	93	84	90	98	116	78	95	143	103	83	102	92	98	80	84	84	128	82	139	86	90	105	72	80
130	117	109	121	121	124	117	130	117	126	128	118	137	110	122	95	114	118	105	103	139	91	126	129	134	124	120	110	119	118	116	102	90	117	119	134	116	126	128	115	112	126	123	108	94	129	116	118	114	113	110	111
143	110	97	112	117	123	106	113	106	122	126	115	142	107	115	106	101	114	102	109	142	91	105	142	140	109	122	111	117	99	118	111	99	109	120	147	106	110	121	105	116	117	116	108	79	118	130	119	119	112	118	99
134	101	122	118	118	120	116	102	127	109	130	109	139	101	120	106	108	126	104	112	139	81	115	139	138	117	125	96	111	94	114	97	105	111	124	144	112	117	130	115	113	118	121	95	91	112	118	114	117	99	114	106
140	105	111	122	111	107	123	106	105	112	109	109	145	107	101	101	101	104	104	105	137	115	96	140	139	101	108	111	102	97	101	118	115	105	102	138	108	106	106	103	109	120	106	115	110	105	116	103	105	82	106	117
146	113	129	115	82	81	124	126	107	129	97	109	133	99	97	120	107	99	86	79	147	109	84	143	140	91	95	72	74	99	94	103	113	84	86	145	100	87	107	88	113	91	98	107	121	68	139	98	105	96	107	95
148	95	111	110	115	115	106	114	95	107	115	99	132	98	93	97	92	103	97	100	147	112	95	145	146	98	107	102	107	92	108	114	110	105	97	149	85	101	108	104	91	111	101	96	110	113	118	94	103	98	84	84
131	95	115	113	126	111	110	118	75	107	126	98	141	88	105	103	102	101	119	110	137	113	111	131	130	104	107	96	107	93	112	108	114	99	94	135	100	111	105	100	89	115	106	87	115	120	106	95	95	100	89	99
139	97	118	110	114	119	99	98	109	112	124	91	133	107	116	118	84	110	103	122	137	129	112	133	137	116	117	113	120	115	111	121	109	117	116	136	114	115	119	109	113	127	115	120	117	114	104	106	111	89	112	114
134	88	120	98	121	114	106	108	107	101	120	103	130	102	116	111	103	118	115	112	141	113	106	124	128	111	107	114	108	117	110	112	113	112	110	130	103	115	112	119	109	122	112	107	118	113	105	112	102	107	113	117
144	119	126	121	96	101	120	106	124	119	107	117	144	114	111	118	110	103	103	96	144	112	100	143	146	109	101	108	98	114	93	108	110	100	105	142	98	106	108	95	115	110	108	117	123	95	129	102	106	97	121	112
97	113	131	96	148	138	120	119	136	108	146	113	143	127	117	128	125	130	123	143	123	136	133	88	97	135	136	128	133	139	140	129	125	142	134	92	134	119	137	135	124	134	136	126	131	134	72	130	127	131	133	134
140	98	132	113	129	122	104	103	114	102	138	98	136	90	96	88	108	110	88	105	140	93	107	138	131	99	114	95	104	98	121	93	112	114	118	140	108	97	117	108	107	113	114	90	102	112	103	103	113	91	106	91
141	102	121	123	102	90	118	134	118	125	113	112	140	86	81	81	94	63	89	94	146	112	100	140	140	107	97	85	94	102	104	101	111	85	105	141	98	85	92	94	92	96	92	75	115	102	124	92	81	90	90	94
149	98	112	115	112	110	111	112	88	111	120	110	133	93	93	68	105	103	103	86	142	105	109	145	146	104	112	95	104	79	104	93	103	101	81	145	90	99	118	93	82	112	112	75	108	116	121	83	92	89	70	77
140	114	128	126	88	74	126	126	109	129	98	118	135	85	87	110	104	91	85	86	151	108	74	144	138	87	78	97	96	102	81	112	115	82	89	142	109	85	90	102	107	92	79	95	123	80	138	99	94	101	102	101
145	111	113	131	88	77	128	131	94	128	98	127	138	81	93	102	99	81	98	87	151	99	95	145	141	101	82	96	79	106	86	96	114	91	77	144	99	93	93	92	97	96	83	92	116	93	139	96	76	94	94	96
148	111	125	119	101	95	131	125	101	120	103	114	137	78	90	95	125	102	123	90	143	103	91	144	135	87	94	94	104	77	95	100	121	79	92	145	90	96	99	102	82	101	90	68	124	95	132	89	80	106	106	93
134	106	123	124	105	107	117	124	111	119	116	115	137	53	72	83	116	95	106	83	147	95	90	138	135	89	100	100	103	83	99	82	118	74	100	133	101	80	106	107	97	105	93	58	118	98	114	95	79	88	97	69
148	112	122	123	90	91	124	123	111	125	101	121	138	70	89	80	115	101	98	48	147	98	84	146	148	97	91	90	81	93	83	87	120	60	86	151	100	89	92	89	90	105	88	73	122	87	135	89	79	89	98	77
142	108	117	128	92	89	133	127	108	126	99	125	134	87	74	92	96	78	81	80	141	112	85	140	139	92	83	92	103	91	84	99	102	78	100	139	107	81	98	95	79	96	89	93	130	91	120	77	88	86	86	77
149	113	127	119	117	118	107	118	107	119	118	108	139	85	90	81	108	104	82	54	143	106	105	140	144	94	116	101	101	83	106	97	113	82	108	143	101	94	113	76	104	106	110	69	112	107	123	100	95	86	98	74
110	108	137	88	146	138	89	93	115	112	150	93	145	119	114	98	108	122	121	127	133	123	140	114	118	132	141	113	121	121	135	122	116	134	128	117	119	115	137	125	114	133	141	105	124	140	106	112	115	115	118	121

146	109	129	127	86	84	130	123	108	123	98	126	130	98	79	111	108	103	110	94	144	118	90	144	140	93	94	93	102	85	87	107	113	86	91	141	104	70	105	98	101	92	97	97	127	70	132	104	104	106	96	92
143	113	133	130	90	85	130	124	127	126	103	119	136	97	82	109	108	100	86	91	149	111	83	147	146	88	99	76	95	89	82	112	107	74	99	144	108	84	105	82	105	98	89	94	123	69	133	104	99	90	112	99
108	119	141	114	145	137	109	109	135	121	147	103	142	119	127	114	122	136	122	130	109	130	135	120	111	134	136	122	130	131	134	129	120	137	131	119	129	128	135	137	122	139	138	121	127	140	111	120	126	125	120	134
113	119	136	96	141	140	105	101	129	114	148	98	146	118	122	118	117	130	127	136	117	126	137	112	105	134	136	119	132	128	140	132	118	141	137	118	124	118	143	129	123	129	133	123	124	137	101	121	130	129	126	132
151	100	117	120	99	84	120	122	102	118	116	118	138	91	78	104	107	83	93	95	143	108	87	143	138	75	83	82	89	65	92	111	103	76	90	144	94	86	92	86	81	75	80	81	111	78	125	92	100	105	93	84
148	107	124	120	111	108	117	115	110	117	114	117	141	79	92	83	113	101	107	66	148	99	100	144	142	100	107	112	101	92	99	98	119	78	96	142	94	100	101	92	102	120	107	74	115	110	125	100	83	92	99	84
132	109	132	127	116	108	117	128	129	119	121	120	134	95	109	88	124	108	104	99	136	109	96	140	144	111	108	109	106	108	103	109	123	89	95	140	116	109	107	111	119	121	106	82	121	108	129	108	95	103	110	92
145	105	103	119	121	106	119	115	108	117	132	116	133	84	92	96	97	96	91	101	143	100	97	148	144	96	110	94	106	92	108	99	91	97	100	145	103	98	117	94	105	109	111	92	94	97	124	102	104	95	81	85
143	116	123	131	101	106	118	127	121	128	113	105	140	93	78	94	112	105	84	88	149	105	91	147	142	83	104	104	98	75	97	111	128	77	104	145	109	80	118	100	107	101	102	75	124	91	134	105	97	95	94	85
130	104	120	92	140	131	95	103	114	111	134	91	139	110	102	103	108	112	118	126	128	120	124	130	132	117	132	111	130	107	136	130	118	125	127	128	123	108	131	133	100	119	127	106	106	123	111	114	123	114	115	124
151	104	116	120	94	94	118	125	103	118	106	113	137	74	82	91	115	91	99	81	143	107	85	149	142	81	94	96	96	69	92	103	123	57	88	148	98	81	101	92	98	97	92	74	117	88	129	91	77	93	87	74
152	120	124	131	94	105	127	117	100	126	104	114	138	103	93	90	113	104	86	75	146	109	102	144	142	93	103	86	110	71	93	114	106	84	103	148	99	91	104	96	96	95	102	102	122	87	127	92	114	96	94	88
144	118	132	133	81	86	140	133	105	137	91	134	135	94	93	93	123	96	105	64	146	117	102	145	144	100	100	102	97	110	83	104	123	63	83	145	99	85	103	86	102	106	96	87	139	88	143	94	86	107	89	88

1	1	1	1	1	1	1	1	1	1	1	1	2	1	1	1	1	2	1	1	1	1	1	1	1	1	1	1	1	1	1	1	1	1	1	1	1	1	1	1	1	1	1	1	1	1	1	1	1	1	1	1
'Nou 5 gou'	'Nou 6 gou'	'Nou 7 gou'	'Nova'	'Ooitakaken-4'	'Orland'	'Osceola'	'Page'	'Pearl'	'Pixie mandarin'	'Robinson'	'Saga Mandarin'	'Sagakashi 34'	'Seihou'	'Seinannohikari'	'Seminole'	'Setomi'	'Shiranuhi'	'Southern Yellow'	'Summer Fresh'	Sweet Spring'	'Tamami'	'Tanikawa Buntan'	'Tsunokagayaki'	'Tsunokaori'	'Tsunonozomi'	'Willking'	'Yellow Pummelo'	'Youkou'	E-647	EnOw21	H・FD-1	HF9	KyOw14	KyOw21	No.1408	Okitsu-46	Okitsu-56	Strain 01	Strain 02	Strain 03	Strain 04	Strain 05	Strain 06	Strain 07	Strain 08	Strain 09	Strain 10	Strain 11	Strain 12	Strain 13	Strain 14
B040	B041	B042	B043	B044	B045	B047	B048	B049	B050	B051	B052	B053	B054	B055	B056	B057	B058	B059	B060	B061	B062	B063	B064	B065	B066	B067	B068	B069	B070	B071	B072	B073	B074	B075	B076	B077	B078	C001	C002	C003	C004	C005	C006	C007	C008	C009	C010	C011	C012	C013	C014
0	0	0	0	1	0	0	0	0	0	0	0	0	0	0	0	0	0	0	2	0	0	0	0	5	0	0	2	0	0	0	0	0	0	0	0	0	0	0	0	0	0	0	0	2	0	0	0	0	0	0	0
128	115	117	136	104	111	129	126	91	132	140	102	116	118	118	100	124	116	89	106	92	132	105	120	107	121	130	94	122	118	119	142	113	105	105	104	109	135	132	127	120	109	108	124	78	97	123	127	119	130	101	126
145	134	112	141	132	139	143	136	130	144	151	143	145	121	138	140	139	145	127	99	134	141	98	131	136	136	143	117	137	138	135	129	136	129	141	134	136	147	135	144	139	123	134	138	114	138	137	144	135	131	133	143
132	128	136	128	119	111	129	125	106	134	131	125	119	124	129	120	124	119	114	101	107	129	88	130	114	125	141	88	124	114	120	142	123	111	115	125	116	134	126	126	113	115	121	119	108	118	124	128	129	138	109	123
146	138	107	132	143	129	139	134	131	141	139	138	138	127	138	138	140	138	142	114	136	144	110	132	141	136	137	119	140	135	140	136	140	133	141	134	134	147	131	138	139	123	141	142	124	136	134	139	133	130	137	132
83	102	147	107	112	120	102	110	129	93	92	104	104	119	106	112	110	104	116	131	114	101	142	113	116	99	93	129	100	103	110	148	109	119	114	112	121	87	101	103	106	131	118	104	119	113	107	91	111	112	118	98
135	128	138	132	134	139	130	132	130	136	137	145	130	135	139	137	139	130	144	142	144	136	131	134	128	127	126	131	131	137	135	139	131	131	133	127	136	135	131	138	131	118	140	140	133	125	135	136	136	137	118	132
120	98	131	112	78	93	110	110	95	110	118	77	94	101	84	85	106	94	116	106	68	113	112	101	78	110	125	97	111	102	79	146	93	75	81	96	85	121	108	99	88	103	78	105	97	98	102	104	86	99	87	102
109	101	135	123	116	124	126	123	119	116	134	115	106	129	127	126	125	106	115	138	107	120	117	116	108	107	123	126	106	120	127	139	112	109	109	137	113	123	115	130	117	127	125	121	112	114	121	119	111	112	112	127
80	96	145	74	113	113	76	70	119	100	63	105	101	116	100	113	87	101	131	139	119	100	137	94	103	86	85	132	95	95	107	147	107	108	119	98	117	89	100	70	99	127	107	102	122	108	99	86	99	100	121	93
80	96	145	74	113	113	76	70	119	100	63	105	101	116	100	113	87	101	131	139	119	100	137	94	103	86	85	132	95	95	107	147	107	108	119	98	117	89	100	70	99	127	107	102	122	108	99	86	99	100	121	93
80	96	145	74	113	113	76	70	119	100	63	105	101	116	100	113	87	101	131	139	119	100	137	94	103	86	85	132	95	95	107	147	107	108	119	98	117	89	100	70	99	127	107	102	122	108	99	86	99	100	121	93
80	96	145	74	113	113	76	70	119	100	63	105	101	116	100	113	87	101	131	139	119	100	137	94	103	86	85	132	95	95	107	147	107	108	119	98	117	89	100	70	99	127	107	102	122	108	99	86	99	100	121	93
94	96	144	108	122	118	92	105	131	94	89	111	108	112	95	111	115	108	126	135	123	97	142	103	118	98	76	142	96	116	106	148	108	120	121	110	123	86	98	112	112	132	117	111	121	114	116	88	102	100	112	108
96	107	144	97	100	103	88	91	100	93	74	90	99	101	98	93	96	99	115	129	108	82	138	83	112	100	93	130	100	102	95	145	104	114	119	99	96	85	98	87	109	134	101	82	132	105	101	81	92	92	122	90
129	128	129	126	113	115	127	127	107	136	137	123	120	121	128	115	130	120	115	121	119	131	117	123	118	126	133	108	126	116	123	140	125	117	119	124	113	141	131	122	127	114	119	124	121	117	124	131	126	130	112	125
80	97	144	87	112	92	75	91	131	80	63	108	110	107	93	92	97	110	130	142	123	94	142	100	104	100	90	144	79	101	108	144	90	108	116	100	120	80	85	103	91	139	113	104	121	102	114	85	98	95	116	86
80	97	144	87	112	92	75	91	131	80	63	108	110	107	93	92	97	110	130	142	123	94	142	100	104	100	90	144	79	101	108	144	90	108	116	100	120	80	85	103	91	139	113	104	121	102	114	85	98	95	116	86
148	141	116	146	148	150	147	150	133	142	149	144	140	133	144	147	146	140	142	115	143	145	115	139	147	142	135	119	148	151	142	134	154	145	148	138	145	144	138	144	153	129	147	147	129	141	138	146	141	137	148	149
104	107	146	125	118	125	109	120	132	110	110	114	112	122	113	128	129	112	115	138	121	104	137	115	117	113	107	136	108	121	118	148	108	109	116	114	123	99	114	120	119	135	127	111	125	114	119	109	122	122	111	116
127	110	140	132	131	127	123	120	129	124	126	129	120	125	123	128	126	120	126	135	132	123	129	116	119	124	120	137	121	132	120	140	124	126	126	129	130	120	120	135	125	124	125	124	126	125	125	130	127	122	120	134
118	114	126	108	96	102	123	107	115	118	115	94	93	111	102	104	110	93	111	105	94	114	113	105	119	107	121	104	102	96	104	144	106	106	111	106	99	117	115	111	119	112	93	92	123	115	104	113	95	108	99	109
96	89	143	104	121	105	94	95	127	86	85	115	104	114	89	114	112	104	132	133	118	91	137	103	112	105	85	134	89	120	108	146	111	115	122	100	125	79	103	121	106	139	110	112	123	115	120	89	103	94	120	109
128	112	141	126	128	122	110	124	128	116	114	112	116	128	118	118	130	116	128	132	118	122	129	119	124	113	125	134	109	127	125	142	124	123	124	134	125	117	116	129	112	131	128	117	122	115	121	115	120	114	120	127
126	128	113	120	113	99	131	119	100	138	136	117	119	125	122	101	124	119	113	93	104	124	119	127	130	126	129	98	118	123	130	136	130	126	125	121	113	126	133	120	139	112	120	120	101	115	121	129	124	122	120	133
126	128	113	120	113	99	131	119	100	138	136	117	119	125	122	101	124	119	113	93	104	124	119	127	130	126	129	98	118	123	130	136	130	126	125	121	113	126	133	120	139	112	120	120	101	115	121	129	124	122	120	133
126	128	113	120	113	99	131	119	100	138	136	117	119	125	122	101	124	119	113	93	104	124	119	127	130	126	129	98	118	123	130	136	130	126	125	121	113	126	133	120	139	112	120	120	101	115	121	129	124	122	120	133
135	128	148	136	137	137	127	128	138	124	131	136	132	139	131	140	136	132	138	144	131	132	135	130	135	130	125	144	132	140	124	152	136	136	136	138	137	134	127	140	134	132	138	129	136	138	135	133	133	132	132	139
129	125	107	118	118	104	126	121	95	135	132	124	121	106	119	115	123	121	109	82	90	134	79	113	116	126	136	58	125	123	120	138	124	108	121	114	111	135	129	130	130	111	111	113	102	110	124	128	124	132	119	130
125	123	140	126	125	126	124	125	129	130	125	128	121	122	120	119	126	121	134	131	122	113	129	121	125	115	121	132	118	120	131	140	120	126	126	129	127	126	121	126	126	129	122	118	133	124	126	117	125	117	112	121
119	105	140	139	117	138	127	128	119	116	133	115	126	132	113	136	139	126	127	132	116	123	131	120	119	118	120	128	120	141	116	144	120	115	120	130	124	120	123	128	125	128	131	115	130	123	129	131	128	122	112	141
92	92	149	103	117	108	87	98	131	93	80	107	94	111	101	102	101	94	143	142	123	99	140	104	107	98	90	137	86	108	113	146	97	115	107	108	108	90	93	118	100	135	113	108	125	102	106	90	104	87	111	105
142	141	113	140	146	137	145	144	132	140	145	141	143	128	141	140	151	143	135	112	135	149	110	135	142	139	129	113	145	146	141	135	142	135	141	138	142	145	137	146	147	132	144	147	123	140	138	139	135	130	139	140
123	130	125	120	126	116	122	118	114	135	131	133	123	119	139	117	130	123	127	124	124	131	105	120	120	120	132	107	125	121	131	135	126	118	119	129	124	134	123	136	134	117	127	130	114	120	127	127	119	128	117	129
115	112	127	115	91	101	117	103	94	119	123	101	107	101	113	88	117	107	106	110	81	114	108	105	99	117	117	96	124	107	99	146	109	92	98	104	102	114	117	111	118	104	98	103	108	103	117	118	110	118	97	116
126	121	115	136	110	124	139	130	102	133	145	119	132	127	121	114	131	132	111	84	102	137	114	121	115	122	136	90	129	127	120	134	122	109	115	131	123	138	131	133	132	111	121	129	105	109	130	134	127	124	104	138
130	119	129	130	113	112	125	131	105	127	137	114	112	112	113	107	123	112	119	119	108	129	109	112	117	120	131	109	122	115	112	139	119	114	116	125	107	133	127	118	117	102	109	124	127	113	120	122	115	124	90	120
130	119	129	130	113	112	125	131	105	127	137	114	112	112	113	107	123	112	119	119	108	129	109	112	117	120	131	109	122	115	112	139	119	114	116	125	107	133	127	118	117	102	109	124	127	113	120	122	115	124	90	120
130	119	129	130	113	112	125	131	105	127	137	114	112	112	113	107	123	112	119	119	108	129	109	112	117	120	131	109	122	115	112	139	119	114	116	125	107	133	127	118	117	102	109	124	127	113	120	122	115	124	90	120
130	119	129	130	113	112	125	131	105	127	137	114	112	112	113	107	123	112	119	120	108	129	109	112	117	120	131	109	122	115	112	139	119	114	116	125	107	133	127	118	117	103	109	124	128	113	120	122	115	125	90	120
130	119	129	130	113	112	125	131	105	127	137	114	112	112	113	107	123	112	119	119	108	129	109	112	117	120	131	109	122	115	112	139	119	114	116	125	107	133	127	118	117	102	109	124	127	113	120	122	115	124	90	120
138	137	141	138	136	139	134	135	136	135	134	138	135	141	142	140	138	135	138	138	141	135	136	135	140	138	135	135	138	141	140	145	141	136	142	140	143	137	137	138	142	127	145	137	141	143	134	136	139	135	136	140
139	134	125	137	137	130	141	137	129	132	139	142	139	135	139	130	130	139	129	129	132	140	122	134	134	137	139	129	141	134	139	133	135	135	136	141	135	142	137	138	137	131	132	137	128	138	132	137	137	140	136	138
117	119	122	119	99	103	125	123	95	117	129	106	120	112	114	90	108	120	94	113	109	115	117	121	114	121	116	105	112	115	123	147	114	110	110	97	110	124	128	112	130	111	101	121	98	110	125	114	110	121	111	108
117	119	122	119	98	102	125	123	95	117	128	105	118	112	114	90	107	118	92	112	108	113	117	120	114	119	116	105	110	114	122	147	113	109	110	96	109	123	128	112	129	111	101	120	98	109	124	112	108	120	110	107
130	123	129	132	125	130	136	127	132	130	136	130	125	120	125	126	123	125	131	133	123	127	132	125	122	124	138	132	130	132	125	137	125	118	130	133	134	126	125	134	132	130	119	126	135	127	125	135	126	124	123	136
134	121	129	129	126	130	127	123	119	128	127	123	122	123	122	120	136	122	124	126	123	130	123	121	125	124	126	123	126	131	123	139	132	124	125	126	125	131	125	125	134	124	126	120	126	127	126	131	128	124	128	137
118	124	136	125	129	132	122	122	131	123	127	129	124	125	126	121	125	124	126	137	129	118	126	117	115	119	117	129	120	122	125	135	122	117	116	132	131	124	123	124	123	132	127	121	124	119	126	116	124	121	116	123
122	118	141	123	119	116	119	128	125	122	121	114	118	122	114	118	129	118	126	136	119	120	124	123	130	120	111	134	120	123	120	147	117	122	128	121	125	115	116	125	121	124	119	127	134	122	128	116	114	124	117	117
139	131	117	135	125	125	136	132	111	141	145	131	128	113	129	133	132	128	120	103	116	137	71	124	122	131	144	89	129	129	123	137	126	111	127	122	119	142	129	142	133	119	126	131	110	119	129	133	128	137	123	139
114	103	123	122	103	117	131	127	110	109	128	103	104	113	99	112	111	104	100	112	95	118	104	118	120	113	111	113	121	113	112	132	109	118	130	120	117	121	118	116	117	122	108	120	121	119	116	106	109	117	122	111
133	127	122	131	126	130	134	130	104	138	140	133	123	129	140	124	130	123	101	105	111	131	100	123	133	131	130	115	136	120	126	129	134	124	132	131	118	133	134	134	136	106	127	130	112	121	127	134	129	126	105	131
121	108	137	128	115	126	120	119	120	112	120	108	111	117	103	120	125	111	126	121	100	123	124	116	119	107	114	118	116	121	101	147	120	115	116	114	108	112	106	120	115	124	116	110	124	122	111	121	115	112	124	124
135	133	131	127	132	122	142	134	127	138	137	135	131	135	132	129	125	131	113	134	129	128	114	128	133	137	137	125	135	128	133	139	133	129	132	137	131	135	136	126	138	127	126	132	128	132	133	134	128	134	127	128
102	78	126	116	102	100	100	101	106	79	105	89	100	105	83	103	118	100	124	121	93	100	120	89	112	95	90	124	104	120	81	137	107	115	113	97	112	99	91	109	102	122	103	98	119	115	113	91	86	93	120	114
123	127	124	131	122	115	127	127	104	137	138	124	128	119	120	120	132	128	98	92	98	134	79	119	124	132	133	82	133	121	114	145	127	117	127	115	113	134	135	128	125	116	113	120	109	120	122	132	131	141	116	133
128	115	118	137	106	113	129	126	93	132	140	104	118	119	120	102	125	118	90	108	94	132	106	121	105	121	130	96	122	120	121	142	114	107	106	106	110	135	132	129	121	110	110	125	78	98	124	127	121	130	102	128

75	84	146	113	115	118	100	111	127	93	96	104	101	107	102	115	116	101	115	137	98	89	147	101	107	96	87	134	94	117	103	150	98	97	104	101	118	75	97	121	111	133	109	101	116	103	113	83	102	91	109	108
75	85	146	113	115	118	100	111	127	94	96	104	101	108	102	115	116	101	115	137	98	89	147	102	108	96	87	134	94	117	104	150	98	98	104	102	118	75	97	121	111	133	110	101	116	103	113	83	102	91	109	108
75	84	146	113	115	118	100	111	127	93	96	104	101	107	102	115	116	101	115	137	98	89	147	101	107	96	87	134	94	117	103	150	98	97	104	101	118	75	97	121	111	133	109	101	116	103	113	83	102	91	109	108
75	84	146	113	115	118	100	111	127	93	96	104	101	107	102	115	116	101	115	137	98	89	147	101	107	96	87	134	94	117	103	150	98	97	104	101	118	75	97	121	111	133	109	101	116	103	113	83	102	91	109	108
75	84	146	113	115	118	100	111	127	93	96	104	101	107	102	115	116	101	115	137	98	89	147	101	107	96	87	134	94	117	103	150	98	97	104	101	118	75	97	121	111	133	109	101	116	103	113	83	102	91	109	108
75	84	146	113	115	118	100	111	127	93	96	104	101	107	102	115	116	101	115	137	98	89	147	101	107	96	87	134	94	117	103	150	98	97	104	101	118	75	97	121	111	133	109	101	116	103	113	83	102	91	109	108
75	84	146	113	115	118	100	111	127	93	96	104	101	107	102	115	116	101	115	137	98	89	147	101	107	96	87	134	94	117	103	150	98	97	104	101	118	75	97	121	111	133	109	101	116	103	113	83	102	91	109	108
75	84	146	113	115	118	100	111	127	93	96	104	101	107	102	115	116	101	115	137	98	89	147	101	107	96	87	134	94	117	103	150	98	97	104	101	118	75	97	121	111	133	109	101	116	103	113	83	102	91	109	108
75	84	146	113	115	118	100	111	127	93	96	104	101	107	102	115	116	101	115	137	98	89	147	101	107	96	87	134	94	117	103	150	98	97	104	101	118	75	97	121	111	133	109	101	116	103	113	83	102	91	109	108
75	84	146	113	115	118	100	111	127	93	96	104	101	107	102	115	116	101	115	137	98	89	147	101	107	96	87	134	94	117	103	150	98	97	104	101	118	75	97	121	111	133	109	101	116	103	113	83	102	91	109	108
75	84	146	113	115	118	100	111	127	93	96	104	101	107	102	115	116	101	115	137	98	89	147	101	107	96	87	134	94	117	103	150	98	97	104	101	118	75	97	121	111	133	109	101	116	103	113	83	102	91	109	108
75	84	146	113	115	118	100	111	127	93	96	104	101	107	102	115	116	101	115	137	98	89	147	101	107	96	87	134	94	117	103	150	98	97	104	101	118	75	97	121	111	133	109	101	116	103	113	83	102	91	109	108
75	84	146	113	115	118	100	111	127	93	96	104	101	107	102	115	116	101	115	137	98	89	147	101	107	96	87	134	94	117	103	150	98	97	104	101	118	75	97	121	111	133	109	101	116	103	113	83	102	91	109	108
75	84	146	113	115	118	100	111	127	93	96	104	101	107	102	115	116	101	115	137	98	89	147	101	107	96	87	134	94	117	103	150	98	97	104	101	118	75	97	121	111	133	109	101	116	103	113	83	102	91	109	108
75	84	146	113	115	118	100	111	127	93	96	104	101	107	102	115	116	101	115	137	98	89	147	101	107	96	87	134	94	117	103	150	98	97	104	101	118	75	97	121	111	133	109	101	116	103	113	83	102	91	109	108
75	84	146	113	115	118	100	111	127	93	96	104	101	107	102	115	116	101	115	137	98	89	147	101	107	96	87	134	94	117	103	150	98	97	104	101	118	75	97	121	111	133	109	101	116	103	113	83	102	91	109	108
125	123	140	126	125	126	124	125	129	130	125	128	121	122	120	119	126	121	134	131	122	113	129	121	125	115	121	132	118	120	131	140	120	126	126	129	127	126	121	126	126	129	122	118	133	124	126	117	125	117	112	121
100	93	151	114	117	117	94	111	124	86	92	113	102	112	107	109	106	102	127	138	126	91	148	114	110	97	88	142	92	111	116	149	108	119	115	113	119	91	92	106	101	127	120	106	121	105	111	87	99	99	114	92
118	121	141	120	128	124	108	125	127	126	114	115	113	130	114	125	132	113	135	134	125	123	132	127	133	119	121	132	114	117	131	142	122	124	132	133	125	118	124	123	116	130	131	130	132	122	123	117	121	124	120	120
117	122	142	121	128	124	107	124	128	125	113	116	112	130	115	126	131	112	136	135	125	124	132	128	131	120	121	133	113	115	131	143	122	122	130	133	124	119	124	124	115	129	132	130	131	121	122	118	122	124	118	118
114	123	137	120	108	111	125	123	106	131	132	117	116	123	122	114	125	116	119	117	113	125	124	123	112	124	127	105	122	118	124	144	120	117	114	120	120	123	128	122	125	114	116	123	109	114	127	123	125	130	113	126
129	120	147	134	136	135	127	129	133	127	127	138	129	138	127	140	138	129	133	139	127	129	140	133	137	125	124	141	131	138	129	147	128	132	131	134	135	123	128	138	135	133	142	128	133	133	139	126	133	126	123	136
122	123	122	122	105	104	126	116	102	131	140	121	120	116	125	99	124	120	101	108	104	129	121	118	119	128	136	107	131	121	113	146	119	116	118	115	116	129	136	118	126	115	105	116	110	119	130	132	116	129	116	132
120	98	131	112	78	93	110	110	95	110	118	77	94	101	84	85	106	94	116	106	68	113	112	101	78	110	125	97	111	102	79	146	93	75	81	96	85	121	108	99	88	103	78	105	97	98	102	104	86	99	87	102
120	115	141	126	127	129	116	119	131	118	113	111	111	129	118	126	132	111	124	136	125	124	132	116	125	115	115	133	114	127	118	147	125	127	130	128	121	114	112	127	122	125	132	122	126	119	115	118	120	119	124	123
120	98	131	112	79	94	110	110	95	110	118	77	94	102	84	85	106	94	116	106	69	113	112	101	79	110	125	97	111	103	79	146	94	76	82	96	86	121	108	99	88	104	78	106	97	98	102	104	86	100	88	103
120	98	131	112	78	93	110	110	96	110	118	77	94	101	85	86	106	94	116	106	69	114	112	101	78	111	126	97	111	103	79	147	93	75	82	96	85	121	109	99	89	103	78	105	98	98	102	105	87	99	88	103
143	137	146	137	146	137	136	136	136	140	137	150	136	135	145	140	142	136	144	143	145	144	137	143	143	142	139	143	140	137	143	145	147	148	144	142	143	143	140	147	142	117	146	143	142	138	143	144	141	138	136	141
131	125	144	133	129	127	125	131	121	134	141	135	129	129	137	128	137	129	140	137	129	133	128	133	133	130	122	131	137	130	133	143	139	139	130	127	131	132	132	140	141	114	132	139	137	129	141	133	125	135	128	136
137	137	152	133	133	131	130	125	137	132	135	133	131	131	136	133	144	131	149	141	134	130	142	135	137	136	125	141	137	137	135	143	139	142	134	130	135	127	137	143	143	127	138	136	142	137	143	134	132	133	131	140
149	139	114	139	148	144	141	138	136	141	141	144	143	134	148	139	142	143	139	111	145	147	108	138	149	139	134	124	141	142	143	130	149	141	147	137	142	146	138	141	142	125	145	141	130	138	134	145	140	134	136	138
148	147	144	148	150	154	146	147	152	148	149	151	155	147	152	154	157	155	158	149	156	153	144	152	155	153	145	151	155	154	152	148	157	153	158	151	158	149	152	154	157	136	155	154	156	154	157	157	152	151	142	157
137	134	145	135	127	134	141	132	133	132	137	132	128	134	139	131	138	128	142	136	132	130	140	130	136	138	132	136	135	137	137	139	141	137	133	135	139	132	136	141	141	123	135	135	136	135	144	138	129	133	138	138
130	121	140	138	121	133	131	133	122	121	127	119	121	129	107	125	131	121	128	136	112	121	132	120	124	118	126	134	120	135	111	144	119	116	121	125	116	124	123	117	121	129	127	110	129	130	125	123	128	123	120	130
95	73	143	103	100	95	87	106	114	71	92	100	101	103	95	95	108	101	130	127	102	91	133	103	94	86	97	129	86	104	102	138	93	109	109	104	104	88	71	96	78	128	111	95	119	94	105	84	100	91	117	95
121	125	129	117	122	121	119	119	100	134	128	121	113	123	127	118	121	113	116	118	119	128	113	120	119	121	129	103	124	116	119	141	129	115	124	115	118	126	127	120	129	100	117	120	123	114	120	127	123	133	114	122
119	105	140	139	117	138	127	128	119	116	133	115	126	132	113	136	139	126	127	132	116	123	131	120	119	118	120	128	120	141	116	144	120	115	120	130	124	120	123	128	125	128	131	115	130	123	129	131	128	122	112	141
122	115	116	126	112	108	135	122	95	136	139	116	117	114	123	105	122	117	82	105	81	131	105	119	107	128	134	105	130	116	117	139	113	105	119	113	110	138	134	121	128	117	103	118	96	113	124	129	120	128	117	126
122	116	124	119	98	102	125	116	96	127	132	109	110	108	114	95	116	110	103	88	89	122	111	117	105	118	133	86	118	107	116	138	109	103	106	110	106	128	122	114	123	112	95	113	105	98	117	118	113	127	104	117
122	116	124	119	98	102	125	116	96	127	132	109	110	108	114	95	116	110	103	88	89	122	111	117	105	118	133	86	118	107	116	138	109	103	106	110	106	128	122	114	123	112	95	113	105	98	117	118	113	127	104	117
122	116	124	119	98	102	125	116	96	127	132	109	110	108	114	95	116	110	103	88	89	122	111	117	105	118	133	86	118	107	116	138	109	103	106	110	106	128	122	114	123	112	95	113	105	98	117	118	113	127	104	117
122	116	124	119	98	102	125	116	96	127	132	109	110	108	114	95	116	110	103	88	89	122	111	117	105	118	133	86	118	107	116	138	109	103	106	110	106	128	122	114	123	112	95	113	105	98	117	118	113	127	104	117
97	113	145	120	109	111	113	113	117	108	106	109	107	99	112	99	106	107	105	129	114	100	131	106	105	112	100	123	106	109	114	146	105	98	106	106	112	91	104	117	108	124	105	110	121	98	112	101	108	109	103	102
119	114	137	123	116	104	109	120	111	120	113	121	121	111	115	101	132	121	125	124	111	126	125	124	115	116	119	112	121	118	118	139	108	117	109	113	117	118	116	116	105	126	116	111	129	107	124	115	123	121	94	114
144	140	120	144	143	145	144	147	127	138	147	147	144	130	142	140	143	144	119	99	139	147	83	136	143	146	137	93	143	134	136	136	148	137	142	141	134	146	147	141	138	114	143	142	120	134	139	145	143	141	128	139
117	126	124	117	119	115	127	126	104	128	123	125	123	118	122	111	117	123	101	106	105	117	103	121	125	120	124	100	128	118	123	143	122	117	118	119	121	125	120	117	128	117	113	120	118	112	122	119	120	128	122	118
119	117	128	126	118	122	133	130	109	121	129	124	119	124	127	123	116	119	79	109	109	126	120	123	115	130	134	106	131	119	116	145	128	117	115	124	114	130	137	112	120	119	110	114	104	122	122	128	128	135	123	120
81	97	148	92	117	96	91	88	129	98	79	125	88	111	104	95	78	88	129	138	120	103	145	108	114	104	85	142	84	109	121	144	102	124	116	113	125	88	107	113	105	132	115	118	121	109	119	85	102	101	113	98
81	97	148	92	117	96	91	88	129	98	79	125	88	111	104	95	78	88	129	138	120	103	145	108	114	104	85	142	84	109	121	144	102	124	116	113	125	88	107	113	105	132	115	118	121	109	119	85	102	101	113	98
81	97	148	92	117	96	91	88	129	98	79	125	88	111	104	95	78	88	129	138	120	103	145	108	114	104	85	142	84	109	121	144	102	124	116	113	125	88	107	113	105	132	115	118	121	109	119	85	102	101	113	98
81	97	148	92	117	96	91	88	129	98	79	125	88	111	104	95	78	88	129	138	120	103	145	108	114	104	85	142	84	109	121	144	102	124	116	113	125	88	107	113	105	132	115	118	121	109	119	85	102	101	113	98
100	89	144	102	104	95	79	96	120	83	88	105	103	98	104	95	115	103	143	138	127	99	133	115	103	100	97	131	90	102	113	148	94	115	106	97	109	94	93	115	100	127	103	108	120	101	114	96	101	101	99	102
151	136	113	140	139	146	148	145	137	135	145	139	140	129	145	141	143	140	140	114	143	145	114	139	142	140	134	120	144	140	145	135	147	136	139	139	135	144	134	141	140	126	144	146	131	137	134	146	141	136	138	139
127	110	140	132	131	127	123	120	129	124	126	129	120	125	123	128	126	120	126	135	132	123	129	116	119	124	120	137	121	132	120	140	124	126	126	129	130	120	120	135	125	124	125	124	126	125	125	130	127	122	120	134
118	113	120	131	105	116	126	123	94	126	142	108	104	117	117	103	126	104	90	103	104	126	100	115	120	122	130	100	128	119	114	137	123	117	123	110	113	132	130	125	125	95	109	122	109	112	124	123	112	126	100	130
126	97	134	122	70	98	117	120	107	107	118	74	88	92	88	87	106	88	110	117	68	103	131	94	82	104	119	117	101	107	80	145	88	64	60	95	82	111	102	114	91	116	79	87	115	100	99	113	89	97	89	107
127	98	134	123	70	99	118	120	107	108	119	75	89	93	88	88	107	89	110	118	69	103	131	94	83	104	119	118	101	108	80	145	89	65	61	95	83	111	103	115	91	117	79	88	116	101	100	113	89	97	89	108
126	97	134	122	70	98	117	120	107	107	118	74	88	92	88	87	106	88	110	117	68	103	131	94	82	104	119	117	101	107	80	145	88	64	60	95	82	111	102	114	91	116	79	87	115	100	99	113	89	97	89	107
126	97	134	122	70	98	117	120	107	107	118	74	88	92	88	87	106	88	110	117	68	103	131	94	82	104	119	117	101	107	80	145	88	64	60	95	82	111	102	114	91	116	79	87	115	100	99	113	89	97	89	107
126	97	134	122	70	98	117	120	107	107	118	74	88	92	88	87	106	88	110	117	68	103	131	94	82	104	119	117	101	107	80	145	88	64	60	95	82	111	102	114	91	116	79	87	115	100	99	113	89	97	89	107
126	97	134	122	70	98	117	120	107	107	118	74	88	92	88	87	106	88	110	117	68	103	131	94	82	104	119	117	101	107	80	145	88	64	60	95	82	111	102	114	91	116	79	87	115	100	99	113	89	97	89	107
126	97	134	122	70	98	117	120	107	107	118	74	88	92	88	87	106	88	110	117	68	103	131	94	82	104	119	117	101	107	80	145	88	64	60	95	82	111	102	114	91	116	79	87	115	100	99	113	89	97	89	107
126	97	134	122	70	98	117	120	107	107	118	74	88	92	88	87	106	88	110	117	68	103	131	94	82	104	119	117	101	107	80	145	88	64	60	95	82	111	102	114	91	116	79	87	115	100	99	113	89	97	89	107
126	97	134	122	70	98	117	120	107	107	118	74	88	92	88	87	106	88	110	117	68	103	131	94	82	104	119	117	101	107	80	145	88	64	60	95	82	111	102	114	91	116	79	87	115	100	99	113	89	97	89	107
126	97	134	122	70	98	117	120	107	107	118	74	88	92	88	87	106	88	110	117	68	103	131	94	82	104	119	117	101	107	80	145	88	64	60	95	82	111	102	114	91	116	79	87	115	100	99	113	89	97	89	107
126	97	134	122	70	98	117	120	107	107	118	74	88	92	88	87	106	88	110	117	68	103	131	94	82	104	119	117	101	107	80	145	88	64	60	95	82	111	102	114	91	116	79	87	115	100	99	113	89	97	89	107
126	97	134	122	70	98	117	120	107	107	118	74	88	92	88	87	106	88	110	117	68	103	131	94	82	104	119	117	101	107	80	145	88	64	60	95	82	111	102	114	91	116	79	87	115	100	99	113	89	97	89	107
126	97	134	122	70	98	117	120	107	107	118	74	88	92	88	87	106	88	110	117	68	103	131	94	82	104	119	117	101	107	80	145	88	64	60	95	82	111	102	114	91	116	79	87	115	100	99	113	89	97	89	107
126	97	134	122	70	98	117	120	107	107	118	74	88	92	88	87	106	88	110	117	68	103	131	94	82	104	119	117	101	107	80	145	88	64	60	95	82	111	102	114	91	116	79	87	115	100	99	113	89	97	89	107
126	97	134	122	70	98	117	120	107	107	118	74	88	92	88	87	106	88	110	117	68	103	131	94	82	104	119	117	101	107	80	145	88	64	60	95	82	111	102	114	91	116	79	87	115	100	99	113	89	97	89	107
126	97	134	122	70	98	117	120	107	107	118	74	88	92	88	87	106	88	110	117	68	103	131	94	82	104	119	117	101	107	80	145	88	64	60	95	82	111	102	114	91	116	79	87	115	100	99	113	89	97	89	107
126	97	134	122	70	98	117	120	107	107	118	74	88	92	88	87	106	88	110	117	68	103	131	94	82	104	119	117	101	107	80	145	88	64	60	95	82	111	102	114	91	116	79	87	115	100	99	113	89	97	89	107
126	97	134	122	70	98	117	120	107	107	118	74	88	92	88	87	106	88	110	117	68	103	131	94	82	104	119	117	101	107	80	145	88	64	60	95	82	111	102	114	91	116	79	87	115	100	99	113	89	97	89	107
126	97	134	122	70	98	117	120	107	107	118	74	88	92	88	87	106	88	110	117	68	103	131	94	82	104	119	117	101	107	80	145	88	64	60	95	82	111	102	114	91	116	79	87	115	100	99	113	89	97	89	107
126	97	134	122	70	98	117	120	107	107	118	74	88	92	88	87	106	88	110	117	68	103	131	94	82	104	119	117	101	107	80	145	88	64	60	95	82	111	102	114	91	116	79	87	115	100	99	113	89	97	89	107

126	97	134	122	70	98	117	120	107	107	118	74	88	92	88	87	106	88	110	117	68	103	131	94	82	104	119	117	101	107	80	145	88	64	60	95	82	111	102	114	91	116	79	87	115	100	99	113	89	97	89	107
114	123	137	120	108	111	125	123	106	131	132	117	116	123	122	114	125	116	119	117	113	125	124	123	112	124	127	105	122	118	124	144	120	117	114	120	120	123	128	122	125	114	116	123	109	114	127	123	125	130	113	126
115	107	145	123	127	127	109	122	132	111	105	122	114	127	111	126	130	114	132	142	130	117	145	123	128	118	105	147	115	127	119	149	120	130	128	123	127	105	114	127	121	126	131	125	129	127	128	115	122	118	115	121
110	113	147	129	130	134	113	121	135	114	113	126	120	126	111	130	126	120	129	140	131	116	144	118	128	119	108	142	117	130	117	149	121	127	130	120	131	107	118	133	120	128	135	127	134	128	130	118	123	120	117	123
111	113	141	124	112	105	119	116	96	131	132	111	112	111	116	103	129	112	97	106	77	123	115	119	107	126	129	106	121	116	114	144	107	100	113	111	109	120	121	123	115	121	114	118	109	97	121	119	120	118	117	122
86	104	144	118	123	126	97	109	126	95	98	110	110	119	106	119	116	110	124	129	113	99	147	102	118	106	100	139	97	116	104	146	112	113	119	107	117	81	98	109	115	129	117	107	119	119	107	93	110	105	116	107
121	125	129	117	121	121	119	119	100	134	128	121	113	123	126	118	121	113	116	118	119	128	113	119	119	120	129	103	124	116	118	141	129	115	124	115	118	126	127	120	128	100	117	120	123	114	120	127	123	133	114	122
121	125	129	117	121	121	119	119	100	134	128	121	113	123	126	118	121	113	116	118	119	128	113	119	119	120	129	103	124	116	118	141	129	115	124	115	118	126	127	120	128	100	117	120	123	114	120	127	123	133	114	122
121	125	129	117	121	121	119	119	100	134	128	121	113	123	126	118	121	113	116	118	119	128	113	119	119	120	129	103	124	116	118	141	129	115	124	115	118	126	127	120	128	100	117	120	123	114	120	127	123	133	114	122
122	125	129	117	121	121	119	119	100	134	128	121	113	123	126	118	121	113	116	118	119	128	113	119	119	120	129	103	124	116	118	142	129	115	124	115	118	126	127	120	128	100	117	120	123	114	120	127	123	133	114	122
121	125	129	117	121	121	119	119	100	134	128	121	113	123	126	118	121	113	116	118	119	128	113	119	119	120	129	103	124	116	118	141	129	115	124	115	118	126	127	120	128	100	117	120	123	114	120	127	123	133	114	122
120	128	145	128	131	135	121	126	131	128	127	128	131	131	132	132	140	131	136	135	126	126	132	134	125	128	131	128	132	122	136	146	122	129	127	135	131	122	124	125	124	135	135	126	132	122	125	128	134	131	127	127
143	137	114	137	138	142	147	144	127	135	142	140	139	133	143	139	142	139	130	100	129	142	97	136	140	138	136	109	141	137	139	133	143	138	139	138	132	143	139	148	143	118	139	138	115	139	129	142	139	133	137	143
91	92	147	105	121	116	82	107	128	86	79	102	96	108	97	104	106	96	129	141	115	93	141	101	112	95	85	143	84	112	103	149	107	108	107	105	117	78	94	114	102	128	118	106	121	99	115	92	104	102	115	101
94	103	150	122	123	129	115	123	131	110	108	118	105	125	114	127	130	105	121	138	127	107	145	123	124	121	102	144	104	124	122	148	112	117	120	119	132	87	109	131	118	133	123	124	121	117	125	107	113	115	108	116
112	122	126	78	91	69	94	78	99	121	98	100	92	94	112	87	83	92	119	120	106	105	115	101	82	109	130	102	97	77	107	143	95	92	97	104	78	122	113	82	103	115	81	88	125	97	88	106	100	114	112	88
113	123	126	80	92	70	96	80	100	122	99	101	92	95	112	88	84	92	120	120	106	105	117	101	83	110	131	104	97	78	107	143	97	92	98	105	78	122	113	84	105	116	82	88	126	98	89	107	101	114	113	90
112	122	126	78	91	69	94	78	99	121	98	100	92	94	112	87	83	92	119	120	106	105	115	101	82	109	130	102	97	77	107	143	95	92	97	104	78	122	113	82	103	115	81	88	125	97	88	106	100	114	112	88
112	122	126	78	91	69	94	78	99	121	98	100	92	94	112	87	83	92	119	120	106	105	115	101	82	109	130	102	97	77	107	143	95	92	97	104	78	122	113	82	103	115	81	88	125	97	88	106	100	114	112	88
112	122	126	78	91	69	94	78	99	121	98	100	92	94	112	87	83	92	119	120	106	105	115	101	82	109	130	102	97	77	107	143	95	92	97	104	78	122	113	82	103	115	81	88	125	97	88	106	100	114	112	88
112	122	126	78	91	69	94	78	99	121	98	100	92	94	112	87	83	92	119	120	106	105	115	101	82	109	130	102	97	77	107	143	95	92	97	104	78	122	113	82	103	115	81	88	125	97	88	106	100	114	112	88
112	122	126	78	91	69	94	78	99	121	98	100	92	94	112	87	83	92	119	120	106	105	115	101	82	109	130	102	97	77	107	143	95	92	97	104	78	122	113	82	103	115	81	88	125	97	88	106	100	114	112	88
113	122	126	79	92	69	95	78	99	121	98	100	92	95	113	87	83	92	119	120	106	105	115	100	83	109	130	102	97	77	108	143	95	93	98	105	79	122	113	82	104	115	82	88	125	98	89	106	100	114	112	88
112	122	126	78	91	69	94	78	99	121	98	100	92	94	112	87	83	92	119	120	106	105	115	101	82	109	130	102	97	77	107	143	95	92	97	104	78	122	113	82	103	115	81	88	125	97	88	106	100	114	112	88
112	122	126	78	91	69	94	78	99	121	98	100	92	94	112	87	83	92	119	120	106	105	115	101	82	109	130	102	97	77	107	143	95	92	97	104	78	122	113	82	103	115	81	88	125	97	88	106	100	114	112	88
112	122	126	78	91	69	94	78	99	121	98	100	92	94	112	87	83	92	119	120	106	105	115	101	82	109	130	102	97	77	107	143	95	92	97	104	78	122	113	82	103	115	81	88	125	97	88	106	100	114	112	88
112	122	126	78	91	69	94	78	99	121	98	100	92	94	112	87	83	92	119	120	106	105	115	101	82	109	130	102	97	77	107	143	95	92	97	104	78	122	113	82	103	115	81	88	125	97	88	106	100	114	112	88
112	122	126	78	91	69	94	78	99	121	98	100	92	94	112	87	83	92	119	120	106	105	115	101	82	109	130	102	97	77	107	143	95	92	97	104	78	122	113	82	103	115	81	88	125	97	88	106	100	114	112	88
112	122	126	78	91	69	94	78	99	121	98	100	92	94	112	87	83	92	119	120	106	105	115	101	82	109	130	102	97	77	107	143	95	92	97	104	78	122	113	82	103	115	81	88	125	97	88	106	100	114	112	88
112	122	126	78	91	69	94	78	99	121	98	100	92	94	112	87	83	92	119	120	106	105	115	101	82	109	130	102	97	77	107	143	95	92	97	104	78	122	113	82	103	115	81	88	125	97	88	106	100	114	112	88
112	122	126	78	91	69	94	78	99	121	98	100	92	94	112	87	83	92	119	120	106	105	115	101	82	109	130	102	97	77	107	143	95	92	97	104	78	122	113	82	103	115	81	88	125	97	88	106	100	114	112	88
112	122	126	78	91	69	94	78	99	121	98	100	92	94	112	87	83	92	119	120	106	105	115	101	82	109	130	102	97	77	107	143	95	92	97	104	78	122	113	82	103	115	81	88	125	97	88	106	100	114	112	88
112	122	126	78	91	69	94	78	99	121	98	100	92	94	112	87	83	92	119	120	106	105	115	101	82	109	130	102	97	77	107	143	95	92	97	104	78	122	113	82	103	115	81	88	125	97	88	106	100	114	112	88
111	122	126	79	91	68	93	78	98	121	99	100	93	94	111	87	84	93	118	121	105	105	114	101	82	108	129	103	98	76	107	143	94	92	96	104	77	123	112	81	102	115	81	89	124	97	87	105	99	115	111	87
117	112	143	130	130	134	122	126	134	110	119	130	129	132	120	136	134	129	132	139	130	121	145	124	130	118	115	144	120	142	125	145	126	131	128	129	136	115	118	137	128	138	132	126	132	129	131	120	129	124	124	134
116	111	143	128	131	131	119	126	137	109	116	127	122	132	112	136	133	122	132	140	132	119	144	125	130	121	112	144	114	140	123	145	130	134	135	127	136	111	121	135	125	131	134	129	131	130	132	117	128	124	123	133
117	111	143	128	131	131	119	126	137	109	116	127	122	132	112	136	133	122	132	140	132	119	144	125	130	121	112	144	114	140	123	145	130	134	135	127	136	112	121	135	125	131	134	129	131	130	132	117	128	124	123	133
116	111	143	128	131	131	119	126	137	109	116	127	122	132	112	136	133	122	132	140	132	119	144	125	130	121	112	144	114	140	123	145	130	134	135	127	136	111	121	135	125	131	134	129	131	130	132	117	128	124	123	133
117	112	143	130	130	134	122	126	134	110	119	130	129	132	120	136	134	129	132	139	130	121	145	124	130	118	115	144	120	142	125	145	126	131	128	129	136	115	118	137	128	138	132	126	132	129	131	120	129	124	124	134
117	112	143	130	130	134	122	126	134	110	119	130	129	132	120	136	134	129	132	139	130	121	145	124	130	118	115	144	120	142	125	145	126	131	128	129	136	115	118	137	128	138	132	126	132	129	131	120	129	124	124	134
119	115	144	126	126	133	114	128	138	107	110	123	124	135	119	132	130	124	129	141	131	120	148	125	133	123	108	145	120	134	125	145	125	130	133	126	132	109	120	133	123	132	133	121	132	135	132	119	124	125	119	124
116	111	143	128	131	131	119	126	137	109	116	127	122	132	112	136	133	122	132	140	132	119	144	125	130	121	112	144	114	140	123	145	130	134	135	127	136	111	121	135	125	131	134	129	131	130	132	117	128	124	123	133
116	111	143	128	131	131	119	126	137	109	116	127	122	132	112	136	133	122	132	140	132	119	144	125	130	121	112	144	114	140	123	145	130	134	135	127	136	111	121	135	125	131	134	129	131	130	132	117	128	124	123	133
116	111	143	128	131	131	119	126	137	109	116	127	122	132	112	136	133	122	132	140	132	119	144	125	130	121	112	144	114	140	123	145	130	134	135	127	136	111	121	135	125	131	134	129	131	130	132	117	128	124	123	133
117	112	143	130	130	134	122	126	134	110	119	130	129	132	120	136	134	129	132	139	130	121	145	124	130	118	115	144	120	142	125	145	126	131	128	129	136	115	118	137	128	138	132	126	132	129	131	120	129	124	124	134
116	111	143	128	131	131	119	126	137	109	116	127	122	132	112	136	133	122	132	140	132	119	144	125	130	121	112	144	114	140	123	145	130	134	135	127	136	111	121	135	125	131	134	129	131	130	132	117	128	124	123	133
128	112	141	126	128	122	110	124	128	116	114	112	116	128	118	118	130	116	128	132	118	122	129	119	124	113	125	134	109	127	125	142	124	123	124	134	125	117	116	129	112	131	128	117	122	115	121	115	120	114	120	127
123	130	125	120	126	116	122	118	114	135	131	133	123	119	139	117	130	123	127	124	124	131	105	120	120	120	132	107	125	121	131	135	126	118	119	129	124	134	123	136	134	117	127	130	114	120	127	127	119	128	117	129
100	96	143	91	100	98	83	88	116	87	98	110	103	106	112	97	98	103	134	137	114	90	131	104	97	86	101	126	88	95	115	144	90	107	96	114	101	111	98	101	96	127	108	101	113	94	109	93	99	103	108	100
100	96	143	91	100	98	83	88	116	87	98	110	103	106	112	97	98	103	134	137	114	90	131	104	97	86	101	126	88	95	115	144	90	107	96	114	101	111	98	101	96	127	108	101	113	94	109	93	99	103	108	100
100	96	143	91	100	98	83	88	116	87	98	110	103	106	112	97	98	103	134	137	114	90	131	104	97	86	101	126	88	95	115	144	90	107	96	114	101	111	98	101	96	127	108	101	113	94	109	93	99	103	108	100
100	96	143	91	100	98	83	88	116	87	98	110	103	106	112	97	98	103	134	137	114	90	131	104	97	86	101	126	88	95	115	144	90	107	96	114	101	111	98	101	96	127	108	101	113	94	109	93	99	103	108	100
98	99	141	93	116	99	87	88	103	101	102	115	103	102	107	107	106	103	127	129	117	104	113	97	85	105	108	114	94	103	107	143	99	97	100	103	105	107	103	97	94	126	105	110	112	90	96	90	99	102	111	109
122	117	122	115	99	113	126	116	106	119	115	112	113	109	103	105	119	113	110	89	99	123	117	114	120	117	121	95	109	110	116	138	112	112	116	111	108	120	125	109	120	119	111	106	115	114	120	121	118	114	112	118
93	103	142	100	115	96	82	88	108	106	95	102	98	92	100	91	106	98	127	130	110	103	124	96	95	95	99	122	92	93	98	139	97	113	96	97	106	101	101	109	93	127	96	110	108	87	91	81	99	101	112	99
118	121	141	120	128	124	108	125	127	126	114	115	113	130	114	125	132	113	135	134	125	123	132	127	133	119	121	132	114	117	131	142	122	124	132	133	125	118	124	123	116	130	131	130	132	122	123	117	121	124	120	120
121	100	132	113	80	95	111	112	96	111	119	78	96	102	86	86	107	96	116	106	69	113	114	103	80	111	125	99	113	104	80	146	95	76	82	98	86	122	109	99	89	103	80	106	98	99	104	104	87	99	89	102
144	140	120	144	143	145	144	147	127	138	147	147	144	130	142	140	143	144	119	99	139	147	83	136	143	146	137	93	143	134	136	136	148	137	142	141	134	146	147	141	138	114	143	142	120	134	139	145	143	141	128	139
123	100	144	122	125	122	107	119	132	110	110	113	115	120	104	117	131	115	137	137	124	116	129	111	118	107	104	141	110	123	110	145	113	122	117	118	124	117	107	124	104	128	123	120	122	118	123	115	115	109	105	118
120	98	131	112	78	93	110	110	95	110	118	77	94	101	84	85	106	94	116	106	68	113	112	101	78	110	125	97	111	102	79	146	93	75	81	96	85	121	108	99	88	103	78	105	97	98	102	104	86	99	87	102
120	98	131	112	78	93	110	110	95	110	118	77	94	101	84	85	106	94	116	106	68	113	112	101	78	110	125	97	111	102	79	146	93	75	81	96	85	121	108	99	88	103	78	105	97	98	102	104	86	99	87	102
120	98	131	112	78	93	110	110	95	110	118	77	94	101	84	85	106	94	116	106	68	113	112	101	78	110	125	97	111	102	79	146	93	75	81	96	85	121	108	99	88	103	78	105	97	98	102	104	86	99	87	102
149	138	114	143	149	149	144	146	136	135	145	146	143	128	147	141	145	143	143	115	148	148	100	139	150	138	135	120	143	146	146	133	150	144	147	140	146	145	134	148	147	125	147	148	132	137	140	145	136	136	141	141
115	112	127	115	91	101	117	103	94	119	123	101	107	101	113	88	117	107	106	110	81	114	108	105	99	117	117	96	124	107	99	146	109	92	98	104	102	114	117	111	118	104	98	103	108	103	117	118	110	118	97	116
131	118	137	133	114	118	125	131	116	125	129	102	106	126	112	117	133	106	129	129	108	121	124	109	131	112	125	130	111	123	107	138	117	119	122	125	117	127	109	132	119	119	118	118	128	125	120	127	104	113	118	122
125	124	123	128	118	113	132	130	104	132	142	127	124	127	130	119	128	124	107	109	100	137	105	124	113	126	131	101	132	123	119	142	121	109	118	120	121	139	136	133	130	102	117	128	98	118	126	128	121	124	104	133
100	95	147	103	110	113	87	97	129	88	76	108	106	105	103	102	107	106	132	144	121	83	142	96	111	97	84	139	85	105	101	146	95	115	117	101	118	74	84	100	99	133	101	98	122	102	107	85	92	92	121	93
81	84	146	99	114	105	86	91	119	84	76	100	92	97	90	99	103	92	125	137	110	75	138	83	103	86	67	138	80	106	88	142	98	110	118	95	117	63	87	102	103	133	106	99	118	101	112	72	93	83	117	97
131	125	121	132	98	119	133	126	97	133	144	112	116	125	120	112	129	116	93	108	104	130	115	122	118	126	136	107	128	118	120	141	123	116	115	116	114	139	139	118	128	109	111	125	108	110	123	126	121	133	109	129
130	134	122	121	112	106	129	119	103	137	127	126	127	113	128	107	128	127	100	101	109	128	97	131	118	134	137	96	124	116	130	145	128	121	118	126	115	130	136	118	126	121	114	111	110	123	130	130	135	128	124	124
99	100	134	122	94	116	123	131	118	111	116	79	89	113	81	109	121	89	86	119	63	108	139	107	116	111	115	127	113	107	97	143	92	96	105	103	105	108	119	108	110	120	98	104	114	117	114	98	94	100	110	107
131	126	126	125	112	114	131	126	103	132	139	125	123	120	127	107	129	123	110	114	101	137	95	124	119	134	141	89	134	116	116	141	115	111	115	119	107	136	136	128	128	119	110	122	114	116	127	135	128	138	112	129
102	94	145	108	121	111	95	102	131	93	90	122	117	110	109	117	115	117	129	143	122	95	142	103	116	104	88	146	109	119	104	147	104	119	124	107	122	91	96	109	109	138	114	102	127	116	121	96	108	102	121	109
127	116	113	134	109	112	122	125	92	128	134	102	118	115	114	105	124	118	101	109	101	130	105	113	106	111	126	97	118	117	115	143	115	102	99	105	107	133	121	119	114	116	113	117	90	95	118	124	121	133	107	120

132	132	145	136	133	141	134	134	138	133	135	138	135	140	137	141	141	135	143	140	133	131	136	133	138	133	133	136	135	139	135	151	131	134	134	136	136	128	130	134	139	132	135	125	141	137	139	136	138	136	132	137
105	99	134	103	88	91	96	97	105	99	111	88	97	63	101	72	90	97	123	127	96	87	113	93	76	75	103	117	94	86	95	139	75	92	80	97	80	108	82	103	98	118	70	89	115	72	87	83	85	86	94	88
92	105	137	95	76	88	81	93	116	106	85	100	87	99	101	86	75	87	119	118	100	89	126	87	81	77	104	119	80	69	105	140	80	88	79	111	86	103	89	94	78	117	87	70	102	84	81	83	94	89	92	65
118	113	130	101	81	107	114	97	113	122	114	80	78	91	99	91	85	78	111	119	90	98	124	98	84	94	118	114	97	77	96	146	92	74	79	110	67	114	99	99	99	108	81	93	112	91	81	108	84	101	83	94
115	111	131	119	98	95	110	113	55	113	115	91	108	111	104	88	124	108	107	105	98	106	127	108	124	105	105	114	112	111	105	139	111	129	119	103	109	109	108	109	116	126	101	103	123	108	121	99	93	106	111	110
109	86	136	110	99	108	103	106	106	87	96	77	85	105	83	96	101	85	124	123	92	66	138	67	96	70	89	139	80	112	75	137	101	97	101	100	100	92	74	109	97	126	96	95	123	100	98	77	73	66	120	99
109	116	132	79	98	96	79	88	102	116	90	92	91	110	104	93	96	91	127	128	116	100	124	101	105	93	112	111	91	87	101	146	105	109	108	106	91	117	101	77	100	113	92	89	125	104	95	90	89	104	119	86
94	109	136	96	102	90	94	97	119	116	92	105	76	94	93	96	94	76	126	142	113	83	127	88	81	81	110	130	69	79	100	144	81	91	96	102	78	94	80	96	92	129	92	90	127	86	78	84	92	90	102	89
151	146	61	148	142	147	148	149	140	145	151	144	143	141	147	148	144	143	136	133	145	148	120	144	140	143	148	136	145	144	145	83	145	140	143	144	143	153	141	145	152	137	143	140	129	144	140	152	148	145	140	151
114	118	138	120	119	114	114	117	121	128	115	114	109	105	110	110	119	109	117	124	115	108	111	110	104	115	123	112	101	99	119	145	101	112	110	122	106	112	109	119	108	114	111	119	121	96	99	111	117	111	79	106
93	82	143	102	93	97	82	97	118	93	87	94	97	103	92	101	103	97	135	138	114	85	130	90	100	60	83	125	79	92	78	146	76	107	111	92	102	91	84	91	82	132	86	88	118	93	107	84	94	95	107	93
147	140	109	139	149	142	143	142	133	141	147	142	141	137	145	142	147	141	132	109	139	151	116	135	145	138	138	121	146	147	139	131	147	138	143	140	138	150	136	144	145	130	145	146	125	142	134	146	135	134	141	142
146	135	116	140	145	141	144	142	133	136	143	141	147	127	138	139	147	147	139	112	136	150	110	136	137	140	136	114	143	147	140	137	143	136	138	140	140	143	135	148	143	129	143	147	124	135	137	139	138	128	141	141
109	102	133	103	63	94	83	76	123	95	102	80	100	104	93	100	95	100	131	133	100	102	127	95	91	87	108	129	99	89	88	146	88	89	98	110	99	110	101	76	79	125	95	82	111	98	96	94	96	112	111	94
94	75	149	107	107	102	89	95	112	79	94	93	103	104	81	111	114	103	133	133	101	84	134	75	99	76	77	133	88	118	71	145	100	113	115	90	115	83	80	111	97	135	98	99	120	104	112	80	88	82	122	114
88	95	128	95	97	82	64	77	98	105	78	73	95	83	83	86	103	95	123	126	107	97	127	83	98	93	95	115	91	93	83	140	101	102	109	79	108	93	102	85	103	116	90	92	119	92	104	84	95	99	111	93
96	98	140	71	105	76	68	69	105	108	74	87	87	97	93	83	108	87	128	127	103	97	122	82	104	103	101	122	83	81	93	141	104	103	115	95	108	95	90	116	99	124	93	119	119	89	103	94	92	89	101	91
108	99	135	97	76	83	86	97	120	109	93	76	108	86	85	87	106	108	125	135	89	106	116	94	63	96	121	115	104	79	81	152	68	71	67	97	88	116	99	76	59	130	76	82	118	82	96	104	101	109	109	73
95	85	141	101	107	100	80	91	118	87	86	104	98	101	93	93	110	98	136	141	108	90	137	82	108	84	94	132	87	103	95	144	88	112	117	101	108	84	75	106	95	130	99	88	117	102	105	76	88	84	120	102
114	109	138	115	117	115	106	108	114	114	109	105	86	87	110	106	119	86	127	120	107	102	116	95	99	104	113	119	97	104	102	138	115	107	106	110	82	107	91	126	108	115	103	102	129	90	85	110	101	96	95	108
118	105	129	129	119	111	115	119	115	107	118	104	100	118	103	107	115	100	113	119	100	119	119	105	128	101	111	119	104	117	106	137	121	127	121	115	109	117	115	112	118	114	112	97	121	123	106	112	115	114	116	125
84	90	143	91	88	90	84	87	123	104	84	100	86	93	101	89	87	86	130	145	107	76	131	90	73	77	104	137	67	77	102	148	71	89	91	99	103	85	78	95	83	131	92	87	109	77	99	74	83	83	102	75
84	97	139	104	99	99	100	111	122	100	94	94	69	105	75	100	97	69	114	127	88	94	137	99	104	93	99	129	72	99	103	133	75	96	107	106	110	87	96	108	103	131	100	99	108	101	99	78	95	88	107	97
145	136	116	146	146	145	149	148	135	141	147	143	143	135	147	142	147	143	132	113	140	148	112	138	146	136	136	115	144	145	144	127	145	139	144	142	144	146	135	141	140	134	149	144	125	142	136	144	139	134	142	140
112	105	137	115	103	103	100	105	106	110	107	101	90	104	103	99	108	90	118	130	89	103	128	94	92	105	116	117	99	97	99	143	100	91	88	100	89	110	96	111	102	119	92	103	112	91	87	103	93	96	106	101
93	88	140	89	87	98	84	89	113	95	81	101	89	93	107	88	84	89	124	114	112	89	129	87	80	79	102	119	70	80	104	140	87	97	89	105	97	101	83	99	80	113	92	87	109	84	85	86	94	94	97	73
102	75	145	120	104	117	97	105	113	73	102	95	101	104	90	113	117	101	125	130	104	81	136	92	107	88	69	133	96	120	90	145	109	112	115	109	110	88	89	115	107	135	111	101	126	108	114	85	94	88	121	122
104	99	139	93	114	103	93	90	115	114	94	93	60	106	87	96	96	60	119	134	90	96	127	84	101	88	105	130	68	92	107	145	97	98	108	101	91	104	90	116	102	127	101	112	121	100	74	94	88	89	107	103
98	94	130	125	89	98	102	112	97	104	117	79	96	100	65	97	110	96	116	117	81	107	129	89	97	104	105	118	101	107	63	137	93	88	101	83	104	98	99	106	92	116	84	91	113	100	106	100	90	95	98	106
89	90	141	95	113	115	84	90	119	75	92	87	106	108	90	110	102	106	124	127	112	94	132	90	100	92	83	128	89	110	85	148	106	108	108	99	117	93	98	101	90	126	99	107	115	106	106	91	91	97	116	94
95	83	144	110	105	102	83	90	107	78	100	102	106	99	88	100	110	106	133	131	108	89	129	80	99	82	71	128	88	112	91	141	89	114	108	98	116	93	82	109	94	132	100	101	110	100	109	73	84	77	112	105
100	111	131	89	87	94	98	83	113	118	110	92	81	65	93	88	82	81	121	127	85	94	112	85	44	89	116	111	95	74	95	147	81	55	54	102	74	109	88	97	85	118	75	90	105	67	78	92	77	89	87	82
130	119	129	130	113	112	125	131	105	127	137	114	112	112	113	107	123	112	119	119	108	129	109	112	117	120	131	109	122	115	112	139	119	114	116	125	107	133	127	118	117	102	109	124	127	113	120	122	115	124	90	120
76	100	144	54	109	80	55	84	118	85	48	106	99	101	106	92	85	99	131	136	124	87	132	99	100	92	87	128	82	92	105	146	98	107	110	98	115	81	96	93	85	131	103	93	124	94	114	76	103	100	120	78
143	129	110	135	123	120	138	133	113	128	140	126	129	119	126	120	132	129	123	97	110	140	90	121	127	128	136	76	134	137	123	138	130	119	120	120	109	138	126	136	132	121	120	120	119	130	122	141	130	140	128	136
105	95	134	116	106	97	95	104	102	105	98	78	95	87	75	93	110	95	126	123	88	93	128	73	93	81	96	122	91	108	52	148	102	91	104	52	105	104	83	107	100	119	68	95	118	92	102	96	86	93	113	103
105	90	141	105	99	94	99	104	106	107	108	84	77	83	93	96	104	77	123	132	95	79	124	67	82	64	100	123	87	101	63	148	85	79	91	80	92	100	67	125	99	116	72	106	114	86	99	86	70	77	95	102
111	111	137	89	82	51	96	72	105	105	93	92	88	71	102	41	88	88	118	125	95	106	115	100	102	101	106	114	94	82	99	144	96	105	99	98	95	106	107	101	104	113	81	94	120	92	109	92	93	103	105	94
99	96	132	105	90	108	101	94	104	112	105	63	82	91	80	105	102	82	127	125	83	96	126	88	92	93	117	124	89	99	81	141	87	85	104	91	99	98	92	94	100	121	84	88	124	108	87	94	90	90	110	104
105	107	138	97	85	99	90	90	124	110	100	82	91	86	90	94	99	91	130	130	102	101	120	95	89	99	117	124	97	75	100	150	75	98	98	106	73	117	95	87	90	121	74	82	115	84	55	101	76	94	108	78
0	91	147	94	112	112	90	97	125	95	100	114	106	111	102	119	101	106	111	137	109	96	143	99	96	93	92	129	95	101	109	145	90	107	101	100	124	62	107	107	111	130	108	110	106	93	108	86	106	109	112	106
91	0	142	115	103	107	101	106	112	72	106	90	94	102	94	108	108	94	123	124	88	93	129	99	95	77	86	126	87	115	97	140	89	107	103	99	113	90	83	115	97	127	94	102	107	96	110	91	93	91	109	110
147	142	0	141	133	139	140	141	131	141	148	135	133	132	133	139	135	133	124	119	133	145	124	130	134	136	149	121	140	140	136	90	140	129	138	136	136	146	137	132	148	128	134	130	119	142	132	145	138	138	137	146
94	115	141	0	102	77	70	54	121	110	58	116	95	99	115	94	88	95	130	128	117	98	132	103	99	109	106	123	100	83	121	145	103	105	106	107	104	103	103	90	102	124	100	104	127	100	106	95	95	101	118	78
112	103	133	102	0	95	111	102	114	98	116	74	93	95	89	85	97	93	116	126	89	96	127	101	84	95	118	117	105	86	90	146	73	78	82	106	82	116	102	86	95	115	75	72	113	93	99	98	89	104	96	97
112	107	139	77	95	0	83	80	97	112	85	95	96	91	101	52	93	96	127	121	97	108	117	103	101	105	111	114	94	84	103	144	94	110	101	93	94	115	103	103	99	128	81	93	116	98	115	98	95	104	113	84
90	101	140	70	111	83	0	69	113	97	57	96	103	98	91	92	105	103	139	136	121	99	131	98	102	94	96	127	86	81	99	149	94	105	107	91	105	89	92	96	84	127	102	97	118	84	96	84	98	101	112	77
97	106	141	54	102	80	69	0	117	108	75	107	96	92	106	94	92	96	134	130	104	105	126	93	93	96	101	125	95	81	109	142	103	107	104	99	102	98	99	90	103	131	100	102	114	100	94	95	96	97	114	92
125	112	131	121	114	97	113	117	0	126	126	99	113	108	110	95	126	113	117	103	95	114	111	107	119	111	116	102	121	122	104	134	121	115	114	101	115	124	112	120	117	113	106	119	116	105	126	109	97	113	108	118
95	72	141	110	98	112	97	108	126	0	96	96	108	106	93	100	105	108	128	127	111	94	133	100	99	94	81	127	96	119	92	143	99	113	106	107	109	93	91	106	94	131	107	99	120	101	108	90	100	101	121	109
100	106	148	58	116	85	57	75	126	96	0	99	88	110	98	93	91	88	134	130	124	91	138	100	111	101	90	127	79	84	109	150	106	109	114	101	111	85	90	82	86	136	113	88	126	101	100	83	104	95	120	64
114	90	135	116	74	95	96	107	99	96	99	0	85	104	67	82	114	85	127	125	80	92	132	94	96	90	110	124	91	98	71	145	95	90	97	97	86	108	87	90	87	124	81	89	120	94	95	90	76	87	113	97
106	94	133	95	93	96	103	96	113	108	88	85	0	96	87	96	70	0	120	122	91	92	131	86	98	79	109	118	56	75	97	136	98	95	96	107	82	100	82	104	93	113	94	82	121	107	72	84	75	80	99	88
111	102	132	99	95	91	98	92	108	106	110	104	96	0	104	78	106	96	125	122	105	95	102	95	77	97	107	114	108	89	97	145	94	86	88	95	90	107	86	108	106	119	81	87	137	71	97	92	93	93	105	94
102	94	133	115	89	101	91	106	110	93	98	67	87	104	0	100	113	87	130	120	83	105	133	92	96	94	98	121	77	109	62	144	93	97	108	80	98	100	94	93	77	129	87	98	117	109	100	88	92	97	111	108
119	108	139	94	85	52	92	94	95	100	93	82	96	78	100	0	91	96	124	123	96	101	122	99	102	104	104	117	95	89	97	142	97	105	94	97	90	111	99	96	96	119	73	94	117	76	109	95	87	95	100	86
101	108	135	88	97	93	105	92	126	105	91	114	70	106	113	91	0	70	120	127	118	99	132	94	90	87	112	124	76	85	112	140	95	96	94	113	99	107	106	94	98	121	84	88	116	105	87	94	98	106	104	80
106	94	133	95	93	96	103	96	113	108	88	85	0	96	87	96	70	0	120	122	91	92	131	86	98	79	109	118	56	75	97	136	98	95	96	107	82	100	82	104	93	113	94	82	121	107	72	84	75	80	99	88
111	123	124	130	116	127	139	134	117	128	134	127	120	125	130	124	120	120	0	115	102	117	105	117	126	130	124	118	131	118	122	148	128	121	122	132	114	124	137	127	134	126	118	114	112	130	120	125	126	128	118	125
137	124	119	128	126	121	136	130	103	127	130	125	122	122	120	123	127	122	115	0	99	142	115	125	139	134	132	81	128	133	121	129	143	131	137	124	122	137	130	134	128	112	129	124	110	130	130	134	129	127	127	132
109	88	133	117	89	97	121	104	95	111	124	80	91	105	83	96	118	91	102	99	0	113	118	98	91	102	117	107	108	110	83	142	92	78	89	99	85	114	109	110	101	125	91	83	101	102	100	104	96	97	110	120
96	93	145	98	96	108	99	105	114	94	91	92	92	95	105	101	99	92	117	142	113	0	133	80	92	75	83	141	76	95	102	147	92	98	99	104	96	68	71	98	105	129	96	83	128	89	95	56	65	65	113	82
143	129	124	132	127	117	131	126	111	133	138	132	131	102	133	122	132	131	105	115	118	133	0	120	123	128	130	91	130	127	120	142	126	122	126	128	115	141	130	139	124	113	117	127	120	119	125	136	126	138	117	132
99	99	130	103	101	103	98	93	107	100	100	94	86	95	92	99	94	86	117	125	98	80	120	0	84	66	104	113	81	104	63	139	99	79	93	90	93	104	75	114	103	121	84	90	114	93	88	88	74	80	109	101
96	95	134	99	84	101	102	93	119	99	111	96	98	77	96	102	90	98	126	139	91	92	123	84	0	87	116	118	87	96	95	147	78	50	50	101	87	112	81	99	83	128	84	94	106	69	87	92	100	95	95	95
93	77	136	109	95	105	94	96	111	94	101	90	79	97	94	104	87	79	130	134	102	75	128	66	87	0	86	122	62	95	83	142	77	95	84	98	92	94	53	102	92	125	90	85	116	85	84	63	74	84	101	96
92	86	149	106	118	111	96	101	116	81	90	110	109	107	98	104	112	109	124	132	117	83	130	104	116	86	0	137	98	122	99	147	110	124	115	96	123	78	91	111	108	130	111	115	119	102	120	71	92	90	112	102
129	126	121	123	117	114	127	125	102	127	127	124	118	114	121	117	124	118	118	81	107	141	91	113	118	122	137	0	122	119	121	141	124	110	117	116	116	134	130	128	128	111	115	117	111	116	118	132	120	136	120	127
95	87	140	100	105	94	86	95	121	96	79	91	56	108	77	95	76	56	131	128	108	76	130	81	87	62	98	122	0	90	103	141	85	92	94	99	93	83	70	111	87	126	98	84	114	86	78	71	86	77	101	81
101	115	140	83	86	84	81	81	122	119	84	98	75	89	109	89	85	75	118	133	110	95	127	104	96	95	122	119	90	0	114	148	80	98	94	115	80	108	97	80	80	121	87	82	118	92	75	97	93	103	98	40
109	97	136	121	90	103	99	109	104	92	109	71	97	97	62	97	112	97	122	121	83	102	120	63	95	83	99	121	103	114	0	149	106	88	99	70	86	107	90	97	86	119	73	90	118	107	104	104	86	102	115	111
145	140	90	145	146	144	149	142	134	143	150	145	136	145	144	142	140	136	148	129	142	147	142	139	147	142	147	141	141	148	149	0	146	148	148	147	147	148	144	149	148	142	148	144	133	148	144	147	140	136	146	148
90	89	140	103	73	94	94	103	121	99	106	95	98	94	93	97	95	98	128	143	92	92	126	99	78	77	110	124	85	80	106	146	0	78	72	103	87	96	88	100	86	133	76	81	108	76	90	80	90	90	90	81
107	107	129	105	78	110	105	107	115	113	109	90	95	86	97	105	96	95	121	131	78	98	122	79	50	95	124	110	92	98	88	148	78	0	61	104	82	109	97	103	90	122	92	84	114	79	93	103	98	99	96	95
101	103	138	106	82	101	107	104	114	106	114	97	96	88	108	94	94	96	122	137	89	99	126	93	50	84	115	117	94	94	99	148	72	61	0	113	76	108	90	107	85	130	88	92	105	73	87	97	94	97	89	96

100	99	136	107	106	93	91	99	101	107	101	97	107	95	80	97	113	107	132	124	99	104	128	90	101	98	96	116	99	115	70	147	103	104	113	0	111	97	102	105	102	122	65	105	118	99	106	98	96	110	117	107
124	113	136	104	82	94	105	102	115	109	111	86	82	90	98	90	99	82	114	122	85	96	115	93	87	92	123	116	93	80	86	147	87	82	76	111	0	119	94	98	103	117	80	71	126	90	63	108	94	91	106	91
62	90	146	103	116	115	89	98	124	93	85	108	100	107	100	111	107	100	124	137	114	68	141	104	112	94	78	134	83	108	107	148	96	109	108	97	119	0	88	110	111	134	111	99	125	96	108	71	97	92	112	101
107	83	137	103	102	103	92	99	112	91	90	87	82	86	94	99	106	82	137	130	109	71	130	75	81	53	91	130	70	97	90	144	88	97	90	102	94	88	0	108	92	127	98	85	123	82	83	77	76	75	108	90
107	115	132	90	86	103	96	90	120	106	82	90	104	108	93	96	94	104	127	134	110	98	139	114	99	102	111	128	111	80	97	149	100	103	107	105	98	110	108	0	84	126	96	79	128	110	98	99	109	117	128	76
111	97	148	102	95	99	84	103	117	94	86	87	93	106	77	96	98	93	134	128	101	105	124	103	83	92	108	128	87	80	86	148	86	90	85	102	103	111	92	84	0	134	99	100	114	90	101	95	94	109	101	70
130	127	128	124	115	128	127	131	113	131	136	124	113	119	129	119	121	113	126	112	125	129	113	121	128	125	130	111	126	121	119	142	133	122	130	122	117	134	127	126	134	0	113	128	120	125	125	132	114	125	105	124
108	94	134	100	75	81	102	100	106	107	113	81	94	81	87	73	84	94	118	129	91	96	117	84	84	90	111	115	98	87	73	148	76	92	88	65	80	111	98	96	99	113	0	79	113	92	82	99	79	101	104	96
110	102	130	104	72	93	97	102	119	99	88	89	82	87	98	94	88	82	114	124	83	83	127	90	94	85	115	117	84	82	90	144	81	84	92	105	71	99	85	79	100	128	79	0	118	102	79	101	95	93	115	84
106	107	119	127	113	116	118	114	116	120	126	120	121	137	117	117	116	121	112	110	101	128	120	114	106	116	119	111	114	118	118	133	108	114	105	118	126	125	123	128	114	120	113	118	0	115	120	119	115	116	105	125
93	96	142	100	93	98	84	100	105	101	101	94	107	71	109	76	105	107	130	130	102	89	119	93	69	85	102	116	86	92	107	148	76	79	73	99	90	96	82	110	90	125	92	102	115	0	87	88	97	95	90	82
108	110	132	106	99	115	96	94	126	108	100	95	72	97	100	109	87	72	120	130	100	95	125	88	87	84	120	118	78	75	104	144	90	93	87	106	63	108	83	98	101	125	82	79	120	87	0	100	93	97	109	91
86	91	145	95	98	98	84	95	109	90	83	90	84	92	88	95	94	84	125	134	104	56	136	88	92	63	71	132	71	97	104	147	80	103	97	98	108	71	77	99	95	132	99	101	119	88	100	0	68	59	108	83
106	93	138	95	89	95	98	96	97	100	104	76	75	93	92	87	98	75	126	129	96	65	126	74	100	74	92	120	86	93	86	140	90	98	94	96	94	97	76	109	94	114	79	95	115	97	93	68	0	63	99	85
109	91	138	101	104	104	101	97	113	101	95	87	80	93	97	95	106	80	128	127	97	65	138	80	95	84	90	136	77	103	102	136	90	99	97	110	91	92	75	117	109	125	101	93	116	95	97	59	63	0	106	93
112	109	137	118	96	113	112	114	108	121	120	113	99	105	111	100	104	99	118	127	110	113	117	109	95	101	112	120	101	98	115	146	90	96	89	117	106	112	108	128	101	105	104	115	105	90	109	108	99	106	0	104
106	110	146	78	97	84	77	92	118	109	64	97	88	94	108	86	80	88	125	132	120	82	132	101	95	96	102	127	81	40	111	148	81	95	96	107	91	101	90	76	70	124	96	84	125	82	91	83	85	93	104	0
92	73	144	107	82	102	84	97	117	83	92	87	103	97	76	107	112	103	121	128	85	86	129	87	77	88	94	125	89	107	72	142	86	82	82	94	93	92	86	105	82	142	92	77	110	88	92	94	93	88	109	105
150	141	115	144	150	146	142	143	126	139	142	140	144	133	141	141	146	144	138	108	143	148	112	136	147	141	131	113	143	149	138	135	150	140	146	136	142	143	140	141	147	126	143	144	131	139	134	141	137	131	142	140
102	94	138	108	98	94	107	113	112	98	113	96	91	92	103	84	111	91	128	128	85	98	125	104	90	94	109	123	94	107	96	142	76	85	85	94	107	98	87	122	98	120	82	106	112	89	107	91	84	85	89	103
110	93	134	109	72	91	87	102	114	94	87	61	92	95	69	86	106	92	129	134	96	85	133	86	85	92	109	134	90	95	72	144	81	89	97	100	84	104	79	78	77	136	88	64	109	106	98	88	83	87	117	93
105	85	138	114	100	104	103	100	112	97	107	94	99	91	97	103	108	99	117	133	98	77	131	85	86	83	92	132	100	115	82	143	98	88	74	105	101	86	91	107	93	131	102	103	121	95	110	89	83	82	102	110
92	82	140	103	108	102	93	95	117	81	85	97	101	107	87	104	103	101	133	135	106	90	140	84	102	90	90	135	86	109	97	138	89	111	116	107	113	86	82	114	96	136	103	99	119	106	107	80	91	78	120	107
95	82	143	106	96	96	87	99	110	99	107	93	84	92	92	104	99	84	134	130	100	93	126	96	97	54	93	125	81	96	93	147	66	100	96	94	96	92	64	109	87	127	88	97	120	95	94	73	83	87	104	101
98	110	131	97	99	98	103	104	117	112	88	92	84	103	80	102	95	84	121	127	99	85	133	74	96	67	97	122	72	100	80	145	94	89	98	80	94	94	89	94	98	121	92	87	133	102	94	84	91	99	108	89
101	116	143	113	85	94	94	108	128	108	92	102	92	104	95	92	103	92	124	140	100	101	137	101	86	100	117	128	84	86	97	152	81	81	83	110	86	106	101	96	91	137	95	84	109	90	96	101	109	100	113	97
107	90	128	121	98	97	107	105	109	94	117	91	95	84	93	92	106	95	107	125	93	93	124	89	91	88	108	119	103	104	77	149	96	96	79	94	90	100	91	108	106	118	77	94	124	101	97	94	93	102	105	111
78	88	139	101	103	105	91	100	122	88	95	101	96	96	87	110	105	96	123	136	107	77	131	85	88	81	91	132	76	104	92	141	82	97	107	100	102	80	70	100	106	129	101	85	109	85	92	83	96	84	112	101
101	105	130	103	73	104	101	101	111	111	114	82	98	84	96	90	98	98	128	124	76	107	123	99	78	90	120	108	100	90	97	148	68	73	79	88	78	113	96	98	104	116	68	80	109	82	71	94	84	98	103	97
107	84	134	105	94	110	103	99	115	90	93	77	68	102	83	108	92	68	127	121	91	91	133	70	93	67	104	124	82	96	66	138	102	78	97	98	86	103	80	90	85	125	99	77	119	110	94	91	75	88	120	94
90	99	141	106	96	115	103	111	120	94	103	97	98	104	93	115	91	98	114	132	107	65	137	73	83	78	104	132	85	99	72	148	90	82	83	94	97	91	95	96	99	127	91	99	121	101	100	72	95	96	107	91
96	93	131	113	101	106	91	107	121	96	97	100	82	100	86	103	101	82	118	130	105	79	132	87	101	76	101	124	71	90	97	140	92	104	109	108	89	85	75	93	103	126	103	78	118	95	75	83	94	84	119	98
98	106	141	108	93	83	92	95	113	107	93	89	95	93	94	80	88	95	123	132	92	92	128	91	74	91	112	128	96	88	89	150	75	67	68	94	86	104	93	89	77	138	81	90	117	91	90	86	89	94	106	82
95	81	137	116	104	110	98	108	110	92	102	89	100	99	78	112	107	100	118	131	95	91	133	72	100	87	93	126	99	121	58	144	101	94	108	89	107	94	90	104	106	130	89	95	123	107	110	95	93	91	125	114
109	102	126	109	89	107	107	104	112	106	121	94	102	95	93	106	109	102	118	115	88	111	122	102	94	98	119	111	109	104	100	148	76	95	95	89	83	122	99	94	111	126	82	90	110	98	91	108	90	109	122	102
108	113	139	95	92	74	98	94	106	110	103	94	91	70	95	67	104	91	119	119	107	102	111	81	95	102	111	116	94	91	85	149	101	105	95	89	87	111	108	101	105	111	78	96	119	93	108	91	89	100	103	92
98	112	135	90	88	87	96	86	108	109	104	90	94	101	77	86	98	94	120	112	70	108	118	97	95	100	113	106	98	85	89	143	89	105	107	91	76	118	106	90	97	115	71	88	111	99	82	100	89	101	113	99
98	100	136	91	66	99	86	85	123	100	89	83	91	99	104	90	90	91	135	135	105	86	123	87	79	83	110	124	86	80	103	144	73	79	88	112	96	104	93	91	92	115	90	87	117	67	85	88	89	93	99	75
106	104	139	99	111	104	87	102	111	112	92	106	110	121	112	102	103	110	127	131	121	98	128	95	99	89	109	121	102	100	100	147	110	102	102	123	101	114	97	90	86	122	105	98	110	107	100	101	100	105	116	99
98	94	140	112	99	93	88	94	117	105	84	90	95	94	90	86	97	95	127	138	105	79	134	74	90	81	98	130	80	104	79	148	87	90	99	72	103	85	87	106	100	139	79	87	119	96	101	79	87	85	113	98
86	107	148	95	108	94	92	98	126	103	87	105	108	108	96	98	87	108	132	139	115	91	139	96	93	91	96	133	87	95	107	154	92	98	100	99	114	98	99	99	93	131	95	107	113	91	110	78	99	97	112	85
96	100	131	99	98	95	87	87	100	115	104	95	99	93	98	95	100	99	126	139	114	85	120	66	82	73	97	122	91	89	96	140	89	96	99	101	105	106	85	89	90	123	82	101	110	88	93	84	84	92	103	91
95	95	133	95	104	102	94	90	108	106	104	90	89	99	97	93	94	89	133	129	101	77	133	64	91	66	92	132	81	96	91	141	96	94	95	101	95	100	78	97	106	116	91	95	108	91	93	67	76	66	112	87
98	110	140	99	113	101	100	100	111	105	96	98	85	101	98	101	79	85	116	134	116	72	138	77	89	95	98	132	91	93	86	145	112	91	91	96	98	82	93	98	103	133	93	106	125	104	97	75	93	90	124	89
94	103	143	95	101	95	81	86	120	103	95	104	102	102	106	91	91	102	125	141	108	99	125	99	83	84	110	127	93	74	96	147	74	98	76	106	83	101	100	96	80	128	79	92	100	71	75	93	93	96	102	84
99	82	142	106	87	95	93	95	119	85	101	78	93	90	95	86	98	93	129	132	86	93	129	97	64	73	100	128	81	101	88	148	67	75	72	101	82	104	70	95	80	132	94	91	109	74	84	82	84	80	107	91
91	94	148	113	89	103	81	104	116	88	96	86	109	104	89	92	90	109	125	133	105	96	129	102	81	90	102	126	94	97	85	149	75	88	84	92	96	90	101	89	73	131	81	91	113	86	91	98	110	111	101	93
103	106	139	78	81	83	85	73	115	110	93	95	79	85	91	84	81	79	130	130	88	97	121	93	73	86	114	123	87	73	96	144	74	78	87	93	82	110	91	97	91	122	73	93	112	94	87	89	79	86	99	85
122	127	123	115	110	103	105	113	115	131	118	117	117	89	113	96	116	117	118	119	108	130	104	111	107	121	128	107	124	106	116	146	114	114	109	98	90	134	122	108	116	123	95	104	120	109	106	121	114	122	119	104
113	104	123	97	81	99	97	99	103	108	119	92	97	82	104	87	97	97	104	121	95	106	109	94	96	97	122	104	106	91	92	151	96	96	96	92	81	116	109	107	110	101	71	94	117	95	88	100	90	105	100	102
105	113	137	110	80	97	106	108	125	125	109	96	93	86	103	93	103	93	123	136	79	94	120	96	85	96	120	126	95	80	96	150	60	69	86	95	84	106	99	102	95	127	78	74	120	96	90	100	94	98	96	90
112	108	130	110	77	100	106	112	104	114	109	66	87	103	75	98	110	87	115	119	93	98	125	88	99	100	123	114	93	93	81	142	85	86	88	99	76	115	102	93	99	117	70	93	117	111	82	100	88	92	97	106
81	87	146	91	111	100	79	80	116	95	83	84	96	94	94	100	102	96	138	140	111	86	133	88	82	81	96	131	80	88	90	141	86	96	85	89	99	83	85	92	95	128	92	87	113	86	87	83	87	85	108	84
133	130	123	126	119	120	116	126	109	128	120	110	109	116	108	106	119	109	128	122	118	125	120	110	119	116	135	120	118	118	108	143	117	117	122	119	102	124	115	104	123	106	111	111	130	118	106	127	113	115	100	121
124	112	135	115	106	109	117	118	115	117	117	101	106	114	113	101	121	106	118	122	106	119	125	112	110	115	128	114	112	101	119	150	106	116	115	124	103	128	113	101	106	113	111	114	131	107	108	121	110	115	101	108
126	121	127	111	115	105	104	109	108	125	111	115	114	100	109	94	111	114	121	123	120	124	105	113	107	119	128	103	112	109	110	143	111	107	110	109	104	115	120	107	116	101	101	103	128	103	114	124	111	117	86	110
128	102	133	107	94	76	101	101	118	108	89	96	90	104	92	77	105	90	117	108	88	111	118	110	115	105	114	115	88	96	98	149	111	104	113	107	92	118	103	104	92	131	97	93	116	112	108	110	109	106	108	100
67	92	141	72	104	89	79	85	119	88	75	96	89	107	101	95	98	89	125	126	106	94	133	96	94	83	84	119	79	97	104	143	93	107	104	95	109	87	90	92	103	123	103	105	116	71	97	70	95	95	110	86
103	103	134	111	93	92	96	99	102	104	111	93	104	110	91	97	107	104	118	109	57	121	125	106	99	100	113	105	107	104	91	146	68	99	102	88	87	118	113	95	101	125	83	89	112	97	91	103	100	111	112	109
114	96	119	118	92	108	114	112	97	111	123	86	97	88	81	104	116	97	118	107	62	109	112	100	93	104	116	100	107	113	96	136	92	93	104	83	101	114	103	107	105	123	94	99	107	99	105	99	92	103	111	117
120	107	125	111	100	85	118	116	93	109	120	97	109	111	113	88	117	109	116	109	89	117	125	124	117	112	119	105	109	116	113	140	108	118	109	94	97	121	105	114	120	122	95	98	114	103	114	114	98	114	126	114
111	99	128	104	111	99	111	106	98	108	120	112	104	111	114	108	124	104	111	101	71	117	118	118	105	109	120	93	111	110	110	135	105	113	108	112	104	110	106	121	114	118	104	105	106	96	111	109	104	106	111	119
110	104	135	104	110	92	92	99	118	98	86	110	91	117	105	96	103	91	122	121	109	113	131	111	114	107	118	113	83	100	110	146	109	112	113	113	105	108	100	116	91	136	110	101	113	101	102	110	108	107	115	110
134	124	114	129	128	129	141	135	117	132	136	136	123	133	134	132	126	123	125	95	126	139	105	121	134	128	132	69	130	134	134	129	135	124	135	124	129	140	131	138	140	111	134	130	101	136	129	138	125	128	128	136
112	109	126	98	93	92	112	98	100	108	114	93	96	99	94	96	93	96	120	91	80	114	106	95	90	100	129	83	100	96	89	143	110	105	100	106	76	124	107	93	107	108	84	91	116	97	85	117	104	119	111	107
109	97	137	104	99	103	99	96	92	100	103	87	85	96	98	86	85	85	120	120	105	66	125	68	86	74	87	125	81	99	85	141	103	91	86	97	89	89	80	104	95	118	92	99	117	96	92	67	52	71	101	90
109	110	135	113	92	99	103	104	113	113	104	83	93	91	86	88	106	93	106	124	77	106	122	104	90	107	118	115	101	90	88	145	83	79	88	97	70	100	103	103	89	133	85	78	119	92	74	109	99	104	108	100
90	93	145	94	94	103	92	101	113	98	98	97	93	99	100	96	99	93	128	135	109	64	130	86	94	65	75	123	75	99	101	150	85	100	104	97	106	83	89	95	102	125	92	103	122	83	105	30	74	72	108	87
96	101	144	95	97	97	94	101	109	98	94	87	82	98	88	90	104	82	117	128	100	58	126	73	105	83	85	126	85	94	82	142	92	106	103	110	92	87	81	110	101	125	90	104	114	98	96	51	56	54	109	88
94	88	139	114	97	104	93	103	117	96	100	96	107	85	92	106	109	107	123	141	90	89	126	89	51	87	107	124	85	97	93	148	73	55	61	98	92	95	83	108	88	138	86	77	98	71	88	94	101	91	97	96
100	98	142	106	84	95	95	105	120	97	107	92	98	75	103	84	93	98	130	128	104	84	109	94	72	81	100	113	88	88	96	147	70	91	69	100	70	97	88	109	93	121	75	86	114	66	80	85	80	85	96	85
86	103	138	87	91	90	83	86	121	106	94	101	81	79	92	92	88	81	126	138	108	85	131	90	80	86	113	128	76	67	92	148	75	87	87	96	72	93	84	96	84	126	75	83	117	76	81	89	85	86	97	86
111	98	134	101	89	89	93	92	110	103	82	82	82	91	84	91	90	82	125	122	93	82	124	88	99	79	95	127	80	90	70	144	89	101	108	87	80	91	69	89	85	123	78	49	122	108	83	86	80	85	115	83
114	111	129	92	89	85	97	92	110	111	112	96	83	89	90	87	87	83	115	120	93	107	121	96	84	93	124	119	98	83	91	144	86	88	91	106	58	122	100	91	92	117	84	89	124	103	81	107	87	102	105	94
130	127	118	132	115	122	138	128	94	136	143	115	123	114	128	114	136	123	101	98	104	130	117	120	124	124	129	100	137	120	121	141	134	118	118	115	118	135	128	127	132	109	119	127	110	110	130	132	116	124	121	126

74	101	137	95	99	112	77	105	123	91	83	112	100	106	105	107	101	100	116	136	112	101	131	86	96	87	100	122	97	90	100	150	88	103	97	105	105	76	99	94	88	126	99	94	113	96	90	83	108	105	112	92
94	91	137	88	101	85	68	87	117	99	71	106	64	95	98	90	62	64	134	131	122	97	136	99	99	88	99	122	76	77	109	143	92	108	105	100	108	91	86	93	86	125	104	82	123	96	100	87	102	105	109	77
137	127	101	137	125	127	138	130	110	135	141	131	128	113	136	129	127	128	117	109	126	140	113	118	126	125	136	101	131	123	131	118	134	126	126	130	129	142	131	136	139	122	124	122	120	123	133	141	126	133	122	134
133	126	104	133	125	124	138	135	101	135	142	125	132	127	132	127	128	132	116	92	115	148	122	124	126	129	140	88	136	132	129	124	134	125	126	125	127	142	136	132	135	115	122	130	106	128	133	141	130	133	126	134
90	84	135	98	85	99	80	99	113	84	89	75	95	103	81	96	105	95	119	131	86	91	119	88	76	84	107	119	86	93	82	146	85	83	90	105	93	99	94	93	80	126	90	87	104	90	93	86	98	87	107	96
113	101	141	114	91	98	104	107	118	109	117	95	95	95	105	92	106	95	119	136	90	90	121	103	77	93	121	128	93	90	97	150	81	80	79	114	55	100	93	99	100	130	89	91	119	82	82	106	101	85	107	101
104	105	133	99	103	107	107	97	125	108	116	114	104	97	117	103	108	104	139	141	113	94	126	111	91	93	109	127	100	99	113	138	88	104	98	115	101	108	98	100	107	129	102	110	121	86	103	95	97	106	103	101
104	98	135	104	90	99	104	107	108	107	112	82	89	90	100	89	103	89	126	129	90	96	115	99	100	85	117	121	92	93	99	144	92	106	103	108	91	110	99	101	102	120	85	87	128	90	94	93	91	99	109	96
109	109	141	92	82	96	87	82	116	105	93	94	97	97	97	97	87	97	136	136	112	98	128	102	72	85	118	123	77	67	115	147	76	88	86	101	97	112	86	90	72	121	91	94	115	80	86	95	91	97	97	67
122	119	121	132	112	117	125	131	96	127	132	111	127	115	116	112	128	127	102	104	105	122	106	121	114	119	124	96	130	126	116	139	120	103	108	125	117	122	128	118	114	104	120	114	104	100	136	124	115	124	101	120
95	87	138	95	67	96	94	101	124	81	102	82	90	86	92	97	96	90	130	142	93	84	123	88	55	76	114	123	80	86	89	147	59	61	76	94	82	102	81	93	81	123	78	82	113	73	85	87	86	92	98	87
101	103	133	94	97	92	78	102	120	100	81	85	93	103	79	94	89	93	117	127	102	102	132	102	100	104	119	121	82	84	89	152	95	93	96	101	79	100	101	80	85	130	86	70	119	110	83	103	100	104	124	86
84	101	140	89	93	113	95	92	126	96	90	104	88	96	94	107	84	88	129	140	111	74	142	95	77	90	101	143	75	88	104	145	80	85	103	108	93	86	78	91	106	128	95	90	119	99	83	76	93	84	114	87

1	1	1	1	1	1	1	1	1	1	1	1	1	1	1	1	1	1	1	1	1	1	1	1	1	1	1	1	1	1	1	1	1	1	1	1	1	1	1	1	1	1	1	1	1	1	1	1	1	1	1	1
Strain 15	Strain 16	Strain 17	Strain 18	Strain 19	Strain 20	Strain 21	Strain 22	Strain 23	Strain 24	Strain 25	Strain 26	Strain 27	Strain 28	Strain 29	Strain 30	Strain 31	Strain 32	Strain 33	Strain 34	Strain 35	Strain 36	Strain 37	Strain 38	Strain 39	Strain 40	Strain 41	Strain 42	Strain 43	Strain 44	Strain 45	Strain 46	Strain 47	Strain 48	Strain 49	Strain 50	Strain 51	Strain 52	Strain 53	Strain 54	Strain 55	Strain 56	Strain 57	Strain 58	Strain 59	Strain 60	Strain 61	Strain 62	Strain 63	Strain 64	Strain 65	Strain 66
C015	C016	C017	C018	C019	C020	C021	C022	C023	C024	C025	C026	C027	C028	C029	C030	C031	C032	C033	C034	C035	C036	C037	C038	C039	C040	C041	C042	C043	C044	C045	C046	C047	C048	C049	C050	C051	C052	C053	C054	C055	C056	C057	C058	C059	C060	C061	C062	C063	C064	C065	C066
0	0	0	0	0	0	0	0	0	0	0	0	0	0	0	0	0	0	0	0	0	2	0	1	0	0	0	0	0	0	0	0	0	0	1	2	0	0	0	0	0	0	0	0	0	0	0	0	0	0	0	0
126	135	119	123	124	131	121	112	118	112	124	98	125	128	129	119	129	97	110	109	116	127	122	128	121	121	122	115	117	122	120	101	96	112	111	129	117	109	101	106	117	94	94	81	94	115	110	102	113	104	122	127
139	83	131	139	139	144	137	145	138	137	139	133	136	133	135	135	134	131	138	134	133	141	139	140	132	126	147	141	137	140	134	123	127	126	135	145	137	137	132	136	142	141	124	132	131	138	86	132	140	143	138	144
128	132	123	133	132	134	116	123	128	115	134	114	122	124	128	126	134	127	123	112	123	113	133	135	124	133	128	123	127	124	124	123	108	113	118	132	128	118	109	107	122	116	116	104	92	115	103	96	127	118	126	126
141	72	137	143	144	140	132	144	145	136	139	131	134	138	137	139	137	132	130	138	135	139	145	144	132	127	140	142	140	145	139	125	135	133	137	141	131	138	136	135	137	142	127	133	129	142	90	129	138	143	138	140
98	147	112	107	112	98	105	106	98	105	99	109	102	98	104	104	103	114	111	116	112	113	99	98	119	96	99	112	107	101	116	123	119	106	121	101	127	126	129	111	88	112	121	123	118	108	136	116	110	104	97	105
130	127	134	143	134	139	131	135	138	140	140	133	133	137	133	139	138	138	137	142	129	127	133	142	124	136	142	131	132	135	135	128	128	134	140	127	131	135	123	144	123	141	136	138	131	140	132	136	135	136	129	138
102	145	89	82	93	116	105	96	99	83	114	70	101	103	118	82	104	80	91	85	97	104	105	105	110	104	111	98	78	99	81	107	84	88	70	100	102	104	90	95	108	87	78	92	85	112	125	87	86	87	108	97
122	145	116	122	112	110	115	124	121	127	110	114	106	123	119	120	128	119	122	118	117	109	127	127	110	108	129	113	111	120	107	127	115	122	118	111	123	121	123	126	114	120	123	121	114	116	125	122	116	119	114	117
107	139	125	98	103	91	98	88	109	115	92	104	92	98	93	96	96	106	112	94	92	85	94	82	86	83	90	96	100	92	88	116	109	112	111	83	122	118	116	118	75	104	117	133	131	113	128	116	88	113	87	105
107	139	125	98	103	91	98	88	109	115	92	104	92	98	93	96	96	106	112	94	92	85	94	82	86	83	90	96	100	92	88	116	109	112	111	83	122	118	116	118	75	104	117	133	131	113	128	116	88	113	87	105
107	139	125	98	103	91	98	88	109	115	92	104	92	98	93	96	96	106	112	94	92	85	94	82	86	83	90	96	100	92	88	116	109	112	111	83	122	118	116	118	75	104	117	133	131	113	128	116	88	113	87	105
107	139	125	98	103	91	98	88	109	115	92	104	92	98	93	96	96	106	112	94	92	85	94	82	86	83	90	96	100	92	88	116	109	112	111	83	122	118	116	118	75	104	117	133	131	113	128	116	88	113	87	105
91	144	107	99	109	95	98	106	100	108	93	122	108	94	95	114	94	118	107	119	118	113	92	97	107	105	102	110	102	114	115	122	116	117	121	99	115	125	120	108	88	122	131	127	124	106	141	135	109	118	92	91
106	141	126	81	96	74	98	89	114	102	89	106	92	97	94	101	86	108	98	94	97	94	86	102	93	96	87	104	108	96	99	117	100	107	102	89	112	109	110	101	97	100	109	114	120	106	135	109	86	93	88	87
124	134	131	127	129	138	128	121	127	132	127	117	125	127	128	125	132	119	112	117	120	122	132	139	116	123	125	121	133	131	122	118	106	122	116	127	117	107	112	123	119	116	124	115	117	122	117	110	124	112	125	128
91	149	100	85	105	73	100	91	82	114	73	108	101	101	90	96	100	115	110	113	96	104	78	84	100	103	103	99	89	92	93	113	119	96	117	90	121	130	118	106	77	118	123	114	126	82	143	130	103	106	97	93
91	149	100	85	105	73	100	91	82	114	73	108	101	101	90	96	100	115	110	113	96	104	78	84	100	103	103	99	89	92	93	113	119	96	117	90	121	130	118	106	77	118	123	114	126	82	143	130	103	106	97	93
146	62	144	149	146	150	142	149	151	143	147	143	141	145	141	149	137	138	141	148	141	148	146	148	139	136	148	154	148	151	151	135	147	144	144	147	134	146	142	145	147	147	128	138	133	150	96	135	144	151	142	147
98	146	112	114	114	118	116	113	108	119	100	123	110	105	109	123	112	124	125	130	118	129	115	112	127	118	110	119	117	113	130	138	131	115	123	108	130	127	119	123	111	114	131	121	126	114	142	136	123	112	111	115
115	139	133	122	113	119	124	132	122	120	112	135	121	123	120	128	110	134	129	134	126	123	119	133	118	126	121	123	126	120	126	141	131	127	129	123	122	128	124	134	128	135	134	135	127	121	135	129	122	124	135	129
115	131	116	109	114	116	112	100	109	102	114	97	104	109	114	108	116	101	94	88	111	121	107	121	109	104	119	115	117	115	103	110	87	96	90	106	113	105	104	88	113	105	105	110	102	104	114	94	108	101	110	110
94	142	104	100	102	79	102	102	99	105	93	124	98	92	101	107	95	122	113	120	117	111	91	87	102	105	85	114	107	108	113	129	116	114	121	99	125	131	130	109	90	123	123	128	119	99	140	130	106	118	94	99
110	148	121	112	118	114	116	129	118	124	112	127	110	126	116	121	120	128	123	125	120	117	116	128	130	119	118	115	114	118	119	133	128	132	126	119	112	116	119	125	122	122	131	117	116	109	141	128	119	118	123	124
130	121	126	129	130	132	119	122	131	120	126	119	125	129	116	132	129	116	108	107	121	126	130	134	123	113	123	133	135	129	123	118	103	129	107	137	119	121	116	107	118	108	112	97	103	117	93	92	122	121	123	130
130	121	126	129	130	132	119	122	131	120	126	119	125	129	116	132	129	116	108	107	121	126	130	134	123	113	123	133	135	129	123	118	103	129	107	137	119	121	116	107	118	108	112	97	103	117	93	92	122	121	123	130
130	121	126	129	130	132	119	122	131	120	126	119	125	129	116	132	129	116	108	107	121	126	130	134	123	113	123	133	135	129	123	118	103	129	107	137	119	121	116	107	118	108	112	97	103	117	93	92	122	121	123	130
128	148	138	134	128	131	128	139	133	123	126	142	127	133	132	133	121	140	141	138	132	138	135	134	141	134	128	136	132	132	130	147	142	139	145	134	131	140	136	136	132	132	140	138	132	135	143	136	132	140	137	134
126	133	122	132	132	133	126	126	127	113	132	107	126	130	128	121	127	111	114	97	125	127	130	127	122	126	129	130	128	129	120	103	100	126	116	137	120	113	106	99	118	105	72	93	70	119	83	73	123	119	122	126
126	144	126	124	126	121	125	120	130	125	120	128	129	121	123	126	127	129	119	121	122	130	127	123	122	115	131	126	131	126	119	127	128	121	121	120	127	128	123	129	125	123	125	131	125	136	140	128	127	127	117	118
109	142	127	118	108	120	124	123	122	118	113	125	120	122	122	127	117	124	134	132	126	127	125	127	129	120	133	131	122	116	132	142	130	125	121	123	128	123	131	127	132	120	121	127	120	129	129	129	128	124	126	135
96	145	110	96	108	64	99	100	107	113	95	111	105	109	93	107	105	122	116	114	104	106	95	98	105	101	102	96	86	99	104	120	126	104	119	93	115	125	121	102	93	112	123	119	122	103	142	123	102	109	97	95
142	54	136	144	139	142	134	147	144	139	138	135	144	145	140	140	138	134	133	140	140	140	144	142	133	130	148	147	138	149	143	129	142	130	139	139	133	141	140	139	140	145	131	127	126	142	82	140	141	143	138	140
132	116	122	131	129	135	127	127	127	125	126	115	128	124	130	126	134	129	116	120	124	125	127	133	117	119	132	118	126	128	122	116	110	114	123	126	130	119	111	128	116	124	121	121	113	125	101	120	122	127	127	128
116	136	99	111	109	126	120	109	109	94	118	95	114	112	119	100	115	103	99	96	103	117	110	113	110	111	113	117	107	108	102	112	101	98	101	114	117	110	101	105	113	95	95	97	88	119	113	98	102	98	114	111
121	129	117	127	123	140	133	129	124	114	129	113	131	119	124	127	118	128	125	114	118	128	131	131	133	123	134	126	118	124	131	122	113	123	116	136	123	120	122	116	123	115	112	115	103	124	105	102	128	119	132	134
125	140	113	121	117	132	123	115	124	110	127	117	129	128	122	119	123	120	111	118	119	120	133	128	116	120	129	120	122	124	115	123	104	119	105	128	94	79	91	110	121	110	115	117	118	123	131	102	115	108	123	116
125	140	113	121	117	132	123	115	124	110	127	117	129	128	122	119	123	120	111	118	119	120	133	128	116	120	129	120	122	124	115	123	104	119	105	128	94	79	91	110	121	110	115	117	118	123	131	102	115	108	123	116
125	140	113	121	117	132	123	115	124	110	127	117	129	128	122	119	123	120	111	118	119	120	133	128	116	120	129	120	122	124	115	123	104	119	105	128	94	79	91	110	121	110	115	117	118	123	131	102	115	108	123	116
125	141	113	121	117	132	123	115	124	110	127	117	129	128	122	119	123	120	111	118	120	120	133	128	116	121	129	120	122	124	115	123	104	119	105	128	95	80	91	110	121	110	115	117	118	123	132	102	115	108	123	116
125	140	113	121	117	132	123	115	124	110	127	117	129	128	122	119	123	120	111	118	119	120	133	128	116	120	129	120	122	124	115	123	104	119	105	128	94	79	91	110	121	110	115	117	118	123	131	102	115	108	123	116
134	135	144	144	140	134	132	144	137	135	135	142	138	133	134	135	134	144	138	140	140	132	134	144	137	140	131	142	141	135	142	141	133	142	139	137	135	140	130	142	134	137	142	142	139	140	130	142	135	139	137	141
139	127	136	137	133	139	136	140	140	130	132	136	142	140	138	133	138	134	137	134	138	132	140	141	133	138	138	134	141	137	135	131	129	137	134	138	131	137	129	138	135	138	133	131	134	134	135	130	133	129	142	139
129	137	119	124	124	132	113	109	119	108	119	98	120	116	121	119	124	99	104	107	110	128	116	113	114	111	110	126	116	121	118	99	82	117	104	129	126	115	107	104	109	108	103	69	112	112	117	104	109	112	108	115
128	137	117	123	122	132	112	108	118	106	118	97	118	114	119	117	123	99	103	106	109	128	114	113	114	110	109	125	114	119	116	99	81	116	103	128	126	115	107	103	109	107	102	68	111	111	117	104	107	111	107	115
124	145	122	133	123	127	127	127	130	125	127	124	126	129	128	128	126	131	133	129	125	136	128	129	124	130	127	134	132	129	121	135	125	127	123	131	129	138	128	135	135	130	129	138	135	134	136	130	130	119	134	127
128	125	133	120	123	129	133	126	129	124	126	121	121	134	125	129	129	122	115	126	127	122	133	137	131	118	131	126	130	133	129	129	125	133	121	117	127	127	118	127	131	129	125	119	124	128	130	130	121	122	133	133
118	139	128	125	120	127	124	125	124	120	120	128	126	119	121	124	118	127	120	128	122	123	121	127	119	116	118	119	125	122	121	126	127	125	123	115	127	138	120	139	119	129	130	133	126	136	137	130	118	118	118	116
129	146	116	119	123	126	115	109	132	114	124	125	120	129	121	130	118	123	121	123	123	133	133	116	125	121	126	130	124	129	122	136	123	124	115	122	119	108	114	118	115	116	120	120	120	129	138	133	126	123	117	111
129	114	121	131	133	139	135	143	127	127	131	118	134	126	136	124	129	115	126	119	124	134	130	132	130	134	141	125	126	127	121	97	113	123	125	139	133	135	121	123	137	125	108	117	110	123	93	111	128	129	129	131
124	132	113	114	121	113	112	108	130	117	119	108	104	120	115	119	112	103	122	104	117	129	126	122	117	113	116	123	114	120	105	119	109	121	112	126	116	109	117	113	114	103	98	116	106	124	117	110	110	109	108	107
136	108	122	140	130	141	135	130	134	131	128	120	132	127	124	133	134	132	123	125	121	133	134	140	121	119	135	126	135	132	128	123	119	119	123	135	122	125	123	127	128	123	127	118	112	129	101	120	125	128	130	126
109	146	122	105	110	110	119	115	115	108	117	105	101	122	111	118	99	105	127	113	124	119	117	124	130	115	119	117	99	115	114	128	119	115	115	111	111	111	114	111	124	111	106	111	100	115	135	114	112	113	124	118
140	139	135	140	130	137	138	128	136	133	126	134	132	132	133	135	135	132	133	135	133	136	140	133	124	137	126	134	140	139	124	136	123	134	133	136	119	125	117	129	132	133	136	130	128	133	134	129	125	128	133	126
97	136	112	89	85	81	95	101	120	91	91	107	88	107	98	107	76	106	105	102	108	114	105	110	106	90	108	115	97	107	107	121	100	116	100	102	116	107	108	104	104	100	99	111	106	110	122	107	99	111	106	95
123	122	130	131	138	130	130	134	124	122	128	115	133	124	124	121	123	112	112	108	131	122	127	133	119	129	133	125	129	122	123	99	104	119	116	132	122	119	116	117	121	114	106	110	102	124	106	98	130	118	127	126
126	135	120	124	124	131	122	114	119	113	124	99	127	128	130	120	130	99	112	111	117	127	123	128	122	122	123	115	118	122	122	102	98	113	113	129	119	111	103	108	118	96	96	82	95	115	111	104	114	106	123	128

83	153	89	99	105	92	105	100	103	102	96	111	100	93	100	108	86	114	115	117	115	119	92	98	115	98	102	108	96	113	119	127	116	98	107	87	133	124	123	113	82	108	113	120	115	106	145	128	110	108	90	89
84	153	90	99	105	92	105	100	103	103	96	111	101	94	100	108	87	114	116	117	115	119	93	98	115	98	103	108	96	113	119	127	117	98	107	88	133	124	123	113	82	108	113	120	115	106	145	128	110	108	90	89
83	153	89	99	105	92	105	100	103	102	96	111	100	93	100	108	86	114	115	117	115	119	92	98	115	98	102	108	96	113	119	127	116	98	107	87	133	124	123	113	82	108	113	120	115	106	145	128	110	108	90	89
83	153	89	99	105	92	105	100	103	102	96	111	100	93	100	108	86	114	115	117	115	119	92	98	115	98	102	108	96	113	119	127	116	98	107	87	133	124	123	113	82	108	113	120	115	106	145	128	110	108	90	89
83	153	89	99	105	92	105	100	103	102	96	111	100	93	100	108	86	114	115	117	115	119	92	98	115	98	102	108	96	113	119	127	116	98	107	87	133	124	123	113	82	108	113	120	115	106	145	128	110	108	90	89
83	153	89	99	105	92	105	100	103	102	96	111	100	93	100	108	86	114	115	117	115	119	92	98	115	98	102	108	96	113	119	127	116	98	107	87	133	124	123	113	82	108	113	120	115	106	145	128	110	108	90	89
83	153	89	99	105	92	105	100	103	102	96	111	100	93	100	108	86	114	115	117	115	119	92	98	115	98	102	108	96	113	119	127	116	98	107	87	133	124	123	113	82	108	113	120	115	106	145	128	110	108	90	89
83	153	89	99	105	92	105	100	103	102	96	111	100	93	100	108	86	114	115	117	115	119	92	98	115	98	102	108	96	113	119	127	116	98	107	87	133	124	123	113	82	108	113	120	115	106	145	128	110	108	90	89
83	153	89	99	105	92	105	100	103	102	96	111	100	93	100	108	86	114	115	117	115	119	92	98	115	98	102	108	96	113	119	127	116	98	107	87	133	124	123	113	82	108	113	120	115	106	145	128	110	108	90	89
83	153	89	99	105	92	105	100	103	102	96	111	100	93	100	108	86	114	115	117	115	119	92	98	115	98	102	108	96	113	119	127	116	98	107	87	133	124	123	113	82	108	113	120	115	106	145	128	110	108	90	89
83	153	89	99	105	92	105	100	103	102	96	111	100	93	100	108	86	114	115	117	115	119	92	98	115	98	102	108	96	113	119	127	116	98	107	87	133	124	123	113	82	108	113	120	115	106	145	128	110	108	90	89
83	153	89	99	105	92	105	100	103	102	96	111	100	93	100	108	86	114	115	117	115	119	92	98	115	98	102	108	96	113	119	127	116	98	107	87	133	124	123	113	82	108	113	120	115	106	145	128	110	108	90	89
83	153	89	99	105	92	105	100	103	102	96	111	100	93	100	108	86	114	115	117	115	119	92	98	115	98	102	108	96	113	119	127	116	98	107	87	133	124	123	113	82	108	113	120	115	106	145	128	110	108	90	89
83	153	89	99	105	92	105	100	103	102	96	111	100	93	100	108	86	114	115	117	115	119	92	98	115	98	102	108	96	113	119	127	116	98	107	87	133	124	123	113	82	108	113	120	115	106	145	128	110	108	90	89
83	153	89	99	105	92	105	100	103	102	96	111	100	93	100	108	86	114	115	117	115	119	92	98	115	98	102	108	96	113	119	127	116	98	107	87	133	124	123	113	82	108	113	120	115	106	145	128	110	108	90	89
83	153	89	99	105	92	105	100	103	102	96	111	100	93	100	108	86	114	115	117	115	119	92	98	115	98	102	108	96	113	119	127	116	98	107	87	133	124	123	113	82	108	113	120	115	106	145	128	110	108	90	89
126	144	126	124	126	121	125	120	130	125	120	128	129	121	123	126	127	129	119	121	122	130	127	123	122	115	131	126	131	126	119	127	128	121	121	120	127	128	123	129	125	123	125	131	125	136	140	128	127	127	117	118
106	147	105	97	101	95	96	101	102	115	95	113	107	101	97	103	102	115	108	119	111	104	109	95	112	102	105	108	94	108	112	132	119	117	122	98	124	119	122	116	102	119	130	122	124	107	141	129	101	117	93	103
122	146	123	119	129	121	111	120	122	132	118	126	117	128	114	131	129	131	128	123	126	116	125	126	121	124	121	122	126	127	119	130	125	124	125	122	109	121	121	128	113	119	138	128	123	121	141	132	127	117	114	117
123	146	121	120	130	122	111	120	120	133	119	126	116	127	114	129	130	131	128	124	124	117	126	124	121	125	122	121	124	126	117	130	125	124	126	121	110	121	121	129	112	120	139	128	124	120	141	133	128	117	115	118
123	137	132	123	121	126	126	126	122	125	122	110	128	120	127	117	126	117	113	109	116	115	121	129	115	123	123	126	124	123	116	117	106	118	124	126	124	110	120	119	121	117	118	110	113	124	120	108	121	114	121	124
118	146	134	135	127	127	127	131	130	132	130	143	123	126	133	132	126	136	140	140	135	133	129	131	140	130	129	137	134	138	134	137	143	132	141	129	131	135	131	135	127	126	136	136	129	137	142	140	134	130	130	131
129	135	122	121	114	127	128	121	118	116	120	110	125	124	124	115	126	105	107	106	123	118	120	131	116	120	127	116	127	126	115	111	93	112	117	129	124	109	110	115	121	107	117	99	112	106	115	107	120	117	125	129
102	145	89	82	93	116	105	96	99	83	114	70	101	103	118	82	104	80	91	85	97	104	105	105	110	104	111	98	78	99	81	107	84	88	70	100	102	104	90	95	108	87	78	92	85	112	125	87	86	87	108	97
115	136	126	122	119	115	120	126	119	122	117	131	114	128	116	128	113	129	126	127	120	123	124	128	124	119	121	121	121	122	130	135	126	132	130	119	121	112	124	128	114	123	129	128	128	115	132	127	121	120	123	121
102	145	90	82	93	116	105	97	100	83	114	71	101	103	119	83	104	81	92	85	98	104	105	106	110	104	111	98	79	99	82	108	85	89	70	100	102	105	91	95	108	87	78	93	85	112	125	87	86	88	108	97
102	145	89	82	94	116	105	97	99	83	114	70	101	103	118	82	104	80	91	85	97	104	105	105	110	105	111	99	78	99	81	107	84	88	70	100	102	104	90	95	108	87	78	92	85	112	125	87	87	87	108	98
146	138	142	147	140	143	141	140	142	140	144	147	139	146	139	149	143	151	136	142	144	138	147	144	142	144	145	138	147	149	141	141	138	147	148	141	137	143	127	144	137	149	146	140	137	137	138	139	140	147	145	142
136	132	135	140	131	135	125	139	140	125	138	135	133	135	131	138	133	137	126	127	134	134	139	137	130	134	131	136	136	135	128	129	126	134	138	134	130	133	123	136	128	131	132	117	124	142	132	127	127	139	132	135
135	147	134	141	129	133	137	129	140	132	134	141	137	136	139	144	135	141	130	132	138	146	137	142	140	139	136	138	141	136	134	138	140	135	142	134	136	139	136	136	132	138	132	131	128	141	146	135	134	141	137	132
147	47	140	151	144	152	139	144	152	140	144	140	141	146	142	149	142	145	140	147	133	145	147	146	139	135	151	144	145	145	146	128	138	139	147	145	125	142	132	135	142	149	133	139	131	145	96	134	140	148	141	147
150	140	153	157	152	148	149	152	157	153	149	155	154	153	149	159	151	158	152	153	155	152	152	154	150	154	153	159	158	155	153	149	149	151	154	151	141	151	143	151	148	158	153	155	152	154	149	153	154	158	156	155
133	148	139	135	130	131	142	137	140	137	131	138	131	138	135	140	130	137	130	135	130	146	134	145	134	136	131	138	143	135	132	145	137	136	138	138	143	137	139	139	140	135	129	133	133	142	136	137	129	136	140	133
120	148	128	110	121	125	135	119	117	117	118	124	121	120	119	118	111	124	126	124	127	129	128	127	134	122	125	119	121	113	128	134	131	123	111	121	124	128	121	125	132	119	116	132	132	134	137	125	123	118	126	127
82	141	84	86	90	75	78	97	99	106	72	108	90	102	64	100	94	111	106	109	101	91	104	99	103	96	105	105	81	84	96	122	111	107	111	95	121	102	118	97	92	105	105	99	106	88	130	102	103	102	96	103
129	133	124	133	126	136	124	120	136	114	127	112	123	125	127	125	127	120	117	114	114	116	131	134	114	121	120	122	131	131	119	120	99	123	120	117	117	113	105	131	111	119	122	115	117	127	117	107	118	115	125	131
109	142	127	118	108	120	124	123	122	118	113	125	120	122	122	127	117	124	134	132	126	127	125	127	129	120	133	131	122	116	132	142	130	125	121	123	128	123	131	127	132	120	121	127	120	129	129	129	128	124	126	135
123	132	117	120	122	127	127	120	121	117	118	99	124	128	125	116	124	101	111	97	123	123	123	128	113	123	129	119	117	123	113	104	95	108	111	127	123	116	114	109	124	99	94	90	91	121	111	99	121	103	125	126
122	134	109	119	122	128	123	109	115	111	116	95	121	118	119	112	124	104	105	96	110	122	117	125	115	114	127	107	117	116	108	103	96	102	108	123	117	106	98	106	112	99	101	91	98	113	109	91	116	100	114	121
122	134	109	119	122	128	123	109	115	111	116	95	121	118	119	112	124	104	105	96	110	122	117	125	115	114	127	107	117	116	108	103	96	102	108	123	117	106	98	106	112	99	101	91	98	113	109	91	116	100	114	121
122	134	109	119	122	128	123	109	115	111	116	95	121	118	119	112	124	104	105	96	110	122	117	125	115	114	127	107	117	116	108	103	96	102	108	123	117	106	98	106	112	99	101	91	98	113	109	91	116	100	114	121
122	134	109	119	122	128	123	109	115	111	116	95	121	118	119	112	124	104	105	96	110	122	117	125	115	114	127	107	117	116	108	103	96	102	108	123	117	106	98	106	112	99	101	91	98	113	109	91	116	100	114	121
103	145	105	111	113	106	102	107	110	112	105	108	116	105	110	105	108	119	109	118	111	128	101	102	102	120	97	117	108	110	117	123	109	102	112	109	124	127	115	109	102	119	118	121	122	113	136	118	111	102	102	96
106	145	111	108	112	119	117	112	117	115	121	118	122	127	122	109	121	125	109	112	121	106	114	122	115	127	129	111	111	115	118	113	108	115	110	115	121	104	111	105	111	104	121	114	114	107	131	122	121	102	120	118
144	90	144	146	147	150	144	146	142	142	142	139	142	137	140	140	141	141	132	137	137	132	141	144	134	134	141	138	146	140	146	118	130	130	136	143	128	128	120	131	143	141	135	131	129	137	92	129	139	139	136	143
133	133	117	138	118	138	121	112	128	103	125	113	122	119	123	124	120	113	116	110	123	128	131	126	118	118	121	125	133	127	115	113	91	126	116	136	114	119	112	111	112	112	118	113	112	121	111	103	120	115	116	112
114	141	126	121	120	135	135	120	118	110	126	118	123	118	123	123	112	111	113	112	130	118	125	129	133	129	117	111	128	123	132	106	101	125	120	124	129	119	114	125	124	117	115	107	118	122	120	107	124	104	127	126
110	153	107	108	107	69	105	109	103	117	100	121	112	106	104	104	108	126	108	109	105	109	99	83	101	108	108	99	100	102	104	123	123	117	124	99	131	122	120	105	85	112	123	123	121	100	143	128	104	123	94	95
110	153	107	108	107	69	105	109	103	117	100	121	112	106	104	104	108	126	108	109	105	109	99	83	101	108	108	99	100	102	104	123	123	117	124	99	131	122	120	105	85	112	123	123	121	100	143	128	104	123	94	95
110	153	107	108	107	69	105	109	103	117	100	121	112	106	104	104	108	126	108	109	105	109	99	83	101	108	108	99	100	102	104	123	123	117	124	99	131	122	120	105	85	112	123	123	121	100	143	128	104	123	94	95
110	153	107	108	107	69	105	109	103	117	100	121	112	106	104	104	108	126	108	109	105	109	99	83	101	108	108	99	100	102	104	123	123	117	124	99	131	122	120	105	85	112	123	123	121	100	143	128	104	123	94	95
102	144	90	98	100	94	95	107	90	99	104	110	109	110	106	102	111	110	100	109	107	113	96	99	114	114	106	104	92	96	97	119	110	111	114	85	118	111	114	101	94	114	117	112	114	90	137	118	100	112	109	110
145	66	143	148	137	148	135	149	148	140	146	138	140	142	142	145	143	142	144	151	137	145	147	149	139	137	145	152	142	144	147	133	139	137	142	148	132	134	135	133	146	152	132	130	132	142	94	133	141	145	140	149
115	139	133	122	113	119	124	132	122	120	112	135	121	123	120	128	110	134	129	134	126	123	119	133	118	126	121	123	126	120	126	141	131	127	129	123	122	128	124	134	128	135	134	135	127	121	135	129	122	124	135	129
128	136	114	121	119	138	123	119	130	101	125	104	122	123	127	119	124	101	107	100	111	125	125	132	118	124	125	119	120	122	110	108	98	116	109	126	110	108	106	109	118	104	109	98	104	121	114	106	113	105	125	121
73	142	82	82	80	111	95	84	103	78	110	92	91	97	114	86	94	98	92	105	106	113	97	115	106	110	98	114	94	103	93	116	83	76	66	101	111	112	112	88	125	97	88	100	104	109	130	104	92	66	119	101
74	142	83	83	80	111	96	84	104	79	111	93	92	98	114	87	95	99	93	106	107	113	97	116	106	111	98	114	95	104	94	117	84	77	66	102	111	113	112	89	126	98	89	101	105	109	131	105	92	67	119	101
73	142	82	82	80	111	95	84	103	78	110	92	91	97	114	86	94	98	92	105	106	113	97	115	106	110	98	114	94	103	93	116	83	76	66	101	111	112	112	88	125	97	88	100	104	109	130	104	92	66	119	101
73	142	82	82	80	111	95	84	103	78	110	92	91	97	114	86	94	98	92	105	106	113	97	115	106	110	98	114	94	103	93	116	83	76	66	101	111	112	112	88	125	97	88	100	104	109	130	104	92	66	119	101
73	142	82	82	80	111	95	84	103	78	110	92	91	97	114	86	94	98	92	105	106	113	97	115	106	110	98	114	94	103	93	116	83	76	66	101	111	112	112	88	125	97	88	100	104	109	130	104	92	66	119	101
73	142	82	82	80	111	95	84	103	78	110	92	91	97	114	86	94	98	92	105	106	113	97	115	106	110	98	114	94	103	93	116	83	76	66	101	111	112	112	88	125	97	88	100	104	109	130	104	92	66	119	101
73	142	82	82	80	111	95	84	103	78	110	92	91	97	114	86	94	98	92	105	106	113	97	115	106	110	98	114	94	103	93	116	83	76	66	101	111	112	112	88	125	97	88	100	104	109	130	104	92	66	119	101
73	142	82	82	80	111	95	84	103	78	110	92	91	97	114	86	94	98	92	105	106	113	97	115	106	110	98	114	94	103	93	116	83	76	66	101	111	112	112	88	125	97	88	100	104	109	130	104	92	66	119	101
73	142	82	82	80	111	95	84	103	78	110	92	91	97	114	86	94	98	92	105	106	113	97	115	106	110	98	114	94	103	93	116	83	76	66	101	111	112	112	88	125	97	88	100	104	109	130	104	92	66	119	101
73	142	82	82	80	111	95	84	103	78	110	92	91	97	114	86	94	98	92	105	106	113	97	115	106	110	98	114	94	103	93	116	83	76	66	101	111	112	112	88	125	97	88	100	104	109	130	104	92	66	119	101
73	142	82	82	80	111	95	84	103	78	110	92	91	97	114	86	94	98	92	105	106	113	97	115	106	110	98	114	94	103	93	116	83	76	66	101	111	112	112	88	125	97	88	100	104	109	130	104	92	66	119	101
73	142	82	82	80	111	95	84	103	78	110	92	91	97	114	86	94	98	92	105	106	113	97	115	106	110	98	114	94	103	93	116	83	76	66	101	111	112	112	88	125	97	88	100	104	109	130	104	92	66	119	101
73	142	82	82	80	111	95	84	103	78	110	92	91	97	114	86	94	98	92	105	106	113	97	115	106	110	98	114	94	103	93	116	83	76	66	101	111	112	112	88	125	97	88	100	104	109	130	104	92	66	119	101
73	142	82	82	80	111	95	84	103	78	110	92	91	97	114	86	94	98	92	105	106	113	97	115	106	110	98	114	94	103	93	116	83	76	66	101	111	112	112	88	125	97	88	100	104	109	130	104	92	66	119	101
73	142	82	82	80	111	95	84	103	78	110	92	91	97	114	86	94	98	92	105	106	113	97	115	106	110	98	114	94	103	93	116	83	76	66	101	111	112	112	88	125	97	88	100	104	109	130	104	92	66	119	101
73	142	82	82	80	111	95	84	103	78	110	92	91	97	114	86	94	98	92	105	106	113	97	115	106	110	98	114	94	103	93	116	83	76	66	101	111	112	112	88	125	97	88	100	104	109	130	104	92	66	119	101
73	142	82	82	80	111	95	84	103	78	110	92	91	97	114	86	94	98	92	105	106	113	97	115	106	110	98	114	94	103	93	116	83	76	66	101	111	112	112	88	125	97	88	100	104	109	130	104	92	66	119	101
73	142	82	82	80	111	95	84	103	78	110	92	91	97	114	86	94	98	92	105	106	113	97	115	106	110	98	114	94	103	93	116	83	76	66	101	111	112	112	88	125	97	88	100	104	109	130	104	92	66	119	101
73	142	82	82	80	111	95	84	103	78	110	92	91	97	114	86	94	98	92	105	106	113	97	115	106	110	98	114	94	103	93	116	83	76	66	101	111	112	112	88	125	97	88	100	104	109	130	104	92	66	119	101
73	142	82	82	80	111	95	84	103	78	110	92	91	97	114	86	94	98	92	105	106	113	97	115	106	110	98	114	94	103	93	116	83	76	66	101	111	112	112	88	125	97	88	100	104	109	130	104	92	66	119	101
73	142	82	82	80	111	95	84	103	78	110	92	91	97	114	86	94	98	92	105	106	113	97	115	106	110	98	114	94	103	93	116	83	76	66	101	111	112	112	88	125	97	88	100	104	109	130	104	92	66	119	101

73	142	82	82	80	111	95	84	103	78	110	92	91	97	114	86	94	98	92	105	106	113	97	115	106	110	98	114	94	103	93	116	83	76	66	101	111	112	112	88	125	97	88	100	104	109	130	104	92	66	119	101
123	137	132	123	121	126	126	126	122	125	122	110	128	120	127	117	126	117	113	109	116	115	121	129	115	123	123	126	124	123	116	117	106	118	124	126	124	110	120	119	121	117	118	110	113	124	120	108	121	114	121	124
113	145	121	119	119	112	116	120	117	117	114	133	116	121	113	129	110	131	127	132	125	122	118	118	126	124	119	127	119	124	127	140	128	129	128	120	119	123	125	122	117	128	135	135	129	119	144	138	127	128	120	118
115	145	122	119	117	111	121	121	120	120	115	135	120	115	116	126	110	132	130	129	129	128	115	118	126	125	116	128	122	121	128	138	129	125	131	122	126	128	126	121	120	127	131	139	132	114	142	138	127	130	123	117
104	142	112	117	123	120	118	122	104	118	113	107	122	116	124	112	117	105	112	103	121	126	106	124	113	124	116	107	109	114	111	111	106	106	111	120	119	118	118	108	105	91	99	96	87	114	124	108	120	94	114	109
101	146	106	101	108	96	108	103	101	113	94	110	112	91	101	104	94	115	113	114	117	111	92	99	111	101	105	113	104	104	119	130	118	102	111	92	123	122	127	114	95	110	125	127	123	106	143	126	107	106	100	101
128	133	124	133	126	136	124	119	136	114	127	112	122	124	127	125	126	120	117	114	114	116	130	134	114	121	120	122	131	131	119	120	99	123	120	117	117	113	105	131	111	119	122	115	117	127	117	107	117	115	125	130
128	133	124	133	126	136	124	119	136	114	127	112	122	124	127	125	126	120	117	114	114	116	130	134	114	121	120	122	131	131	119	120	99	123	120	117	117	113	105	131	111	119	122	115	117	127	117	107	117	115	125	130
128	133	124	133	126	136	124	119	136	114	127	112	122	124	127	125	126	120	117	114	114	116	130	134	114	121	120	122	131	131	119	120	99	123	120	117	117	113	105	131	111	119	122	115	117	127	117	107	117	115	125	130
128	133	124	133	126	136	124	119	136	115	127	112	122	124	127	125	127	120	117	114	114	116	130	134	114	121	121	122	131	131	119	120	99	123	121	118	117	113	105	131	112	119	122	115	117	127	117	107	117	115	125	130
128	133	124	133	126	136	124	119	136	114	127	112	122	124	127	125	126	120	117	114	114	116	130	134	114	121	120	122	131	131	119	120	99	123	120	117	117	113	105	131	111	119	122	115	117	127	117	107	117	115	125	130
125	142	132	129	126	119	122	133	125	135	116	128	131	129	122	130	129	128	135	128	127	122	132	133	129	126	132	121	127	122	124	133	128	126	133	126	129	131	127	142	124	121	134	129	122	130	139	131	135	125	130	133
143	81	141	140	143	144	138	144	143	140	139	131	138	140	135	141	141	138	134	134	136	131	144	147	136	127	141	142	138	145	140	128	135	128	139	140	132	137	138	136	139	143	136	133	127	139	86	126	137	142	138	141
95	147	96	101	98	97	100	96	93	105	87	117	97	99	97	112	96	122	113	122	110	116	96	91	113	105	96	105	99	95	107	125	119	107	124	93	126	131	122	108	84	118	119	120	123	98	145	129	101	107	100	100
105	147	105	117	120	114	112	113	119	117	110	130	116	111	115	121	116	128	121	128	121	131	109	116	121	126	115	119	119	126	125	137	129	119	117	110	126	135	130	125	105	122	134	128	127	120	143	142	125	114	111	104
111	140	118	103	119	113	103	101	97	104	110	87	103	108	112	94	118	97	92	74	91	86	106	102	88	95	99	75	107	103	84	96	75	92	91	99	109	88	85	101	93	74	111	102	102	100	125	67	97	87	100	113
112	141	119	103	120	113	105	101	97	105	110	88	103	108	112	95	118	99	93	76	93	88	106	103	90	96	99	77	108	105	85	98	77	93	92	101	109	90	87	101	94	76	112	103	103	100	126	68	98	89	101	113
111	140	118	103	119	113	103	101	97	104	110	87	103	108	112	94	118	97	92	74	91	86	106	102	88	95	99	75	107	103	84	96	75	92	91	99	109	88	85	101	93	74	111	102	102	100	125	67	97	87	100	113
111	140	118	103	119	113	103	101	97	104	110	87	103	108	112	94	118	97	92	74	91	86	106	102	88	95	99	75	107	103	84	96	75	92	91	99	109	88	85	101	93	74	111	102	102	100	125	67	97	87	100	113
111	140	118	103	119	113	103	101	97	104	110	87	103	108	112	94	118	97	92	74	91	86	106	102	88	95	99	75	107	103	84	96	75	92	91	99	109	88	85	101	93	74	111	102	102	100	125	67	97	87	100	113
111	140	118	103	119	113	103	101	97	104	110	87	103	108	112	94	118	97	92	74	91	86	106	102	88	95	99	75	107	103	84	96	75	92	91	99	109	88	85	101	93	74	111	102	102	100	125	67	97	87	100	113
111	140	118	103	119	113	103	101	97	104	110	87	103	108	112	94	118	97	92	74	91	86	106	102	88	95	99	75	107	103	84	96	75	92	91	99	109	88	85	101	93	74	111	102	102	100	125	67	97	87	100	113
112	140	118	103	119	113	104	100	97	104	111	88	102	108	112	94	118	97	91	75	91	86	106	102	87	94	98	76	107	104	85	96	75	93	91	100	109	88	85	101	93	75	111	102	102	100	125	67	97	88	100	113
111	140	118	103	119	113	103	101	97	104	110	87	103	108	112	94	118	97	92	74	91	86	106	102	88	95	99	75	107	103	84	96	75	92	91	99	109	88	85	101	93	74	111	102	102	100	125	67	97	87	100	113
111	140	118	103	119	113	103	101	97	104	110	87	103	108	112	94	118	97	92	74	91	86	106	102	88	95	99	75	107	103	84	96	75	92	91	99	109	88	85	101	93	74	111	102	102	100	125	67	97	87	100	113
111	140	118	103	119	113	103	101	97	104	110	87	103	108	112	94	118	97	92	74	91	86	106	102	88	95	99	75	107	103	84	96	75	92	91	99	109	88	85	101	93	74	111	102	102	100	125	67	97	87	100	113
111	140	118	103	119	113	103	101	97	104	110	87	103	108	112	94	118	97	92	74	91	86	106	102	88	95	99	75	107	103	84	96	75	92	91	99	109	88	85	101	93	74	111	102	102	100	125	67	97	87	100	113
111	140	118	103	119	113	103	101	97	104	110	87	103	108	112	94	118	97	92	74	91	86	106	102	88	95	99	75	107	103	84	96	75	92	91	99	109	88	85	101	93	74	111	102	102	100	125	67	97	87	100	113
111	140	118	103	119	113	103	101	97	104	110	87	103	108	112	94	118	97	92	74	91	86	106	102	88	95	99	75	107	103	84	96	75	92	91	99	109	88	85	101	93	74	111	102	102	100	125	67	97	87	100	113
111	140	118	103	119	113	103	101	97	104	110	87	103	108	112	94	118	97	92	74	91	86	106	102	88	95	99	75	107	103	84	96	75	92	91	99	109	88	85	101	93	74	111	102	102	100	125	67	97	87	100	113
111	140	118	103	119	113	103	101	97	104	110	87	103	108	112	94	118	97	92	74	91	86	106	102	88	95	99	75	107	103	84	96	75	92	91	99	109	88	85	101	93	74	111	102	102	100	125	67	97	87	100	113
111	140	118	103	119	113	103	101	97	104	110	87	103	108	112	94	118	97	92	74	91	86	106	102	88	95	99	75	107	103	84	96	75	92	91	99	109	88	85	101	93	74	111	102	102	100	125	67	97	87	100	113
111	140	118	103	119	113	103	101	97	104	110	87	103	108	112	94	118	97	92	74	91	86	106	102	88	95	99	75	107	103	84	96	75	92	91	99	109	88	85	101	93	74	111	102	102	100	125	67	97	87	100	113
111	141	118	102	118	112	102	100	98	104	109	86	103	108	111	93	118	96	92	73	91	85	106	103	87	94	99	74	106	102	83	95	75	91	90	99	108	89	86	102	93	73	110	102	103	101	126	68	96	86	101	112
109	145	123	126	119	116	121	121	125	122	118	138	122	120	122	134	118	138	132	141	132	128	127	122	132	128	121	132	126	129	136	141	135	133	133	124	130	129	136	130	122	131	134	136	127	129	145	139	131	128	127	125
112	145	129	124	117	116	119	124	123	119	114	139	121	120	120	135	115	138	129	136	131	126	127	122	134	128	117	131	127	126	132	142	133	137	139	127	130	129	135	129	120	131	133	139	128	129	145	137	130	129	125	122
112	145	129	124	117	116	119	124	123	119	114	139	121	120	120	135	115	138	129	136	131	126	127	122	134	128	117	131	127	126	132	142	133	137	139	127	130	129	135	129	120	131	133	139	129	129	145	137	130	129	125	122
112	145	129	124	117	116	119	124	123	119	114	139	121	120	120	135	115	138	129	136	131	126	127	122	134	128	117	131	127	126	132	142	133	137	139	127	130	129	135	129	120	131	133	139	128	129	145	137	130	129	125	122
109	145	123	126	119	116	121	121	125	122	118	138	122	120	122	134	118	138	132	141	132	128	127	122	132	128	121	132	126	129	136	141	135	133	133	124	130	129	136	130	122	131	134	136	127	129	145	139	131	128	127	125
109	145	123	126	119	116	121	121	125	122	118	138	122	120	122	134	118	138	132	141	132	128	127	122	132	128	121	132	126	129	136	141	135	133	133	124	130	129	136	130	122	131	134	136	127	129	145	139	131	128	127	125
112	146	125	117	120	115	120	124	120	126	114	135	119	119	119	131	114	131	133	134	126	127	118	123	134	128	118	128	125	124	128	137	130	130	136	125	125	130	132	132	120	127	134	131	126	123	141	143	131	129	125	124
112	145	129	124	117	116	119	124	123	119	114	139	121	120	120	135	115	138	129	136	131	126	127	122	134	128	117	131	127	126	132	142	133	137	139	127	130	129	135	129	120	131	133	139	128	129	145	137	130	129	125	122
112	145	129	124	117	116	119	124	123	119	114	139	121	120	120	135	115	138	129	136	131	126	127	122	134	128	117	131	127	126	132	142	133	137	139	127	130	129	135	129	120	131	133	139	128	129	145	137	130	129	125	122
112	145	129	124	117	116	119	124	123	119	114	139	121	120	120	135	115	138	129	136	131	126	127	122	134	128	117	131	127	126	132	142	133	137	139	127	130	129	135	129	120	131	133	139	128	129	145	137	130	129	125	122
109	145	123	126	119	116	121	121	125	122	118	138	122	120	122	134	118	138	132	141	132	128	127	122	132	128	121	132	126	129	136	141	135	133	133	124	130	129	136	130	122	131	134	136	127	129	145	139	131	128	127	125
112	145	129	124	117	116	119	124	123	119	114	139	121	120	120	135	115	138	129	136	131	126	127	122	134	128	117	131	127	126	132	142	133	137	139	127	130	129	135	129	120	131	133	139	128	129	145	137	130	129	125	122
110	148	121	112	118	114	116	129	118	124	112	127	110	126	116	121	120	128	123	125	120	117	116	128	130	119	118	115	114	118	119	133	128	132	126	119	112	116	119	125	122	122	131	117	116	109	141	128	119	118	123	124
132	116	122	131	129	135	127	127	127	125	126	115	128	124	130	126	134	129	116	120	124	125	127	133	117	119	132	118	126	128	122	116	110	114	123	126	130	119	111	128	116	124	121	121	113	125	101	120	122	127	127	128
108	143	102	102	104	85	99	108	88	111	95	108	99	99	101	104	117	105	111	101	95	99	104	95	98	88	107	82	90	93	95	126	109	107	113	93	124	107	118	106	101	102	118	114	110	98	128	105	101	118	97	108
108	143	102	102	104	85	99	108	88	111	95	108	99	99	101	104	117	105	111	101	95	99	104	95	98	88	107	82	90	93	95	126	109	107	113	93	124	107	118	106	101	102	118	114	110	98	128	105	101	118	97	108
108	143	102	102	104	85	99	108	88	111	95	108	99	99	101	104	117	105	111	101	95	99	104	95	98	88	107	82	90	93	95	126	109	107	113	93	124	107	118	106	101	102	118	114	110	98	128	105	101	118	97	108
108	143	102	102	104	85	99	108	88	111	95	108	99	99	101	104	117	105	111	101	95	99	104	95	98	88	107	82	90	93	95	126	109	107	113	93	124	107	118	106	101	102	118	114	110	98	128	105	101	118	97	108
97	139	108	99	106	97	105	106	107	117	97	107	100	102	100	99	111	103	99	95	105	88	104	111	79	96	98	76	93	96	93	107	98	113	98	89	118	112	106	120	83	100	122	115	109	100	129	104	94	90	88	96
120	125	120	111	123	117	118	106	120	119	120	106	113	117	112	118	115	110	109	96	106	126	124	122	118	110	132	120	121	118	116	112	94	109	106	126	117	90	99	94	110	101	93	104	117	110	107	93	124	116	120	128
92	141	105	95	103	96	97	107	96	106	98	104	105	102	104	90	106	109	91	96	108	89	83	104	90	98	96	71	95	94	96	102	99	105	104	81	120	113	111	115	87	99	113	113	109	101	138	101	94	84	91	93
122	146	123	119	129	121	111	120	122	132	118	126	117	128	114	131	129	131	128	123	126	116	125	126	121	124	121	122	126	127	119	130	125	124	125	122	109	121	121	128	113	119	138	128	123	121	141	132	127	117	114	117
104	145	89	84	94	118	106	97	100	84	115	72	102	103	119	83	105	82	93	87	98	105	107	105	112	104	112	99	79	100	83	108	86	90	72	102	103	105	91	97	109	88	80	94	87	114	126	89	87	89	108	97
144	90	144	146	147	150	144	146	142	142	142	139	142	137	140	140	141	141	132	137	137	132	141	144	134	134	141	138	146	140	146	118	130	130	136	143	128	128	120	131	143	141	135	131	129	137	92	129	139	139	136	143
100	141	109	108	110	114	110	120	110	113	110	127	110	118	114	113	107	123	119	123	116	119	111	111	118	110	123	118	108	109	118	132	128	119	119	103	124	118	120	113	114	125	117	129	125	113	141	135	116	120	120	116
102	145	89	82	93	116	105	96	99	83	114	70	101	103	118	82	104	80	91	85	97	104	105	105	110	104	111	98	78	99	81	107	84	88	70	100	102	104	90	95	108	87	78	92	85	112	125	87	86	87	108	97
102	145	89	82	93	116	105	96	99	83	114	70	101	103	118	82	104	80	91	85	97	104	105	105	110	104	111	98	78	99	81	107	84	88	70	100	102	104	90	95	108	87	78	92	85	112	125	87	86	87	108	97
102	145	89	82	93	116	105	96	99	83	114	70	101	103	118	82	104	80	91	85	97	104	105	105	110	104	111	98	78	99	81	107	84	88	70	100	102	104	90	95	108	87	78	92	85	112	125	87	86	87	108	97
148	73	138	149	148	151	141	150	151	146	140	139	142	146	140	149	144	140	136	146	138	150	145	146	140	136	151	147	145	149	148	123	140	139	149	147	130	143	134	140	146	148	131	139	134	144	97	140	141	149	140	145
116	136	99	111	109	126	120	109	109	94	118	95	114	112	119	100	115	103	99	96	103	117	110	113	110	111	113	117	107	108	102	112	101	98	101	114	117	110	101	105	113	95	95	97	88	119	113	98	102	98	114	111
118	143	114	109	119	124	116	105	129	123	119	120	111	127	119	124	113	119	123	120	116	129	128	126	120	113	129	132	120	132	116	136	115	113	107	121	109	97	122	111	129	111	115	118	120	126	131	132	121	112	128	113
126	123	117	134	120	134	122	126	123	120	125	102	128	129	124	125	126	111	116	115	116	118	131	132	120	117	124	117	125	121	117	117	103	111	117	129	121	112	118	122	115	110	113	110	98	121	96	113	123	115	126	131
88	149	104	89	106	75	101	102	89	117	81	108	99	97	88	105	87	115	104	114	102	104	82	91	100	102	92	95	93	96	106	122	114	103	115	87	121	117	118	111	82	115	126	114	121	96	148	129	102	112	88	88
87	145	99	87	92	69	92	92	100	105	73	115	83	84	86	103	75	114	92	110	105	103	72	94	90	89	83	99	94	97	102	130	107	109	105	78	124	123	120	107	81	115	111	119	114	101	138	122	90	110	74	77
128	129	130	131	124	138	127	119	126	114	130	109	124	123	124	126	128	107	109	107	111	126	134	137	126	124	125	118	127	128	116	104	97	126	111	134	117	106	116	123	124	106	110	99	106	120	120	104	118	111	126	128
126	131	128	123	134	134	131	126	117	126	131	115	130	127	122	120	133	116	110	111	117	121	121	126	118	120	127	122	131	124	121	103	95	118	115	138	130	113	102	106	126	114	118	98	108	106	119	103	134	112	126	131
97	148	94	96	105	114	110	95	102	101	106	93	101	98	107	102	100	92	106	101	114	134	108	111	123	108	113	115	104	114	104	120	110	83	89	113	117	106	127	105	107	95	75	109	107	124	136	114	118	88	109	94
128	126	119	125	130	138	131	126	124	116	131	106	125	125	132	119	131	112	117	107	125	129	129	136	131	129	132	121	124	126	117	116	108	115	118	137	126	122	109	112	129	107	107	112	101	119	108	102	125	111	129	128
91	148	112	98	110	85	98	105	103	106	89	127	105	99	105	111	92	121	121	119	119	108	91	96	107	114	99	111	104	110	115	126	118	110	129	101	128	126	130	109	97	115	126	124	120	107	146	138	113	120	98	98
123	130	123	123	126	130	118	107	121	111	127	103	116	125	120	120	127	104	111	112	115	120	123	127	114	118	119	117	117	121	128	96	100	119	113	127	118	115	109	109	109	99	98	91	103	117	113	98	112	110	118	127

131	145	140	137	133	135	130	141	134	132	128	139	136	134	136	134	133	135	139	140	132	134	135	140	137	137	143	137	140	136	138	141	139	133	139	130	137	142	139	145	133	132	141	133	130	144	143	136	140	133	135	138
95	135	87	98	94	98	84	95	97	95	92	72	102	93	97	88	107	88	81	98	92	106	92	105	80	86	102	85	81	84	74	92	75	93	94	91	110	107	101	107	99	98	88	107	102	114	127	90	86	93	85	81
83	138	93	89	104	108	92	96	86	104	96	81	93	93	88	77	103	104	95	92	60	95	91	89	88	73	100	93	88	80	74	107	97	88	98	92	122	115	120	101	97	93	105	116	116	111	117	96	81	93	87	93
101	137	107	97	101	119	101	98	103	90	105	77	91	98	105	93	112	92	88	82	91	104	106	106	99	94	89	85	95	103	84	110	82	81	82	101	95	106	106	101	120	97	103	118	111	118	128	88	81	68	110	102
117	136	118	100	112	104	102	110	123	110	116	110	111	121	116	108	116	103	100	99	112	113	110	120	96	101	109	118	115	112	110	115	101	117	103	108	114	101	108	101	107	92	102	84	103	110	125	108	94	105	104	99
86	135	89	83	76	73	89	87	110	84	93	102	64	81	83	86	75	107	106	97	86	96	81	111	92	63	71	109	83	98	85	133	101	109	99	89	118	114	126	104	99	103	101	110	118	103	130	110	63	103	91	81
120	139	117	91	122	104	97	91	115	112	111	90	95	109	96	100	125	101	93	82	101	71	101	105	91	87	96	96	100	115	101	100	86	105	97	99	105	102	104	104	86	97	119	103	115	103	123	88	89	103	85	98
90	149	103	87	106	86	90	82	75	105	87	102	95	92	81	77	109	109	97	89	94	95	74	88	75	95	91	84	92	92	78	90	95	80	91	91	103	109	112	105	79	100	110	122	112	96	143	105	94	86	86	87
149	124	143	146	145	150	148	144	148	140	141	139	145	146	140	147	146	141	150	144	141	145	148	152	139	143	147	149	147	150	145	138	136	141	141	148	139	142	139	137	147	147	137	137	141	144	123	140	146	142	151	151
117	140	117	116	111	108	110	115	112	113	111	114	121	112	114	117	125	122	109	115	119	113	115	122	107	121	115	104	119	114	108	115	101	109	105	117	91	91	81	115	109	112	113	129	113	112	136	93	112	105	108	99
90	145	102	90	90	80	63	77	96	101	76	104	78	94	77	106	84	111	101	98	91	89	87	89	86	77	95	93	98	86	94	123	99	95	106	87	126	105	115	96	84	95	111	112	106	100	133	107	100	109	74	95
144	46	138	149	141	149	138	144	150	143	143	138	140	145	141	144	139	137	140	145	140	143	149	150	137	133	150	144	140	148	147	130	142	137	140	143	129	142	139	140	143	145	131	133	124	143	88	138	140	145	144	145
136	64	139	141	139	139	138	153	143	142	139	135	144	141	142	141	141	137	137	142	141	144	147	145	134	131	146	143	136	144	144	131	141	135	139	141	134	140	138	139	140	146	130	137	128	146	97	131	140	146	138	141
85	144	110	74	100	98	95	102	95	108	98	89	87	95	91	89	94	100	101	97	62	99	102	98	80	95	112	92	85	82	86	120	87	95	101	93	124	109	117	101	91	98	104	116	111	109	135	99	107	104	87	101
75	138	109	88	82	52	78	98	109	96	73	114	83	89	87	106	69	115	102	106	108	96	82	106	87	86	95	106	94	97	101	132	106	109	107	85	120	122	125	108	95	107	107	117	107	101	136	114	97	112	78	82
86	139	111	82	89	97	102	88	96	87	90	98	92	90	92	95	81	103	77	98	89	101	84	96	82	88	89	92	98	87	92	102	89	106	84	78	110	111	96	111	72	102	96	113	114	108	128	95	85	95	97	96
93	140	97	98	100	93	103	102	97	100	97	111	103	95	105	97	99	114	88	99	92	110	89	97	87	99	95	93	99	100	81	118	100	110	82	93	119	117	111	102	74	107	107	120	108	98	133	104	94	104	96	79
74	147	92	71	89	110	104	98	83	89	97	80	96	96	106	70	90	88	92	91	70	99	89	99	89	101	110	81	79	75	84	100	90	80	89	84	118	99	94	97	99	92	93	115	117	114	139	98	102	79	102	106
91	147	106	79	102	50	77	104	94	105	79	104	94	100	78	100	91	109	109	107	101	90	80	99	87	86	106	91	85	93	92	116	110	100	110	90	116	118	114	101	94	108	112	111	110	93	140	121	104	104	81	86
98	134	105	110	105	107	105	111	108	105	105	108	106	109	106	113	111	111	101	110	114	112	106	126	99	117	107	101	109	116	102	106	103	106	104	98	102	111	97	118	103	114	108	121	112	108	129	93	101	93	112	96
114	133	127	104	116	109	115	108	118	109	103	117	109	122	107	124	105	112	110	107	121	117	115	125	115	110	119	110	120	118	117	123	104	114	119	116	90	99	105	115	113	110	114	109	113	110	125	112	111	103	115	114
92	152	95	75	96	76	93	96	69	105	79	89	95	93	89	69	106	104	93	92	79	96	74	71	85	85	97	75	70	81	69	106	106	82	100	78	117	109	111	105	84	105	99	117	112	100	142	114	85	101	82	91
83	147	76	92	108	91	92	92	88	109	80	100	93	91	85	98	91	100	101	93	100	120	87	98	97	100	96	99	89	97	86	119	116	85	93	95	119	120	124	102	86	97	94	116	110	105	134	118	105	81	89	77
144	48	138	150	138	147	134	149	148	143	143	141	141	148	138	145	142	139	141	149	137	138	147	151	133	135	152	146	141	143	148	132	147	139	145	143	134	147	144	138	145	149	135	136	130	142	92	140	141	145	142	144
93	144	92	90	102	106	110	103	95	96	103	96	105	102	107	89	95	99	106	98	105	112	97	109	112	106	103	85	90	104	95	114	96	94	94	103	116	106	112	108	100	85	100	114	103	98	134	108	98	90	109	99
69	137	90	94	103	98	93	103	97	105	95	95	91	89	90	92	107	112	97	101	71	89	85	98	80	85	107	92	86	74	92	110	99	100	98	83	126	110	117	106	87	101	111	115	115	106	119	97	85	99	85	93
83	137	115	97	84	70	86	113	108	99	86	124	92	95	91	107	82	121	117	114	116	108	93	114	103	107	96	117	98	99	115	131	111	118	111	102	128	121	130	106	107	108	105	119	112	108	137	117	92	118	90	93
107	144	97	99	109	96	103	92	94	108	97	96	97	102	88	89	109	103	100	89	100	104	94	102	96	93	106	90	84	104	86	103	96	98	90	92	115	105	115	103	88	104	100	109	119	95	135	108	94	93	102	92
77	134	82	82	86	93	94	90	101	88	85	94	86	93	95	91	75	106	104	97	100	111	95	104	98	94	92	99	103	88	94	124	101	90	85	98	112	116	113	109	113	91	89	113	109	115	124	107	92	82	107	97
93	130	105	93	95	84	99	100	114	96	86	109	89	86	101	105	84	109	105	100	98	93	100	97	95	86	98	97	98	92	102	128	100	122	103	80	126	117	118	120	91	111	115	127	122	110	134	113	96	112	92	96
80	136	105	85	89	58	77	106	109	101	80	106	97	101	82	97	83	109	99	107	104	92	92	107	81	81	97	99	87	92	97	120	108	108	106	84	123	116	121	106	98	101	106	115	112	108	136	114	92	112	79	83
98	138	84	96	85	113	95	97	88	90	93	66	89	99	98	75	105	81	79	79	74	103	95	95	86	82	89	72	83	89	57	92	83	79	94	84	108	108	95	115	107	96	87	120	107	117	126	90	75	75	95	92
125	140	113	121	117	132	123	115	124	110	127	117	129	128	122	119	123	120	111	118	119	120	133	128	116	120	129	120	122	124	115	123	104	119	105	128	94	79	91	110	121	110	115	117	118	123	131	102	115	108	123	116
97	143	105	95	103	82	98	98	92	116	85	111	96	94	93	96	100	120	97	100	92	86	92	76	91	95	100	90	100	82	95	117	101	110	116	82	129	118	112	105	68	113	120	114	113	95	134	112	102	116	80	93
133	96	126	133	130	138	122	127	140	117	140	114	128	140	130	129	134	112	122	114	136	131	133	139	130	136	138	139	126	131	127	107	119	122	123	139	116	130	118	116	139	118	106	104	105	129	72	103	124	121	138	139
90	137	86	81	93	93	90	71	97	90	87	91	81	87	95	89	78	93	93	93	103	106	68	100	88	89	87	99	89	96	90	107	92	87	85	86	118	119	114	103	98	94	95	106	112	102	130	103	92	83	99	96
86	138	65	92	73	98	65	83	93	85	80	97	84	76	92	89	81	99	93	100	94	105	71	99	78	89	84	87	85	93	74	119	92	85	87	90	114	119	117	105	105	103	95	111	102	106	127	113	81	92	94	76
106	144	100	98	99	101	96	104	93	86	106	98	102	112	107	86	113	112	69	83	85	109	90	97	90	106	98	97	96	94	73	99	80	98	101	105	113	112	99	82	96	98	100	89	107	97	131	91	90	89	101	94
85	144	93	65	107	102	88	94	110	97	96	82	82	92	102	93	89	93	96	80	87	104	79	110	80	89	91	99	92	91	81	114	80	81	69	72	110	118	114	106	107	84	89	112	113	121	133	106	90	70	102	94
91	138	100	76	112	103	100	107	98	108	94	70	97	113	92	87	110	75	85	70	77	93	100	102	84	95	117	72	83	94	76	85	80	82	81	80	111	99	106	117	95	84	99	114	117	112	134	91	94	77	101	96
92	150	102	110	105	92	95	98	101	107	78	101	107	90	96	98	95	109	108	98	98	106	98	86	96	95	98	94	99	91	103	122	113	105	112	81	133	124	126	128	67	103	114	120	111	110	134	112	109	109	90	96
73	141	94	93	85	82	82	110	116	90	88	105	84	99	93	106	81	102	113	112	100	104	94	107	100	95	110	103	82	94	106	127	104	113	108	87	130	112	121	102	92	103	96	107	99	104	124	109	97	110	93	101
144	115	138	134	138	140	143	131	143	128	139	130	134	141	131	141	137	126	139	135	136	139	140	148	131	133	140	143	142	148	139	123	123	137	130	146	123	135	127	133	141	134	119	125	128	135	114	126	137	135	145	144
107	144	108	109	114	103	106	97	113	121	101	103	105	106	113	108	116	109	95	90	91	99	112	95	99	95	99	95	106	113	78	115	97	110	110	91	126	115	111	107	72	111	118	111	104	104	129	98	104	113	94	95
82	150	98	72	100	108	96	99	85	98	103	73	94	96	101	93	104	89	92	88	66	111	99	108	98	104	113	101	87	89	81	110	81	80	77	111	119	106	115	94	104	93	92	100	111	110	128	93	99	92	94	97
102	146	94	91	104	102	96	98	94	97	105	104	110	115	106	83	110	107	74	87	99	104	93	94	95	102	101	95	95	103	83	103	99	97	100	100	120	109	105	76	89	92	108	85	99	92	129	92	103	99	103	97
84	142	107	87	103	93	87	103	94	107	91	101	103	103	91	92	98	107	98	96	86	87	88	92	87	94	100	81	93	81	85	105	97	106	106	79	116	117	104	101	79	96	114	118	111	92	141	112	99	103	92	94
97	143	113	102	100	95	99	104	108	105	100	101	99	111	107	95	108	104	94	86	85	102	94	98	87	90	100	86	95	104	73	113	99	108	112	80	126	118	109	101	85	99	112	116	106	99	135	98	96	104	101	101
117	126	112	114	112	117	110	117	128	109	122	111	115	120	121	113	110	112	106	108	123	111	117	126	100	108	111	120	119	116	115	115	103	125	104	116	109	115	108	118	119	102	97	93	98	118	117	100	92	113	113	109
83	139	98	94	97	81	99	112	108	94	88	111	90	94	96	107	92	106	110	109	100	112	105	103	115	106	105	103	85	88	110	131	108	125	114	95	128	117	125	108	88	104	111	109	108	98	132	108	100	113	98	98
92	142	113	87	107	85	107	88	92	117	95	114	93	103	97	93	102	121	103	104	89	92	84	87	104	104	96	95	101	96	93	118	119	109	109	83	120	117	111	89	75	111	123	120	120	86	136	114	103	104	98	94
87	140	96	61	94	97	93	92	102	91	101	82	77	97	100	89	89	94	94	90	83	106	90	105	95	90	98	104	78	86	95	117	92	96	66	84	110	101	115	96	96	93	86	97	112	110	136	93	87	83	97	87
103	144	91	92	99	101	84	84	92	95	96	98	68	98	82	95	100	102	91	94	91	110	95	108	99	89	85	102	93	109	79	117	97	93	87	96	109	106	114	90	89	104	97	109	104	91	123	96	85	93	93	82
97	133	92	95	91	107	92	103	104	84	96	84	102	104	100	93	99	95	70	101	99	121	94	108	93	99	101	102	90	104	85	89	82	86	103	94	116	114	100	104	107	110	88	111	111	117	133	99	96	91	99	98
76	141	103	69	97	87	92	80	95	93	87	96	83	93	86	94	78	93	95	77	104	112	90	96	98	97	98	106	95	89	91	113	104	103	75	94	108	113	109	92	101	91	81	113	114	105	134	94	98	86	100	88
107	141	84	86	103	104	104	102	92	92	110	90	108	115	103	80	112	106	67	86	90	102	86	98	95	93	101	91	86	92	84	96	87	93	98	100	106	101	94	77	95	97	104	88	108	96	132	96	86	88	96	90
112	146	111	106	108	103	99	95	103	106	105	98	92	91	101	88	107	109	104	98	90	103	97	87	100	94	79	91	98	90	81	116	97	103	110	102	119	121	111	105	98	107	116	117	124	103	126	93	85	106	99	104
103	144	91	92	99	101	84	84	92	95	96	98	68	98	82	95	100	102	91	94	91	110	95	108	99	89	85	102	93	109	79	117	97	93	87	96	109	106	114	90	89	104	97	109	104	91	123	96	85	93	93	82
121	138	128	129	117	133	134	121	124	107	123	128	127	114	118	123	118	118	119	120	135	127	127	132	126	133	116	125	129	125	130	118	104	123	115	138	128	118	121	117	125	118	118	116	111	122	125	120	120	106	128	117
128	108	128	134	133	135	130	127	140	125	136	124	121	132	130	132	131	115	119	112	135	131	138	139	139	129	134	141	132	133	130	119	121	136	119	140	122	122	123	108	126	109	107	109	101	121	95	91	120	124	135	128
85	143	85	96	98	106	100	99	100	93	107	76	91	107	105	92	95	88	107	70	105	121	105	115	114	101	116	108	86	105	88	108	95	79	93	111	118	106	120	88	106	57	62	89	71	109	126	80	105	77	109	100
86	148	98	85	77	90	93	85	101	93	77	107	91	65	79	92	91	111	102	108	86	98	79	91	85	77	72	99	93	96	97	130	106	94	98	86	125	119	124	111	92	121	109	117	117	113	139	114	66	106	64	58
129	112	125	133	131	140	126	133	137	124	131	123	133	137	132	128	133	122	111	118	123	128	134	139	120	133	138	125	129	129	121	104	109	120	125	133	120	125	105	118	133	125	112	125	118	131	105	106	125	122	130	126
87	136	104	86	85	84	96	74	101	89	85	99	70	73	87	91	72	102	81	97	87	95	74	96	66	64	77	99	97	102	93	111	94	96	88	88	110	112	113	110	96	106	100	124	118	111	121	95	68	104	86	73
77	147	90	85	86	102	97	96	86	91	88	78	93	83	101	74	100	94	95	95	79	99	90	93	82	91	89	83	64	81	73	107	96	85	99	82	119	110	107	115	94	99	93	117	105	114	134	90	86	90	94	105
88	141	94	92	83	90	54	67	100	88	81	90	67	78	76	91	87	98	102	100	83	89	81	91	73	66	95	84	73	90	86	121	97	96	100	81	116	115	119	105	83	100	104	112	109	107	128	100	74	107	65	83
94	131	109	109	92	90	93	97	117	108	91	120	104	104	101	112	93	119	111	113	110	109	98	96	97	92	98	110	100	102	114	128	122	120	123	96	135	128	128	114	84	113	116	119	120	118	132	129	87	118	75	85
125	113	123	134	132	135	125	122	128	119	132	108	124	132	124	128	126	111	116	106	124	121	130	133	122	132	132	127	128	126	123	107	104	126	114	131	120	114	103	115	119	105	100	105	93	113	69	83	125	115	123	126
89	143	94	90	100	86	81	72	84	103	76	100	82	85	71	96	99	109	94	98	86	102	80	87	91	81	91	93	81	94	87	124	106	95	93	80	118	112	112	88	79	107	107	109	111	83	130	100	81	101	75	85
107	149	107	95	115	109	96	100	86	104	104	90	96	99	90	88	121	104	91	85	80	100	104	95	89	96	93	74	101	97	73	106	91	80	93	88	118	101	109	96	97	104	113	116	110	100	134	96	99	90	99	94
72	138	96	72	82	97	93	80	97	77	92	97	66	72	97	89	58	100	85	89	103	100	79	107	96	91	86	96	88	85	96	116	92	96	81	90	108	119	110	98	104	91	96	113	110	110	134	89	85	88	101	82
142	135	142	144	143	138	147	145	152	149	141	148	138	148	140	150	144	148	149	143	144	147	148	154	140	141	145	147	148	149	144	146	151	150	142	141	143	150	143	149	143	146	136	140	135	146	129	143	141	145	150	142
86	150	76	81	98	89	66	94	81	96	82	68	102	90	92	75	101	76	101	89	73	110	87	92	89	96	112	74	67	75	74	114	96	60	85	86	117	106	111	104	93	68	92	108	105	109	135	110	103	83	85	92
82	140	85	89	88	111	100	89	81	96	97	73	78	82	104	67	94	95	105	105	79	102	90	98	96	94	91	98	75	88	78	114	96	69	86	96	117	116	107	104	107	99	93	118	113	112	124	105	91	79	100	106
82	146	85	97	74	116	96	98	83	79	107	79	97	83	109	68	108	95	95	107	88	102	99	100	99	95	91	76	72	84	87	109	96	86	88	85	122	115	110	113	104	102	104	109	108	113	135	100	86	88	104	103

94	136	94	100	105	107	94	80	110	94	100	88	98	94	108	94	89	89	89	91	112	123	72	99	101	101	96	106	101	92	93	98	92	95	99	89	119	124	109	107	95	88	83	94	112	113	124	106	97	97	97	110
93	142	107	84	101	113	96	94	86	90	102	78	86	97	89	86	107	83	87	76	96	101	103	114	105	95	98	83	82	96	82	90	81	84	76	99	102	103	104	92	109	87	101	97	104	105	129	76	89	70	106	92
92	143	98	104	86	86	92	94	106	100	80	113	103	91	85	104	94	122	111	118	104	114	85	98	106	100	82	101	104	90	110	134	116	106	115	83	124	128	115	118	87	118	114	121	110	108	140	124	89	100	83	87
86	140	87	79	91	82	64	89	101	91	70	96	80	95	75	93	90	99	108	106	93	97	87	99	85	78	93	100	70	101	91	122	109	99	102	85	115	113	120	103	90	113	103	105	106	100	131	107	80	103	89	81
105	141	122	78	107	114	109	94	96	108	100	98	90	96	93	89	104	94	101	90	91	90	106	99	89	97	98	96	95	89	97	108	107	102	93	92	104	101	107	104	92	95	107	114	121	116	138	93	104	103	95	110
82	147	98	77	93	96	87	98	91	106	106	104	85	99	103	77	106	111	105	97	92	86	100	93	90	106	103	80	80	73	91	116	110	95	99	95	123	106	116	92	103	101	105	120	114	91	140	107	95	89	102	101
142	126	120	136	131	136	127	121	137	118	129	116	125	127	126	138	130	126	111	115	115	122	139	131	123	116	133	128	132	131	122	123	101	127	117	128	106	113	101	131	123	125	123	122	118	136	111	108	118	133	125	125
92	143	82	88	102	103	88	92	95	77	101	68	99	91	103	81	89	82	78	71	90	105	79	95	82	91	93	79	94	81	73	95	71	78	70	92	111	111	101	97	103	83	94	95	104	110	134	84	92	85	92	90
77	144	106	64	103	99	97	87	84	94	85	80	77	99	78	90	95	90	96	88	87	98	87	107	101	95	106	92	91	91	93	104	94	74	93	87	111	114	103	93	105	89	99	98	105	101	130	91	99	78	103	104
110	131	112	109	121	119	120	133	109	124	109	109	119	121	118	117	123	110	119	111	117	110	119	113	110	108	125	100	109	113	112	120	117	120	117	113	130	131	128	116	116	112	107	114	106	113	101	116	117	119	122	114
88	139	89	106	95	106	95	102	90	101	85	82	110	101	95	91	107	98	93	99	67	107	96	91	88	91	104	71	74	86	94	109	95	96	111	86	118	107	103	112	71	97	99	103	96	101	136	97	96	92	83	98
92	134	107	98	110	107	94	94	96	97	92	71	94	100	75	90	110	91	108	82	85	100	101	110	93	93	97	75	84	91	87	106	88	90	82	87	106	108	114	108	97	91	105	114	111	102	129	85	92	74	105	96
94	141	91	88	89	80	73	84	101	94	83	94	91	72	83	86	95	108	91	100	88	101	79	78	84	67	75	93	82	98	89	121	100	100	100	83	127	121	124	110	70	103	99	114	109	110	138	117	67	109	30	51
93	137	84	83	83	91	83	91	109	93	96	84	75	95	94	89	93	90	89	89	89	100	87	99	84	76	93	93	84	110	79	114	90	94	88	87	113	110	111	109	95	100	92	98	104	108	125	104	52	99	74	56
88	131	85	87	82	78	87	99	100	102	84	98	88	96	84	94	91	109	100	101	93	105	85	97	92	66	90	96	80	111	86	122	105	98	92	85	115	115	117	106	95	111	103	114	106	107	128	119	71	104	72	54
109	142	89	117	102	120	104	108	113	105	112	103	120	107	119	106	125	122	103	113	99	116	113	112	103	112	124	102	107	101	99	119	100	96	97	108	100	101	86	108	110	112	111	126	111	115	128	111	101	108	108	109
105	140	103	93	110	107	101	89	97	111	101	97	94	91	98	82	114	102	92	99	75	99	98	85	91	87	89	84	91	93	85	104	102	90	106	84	121	108	110	100	86	109	117	114	119	110	136	107	90	100	87	88
0	145	94	72	82	91	96	109	83	98	82	100	84	84	93	89	71	101	105	97	86	102	77	106	92	102	101	87	79	83	100	111	103	93	89	78	127	125	121	102	96	96	96	112	104	102	141	107	98	71	101	84
145	0	142	151	141	144	138	145	151	138	141	140	141	144	140	144	137	136	139	144	138	145	146	143	137	132	145	146	143	143	147	133	141	141	142	144	125	141	132	142	146	144	135	135	132	146	89	135	137	148	138	146
94	142	0	102	93	115	92	100	91	86	96	89	103	94	86	83	105	104	102	102	93	116	92	90	103	97	111	95	75	91	77	117	101	83	96	97	124	114	119	103	98	94	97	101	106	96	129	117	94	91	99	92
72	151	102	0	90	84	98	93	88	99	84	90	75	91	88	76	83	90	92	83	92	86	77	101	91	92	98	85	70	84	88	108	100	73	68	76	113	111	116	95	100	94	96	113	122	100	144	104	88	73	100	88
82	141	93	90	0	95	87	98	100	71	94	113	87	83	104	84	78	115	103	117	102	98	90	117	97	95	89	100	93	88	94	129	109	107	99	91	122	121	113	110	113	114	105	122	113	107	134	112	72	97	106	89
91	144	115	84	95	0	88	105	106	101	80	114	94	99	89	95	91	115	114	100	107	89	86	100	86	94	98	97	87	90	96	129	114	107	104	91	119	120	122	102	97	105	107	121	109	94	138	118	96	112	90	85
96	138	92	98	87	88	0	86	111	84	86	89	83	100	86	101	96	96	104	100	97	97	96	99	83	82	90	106	86	97	81	120	90	91	103	93	112	119	117	100	97	92	101	98	101	107	128	107	93	97	79	92
109	145	100	93	98	105	86	0	109	100	86	93	76	83	86	103	95	98	85	99	96	109	90	90	98	81	88	112	102	112	93	112	98	82	92	96	110	113	109	97	86	97	104	110	119	106	131	104	91	93	86	94
83	151	91	88	100	106	111	109	0	107	91	97	105	87	92	65	107	100	100	100	99	102	76	74	101	99	104	76	83	70	80	106	114	81	92	103	121	114	115	96	101	103	113	119	118	83	142	115	110	89	101	101
98	138	86	99	71	101	84	100	107	0	111	100	97	81	106	87	74	101	95	99	105	110	94	105	102	100	86	105	93	94	94	117	87	101	86	98	112	116	108	102	107	99	99	102	105	113	135	95	82	92	105	98
82	141	96	84	94	80	86	86	91	111	0	92	93	86	53	101	80	98	102	100	92	111	87	90	91	82	95	95	82	87	89	130	108	88	108	84	124	122	121	113	90	110	108	109	111	104	133	122	104	98	89	93
100	140	89	90	113	114	89	93	97	100	92	0	99	108	90	87	112	51	97	78	73	112	97	104	102	85	112	87	66	99	77	99	79	63	91	87	111	107	104	109	97	75	86	84	106	120	131	89	96	79	94	104
84	141	103	75	87	94	83	76	105	97	93	99	0	77	86	93	77	97	97	100	96	89	90	110	101	75	82	98	85	97	86	121	104	105	99	84	118	116	118	100	95	101	97	111	108	102	127	95	71	96	97	89
84	144	94	91	83	99	100	83	87	81	86	108	77	0	95	74	75	105	93	112	101	108	75	89	93	88	70	94	90	82	99	129	100	96	87	91	131	130	129	113	91	112	108	132	120	116	136	103	87	115	77	83
93	140	86	88	104	89	86	86	92	106	53	90	86	95	0	103	90	98	102	98	93	93	89	101	91	80	87	97	78	96	96	118	104	92	100	94	115	111	120	105	89	112	109	106	116	99	127	106	101	96	92	91
89	144	83	76	84	95	101	103	65	87	101	87	93	74	103	0	100	88	98	89	93	94	58	74	87	90	86	87	60	64	64	98	102	78	83	91	125	117	119	93	108	88	98	111	122	106	139	111	82	86	98	101
71	137	105	83	78	91	96	95	107	74	80	112	77	75	90	100	0	113	106	107	100	114	82	108	104	95	86	112	98	91	108	128	101	106	97	84	121	121	123	110	99	104	99	119	115	117	131	110	97	100	98	89
101	136	104	90	115	115	96	98	100	101	98	51	97	105	98	88	113	0	90	87	97	119	96	112	103	98	113	99	77	105	78	92	92	91	89	99	118	114	114	114	107	78	88	87	104	115	126	104	105	89	108	108
105	139	102	92	103	114	104	85	100	95	102	97	97	93	102	98	106	90	0	87	108	112	90	100	96	93	92	98	105	111	94	90	75	102	89	92	106	114	101	97	101	108	108	108	114	110	130	93	88	100	91	91
97	144	102	83	117	100	100	99	100	99	100	78	100	112	98	89	107	87	87	0	94	98	101	96	89	98	107	74	94	96	72	89	79	85	82	85	106	102	100	86	92	53	86	95	94	96	127	68	96	78	103	97
86	138	93	92	102	107	97	96	99	105	92	73	96	101	93	93	100	97	108	94	0	106	101	93	82	80	108	86	83	84	78	120	96	87	105	82	120	106	112	109	84	99	103	112	118	115	127	106	99	94	89	98
102	145	116	86	98	89	97	109	102	110	111	112	89	108	93	94	114	119	112	98	106	0	99	105	73	98	100	76	98	96	106	110	103	111	99	91	112	109	108	117	95	118	134	124	123	101	132	102	85	98	103	98
77	146	92	77	90	86	96	90	76	94	87	97	90	75	89	58	82	96	90	101	101	99	0	93	83	88	83	93	85	80	85	105	100	84	93	82	124	132	121	98	101	103	104	116	119	98	137	121	83	91	87	86
106	143	90	101	117	100	99	90	74	105	90	104	110	89	101	74	108	112	100	96	93	105	93	0	97	83	91	99	88	86	94	117	106	97	110	98	130	120	124	104	90	105	116	118	121	104	135	124	101	115	76	101
92	137	103	91	97	86	83	98	101	102	91	102	101	93	91	87	104	103	96	89	82	73	83	97	0	80	93	85	97	87	79	108	90	95	87	85	115	111	114	113	97	110	106	123	113	115	124	107	87	102	79	80
102	132	97	92	95	94	82	81	99	100	82	85	75	88	80	90	95	98	93	98	80	98	88	83	80	0	75	96	86	104	87	116	102	95	105	82	122	110	119	111	87	103	106	115	115	123	122	111	75	113	68	85
101	145	111	98	89	98	90	88	104	86	95	112	82	70	87	86	86	113	92	107	108	100	83	91	93	75	0	103	103	101	91	125	105	111	100	91	121	129	122	112	95	117	118	117	118	113	135	113	66	98	91	84
87	146	95	85	100	97	106	112	76	105	95	87	98	94	97	87	112	99	98	74	86	76	93	99	85	96	103	0	79	70	84	104	96	91	91	70	114	108	101	118	85	87	116	121	109	96	146	99	91	77	100	92
79	143	75	70	93	87	86	102	83	93	82	66	85	90	78	60	98	77	105	94	83	98	85	88	97	86	103	79	0	82	76	112	100	85	92	84	120	108	119	95	83	89	99	91	104	101	140	104	85	94	89	91
83	143	91	84	88	90	97	112	70	94	87	99	97	82	96	64	91	105	111	96	84	96	80	86	87	104	101	70	82	0	81	117	111	96	88	86	125	115	115	105	103	91	100	122	116	98	136	105	93	88	104	111
100	147	77	88	94	96	81	93	80	94	89	77	86	99	96	64	108	78	94	72	78	106	85	94	79	87	91	84	76	81	0	102	86	82	88	94	109	115	103	92	107	84	93	107	104	97	132	103	77	81	102	90
111	133	117	108	129	129	120	112	106	117	130	99	121	129	118	98	128	92	90	89	120	110	105	117	108	116	125	104	112	117	102	0	79	102	107	114	112	117	99	114	114	103	103	107	131	119	124	99	113	94	120	119
103	141	101	100	109	114	90	98	114	87	108	79	104	100	104	102	101	92	75	79	96	103	100	106	90	102	105	96	100	111	86	79	0	98	84	104	110	105	92	101	103	95	104	99	108	106	122	82	97	86	94	99
93	141	83	73	107	107	91	82	81	101	88	63	105	96	92	78	106	91	102	85	87	111	84	97	95	95	111	91	85	96	82	102	98	0	92	93	113	104	114	91	106	81	93	117	115	112	135	111	110	65	99	107
89	142	96	68	99	104	103	92	92	86	108	91	99	87	100	83	97	89	89	82	105	99	93	110	87	105	100	91	92	88	88	107	84	92	0	92	110	112	111	99	107	91	97	114	118	110	130	93	90	80	103	82
78	144	97	76	91	91	93	96	103	98	84	87	84	91	94	91	84	99	92	85	82	91	82	98	85	82	91	70	84	86	94	114	104	93	92	0	121	116	108	114	76	102	108	116	111	111	141	111	88	87	92	99
127	125	124	113	122	119	112	110	121	112	124	111	118	131	115	125	121	118	106	106	120	112	124	130	115	122	121	114	120	125	109	112	110	113	110	121	0	80	70	113	123	112	119	123	121	124	133	105	110	105	125	115
125	141	114	111	121	120	119	113	114	116	122	107	116	130	111	117	121	114	114	102	106	109	132	120	111	110	129	108	108	115	115	117	105	104	112	116	80	0	87	97	109	98	108	112	108	113	128	95	119	109	115	120
121	132	119	116	113	122	117	109	115	108	121	104	118	129	120	119	123	114	101	100	112	108	121	124	114	119	122	101	119	115	103	99	92	114	111	108	70	87	0	112	113	114	117	114	112	114	125	92	104	102	118	119
102	142	103	95	110	102	100	97	96	102	113	109	100	113	105	93	110	114	97	86	109	117	98	104	113	111	112	118	95	105	92	114	101	91	99	114	113	97	112	0	117	92	98	101	94	67	127	97	113	99	114	106
96	146	98	100	113	97	97	86	101	107	90	97	95	91	89	108	99	107	101	92	84	95	101	90	97	87	95	85	83	103	107	114	103	106	107	76	123	109	113	117	0	96	113	106	103	100	136	94	102	112	68	80
96	144	94	94	114	105	92	97	103	99	110	75	101	112	112	88	104	78	108	53	99	118	103	105	110	103	117	87	89	91	84	103	95	81	91	102	112	98	114	92	96	0	84	94	86	105	129	85	103	79	101	108
96	135	97	96	105	107	101	104	113	99	108	86	97	108	109	98	99	88	108	86	103	134	104	116	106	106	118	116	99	100	93	103	104	93	97	108	119	108	117	98	113	84	0	98	81	127	113	86	100	99	107	107
112	135	101	113	122	121	98	110	119	102	109	84	111	132	106	111	119	87	108	95	112	124	116	118	123	115	117	121	91	122	107	107	99	117	114	116	123	112	114	101	106	94	98	0	82	97	120	96	114	100	116	118
104	132	106	122	113	109	101	119	118	105	111	106	108	120	116	122	115	104	114	94	118	123	119	121	113	115	118	109	104	116	104	131	108	115	118	111	121	108	112	94	103	86	81	82	0	98	113	85	116	114	107	109
102	146	96	100	107	94	107	106	83	113	104	120	102	116	99	106	117	115	110	96	115	101	98	104	115	123	113	96	101	98	97	119	106	112	110	111	124	113	114	67	100	105	127	97	98	0	128	100	112	105	121	106
141	89	129	144	134	138	128	131	142	135	133	131	127	136	127	139	131	126	130	127	127	132	137	135	124	122	135	146	140	136	132	124	122	135	130	141	133	128	125	127	136	129	113	120	113	128	0	113	130	142	129	138
107	135	117	104	112	118	107	104	115	95	122	89	95	103	106	111	110	104	93	68	106	102	121	124	107	111	113	99	104	105	103	99	82	111	93	111	105	95	92	97	94	85	86	96	85	100	113	0	95	104	110	106
98	137	94	88	72	96	93	91	110	82	104	96	71	87	101	82	97	105	88	96	99	85	83	101	87	75	66	91	85	93	77	113	97	110	90	88	110	119	104	113	102	103	100	114	116	112	130	95	0	95	79	69
71	148	91	73	97	112	97	93	89	92	98	79	96	115	96	86	100	89	100	78	94	98	91	115	102	113	98	77	94	88	81	94	86	65	80	87	105	109	102	99	112	79	99	100	114	105	142	104	95	0	121	97
101	138	99	100	106	90	79	86	101	105	89	94	97	77	92	98	98	108	91	103	89	103	87	76	79	68	91	100	89	104	102	120	94	99	103	92	125	115	118	114	68	101	107	116	107	121	129	110	79	121	0	54
84	146	92	88	89	85	92	94	101	98	93	104	89	83	91	101	89	108	91	97	98	98	86	101	80	85	84	92	91	111	90	119	99	107	82	99	115	120	119	106	80	108	107	118	109	106	138	106	69	97	54	0
66	142	91	79	78	92	97	98	83	97	76	89	94	86	93	76	88	100	103	110	90	102	86	96	91	101	102	78	76	76	92	117	103	86	95	84	127	124	114	112	101	110	89	116	106	111	138	110	100	84	101	103
81	134	93	93	89	99	85	100	89	100	89	72	103	100	94	87	108	88	84	90	71	104	84	100	88	91	99	72	82	77	76	95	86	85	101	80	115	117	106	114	98	98	93	106	106	113	127	94	81	81	88	85
85	150	104	86	93	80	77	88	84	100	80	88	89	90	81	92	99	97	85	91	97	103	83	100	83	95	88	80	89	91	79	103	87	75	87	85	106	112	109	101	87	103	105	113	100	93	142	96	100	85	89	84
83	137	109	59	95	84	82	84	107	98	85	89	70	103	83	89	90	102	96	83	94	93	84	109	92	88	93	96	88	91	84	112	99	80	95	81	109	118	111	90	107	96	92	105	110	98	131	92	77	83	98	87
104	147	109	85	99	102	96	93	86	92	113	88	90	102	102	81	110	81	89	75	99	94	100	110	91	102	103	79	88	88	76	85	86	95	84	95	104	98	100	103	97	83	95	114	110	104	132	85	91	84	107	99
127	119	122	131	124	143	133	128	129	118	132	116	123	128	123	129	124	107	111	116	117	136	121	136	122	116	126	119	131	127	125	102	104	129	117	137	120	127	123	130	130	117	99	119	114	135	97	114	114	110	129	128

89	144	92	97	102	93	106	108	82	104	89	96	97	92	91	94	99	109	105	96	90	92	92	94	80	95	108	78	95	71	97	115	105	94	107	88	122	109	115	116	85	101	115	129	114	101	134	110	97	96	88	89
103	144	110	92	104	90	91	100	91	109	90	105	93	101	77	93	100	110	100	105	84	99	93	95	100	92	90	91	94	89	89	120	112	103	120	88	117	106	99	96	84	111	112	112	116	87	124	101	98	115	95	108
132	120	140	130	134	136	137	132	138	128	131	126	129	139	137	138	130	126	126	125	131	138	126	143	122	132	135	129	140	135	134	106	113	129	124	136	126	128	120	130	137	131	119	121	115	129	102	114	128	123	136	136
132	115	131	133	135	138	133	133	139	129	139	120	128	140	126	138	127	123	123	123	133	127	134	140	126	127	135	128	133	137	138	102	104	136	119	136	119	121	117	131	130	124	112	114	112	130	94	104	128	126	137	136
78	140	92	67	89	82	100	102	91	94	87	88	85	92	83	78	84	96	96	89	83	86	94	92	91	88	107	85	70	77	90	111	94	97	81	80	119	102	105	109	90	99	92	115	111	103	131	96	91	95	86	93
93	144	99	90	91	101	90	107	80	103	92	90	95	103	91	89	112	96	96	97	96	100	93	111	95	99	97	75	79	83	80	101	93	87	96	101	103	113	109	101	111	98	102	110	102	102	140	99	93	75	106	93
112	143	100	113	107	110	101	115	105	109	98	98	107	110	100	107	112	111	110	97	98	111	105	112	102	100	112	84	91	103	94	108	112	93	121	98	114	109	113	115	104	102	107	122	106	114	137	102	100	104	93	101
104	147	103	84	109	111	97	99	114	104	105	77	94	114	99	108	114	94	91	85	84	105	111	111	92	92	113	97	91	109	98	112	81	91	98	91	99	96	100	108	89	100	102	97	109	113	137	92	100	88	93	94
104	146	94	98	114	103	84	95	94	105	98	88	94	105	97	96	120	101	103	98	76	105	99	93	91	94	106	89	88	93	75	115	102	97	100	87	114	110	103	104	98	100	111	113	110	100	133	104	97	108	94	104
115	131	109	118	114	135	132	120	126	115	124	114	111	125	117	118	116	121	119	117	106	108	126	129	116	115	126	116	122	109	127	119	107	128	110	120	118	116	110	126	116	111	111	113	115	129	100	110	112	101	125	128
82	150	75	74	95	88	82	94	76	94	76	65	79	79	86	74	100	79	92	93	72	108	92	81	90	91	103	85	48	72	72	119	88	80	84	83	121	105	111	104	88	96	91	96	104	100	137	97	96	94	85	97
80	145	116	68	104	91	101	101	87	95	97	92	90	101	87	83	93	101	95	87	106	93	93	95	107	109	96	95	87	84	103	102	90	96	78	91	107	108	107	93	100	103	107	106	116	92	139	94	96	81	112	104
93	146	97	83	109	86	93	95	82	106	70	88	92	76	80	82	99	97	111	93	89	106	77	83	90	87	83	89	77	92	70	119	115	74	105	86	117	126	126	107	89	103	107	126	114	103	143	118	93	102	88	86

1	1	1	1	1	1	1	1	1	1	1	1	1	1	1	1	1	1	1
Strain 67	Strain 68	Strain 69	Strain 70	Strain 71	Strain 72	Strain 73	Strain 74	Strain 75	Strain 76	Strain 77	Strain 78	Strain 79	Strain 80	Strain 81	Strain 82	Strain 83	Strain 84	Strain 85
C067	C068	C069	C070	C071	C072	C073	C074	C075	C076	C077	C078	C079	C080	C081	C082	C083	C084	C085
3	0	0	0	1	0	0	0	1	0	0	2	0	0	0	0	1	0	0
115	110	121	129	113	89	129	121	105	98	115	112	120	117	109	91	112	122	136
137	136	141	137	135	110	139	145	115	115	135	143	138	137	136	115	137	142	139
123	123	123	123	124	118	126	123	118	113	119	122	129	112	116	101	118	126	142
141	136	139	134	134	115	144	137	112	111	139	143	135	141	135	128	142	138	138
111	115	107	104	122	132	93	103	138	140	101	124	121	115	110	127	110	106	94
133	132	129	138	131	136	130	128	139	133	138	131	127	137	131	134	130	147	133
89	89	106	104	88	118	113	116	130	117	81	94	114	91	95	103	78	97	109
113	116	119	127	117	126	115	126	126	133	101	106	118	104	118	117	107	130	123
107	110	99	91	105	129	83	83	140	142	90	115	110	115	96	129	108	102	85
107	110	99	91	105	129	83	83	140	142	90	115	110	115	96	129	108	102	85
107	110	99	91	105	129	83	83	140	142	90	115	110	115	96	129	108	102	85
107	110	99	91	105	129	83	83	140	142	90	115	110	115	96	129	108	102	85
111	115	100	106	115	140	100	97	143	140	115	120	121	127	114	134	114	101	86
103	104	92	76	103	127	89	84	134	136	90	106	111	101	107	124	105	90	92
126	122	120	128	117	117	129	126	122	117	127	122	133	111	125	112	125	130	140
97	107	85	88	108	144	85	76	139	143	96	107	113	116	84	132	91	91	71
97	107	85	88	108	144	85	76	139	143	96	107	113	116	84	132	91	91	71
146	134	150	142	147	118	145	142	120	119	143	144	141	146	149	131	149	150	145
110	124	117	121	131	140	107	112	139	140	113	123	125	124	115	130	107	117	117
122	132	122	121	130	135	127	119	133	134	130	128	132	124	134	130	129	129	121
119	108	105	98	103	113	116	114	119	117	113	120	118	101	106	109	105	103	117
103	117	95	99	121	143	99	105	144	140	104	116	124	122	111	132	102	106	89
121	124	123	122	127	139	121	116	141	139	113	122	131	108	123	130	117	117	128
137	127	130	125	122	85	133	124	108	94	127	122	126	117	128	88	130	124	138
137	127	130	125	122	85	133	124	108	94	127	122	126	117	128	88	130	124	138
137	127	130	125	122	85	133	124	108	94	127	122	126	117	128	88	130	124	138
133	139	134	123	137	150	132	131	150	150	133	139	135	139	139	144	134	136	130
121	115	127	123	115	99	126	122	103	93	118	125	126	113	125	87	121	117	140
122	122	119	125	126	142	125	125	139	146	120	126	119	122	122	131	123	137	123
111	128	121	124	128	132	128	129	130	139	113	123	130	128	134	115	116	127	129
99	101	84	93	116	145	97	77	144	148	103	104	102	120	103	138	105	101	86
140	136	147	139	145	118	139	145	115	111	140	144	140	140	148	128	143	140	142
126	125	123	131	121	114	119	124	111	109	125	126	124	123	125	112	122	130	129
111	106	112	108	113	108	109	113	119	119	100	107	109	105	116	104	104	120	118
120	128	133	136	128	84	131	141	105	97	126	125	125	124	130	78	126	128	132
124	118	122	130	112	124	127	123	127	124	111	115	121	94	124	106	117	122	139
124	118	122	130	112	124	127	123	127	124	111	115	121	94	124	106	117	122	139
124	118	122	130	112	124	127	123	127	124	111	115	121	94	124	106	117	122	139
124	118	122	130	112	125	127	123	127	125	111	115	121	94	124	107	117	122	139
124	118	122	130	112	124	127	123	127	124	111	115	121	94	124	106	117	122	139
139	145	142	139	138	142	135	134	147	139	136	145	140	143	139	137	140	138	131
132	138	142	137	131	134	135	139	128	131	133	137	135	129	140	131	137	133	139
121	110	120	127	116	98	121	124	114	107	122	116	125	114	107	100	110	114	121
121	109	119	126	115	97	120	124	114	107	121	116	124	113	106	99	108	113	121
125	128	118	129	124	138	129	138	134	141	126	121	130	130	127	130	118	133	127
125	133	133	120	126	127	127	125	131	131	117	131	135	120	136	127	129	126	135
112	121	116	129	126	138	125	126	133	143	123	125	124	122	123	131	129	136	123
128	127	126	125	126	144	126	123	148	142	121	126	131	101	120	123	118	124	132
121	112	134	132	123	105	132	132	106	112	122	125	126	128	132	100	124	130	138
126	111	127	113	112	114	116	116	124	119	105	115	118	93	118	111	112	119	118
135	126	133	128	132	96	121	130	106	109	131	128	130	125	132	101	134	141	132
117	116	119	105	118	132	109	120	136	128	100	114	123	97	124	130	108	108	119
135	135	136	135	131	130	136	137	130	136	139	130	130	124	127	128	135	136	134
107	108	103	87	109	127	103	102	125	122	81	117	117	99	118	116	98	97	116
123	112	129	123	124	100	120	130	110	103	113	128	126	118	138	103	124	122	139
115	110	122	130	115	91	129	122	105	99	115	113	121	118	111	93	113	123	136

95	114	99	110	125	138	91	104	141	139	95	115	115	112	108	129	104	107	97
95	114	99	110	125	138	91	104	141	139	95	115	115	112	109	129	105	107	97
95	114	99	110	125	138	91	104	141	139	95	115	115	112	108	129	104	107	97
95	114	99	110	125	138	91	104	141	139	95	115	115	112	108	129	104	107	97
95	114	99	110	125	138	91	104	141	139	95	115	115	112	108	129	104	107	97
95	114	99	110	125	138	91	104	141	139	95	115	115	112	108	129	104	107	97
95	114	99	110	125	138	91	104	141	139	95	115	115	112	108	129	104	107	97
95	114	99	110	125	138	91	104	141	139	95	115	115	112	108	129	104	107	97
95	114	99	110	125	138	91	104	141	139	95	115	115	112	108	129	104	107	97
95	114	99	110	125	138	91	104	141	139	95	115	115	112	108	129	104	107	97
95	114	99	110	125	138	91	104	141	139	95	115	115	112	108	129	104	107	97
95	114	99	110	125	138	91	104	141	139	95	115	115	112	108	129	104	107	97
95	114	99	110	125	138	91	104	141	139	95	115	115	112	108	129	104	107	97
95	114	99	110	125	138	91	104	141	139	95	115	115	112	108	129	104	107	97
95	114	99	110	125	138	91	104	141	139	95	115	115	112	108	129	104	107	97
95	114	99	110	125	138	91	104	141	139	95	115	115	112	108	129	104	107	97
122	122	119	125	126	142	125	125	139	146	120	126	119	122	122	131	123	137	123
106	108	106	101	117	143	103	93	147	148	100	121	114	121	99	130	102	107	96
128	129	117	129	124	138	119	119	145	137	122	121	124	118	121	127	125	125	119
126	129	118	129	125	139	118	118	146	138	120	121	125	119	119	128	123	126	118
122	112	121	125	123	117	120	124	125	119	117	117	120	116	128	107	119	124	129
130	135	128	129	137	144	132	132	144	144	135	136	133	140	138	135	134	136	132
126	122	113	124	101	111	121	123	120	115	116	118	132	119	128	106	121	121	132
89	89	106	104	88	118	113	116	130	117	81	94	114	91	95	103	78	97	109
123	129	122	117	125	134	112	114	145	132	113	125	132	123	131	133	124	117	125
90	90	106	104	88	118	113	116	130	117	82	95	114	91	96	103	79	97	109
90	90	106	104	88	118	113	116	130	117	81	94	114	92	95	104	78	97	109
147	146	141	141	140	140	141	135	146	143	144	142	142	146	136	142	146	147	142
138	130	134	136	132	134	136	126	138	139	137	135	128	136	130	130	136	139	138
138	129	130	136	137	142	136	135	144	152	140	135	132	146	136	146	135	146	138
146	137	146	137	144	112	147	143	109	109	147	146	134	145	141	128	153	148	145
155	154	150	153	156	154	151	149	148	146	154	158	147	157	154	151	154	156	150
137	134	136	137	136	126	138	136	140	141	133	137	132	140	140	132	137	141	139
114	132	129	115	119	137	122	128	142	142	110	126	128	122	133	125	119	121	127
89	97	96	88	103	137	96	82	141	131	73	97	106	107	95	119	84	92	97
126	127	126	124	114	111	120	118	117	111	123	127	129	113	123	107	122	125	129
111	128	121	124	128	132	128	129	130	139	113	123	130	128	134	115	116	127	129
109	117	119	124	110	99	124	129	108	103	109	113	122	109	128	98	109	121	132
110	107	112	122	110	93	121	109	104	104	105	112	112	105	110	94	110	120	122
110	107	112	122	110	93	121	109	104	104	105	112	112	105	110	94	110	120	122
110	107	112	122	110	93	121	109	104	104	105	112	112	105	110	94	110	120	122
110	107	112	122	110	93	121	109	104	104	105	112	112	105	110	94	110	120	122
96	103	93	109	116	127	110	111	129	129	119	110	118	121	110	124	108	107	102
112	116	113	114	115	129	120	116	134	132	119	118	124	110	121	110	118	121	132
144	133	144	139	143	107	137	138	104	102	139	143	139	140	144	115	148	143	148
126	123	125	125	118	103	118	126	120	116	123	123	130	114	122	97	126	125	127
117	125	122	123	116	103	121	129	120	110	114	124	132	127	130	99	126	116	141
117	114	92	108	104	143	89	74	145	142	104	113	110	115	108	138	107	105	89
117	114	92	108	104	143	89	74	145	142	104	113	110	115	108	138	107	105	89
117	114	92	108	104	143	89	74	145	142	104	113	110	115	108	138	107	105	89
117	114	92	108	104	143	89	74	145	142	104	113	110	115	108	138	107	105	89
101	94	89	104	105	138	100	89	142	139	101	104	99	112	96	128	85	98	97
140	133	144	142	141	109	147	139	104	106	144	139	133	143	138	132	143	145	143
122	132	122	121	130	135	127	119	133	134	130	128	132	124	134	130	129	129	121
135	122	125	126	111	96	124	122	111	118	123	120	123	108	127	95	118	130	125
75	90	95	90	98	117	116	118	126	121	94	88	120	103	102	109	84	93	115
76	91	95	90	98	117	117	119	126	121	94	88	121	104	102	110	85	94	116
75	90	95	90	98	117	116	118	126	121	94	88	120	103	102	109	84	93	115
75	90	95	90	98	117	116	118	126	121	94	88	120	103	102	109	84	93	115
75	90	95	90	98	117	116	118	126	121	94	88	120	103	102	109	84	93	115
75	90	95	90	98	117	116	118	126	121	94	88	120	103	102	109	84	93	115
75	90	95	90	98	117	116	118	126	121	94	88	120	103	102	109	84	93	115
75	90	95	90	98	117	116	118	126	121	94	88	120	103	102	109	84	93	115
75	90	95	90	98	117	116	118	126	121	94	88	120	103	102	109	84	93	115
75	90	95	90	98	117	116	118	126	121	94	88	120	103	102	109	84	93	115
75	90	95	90	98	117	116	118	126	121	94	88	120	103	102	109	84	93	115
75	90	95	90	98	117	116	118	126	121	94	88	120	103	102	109	84	93	115
75	90	95	90	98	117	116	118	126	121	94	88	120	103	102	109	84	93	115
75	90	95	90	98	117	116	118	126	121	94	88	120	103	102	109	84	93	115
75	90	95	90	98	117	116	118	126	121	94	88	120	103	102	109	84	93	115
75	90	95	90	98	117	116	118	126	121	94	88	120	103	102	109	84	93	115
75	90	95	90	98	117	116	118	126	121	94	88	120	103	102	109	84	93	115
75	90	95	90	98	117	116	118	126	121	94	88	120	103	102	109	84	93	115
75	90	95	90	98	117	116	118	126	121	94	88	120	103	102	109	84	93	115
75	90	95	90	98	117	116	118	126	121	94	88	120	103	102	109	84	93	115

75	90	95	90	98	117	116	118	126	121	94	88	120	103	102	109	84	93	115
122	112	121	125	123	117	120	124	125	119	117	117	120	116	128	107	119	124	129
125	130	121	117	130	147	114	114	148	146	118	129	133	129	126	139	123	120	119
125	132	123	121	130	147	117	114	147	145	124	132	132	129	129	140	126	126	117
107	110	109	125	108	98	117	125	116	110	120	97	118	113	125	101	114	120	120
108	116	103	107	124	142	94	113	147	143	106	119	121	124	119	131	111	104	91
126	127	126	123	114	111	119	118	117	111	122	127	129	113	123	107	122	125	129
126	127	126	123	114	111	119	118	117	111	122	127	129	113	123	107	122	125	129
126	127	126	123	114	111	119	118	117	111	122	127	129	113	123	107	122	125	129
126	127	126	123	114	111	119	118	117	111	122	127	129	113	123	107	122	125	129
126	127	126	123	114	111	119	118	117	111	122	127	129	113	123	107	122	125	129
120	127	121	129	128	138	122	132	143	140	121	124	130	132	130	129	129	131	127
143	131	148	133	141	116	134	142	109	102	135	141	142	135	141	124	142	141	141
100	108	102	103	119	141	89	93	146	146	106	107	112	119	106	127	102	101	94
116	125	114	128	132	139	114	123	146	147	125	128	121	128	118	131	122	126	112
110	98	89	100	73	115	107	88	115	112	100	97	96	94	89	112	95	94	101
108	99	89	100	74	116	109	89	115	114	102	97	97	95	90	113	96	95	101
110	98	89	100	73	115	107	88	115	112	100	97	96	94	89	112	95	94	101
110	98	89	100	73	115	107	88	115	112	100	97	96	94	89	112	95	94	101
110	98	89	100	73	115	107	88	115	112	100	97	96	94	89	112	95	94	101
110	98	89	100	73	115	107	88	115	112	100	97	96	94	89	112	95	94	101
110	98	89	100	73	115	107	88	115	112	100	97	96	94	89	112	95	94	101
110	99	90	101	72	115	108	88	115	112	100	96	97	94	90	112	96	94	102
110	98	89	100	73	115	107	88	115	112	100	97	96	94	89	112	95	94	101
110	98	89	100	73	115	107	88	115	112	100	97	96	94	89	112	95	94	101
110	98	89	100	73	115	107	88	115	112	100	97	96	94	89	112	95	94	101
110	98	89	100	73	115	107	88	115	112	100	97	96	94	89	112	95	94	101
110	98	89	100	73	115	107	88	115	112	100	97	96	94	89	112	95	94	101
110	98	89	100	73	115	107	88	115	112	100	97	96	94	89	112	95	94	101
110	98	89	100	73	115	107	88	115	112	100	97	96	94	89	112	95	94	101
110	98	89	100	73	115	107	88	115	112	100	97	96	94	89	112	95	94	101
110	98	89	100	73	115	107	88	115	112	100	97	96	94	89	112	95	94	101
110	98	89	100	73	115	107	88	115	112	100	97	96	94	89	112	95	94	101
110	98	89	100	73	115	107	88	115	112	100	97	96	94	89	112	95	94	101
109	97	88	99	72	114	106	89	116	113	99	96	95	93	89	111	95	93	100
122	131	124	125	134	147	123	128	150	146	128	130	135	133	135	140	126	128	122
126	132	128	122	134	145	122	122	152	146	124	131	133	132	135	142	127	126	120
126	132	128	122	134	145	122	122	152	146	124	131	133	132	135	142	127	126	120
126	132	128	122	134	145	122	122	152	146	124	131	133	132	135	142	127	126	120
122	131	124	125	134	147	123	128	150	146	128	130	135	133	135	140	126	128	122
122	131	124	125	134	147	123	128	150	146	128	130	135	133	135	140	126	128	122
127	130	126	121	130	144	121	116	148	146	129	132	139	131	129	140	128	126	119
126	132	128	122	134	145	122	122	152	146	124	131	133	132	135	142	127	126	120
126	132	128	122	134	145	122	122	152	146	124	131	133	132	135	142	127	126	120
126	132	128	122	134	145	122	122	152	146	124	131	133	132	135	142	127	126	120
122	131	124	125	134	147	123	128	150	146	128	130	135	133	135	140	126	128	122
126	132	128	122	134	145	122	122	152	146	124	131	133	132	135	142	127	126	120
121	124	123	122	127	139	121	116	141	139	113	122	131	108	123	130	117	117	128
126	125	123	131	121	114	119	124	111	109	125	126	124	123	125	112	122	130	129
100	96	86	107	102	126	97	85	131	140	94	102	79	105	89	118	87	110	96
100	96	86	107	102	126	97	85	131	140	94	102	79	105	89	118	87	110	96
100	96	86	107	102	126	97	85	131	140	94	102	79	105	89	118	87	110	96
100	96	86	107	102	126	97	85	131	140	94	102	79	105	89	118	87	110	96
91	101	88	115	96	127	93	102	132	130	100	99	100	106	106	110	96	106	96
120	124	120	110	107	94	119	113	114	99	107	118	128	107	120	103	114	108	126
92	92	85	105	100	127	98	96	132	128	97	101	104	104	106	122	102	105	98
128	129	117	129	124	138	119	119	145	137	122	121	124	118	121	127	125	125	119
91	91	108	106	89	118	114	118	132	118	83	95	115	93	96	103	80	99	110
144	133	144	139	143	107	137	138	104	102	139	143	139	140	144	115	148	143	148
120	118	123	112	119	141	115	112	143	142	105	125	124	118	124	126	109	118	121
89	89	106	104	88	118	113	116	130	117	81	94	114	91	95	103	78	97	109
89	89	106	104	88	118	113	116	130	117	81	94	114	91	95	103	78	97	109
89	89	106	104	88	118	113	116	130	117	81	94	114	91	95	103	78	97	109
148	134	148	142	149	110	146	143	108	113	151	148	132	145	143	130	151	152	144
111	106	112	108	113	108	109	113	119	119	100	107	109	105	116	104	104	120	118
125	123	122	117	127	137	129	133	141	136	117	124	132	103	123	120	116	124	132
119	124	123	128	119	88	127	130	114	96	120	120	127	119	131	92	120	131	133
101	105	90	92	117	146	86	90	145	141	99	111	116	121	101	140	94	94	81
95	107	91	89	118	138	84	85	137	140	84	108	108	106	106	131	94	105	86
131	117	124	133	107	89	130	130	109	105	120	117	117	119	118	95	118	127	140
125	124	123	127	118	93	123	124	109	101	122	115	128	115	127	103	125	117	133
101	111	111	108	107	115	108	127	135	129	102	110	129	108	121	114	103	100	105
120	119	125	126	119	112	123	126	121	114	118	117	119	117	128	111	118	126	137
103	116	101	99	118	150	98	103	147	148	116	114	121	132	113	134	106	104	91
114	115	121	125	108	93	126	119	103	98	118	117	120	116	105	91	113	114	134

137	137	138	134	139	145	130	136	142	146	138	141	134	133	140	139	137	138	135
78	53	70	87	85	119	98	97	119	118	91	79	95	84	93	110	74	103	94
90	72	89	74	90	114	79	82	127	122	78	92	109	92	78	102	82	93	93
95	83	80	92	81	98	111	109	114	118	104	83	88	96	94	103	91	90	93
125	116	115	96	108	108	108	108	122	117	107	113	124	97	112	108	115	113	123
102	95	101	78	104	122	103	100	136	130	83	101	108	96	105	112	91	104	96
123	106	98	81	82	121	110	86	122	127	93	107	104	91	84	118	99	86	105
90	83	48	80	54	127	94	91	130	136	95	66	99	101	88	126	81	75	64
143	147	147	141	143	133	144	149	109	117	143	148	136	143	149	128	143	146	146
103	95	98	112	106	123	118	111	130	126	108	99	109	100	105	120	107	109	117
91	90	84	85	105	140	90	83	135	137	87	100	96	97	91	124	85	102	102
144	138	146	140	140	114	144	147	120	112	143	144	140	148	147	130	149	144	145
135	135	148	139	144	118	140	146	111	105	138	142	144	144	142	132	142	142	144
87	89	97	92	94	132	93	88	134	134	75	100	111	96	83	117	81	93	100
94	100	91	83	116	141	94	99	136	136	83	107	108	110	104	132	94	103	100
94	100	90	92	101	113	93	76	122	119	82	112	109	94	104	111	96	86	102
104	103	81	103	101	121	102	95	130	132	89	101	106	106	98	130	96	110	97
77	83	93	91	83	121	85	89	131	128	65	92	108	92	75	107	69	71	110
95	99	83	84	106	135	87	82	134	140	92	99	103	108	97	136	92	93	83
100	82	87	99	97	122	107	112	129	132	111	98	109	99	111	130	103	114	104
121	118	120	102	113	116	113	107	120	118	103	119	123	91	128	118	123	106	123
79	74	60	78	82	134	86	74	137	141	76	78	89	97	77	125	57	84	63
92	100	86	100	108	128	91	99	131	137	90	96	95	100	104	127	88	103	83
145	133	151	139	143	117	141	144	119	118	144	142	140	145	145	128	148	148	145
90	101	100	107	101	119	104	108	129	124	94	94	116	103	109	123	98	99	99
96	80	89	81	94	115	70	84	128	118	86	100	109	98	80	108	81	91	85
99	106	92	98	113	137	105	105	135	143	92	101	107	117	118	131	101	104	103
102	107	89	95	76	125	98	82	137	129	86	92	111	94	100	133	92	96	86
82	97	90	79	104	114	101	105	122	123	81	102	119	105	107	100	98	96	102
101	105	105	96	106	133	92	98	139	129	75	120	121	109	101	119	97	95	106
90	93	88	89	110	141	97	89	138	133	80	107	106	111	102	127	92	102	96
68	58	73	93	69	105	97	94	121	123	81	74	82	92	75	106	74	102	87
124	118	122	130	112	124	127	123	127	124	111	115	121	94	124	106	117	122	139
95	98	87	91	107	140	70	69	140	137	78	110	108	97	91	123	88	87	88
132	114	135	120	123	106	132	133	111	101	125	125	129	124	134	111	129	127	143
89	95	89	77	100	112	104	104	120	121	92	100	108	102	105	114	91	92	94
80	79	79	88	95	115	104	99	126	130	100	83	95	104	97	123	77	114	86
106	88	89	86	86	115	106	90	125	129	105	92	103	95	95	114	93	96	107
106	97	98	86	98	118	96	112	120	126	93	99	110	81	94	115	87	94	89
93	69	77	77	74	121	92	99	134	132	84	84	92	85	85	124	74	88	88
94	100	86	111	114	130	74	94	137	133	90	113	104	104	109	122	95	101	84
88	98	103	98	111	127	101	91	127	126	84	101	105	98	109	119	87	103	101
139	142	138	134	129	118	137	137	101	104	135	141	133	135	141	121	138	133	140
114	106	87	101	92	132	95	88	137	133	98	114	99	104	92	132	95	94	89
97	84	91	89	89	115	99	101	125	125	85	91	103	90	82	112	67	97	93
104	95	90	89	85	122	112	85	127	124	99	98	107	99	96	117	96	92	113
93	95	83	93	97	138	77	68	138	138	80	104	107	104	87	125	94	78	95
103	105	86	92	92	128	105	87	130	135	99	107	97	107	82	131	101	102	92
117	120	121	110	110	94	123	117	110	101	113	118	125	108	116	96	124	120	126
96	97	106	103	111	136	91	99	135	135	84	109	108	107	105	127	81	100	96
100	107	94	82	112	143	83	71	141	142	89	117	116	112	93	132	102	81	90
96	92	101	82	96	115	112	106	131	125	75	95	114	82	94	111	82	85	104
107	98	81	82	83	123	100	64	128	132	95	95	104	89	97	127	90	93	88
85	75	79	91	89	114	106	95	113	127	103	95	97	90	97	115	86	103	96
92	103	92	84	90	128	105	98	136	132	81	105	117	100	97	116	92	79	94
106	84	92	91	87	114	107	90	129	127	96	92	103	89	97	112	97	94	107
109	93	88	90	87	136	101	62	127	128	105	106	108	103	87	128	96	89	84
107	98	81	82	83	123	100	64	128	132	95	95	104	89	97	127	90	93	88
123	130	126	125	115	101	116	134	117	116	119	119	139	126	136	102	130	117	129
141	128	138	122	120	98	136	131	109	92	131	136	141	129	136	104	142	127	140
90	104	108	93	93	104	112	122	126	115	86	90	113	90	112	105	93	102	111
89	84	85	82	107	130	101	97	140	148	91	90	94	96	98	122	84	102	74
126	109	131	124	121	117	131	136	113	122	119	121	126	115	128	106	123	132	142
89	94	90	88	96	120	86	99	118	124	88	103	111	99	102	121	88	102	95
51	72	80	99	84	124	96	99	126	126	76	77	91	100	72	114	55	100	77
87	81	86	79	93	124	87	88	125	129	84	93	93	85	85	119	76	104	90
107	100	113	95	124	129	100	99	136	140	107	121	109	117	118	124	114	119	101
124	113	128	127	119	100	122	122	101	88	119	128	127	121	123	96	123	121	143
85	88	76	80	98	137	97	76	131	136	86	93	100	92	77	130	80	82	75
97	88	67	90	83	120	90	77	123	132	93	90	99	93	67	126	86	84	88
93	96	92	70	91	121	100	109	131	129	82	97	113	99	115	116	89	89	104
148	147	148	144	144	141	150	143	118	124	146	150	138	144	147	139	147	152	145
73	70	75	89	86	134	88	92	134	134	85	81	88	92	76	120	59	95	80
55	91	87	101	88	118	103	108	126	125	83	80	104	106	88	103	61	93	85
61	69	87	108	91	118	97	105	126	126	90	79	98	103	86	108	76	96	103

98	100	96	87	106	115	105	100	130	125	105	114	115	108	101	125	94	101	108
92	70	72	80	58	118	105	108	129	127	93	55	101	91	97	117	82	79	93
95	97	93	91	122	135	76	91	142	142	99	100	108	110	112	122	102	100	86
83	88	84	69	100	128	99	86	131	136	94	93	98	99	86	128	81	101	78
108	109	96	89	91	127	94	93	136	132	93	99	100	101	90	118	93	80	91
88	93	84	85	92	132	88	86	139	135	80	100	107	102	72	114	81	85	106
138	121	126	123	117	109	126	125	122	115	126	130	129	120	121	104	123	130	128
86	75	75	78	84	119	99	104	124	122	90	89	102	85	91	120	78	86	95
77	86	83	49	89	127	94	82	122	130	87	91	110	87	94	114	82	70	90
98	114	117	122	124	110	113	123	120	106	104	119	121	128	115	104	113	119	119
71	66	76	108	103	110	96	96	123	128	90	82	86	90	80	100	73	110	99
88	80	81	83	81	130	90	100	133	133	93	82	103	94	86	136	85	83	83
94	85	89	86	107	132	83	87	141	141	86	106	95	93	95	124	87	103	76
101	80	85	80	87	116	108	102	126	130	98	101	97	91	91	115	86	100	93
91	85	86	85	102	124	105	105	133	133	87	85	106	99	97	124	92	104	84
97	96	97	115	105	121	112	109	122	126	107	107	103	109	97	101	98	124	114
96	85	86	83	94	126	92	77	134	134	96	101	101	96	67	120	87	86	87
66	81	85	83	104	127	89	103	132	132	78	93	112	104	104	115	82	80	93
142	134	150	137	147	119	144	144	120	115	140	144	143	147	146	131	150	145	146
91	93	104	109	109	122	92	110	140	131	92	99	100	103	94	109	75	116	97
79	93	86	59	85	131	97	92	130	133	67	90	113	84	98	118	74	68	83
78	89	93	95	99	124	102	104	134	135	89	91	107	109	114	114	95	104	109
92	99	80	84	102	143	93	90	136	138	82	101	110	111	103	135	88	91	86
97	85	77	82	96	133	106	91	137	133	100	90	101	97	84	132	82	101	93
98	100	88	84	93	128	108	100	132	133	102	107	115	99	95	120	94	101	95
83	89	84	107	86	129	82	91	138	139	91	80	105	114	94	126	76	87	82
97	100	100	98	92	118	104	109	128	129	94	103	109	104	105	115	94	95	106
76	89	80	85	113	132	89	90	131	139	87	92	98	105	98	124	76	97	70
89	72	88	89	88	116	96	105	126	120	88	90	98	77	88	114	65	92	88
94	103	89	70	90	123	97	93	129	128	85	95	107	94	94	111	79	90	92
86	100	90	103	102	128	92	101	139	140	92	103	110	114	105	125	79	101	76
93	94	81	83	102	123	91	77	137	126	83	91	100	99	97	117	86	87	80
76	87	92	89	81	129	94	93	138	138	78	89	107	108	96	118	74	83	82
88	108	99	90	110	124	99	100	130	127	84	112	112	114	120	116	100	93	99
100	88	97	102	81	107	109	110	126	123	96	96	111	94	101	121	79	101	97
103	84	85	96	89	111	105	100	126	123	96	96	110	91	103	119	92	95	111
110	90	91	83	75	116	96	105	125	123	89	97	97	85	98	117	93	87	93
90	71	97	94	99	117	90	84	131	133	83	96	98	84	76	106	72	106	89
102	104	103	93	94	136	92	99	138	127	86	100	111	105	105	108	108	93	106
86	84	83	84	100	121	92	93	126	134	94	93	105	111	99	126	92	93	77
96	100	100	109	110	136	94	95	143	140	92	111	112	111	93	129	81	95	83
91	88	83	92	91	122	80	100	122	126	91	95	102	92	91	116	90	107	90
101	91	95	88	102	116	95	92	132	127	88	99	100	92	94	115	91	109	87
102	99	88	93	103	126	108	90	135	135	107	97	112	113	106	126	103	96	83
78	72	80	96	79	119	78	91	129	128	85	75	84	97	89	116	85	95	89
76	82	89	88	88	131	95	94	140	133	70	79	91	91	88	122	48	87	77
76	77	91	91	88	127	71	89	135	137	77	83	103	109	93	109	72	84	92
92	76	79	84	76	125	97	89	134	138	90	80	94	98	75	127	72	103	70
117	95	103	112	85	102	115	120	106	102	111	101	108	112	115	119	119	102	119
103	86	87	99	86	104	105	112	113	104	94	93	112	81	102	107	88	90	115
86	85	75	80	95	129	94	103	129	136	97	87	93	91	97	128	80	96	74
95	101	87	95	84	117	107	120	124	119	81	96	121	98	100	110	84	78	105
84	80	85	81	95	137	88	88	136	136	80	101	98	91	87	120	83	91	86
127	115	106	109	104	120	122	117	126	119	119	103	114	99	114	118	121	107	117
124	117	112	118	98	127	109	106	128	121	102	113	109	96	110	116	105	108	126
114	106	109	111	100	123	115	99	120	117	105	109	113	100	103	110	111	107	126
112	114	101	90	103	130	116	96	130	131	109	101	115	108	104	126	104	93	107
101	98	87	107	97	130	85	84	137	130	90	111	104	89	98	116	88	100	89
110	98	103	96	83	117	101	111	131	124	99	98	102	100	100	111	96	103	103
89	93	105	92	95	99	115	112	119	112	92	102	107	102	111	111	91	107	107
116	106	113	105	114	119	129	112	121	114	115	110	122	97	113	113	96	106	126
106	106	100	110	110	114	114	116	115	112	111	102	106	109	110	115	104	116	114
111	113	93	98	104	135	101	87	129	130	103	102	114	113	100	129	100	92	103
138	127	142	131	132	97	134	124	102	94	131	140	137	137	133	100	137	139	143
110	94	96	92	85	114	110	101	114	104	96	99	102	92	104	110	97	94	118
100	81	100	77	91	114	97	98	128	128	91	93	100	100	97	112	96	96	93
84	81	85	83	84	110	96	115	123	126	95	75	104	88	108	101	94	81	102
101	88	89	98	107	129	88	95	136	137	86	106	93	93	94	125	85	112	88
103	85	84	87	99	128	89	108	136	136	93	93	101	94	104	128	97	104	86
0	75	75	88	102	128	96	106	128	132	75	77	102	107	91	109	66	89	87
75	0	70	84	87	118	92	99	125	130	88	61	89	89	89	118	68	97	93
75	70	0	77	75	128	96	92	128	136	92	67	91	99	87	127	72	78	72
88	84	77	0	88	126	97	79	131	131	86	92	107	92	95	123	87	77	82
102	87	75	88	0	115	105	94	130	125	91	68	106	98	88	116	85	76	90
128	118	128	126	115	0	134	133	77	72	127	115	115	121	131	82	136	130	135

96	92	96	97	105	134	0	90	137	129	73	98	106	100	101	122	82	84	82
106	99	92	79	94	133	90	0	133	131	89	111	104	101	83	128	94	91	88
128	125	128	131	130	77	137	133	0	64	133	132	119	123	125	110	136	129	144
132	130	136	131	125	72	129	131	64	0	127	132	126	126	134	95	138	122	148
75	88	92	86	91	127	73	89	133	127	0	87	107	84	94	115	59	74	103
77	61	67	92	68	115	98	111	132	132	87	0	92	97	98	123	75	91	86
102	89	91	107	106	115	106	104	119	126	107	92	0	106	98	121	92	125	92
107	89	99	92	98	121	100	101	123	126	84	97	106	0	108	114	80	99	113
91	89	87	95	88	131	101	83	125	134	94	98	98	108	0	123	70	100	86
109	118	127	123	116	82	122	128	110	95	115	123	121	114	123	0	120	120	140
66	68	72	87	85	136	82	94	136	138	59	75	92	80	70	120	0	83	78
89	97	78	77	76	130	84	91	129	122	74	91	125	99	100	120	83	0	90
87	93	72	82	90	135	82	88	144	148	103	86	92	113	86	140	78	90	0
