# Supplementary material for: Hybrid Origins of Citrus Varieties Inferred from DNA Marker Analysis of Nuclear and Organelle Genomes
Source: PLoS One. 2016 Nov 30;11(11):e0166969. doi: 10.1371/journal.pone.0166969 (PMC5130255; doi:10.1371/journal.pone.0166969)
Supplement: S7 Table — (PDF) [file pone.0166969.s010.pdf]

S7 Table. Summary of the Hardy-Weinberg equiblium test of the 169 certified DNA markers with the 101 native varieties.

Locus	Marker	Rep [1)]	p-value	Rejected [2)]
1	CTVR01	*	0.1924	
4	CUBER410	*	0.5478	
8	CUBER918	*	0.1062	
9	CUBER920	*	0.6338	
12	CUBER939	*	0.0805	
13	CUBER951	*	0.0684	
14	GRW2011	*	0.3408	
17	GRW3011	*	0.3135	
18	GSR10S09	*	0.2013	
19	GSR10S17	*	0.0056	x
20	GSR10S29	*	0.8076	
28	GSR3121	*	0.2444	
30	GSR3128	*	0.0059	x
31	GSR3136	*	0.0406	x
33	GSR3138	*	0.0033	x
34	GSR3140	*	0.1780	
42	GSR6101	*	0.7697	
43	GSR6122	*	0.0020	x
44	GSR6129	*	0.5084	
45	GSR6133	*	0.4673	
46	GSR6135	*	0.0036	x
48	NSX23	*	0.0959	
49	NSX32	*	0.0169	x
51	NSX38	*	0.5999	
52	NSX39	*	0.6486	
53	NSX43	*	0.3691	
58	NSX82	*	0.0250	x
59	NSX84	*	0.0961	
60	NSX115	*	1.0000	
62	NSX121	*	0.0012	x
63	NSX132	*	0.6547	
64	NSX137	*	0.4597	
65	NSX141	*	0.6636	
66	NSX145	*	0.2653	
67	NSX150	*	0.7538	
68	NSX153	*	0.3748	
69	NSX156	*	0.5377	
70	NSX160	*	0.4633	
71	NSX162	*	0.3644	
72	NSX165	*	0.0056	x
73	NSX169	*	0.1483	
74	NSX170	*	0.4661	
77	NSX187	*	1.0000	
78	SRPE02	*	0.0029	x
80	SRPE05	*	0.7385	
81	TSRA101	*	0.0002	x
82	TSRA103	*	0.0055	x
86	TSRA110	*	0.0450	x
87	TSRA117	*	0.0040	x
89	TSGR264	*	0.0558	
91	TSGR271	*	0.4698	
96	NSX70	*	0.7366	
99	NSX118	*	0.3334	

102	NSX161	*	0.0098	x
105	SSR07A05	*	0.1783	
106	SSR07A07	*	0.0158	x
107	SSR07A14	*	0.2306	
109	SSR07A19	*	0.8423	
110	SSR07A23	*	0.0369	x
111	SSR07A24	*	0.0758	
112	SSR07A20	*	0.2663	
113	SSR07B01	*	0.0646	
115	SSR07B08	*	0.0707	
117	SSR07B14	*	0.8334	
118	SSR07B27	*	0.9399	
121	SSR08A02	*	1.0000	
122	SSR08A06	*	0.9102	
124	SSR08A09	*	0.0920	
126	SSR08A15	*	0.4627	
127	SSR08A16	*	0.4642	
129	SSR08B06	*	0.3170	
131	SSR08B15	*	0.3530	
132	SSR08B20	*	0.0236	x
136	SSR08B27	*	0.0899	
137	SSR08B28	*	0.1318	
138	SSR08B29	*	0.7619	
140	SSR08B32	*	0.8080	
147	SSR08B60	*	0.0055	x
148	SSR08B62	*	0.9550	
150	SSR08B66	*	0.5442	
151	SSR08B68	*	1.0000	
152	SSR08B75	*	0.0145	x
153	SSR08B78	*	0.1003	
155	SSR08B84	*	0.3386	
156	SSR08B85	*	0.0829	
157	SSR08B88	*	0.6318	
158	SSR08B90	*	0.2737	
159	SSR08B92	*	0.0211	x
160	SSR08B95	*	0.4630	
161	SSR11A06	*	0.0112	x
163	SSR11A27	*	0.8417	
165	TSGR904	*	0.8246	
166	TSGR905	*	0.2106	
168	TSR8EM15	*	1.0000	
169	TSR8EM24	*	0.7802	
172	TSRB09	*	0.9581	
173	TSRB11	*	0.6065	
174	TSRB29	*	0.1511	
175	TSRB38	*	0.6662	
176	TSRB39	*	0.0166	x
177	TSRF101	*	0.3102	
178	TSRF105	*	0.6936	
179	TSRF116	*	0.6843	
181	TSRF124	*	0.2832	
182	TSRF136	*	0.1033	
183	TSRF138	*	0.1049	
184	TSRF144	*	0.0962	
185	TSRF157	*	0.6078	
186	TSRF160	*	0.4292	
187	TSRF161	*	0.1545	

188	TSRF162	*	0.0849	
189	TSRF164	*	0.6574	
190	TSRF167	*	0.1020	
191	TSRF169	*	0.1853	
193	TSRF184	*	0.3629	
194	TSRF187	*	0.5498	
196	TSRF192	*	0.0044	x
197	TSRF194	*	0.5335	
198	TSRF195	*	0.9217	
199	TSRF198	*	1.0000	
200	TSRF204	*	0.8862	
201	TSRF208	*	1.0000	
202	TSRF211	*	0.7136	
203	TSRF215	*	0.5219	
204	TSRF218	*	0.8829	
206	TSRF225	*	1.0000	
207	TSRF229	*	0.1847	
208	TSRF230	*	0.8097	
210	TSRF237	*	0.1306	
212	TSRN214	*	0.1480	
213	TSRP06	*	0.2896	
214	TSRP07	*	0.8204	
215	CiBE0246	*	0.3727	
216	CiBE0447	*	0.0009	x
217	CiBE1644	*	0.0017	x
218	CiBE2165	*	0.1088	
219	CiBE2227	*	0.0000	x
220	CiBE4265	*	0.3240	
221	CX0004	*	0.2501	
223	CX0035	*	0.4254	
224	CX2004	*	0.5538	
227	CX2040	*	0.1184	
228	CX3001	*	0.1008	
230	CX4005	*	0.5349	
235	CX5039	*	0.3589	
236	CX6024	*	0.1262	
237	CX6035	*	0.7129	
238	CX6037	*	0.5764	
239	CX6F21	*	0.1110	
241	F02	*	0.0327	x
245	F40	*	0.3108	
246	F50	*	0.0347	x
247	F79	*	0.2699	
2	CTVR04	**	0.1902	
6	CUBER602	**	0.0677	
11	CUBER935	**	0.5688	
90	TSGR265	**	0.8227	
133	SSR08B21	**	0.8939	
135	SSR08B25	**	0.0247	x
141	SSR08B35	**	0.0963	
143	SSR08B40	**	0.1220	
146	SSR08B54	**	0.0926	
164	TSGR901	**	0.3131	
195	TSRF191	**	0.5219	
205	TSRF206	**	0.1547	
209	TSRF234	**	0.8105	
234	CX5022	**	0.0801	

242	F03		**	0.9551		
243	F16		**	0.4321		
		169			31	138

HWE test for multi allelic data was evaluated by MCMC method (Guo and Thompson, 1994?) that is implemented on Arlequin (ver. 3.5).
The test was conducted 10 times, then any DNA markers that showed *p*-value smaller than 0.05 were rejected from the following structure analysis.

1) Representative markers (see Table xx).
2) "x" indicates the rejected markers by $p < .05$.
