# Supplementary material for: Hybrid Origins of Citrus Varieties Inferred from DNA Marker Analysis of Nuclear and Organelle Genomes
Source: PLoS One. 2016 Nov 30;11(11):e0166969. doi: 10.1371/journal.pone.0166969 (PMC5130255; doi:10.1371/journal.pone.0166969)
Supplement: S8 Table — (PDF) [file pone.0166969.s011.pdf]

S8 Table. Inferred admixture population (Q) of 101 native citrus varieties for a *K* = 3 population model

		Pummelo	Citron	Mandarin
		Inferred populations		
ID	Variety name	P1 (pummelo)	P2 (citron)	P3 (mandarin)
A110	Pummelo whitetype	0.9960	0.0020	0.0020
A196	Uchimurasaki	0.9960	0.0020	0.0020
A032	Hirado buntan	0.9950	0.0020	0.0030
A004	Banpeiyu	0.9940	0.0030	0.0030
A018	Egami buntan	0.9930	0.0040	0.0030
A145	Suisho buntan	0.9850	0.0060	0.0090
A088	Mato buntan	0.9657	0.0293	0.0050
A191	Tosa buntan	0.8949	0.0242	0.0809
A002	Anseikan	0.8798	0.0110	0.1092
A027	Hanayu	0.0020	0.9900	0.0080
A208	Yuzu	0.0040	0.9888	0.0072
A089	Mexican lime	0.0260	0.9710	0.0030
A041	Ichanchii	0.1083	0.8857	0.0060
A079	Kourai Tachibana	0.0030	0.8456	0.1514
A085	Lemon	0.1539	0.8329	0.0132
A087	Limonia	0.0070	0.8137	0.1793
A111	Rokugatsumikan	0.0931	0.8099	0.0970
A042	Ichang lemon	0.3903	0.6057	0.0040
A006	Bergamot	0.3430	0.6319	0.0251
A053	Kikudaidai	0.3611	0.5993	0.0396
A015	Dada	0.4270	0.4479	0.1252
A046	Jabon	0.3390	0.5102	0.1508
A036	Hyuganatsu	0.3711	0.4090	0.2199
A090	Meyer lemon	0.0975	0.6821	0.2203
A086	Lemonade	0.2180	0.5499	0.2321
A049	Kaikoukan	0.7555	0.0040	0.2405
A045	Jabara	0.2050	0.5510	0.2440
A198	Unzoki	0.2240	0.5042	0.2718
A082	Kunenbo	0.0350	0.6736	0.2914
A033	Hiroshimanatsubuntan	0.6491	0.0555	0.2954
A144	Sudachi	0.0070	0.6941	0.2989
A051	Kawachi bankan	0.6940	0.0050	0.3010
A175	Tachibana	0.0020	0.6876	0.3104
A048	Kabuchi	0.1930	0.4958	0.3112
A172	Tachibana	0.0020	0.6730	0.3250
A047	Kabosu	0.0940	0.5789	0.3271
A141	Sour orange	0.4132	0.2445	0.3422
A073	Kizu	0.1120	0.5415	0.3465
A091	Mochiyu	0.1490	0.5018	0.3492
A055	Kinkoji	0.6383	0.0040	0.3577
A024	Grapefruit	0.6276	0.0040	0.3684
A028	Hassaku	0.6256	0.0030	0.3714
A199	USSR Tangelo	0.6143	0.0053	0.3804
A023	Girimikan	0.0191	0.5983	0.3826
A205	Yuge hyoukan	0.6020	0.0050	0.3930
A174	Tachibana	0.0020	0.5984	0.3996
A003	Asahikan	0.5938	0.0040	0.4022
A136	Shiikuwasha	0.0030	0.5832	0.4138
A035	Hyoukan	0.5715	0.0040	0.4245
A192	Twukkuni	0.0301	0.5427	0.4272
A203	Yamamikan	0.5597	0.0040	0.4363
A076	Koji	0.0313	0.5266	0.4421
A135	Shiikuwasha	0.0020	0.5551	0.4429
A030	Henka mikan	0.0370	0.5113	0.4517
A101	Oogonkan	0.2876	0.2589	0.4535
A095	Naruto	0.5300	0.0050	0.4650
A202	Yamabuki	0.5230	0.0110	0.4660

A050	Kawabata	0.3293	0.1938	0.4769
A134	Satsuma Kikoku	0.4396	0.0711	0.4893
A001	Andoukan	0.4962	0.0040	0.4998
A052	Keraji	0.1570	0.3401	0.5029
A112	Sanbokan	0.4861	0.0068	0.5071
A008	Binkitsu	0.2164	0.2745	0.5091
A207	Yuukunibu	0.4830	0.0040	0.5130
A098	Natsudaidai	0.4670	0.0060	0.5270
A103	Ootoukan	0.4619	0.0072	0.5309
A080	Kotokan	0.4378	0.0200	0.5422
A187	Tengu	0.4492	0.0030	0.5478
A021	Funadoko	0.4300	0.0060	0.5640
A137	Shunkokan	0.4053	0.0240	0.5707
A197	Ujukitsu	0.4090	0.0060	0.5850
A044	Iyo	0.4089	0.0030	0.5881
A104	Oukan	0.3669	0.0252	0.6079
A019	Fukure mikan	0.0060	0.3601	0.6339
A162	Sweet orange	0.3607	0.0050	0.6343
A081	Kunenbo	0.3542	0.0040	0.6417
A147	Suruga Yuko	0.0100	0.2996	0.6904
A125	Satsuma mandarin	0.2892	0.0050	0.7058
A204	Yatsushiro	0.2430	0.0050	0.7520
A100	Nidonari mikan	0.1580	0.0680	0.7740
A186	Temple	0.2220	0.0040	0.7740
A054	King mandarin	0.1773	0.0282	0.7945
A188	Tizon	0.1665	0.0092	0.8242
A183	Tankan	0.1130	0.0060	0.8810
A014	Cravo mandarin	0.1000	0.0040	0.8960
A009	Clementine	0.0710	0.0040	0.9250
A092	Murcott	0.0640	0.0070	0.9290
A146	Sunki	0.0020	0.0674	0.9306
A109	Ponkitsu	0.0350	0.0243	0.9407
A013	Cleopatra	0.0030	0.0397	0.9573
A206	Youpi ju (Yuhikitsu)	0.0020	0.0270	0.9710
A107	Ponkan	0.0045	0.0140	0.9815
A005	Ben Di Zao	0.0040	0.0120	0.9840
A022	Genshokan	0.0030	0.0120	0.9850
A031	Hickson	0.0070	0.0060	0.9870
A075	Kobeni mikan	0.0020	0.0070	0.9910
A059	Kishu mandarin	0.0020	0.0050	0.9930
A138	Sokitsu	0.0020	0.0040	0.9940
A201	Willowleaf mandarin	0.0020	0.0040	0.9940
A016	Dancy	0.0020	0.0030	0.9950
A200	Willowleaf mandarin	0.0020	0.0030	0.9950

P1, P2 and P3: The inferred three populations that correspond to pummelo, citron, and mandarin.
