# Supplementary material for: Hybrid Origins of Citrus Varieties Inferred from DNA Marker Analysis of Nuclear and Organelle Genomes
Source: PLoS One. 2016 Nov 30;11(11):e0166969. doi: 10.1371/journal.pone.0166969 (PMC5130255; doi:10.1371/journal.pone.0166969)
Supplement: S9 Table — (PDF) [file pone.0166969.s012.pdf]

Table S9. Genotypes of all plant samples obtained with 11 SSR markers for organelles

A: indigenous varieties

No.	Rep 1)	Variety	Strain	Chloroplast markers								Mitochondria markers			Class	Same GT	Organellar type
				CSS03	CSS04	CSL01	CSL09	ccmp2.2	ccmp6.2	ccmp7.2	ccmp10.2	*rrn* 5/ *rrn* 18-1	*nad* 2/ 4-3	*nad* 7/ 1-2			
A001	*	Andoukan		315	234	379	298	210	143	148	130	274	269	172	C04	67	pummelo type
A002	*	Anseikan		315	234	379	298	210	143	148	130	274	269	172	C04	67	pummelo type
A003	*	Asahikan		315	234	379	298	210	143	148	130	274	269	172	C04	67	pummelo type
A004	*	Banpeiyu		315	234	379	298	210	143	148	130	274	269	172	C04	67	pummelo type
A005	*	Ben Di Zao		315	235	372	303	210	141	148	130	269	253	164	C12	192	Mandarin type
A006	*	Bergamot		315	233	377	305	210	141	148	130	274	269	172	C06	13	Lemon type
A007		Bendi Guangju		315	233	377	298	210	143	148	130	274	269	172	C07	45	Sweet orange type
A008	*	Binkitsu		315	233	377	298	210	143	148	130	274	269	172	C07	45	Sweet orange type
A009	*	Clementine		315	235	372	303	210	141	148	130	269	253	164	C12	192	Mandarin type
A010		Clementine	A Peau Fin	315	235	372	303	210	141	148	130	269	253	164	C12	192	Mandarin type
A011		Clementine	Caffin	315	235	372	303	210	141	148	130	269	253	164	C12	192	Mandarin type
A012		Clementine	de Nules	315	235	372	303	210	141	148	130	269	253	164	C12	192	Mandarin type
A013	*	Cleopatra		315	234	370	304	210	141	148	130	269	261	164	C17	1	Cleopatra type
A014	*	Cravo mandarin		315	235	372	303	210	141	148	130	269	253	164	C12	192	Mandarin type
A015	*	Dada		315	234	379	298	210	143	148	130	274	269	172	C04	67	pummelo type
A016	*	Dancy		315	235	372	303	210	141	148	130	269	253	164	C12	192	Mandarin type
A017		Dancy	Obenimikan	315	235	372	303	210	141	148	130	269	253	164	C12	192	Mandarin type
A018	*	Egami buntan		315	234	379	298	210	143	148	130	274	269	172	C04	67	pummelo type
A019	*	Fukure mikan		315	235	372	303	210	141	148	130	269	253	164	C12	192	Mandarin type
A020		Fukushukan		315	233	377	305	210	141	148	130	274	269	172	C06	13	Lemon type
A021	*	Funadoko		315	234	379	298	210	143	148	130	274	269	172	C04	67	pummelo type
A022	*	Genshokan		315	235	372	303	210	141	148	130	269	253	164	C12	192	Mandarin type
A023	*	Girimikan		315	234	378	304	210	142	148	130	274	269	172	C18	6	Koji type
A024	*	Grapefruit	Marsh	315	234	379	298	210	143	148	130	274	269	172	C04	67	pummelo type
A025		Grapefruit	Red blush	315	234	379	298	210	143	148	130	274	269	172	C04	67	pummelo type
A026		Grapefruit	Triumph	315	234	379	298	210	143	148	130	274	269	172	C04	67	pummelo type
A027	*	Hanayu		315	233	371	311	210	139	159	129	274	261	172	C09	3	Yuzu type
A028	*	Hassaku		315	234	379	298	210	143	148	130	274	269	172	C04	67	pummelo type
A029		Hebesu		315	233	377	298	210	143	148	130	274	269	172	C07	45	Sweet orange type
A030	*	Henka mikan		315	233	377	298	210	143	148	130	274	269	172	C07	45	Sweet orange type
A031	*	Hickson		315	235	372	303	210	141	148	130	269	253	164	C12	192	Mandarin type
A032	*	Hirado buntan		315	234	379	298	210	143	148	130	274	269	172	C04	67	pummelo type
A033	*	Hiroshimanatsubuntan		315	233	377	305	210	141	148	130	274	269	172	C06	13	Lemon type
A034		Houraikan		315	234	379	298	210	143	148	130	274	269	172	C04	67	pummelo type
A035	*	Hyoukan		315	234	379	298	210	143	148	130	274	269	172	C04	67	pummelo type
A036	*	Hyuganatsu		315	233	377	298	210	144	148	130	274	269	172	C05	17	Hyuganatsu type
A037		Hyuganatsu	Ihara 1	315	233	377	298	210	144	148	130	274	269	172	C05	17	Hyuganatsu type
A038		Hyuganatsu	Muroto Konatsu	315	233	377	298	210	144	148	130	274	269	172	C05	17	Hyuganatsu type
A039		Hyuganatsu	Orange Hyuga	315	233	377	298	210	144	148	130	274	269	172	C05	17	Hyuganatsu type
A040		Hyuganatsu	Shoukakukei Hyuganatsu	315	233	377	298	210	144	148	130	274	269	172	C05	17	Hyuganatsu type
A041	*	Ichanchii		315	237	371	306	224	149	159	129	274	261	172	C01	1	C. ichangensis type
A042	*	Ichang lemon		315	233	377	298	210	145	148	130	274	269	172	C10	1	Ichang lemon type
A043		Iyo	Miyauchi Iyo	315	234	379	298	210	143	148	130	274	269	172	C04	67	pummelo type
A044	*	Iyo	Ootani Iyo	315	234	379	298	210	143	148	130	274	269	172	C04	67	pummelo type
A045	*	Jabara		315	233	377	298	210	143	148	130	274	269	172	C07	45	Sweet orange type
A046	*	Jabon		315	234	379	298	210	143	148	130	274	269	172	C04	67	pummelo type
A047	*	Kabosu		315	233	377	298	210	143	148	130	274	269	172	C07	45	Sweet orange type
A048	*	Kabuchi		315	233	377	298	210	143	148	130	274	269	172	C07	45	Sweet orange type
A049	*	Kaikoukan		315	234	379	298	210	143	148	130	274	269	172	C04	67	pummelo type
A050	*	Kawabata		315	233	377	298	210	144	148	130	274	269	172	C05	17	Hyuganatsu type
A051	*	Kawachi bankan		315	234	379	298	210	143	148	130	274	269	172	C04	67	pummelo type
A052	*	Keraji		315	233	377	298	210	143	148	130	274	269	172	C07	45	Sweet orange type
A053	*	Kikudaidai		315	234	379	298	210	143	148	130	274	269	172	C04	67	pummelo type
A054	*	King mandarin		315	235	372	303	210	141	148	130	269	253	164	C12	192	Mandarin type
A055	*	Kinkoji		315	234	379	298	210	143	148	130	274	269	172	C04	67	pummelo type
A056		Kinukawa		315	234	379	298	210	143	148	130	274	269	172	C04	67	pummelo type
A057		Kishu mandarin	Hira Kishu	315	235	372	303	210	141	148	130	269	253	164	C12	192	Mandarin type
A058		Kishu mandarin	Hisago Komikan	315	235	372	303	210	141	148	130	269	253	164	C12	192	Mandarin type
A059	*	Kishu mandarin	Kishu	315	235	372	303	210	141	148	130	269	253	164	C12	192	Mandarin type
A060		Kishu mandarin	Kishu mikan	315	235	372	303	210	141	148	130	269	253	164	C12	192	Mandarin type
A061		Kishu mandarin	Kishu mikan Ihara Ichijoji	315	235	372	303	210	141	148	130	269	253	164	C12	192	Mandarin type
A062		Kishu mandarin	Komikan Fukuyama (Kinkou PEARL)	315	235	372	303	210	141	148	130	269	253	164	C12	192	Mandarin type
A063		Kishu mandarin	Komikan Kawachi	315	235	372	303	210	141	148	130	269	253	164	C12	192	Mandarin type
A064		Kishu mandarin	Komikan Tensui	315	235	372	303	210	141	148	130	269	253	164	C12	192	Mandarin type
A065		Kishu mandarin	Kouda mikan	315	235	372	303	210	141	148	130	269	253	164	C12	192	Mandarin type
A066		Kishu mandarin	Mukaku Kishu (seedless Kishu)	315	235	372	303	210	141	148	130	269	253	164	C12	192	Mandarin type
A067		Kishu mandarin	Nan feng mi ju	315	235	372	303	210	141	148	130	269	253	164	C12	192	Mandarin type

A068		Kishu mandarin	Ozaki Komikan	315	235	372	303	210	141	148	130	269	253	164	C12	192	Mandarin type
A069		Kishu mandarin	Sakurajima Komikan Matsuura	315	235	372	303	210	141	148	130	269	253	164	C12	192	Mandarin type
A070		Kishu mandarin	Sakurajima Komikan senbatsu 1gou	315	235	372	303	210	141	148	130	269	253	164	C12	192	Mandarin type
A071		Kishu mandarin	Sakurajima Komikan Shirahama	315	235	372	303	210	141	148	130	269	253	164	C12	192	Mandarin type
A072		Kishu mandarin	Taka Mkan	315	235	372	303	210	141	148	130	269	253	164	C12	192	Mandarin type
A073	*	Kizu		315	233	377	298	210	143	148	130	274	269	172	C07	45	Sweet orange type
A074		Kobayashi mikan		315	234	379	298	210	143	148	130	274	269	172	C04	67	pummelo type
A075	*	Kobeni mikan		315	235	372	303	210	141	148	130	269	253	164	C12	192	Mandarin type
A076	*	Koji		315	234	378	304	210	142	148	130	274	269	172	C18	6	Koji type
A077		Komikan 2009-130		315	234	378	304	210	142	148	130	274	269	172	C18	6	Koji type
A078		Konejime		315	233	377	299	210	143	148	130	274	269	172	C08	2	Satsumakikoku type
A079	*	Kourai Tachibana		315	233	371	311	210	139	159	129	274	261	172	C09	3	Yuzu type
A080	*	Kotokan		315	234	379	298	210	143	148	130	274	269	172	C04	67	pummelo type
A081	*	Kunenbo		315	233	377	298	210	143	148	130	274	269	172	C07	45	Sweet orange type
A082	*	Kunenbo	Kagoshima 0007	335	233	374	310	210	141	148	130	269	253	164	C11	1	Kunenbo_B type
A083		Kunenbo	Kagoshima 0027	315	233	377	298	210	143	148	130	274	269	172	C07	45	Sweet orange type
A084		Kunenbo	Kunenbo Kamikoshikijima	315	233	377	298	210	143	148	130	274	269	172	C07	45	Sweet orange type
A085	*	Lemon	Lisbon	315	233	377	305	210	141	148	130	274	269	172	C06	13	Lemon type
A086	*	Lemonade	LEMONADE	315	233	377	298	210	144	148	130	274	269	172	C05	17	Hyuganatsu type
A087	*	Limonia		315	233	370	304	210	141	148	130	269	261	164	C03	1	Limonia type
A088	*	Mato buntan		315	234	379	298	210	143	148	130	274	269	172	C04	67	pummelo type
A089	*	Mexican lime		315	233	379	305	217	148	148	130	274	261	172	C02	1	Mexican lime
A090	*	Meyer lemon		315	233	377	298	210	143	148	130	274	269	172	C07	45	Sweet orange type
A091	*	Mochiyu		315	233	377	298	210	143	148	130	274	269	172	C07	45	Sweet orange type
A092	*	Murcott		315	235	372	303	210	141	148	130	269	253	164	C12	192	Mandarin type
A093		Myrttle leaf orange	Chinott	315	233	377	305	210	141	148	130	274	269	172	C06	13	Lemon type
A094		Nansho daidai		315	233	377	298	210	143	148	130	274	269	172	C07	45	Sweet orange type
A095	*	Naruto		315	234	379	298	210	143	148	130	274	269	172	C04	67	pummelo type
A096		Natsudaidai		315	234	379	298	210	143	148	130	274	269	172	C04	67	pummelo type
A097		Natsudaidai	Beniamanatsu	315	234	379	298	210	143	148	130	274	269	172	C04	67	pummelo type
A098	*	Natsudaidai	Kawano Natsudaidai	315	234	379	298	210	143	148	130	274	269	172	C04	67	pummelo type
A099		Natsudaidai	Tachibana orange	315	234	379	298	210	143	148	130	274	269	172	C04	67	pummelo type
A100	*	Nidonari mikan		315	235	372	303	210	141	148	130	269	253	164	C12	192	Mandarin type
A101	*	Oogonkan		315	233	377	298	210	144	148	130	274	269	172	C05	17	Hyuganatsu type
A102		Ootachibana		315	234	379	298	210	143	148	130	274	269	172	C04	67	pummelo type
A103	*	Ootoukan		315	234	379	298	210	143	148	130	274	269	172	C04	67	pummelo type
A104	*	Oukan		315	234	379	298	210	143	148	130	274	269	172	C04	67	pummelo type
A105		Ponkan	Ihara ponkan	315	235	372	303	210	141	148	130	269	253	164	C12	192	Mandarin type
A106		Ponkan	Morita ponkan	315	235	372	303	210	141	148	130	269	253	164	C12	192	Mandarin type
A107	*	Ponkan	Oota ponkan	315	235	372	303	210	141	148	130	269	253	164	C12	192	Mandarin type
A108		Ponkan	Yoshida ponkan	315	235	372	303	210	141	148	130	269	253	164	C12	192	Mandarin type
A109	*	Ponkitsu		315	235	370	305	210	141	148	130	269	261	164	C13	7	Sunki type
A110	*	Pummelo whitetype		315	234	379	298	210	143	148	130	274	269	172	C04	67	pummelo type
A111	*	Rokugatsumikan		315	233	377	305	210	141	148	130	274	269	172	C06	13	Lemon type
A112	*	Sanbokan		315	234	379	298	210	143	148	130	274	269	172	C04	67	pummelo type
A113		Satsuma mandarin	Aoshima Unshu	315	235	372	303	210	141	148	130	269	253	164	C12	192	Mandarin type
A114		Satsuma mandarin	Dobashi Beni	315	235	372	303	210	141	148	130	269	253	164	C12	192	Mandarin type
A115		Satsuma mandarin	Haraguchi Wase	315	235	372	303	210	141	148	130	269	253	164	C12	192	Mandarin type
A116		Satsuma mandarin	Imamura Unshu	315	235	372	303	210	141	148	130	269	253	164	C12	192	Mandarin type
A117		Satsuma mandarin	Iwasaki Wase	315	235	372	303	210	141	148	130	269	253	164	C12	192	Mandarin type
A118		Satsuma mandarin	Juman Unshu	315	235	372	303	210	141	148	130	269	253	164	C12	192	Mandarin type
A119		Satsuma mandarin	Jutaro Unshu NC	315	235	372	303	210	141	148	130	269	253	164	C12	192	Mandarin type
A120		Satsuma mandarin	Kinokuni Unshu	315	235	372	303	210	141	148	130	269	253	164	C12	192	Mandarin type
A121		Satsuma mandarin	Kuno Unshu	315	235	372	303	210	141	148	130	269	253	164	C12	192	Mandarin type
A122		Satsuma mandarin	Miyagawa Wase	315	235	372	303	210	141	148	130	269	253	164	C12	192	Mandarin type
A123		Satsuma mandarin	Nagahashi Unshu NC	315	235	372	303	210	141	148	130	269	253	164	C12	192	Mandarin type
A124		Satsuma mandarin	Niu Unshu	315	235	372	303	210	141	148	130	269	253	164	C12	192	Mandarin type
A125	*	Satsuma mandarin	Okitsu Wase (NC)	315	235	372	303	210	141	148	130	269	253	164	C12	192	Mandarin type
A126		Satsuma mandarin	Original tree	315	235	372	303	210	141	148	130	269	253	164	C12	192	Mandarin type
A127		Satsuma mandarin	Otsu-4 (NC)	315	235	372	303	210	141	148	130	269	253	164	C12	192	Mandarin type
A128		Satsuma mandarin	Shirakawa Unshu	315	235	372	303	210	141	148	130	269	253	164	C12	192	Mandarin type
A129		Satsuma mandarin	Sugiyama Unshu	315	235	372	303	210	141	148	130	269	253	164	C12	192	Mandarin type
A130		Satsuma mandarin	Suruga Beni	315	235	372	303	210	141	148	130	269	253	164	C12	192	Mandarin type
A131		Satsuma mandarin	Ueno Wase	315	235	372	303	210	141	148	130	269	253	164	C12	192	Mandarin type
A132		Satsuma mandarin	Yamada Unshu NC	315	235	372	303	210	141	148	130	269	253	164	C12	192	Mandarin type
A133		Satsuma mandarin	Yamashita Beni	315	235	372	303	210	141	148	130	269	253	164	C12	192	Mandarin type
A134	*	Satsuma Kikoku		315	233	377	299	210	143	148	130	274	269	172	C08	2	Satsumakikoku type
A135	*	Shiikuwasha		315	235	370	305	210	141	148	130	269	261	164	C13	7	Sunki type
A136	*	Shiikuwasha	Oogimi Kuganii	335	234	374	310	210	142	148	130	269	253	164	C16	1	Ogimikugani type
A137	*	Shunkokan		315	234	379	298	210	143	148	130	274	269	172	C04	67	pummelo type
A138	*	Sokitsu		315	235	372	303	210	141	148	130	269	253	164	C12	192	Mandarin type
A139		Sour orange	Bouquet de Fleurs	315	233	377	305	210	141	148	130	274	269	172	C06	13	Lemon type
A140		Sour orange	Chaozhouchen	315	233	377	305	210	141	148	130	274	269	172	C06	13	Lemon type

A141	*	Sour orange	Daidai	315	233	377	305	210	141	148	130	274	269	172	C06	13	Lemon type
A142		Sour orange	Kaiseito	315	233	377	305	210	141	148	130	274	269	172	C06	13	Lemon type
A143		Sour orange	Za daidai	315	233	377	305	210	141	148	130	274	269	172	C06	13	Lemon type
A144	*	Sudachi		315	234	378	304	210	142	148	130	274	269	172	C18	6	Koji type
A145	*	Suisho buntan		315	234	379	298	210	143	148	130	274	269	172	C04	67	pummelo type
A146	*	Sunki		315	235	370	305	210	141	148	130	269	261	164	C13	7	Sunki type
A147	*	Suruga Yuko		315	235	372	303	210	141	148	130	269	253	164	C12	192	Mandarin type
A148		Sweet orange	Cadenera	315	233	377	298	210	143	148	130	274	269	172	C07	45	Sweet orange type
A149		Sweet orange	Cara Cara	315	233	377	298	210	143	148	130	274	269	172	C07	45	Sweet orange type
A150		Sweet orange	Crescent	315	233	377	298	210	143	148	130	274	269	172	C07	45	Sweet orange type
A151		Sweet orange	Hamlin	315	233	377	298	210	143	148	130	274	269	172	C07	45	Sweet orange type
A152		Sweet orange	Jin Cheng	315	233	377	298	210	143	148	130	274	269	172	C07	45	Sweet orange type
A153		Sweet orange	Joppa	315	233	377	298	210	143	148	130	274	269	172	C07	45	Sweet orange type
A154		Sweet orange	Mediterranean Sweet Orange	315	233	377	298	210	143	148	130	274	269	172	C07	45	Sweet orange type
A155		Sweet orange	Moro NC	315	233	377	298	210	143	148	130	274	269	172	C07	45	Sweet orange type
A156		Sweet orange	Parson Brown	315	233	377	298	210	143	148	130	274	269	172	C07	45	Sweet orange type
A157		Sweet orange	Pineapple	315	233	377	298	210	143	148	130	274	269	172	C07	45	Sweet orange type
A158		Sweet orange	Santa Catarina	315	233	377	298	210	143	148	130	274	269	172	C07	45	Sweet orange type
A159		Sweet orange	Seike navel	315	233	377	298	210	143	148	130	274	269	172	C07	45	Sweet orange type
A160		Sweet orange	Shamouti	315	233	377	298	210	143	148	130	274	269	172	C07	45	Sweet orange type
A161		Sweet orange	Tong Zi Gan	315	233	377	298	210	143	148	130	274	269	172	C07	45	Sweet orange type
A162	*	Sweet orange	Trovita	315	233	377	298	210	143	148	130	274	269	172	C07	45	Sweet orange type
A163		Sweet orange	Washington navel	315	233	377	298	210	143	148	130	274	269	172	C07	45	Sweet orange type
A164		Sweet orange	Valencia	315	233	377	298	210	143	148	130	274	269	172	C07	45	Sweet orange type
A165		Sweet orange	Wu Yue Cheng	315	233	377	298	210	143	148	130	274	269	172	C07	45	Sweet orange type
A166		Sweet orange	Xue Gan	315	233	377	298	210	143	148	130	274	269	172	C07	45	Sweet orange type
A167		Sweet orange	Yinzi Gan	315	233	377	298	210	143	148	130	274	269	172	C07	45	Sweet orange type
A168		Tachibana		335	233	374	310	210	142	148	130	269	253	164	C14	11	Tachibana type
A169		Tachibana	Anettaishijou	335	233	374	310	210	142	148	130	269	253	164	C14	11	Tachibana type
A170		Tachibana	Botanical garden	335	233	374	310	210	142	148	130	269	253	164	C14	11	Tachibana type
A171		Tachibana	Hananoiwaya	335	233	374	310	210	142	148	130	269	253	164	C14	11	Tachibana type
A172	*	Tachibana	Heda 1	335	233	374	310	210	142	148	130	269	253	164	C14	11	Tachibana type
A173		Tachibana	Heda 2	335	233	374	310	210	142	148	130	269	253	164	C14	11	Tachibana type
A174	*	Tachibana	Ishinami Minka	315	234	371	305	210	141	148	130	269	261	164	C15	1	Tachibana_C type
A175	*	Tachibana	Ishinami No.1	335	233	374	310	210	142	148	130	269	253	164	C14	11	Tachibana type
A176		Tachibana	Ishinami No.2	335	233	374	310	210	142	148	130	269	253	164	C14	11	Tachibana type
A177		Tachibana	Oodomari OP-2	335	233	374	310	210	142	148	130	269	253	164	C14	11	Tachibana type
A178		Tachibana	Okitsu	335	233	374	310	210	142	148	130	269	253	164	C14	11	Tachibana type
A179		Tachibana	Reizanji	335	233	374	310	210	142	148	130	269	253	164	C14	11	Tachibana type
A180		Tajima mikan		315	234	378	304	210	142	148	130	274	269	172	C18	6	Koji type
A181		Takumanatsukunenbo		315	233	377	305	210	141	148	130	274	269	172	C06	13	Lemon type
A182		Tankan		315	235	370	305	210	141	148	130	269	261	164	C13	7	Sunki type
A183	*	Tankan	Taishun	315	235	370	305	210	141	148	130	269	261	164	C13	7	Sunki type
A184		Tankan	Tarumizu 1	315	235	370	305	210	141	148	130	269	261	164	C13	7	Sunki type
A185		Tankan	T-132	315	235	370	305	210	141	148	130	269	261	164	C13	7	Sunki type
A186	*	Temple		315	235	372	303	210	141	148	130	269	253	164	C12	192	Mandarin type
A187	*	Tengu		315	233	377	298	210	144	148	130	274	269	172	C05	17	Hyuganatsu type
A188	*	Tizon		315	233	377	298	210	143	148	130	274	269	172	C07	45	Sweet orange type
A189		Toukan		315	234	378	304	210	142	148	130	274	269	172	C18	6	Koji type
A190		Tookunin		315	233	377	298	210	143	148	130	274	269	172	C07	45	Sweet orange type
A191	*	Tosa buntan		315	234	379	298	210	143	148	130	274	269	172	C04	67	pummelo type
A192	*	Twukkuni		315	233	377	298	210	143	148	130	274	269	172	C07	45	Sweet orange type
A193		Twukkunin		315	233	377	298	210	143	148	130	274	269	172	C07	45	Sweet orange type
A194		Twukunihu		315	233	377	298	210	143	148	130	274	269	172	C07	45	Sweet orange type
A195		Twuukuribu		315	233	377	298	210	143	148	130	274	269	172	C07	45	Sweet orange type
A196	*	Uchimurasaki		315	234	379	298	210	143	148	130	274	269	172	C04	67	pummelo type
A197	*	Ujukitsu		315	234	379	298	210	143	148	130	274	269	172	C04	67	pummelo type
A198	*	Unzoki		315	233	377	298	210	143	148	130	274	269	172	C07	45	Sweet orange type
A199	*	USSR Tangelo		315	235	372	303	210	141	148	130	269	253	164	C12	192	Mandarin type
A200	*	Willowleaf mandarin	Mediterranean mandarin	315	235	372	303	210	141	148	130	269	253	164	C12	192	Mandarin type
A201	*	Willowleaf mandarin	Willowleaf mandarin	315	235	372	303	210	141	148	130	269	253	164	C12	192	Mandarin type
A202	*	Yamabuki		315	234	379	298	210	143	148	130	274	269	172	C04	67	pummelo type
A203	*	Yamamikan		315	233	377	298	210	143	148	130	274	269	172	C07	45	Sweet orange type
A204	*	Yatsushiro		315	233	377	298	210	143	148	130	274	269	172	C07	45	Sweet orange type
A205	*	Yuge hyoukan		315	234	379	298	210	143	148	130	274	269	172	C04	67	pummelo type
A206	*	Youpi ju (Yuhikitsu)		315	235	372	303	210	141	148	130	269	253	164	C12	192	Mandarin type
A207	*	Yuukunibu		315	234	379	298	210	143	148	130	274	269	172	C04	67	pummelo type
A208	*	Yuzu		315	233	371	311	210	139	159	129	274	261	172	C09	3	Yuzu type

B: hybrid varieties

No.	Rep 1)	Variety	Chloroplast markers								Mitochondria markers			Class	Same GT	Organellar type
			CSS03	CSS04	CSL01	CSL09	ccmp2.2	ccmp6.2	ccmp7.2	ccmp10.2	*rrn* 5/ *rrn* 18-1	*nad* 2/ 4-3	*nad* 7/ 1-2			
B001	*	Akemi	315	235	372	303	210	141	148	130	269	253	164	C12	192	Mandarin type
B002	*	Aki Marine	315	235	372	303	210	141	148	130	269	253	164	C12	192	Mandarin type
B003	*	Aki Tangor	315	235	372	303	210	141	148	130	269	253	164	C12	192	Mandarin type
B004	*	All spice	315	234	379	298	210	143	148	130	274	269	172	C04	67	pummelo type
B005	*	Amaka	315	235	372	303	210	141	148	130	269	253	164	C12	192	Mandarin type
B006	*	Ariake	315	233	377	298	210	143	148	130	274	269	172	C07	45	Sweet orange type
B007	*	Asumi	315	235	372	303	210	141	148	130	269	253	164	C12	192	Mandarin type
B008	*	Aurastar	315	234	379	298	210	143	148	130	274	269	172	C04	67	pummelo type
B009	*	Awa Orange	315	233	377	298	210	144	148	130	274	269	172	C05	17	Hyuganatsu type
B010	*	Benibae	315	235	372	303	210	141	148	130	269	253	164	C12	192	Mandarin type
B011	*	Benimadoka	315	234	379	298	210	143	148	130	274	269	172	C04	67	pummelo type
B012	*	Chandler pummelo	315	234	379	298	210	143	148	130	274	269	172	C04	67	pummelo type
B013	*	Ehimekashi 28	315	235	372	303	210	141	148	130	269	253	164	C12	192	Mandarin type
B014	*	Encore	315	235	372	303	210	141	148	130	269	253	164	C12	192	Mandarin type
B015	*	Fairchild	315	235	372	303	210	141	148	130	269	253	164	C12	192	Mandarin type
B016	*	Fortune	315	235	372	303	210	141	148	130	269	253	164	C12	192	Mandarin type
B017	*	Harehime	315	235	372	303	210	141	148	130	269	253	164	C12	192	Mandarin type
B018	*	Hareyaka	315	235	372	303	210	141	148	130	269	253	164	C12	192	Mandarin type
B019	*	Haruhi	315	235	372	303	210	141	148	130	269	253	164	C12	192	Mandarin type
B020	*	Haruka	315	233	377	298	210	144	148	130	274	269	172	C05	17	Hyuganatsu type
B021	*	Harumi	315	235	372	303	210	141	148	130	269	253	164	C12	192	Mandarin type
B022	*	Hayaka	315	235	372	303	210	141	148	130	269	253	164	C12	192	Mandarin type
B023	*	Hayasaki	315	234	379	298	210	143	148	130	274	269	172	C04	67	pummelo type
B024	*	Himekoharu	315	235	372	303	210	141	148	130	269	253	164	C12	192	Mandarin type
B025	*	Hiroshimakaken 11	315	235	372	303	210	141	148	130	269	253	164	C12	192	Mandarin type
B026	*	Honey	315	235	372	303	210	141	148	130	269	253	164	C12	192	Mandarin type
B027	*	Kanpei	315	235	372	303	210	141	148	130	269	253	164	C12	192	Mandarin type
B028	*	Kara mandarin	315	235	372	303	210	141	148	130	269	253	164	C12	192	Mandarin type
B029	*	Kincy mandarin	315	235	372	303	210	141	148	130	269	253	164	C12	192	Mandarin type
B030	*	Kinnow mandarin	315	235	372	303	210	141	148	130	269	253	164	C12	192	Mandarin type
B031	*	Kiyomi	315	235	372	303	210	141	148	130	269	253	164	C12	192	Mandarin type
B032		Kuchinotsu-41 *	315	233	377	298	210	144	148	130	274	269	172	C05	17	Hyuganatsu type
B033	*	Lee	315	235	372	303	210	141	148	130	269	253	164	C12	192	Mandarin type
B034	*	May pummelo	315	234	379	298	210	143	148	130	274	269	172	C04	67	pummelo type
B035	*	Mihaya	315	235	372	303	210	141	148	130	269	253	164	C12	192	Mandarin type
B036	*	Mihocore	315	235	372	303	210	141	148	130	269	253	164	C12	192	Mandarin type
B037	*	Mineola	315	234	379	298	210	143	148	130	274	269	172	C04	67	pummelo type
B038	*	Nankou	315	235	372	303	210	141	148	130	269	253	164	C12	192	Mandarin type
B039	*	Nishinokaori	315	235	372	303	210	141	148	130	269	253	164	C12	192	Mandarin type
B040	*	Nou 5 gou	315	235	372	303	210	141	148	130	269	253	164	C12	192	Mandarin type
B041	*	Nou 6 gou	315	235	372	303	210	141	148	130	269	253	164	C12	192	Mandarin type
B042	*	Nou 7 gou	315	234	379	298	210	143	148	130	274	269	172	C04	67	pummelo type
B043	*	Nova	315	235	372	303	210	141	148	130	269	253	164	C12	192	Mandarin type
B044	*	Ooitakaken-4	315	235	372	303	210	141	148	130	269	253	164	C12	192	Mandarin type
B045	*	Orland	315	234	379	298	210	143	148	130	274	269	172	C04	67	pummelo type
B046		Oroblanco	315	234	379	298	210	143	148	130	274	269	172	C04	67	pummelo type
B047	*	Osceola	315	235	372	303	210	141	148	130	269	253	164	C12	192	Mandarin type
B048	*	Page	315	234	379	298	210	143	148	130	274	269	172	C04	67	pummelo type
B049	*	Pearl	315	234	379	298	210	143	148	130	274	269	172	C04	67	pummelo type
B050	*	Pixie mandarin	315	235	372	303	210	141	148	130	269	253	164	C12	192	Mandarin type
B051	*	Robinson	315	235	372	303	210	141	148	130	269	253	164	C12	192	Mandarin type
B052	*	Saga Mandarin	315	235	372	303	210	141	148	130	269	253	164	C12	192	Mandarin type
B053		Sagakashi 34	315	235	372	303	210	141	148	130	269	253	164	C12	192	Mandarin type
B054	*	Seihou	315	235	372	303	210	141	148	130	269	253	164	C12	192	Mandarin type
B055	*	Seinannohikari	315	235	372	303	210	141	148	130	269	253	164	C12	192	Mandarin type
B056	*	Seminor	315	234	379	298	210	143	148	130	274	269	172	C04	67	pummelo type
B057	*	Setomi	315	235	372	303	210	141	148	130	269	253	164	C12	192	Mandarin type
B058	*	Shiranuhi	315	235	372	303	210	141	148	130	269	253	164	C12	192	Mandarin type
B059	*	Southern Yellow	315	233	377	298	210	144	148	130	274	269	172	C05	17	Hyuganatsu type
B060	*	Summer Fresh	315	234	379	298	210	143	148	130	274	269	172	C04	67	pummelo type
B061	*	Sweet Spring	315	235	372	303	210	141	148	130	269	253	164	C12	192	Mandarin type
B062	*	Tamami	315	235	372	303	210	141	148	130	269	253	164	C12	192	Mandarin type
B063	*	Tanikawa Buntan	315	233	377	298	210	144	148	130	274	269	172	C05	17	Hyuganatsu type
B064	*	Tsunokagayaki	315	235	372	303	210	141	148	130	269	253	164	C12	192	Mandarin type
B065	*	Tsunokaori	315	235	372	303	210	141	148	130	269	253	164	C12	192	Mandarin type
B066	*	Tsunonozomi	315	235	372	303	210	141	148	130	269	253	164	C12	192	Mandarin type
B067	*	Willking	315	235	372	303	210	141	148	130	269	253	164	C12	192	Mandarin type
B068	*	Yellow Pummelo	315	234	379	298	210	143	148	130	274	269	172	C04	67	pummelo type

B069	*	Youkou	315	235	372	303	210	141	148	130	269	253	164	C12	192	Mandarin type
B070	*	E-647	315	235	372	303	210	141	148	130	269	253	164	C12	192	Mandarin type
B071	*	EnOw21	315	235	372	303	210	141	148	130	269	253	164	C12	192	Mandarin type
B072	*	H・FD-1	315	234	379	298	210	143	148	130	274	269	172	C04	67	pummelo type
B073	*	HF9	315	235	372	303	210	141	148	130	269	253	164	C12	192	Mandarin type
B074	*	KyOw14	315	235	372	303	210	141	148	130	269	253	164	C12	192	Mandarin type
B075	*	KyOw21	315	235	372	303	210	141	148	130	269	253	164	C12	192	Mandarin type
B076	*	No.1408	315	235	372	303	210	141	148	130	269	253	164	C12	192	Mandarin type
B077	*	Okitsu-46	315	235	372	303	210	141	148	130	269	253	164	C12	192	Mandarin type
B078	*	Okitsu-56	315	235	372	303	210	141	148	130	269	253	164	C12	192	Mandarin type

C: Selected strains

No.	Rep 1)	Lines	Chloroplast markers								Mitochondria markers			Class	Same GT	Organellar type
			CSS03	CSS04	CSL01	CSL09	ccmp2.2	ccmp6.2	ccmp7.2	ccmp10.2	*rrn* 5/ *rrn* 18-1	*nad* 2/ 4-3	*nad* 7/ 1-2			
C001	*	Strain 01	315	235	372	303	210	141	148	130	269	253	164	C12	192	Mandarin type
C002	*	Strain 02	315	235	372	303	210	141	148	130	269	253	164	C12	192	Mandarin type
C003	*	Strain 03	315	235	372	303	210	141	148	130	269	253	164	C12	192	Mandarin type
C004	*	Strain 04	315	233	377	305	210	141	148	130	274	269	172	C06	13	Lemon type
C005	*	Strain 05	315	235	372	303	210	141	148	130	269	253	164	C12	192	Mandarin type
C006	*	Strain 06	315	235	372	303	210	141	148	130	269	253	164	C12	192	Mandarin type
C007	*	Strain 07	315	234	379	298	210	143	148	130	274	269	172	C04	67	pummelo type
C008	*	Strain 08	315	235	372	303	210	141	148	130	269	253	164	C12	192	Mandarin type
C009	*	Strain 09	315	235	372	303	210	141	148	130	269	253	164	C12	192	Mandarin type
C010	*	Strain 10	315	235	372	303	210	141	148	130	269	253	164	C12	192	Mandarin type
C011	*	Strain 11	315	235	372	303	210	141	148	130	269	253	164	C12	192	Mandarin type
C012	*	Strain 12	315	235	372	303	210	141	148	130	269	253	164	C12	192	Mandarin type
C013	*	Strain 13	315	235	372	303	210	141	148	130	269	253	164	C12	192	Mandarin type
C014	*	Strain 14	315	235	372	303	210	141	148	130	269	253	164	C12	192	Mandarin type
C015	*	Strain 15	315	235	372	303	210	141	148	130	269	253	164	C12	192	Mandarin type
C016	*	Strain 16	315	234	379	298	210	143	148	130	274	269	172	C04	67	pummelo type
C017	*	Strain 17	315	235	372	303	210	141	148	130	269	253	164	C12	192	Mandarin type
C018	*	Strain 18	315	235	372	303	210	141	148	130	269	253	164	C12	192	Mandarin type
C019	*	Strain 19	315	235	372	303	210	141	148	130	269	253	164	C12	192	Mandarin type
C020	*	Strain 20	315	235	372	303	210	141	148	130	269	253	164	C12	192	Mandarin type
C021	*	Strain 21	315	235	372	303	210	141	148	130	269	253	164	C12	192	Mandarin type
C022	*	Strain 22	315	235	372	303	210	141	148	130	269	253	164	C12	192	Mandarin type
C023	*	Strain 23	315	235	372	303	210	141	148	130	269	253	164	C12	192	Mandarin type
C024	*	Strain 24	315	235	372	303	210	141	148	130	269	253	164	C12	192	Mandarin type
C025	*	Strain 25	315	235	372	303	210	141	148	130	269	253	164	C12	192	Mandarin type
C026	*	Strain 26	315	235	372	303	210	141	148	130	269	253	164	C12	192	Mandarin type
C027	*	Strain 27	315	235	372	303	210	141	148	130	269	253	164	C12	192	Mandarin type
C028	*	Strain 28	315	235	372	303	210	141	148	130	269	253	164	C12	192	Mandarin type
C029	*	Strain 29	315	235	372	303	210	141	148	130	269	253	164	C12	192	Mandarin type
C030	*	Strain 30	315	235	372	303	210	141	148	130	269	253	164	C12	192	Mandarin type
C031	*	Strain 31	315	235	372	303	210	141	148	130	269	253	164	C12	192	Mandarin type
C032	*	Strain 32	315	235	372	303	210	141	148	130	269	253	164	C12	192	Mandarin type
C033	*	Strain 33	315	235	372	303	210	141	148	130	269	253	164	C12	192	Mandarin type
C034	*	Strain 34	315	235	372	303	210	141	148	130	269	253	164	C12	192	Mandarin type
C035	*	Strain 35	315	235	372	303	210	141	148	130	269	253	164	C12	192	Mandarin type
C036	*	Strain 36	315	235	372	303	210	141	148	130	269	253	164	C12	192	Mandarin type
C037	*	Strain 37	315	235	372	303	210	141	148	130	269	253	164	C12	192	Mandarin type
C038	*	Strain 38	315	235	372	303	210	141	148	130	269	253	164	C12	192	Mandarin type
C039	*	Strain 39	315	235	372	303	210	141	148	130	269	253	164	C12	192	Mandarin type
C040	*	Strain 40	315	235	372	303	210	141	148	130	269	253	164	C12	192	Mandarin type
C041	*	Strain 41	315	235	372	303	210	141	148	130	269	253	164	C12	192	Mandarin type
C042	*	Strain 42	315	235	372	303	210	141	148	130	269	253	164	C12	192	Mandarin type
C043	*	Strain 43	315	235	372	303	210	141	148	130	269	253	164	C12	192	Mandarin type
C044	*	Strain 44	315	235	372	303	210	141	148	130	269	253	164	C12	192	Mandarin type
C045	*	Strain 45	315	235	372	303	210	141	148	130	269	253	164	C12	192	Mandarin type
C046	*	Strain 46	315	235	372	303	210	141	148	130	269	253	164	C12	192	Mandarin type
C047	*	Strain 47	315	235	372	303	210	141	148	130	269	253	164	C12	192	Mandarin type
C048	*	Strain 48	315	235	372	303	210	141	148	130	269	253	164	C12	192	Mandarin type
C049	*	Strain 49	315	235	372	303	210	141	148	130	269	253	164	C12	192	Mandarin type
C050	*	Strain 50	315	235	372	303	210	141	148	130	269	253	164	C12	192	Mandarin type
C051	*	Strain 51	315	233	377	298	210	144	148	130	274	269	172	C05	17	Hyuganatsu type
C052	*	Strain 52	315	233	377	298	210	144	148	130	274	269	172	C05	17	Hyuganatsu type
C053	*	Strain 53	315	233	377	298	210	144	148	130	274	269	172	C05	17	Hyuganatsu type
C054	*	Strain 54	315	234	379	298	210	143	148	130	274	269	172	C04	67	pummelo type
C055	*	Strain 55	315	235	372	303	210	141	148	130	269	253	164	C12	192	Mandarin type
C056	*	Strain 56	315	235	372	303	210	141	148	130	269	253	164	C12	192	Mandarin type

C057	*	Strain 57	315	235	372	303	210	141	148	130	269	253	164	C12	192	Mandarin type
C058	*	Strain 58	315	234	379	298	210	143	148	130	274	269	172	C04	67	pummelo type
C059	*	Strain 59	315	235	372	303	210	141	148	130	269	253	164	C12	192	Mandarin type
C060	*	Strain 60	315	234	379	298	210	143	148	130	274	269	172	C04	67	pummelo type
C061	*	Strain 61	315	234	379	298	210	143	148	130	274	269	172	C04	67	pummelo type
C062	*	Strain 62	315	234	379	298	210	143	148	130	274	269	172	C04	67	pummelo type
C063	*	Strain 63	315	235	372	303	210	141	148	130	269	253	164	C12	192	Mandarin type
C064	*	Strain 64	315	235	372	303	210	141	148	130	269	253	164	C12	192	Mandarin type
C065	*	Strain 65	315	235	372	303	210	141	148	130	269	253	164	C12	192	Mandarin type
C066	*	Strain 66	315	235	372	303	210	141	148	130	269	253	164	C12	192	Mandarin type
C067	*	Strain 67	315	235	372	303	210	141	148	130	269	253	164	C12	192	Mandarin type
C068	*	Strain 68	315	235	372	303	210	141	148	130	269	253	164	C12	192	Mandarin type
C069	*	Strain 69	315	235	372	303	210	141	148	130	269	253	164	C12	192	Mandarin type
C070	*	Strain 70	315	235	372	303	210	141	148	130	269	253	164	C12	192	Mandarin type
C071	*	Strain 71	315	234	379	298	210	143	148	130	274	269	172	C04	67	pummelo type
C072	*	Strain 72	315	235	372	303	210	141	148	130	269	253	164	C12	192	Mandarin type
C073	*	Strain 73	315	235	372	303	210	141	148	130	269	253	164	C12	192	Mandarin type
C074	*	Strain 74	315	235	372	303	210	141	148	130	269	253	164	C12	192	Mandarin type
C075	*	Strain 75	315	235	372	303	210	141	148	130	269	253	164	C12	192	Mandarin type
C076	*	Strain 76	315	235	372	303	210	141	148	130	269	253	164	C12	192	Mandarin type
C077	*	Strain 77	315	235	372	303	210	141	148	130	269	253	164	C12	192	Mandarin type
C078	*	Strain 78	315	235	372	303	210	141	148	130	269	253	164	C12	192	Mandarin type
C079	*	Strain 79	315	235	372	303	210	141	148	130	269	253	164	C12	192	Mandarin type
C080	*	Strain 80	315	235	372	303	210	141	148	130	269	253	164	C12	192	Mandarin type
C081	*	Strain 81	315	235	372	303	210	141	148	130	269	253	164	C12	192	Mandarin type
C082	*	Strain 82	315	235	372	303	210	141	148	130	269	253	164	C12	192	Mandarin type
C083	*	Strain 83	315	235	372	303	210	141	148	130	269	253	164	C12	192	Mandarin type
C084	*	Strain 84	315	235	372	303	210	141	148	130	269	253	164	C12	192	Mandarin type
C085	*	Strain 85	315	235	372	303	210	141	148	130	269	253	164	C12	192	Mandarin type

D. Numbers of mismatched genotypes between all pairs of cytotype classes

Class	Cytotypes	Classes																	
		C01	C02	C03	C04	C05	C06	C07	C08	C09	C10	C11	C12	C13	C14	C15	C16	C17	C18
C01	C. ichangensis type	0	7	9	8	8	8	8	8	4	8	11	10	9	11	8	11	9	8
C02	Mexican lime	7	0	6	5	5	4	5	5	6	5	8	8	6	8	6	9	7	6
C03	Limonia type	9	6	0	7	6	5	6	6	7	6	4	4	2	5	3	6	1	6
C04	pummelo type	8	5	7	0	3	4	2	3	7	3	8	7	7	8	6	7	6	3
C05	Hyuganatsu type	8	5	6	3	0	2	1	2	6	1	7	7	7	7	7	8	7	4
C06	Lemon type	8	4	5	4	2	0	2	2	6	2	6	6	5	7	5	8	6	4
C07	Sweet orange type	8	5	6	2	1	2	0	1	6	1	7	7	7	7	7	8	7	4
C08	Satsumakikoku type	8	5	6	3	2	2	1	0	6	2	7	7	7	7	7	8	7	4
C09	Yuzu type	4	6	7	7	6	6	6	6	0	6	9	9	8	9	7	10	8	7
C10	Ichang lemon type	8	5	6	3	1	2	1	2	6	0	7	7	7	7	7	8	7	4
C11	Kunenbo_B type	11	8	4	8	7	6	7	7	9	7	0	4	5	1	5	2	5	8
C12	Mandarin type	10	8	4	7	7	6	7	7	9	7	4	0	3	5	4	5	4	7
C13	Sunki type	9	6	2	7	7	5	7	7	8	7	5	3	0	6	2	6	2	7
C14	Tachibana type	11	8	5	8	7	7	7	7	9	7	1	5	6	0	6	1	6	7
C15	Tachibana C type	8	6	3	6	7	5	7	7	7	7	5	4	2	6	0	5	2	6
C16	Ogimikugani type	11	9	6	7	8	8	8	8	10	8	2	5	6	1	5	0	5	6
C17	Cleopatra type	9	7	1	6	7	6	7	7	8	7	5	4	2	6	2	5	0	5
C18	Koji type	8	6	6	3	4	4	4	4	7	4	8	7	7	7	6	6	5	0
