# Supplementary material for: Hybrid Origins of Citrus Varieties Inferred from DNA Marker Analysis of Nuclear and Organelle Genomes
Source: PLoS One. 2016 Nov 30;11(11):e0166969. doi: 10.1371/journal.pone.0166969 (PMC5130255; doi:10.1371/journal.pone.0166969)
Supplement: S10 Table — (PDF) [file pone.0166969.s013.pdf]

**S10 Table. Numbers of DNA markers that inconsistent on allele sharing test between all combinations of representative native varietites**

| | | | | | | | | | | | | | | | | | | | | | | | | | | | | | | | | | | | | |
|---|---|---|---|---|---|---|---|---|---|---|---|---|---|---|---|---|---|---|---|---|---|---|---|---|---|---|---|---|---|---|---|---|---|---|---|---|
| Inferred combinations by allele sharing test | | | | | | | | | | | | | | | | | | | | | | | | | | | | | | | | | | | | |
| No unshared alleles | | 74 | | 3 | 0 | 1 | 0 | 2 | 1 | 0 | 2 | 1 | 2 | 0 | 2 | 0 | 2 | 1 | 1 | 0 | 1 | 1 | 1 | 1 | 1 | 0 | 1 | 1 | 1 | 0 | 0 | 2 | 0 | 1 | 1 | 2 |
| Up to 4 unshared alleles allowed | | 92 | | 8 | 1 | 4 | 0 | 5 | 2 | 0 | 5 | 1 | 6 | 0 | 3 | 0 | 7 | 3 | 3 | 4 | 4 | 3 | 6 | 3 | 2 | 1 | 1 | 5 | 1 | 1 | 1 | 8 | 2 | 1 | 3 | 4 |
| | | | Variety | Andoukan | Anseikan | Asahikan | Banpeiyu | Ben Di Zao | Bergamot | Binkitsu | Clementine | Cleopatra | Cravo mandarin | Dada | Dancy | Egami buntan | Fukure mikan | Funadoko | Genshokan | Girimikan | Grapefruit | Hanayu | Hassaku | Henka mikan | Hickson | Hirado buntan | Hiroshimanatsubuntan | Hyoukan | Hyuganatsu | Ichanchii | Ichang lemon | Iyo | Jabara | Jabon | Kabosu | Kabuchi |
| | | | ID | A001 | A002 | A003 | A004 | A005 | A006 | A008 | A009 | A013 | A014 | A015 | A016 | A018 | A019 | A021 | A022 | A023 | A024 | A027 | A028 | A030 | A031 | A032 | A033 | A035 | A036 | A041 | A042 | A044 | A045 | A046 | A047 | A048 |
| No | ID | Variety | ND | 0 | 0 | 0 | 0 | 0 | 0 | 0 | 0 | 0 | 0 | 0 | 0 | 1 | 0 | 0 | 0 | 0 | 0 | 2 | 0 | 0 | 0 | 0 | 0 | 0 | 0 | 0 | 0 | 0 | 2 | 0 | 0 | 0 |
| 1 | A001 | Andoukan | 0 | 0 | 10 | 5 | 10 | 11 | 14 | 13 | 17 | 14 | 11 | 12 | 11 | 24 | 8 | 6 | 13 | 11 | 7 | 26 | 8 | 11 | 14 | 14 | 7 | 7 | 11 | 25 | 17 | 3 | 15 | 10 | 11 | 12 |
| 2 | A002 | Anseikan | 0 | 10 | 0 | 13 | 6 | 47 | 29 | 31 | 54 | 58 | 45 | 15 | 54 | 18 | 48 | 20 | 59 | 41 | 6 | 59 | 3 | 41 | 51 | 12 | 11 | 11 | 14 | 39 | 22 | 19 | 28 | 21 | 41 | 25 |
| 3 | A003 | Asahikan | 0 | 5 | 13 | 0 | 9 | 15 | 21 | 22 | 21 | 23 | 14 | 13 | 24 | 16 | 18 | 7 | 24 | 16 | 6 | 44 | 9 | 17 | 23 | 11 | 8 | 6 | 15 | 30 | 19 | 6 | 18 | 9 | 14 | 9 |
| 4 | A004 | Banpeiyu | 0 | 10 | 6 | 9 | 0 | 68 | 33 | 45 | 66 | 75 | 63 | 29 | 72 | 12 | 60 | 29 | 72 | 51 | 8 | 70 | 9 | 54 | 69 | 8 | 15 | 14 | 30 | 54 | 23 | 24 | 44 | 26 | 49 | 46 |
| 5 | A005 | Ben Di Zao | 0 | 11 | 47 | 15 | 68 | 0 | 32 | 14 | 6 | 11 | 10 | 27 | 5 | 71 | 7 | 0 | 8 | 12 | 26 | 34 | 17 | 11 | 10 | 70 | 26 | 18 | 24 | 40 | 40 | 8 | 26 | 20 | 10 | 17 |
| 6 | A006 | Bergamot | 0 | 14 | 29 | 21 | 33 | 32 | 0 | 26 | 38 | 34 | 33 | 21 | 40 | 35 | 30 | 25 | 36 | 24 | 14 | 38 | 26 | 32 | 40 | 34 | 18 | 18 | 22 | 12 | 16 | 23 | 32 | 22 | 26 | 32 |
| 7 | A008 | Binkitsu | 0 | 13 | 31 | 22 | 45 | 14 | 26 | 0 | 17 | 24 | 19 | 21 | 20 | 50 | 8 | 15 | 22 | 13 | 18 | 23 | 10 | 12 | 19 | 44 | 27 | 5 | 13 | 34 | 21 | 10 | 14 | 18 | 14 | 13 |
| 8 | A009 | Clementine | 0 | 17 | 54 | 21 | 66 | 6 | 38 | 17 | 0 | 15 | 1 | 26 | 10 | 73 | 13 | 15 | 9 | 17 | 19 | 38 | 26 | 18 | 13 | 65 | 33 | 22 | 30 | 44 | 45 | 12 | 26 | 31 | 19 | 26 |
| 9 | A013 | Cleopatra | 0 | 14 | 58 | 23 | 75 | 11 | 34 | 24 | 15 | 0 | 13 | 29 | 13 | 80 | 10 | 24 | 13 | 17 | 35 | 33 | 30 | 18 | 12 | 77 | 39 | 28 | 25 | 41 | 49 | 16 | 30 | 31 | 26 | 24 |
| 10 | A014 | Cravo mandarin | 0 | 11 | 45 | 14 | 63 | 10 | 33 | 19 | 1 | 13 | 0 | 23 | 9 | 64 | 13 | 17 | 7 | 15 | 15 | 37 | 19 | 14 | 3 | 62 | 25 | 19 | 25 | 42 | 42 | 9 | 29 | 26 | 18 | 24 |
| 11 | A015 | Dada | 0 | 12 | 15 | 13 | 29 | 27 | 21 | 21 | 26 | 29 | 23 | 0 | 29 | 25 | 11 | 15 | 28 | 16 | 11 | 19 | 9 | 24 | 28 | 23 | 12 | 14 | 18 | 26 | 21 | 13 | 23 | 13 | 21 | 11 |
| 12 | A016 | Dancy | 0 | 11 | 54 | 24 | 72 | 5 | 40 | 20 | 10 | 13 | 9 | 29 | 0 | 78 | 11 | 15 | 7 | 12 | 25 | 37 | 23 | 16 | 5 | 72 | 38 | 23 | 29 | 46 | 52 | 0 | 30 | 33 | 18 | 27 |
| 13 | A018 | Egami buntan | 1 | 24 | 18 | 16 | 12 | 71 | 35 | 50 | 73 | 80 | 64 | 25 | 78 | 1 | 67 | 33 | 74 | 60 | 16 | 70 | 20 | 59 | 74 | 13 | 16 | 23 | 33 | 54 | 26 | 37 | 44 | 36 | 54 | 49 |
| 14 | A019 | Fukure mikan | 0 | 8 | 48 | 18 | 60 | 7 | 30 | 8 | 13 | 10 | 13 | 11 | 11 | 67 | 0 | 16 | 13 | 2 | 27 | 16 | 19 | 9 | 12 | 61 | 28 | 17 | 13 | 33 | 35 | 11 | 24 | 18 | 13 | 10 |
| 15 | A021 | Funadoko | 0 | 6 | 20 | 7 | 29 | 0 | 25 | 15 | 15 | 24 | 17 | 15 | 15 | 33 | 16 | 0 | 20 | 16 | 9 | 38 | 7 | 14 | 19 | 32 | 12 | 8 | 16 | 39 | 22 | 6 | 19 | 13 | 8 | 12 |
| 16 | A022 | Genshokan | 0 | 13 | 59 | 24 | 72 | 8 | 36 | 22 | 9 | 13 | 7 | 28 | 7 | 74 | 13 | 20 | 0 | 18 | 28 | 34 | 26 | 13 | 6 | 69 | 40 | 19 | 22 | 41 | 50 | 8 | 32 | 28 | 22 | 25 |
| 17 | A023 | Girimikan | 0 | 11 | 41 | 16 | 51 | 12 | 24 | 13 | 17 | 17 | 15 | 16 | 12 | 60 | 2 | 16 | 18 | 0 | 24 | 12 | 23 | 11 | 13 | 53 | 29 | 13 | 7 | 29 | 29 | 15 | 20 | 17 | 15 | 8 |
| 18 | A024 | Grapefruit | 0 | 7 | 6 | 6 | 8 | 26 | 14 | 18 | 19 | 35 | 15 | 11 | 25 | 16 | 27 | 9 | 28 | 24 | 0 | 42 | 8 | 24 | 21 | 13 | 10 | 5 | 14 | 30 | 13 | 12 | 22 | 11 | 15 | 24 |
| 19 | A027 | Hanayu | 2 | 26 | 59 | 44 | 70 | 34 | 38 | 23 | 38 | 33 | 37 | 19 | 37 | 70 | 16 | 38 | 34 | 12 | 42 | 0 | 42 | 18 | 34 | 72 | 48 | 37 | 16 | 29 | 22 | 34 | 19 | 21 | 17 | 19 |
| 20 | A028 | Hassaku | 0 | 8 | 3 | 9 | 9 | 17 | 26 | 10 | 26 | 30 | 19 | 9 | 23 | 20 | 19 | 7 | 26 | 23 | 8 | 42 | 0 | 15 | 25 | 12 | 11 | 7 | 9 | 39 | 19 | 4 | 19 | 14 | 21 | 14 |
| 21 | A030 | Henka mikan | 0 | 11 | 41 | 17 | 54 | 11 | 32 | 12 | 18 | 18 | 14 | 24 | 16 | 59 | 9 | 14 | 13 | 11 | 24 | 18 | 15 | 0 | 14 | 52 | 26 | 6 | 18 | 27 | 20 | 12 | 18 | 13 | 11 | 24 |
| 22 | A031 | Hickson | 0 | 14 | 51 | 23 | 69 | 10 | 40 | 19 | 13 | 12 | 3 | 28 | 5 | 74 | 12 | 19 | 6 | 13 | 21 | 34 | 25 | 14 | 0 | 68 | 33 | 18 | 29 | 44 | 48 | 5 | 26 | 31 | 21 | 25 |
| 23 | A032 | Hirado buntan | 0 | 14 | 12 | 11 | 8 | 70 | 34 | 44 | 65 | 77 | 62 | 23 | 72 | 13 | 61 | 32 | 69 | 53 | 13 | 72 | 12 | 52 | 68 | 0 | 12 | 17 | 29 | 54 | 25 | 27 | 42 | 31 | 51 | 44 |
| 24 | A033 | Hiroshimanatsubuntan | 0 | 7 | 11 | 8 | 15 | 26 | 18 | 27 | 33 | 39 | 25 | 12 | 38 | 16 | 28 | 12 | 40 | 29 | 10 | 48 | 11 | 26 | 33 | 12 | 0 | 15 | 10 | 29 | 17 | 12 | 23 | 15 | 27 | 19 |
| 25 | A035 | Hyoukan | 0 | 7 | 11 | 6 | 14 | 18 | 18 | 5 | 22 | 28 | 19 | 14 | 23 | 23 | 17 | 8 | 19 | 13 | 5 | 37 | 7 | 6 | 18 | 17 | 15 | 0 | 9 | 32 | 13 | 11 | 14 | 6 | 14 | 14 |
| 26 | A036 | Hyuganatsu | 0 | 11 | 14 | 15 | 30 | 24 | 22 | 13 | 30 | 25 | 25 | 18 | 29 | 33 | 13 | 16 | 22 | 7 | 14 | 16 | 9 | 18 | 29 | 29 | 10 | 9 | 0 | 28 | 25 | 13 | 17 | 16 | 22 | 11 |
| 27 | A041 | Ichanchii | 0 | 25 | 39 | 30 | 54 | 40 | 12 | 34 | 44 | 41 | 42 | 26 | 46 | 54 | 33 | 39 | 41 | 29 | 30 | 29 | 39 | 27 | 44 | 54 | 29 | 32 | 28 | 0 | 20 | 31 | 25 | 25 | 23 | 33 |
| 28 | A042 | Ichang lemon | 0 | 17 | 22 | 19 | 23 | 40 | 16 | 21 | 45 | 49 | 42 | 21 | 52 | 26 | 35 | 22 | 50 | 29 | 13 | 22 | 19 | 20 | 48 | 25 | 17 | 13 | 25 | 20 | 0 | 24 | 16 | 19 | 8 | 31 |
| 29 | A044 | Iyo | 0 | 3 | 19 | 6 | 24 | 8 | 23 | 10 | 12 | 16 | 9 | 13 | 0 | 37 | 11 | 6 | 8 | 15 | 12 | 34 | 4 | 12 | 5 | 27 | 12 | 11 | 13 | 31 | 24 | 0 | 14 | 15 | 10 | 10 |
| 30 | A045 | Jabara | 2 | 15 | 28 | 18 | 44 | 26 | 32 | 14 | 26 | 30 | 29 | 23 | 30 | 44 | 24 | 19 | 32 | 20 | 22 | 19 | 19 | 18 | 26 | 42 | 23 | 14 | 17 | 25 | 16 | 14 | 2 | 19 | 12 | 20 |
| 31 | A046 | Jabon | 0 | 10 | 21 | 9 | 26 | 20 | 22 | 18 | 31 | 31 | 26 | 13 | 33 | 36 | 18 | 13 | 28 | 17 | 11 | 21 | 14 | 13 | 31 | 31 | 15 | 6 | 16 | 25 | 19 | 15 | 19 | 0 | 10 | 14 |

| | | | | | | | | | | | | | | | | | | | | | | | | | | | | | | | | | | | | |
|---|---|---|---|---|---|---|---|---|---|---|---|---|---|---|---|---|---|---|---|---|---|---|---|---|---|---|---|---|---|---|---|---|---|---|---|---|
| 32 | A047 | Kabosu | 0 | 11 | 41 | 14 | 49 | 10 | 26 | 14 | 19 | 26 | 18 | 21 | 18 | 54 | 13 | 8 | 22 | 15 | 15 | 17 | 21 | 11 | 21 | 51 | 27 | 14 | 22 | 23 | 8 | 10 | 12 | 10 | 0 | 21 |
| 33 | A048 | Kabuchi | 0 | 12 | 25 | 9 | 46 | 17 | 32 | 13 | 26 | 24 | 24 | 11 | 27 | 49 | 10 | 12 | 25 | 8 | 24 | 19 | 14 | 24 | 25 | 44 | 19 | 14 | 11 | 33 | 31 | 10 | 20 | 14 | 21 | 0 |
| 34 | A049 | Kaikoukan | 0 | 0 | 10 | 4 | 7 | 30 | 27 | 27 | 39 | 42 | 31 | 11 | 41 | 17 | 29 | 7 | 39 | 35 | 6 | 53 | 4 | 29 | 40 | 10 | 9 | 10 | 9 | 38 | 19 | 0 | 21 | 16 | 25 | 4 |
| 35 | A050 | Kawabata | 0 | 13 | 26 | 9 | 40 | 12 | 31 | 9 | 28 | 22 | 23 | 16 | 19 | 41 | 8 | 10 | 19 | 10 | 17 | 29 | 4 | 13 | 23 | 38 | 21 | 12 | 8 | 36 | 29 | 7 | 20 | 15 | 16 | 6 |
| 36 | A051 | Kawachi bankan | 0 | 8 | 8 | 12 | 13 | 30 | 23 | 23 | 35 | 38 | 29 | 18 | 39 | 17 | 26 | 18 | 37 | 25 | 8 | 42 | 11 | 21 | 36 | 10 | 8 | 8 | 14 | 36 | 21 | 18 | 23 | 14 | 29 | 20 |
| 37 | A052 | Keraji | 0 | 13 | 37 | 9 | 52 | 17 | 41 | 10 | 22 | 26 | 20 | 15 | 18 | 58 | 7 | 15 | 20 | 10 | 23 | 24 | 10 | 10 | 20 | 53 | 27 | 9 | 10 | 41 | 33 | 14 | 24 | 18 | 19 | 0 |
| 38 | A053 | Kikudaidai | 0 | 9 | 20 | 16 | 25 | 31 | 27 | 26 | 38 | 34 | 36 | 14 | 33 | 30 | 20 | 17 | 33 | 18 | 18 | 1 | 13 | 27 | 38 | 24 | 13 | 17 | 14 | 33 | 21 | 10 | 21 | 15 | 20 | 19 |
| 39 | A054 | King mandarin | 0 | 11 | 41 | 11 | 51 | 13 | 34 | 10 | 19 | 18 | 15 | 21 | 18 | 57 | 15 | 14 | 11 | 10 | 17 | 28 | 15 | 12 | 16 | 51 | 25 | 11 | 19 | 35 | 32 | 10 | 31 | 22 | 19 | 15 |
| 40 | A055 | Kinkoji | 0 | 10 | 16 | 11 | 21 | 26 | 27 | 13 | 32 | 33 | 29 | 12 | 31 | 21 | 21 | 12 | 29 | 33 | 13 | 46 | 6 | 18 | 34 | 20 | 16 | 7 | 8 | 44 | 23 | 7 | 20 | 18 | 23 | 8 |
| 41 | A059 | Kishu mandarin | 0 | 0 | 52 | 14 | 70 | 6 | 34 | 14 | 7 | 10 | 9 | 26 | 7 | 76 | 0 | 17 | 8 | 10 | 28 | 28 | 23 | 3 | 6 | 69 | 32 | 13 | 25 | 39 | 44 | 8 | 27 | 21 | 13 | 21 |
| 42 | A073 | Kizu | 0 | 15 | 37 | 15 | 54 | 13 | 35 | 10 | 22 | 23 | 22 | 24 | 18 | 51 | 17 | 10 | 21 | 19 | 23 | 16 | 16 | 12 | 19 | 49 | 31 | 10 | 15 | 19 | 8 | 9 | 8 | 16 | 5 | 10 |
| 43 | A075 | Kobeni mikan | 0 | 14 | 57 | 26 | 75 | 3 | 36 | 16 | 16 | 12 | 14 | 32 | 9 | 80 | 10 | 20 | 13 | 12 | 30 | 38 | 29 | 15 | 14 | 75 | 33 | 21 | 26 | 43 | 45 | 18 | 29 | 33 | 16 | 25 |
| 44 | A076 | Koji | 0 | 16 | 42 | 22 | 54 | 10 | 29 | 8 | 14 | 14 | 13 | 12 | 14 | 63 | 0 | 19 | 20 | 3 | 28 | 14 | 23 | 19 | 16 | 56 | 30 | 27 | 14 | 34 | 35 | 16 | 19 | 22 | 17 | 13 |
| 45 | A079 | Kourai Tachibana | 1 | 25 | 60 | 37 | 63 | 25 | 33 | 23 | 29 | 24 | 29 | 25 | 28 | 63 | 17 | 30 | 21 | 13 | 33 | 5 | 36 | 19 | 33 | 67 | 39 | 37 | 16 | 29 | 25 | 29 | 18 | 18 | 24 | 28 |
| 46 | A080 | Kotokan | 0 | 5 | 19 | 8 | 20 | 9 | 15 | 11 | 17 | 14 | 16 | 11 | 9 | 30 | 10 | 13 | 7 | 13 | 9 | 27 | 9 | 11 | 7 | 19 | 15 | 7 | 14 | 25 | 19 | 6 | 12 | 12 | 11 | 13 |
| 47 | A081 | Kunenbo-A | 0 | 8 | 13 | 0 | 36 | 4 | 26 | 7 | 8 | 10 | 7 | 14 | 9 | 39 | 3 | 3 | 7 | 8 | 5 | 33 | 0 | 0 | 8 | 33 | 13 | 0 | 10 | 34 | 23 | 3 | 2 | 12 | 0 | 0 |
| 48 | A082 | Kunenbo-B | 0 | 11 | 40 | 20 | 57 | 14 | 16 | 12 | 23 | 16 | 21 | 8 | 22 | 60 | 7 | 13 | 16 | 5 | 22 | 10 | 26 | 15 | 21 | 60 | 21 | 15 | 10 | 30 | 29 | 19 | 27 | 15 | 16 | 7 |
| 49 | A085 | Lemon | 0 | 22 | 34 | 28 | 46 | 29 | 4 | 29 | 34 | 32 | 35 | 24 | 40 | 50 | 29 | 29 | 38 | 21 | 23 | 37 | 30 | 31 | 38 | 48 | 18 | 25 | 22 | 1 | 23 | 28 | 27 | 29 | 30 | 34 |
| 50 | A086 | Lemonade | 0 | 15 | 29 | 22 | 42 | 26 | 19 | 26 | 30 | 25 | 18 | 19 | 26 | 48 | 20 | 23 | 24 | 22 | 22 | 37 | 28 | 22 | 21 | 39 | 16 | 24 | 20 | 19 | 28 | 16 | 25 | 25 | 22 | 21 |
| 51 | A087 | Limonia | 0 | 24 | 52 | 37 | 62 | 21 | 19 | 24 | 25 | 14 | 27 | 27 | 23 | 67 | 19 | 30 | 26 | 19 | 35 | 31 | 37 | 24 | 24 | 63 | 36 | 30 | 25 | 21 | 38 | 23 | 33 | 31 | 25 | 29 |
| 52 | A088 | Mato buntan | 0 | 18 | 10 | 13 | 10 | 67 | 32 | 42 | 69 | 79 | 61 | 16 | 74 | 13 | 62 | 27 | 75 | 56 | 16 | 72 | 12 | 54 | 71 | 7 | 15 | 19 | 28 | 54 | 19 | 29 | 42 | 29 | 49 | 45 |
| 53 | A089 | Mexican lime | 1 | 43 | 56 | 47 | 61 | 54 | 32 | 44 | 58 | 57 | 60 | 49 | 60 | 65 | 49 | 48 | 60 | 43 | 49 | 38 | 52 | 40 | 61 | 62 | 50 | 43 | 44 | 25 | 35 | 49 | 42 | 40 | 41 | 50 |
| 54 | A090 | Meyer lemon | 0 | 17 | 40 | 25 | 51 | 21 | 25 | 24 | 24 | 27 | 27 | 28 | 24 | 60 | 20 | 19 | 25 | 26 | 26 | 33 | 28 | 24 | 22 | 51 | 31 | 19 | 21 | 22 | 30 | 15 | 30 | 26 | 21 | 22 |
| 55 | A091 | Mochiyu | 0 | 11 | 39 | 11 | 49 | 11 | 29 | 16 | 19 | 24 | 12 | 20 | 20 | 51 | 17 | 14 | 23 | 16 | 20 | 18 | 11 | 13 | 21 | 50 | 19 | 14 | 14 | 25 | 13 | 8 | 16 | 9 | 4 | 17 |
| 56 | A092 | Murcott | 0 | 10 | 42 | 22 | 61 | 10 | 34 | 12 | 13 | 12 | 10 | 28 | 3 | 68 | 12 | 13 | 10 | 10 | 18 | 34 | 16 | 18 | 7 | 58 | 29 | 14 | 26 | 39 | 37 | 9 | 29 | 32 | 17 | 21 |
| 57 | A095 | Naruto | 0 | 5 | 8 | 5 | 9 | 10 | 20 | 14 | 18 | 20 | 13 | 10 | 16 | 17 | 8 | 9 | 15 | 9 | 4 | 30 | 5 | 13 | 18 | 12 | 6 | 5 | 19 | 27 | 17 | 9 | 18 | 9 | 12 | 17 |
| 58 | A098 | Natsudaidai | 0 | 6 | 11 | 9 | 17 | 7 | 18 | 9 | 15 | 12 | 10 | 15 | 7 | 25 | 5 | 9 | 10 | 8 | 7 | 27 | 9 | 7 | 10 | 12 | 6 | 3 | 15 | 27 | 14 | 4 | 11 | 10 | 8 | 11 |
| 59 | A100 | Nidonari mikan | 0 | 7 | 38 | 15 | 56 | 12 | 20 | 22 | 13 | 15 | 12 | 25 | 12 | 59 | 10 | 18 | 14 | 19 | 19 | 36 | 22 | 13 | 11 | 57 | 18 | 17 | 21 | 38 | 36 | 9 | 17 | 26 | 20 | 20 |
| 60 | A101 | Oogonkan | 0 | 7 | 26 | 16 | 37 | 13 | 20 | 13 | 24 | 19 | 18 | 17 | 20 | 37 | 10 | 13 | 20 | 10 | 12 | 26 | 13 | 21 | 23 | 34 | 16 | 12 | 15 | 28 | 30 | 11 | 26 | 19 | 15 | 18 |
| 61 | A103 | Ootoukan | 0 | 11 | 25 | 10 | 21 | 0 | 23 | 13 | 15 | 25 | 16 | 18 | 18 | 33 | 15 | 2 | 18 | 21 | 11 | 40 | 12 | 15 | 23 | 23 | 13 | 10 | 16 | 31 | 19 | 8 | 20 | 13 | 9 | 16 |
| 62 | A104 | Oukan | 0 | 8 | 24 | 5 | 26 | 10 | 24 | 16 | 17 | 14 | 15 | 17 | 12 | 39 | 8 | 8 | 14 | 13 | 14 | 33 | 14 | 10 | 15 | 28 | 14 | 9 | 19 | 31 | 29 | 7 | 19 | 12 | 11 | 17 |
| 63 | A107 | Ponkan | 0 | 13 | 49 | 17 | 67 | 3 | 37 | 12 | 4 | 10 | 3 | 27 | 0 | 72 | 11 | 11 | 4 | 11 | 18 | 31 | 18 | 11 | 0 | 66 | 33 | 14 | 27 | 39 | 44 | 1 | 24 | 27 | 11 | 19 |
| 64 | A109 | Ponkitsu | 0 | 16 | 55 | 32 | 68 | 15 | 44 | 21 | 14 | 16 | 14 | 27 | 11 | 72 | 15 | 23 | 5 | 20 | 21 | 35 | 31 | 16 | 13 | 70 | 44 | 20 | 25 | 43 | 51 | 13 | 28 | 34 | 27 | 28 |
| 65 | A110 | Pummelo whitetype | 1 | 17 | 13 | 19 | 10 | 72 | 36 | 45 | 72 | 78 | 64 | 27 | 76 | 20 | 66 | 31 | 74 | 62 | 16 | 74 | 13 | 58 | 72 | 7 | 15 | 20 | 29 | 55 | 32 | 27 | 48 | 29 | 58 | 46 |
| 66 | A111 | Rokugatsumikan | 0 | 13 | 36 | 18 | 48 | 19 | 12 | 14 | 25 | 22 | 21 | 8 | 30 | 54 | 7 | 18 | 26 | 7 | 17 | 14 | 22 | 19 | 28 | 51 | 21 | 14 | 11 | 27 | 24 | 21 | 24 | 14 | 16 | 9 |
| 67 | A112 | Sanbokan | 0 | 1 | 13 | 6 | 18 | 9 | 20 | 12 | 18 | 19 | 18 | 16 | 21 | 31 | 8 | 6 | 16 | 17 | 5 | 30 | 11 | 9 | 16 | 16 | 8 | 6 | 12 | 24 | 19 | 5 | 17 | 10 | 12 | 10 |
| 68 | A125 | Satsuma mandarin | 0 | 2 | 27 | 10 | 46 | 9 | 30 | 13 | 13 | 13 | 8 | 18 | 15 | 48 | 6 | 13 | 12 | 16 | 15 | 34 | 10 | 9 | 14 | 42 | 19 | 8 | 14 | 39 | 34 | 11 | 17 | 21 | 14 | 16 |
| 69 | A134 | Satsuma Kikoku | 0 | 7 | 17 | 13 | 18 | 17 | 17 | 12 | 26 | 18 | 22 | 17 | 23 | 24 | 17 | 18 | 15 | 15 | 8 | 30 | 12 | 22 | 24 | 27 | 15 | 11 | 15 | 26 | 18 | 11 | 21 | 10 | 21 | 18 |
| 70 | A135 | Shiikuwasha | 0 | 13 | 51 | 26 | 69 | 12 | 30 | 16 | 15 | 9 | 14 | 7 | 14 | 75 | 6 | 18 | 13 | 7 | 30 | 16 | 31 | 15 | 16 | 72 | 34 | 24 | 13 | 32 | 39 | 14 | 29 | 20 | 19 | 6 |
| 71 | A136 | Shiikuwasha_Ogimikugani | 0 | 13 | 53 | 24 | 67 | 13 | 33 | 13 | 21 | 10 | 18 | 9 | 16 | 76 | 5 | 20 | 15 | 4 | 31 | 12 | 30 | 14 | 14 | 72 | 35 | 23 | 10 | 33 | 39 | 15 | 32 | 19 | 16 | 5 |
| 72 | A137 | Shunkokan | 0 | 8 | 11 | 11 | 26 | 14 | 25 | 14 | 20 | 21 | 14 | 14 | 15 | 31 | 7 | 9 | 18 | 19 | 9 | 26 | 8 | 15 | 12 | 26 | 11 | 11 | 19 | 29 | 24 | 6 | 15 | 17 | 16 | 16 |
| 73 | A138 | Sokitsu | 0 | 6 | 57 | 18 | 74 | 5 | 40 | 14 | 10 | 10 | 12 | 35 | 11 | 80 | 3 | 18 | 10 | 12 | 28 | 35 | 23 | 6 | 14 | 73 | 33 | 18 | 29 | 45 | 47 | 16 | 30 | 25 | 17 | 25 |
| 74 | A141 | Sour orange | 0 | 10 | 15 | 10 | 24 | 9 | 0 | 16 | 18 | 18 | 17 | 14 | 21 | 29 | 15 | 12 | 19 | 15 | 9 | 32 | 20 | 20 | 21 | 25 | 0 | 12 | 14 | 16 | 12 | 13 | 20 | 14 | 10 | 17 |
| 75 | A144 | Sudachi | 0 | 19 | 48 | 22 | 54 | 12 | 26 | 15 | 22 | 17 | 17 | 26 | 18 | 55 | 10 | 16 | 18 | 8 | 25 | 12 | 26 | 15 | 24 | 57 | 27 | 27 | 21 | 20 | 14 | 21 | 12 | 15 | 12 | 25 |
| 76 | A145 | Suisho buntan | 0 | 18 | 9 | 14 | 9 | 61 | 34 | 39 | 60 | 69 | 49 | 20 | 64 | 14 | 50 | 29 | 63 | 51 | 11 | 64 | 9 | 48 | 62 | 1 | 14 | 16 | 20 | 46 | 25 | 28 | 42 | 25 | 43 | 35 |
| 77 | A146 | Sunki | 0 | 14 | 58 | 28 | 75 | 11 | 36 | 19 | 11 | 6 | 7 | 27 | 6 | 81 | 11 | 20 | 7 | 12 | 30 | 31 | 28 | 12 | 5 | 75 | 36 | 26 | 25 | 43 | 47 | 7 | 27 | 28 | 18 | 24 |

| | | | | | | | | | | | | | | | | | | | | | | | | | | | | | | | | | | | | |
|---|---|---|---|---|---|---|---|---|---|---|---|---|---|---|---|---|---|---|---|---|---|---|---|---|---|---|---|---|---|---|---|---|---|---|---|---|
| 78 | A147 | Suruga Yuko | 0 | 12 | 52 | 29 | 67 | 14 | 38 | 17 | 11 | 15 | 17 | 25 | 21 | 71 | 3 | 26 | 20 | 11 | 32 | 22 | 30 | 16 | 13 | 67 | 31 | 27 | 23 | 41 | 39 | 20 | 26 | 23 | 25 | 17 |
| 79 | A162 | Sweet orange | 0 | 6 | 19 | 10 | 31 | 7 | 23 | 9 | 0 | 13 | 0 | 12 | 6 | 35 | 14 | 5 | 10 | 13 | 0 | 34 | 9 | 13 | 6 | 27 | 11 | 6 | 15 | 34 | 24 | 5 | 11 | 17 | 7 | 14 |
| 80 | A172 | Tachibana-A | 1 | 22 | 61 | 33 | 71 | 23 | 44 | 15 | 29 | 19 | 27 | 20 | 24 | 77 | 8 | 28 | 17 | 5 | 37 | 0 | 36 | 28 | 29 | 77 | 45 | 33 | 17 | 41 | 49 | 25 | 42 | 28 | 30 | 12 |
| 81 | A174 | Tachibana-C | 0 | 20 | 57 | 30 | 77 | 12 | 36 | 10 | 28 | 17 | 23 | 13 | 18 | 79 | 4 | 24 | 22 | 8 | 39 | 14 | 32 | 24 | 25 | 79 | 37 | 30 | 12 | 39 | 47 | 22 | 35 | 24 | 21 | 6 |
| 82 | A175 | Tachibana-B | 0 | 19 | 58 | 34 | 70 | 22 | 40 | 21 | 29 | 18 | 25 | 22 | 21 | 76 | 10 | 28 | 19 | 1 | 43 | 9 | 36 | 24 | 27 | 76 | 42 | 34 | 0 | 39 | 44 | 24 | 35 | 24 | 31 | 12 |
| 83 | A183 | Tankan | 0 | 13 | 49 | 25 | 56 | 13 | 38 | 11 | 8 | 17 | 7 | 25 | 6 | 62 | 15 | 18 | 0 | 15 | 15 | 36 | 24 | 17 | 6 | 57 | 36 | 13 | 26 | 41 | 42 | 12 | 26 | 32 | 21 | 26 |
| 84 | A186 | Temple | 0 | 11 | 35 | 21 | 47 | 5 | 27 | 17 | 1 | 14 | 3 | 22 | 9 | 49 | 13 | 11 | 10 | 15 | 5 | 32 | 19 | 11 | 5 | 43 | 19 | 13 | 25 | 35 | 35 | 8 | 17 | 24 | 18 | 28 |
| 85 | A187 | Tengu | 0 | 10 | 21 | 9 | 29 | 13 | 31 | 8 | 11 | 25 | 13 | 16 | 15 | 33 | 20 | 6 | 20 | 17 | 9 | 41 | 6 | 11 | 9 | 22 | 11 | 3 | 7 | 40 | 26 | 13 | 24 | 15 | 17 | 15 |
| 86 | A188 | Tizon | 0 | 9 | 36 | 19 | 52 | 8 | 26 | 21 | 8 | 0 | 6 | 20 | 10 | 57 | 11 | 11 | 8 | 15 | 1 | 29 | 15 | 16 | 9 | 53 | 28 | 13 | 25 | 34 | 38 | 6 | 16 | 24 | 14 | 22 |
| 87 | A191 | Tosa buntan | 0 | 14 | 12 | 8 | 10 | 53 | 31 | 33 | 51 | 61 | 41 | 11 | 56 | 15 | 46 | 20 | 53 | 45 | 12 | 53 | 7 | 41 | 53 | 6 | 13 | 12 | 17 | 44 | 24 | 21 | 37 | 20 | 38 | 20 |
| 88 | A192 | Twukkuni | 1 | 21 | 47 | 26 | 68 | 21 | 38 | 14 | 28 | 22 | 24 | 13 | 28 | 67 | 10 | 22 | 27 | 13 | 30 | 21 | 28 | 16 | 22 | 66 | 34 | 15 | 14 | 41 | 37 | 24 | 36 | 26 | 21 | 8 |
| 89 | A196 | Uchimurasaki | 0 | 18 | 8 | 14 | 6 | 76 | 40 | 47 | 73 | 84 | 67 | 25 | 79 | 13 | 71 | 30 | 79 | 64 | 10 | 79 | 11 | 59 | 79 | 10 | 14 | 15 | 27 | 56 | 28 | 30 | 45 | 33 | 58 | 46 |
| 90 | A197 | Ujukitsu | 0 | 7 | 13 | 7 | 26 | 8 | 23 | 11 | 14 | 17 | 7 | 15 | 18 | 34 | 5 | 12 | 11 | 8 | 5 | 32 | 7 | 6 | 13 | 28 | 12 | 3 | 15 | 29 | 18 | 7 | 11 | 7 | 8 | 8 |
| 91 | A198 | Unzoki | 0 | 8 | 22 | 3 | 43 | 16 | 34 | 13 | 23 | 21 | 19 | 9 | 26 | 48 | 6 | 9 | 24 | 7 | 18 | 14 | 10 | 16 | 22 | 41 | 18 | 9 | 13 | 29 | 30 | 14 | 17 | 13 | 20 | 3 |
| 92 | A199 | USSR Tangelo | 0 | 3 | 10 | 7 | 15 | 22 | 23 | 23 | 30 | 30 | 19 | 19 | 30 | 18 | 16 | 11 | 25 | 20 | 10 | 40 | 11 | 16 | 23 | 11 | 10 | 4 | 14 | 35 | 24 | 15 | 21 | 12 | 19 | 20 |
| 93 | A200 | Med mandarin | 0 | 11 | 61 | 29 | 77 | 5 | 38 | 20 | 8 | 9 | 7 | 31 | 8 | 80 | 9 | 19 | 8 | 14 | 27 | 30 | 30 | 10 | 10 | 76 | 36 | 21 | 26 | 40 | 52 | 12 | 29 | 34 | 22 | 26 |
| 94 | A201 | Willowleaf mandarin | 0 | 8 | 57 | 24 | 70 | 6 | 33 | 20 | 0 | 8 | 0 | 27 | 8 | 74 | 6 | 16 | 3 | 13 | 26 | 30 | 26 | 7 | 5 | 69 | 33 | 17 | 28 | 38 | 47 | 11 | 27 | 31 | 20 | 27 |
| 95 | A202 | Yamabuki | 0 | 1 | 10 | 5 | 13 | 15 | 16 | 11 | 21 | 16 | 16 | 12 | 21 | 25 | 5 | 10 | 17 | 14 | 7 | 28 | 6 | 10 | 18 | 15 | 7 | 9 | 10 | 28 | 16 | 8 | 14 | 13 | 18 | 10 |
| 96 | A203 | Yamamikan | 0 | 7 | 11 | 6 | 12 | 19 | 22 | 13 | 10 | 29 | 9 | 9 | 15 | 23 | 23 | 7 | 21 | 16 | 4 | 43 | 7 | 20 | 12 | 15 | 10 | 6 | 14 | 31 | 21 | 10 | 19 | 11 | 18 | 15 |
| 97 | A204 | Yatsushiro | 0 | 8 | 33 | 7 | 55 | 9 | 41 | 12 | 21 | 21 | 16 | 23 | 13 | 59 | 5 | 14 | 18 | 23 | 25 | 34 | 4 | 14 | 11 | 52 | 19 | 19 | 18 | 43 | 38 | 4 | 24 | 21 | 18 | 9 |
| 98 | A205 | Yuge hyoukan | 0 | 9 | 12 | 4 | 8 | 18 | 19 | 14 | 27 | 24 | 20 | 16 | 25 | 15 | 14 | 19 | 24 | 18 | 8 | 34 | 7 | 12 | 27 | 10 | 9 | 5 | 15 | 30 | 17 | 8 | 19 | 9 | 20 | 18 |
| 99 | A206 | Youpi ju | 0 | 13 | 63 | 30 | 77 | 9 | 37 | 24 | 15 | 8 | 14 | 34 | 9 | 80 | 10 | 25 | 13 | 17 | 34 | 34 | 35 | 17 | 14 | 78 | 40 | 29 | 29 | 43 | 57 | 12 | 36 | 38 | 27 | 29 |
| 100 | A207 | Yuukunibu | 0 | 0 | 16 | 7 | 19 | 15 | 20 | 16 | 23 | 22 | 15 | 16 | 16 | 31 | 13 | 10 | 21 | 14 | 11 | 36 | 11 | 13 | 19 | 20 | 12 | 9 | 18 | 35 | 21 | 5 | 22 | 16 | 16 | 21 |
| 101 | A208 | Yuzu | 0 | 22 | 50 | 36 | 60 | 24 | 31 | 21 | 35 | 32 | 30 | 24 | 32 | 59 | 22 | 26 | 30 | 17 | 34 | 2 | 37 | 2 | 33 | 58 | 34 | 31 | 19 | 21 | 1 | 26 | 3 | 0 | 1 | 24 |

ND: no data

Mismatch <5
Mismatch <10

| | | | | | | | | | | | | | | | | | | | | | | | | | | | | | | | | | | | | | | | | | | |
|---|---|---|---|---|---|---|---|---|---|---|---|---|---|---|---|---|---|---|---|---|---|---|---|---|---|---|---|---|---|---|---|---|---|---|---|---|---|---|---|---|---|---|
| 5 | 1 | 2 | 2 | 0 | 1 | 1 | 12 | 1 | 1 | 2 | 0 | 0 | 16 | 1 | 1 | 0 | 0 | 0 | 0 | 0 | 1 | 1 | 2 | 0 | 2 | 1 | 1 | 1 | 2 | 1 | 0 | 1 | 2 | 3 | 0 | 0 | 0 | 1 | 2 | 6 | 0 | 1 |
| 9 | 4 | 3 | 3 | 1 | 1 | 2 | 18 | 2 | 3 | 4 | 1 | 2 | 30 | 3 | 3 | 1 | 1 | 0 | 0 | 2 | 3 | 2 | 8 | 8 | 4 | 1 | 5 | 4 | 11 | 1 | 0 | 2 | 9 | 4 | 0 | 3 | 5 | 4 | 5 | 6 | 1 | 2 |
| Kaikoukan | Kawabata | Kawachi bankan | Keraji | Kikudaidai | King mandarin | Kinkoji | Kishu mandarin | Kizu | Kobeni mikan | Koji | Kourai Tachibana | Kotokan | Kunenbo-A | Kunenbo-B | Lemon | Lemonade | Limonia | Mato buntan | Mexican lime | Meyer lemon | Mochiyu | Murcott | Naruto | Natsudaidai | Nidonari mikan | Oogonkan | Ootoukan | Oukan | Ponkan | Ponkitsu | Pummelo whitetype | Rokugatsumikan | Sanbokan | Satsuma mandarin | Satsuma Kikoku | Shiikuwasha | Shiikuwasha_Ogimikug | Shunkokan | Sokitsu | Sour orange | Sudachi | Suisho buntan |
| A049 | A050 | A051 | A052 | A053 | A054 | A055 | A059 | A073 | A075 | A076 | A079 | A080 | A081 | A082 | A085 | A086 | A087 | A088 | A089 | A090 | A091 | A092 | A095 | A098 | A100 | A101 | A103 | A104 | A107 | A109 | A110 | A111 | A112 | A125 | A134 | A135 | A136 | A137 | A138 | A141 | A144 | A145 |
| 0 | 0 | 0 | 0 | 0 | 0 | 0 | 0 | 0 | 0 | 1 | 0 | 0 | 0 | 0 | 0 | 0 | 0 | 0 | 1 | 0 | 0 | 0 | 0 | 0 | 0 | 0 | 0 | 0 | 0 | 0 | 1 | 0 | 0 | 0 | 0 | 0 | 0 | 0 | 0 | 0 | 0 | 0 |
| 0 | 13 | 8 | 13 | 9 | 11 | 10 | 0 | 15 | 14 | 16 | 25 | 5 | 8 | 11 | 22 | 15 | 24 | 18 | 43 | 17 | 11 | 10 | 5 | 6 | 7 | 7 | 11 | 8 | 13 | 16 | 17 | 13 | 1 | 2 | 7 | 13 | 13 | 8 | 6 | 10 | 19 | 18 |
| 10 | 26 | 8 | 37 | 20 | 41 | 16 | 52 | 37 | 57 | 42 | 60 | 19 | 13 | 40 | 34 | 29 | 52 | 10 | 56 | 40 | 39 | 42 | 8 | 11 | 38 | 26 | 25 | 24 | 49 | 55 | 13 | 36 | 13 | 27 | 17 | 51 | 53 | 11 | 57 | 15 | 48 | 9 |
| 4 | 9 | 12 | 9 | 16 | 11 | 11 | 14 | 15 | 26 | 22 | 37 | 8 | 0 | 20 | 28 | 22 | 37 | 13 | 47 | 25 | 11 | 22 | 5 | 9 | 15 | 16 | 10 | 5 | 17 | 32 | 19 | 18 | 6 | 10 | 13 | 26 | 24 | 11 | 18 | 10 | 22 | 14 |
| 7 | 40 | 13 | 52 | 25 | 51 | 21 | 70 | 54 | 75 | 54 | 63 | 20 | 36 | 57 | 46 | 42 | 62 | 10 | 61 | 51 | 49 | 61 | 9 | 17 | 56 | 37 | 21 | 26 | 67 | 68 | 10 | 48 | 18 | 46 | 18 | 69 | 67 | 26 | 74 | 24 | 54 | 9 |
| 30 | 12 | 30 | 17 | 31 | 13 | 26 | 6 | 13 | 3 | 10 | 25 | 9 | 4 | 14 | 29 | 26 | 21 | 67 | 54 | 21 | 11 | 10 | 10 | 7 | 12 | 13 | 0 | 10 | 3 | 15 | 72 | 19 | 9 | 9 | 17 | 12 | 13 | 14 | 5 | 9 | 12 | 61 |
| 27 | 31 | 23 | 41 | 27 | 34 | 27 | 34 | 35 | 36 | 29 | 33 | 15 | 26 | 16 | 4 | 19 | 19 | 32 | 32 | 25 | 29 | 34 | 20 | 18 | 20 | 20 | 23 | 24 | 37 | 44 | 36 | 12 | 20 | 30 | 17 | 30 | 33 | 25 | 40 | 0 | 26 | 34 |
| 27 | 9 | 23 | 10 | 26 | 10 | 13 | 14 | 10 | 16 | 8 | 23 | 11 | 7 | 12 | 29 | 26 | 24 | 42 | 44 | 24 | 16 | 12 | 14 | 9 | 22 | 13 | 13 | 16 | 12 | 21 | 45 | 14 | 12 | 13 | 12 | 16 | 13 | 14 | 14 | 16 | 15 | 39 |
| 39 | 28 | 35 | 22 | 38 | 19 | 32 | 7 | 22 | 16 | 14 | 29 | 17 | 8 | 23 | 34 | 30 | 25 | 69 | 58 | 24 | 19 | 13 | 18 | 15 | 13 | 24 | 15 | 17 | 4 | 14 | 72 | 25 | 18 | 13 | 26 | 15 | 21 | 20 | 10 | 18 | 22 | 60 |
| 42 | 22 | 38 | 26 | 34 | 18 | 33 | 10 | 23 | 12 | 14 | 24 | 14 | 10 | 16 | 32 | 25 | 14 | 79 | 57 | 27 | 24 | 12 | 20 | 12 | 15 | 19 | 25 | 14 | 10 | 16 | 78 | 22 | 19 | 13 | 18 | 9 | 10 | 21 | 10 | 18 | 17 | 69 |
| 31 | 23 | 29 | 20 | 36 | 15 | 29 | 9 | 22 | 14 | 13 | 29 | 16 | 7 | 21 | 35 | 18 | 27 | 61 | 60 | 27 | 12 | 10 | 13 | 10 | 12 | 18 | 16 | 15 | 3 | 14 | 64 | 21 | 18 | 8 | 22 | 14 | 18 | 14 | 12 | 17 | 17 | 49 |
| 11 | 16 | 18 | 15 | 14 | 21 | 12 | 26 | 24 | 32 | 12 | 25 | 11 | 14 | 8 | 24 | 19 | 27 | 16 | 49 | 28 | 20 | 28 | 10 | 15 | 25 | 17 | 18 | 17 | 27 | 27 | 27 | 8 | 16 | 18 | 17 | 7 | 9 | 14 | 35 | 14 | 26 | 20 |
| 41 | 19 | 39 | 18 | 33 | 18 | 31 | 7 | 18 | 9 | 14 | 28 | 9 | 9 | 22 | 40 | 26 | 23 | 74 | 60 | 24 | 20 | 3 | 16 | 7 | 12 | 20 | 18 | 12 | 0 | 11 | 76 | 30 | 21 | 15 | 23 | 14 | 16 | 15 | 11 | 21 | 18 | 64 |
| 17 | 41 | 17 | 58 | 30 | 57 | 21 | 76 | 51 | 80 | 63 | 63 | 30 | 39 | 60 | 50 | 48 | 67 | 13 | 65 | 60 | 51 | 68 | 17 | 25 | 59 | 37 | 33 | 39 | 72 | 72 | 20 | 54 | 31 | 48 | 24 | 75 | 76 | 31 | 80 | 29 | 55 | 14 |
| 29 | 8 | 26 | 7 | 20 | 15 | 21 | 0 | 17 | 10 | 0 | 17 | 10 | 3 | 7 | 29 | 20 | 19 | 62 | 49 | 20 | 17 | 12 | 8 | 5 | 10 | 10 | 15 | 8 | 11 | 15 | 66 | 7 | 8 | 6 | 17 | 6 | 5 | 7 | 3 | 15 | 10 | 50 |
| 7 | 10 | 18 | 15 | 17 | 14 | 12 | 17 | 10 | 20 | 19 | 30 | 13 | 3 | 13 | 29 | 23 | 30 | 27 | 48 | 19 | 14 | 13 | 9 | 9 | 18 | 13 | 2 | 8 | 11 | 23 | 31 | 18 | 6 | 13 | 18 | 18 | 20 | 9 | 18 | 12 | 16 | 29 |
| 39 | 19 | 37 | 20 | 33 | 11 | 29 | 8 | 21 | 13 | 20 | 21 | 7 | 7 | 16 | 38 | 24 | 26 | 75 | 60 | 25 | 23 | 10 | 15 | 10 | 14 | 20 | 18 | 14 | 4 | 5 | 74 | 26 | 16 | 12 | 15 | 13 | 15 | 18 | 10 | 19 | 18 | 63 |
| 35 | 10 | 25 | 10 | 18 | 10 | 33 | 10 | 19 | 12 | 3 | 13 | 13 | 8 | 5 | 21 | 22 | 19 | 56 | 43 | 26 | 16 | 10 | 9 | 8 | 19 | 10 | 21 | 13 | 11 | 20 | 62 | 7 | 17 | 16 | 15 | 7 | 4 | 19 | 12 | 15 | 8 | 51 |
| 6 | 17 | 8 | 23 | 18 | 17 | 13 | 28 | 23 | 30 | 28 | 33 | 9 | 5 | 22 | 23 | 22 | 35 | 16 | 49 | 26 | 20 | 18 | 4 | 7 | 19 | 12 | 11 | 14 | 18 | 21 | 16 | 17 | 5 | 15 | 8 | 30 | 31 | 9 | 28 | 9 | 25 | 11 |
| 53 | 29 | 42 | 24 | 1 | 28 | 46 | 28 | 16 | 38 | 14 | 5 | 27 | 33 | 10 | 37 | 37 | 31 | 72 | 38 | 33 | 18 | 34 | 30 | 27 | 36 | 26 | 40 | 33 | 31 | 35 | 74 | 14 | 30 | 34 | 30 | 16 | 12 | 26 | 35 | 32 | 12 | 64 |
| 4 | 4 | 11 | 10 | 13 | 15 | 6 | 23 | 16 | 29 | 23 | 36 | 9 | 0 | 26 | 30 | 28 | 37 | 12 | 52 | 28 | 11 | 16 | 5 | 9 | 22 | 13 | 12 | 14 | 18 | 31 | 13 | 22 | 11 | 10 | 12 | 31 | 30 | 8 | 23 | 20 | 26 | 9 |
| 29 | 13 | 21 | 10 | 27 | 12 | 18 | 3 | 12 | 15 | 19 | 19 | 11 | 0 | 15 | 31 | 22 | 24 | 54 | 40 | 24 | 13 | 18 | 13 | 7 | 13 | 21 | 15 | 10 | 11 | 16 | 58 | 19 | 9 | 9 | 22 | 15 | 14 | 15 | 6 | 20 | 15 | 48 |
| 40 | 23 | 36 | 20 | 38 | 16 | 34 | 6 | 19 | 14 | 16 | 33 | 7 | 8 | 21 | 38 | 21 | 24 | 71 | 61 | 22 | 21 | 7 | 18 | 10 | 11 | 23 | 23 | 15 | 0 | 13 | 72 | 28 | 16 | 14 | 24 | 16 | 14 | 12 | 14 | 21 | 24 | 62 |
| 10 | 38 | 10 | 53 | 24 | 51 | 20 | 69 | 49 | 75 | 56 | 67 | 19 | 33 | 60 | 48 | 39 | 63 | 7 | 62 | 51 | 50 | 58 | 12 | 12 | 57 | 34 | 23 | 28 | 66 | 70 | 7 | 51 | 16 | 42 | 27 | 72 | 72 | 26 | 73 | 25 | 57 | 1 |
| 9 | 21 | 8 | 27 | 13 | 25 | 16 | 32 | 31 | 33 | 30 | 39 | 15 | 13 | 21 | 18 | 16 | 36 | 15 | 50 | 31 | 19 | 29 | 6 | 6 | 18 | 16 | 13 | 14 | 33 | 44 | 15 | 21 | 8 | 19 | 15 | 34 | 35 | 11 | 33 | 0 | 27 | 14 |
| 10 | 12 | 8 | 9 | 17 | 11 | 7 | 13 | 10 | 21 | 27 | 37 | 7 | 0 | 15 | 25 | 24 | 30 | 19 | 43 | 19 | 14 | 14 | 5 | 3 | 17 | 12 | 10 | 9 | 14 | 20 | 20 | 14 | 6 | 8 | 11 | 24 | 23 | 11 | 18 | 12 | 27 | 16 |
| 9 | 8 | 14 | 10 | 14 | 19 | 8 | 25 | 15 | 26 | 14 | 16 | 14 | 10 | 10 | 22 | 20 | 25 | 28 | 44 | 21 | 14 | 26 | 19 | 15 | 21 | 15 | 16 | 19 | 27 | 25 | 29 | 11 | 12 | 14 | 15 | 13 | 10 | 19 | 29 | 14 | 21 | 20 |
| 38 | 36 | 36 | 41 | 33 | 35 | 44 | 39 | 19 | 43 | 34 | 29 | 25 | 34 | 30 | 1 | 19 | 21 | 54 | 25 | 22 | 25 | 39 | 27 | 27 | 38 | 28 | 31 | 31 | 39 | 43 | 55 | 27 | 24 | 39 | 26 | 32 | 33 | 29 | 45 | 16 | 20 | 46 |
| 19 | 29 | 21 | 33 | 21 | 32 | 23 | 44 | 8 | 45 | 35 | 25 | 19 | 23 | 29 | 23 | 28 | 38 | 19 | 35 | 30 | 13 | 37 | 17 | 14 | 36 | 30 | 19 | 29 | 44 | 51 | 32 | 24 | 19 | 34 | 18 | 39 | 39 | 24 | 47 | 12 | 14 | 25 |
| 0 | 7 | 18 | 14 | 10 | 10 | 7 | 8 | 9 | 18 | 16 | 29 | 6 | 3 | 19 | 28 | 16 | 23 | 29 | 49 | 15 | 8 | 9 | 9 | 4 | 9 | 11 | 8 | 7 | 1 | 13 | 27 | 21 | 5 | 11 | 11 | 14 | 15 | 6 | 16 | 13 | 21 | 28 |
| 21 | 20 | 23 | 24 | 21 | 31 | 20 | 27 | 8 | 29 | 19 | 18 | 12 | 2 | 27 | 27 | 25 | 33 | 42 | 42 | 30 | 16 | 29 | 18 | 11 | 17 | 26 | 20 | 19 | 24 | 28 | 48 | 24 | 17 | 17 | 21 | 29 | 32 | 15 | 30 | 20 | 12 | 42 |
| 16 | 15 | 14 | 18 | 15 | 22 | 18 | 21 | 16 | 33 | 22 | 18 | 12 | 12 | 15 | 29 | 25 | 31 | 29 | 40 | 26 | 9 | 32 | 9 | 10 | 26 | 19 | 13 | 12 | 27 | 34 | 29 | 14 | 10 | 21 | 10 | 20 | 19 | 17 | 25 | 14 | 15 | 25 |

| | | | | | | | | | | | | | | | | | | | | | | | | | | | | | | | | | | | | | | | | | | |
|---|---|---|---|---|---|---|---|---|---|---|---|---|---|---|---|---|---|---|---|---|---|---|---|---|---|---|---|---|---|---|---|---|---|---|---|---|---|---|---|---|---|---|
| 25 | 16 | 29 | 19 | 20 | 19 | 23 | 13 | 5 | 16 | 17 | 24 | 11 | 0 | 16 | 30 | 22 | 25 | 49 | 41 | 21 | 4 | 17 | 12 | 8 | 20 | 15 | 9 | 11 | 11 | 27 | 58 | 16 | 12 | 14 | 21 | 19 | 16 | 16 | 17 | 10 | 12 | 43 |
| 4 | 6 | 20 | 0 | 19 | 15 | 8 | 21 | 10 | 25 | 13 | 28 | 13 | 0 | 7 | 34 | 21 | 29 | 45 | 50 | 22 | 17 | 21 | 17 | 11 | 20 | 18 | 16 | 17 | 19 | 28 | 46 | 9 | 10 | 16 | 18 | 6 | 5 | 16 | 25 | 17 | 25 | 35 |
| 0 | 8 | 14 | 23 | 13 | 26 | 7 | 35 | 18 | 43 | 30 | 49 | 12 | 0 | 34 | 37 | 22 | 48 | 11 | 57 | 27 | 21 | 33 | 8 | 8 | 26 | 20 | 10 | 19 | 30 | 45 | 13 | 36 | 0 | 15 | 14 | 38 | 39 | 13 | 39 | 17 | 36 | 13 |
| 8 | 0 | 24 | 5 | 18 | 15 | 2 | 16 | 7 | 25 | 13 | 26 | 15 | 0 | 19 | 37 | 21 | 32 | 39 | 54 | 22 | 13 | 17 | 16 | 12 | 21 | 9 | 10 | 17 | 15 | 21 | 38 | 22 | 13 | 11 | 13 | 22 | 17 | 16 | 21 | 19 | 18 | 31 |
| 14 | 24 | 0 | 24 | 19 | 25 | 15 | 24 | 28 | 33 | 29 | 36 | 10 | 12 | 24 | 32 | 23 | 38 | 11 | 48 | 28 | 24 | 30 | 3 | 7 | 29 | 18 | 14 | 17 | 34 | 36 | 16 | 19 | 7 | 23 | 12 | 31 | 30 | 7 | 30 | 12 | 35 | 14 |
| 23 | 5 | 24 | 0 | 22 | 11 | 23 | 16 | 19 | 22 | 14 | 31 | 18 | 0 | 13 | 39 | 31 | 34 | 56 | 58 | 23 | 5 | 14 | 15 | 12 | 24 | 12 | 15 | 21 | 17 | 24 | 53 | 9 | 17 | 8 | 22 | 13 | 11 | 16 | 19 | 25 | 23 | 43 |
| 13 | 18 | 19 | 22 | 0 | 30 | 18 | 32 | 16 | 41 | 20 | 21 | 12 | 17 | 15 | 35 | 25 | 34 | 27 | 46 | 28 | 19 | 31 | 12 | 8 | 26 | 26 | 19 | 18 | 31 | 37 | 31 | 15 | 17 | 24 | 16 | 19 | 17 | 12 | 39 | 17 | 22 | 27 |
| 26 | 15 | 25 | 11 | 30 | 0 | 20 | 12 | 21 | 14 | 13 | 23 | 12 | 6 | 14 | 30 | 22 | 22 | 57 | 52 | 17 | 11 | 0 | 13 | 9 | 14 | 12 | 17 | 17 | 13 | 19 | 52 | 19 | 15 | 12 | 13 | 16 | 18 | 16 | 14 | 15 | 14 | 44 |
| 7 | 2 | 15 | 23 | 18 | 20 | 0 | 26 | 9 | 34 | 28 | 43 | 13 | 0 | 33 | 37 | 25 | 43 | 19 | 58 | 30 | 23 | 22 | 9 | 10 | 19 | 16 | 11 | 17 | 26 | 26 | 21 | 31 | 13 | 6 | 16 | 31 | 32 | 13 | 33 | 16 | 34 | 10 |
| 35 | 16 | 24 | 16 | 32 | 12 | 26 | 0 | 16 | 3 | 13 | 25 | 5 | 0 | 12 | 32 | 23 | 19 | 71 | 56 | 23 | 17 | 8 | 0 | 1 | 0 | 17 | 17 | 0 | 7 | 14 | 75 | 20 | 0 | 0 | 17 | 6 | 9 | 3 | 0 | 15 | 16 | 61 |
| 18 | 7 | 28 | 19 | 16 | 21 | 9 | 16 | 0 | 22 | 16 | 22 | 14 | 0 | 24 | 31 | 22 | 28 | 48 | 36 | 24 | 16 | 16 | 16 | 9 | 14 | 18 | 10 | 14 | 11 | 24 | 55 | 27 | 13 | 12 | 21 | 23 | 21 | 14 | 23 | 19 | 10 | 44 |
| 43 | 25 | 33 | 22 | 41 | 14 | 34 | 3 | 22 | 0 | 14 | 26 | 12 | 12 | 15 | 34 | 29 | 24 | 78 | 60 | 24 | 20 | 11 | 16 | 12 | 13 | 15 | 18 | 15 | 10 | 16 | 80 | 23 | 21 | 13 | 20 | 11 | 16 | 21 | 0 | 16 | 12 | 69 |
| 30 | 13 | 29 | 14 | 20 | 13 | 28 | 13 | 16 | 14 | 0 | 12 | 10 | 13 | 6 | 25 | 22 | 21 | 53 | 45 | 29 | 21 | 11 | 18 | 14 | 18 | 10 | 21 | 11 | 8 | 24 | 61 | 8 | 16 | 21 | 19 | 10 | 8 | 17 | 13 | 17 | 7 | 53 |
| 49 | 26 | 36 | 31 | 21 | 23 | 43 | 25 | 22 | 26 | 12 | 1 | 24 | 28 | 16 | 36 | 34 | 29 | 68 | 39 | 38 | 20 | 31 | 27 | 28 | 34 | 22 | 28 | 32 | 25 | 26 | 68 | 18 | 24 | 31 | 22 | 20 | 19 | 32 | 28 | 23 | 14 | 61 |
| 12 | 15 | 10 | 18 | 12 | 12 | 13 | 5 | 14 | 12 | 10 | 24 | 0 | 8 | 10 | 19 | 13 | 22 | 18 | 41 | 11 | 14 | 15 | 6 | 4 | 5 | 10 | 7 | 3 | 9 | 13 | 27 | 11 | 9 | 11 | 11 | 12 | 13 | 10 | 10 | 9 | 17 | 21 |
| 0 | 0 | 12 | 0 | 17 | 6 | 0 | 0 | 0 | 12 | 13 | 28 | 8 | 0 | 14 | 26 | 15 | 25 | 33 | 50 | 11 | 0 | 12 | 12 | 4 | 3 | 10 | 3 | 4 | 3 | 14 | 37 | 13 | 6 | 0 | 10 | 12 | 12 | 8 | 6 | 13 | 16 | 27 |
| 34 | 19 | 24 | 13 | 15 | 14 | 33 | 12 | 24 | 15 | 6 | 16 | 10 | 14 | 0 | 20 | 21 | 19 | 58 | 42 | 25 | 21 | 19 | 15 | 11 | 13 | 11 | 16 | 17 | 19 | 24 | 60 | 2 | 12 | 16 | 17 | 6 | 4 | 18 | 16 | 0 | 15 | 49 |
| 37 | 37 | 32 | 39 | 35 | 30 | 37 | 32 | 31 | 34 | 25 | 36 | 19 | 26 | 20 | 0 | 16 | 8 | 46 | 11 | 9 | 30 | 32 | 24 | 21 | 25 | 17 | 29 | 27 | 35 | 40 | 48 | 15 | 22 | 30 | 22 | 30 | 33 | 22 | 35 | 0 | 24 | 44 |
| 22 | 21 | 23 | 31 | 25 | 22 | 25 | 23 | 22 | 29 | 22 | 34 | 13 | 15 | 21 | 16 | 0 | 10 | 43 | 18 | 4 | 22 | 25 | 22 | 16 | 22 | 15 | 22 | 25 | 23 | 34 | 45 | 27 | 17 | 23 | 18 | 22 | 22 | 19 | 29 | 15 | 24 | 41 |
| 48 | 32 | 38 | 34 | 34 | 22 | 43 | 19 | 28 | 24 | 21 | 29 | 22 | 25 | 19 | 8 | 10 | 0 | 62 | 7 | 1 | 30 | 19 | 24 | 23 | 23 | 22 | 27 | 21 | 19 | 33 | 66 | 19 | 24 | 26 | 27 | 18 | 10 | 26 | 25 | 18 | 26 | 60 |
| 11 | 39 | 11 | 56 | 27 | 57 | 19 | 71 | 48 | 78 | 53 | 68 | 18 | 33 | 58 | 46 | 43 | 62 | 0 | 63 | 57 | 50 | 63 | 10 | 13 | 57 | 36 | 21 | 32 | 67 | 73 | 11 | 50 | 18 | 44 | 23 | 75 | 71 | 24 | 73 | 21 | 56 | 15 |
| 57 | 54 | 48 | 58 | 46 | 52 | 58 | 56 | 36 | 60 | 45 | 39 | 41 | 50 | 42 | 11 | 18 | 7 | 63 | 1 | 6 | 43 | 54 | 44 | 42 | 56 | 49 | 44 | 45 | 54 | 59 | 66 | 43 | 40 | 60 | 42 | 53 | 51 | 49 | 61 | 38 | 36 | 62 |
| 27 | 22 | 28 | 23 | 28 | 17 | 30 | 23 | 24 | 24 | 29 | 38 | 11 | 11 | 25 | 9 | 4 | 1 | 57 | 6 | 0 | 18 | 22 | 20 | 14 | 25 | 22 | 16 | 23 | 19 | 28 | 54 | 29 | 18 | 20 | 28 | 23 | 24 | 22 | 24 | 22 | 27 | 48 |
| 21 | 13 | 24 | 5 | 19 | 11 | 23 | 17 | 16 | 20 | 21 | 20 | 14 | 0 | 21 | 30 | 22 | 30 | 50 | 43 | 18 | 0 | 19 | 13 | 10 | 19 | 17 | 8 | 16 | 15 | 28 | 54 | 19 | 15 | 14 | 21 | 20 | 20 | 12 | 17 | 16 | 7 | 44 |
| 33 | 17 | 30 | 14 | 31 | 0 | 22 | 8 | 16 | 11 | 11 | 31 | 15 | 12 | 19 | 32 | 25 | 19 | 63 | 54 | 22 | 19 | 0 | 12 | 7 | 14 | 12 | 16 | 14 | 5 | 19 | 65 | 22 | 21 | 15 | 20 | 14 | 15 | 12 | 11 | 16 | 18 | 57 |
| 8 | 16 | 3 | 15 | 12 | 13 | 9 | 0 | 16 | 16 | 18 | 27 | 6 | 12 | 15 | 24 | 22 | 24 | 10 | 44 | 20 | 13 | 12 | 0 | 1 | 11 | 9 | 7 | 7 | 17 | 25 | 16 | 13 | 4 | 8 | 12 | 18 | 16 | 4 | 8 | 11 | 19 | 13 |
| 8 | 12 | 7 | 12 | 8 | 9 | 10 | 1 | 9 | 12 | 14 | 28 | 4 | 4 | 11 | 21 | 16 | 23 | 13 | 42 | 14 | 10 | 7 | 1 | 0 | 7 | 11 | 5 | 4 | 8 | 19 | 19 | 11 | 7 | 9 | 13 | 12 | 14 | 5 | 4 | 10 | 17 | 16 |
| 26 | 21 | 29 | 24 | 26 | 14 | 19 | 0 | 14 | 13 | 18 | 34 | 5 | 3 | 13 | 25 | 22 | 23 | 57 | 56 | 25 | 19 | 14 | 11 | 7 | 0 | 17 | 15 | 9 | 10 | 13 | 63 | 15 | 15 | 8 | 16 | 14 | 19 | 4 | 10 | 0 | 22 | 50 |
| 20 | 9 | 18 | 12 | 26 | 12 | 16 | 17 | 18 | 15 | 10 | 22 | 10 | 10 | 11 | 17 | 15 | 22 | 36 | 49 | 22 | 17 | 12 | 9 | 11 | 17 | 0 | 11 | 14 | 17 | 24 | 40 | 12 | 15 | 15 | 11 | 16 | 15 | 14 | 18 | 11 | 10 | 22 |
| 10 | 10 | 14 | 15 | 19 | 17 | 11 | 17 | 10 | 18 | 21 | 28 | 7 | 3 | 16 | 29 | 22 | 27 | 21 | 44 | 16 | 8 | 16 | 7 | 5 | 15 | 11 | 0 | 10 | 13 | 27 | 26 | 21 | 4 | 17 | 14 | 25 | 24 | 13 | 16 | 12 | 18 | 25 |
| 19 | 17 | 17 | 21 | 18 | 17 | 17 | 0 | 14 | 15 | 11 | 32 | 3 | 4 | 17 | 27 | 25 | 21 | 32 | 45 | 23 | 16 | 14 | 7 | 4 | 9 | 14 | 10 | 0 | 12 | 18 | 34 | 16 | 7 | 6 | 15 | 12 | 13 | 11 | 12 | 11 | 17 | 28 |
| 30 | 15 | 34 | 17 | 31 | 13 | 26 | 7 | 11 | 10 | 8 | 25 | 9 | 3 | 19 | 35 | 23 | 19 | 67 | 54 | 19 | 15 | 5 | 17 | 8 | 10 | 17 | 13 | 12 | 0 | 10 | 69 | 26 | 15 | 14 | 19 | 12 | 15 | 11 | 8 | 21 | 15 | 59 |
| 45 | 21 | 36 | 24 | 37 | 19 | 26 | 14 | 24 | 16 | 24 | 26 | 13 | 14 | 24 | 40 | 34 | 33 | 73 | 59 | 28 | 28 | 19 | 25 | 19 | 13 | 24 | 27 | 18 | 10 | 0 | 72 | 30 | 26 | 13 | 19 | 14 | 20 | 16 | 17 | 30 | 25 | 56 |
| 13 | 38 | 16 | 53 | 31 | 52 | 21 | 75 | 55 | 80 | 61 | 68 | 27 | 37 | 60 | 48 | 45 | 66 | 11 | 66 | 54 | 54 | 65 | 16 | 19 | 63 | 40 | 26 | 34 | 69 | 72 | 0 | 56 | 21 | 48 | 30 | 74 | 74 | 30 | 77 | 28 | 63 | 10 |
| 36 | 22 | 19 | 9 | 15 | 19 | 31 | 20 | 27 | 23 | 8 | 18 | 11 | 13 | 2 | 15 | 27 | 19 | 50 | 43 | 29 | 19 | 22 | 13 | 11 | 15 | 12 | 21 | 16 | 26 | 30 | 56 | 0 | 17 | 17 | 15 | 6 | 6 | 18 | 23 | 0 | 21 | 41 |
| 0 | 13 | 7 | 17 | 17 | 15 | 13 | 0 | 13 | 21 | 16 | 24 | 9 | 6 | 12 | 22 | 17 | 24 | 18 | 40 | 18 | 15 | 21 | 4 | 7 | 15 | 15 | 4 | 7 | 15 | 26 | 21 | 17 | 0 | 8 | 11 | 14 | 13 | 5 | 10 | 10 | 18 | 19 |
| 15 | 11 | 23 | 8 | 24 | 12 | 6 | 0 | 12 | 13 | 21 | 31 | 11 | 0 | 16 | 30 | 23 | 26 | 44 | 60 | 20 | 14 | 15 | 8 | 9 | 8 | 15 | 17 | 6 | 14 | 13 | 48 | 17 | 8 | 0 | 13 | 14 | 15 | 8 | 6 | 15 | 21 | 35 |
| 14 | 13 | 12 | 22 | 16 | 13 | 16 | 17 | 21 | 20 | 19 | 22 | 11 | 10 | 17 | 22 | 18 | 27 | 23 | 42 | 28 | 21 | 20 | 12 | 13 | 16 | 11 | 14 | 15 | 19 | 19 | 30 | 15 | 11 | 13 | 0 | 16 | 19 | 15 | 19 | 14 | 16 | 23 |
| 38 | 22 | 31 | 13 | 19 | 16 | 31 | 6 | 23 | 11 | 10 | 20 | 12 | 12 | 6 | 30 | 22 | 18 | 75 | 53 | 23 | 20 | 14 | 18 | 12 | 14 | 16 | 25 | 12 | 12 | 14 | 74 | 6 | 14 | 14 | 16 | 0 | 4 | 17 | 10 | 16 | 20 | 63 |
| 39 | 17 | 30 | 11 | 17 | 18 | 32 | 9 | 21 | 16 | 8 | 19 | 13 | 12 | 4 | 33 | 22 | 10 | 71 | 51 | 24 | 20 | 15 | 16 | 14 | 19 | 15 | 24 | 13 | 15 | 20 | 74 | 6 | 13 | 15 | 19 | 4 | 0 | 18 | 14 | 17 | 18 | 61 |
| 13 | 16 | 7 | 16 | 12 | 16 | 13 | 3 | 14 | 21 | 17 | 32 | 10 | 8 | 18 | 22 | 19 | 26 | 24 | 49 | 22 | 12 | 12 | 4 | 5 | 4 | 14 | 13 | 11 | 11 | 16 | 30 | 18 | 5 | 8 | 15 | 17 | 18 | 0 | 15 | 12 | 25 | 24 |
| 39 | 21 | 30 | 19 | 39 | 14 | 33 | 0 | 23 | 0 | 13 | 28 | 10 | 6 | 16 | 35 | 29 | 25 | 73 | 61 | 24 | 17 | 11 | 8 | 4 | 10 | 18 | 16 | 12 | 8 | 17 | 77 | 23 | 10 | 6 | 19 | 10 | 14 | 15 | 0 | 19 | 14 | 66 |
| 17 | 19 | 12 | 25 | 17 | 15 | 16 | 15 | 19 | 16 | 17 | 23 | 9 | 13 | 0 | 0 | 15 | 18 | 21 | 38 | 22 | 16 | 16 | 11 | 10 | 0 | 11 | 12 | 11 | 21 | 30 | 28 | 0 | 10 | 15 | 14 | 16 | 17 | 12 | 19 | 0 | 13 | 25 |
| 36 | 18 | 35 | 23 | 22 | 14 | 34 | 16 | 10 | 12 | 7 | 14 | 17 | 16 | 15 | 24 | 24 | 26 | 56 | 36 | 27 | 7 | 18 | 19 | 17 | 22 | 10 | 18 | 17 | 15 | 25 | 63 | 21 | 18 | 21 | 16 | 20 | 18 | 25 | 14 | 13 | 0 | 51 |
| 13 | 31 | 14 | 43 | 27 | 44 | 10 | 61 | 44 | 69 | 53 | 61 | 21 | 27 | 49 | 44 | 41 | 60 | 15 | 62 | 48 | 44 | 57 | 13 | 16 | 50 | 22 | 25 | 28 | 59 | 56 | 10 | 41 | 19 | 35 | 23 | 63 | 61 | 24 | 66 | 25 | 51 | 0 |
| 45 | 22 | 40 | 19 | 33 | 20 | 35 | 7 | 15 | 10 | 13 | 24 | 11 | 10 | 16 | 38 | 23 | 15 | 81 | 55 | 23 | 17 | 11 | 18 | 12 | 13 | 18 | 25 | 14 | 5 | 11 | 80 | 23 | 19 | 16 | 19 | 6 | 7 | 17 | 9 | 17 | 18 | 70 |

| | | | | | | | | | | | | | | | | | | | | | | | | | | | | | | | | | | | | | | | | | | |
|---|---|---|---|---|---|---|---|---|---|---|---|---|---|---|---|---|---|---|---|---|---|---|---|---|---|---|---|---|---|---|---|---|---|---|---|---|---|---|---|---|---|---|
| 37 | 21 | 27 | 18 | 27 | 13 | 33 | 0 | 22 | 16 | 0 | 20 | 14 | 16 | 11 | 32 | 26 | 27 | 65 | 51 | 30 | 25 | 16 | 10 | 13 | 14 | 16 | 22 | 15 | 14 | 22 | 72 | 13 | 7 | 18 | 24 | 12 | 10 | 10 | 8 | 22 | 21 | 63 |
| 9 | 9 | 14 | 13 | 20 | 11 | 8 | 11 | 8 | 13 | 10 | 27 | 13 | 1 | 21 | 22 | 13 | 28 | 25 | 55 | 21 | 9 | 6 | 11 | 5 | 9 | 12 | 4 | 12 | 4 | 8 | 33 | 16 | 12 | 6 | 14 | 17 | 21 | 7 | 12 | 12 | 18 | 19 |
| 46 | 20 | 40 | 20 | 16 | 21 | 39 | 20 | 34 | 25 | 5 | 11 | 18 | 24 | 9 | 42 | 35 | 26 | 75 | 55 | 36 | 30 | 27 | 26 | 22 | 30 | 21 | 32 | 25 | 23 | 25 | 78 | 13 | 23 | 29 | 15 | 5 | 5 | 23 | 22 | 32 | 20 | 66 |
| 47 | 17 | 35 | 13 | 20 | 17 | 34 | 14 | 25 | 12 | 2 | 12 | 18 | 21 | 5 | 32 | 28 | 18 | 76 | 53 | 32 | 25 | 14 | 22 | 19 | 24 | 0 | 24 | 18 | 18 | 19 | 81 | 8 | 22 | 22 | 21 | 5 | 5 | 23 | 15 | 21 | 8 | 67 |
| 42 | 16 | 36 | 21 | 16 | 23 | 41 | 16 | 28 | 22 | 9 | 8 | 20 | 23 | 9 | 38 | 32 | 23 | 75 | 51 | 34 | 26 | 26 | 22 | 21 | 28 | 20 | 31 | 24 | 20 | 24 | 75 | 14 | 22 | 27 | 21 | 9 | 4 | 26 | 22 | 28 | 17 | 67 |
| 39 | 18 | 32 | 19 | 34 | 12 | 26 | 12 | 23 | 15 | 18 | 28 | 12 | 12 | 20 | 37 | 31 | 30 | 61 | 58 | 26 | 26 | 9 | 17 | 14 | 14 | 20 | 20 | 17 | 8 | 0 | 62 | 22 | 20 | 11 | 18 | 16 | 20 | 14 | 14 | 23 | 20 | 46 |
| 32 | 21 | 25 | 18 | 31 | 20 | 23 | 9 | 20 | 19 | 17 | 28 | 11 | 5 | 19 | 25 | 18 | 23 | 42 | 54 | 25 | 17 | 15 | 13 | 9 | 10 | 18 | 15 | 14 | 3 | 14 | 50 | 20 | 17 | 12 | 20 | 17 | 17 | 14 | 14 | 14 | 21 | 35 |
| 8 | 8 | 12 | 10 | 29 | 10 | 9 | 20 | 13 | 17 | 17 | 36 | 14 | 2 | 22 | 32 | 19 | 33 | 24 | 52 | 20 | 14 | 8 | 8 | 7 | 21 | 11 | 12 | 14 | 10 | 14 | 27 | 23 | 6 | 12 | 20 | 26 | 28 | 13 | 20 | 18 | 23 | 19 |
| 27 | 16 | 29 | 21 | 29 | 14 | 24 | 8 | 12 | 11 | 13 | 27 | 8 | 4 | 18 | 23 | 22 | 22 | 50 | 53 | 24 | 20 | 9 | 16 | 8 | 9 | 15 | 8 | 12 | 4 | 16 | 53 | 20 | 12 | 8 | 15 | 12 | 12 | 11 | 9 | 15 | 18 | 44 |
| 8 | 22 | 12 | 28 | 22 | 35 | 11 | 54 | 35 | 60 | 42 | 52 | 15 | 16 | 43 | 41 | 36 | 60 | 11 | 64 | 45 | 35 | 46 | 10 | 15 | 46 | 22 | 21 | 26 | 47 | 50 | 14 | 35 | 17 | 29 | 18 | 54 | 53 | 17 | 58 | 21 | 44 | 0 |
| 36 | 18 | 30 | 10 | 26 | 16 | 28 | 19 | 25 | 19 | 13 | 27 | 22 | 8 | 12 | 35 | 29 | 28 | 67 | 54 | 28 | 21 | 15 | 25 | 17 | 27 | 15 | 27 | 22 | 24 | 23 | 68 | 10 | 23 | 12 | 18 | 1 | 6 | 23 | 22 | 23 | 22 | 51 |
| 12 | 42 | 5 | 58 | 29 | 59 | 16 | 80 | 55 | 82 | 66 | 75 | 25 | 35 | 63 | 52 | 48 | 71 | 8 | 70 | 58 | 57 | 65 | 5 | 11 | 61 | 45 | 21 | 36 | 72 | 77 | 13 | 55 | 24 | 48 | 27 | 79 | 77 | 23 | 80 | 26 | 65 | 12 |
| 9 | 10 | 0 | 8 | 15 | 10 | 11 | 0 | 8 | 13 | 11 | 25 | 8 | 5 | 12 | 23 | 17 | 26 | 27 | 46 | 16 | 6 | 12 | 4 | 6 | 9 | 13 | 5 | 9 | 12 | 21 | 31 | 8 | 4 | 8 | 10 | 14 | 13 | 8 | 6 | 13 | 16 | 24 |
| 4 | 9 | 20 | 4 | 19 | 16 | 10 | 18 | 14 | 25 | 9 | 26 | 12 | 0 | 6 | 29 | 17 | 25 | 39 | 49 | 24 | 19 | 20 | 14 | 10 | 20 | 17 | 20 | 12 | 20 | 26 | 46 | 6 | 9 | 11 | 17 | 1 | 4 | 15 | 22 | 18 | 20 | 34 |
| 13 | 15 | 12 | 17 | 21 | 16 | 14 | 15 | 18 | 26 | 27 | 32 | 9 | 7 | 21 | 28 | 23 | 35 | 8 | 50 | 25 | 17 | 23 | 7 | 6 | 18 | 12 | 15 | 11 | 27 | 24 | 16 | 19 | 10 | 0 | 10 | 28 | 27 | 14 | 20 | 13 | 21 | 7 |
| 45 | 26 | 37 | 23 | 35 | 15 | 35 | 4 | 21 | 8 | 16 | 24 | 10 | 9 | 16 | 35 | 22 | 16 | 80 | 55 | 19 | 18 | 8 | 18 | 11 | 12 | 18 | 15 | 16 | 7 | 15 | 82 | 25 | 16 | 9 | 19 | 9 | 11 | 15 | 5 | 18 | 18 | 70 |
| 40 | 24 | 32 | 21 | 34 | 12 | 31 | 3 | 22 | 8 | 15 | 25 | 9 | 5 | 17 | 33 | 17 | 18 | 71 | 54 | 22 | 18 | 9 | 13 | 9 | 11 | 20 | 15 | 12 | 6 | 14 | 73 | 22 | 12 | 8 | 18 | 8 | 12 | 14 | 4 | 16 | 18 | 61 |
| 0 | 12 | 9 | 17 | 12 | 13 | 12 | 5 | 16 | 19 | 8 | 27 | 7 | 6 | 12 | 21 | 16 | 29 | 15 | 40 | 24 | 16 | 20 | 9 | 6 | 13 | 11 | 15 | 10 | 18 | 16 | 19 | 15 | 3 | 9 | 10 | 11 | 18 | 13 | 10 | 12 | 19 | 20 |
| 8 | 12 | 8 | 14 | 16 | 13 | 16 | 23 | 17 | 20 | 16 | 37 | 8 | 4 | 20 | 26 | 19 | 36 | 13 | 51 | 24 | 15 | 11 | 8 | 6 | 21 | 11 | 10 | 13 | 11 | 20 | 17 | 18 | 9 | 14 | 15 | 27 | 27 | 16 | 25 | 12 | 24 | 10 |
| 6 | 4 | 27 | 9 | 21 | 12 | 6 | 0 | 8 | 21 | 16 | 34 | 16 | 0 | 22 | 40 | 23 | 27 | 49 | 58 | 23 | 16 | 12 | 11 | 6 | 7 | 18 | 9 | 8 | 8 | 19 | 53 | 29 | 5 | 5 | 19 | 18 | 15 | 0 | 14 | 18 | 23 | 45 |
| 11 | 16 | 0 | 15 | 15 | 20 | 9 | 13 | 21 | 26 | 17 | 31 | 7 | 11 | 16 | 24 | 20 | 30 | 7 | 40 | 22 | 17 | 23 | 0 | 6 | 18 | 21 | 13 | 10 | 20 | 31 | 15 | 10 | 4 | 25 | 8 | 23 | 21 | 6 | 17 | 10 | 23 | 8 |
| 49 | 26 | 41 | 27 | 40 | 14 | 38 | 9 | 26 | 9 | 18 | 25 | 12 | 13 | 20 | 36 | 24 | 18 | 81 | 55 | 25 | 21 | 10 | 21 | 12 | 12 | 18 | 26 | 15 | 10 | 15 | 83 | 30 | 25 | 14 | 15 | 5 | 13 | 19 | 11 | 21 | 16 | 74 |
| 7 | 18 | 9 | 17 | 16 | 13 | 17 | 5 | 23 | 16 | 22 | 31 | 9 | 12 | 15 | 27 | 24 | 28 | 22 | 49 | 22 | 14 | 13 | 6 | 7 | 11 | 12 | 15 | 9 | 19 | 21 | 23 | 17 | 1 | 6 | 13 | 19 | 18 | 9 | 12 | 15 | 24 | 26 |
| 43 | 25 | 33 | 29 | 16 | 24 | 40 | 25 | 2 | 29 | 19 | 2 | 20 | 30 | 16 | 33 | 30 | 29 | 57 | 35 | 27 | 2 | 32 | 21 | 22 | 32 | 27 | 26 | 25 | 26 | 38 | 64 | 19 | 25 | 33 | 28 | 22 | 17 | 29 | 29 | 21 | 3 | 55 |

| | | | | | | | | | | | | | | | | | | | | | | | | |
|---|---|---|---|---|---|---|---|---|---|---|---|---|---|---|---|---|---|---|---|---|---|---|---|---|
| 0 | 2 | 8 | 1 | 1 | 1 | 3 | 1 | 1 | 2 | 1 | 0 | 0 | 3 | 1 | 1 | 1 | 2 | 1 | 1 | 3 | 3 | 1 | 1 | 1 |
| 2 | 3 | 12 | 3 | 4 | 4 | 3 | 5 | 5 | 5 | 2 | 1 | 0 | 6 | 7 | 4 | 4 | 9 | 4 | 4 | 7 | 6 | 2 | 3 | 10 |
| Sunki | Suruga Yuko | Sweet orange | Tachibana-A | Tachibana-C | Tachibana-B | Tankan | Temple | Tengu | Tizon | Tosa buntan | Twukkuni | Uchimurasaki | Ujukitsu | Unzoki | USSR Tangelo | Med mandarin | Willowleaf mandarin | Yamabuki | Yamamikan | Yatsushiro | Yuge hyoukan | Youpi ju | Yuukunibu | Yuzu |
| A146 | A147 | A162 | A172 | A174 | A175 | A183 | A186 | A187 | A188 | A191 | A192 | A196 | A197 | A198 | A199 | A200 | A201 | A202 | A203 | A204 | A205 | A206 | A207 | A208 |
| 0 | 0 | 0 | 1 | 0 | 0 | 0 | 0 | 0 | 0 | 0 | 1 | 0 | 0 | 0 | 0 | 0 | 0 | 0 | 0 | 0 | 0 | 0 | 0 | 0 |
| 14 | 12 | 6 | 22 | 20 | 19 | 13 | 11 | 10 | 9 | 14 | 21 | 18 | 7 | 8 | 3 | 11 | 8 | 1 | 7 | 8 | 9 | 13 | 0 | 22 |
| 58 | 52 | 19 | 61 | 57 | 58 | 49 | 35 | 21 | 36 | 12 | 47 | 8 | 13 | 22 | 10 | 61 | 57 | 10 | 11 | 33 | 12 | 63 | 16 | 50 |
| 28 | 29 | 10 | 33 | 30 | 34 | 25 | 21 | 9 | 19 | 8 | 26 | 14 | 7 | 3 | 7 | 29 | 24 | 5 | 6 | 7 | 4 | 30 | 7 | 36 |
| 75 | 67 | 31 | 71 | 77 | 70 | 56 | 47 | 29 | 52 | 10 | 68 | 6 | 26 | 43 | 15 | 77 | 70 | 13 | 12 | 55 | 8 | 77 | 19 | 60 |
| 11 | 14 | 7 | 23 | 12 | 22 | 13 | 5 | 13 | 8 | 53 | 21 | 76 | 8 | 16 | 22 | 5 | 6 | 15 | 19 | 9 | 18 | 9 | 15 | 24 |
| 36 | 38 | 23 | 44 | 36 | 40 | 38 | 27 | 31 | 26 | 31 | 38 | 40 | 23 | 34 | 23 | 38 | 33 | 16 | 22 | 41 | 19 | 37 | 20 | 31 |
| 19 | 17 | 9 | 15 | 10 | 21 | 11 | 17 | 8 | 21 | 33 | 14 | 47 | 11 | 13 | 23 | 20 | 20 | 11 | 13 | 12 | 14 | 24 | 16 | 21 |
| 11 | 11 | 0 | 29 | 28 | 29 | 8 | 1 | 11 | 8 | 51 | 28 | 73 | 14 | 23 | 30 | 8 | 0 | 21 | 10 | 21 | 27 | 15 | 23 | 35 |
| 6 | 15 | 13 | 19 | 17 | 18 | 17 | 14 | 25 | 0 | 61 | 22 | 84 | 17 | 21 | 30 | 9 | 8 | 16 | 29 | 21 | 24 | 8 | 22 | 32 |
| 7 | 17 | 0 | 27 | 23 | 25 | 7 | 3 | 13 | 6 | 41 | 24 | 67 | 7 | 19 | 19 | 7 | 0 | 16 | 9 | 16 | 20 | 14 | 15 | 30 |
| 27 | 25 | 12 | 20 | 13 | 22 | 25 | 22 | 16 | 20 | 11 | 13 | 25 | 15 | 9 | 19 | 31 | 27 | 12 | 9 | 23 | 16 | 34 | 16 | 24 |
| 6 | 21 | 6 | 24 | 18 | 21 | 6 | 9 | 15 | 10 | 56 | 28 | 79 | 18 | 26 | 30 | 8 | 8 | 21 | 15 | 13 | 25 | 9 | 16 | 32 |
| 81 | 71 | 35 | 77 | 79 | 76 | 62 | 49 | 33 | 57 | 15 | 67 | 13 | 34 | 48 | 18 | 80 | 74 | 25 | 23 | 59 | 15 | 80 | 31 | 59 |
| 11 | 3 | 14 | 8 | 4 | 10 | 15 | 13 | 20 | 11 | 46 | 10 | 71 | 5 | 6 | 16 | 9 | 6 | 5 | 23 | 5 | 14 | 10 | 13 | 22 |
| 20 | 26 | 5 | 28 | 24 | 28 | 18 | 11 | 6 | 11 | 20 | 22 | 30 | 12 | 9 | 11 | 19 | 16 | 10 | 7 | 14 | 19 | 25 | 10 | 26 |
| 7 | 20 | 10 | 17 | 22 | 19 | 0 | 10 | 20 | 8 | 53 | 27 | 79 | 11 | 24 | 25 | 8 | 3 | 17 | 21 | 18 | 24 | 13 | 21 | 30 |
| 12 | 11 | 13 | 5 | 8 | 1 | 15 | 15 | 17 | 15 | 45 | 13 | 64 | 8 | 7 | 20 | 14 | 13 | 14 | 16 | 23 | 18 | 17 | 14 | 17 |
| 30 | 32 | 0 | 37 | 39 | 43 | 15 | 5 | 9 | 1 | 12 | 30 | 10 | 5 | 18 | 10 | 27 | 26 | 7 | 4 | 25 | 8 | 34 | 11 | 34 |
| 31 | 22 | 34 | 0 | 14 | 9 | 36 | 32 | 41 | 29 | 53 | 21 | 79 | 32 | 14 | 40 | 30 | 30 | 28 | 43 | 34 | 34 | 34 | 36 | 2 |
| 28 | 30 | 9 | 36 | 32 | 36 | 24 | 19 | 6 | 15 | 7 | 28 | 11 | 7 | 10 | 11 | 30 | 26 | 6 | 7 | 4 | 7 | 35 | 11 | 37 |
| 12 | 16 | 13 | 28 | 24 | 24 | 17 | 11 | 11 | 16 | 41 | 16 | 59 | 6 | 16 | 16 | 10 | 7 | 10 | 20 | 14 | 12 | 17 | 13 | 2 |
| 5 | 13 | 6 | 29 | 25 | 27 | 6 | 5 | 9 | 9 | 53 | 22 | 79 | 13 | 22 | 23 | 10 | 5 | 18 | 12 | 11 | 27 | 14 | 19 | 33 |
| 75 | 67 | 27 | 77 | 79 | 76 | 57 | 43 | 22 | 53 | 6 | 66 | 10 | 28 | 41 | 11 | 76 | 69 | 15 | 15 | 52 | 10 | 78 | 20 | 58 |
| 36 | 31 | 11 | 45 | 37 | 42 | 36 | 19 | 11 | 28 | 13 | 34 | 14 | 12 | 18 | 10 | 36 | 33 | 7 | 10 | 19 | 9 | 40 | 12 | 34 |
| 26 | 27 | 6 | 33 | 30 | 34 | 13 | 13 | 3 | 13 | 12 | 15 | 15 | 3 | 9 | 4 | 21 | 17 | 9 | 6 | 19 | 5 | 29 | 9 | 31 |
| 25 | 23 | 15 | 17 | 12 | 0 | 26 | 25 | 7 | 25 | 17 | 14 | 27 | 15 | 13 | 14 | 26 | 28 | 10 | 14 | 18 | 15 | 29 | 18 | 19 |
| 43 | 41 | 34 | 41 | 39 | 39 | 41 | 35 | 40 | 34 | 44 | 41 | 56 | 29 | 29 | 35 | 40 | 38 | 28 | 31 | 43 | 30 | 43 | 35 | 21 |
| 47 | 39 | 24 | 49 | 47 | 44 | 42 | 35 | 26 | 38 | 24 | 37 | 28 | 18 | 30 | 24 | 52 | 47 | 16 | 21 | 38 | 17 | 57 | 21 | 1 |
| 7 | 20 | 5 | 25 | 22 | 24 | 12 | 8 | 13 | 6 | 21 | 24 | 30 | 7 | 14 | 15 | 12 | 11 | 8 | 10 | 4 | 8 | 12 | 5 | 26 |
| 27 | 26 | 11 | 42 | 35 | 35 | 26 | 17 | 24 | 16 | 37 | 36 | 45 | 11 | 17 | 21 | 29 | 27 | 14 | 19 | 24 | 19 | 36 | 22 | 3 |
| 28 | 23 | 17 | 28 | 24 | 24 | 32 | 24 | 15 | 24 | 20 | 26 | 33 | 7 | 13 | 12 | 34 | 31 | 13 | 11 | 21 | 9 | 38 | 16 | 0 |

| | | | | | | | | | | | | | | | | | | | | | | | | |
|---|---|---|---|---|---|---|---|---|---|---|---|---|---|---|---|---|---|---|---|---|---|---|---|---|
| 18 | 25 | 7 | 30 | 21 | 31 | 21 | 18 | 17 | 14 | 38 | 21 | 58 | 8 | 20 | 19 | 22 | 20 | 18 | 18 | 18 | 20 | 27 | 16 | 1 |
| 24 | 17 | 14 | 12 | 6 | 12 | 26 | 28 | 15 | 22 | 20 | 8 | 46 | 8 | 3 | 20 | 26 | 27 | 10 | 15 | 9 | 18 | 29 | 21 | 24 |
| 45 | 37 | 9 | 46 | 47 | 42 | 39 | 32 | 8 | 27 | 8 | 36 | 12 | 9 | 4 | 13 | 45 | 40 | 0 | 8 | 6 | 11 | 49 | 7 | 43 |
| 22 | 21 | 9 | 20 | 17 | 16 | 18 | 21 | 8 | 16 | 22 | 18 | 42 | 10 | 9 | 15 | 26 | 24 | 12 | 12 | 4 | 16 | 26 | 18 | 25 |
| 40 | 27 | 14 | 40 | 35 | 36 | 32 | 25 | 12 | 29 | 12 | 30 | 5 | 0 | 20 | 12 | 37 | 32 | 9 | 8 | 27 | 0 | 41 | 9 | 33 |
| 19 | 18 | 13 | 20 | 13 | 21 | 19 | 18 | 10 | 21 | 28 | 10 | 58 | 8 | 4 | 17 | 23 | 21 | 17 | 14 | 9 | 15 | 27 | 17 | 29 |
| 33 | 27 | 20 | 16 | 20 | 16 | 34 | 31 | 29 | 29 | 22 | 26 | 29 | 15 | 19 | 21 | 35 | 34 | 12 | 16 | 21 | 15 | 40 | 16 | 16 |
| 20 | 13 | 11 | 21 | 17 | 23 | 12 | 20 | 10 | 14 | 35 | 16 | 59 | 10 | 16 | 16 | 15 | 12 | 13 | 13 | 12 | 20 | 14 | 13 | 24 |
| 35 | 33 | 8 | 39 | 34 | 41 | 26 | 23 | 9 | 24 | 11 | 28 | 16 | 11 | 10 | 14 | 35 | 31 | 12 | 16 | 6 | 9 | 38 | 17 | 40 |
| 7 | 0 | 11 | 20 | 14 | 16 | 12 | 9 | 20 | 8 | 54 | 19 | 80 | 0 | 18 | 15 | 4 | 3 | 5 | 23 | 0 | 13 | 9 | 5 | 25 |
| 15 | 22 | 8 | 34 | 25 | 28 | 23 | 20 | 13 | 12 | 35 | 25 | 55 | 8 | 14 | 18 | 21 | 22 | 16 | 17 | 8 | 21 | 26 | 23 | 2 |
| 10 | 16 | 13 | 25 | 12 | 22 | 15 | 19 | 17 | 11 | 60 | 19 | 82 | 13 | 25 | 26 | 8 | 8 | 19 | 20 | 21 | 26 | 9 | 16 | 29 |
| 13 | 0 | 10 | 5 | 2 | 9 | 18 | 17 | 17 | 13 | 42 | 13 | 66 | 11 | 9 | 27 | 16 | 15 | 8 | 16 | 16 | 17 | 18 | 22 | 19 |
| 24 | 20 | 27 | 11 | 12 | 8 | 28 | 28 | 36 | 27 | 52 | 27 | 75 | 25 | 26 | 32 | 24 | 25 | 27 | 37 | 34 | 31 | 25 | 31 | 2 |
| 11 | 14 | 13 | 18 | 18 | 20 | 12 | 11 | 14 | 8 | 15 | 22 | 25 | 8 | 12 | 9 | 10 | 9 | 7 | 8 | 16 | 7 | 12 | 9 | 20 |
| 10 | 16 | 1 | 24 | 21 | 23 | 12 | 5 | 2 | 4 | 16 | 8 | 35 | 5 | 0 | 7 | 9 | 5 | 6 | 4 | 0 | 11 | 13 | 12 | 30 |
| 16 | 11 | 21 | 9 | 5 | 9 | 20 | 19 | 22 | 18 | 43 | 12 | 63 | 12 | 6 | 21 | 16 | 17 | 12 | 20 | 22 | 16 | 20 | 15 | 16 |
| 38 | 32 | 22 | 42 | 32 | 38 | 37 | 25 | 32 | 23 | 41 | 35 | 52 | 23 | 29 | 28 | 35 | 33 | 21 | 26 | 40 | 24 | 36 | 27 | 33 |
| 23 | 26 | 13 | 35 | 28 | 32 | 31 | 18 | 19 | 22 | 36 | 29 | 48 | 17 | 17 | 23 | 22 | 17 | 16 | 19 | 23 | 20 | 24 | 24 | 30 |
| 15 | 27 | 28 | 26 | 18 | 23 | 30 | 23 | 33 | 22 | 60 | 28 | 71 | 26 | 25 | 35 | 16 | 18 | 29 | 36 | 27 | 30 | 18 | 28 | 29 |
| 81 | 65 | 25 | 75 | 76 | 75 | 61 | 42 | 24 | 50 | 11 | 67 | 8 | 27 | 39 | 8 | 80 | 71 | 15 | 13 | 49 | 7 | 81 | 22 | 57 |
| 55 | 51 | 55 | 55 | 53 | 51 | 58 | 54 | 52 | 53 | 64 | 54 | 70 | 46 | 49 | 50 | 55 | 54 | 40 | 51 | 58 | 40 | 55 | 49 | 35 |
| 23 | 30 | 21 | 36 | 32 | 34 | 26 | 25 | 20 | 24 | 45 | 28 | 58 | 16 | 24 | 25 | 19 | 22 | 24 | 24 | 23 | 22 | 25 | 22 | 27 |
| 17 | 25 | 9 | 30 | 25 | 26 | 26 | 17 | 14 | 20 | 35 | 21 | 57 | 6 | 19 | 17 | 18 | 18 | 16 | 15 | 16 | 17 | 21 | 14 | 2 |
| 11 | 16 | 6 | 27 | 14 | 26 | 9 | 15 | 8 | 9 | 46 | 15 | 65 | 12 | 20 | 23 | 8 | 9 | 20 | 11 | 12 | 23 | 10 | 13 | 32 |
| 18 | 10 | 11 | 26 | 22 | 22 | 17 | 13 | 8 | 16 | 10 | 25 | 5 | 4 | 14 | 7 | 18 | 13 | 9 | 8 | 11 | 0 | 21 | 6 | 21 |
| 12 | 13 | 5 | 22 | 19 | 21 | 14 | 9 | 7 | 8 | 15 | 17 | 11 | 6 | 10 | 6 | 11 | 9 | 6 | 6 | 6 | 6 | 12 | 7 | 22 |
| 13 | 14 | 9 | 30 | 24 | 28 | 14 | 10 | 21 | 9 | 46 | 27 | 61 | 9 | 20 | 18 | 12 | 11 | 13 | 21 | 7 | 18 | 12 | 11 | 32 |
| 18 | 16 | 12 | 21 | 0 | 20 | 20 | 18 | 11 | 15 | 22 | 15 | 45 | 13 | 17 | 12 | 18 | 20 | 11 | 11 | 18 | 21 | 18 | 12 | 27 |
| 25 | 22 | 4 | 32 | 24 | 31 | 20 | 15 | 12 | 8 | 21 | 27 | 21 | 5 | 20 | 15 | 15 | 15 | 15 | 10 | 9 | 13 | 26 | 15 | 26 |
| 14 | 15 | 12 | 25 | 18 | 24 | 17 | 14 | 14 | 12 | 26 | 22 | 36 | 9 | 12 | 11 | 16 | 12 | 10 | 13 | 8 | 10 | 15 | 9 | 25 |
| 5 | 14 | 4 | 23 | 18 | 20 | 8 | 3 | 10 | 4 | 47 | 24 | 72 | 12 | 20 | 27 | 7 | 6 | 18 | 11 | 8 | 20 | 10 | 19 | 26 |
| 11 | 22 | 8 | 25 | 19 | 24 | 0 | 14 | 14 | 16 | 50 | 23 | 77 | 21 | 26 | 24 | 15 | 14 | 16 | 20 | 19 | 31 | 15 | 21 | 38 |
| 80 | 72 | 33 | 78 | 81 | 75 | 62 | 50 | 27 | 53 | 14 | 68 | 13 | 31 | 46 | 16 | 82 | 73 | 19 | 17 | 53 | 15 | 83 | 23 | 64 |
| 23 | 13 | 16 | 13 | 8 | 14 | 22 | 20 | 23 | 20 | 35 | 10 | 55 | 8 | 6 | 19 | 25 | 22 | 15 | 18 | 29 | 10 | 30 | 17 | 19 |
| 19 | 7 | 12 | 23 | 22 | 22 | 20 | 17 | 6 | 12 | 17 | 23 | 24 | 4 | 9 | 10 | 16 | 12 | 3 | 9 | 5 | 4 | 25 | 1 | 25 |
| 16 | 18 | 6 | 29 | 22 | 27 | 11 | 12 | 12 | 8 | 29 | 12 | 48 | 8 | 11 | 0 | 9 | 8 | 9 | 14 | 5 | 25 | 14 | 6 | 33 |
| 19 | 24 | 14 | 15 | 21 | 21 | 18 | 20 | 20 | 15 | 18 | 18 | 27 | 10 | 17 | 10 | 19 | 18 | 10 | 15 | 19 | 8 | 15 | 13 | 28 |
| 6 | 12 | 17 | 5 | 5 | 9 | 16 | 17 | 26 | 12 | 54 | 1 | 79 | 14 | 1 | 28 | 9 | 8 | 11 | 27 | 18 | 23 | 5 | 19 | 22 |
| 7 | 10 | 21 | 5 | 5 | 4 | 20 | 17 | 28 | 12 | 53 | 6 | 77 | 13 | 4 | 27 | 11 | 12 | 18 | 27 | 15 | 21 | 13 | 18 | 17 |
| 17 | 10 | 7 | 23 | 23 | 26 | 14 | 14 | 13 | 11 | 17 | 23 | 23 | 8 | 15 | 14 | 15 | 14 | 13 | 16 | 0 | 6 | 19 | 9 | 29 |
| 9 | 8 | 12 | 22 | 15 | 22 | 14 | 14 | 20 | 9 | 58 | 22 | 80 | 6 | 22 | 20 | 5 | 4 | 10 | 25 | 14 | 17 | 11 | 12 | 29 |
| 17 | 22 | 12 | 32 | 21 | 28 | 23 | 14 | 18 | 15 | 21 | 23 | 26 | 13 | 18 | 13 | 18 | 16 | 12 | 12 | 18 | 10 | 21 | 15 | 21 |
| 18 | 21 | 18 | 20 | 8 | 17 | 20 | 21 | 23 | 18 | 44 | 22 | 65 | 16 | 20 | 21 | 18 | 18 | 19 | 24 | 23 | 23 | 16 | 24 | 3 |
| 70 | 63 | 19 | 66 | 67 | 67 | 46 | 35 | 19 | 44 | 0 | 51 | 12 | 24 | 34 | 7 | 70 | 61 | 20 | 10 | 45 | 8 | 74 | 26 | 55 |
| 0 | 14 | 11 | 17 | 15 | 19 | 12 | 9 | 22 | 9 | 62 | 23 | 86 | 16 | 24 | 30 | 3 | 3 | 15 | 23 | 17 | 28 | 7 | 18 | 27 |

| | | | | | | | | | | | | | | | | | | | | | | | | |
|---|---|---|---|---|---|---|---|---|---|---|---|---|---|---|---|---|---|---|---|---|---|---|---|---|
| 14 | 0 | 20 | 15 | 12 | 15 | 21 | 18 | 24 | 17 | 52 | 24 | 73 | 12 | 15 | 30 | 11 | 10 | 13 | 29 | 11 | 18 | 17 | 17 | 27 |
| 11 | 20 | 0 | 27 | 23 | 30 | 0 | 0 | 0 | 0 | 14 | 21 | 33 | 5 | 11 | 9 | 14 | 11 | 8 | 0 | 4 | 14 | 18 | 12 | 32 |
| 17 | 15 | 27 | 0 | 4 | 2 | 26 | 26 | 38 | 22 | 57 | 15 | 84 | 23 | 11 | 35 | 18 | 18 | 20 | 39 | 26 | 28 | 18 | 30 | 23 |
| 15 | 12 | 23 | 4 | 0 | 7 | 22 | 23 | 33 | 22 | 55 | 9 | 85 | 18 | 5 | 32 | 18 | 17 | 21 | 31 | 27 | 23 | 16 | 24 | 25 |
| 19 | 15 | 30 | 2 | 7 | 0 | 25 | 27 | 38 | 24 | 58 | 20 | 80 | 20 | 15 | 36 | 17 | 16 | 22 | 38 | 27 | 28 | 18 | 25 | 19 |
| 12 | 21 | 0 | 26 | 22 | 25 | 0 | 10 | 5 | 12 | 40 | 24 | 64 | 16 | 23 | 18 | 10 | 10 | 20 | 8 | 16 | 26 | 13 | 17 | 34 |
| 9 | 18 | 0 | 26 | 23 | 27 | 10 | 0 | 7 | 5 | 35 | 20 | 53 | 11 | 26 | 20 | 7 | 3 | 13 | 12 | 16 | 21 | 20 | 15 | 34 |
| 22 | 24 | 0 | 38 | 33 | 38 | 5 | 7 | 0 | 6 | 13 | 20 | 28 | 7 | 12 | 4 | 19 | 19 | 8 | 1 | 15 | 13 | 27 | 13 | 30 |
| 9 | 17 | 0 | 22 | 22 | 24 | 12 | 5 | 6 | 0 | 40 | 19 | 62 | 11 | 17 | 20 | 12 | 9 | 12 | 9 | 12 | 23 | 14 | 16 | 32 |
| 62 | 52 | 14 | 57 | 55 | 58 | 40 | 35 | 13 | 40 | 0 | 40 | 12 | 15 | 20 | 12 | 63 | 55 | 13 | 5 | 32 | 4 | 65 | 21 | 46 |
| 23 | 24 | 21 | 15 | 9 | 20 | 24 | 20 | 20 | 19 | 40 | 1 | 73 | 17 | 7 | 25 | 19 | 21 | 20 | 25 | 25 | 25 | 22 | 28 | 32 |
| 86 | 73 | 33 | 84 | 85 | 80 | 64 | 53 | 28 | 62 | 12 | 73 | 0 | 29 | 44 | 19 | 84 | 77 | 19 | 15 | 55 | 8 | 87 | 26 | 65 |
| 16 | 12 | 5 | 23 | 18 | 20 | 16 | 11 | 7 | 11 | 15 | 17 | 29 | 0 | 5 | 5 | 15 | 9 | 7 | 8 | 5 | 0 | 18 | 8 | 25 |
| 24 | 15 | 11 | 11 | 5 | 15 | 23 | 26 | 12 | 17 | 20 | 7 | 44 | 5 | 0 | 14 | 24 | 22 | 8 | 11 | 9 | 18 | 27 | 15 | 24 |
| 30 | 30 | 9 | 35 | 32 | 36 | 18 | 20 | 4 | 20 | 12 | 25 | 19 | 5 | 14 | 0 | 27 | 23 | 9 | 7 | 19 | 7 | 30 | 6 | 33 |
| 3 | 11 | 14 | 18 | 18 | 17 | 10 | 7 | 19 | 12 | 63 | 19 | 84 | 15 | 24 | 27 | 0 | 3 | 17 | 26 | 14 | 27 | 0 | 17 | 30 |
| 3 | 10 | 11 | 18 | 17 | 16 | 10 | 3 | 19 | 9 | 55 | 21 | 77 | 9 | 22 | 23 | 3 | 0 | 12 | 22 | 13 | 22 | 2 | 12 | 29 |
| 15 | 13 | 8 | 20 | 21 | 22 | 20 | 13 | 8 | 12 | 13 | 20 | 19 | 7 | 8 | 9 | 17 | 12 | 0 | 10 | 14 | 5 | 17 | 4 | 25 |
| 23 | 29 | 0 | 39 | 31 | 38 | 8 | 12 | 1 | 9 | 5 | 25 | 15 | 8 | 11 | 7 | 26 | 22 | 10 | 0 | 16 | 6 | 29 | 13 | 33 |
| 17 | 11 | 4 | 26 | 27 | 27 | 16 | 16 | 15 | 12 | 32 | 25 | 55 | 5 | 9 | 19 | 14 | 13 | 14 | 16 | 0 | 19 | 19 | 10 | 33 |
| 28 | 18 | 14 | 28 | 23 | 28 | 26 | 21 | 13 | 23 | 4 | 25 | 8 | 0 | 18 | 7 | 27 | 22 | 5 | 6 | 19 | 0 | 29 | 12 | 30 |
| 7 | 17 | 18 | 18 | 16 | 18 | 13 | 20 | 27 | 14 | 65 | 22 | 87 | 18 | 27 | 30 | 0 | 2 | 17 | 29 | 19 | 29 | 0 | 20 | 34 |
| 18 | 17 | 12 | 30 | 24 | 25 | 17 | 15 | 13 | 16 | 21 | 28 | 26 | 8 | 15 | 6 | 17 | 12 | 4 | 13 | 10 | 12 | 20 | 0 | 28 |
| 27 | 27 | 32 | 23 | 25 | 19 | 34 | 34 | 30 | 32 | 46 | 32 | 65 | 25 | 24 | 33 | 30 | 29 | 25 | 33 | 33 | 30 | 34 | 28 | 0 |
