# Supplementary material for: Hybrid Origins of Citrus Varieties Inferred from DNA Marker Analysis of Nuclear and Organelle Genomes
Source: PLoS One. 2016 Nov 30;11(11):e0166969. doi: 10.1371/journal.pone.0166969 (PMC5130255; doi:10.1371/journal.pone.0166969)
Supplement: S11 Table — (PDF) [file pone.0166969.s014.pdf]

S11 Table. Representative genotypes of the 22 indigenous citrus varieties and their inferred parental varieties

#	Marker	Parent 1	Parent 2	Offspring
		A049	A059	A001
		Kaikoukan	Kishu	**Andoukan**
12	CUBER939	205/214	209/214	205/214
13	CUBER951	158/161	149/155	155/158
19	GSR10S17	124/161	124/149	149/161
33	GSR3138	132/162	132/137	137/162
43	GSR6122	195/208	208/213	208/213
45	GSR6133	262/274	256/274	256/262
48	NSX23	245/247	245/246	246/247
53	NSX43	131/160	160/167	131/167
62	NSX121	212/223	208/212	212/223
68	NSX153	138/141	141/147	141/141
69	NSX156	207/216	216/219	207/219
73	NSX169	162/171	171/182	162/182
74	NSX170	177/183	171/183	171/177
81	TSRA101	169/183	165/183	169/183
82	TSRA103	174/188	184/188	174/188
86	TSRA110	145/152	160/161	152/160
87	TSRA117	161/164	160/161	160/164
112	SSR07A20	198/201	195/198	195/201
113	SSR07B01	130/135	133/135	130/135
122	SSR08A06	120/124	120/128	124/128
132	SSR08B20	146/153	148/151	146/151
138	SSR08B29	159/165	159/168	165/168
148	SSR08B62	262/280	262/269	262/269
156	SSR08B85	185/194	185/191	185/191
159	SSR08B92	127/130	130/138	130/130
160	SSR08B95	161/164	152/161	152/164
173	TSRB11	225/234	228/234	225/228
182	TSRF136	203/214	206/214	203/206
187	TSRF161	246/254	246/249	246/249
191	TSRF169	302/304	299/304	302/304
207	TSRF229	281/283	278/283	281/283
216	CiBE0447	309/325	325/328	309/328
217	CiBE1644	366/378	378/394	366/378
224	CX2004	196/199	202/206	196/206
227	CX2040	106/110	110/114	106/110
228	CX3001	423/426	417/423	417/426

#	Marker	Parent 1	Parent 2	Offspring
		A085	A141	A006
		Lemon	Sour orange	**Bergamot**
1	CTVR01	292/313	298/313	298/313
8	CUBER918	93/102	102/105	102/105
17	GRW3011	84/90	87/90	87/90
19	GSR10S17	161/167	124/161	161/167
28	GSR3121	141/144	144/152	144/152
31	GSR3136	98/117	117/126	117/126
33	GSR3138	151/164	134/164	151/164
34	GSR3140	177/184	177/180	177/177
42	GSR6101	210/231	210/237	210/210
43	GSR6122	176/208	195/208	176/195
48	NSX23	243/246	246/247	243/246
51	NSX38	239/247	235/247	235/239
53	NSX43	125/167	131/167	131/167
62	NSX121	202/208	208/218	208/218
66	NSX145	129/138	128/138	128/138
72	NSX165	285/295	275/285	285/295
73	NSX169	150/162	162/171	150/162
82	TSRA103	185/188	174/188	174/185
86	TSRA110	153/161	145/161	145/153
87	TSRA117	157/163	160/163	157/163
89	TSGR264	84/167	113/167	84/113
105	SSR07A05	134/142	131/142	134/142
111	SSR07A24	143/147	147/156	147/156
112	SSR07A20	192/195	195/200	195/200
117	SSR07B14	205/217	205/211	205/205
118	SSR07B27	179/185	185/197	185/197
129	SSR08B06	118/121	115/121	118/121
131	SSR08B15	89/95	89/98	89/89
132	SSR08B20	139/145	145/146	145/146
152	SSR08B75	176/189	172/189	176/189
159	SSR08B92	124/132	132/138	132/132
160	SSR08B95	158/161	152/161	152/158
166	TSGR905	241/243	241/242	242/243
172	TSRB09	130/133	130/136	130/136
175	TSRB38	197/211	206/211	211/211
176	TSRB39	364/376	364/370	370/376
178	TSRF105	251/265	259/265	265/265
181	TSRF124	436/438	438/442	438/438
184	TSRF144	214/223	217/223	214/217
185	TSRF157	288/293	288/290	288/288
188	TSRF162	177/186	182/186	186/186
189	TSRF164	224/225	225/230	224/225
196	TSRF192	178/180	175/178	178/180
202	TSRF211	229/240	229/234	229/234
203	TSRF215	232/238	238/243	238/243
213	TSRP06	204/206	202/204	204/204
215	CiBE0246	230/236	233/236	236/236
216	CiBE0447	316/325	309/325	316/325
217	CiBE1644	366/369	366/387	366/387
218	CiBE2165	285/298	285/323	285/285
219	CiBE2227	162/176	160/176	162/176
220	CiBE4265	242/253	249/253	242/253
224	CX2004	199/217	199/202	199/217
227	CX2040	102/103	102/114	102/102
228	CX3001	417/430	417/426	417/430
234	CX5039	417/437	432/437	437/437
236	CX6035	246/258	246/252	246/252

#	Marker	Parent 1	Parent 2	Offspring
		A201	A162	A009
		Willowleaf mandarin	Sweet orange	**Clementine**
1	CTVR01	301/313	298/313	301/313
12	CUBER939	209/213	209/214	209/213
19	GSR10S17	124/149	149/161	124/149
51	NSX38	245/249	239/247	245/247
53	NSX43	153/160	131/167	160/167
69	NSX156	204/216	204/207	204/216
73	NSX169	171/182	162/182	162/182
82	TSRA103	184/204	188/192	184/192
86	TSRA110	152/175	145/161	145/175
105	SSR07A05	131/140	137/140	131/140
112	SSR07A20	195/198	198/200	198/200
118	SSR07B27	182/185	188/191	185/188
132	SSR08B20	150/153	151/153	153/153
148	SSR08B62	263/269	262/274	269/274
152	SSR08B75	176/189	176/193	176/193
156	SSR08B85	191/200	185/191	191/191
160	SSR08B95	152/164	161/164	164/164
173	TSRB11	228/234	225/234	234/234
216	CiBE0447	309/328	319/328	309/328
218	CiBE2165	287/298	281/287	287/287
224	CX2004	202/206	199/206	202/206
234	CX5039	437/442	432/437	437/437

#	Marker	Parent 1	Parent 2	Offspring
		A201	A162	A014
		Willowleaf mandarin	Sweet orange	**Cravo mandarin**
1	CTVR01	301/313	298/313	313/313
12	CUBER939	209/213	209/214	209/214
19	GSR10S17	124/149	149/161	124/149
51	NSX38	245/249	239/247	245/247
53	NSX43	153/160	131/167	131/160
69	NSX156	204/216	204/207	204/204
73	NSX169	171/182	162/182	182/182
82	TSRA103	184/204	188/192	188/204
86	TSRA110	152/175	145/161	152/161
105	SSR07A05	131/140	137/140	137/140
112	SSR07A20	195/198	198/200	198/200
118	SSR07B27	182/185	188/191	182/188
132	SSR08B20	150/153	151/153	151/153
148	SSR08B62	263/269	262/274	263/274
152	SSR08B75	176/189	176/193	176/176
156	SSR08B85	191/200	185/191	191/200
160	SSR08B95	152/164	161/164	152/164
173	TSRB11	228/234	225/234	234/234
216	CiBE0447	309/328	319/328	309/328

#	Marker	Parent 1	Parent 2	Offspring
		A059	A076	A019
		Kishu	Koji	**Fukure mikan**
1	CTVR01	313/320	301/304	301/313
13	CUBER951	149/155	158/161	149/161
19	GSR10S17	124/149	137/149	149/149
42	GSR6101	210/211	210/249	210/210
45	GSR6133	256/274	274/280	274/280
48	NSX23	245/246	245/253	245/246
51	NSX38	249/253	247/251	249/251
62	NSX121	208/212	210/220	212/220
69	NSX156	216/219	204/219	219/219
73	NSX169	171/182	162/165	165/171
74	NSX170	171/183	171/177	177/183
82	TSRA103	184/188	188/192	188/192
86	TSRA110	160/161	152/161	152/160
87	TSRA117	160/161	162/170	160/162
105	SSR07A05	131/140	131/134	131/134
113	SSR07B01	133/135	131/135	131/135
137	SSR08B28	180/184	180/183	183/184
148	SSR08B62	262/269	257/262	262/269
152	SSR08B75	176/185	185/189	185/185

218	CiBE2165	287/298	281/287	281/287
224	CX2004	202/206	199/206	199/206
234	CX5039	437/442	432/437	432/437

#	Marker	Parent 1	Parent 2	Offspring
		A208	A172	A027
		Yuzu	Tachibana-A	**Hanayu**
13	CUBER951	155/161	158/164	155/164
17	GRW3011	87/100	87/90	90/100
28	GSR3121	144/158	144/152	144/158
31	GSR3136	110/117	123/129	110/123
42	GSR6101	210/231	210/211	211/231
45	GSR6133	268/286	256/280	268/280
46	GSR6135	170/176	176/183	170/176
48	NSX23	245/247	245/246	245/245
49	NSX32	252/261	252/253	253/261
51	NSX38	235/251	245/249	235/249
72	NSX165	290/316	286/300	290/300
80	SRPE05	175/180	180/186	180/180
82	TSRA103	185/196	192/200	185/200
89	TSGR264	113/128	128/134	113/128
129	SSR08B06	112/118	115/121	118/121
131	SSR08B15	95/98	95/101	95/95
132	SSR08B20	143/145	150/153	145/153
137	SSR08B28	167/179	126/184	126/167
138	SSR08B29	156/159	159/162	156/159
140	SSR08B32	82/92	96/99	82/99
153	SSR08B78	131/140	131/137	131/131
156	SSR08B85	177/191	183/191	177/183
157	SSR08B88	257/265	268/271	257/271
169	TSR8EM24	146/156	156/159	146/159
172	TSRB09	122/133	133/136	122/133
174	TSRB29	162/168	162/165	165/168
191	TSRF169	299/304	304/307	304/304
196	TSRF192	168/171	171/175	168/175
207	TSRF229	278/283	283/286	283/283
212	TSRN214	134/140	140/146	140/140
216	CiBE0447	319/334	319/325	319/334
217	CiBE1644	369/381	369/394	369/369
219	CiBE2227	165/170	164/167	165/167
220	CiBE4265	237/249	249/253	237/249
221	CX0004	273/276	273/279	273/276
224	CX2004	201/204	208/214	204/214
227	CX2040	108/116	126/128	116/126
228	CX3001	416/423	423/426	416/426
237	CX6037	165/177	153/165	153/177
244	F40	80/95	80/90	80/80
246	F79	169/172	172/174	172/172

#	Marker	Parent 1	Parent 2	Offspring
		A049	A016	A044
		Kaikoukan	Dancy	**Iyo**
4	CUBER410	253/264	253/260	253/264
12	CUBER939	205/214	209/214	205/214
13	CUBER951	158/161	149/161	149/158
33	GSR3138	132/162	134/137	137/162
34	GSR3140	168/177	168/180	168/177
49	NSX32	248/252	252/259	252/252
53	NSX43	131/160	153/167	131/167
62	NSX121	212/223	208/220	212/220
69	NSX156	207/216	204/216	204/207
73	NSX169	162/171	179/182	162/182
74	NSX170	177/183	174/177	177/183
81	TSRA101	169/183	165/183	169/183
82	TSRA103	174/188	177/184	174/177
86	TSRA110	145/152	152/161	145/161
113	SSR07B01	130/135	131/135	130/131
122	SSR08A06	120/124	120/128	124/128
132	SSR08B20	146/153	151/153	146/151
150	SSR08B66	96/108	96/113	96/96
156	SSR08B85	185/194	185/191	185/191
176	TSRB39	367/370	364/396	364/370
184	TSRF144	220/234	220/223	220/223
221	CX0004	261/273	273/276	261/273
224	CX2004	196/199	206/208	196/206
227	CX2040	106/110	110/112	110/112

#	Marker	Parent 1	Parent 2	Offspring
		A081	A208	A045
		Kunenbo-A	Yuzu	**Jabara**
1	CTVR01	298/313	304/326	298/304
12	CUBER939	209/214	196/214	196/209
13	CUBER951	149/161	155/161	149/161
17	GRW3011	87/90	87/100	90/100
19	GSR10S17	124/161	137/149	149/161
33	GSR3138	132/155	126/132	126/155
34	GSR3140	168/177	165/174	174/177
42	GSR6101	210/243	210/231	231/243
45	GSR6133	268/274	268/286	268/274
51	NSX38	239/249	235/251	249/251

153	SSR08B78	134/137	131/134	131/137
159	SSR08B92	130/138	132/138	130/138
172	TSRB09	133/136	122/133	133/133
198	TSRF195	163/169	158/169	158/169
216	CiBE0447	325/328	319/325	319/325
219	CiBE2227	164/170	164/167	167/170
224	CX2004	202/206	206/212	202/212
227	CX2040	110/114	114/126	110/126
228	CX3001	417/423	423/426	423/426

#	Marker	Parent 1	Parent 2	Offspring
		A081	A208	A030
		Kunenbo-A	Yuzu	**Henka mikan**
1	CTVR01	298/313	304/326	313/326
12	CUBER939	209/214	196/214	196/209
13	CUBER951	149/161	155/161	149/161
17	GRW3011	87/90	87/100	87/90
19	GSR10S17	124/161	137/149	124/137
33	GSR3138	132/155	126/132	132/132
34	GSR3140	168/177	165/174	165/168
42	GSR6101	210/243	210/231	210/210
45	GSR6133	268/274	268/286	268/268
51	NSX38	239/249	235/251	235/249
63	NSX132	154/161	154/168	161/168
70	NSX160	162/168	156/162	156/168
72	NSX165	278/300	290/316	278/290
73	NSX169	162/171	153/174	153/171
81	TSRA101	165/183	169/174	165/174
82	TSRA103	184/188	185/196	184/185
86	TSRA110	152/161	145/161	161/161
87	TSRA117	161/170	161/174	161/174
89	TSGR264	113/137	113/128	113/137
110	SSR07A23	164/182	184/193	182/193
113	SSR07B01	133/135	130/131	130/133
117	SSR07B14	205/211	208/211	205/208
118	SSR07B27	176/191	188/191	191/191
124	SSR08A09	94/102	94/110	94/110
126	SSR08A15	135/138	132/135	132/135
127	SSR08A16	141/144	138/141	138/141
129	SSR08B06	115/121	112/118	115/118
131	SSR08B15	89/95	95/98	95/95
132	SSR08B20	151/153	143/145	143/153
137	SSR08B28	180/184	167/179	167/184
138	SSR08B29	159/171	156/159	159/171
140	SSR08B32	92/102	82/92	92/102
152	SSR08B75	176/185	176/189	176/176
153	SSR08B78	131/134	131/140	134/140
156	SSR08B85	191/194	177/191	191/191
157	SSR08B88	265/274	257/265	265/274
159	SSR08B92	130/135	135/141	135/135
160	SSR08B95	161/164	149/152	152/161
161	SSR11A06	240/255	255/258	240/258
172	TSRB09	130/136	122/133	133/136
174	TSRB29	162/165	162/168	165/168
176	TSRB39	364/367	364/376	364/376
181	TSRF124	436/442	436/438	436/436
182	TSRF136	206/214	194/206	194/214
184	TSRF144	220/234	223/229	229/234
188	TSRF162	182/186	186/189	186/189
196	TSRF192	171/175	168/171	171/175
200	TSRF204	203/206	200/206	200/206
203	TSRF215	238/243	235/243	238/243
208	TSRF230	212/224	212/215	215/224
210	TSRF237	177/183	171/177	171/183
215	CiBE0246	233/236	230/233	233/233
216	CiBE0447	325/328	319/334	328/334
217	CiBE1644	366/378	369/381	366/381
218	CiBE2165	281/298	285/289	289/298
219	CiBE2227	160/170	165/170	160/165
224	CX2004	196/206	201/204	201/206
227	CX2040	110/118	108/116	110/116
228	CX3001	420/423	416/423	416/423
230	CX4005	368/372	368/369	368/368
237	CX6037	165/171	165/177	165/165
238	CX6F21	146/152	140/146	140/146
244	F40	90/99	80/95	95/99

#	Marker	Parent 1	Parent 2	Offspring
		A081	A208	A047
		Kunenbo-A	Yuzu	**Kabosu**
1	CTVR01	298/313	304/326	304/313
12	CUBER939	209/214	196/214	214/214
13	CUBER951	149/161	155/161	161/161
17	GRW3011	87/90	87/100	87/100
19	GSR10S17	124/161	137/149	137/161
33	GSR3138	132/155	126/132	132/155
34	GSR3140	168/177	165/174	165/177
42	GSR6101	210/243	210/231	210/231
45	GSR6133	268/274	268/286	274/286
51	NSX38	239/249	235/251	235/239

63	NSX132	154/161	154/168	161/168
70	NSX160	162/168	156/162	156/168
72	NSX165	278/300	290/316	278/316
73	NSX169	162/171	153/174	153/162
81	TSRA101	165/183	169/174	165/174
82	TSRA103	184/188	185/196	184/185
86	TSRA110	152/161	145/161	161/161
87	TSRA117	161/170	161/174	170/174
89	TSGR264	113/137	113/128	113/128
110	SSR07A23	164/182	184/193	182/193
113	SSR07B01	133/135	130/131	131/133
117	SSR07B14	205/211	208/211	205/208
118	SSR07B27	176/191	188/191	188/191
124	SSR08A09	94/102	94/110	94/102
126	SSR08A15	135/138	132/135	132/138
127	SSR08A16	141/144	138/141	138/144
129	SSR08B06	115/121	112/118	118/121
131	SSR08B15	89/95	95/98	89/98
132	SSR08B20	151/153	143/145	143/151
137	SSR08B28	180/184	167/179	179/180
138	SSR08B29	159/171	156/159	156/159
140	SSR08B32	92/102	82/92	82/92
152	SSR08B75	176/185	176/189	185/189
153	SSR08B78	131/134	131/140	134/140
156	SSR08B85	191/194	177/191	177/194
157	SSR08B88	265/274	257/265	257/265
159	SSR08B92	130/135	135/141	135/135
160	SSR08B95	161/164	149/152	152/161
161	SSR11A06	240/255	255/258	240/258
172	TSRB09	130/136	122/133	122/130
174	TSRB29	162/165	162/168	162/162
176	TSRB39	364/367	364/376	364/364
181	TSRF124	436/442	436/438	436/436
182	TSRF136	206/214	194/206	206/206
184	TSRF144	220/234	223/229	220/223
188	TSRF162	182/186	186/189	186/189
196	TSRF192	171/175	168/171	168/171
200	TSRF204	203/206	200/206	200/203
203	TSRF215	238/243	235/243	235/238
208	TSRF230	212/224	212/215	215/224
215	CiBE0246	233/236	230/233	230/236
216	CiBE0447	325/328	319/334	325/334
217	CiBE1644	366/378	369/381	366/369
218	CiBE2165	281/298	285/289	289/298
219	CiBE2227	160/170	165/170	160/165
224	CX2004	196/206	201/204	201/206
227	CX2040	110/118	108/116	108/118
228	CX3001	420/423	416/423	423/423
230	CX4005	368/372	368/369	368/369
237	CX6037	165/171	165/177	171/177
238	CX6F21	146/152	140/146	140/146
244	F40	90/99	80/95	80/90

#	Marker	Parent 1<br>A048<br>Kabuchi	Parent 2<br>A081<br>Kunenbo-A	Offspring<br>A052<br>**Keraji**
1	CTVR01	298/301	298/313	301/313
8	CUBER918	99/105	102/105	99/102
13	CUBER951	149/158	149/161	149/161
19	GSR10S17	124/149	124/161	124/149
34	GSR3140	162/168	168/177	162/168
42	GSR6101	211/243	210/243	210/211
45	GSR6133	274/280	268/274	268/280
46	GSR6135	176/183	170/176	176/183
51	NSX38	239/251	239/249	239/251
69	NSX156	216/222	207/216	207/222
70	NSX160	156/162	162/168	156/162
72	NSX165	285/300	278/300	278/300
73	NSX169	165/171	162/171	162/171
89	TSGR264	113/128	113/137	113/128
111	SSR07A24	153/156	147/153	147/156
113	SSR07B01	131/135	133/135	133/135
115	SSR07B08	187/190	190/193	190/190
118	SSR07B27	185/191	176/191	185/191
132	SSR08B20	150/153	151/153	150/151
136	SSR08B27	96/102	96/105	102/105
137	SSR08B28	180/195	180/184	180/195
140	SSR08B32	92/99	92/102	99/102
148	SSR08B62	257/262	262/274	257/262
156	SSR08B85	194/200	191/194	191/200
157	SSR08B88	265/271	265/274	271/274
159	SSR08B92	130/138	130/135	130/135
160	SSR08B95	152/161	161/164	152/164
163	SSR11A27	216/233	222/233	216/222
172	TSRB09	130/133	130/136	133/136
175	TSRB38	206/208	206/211	206/208
178	TSRF105	259/262	259/265	259/262
184	TSRF144	220/226	220/234	220/226
187	TSRF161	246/249	249/254	249/254
198	TSRF195	158/169	163/169	163/169
200	TSRF204	206/209	203/206	206/206

63	NSX132	154/161	154/168	154/168
70	NSX160	162/168	156/162	156/162
72	NSX165	278/300	290/316	290/300
73	NSX169	162/171	153/174	162/174
81	TSRA101	165/183	169/174	174/183
82	TSRA103	184/188	185/196	185/188
86	TSRA110	152/161	145/161	145/152
87	TSRA117	161/170	161/174	161/170
89	TSGR264	113/137	113/128	113/128
110	SSR07A23	164/182	184/193	164/184
113	SSR07B01	133/135	130/131	130/135
117	SSR07B14	205/211	208/211	211/211
118	SSR07B27	176/191	188/191	176/191
124	SSR08A09	94/102	94/110	94/102
126	SSR08A15	135/138	132/135	135/138
127	SSR08A16	141/144	138/141	141/144
129	SSR08B06	115/121	112/118	115/118
131	SSR08B15	89/95	95/98	95/98
132	SSR08B20	151/153	143/145	143/153
137	SSR08B28	180/184	167/179	179/180
138	SSR08B29	159/171	156/159	159/159
140	SSR08B32	92/102	82/92	92/92
152	SSR08B75	176/185	176/189	185/189
153	SSR08B78	131/134	131/140	131/134
156	SSR08B85	191/194	177/191	177/191
157	SSR08B88	265/274	257/265	265/265
159	SSR08B92	130/135	135/141	130/135
160	SSR08B95	161/164	149/152	149/164
161	SSR11A06	240/255	255/258	240/258
172	TSRB09	130/136	122/133	122/136
174	TSRB29	162/165	162/168	162/162
176	TSRB39	364/367	364/376	364/367
181	TSRF124	436/442	436/438	436/438
182	TSRF136	206/214	194/206	194/206
184	TSRF144	220/234	223/229	220/223
188	TSRF162	182/186	186/189	186/186
196	TSRF192	171/175	168/171	171/171
200	TSRF204	203/206	200/206	203/206
203	TSRF215	238/243	235/243	235/243
208	TSRF230	212/224	212/215	212/215
210	TSRF237	177/183	171/177	177/183
215	CiBE0246	233/236	230/233	233/236
216	CiBE0447	325/328	319/334	319/325
217	CiBE1644	366/378	369/381	369/378
218	CiBE2165	281/298	285/289	285/298
219	CiBE2227	160/170	165/170	165/170
224	CX2004	196/206	201/204	201/206
227	CX2040	110/118	108/116	110/116
228	CX3001	420/423	416/423	416/423
230	CX4005	368/372	368/369	369/372
237	CX6037	165/171	165/177	165/177
238	CX6F21	146/152	140/146	140/152
244	F40	90/99	80/95	80/99

#	Marker	Parent 1<br>A081<br>Kunenbo-A	Parent 2<br>A208<br>Yuzu	Offspring<br>A073<br>**Kizu**
1	CTVR01	298/313	304/326	298/304
12	CUBER939	209/214	196/214	196/214
13	CUBER951	149/161	155/161	161/161
17	GRW3011	87/90	87/100	87/100
19	GSR10S17	124/161	137/149	124/149
33	GSR3138	132/155	126/132	126/132
34	GSR3140	168/177	165/174	168/174
42	GSR6101	210/243	210/231	231/243
45	GSR6133	268/274	268/286	268/274
51	NSX38	239/249	235/251	249/251
63	NSX132	154/161	154/168	154/168
70	NSX160	162/168	156/162	156/168
72	NSX165	278/300	290/316	300/316
73	NSX169	162/171	153/174	153/171
81	TSRA101	165/183	169/174	174/183
82	TSRA103	184/188	185/196	185/188
86	TSRA110	152/161	145/161	145/161
87	TSRA117	161/170	161/174	161/174
89	TSGR264	113/137	113/128	113/128
110	SSR07A23	164/182	184/193	182/193
113	SSR07B01	133/135	130/131	130/135
117	SSR07B14	205/211	208/211	205/208
118	SSR07B27	176/191	188/191	188/191
124	SSR08A09	94/102	94/110	94/102
126	SSR08A15	135/138	132/135	132/135
127	SSR08A16	141/144	138/141	138/141
129	SSR08B06	115/121	112/118	112/115
131	SSR08B15	89/95	95/98	89/98
132	SSR08B20	151/153	143/145	143/153
137	SSR08B28	180/184	167/179	179/180
138	SSR08B29	159/171	156/159	159/159
140	SSR08B32	92/102	82/92	92/92
152	SSR08B75	176/185	176/189	176/176
153	SSR08B78	131/134	131/140	134/140
156	SSR08B85	191/194	177/191	177/194

216	CiBE0447	319/325	325/328	319/325
218	CiBE2165	281/294	281/298	294/298
219	CiBE2227	167/170	160/170	160/170
224	CX2004	196/212	196/206	196/212
227	CX2040	102/118	110/118	118/118
234	CX5039	432/442	432/437	437/442
237	CX6037	153/171	165/171	153/165
244	F40	80/90	90/99	80/99

#	Marker	Parent 1<br>A081<br>Kunenbo-A	Parent 2<br>A208<br>Yuzu	Offspring<br>A091<br>**Mochiyu**
1	CTVR01	298/313	304/326	313/326
12	CUBER939	209/214	196/214	196/209
13	CUBER951	149/161	155/161	149/161
17	GRW3011	87/90	87/100	87/100
19	GSR10S17	124/161	137/149	124/149
33	GSR3138	132/155	126/132	126/132
34	GSR3140	168/177	165/174	168/174
42	GSR6101	210/243	210/231	210/210
45	GSR6133	268/274	268/286	268/286
51	NSX38	239/249	235/251	239/251
63	NSX132	154/161	154/168	161/168
70	NSX160	162/168	156/162	162/162
72	NSX165	278/300	290/316	290/300
73	NSX169	162/171	153/174	162/174
81	TSRA101	165/183	169/174	165/174
82	TSRA103	184/188	185/196	184/185
86	TSRA110	152/161	145/161	152/161
87	TSRA117	161/170	161/174	161/170
89	TSGR264	113/137	113/128	113/128
110	SSR07A23	164/182	184/193	164/184
113	SSR07B01	133/135	130/131	130/133
117	SSR07B14	205/211	208/211	211/211
118	SSR07B27	176/191	188/191	176/188
124	SSR08A09	94/102	94/110	94/102
126	SSR08A15	135/138	132/135	135/138
127	SSR08A16	141/144	138/141	141/144
129	SSR08B06	115/121	112/118	118/121
131	SSR08B15	89/95	95/98	95/95
132	SSR08B20	151/153	143/145	143/151
137	SSR08B28	180/184	167/179	179/180
138	SSR08B29	159/171	156/159	159/171
140	SSR08B32	92/102	82/92	92/102
152	SSR08B75	176/185	176/189	176/176
153	SSR08B78	131/134	131/140	131/131
156	SSR08B85	191/194	177/191	191/191
157	SSR08B88	265/274	257/265	265/274
159	SSR08B92	130/135	135/141	130/135
160	SSR08B95	161/164	149/152	152/164
161	SSR11A06	240/255	255/258	240/255
172	TSRB09	130/136	122/133	133/136
174	TSRB29	162/165	162/168	165/168
176	TSRB39	364/367	364/376	364/367
181	TSRF124	436/442	436/438	438/442
182	TSRF136	206/214	194/206	206/214
184	TSRF144	220/234	223/229	220/223
188	TSRF162	182/186	186/189	182/186
196	TSRF192	171/175	168/171	168/175
200	TSRF204	203/206	200/206	200/206
203	TSRF215	238/243	235/243	235/243
208	TSRF230	212/224	212/215	215/224
210	TSRF237	177/183	171/177	177/177
215	CiBE0246	233/236	230/233	230/233
216	CiBE0447	325/328	319/334	319/328
217	CiBE1644	366/378	369/381	369/378
218	CiBE2165	281/298	285/289	281/285
219	CiBE2227	160/170	165/170	160/165
224	CX2004	196/206	201/204	196/201
227	CX2040	110/118	108/116	116/118
228	CX3001	420/423	416/423	416/423
230	CX4005	368/372	368/369	369/372
237	CX6037	165/171	165/177	165/165
238	CX6F21	146/152	140/146	140/146
244	F40	90/99	80/95	95/99

#	Marker	Parent 1<br>A049<br>Kaikoukan	Parent 2<br>A059<br>Kishu	Offspring<br>A112<br>**Sanbokan**
12	CUBER939	205/214	209/214	205/214
13	CUBER951	158/161	149/155	155/158
19	GSR10S17	124/161	124/149	124/161
33	GSR3138	132/162	132/137	132/162
43	GSR6122	195/208	208/213	208/213
45	GSR6133	262/274	256/274	256/262
48	NSX23	245/247	245/246	245/247
53	NSX43	131/160	160/167	131/160
62	NSX121	212/223	208/212	208/223
68	NSX153	138/141	141/147	141/141
69	NSX156	207/216	216/219	216/219
73	NSX169	162/171	171/182	162/171

157	SSR08B88	265/274	257/265	265/265
159	SSR08B92	130/135	135/141	135/141
160	SSR08B95	161/164	149/152	149/161
161	SSR11A06	240/255	255/258	240/258
172	TSRB09	130/136	122/133	122/130
174	TSRB29	162/165	162/168	165/168
176	TSRB39	364/367	364/376	364/367
181	TSRF124	436/442	436/438	436/436
182	TSRF136	206/214	194/206	206/214
184	TSRF144	220/234	223/229	220/223
188	TSRF162	182/186	186/189	186/189
196	TSRF192	171/175	168/171	168/175
200	TSRF204	203/206	200/206	200/206
203	TSRF215	238/243	235/243	235/243
208	TSRF230	212/224	212/215	212/215
210	TSRF237	177/183	171/177	177/183
215	CiBE0246	233/236	230/233	230/233
216	CiBE0447	325/328	319/334	325/334
217	CiBE1644	366/378	369/381	369/378
218	CiBE2165	281/298	285/289	289/298
219	CiBE2227	160/170	165/170	165/170
224	CX2004	196/206	201/204	201/206
227	CX2040	110/118	108/116	116/118
228	CX3001	420/423	416/423	423/423
230	CX4005	368/372	368/369	369/372
237	CX6037	165/171	165/177	171/177
238	CX6F21	146/152	140/146	140/152
244	F40	90/99	80/95	80/90

#	Marker	Parent 1<br>A059<br>Kishu	Parent 2<br>A141<br>Sour orange	Offspring<br>A100<br>**Nidonari mikan**
1	CTVR01	313/320	298/313	298/320
12	CUBER939	209/214	205/218	209/218
19	GSR10S17	124/149	124/161	149/161
33	GSR3138	132/137	134/164	137/164
42	GSR6101	210/211	210/237	211/237
43	GSR6122	208/213	195/208	208/213
48	NSX23	245/246	246/247	245/246
51	NSX38	249/253	235/247	235/249
53	NSX43	160/167	131/167	131/167
62	NSX121	208/212	208/218	208/212
69	NSX156	216/219	207/216	216/219
70	NSX160	156/168	162/168	162/168
73	NSX169	171/182	162/171	171/182
74	NSX170	171/183	177/183	177/183
81	TSRA101	165/183	169/183	165/169
82	TSRA103	184/188	174/188	184/188
86	TSRA110	160/161	145/161	161/161
87	TSRA117	160/161	160/163	160/160
105	SSR07A05	131/140	131/142	140/142
112	SSR07A20	195/198	195/200	195/200
113	SSR07B01	133/135	130/131	130/133
118	SSR07B27	185/191	185/197	191/197
122	SSR08A06	120/128	120/124	120/120
132	SSR08B20	148/151	145/146	145/148
137	SSR08B28	180/184	175/180	175/180
148	SSR08B62	262/269	269/274	262/274
152	SSR08B75	176/185	172/189	172/176
156	SSR08B85	185/191	179/191	179/185
159	SSR08B92	130/138	132/138	130/138
172	TSRB09	133/136	130/136	130/133
173	TSRB11	228/234	225/228	228/234
191	TSRF169	299/304	304/307	299/304
207	TSRF229	278/283	283/286	278/283
216	CiBE0447	325/328	309/325	325/328
217	CiBE1644	378/394	366/387	378/387
219	CiBE2227	164/170	160/176	160/170
224	CX2004	202/206	199/202	199/206
227	CX2040	110/114	102/114	110/114
228	CX3001	417/423	417/426	417/423

#	Marker	Parent 1<br>A059<br>Kishu	Parent 2<br>A081<br>Kunenbo-A	Offspring<br>A125<br>**Satsuma**
1	CTVR01	313/320	298/313	298/313
13	CUBER951	149/155	149/161	155/161
19	GSR10S17	124/149	124/161	149/161
33	GSR3138	132/137	132/155	137/155
42	GSR6101	210/211	210/243	210/243
45	GSR6133	256/274	268/274	256/274
48	NSX23	245/246	245/247	246/247
51	NSX38	249/253	239/249	249/249
53	NSX43	160/167	131/160	131/167
62	NSX121	208/212	212/218	208/212
68	NSX153	141/147	138/141	138/141
69	NSX156	216/219	207/216	207/219
70	NSX160	156/168	162/168	168/168
73	NSX169	171/182	162/171	171/182
86	TSRA110	160/161	152/161	152/160
87	TSRA117	160/161	161/170	160/161

74	NSX170	177/183	171/183	177/183
81	TSRA101	169/183	165/183	169/183
82	TSRA103	174/188	184/188	174/188
86	TSRA110	145/152	160/161	145/161
87	TSRA117	161/164	160/161	161/164
112	SSR07A20	198/201	195/198	198/201
113	SSR07B01	130/135	133/135	130/135
122	SSR08A06	120/124	120/128	124/128
132	SSR08B20	146/153	148/151	146/148
138	SSR08B29	159/165	159/168	159/168
148	SSR08B62	262/280	262/269	269/280
156	SSR08B85	185/194	185/191	185/191
159	SSR08B92	127/130	130/138	127/138
160	SSR08B95	161/164	152/161	161/161
173	TSRB11	225/234	228/234	225/234
182	TSRF136	203/214	206/214	203/214
187	TSRF161	246/254	246/249	246/254
191	TSRF169	302/304	299/304	299/302
207	TSRF229	281/283	278/283	278/281
216	CiBE0447	309/325	325/328	309/328
217	CiBE1644	366/378	378/394	366/394
224	CX2004	196/199	202/206	196/202
227	CX2040	106/110	110/114	110/114
228	CX3001	423/426	417/423	423/426

#	Marker	Parent 1<br>A059<br>Kishu	Parent 2<br>A075<br>Kobeni mikan	Offspring<br>A138<br>**Sokitsu**
1	CTVR01	313/320	307/313	307/313
13	CUBER951	149/155	149/161	149/161
51	NSX38	249/253	247/253	247/249
53	NSX43	160/167	153/167	153/160
62	NSX121	208/212	208/210	210/212
70	NSX160	156/168	162/168	156/162
86	TSRA110	160/161	152/160	152/161
87	TSRA117	160/161	161/169	161/169
132	SSR08B20	148/151	151/153	148/151
138	SSR08B29	159/168	159/162	159/168
152	SSR08B75	176/185	189/193	185/193
153	SSR08B78	134/137	131/137	131/137
160	SSR08B95	152/161	152/164	161/164
217	CiBE1644	378/394	394/403	394/403
227	CX2040	110/114	108/114	110/114

#	Marker	Parent 1<br>A201<br>Willowleaf mandarin	Parent 2<br>A162<br>Sweet orange	Offspring<br>A186<br>**Temple**
1	CTVR01	301/313	298/313	313/313
12	CUBER939	209/213	209/214	209/213
19	GSR10S17	124/149	149/161	149/161
51	NSX38	245/249	239/247	247/249
53	NSX43	153/160	131/167	131/160
69	NSX156	204/216	204/207	207/216
73	NSX169	171/182	162/182	162/171
82	TSRA103	184/204	188/192	184/192
86	TSRA110	152/175	145/161	145/175
105	SSR07A05	131/140	137/140	131/137
112	SSR07A20	195/198	198/200	195/200
132	SSR08B20	150/153	151/153	150/153
148	SSR08B62	263/269	262/274	263/274
156	SSR08B85	191/200	185/191	185/191
160	SSR08B95	152/164	161/164	161/164
173	TSRB11	228/234	225/234	225/234
218	CiBE2165	287/298	281/287	287/287
224	CX2004	202/206	199/206	199/202
234	CX5039	437/442	432/437	437/437

#	Marker	Parent 1<br>A059<br>Kishu	Parent 2<br>A081<br>Kunenbo-A	Offspring<br>A204<br>**Yatsushiro**
1	CTVR01	313/320	298/313	298/320
13	CUBER951	149/155	149/161	155/161
19	GSR10S17	124/149	124/161	124/149
33	GSR3138	132/137	132/155	132/137
42	GSR6101	210/211	210/243	211/243
45	GSR6133	256/274	268/274	256/268
48	NSX23	245/246	245/247	245/247
51	NSX38	249/253	239/249	239/249
53	NSX43	160/167	131/160	160/167
62	NSX121	208/212	212/218	208/218
68	NSX153	141/147	138/141	138/147
69	NSX156	216/219	207/216	207/219
70	NSX160	156/168	162/168	162/168
73	NSX169	171/182	162/171	171/182
86	TSRA110	160/161	152/161	161/161
87	TSRA117	160/161	161/170	160/161
105	SSR07A05	131/140	134/140	131/134

105	SSR07A05	131/140	134/140	134/140
112	SSR07A20	195/198	198/200	195/200
118	SSR07B27	185/191	176/191	176/191
132	SSR08B20	148/151	151/153	148/151
138	SSR08B29	159/168	159/171	168/171
148	SSR08B62	262/269	262/274	262/274
153	SSR08B78	134/137	131/134	131/137
156	SSR08B85	185/191	191/194	191/194
159	SSR08B92	130/138	130/135	130/138
160	SSR08B95	152/161	161/164	152/164
172	TSRB09	133/136	130/136	130/136
187	TSRF161	246/249	249/254	249/254
217	CiBE1644	378/394	366/378	366/394
219	CiBE2227	164/170	160/170	160/170
224	CX2004	202/206	196/206	196/206
227	CX2040	110/114	110/118	110/118
228	CX3001	417/423	420/423	417/420

#	Marker	Parent 1<br>A059<br>Kishu	Parent 2<br>A076<br>Koji	Offspring<br>A147<br>**Suruga Yuko**
1	CTVR01	313/320	301/304	304/313
13	CUBER951	149/155	158/161	155/158
19	GSR10S17	124/149	137/149	124/137
42	GSR6101	210/211	210/249	211/249
45	GSR6133	256/274	274/280	256/274
48	NSX23	245/246	245/253	245/246
51	NSX38	249/253	247/251	251/253
62	NSX121	208/212	210/220	212/220
69	NSX156	216/219	204/219	204/219
73	NSX169	171/182	162/165	165/182
74	NSX170	171/183	171/177	171/171
82	TSRA103	184/188	188/192	184/192
86	TSRA110	160/161	152/161	152/161
87	TSRA117	160/161	162/170	160/162
105	SSR07A05	131/140	131/134	131/134
113	SSR07B01	133/135	131/135	131/133
137	SSR08B28	180/184	180/183	180/183
148	SSR08B62	262/269	257/262	257/269
152	SSR08B75	176/185	185/189	185/189
153	SSR08B78	134/137	131/134	134/137
159	SSR08B92	130/138	132/138	138/138
172	TSRB09	133/136	122/133	122/133
198	TSRF195	163/169	158/169	158/163
216	CiBE0447	325/328	319/325	325/328
219	CiBE2227	164/170	164/167	167/170
224	CX2004	202/206	206/212	202/212
227	CX2040	110/114	114/126	110/114
228	CX3001	417/423	423/426	417/426

#	Marker	Parent 1<br>A162<br>Sweet orange	Parent 2<br>A013<br>Cleopatra	Offspring<br>A188<br>**Tizon**
1	CTVR01	298/313	301/313	313/313
9	CUBER920	158/162	162/170	158/162
12	CUBER939	209/214	209/213	213/214
31	GSR3136	117/131	113/126	126/131
34	GSR3140	168/177	168/171	171/177
49	NSX32	252/262	252/259	259/262
51	NSX38	239/247	249/253	239/249
62	NSX121	208/210	208/212	210/212
82	TSRA103	188/192	184/192	188/192
105	SSR07A05	137/140	131/140	131/137
112	SSR07A20	198/200	192/198	198/200
118	SSR07B27	188/191	185/191	185/188
131	SSR08B15	89/101	89/98	89/89
147	SSR08B60	125/129	118/133	118/129
153	SSR08B78	131/134	131/137	134/137
173	TSRB11	225/234	228/234	225/234
181	TSRF124	436/442	436/438	436/438
216	CiBE0447	319/328	309/322	309/328
219	CiBE2227	160/170	164/170	160/164
227	CX2040	110/112	108/110	108/110
228	CX3001	417/423	417/426	423/426

#	Marker	Parent 1<br>A001<br>Andoukan	Parent 2<br>A112<br>Sanbokan	Offspring<br>A207<br>**Yuukunibu**
8	CUBER918	96/102	102/105	96/102
19	GSR10S17	149/161	124/161	149/161
33	GSR3138	137/162	132/162	137/162
48	NSX23	246/247	245/247	246/247
51	NSX38	239/249	239/253	239/249
53	NSX43	131/167	131/160	131/167
62	NSX121	212/223	208/223	212/223
69	NSX156	207/219	216/219	207/219
70	NSX160	162/168	156/162	162/168
73	NSX169	162/182	162/171	162/182
74	NSX170	171/177	177/183	171/177
87	TSRA117	160/164	161/164	160/164

112	SSR07A20	195/198	198/200	195/198
118	SSR07B27	185/191	176/191	185/191
132	SSR08B20	148/151	151/153	148/151
138	SSR08B29	159/168	159/171	168/171
148	SSR08B62	262/269	262/274	262/269
153	SSR08B78	134/137	131/134	131/137
156	SSR08B85	185/191	191/194	185/194
159	SSR08B92	130/138	130/135	130/135
160	SSR08B95	152/161	161/164	161/161
172	TSRB09	133/136	130/136	130/133
187	TSRF161	246/249	249/254	246/254
217	CiBE1644	378/394	366/378	378/378
219	CiBE2227	164/170	160/170	160/164
224	CX2004	202/206	196/206	196/202
227	CX2040	110/114	110/118	110/114
228	CX3001	417/423	420/423	420/423

105	SSR07A05	134/140	131/134	134/140
111	SSR07A24	147/156	147/153	147/156
112	SSR07A20	195/201	198/201	195/201
132	SSR08B20	146/151	146/148	146/151
138	SSR08B29	165/168	159/168	165/168
148	SSR08B62	262/269	269/280	262/269
173	TSRB11	225/228	225/234	225/228
177	TSRF101	152/161	154/161	152/161
182	TSRF136	203/206	203/214	203/206
187	TSRF161	246/249	246/254	246/246
191	TSRF169	302/304	299/302	302/304
207	TSRF229	281/283	278/281	281/283
217	CiBE1644	366/378	366/394	366/378
224	CX2004	196/206	196/202	196/206
227	CX2040	106/110	110/114	110/110
228	CX3001	417/426	423/426	417/426
